# Induction of epigenetic variation in Arabidopsis by over-expression of *DNA METHYLTRANSFERASE1 (MET1)*

**DOI:** 10.1371/journal.pone.0192170

**Published:** 2018-02-21

**Authors:** Samuel Brocklehurst, Michael Watson, Ian M. Carr, Suzan Out, Iris Heidmann, Peter Meyer

**Affiliations:** 1 Center for Plant Sciences, University of Leeds, Leeds, United Kingdom; 2 School of Medicine Institute of Biomed. & Clin. Sciences (LIBACS), University of Leeds, Leeds, United Kingdom; 3 Enza Zaden Research and Development B.V., Enkhuizen, NL; National Taiwan University, TAIWAN

## Abstract

Epigenetic marks such as DNA methylation and histone modification can vary among plant accessions creating epi-alleles with different levels of expression competence. Mutations in epigenetic pathway functions are powerful tools to induce epigenetic variation. As an alternative approach, we investigated the potential of over-expressing an epigenetic function, using *DNA METHYLTRANSFERASE1 (MET1)* for proof-of-concept. In *Arabidopsis thaliana*, MET1 controls maintenance of cytosine methylation at symmetrical CG positions. At some loci, which contain dense DNA methylation in CG- and non-CG context, loss of MET1 causes joint loss of all cytosines methylation marks. We find that over-expression of both catalytically active and inactive versions of *MET1* stochastically generates new epi-alleles at loci encoding transposable elements, non-coding RNAs and proteins, which results for most loci in an increase in expression. Individual transformants share some common phenotypes and genes with altered gene expression. Altered expression states can be transmitted to the next generation, which does not require the continuous presence of the *MET1* transgene. Long-term stability and epigenetic features differ for individual loci. Our data show that over-expression of *MET1*, and potentially of other genes encoding epigenetic factors, offers an alternative strategy to identify epigenetic target genes and to create novel epi-alleles.

## Introduction

DNA methylation patterns in plants influence a number of molecular mechanisms, including transcription [1], repair [2] and recombination [3], with implications for plant development [4], genome structure [5] and evolution [6]. The responsiveness of DNA methylation patterns to environmental stress [7] has been proposed to act as a molecular switch for evolutionary adaptation of plants to environmental change [8]. In support of this model, various biotic [9] and abiotic stress conditions [10] have been shown to alter DNA methylation profiles. The epi-genotype has therefore emerged as an additional factor to genetic mutations in shaping phenotypic diversity [11], [12].

Cytosine methylation in *Arabidopsis* occurs in three sequence contexts. The most prominent methylation mark at CG sites is faithfully propagated by maintenance DNA METHYLTRANSFERASE1 (MET1), a plant homolog of the mammalian DNA methyltransferase 1 (Dnmt1). Non-symmetrical CHH methylation (H representing C, T or A) is controlled by the RNA-directed DNA methylation (RdDM) pathway with 24nt small RNAs (siRNAs) acting as guides for *de novo* DOMAINS REARRANGED METHYLTRANSFERASE 2 (DRM2). A third DNA methyltransferase, CHROMOMETHYLASE3 (CMT3), which is exclusively found in plants, predominantly controls CHG methylation [13] in combination with histone methyltransferases [14]. The RdDM pathway predominantly controls repeats in heterochromatic regions and in dispersed transposons, and related sequences in euchromatic regions [15]. At a number of loci, RdDM-mediated DNA methylation is supported by the Snf2 remodeler DRD1, which forms a complex with RdDM pathway proteins [16]. An RdDM-independent DNA methylation pathway is controlled by DDM1, another Snf2 family nucleosome remodeler, which facilitates access to heterochromatic regions for DNA methyltransferases, especially for CHROMOMETHYLASE 2 (CMT2), which controls the majority of methylation at CHH sites in pericentromeric heterochromatin [17].

The analysis of distinct genomic loci has helped to establish mechanistic models that allocate specific functions to the different DNA methyltransferases. MET1 has mainly been discussed in the context of its maintenance function for CG methylation marks, providing more stable epigenetic patterns than the target loci of the RdDM pathway, which show a higher level of epigenetic variation in *Arabidopsis* accessions [18]. The role of MET1, however, is not strictly limited to maintenance of CG methylation. At some genetic regions with dense DNA methylation in all sequence contexts, elimination of MET1 activity causes a loss of all methylation marks [19], which can result in heritable loss of dense methylation patterns creating novel epi-alleles and states of expression [20]. At many of these loci, dense DNA methylation is independent of DRM2 and other components of the RNA-directed DNA methylation (RdDM) pathway. Instead, dense methylation at these loci requires the nucleosome remodeler DDM1, with CHH methylation being controlled by CHROMOMETHYLASE 2 (CMT2) and CHG methylation by CHROMOMETHYLASE3 (CMT3) [20].

There are several mechanistic options that could explain how MET1 depletion could result in a loss of CG and non-CG marks in dense methylation region. MET1 may be part of a multi-protein complex that also contains CMT2 and/or CMT3 and that would be non-functional without MET1. Alternatively, MET1 depletion would be have an indirect effect on other epigenetic factors that it interacts with, and that are required for dense methylation. This could involve interaction of MET1 with histone regulators like HISTONE DEACETYLASE6 (HDA6), for which direct binding to MET1 has been demonstrated [21] and which has been proposed to recruit MET1 to certain target loci as the initial step in establishing subsequent non-CG methylation [22]. Finally, depletion of MET1-controlled CG-methylation in dense methylation region could remove epigenetic marks established by CG-methylation, which may be required to recruit CMT2 and CMT3. An indirect effect of MET1 on non-CG methylation has, for example, been observed at certain loci that lose their H3K9 methylation patterns in a *met1* mutant, which resulted in a loss of CHG and CHH methylation marks [23].

Any MET1 function that involves interaction with other epigenetic factors would not only be sensitive to MET1 depletion but may also be disturbed by an increase in MET1 concentration, if this causes an imbalance in the availability or function of MET1-binding partners. Any effect that was induced by interaction of MET1 with other factors, would not necessarily require an increase in MET1 protein levels with a functional catalytic activity. To investigate this option, we tested the effect of introducing high levels of catalytically active and inactive MET1 proteins into Arabidopsis. We find that, independent of the catalytic ability of the *MET1* transgene, its expression induces heritable epi-alleles at distinct loci with altered expression levels and epigenetic marks.

## Materials and methods

### Construction of plasmids and plant transformation

DNA fragments with compatible ends were ligated in a reaction incubated for 17 h at 4 ^o^C using 1 U of T4 DNA ligase (Promega). De-phosphorylation was carried out using calf intestinal alkaline phosphatase (Promega) according to the manufacturer’s instructions. 5’ overhangs produced after amplicon assembly were filled by PCR using the Phusion high-fidelity PCR kit (Finnzymes). *Arabidopsis* transformation was carried out by floral dip [24].

The *MET1* cDNA [25] was cut from p-GEM T easy (Promega) using *EcoR*I and was subsequently ligated into pGreen II 0179 35S-*NOS*, which contains a single *EcoR*I site in the polylinker region between the promoter and terminator. To remove the catalytic function from MET1 we followed the strategy documented by Hsieh et al [26] and exchanged the cysteine residue in the active site loop region in MET1 GGPP**C**QGFSGMNRFN by a serine residue. Site-directed mutagenesis and subsequent assembly-PCR were used to mutate the cysteine codon (TGT) to a serine codon (TCT) within the *MET1* coding sequence.

### Plant material

T1 transformants A1, A2, I1 and I2 were selected on hygromycin medium and were selfed. T2 progeny plants of each line were grown without selections and were genotyped. To differentiate between transformants that had retained or lost the *MET1* transgene, respectively, primers were designed annealing either side of an intron of the *MET1* gene. These primers amplify part of the endogenous *MET1* gene yielding a 1161bp fragment, while amplification of a part of the *MET1* cDNA transgene without the intron produces a 786bp fragment. Plant with (+) and without (-) the transgene we isolated and selfed. T3 seeds of these plant were placed on hygromycin selection to confirm that the transgene had been lost in (-) plants and to identify (+) lines that were homozygous for the transgene. One (-) plant and one (+) plant, homozygous for the transgene, were selected for each line. For transcript profiling, qRT-PCR and bisulphite sequencing analysis, three replica samples were prepared, each contained ten pooled four-week old seedlings of the T3 generation. Control plants were derived from non-transgenic seeds raised from a transformation experiment after culture of seeds on selection-free media.

### Plant analysis

Seeds were sterilised by washing in 70% ethanol for 2 minutes, soaking in 30% bleach (4.8% active hypochloride) for 10 minutes and washing 3 times with sterilised water. Sterilised seeds were sown on MS15 medium (4.4g/l Murashige and Skoog plus vitamins; 15g/l Sucrose; 1% agar; pH 5.8) and germinated under long day conditions (25oC, 16 hour photoperiod). After four weeks seedlings were harvested for molecular analysis. For flowering analysis seedling were grown on MS15 medium under long day conditions, and were transferred to soil after four weeks. For bolting analysis, leaves above 1cm in length were counted, once the primary bolt reached 1cm in height from the base of the plant.

### Sequencing and data analysis

Next generation sequencing libraries were created from mRNA using the TruSeq Stranded mRNA kit (Illumina) and sequenced on a HiSeq 2500 to generate 50 bp single end sequence data. The data was aligned to the Arabidopsis genome (TAIR web site [https://www.arabidopsis.org]) using the STAR aligner [27]. Reads mapping to each transcript were determined using the R package rsubRead [28] and pairwise comparisons between the wild type sample and each of the modified samples were performed using the R package DeSeq2 [29] to identify transcripts whose expression varied markedly between the control and experimental sample for each condition Reads were used to calculate the mean value of read mapping to a transcript in all sample in the analysis (base Mean), the change in expression between the control sample and the test sample given as a Log to the base 2 value (log2FoldChange), the standard error of variation for the log2FoldChange values in the analysis (lfcSE = log fold change Standard Error), the Wald statistic; the log2FoldChange divided by lfcSE, the probability the result is real; the log2FoldChange divided by lfcSE, compared to a standard Normal distribution to generate a two-tailed pvalue (pvalue) and the pvalue adjusted for multiple testing using the Benjamini-Hochberg test (Padj).

Raw data were submitted to the short read archive of NCBI BioProject database under SubmissionID SUB2885208, BioProject ID PRJNA395995 for the following Datasets:

**Table:** 

Accession	Sample Name	Organism	Tax ID	BioProject
SAMN07419160	WT_1	Arabidopsis thaliana	3702	PRJNA395995
SAMN07419161	WT_2	Arabidopsis thaliana	3702	PRJNA395995
SAMN07419162	WT_3	Arabidopsis thaliana	3702	PRJNA395995
SAMN07419163	A1+_1	Arabidopsis thaliana	3702	PRJNA395995
SAMN07419164	A1+_2	Arabidopsis thaliana	3702	PRJNA395995
SAMN07419165	A1+_3	Arabidopsis thaliana	3702	PRJNA395995
SAMN07419166	A1-_1	Arabidopsis thaliana	3702	PRJNA395995
SAMN07419167	A1-_2	Arabidopsis thaliana	3702	PRJNA395995
SAMN07419168	A1-_3	Arabidopsis thaliana	3702	PRJNA395995
SAMN07419169	A2+_1	Arabidopsis thaliana	3702	PRJNA395995
SAMN07419170	A2+_2	Arabidopsis thaliana	3702	PRJNA395995
SAMN07419171	A2+_3	Arabidopsis thaliana	3702	PRJNA395995
SAMN07419172	A2-_1	Arabidopsis thaliana	3702	PRJNA395995
SAMN07419173	A2-_2	Arabidopsis thaliana	3702	PRJNA395995
SAMN07419174	A2-_3	Arabidopsis thaliana	3702	PRJNA395995

### Quantitative RT-PCR assay

Gene expression was analysed using SsoFast EvaGreen supermix (BioRad) on the Fluidigm Biomark 96.96 Dynamic Array according to the manufacturer’s protocol. Data analysis was carried out utilizing the Fluidigm Gene Expression Analysis software using ACTIN 2 (*AT3G18780*) as the reference gene. Primers are listed in S8 Table.

### ChIP analysis

28-day-old seedlings were harvested and cross-linked with 1% formaldehyde. Chromatin was extracted using the ChromaFlash Plant Chromatin Extraction Kit (Epigentek) and sheared to 200-500bp fragments using a Bioruptor (Diagenode). ChIP was carried out using the EpiQuik Plant ChIP Kit (Epigentek). Input samples and immunoprecipitated samples were analysed using SsoFast EvaGreen supermix (BioRad) on the Fluidigm Biomark 96.96 Dynamic Array according to the manufacturer’s protocol. ChIP-qPCR results were first normalized with input sample. Relative enrichment was then calculated via the enrichment of the signal (antibody of interest) compared to the enrichment of the noise (negative control). Primers used for amplification are listed in S8 Table. Antibodies used for ChIP: anti-acetyl-histone H4K5K8K12K16 (06–866; Millipore), H3K4me3 (07–473, Millipore), H3K9me3 (07–442, Millipore), normal rabbit IgG (12–370, Millipore).

### Bisulphite analysis

Genomic DNA was isolated [30] and subjected to bisulfite treatment using an EZ DNA Methylation-lightning kit (Zymo Research) according to the manufacturer's instructions. Regions containing dense methylation for *At3G01345* (Chr3: 129684..129860–177 bp), *At3G27473* (Chr3: 10171884..10172090–207 bp), and *At3G30720* (Chr3: 12348994..12349109–116 bp) *AT5G34850* (Chr5: 13111304..13111574 – 271bp) were amplified by primers listed S8 Table. For each line, 10 clones were sequenced and sequences were exported into the BioEdit program [31]. Aligned sequences were saved in FASTA format and analysed by the CyMATE programme [32].

### Data analysis

The ThaleMine platform https://apps.araport.org/thalemine/begin.do was used to extract the annotation for extracted genes. DNA methylation patterns were extracted from the Neomorph platform http://neomorph.salk.edu/epigenome/epigenome.html to identify genes with dense DNA methylation patterns.

## Results and discussion

### Phenotypic changes in MET1 over-expression lines

To investigate the effects of *MET1* over-expression, *Arabidopsis* was transformed with a construct which contained the *MET1* cDNA under the control of the 35S promoter and with a second construct carrying a point mutation in the *MET1* cDNA introducing a C/S replacement in the active site loop region that renders the MET1 protein catalytically inactive [26]. For each construct, two transgenic lines were selected; lines A1 and A2 contain the cDNA encoding a catalytically active MET1, and lines I1 and I2 contain the cDNA encoding a catalytically inactive MET1. To identify heritable effects that do not require continuous presence of the *MET1* transgene, each line was selfed and plants were selected from the T2 generation that had retained the transgene (labelled ‘+’) as well as plants that had lost the transgene (labelled ‘-‘). In plants that had retained the transgene, *MET1* transcript levels were found to be increased about 3-fold in A1+ and I1+, and about 15-fold in A2+ and I1+. In lines that had lost the transgene, *MET1* transcript levels had been restored to wildtype levels (S1 Fig).

**Fig 1:**
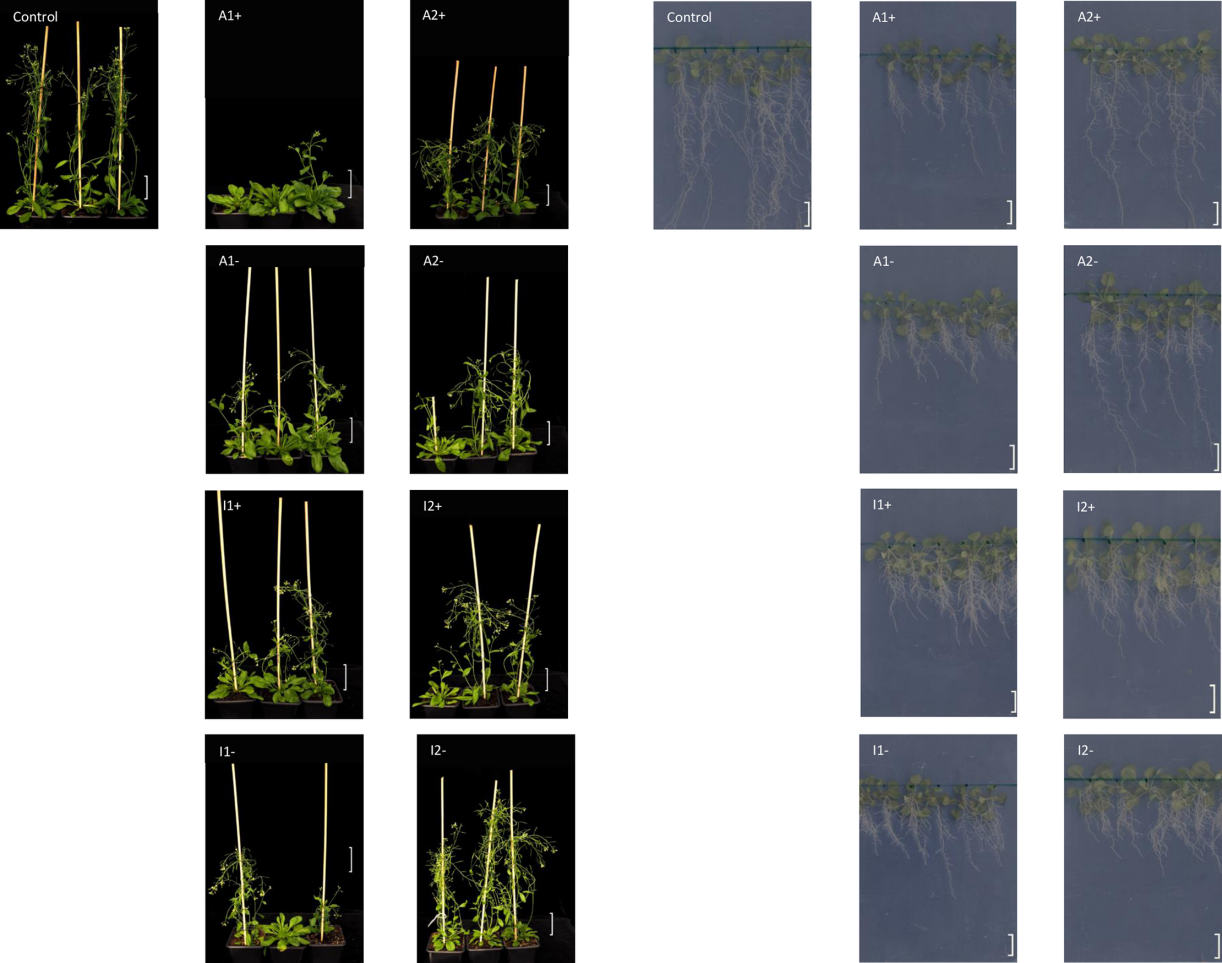
Shoot and root phenotypes in wildtype control plants, in MET1 transformants (+) and in lines derived from MET1 transformants, from which the transgene has been removed (-). Lines A1 and A2 express a catalytically active MET1 transgene, lines I1 and I2 express a catalytically inactive MET transgene. Images were taken eight weeks after stratification. The scale bar for shoot images indicates 5cm, the scale bar for root images indicates 10mm.

Among the *MET1* lines specific shoot and root phenotypes were observed (Fig 1). In all lines, primary root length was reduced (S2A Fig) and several lines showed an increase in secondary roots (S2B Fig) and a delay in bolting (S2C Fig). Similar common phenotypes were present in different lines, which were also retained in *MET1* lines that had lost the transgene, suggesting that that phenotypic changes represent heritable changes induced at common target loci. There was no direct correlation detectable between the transgene expression levels and the severity of individual phenotypes in individual lines.

We do not observe a direct quantitative correlation between the severity of individual phenotypes and the expression level of the *MET1* transgene construct. Equally, the expression changes that are observed for specific loci in in *MET1* transformants, do not occur more frequently when the *MET1* transgene is expressed more strongly. This suggests that the epigenetic changes that are induced by *MET1* over-expression are stochastic events, for which increased MET1 levels are required but not always sufficient. This might, for example, be expected if target genes are only susceptible to increased MET1 levels during a short developmental period, and if epigenetic changes not only depend on the local concentration of MET1 but also on the local concentration of proteins that interact with MET1.

A reduction in primary root length has been reported for *Arabidopsis* seedlings treated with the DNA methylation inhibitor 5‐azacytidine [33], which suggests that the phenotype is associated with cytosine hypomethylation. Among the *MET1* over-expression lines, we did not observe any defect in leaf shape or size, nor in flower structure or floral organ identity, which have been reported for *ddm1* [34] and for *MET1* antisense lines [4], but the delay in bolting resembles phenotypes observed in some mutants associated with DNA methylation pathways. Both the *HDA6* mutant *axe1-5* and *HDA6* RNAi lines display late flowering phenotypes [35]. When grown in long-day photoperiod, *ddm1-2* mutant plants also flower late [34], while they flower early under short day conditions [36]. Plants with altered MET1 functions show a range of flowering time effects. In *met1*-3 mutants, a consistent delay in flowering is observed [37], *met1-2* mutant plants exhibit normal morphology and development, and *met1-1* mutants are late flowering [38]. Demethylation of DNA via 5-azacytidine (5-azaC) treatment or via expression of a *MET1* antisense gene causes early flowering, with the promotion of flowering being directly proportional to the decrease in methylation in *MET1* antisense lines [36].

With regard to the maintenance of phenotypes in lines that had lost the *MET1* transgene, at least partial heritability of phenotypes has been reported for *MET1* antisense lines when the antisense transgene had been lost via segregation [36] and for derivatives of a *met1-1* mutant with restored wildtype MET1 levels. The at least partial transmission of the late flowering phenotype in these lines was explained by the inheritance of *fwa* epigenetic alleles activated in the *met1-1* mutant [38]. As the *met1-1* allele encodes a MET1 protein with a single aminoacid substitution, it is possible that some of the induced phenotypes are generated by changes in protein structure and interaction, which may induce similar effects as an increase in MET1 concentration.

### Expression changes in MET1 over-expression lines

To identify potential target loci for *MET1* over-expression, pools of 4-week-old T3 seedlings of lines A1+ (S1 Table), A1- (S2 Table), A2+ (S3 Table) and A2- (S4 Table) were used for transcript profiling. In each line except line A2-, the majority of genes with altered transcript profiles show an increase in expression. Applying a cut-off of a log2-fold change of 2.5, increased expression levels were observed in 644 genes in A1+, 565 genes in A1-, 22 in A2+ and 37 in A2-. Reduced expression was observed in 240 genes in A1+, 77 genes in A1-, 0 genes in A2+ and 85 genes A2-. The three major categories of genes with altered gene expression were transposable elements (S5 Table), genes expressing non-coding transcripts (S6 Table) and coding genes (S7 Table).

The majority of genes encoding transposable elements are up-regulated. An exception is the down-regulated gene *AT5G34853*, *MUSTANG 8* (MUG8), which encodes a member of a domesticated transposable element gene family MUSTANG. Members of this family are derived from transposable elements genes but gained functions in plant fitness and flower development [39]. To assess the efficiency and frequency of heritable expression changes, we compared transcript data from lines A1+ and A1-. Heritability frequencies differed among the individual categories of transposable element genes and non-coding RNA (Table 1), with high heritability levels for snRNAs (100%), snoRNA (98%), ncRNAs (82%) and pseudogene TEs (80%), and low heritability rates for CACTA-like TEs of Tnp1/En/Spm (16.7%) and Tnp2/En/Spm types (21.9%) and for Ty1-Copia-like retrotransposons (36.8%). This suggests that the transcript changes induced after *MET1* over-expression at individual genetic loci are maintained with different levels of efficiency. This resembles observations made in *met1*-*1*, *met1-3* [40] and *ddm1*-2 lines [41, 42], where induced hypomethylation of repeat sequences was either fully reversed or could be stably inherited for at least eight generations. Heritable activation in *ddm1* has, for example, been reported for the CACTA family members CAC1-CAC4 [43], [44], [41] and for LTR-retrotransposons (*ATGP3*, *ATCOPIA13*, *ATCOPIA21*, *ATCOPIA57*, *ATCOPIA93*/*EVD*) [45]. In *met1* lines, ATLANTYS2 and VANDAL21, family member show particularly high heritability levels [40].

**Table 1:** Summary of transposable elements and genes expressing non-coding RNAs with altered transcript levels and their heritability rates. Data were compiled for different categories of transposable elements (S5 Table) and genes expressing non-coding RNAs (S6 Table) that showed at least log2-fold changes of +/- 2.5 in line A1+ compared to wildtype. For each gene the values in A1+ and A1- were compared to score the heritability of expression changes.

	No of genes	Genes with heritable changes	Percentage heritable changes
**Transposable elements**			
CACTA-like transposase family (En/Spm)	3	0	0
CACTA-like transposase family (Ptta/En/Spm)	59	39	66.1
CACTA-like transposase family (Tnp1/En/Spm)	18	3	16.7
CACTA-like transposase family (Tnp2/En/Spm)	32	7	21.9
CACTA-like transposase family, putative	4	0	0
copia-like retrotransposon family (Ty1-Copia-element)	19	7	36.8
gypsy-like retrotransposon family (Athila)	59	39	66.1
gypsy-like retrotransposon pseudogene (Athila)	4	4	100
gypsy-like retrotransposon genes and pseudogenes (Athila)	63	43	68.3
gypsy-like retrotransposon family (Ty3-element)	26	16	61.5
hAT-like transposase family (hobo/Ac/Tam3)	12	9	75
Mutator-like transposase family	24	16	66.7
non-LTR retrotransposon family (LINE)	11	8	72.7
transposable element gene	64	37	57.8
transposable element gene; pseudogene, hypothetical protein	86	69	80.2
**non-coding RNAs**			
miRNAs	4	4	100
NATs	10	5	50
ncRNA	50	41	82
rRNAs	2	0	0
snoRNAs	57	56	98.2
snRNAs	8	8	100
tRNAs	2	2	100

The group of heritably up-regulated TEs in *MET1* over-expression lines overlaps with many genes activated in *met1*, *ddm1* and *hda6* mutants but do not exactly match the activation profile in any of these lines (S5 Table). This is illustrated by *AT3G02515* which is upregulated only in *met1-1*, but not in *ddm1-2* or *hda6-5*, *AT1G50735*, which is activated in *met1-1*, *ddm1-2*, and *hda6-5*, *AT3G42658*, which is upregulated in *met1-1*, *ddm1-2*, *hda6-5* and *suvh4*, *AT2G04770* and *AT5G19015*, which are jointly and additively regulated by MET1 and HAD6 [21], and *AT3G31442*, for which strong activated is only observed in *ddm1-2* [46]. Some TEs activated in *MET1* over-expression lines also deviate in their heritability levels. While, for example, Athila elements that are activated in *met1* mutants are efficiently silenced again after re-introduction of a MET1 transgene copy [40], two third of all Athila elements activated in *MET1* over-expression lines, retain this status after removal of the *MET1* transgene (Table 1).

To differentiate between potential primary and secondary targets of MET1-based epigenetic modifications, we used the methylome genome browser http://neomorph.salk.edu/ [23] to screen genes with altered transcript levels for the presence of dense methylation patterns. We identified 31 primary target candidate genes with heritable dense methylation. These genes were entered into Table 2, arbitrarily grouped into three categories, based on the presence of dense methylation in the promoter or 5’ region (upstream), in the gene region (genic) or in the genomic region into which the gene is embedded (region).

**Table 2:** List of all coding genes with heritably increased (negative log2-fold change) or reduced (positive log2-fold change) transcript levels in the A1 lines with dense cytosine methylation in all three sequence contexts (CG, CHG, CHH).

Gene ID	line	log2-fold change	pvalue	Location of dense C methylation	Annotation
**Increased transcript levels**					
*AT2G34130*	A1+	-6.343	7.37E-42	genic	MEE19 maternal effect embryo arrest 19; hypothetical protein
	A1-	-1.855	0.000285		
	A2+	-2.970	5.17E-62		
*AT3G01345*	A1+	-7.076	4.86E-76	genic	Expressed protein
* *	A1-	-6.829	5.84E-86		
* *	A2+	-0.883	6.86E-08		
*AT3G21570*	A1+	-3.451	2.47E-09	genic	proline-rich nuclear receptor coactivator
	A1-	-1.949	0.000119		
*AT3G24542*	A1+	-5.551	8.93E-28	genic	Beta-galactosidase related protein
	A1-	-4.889	3.90E-26		
*AT3G53910*	A1+	-4.682	3.88E-18	genic	Malate dehydrogenase-like protein
	A1-	-3.701	8.64E-14		
*AT4G18150*	A1+	-5.660	7.99E-34	genic	Serine/Threonine-kinase, putative
	A1-	-5.388	3.45E-37		
*AT5G15360*	A1+	-4.214	6.81E-14	genic	Transmembrane protein
	A1-	-6.021	7.80E-51		
*AT5G26270*	A1+	-3.470	2.04E-09	genic	Transmembrane protein
	A1-	-5.411	4.71E-64		
*AT5G35375*	A1+	-3.379	4.08E-09	genic	Transmembrane protein
	A1-	-2.398	2.47E-06		
*AT5G01080*	A1+	-2.550	1.39E-05	upstream/ genic	Beta-galactosidase related protein
	A1-	-4.218	5.14E-21		
*AT3G27473*	A1+	-2.984	9.32E-09	upstream	Cysteine/Histidine-rich C1 domain family protein
* *	A1-	-1.797	0.000464		
*AT3G30775*	A1+	-1.205	1.18E-08	upstream	EARLY RESPONSIVE TO DEHYDRATION 5 (ERD5); Encodes a proline oxidase, its mRNA expression induced by high levels of Al and by osmotic stress. The promoter contains an L-proline-inducible element.
	A1-	-2.719	6.01E-38		
*AT4G09430*	A1+	-2.531	2.12E-06	upstream	Disease resistance protein (TIR-NBS-LRR class) family; with Natural antisense transcript At4G09432, FUNCTIONS IN: transmembrane receptor activity, ATP binding.
	A1-	-2.721	6.51E-09		
*AT4G25530*	A1+	-10.316	7.26E-118	upstream	FLOWERING WAGENINGEN, FWA, HDG6, HOMEODOMAIN GLABROUS6
	A1-	-3.858	3.10E-14		
	A2+	-2.503	7.00E-44		
*AT5G23240*	A1+	-2.881	9.78E-30	upstream	ATDJC17, DJC76, DNA J PROTEIN C76, DNAJ heat shock N-terminal domain-containing protein
	A1-	-2.544	1.21E-20		
	A2-	-3.046	1.10E-41		
*AT5G24240*	A1+	-4.144	3.44E-14	upstream	Phosphoinositide 4-kinase PI4Kc3, Overexpression mutants display late-flowering phenotype.
	A1-	-4.524	3.52E-24		
*AT2G06904*	A1+	-5.267	1.48E-23	region	Nucleic acid / zinc ion binding protein
	A1-	-4.457	1.88E-20		
	A2+	-1.449	7.54E-17		
*AT2G07240*	A1+	-2.203	1.99E-04	region	Cysteine-type peptidase
	A1-	-3.788	2.22E-15		
*AT2G11778*	A1+	-9.461	1.53E-116	region	Transmembrane protein
	A1-	-8.981	6.08E-135		
	A2+	-2.911	2.75E-59		
*AT3G28193*	A1+	-3.823	3.92E-12	region	Transmembrane protein
	A1-	-4.477	2.58E-22		
*AT3G30720*	A1+	-4.185	2.57E-117	region	QQS qua-quine starch
* *	A1-	-3.951	2.41E-95		
* *	A2+	-0.748	6.70E-06		
*AT3G30770*	A1+	-5.097	4.01E-23	region	Eukaryotic aspartyl protease family protein
	A1-	-5.149	2.83E-31		
*AT3G31910*	A1+	-4.855	7.50E-24	region	Ulp1 protease family protein (DUF1985)
	A1-	-3.709	1.98E-15		
*AT3G42723*	A1+	-4.205	2.11E-15	region	ATP binding / aminoacyl-tRNA ligase/ nucleotide binding protein
	A1-	-2.870	5.22E-09		
*AT3G44070*	A1+	-5.709	4.19E-28	region	Glycosyl hydrolase family 35 protein
	A1-	-5.617	6.42E-37		
*AT3G44265*	A1+	-6.376	7.87E-46	region	Beta-galactosidase-like protein
	A1-	-5.866	2.31E-47		
	A2+	-0.549	8.62E-05		
*AT4G03950*	A1+	-3.246	2.27E-08	region	Nucleotide/sugar transporter family protein
	A1-	-3.250	4.93E-11		
*AT5G45570*	A1+	-4.759	5.05E-17	region	Ulp1 protease family protein
	A1-	-3.487	4.75E-13		
	A2+	-1.290	8.65E-14		
**Reduced transcript levels**					
*AT5G34850*	A1+	7.956	1.09E-105	upstream	Purple acid phosphatase 26
	A1-	7.971	6.56E-111		
**Antagonistic transcript level changes in A1+ and A1-**					
*AT3G50770*	A1+	2.816	3.50E-15	upstream	CML41, calmodulin-like 41 FUNCTIONS IN: calcium ion binding
	A1-	-0.948	0.00018459		
*AT4G00130*	A1+	2.452	6.56E-08	region	DNA-binding storekeeper protein-related transcriptional regulator
	A1-	-3.355	4.03E-63		

Several of the genes listed in Table 2 have been shown to be sensitive to DNA methylation changes. The up-regulated gene *AT4G25530*, *FLOWERING WAGENINGEN (FWA)*, is imprinted in the endosperm under the control of MET1 [47] and DDM1 [48]. Silencing is most likely mediated by transposable-element-derived tandem repeats in the promoter region [49]. In line A1-, *FWA* activation is retained, which suggest that, at least in some lines, *MET1* over-expression can induce a heritable activation. In contrast, an *FWA* allele activated in *ddm1-2*, was efficiently re-methylated and re-silenced upon restoration of the DDM1 function. Only in some rare cases, further hypomethylation and reactivation of FWA alleles could occur [41]. The up-regulated gene *AT4G03950*, which encodes a Nucleotide/sugar transporter family protein, is activated in some but not all biological replicas of 9-day-old seedlings of a *ddm1-2* mutant [49]. The up-regulated gene *AT3G30720*, *Qua-Quine Starch (QQS)* is embedded within a TE-rich region and its expression levels are increased in *met1*, *ddc* (*ddm1/ddm2/cmt3*), *ddm1* and in the *RNA-DEPENDENT RNA POLYMERASE 2 mutant rdr2*. QQS expression levels, vary considerably among natural accessions, which correlates negatively with the DNA methylation level of repeated sequences located within the 5′end of the gene. DNA methylation and expression variants can be inherited for several generations. [50].

A large number of the genes with dense methylation marks and altered expression in *MET1* over-expression lines, also show modified expression profiles in the *hda6* mutant *axe1–5*. The upregulated gene *AT3G30720* is also upregulated in *axe1–5* and the downregulated gene *AT5G13170* is also downregulated in *axe1–5* [51]. *HDA6* regulates cold acclimation under low temperature condition. Ten of the genes *activated in MET1 over-expression lines* (*AT3G01345*, *AT3G27473*, *AT3G30770*, *AT3G44070*, *AT5G01080*, *AT5G15360*, *AT5G24240*, *AT5G26270*, *AT5G35375* and *AT5G45570*), are upregulated in *axe1–5* after cold treatment [51].

Two genes listed in Table 2 are regulated by DNA methylation.The up-regulated gene *AT3G50770*, *calmodulin-like 41 (CML41*,*)* contains transposon promoter insertions [52]. Its increased expression, in response to elevated temperature, correlates with reduced promoter DNA methylation [53]. The down-regulated gene *AT3G18610*, *nucleolin like 2 (NOR2)*, is involved in epigenetic regulation, as its disruption induces rDNA hypermethylation [54].

### Epigenetic changes in selected target genes

**Fig 2:**
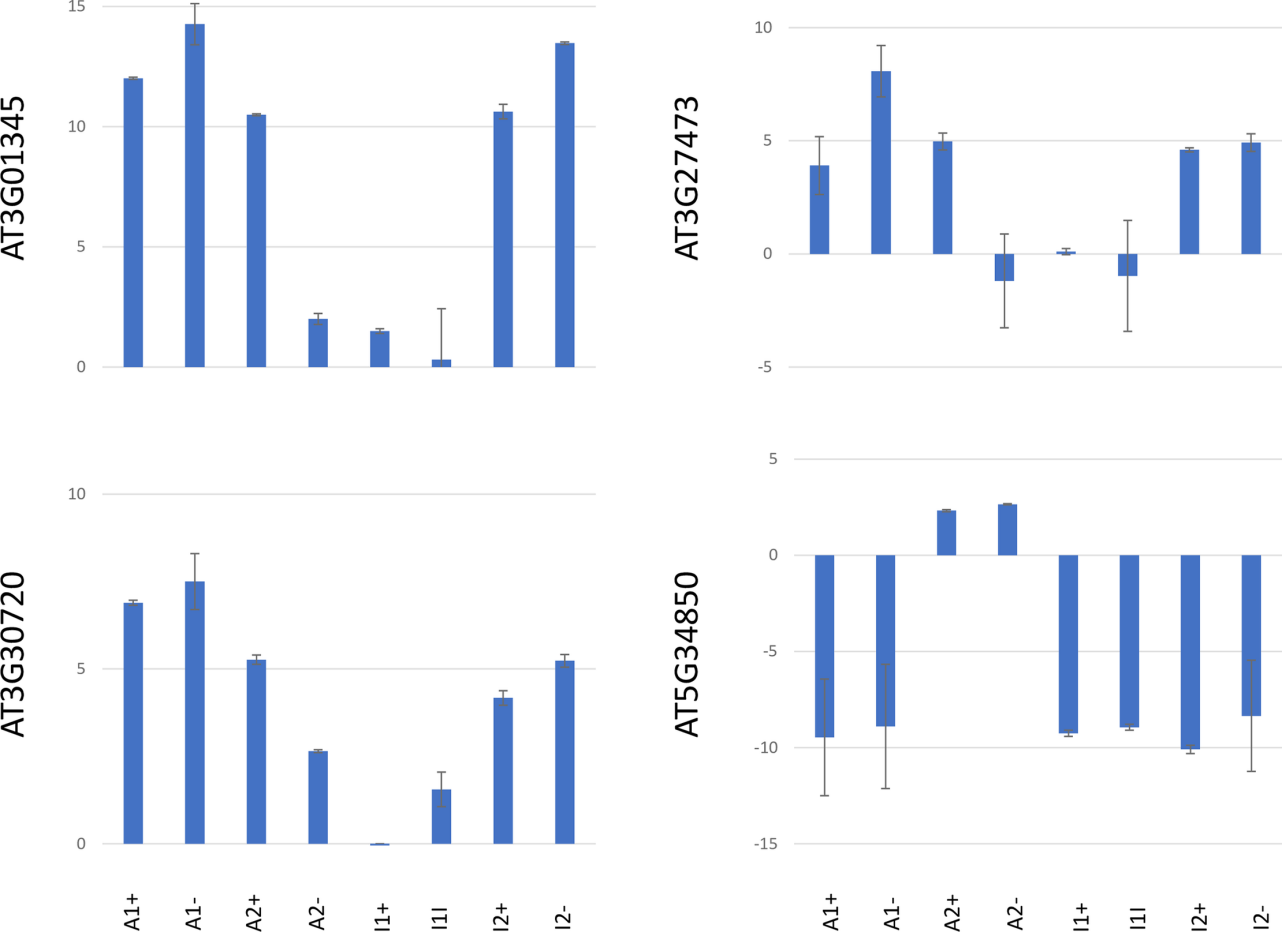
RT-PCR analysis of four genes with dense methylation in MET1 transformants with (+) and without the transgene (-). Lines A1 and A2 express a catalytically active MET1 transgene, lines I1 and I2 express a catalytically inactive MET transgene. The mean and the standard error are shown for three biological replicates each tested in three technical replicates. Values on the y-axis represent the log2-fold-difference compared to the control line.

We selected four genes from Table 2 for further analysis of expression changes and epigenetic features. We selected three genes with increased transcript levels that contained dense DNA methylation marks in the upstream region (*At3G27473)*, in the genic region *(At3G01345)* or in the chromosomal environment (*At3G30720)*, respectively, and one gene with reduced transcript levels and dense methylation in the upstream region (*At5G34850*). For q-RTR analysis, transcript samples were prepared from T3 seedling pools for all eight *MET*-overexpression lines. Similar to the observed phenotypes, expression changes of the four analysed genes occur independently of expression levels, catalytic activity or conservation of the *MET1* transgene. Within individual lines, expression changes occur stochastically and with different intensity, inducing an increase in expression for all genes except *At5G34850*, which displays a significant reduction in expression in six out of eight *MET1-*overexpression lines. In most *MET1*- overexpression lines that have lost the transgene, expression changes are conserved (Fig 2).

**Fig 3:**
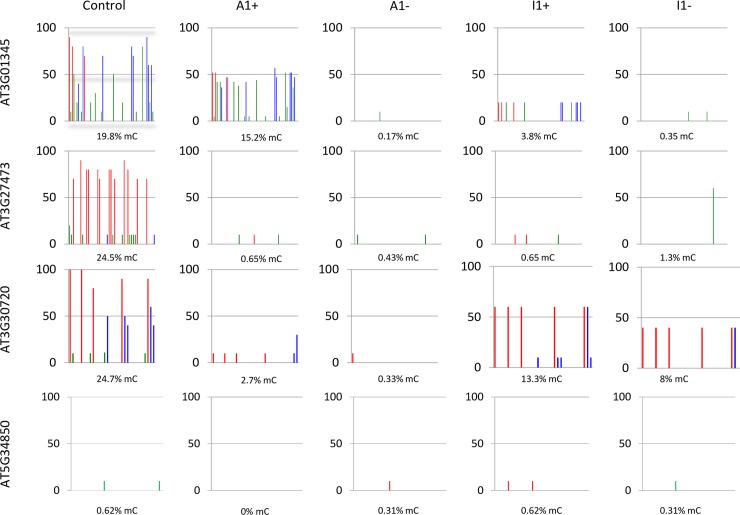
DNA methylation analysis of regions (S3 Fig) of genes *AT3G01345*, *AT3G27473*, *AT3G30720*, *AT5G34850* in *MET1* transformants (+) and in lines derived from *MET1* transformants, from which the transgene has been removed (-). Lines A expresses a catalytically active *MET1* transgene, line I1 expresses a catalytically inactive *MET* transgene. Red bars denote CG methylation, blue bars CHG methylation and green bars CHH methylation.

To investigate if expression changes in the four genes were associated with epigenetic changes, we compared cytosine methylation and histone marks in wildtype and *MET1* over-expression lines. Bisulphite sequencing analysis of densely methylated regions (S3 Fig) identified a reduction or loss of methylation marks for all three genes, independent of the expression levels of the three activated genes in different lines (Fig 3). This suggests that *MET1* overexpression induced heritable hypomethylation at these loci, which was, however, not in all cases sufficient to increase gene expression. The analysis of the silenced gene *At5G34850*, turned out to be more complicated. PCR-analysis of the locus (S4 Fig) revealed that the upstream region of the gene, which contains multiple repetitive elements, had been deleted or rearranged in all six lines, in which the gene had been silenced. Moreover, a central region of *At5G34850* also could not be amplified in lines A1+ and A1-, suggesting extensive rearrangement of the locus in the six lines that may be the result of transposon activity. Bisulphite analysis of the 5’ region of the gene, which had been retained in all eight lines, did not give any indication for DNA methylation changes (Fig 3).

**Fig 4:**
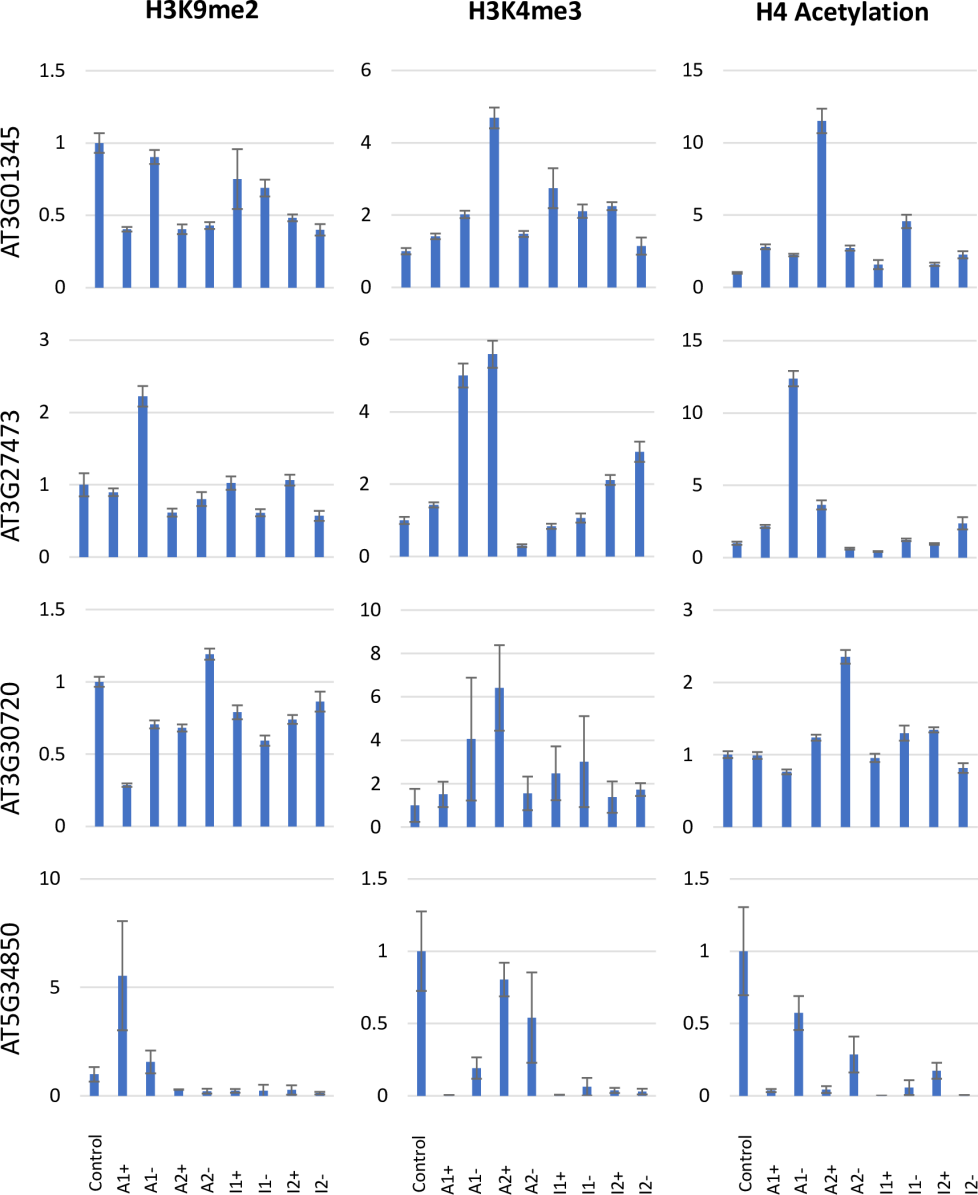
ChIP analysis of genes *At3G27473*, *At3G01345*, *At3G30720* and At5G34850 for H3K9me2, H3K4me3 and H4 acetylation marks. The means and the standard errors are shown for three biological replicates each tested in three technical replicates. Values on the y-axis represent the fold-difference of histone mark levels compared to the control line.

To investigate if expression changes correspond to changes in specific histone marks, we compared histone 4 acetylation and histone 3 methylation (H3K9me2, H3K4me3) marks of *At5G34850*, *At3G01345*, *At3G27473* and *At3G30720* in wildtype and in the eight *MET1* over-expression lines (Fig 4). H3K9me2 levels were moderately reduced for *AT3G01345* in most lines and H4 acetylation levels were increased in some lines for *AT3G01345*, *AT3G27473* and *AT3G30720*. Among the three histone marks tested, H3K4me3 levels show the most significant changes. While there was no uniform correlation between expression changes and individual H3K4me3 marks, some locus-specific correlations were detectable. Increased H3K4me3 levels correlated in all *MET1* overexpression lines with enhanced *At3G27473* expression, and in seven out of eight *MET1* overexpression lines with enhanced expression of *At3G01345*. In the six line with reduced expression of *At5G34850* H3K4me3 levels are also significantly reduced. As in all six lines, this reduction correlates with deletions and/or rearrangements of the locus, it is unclear if silencing of *At5G34850* is the consequence of H3K4me3 reduction or of the loss of upstream regions that are required for gene expression. It also unclear if H3K4me3 reduction is causally linked to DNA rearrangements or expression changes.

The expression analysis had identified genetic loci for which the presence of the *MET1* transgene was not required to maintained expression changes in *MET1* over-expression lines. This suggests that for certain loci epigenetic changes that alter gene expression, once they had been induced by enhanced *MET1* expression, are inherited without the need for increased *MET1* levels. This does, however, not exclude the possibility that in lines that have maintained the *MET1* transgene, enhanced *MET1* levels continuously induce new epigenetic changes. The expression analysis in T3 populations (Fig 2) had shown no indication for a specific enhancement of expression changes in *MET1* over-expression lines that had retained the *MET1* transgene. Such effects may, however, be obscured by the stable propagation of initial *MET1*-induced epigenetic states, and might become more easily detectable in later generations, especially at loci with semi-stable epigenetic changes. If the presence of the *MET1* transgene favours the induction of new epigenetic changes, expression changes at loci with semi-stable epigenetic states would be expected to revert to wildtype levels in progeny of *MET1* over-expression lines that have lost the transgene but could be re-established in progeny that has retained the transgenes.

**Fig 5:**
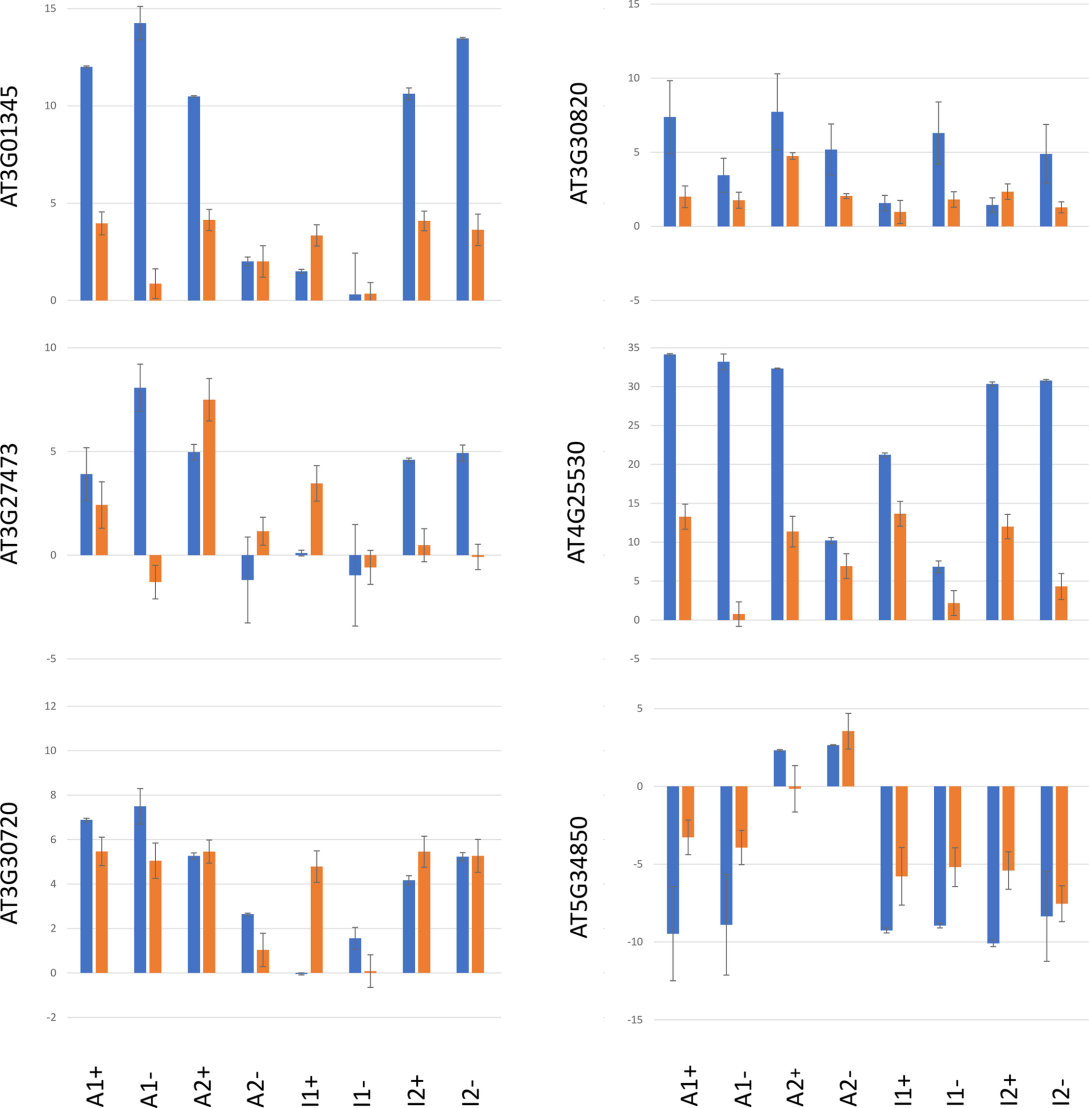
Comparison of expression profiles of genes *AT3G01345*, *AT3G27473*, *AT3G30720*, *AT3G30820*, *AT4G25530 and AT5G34850* in MET1 lines. T3 seeds are labelled in blue, T4 seeds are labelled in orange.

To investigate this option and to test the long-term stability of *MET1*-induced expression changes, we compared the expression profiles of six genes in the T3 and T4 generation (Fig 5). In most lines, enhanced expression of genes observed in the T3 generation, is also detectable in the T4 generation, although at a lower levels. In a few lines, enhanced expression is restored to wildtype levels in the T4 generation. A comparison of the four (-) lines that have lost the *MET1* transgene, suggests locus-specific differences in the efficiency of maintaining expression levels, with enhanced states being preserved for *At3G30720* but reset for *At5G34850*. This corresponds to previous reports about locus-specific differences in the maintenance of epigenetic changes [55] [56]. For all genes except *At3G30720*, the analysis implies that enhanced expression can be maintained in the T4 generation at a reduced level, with a tendency to be reset to the original levels over subsequent generation. The stable epigenetic state of *At3G30720* confirms reports about a *ddm1*-derived hypomethylated epiallele of *At3G30720* that was inherited for least eight generations [50]. In some lines, enhanced expression levels are higher in the T4 generation than in the T3 generation. This applies to *At3G30720* in lines I1+ and I2+, *At3G27473* in lines A2+ and I2+ and *At3G01345* in line I1+ (Fig 5). All lines have retained the MET1 transgene, which suggests that epigenetic changes can be continuously induced in lines that have retained increased *MET1* expression.

**Fig 6:**
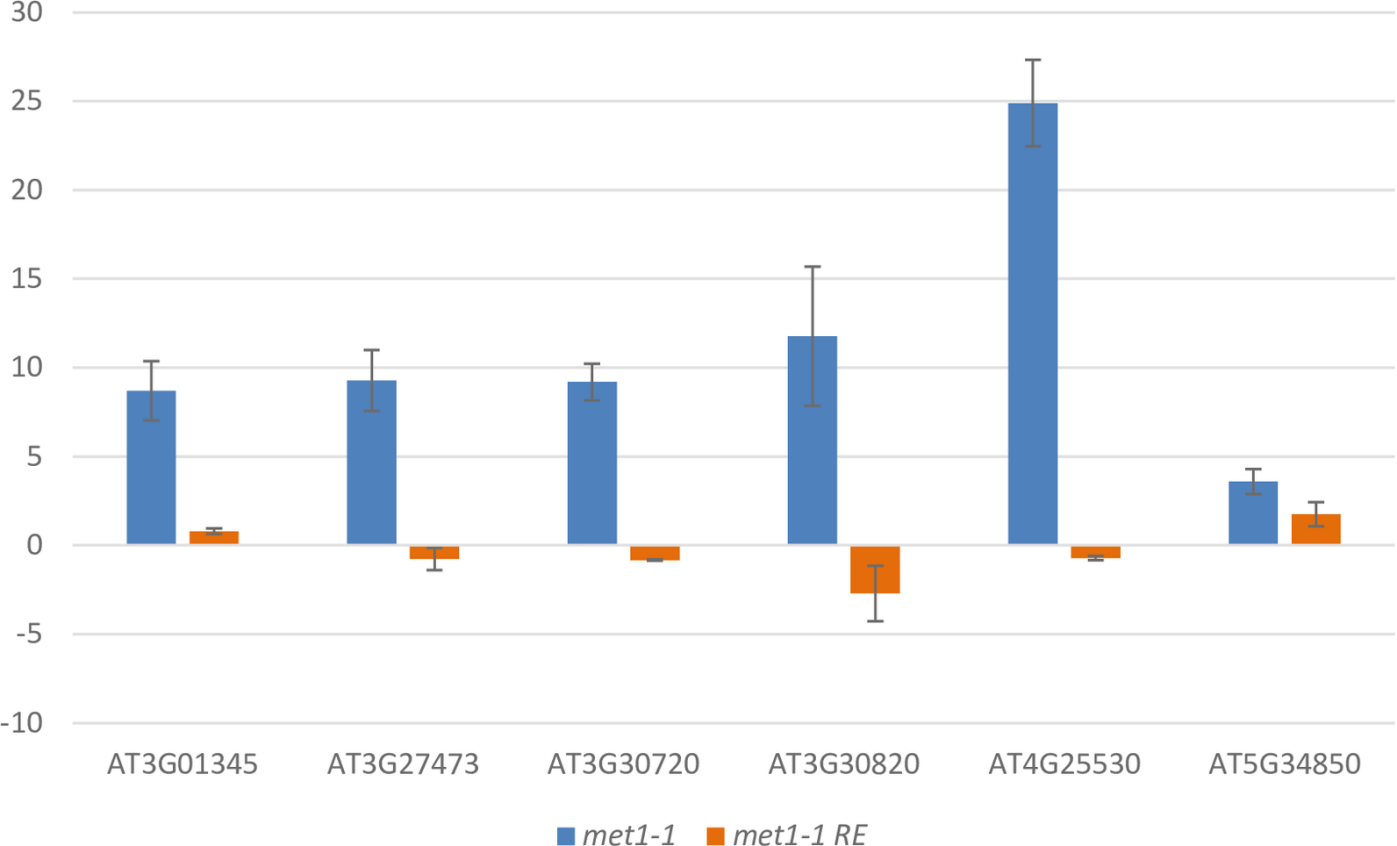
Comparison of expression profiles of genes *AT3G01345*, *AT3G27473*, *AT3G30720*, *AT3G30820*, *AT4G25530 and AT5G34850* in the *met1-1* mutant and *met1-1 RE*. The mean and the standard error are shown for three biological replicates each tested in three technical replicates. Values on the y-axis represent the fold-difference compared to the control line.

To compare the effects of *MET1* over-expression with *MET1* mutation, we examined the expression of the six genes in a *met1-1* mutant and in a met1 derived line *met1-1RE*, from which the *met1* mutant alleles had been replaced by *MET1* wildtype alleles. No significant expression changes were observed in *met1-1* or *met1-1RE* for *AT5G34850*, the locus that had been rearranged in some MET1 over-expression lines. The five genes, however, that had shown increased expression in *MET1* over-expression lines were also more highly expressed in the *met1-1* mutant. This suggests that all five genes respond in a similar way to an increase and to a reduction in MET1 levels. Enhanced expression of all five genes in *met1-1* was reversible as wildtype expression levels were restored in *met1-1RE* (Fig 6).

Our data show that *MET1* over-expression can be employed to induce epigenetic changes, with enhanced *MET1* expression levels being required but not always sufficient to induce epigenetic changes. There is not direct correlation between the level of enhanced *MET1* expression and the efficiency of the induction of epigenetic changes. This implies that recombinant MET1 proteins do not act like a transcription factor or like any other concentration-dependent gene regulator. *MET1* over-expression acts stochastically but not randomly as it induces similar changes in epigenetic and expression states at specific target loci in different MET1-overexpression lines. This resembles position-effect-variegation effects where epigenetic changes also occur stochastically but with defined probability for individual loci [57].

As increased transcript levels are stable in *MET1* over-expression lines (S1 Fig) and as there is no indication that enhanced MET1 protein levels are subject to degradation (S5 Fig), MET1 over-expression does not generate co-suppression or protein degradation effects that would resemble a *met1* mutant. Yet, some of the genes with altered expression in *MET1* over-expression lines, have also been reported to be affected in *met1* mutants, while other genes with altered expression match genes with modified expression in *ddm1* and *hda6* mutants. Increased expression correlated with hypomethylation and with an increase in H3K4me3 marks, which may occur either as a consequence of hypomethylation or due to an interaction of MET1 with histone modifier proteins like HDA6. Changes in MET1 levels may affect the stability of complexes to which MET1 and histone modifier functions contribute, altering the epigenetic state of target loci like some transposable elements, which are jointly activated in *met1* and *hda6* mutants, correlating with H3K4 methylation levels [21].

Alternatively an increase in MET1 levels may cause epigenetic changes at loci that are controlled by histone modification without a direct involvement of MET1, if the activity of binding partners like HDA6 [21] is altered by their interaction with MET1, and if this impairs the regulation of the target loci of the binding partner. Stochiometric imbalances can sequester complex partners and disrupt a multiprotein complex into non-functional subassemblies. One of the earliest examples demonstrating this effect is the over-expression of either histone H2A-H2B or histone H3-H4 gene pairs in yeast, which causes aberrant chromosome segregation [58] and which alters transcription due to disturbance of the histone octamer [59] [60]. If a protein with a catalytic function is involved in a multi-protein interaction, over-expression of a catalytically inactive version of the protein is sufficient to disturb interactions with binding partners [61].

While the mechanisms involved in *MET1* over-expression remain unclear, our data show that *MET1* over-expression offers a new strategy to induce variants with novel combinations of epi-alleles. Selective MET1 over-expression may be used to limit epigenetic changes to certain tissue types and potentially to distinct MET1 target loci, which will be especially relevant in species where the induction of epigenetic changes in all plant tissues creates unfavourable phenotypes or lethal effects. Spatial and temporal over-expression of MET1, also offers the opportunity to test if target loci alter their susceptibility to *MET1* over-expression in different tissue types, developmental stages and/or under specific growth or stress conditions.

## Conclusions

Epigenetic states contribute to the variation in gene expression and phenotypes in plants. A temporary increase in levels of DNA methyltransferase MET1 induces heritable epigenetic changes at specific loci. Over-expression of *MET1* provides a new tool to generate novel epi-alleles, and to identify and analyse epigenetic target loci and phenotypes. *MET1* over-expression serves as a proof-of-concept study that should stimulate a wider application of over-expressing epigenetic regulator genes to examine the significance and targets of epigenetic regulation in different species.

## Supporting information

S1 Fig Comparison of *MET1* expression levels in wildtype, in *MET1* transformants (+), in lines derived from *MET1* transformants, from which the transgene has been removed (-). In A1+ and I2+, MET1 expression is about 3-fold higher compared to wildtype. In A2+ and I1+, MET1 levels increase are about 15-fold compared to wildtype.
(PDF)

S2 Fig Phenotypic analysis of MET1 transformants with (+) and without the transgene (-). A) Primary root length at four weeks of development. B) Number of secondary roots greater than 2mm per mm of primary root length, at four weeks of development. C) Bolting time was analysed by counting the number of basal rosette leaves upon bolting. The parameter used to determine when bolting had occurred was defined, as the stem reaching a minimum of 1 cm in vertical height, for a basal rosette leaf to be counted in the study the leaf had to be at least 1 cm in length and 0.5cm in width. The significance of a change from wildtype is indicated by asterisks (if present): * = P <0.05, ** = P<0.01 *** = P<0.005, calculated by Student’s two-tailed t-test.
(PDF)

S3 Fig Genes with dense DNA methylation patterns in the genic region (AT3G01345), in the upstream region (AT3G27473 and AT5G34850) and in the gene and its surrounding region (AT3G30720). Boxes label sections that were analysed by bisulphite sequencing (Fig 3).
(PDF)

S4 Fig Deletions upstream of *AT5G34850* in MET1 transformants. A) Region of the *AT5G34850* locus, which was mapped using four different primer pairs (Pp1-Pp4). B) PCR analysis of *AT5G34850* regions in *MET1* transformants (+) and in lines derived from *MET1* transformants, from which the transgene has been removed (-). A lines express a catalytically active *MET1* transgene, I lines express a catalytically inactive MET transgene. Actin was used as an internal reference for DNA concentrations. Lack of PCR fragments in some lines indicates absence of at least one of the primer pairs.
(PDF)

S5 Fig Analysis of a recombinant FLAG-tagged MET1 shows no indication for MET1 instability. To assess if increasing the amount of MET1 protein induced protein degradation, a Western blot was carried out for a 35S-*FLAG-MET1* transformant and a wild type control. The expected size of the FLAG-tagged MET1 protein is 176 kDa. Actin (40 kDa) was used as an internal control for protein concentration. An unspecific ~50kDa protein is present in both samples.
(PDF)

S1 Table List of genes with altered transcript levels in line A1+. (PDF)

S2 Table List of genes with altered transcript levels in line A1-. (PDF)

S3 Table List of genes with altered transcript levels in line A2+. (PDF)

S4 Table List of genes with altered transcript levels in line A2-. (PDF)

S5 Table List of transposable elements with at least log2-fold increases (negative log2-fold change) or decreases (positive log2-fold change) of 2.5 in at least one of the four lines A1+, A1-, A2+ or A2-. (PDF)

S6 Table List of non-coding RNAs with at least log2-fold increases (negative log2-fold change) or decreases (positive log2-fold change) of 2.5 in at least one of the four lines A1+, A1-, A2+ or A2-. (PDF)

S7 Table List of coding genes at least log2-fold increases (negative log2-fold change) or decreases (positive log2-fold change) of 2.5 in at least one of the four lines A1+, A1, A2+ or A2-. (PDF)

S8 Table List of primers. (PDF)
