# Supplementary figures and images for: Induction of epigenetic variation in Arabidopsis by over-expression of DNA METHYLTRANSFERASE1 (MET1)
Source: PLoS One. 2018 Feb 21;13(2):e0192170. doi: 10.1371/journal.pone.0192170 (PMC5821449; doi:10.1371/journal.pone.0192170)

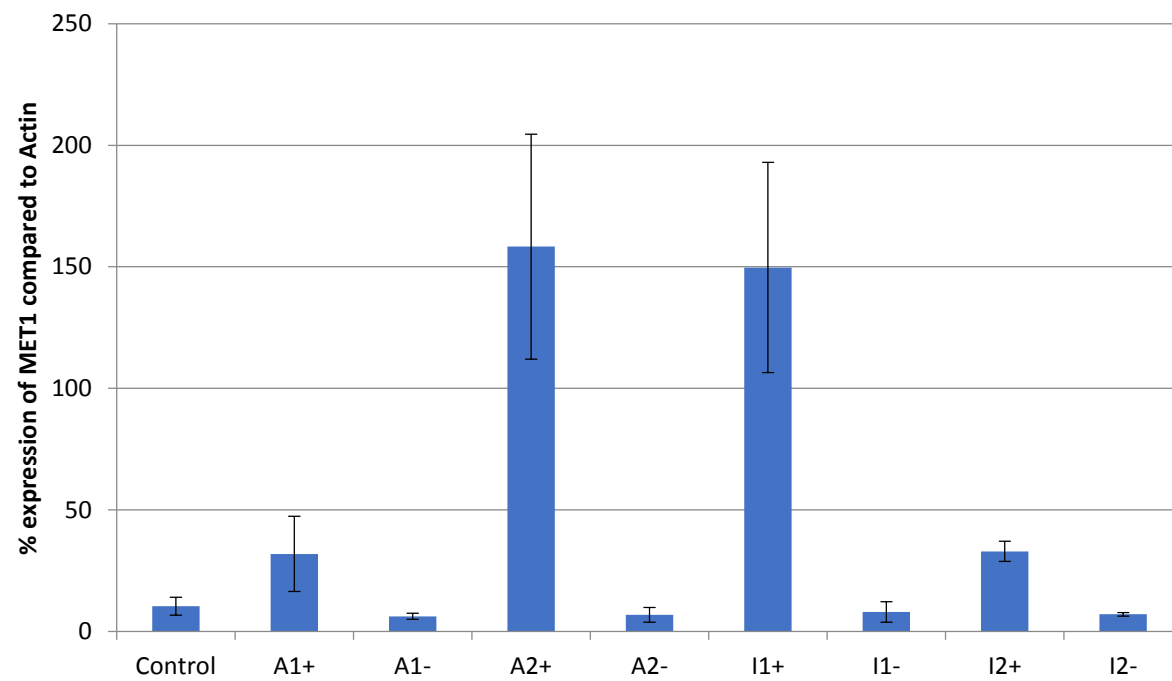

Supplement: S1 Fig — In A1+ and I2+, MET1 expression is about 3-fold higher compared to wildtype. In A2+ and I1+, MET1 levels increase are about 15-fold compared to wildtype. (PDF) [file pone.0192170.s001.pdf]

A

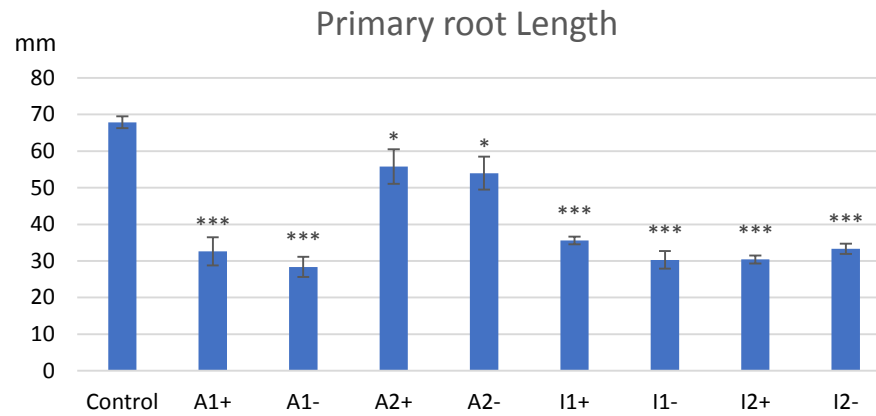

B

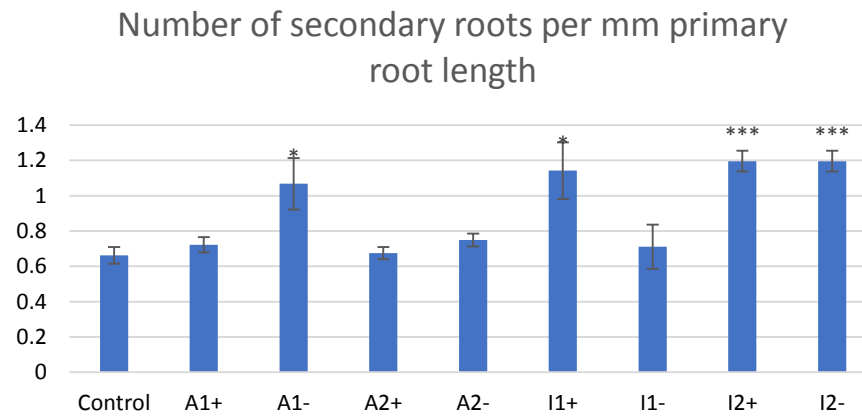

C

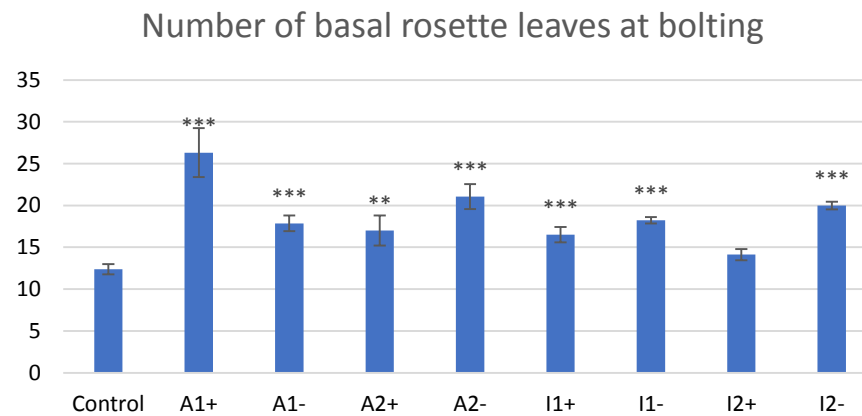

Supplement: S2 Fig — A) Primary root length at four weeks of development. B) Number of secondary roots greater than 2mm per mm of primary root length, at four weeks of development. C) Bolting time was analysed by counting the number of basal rosette leaves upon bolting. The parameter used to determine when bolting had occurred was defined, as the stem reaching a minimum of 1 cm in vertical height, for a basal rosette leaf to be counted in the study the leaf had to be at least 1 cm in length and 0.5cm in width. The significance of a change from wildtype is indicated by asterisks (if present): * = P <0.05, ** = P<0.01 *** = P<0.005, calculated by Student’s two-tailed t-test. (PDF) [file pone.0192170.s002.pdf]

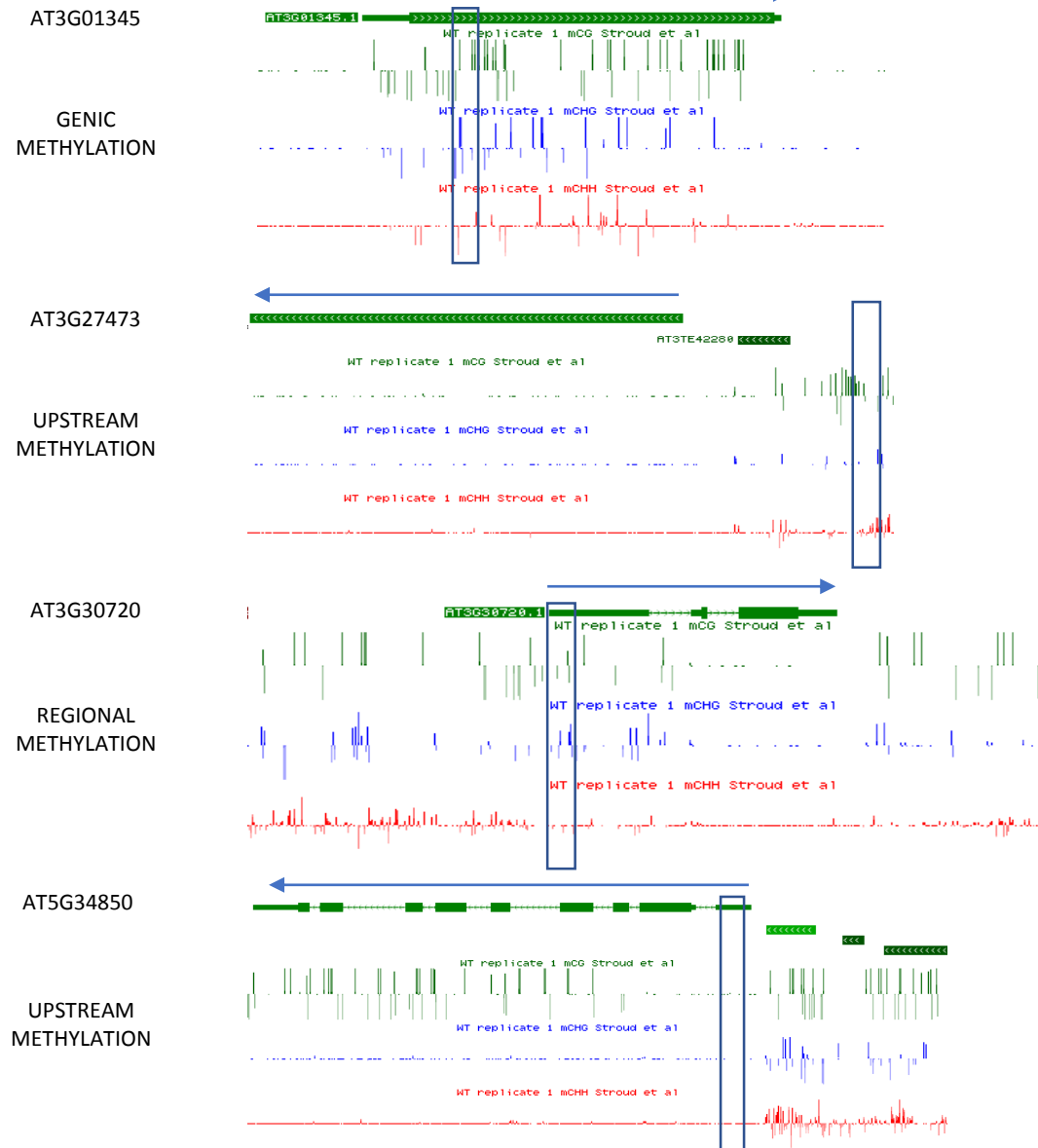

Supplement: S3 Fig — Boxes label sections that were analysed by bisulphite sequencing (Fig 3). (PDF) [file pone.0192170.s003.pdf]

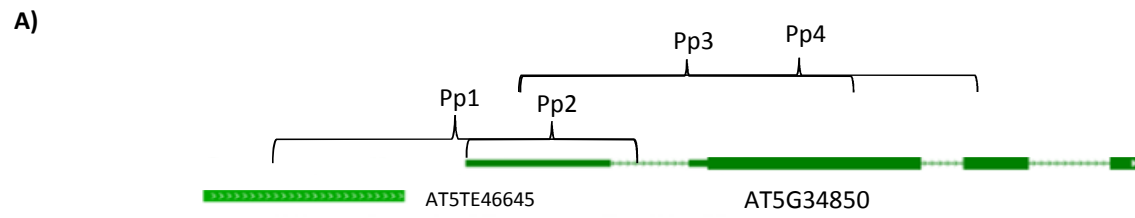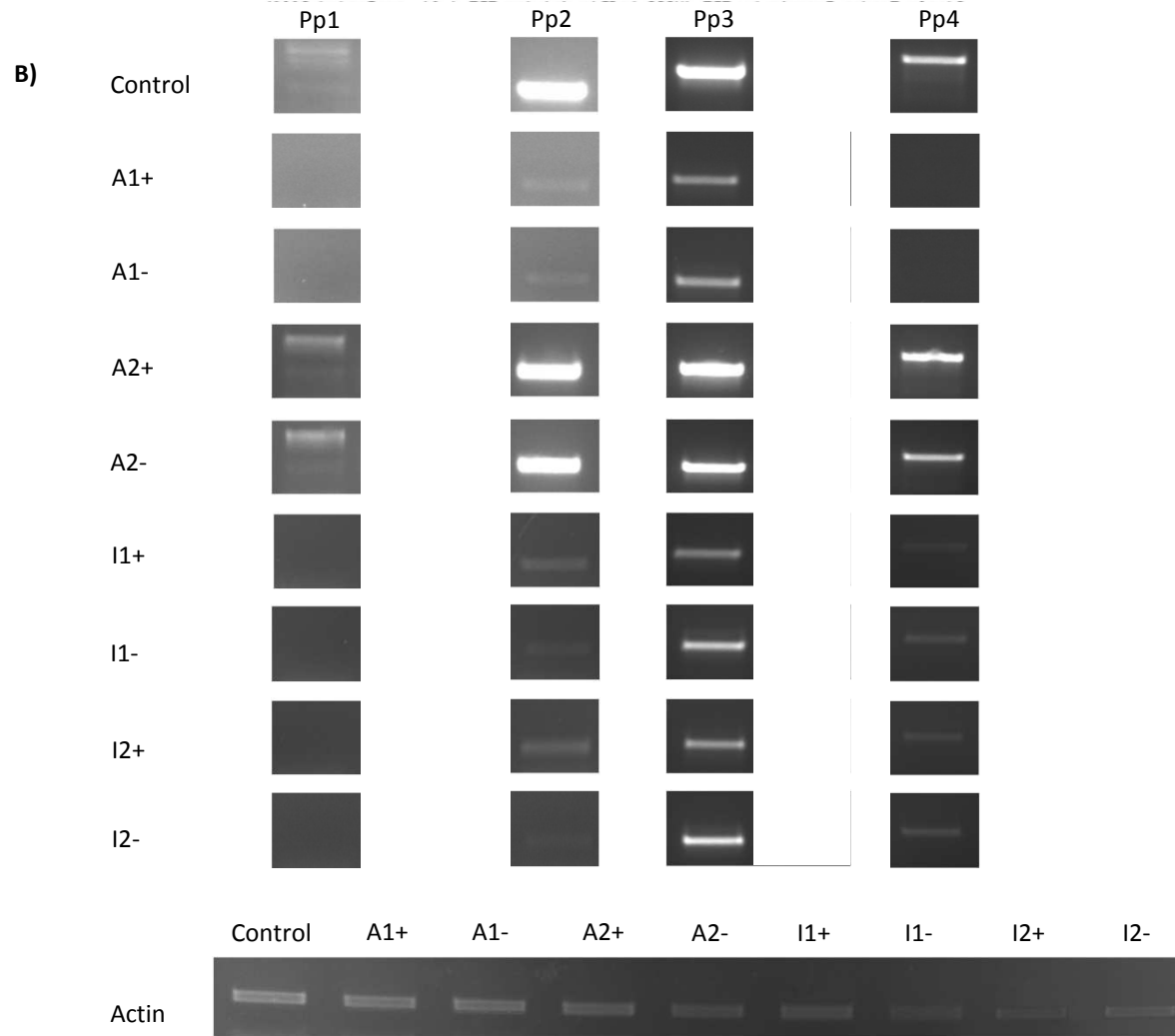

Supplement: S4 Fig — A) Region of the AT5G34850 locus, which was mapped using four different primer pairs (Pp1-Pp4). B) PCR analysis of AT5G34850 regions in MET1 transformants (+) and in lines derived from MET1 transformants, from which the transgene has been removed (-). A lines express a catalytically active MET1 transgene, I lines express a catalytically inactive MET transgene. Actin was used as an internal reference for DNA concentrations. Lack of PCR fragments in some lines indicates absence of at least one of the primer pairs. (PDF) [file pone.0192170.s004.pdf]

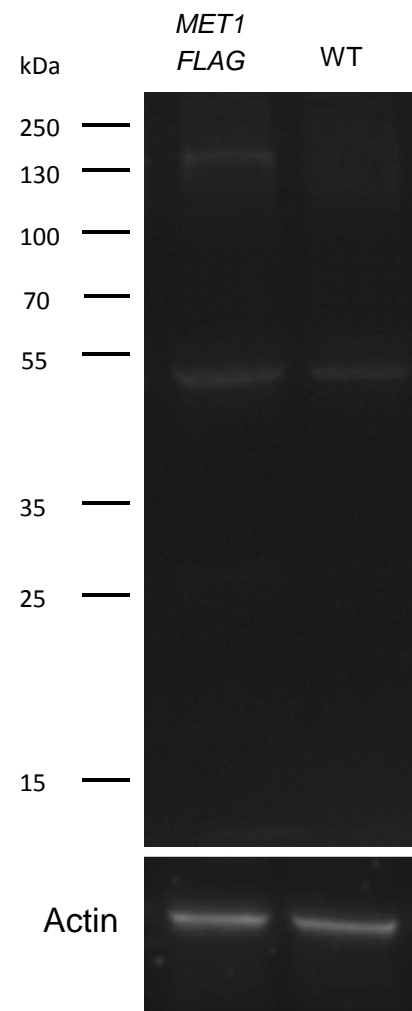

Supplement: S5 Fig — To assess if increasing the amount of MET1 protein induced protein degradation, a Western blot was carried out for a 35S-FLAG-MET1 transformant and a wild type control. The expected size of the FLAG-tagged MET1 protein is 176 kDa. Actin (40 kDa) was used as an internal control for protein concentration. An unspecific ~50kDa protein is present in both samples. (PDF) [file pone.0192170.s005.pdf]
