# Supplementary material for: Induction of epigenetic variation in Arabidopsis by over-expression of DNA METHYLTRANSFERASE1 (MET1)
Source: PLoS One. 2018 Feb 21;13(2):e0192170. doi: 10.1371/journal.pone.0192170 (PMC5821449; doi:10.1371/journal.pone.0192170)
Supplement: S1 Table — (PDF) [file pone.0192170.s006.pdf]

S1 Table: List of genes with altered transcript levels in line A1+

	baseMean	log2FoldChange	lfcSE	stat	pvalue	padj	control 1	control 2	control 3	A1+ 4	A1+ 5	A1+ 6
AT1G42050	2313.88	-10.64797595	0.374367	-28.4426	6.02E-178	3.09E-174	0.640149	0	0.694589	12.4897	11.99814	11.9812
AT2G11780	2589.918	-10.32601037	0.356584	-28.9581	2.22E-184	1.42E-180	0.640149	1.06174	1.513627	11.77496	12.59233	12.51647
AT4G25530	983.3379	-10.31630517	0.44697	-23.0805	7.26E-118	2.12E-114	0	0	0	0	11.17218	11.35043
AT2G11778	758.0734	-9.461332801	0.412285	-22.9485	1.53E-116	3.01E-113	0	0	0	10.85072	10.50153	10.29354
AT3G06465	1978.681	-9.439225726	0.409282	-23.0629	1.09E-117	2.55E-114	1.082007	1.395705	0.694589	12.76321	11.49137	10.99486
AT3G30620	1192.134	-9.287246728	0.354998	-26.1614	7.31E-151	2.68E-147	1.419792	1.06174	1.161466	11.6097	10.88591	11.05962
AT1G67105	1941.538	-8.814609957	0.231995	-37.9948	0.00E+00	0.00E+00	3.537458	2.419482	2.413855	11.97572	11.88737	11.89818
AT4G06566	984.5216	-8.673823152	0.373822	-23.2031	4.24E-119	1.09E-115	1.082007	2.091646	0.694589	11.4891	10.8102	10.27038
AT1G42040	397.1412	-8.64726995	0.426366	-20.2813	1.88E-91	2.54E-88	0	0	0	9.933734	9.533458	9.381005
AT1G40310	396.2297	-8.49382704	0.437181	-19.4286	4.42E-84	4.73E-81	0	0	0	10.11868	9.153175	9.449617
AT3G43862	489.4929	-8.421276414	0.420798	-20.0127	4.27E-89	5.49E-86	0.640149	0	0.694589	10.52402	9.470914	9.563547
AT4G08091	385.4899	-8.405287875	0.400963	-20.9627	1.44E-97	2.05E-94	0	1.06174	0	9.718646	9.32622	9.697625
AT3G46487	439.5033	-8.217801045	0.375059	-21.9107	2.05E-106	3.52E-103	1.419792	0	1.161466	9.434343	9.904985	9.946192
AT4G06485	674.2271	-8.171029262	0.427674	-19.1057	2.26E-81	2.15E-78	0.640149	1.06174	1.161466	11.22906	9.461046	9.876972
AT1G40121	288.464	-8.13015732	0.442249	-18.3837	1.78E-75	1.20E-72	0	0	0	8.577394	9.446117	9.353722
AT4G06748	228.014	-8.030337014	0.434207	-18.4942	2.30E-76	1.74E-73	0	0	0	8.829096	8.712195	8.956749
AT3G30836	252.4687	-8.026060757	0.441515	-18.1785	7.65E-74	4.79E-71	0	0	0	9.052569	8.506912	9.283167
AT3G30170	266.5284	-7.923117705	0.409314	-19.357	1.78E-83	1.83E-80	0.640149	0	0.694589	9.293513	8.862188	8.989026
AT5G33389	311.7158	-7.879526329	0.427783	-18.4195	9.17E-76	6.55E-73	0	0.626414	0.694589	8.55808	9.379578	9.695454
AT4G06615	202.6354	-7.792073358	0.443981	-17.5505	5.90E-69	3.52E-66	0	0	0	8.981856	8.35535	8.591379
AT4G06698	223.9999	-7.726582487	0.411031	-18.798	7.84E-79	6.71E-76	1.082007	0	0	8.717459	8.72049	8.97477
AT4G03910	237.4061	-7.71361368	0.459098	-16.8017	2.37E-63	1.05E-60	0	0	0	7.97503	9.177483	9.221112
AT1G39110	288.1867	-7.655498912	0.457105	-16.7478	5.88E-63	2.56E-60	0	0.626414	0	7.97503	9.475822	9.584822
AT3G42720	333.0891	-7.654669261	0.45144	-16.9561	1.73E-64	8.41E-62	0.640149	0.626414	0	10.18943	8.695459	8.708006
AT2G03965	227.8225	-7.625605269	0.399321	-19.0964	2.70E-81	2.48E-78	0.640149	0.626414	0.694589	8.812891	8.977427	8.69502
AT4G06622	176.4488	-7.618140242	0.447345	-17.0297	4.95E-65	2.44E-62	0	0	0	8.104931	8.712195	8.519541
AT4G03860	310.6504	-7.591195362	0.450615	-16.8463	1.12E-63	5.12E-61	0.640149	0	0.694589	10.05581	8.428064	8.835761
AT1G49090	299.5863	-7.539912221	0.436738	-17.2641	8.76E-67	4.79E-64	1.082007	0.626414	0	8.148628	9.542844	9.587167
AT3G30610	184.304	-7.498788266	0.437151	-17.1538	5.89E-66	3.15E-63	0	0.626414	0	8.839799	8.086794	8.563072
AT1G40230	145.7034	-7.400103041	0.451208	-16.4006	1.89E-60	7.97E-58	0	0	0	8.464361	8.073902	7.991862
AT2G14180	275.7196	-7.39698779	0.425291	-17.3928	9.36E-68	5.47E-65	1.419792	0	0.694589	9.755854	8.678527	8.57257
AT5G32404	170.4021	-7.395169265	0.461545	-16.0226	8.88E-58	3.11E-55	0	0	0	8.967288	7.923931	8.139753

AT3G30695	231.5052	-7.392886409	0.425585	-17.3711	1.37E-67	7.63E-65	0	1.06174	0.694589	9.409413	8.40766	8.534198
AT1G20400	183.2095	-7.293200594	0.453012	-16.0994	2.58E-58	9.33E-56	0.640149	0	0	7.697535	8.678527	8.920019
AT3G43863	206.7371	-7.278904671	0.424544	-17.1452	6.82E-66	3.58E-63	0.640149	0	1.161466	9.209354	8.333883	8.353172
AT3G30837	164.3579	-7.263472645	0.425416	-17.0738	2.32E-65	1.17E-62	0	1.06174	0	8.471261	7.994038	8.563072
AT2G06720	158.1724	-7.211839279	0.447919	-16.1008	2.52E-58	9.25E-56	0	0	0.694589	8.694056	8.333883	7.745439
AT5G33050	132.0417	-7.165044447	0.438202	-16.351	4.28E-60	1.72E-57	0.640149	0	0	8.207663	7.850244	8.067702
AT1G50850	159.3496	-7.160355178	0.473568	-15.12	1.20E-51	3.30E-49	0	0	0	7.308122	8.516472	8.762964
AT1G33130	206.7877	-7.139678165	0.472088	-15.1236	1.13E-51	3.16E-49	0	0.626414	0	7.353641	9.270813	8.851451
AT3G44042	118.517	-7.116949895	0.458963	-15.5066	3.13E-54	1.01E-51	0	0	0	8.191041	7.804154	7.63192
AT3G01345	198.4049	-7.075913201	0.383439	-18.4538	4.86E-76	3.57E-73	1.419792	1.395705	0	8.922678	8.468027	8.45425
AT3G31540	115.2923	-7.070074629	0.460817	-15.3425	3.98E-53	1.25E-50	0	0	0	8.191041	7.673524	7.63192
AT3G43867	129.4578	-7.021407763	0.448082	-15.6699	2.43E-55	8.00E-53	0	0	0.694589	8.311275	8.086794	7.566881
AT3G32240	142.4994	-6.975919031	0.479714	-14.5418	6.58E-48	1.63E-45	0	0	0	8.887003	7.491614	7.676644
AT4G29200	124.6455	-6.968148462	0.425508	-16.3761	2.84E-60	1.16E-57	0	0.626414	0.694589	7.97503	8.021154	7.89677
AT2G04655	99.48946	-6.95876906	0.459719	-15.137	9.23E-52	2.61E-49	0	0	0	7.777648	7.567152	7.576354
AT4G06628	99.95979	-6.952400128	0.460127	-15.1097	1.40E-51	3.82E-49	0	0	0	7.649677	7.788457	7.498771
AT5G36655	320.7869	-6.928219905	0.486665	-14.2361	5.47E-46	1.11E-43	1.082007	0.626414	0	7.074357	9.919469	9.684546
AT2G13310	179.2185	-6.925891881	0.478086	-14.4867	1.47E-47	3.47E-45	0.640149	0	0	7.03764	8.942394	8.827852
AT4G16215	111.1074	-6.923919249	0.44445	-15.5786	1.02E-54	3.31E-52	0.640149	0	0	7.574773	7.966403	7.834975
AT2G13050	123.0577	-6.871474786	0.478353	-14.3649	8.60E-47	1.84E-44	0	0	0	7.110162	8.525969	7.873905
AT1G50735	122.6148	-6.854758422	0.479255	-14.303	2.10E-46	4.35E-44	0	0	0	7.110162	8.544777	7.827061
AT3G32043	102.8737	-6.851832872	0.444402	-15.4181	1.24E-53	3.93E-51	0.640149	0	0	7.697535	7.756543	7.613635
AT4G05275	95.99771	-6.831379575	0.467271	-14.6197	2.10E-48	5.30E-46	0	0	0	7.935661	7.283413	7.478706
AT2G13400	99.29592	-6.80844549	0.470754	-14.4629	2.08E-47	4.77E-45	0	0	0	7.074357	7.865285	7.850673
AT3G30663	132.3126	-6.802671775	0.486891	-13.9716	2.32E-44	4.45E-42	0	0	0	6.795248	8.344656	8.494778
AT2G13160	101.9937	-6.786868229	0.473587	-14.3308	1.41E-46	2.94E-44	0	0	0	7.984706	7.850244	7.032351
AT5G30762	87.8641	-6.770612261	0.466264	-14.521	8.92E-48	2.18E-45	0	0	0	7.245106	7.452309	7.66781
AT3G29648	128.1717	-6.769604743	0.468572	-14.4473	2.61E-47	5.87E-45	0	0.626414	0	8.676251	7.411902	7.585765
AT1G67240	107.0372	-6.768719782	0.430596	-15.7194	1.11E-55	3.72E-53	0	0.626414	0.694589	7.766472	7.656331	7.811102
AT5G32511	87.29709	-6.742902359	0.468033	-14.4069	4.68E-47	1.03E-44	0	0	0	7.720882	7.166841	7.427286
AT3G32966	129.2495	-6.67245426	0.476104	-14.0147	1.27E-44	2.45E-42	0.640149	0	0	6.838581	8.323029	8.423089
AT3G33225	112.8333	-6.652854233	0.488538	-13.6179	3.14E-42	5.63E-40	0	0	0	8.577394	7.26084	7.176347
AT5G28165	345.5481	-6.624176079	0.506477	-13.0789	4.35E-39	6.81E-37	0.640149	0.626414	0.694589	6.448866	9.982908	9.929658
AT3G42431	104.9529	-6.609103929	0.487636	-13.5534	7.57E-42	1.34E-39	0	0	0	8.415115	7.370331	6.976208

AT2G14230	230.524	-6.580635482	0.333787	-19.7151	1.60E-86	1.96E-83	2.121327	1.894767	1.79647	9.175955	8.678527	8.605328
AT2G13547	86.39252	-6.572027482	0.454139	-14.4714	1.84E-47	4.29E-45	0	0	0.694589	7.383209	7.673524	7.225339
AT2G33175	85.31554	-6.56508346	0.454376	-14.4486	2.56E-47	5.82E-45	0.640149	0	0	7.612711	7.432247	7.188751
AT3G30744	83.66179	-6.561245225	0.453449	-14.4697	1.88E-47	4.36E-45	0	0	0.694589	7.245106	7.349087	7.566881
AT4G03760	103.8914	-6.483385173	0.475707	-13.6289	2.69E-42	4.88E-40	0	0	0.694589	8.386201	7.066287	7.295843
AT3G29730	124.6605	-6.462805545	0.453907	-14.2382	5.31E-46	1.08E-43	0.640149	0.626414	0.694589	6.96128	8.161812	8.401935
AT2G15555	159.3943	-6.451780753	0.447144	-14.4289	3.41E-47	7.61E-45	1.419792	1.06174	0	9.084419	7.673524	7.711452
AT2G06180	114.3909	-6.446006064	0.483768	-13.3246	1.67E-40	2.74E-38	0	0	0.694589	6.980752	8.61765	7.406194
AT5G28923	137.9761	-6.423606911	0.496719	-12.9321	2.97E-38	4.38E-36	0	0.626414	0	6.332435	8.477847	8.619142
AT3G44265	87.00657	-6.375912696	0.448673	-14.2106	7.87E-46	1.57E-43	0.640149	0.626414	0	7.685719	7.567152	7.004552
AT2G34130	79.88609	-6.343086398	0.467942	-13.5553	7.37E-42	1.32E-39	0	0.626414	0	7.338627	7.707307	6.793345
AT4G07518	84.2498	-6.336405263	0.473468	-13.383	7.61E-41	1.27E-38	0	0.626414	0	6.656843	7.491614	7.827061
AT4G08080	106.3366	-6.320723921	0.489427	-12.9145	3.73E-38	5.47E-36	0.640149	0	0	8.570985	6.985988	7.059624
AT4G05715	63.73704	-6.309307301	0.481568	-13.1016	3.22E-39	5.08E-37	0	0	0	7.245106	6.574739	7.112669
AT4G04430	94.56974	-6.300144286	0.441509	-14.2696	3.39E-46	6.96E-44	1.082007	0.626414	0	7.625138	7.909492	7.032351
AT2G14730	75.10872	-6.296267267	0.49124	-12.8171	1.32E-37	1.87E-35	0	0	0	7.864069	6.810594	6.760481
AT2G05660	68.06213	-6.266019682	0.46349	-13.5192	1.20E-41	2.09E-39	0.640149	0	0	7.276958	6.779171	7.188751
AT3G30825	58.52424	-6.155445932	0.488007	-12.6134	1.78E-36	2.46E-34	0	0	0	7.179211	6.336481	7.004552
AT3G30827	73.11562	-6.098885778	0.482267	-12.6463	1.17E-36	1.63E-34	0.640149	0	0	6.301797	7.452309	7.547747
AT2G11110	63.33744	-6.093281014	0.495925	-12.2867	1.07E-34	1.31E-32	0	0	0	6.138016	7.370331	7.201051
AT2G23490	56.41114	-6.079467959	0.490454	-12.3956	2.76E-35	3.55E-33	0	0	0	7.127736	6.92986	6.316346
AT2G10490	52.61122	-6.078189192	0.486921	-12.4829	9.25E-36	1.24E-33	0	0	0	6.420628	6.871459	6.85691
AT5G33255	57.34452	-6.053831986	0.467921	-12.9377	2.76E-38	4.09E-36	0	0.626414	0	6.680852	6.985988	6.872373
AT1G37040	94.81795	-6.052648744	0.484342	-12.4967	7.79E-36	1.05E-33	0	0.626414	0.694589	6.205775	8.007659	7.904311
AT1G35300	61.43518	-6.041256322	0.498139	-12.1276	7.54E-34	8.80E-32	0	0	0	6.066916	7.166841	7.329843
AT2G06760	50.8742	-6.017562348	0.489692	-12.2885	1.04E-34	1.28E-32	0	0	0	6.999965	6.460523	6.526408
AT2G12240	71.75753	-6.015937683	0.507157	-11.8621	1.86E-32	2.08E-30	0	0	0	5.872138	7.391266	7.694153
AT4G03865	77.40735	-6.009211053	0.510892	-11.7622	6.11E-32	6.60E-30	0	0	0	8.131308	6.152528	6.85691
AT2G06590	66.17539	-6.007148626	0.503855	-11.9224	9.05E-33	1.02E-30	0	0	0	7.743857	6.810594	6.198607
AT4G03005	67.31185	-5.994397697	0.436943	-13.719	7.82E-43	1.46E-40	1.082007	0	0.694589	7.145099	7.09209	6.99045
AT2G13300	55.15827	-5.993806417	0.469813	-12.7579	2.82E-37	3.96E-35	0.640149	0	0	6.773083	6.958197	6.639175
AT4G02314	62.19426	-5.987196515	0.479157	-12.4953	7.92E-36	1.07E-33	0.640149	0	0	7.09237	7.305638	6.338783
AT1G36260	58.36769	-5.975411949	0.499644	-11.9593	5.80E-33	6.60E-31	0	0	0	7.368501	6.900955	6.096967
AT3G28899	86.38862	-5.973762859	0.402168	-14.8539	6.56E-50	1.70E-47	1.082007	0.626414	1.513627	7.212535	7.510873	7.547747

AT5G33391	57.14864	-5.939408823	0.501626	-11.8403	2.42E-32	2.68E-30	0	0	0	6.030007	6.841348	7.373965
AT1G40135	57.3646	-5.934627838	0.47873	-12.3966	2.73E-35	3.52E-33	0.640149	0	0	6.301797	6.958197	7.163834
AT2G06670	65.27555	-5.929783281	0.508143	-11.6695	1.82E-31	1.91E-29	0	0	0	7.788738	6.680573	6.148682
AT3G30713	45.05528	-5.913694274	0.490246	-12.0627	1.66E-33	1.92E-31	0	0	0	6.503738	6.537663	6.486772
AT1G42410	56.5877	-5.902700771	0.478973	-12.3237	6.76E-35	8.43E-33	0	0	0.694589	6.96128	7.11744	6.293554
AT3G29612	56.30174	-5.900255931	0.479925	-12.2941	9.74E-35	1.21E-32	0	0.626414	0	6.238498	7.11744	6.976208
AT5G32228	63.98523	-5.896961252	0.509395	-11.5764	5.43E-31	5.43E-29	0	0	0	7.799743	6.420349	6.293554
AT3G30218	69.92282	-5.8868097	0.453648	-12.9766	1.66E-38	2.54E-36	0	1.06174	0.694589	6.503738	7.370331	7.363061
AT3G42360	61.8892	-5.881049952	0.508495	-11.5656	6.16E-31	6.13E-29	0	0	0	7.46844	7.142352	5.806294
AT2G05700	50.6876	-5.873841213	0.473502	-12.4051	2.45E-35	3.20E-33	0	0	0.694589	6.656843	6.841348	6.506726
AT3G32677	71.7235	-5.8473802	0.499871	-11.6978	1.31E-31	1.39E-29	0.640149	0	0	5.829815	7.472095	7.63192
AT1G38360	63.90988	-5.84598928	0.47157	-12.3969	2.72E-35	3.52E-33	0.640149	0	0.694589	7.561902	6.537663	6.692417
AT5G32197	59.1617	-5.824269697	0.510337	-11.4126	3.62E-30	3.48E-28	0	0	0	7.63746	6.537663	6.04333
AT2G10640	73.36932	-5.802083302	0.486319	-11.9306	8.20E-33	9.28E-31	0	0.626414	0.694589	5.99213	7.472095	7.649977
AT1G37340	40.74923	-5.77797446	0.494891	-11.6752	1.71E-31	1.79E-29	0	0	0	6.420628	6.292644	6.382637
AT1G43840	47.59025	-5.715878568	0.482166	-11.8546	2.04E-32	2.27E-30	0	0	0.694589	6.301797	6.958197	6.404074
AT3G06955	83.22511	-5.712283617	0.392543	-14.552	5.67E-48	1.42E-45	1.419792	1.395705	1.161466	7.649677	7.190921	7.237331
AT3G44070	62.39451	-5.709075794	0.519402	-10.9916	4.19E-28	3.58E-26	0	0	0	7.842944	6.460523	5.899804
AT3G30820	44.40284	-5.681777104	0.481834	-11.792	4.29E-32	4.70E-30	0.640149	0	0	6.704469	6.152528	6.545826
AT3G29736	125.2075	-5.665701496	0.347715	-16.2941	1.09E-59	4.23E-57	1.419792	2.559128	1.79647	8.240343	7.603488	7.956026
AT4G18150	49.63982	-5.659925101	0.466878	-12.1229	7.99E-34	9.29E-32	1.082007	0	0	6.301797	6.747048	6.825478
AT2G16000	43.54043	-5.649153489	0.481602	-11.7299	8.95E-32	9.59E-30	0	0	0.694589	6.530411	6.610887	6.198607
AT3G29739	86.72034	-5.641600726	0.392403	-14.3771	7.21E-47	1.56E-44	1.419792	1.395705	1.513627	7.777648	7.040014	7.384788
AT1G35600	293.662	-5.634348844	0.552721	-10.1938	2.11E-24	1.49E-22	0	0	0	7.732415	9.55681	9.642326
AT3G30780	42.5528	-5.59615046	0.509534	-10.9829	4.62E-28	3.92E-26	0	0	0	6.838581	6.537663	5.671366
AT3G30751	37.71811	-5.59277317	0.504919	-11.0766	1.63E-28	1.43E-26	0	0	0	6.238498	5.82339	6.602557
AT5G32306	62.13315	-5.587318713	0.525971	-10.6229	2.33E-26	1.80E-24	0	0	0	7.915566	6.292644	5.806294
AT3G32070	36.96792	-5.584574785	0.503054	-11.1013	1.24E-28	1.11E-26	0	0	0	6.172293	6.499609	5.958939
AT5G31719	76.02572	-5.580973018	0.505526	-11.0399	2.45E-28	2.12E-26	0	0.626414	0.694589	8.182658	6.610887	6.123056
AT3G29734	149.003	-5.567399774	0.328744	-16.9354	2.47E-64	1.17E-61	1.693301	2.803425	2.413855	8.55808	7.894906	8.074403
AT3G45270	59.04369	-5.56344185	0.489638	-11.3624	6.44E-30	6.08E-28	0	0	1.161466	7.600176	6.200761	6.466537
AT5G29975	75.32013	-5.555317167	0.506839	-10.9607	5.90E-28	4.99E-26	0	0.626414	0.694589	8.191041	6.460523	6.198607
AT3G24542	38.6852	-5.551372524	0.508217	-10.9232	8.93E-28	7.50E-26	0	0	0	5.646896	6.574739	6.486772
AT2G10190	42.99822	-5.543564385	0.491111	-11.2878	1.51E-29	1.39E-27	0	0.626414	0	6.901235	6.102626	6.17386

AT1G40118	38.54336	-5.537633727	0.509351	-10.8719	1.57E-27	1.29E-25	0	0	0	5.646896	6.379024	6.657142
AT5G32107	66.2424	-5.537367429	0.500278	-11.0686	1.78E-28	1.56E-26	0	0.626414	0.694589	7.915566	6.420349	6.17386
AT3G30393	57.62638	-5.531672528	0.492444	-11.2331	2.80E-29	2.54E-27	0.640149	0.626414	0	6.102904	6.460523	7.576354
AT3G29700	34.89875	-5.508519685	0.505533	-10.8965	1.20E-27	9.93E-26	0	0	0	6.066916	6.420349	5.899804
AT2G06800	45.42103	-5.508323982	0.518533	-10.6229	2.33E-26	1.80E-24	0	0	0	5.319969	6.841348	6.917792
AT2G11775	33.24581	-5.4911065	0.505039	-10.8726	1.56E-27	1.29E-25	0	0	0	6.205775	5.941644	6.070398
AT4G08093	44.75077	-5.475598695	0.476486	-11.4916	1.45E-30	1.43E-28	0	0.626414	0.694589	6.632428	5.941644	6.777007
AT4G06835	36.60339	-5.452186262	0.486958	-11.1964	4.25E-29	3.83E-27	0.640149	0	0	6.205775	6.200761	6.222936
AT3G30767	47.17308	-5.451382856	0.523607	-10.4112	2.20E-25	1.63E-23	0	0	0	5.19216	6.779171	7.125632
AT2G14240	37.94688	-5.349899425	0.498178	-10.7389	6.68E-27	5.35E-25	0	0.626414	0	6.704469	6.152528	5.773726
AT1G38185	50.75257	-5.345378674	0.515317	-10.373	3.29E-25	2.41E-23	0	0	0.694589	7.535811	5.82339	6.015745
AT2G06250	43.42006	-5.338637405	0.527086	-10.1286	4.13E-24	2.87E-22	0	0	0	7.292624	5.476813	5.958939
AT4G08593	33.10209	-5.335285676	0.516174	-10.3362	4.83E-25	3.51E-23	0	0	0	5.545934	5.941644	6.545826
AT2G06370	39.81284	-5.33448456	0.52358	-10.1885	2.23E-24	1.57E-22	0	0	0	5.123743	6.779171	6.602557
AT3G35707	36.84949	-5.32734791	0.520958	-10.2261	1.52E-24	1.08E-22	0	0	0	6.880651	5.883728	5.561185
AT2G13040	40.94806	-5.31259129	0.525966	-10.1006	5.49E-24	3.76E-22	0	0	0	5.051918	6.871459	6.620982
AT2G05914	86.91758	-5.30536615	0.459787	-11.5387	8.41E-31	8.32E-29	1.693301	1.395705	0.694589	8.240343	7.013254	6.466537
AT4G25580	63.81279	-5.296888925	0.429769	-12.325	6.65E-35	8.33E-33	1.082007	1.06174	1.513627	6.301797	7.283413	7.201051
AT2G04320	36.2758	-5.293588158	0.498803	-10.6126	2.60E-26	2.00E-24	0.640149	0	0	6.205775	6.574739	5.671366
AT2G06490	42.74231	-5.281807795	0.488898	-10.8035	3.31E-27	2.66E-25	0.640149	0.626414	0	6.476563	6.871459	5.706299
AT1G39830	30.62046	-5.275208582	0.516141	-10.2205	1.61E-24	1.14E-22	0	0	0	6.391825	5.69457	5.671366
AT2G06904	38.91945	-5.266862381	0.52655	-10.0026	1.48E-23	9.96E-22	0	0	0	6.859769	6.420349	5.117892
AT2G12380	28.79047	-5.221175648	0.517055	-10.0979	5.64E-24	3.86E-22	0	0	0	5.437371	5.941644	6.148682
AT1G40074	29.81428	-5.220702889	0.518724	-10.0645	7.93E-24	5.38E-22	0	0	0	6.391825	5.553109	5.671366
AT5G29040	30.68511	-5.187304883	0.496938	-10.4385	1.65E-25	1.23E-23	0.640149	0	0	5.829815	6.102626	5.929675
AT2G14950	32.43348	-5.179309379	0.501377	-10.3302	5.15E-25	3.73E-23	0.640149	0	0	6.362435	6.050936	5.598855
AT3G30670	34.92976	-5.176798218	0.527826	-9.80777	1.04E-22	6.50E-21	0	0	0	4.97633	6.646151	6.338783
AT5G35792	30.35677	-5.153837866	0.523083	-9.85282	6.66E-23	4.19E-21	0	0	0	5.319969	6.499609	5.773726
AT3G32295	35.57389	-5.13950104	0.530815	-9.68229	3.59E-22	2.14E-20	0	0	0	6.880651	5.997325	5.117892
AT3G44325	30.6598	-5.116309896	0.503038	-10.1708	2.68E-24	1.87E-22	0.640149	0	0	6.301797	5.883728	5.598855
AT4G06538	39.93587	-5.108562881	0.454009	-11.2521	2.26E-29	2.06E-27	0	1.395705	0.694589	6.448866	6.336481	6.17386
AT3G30665	25.23116	-5.102905165	0.51877	-9.83655	7.84E-23	4.90E-21	0	0	0	5.545934	5.760417	5.740407
AT3G30770	35.71235	-5.09746376	0.514698	-9.9038	4.01E-23	2.57E-21	0.640149	0	0	6.859769	5.396255	5.869303
AT4G08598	30.39889	-5.09407699	0.528033	-9.64726	5.05E-22	2.96E-20	0	0	0	6.582321	5.123469	5.773726

AT2G06002	148.6793	-5.092286856	0.27213	-18.7127	3.90E-78	3.13E-75	3.046206	3.10637	2.714973	8.113777	8.086794	8.347651
AT3G30165	28.09512	-5.091907007	0.524228	-9.71315	2.65E-22	1.59E-20	0	0	0	6.301797	5.760417	5.265734
AT2G00430	61.3488	-5.084523901	0.417849	-12.1683	4.58E-34	5.40E-32	1.419792	1.395705	1.513627	7.426454	6.499609	6.692417
AT2G11650	31.15266	-5.074494995	0.52973	-9.5794	9.76E-22	5.60E-20	0	0	0	6.656843	5.69457	5.218117
AT2G12385	50.91524	-5.06034672	0.488945	-10.3495	4.21E-25	3.06E-23	0	1.666709	0	5.597298	6.900955	7.112669
AT2G20460	43.63909	-5.051720658	0.526892	-9.58776	9.00E-22	5.19E-20	0	0	0.694589	7.338627	5.997325	5.265734
AT2G06845	25.75645	-5.046319667	0.522982	-9.64913	4.96E-22	2.91E-20	0	0	0	5.786214	5.997325	5.265734
AT3G30746	24.2371	-5.029104576	0.521772	-9.63852	5.50E-22	3.20E-20	0	0	0	5.437371	5.82339	5.598855
AT3G42658	74.34394	-5.027691351	0.374934	-13.4095	5.32E-41	8.93E-39	2.121327	1.666709	1.79647	7.426454	7.327526	6.760481
AT2G16670	46.2901	-5.024897855	0.517189	-9.71578	2.58E-22	1.56E-20	0	1.06174	0	7.397768	6.050936	5.482759
AT4G07490	38.76431	-5.011670636	0.524664	-9.55215	1.27E-21	7.17E-20	0.640149	0	0	7.127736	5.760417	5.356498
AT2G04885	42.26849	-5.00770647	0.528246	-9.47987	2.55E-21	1.41E-19	0	0	0.694589	7.308122	5.883728	5.265734
AT2G12520	36.04488	-4.990043964	0.52179	-9.56332	1.14E-21	6.47E-20	0.640149	0	0	5.051918	6.871459	6.096967
AT1G40077	32.5651	-4.968828373	0.53729	-9.24794	2.29E-20	1.18E-18	0	0	0	4.626795	6.610887	6.246861
AT1G35995	91.8519	-4.959512262	0.548113	-9.04833	1.45E-19	7.16E-18	0.640149	0.626414	0	4.021447	7.804154	8.291245
AT1G37160	34.43855	-4.953358593	0.540323	-9.1674	4.85E-20	2.45E-18	0	0	0	7.03764	5.220243	5.356498
AT5G33254	22.47099	-4.925930927	0.525751	-9.36933	7.30E-21	3.92E-19	0	0	0	5.741253	5.396255	5.399825
AT1G36630	1211.524	-4.924761673	0.56023	-8.79061	1.49E-18	6.72E-17	1.92313	3.634384	1.161466	10.56814	11.77538	11.12482
AT1G34530	29.12105	-4.920605236	0.514522	-9.56346	1.14E-21	6.47E-20	0.640149	0	0	5.694846	6.420349	5.31183
AT3G43523	29.46267	-4.920589921	0.492396	-9.99316	1.63E-23	1.09E-21	1.082007	0	0	6.102904	5.941644	5.598855
AT2G23720	42.35022	-4.916868193	0.547708	-8.97718	2.78E-19	1.35E-17	0	0	0	4.164539	6.900955	6.90281
AT2G01840	36.41723	-4.89404183	0.490592	-9.97579	1.95E-23	1.29E-21	0.640149	1.06174	0	6.205775	6.680573	5.441888
AT3G33151	39.06834	-4.878310784	0.499531	-9.76578	1.58E-22	9.75E-21	1.082007	0.626414	0	6.921529	6.200761	5.356498
AT4G07965	26.09807	-4.867525906	0.513653	-9.4763	2.63E-21	1.46E-19	0.640149	0	0	6.172293	5.310931	5.561185
AT3G31910	31.3262	-4.854681261	0.482098	-10.0699	7.50E-24	5.10E-22	1.082007	0.626414	0	6.301797	5.553109	5.987621
AT2G07460	25.66964	-4.847415921	0.51293	-9.45044	3.37E-21	1.85E-19	0	0	0.694589	5.19216	5.997325	5.806294
AT5G29408	22.92036	-4.845329052	0.531279	-9.12013	7.50E-20	3.74E-18	0	0	0	5.051918	5.997325	5.441888
AT3G32377	21.02635	-4.841095975	0.52878	-9.15521	5.43E-20	2.73E-18	0	0	0	5.597298	5.220243	5.441888
AT1G42745	30.97305	-4.838128019	0.542729	-8.91444	4.90E-19	2.33E-17	0	0	0	4.414102	6.574739	6.17386
AT2G26630	28.12172	-4.831968221	0.519097	-9.30842	1.30E-20	6.80E-19	0	0	0.694589	5.051918	6.379024	5.773726
AT3G62475	21.69069	-4.819757492	0.532024	-9.05929	1.31E-19	6.51E-18	0	0	0	5.597298	4.907958	5.773726
AT4G08720	27.14562	-4.801278484	0.497133	-9.65794	4.55E-22	2.68E-20	0	1.06174	0	5.492674	6.050936	5.740407
AT5G32624	137.9944	-4.796419146	0.567593	-8.45045	2.90E-17	1.17E-15	0	0	0	6.476563	8.516472	8.553511
AT3G25719	32.87888	-4.784275319	0.513514	-9.31674	1.20E-20	6.30E-19	0	0.626414	0.694589	4.896561	6.292644	6.506726

AT3G43154	27.68115	-4.781751781	0.52288	-9.14502	5.96E-20	2.98E-18	0	0	0.694589	6.476563	5.310931	5.31183
AT4G09380	44.09944	-4.776702752	0.531431	-8.98839	2.51E-19	1.22E-17	0.640149	0.626414	0	4.414102	6.985988	6.887672
AT5G45570	297.1668	-4.758692797	0.567491	-8.38549	5.05E-17	2.01E-15	0	0.626414	1.161466	3.242825	9.689499	9.890325
AT3G29634	31.4577	-4.756759299	0.514064	-9.25324	2.18E-20	1.13E-18	0.640149	0.626414	0	5.051918	5.883728	6.620982
AT1G10070	2134.618	-4.74987205	0.145415	-32.6642	5.05E-234	6.48E-230	7.406536	7.212852	6.918555	11.87526	12.16546	11.9745
AT2G04000	20.43605	-4.73139976	0.534161	-8.85763	8.17E-19	3.78E-17	0	0	0	5.646896	5.476813	4.953151
AT4G06672	19.50143	-4.728714589	0.532526	-8.87979	6.70E-19	3.12E-17	0	0	0	5.25748	5.476813	5.218117
AT3G14670	21.86554	-4.721120373	0.537227	-8.78795	1.52E-18	6.85E-17	0	0	0	4.812122	5.997325	5.399825
AT2G12650	25.74525	-4.716560307	0.500315	-9.42718	4.21E-21	2.30E-19	0.640149	0.626414	0	5.786214	5.941644	5.31183
AT4G19240	25.36425	-4.68726939	0.501758	-9.34169	9.48E-21	5.02E-19	1.082007	0	0	5.545934	5.997325	5.441888
AT3G53910	20.87058	-4.682393155	0.539314	-8.68213	3.88E-18	1.70E-16	0	0	0	5.437371	4.654502	5.899804
AT4G03770	22.73553	-4.672097577	0.518818	-9.00527	2.15E-19	1.05E-17	0	0	0.694589	5.492674	5.883728	5.117892
AT2G06965	20.6246	-4.668158643	0.538657	-8.66629	4.46E-18	1.94E-16	0	0	0	5.872138	5.310931	4.831847
AT5G19097	137.669	-4.667496711	0.548982	-8.50209	1.86E-17	7.71E-16	1.419792	1.395705	0.694589	3.684038	8.61765	8.712309
AT1G36085	34.00774	-4.661094974	0.553914	-8.41483	3.93E-17	1.58E-15	0	0	0	3.862584	6.574739	6.602557
AT1G49080	103.5103	-4.649504435	0.569144	-8.16929	3.10E-16	1.16E-14	0	0	0	6.066916	8.185975	8.054206
AT1G33135	29.39985	-4.644033387	0.551247	-8.4246	3.62E-17	1.46E-15	0	0	0	4.021447	6.247434	6.446015
AT5G33240	18.71489	-4.637454159	0.536915	-8.63722	5.76E-18	2.48E-16	0	0	0	4.896561	5.476813	5.356498
AT4G07942	995.5493	-4.631419905	0.575525	-8.0473	8.46E-16	3.04E-14	0	0	0	11.94073	9.722914	10.22894
AT1G59930	171.4759	-4.6002686	0.378181	-12.1642	4.82E-34	5.66E-32	3.669966	2.264856	3.6007	8.393484	8.956509	7.427286
AT3G44006	17.86879	-4.584242674	0.539197	-8.50199	1.86E-17	7.71E-16	0	0	0	5.379864	4.786789	5.356498
AT4G22415	24.49972	-4.571054369	0.509793	-8.96649	3.06E-19	1.48E-17	0.640149	0	0.694589	4.97633	5.997325	5.740407
AT1G38430	38.4637	-4.570617931	0.493952	-9.25316	2.18E-20	1.13E-18	1.082007	1.395705	0	6.880651	6.247434	5.218117
AT2G13463	20.19239	-4.548922986	0.523837	-8.68385	3.83E-18	1.68E-16	0.640149	0	0	5.646896	4.907958	5.441888
AT3G33166	97.52277	-4.547974891	0.436861	-10.4106	2.22E-25	1.63E-23	2.295555	2.911631	1.513627	8.013353	7.938228	6.17386
AT4G15242	24.62059	-4.544712102	0.51247	-8.86826	7.43E-19	3.45E-17	0.640149	0	0.694589	4.896561	6.050936	5.740407
AT2G16140	71.82996	-4.533267122	0.409067	-11.082	1.53E-28	1.36E-26	1.693301	1.894767	2.714973	7.788738	6.646151	6.674887
AT1G43815	27.47875	-4.533134401	0.554751	-8.17148	3.05E-16	1.14E-14	0	0	0	3.862584	6.152528	6.360876
AT1G41775	25.10013	-4.527056597	0.552851	-8.18857	2.64E-16	9.97E-15	0	0	0	6.607592	4.786789	4.831847
AT4G06575	24.43512	-4.521772692	0.552315	-8.18695	2.68E-16	1.01E-14	0	0	0	6.530411	5.019734	4.62832
AT4G08710	19.75452	-4.500231872	0.526332	-8.55017	1.23E-17	5.19E-16	0.640149	0	0	4.97633	5.220243	5.706299
AT2G13320	23.02596	-4.494214413	0.552042	-8.14108	3.92E-16	1.45E-14	0	0	0	4.021447	5.941644	5.987621
AT5G37385	33.84639	-4.490466078	0.560848	-8.00657	1.18E-15	4.19E-14	0	0	0	3.480233	6.610887	6.602557
AT1G41680	17.06207	-4.475369504	0.543723	-8.23097	1.86E-16	7.09E-15	0	0	0	4.626795	5.310931	5.356498

AT2G12250	23.6124	-4.463286696	0.537296	-8.30694	9.82E-17	3.81E-15	0	0	0.694589	4.414102	5.69457	6.148682
AT4G08013	30.359	-4.456991526	0.560312	-7.95448	1.80E-15	6.31E-14	0	0	0	3.684038	5.997325	6.760481
AT3G31920	24.56281	-4.448211334	0.556243	-7.99688	1.28E-15	4.52E-14	0	0	0	4.021447	5.553109	6.446015
AT1G42500	20.86884	-4.444168111	0.531476	-8.36194	6.17E-17	2.44E-15	0	0	0.694589	5.741253	5.625572	4.62832
AT4G33980	97.98175	-4.438744966	0.342204	-12.9711	1.79E-38	2.70E-36	2.295555	2.911631	3.271714	7.074357	7.491614	8.005951
AT2G06740	15.99453	-4.42987978	0.544681	-8.13297	4.19E-16	1.54E-14	0	0	0	5.19216	4.654502	5.218117
AT1G35500	19.00279	-4.415102532	0.550653	-8.01794	1.08E-15	3.84E-14	0	0	0	4.164539	5.625572	5.635566
AT3G37820	24.98768	-4.388329417	0.543274	-8.07756	6.61E-16	2.39E-14	0	0	0.694589	5.913254	6.200761	4.208431
AT4G18410	32.17815	-4.373670672	0.541179	-8.08175	6.38E-16	2.32E-14	0	0	1.161466	4.164539	6.247434	6.657142
AT3G32230	24.95158	-4.372631122	0.545465	-8.01633	1.09E-15	3.88E-14	0.640149	0	0	6.476563	5.396255	4.391288
AT2G05695	16.96338	-4.361354368	0.54997	-7.93017	2.19E-15	7.65E-14	0	0	0	5.646896	5.019734	4.474702
AT3G32300	24.18502	-4.357795385	0.559969	-7.78221	7.13E-15	2.36E-13	0	0	0	6.476563	3.710678	5.522505
AT5G32563	26.6796	-4.355785642	0.561719	-7.75439	8.88E-15	2.90E-13	0	0	0	3.480233	6.247434	6.246861
AT3G24517	16.23761	-4.352236444	0.548946	-7.92835	2.22E-15	7.74E-14	0	0	0	5.437371	5.123469	4.474702
AT2G11340	22.45325	-4.341641597	0.558794	-7.76966	7.87E-15	2.59E-13	0	0	0	6.476563	4.654502	4.553554
AT2G05015	15.91492	-4.340576814	0.548734	-7.91017	2.57E-15	8.87E-14	0	0	0	5.19216	5.310931	4.474702
AT2G05915	33.13792	-4.297532404	0.507426	-8.46928	2.47E-17	1.01E-15	1.419792	0.626414	0.694589	6.859769	5.625572	5.065041
AT3G42622	27.00217	-4.297194476	0.538731	-7.97651	1.51E-15	5.30E-14	0.640149	0	0.694589	6.680852	4.50885	5.265734
AT5G44416	23.81819	-4.286556985	0.56235	-7.62257	2.49E-14	7.83E-13	0	0	0	3.480233	6.050936	6.096967
AT1G40115	16.54703	-4.278957554	0.553688	-7.7281	1.09E-14	3.56E-13	0	0	0	5.646896	5.019734	4.302755
AT1G36520	272.7695	-4.269531415	0.578019	-7.38649	1.51E-13	4.43E-12	0	0	1.161466	10.01153	8.468027	7.977634
AT5G26970	38.68469	-4.26940738	0.523857	-8.14994	3.64E-16	1.35E-14	1.419792	0.626414	0.694589	4.29471	6.499609	6.932619
AT4G06517	141.5975	-4.26604675	0.57849	-7.37445	1.65E-13	4.81E-12	0	0	0	9.128733	6.92986	7.416778
AT2G11050	21.39407	-4.262227002	0.507776	-8.39392	4.70E-17	1.88E-15	0.640149	0.626414	0.694589	5.872138	4.786789	5.441888
AT4G06621	20.22465	-4.259739582	0.5601	-7.60532	2.84E-14	8.89E-13	0	0	0	3.684038	5.69457	5.899804
AT4G05025	14.31584	-4.252793644	0.550896	-7.71977	1.17E-14	3.79E-13	0	0	0	5.19216	4.786789	4.62832
AT5G32516	14.7594	-4.252176722	0.552025	-7.70286	1.33E-14	4.29E-13	0	0	0	5.379864	4.654502	4.62832
AT2G23500	17.54686	-4.220446014	0.5399	-7.81709	5.41E-15	1.82E-13	0.640149	0	0	5.694846	5.019734	4.553554
AT3G32950	15.49031	-4.218748586	0.554886	-7.60291	2.90E-14	9.05E-13	0	0	0	4.164539	5.476813	5.065041
AT5G15360	47.79843	-4.214131567	0.562528	-7.49142	6.81E-14	2.06E-12	0.640149	0	0.694589	2.958503	7.166841	7.09959
AT5G15420	17.42866	-4.21361166	0.539547	-7.80953	5.74E-15	1.92E-13	0	0.626414	0	4.524365	5.69457	5.01018
AT3G30396	95.63449	-4.206484277	0.57827	-7.27426	3.48E-13	9.78E-12	0	0	0	5.786214	7.835043	8.19614
AT3G42723	33.61586	-4.205259147	0.530002	-7.93441	2.11E-15	7.40E-14	0	0.626414	1.513627	7.03764	5.019734	5.265734
AT3G30720	311.0296	-4.184980052	0.181751	-23.0259	2.57E-117	5.50E-114	4.908884	4.97492	5.024124	9.043339	9.195448	9.375589

AT3G30765	14.68414	-4.181766371	0.55546	-7.52847	5.13E-14	1.57E-12	0	0	0	4.164539	5.310931	5.065041
AT2G13175	17.32411	-4.180599895	0.519669	-8.04473	8.64E-16	3.10E-14	0	0.626414	0.694589	5.319969	5.123469	4.953151
AT1G38260	42.32417	-4.1710324	0.505686	-8.24826	1.61E-16	6.15E-15	1.92313	1.06174	0.694589	7.276958	5.997325	5.065041
AT1G38330	42.32417	-4.1710324	0.505686	-8.24826	1.61E-16	6.15E-15	1.92313	1.06174	0.694589	7.276958	5.997325	5.065041
AT2G14970	15.2081	-4.154419067	0.557762	-7.44837	9.45E-14	2.83E-12	0	0	0	4.021447	5.476813	5.065041
AT5G24240	48.62145	-4.144285788	0.546685	-7.58075	3.44E-14	1.06E-12	0.640149	1.395705	0.694589	3.480233	7.190921	7.073069
AT5G19015	13.75844	-4.104313733	0.557344	-7.36405	1.78E-13	5.17E-12	0	0	0	4.896561	5.220243	4.208431
AT5G31087	14.19375	-4.100557623	0.55876	-7.33867	2.16E-13	6.20E-12	0	0	0	4.021447	5.310931	5.01018
AT3G06715	16.70201	-4.09198506	0.5634	-7.26301	3.79E-13	1.06E-11	0	0	0	5.953231	4.50885	4.208431
AT2G12500	59.24283	-4.087216549	0.511742	-7.98687	1.38E-15	4.88E-14	1.419792	2.091646	1.513627	7.95548	6.102626	5.117892
AT5G32515	101.4419	-4.081239332	0.580742	-7.02763	2.10E-12	5.49E-11	0	0	0	5.786214	8.267496	7.956026
AT1G59920	83.40661	-4.071940809	0.370505	-10.9903	4.26E-28	3.62E-26	3.046206	2.419482	3.176244	7.179211	7.909492	6.620982
AT3G27327	12.74275	-4.071840161	0.55774	-7.30061	2.86E-13	8.12E-12	0	0	0	4.29471	4.786789	5.01018
AT3G43573	18.1765	-4.062700462	0.552118	-7.35839	1.86E-13	5.39E-12	0	0.626414	0	6.030007	4.654502	4.391288
AT1G80160	164.053	-4.056145299	0.239678	-16.9233	3.03E-64	1.41E-61	4.150129	4.201568	3.985697	8.311275	8.525969	7.977634
AT4G02960	21.14365	-4.056091184	0.482172	-8.41213	4.03E-17	1.62E-15	0.640149	1.06174	1.161466	5.19216	5.553109	5.441888
AT2G06562	15.87566	-4.055738039	0.563705	-7.19479	6.26E-13	1.73E-11	0	0	0	5.741253	4.907958	3.881636
AT1G36460	171.743	-4.044193077	0.582499	-6.94283	3.84E-12	9.79E-11	0	0	0	6.448866	8.728738	9.027518
AT2G23480	15.00001	-4.043426221	0.543909	-7.43401	1.05E-13	3.14E-12	0.640149	0	0	4.812122	5.396255	4.474702
AT2G06906	26.35259	-4.041446494	0.563255	-7.17516	7.22E-13	1.99E-11	0	0	0.694589	6.270495	6.200761	3.284433
AT3G23085	26.42638	-4.035543067	0.45538	-8.86193	7.86E-19	3.64E-17	0.640149	1.894767	1.161466	5.545934	5.625572	5.929675
AT5G28523	104.7441	-4.031632353	0.57632	-6.99547	2.64E-12	6.84E-11	0.640149	0.626414	0.694589	1.429371	8.173944	8.401935
AT4G09425	43.89417	-4.030065422	0.42381	-9.50912	1.92E-21	1.07E-19	1.419792	2.419482	2.032847	6.773083	5.553109	6.657142
AT3G44215	125.3664	-4.020795708	0.579478	-6.93865	3.96E-12	1.00E-10	0	0.626414	0.694589	9.020001	6.610887	7.09959
AT2G15750	17.37036	-4.019607233	0.512298	-7.84623	4.29E-15	1.45E-13	0.640149	0.626414	0.694589	5.437371	5.019734	4.893774
AT5G37125	23.60252	-4.017834425	0.562407	-7.144	9.07E-13	2.48E-11	0.640149	0	0	3.242825	6.152528	5.987621
AT2G13870	178.891	-4.013723005	0.583008	-6.88451	5.80E-12	1.45E-10	0	0	0	6.476563	8.854671	9.034408
AT2G15940	25.51027	-4.006926993	0.527346	-7.59828	3.00E-14	9.36E-13	0.640149	0.626414	1.161466	6.556599	5.123469	4.62832
AT2G12980	13.26168	-4.006133564	0.561661	-7.13265	9.85E-13	2.68E-11	0	0	0	5.19216	4.907958	3.998987
AT4G33465	67.31289	-4.005653459	0.454966	-8.80429	1.32E-18	6.01E-17	2.719161	2.419482	1.79647	5.437371	7.707307	7.151212
AT2G03865	336307.6	-4.001257987	0.433606	-9.22788	2.76E-20	1.42E-18	14.25787	14.42946	14.8395	19.20687	19.99666	18.1733
AT3G43128	16.28566	-3.995756942	0.567041	-7.04668	1.83E-12	4.83E-11	0	0	0	5.953231	4.50885	3.998987
AT2G13431	45.38261	-3.992261146	0.383999	-10.3965	2.57E-25	1.89E-23	2.836608	1.395705	2.032847	6.503738	6.610887	6.246861
AT2G04770	526.6598	-3.990396274	0.584242	-6.83004	8.49E-12	2.08E-10	0	0	0	7.945604	10.55817	10.46014

AT2G12066	11.95118	-3.985727034	0.5602	-7.11483	1.12E-12	3.04E-11	0	0	0	4.896561	4.654502	4.302755
AT3G42100	20.4708	-3.985051845	0.486868	-8.18508	2.72E-16	1.02E-14	0.640149	0.626414	1.513627	5.123743	5.476813	5.441888
AT1G47650	26.0544	-3.983552009	0.574536	-6.93351	4.11E-12	1.04E-10	0	0	0	2.604042	6.200761	6.316346
AT4G04400	14.12851	-3.969424199	0.565396	-7.02061	2.21E-12	5.77E-11	0	0	0	5.646896	4.164273	4.302755
AT1G40125	85.91104	-3.942146824	0.582616	-6.76629	1.32E-11	3.15E-10	0	0	0	5.492674	7.966403	7.803055
AT3G29738	12.64206	-3.941544236	0.564727	-6.97956	2.96E-12	7.61E-11	0	0	0	4.896561	3.710678	5.168875
AT5G17125	21.33479	-3.935020404	0.573344	-6.86328	6.73E-12	1.67E-10	0	0	0	3.242825	6.460523	5.065041
AT2G11950	12.4214	-3.93103733	0.563445	-6.97679	3.02E-12	7.75E-11	0	0	0	4.524365	5.220243	4.107507
AT1G25430	133.4517	-3.923469067	0.376134	-10.431	1.79E-25	1.33E-23	3.22918	4.158291	3.445564	6.838581	8.233121	8.464489
AT3G31450	11.35319	-3.918886544	0.562589	-6.96581	3.27E-12	8.36E-11	0	0	0	4.896561	4.346824	4.391288
AT4G33467	84.12515	-3.905767448	0.457284	-8.54123	1.33E-17	5.58E-16	3.046206	2.911631	2.032847	5.545934	8.060895	7.468567
AT5G35146	16.80326	-3.897551643	0.571522	-6.8196	9.13E-12	2.23E-10	0	0	0	6.102904	4.346824	3.881636
AT1G38194	11.94482	-3.862172795	0.5666	-6.8164	9.34E-12	2.28E-10	0	0	0	5.25748	4.164273	4.208431
AT2G12830	12.49599	-3.858471963	0.567616	-6.79768	1.06E-11	2.57E-10	0	0	0	5.379864	4.346824	3.998987
AT1G47265	26.30013	-3.843876634	0.571974	-6.72037	1.81E-11	4.25E-10	0	0.626414	0	2.604042	6.379024	6.148682
AT3G43680	18.15934	-3.836278298	0.564162	-6.79996	1.05E-11	2.53E-10	0.640149	0	0	6.205775	4.346824	4.107507
AT1G36540	10.59385	-3.834286537	0.565284	-6.78294	1.18E-11	2.83E-10	0	0	0	4.722432	4.164273	4.474702
AT1G40124	10.60021	-3.827523708	0.565321	-6.77053	1.28E-11	3.06E-10	0	0	0	4.164539	4.654502	4.553554
AT3G33595	22.06661	-3.826915759	0.514835	-7.43329	1.06E-13	3.15E-12	0.640149	1.395705	0.694589	5.379864	6.102626	4.474702
AT3G28193	12.67188	-3.823125103	0.55088	-6.94003	3.92E-12	9.95E-11	0	0	0.694589	4.414102	5.123469	4.474702
AT4G07493	18.75847	-3.821530272	0.575771	-6.63724	3.20E-11	7.31E-10	0	0	0	6.391825	3.710678	4.208431
AT5G04935	18.47625	-3.819838026	0.575639	-6.63582	3.23E-11	7.37E-10	0	0	0	6.362435	3.710678	4.208431
AT4G03775	17.08434	-3.817532925	0.563008	-6.7806	1.20E-11	2.86E-10	0.640149	0	0	5.913254	5.019734	3.613718
AT2G12300	113.4275	-3.811981822	0.585169	-6.51432	7.30E-11	1.60E-09	0	0	0	5.786214	8.161812	8.417829
AT2G03875	1135214	-3.80302403	0.42041	-9.04598	1.48E-19	7.30E-18	16.39907	16.40548	16.87329	20.97295	21.71353	19.96141
AT1G06963	14.60395	-3.802095599	0.557931	-6.81464	9.45E-12	2.30E-10	0.640149	0	0	4.626795	5.625572	3.998987
AT4G06586	11.48097	-3.789070716	0.569323	-6.6554	2.83E-11	6.51E-10	0	0	0	5.19216	3.710678	4.474702
AT3G41768	3791353	-3.786996061	0.509863	-7.42748	1.11E-13	3.28E-12	16.81473	18.40595	17.46229	22.63102	23.52563	21.75266
AT5G29562	117.7994	-3.783534474	0.58463	-6.47167	9.69E-11	2.08E-09	0.640149	0	0	5.786214	8.244671	8.45425
AT4G33150	1769.791	-3.778890425	0.122173	-30.9308	4.61E-210	3.95E-206	8.059837	7.875296	7.662533	11.65054	11.75533	11.66559
AT4G06511	16.19199	-3.775033597	0.575339	-6.56141	5.33E-11	1.18E-09	0	0	0	6.102904	4.164273	3.753887
AT1G42110	10.64771	-3.77365409	0.567582	-6.64865	2.96E-11	6.79E-10	0	0	0	4.29471	4.907958	4.107507
AT5G13475	74.17437	-3.772315256	0.579422	-6.51048	7.49E-11	1.63E-09	0	1.06174	0.694589	1.429371	7.690514	7.889188
AT4G07600	11.44116	-3.769067357	0.569711	-6.61575	3.70E-11	8.36E-10	0	0	0	5.19216	4.346824	3.881636

AT3G06365	2827304	-3.768484393	0.417599	-9.02417	1.81E-19	8.90E-18	17.66549	17.94396	18.17946	22.28838	23.02836	21.27035
AT4G18420	16.61863	-3.767336543	0.57566	-6.54438	5.97E-11	1.32E-09	0	0	0	2.958503	5.941644	5.065041
AT3G02515	105.5169	-3.762202139	0.259474	-14.4994	1.22E-47	2.91E-45	3.731919	3.634384	3.928251	7.755209	7.756543	7.373965
AT2G12840	12.44915	-3.758407167	0.571858	-6.57228	4.96E-11	1.11E-09	0	0	0	5.492674	4.164273	3.881636
AT3G32226	14.08124	-3.757195693	0.573342	-6.55315	5.63E-11	1.25E-09	0	0	0	4.626795	5.69457	3.458449
AT2G01010	3295391	-3.751053554	0.507864	-7.38594	1.51E-13	4.44E-12	16.6923	18.26786	17.33721	22.42886	23.31828	21.55438
AT5G45095	29.27182	-3.750298914	0.546821	-6.85837	6.97E-12	1.72E-10	1.693301	0.626414	0	3.684038	6.336481	6.360876
AT1G49070	66.00071	-3.73890785	0.58514	-6.38976	1.66E-10	3.46E-09	0	0	0	5.051918	7.491614	7.538084
AT3G31909	9.821241	-3.737566177	0.568165	-6.57831	4.76E-11	1.07E-09	0	0	0	4.524365	4.164273	4.391288
AT1G08997	22.67606	-3.726692692	0.580284	-6.42218	1.34E-10	2.84E-09	0	0	0	2.133098	6.102626	6.04333
AT4G07520	9.914906	-3.72463792	0.568841	-6.54776	5.84E-11	1.29E-09	0	0	0	4.021447	4.50885	4.553554
AT5G42900	53.91136	-3.723401929	0.393792	-9.45525	3.22E-21	1.77E-19	2.836608	2.911631	2.032847	6.138016	6.420349	7.272722
AT5G31981	10.39961	-3.708274769	0.570557	-6.49939	8.06E-11	1.75E-09	0	0	0	4.97633	4.164273	3.998987
AT4G15245	10.03578	-3.705724726	0.569863	-6.50283	7.88E-11	1.72E-09	0	0	0	3.862584	4.654502	4.553554
AT3G42258	21.03263	-3.702353154	0.523994	-7.06563	1.60E-12	4.23E-11	0.640149	1.395705	0.694589	5.646896	5.883728	4.107507
AT5G28865	18.06364	-3.70174597	0.56961	-6.49873	8.10E-11	1.76E-09	0	0	0.694589	2.958503	5.476813	5.869303
AT3G60176	10.84317	-3.680913961	0.572615	-6.42825	1.29E-10	2.73E-09	0	0	0	5.19216	3.955229	3.998987
AT4G08016	88.19143	-3.679891125	0.585287	-6.28732	3.23E-10	6.44E-09	0	0	0.694589	5.545934	7.411902	8.296986
AT5G32473	9.922484	-3.6599421	0.57187	-6.39995	1.55E-10	3.25E-09	0	0	0	4.896561	3.955229	4.107507
AT5G28927	10.49896	-3.653404815	0.573042	-6.37545	1.82E-10	3.78E-09	0	0	0	3.480233	4.786789	4.767144
AT2G04290	9.942779	-3.648465654	0.571989	-6.37855	1.79E-10	3.70E-09	0	0	0	4.722432	4.50885	3.753887
AT1G06407	10.30406	-3.632709526	0.573663	-6.33248	2.41E-10	4.92E-09	0	0	0	3.480233	4.654502	4.831847
AT1G08630	321.5537	-3.625969571	0.252762	-14.3454	1.14E-46	2.40E-44	5.843167	5.43997	4.910056	8.796502	9.566046	9.23314
AT1G42070	9.048632	-3.623895878	0.571609	-6.33982	2.30E-10	4.70E-09	0	0	0	4.29471	4.164273	4.302755
AT4G06518	13.80013	-3.619401566	0.578358	-6.25807	3.90E-10	7.66E-09	0	0	0	5.829815	4.164273	3.458449
AT4G02865	10.91212	-3.618178917	0.558043	-6.48369	8.95E-11	1.93E-09	0	0	0.694589	4.524365	4.786789	4.107507
AT4G09355	10.20336	-3.614067882	0.574762	-6.28794	3.22E-10	6.42E-09	0	0	0	4.414102	3.416038	5.01018
AT3G47330	9.558652	-3.611570629	0.573163	-6.30112	2.96E-10	5.95E-09	0	0	0	3.684038	4.50885	4.62832
AT2G11115	22.81775	-3.610496491	0.5831	-6.1919	5.94E-10	1.14E-08	0	0	0	6.795248	4.346824	3.08651
AT3G45380	11.27346	-3.608644728	0.576027	-6.26472	3.74E-10	7.39E-09	0	0	0	5.379864	3.955229	3.753887
AT2G17690	42.0385	-3.60863246	0.429633	-8.39934	4.49E-17	1.80E-15	1.92313	2.803425	2.032847	6.880651	6.336481	5.482759
AT1G36200	10.45347	-3.602817924	0.574529	-6.27091	3.59E-10	7.12E-09	0	0	0	4.626795	4.907958	3.458449
AT1G41840	10.15787	-3.577241438	0.575691	-6.21382	5.17E-10	1.00E-08	0	0	0	5.123743	3.710678	3.998987
AT5G04995	9.519398	-3.570653082	0.574917	-6.21073	5.27E-10	1.02E-08	0	0	0	4.896561	3.710678	4.107507

AT5G32517	13.96147	-3.555599316	0.58045	-6.12559	9.03E-10	1.69E-08	0	0	0	5.913254	3.955229	3.458449
AT3G29610	8.692379	-3.553974273	0.573922	-6.19243	5.92E-10	1.14E-08	0	0	0	4.021447	4.164273	4.391288
AT3G24516	8.571508	-3.535124883	0.574572	-6.15262	7.62E-10	1.45E-08	0	0	0	4.164539	3.955229	4.391288
AT1G42367	11.15259	-3.529274579	0.578702	-6.0986	1.07E-09	2.00E-08	0	0	0	5.437371	3.710678	3.753887
AT1G54430	11.53868	-3.527397112	0.550331	-6.40959	1.46E-10	3.06E-09	1.082007	0	0	4.626795	4.164273	4.831847
AT4G06702	9.949689	-3.52727803	0.577211	-6.1109	9.91E-10	1.85E-08	0	0	0	5.123743	3.710678	3.881636
AT2G05000	12.94335	-3.525960652	0.580521	-6.07379	1.25E-09	2.31E-08	0	0	0	2.604042	5.123469	5.31183
AT3G42181	17.33479	-3.518097774	0.583311	-6.03126	1.63E-09	2.96E-08	0	0	0	6.332435	4.164273	3.08651
AT3G30823	10.88364	-3.516683083	0.578888	-6.0749	1.24E-09	2.30E-08	0	0	0	5.379864	3.416038	3.998987
AT3G57520	4407.309	-3.512732436	0.141791	-24.774	1.71E-135	5.49E-132	9.503666	9.47722	9.28483	12.97007	13.22264	12.73231
AT1G05147	36.59309	-3.503191721	0.426228	-8.21905	2.05E-16	7.77E-15	2.719161	2.419482	1.161466	5.913254	6.646151	5.671366
AT1G43590	68.23316	-3.500057452	0.323238	-10.8281	2.53E-27	2.05E-25	3.848373	3.356609	2.413855	7.127736	6.841348	7.032351
AT2G13430	28.78206	-3.494282304	0.440881	-7.92568	2.27E-15	7.86E-14	2.591298	1.666709	1.161466	5.99213	5.997325	5.31183
AT2G02205	15.88582	-3.488971403	0.583267	-5.98177	2.21E-09	3.95E-08	0	0	0	2.133098	5.760417	5.31183
AT4G04945	9.015737	-3.487811511	0.577061	-6.04409	1.50E-09	2.75E-08	0	0	0	4.812122	3.955229	3.753887
AT1G38167	13.28944	-3.482941615	0.582058	-5.98384	2.18E-09	3.91E-08	0	0	0	5.872138	3.416038	3.753887
AT1G78350	12.29228	-3.47916684	0.581207	-5.98611	2.15E-09	3.86E-08	0	0	0	2.604042	5.310931	4.953151
AT2G15800	58.53319	-3.470068077	0.415514	-8.35126	6.75E-17	2.65E-15	3.22918	3.194694	2.032847	7.46844	5.883728	6.620982
AT5G26270	181.761	-3.469700538	0.578808	-5.99456	2.04E-09	3.68E-08	2.450992	2.264856	2.032847	5.545934	9.115929	8.908815
AT4G04426	10.0131	-3.467766735	0.565575	-6.1314	8.71E-10	1.64E-08	0	0.626414	0	4.896561	3.955229	4.107507
AT1G27570	25.34111	-3.467677433	0.566027	-6.12635	8.99E-10	1.69E-08	1.082007	1.06174	0	2.604042	6.336481	6.04333
AT4G02795	137.261	-3.465599149	0.228376	-15.175	5.18E-52	1.50E-49	4.509766	4.284411	4.53998	8.224096	7.909492	7.811102
AT3G31440	11.31867	-3.463667004	0.553352	-6.25942	3.86E-10	7.61E-09	0	1.06174	0	4.021447	4.786789	4.699401
AT5G28524	8.846814	-3.462843375	0.577524	-5.99602	2.02E-09	3.65E-08	0	0	0	3.480233	4.654502	4.302755
AT3G05595	12.67838	-3.459007995	0.572411	-6.04288	1.51E-09	2.76E-08	0	0.626414	0	2.958503	5.220243	5.065041
AT3G21570	9.18913	-3.450577262	0.57865	-5.96315	2.47E-09	4.39E-08	0	0	0	3.242825	4.654502	4.553554
AT2G12260	8.215255	-3.447059269	0.577419	-5.96977	2.38E-09	4.23E-08	0	0	0	3.862584	3.955229	4.474702
AT3G42650	12.28848	-3.440434667	0.582305	-5.9083	3.46E-09	5.96E-08	0	0	0	5.545934	4.50885	2.85705
AT4G06735	16.62853	-3.439225715	0.584871	-5.88031	4.09E-09	6.98E-08	0	0	0	6.270495	2.545235	4.302755
AT1G52850	350.5032	-3.432665812	0.58982	-5.81986	5.89E-09	9.83E-08	0.640149	0	0	7.03764	9.841749	10.04502
AT1G22120	8.994222	-3.429829523	0.579181	-5.92186	3.18E-09	5.54E-08	0	0	0	3.242825	4.50885	4.62832
AT5G29058	8.980953	-3.425227195	0.579188	-5.91384	3.34E-09	5.78E-08	0	0	0	3.242825	4.654502	4.474702
AT5G39520	178.821	-3.425186249	0.230456	-14.8626	5.76E-50	1.51E-47	5.037115	4.507728	4.93943	8.059868	8.644055	8.347651
AT2G12460	26.92456	-3.424317392	0.488577	-7.00875	2.40E-12	6.25E-11	1.92313	1.666709	1.513627	4.29471	6.292644	5.899804

AT2G06790	8.094384	-3.42089319	0.578277	-5.91566	3.31E-09	5.73E-08	0	0	0	4.021447	3.710678	4.474702
AT2G06425	8.363999	-3.418875375	0.578343	-5.9115	3.39E-09	5.85E-08	0	0	0	4.524365	4.164273	3.613718
AT2G11640	10.40652	-3.416632881	0.581573	-5.87482	4.23E-09	7.20E-08	0	0	0	5.319969	3.045364	4.107507
AT1G07347	7.98054	-3.408031299	0.578028	-5.89596	3.72E-09	6.39E-08	0	0	0	3.862584	4.346824	3.998987
AT1G40075	19.84326	-3.393708314	0.576467	-5.88708	3.93E-09	6.72E-08	0.640149	0.626414	0	2.133098	5.625572	6.070398
AT1G34967	12.68854	-3.393419248	0.574526	-5.90646	3.50E-09	6.02E-08	0.640149	0	0	3.242825	5.625572	4.302755
AT5G35375	24.18782	-3.378758205	0.57452	-5.88101	4.08E-09	6.96E-08	1.082007	0	0.694589	2.133098	6.200761	6.096967
AT4G06650	9.50491	-3.378279529	0.581366	-5.81094	6.21E-09	1.03E-07	0	0	0	2.958503	4.907958	4.474702
AT3G43000	14.47794	-3.368654773	0.578486	-5.82322	5.77E-09	9.66E-08	0.640149	0	0	5.829815	4.654502	2.85705
AT4G06541	15.36552	-3.356452892	0.58611	-5.72666	1.02E-08	1.65E-07	0	0	0	6.172293	2.545235	4.107507
AT1G34610	8.906331	-3.35427951	0.567737	-5.90816	3.46E-09	5.96E-08	0.640149	0	0	3.862584	4.346824	4.391288
AT3G33575	13.98453	-3.349257118	0.57841	-5.79046	7.02E-09	1.16E-07	0	0.626414	0	5.25748	5.396255	2.584062
AT1G68050	102.6629	-3.346568762	0.315738	-10.5992	3.01E-26	2.30E-24	3.466342	4.158291	4.33422	6.921529	7.835043	7.803055
AT2G37125	10.4983	-3.346260371	0.583578	-5.73404	9.81E-09	1.58E-07	0	0	0	2.604042	4.654502	5.117892
AT5G33427	9.976895	-3.345466481	0.583098	-5.7374	9.61E-09	1.55E-07	0	0	0	5.319969	3.416038	3.613718
AT5G27160	15.60236	-3.336432464	0.580764	-5.7449	9.20E-09	1.49E-07	0.640149	0	0	2.133098	5.553109	5.482759
AT2G12540	10.9643	-3.335822606	0.54286	-6.1449	8.00E-10	1.51E-08	1.082007	0.626414	0	4.29471	4.50885	4.62832
AT4G07810	15.62889	-3.332761294	0.580978	-5.73647	9.67E-09	1.56E-07	0.640149	0	0	2.133098	5.396255	5.635566
AT3G15440	38.60537	-3.327733521	0.397758	-8.36623	5.95E-17	2.36E-15	3.140592	1.894767	2.413855	5.872138	6.610887	5.987621
AT2G12910	11.51216	-3.324304621	0.576139	-5.76997	7.93E-09	1.30E-07	0	0.626414	0	5.492674	3.955229	3.458449
AT2G12810	9.963626	-3.311241178	0.583952	-5.6704	1.42E-08	2.25E-07	0	0	0	5.319969	3.710678	3.284433
AT1G36470	7.637555	-3.311198603	0.581098	-5.69817	1.21E-08	1.93E-07	0	0	0	3.480233	4.164273	4.302755
AT2G06245	10.61536	-3.310250499	0.584572	-5.66269	1.49E-08	2.35E-07	0	0	0	5.492674	3.416038	3.458449
AT3G42445	9.130249	-3.308681192	0.583257	-5.67277	1.41E-08	2.22E-07	0	0	0	5.123743	3.416038	3.613718
AT5G04985	8.67342	-3.307702272	0.582592	-5.67756	1.37E-08	2.16E-07	0	0	0	4.896561	3.955229	3.284433
AT2G04365	7.712262	-3.296246642	0.581375	-5.66974	1.43E-08	2.26E-07	0	0	0	4.164539	4.346824	3.458449
AT4G07250	7.261675	-3.294326627	0.58113	-5.66883	1.44E-08	2.27E-07	0	0	0	4.021447	3.710678	4.107507
AT3G29695	7.752736	-3.283500097	0.582044	-5.64133	1.69E-08	2.64E-07	0	0	0	4.524365	3.955229	3.458449
AT2G09840	16.88487	-3.283454282	0.58785	-5.58553	2.33E-08	3.58E-07	0	0	0	6.391825	3.710678	2.85705
AT3G42353	7.591391	-3.282026226	0.581734	-5.6418	1.68E-08	2.63E-07	0	0	0	4.29471	4.164273	3.458449
AT1G48285	17.55682	-3.282024996	0.588052	-5.58118	2.39E-08	3.66E-07	0	0	0	1.429371	5.310931	6.04333
AT4G03900	7.174368	-3.28011116	0.581314	-5.64258	1.68E-08	2.62E-07	0	0	0	3.862584	3.955229	3.998987
AT3G31955	7.348981	-3.277026102	0.58198	-5.63082	1.79E-08	2.79E-07	0	0	0	4.164539	3.416038	4.208431
AT3G42721	7.187637	-3.271409496	0.581808	-5.62284	1.88E-08	2.92E-07	0	0	0	3.862584	3.710678	4.208431

AT2G13000	11.79117	-3.270020969	0.586069	-5.57959	2.41E-08	3.69E-07	0	0	0	5.19216	5.019734	2.247075
AT1G40072	9.486502	-3.269917428	0.584646	-5.59298	2.23E-08	3.43E-07	0	0	0	5.25748	3.416038	3.458449
AT5G04965	11.25383	-3.263720476	0.586089	-5.56864	2.57E-08	3.92E-07	0	0	0	5.646896	3.416038	3.284433
AT5G05005	11.25383	-3.263720476	0.586089	-5.56864	2.57E-08	3.92E-07	0	0	0	5.646896	3.416038	3.284433
AT2G05950	14.54332	-3.26250043	0.521525	-6.25569	3.96E-10	7.76E-09	1.419792	1.06174	0.694589	5.379864	3.955229	4.893774
AT1G26558	113.7048	-3.254951061	0.384965	-8.4552	2.79E-17	1.13E-15	4.056385	3.75549	4.420076	6.332435	8.137237	8.100899
AT5G38005	69.57464	-3.250171608	0.473362	-6.86614	6.60E-12	1.64E-10	3.140592	3.012284	3.176244	4.722432	7.707307	7.341001
AT4G03950	18.92254	-3.246353665	0.580751	-5.58992	2.27E-08	3.49E-07	0.640149	0.626414	0	6.530411	3.045364	3.881636
AT4G06603	48.5154	-3.243955205	0.346997	-9.34867	8.88E-21	4.73E-19	2.836608	3.502171	2.714973	6.773083	6.499609	6.17386
AT5G38190	10.99572	-3.239844044	0.586368	-5.52527	3.29E-08	4.95E-07	0	0	0	2.133098	5.123469	4.893774
AT5G32436	15.22706	-3.23772449	0.58815	-5.50493	3.69E-08	5.52E-07	0	0	0	1.429371	5.553109	5.482759
AT5G60100	101.2135	-3.235095976	0.297709	-10.8666	1.66E-27	1.36E-25	4.194811	4.362754	3.806003	6.921529	7.835043	7.720024
AT2G13930	7.113599	-3.234881943	0.582893	-5.5497	2.86E-08	4.34E-07	0	0	0	3.684038	3.710678	4.302755
AT1G43060	36.70382	-3.23134805	0.525245	-6.15207	7.65E-10	1.45E-08	2.450992	2.091646	1.161466	3.684038	6.499609	6.793345
AT3G28923	7.543222	-3.230542589	0.58382	-5.53345	3.14E-08	4.74E-07	0	0	0	3.684038	3.416038	4.62832
AT3G09170	8.207761	-3.218892902	0.572302	-5.62446	1.86E-08	2.89E-07	0.640149	0	0	3.684038	4.346824	4.208431
AT1G08740	7.671788	-3.213353548	0.58377	-5.50449	3.70E-08	5.52E-07	0	0	0	3.684038	4.654502	3.458449
AT3G26530	7.671788	-3.213353548	0.58377	-5.50449	3.70E-08	5.52E-07	0	0	0	3.684038	4.654502	3.458449
AT5G44890	7.671788	-3.213353548	0.58377	-5.50449	3.70E-08	5.52E-07	0	0	0	3.684038	4.654502	3.458449
AT4G06578	7.026292	-3.209033103	0.583432	-5.50027	3.79E-08	5.65E-07	0	0	0	3.480233	3.955229	4.208431
AT5G27180	14.39435	-3.196834036	0.588567	-5.43155	5.59E-08	8.13E-07	0	0	0	1.429371	5.553109	5.31183
AT2G15410	6.758013	-3.185850556	0.583556	-5.45938	4.78E-08	7.02E-07	0	0	0	3.862584	3.955229	3.753887
AT4G01980	21.71668	-3.183229072	0.506142	-6.28921	3.19E-10	6.38E-09	1.693301	1.666709	1.513627	6.270495	4.786789	4.474702
AT3G31442	9.713356	-3.178959466	0.565177	-5.62471	1.86E-08	2.89E-07	0	1.06174	0	3.684038	4.786789	4.302755
AT3G47320	48.14708	-3.163636177	0.59116	-5.35158	8.72E-08	1.23E-06	0	0	0	4.29471	7.11744	7.059624
AT2G10480	6.724449	-3.155970598	0.584605	-5.39846	6.72E-08	9.68E-07	0	0	0	4.164539	3.416038	3.881636
AT1G36920	14.2988	-3.149823062	0.578502	-5.4448	5.19E-08	7.60E-07	0	0.626414	0.694589	2.133098	5.220243	5.522505
AT5G38595	13.36673	-3.144458412	0.589084	-5.33788	9.40E-08	1.32E-06	0	0	0	1.429371	5.476813	5.168875
AT3G33172	17.45997	-3.139435637	0.568836	-5.51905	3.41E-08	5.12E-07	0	1.395705	0.694589	5.379864	5.82339	2.85705
AT2G12320	9.547271	-3.137967331	0.587639	-5.33995	9.30E-08	1.31E-06	0	0	0	5.319969	3.710678	2.85705
AT4G07560	8.801201	-3.130658983	0.587369	-5.32997	9.82E-08	1.38E-06	0	0	0	5.19216	3.045364	3.458449
AT5G27500	12.96364	-3.126901056	0.589237	-5.30669	1.12E-07	1.56E-06	0	0	0	1.429371	5.396255	5.168875
AT2G04310	7.70773	-3.12666905	0.57464	-5.44109	5.30E-08	7.73E-07	0	0	0.694589	4.29471	3.955229	3.753887
AT1G47657	11.51596	-3.112046224	0.583604	-5.33246	9.69E-08	1.36E-06	0	0.626414	0	2.133098	5.019734	5.117892

AT3G06505	12.631	-3.109406142	0.539833	-5.75994	8.41E-09	1.38E-07	0	1.395705	1.161466	5.25748	4.164273	4.302755
AT3G30418	8.167839	-3.103249691	0.578345	-5.36574	8.06E-08	1.15E-06	0.640149	0	0	4.626795	3.045364	4.208431
AT5G32420	8.23677	-3.102718818	0.587535	-5.28091	1.29E-07	1.78E-06	0	0	0	5.051918	3.045364	3.458449
AT5G32103	17.64436	-3.099206531	0.583577	-5.31071	1.09E-07	1.53E-06	0	0.626414	0.694589	1.429371	5.883728	5.522505
AT1G54420	6.415029	-3.090381946	0.585949	-5.27415	1.33E-07	1.85E-06	0	0	0	3.480233	3.710678	4.107507
AT5G32434	6.415029	-3.090381946	0.585949	-5.27415	1.33E-07	1.85E-06	0	0	0	3.480233	3.710678	4.107507
AT4G04293	33.66428	-3.086520941	0.47527	-6.49425	8.34E-11	1.81E-09	1.92313	2.803425	2.235893	6.795248	5.760417	4.767144
AT3G33154	10.61754	-3.078259517	0.573889	-5.36386	8.15E-08	1.16E-06	0.640149	0.626414	0	5.051918	4.654502	2.85705
AT1G21020	7.181395	-3.068866846	0.58696	-5.22841	1.71E-07	2.32E-06	0	0	0	3.480233	4.654502	3.284433
AT3G42356	8.169758	-3.067036865	0.588033	-5.21575	1.83E-07	2.48E-06	0	0	0	4.812122	4.164273	2.584062
AT1G35614	12.83508	-3.065403994	0.590106	-5.19467	2.05E-07	2.75E-06	0	0	0	1.429371	4.786789	5.635566
AT1G50860	6.549168	-3.063565776	0.586847	-5.22039	1.79E-07	2.42E-06	0	0	0	3.242825	3.710678	4.302755
AT3G31410	8.387492	-3.055559997	0.580763	-5.26128	1.43E-07	1.97E-06	0	0.626414	0	4.896561	3.045364	3.881636
AT5G29032	6.933295	-3.046648906	0.58746	-5.18614	2.15E-07	2.87E-06	0	0	0	4.524365	3.710678	3.08651
AT5G34834	9.950566	-3.046017216	0.559662	-5.4426	5.25E-08	7.68E-07	0	1.06174	0.694589	3.480233	4.786789	4.474702
AT3G42256	23.47683	-3.039294639	0.535532	-5.67528	1.38E-08	2.19E-07	1.693301	1.395705	1.79647	6.607592	4.50885	4.107507
AT5G35057	656.002	-3.031289858	0.592196	-5.11873	3.08E-07	4.00E-06	0	0	0	7.755209	10.73849	10.9762
AT4G07939	9.281639	-3.031238869	0.583024	-5.19917	2.00E-07	2.69E-06	0	0.626414	0	5.19216	3.710678	3.08651
AT3G31908	7.986114	-3.021996469	0.5891	-5.12985	2.90E-07	3.79E-06	0	0	0	3.684038	2.545235	5.01018
AT3G32290	8.048888	-3.020331108	0.588869	-5.12904	2.91E-07	3.81E-06	0	0	0	4.896561	3.955229	2.584062
AT4G07485	9.231492	-3.019641153	0.589597	-5.12154	3.03E-07	3.94E-06	0	0	0	5.379864	3.045364	3.08651
AT3G33528	13.62362	-3.017731012	0.555384	-5.43359	5.52E-08	8.05E-07	1.082007	0.626414	1.161466	5.646896	3.045364	4.553554
AT2G15815	7.465989	-3.014141415	0.579286	-5.2032	1.96E-07	2.64E-06	0	0	0.694589	4.524365	3.416038	3.753887
AT5G32630	35.22661	-3.010235297	0.589208	-5.10895	3.24E-07	4.19E-06	0.640149	1.06174	0	0	6.336481	7.032351
AT4G19239	6.254352	-3.002526271	0.58758	-5.10999	3.22E-07	4.17E-06	0	0	0	3.684038	4.164273	3.284433
AT3G33178	10.96235	-3.000845182	0.56683	-5.29409	1.20E-07	1.66E-06	0.640149	0.626414	0.694589	4.896561	4.907958	2.85705
AT1G47660	10.72677	-2.996255982	0.590401	-5.07495	3.88E-07	4.96E-06	0	0	0	1.429371	5.019734	5.01018
AT3G27473	12.82813	-2.984249192	0.519665	-5.74264	9.32E-09	1.51E-07	1.082007	0.626414	1.79647	5.051918	4.164273	4.62832
AT3G30400	6.113186	-2.977972562	0.588291	-5.06208	4.15E-07	5.28E-06	0	0	0	4.164539	3.045364	3.753887
AT2G29240	7.361783	-2.976907305	0.58013	-5.13145	2.88E-07	3.77E-06	0.640149	0	0	3.684038	4.50885	3.458449
AT5G17460	89.5997	-2.972905501	0.319786	-9.29654	1.45E-20	7.59E-19	4.056385	4.113676	4.377786	6.530411	7.638931	7.622806
AT5G33384	7.160516	-2.971755705	0.580382	-5.12035	3.05E-07	3.96E-06	0.640149	0	0	4.414102	3.710678	3.458449
AT2G10010	6.563773	-2.967269897	0.588694	-5.04043	4.64E-07	5.87E-06	0	0	0	4.29471	3.955229	2.85705
AT3G42083	14.3893	-2.966820663	0.579258	-5.12176	3.03E-07	3.94E-06	0.640149	0.626414	0.694589	6.066916	2.545235	3.881636

AT3G33160	14.97013	-2.962665316	0.591431	-5.00932	5.46E-07	6.83E-06	0	0	0	6.205775	3.955229	1.806583
AT4G05510	17.339	-2.961154516	0.586697	-5.04716	4.48E-07	5.68E-06	0	0	1.161466	6.391825	2.545235	3.998987
AT5G00480	10.29715	-2.957214456	0.590727	-5.00606	5.56E-07	6.93E-06	0	0	0	1.429371	5.123469	4.767144
AT3G33377	7.386527	-2.949480582	0.581486	-5.07232	3.93E-07	5.02E-06	0	0.626414	0	4.164539	4.346824	3.08651
AT3G34299	7.386527	-2.949480582	0.581486	-5.07232	3.93E-07	5.02E-06	0	0.626414	0	4.164539	4.346824	3.08651
AT1G15380	63.70528	-2.938363717	0.355195	-8.27255	1.31E-16	5.04E-15	4.360866	3.431225	2.844834	6.680852	7.166841	6.692417
AT4G06585	6.875839	-2.936641551	0.581079	-5.05378	4.33E-07	5.50E-06	0	0.626414	0	4.29471	3.710678	3.458449
AT3G33136	15.67478	-2.935481478	0.586715	-5.00325	5.64E-07	7.03E-06	0	1.06174	0	5.786214	5.220243	1.806583
AT4G28960	9.920596	-2.934219413	0.590921	-4.9655	6.85E-07	8.44E-06	0	0	0	1.429371	4.786789	5.01018
AT3G42993	6.8783	-2.933868584	0.581065	-5.04913	4.44E-07	5.62E-06	0.640149	0	0	4.29471	3.710678	3.458449
AT1G61510	6.596001	-2.930731827	0.589573	-4.97094	6.66E-07	8.21E-06	0	0	0	2.604042	3.955229	4.391288
AT4G05592	10.95132	-2.930310918	0.591206	-4.9565	7.18E-07	8.80E-06	0	0	0	5.694846	1.77337	3.753887
AT4G06474	9.757511	-2.928834601	0.579396	-5.05498	4.30E-07	5.47E-06	0.640149	0.626414	0	5.319969	3.045364	3.613718
AT1G35590	5.905009	-2.923907162	0.58914	-4.96301	6.94E-07	8.54E-06	0	0	0	4.164539	3.045364	3.613718
AT4G07937	6.556746	-2.920396715	0.589772	-4.95174	7.36E-07	9.00E-06	0	0	0	4.524365	2.545235	3.753887
AT5G36650	55.60166	-2.914816715	0.592314	-4.92107	8.61E-07	1.04E-05	0	0	0	4.29471	7.190921	7.416778
AT3G20340	165.3621	-2.911605911	0.314404	-9.2607	2.03E-20	1.06E-18	5.548318	4.541804	5.024124	7.788738	8.793078	7.850673
AT4G04530	7.152938	-2.906265746	0.582749	-4.98716	6.13E-07	7.60E-06	0.640149	0	0	2.958503	4.346824	4.107507
AT3G30690	8.901776	-2.901552641	0.591048	-4.90916	9.15E-07	1.10E-05	0	0	0	5.25748	1.77337	3.881636
AT2G40955	32.84942	-2.900129347	0.455557	-6.36612	1.94E-10	4.00E-09	2.719161	2.419482	2.714973	4.626795	5.941644	6.620982
AT2G01422	50.41606	-2.896083847	0.592336	-4.88926	1.01E-06	1.20E-05	0	0	0	4.164539	7.013254	7.307265
AT4G28970	118.3129	-2.889032433	0.592383	-4.87696	1.08E-06	1.28E-05	0	0	0	5.25748	8.497289	8.291245
AT1G44040	6.556195	-2.8885012	0.590044	-4.8954	9.81E-07	1.17E-05	0	0	0	2.604042	4.50885	3.753887
AT5G32825	22.48943	-2.887557775	0.589966	-4.89445	9.86E-07	1.18E-05	0.640149	0	0.694589	0	6.200761	5.958939
AT2G14350	8.326572	-2.886210062	0.585457	-4.92984	8.23E-07	9.99E-06	0	0	0.694589	5.051918	3.416038	3.08651
AT5G23240	131.497	-2.881448953	0.254416	-11.3258	9.78E-30	9.18E-28	4.88181	4.541804	5.204319	7.522586	7.865285	8.171351
AT5G32623	62.21899	-2.881159269	0.592264	-4.86465	1.15E-06	1.36E-05	0	0	0.694589	4.414102	7.214606	7.685425
AT2G04990	6.691553	-2.877630879	0.590426	-4.87382	1.09E-06	1.30E-05	0	0	0	4.722432	3.045364	3.08651
AT1G38460	10.17871	-2.864562092	0.591635	-4.84177	1.29E-06	1.51E-05	0	0	0	5.597298	1.77337	3.613718
AT2G11773	5.38741	-2.862246326	0.589743	-4.85338	1.21E-06	1.43E-05	0	0	0	3.480233	3.416038	3.753887
AT3G06115	7.343291	-2.85385029	0.59105	-4.82844	1.38E-06	1.61E-05	0	0	0	4.97633	2.545235	3.284433
AT3G28917	7.990181	-2.852077421	0.577601	-4.9378	7.90E-07	9.61E-06	0.640149	0.626414	0	4.812122	3.045364	3.753887
AT5G32490	7.330022	-2.8496939	0.591064	-4.8213	1.43E-06	1.66E-05	0	0	0	4.97633	3.045364	2.85705
AT5G57640	45.98899	-2.849655253	0.402696	-7.07643	1.48E-12	3.94E-11	3.605233	2.911631	3.074005	5.492674	7.040014	6.270397

AT5G32591	5.878471	-2.847944912	0.590251	-4.82497	1.40E-06	1.63E-05	0	0	0	4.164539	3.710678	2.85705
AT4G08092	5.689921	-2.844170036	0.590102	-4.8198	1.44E-06	1.67E-05	0	0	0	3.242825	4.164273	3.284433
AT3G45300	3240.753	-2.841197857	0.1634	-17.388	1.02E-67	5.80E-65	9.915985	9.388714	9.381634	12.29734	12.58099	12.5475
AT5G28430	54.3953	-2.835731514	0.511775	-5.54098	3.01E-08	4.55E-07	3.140592	3.194694	2.714973	3.684038	7.040014	7.352073
AT5G45085	5.770201	-2.832287813	0.590627	-4.79539	1.62E-06	1.87E-05	0	0	0	3.862584	2.545235	4.107507
AT2G12050	5.717127	-2.829051312	0.590371	-4.79199	1.65E-06	1.90E-05	0	0	0	3.862584	3.955229	2.85705
AT2G14010	8.227388	-2.823423178	0.587267	-4.80774	1.53E-06	1.77E-05	0.640149	0	0	2.133098	4.654502	4.474702
AT5G34835	6.612432	-2.818312103	0.583768	-4.82779	1.38E-06	1.61E-05	0	0	0.694589	3.242825	4.346824	3.613718
AT2G01021	48409.17	-2.816848554	0.397943	-7.07852	1.46E-12	3.89E-11	13.09401	12.86828	13.50109	16.36478	17.01876	15.47261
AT5G32566	217.3743	-2.812918788	0.592362	-4.74865	2.05E-06	2.33E-05	0	0	0	6.030007	9.363777	9.18751
AT4G03830	9.970536	-2.80871499	0.591977	-4.74463	2.09E-06	2.37E-05	0	0	0	5.597298	1.77337	3.458449
AT1G39990	7.417329	-2.795888492	0.591633	-4.72572	2.29E-06	2.58E-05	0	0	0	5.051918	2.545235	3.08651
AT4G06604	5.488654	-2.795146891	0.590839	-4.73081	2.24E-06	2.52E-05	0	0	0	4.164539	3.045364	3.284433
AT2G10000	6.357422	-2.793155777	0.583965	-4.78309	1.73E-06	1.98E-05	0	0	0.694589	3.684038	4.164273	3.284433
AT5G23235	116.5041	-2.788420881	0.271167	-10.2831	8.40E-25	6.05E-23	4.738256	4.400386	5.15509	7.292624	7.707307	7.998924
AT2G21660	2116.119	-2.784290204	0.239589	-11.6211	3.22E-31	3.28E-29	8.329003	9.465063	8.866268	11.56282	11.94249	12.05208
AT3G59930	57.04168	-2.773030262	0.351384	-7.89175	2.98E-15	1.02E-13	4.104018	3.634384	3.271714	7.212535	6.247434	6.404074
AT1G33570	97.23937	-2.771920546	0.592328	-4.67971	2.87E-06	3.19E-05	0	0	0	4.896561	8.047769	8.189982
AT5G35061	6.300516	-2.771599765	0.59148	-4.68587	2.79E-06	3.11E-05	0	0	0	2.133098	4.164273	4.208431
AT5G28926	5.326641	-2.770383105	0.591131	-4.68658	2.78E-06	3.10E-05	0	0	0	3.242825	3.045364	4.107507
AT4G06590	6.201161	-2.763144317	0.591532	-4.67116	2.99E-06	3.31E-05	0	0	0	4.626795	3.045364	2.85705
AT5G33150	6.201161	-2.763144317	0.591532	-4.67116	2.99E-06	3.31E-05	0	0	0	4.626795	3.045364	2.85705
AT3G29792	11.0668	-2.762241922	0.573056	-4.8202	1.43E-06	1.67E-05	0.640149	1.06174	0.694589	5.545934	3.416038	3.284433
AT2G42530	516.0136	-2.760085218	0.269024	-10.2596	1.07E-24	7.67E-23	7.003778	6.689579	7.052287	8.942675	10.18526	10.07874
AT3G33205	13.97319	-2.754585845	0.540044	-5.10067	3.38E-07	4.36E-06	1.693301	0.626414	1.513627	4.524365	5.553109	3.613718
AT1G40076	4.971056	-2.751362058	0.591039	-4.65513	3.24E-06	3.55E-05	0	0	0	3.480233	3.416038	3.458449
AT3G43290	5.206438	-2.748651684	0.591242	-4.64895	3.34E-06	3.65E-05	0	0	0	4.021447	3.045364	3.284433
AT3G29732	8.658606	-2.743504569	0.573081	-4.78729	1.69E-06	1.94E-05	0	0	1.513627	3.684038	3.955229	4.62832
AT2G11410	5.119132	-2.742119538	0.591218	-4.63809	3.52E-06	3.83E-05	0	0	0	3.862584	3.416038	3.08651
AT2G06335	7.773582	-2.741529218	0.59207	-4.63041	3.65E-06	3.96E-05	0	0	0	5.19216	2.545235	2.85705
AT1G34590	229.8556	-2.73056217	0.592243	-4.61055	4.02E-06	4.31E-05	0	0	0.694589	5.953231	9.431032	9.297557
AT4G09370	7.670451	-2.728630574	0.59212	-4.60824	4.06E-06	4.35E-05	0	0	0	1.429371	4.346824	4.699401
AT3G42057	12.15263	-2.722953697	0.544301	-5.00266	5.65E-07	7.04E-06	1.92313	0	1.161466	4.626795	5.019734	3.881636
AT1G39190	6.075207	-2.720491937	0.585635	-4.64537	3.39E-06	3.71E-05	0	0	0.694589	3.480233	4.164273	3.284433

AT2G10540	12.13054	-2.719895697	0.588995	-4.61786	3.88E-06	4.18E-05	0	1.06174	0	1.429371	5.220243	5.117892
AT5G34623	6.478444	-2.719331581	0.587536	-4.62837	3.69E-06	3.99E-05	0	0.626414	0	2.604042	3.955229	4.302755
AT4G05593	8.915713	-2.719121788	0.592279	-4.59095	4.41E-06	4.69E-05	0	0	0	5.437371	1.77337	3.284433
AT4G15096	25.62242	-2.715326602	0.487932	-5.56497	2.62E-08	4.00E-07	2.450992	2.264856	2.413855	4.021447	5.553109	6.338783
AT1G24260	10.92815	-2.708085899	0.563767	-4.80355	1.56E-06	1.80E-05	1.082007	0.626414	1.161466	2.604042	5.123469	4.62832
AT5G33387	5.89743	-2.705263841	0.591919	-4.57033	4.87E-06	5.12E-05	0	0	0	2.133098	3.955229	4.208431
AT2G10070	11.19248	-2.70442838	0.591354	-4.57328	4.80E-06	5.07E-05	0.640149	0	0	5.786214	1.77337	3.458449
AT5G28230	8.983599	-2.703120278	0.590457	-4.57801	4.69E-06	4.96E-05	0	0.626414	0	1.429371	4.654502	4.831847
AT3G05955	10.8952	-2.694619683	0.535407	-5.03285	4.83E-07	6.09E-06	0	1.395705	1.79647	4.524365	4.50885	4.208431
AT2G36270	16.38157	-2.691760413	0.46795	-5.75224	8.81E-09	1.44E-07	2.121327	1.395705	2.235893	4.524365	5.123469	5.117892
AT3G42090	5.712595	-2.664721916	0.586851	-4.54071	5.61E-06	5.83E-05	0	0	0.694589	4.021447	3.416038	3.284433
AT1G37063	5.992983	-2.660225746	0.592192	-4.49217	7.05E-06	7.22E-05	0	0	0	4.626795	3.045364	2.584062
AT5G47240	85.70836	-2.659040177	0.261879	-10.1537	3.19E-24	2.23E-22	4.321109	4.324114	4.786189	6.921529	7.349087	7.395531
AT5G49160	621.2418	-2.656109467	0.231215	-11.4876	1.52E-30	1.49E-28	6.851629	7.723975	7.258633	10.304	10.20312	9.662479
AT3G33130	12.92967	-2.65192295	0.577416	-4.59274	4.37E-06	4.65E-05	1.419792	0.626414	0.694589	5.872138	3.416038	3.08651
AT4G14120	1780.357	-2.645311716	0.226849	-11.6611	2.01E-31	2.10E-29	8.965009	8.521482	9.01509	11.02379	12.02852	11.57162
AT5G37665	8.593781	-2.643720891	0.591085	-4.47265	7.73E-06	7.82E-05	0	0.626414	0	1.429371	4.346824	4.953151
AT4G13540	67.59473	-2.633712947	0.362262	-7.27019	3.59E-13	1.01E-11	4.056385	4.507728	3.271714	6.301797	7.432247	6.793345
AT3G43310	5.085568	-2.631732328	0.592176	-4.44417	8.82E-06	8.83E-05	0	0	0	4.164539	2.545235	3.284433
AT1G31095	4.957119	-2.62521578	0.592181	-4.43313	9.29E-06	9.27E-05	0	0	0	2.604042	3.416038	3.998987
AT4G07526	6.940205	-2.622994882	0.592383	-4.42787	9.52E-06	9.48E-05	0	0	0	4.97633	1.77337	3.284433
AT1G41726	9.910435	-2.618234506	0.592359	-4.42001	9.87E-06	9.80E-05	0	0	0	5.694846	1.77337	2.85705
AT5G38365	5.718954	-2.600843189	0.588277	-4.42112	9.82E-06	9.75E-05	0	0	0.694589	2.958503	4.164273	3.458449
AT4G14130	4892.723	-2.600604314	0.235014	-11.0657	1.84E-28	1.61E-26	10.49711	9.932994	10.51832	12.49817	13.52543	12.94697
AT5G35794	5.272898	-2.599693255	0.592314	-4.38905	1.14E-05	0.0001119	0	0	0	2.133098	3.955229	3.881636
AT2G22710	25.2579	-2.599575319	0.509963	-5.09758	3.44E-07	4.43E-06	2.591298	2.559128	1.79647	6.582321	4.346824	4.699401
AT2G11522	6.718759	-2.596096216	0.592393	-4.38239	1.17E-05	1.15E-04	0	0	0	4.97633	2.545235	2.584062
AT3G33142	6.123527	-2.595942215	0.589309	-4.40506	1.06E-05	0.0001046	0	0.626414	0	3.684038	4.346824	2.584062
AT3G31915	5.501254	-2.588698221	0.592379	-4.37001	1.24E-05	1.21E-04	0	0	0	3.684038	1.77337	4.302755
AT3G06495	4.790084	-2.583775946	0.592301	-4.36227	1.29E-05	0.0001251	0	0	0	4.021447	3.045364	2.85705
AT2G12040	6.101161	-2.580023831	0.582022	-4.43286	9.30E-06	9.28E-05	0.640149	0	0.694589	4.164539	3.045364	3.613718
AT5G34843	5.515859	-2.57841138	0.592382	-4.35262	1.35E-05	0.0001305	0	0	0	4.524365	2.545235	2.85705
AT2G00440	17.02561	-2.575530484	0.455804	-5.65052	1.60E-08	2.51E-07	1.693301	2.419482	2.235893	5.319969	4.654502	4.893774
AT2G13070	5.454538	-2.574391168	0.592372	-4.3459	1.39E-05	1.34E-04	0	0	0	2.133098	4.346824	3.458449

AT5G35791	5.527993	-2.572690926	0.58888	-4.36879	1.25E-05	1.22E-04	0.640149	0	0	3.684038	3.955229	2.85705
AT2G10250	16.33851	-2.568954009	0.476889	-5.3869	7.17E-08	1.03E-06	1.419792	2.419482	2.032847	4.414102	5.396255	4.831847
AT3G45775	12.09993	-2.567748123	0.591152	-4.34363	1.40E-05	1.35E-04	0	0.626414	0.694589	5.913254	3.416038	1.806583
AT3G00620	21.52593	-2.566259022	0.417696	-6.14384	8.05E-10	1.52E-08	2.450992	2.803425	2.235893	5.597298	5.123469	5.117892
AT5G37390	6.226478	-2.562952023	0.592387	-4.32649	1.52E-05	0.0001454	0	0	0	1.429371	4.164273	4.302755
AT1G11810	5.332416	-2.560919533	0.589291	-4.34576	1.39E-05	0.0001343	0.640149	0	0	2.958503	3.416038	3.998987
AT5G15995	17.10439	-2.559318862	0.591212	-4.32894	1.50E-05	0.000144	0.640149	0	1.161466	0	5.625572	5.740407
AT2G15890	5072.829	-2.559109108	0.142634	-17.9418	5.56E-72	3.40E-69	10.46441	10.35627	10.63961	12.8034	13.27251	13.14855
AT5G24470	193.5549	-2.558665807	0.23586	-10.8482	2.03E-27	1.66E-25	5.108917	6.04978	5.900872	8.349224	8.516472	8.296986
AT2G11770	5.79174	-2.555997881	0.581669	-4.39425	1.11E-05	1.09E-04	0.640149	0	0.694589	3.480233	3.416038	3.881636
AT4G06533	6.154328	-2.555797231	0.592381	-4.31445	1.60E-05	1.52E-04	0	0	0	4.812122	2.545235	2.584062
AT5G31302	6.154328	-2.555797231	0.592381	-4.31445	1.60E-05	0.0001525	0	0	0	4.812122	2.545235	2.584062
AT5G35048	6.198689	-2.554255449	0.590537	-4.32531	1.52E-05	1.46E-04	0.640149	0	0	2.133098	3.955229	4.302755
AT5G56870	3218.963	-2.552355602	0.219111	-11.6487	2.33E-31	2.41E-29	10.27906	9.374603	9.562424	12.2313	12.69903	12.34419
AT5G01080	10.05227	-2.55025532	0.586882	-4.34543	1.39E-05	0.0001344	0.640149	0.626414	0.694589	1.429371	4.164273	5.356498
AT1G07343	6.66937	-2.548343605	0.592339	-4.30217	1.69E-05	1.60E-04	0	0	0	1.429371	3.710678	4.767144
AT3G42110	5.074126	-2.540643388	0.588759	-4.31526	1.59E-05	0.0001521	0	0	0.694589	3.480233	3.416038	3.458449
AT2G23710	6.018301	-2.534992023	0.592358	-4.2795	1.87E-05	1.76E-04	0	0	0	1.429371	4.164273	4.208431
AT1G36922	6.461193	-2.530969979	0.592308	-4.27306	1.93E-05	0.0001806	0	0	0	1.429371	3.710678	4.699401
AT4G09430	9.929713	-2.530575437	0.533702	-4.74155	2.12E-06	2.40E-05	1.082007	1.395705	1.513627	4.414102	3.416038	4.699401
AT5G34790	103.3791	-2.528163688	0.287765	-8.78551	1.56E-18	6.99E-17	4.768132	4.816916	4.880072	7.522586	7.923931	6.841279
AT3G06355	28019.63	-2.522475094	0.563272	-4.47826	7.53E-06	7.65E-05	11.46817	10.47014	12.52638	15.56671	16.54516	13.75336
AT5G35796	4.869812	-2.520385507	0.592389	-4.25461	2.09E-05	0.0001943	0	0	0	2.133098	3.710678	3.881636
AT2G04330	4.588265	-2.517404147	0.592392	-4.24956	2.14E-05	0.0001984	0	0	0	2.958503	3.955229	2.85705
AT1G73120	88.25249	-2.515355152	0.557238	-4.51397	6.36E-06	6.56E-05	3.466342	3.277922	4.040944	5.25748	8.301072	7.176347
AT1G21400	3048.344	-2.511997412	0.131302	-19.1315	1.38E-81	1.36E-78	9.901736	9.752886	9.748176	12.10279	12.54349	12.35764
AT1G18050	5.581102	-2.511735589	0.58269	-4.31059	1.63E-05	0.000155	0	0.626414	0.694589	3.480233	3.416038	3.753887
AT1G38390	4.951428	-2.511204797	0.592375	-4.23922	2.24E-05	2.07E-04	0	0	0	4.29471	2.545235	2.85705
AT3G45446	4.951428	-2.511204797	0.592375	-4.23922	2.24E-05	0.0002067	0	0	0	4.29471	2.545235	2.85705
AT3G30700	7.088281	-2.506210132	0.592169	-4.23226	2.31E-05	2.13E-04	0	0	0	5.123743	1.77337	2.85705
AT3G29710	5.243316	-2.505605853	0.589892	-4.24757	2.16E-05	0.0002	0	0.626414	0	3.480233	3.955229	2.85705
AT3G33193	18.57042	-2.50497163	0.533494	-4.69541	2.66E-06	2.97E-05	1.082007	2.091646	2.413855	6.066916	4.654502	3.753887
AT3G33124	5.49568	-2.502161074	0.59234	-4.2242	2.40E-05	0.0002196	0	0	0	3.862584	4.164273	1.806583
AT5G44440	28.15411	-2.500950742	0.541513	-4.61845	3.87E-06	4.17E-05	3.312641	1.395705	1.513627	3.862584	6.610887	5.561185

AT3G33537	7.446898	-2.498130549	0.581715	-4.29443	1.75E-05	1.65E-04	0	1.395705	0	4.29471	4.164273	3.08651
AT1G36020	8.361327	-2.488440663	0.59193	-4.20394	2.62E-05	0.0002383	0	0	0	1.429371	3.045364	5.399825
AT5G28053	9.717679	-2.483764679	0.581543	-4.27099	1.95E-05	0.000182	0	1.06174	1.161466	2.133098	5.123469	4.302755
AT3G19390	45.84889	-2.480730042	0.329323	-7.53282	4.97E-14	1.52E-12	3.391538	3.91999	3.740762	5.99213	6.714193	6.198607
AT1G36080	5.615215	-2.470781631	0.592182	-4.17234	3.01E-05	2.71E-04	0	0	0	1.429371	3.955229	4.208431
AT3G30743	5.779055	-2.470548681	0.591028	-4.18009	2.91E-05	2.62E-04	0	0	0.694589	2.133098	4.164273	3.881636
AT2G33830	8176.052	-2.463630844	0.151521	-16.2594	1.92E-59	7.35E-57	11.44927	10.93586	11.34065	13.83491	13.86924	13.56711
AT3G05485	4.164883	-2.461797936	0.592335	-4.15609	3.24E-05	2.88E-04	0	0	0	3.480233	2.545235	3.458449
AT3G30680	4.164883	-2.461797936	0.592335	-4.15609	3.24E-05	0.0002876	0	0	0	3.480233	2.545235	3.458449
AT3G29639	28.00203	-2.460613672	0.44577	-5.51992	3.39E-08	5.10E-07	2.719161	2.803425	3.074005	6.448866	5.019734	5.01018
AT1G36763	6.228366	-2.46034203	0.592039	-4.15571	3.24E-05	2.88E-04	0	0	0	4.896561	2.545235	2.247075
AT1G40630	4.31296	-2.457487204	0.592309	-4.14899	3.34E-05	2.95E-04	0	0	0	3.862584	2.545235	3.08651
AT2G08685	24.63565	-2.457269481	0.39098	-6.2849	3.28E-10	6.53E-09	2.591298	2.803425	3.074005	5.694846	5.396255	5.265734
AT5G28824	8.214154	-2.447560668	0.590252	-4.14663	3.37E-05	2.98E-04	0	0.626414	0.694589	1.429371	5.019734	3.998987
AT5G35065	5.407038	-2.439424547	0.592049	-4.12031	3.78E-05	3.31E-04	0	0	0	1.429371	3.955229	4.107507
AT5G28773	10.73254	-2.439274964	0.592356	-4.11792	3.82E-05	0.0003345	0.640149	0	0	0	5.123469	4.953151
AT3G33115	5.946151	-2.439086244	0.59195	-4.12043	3.78E-05	3.31E-04	0	0	0	4.812122	2.545235	2.247075
AT3G31406	4.064308	-2.4388599	0.592309	-4.11755	3.83E-05	3.35E-04	0	0	0	3.242825	3.416038	2.85705
AT1G33580	78.31128	-2.437925447	0.590468	-4.12881	3.65E-05	3.21E-04	0	0	0	4.29471	7.772588	7.873905
AT1G23915	130.1135	-2.437924527	0.5904	-4.12927	3.64E-05	0.0003201	0	0	0	4.97633	8.626506	8.479713
AT5G29020	10.53763	-2.426955241	0.592338	-4.09725	4.18E-05	0.0003628	0.640149	0	0	0	5.019734	5.01018
AT3G41979	26417.61	-2.425970833	0.581677	-4.17065	3.04E-05	2.72E-04	10.99518	9.29567	12.27418	15.48118	16.49187	13.66484
AT2G10630	4.224985	-2.425135147	0.592169	-4.09534	4.22E-05	0.0003656	0	0	0	2.958503	2.545235	3.881636
AT5G04925	3.99027	-2.424235441	0.592264	-4.09317	4.26E-05	3.69E-04	0	0	0	2.958503	3.416038	3.08651
AT2G01020	26417.1	-2.423994716	0.581748	-4.16674	3.09E-05	2.76E-04	10.99518	9.291921	12.2758	15.4815	16.49172	13.66401
AT2G14040	5.36598	-2.417287969	0.591558	-4.08631	4.38E-05	3.78E-04	0.640149	0	0	2.133098	3.955229	3.881636
AT3G24514	4.608444	-2.415480271	0.592032	-4.07998	4.50E-05	3.88E-04	0	0	0	4.021447	1.77337	3.458449
AT1G34440	6.5012	-2.409028638	0.592173	-4.06812	4.74E-05	4.06E-04	0.640149	0	0	1.429371	4.50885	3.998987
AT1G20390	99.5742	-2.405269934	0.306497	-7.84761	4.24E-15	1.44E-13	4.768132	5.421786	4.145468	7.162256	7.411902	7.658921
AT3G42712	9.948522	-2.397130215	0.592112	-4.04844	5.16E-05	0.0004381	1.082007	0	0	5.597298	1.77337	3.284433
AT1G40123	4.030744	-2.395856702	0.592076	-4.04654	5.20E-05	0.0004408	0	0	0	3.684038	2.545235	3.08651
AT2G42540	443.0204	-2.394343385	0.255689	-9.36428	7.66E-21	4.10E-19	6.830546	7.147962	7.156022	8.768767	9.755573	9.9148
AT1G76410	117.5158	-2.387026184	0.241698	-9.87605	5.29E-23	3.36E-21	5.345282	4.949759	5.204319	7.338627	7.994038	7.538084
AT5G34780	116.679	-2.383159302	0.205811	-11.5793	5.24E-31	5.26E-29	5.177314	5.16182	5.251924	7.600176	7.788457	7.498771

AT1G42727	128.223	-2.378940273	0.589752	-4.0338	5.49E-05	4.63E-04	0	0	0	4.896561	8.438159	8.628279
AT1G07040	1004.656	-2.375346454	0.138571	-17.1417	7.25E-66	3.72E-63	8.214317	8.249814	8.478136	10.50398	10.87294	10.77185
AT4G28870	6.499631	-2.374886875	0.589771	-4.02679	5.65E-05	0.0004762	0	0	1.161466	2.958503	3.045364	4.62832
AT5G33355	8.607245	-2.37393494	0.552263	-4.29856	1.72E-05	1.63E-04	1.419792	1.06174	1.161466	4.722432	3.045364	3.998987
AT2G11670	4.104782	-2.372099281	0.591892	-4.00766	6.13E-05	0.0005133	0	0	0	3.862584	2.545235	2.85705
AT4G04223	150.452	-2.369698446	0.207848	-11.4011	4.13E-30	3.96E-28	5.857134	5.267421	5.547306	8.078063	7.865285	8.033723
AT2G12630	6.174623	-2.368071845	0.591329	-4.00466	6.21E-05	0.0005188	0	0	0	4.626795	3.710678	1.169325
AT5G52310	634.278	-2.367466056	0.433618	-5.4598	4.77E-08	7.01E-07	7.267032	7.529654	7.092067	8.986679	10.03372	10.7984
AT3G33076	9.32879	-2.361499809	0.524983	-4.49824	6.85E-06	7.03E-05	1.082007	1.666709	1.513627	4.021447	4.346824	4.107507
AT5G30400	9.689196	-2.355903434	0.592152	-3.97855	6.93E-05	0.0005738	0	0.626414	0	0	5.123469	4.62832
AT1G35370	8.371118	-2.355392146	0.589255	-3.99724	6.41E-05	5.33E-04	0	1.06174	0.694589	1.429371	4.346824	4.831847
AT2G45360	36.41566	-2.344938596	0.375601	-6.24317	4.29E-10	8.37E-09	4.104018	3.012284	2.963966	5.913254	5.941644	6.123056
AT4G26290	101.2292	-2.341777536	0.262596	-8.9178	4.76E-19	2.27E-17	5.42246	4.760154	4.685768	7.162256	7.529878	7.566881
AT3G15460	931.457	-2.340467335	0.163029	-14.3561	9.76E-47	2.07E-44	8.494525	8.112011	8.036685	10.3664	10.69916	10.74165
AT5G05130	137.4993	-2.340272509	0.206571	-11.3291	9.41E-30	8.86E-28	5.599069	5.384715	5.428035	7.600176	7.923931	8.02683
AT5G35608	9.675927	-2.335743457	0.592049	-3.94518	7.97E-05	6.50E-04	0	0.626414	0	0	5.220243	4.474702
AT1G53490	161.2118	-2.33112838	0.2392	-9.74553	1.93E-22	1.18E-20	5.977105	5.094538	5.839872	8.224096	7.938228	8.100899
AT1G37735	119.2163	-2.330121748	0.58914	-3.95512	7.65E-05	0.000627	0	0	0	9.11117	5.625572	6.85691
AT1G49680	5.01722	-2.328321651	0.591131	-3.93876	8.19E-05	6.66E-04	0	0	0	1.429371	3.416038	4.302755
AT1G07887	164.463	-2.325583704	0.249035	-9.3384	9.78E-21	5.16E-19	6.002463	5.023964	5.915727	8.224096	8.034523	8.100899
AT1G07337	5.655021	-2.324821063	0.590879	-3.93451	8.34E-05	6.77E-04	0	0	0	1.429371	3.045364	4.699401
AT3G32060	3.748529	-2.323954794	0.591583	-3.92836	8.55E-05	0.0006928	0	0	0	3.480233	2.545235	3.08651
AT5G32471	9.546543	-2.323029423	0.592061	-3.92363	8.72E-05	0.0007059	0	0	0.694589	0	4.654502	5.065041
AT2G06303	3.902963	-2.317476177	0.591539	-3.91771	8.94E-05	7.20E-04	0	0	0	2.604042	3.710678	2.85705
AT5G29037	3.902963	-2.317476177	0.591539	-3.91771	8.94E-05	7.20E-04	0	0	0	2.604042	3.710678	2.85705
AT3G33131	7.514425	-2.317434529	0.592317	-3.91249	9.13E-05	0.0007328	0	0.626414	0	3.684038	5.019734	1.169325
AT1G53480	165.595	-2.316235455	0.251113	-9.22387	2.87E-20	1.47E-18	6.014976	5.023964	5.944987	8.224096	8.034523	8.126918
AT3G06095	14.18957	-2.315022567	0.468688	-4.93937	7.84E-07	9.54E-06	2.121327	2.264856	2.032847	4.97633	4.164273	4.831847
AT5G23115	4.037103	-2.314321536	0.591418	-3.91318	9.11E-05	0.0007314	0	0	0	2.133098	3.710678	3.284433
AT4G03911	3.721323	-2.312121143	0.591475	-3.90908	9.26E-05	7.42E-04	0	0	0	2.604042	3.045364	3.458449
AT4G22485	49.05702	-2.305593198	0.304004	-7.58409	3.35E-14	1.04E-12	4.280226	3.970903	3.6007	6.332435	6.574739	6.270397
AT3G61060	633.4733	-2.305337911	0.295776	-7.79421	6.48E-15	2.16E-13	8.047708	7.207555	7.418413	9.150389	10.49187	10.24685
AT4G22475	58.955	-2.303446146	0.303008	-7.60193	2.92E-14	9.11E-13	4.676581	4.067637	3.806003	6.556599	6.871459	6.526408
AT5G15970	1468.836	-2.298867077	0.390116	-5.89278	3.80E-09	6.51E-08	9.346696	8.089149	8.271346	10.86127	11.43703	11.52835

AT2G12205	7.583145	-2.298824747	0.590165	-3.89522	9.81E-05	7.78E-04	0	0	0	0	4.50885	4.62832
AT1G40137	71.05864	-2.29878721	0.588777	-3.90434	9.45E-05	0.0007542	0	0	0	4.021447	7.804154	7.566881
AT4G35770	23246.87	-2.296128047	0.174047	-13.1926	9.68E-40	1.57E-37	13.02947	12.65681	12.9806	14.90613	15.57279	15.18081
AT4G05610	4.587597	-2.291397863	0.590862	-3.87806	1.05E-04	8.28E-04	0	0	0	1.429371	3.710678	3.881636
AT1G09225	9.21082	-2.289020406	0.592387	-3.86407	1.12E-04	0.000872	0.640149	0.626414	0	0	4.654502	4.953151
AT3G33148	7.002918	-2.287519104	0.592342	-3.86182	1.13E-04	8.79E-04	0	0	0.694589	3.862584	4.786789	1.169325
AT1G33450	8.703844	-2.282212095	0.591854	-3.85604	1.15E-04	9.00E-04	0.640149	0	0	0	4.654502	4.831847
AT4G06642	7.329146	-2.282007143	0.582623	-3.91678	8.97E-05	7.23E-04	1.419792	0.626414	0	2.604042	4.654502	3.753887
AT1G22990	33.02119	-2.274120287	0.375351	-6.05864	1.37E-09	2.52E-08	3.731919	3.502171	2.844834	6.138016	5.310931	5.958939
AT1G44070	3.896605	-2.272749169	0.590921	-3.84611	1.20E-04	9.32E-04	0	0	0	3.862584	2.545235	2.584062
AT5G29571	4.600865	-2.268639194	0.590505	-3.84186	1.22E-04	9.46E-04	0	0	0	1.429371	3.416038	4.107507
AT1G13930	3512.286	-2.267220044	0.188966	-11.9981	3.64E-33	4.15E-31	9.971612	10.1768	10.38856	12.07305	12.84227	12.53268
AT5G32481	5.943011	-2.266219321	0.592312	-3.82605	1.30E-04	1.00E-03	0.640149	0	0	1.429371	3.416038	4.62832
AT2G43795	6.286299	-2.262789679	0.577944	-3.91524	9.03E-05	7.27E-04	0	1.395705	0.694589	3.242825	3.955229	3.753887
AT1G37070	3.855463	-2.257597221	0.590686	-3.82199	0.000132	0.0010172	0	0	0	2.133098	3.045364	3.753887
AT2G12305	3.734592	-2.256043813	0.590698	-3.81929	1.34E-04	1.03E-03	0	0	0	2.604042	2.545235	3.753887
AT4G02490	7.401505	-2.249831953	0.589432	-3.81695	1.35E-04	1.03E-03	0	0	0	0	4.164273	4.831847
AT5G35120	5.284799	-2.248531851	0.589433	-3.81473	1.36E-04	0.0010423	0	0	1.161466	3.242825	3.955229	3.08651
AT1G72060	419.6	-2.246356446	0.284157	-7.90534	2.67E-15	9.19E-14	7.654621	6.545861	6.787392	9.885479	9.337051	9.037841
AT3G10985	1537.327	-2.241456123	0.190332	-11.7765	5.16E-32	5.59E-30	9.422626	8.598378	8.916985	11.37968	11.37664	11.19798
AT2G12770	7.335153	-2.241277638	0.589955	-3.79906	0.000145	0.0011016	1.082007	0	0.694589	1.429371	4.346824	4.474702
AT3G32020	50.6205	-2.234669614	0.587844	-3.80147	0.000144	0.0010929	0	0	0	7.884889	5.625572	4.391288
AT5G04815	7.193327	-2.233707473	0.589204	-3.79106	1.50E-04	1.13E-03	0	0	0	0	4.164273	4.767144
AT3G41345	6.682526	-2.232938978	0.581484	-3.84007	1.23E-04	0.0009512	1.082007	1.06174	0	3.862584	4.346824	2.584062
AT1G53080	5.75458	-2.229739998	0.585495	-3.8083	0.00014	0.0010676	0.640149	0.626414	0.694589	2.133098	4.164273	3.753887
AT5G31355	3.761797	-2.228243506	0.590247	-3.77511	1.60E-04	1.20E-03	0	0	0	3.480233	1.77337	3.458449
AT1G40112	67.49866	-2.220525975	0.587539	-3.77937	1.57E-04	1.18E-03	0	0	0	3.862584	7.740317	7.488773
AT2G13750	4.076909	-2.219083979	0.589904	-3.76177	1.69E-04	1.26E-03	0	0	0	2.133098	2.545235	4.107507
AT1G77950	13.0845	-2.217550677	0.5329	-4.16128	3.16E-05	0.000282	1.92313	1.06174	2.413855	5.379864	3.416038	4.391288
AT3G33181	6.402697	-2.216573054	0.592027	-3.74404	1.81E-04	1.34E-03	0	1.06174	0	4.414102	3.955229	1.806583
AT3G03085	11.64263	-2.21647982	0.587723	-3.7713	0.000162	0.001218	1.082007	1.06174	1.161466	0	5.019734	5.168875
AT5G61380	398.5072	-2.214029114	0.131708	-16.8102	2.05E-63	9.26E-61	7.040965	7.191545	7.162264	9.301278	9.315308	9.467545
AT1G37405	4.469456	-2.213147903	0.589303	-3.75553	1.73E-04	1.29E-03	0.640149	0.626414	0	3.242825	3.045364	3.458449
AT1G35990	10.12334	-2.212258938	0.592337	-3.7348	0.000188	0.0013896	1.082007	0.626414	0	0	4.786789	5.065041

AT4G04635	3.392275	-2.210584781	0.590227	-3.74531	1.80E-04	1.34E-03	0	0	0	2.958503	2.545235	3.284433
AT5G32405	4.049788	-2.207408884	0.592357	-3.72648	1.94E-04	0.0014329	0.640149	0	0	3.480233	2.545235	3.284433
AT1G72070	437.2694	-2.207019354	0.279649	-7.89212	2.97E-15	1.02E-13	7.724859	6.611509	6.96214	9.938721	9.379578	9.098269
AT4G37220	132.9132	-2.206946605	0.333053	-6.62641	3.44E-11	7.82E-10	5.284563	5.421786	5.528099	6.838581	8.487601	7.658921
AT4G06481	5.260849	-2.205293501	0.58922	-3.74274	1.82E-04	1.35E-03	0	0	0	4.722432	1.77337	2.247075
AT2G07240	6.437787	-2.202970542	0.59213	-3.72042	1.99E-04	1.46E-03	0.640149	0	0.694589	4.896561	1.77337	2.85705
AT2G34790	64.25827	-2.201579558	0.267518	-8.22964	1.88E-16	7.15E-15	4.399556	4.639454	4.377786	6.980752	6.714193	6.545826
AT5G28696	5.050235	-2.195765182	0.590572	-3.71803	2.01E-04	1.48E-03	0	0.626414	0.694589	2.133098	3.955229	3.613718
AT5G00490	5.9738	-2.194966777	0.581972	-3.7716	0.000162	0.0012171	0.640149	1.395705	0	3.480233	3.045364	4.107507
AT2G05705	4.456188	-2.192706431	0.589424	-3.72008	1.99E-04	1.47E-03	0.640149	0.626414	0	3.242825	3.416038	3.08651
AT5G32053	208.5001	-2.18646538	0.588479	-3.71545	2.03E-04	1.49E-03	2.719161	0	0.694589	9.597745	8.047769	7.702828
AT3G05475	3.888443	-2.179046978	0.592374	-3.6785	2.35E-04	0.0017015	0.640149	0	0	2.958503	3.045364	3.284433
AT5G28526	5.824636	-2.178033712	0.591986	-3.6792	0.000234	0.0016974	0.640149	0	0.694589	1.429371	3.710678	4.391288
AT1G67235	3.304969	-2.177211387	0.589752	-3.69174	0.000223	0.0016264	0	0	0	2.604042	3.045364	3.08651
AT3G33201	7.478426	-2.17506952	0.579763	-3.75165	1.76E-04	1.31E-03	0	1.395705	1.161466	4.021447	4.50885	2.584062
AT3G29633	17.51041	-2.173319056	0.486391	-4.46825	7.89E-06	7.97E-05	2.295555	2.911631	2.032847	5.545934	3.710678	5.117892
AT4G15530	2035.454	-2.172809025	0.113947	-19.0686	4.60E-81	4.08E-78	9.597137	9.553319	9.378956	11.67241	11.84721	11.5881
AT2G01029	23.50568	-2.166517865	0.390588	-5.54681	2.91E-08	4.41E-07	2.836608	2.911631	3.271714	5.123743	5.625572	5.265734
AT5G44415	5.60319	-2.164863592	0.59187	-3.65767	2.55E-04	1.83E-03	0.640149	0	0.694589	1.429371	3.955229	4.107507
AT3G33084	11.71009	-2.157358367	0.501069	-4.30551	1.67E-05	1.58E-04	1.082007	2.264856	2.235893	4.29471	4.50885	4.474702
AT2G09953	3.258136	-2.155164131	0.589323	-3.65702	0.000255	0.0018341	0	0	0	3.242825	2.545235	2.85705
AT5G31804	3.688427	-2.155124698	0.589036	-3.65873	2.53E-04	1.82E-03	0	0	0	3.862584	2.545235	2.247075
AT2G11380	3.439108	-2.154087663	0.589149	-3.65627	2.56E-04	0.0018374	0	0	0	2.133098	3.045364	3.458449
AT2G12345	3.815657	-2.151835428	0.58902	-3.65325	2.59E-04	1.85E-03	0	0	0	2.133098	3.955229	2.584062
AT2G06220	4.888306	-2.151558602	0.584134	-3.68333	2.30E-04	1.67E-03	0.640149	0.626414	0.694589	2.958503	3.710678	3.284433
AT2G11500	3.318237	-2.14993675	0.589109	-3.64947	0.000263	0.0018772	0	0	0	2.604042	2.545235	3.458449
AT2G05135	3.946751	-2.148304549	0.592391	-3.6265	2.87E-04	2.03E-03	0	0.626414	0	3.242825	3.416038	2.584062
AT3G39230	6.255046	-2.148135414	0.582587	-3.68724	0.000227	0.0016512	0.640149	0.626414	1.161466	4.164539	3.955229	2.247075
AT3G31980	3.701696	-2.145923038	0.588766	-3.64478	0.000268	0.0019086	0	0	0	3.862584	1.77337	2.85705
AT3G62950	384.0475	-2.141758978	0.268102	-7.98861	1.36E-15	4.82E-14	7.568105	6.651072	6.762956	8.962399	9.75153	9.10483
AT2G04010	3.332174	-2.141080242	0.588987	-3.63519	2.78E-04	1.97E-03	0	0	0	3.480233	2.545235	2.584062
AT2G01037	7.626258	-2.139582497	0.559341	-3.82518	1.31E-04	1.01E-03	0.640149	1.06174	1.79647	3.862584	4.346824	3.284433
AT1G30784	7.046764	-2.138161346	0.588504	-3.63322	2.80E-04	1.98E-03	0.640149	1.06174	0.694589	1.429371	3.710678	4.767144
AT3G33169	6.492256	-2.13813752	0.574168	-3.72389	1.96E-04	1.45E-03	0.640149	0.626414	1.513627	4.021447	3.955229	2.85705

AT4G15890	41.08778	-2.134749829	0.325985	-6.54862	5.81E-11	1.29E-09	3.537458	4.284411	3.67243	6.030007	6.152528	6.17386
AT1G62580	18.54191	-2.134049862	0.461194	-4.62723	3.71E-06	4.01E-05	1.693301	2.911631	2.963966	5.492674	4.654502	4.831847
AT5G41080	144.6877	-2.131956416	0.333133	-6.39971	1.56E-10	3.25E-09	5.548318	5.54451	5.709543	6.901235	8.599774	7.786827
AT2G11070	6.80351	-2.128458542	0.587192	-3.62481	2.89E-04	2.04E-03	0	0	0	0	3.710678	4.893774
AT1G54100	4560.439	-2.128446552	0.079487	-26.7772	5.95E-158	2.55E-154	10.72651	10.72913	10.7116	12.7831	12.87748	12.91559
AT1G77960	81.67867	-2.128033162	0.417476	-5.09737	3.44E-07	4.43E-06	3.956125	4.949759	4.93943	8.013353	6.292644	6.360876
AT3G61890	589.2155	-2.127883397	0.157257	-13.5312	1.02E-41	1.80E-39	7.953338	7.77142	7.489642	9.721543	10.03372	9.969746
AT5G52910	238.8817	-2.121660389	0.226966	-9.34791	8.94E-21	4.76E-19	6.145906	6.554232	6.560439	8.996279	8.185975	8.563072
AT5G32433	7.283129	-2.121189021	0.590703	-3.59095	0.000329	0.0022945	0	0	0.694589	0	4.346824	4.62832
AT5G37880	6.125903	-2.121122919	0.587257	-3.61191	3.04E-04	0.0021345	0	0	0	0	4.50885	3.998987
AT5G09440	560.7972	-2.118209535	0.238269	-8.89	6.11E-19	2.87E-17	8.18693	7.153483	7.376074	9.730198	10.01361	9.794149
AT5G32489	3.170829	-2.117551451	0.588657	-3.59726	3.22E-04	2.25E-03	0	0	0	2.958503	3.045364	2.584062
AT4G02312	5.53255	-2.116979049	0.579459	-3.65337	2.59E-04	1.85E-03	1.082007	0.626414	0.694589	3.480233	3.955229	2.85705
AT2G15160	4.140173	-2.115184283	0.592316	-3.57104	3.56E-04	2.46E-03	0	0	0.694589	2.133098	3.710678	3.284433
AT3G30790	4.15411	-2.113253151	0.592335	-3.56767	0.00036	0.0024832	0	0	0.694589	3.242825	3.710678	2.247075
AT5G29646	57.03129	-2.111019728	0.586612	-3.59867	3.20E-04	0.0022358	0	1.06174	0	3.862584	7.166841	7.538084
AT1G13920	50.73202	-2.110868929	0.329412	-6.40799	1.47E-10	3.09E-09	4.150129	4.113676	4.242928	5.694846	6.747048	6.602557
AT1G20440	4156.843	-2.109363138	0.224736	-9.38598	6.23E-21	3.37E-19	10.64029	10.60115	10.38055	12.00157	13.03709	12.95833
AT4G07523	3.949796	-2.108166657	0.587908	-3.58588	0.000336	0.0023337	0	0	0	1.429371	3.955229	3.08651
AT5G32520	5.930994	-2.106951447	0.586971	-3.58953	3.31E-04	2.31E-03	0	0	0	0	4.346824	4.107507
AT4G06507	3.775734	-2.104507392	0.587791	-3.58037	3.43E-04	2.38E-03	0	0	0	4.021447	1.77337	2.584062
AT2G25550	6.854324	-2.101966883	0.590376	-3.56039	0.00037	0.0025476	0	0.626414	0	0	4.346824	4.474702
AT3G43350	6.979813	-2.099288428	0.59199	-3.54616	3.91E-04	2.67E-03	0.640149	1.06174	0	4.97633	3.045364	1.806583
AT3G48130	7.565339	-2.096574908	0.571327	-3.66966	2.43E-04	1.76E-03	1.92313	1.06174	0	3.684038	3.416038	4.391288
AT1G32225	3.230931	-2.092473736	0.587932	-3.55904	3.72E-04	0.0025587	0	0	0	2.133098	3.045364	3.284433
AT1G48680	35.21295	-2.090204485	0.586081	-3.56641	3.62E-04	2.49E-03	0.640149	0	0	2.958503	6.050936	7.125632
AT3G32091	3.11006	-2.087149054	0.587852	-3.55047	3.85E-04	2.64E-03	0	0	0	2.604042	2.545235	3.284433
AT2G15110	7.025157	-2.083277227	0.590116	-3.53029	4.15E-04	2.82E-03	0.640149	0	0	0	4.786789	3.998987
AT1G03090	4854.6	-2.078195483	0.124483	-16.6947	1.43E-62	6.14E-60	10.93636	10.79109	10.79281	12.71133	13.11742	12.97208
AT2G12860	4.359209	-2.077628413	0.591705	-3.51126	4.46E-04	3.00E-03	0.640149	0	0	4.164539	1.77337	2.85705
AT5G51810	17.60442	-2.076589182	0.470747	-4.41126	1.03E-05	1.02E-04	2.836608	2.419482	2.413855	3.862584	5.476813	5.117892
AT2G00560	4.224435	-2.073398883	0.59138	-3.50604	4.55E-04	0.0030619	0	0.626414	0.694589	3.684038	2.545235	3.08651
AT4G07530	3.412571	-2.07141828	0.587405	-3.52639	4.21E-04	2.85E-03	0	0	0	2.133098	3.710678	2.584062
AT5G08725	4.51875	-2.067682219	0.591946	-3.49303	4.78E-04	0.0031939	1.082007	0	0	2.604042	3.416038	3.613718

AT2G27550	3.606228	-2.058600322	0.592142	-3.47653	5.08E-04	3.37E-03	0.640149	0	0	2.604042	3.045364	3.284433
AT1G19530	57.82809	-2.057630352	0.263904	-7.79689	6.35E-15	2.12E-13	4.473957	4.507728	4.420076	6.607592	6.420349	6.692417
AT5G34990	2.975921	-2.05459093	0.58717	-3.49914	0.000467	0.0031335	0	0	0	2.958503	2.545235	2.85705
AT3G25720	4.334649	-2.053898898	0.592092	-3.46889	0.000523	0.0034558	0.640149	0.626414	0	2.604042	2.545235	3.998987
AT1G39590	3.493519	-2.050992455	0.586607	-3.49637	4.72E-04	3.16E-03	0	0	0	3.862584	1.77337	2.584062
AT4G06684	3.493519	-2.050992455	0.586607	-3.49637	4.72E-04	3.16E-03	0	0	0	3.862584	1.77337	2.584062
AT1G02620	53.33118	-2.049672864	0.381342	-5.37489	7.66E-08	1.10E-06	5.012364	3.569791	3.6007	6.632428	6.336481	6.486772
AT5G15690	5.709548	-2.048017512	0.585574	-3.49745	4.70E-04	3.15E-03	0	0	0	0	4.50885	3.753887
AT5G63250	5.709548	-2.048017512	0.585574	-3.49745	0.00047	0.0031474	0	0	0	0	4.50885	3.753887
AT3G38525	33.89393	-2.046227132	0.585099	-3.49723	0.00047	0.0031491	0	0.626414	0	7.353641	4.907958	3.613718
AT5G32169	3.616217	-2.04518936	0.592147	-3.45385	5.53E-04	3.64E-03	0	0	0.694589	2.604042	3.045364	3.284433
AT3G31475	5.51464	-2.04041066	0.585415	-3.48541	4.91E-04	3.27E-03	0	0	0	0	4.346824	3.881636
AT3G60140	48.84578	-2.039798485	0.340786	-5.98556	2.16E-09	3.87E-08	3.848373	4.730912	3.67243	6.530411	6.379024	6.123056
AT1G11595	3.849772	-2.039657832	0.58607	-3.48023	5.01E-04	3.33E-03	0	0	0	4.164539	1.77337	2.247075
AT1G38212	3.849772	-2.039657832	0.58607	-3.48023	5.01E-04	3.33E-03	0	0	0	4.164539	1.77337	2.247075
AT1G04157	148.3826	-2.025346582	0.22798	-8.88389	6.46E-19	3.02E-17	5.870968	5.873683	5.709543	7.535811	8.301072	7.819103
AT5G20260	15.01639	-2.024856714	0.49946	-4.05409	5.03E-05	4.29E-04	3.140592	1.894767	1.513627	4.29471	4.786789	5.065041
AT5G32136	5.554446	-2.019571737	0.584765	-3.45365	5.53E-04	3.64E-03	0	0	0	0	3.710678	4.474702
AT1G53580	962.8011	-2.018959413	0.109647	-18.4133	1.03E-75	7.14E-73	8.583329	8.538459	8.570211	10.4526	10.63478	10.6935
AT1G06570	3360.46	-2.016691321	0.11057	-18.239	2.53E-74	1.62E-71	10.43967	10.23588	10.41167	12.43517	12.50499	12.24707
AT4G03825	4.68315	-2.015817613	0.585002	-3.44583	5.69E-04	3.73E-03	0	0	0	4.524365	2.545235	1.169325
AT5G27340	3.936527	-2.012835677	0.585419	-3.43828	5.85E-04	3.81E-03	0	0	0	1.429371	4.164273	2.584062
AT1G03100	313.5128	-2.010505656	0.196682	-10.2221	1.58E-24	1.12E-22	7.197372	6.778008	6.746431	8.682211	9.248041	8.978347
AT4G06641	3.063227	-2.009589401	0.585713	-3.43101	0.000601	0.0039029	0	0	0	3.242825	1.77337	3.08651
AT3G33197	4.731226	-2.006955366	0.592366	-3.38804	7.04E-04	4.47E-03	0.640149	0	0.694589	4.29471	2.545235	2.247075
AT2G47780	52.0929	-2.005426138	0.399667	-5.01774	5.23E-07	6.55E-06	2.719161	4.607633	4.614715	6.332435	6.574739	6.446015
AT4G12520	39.66593	-2.005291621	0.341264	-5.87608	4.20E-09	7.15E-08	3.605233	3.812433	4.242928	5.597298	6.379024	6.070398
AT3G33545	103.2516	-2.004780168	0.588206	-3.40829	6.54E-04	0.0041962	0	3.194694	1.161466	7.709256	8.301072	6.466537
AT3G33570	4.319587	-2.003849581	0.592231	-3.38356	7.16E-04	4.53E-03	0	1.06174	0	3.480233	3.416038	2.584062
AT1G48290	6.034884	-1.99911861	0.588999	-3.3941	6.89E-04	4.38E-03	0	0.626414	0	0	4.164273	4.302755
AT5G34837	3.351801	-1.998582088	0.585258	-3.41487	6.38E-04	4.11E-03	0	0	0	1.429371	3.416038	3.284433
AT2G38465	39.93504	-1.997544171	0.322309	-6.19761	5.73E-10	1.11E-08	4.056385	4.113676	3.6007	5.786214	6.292644	6.04333
AT1G42130	6.021615	-1.996062193	0.588993	-3.38894	7.02E-04	0.004455	0	0.626414	0	0	4.346824	4.107507
AT4G22390	16.80051	-1.994555194	0.432696	-4.6096	4.03E-06	4.33E-05	2.719161	2.419482	2.844834	4.812122	5.123469	4.62832

AT1G38280	3.567557	-1.993133517	0.584875	-3.40779	6.55E-04	0.0042029	0	0	0	4.021447	1.77337	2.247075
AT1G07985	65.31213	-1.990510786	0.258515	-7.69979	1.36E-14	4.39E-13	4.509766	4.788814	4.720024	6.582321	6.958197	6.657142
AT1G36400	5.707723	-1.989772683	0.589039	-3.378	0.00073	0.0046076	0	0.626414	0	4.896561	1.77337	1.806583
AT1G38410	3.211303	-1.989719752	0.585014	-3.40115	6.71E-04	0.0042914	0	0	0	3.684038	1.77337	2.584062
AT4G04130	7.393416	-1.986738863	0.565029	-3.51617	4.38E-04	2.95E-03	1.082007	1.894767	0.694589	4.414102	3.710678	3.08651
AT3G30819	4.400934	-1.984126983	0.584173	-3.39647	0.000683	0.0043539	0	0	0	4.414102	2.545235	1.169325
AT2G24780	2.989189	-1.980443728	0.584843	-3.38628	7.08E-04	4.49E-03	0	0	0	2.958503	1.77337	3.284433
AT4G07630	8.626273	-1.980331628	0.591709	-3.3468	8.18E-04	0.0050875	0.640149	1.06174	0	5.492674	2.545235	1.169325
AT1G36795	4.206026	-1.980112427	0.584112	-3.38995	6.99E-04	4.44E-03	0	0	0	4.414102	1.77337	1.806583
AT3G42251	4.206026	-1.980112427	0.584112	-3.38995	6.99E-04	0.0044418	0	0	0	4.414102	1.77337	1.806583
AT5G29568	62.89422	-1.979836961	0.58218	-3.40073	6.72E-04	4.30E-03	0	0	0	3.480233	7.26084	7.753812
AT4G34030	2296.686	-1.977718673	0.141012	-14.0252	1.09E-44	2.13E-42	9.879668	9.809929	9.826909	11.52768	12.04182	11.91788
AT1G42510	3.157561	-1.977690562	0.584875	-3.38139	0.000721	0.0045578	0	0	0	2.958503	3.416038	1.806583
AT1G35215	6.15139	-1.976824406	0.591784	-3.34045	0.000836	0.0051796	1.082007	0	0	1.429371	4.786789	3.08651
AT5G15960	84.58832	-1.976247218	0.296021	-6.67605	2.45E-11	5.68E-10	5.42246	4.701066	4.880072	6.632428	7.11744	7.468567
AT2G02100	1105.25	-1.971588565	0.163196	-12.0811	1.33E-33	1.54E-31	9.048027	8.424359	8.807742	10.82671	10.86922	10.67046
AT5G50450	183.4212	-1.971415376	0.184503	-10.685	1.20E-26	9.40E-25	6.347356	6.03789	6.172301	8.182658	8.397349	8.019904
AT5G03090	43.08871	-1.970977144	0.581996	-3.38658	7.08E-04	4.49E-03	0	0	0	2.958503	7.040014	6.932619
AT5G32430	3.348757	-1.970174476	0.591439	-3.33116	8.65E-04	5.32E-03	0	0.626414	0	3.242825	2.545235	2.85705
AT4G16146	153.5434	-1.96938689	0.283515	-6.94632	3.75E-12	9.57E-11	5.459553	5.688357	6.357789	7.412182	8.209741	8.146128
AT5G52300	20.02677	-1.968571926	0.466084	-4.22365	2.40E-05	2.20E-04	1.693301	3.502171	2.844834	5.437371	4.786789	5.065041
AT5G32670	4.066135	-1.96835278	0.590873	-3.33126	8.65E-04	5.32E-03	0	0	0.694589	1.429371	3.710678	3.458449
AT4G27260	3106.138	-1.967890779	0.146536	-13.4294	4.07E-41	6.88E-39	10.50766	10.04638	10.25183	12.07191	12.34787	12.39659
AT2G15970	1030.304	-1.967173403	0.213713	-9.20475	3.42E-20	1.75E-18	8.863144	8.332442	8.738409	10.14912	10.87666	10.93445
AT5G35935	66.82777	-1.964355071	0.290859	-6.75363	1.44E-11	3.42E-10	4.150129	4.949759	4.93943	6.921529	6.499609	6.85691
AT1G53885	40.80328	-1.961407016	0.331643	-5.91421	3.33E-09	5.77E-08	4.23815	3.867214	3.806003	6.138016	6.379024	5.635566
AT1G53903	40.80328	-1.961407016	0.331643	-5.91421	3.33E-09	5.77E-08	4.23815	3.867214	3.806003	6.138016	6.379024	5.635566
AT4G04390	7.6965	-1.959885779	0.586839	-3.33973	0.000839	0.0051899	0	0.626414	0	5.379864	0	2.584062
AT3G00630	3.804767	-1.958651402	0.590862	-3.3149	9.17E-04	5.60E-03	0	0	0.694589	3.862584	1.77337	2.85705
AT5G30545	4.916645	-1.958422419	0.583145	-3.35838	0.000784	0.0048969	0	0	0	0	3.955229	3.998987
AT4G04590	7.753377	-1.954105637	0.586562	-3.33145	8.64E-04	5.32E-03	0	0.626414	0	0	3.045364	5.31183
AT3G30867	36.96839	-1.953380654	0.337674	-5.78482	7.26E-09	1.19E-07	3.903255	3.812433	3.928251	6.332435	5.476813	5.869303
AT4G03870	4.30878	-1.95302106	0.592374	-3.29694	9.77E-04	5.93E-03	0.640149	0.626414	0	3.480233	3.710678	1.806583
AT3G33175	3.755555	-1.952577979	0.583632	-3.34556	8.21E-04	5.11E-03	0	0	0	2.958503	3.955229	1.169325

AT2G13230	3.198035	-1.949971889	0.583796	-3.34016	8.37E-04	5.18E-03	0	0	0	3.684038	2.545235	1.806583
AT4G04775	4.903376	-1.948580496	0.582852	-3.34318	0.000828	0.0051419	0	0	0	0	4.164273	3.753887
AT5G28232	4.903376	-1.948580496	0.582852	-3.34318	0.000828	0.0051419	0	0	0	0	4.164273	3.753887
AT4G25000	31.23208	-1.948455402	0.456308	-4.27004	1.95E-05	1.83E-04	3.140592	4.243584	2.413855	4.97633	5.82339	6.17386
AT5G15685	44.19594	-1.943914967	0.581206	-3.34462	0.000824	0.0051203	0	0	0	2.958503	6.900955	7.138479
AT4G07920	2.814576	-1.941125536	0.583838	-3.32477	0.000885	0.0054256	0	0	0	2.133098	3.045364	2.85705
AT5G01600	7034.492	-1.935090239	0.203498	-9.50916	1.92E-21	1.07E-19	11.66759	10.9593	11.65224	13.25608	13.77581	13.27918
AT5G39410	198.9586	-1.932763499	0.199428	-9.69154	3.28E-22	1.96E-20	6.58411	6.04978	6.346869	8.122569	8.289966	8.509687
AT5G33220	3.143624	-1.932190925	0.583236	-3.31288	9.23E-04	0.0056361	0	0	0	1.429371	3.416038	3.08651
AT1G06957	10.27763	-1.931337182	0.495665	-3.89645	9.76E-05	7.75E-04	1.92313	2.091646	2.032847	4.164539	4.346824	4.107507
AT1G65970	110.6926	-1.93064036	0.222531	-8.67584	4.11E-18	1.79E-16	5.695509	5.561219	5.179914	7.292624	7.548635	7.576354
AT4G04167	2.85505	-1.928012165	0.583208	-3.30587	9.47E-04	5.76E-03	0	0	0	3.242825	1.77337	2.85705
AT4G03970	3.542997	-1.927769619	0.590242	-3.26607	1.09E-03	6.52E-03	0	0.626414	0	2.133098	2.545235	3.753887
AT1G56300	309.4417	-1.927008104	0.18385	-10.4814	1.05E-25	7.95E-24	6.78012	6.990318	7.149752	8.615264	9.134672	9.048089
AT4G06539	5.630371	-1.926723172	0.581861	-3.31131	9.29E-04	5.66E-03	0	0	0	4.896561	0	2.584062
AT5G33434	8.222993	-1.926338706	0.544478	-3.53796	4.03E-04	2.75E-03	1.693301	1.06174	2.032847	4.164539	3.045364	4.302755
AT1G35770	6.111417	-1.92499836	0.591154	-3.25634	1.13E-03	0.0067161	0	0.626414	0.694589	0	4.50885	3.881636
AT4G16690	186.8344	-1.922678272	0.192074	-10.0101	1.38E-23	9.26E-22	6.506476	6.24857	5.973665	8.068994	8.301072	8.291245
AT3G33066	2.929088	-1.917977401	0.582798	-3.29098	9.98E-04	6.03E-03	0	0	0	3.480233	1.77337	2.584062
AT4G07580	2.929088	-1.917977401	0.582798	-3.29098	0.000998	0.0060335	0	0	0	3.480233	1.77337	2.584062
AT1G28330	4912.042	-1.916782375	0.1264	-15.1644	6.09E-52	1.74E-49	11.1746	10.73512	11.02418	12.94706	12.96735	12.86769
AT2G11983	4.721736	-1.915238269	0.58174	-3.29226	9.94E-04	6.01E-03	0	0	0	0	3.710678	4.107507
AT1G67265	321.7396	-1.91463722	0.168504	-11.3626	6.42E-30	6.08E-28	7.343439	6.946471	6.819344	8.996279	8.984333	9.024061
AT2G12990	3.337949	-1.910172265	0.590736	-3.23355	0.001223	0.007209	0.640149	0	0	3.242825	3.045364	2.247075
AT2G06150	5.813255	-1.909306674	0.591088	-3.23016	1.24E-03	0.00728	0.640149	0	0.694589	4.896561	1.77337	1.806583
AT3G18320	70.72807	-1.908234043	0.417412	-4.57158	4.84E-06	5.10E-05	4.578823	5.023964	4.377786	5.19216	7.305638	7.307265
AT5G26350	5.085016	-1.907362853	0.581419	-3.28053	1.04E-03	6.24E-03	0	0	0	0	4.50885	3.284433
AT4G06710	5.12671	-1.90641119	0.58131	-3.27951	0.00104	0.0062548	0	0	0	4.812122	1.77337	1.169325
AT5G33223	3.641595	-1.902728507	0.581766	-3.27061	0.001073	0.0064309	0	0	0	4.164539	1.77337	1.806583
AT5G57670	18.20196	-1.900931491	0.428014	-4.44128	8.94E-06	8.94E-05	2.94521	2.419482	3.176244	4.626795	5.019734	5.168875
AT5G28760	3.651267	-1.898425669	0.590071	-3.21728	0.001294	0.0075852	0	0.626414	0	2.958503	3.710678	1.806583
AT5G39770	6.908844	-1.897761651	0.592221	-3.20448	1.35E-03	0.0078786	1.082007	0.626414	0.694589	0	4.654502	3.998987
AT2G15880	264.1763	-1.891255654	0.187078	-10.1095	5.02E-24	3.46E-22	6.470584	7.008709	6.835058	8.676251	8.544777	8.897523
AT2G38400	957.5344	-1.890858744	0.165055	-11.4559	2.20E-30	2.14E-28	8.828117	8.392033	8.665575	10.32701	10.81602	10.51905

AT1G80920	10908.29	-1.886120886	0.162766	-11.5879	4.75E-31	4.78E-29	12.26728	12.05498	12.12737	13.66345	14.30882	14.17735
AT3G43681	4.520587	-1.884762598	0.592037	-3.18352	1.45E-03	8.37E-03	0	0.626414	0.694589	4.29471	2.545235	1.806583
AT1G37110	4.69273	-1.87814394	0.592127	-3.17186	1.51E-03	8.66E-03	0.640149	0.626414	0.694589	1.429371	3.045364	4.208431
AT5G32483	3.003126	-1.877612338	0.581215	-3.23049	1.24E-03	0.007273	0	0	0	3.684038	1.77337	2.247075
AT5G50715	3.003126	-1.877612338	0.581215	-3.23049	1.24E-03	0.007273	0	0	0	3.684038	1.77337	2.247075
AT4G07570	4.427472	-1.875969921	0.580387	-3.23227	0.001228	0.0072345	0	0	0	4.414102	0	2.85705
AT1G15040	91.73669	-1.87546259	0.283233	-6.62162	3.55E-11	8.06E-10	5.459553	5.40337	4.685768	7.03764	7.585434	6.917792
AT4G04330	300.4983	-1.875089436	0.200503	-9.35193	8.61E-21	4.60E-19	6.592483	6.97169	7.281752	8.664258	8.998046	9.00665
AT1G43200	3.337281	-1.873748503	0.58957	-3.17816	1.48E-03	8.50E-03	0.640149	0	0	2.133098	2.545235	3.613718
AT1G19540	295.0596	-1.869678404	0.164386	-11.3737	5.65E-30	5.38E-28	7.089101	7.032872	6.754717	8.658224	8.970488	8.953118
AT4G06627	7.488322	-1.868233569	0.584328	-3.19724	1.39E-03	8.05E-03	0	0.626414	0	5.379864	0	2.247075
AT2G09388	2.875345	-1.865295914	0.581012	-3.21043	1.33E-03	7.73E-03	0	0	0	2.604042	3.416038	1.806583
AT3G43147	3.554288	-1.863814583	0.580362	-3.21147	0.001321	0.0077105	0	0	0	4.021447	2.545235	1.169325
AT4G06486	3.554288	-1.863814583	0.580362	-3.21147	0.001321	0.0077105	0	0	0	4.021447	2.545235	1.169325
AT3G13450	1424.113	-1.86327504	0.131628	-14.1556	1.72E-45	3.41E-43	9.344219	9.122127	9.260306	10.90779	11.30122	11.15832
AT1G34600	2.619667	-1.86119398	0.58087	-3.20415	1.35E-03	0.0078859	0	0	0	2.133098	2.545235	3.08651
AT5G19230	68.18743	-1.856902317	0.287099	-6.46782	9.94E-11	2.13E-09	5.177314	4.844481	4.33422	6.530411	7.013254	6.760481
AT3G00600	3.359379	-1.856056907	0.580125	-3.19941	1.38E-03	8.00E-03	0	0	0	4.021447	1.77337	1.806583
AT1G42590	46.28536	-1.855252472	0.583319	-3.18051	1.47E-03	8.44E-03	1.419792	1.395705	0	2.604042	7.013254	7.151212
AT4G28040	578.6908	-1.852057704	0.164268	-11.2746	1.75E-29	1.60E-27	7.907093	7.796335	8.116007	9.581894	10.09562	9.777819
AT1G48250	4.305381	-1.84725261	0.579354	-3.18847	1.43E-03	8.26E-03	0	0	0	0	3.710678	3.881636
AT1G43205	2.606399	-1.846690702	0.580391	-3.18181	0.001464	0.0084098	0	0	0	2.133098	3.045364	2.584062
AT1G11210	236.9464	-1.846402885	0.213434	-8.65095	5.11E-18	2.21E-16	6.405541	6.719662	6.819344	8.2723	8.386963	8.908815
AT5G48250	107.7904	-1.846195892	0.236775	-7.79725	6.33E-15	2.11E-13	5.242615	5.762479	5.508634	7.110162	7.529878	7.585765
AT1G32010	24.28841	-1.843761976	0.578074	-3.18949	1.43E-03	0.0082422	0	0	0	2.133098	6.292644	6.04333
AT3G32360	97.38223	-1.843062207	0.578716	-3.18475	0.001449	0.0083435	0	1.06174	0	8.881834	5.220243	6.270397
AT3G05870	276.752	-1.840970674	0.170132	-10.8209	2.74E-27	2.21E-25	6.830546	6.881427	6.976381	8.464361	8.891872	8.908815
AT5G54080	2226.46	-1.837231083	0.131598	-13.9609	2.70E-44	5.10E-42	10.14102	9.687798	9.866634	11.70571	11.79515	11.80708
AT1G37050	2.485528	-1.83587836	0.579983	-3.1654	1.55E-03	8.81E-03	0	0	0	2.604042	2.545235	2.584062
AT3G43291	2.485528	-1.83587836	0.579983	-3.1654	1.55E-03	8.81E-03	0	0	0	2.604042	2.545235	2.584062
AT3G30370	2.646873	-1.833414171	0.579658	-3.16292	0.001562	0.0088795	0	0	0	3.242825	1.77337	2.584062
AT5G32312	2.572834	-1.827535357	0.579444	-3.15395	1.61E-03	0.0091087	0	0	0	2.958503	1.77337	2.85705
AT5G28335	19.43403	-1.825565547	0.509473	-3.58324	3.39E-04	2.35E-03	2.121327	3.194694	2.963966	3.242825	5.396255	5.635566
AT3G26740	12729.47	-1.821719719	0.147564	-12.3453	5.17E-35	6.54E-33	12.31486	12.25663	12.6864	14.08578	14.45798	14.28119

AT2G39980	304.9015	-1.817336647	0.241841	-7.51458	5.71E-14	1.74E-12	7.191872	6.946471	6.896258	8.240343	9.28766	9.020596
AT1G77380	35.76341	-1.81324268	0.349797	-5.18371	2.18E-07	2.90E-06	4.194811	3.75549	3.806003	5.319969	5.997325	6.148682
AT4G10695	8.305412	-1.810199869	0.547961	-3.30352	9.55E-04	5.80E-03	0.640149	1.894767	2.235893	4.164539	3.416038	4.107507
AT4G06504	2.740538	-1.810117948	0.578608	-3.1284	1.76E-03	9.84E-03	0	0	0	1.429371	3.045364	3.08651
AT5G53970	640.8209	-1.806696479	0.153332	-11.7829	4.78E-32	5.21E-30	8.38551	7.975313	7.998818	9.882893	10.12716	9.886522
AT3G32210	40.536	-1.804958342	0.576573	-3.13049	0.001745	0.0097804	0	0	0	7.63746	5.123469	3.613718
AT1G40139	39.5022	-1.803569566	0.576525	-3.12835	1.76E-03	9.84E-03	0	0	0	2.604042	6.985988	6.743763
AT3G51400	82.92076	-1.797501452	0.265667	-6.76599	1.32E-11	3.15E-10	5.154874	5.16182	5.179914	6.503738	7.370331	7.125632
AT3G08860	45.12246	-1.793306971	0.390933	-4.58725	4.49E-06	4.76E-05	3.791321	4.639454	4.145468	5.872138	5.476813	6.872373
AT1G29395	1929.321	-1.787627969	0.180817	-9.88638	4.77E-23	3.06E-21	9.901736	9.422258	9.81309	11.16666	11.71957	11.71788
AT2G19800	1233.924	-1.783897945	0.445721	-4.00227	6.27E-05	0.0005236	8.139144	9.073452	9.079174	10.27275	11.68924	10.55901
AT3G11550	12.305	-1.780784391	0.487513	-3.65279	2.59E-04	1.86E-03	2.836608	1.666709	2.413855	4.164539	4.50885	4.553554
AT5G67060	6.55024	-1.774361172	0.556348	-3.1893	0.001426	0.0082446	1.419792	1.395705	1.513627	3.242825	4.164273	3.284433
AT1G07050	9.820955	-1.772650684	0.529448	-3.34811	8.14E-04	5.07E-03	1.92313	2.264856	1.79647	2.958503	4.346824	4.62832
AT3G43850	151.8745	-1.763247518	0.286996	-6.1438	8.06E-10	1.52E-08	6.558693	5.493187	5.839872	7.799743	8.256129	7.585765
AT4G25490	34.49271	-1.751132465	0.390357	-4.48598	7.26E-06	7.41E-05	3.848373	3.569791	4.195021	4.896561	5.997325	6.222936
AT1G20620	49726.35	-1.74878608	0.142261	-12.2928	9.90E-35	1.22E-32	14.58655	14.3043	14.46684	16.4967	16.04352	16.11295
AT5G23050	626.0735	-1.748018916	0.124062	-14.0898	4.39E-45	8.60E-43	8.164639	8.175792	8.138345	9.835552	9.814884	10.09036
AT3G15630	921.1361	-1.744805278	0.174009	-10.0271	1.16E-23	7.81E-22	8.984228	8.442511	8.609573	10.30786	10.72412	10.37586
AT1G49130	1556.449	-1.741643188	0.183135	-9.51016	1.90E-21	1.07E-19	9.661232	9.361545	9.313157	10.82402	11.26339	11.53626
AT5G57655	8813.038	-1.739215612	0.108551	-16.0221	8.96E-58	3.11E-55	12.13917	11.89426	11.88306	13.62006	13.77482	13.79186
AT3G05945	148.9097	-1.73701198	0.279223	-6.22087	4.94E-10	9.59E-09	6.234178	5.475665	6.268024	7.353641	8.256129	7.866203
AT2G41190	158.3762	-1.736068853	0.225314	-7.70509	1.31E-14	4.23E-13	6.039683	6.227787	6.232868	7.649677	7.756543	8.336545
AT2G23030	51.4888	-1.724621029	0.314032	-5.49187	3.98E-08	5.90E-07	4.987182	4.201568	4.242928	6.172293	6.574739	6.293554
AT4G18650	8.711113	-1.720176143	0.531866	-3.23423	1.22E-03	7.19E-03	1.92313	2.264856	1.513627	4.414102	3.045364	4.107507
AT3G51000	1160.416	-1.717716912	0.122976	-13.9679	2.45E-44	4.66E-42	9.195489	9.010965	8.983712	10.63418	10.94019	10.8185
AT2G06005	68.19266	-1.711114173	0.255279	-6.70292	2.04E-11	4.76E-10	4.908884	4.788814	5.204319	6.530411	6.810594	6.85691
AT4G36670	658.3383	-1.697957012	0.158471	-10.7146	8.69E-27	6.91E-25	8.411773	8.04528	8.28285	9.830195	10.23242	9.855735
AT3G15635	218.8767	-1.689859723	0.194195	-8.70187	3.26E-18	1.44E-16	6.913091	6.459362	6.597764	8.122569	8.572538	8.469581
AT1G22770	836.3205	-1.684881529	0.144674	-11.6461	2.40E-31	2.47E-29	8.346437	8.696181	8.773492	10.18943	10.34669	10.4265
AT4G12510	21.4028	-1.68040482	0.418507	-4.01524	5.94E-05	4.98E-04	2.836608	3.10637	3.740762	4.626795	5.396255	5.265734
AT2G39920	33.29243	-1.674917306	0.370497	-4.52073	6.16E-06	6.36E-05	4.056385	4.020081	3.67243	4.97633	5.941644	6.070398
AT4G32340	451.6961	-1.671618206	0.173295	-9.64606	5.11E-22	2.98E-20	7.740014	7.490932	7.927316	9.163229	9.646611	9.459889
AT5G07010	1013.069	-1.670617606	0.127991	-13.0527	6.14E-39	9.50E-37	8.885464	9.033594	8.793737	10.40835	10.63697	10.72471

AT2G15960	4538.582	-1.670155583	0.171935	-9.7139	2.63E-22	1.58E-20	11.37325	10.82391	10.90826	12.50995	12.97084	12.77098
AT4G38580	207.9583	-1.668731702	0.234729	-7.10919	1.17E-12	3.15E-11	6.297009	6.468249	6.940512	7.915566	8.61765	8.364152
AT4G26288	298.4299	-1.666230788	0.196635	-8.47371	2.38E-17	9.74E-16	7.377757	7.008709	6.96214	8.511981	9.115929	8.835761
AT4G24230	1691.686	-1.659240599	0.157565	-10.5305	6.25E-26	4.77E-24	9.814051	9.586348	9.519305	11.04592	11.60214	11.30512
AT1G52100	57.66916	-1.658989546	0.301234	-5.50731	3.64E-08	5.45E-07	5.037115	4.730912	4.377786	6.102904	6.841348	6.466537
AT5G07440	3154.207	-1.656714486	0.134103	-12.354	4.63E-35	5.89E-33	10.71217	10.41949	10.50978	12.121	12.44806	12.09802
AT4G36900	64.66465	-1.652834427	0.326788	-5.05781	4.24E-07	5.40E-06	4.544707	5.493187	4.377786	6.859769	6.610887	6.506726
AT5G14470	48.19137	-1.647671738	0.327944	-5.02425	5.05E-07	6.35E-06	4.826091	4.284411	4.242928	6.420628	6.499609	5.706299
AT5G57630	508.6428	-1.642725118	0.16561	-9.91927	3.43E-23	2.22E-21	8.235859	7.735063	7.756298	9.52666	9.570642	9.695454
AT1G76590	224.1585	-1.641411143	0.172432	-9.51917	1.75E-21	9.82E-20	6.641729	6.666599	6.947758	8.349224	8.289966	8.596044
AT1G09420	166.7815	-1.63413352	0.211938	-7.71044	1.25E-14	4.06E-13	6.265936	6.338556	6.335866	7.574773	8.312092	8.019904
AT3G51430	318.5363	-1.62998135	0.168743	-9.65955	4.48E-22	2.65E-20	7.494057	7.026869	7.229204	9.043339	8.942394	8.771237
AT1G58180	1494.141	-1.622585957	0.138856	-11.6854	1.51E-31	1.59E-29	9.644215	9.515236	9.324334	10.91413	11.29429	11.20257
AT1G04467	76.50803	-1.621912561	0.259045	-6.26111	3.82E-10	7.54E-09	5.108917	5.183567	5.27515	6.530411	7.26084	6.777007
AT1G10760	2168.207	-1.620826301	0.166301	-9.74631	1.91E-22	1.17E-20	9.789679	10.17068	10.10645	11.33214	11.78034	11.88395
AT4G30650	136.2659	-1.617907046	0.270692	-5.97694	2.27E-09	4.06E-08	5.648094	6.096383	6.244682	7.09237	7.865285	8.019904
AT5G34795	17.68298	-1.616133299	0.453629	-3.56267	3.67E-04	2.53E-03	2.450992	3.634384	2.714973	5.19216	4.786789	4.553554
AT1G21670	522.1583	-1.615836781	0.138626	-11.6561	2.14E-31	2.21E-29	7.946822	7.881962	8.135175	9.455375	9.775615	9.637809
AT1G62770	42.81967	-1.614420357	0.333783	-4.83674	1.32E-06	1.55E-05	4.056385	4.158291	4.720024	5.694846	6.460523	5.958939
AT4G24050	211.2662	-1.608905398	0.19939	-8.06915	7.08E-16	2.55E-14	6.965608	6.459362	6.550955	8.349224	8.516472	8.100899
AT4G39780	110.404	-1.607912671	0.248542	-6.46937	9.84E-11	2.11E-09	5.98984	5.384715	5.759806	7.03764	7.673524	7.437718
AT5G02580	90.80732	-1.607482999	0.277907	-5.78425	7.28E-09	1.20E-07	5.88467	5.094538	5.204319	6.838581	7.391266	7.112669
AT1G20450	1287.734	-1.605519822	0.242118	-6.63116	3.33E-11	7.59E-10	9.379728	9.256448	9.140532	10.13832	11.08375	11.3403
AT5G16370	1904.482	-1.603913191	0.146987	-10.9119	1.01E-27	8.41E-26	10.08277	9.670575	9.802139	11.33876	11.69662	11.41545
AT3G09390	4180.232	-1.602742344	0.122843	-13.0471	6.60E-39	1.02E-36	10.99399	10.93466	11.07882	12.39264	12.65697	12.79196
AT5G16340	200.6704	-1.5943576	0.184598	-8.63692	5.77E-18	2.49E-16	6.787432	6.459362	6.588523	8.087074	8.497289	8.139753
AT1G27670	27.7871	-1.590645504	0.407337	-3.90498	9.42E-05	7.53E-04	4.280226	3.012284	3.525217	5.123743	5.82339	5.399825
AT1G65620	24.14936	-1.588620503	0.424012	-3.74664	0.000179	0.001331	3.466342	3.634384	3.445564	4.29471	5.310931	5.838142
AT4G23870	894.7344	-1.586073966	0.149291	-10.6241	2.30E-26	1.79E-24	9.046504	8.521482	8.750889	10.43507	10.31965	10.43563
AT5G48000	14.92903	-1.585644843	0.474573	-3.34121	0.000834	0.0051699	2.295555	3.502171	2.235893	4.626795	4.654502	4.62832
AT3G26480	31.37847	-1.5836651	0.425848	-3.71885	0.0002	0.0014719	3.669966	4.067637	3.806003	4.29471	5.941644	6.123056
AT1G49000	24.69568	-1.577561932	0.461418	-3.41895	6.29E-04	0.0040575	3.848373	2.264856	3.806003	4.896561	5.941644	4.893774
AT1G22130	9.391173	-1.574273857	0.500184	-3.14739	1.65E-03	9.30E-03	2.295555	2.091646	2.235893	3.862584	4.164273	3.998987
AT4G26530	2276.023	-1.572224002	0.188052	-8.3606	6.24E-17	2.46E-15	9.890744	10.02772	10.42081	11.48995	12.08663	11.58517

AT3G29631	27.98183	-1.570487717	0.393519	-3.99088	6.58E-05	5.47E-04	3.669966	3.634384	3.928251	6.030007	5.310931	4.831847
AT1G08803	22.18367	-1.569582079	0.377161	-4.16157	3.16E-05	2.82E-04	3.312641	3.569791	3.525217	5.123743	5.019734	5.265734
AT5G03545	28.52582	-1.567552974	0.356041	-4.40274	1.07E-05	0.0001055	3.466342	3.91999	3.985697	5.319969	5.760417	5.356498
AT1G64660	2959.83	-1.567324475	0.158674	-9.87765	5.20E-23	3.32E-21	10.26537	10.60619	10.66785	12.38672	12.00239	11.92577
AT4G39070	50.59654	-1.566485744	0.326297	-4.8008	1.58E-06	1.82E-05	4.826091	4.067637	4.849451	6.607592	6.292644	5.899804
AT4G36930	59.58775	-1.564325959	0.288989	-5.41311	6.19E-08	8.97E-07	4.826091	4.949759	4.880072	5.99213	6.610887	6.825478
AT1G58190	105.8848	-1.563988281	0.296621	-5.27268	1.34E-07	1.86E-06	6.276368	5.40337	5.104121	7.45458	7.283413	7.284329
AT1G21680	3308.264	-1.559391327	0.074217	-21.0112	5.18E-98	7.83E-95	10.72079	10.68502	10.71585	12.23839	12.28354	12.29411
AT1G77210	1294.205	-1.55884167	0.15721	-9.91568	3.56E-23	2.29E-21	9.56771	8.98952	9.396272	10.90142	11.0347	10.82944
AT3G05845	27.38837	-1.558097799	0.395494	-3.93962	8.16E-05	0.0006638	3.669966	3.634384	3.868421	5.99213	5.310931	4.767144
AT1G70950	30.99682	-1.556483419	0.348237	-4.46962	7.84E-06	7.93E-05	3.956125	4.243584	3.525217	5.437371	5.760417	5.598855
AT5G22500	2521.757	-1.555567172	0.209172	-7.43679	1.03E-13	3.08E-12	9.923471	10.2169	10.62556	11.46252	12.18507	11.93547
AT2G28840	1415.578	-1.554691894	0.095253	-16.3218	6.91E-60	2.73E-57	9.530062	9.399208	9.520521	10.97487	11.07245	11.093
AT2G14170	1512.196	-1.5499909	0.136079	-11.3904	4.67E-30	4.46E-28	9.625976	9.475018	9.616844	10.86651	11.30122	11.22984
AT1G04133	15.41379	-1.548531794	0.474631	-3.2626	1.10E-03	6.59E-03	3.466342	2.091646	2.714973	4.812122	4.907958	4.208431
AT1G19550	162.8513	-1.543954921	0.179681	-8.59276	8.49E-18	3.62E-16	6.452298	6.459362	6.172301	7.810665	7.966403	8.02683
AT4G19160	2756.381	-1.538390544	0.125765	-12.2323	2.09E-34	2.51E-32	10.66779	10.33334	10.3253	12.1133	11.94514	11.95511
AT2G43620	28.58629	-1.537536442	0.444239	-3.46106	0.000538	0.003549	4.150129	3.277922	3.67243	6.301797	5.019734	4.699401
AT2G22450	467.9567	-1.535577344	0.119552	-12.8445	9.24E-38	1.33E-35	7.969501	7.796335	7.930976	9.394971	9.490449	9.459889
AT3G53980	105.7719	-1.534353076	0.243236	-6.30808	2.83E-10	5.71E-09	5.403549	5.93907	5.792368	7.495767	7.452309	6.976208
AT3G13270	83.36419	-1.528710978	0.250701	-6.09775	1.08E-09	2.00E-08	5.513465	5.421786	5.27515	6.607592	6.985988	7.272722
AT3G09745	201.2681	-1.527622665	0.17374	-8.79259	1.46E-18	6.61E-17	6.506476	6.756404	6.795447	8.232242	8.060895	8.39124
AT2G19450	135.7295	-1.520267595	0.189181	-8.03603	9.28E-16	3.33E-14	5.98984	6.185302	6.208946	7.709256	7.491614	7.786827
AT4G08305	79.27029	-1.520138921	0.289998	-5.2419	1.59E-07	2.17E-06	4.768132	5.267421	5.759806	6.680852	6.92986	7.09959
AT3G62090	229.1829	-1.515883108	0.172236	-8.80121	1.35E-18	6.16E-17	6.697136	6.946471	6.997482	8.349224	8.267496	8.614552
AT2G34655	43.42748	-1.513445176	0.45556	-3.32216	8.93E-04	5.47E-03	4.437236	4.067637	4.461161	4.812122	5.396255	7.08639
AT4G09020	314.1368	-1.504839014	0.195075	-7.71417	1.22E-14	3.95E-13	6.978444	7.460079	7.509364	8.639969	8.831881	9.108099
AT1G36060	26.95504	-1.504182304	0.379411	-3.96452	7.35E-05	6.06E-04	4.104018	3.10637	3.928251	5.319969	5.476813	5.399825
AT2G28900	1510.624	-1.502903992	0.205645	-7.30824	2.71E-13	7.70E-12	9.638161	9.304381	9.779984	10.60781	11.43073	11.25292
AT2G07766	10.77592	-1.501779457	0.480248	-3.12709	1.77E-03	9.88E-03	2.295555	2.559128	2.572256	4.021447	4.164273	4.302755
AT5G54960	257.9474	-1.498237535	0.16368	-9.15346	5.51E-20	2.76E-18	6.886097	7.142421	7.162264	8.457428	8.516472	8.75881
AT4G39090	10398.33	-1.493701603	0.151614	-9.85203	6.72E-23	4.21E-21	12.23772	12.31013	12.60568	13.61851	14.08819	13.98916
AT5G22505	324.1868	-1.49333579	0.221041	-6.75592	1.42E-11	3.37E-10	7.124178	7.319828	7.631374	8.429358	9.236518	8.981915
AT2G29670	1414.403	-1.493275395	0.175941	-8.48735	2.11E-17	8.71E-16	9.495876	9.432511	9.597396	10.56974	11.26481	11.18256

AT5G03240	5032.343	-1.490479369	0.092852	-16.0523	5.51E-58	1.97E-55	11.43741	11.25436	11.38402	12.88608	12.91681	12.77484
AT3G51730	2764.084	-1.489910802	0.152981	-9.73917	2.05E-22	1.25E-20	10.59256	10.31806	10.52983	11.67241	12.21474	12.06139
AT3G15620	129.0359	-1.487610529	0.195948	-7.59185	3.15E-14	9.80E-13	6.255428	6.04978	5.885862	7.46844	7.656331	7.640977
AT4G39110	27.42354	-1.487358818	0.387049	-3.84281	1.22E-04	9.43E-04	3.140592	4.201568	3.868421	5.694846	5.310931	5.218117
AT3G03470	614.7819	-1.487330046	0.185869	-8.00204	1.22E-15	4.34E-14	8.313891	8.310373	8.316823	10.23473	9.533458	9.633278
AT4G39675	17.47777	-1.485273135	0.418077	-3.55263	0.000381	0.0026156	3.046206	3.431225	2.963966	4.812122	5.019734	4.553554
AT5G20630	646.0389	-1.481119947	0.289707	-5.11248	3.18E-07	4.12E-06	9.051068	7.827749	7.748022	9.880304	10.13959	9.712737
AT1G22490	30.81461	-1.478800902	0.353431	-4.18413	2.86E-05	2.58E-04	3.669966	3.867214	4.377786	5.319969	5.625572	5.740407
AT3G07650	138.6815	-1.475428245	0.193297	-7.63294	2.29E-14	7.23E-13	6.357218	6.217283	5.959397	7.799743	7.585434	7.676644
AT5G18937	27.78243	-1.472986598	0.355812	-4.13979	3.48E-05	3.06E-04	4.150129	3.569791	3.806003	5.597298	5.310931	5.356498
AT1G19570	1676.423	-1.469953098	0.089727	-16.3825	2.55E-60	1.06E-57	9.766739	9.787027	9.821989	11.18041	11.29845	11.32493
AT5G45830	152.7401	-1.460879767	0.204152	-7.15585	8.32E-13	2.28E-11	6.488642	6.174482	6.279555	7.732415	8.112236	7.595115
AT5G06690	978.9301	-1.46045942	0.167833	-8.70186	3.26E-18	1.44E-16	8.992161	8.888864	9.126234	10.17471	10.81214	10.43433
AT4G39260	262.3661	-1.460282937	0.331846	-4.40048	1.08E-05	0.0001065	6.063973	7.810381	6.678374	8.471261	8.695459	8.70369
AT5G63810	697.0279	-1.457513487	0.200893	-7.25518	4.01E-13	1.12E-11	8.278	8.722418	8.498015	9.523344	10.14576	10.25866
AT2G15790	113.3654	-1.455507606	0.340775	-4.27117	1.94E-05	1.82E-04	5.726274	6.152596	5.584968	8.131308	6.574739	7.112669
AT3G47340	27827.8	-1.453342233	0.112779	-12.8866	5.36E-38	7.82E-36	13.94397	13.75376	13.85185	15.25884	15.49604	15.18275
AT3G61070	587.952	-1.452363205	0.213918	-6.78934	1.13E-11	2.71E-10	8.395116	8.109173	8.271346	9.184378	10.11777	9.844019
AT1G55510	439.549	-1.452167336	0.122855	-11.8201	3.07E-32	3.39E-30	7.896987	7.824292	7.89019	9.217584	9.436078	9.350965
AT4G11910	18.26634	-1.425563275	0.422644	-3.37297	7.44E-04	4.68E-03	3.466342	3.10637	3.271714	5.319969	4.346824	4.699401
AT1G77000	117.2246	-1.424278144	0.218675	-6.51322	7.36E-11	1.61E-09	6.051879	5.873683	5.959397	7.276958	7.772588	7.213246
AT2G37130	175.0642	-1.42149515	0.219375	-6.47976	9.19E-11	1.98E-09	6.90639	6.32883	6.324778	8.199376	8.007659	7.811102
AT2G19970	16.85476	-1.421165168	0.444094	-3.20014	1.37E-03	0.0079819	3.140592	3.277922	3.074005	5.319969	3.955229	4.62832
AT1G13990	467.3019	-1.421089032	0.11415	-12.4493	1.41E-35	1.86E-33	8.04466	7.965931	7.93827	9.409413	9.451111	9.38909
AT5G64570	1235.728	-1.418284525	0.199999	-7.09147	1.33E-12	3.57E-11	9.791499	9.070537	9.111793	10.92171	10.94019	10.5874
AT5G14920	1149.303	-1.413741482	0.208446	-6.78229	1.18E-11	2.84E-10	9.176119	8.883891	9.623646	10.8652	10.89876	10.3304
AT2G19810	1278.461	-1.4134585	0.147319	-9.59456	8.43E-22	4.88E-20	9.639171	9.208195	9.415997	10.66611	11.00953	10.90562
AT3G20380	32.87377	-1.413107443	0.362475	-3.8985	9.68E-05	0.0007693	4.509766	4.201568	3.445564	5.741253	5.760417	5.441888
AT5G24490	8067.083	-1.412962616	0.132126	-10.6941	1.08E-26	8.58E-25	11.96692	12.08001	12.21761	13.3047	13.7334	13.4987
AT1G28230	46.96053	-1.410551991	0.305016	-4.62452	3.75E-06	4.06E-05	4.509766	4.507728	4.93943	6.476563	5.883728	5.987621
AT5G28145	53.34186	-1.408860175	0.343234	-4.10467	4.05E-05	0.0003522	5.403549	4.243584	4.501109	6.172293	6.574739	6.222936
AT5G10860	3558.208	-1.404465315	0.109016	-12.8831	5.60E-38	8.13E-36	10.98408	10.79153	10.98245	12.18588	12.39851	12.41881
AT2G21820	17.12204	-1.404321128	0.440918	-3.185	1.45E-03	8.34E-03	3.466342	3.10637	2.844834	4.896561	5.123469	4.107507
AT3G51325	14.33272	-1.403155658	0.44626	-3.14426	0.001665	0.0093829	3.22918	2.803425	2.844834	4.29471	4.346824	4.831847

AT3G15450	22160.91	-1.401059147	0.134232	-10.4376	1.67E-25	1.24E-23	13.7107	13.3523	13.5885	14.77032	15.13602	15.00295
AT5G64572	712.7416	-1.400011517	0.204538	-6.84476	7.66E-12	1.89E-10	8.987407	8.332442	8.325192	10.14265	10.16716	9.734054
AT4G29190	2171.534	-1.399069517	0.140392	-9.96549	2.16E-23	1.41E-21	10.42744	9.965525	10.19283	11.51187	11.76338	11.59394
AT2G15830	70.4899	-1.398629745	0.234832	-5.95588	2.59E-09	4.58E-08	5.345282	5.226103	5.251924	6.817077	6.574739	6.709736
AT1G06350	62.00034	-1.381388936	0.262622	-5.26	1.44E-07	1.98E-06	5.305088	4.97492	4.996442	6.362435	6.420349	6.743763
AT2G43830	58.51682	-1.379206093	0.295263	-4.67111	3.00E-06	3.31E-05	5.012364	4.97492	4.996442	6.901235	6.247434	6.04333
AT1G62420	67.76109	-1.375652796	0.283638	-4.85004	1.23E-06	1.45E-05	5.58235	5.204992	4.685768	6.607592	6.779171	6.564985
AT1G04903	1818.167	-1.373530513	0.154209	-8.90691	5.25E-19	2.49E-17	9.996277	10.04118	9.85512	11.49673	11.53788	11.00191
AT1G04907	1817.958	-1.373263816	0.154346	-8.89731	5.72E-19	2.70E-17	9.996277	10.04118	9.85512	11.49673	11.53788	11.00103
AT4G04180	81.67982	-1.372603971	0.226602	-6.05733	1.38E-09	2.54E-08	5.615596	5.457928	5.448611	7.018926	6.680573	6.99045
AT1G30250	36.91426	-1.372054964	0.362654	-3.78337	1.55E-04	1.17E-03	4.61215	3.812433	4.461161	5.379864	5.625572	6.246861
AT1G03620	34.44993	-1.371958129	0.315916	-4.34279	1.41E-05	0.0001359	4.23815	4.362754	4.145468	5.545934	5.82339	5.706299
AT1G09421	45.59226	-1.371196584	0.29722	-4.61341	3.96E-06	4.26E-05	4.509766	4.844481	4.577832	5.741253	6.247434	6.222936
AT2G43400	785.0172	-1.369610662	0.12542	-10.9202	9.23E-28	7.73E-26	8.87177	8.731673	8.689544	9.960952	10.23532	10.23792
AT5G54090	334.8494	-1.367912757	0.155806	-8.77956	1.64E-18	7.35E-17	7.747532	7.414827	7.428806	8.807449	8.906489	9.048089
AT3G20395	115.8197	-1.366878659	0.310226	-4.40608	1.05E-05	0.0001041	6.327428	5.594069	5.82421	6.859769	8.007659	7.151212
AT5G44572	106.1282	-1.366055982	0.247568	-5.5179	3.43E-08	5.15E-07	6.11136	5.762479	5.657465	7.45458	7.472095	6.85691
AT3G46970	761.9748	-1.364692278	0.139423	-9.78811	1.27E-22	7.84E-21	8.620654	8.748186	8.805749	9.893206	10.08281	10.31209
AT1G08513	47.37179	-1.360754808	0.303293	-4.4866	7.24E-06	7.39E-05	5.012364	4.400386	4.685768	6.362435	5.82339	6.17386
AT3G60930	123.9038	-1.360684209	0.26051	-5.22316	1.76E-07	2.39E-06	6.134483	6.338556	5.726492	7.925648	7.142352	7.329843
AT5G59570	36.89867	-1.360598922	0.31946	-4.25905	2.05E-05	1.91E-04	4.280226	4.639454	4.094152	5.953231	5.69457	5.706299
AT1G17665	21.391	-1.348507975	0.381933	-3.53074	4.14E-04	2.81E-03	3.791321	3.431225	3.525217	4.896561	5.019734	5.168875
AT1G28050	105.9803	-1.346866167	0.224492	-5.99963	1.98E-09	3.58E-08	5.756397	6.185302	5.657465	7.162256	7.370331	7.284329
AT1G79440	513.3198	-1.346397313	0.13544	-9.94094	2.76E-23	1.81E-21	8.068868	8.244651	8.197287	9.343255	9.55681	9.675759
AT1G18270	1499.096	-1.34376349	0.128482	-10.4588	1.34E-25	1.00E-23	9.759321	9.749244	9.638275	10.82132	11.18314	11.19337
AT4G13250	1133.897	-1.343220491	0.151121	-8.88838	6.20E-19	2.90E-17	9.26916	9.221396	9.432877	10.34778	10.78669	10.84127
AT5G18130	843.4165	-1.340212132	0.123375	-10.863	1.73E-27	1.42E-25	9.043454	8.803658	8.811718	10.11868	10.35204	10.25719
AT5G64260	1500.025	-1.340124756	0.191592	-6.99468	2.66E-12	6.87E-11	9.901736	9.338708	9.81309	10.71198	11.34614	11.11754
AT5G02020	898.205	-1.339366647	0.149224	-8.97552	2.82E-19	1.37E-17	8.907444	8.994142	9.035637	10.07405	10.29208	10.59674
AT1G26665	340.0273	-1.337937459	0.185329	-7.21927	5.23E-13	1.45E-11	7.910446	7.377575	7.370693	8.917636	8.816485	9.075069
AT5G01520	211.1947	-1.33639861	0.182727	-7.31365	2.60E-13	7.41E-12	6.872409	6.888065	6.918555	7.905412	8.376502	8.428329
AT3G07105	25.39261	-1.332564734	0.408026	-3.26588	1.09E-03	6.53E-03	4.360866	3.75549	2.963966	5.25748	5.220243	5.356498
AT3G02040	409.3181	-1.324746206	0.136255	-9.72254	2.42E-22	1.46E-20	7.985486	7.775006	7.80099	9.11117	9.347801	9.133989
AT1G60740	19.87182	-1.324301293	0.389847	-3.39698	0.000681	0.0043476	3.605233	3.277922	3.6007	4.97633	4.907958	4.893774

AT1G05835	201.6904	-1.320619323	0.16149	-8.1777	2.89E-16	1.08E-14	6.98482	6.792233	6.754717	8.248399	8.060895	8.226539
AT1G22370	227.2371	-1.316865972	0.192247	-6.84986	7.39E-12	1.83E-10	6.735449	7.114389	7.137131	8.059868	8.544777	8.423089
AT4G37580	76.28748	-1.313982913	0.22763	-5.77244	7.81E-09	1.28E-07	5.58235	5.421786	5.320509	6.795248	6.810594	6.777007
AT3G48690	1239.875	-1.312602656	0.14777	-8.88275	6.52E-19	3.04E-17	9.577234	9.406162	9.388306	10.82402	11.01629	10.50051
AT4G36450	35.17657	-1.31151138	0.351634	-3.72976	0.000192	0.001416	4.707748	3.812433	4.289296	5.437371	5.69457	5.958939
AT3G06850	796.288	-1.310270375	0.171052	-7.66006	1.86E-14	5.92E-13	8.831658	8.713102	8.904006	9.814003	10.48215	10.10188
AT5G48180	790.2255	-1.304903394	0.188979	-6.90501	5.02E-12	1.26E-10	8.514493	9.166486	8.65676	9.968286	10.36797	10.07707
AT4G36410	65.95414	-1.303502585	0.327736	-3.97729	6.97E-05	5.76E-04	5.177314	5.346667	5.024124	5.786214	7.166841	6.545826
AT5G67480	1707.38	-1.303193816	0.119296	-10.924	8.85E-28	7.46E-26	10.04971	9.786138	9.957375	11.39064	11.22601	11.12482
AT5G24500	762.3506	-1.300874834	0.131731	-9.87522	5.33E-23	3.38E-21	8.753536	8.807164	8.757088	9.838224	10.20903	10.1909
AT5G20640	26.70819	-1.30028003	0.359697	-3.61493	3.00E-04	2.11E-03	4.150129	3.75549	3.928251	5.492674	4.907958	5.482759
AT3G46640	391.1912	-1.30026121	0.152279	-8.53866	1.36E-17	5.69E-16	7.670527	7.806883	7.930976	8.937702	9.337051	9.088372
AT2G21130	45.38397	-1.298784313	0.307111	-4.22904	2.35E-05	0.0002155	4.88181	4.541804	4.650679	5.597298	6.200761	6.293554
AT5G57240	271.6805	-1.298334013	0.249301	-5.20791	1.91E-07	2.57E-06	7.095007	7.244235	7.407944	7.95548	9.084138	8.596044
AT1G06560	346.1243	-1.29619759	0.15657	-8.27869	1.25E-16	4.81E-15	7.855842	7.533893	7.479679	8.97216	9.051631	8.827852
AT5G38940	41.3572	-1.295231016	0.371546	-3.48606	0.00049	0.0032649	4.797403	4.701066	3.985697	5.25748	5.883728	6.446015
AT1G06460	647.8279	-1.291528028	0.15172	-8.51256	1.70E-17	7.06E-16	8.437566	8.417493	8.744662	9.625844	10.01023	9.8979
AT1G08115	16120.29	-1.285049748	0.156405	-8.21618	2.10E-16	7.95E-15	13.253	13.16011	13.12051	14.59308	14.70893	14.08767
AT3G29575	388.6227	-1.284056417	0.193395	-6.63956	3.15E-11	7.21E-10	7.713388	8.062988	7.58566	8.751866	9.253768	9.291818
AT2G04690	947.9284	-1.281587561	0.1163	-11.0197	3.07E-28	2.64E-26	9.163529	8.95832	9.18106	10.28455	10.41473	10.48173
AT1G10090	442.1025	-1.278138829	0.15006	-8.5175	1.63E-17	6.79E-16	8.170244	7.924557	7.901428	9.102308	9.30982	9.459889
AT4G03510	1007.129	-1.277863087	0.126175	-10.1277	4.16E-24	2.88E-22	9.263927	9.02609	9.270454	10.31938	10.60148	10.51536
AT4G30662	123.1294	-1.277038952	0.277387	-4.60382	4.15E-06	4.43E-05	5.513465	6.107802	6.588523	7.212535	7.548635	7.63192
AT1G26450	31.58589	-1.276982924	0.330044	-3.86913	1.09E-04	8.55E-04	4.360866	4.201568	3.985697	5.545934	5.69457	5.399825
AT5G02140	58.36786	-1.276604998	0.295513	-4.31996	1.56E-05	0.0001493	5.459553	4.816916	4.818167	6.632428	6.420349	6.148682
AT4G21650	380.4193	-1.273359734	0.205759	-6.1886	6.07E-10	1.16E-08	7.267032	8.109173	7.828726	9.010559	9.128451	9.117863
AT3G05800	50.93177	-1.270578344	0.325863	-3.89911	9.65E-05	0.0007678	4.473957	5.071394	4.996442	5.597298	6.379024	6.526408
AT5G37010	62.74424	-1.264900024	0.270754	-4.67177	2.99E-06	3.30E-05	5.061448	5.43997	5.024124	6.172293	6.680573	6.620982
AT3G04070	24.4545	-1.26402172	0.362015	-3.49163	4.80E-04	3.21E-03	3.956125	3.867214	3.740762	5.25748	4.907958	5.356498
AT4G34860	127.7459	-1.263573676	0.216433	-5.83818	5.28E-09	8.88E-08	6.075967	6.206701	6.390061	7.697535	7.11744	7.649977
AT2G18260	53.98631	-1.261173729	0.290746	-4.33772	1.44E-05	0.0001387	4.935458	4.816916	5.104121	6.066916	6.714193	6.015745
AT1G04433	154.1223	-1.259534237	0.175831	-7.16332	7.87E-13	2.16E-11	6.609086	6.367346	6.512382	7.874516	7.756543	7.702828
AT4G01870	1069.207	-1.258368837	0.155555	-8.08956	5.99E-16	2.19E-14	9.143723	9.473915	9.212982	10.40835	10.83333	10.4173
AT3G48360	3280.562	-1.257630953	0.153678	-8.18355	2.76E-16	1.03E-14	11.22461	10.70583	10.71585	12.26792	12.1685	12.10622

AT2G29630	1781.201	-1.251615805	0.147462	-8.48772	2.11E-17	8.69E-16	10.01587	10.02546	10.04274	10.94296	11.46446	11.43381
AT4G33490	424.9526	-1.250563002	0.166703	-7.50173	6.30E-14	1.91E-12	8.062854	7.810381	8.002302	8.907497	9.337051	9.410432
AT3G55450	197.2417	-1.250552119	0.17885	-6.9922	2.71E-12	6.99E-11	6.97204	6.792233	6.827223	7.994319	7.980286	8.375048
AT2G33150	3337.916	-1.24980714	0.117699	-10.6187	2.44E-26	1.88E-24	10.74728	11.04644	11.00867	12.07645	12.29539	12.22691
AT5G52190	249.6134	-1.249680763	0.240193	-5.20282	1.96E-07	2.64E-06	7.175244	7.080015	7.252795	7.915566	8.970488	8.358672
AT1G79360	121.4104	-1.249650919	0.224573	-5.56457	2.63E-08	4.01E-07	6.327428	5.900194	6.172301	7.509239	7.656331	7.112669
AT5G28050	803.3794	-1.247714631	0.170277	-7.32758	2.34E-13	6.72E-12	8.885464	8.800142	8.938966	9.885479	10.50873	9.985829
AT5G49015	47.43726	-1.247056137	0.300576	-4.14889	3.34E-05	0.0002955	4.85422	4.472827	5.051286	5.913254	5.997325	6.360876
AT1G55810	418.1061	-1.23949028	0.159423	-7.77485	7.55E-15	2.49E-13	8.107358	7.764221	7.945526	8.97216	9.390016	9.23314
AT2G40970	146.5532	-1.239058448	0.18637	-6.64839	2.96E-11	6.80E-10	6.532821	6.238216	6.531797	7.561902	7.772588	7.762137
AT1G69480	21.01982	-1.238444373	0.379856	-3.2603	0.001113	0.0066415	3.537458	3.75549	3.67243	5.051918	4.786789	5.065041
AT1G53450	423.5044	-1.238315766	0.151495	-8.17398	2.98E-16	1.12E-14	8.02004	8.012241	7.875068	8.97216	9.248041	9.418355
AT4G24040	122.3998	-1.237953707	0.191418	-6.46727	9.98E-11	2.14E-09	6.255428	6.217283	6.069773	7.58753	7.370331	7.363061
AT4G30660	247.2444	-1.236423274	0.263035	-4.7006	2.59E-06	2.90E-05	6.506476	7.108716	7.658123	8.207663	8.544777	8.605328
AT1G06180	51.77478	-1.233052727	0.285868	-4.31336	1.61E-05	1.53E-04	5.085378	5.071394	4.650679	6.391825	5.883728	6.338783
AT4G15990	27.39648	-1.230101319	0.362195	-3.39624	6.83E-04	4.35E-03	4.280226	4.158291	3.525217	5.437371	5.220243	5.356498
AT3G63215	77.15307	-1.22957023	0.232186	-5.29562	1.19E-07	1.65E-06	5.679876	5.365817	5.508634	6.921529	6.714193	6.709736
AT3G62550	2685.727	-1.228928935	0.160158	-7.67324	1.68E-14	5.35E-13	10.45008	10.65954	10.77255	11.55635	12.14326	11.89913
AT1G18710	141.0955	-1.227445919	0.203211	-6.04025	1.54E-09	2.80E-08	6.609086	6.395572	6.147344	7.697535	7.432247	7.778644
AT2G26740	698.6682	-1.227277431	0.126894	-9.67167	3.98E-22	2.36E-20	8.715708	8.673305	8.702452	9.951114	10.11148	9.742493
AT1G12240	2386.234	-1.224700926	0.104279	-11.7444	7.54E-32	8.11E-30	10.38529	10.518	10.51771	11.73467	11.81755	11.56806
AT3G18530	26.58754	-1.223878404	0.352536	-3.47164	5.17E-04	3.42E-03	4.104018	3.696207	4.145468	5.25748	5.310931	5.31183
AT3G62650	2289.763	-1.223788308	0.137316	-8.91223	5.00E-19	2.38E-17	10.43444	10.23198	10.54961	11.51521	11.87163	11.54352
AT5G57785	340.5843	-1.223777209	0.179823	-6.80544	1.01E-11	2.44E-10	7.806308	7.414827	7.714432	8.790998	9.189485	8.708006
AT2G37200	255.544	-1.223395272	0.15916	-7.68656	1.51E-14	4.84E-13	7.33847	7.212852	7.193077	8.664258	8.506912	8.291245
AT1G70300	231.4505	-1.220753962	0.177221	-6.88832	5.65E-12	1.41E-10	7.372904	6.990318	6.918555	8.450462	8.323029	8.279694
AT3G10410	638.3128	-1.220433866	0.14762	-8.2674	1.37E-16	5.26E-15	8.383099	8.71497	8.595803	9.588255	9.965564	9.853789
AT4G14270	1452.188	-1.219127837	0.143146	-8.51669	1.64E-17	6.83E-16	9.6422	9.687798	9.929283	10.74081	11.04629	11.16383
AT3G47160	531.014	-1.218811442	0.148088	-8.23033	1.87E-16	7.12E-15	8.230503	8.337301	8.36091	9.281787	9.566046	9.746694
AT1G58270	926.1967	-1.214753042	0.109537	-11.0899	1.40E-28	1.24E-26	9.184452	9.086498	9.075872	10.23473	10.47481	10.31067
AT4G27130	949.9798	-1.21419489	0.155315	-7.81761	5.38E-15	1.82E-13	9.372452	8.851991	9.168711	10.43683	10.47726	10.22141
AT1G16510	36.63258	-1.211080472	0.33596	-3.60483	3.12E-04	2.19E-03	4.797403	4.201568	4.289296	6.066916	5.476813	5.598855
AT2G39900	165.7643	-1.207226546	0.22607	-5.34006	9.29E-08	1.31E-06	6.405541	6.861328	6.569861	7.426454	8.112236	7.998924
AT3G30775	1990.53	-1.205019857	0.21129	-5.70315	1.18E-08	1.88E-07	10.45468	9.803799	10.28224	11.40602	11.81174	11.03492

AT3G12750	29.52776	-1.204176489	0.353162	-3.4097	0.00065	0.0041788	4.23815	4.437061	3.67243	5.545934	5.310931	5.441888
AT3G05880	1940.744	-1.204065925	0.215808	-5.57935	2.41E-08	3.69E-07	9.827416	10.11094	10.4913	10.92422	11.74011	11.46793
AT2G45210	60.68302	-1.202535329	0.296979	-4.04923	5.14E-05	0.0004368	5.364967	4.924151	5.204319	6.859769	6.336481	6.015745
AT3G10020	1180.84	-1.198104919	0.195878	-6.11657	9.56E-10	1.79E-08	9.256042	9.813421	9.238316	10.72069	10.94551	10.36223
AT5G46180	750.8985	-1.197042256	0.103219	-11.5971	4.26E-31	4.33E-29	8.803081	8.836631	8.856677	10.09431	9.93381	10.06703
AT1G03470	82.44412	-1.196562393	0.270304	-4.42673	9.57E-06	9.53E-05	5.741414	5.510498	5.603436	6.270495	7.190921	7.018519
AT5G20250	9055.353	-1.19411195	0.138911	-8.59626	8.24E-18	3.52E-16	12.56525	12.32013	12.34843	13.44974	13.87993	13.51234
AT1G64380	49.22676	-1.190480822	0.348003	-3.4209	6.24E-04	4.03E-03	4.88181	4.639454	5.077945	5.319969	6.200761	6.639175
AT1G14890	234.3754	-1.190151621	0.200498	-5.93599	2.92E-09	5.12E-08	7.323457	7.014788	7.052287	8.013353	8.687018	8.319723
AT5G06370	535.0927	-1.189640571	0.136503	-8.71511	2.90E-18	1.29E-16	8.437566	8.140089	8.432382	9.423711	9.58888	9.617306
AT2G26750	83.87814	-1.188763619	0.227067	-5.23529	1.65E-07	2.24E-06	5.710973	5.641983	5.639679	7.056115	6.958197	6.639175
AT3G02525	40.66815	-1.188736742	0.324373	-3.66472	2.48E-04	1.79E-03	4.509766	4.639454	4.685768	6.301797	5.760417	5.441888
AT5G55970	356.2236	-1.188165379	0.138405	-8.58469	9.11E-18	3.87E-16	7.777219	7.667219	7.828726	8.790998	9.018374	9.054882
AT3G02030	191.8089	-1.185958893	0.161499	-7.34343	2.08E-13	6.00E-12	6.978444	6.756404	6.850603	8.09603	8.137237	7.977634
AT5G23660	394.7488	-1.185703817	0.195471	-6.06587	1.31E-09	2.42E-08	7.416003	8.036344	8.138345	9.205221	9.018374	9.10483
AT2G23170	602.7652	-1.184645161	0.205567	-5.76282	8.27E-09	1.35E-07	8.478802	8.536347	8.490592	10.17893	9.514501	9.331517
AT5G58600	139.6227	-1.183478485	0.206839	-5.72175	1.05E-08	1.69E-07	6.286726	6.24857	6.643102	7.368501	7.788457	7.66781
AT1G06980	79.99381	-1.181561304	0.265711	-4.4468	8.72E-06	8.73E-05	5.530997	5.307588	5.870694	6.503738	7.166841	6.743763
AT3G47800	828.0116	-1.181335416	0.131925	-8.95457	3.41E-19	1.65E-17	9.072179	8.855383	8.987232	10.11428	10.36797	10.01573
AT2G46610	167.3919	-1.180029004	0.173687	-6.79401	1.09E-11	2.63E-10	6.830546	6.545861	6.643102	7.965288	7.772588	7.873905
AT3G27940	41.57005	-1.17812802	0.344319	-3.42162	6.22E-04	4.02E-03	4.707748	4.607633	4.650679	6.476563	5.220243	5.740407
AT2G45720	155.0967	-1.176215165	0.204273	-5.75806	8.51E-09	1.39E-07	6.70488	6.404859	6.550955	7.412182	7.966403	7.866203
AT3G16180	150.6874	-1.174305473	0.264178	-4.44514	8.78E-06	8.80E-05	6.317359	6.459362	6.704273	7.018926	7.966403	8.012944
AT4G14548	38.46814	-1.173712799	0.363941	-3.22501	0.00126	0.0074003	4.826091	3.812433	4.786189	5.99213	5.310931	5.987621
AT2G35070	72.6388	-1.172454435	0.274704	-4.26806	1.97E-05	0.000184	5.345282	5.641983	5.407161	7.110162	6.247434	6.564985
AT5G26570	999.2141	-1.1700424	0.131221	-8.91656	4.81E-19	2.29E-17	9.090031	9.393388	9.273341	10.30594	10.39931	10.59674
AT4G26670	234.4929	-1.166538926	0.16887	-6.90791	4.92E-12	1.24E-10	7.077217	7.062516	7.332454	8.224096	8.563343	8.256311
AT2G09660	59.56283	-1.166069632	0.276922	-4.21082	2.54E-05	2.32E-04	5.263741	5.117315	5.129831	6.066916	6.810594	6.246861
AT2G39705	142.0147	-1.165812319	0.179994	-6.47694	9.36E-11	2.02E-09	6.558693	6.357813	6.442294	7.63746	7.510873	7.737017
AT1G46768	88.34436	-1.163863418	0.272242	-4.2751	1.91E-05	0.000179	5.800434	5.54451	5.900872	6.448866	6.92986	7.363061
AT2G38530	443.9405	-1.160670791	0.177408	-6.54237	6.06E-11	1.33E-09	8.170244	8.000036	8.083485	9.610301	9.134672	8.996101
AT1G49720	334.0649	-1.160599933	0.138064	-8.40624	4.23E-17	1.70E-15	7.646601	7.604113	7.792967	8.740488	8.977427	8.851451
AT1G07653	27.83548	-1.157952668	0.346528	-3.34159	8.33E-04	5.17E-03	4.007126	4.362754	3.928251	5.379864	5.220243	5.399825
AT4G27460	33.72596	-1.157618205	0.336096	-3.44431	5.73E-04	3.74E-03	4.23815	4.362754	4.53998	5.99213	5.220243	5.482759

AT1G01420	101.5207	-1.156727055	0.279027	-4.14557	3.39E-05	2.99E-04	6.276368	5.747956	5.743245	6.556599	7.411902	7.373965
AT5G56100	388.9016	-1.156324348	0.225733	-5.12252	3.01E-07	3.93E-06	8.203425	7.516861	7.848216	8.711644	9.451111	8.999626
AT1G08890	302.0378	-1.149750433	0.145007	-7.92892	2.21E-15	7.72E-14	7.448656	7.538119	7.658123	8.590128	8.687018	8.8397
AT1G03580	115.3192	-1.148932422	0.232009	-4.9521	7.34E-07	8.98E-06	6.46147	5.900194	6.001784	7.245106	7.567152	7.176347
AT5G00820	133.0702	-1.148931011	0.201851	-5.69196	1.26E-08	2.00E-07	6.58411	6.141527	6.313604	7.574773	7.638931	7.395531
AT5G49450	758.3349	-1.148307945	0.15553	-7.38321	1.55E-13	4.53E-12	9.017258	8.586184	8.964194	9.905994	10.22661	9.960732
AT2G30615	380.6029	-1.148262571	0.162277	-7.07594	1.48E-12	3.95E-11	7.817066	7.86187	7.930976	8.892154	9.342436	8.855347
AT5G02935	32.70208	-1.148248981	0.341719	-3.36021	0.000779	0.0048716	4.578823	3.867214	4.461161	5.646896	5.625572	5.399825
AT1G77410	30.38994	-1.147745455	0.358755	-3.19925	1.38E-03	8.00E-03	3.669966	4.575094	4.289296	5.545934	5.476813	5.356498
AT3G22460	75.24483	-1.146194043	0.234763	-4.88234	1.05E-06	1.24E-05	5.548318	5.594069	5.468897	6.859769	6.810594	6.486772
AT1G76800	80.24007	-1.142426237	0.23884	-4.78323	1.72E-06	1.98E-05	5.648094	5.457928	5.759806	6.704469	7.09209	6.620982
AT5G07745	4754.557	-1.140081289	0.357753	-3.18678	1.44E-03	8.30E-03	11.55562	11.57289	11.17457	12.87279	13.14195	11.82408
AT5G66170	55.51828	-1.133404509	0.280699	-4.03779	5.40E-05	0.0004561	5.108917	5.307588	4.849451	5.99213	6.574739	6.270397
AT5G66400	260.9132	-1.132316828	0.242011	-4.67878	2.89E-06	3.20E-05	6.6255	7.554903	7.60412	8.525303	8.544777	8.443937
AT1G65330	50.47431	-1.13223537	0.304632	-3.71673	2.02E-04	1.48E-03	4.509766	5.047874	5.228318	6.205775	6.379024	5.869303
AT3G10190	114.9452	-1.131287919	0.265824	-4.25577	2.08E-05	1.93E-04	6.532821	5.610218	6.159877	7.574773	7.166841	7.213246
AT4G27450	4212.472	-1.129831772	0.124833	-9.05074	1.42E-19	7.02E-18	11.51984	11.15156	11.38335	12.36466	12.53057	12.59982
AT4G35750	2444.1	-1.129704108	0.099945	-11.3032	1.26E-29	1.18E-27	10.69526	10.47069	10.55615	11.6769	11.79318	11.67278
AT1G12710	113.6911	-1.124736902	0.204406	-5.50247	3.75E-08	5.59E-07	6.027383	6.025901	6.390061	7.308122	7.283413	7.318598
AT2G22720	1025.91	-1.124198175	0.15279	-7.35782	1.87E-13	5.40E-12	9.407282	9.291921	9.264664	10.76207	10.33323	10.24536
AT1G17460	71.99159	-1.124114968	0.274683	-4.0924	4.27E-05	3.70E-04	5.441126	5.610218	5.407161	6.102904	6.779171	6.961824
AT3G26512	708.2881	-1.122325428	0.153433	-7.31475	2.58E-13	7.36E-12	8.875205	8.610471	8.864354	9.668496	10.15191	9.933349
AT1G28070	89.81199	-1.118242595	0.236192	-4.73446	2.20E-06	2.48E-05	5.98984	5.475665	5.944987	6.859769	6.92986	7.09959
AT3G60530	1081.852	-1.118182099	0.156547	-7.1428	9.15E-13	2.49E-11	9.444763	9.092258	9.619115	10.46476	10.70335	10.43823
AT1G12780	3162.456	-1.115893192	0.089671	-12.4444	1.50E-35	1.97E-33	10.98368	10.96284	10.92992	11.99799	12.19255	12.04911
AT1G20030	35.66785	-1.113215995	0.318257	-3.49785	4.69E-04	3.14E-03	4.578823	4.324114	4.53998	5.379864	5.941644	5.635566
AT2G28110	65.71721	-1.112514365	0.326023	-3.41238	6.44E-04	4.14E-03	5.384387	5.493187	5.15509	7.145099	5.82339	6.316346
AT5G43500	154.0508	-1.11227609	0.295361	-3.76581	0.000166	0.0012426	6.452298	6.651072	6.597764	6.817077	7.880172	8.214456
AT4G34970	104.8286	-1.110865678	0.231107	-4.80672	1.53E-06	1.78E-05	6.255428	5.791094	6.01564	6.980752	7.472095	7.073069
AT4G16190	4761.073	-1.10858546	0.08544	-12.975	1.70E-38	2.58E-36	11.52614	11.49621	11.6386	12.66506	12.72761	12.61397
AT4G02005	72.75988	-1.106912734	0.265913	-4.16269	3.15E-05	2.81E-04	5.870968	5.40337	5.204319	6.530411	6.610887	6.841279
AT2G42790	837.5508	-1.106747936	0.099496	-11.1235	9.64E-29	8.66E-27	9.028102	9.018548	9.102085	10.08983	10.20903	10.18936
AT2G02930	109.9401	-1.105811219	0.327001	-3.38167	0.00072	0.0045553	6.575687	5.951799	5.508634	7.810665	6.747048	7.032351
AT1G80315	54.29642	-1.105710788	0.300918	-3.67446	0.000238	0.0017266	4.826091	4.87153	5.468897	6.420628	6.379024	5.929675

AT3G17810	629.4487	-1.104282946	0.111903	-9.86821	5.72E-23	3.62E-21	8.63896	8.614479	8.672152	9.674487	9.70212	9.867357
AT2G22980	1691.45	-1.100330134	0.114612	-9.6005	7.96E-22	4.61E-20	10.15663	10.10097	9.930198	11.03082	11.19955	11.28504
AT1G13300	31.64578	-1.100237296	0.349675	-3.14646	1.65E-03	0.0093228	4.738256	3.970903	4.145468	5.379864	5.553109	5.561185
AT2G01080	96.92534	-1.099585514	0.223202	-4.9264	8.38E-07	1.02E-05	5.898243	5.703489	6.172301	6.999965	7.214606	6.99045
AT2G09400	51.16121	-1.099173315	0.321356	-3.42042	6.25E-04	4.04E-03	5.037115	4.816916	5.204319	6.172293	5.476813	6.620982
AT1G16490	34.47343	-1.095805306	0.313254	-3.49813	0.000469	0.0031423	4.509766	4.284411	4.577832	5.646896	5.625572	5.561185
AT2G25450	2997.024	-1.094746523	0.102442	-10.6865	1.18E-26	9.28E-25	10.9741	10.89196	10.81378	12.07248	12.05828	11.85505
AT2G22080	218.3665	-1.093909501	0.184166	-5.93981	2.85E-09	5.01E-08	6.991167	7.269875	7.052287	7.925648	8.448184	8.273883
AT3G56210	254.8096	-1.093773923	0.15522	-7.04663	1.83E-12	4.83E-11	7.516233	7.319828	7.16848	8.457428	8.397349	8.489773
AT4G19850	104.1503	-1.092099595	0.212889	-5.1299	2.90E-07	3.79E-06	5.938213	6.073269	6.10908	6.96128	7.432247	7.08639
AT3G01695	18567.23	-1.091995438	0.182477	-5.98428	2.17E-09	3.90E-08	13.66522	13.40677	13.45971	14.77251	14.89014	14.1308
AT3G01715	18566.24	-1.091962414	0.182543	-5.98193	2.21E-09	3.95E-08	13.66515	13.40662	13.45971	14.77242	14.89026	14.13039
AT1G10682	247.2155	-1.091745139	0.300931	-3.62789	0.000286	0.0020217	7.907093	6.914316	6.597764	8.318945	8.736939	8.158795
AT4G20930	158.2015	-1.089695155	0.179566	-6.06848	1.29E-09	2.38E-08	6.823449	6.57083	6.541408	7.755209	7.880172	7.658921
AT3G19990	334.4726	-1.088804976	0.132531	-8.2155	2.11E-16	7.98E-15	7.743778	7.778582	7.680041	8.723251	8.956509	8.827852
AT3G05675	18640.87	-1.087991448	0.181962	-5.97924	2.24E-09	4.01E-08	13.6727	13.41728	13.46785	14.77599	14.89405	14.13731
AT4G22517	82.38763	-1.087034333	0.321298	-3.38326	7.16E-04	4.53E-03	6.286726	5.40337	5.077945	6.582321	6.92986	7.004552
AT5G58390	71.08466	-1.086620006	0.258549	-4.20276	2.64E-05	2.39E-04	5.403549	5.805191	5.204319	6.556599	6.779171	6.545826
AT3G45290	148.5864	-1.085933314	0.263163	-4.12647	3.68E-05	3.23E-04	6.98482	6.367346	6.172301	7.984706	7.283413	7.694153
AT3G05700	18651.34	-1.085695288	0.182128	-5.96117	2.50E-09	4.44E-08	13.67554	13.4185	13.47084	14.77617	14.89439	14.13681
AT4G38470	1426.951	-1.085686698	0.10132	-10.7154	8.61E-27	6.87E-25	9.875385	9.792344	9.822974	10.8132	11.04959	10.90468
AT5G45310	127.1093	-1.08335824	0.187064	-5.79139	6.98E-09	1.15E-07	6.317359	6.227787	6.492702	7.412182	7.432247	7.498771
AT2G36320	633.3782	-1.082757438	0.141677	-7.64244	2.13E-14	6.75E-13	8.616554	8.614479	8.736318	9.496539	9.951537	9.786007
AT1G12730	103.6758	-1.082547692	0.222215	-4.87162	1.11E-06	1.31E-05	6.30722	5.886999	5.930431	7.03764	7.09209	7.329843
AT1G23050	632.5447	-1.079415247	0.141168	-7.64633	2.07E-14	6.55E-13	8.757265	8.467103	8.730029	9.591425	9.926657	9.723435
AT1G23052	632.5447	-1.079415247	0.141168	-7.64633	2.07E-14	6.55E-13	8.757265	8.467103	8.730029	9.591425	9.926657	9.723435
AT2G31810	2588.442	-1.079115691	0.134299	-8.03518	9.34E-16	3.34E-14	10.91982	10.46515	10.6502	11.69617	11.87256	11.77761
AT5G27280	480.392	-1.079026954	0.155428	-6.94228	3.86E-12	9.81E-11	8.156191	8.194656	8.411572	9.119978	9.584342	9.334312
AT1G70850	99.83531	-1.077808501	0.327612	-3.28989	1.00E-03	0.0060541	6.145906	6.299253	5.204319	7.612711	6.92986	6.657142
AT5G57110	307.579	-1.077521901	0.179092	-6.01657	1.78E-09	3.24E-08	7.614069	7.77142	7.459545	8.443461	8.669986	8.981915
AT3G13750	19735.52	-1.076448323	0.137252	-7.84287	4.40E-15	1.49E-13	13.89245	13.52634	13.40146	14.63189	14.76327	14.74165
AT5G54510	1080.161	-1.076384594	0.112586	-9.56054	1.17E-21	6.63E-20	9.456279	9.417677	9.425111	10.44911	10.69495	10.40672
AT3G46440	188.0688	-1.075871172	0.172044	-6.25345	4.01E-10	7.87E-09	6.757957	7.03885	6.940512	7.853545	8.021154	8.13335
AT3G50500	1566.772	-1.07494303	0.09496	-11.3199	1.05E-29	9.77E-28	10.0535	9.863119	10.00243	11.04245	11.04959	11.07557

AT4G34138	579.5256	-1.073079238	0.152446	-7.03909	1.94E-12	5.07E-11	8.512288	8.707483	8.358194	9.41658	9.822611	9.61271
AT2G00600	68.24299	-1.070885665	0.266773	-4.01422	5.96E-05	0.0004999	5.459553	5.762479	5.051286	6.607592	6.460523	6.620982
AT5G63160	1752.078	-1.068003968	0.199147	-5.36288	8.19E-08	1.16E-06	10.57523	9.901353	9.756452	11.08468	11.26481	11.3101
AT3G12490	753.8559	-1.066964999	0.120885	-8.82626	1.08E-18	4.98E-17	8.953678	8.777083	9.023687	9.911077	10.11777	9.966147
AT1G11080	109.8323	-1.066144571	0.247677	-4.30458	1.67E-05	0.0001588	5.88467	6.545861	5.855366	7.110162	7.349087	7.261021
AT3G51330	95.66197	-1.063319613	0.281221	-3.78108	0.000156	0.0011763	6.396005	5.703489	5.508634	6.880651	7.327526	6.90281
AT3G49110	218.3669	-1.063056093	0.1755	-6.05731	1.38E-09	2.54E-08	7.277457	7.196901	6.918555	8.400731	8.047769	8.183799
AT2G32150	2216.726	-1.062109559	0.154262	-6.88512	5.77E-12	1.44E-10	10.46326	10.32786	10.61933	11.27019	11.82334	11.52285
AT3G11690	273.0131	-1.061293339	0.203058	-5.22654	1.73E-07	2.35E-06	7.766158	7.191545	7.32134	8.326574	8.800923	8.464489
AT3G25770	96.14111	-1.061088704	0.296793	-3.57519	0.00035	0.0024214	5.284563	6.174482	6.18462	6.680852	7.013254	7.384788
AT2G40300	381.077	-1.060182898	0.16199	-6.54473	5.96E-11	1.31E-09	7.890211	7.70154	8.185076	8.927704	9.128451	8.992568
AT1G73330	247.4355	-1.059735552	0.184315	-5.7496	8.95E-09	1.46E-07	7.439402	7.32473	7.156022	8.609021	8.468027	8.074403
AT1G21130	1972.269	-1.059668378	0.149139	-7.10526	1.20E-12	3.24E-11	10.57259	10.08083	10.23045	11.50684	11.41036	11.22909
AT3G13310	317.7435	-1.058557815	0.164266	-6.44418	1.16E-10	2.48E-09	7.848869	7.639949	7.519124	8.892154	8.831881	8.524443
AT5G03615	211.4036	-1.058331614	0.28042	-3.7741	1.61E-04	1.21E-03	7.555311	6.635377	6.811422	8.041442	8.61765	7.770414
AT1G27150	507.9139	-1.05609118	0.11536	-9.15474	5.45E-20	2.73E-18	8.409405	8.290468	8.395765	9.413001	9.358471	9.502747
AT1G28260	660.6063	-1.054395151	0.110249	-9.56374	1.14E-21	6.47E-20	8.806684	8.682881	8.734225	9.888059	9.807115	9.717026
AT1G16489	34.85328	-1.049146364	0.311928	-3.36343	7.70E-04	4.82E-03	4.578823	4.324114	4.614715	5.646896	5.625572	5.561185
AT5G51970	3311.798	-1.048803418	0.143524	-7.3075	2.72E-13	7.74E-12	11.12351	11.00391	11.06556	11.81076	12.35987	12.16744
AT1G27630	281.276	-1.04682995	0.147631	-7.09083	1.33E-12	3.58E-11	7.33847	7.596027	7.60412	8.525303	8.55409	8.641876
AT5G66053	151.8467	-1.046222178	0.272361	-3.84131	0.000122	0.0009475	7.003778	6.013812	6.61607	7.323456	7.980286	7.737017
AT2G43535	75.75357	-1.045777432	0.239148	-4.37294	1.23E-05	1.20E-04	5.800434	5.657607	5.364486	6.607592	6.841348	6.657142
AT1G10060	227.0774	-1.044807349	0.204304	-5.114	3.15E-07	4.09E-06	7.267032	6.952817	7.332454	7.95548	8.60874	8.183799
AT5G11090	531.4112	-1.04472145	0.162115	-6.44431	1.16E-10	2.48E-09	8.574902	8.191976	8.485623	9.273916	9.714632	9.457328
AT1G20900	43.83625	-1.044199926	0.291384	-3.58359	3.39E-04	0.0023505	4.738256	4.89808	4.910056	6.172293	5.625572	5.929675
AT5G27930	168.6253	-1.043968552	0.241545	-4.32205	1.55E-05	0.000148	6.858589	6.763641	6.687059	7.368501	7.707307	8.302704
AT3G01475	324.0094	-1.043554029	0.176779	-5.90316	3.57E-09	6.13E-08	7.666567	7.643877	7.812943	8.531918	9.109626	8.641876
AT5G22920	3359.92	-1.043493096	0.157555	-6.62306	3.52E-11	7.99E-10	11.15494	11.01343	11.08951	11.82699	12.46358	12.08936
AT1G08500	34.84145	-1.042571921	0.329867	-3.16058	1.57E-03	0.0089374	4.676581	4.284411	4.53998	5.19216	5.760417	5.806294
AT5G64110	112.0443	-1.042364731	0.27466	-3.79511	0.000148	0.0011169	6.609086	6.084872	5.692391	7.127736	7.040014	7.566881
AT3G49790	791.3508	-1.038990495	0.172799	-6.0127	1.82E-09	3.31E-08	9.336761	8.794854	8.811718	9.888059	10.15803	10.13431
AT5G01215	332.2356	-1.038591062	0.179044	-5.80074	6.60E-09	1.09E-07	7.444036	7.775006	7.988316	8.658224	8.991206	8.779462
AT5G01210	332.2356	-1.038591062	0.179044	-5.80074	6.60E-09	1.09E-07	7.444036	7.775006	7.988316	8.658224	8.991206	8.779462
AT5G49360	11686.16	-1.037144404	0.147238	-7.04401	1.87E-12	4.91E-11	13.12121	12.74046	12.78185	13.85977	14.17186	13.77358

AT1G75590	35.22552	-1.036920136	0.325812	-3.18257	0.00146	0.0083914	4.399556	4.607633	4.577832	5.99213	5.396255	5.399825
AT5G00365	451.6087	-1.034480729	0.166258	-6.22214	4.90E-10	9.52E-09	8.380684	7.96279	8.221402	9.047962	9.475822	9.212026
AT1G75785	67.34413	-1.033689831	0.328976	-3.14214	1.68E-03	9.44E-03	5.085378	5.733286	5.385981	5.741253	7.013254	6.620982
AT5G21170	1769.25	-1.032418453	0.125518	-8.22524	1.95E-16	7.39E-15	10.29582	9.952125	10.25256	11.22396	11.31498	11.11266
AT3G61160	151.9384	-1.031438461	0.24954	-4.13336	3.57E-05	3.15E-04	7.083171	6.185302	6.452518	7.600176	7.638931	7.834975
AT4G25500	759.6592	-1.030903469	0.113542	-9.07946	1.09E-19	5.42E-18	8.948795	8.902041	9.027111	9.854148	10.05356	10.07874
AT5G09130	944.2352	-1.029538912	0.135881	-7.57674	3.54E-14	1.09E-12	9.409653	9.148629	9.242741	10.08534	10.40704	10.42387
AT1G80910	354.6747	-1.028346198	0.16156	-6.36511	1.95E-10	4.02E-09	7.972712	7.749716	7.855939	8.658224	8.884509	9.12756
AT3G63160	1237.029	-1.023206462	0.16151	-6.33526	2.37E-10	4.83E-09	9.67805	9.526979	9.7616	10.42622	11.01797	10.60139
AT4G05150	2490.195	-1.021462147	0.131559	-7.76426	8.21E-15	2.70E-13	10.68159	10.45009	10.86299	11.58367	11.81755	11.71627
AT2G23080	446.9558	-1.021446303	0.13771	-7.41739	1.19E-13	3.54E-12	8.370981	8.100625	8.11922	9.163229	9.177483	9.348202
AT4G30270	1999.462	-1.018483418	0.239951	-4.24454	2.19E-05	2.02E-04	10.82262	9.916043	10.11049	11.60186	11.58747	10.91778
AT5G01740	269.2253	-1.017862263	0.235424	-4.32353	1.54E-05	1.47E-04	7.88681	7.175355	7.162264	8.525303	8.761267	8.202271
AT4G18930	261.7135	-1.015815016	0.164972	-6.15749	7.39E-10	1.41E-08	7.229941	7.363353	7.662533	8.464361	8.516472	8.407252
AT3G05640	242.8707	-1.015443381	0.210745	-4.81835	1.45E-06	1.68E-05	6.830546	7.473382	7.548016	8.318945	8.256129	8.489773
AT5G08000	69.85156	-1.012588161	0.240256	-4.21462	2.50E-05	2.28E-04	5.615596	5.365817	5.603436	6.391825	6.680573	6.639175
AT5G66052	638.7354	-1.011334692	0.219863	-4.59983	4.23E-06	4.51E-05	9.109124	8.173076	8.678698	9.591425	9.972527	9.664701
AT5G55700	502.8771	-1.010933463	0.146227	-6.91347	4.73E-12	1.19E-10	8.38551	8.292971	8.44011	9.124362	9.561435	9.480216
AT4G36040	3435.62	-1.009117737	0.142808	-7.06623	1.59E-12	4.22E-11	11.30833	10.83512	11.24867	12.2067	12.25527	12.03887
AT5G04375	222.1026	-1.004273615	0.169679	-5.91866	3.25E-09	5.63E-08	7.083171	7.131273	7.359871	8.139994	8.428064	8.087712
AT1G20160	156.6433	-1.003444105	0.202675	-4.95101	7.38E-07	9.03E-06	6.506476	6.97169	6.560439	7.884889	7.690514	7.585765
AT2G35170	60.51898	-1.003221947	0.313923	-3.19576	1.39E-03	8.08E-03	5.441126	5.117315	5.320509	5.694846	6.900955	6.316346
AT2G30600	2321.579	-1.001452822	0.106666	-9.38869	6.07E-21	3.29E-19	10.53344	10.6227	10.60909	11.5227	11.76639	11.49627
AT4G11600	499.7129	-1.000912411	0.152911	-6.5457	5.92E-11	1.31E-09	8.430577	8.307899	8.369029	9.079912	9.58888	9.452192
AT5G58375	274.0426	-1.000762598	0.149683	-6.68588	2.30E-11	5.32E-10	7.646601	7.34899	7.509364	8.525303	8.599774	8.443937
AT2G28120	396.701	-0.998867159	0.166928	-5.98384	2.18E-09	3.91E-08	8.18693	7.844911	8.053574	9.175955	9.189485	8.771237
AT1G20693	1890.282	-0.998138897	0.148522	-6.7205	1.81E-11	4.25E-10	10.08203	10.3382	10.43054	11.02497	11.41548	11.43771
AT3G03330	455.8684	-0.990927161	0.136	-7.28621	3.19E-13	8.98E-12	8.351379	8.10633	8.271346	9.335713	9.315308	9.094978
AT2G22660	1163.522	-0.990430286	0.124941	-7.92716	2.24E-15	7.78E-14	9.535501	9.658978	9.606581	10.40475	10.58794	10.78315
AT1G80190	191.5936	-0.989707924	0.180712	-5.4767	4.33E-08	6.41E-07	7.219167	6.840944	6.903729	7.842944	8.099571	8.067702
AT1G20696	1230.303	-0.989139003	0.127438	-7.76173	8.38E-15	2.75E-13	9.544161	9.761949	9.72517	10.47337	10.81795	10.73109
AT5G18540	68.39063	-0.988119491	0.240029	-4.11667	3.84E-05	0.0003361	5.664073	5.43997	5.428035	6.420628	6.680573	6.506726
AT2G30860	2513.255	-0.987343248	0.120885	-8.16759	3.15E-16	1.17E-14	10.64382	10.86697	10.61536	11.85628	11.55885	11.69903
AT1G07390	52.2676	-0.986790568	0.279382	-3.53204	4.12E-04	2.80E-03	5.061448	5.287644	5.024124	6.448866	6.050936	5.929675

AT1G09570	7589.903	-0.98499778	0.11642	-8.4607	2.66E-17	1.08E-15	12.2988	12.29272	12.33433	13.06549	13.38213	13.43521
AT2G36390	546.3935	-0.984650888	0.156355	-6.29753	3.02E-10	6.08E-09	8.402278	8.519345	8.607287	9.201076	9.584342	9.701959
AT3G26580	989.3469	-0.983309925	0.120133	-8.18515	2.72E-16	1.02E-14	9.256042	9.504476	9.340939	10.22252	10.46002	10.4014
AT3G01310	1234.364	-0.982727963	0.113611	-8.64992	5.15E-18	2.23E-16	9.526789	9.755611	9.783025	10.64643	10.62597	10.76979
AT3G58750	724.8077	-0.981436179	0.116918	-8.3942	4.69E-17	1.87E-15	8.956925	8.803658	9.002968	9.816714	10.02705	9.89601
AT2G02710	1760.064	-0.980865625	0.120057	-8.17002	3.08E-16	1.15E-14	10.41155	10.09524	10.07949	11.20339	11.16502	11.21324
AT5G02160	7021.239	-0.979371235	0.165443	-5.9197	3.23E-09	5.60E-08	12.15675	12.01099	12.38659	12.8597	13.47116	13.18248
AT2G25930	434.5188	-0.978904924	0.133458	-7.3349	2.22E-13	6.37E-12	8.285767	8.112011	8.17585	9.277857	8.991206	9.242095
AT5G38200	118.5484	-0.978607883	0.198622	-4.92699	8.35E-07	1.01E-05	6.122968	6.423257	6.379384	7.179211	7.432247	7.329843
AT1G22650	96.7422	-0.977833387	0.247327	-3.95361	7.70E-05	0.0006304	6.276368	5.594069	6.082995	7.110162	7.142352	6.825478
AT1G21770	680.4737	-0.97752002	0.155369	-6.29161	3.14E-10	6.29E-09	9.058643	8.559403	8.831438	9.918669	9.868124	9.680159
AT3G23020	69.12287	-0.974552781	0.247759	-3.93348	8.37E-05	6.79E-04	5.459553	5.657607	5.488902	6.238498	6.747048	6.620982
AT1G23060	82.40337	-0.974464374	0.254136	-3.83442	0.000126	0.0009706	5.530997	5.900194	5.930431	7.110162	6.420349	6.777007
AT2G07175	102.0017	-0.973952275	0.222907	-4.36932	1.25E-05	0.0001217	6.027383	5.860242	6.368627	6.980752	7.190921	7.125632
AT5G57900	247.4924	-0.972734251	0.155493	-6.2558	3.95E-10	7.76E-09	7.411277	7.353794	7.376074	8.564547	8.301072	8.214456
AT2G18050	2542.428	-0.972598362	0.138724	-7.01105	2.37E-12	6.16E-11	10.62761	10.74247	10.83689	11.86088	11.42312	11.84186
AT5G15230	1747.885	-0.971273354	0.133846	-7.25662	3.97E-13	1.11E-11	10.03365	10.16318	10.37519	11.00846	11.34211	11.17634
AT2G38820	442.5939	-0.966853905	0.145811	-6.63089	3.34E-11	7.59E-10	8.425899	8.112011	8.096582	9.088912	9.30982	9.19982
AT2G40070	240.3933	-0.965448486	0.151727	-6.36305	1.98E-10	4.07E-09	7.416003	7.290065	7.31014	8.386201	8.417898	8.158795
AT1G11820	463.3942	-0.964858864	0.145633	-6.62529	3.47E-11	7.87E-10	8.516694	8.142867	8.172762	9.281787	9.30982	9.208984
AT3G17790	233.8628	-0.963998276	0.191392	-5.03678	4.73E-07	5.97E-06	7.251253	7.554903	7.06567	8.400731	8.007659	8.407252
AT4G34180	643.5493	-0.963548748	0.136162	-7.07648	1.48E-12	3.94E-11	8.971444	8.622462	8.674337	9.827509	9.663919	9.719165
AT5G15150	75.1084	-0.963179999	0.236917	-4.06548	4.79E-05	4.11E-04	5.814819	5.475665	5.726492	6.750572	6.460523	6.743763
AT1G62810	227.6224	-0.96116793	0.162008	-5.93286	2.98E-09	5.20E-08	7.387414	7.228629	7.193077	8.415115	8.047769	8.238522
AT1G19370	158.6538	-0.960701271	0.169028	-5.68369	1.32E-08	2.09E-07	6.78012	6.666599	6.787392	7.709256	7.835043	7.63192
AT3G63210	242.7326	-0.959753961	0.171978	-5.58067	2.40E-08	3.67E-07	7.49852	7.125667	7.439125	8.491765	8.161812	8.330959
AT1G52000	584.3946	-0.958387146	0.265967	-3.60341	3.14E-04	2.20E-03	8.560035	8.863826	8.333514	10.21842	9.03842	9.297557
AT4G13530	756.5782	-0.956770981	0.124324	-7.69581	1.41E-14	4.52E-13	8.938978	9.041059	9.018535	9.789368	10.14576	9.95348
AT1G75780	124.9412	-0.954352022	0.279046	-3.42005	0.000626	0.0040433	5.977105	6.603464	6.502575	6.727704	7.673524	7.604405
AT1G18720	416.8494	-0.954050793	0.143654	-6.64132	3.11E-11	7.13E-10	8.156191	7.965931	8.299936	9.010559	9.077695	9.230143
AT5G19860	653.7603	-0.953879242	0.141411	-6.74545	1.53E-11	3.60E-10	8.764694	8.66753	8.929848	9.52666	9.897687	9.826263
AT5G43430	257.4347	-0.953109896	0.155071	-6.14626	7.93E-10	1.50E-08	7.453261	7.305023	7.581008	8.264377	8.506912	8.469581
AT2G01090	335.1594	-0.95032767	0.200506	-4.73964	2.14E-06	2.42E-05	7.876561	7.723975	7.84434	8.734765	9.183496	8.380466
AT1G74550	41.08935	-0.949729078	0.299838	-3.16747	1.54E-03	8.76E-03	4.88181	4.788814	4.818167	5.694846	5.553109	6.096967

AT2G38370	133.2501	-0.947164611	0.201629	-4.69757	2.63E-06	2.94E-05	6.742991	6.468249	6.268024	7.561902	7.370331	7.478706
AT4G21570	450.0549	-0.946422893	0.158562	-5.96879	2.39E-09	4.25E-08	8.225128	8.305422	8.248058	8.922678	9.21906	9.462445
AT5G49440	1323.922	-0.945992333	0.16587	-5.70323	1.18E-08	1.88E-07	10.01509	9.469496	9.888448	10.85072	10.90423	10.54581
AT3G01470	1069.89	-0.945491261	0.160883	-5.87688	4.18E-09	7.12E-08	9.436647	9.29941	9.737764	10.25687	10.70125	10.40937
AT1G54410	3844.184	-0.945341036	0.171956	-5.49758	3.85E-08	5.73E-07	11.17599	11.51938	11.32195	11.95187	12.62639	12.28114
AT4G01610	1041.853	-0.944613861	0.106091	-8.90381	5.40E-19	2.55E-17	9.458571	9.583283	9.37493	10.44386	10.34669	10.47921
AT5G40470	61.05327	-0.944561816	0.29839	-3.16553	1.55E-03	0.00881	5.648094	5.510498	4.818167	6.066916	6.537663	6.466537
AT2G47600	372.4599	-0.944289762	0.177688	-5.31431	1.07E-07	1.50E-06	7.963058	8.018304	7.970641	8.531918	9.071223	9.162571
AT1G33170	346.8659	-0.943961743	0.182947	-5.15975	2.47E-07	3.27E-06	8.047708	7.838071	7.752166	8.564547	8.753203	9.149938
AT5G11520	709.9417	-0.94395701	0.160729	-5.87298	4.28E-09	7.27E-08	8.880344	8.940868	8.916985	9.503287	10.11777	9.940702
AT1G11260	12559.33	-0.941928629	0.170444	-5.52632	3.27E-08	4.93E-07	13.30528	12.74584	13.06464	13.81742	14.28978	13.90485
AT1G06265	55.58119	-0.941486233	0.25679	-3.66637	0.000246	0.0017773	5.325325	5.226103	5.228318	6.102904	6.200761	6.360876
AT1G01300	175.4902	-0.94098783	0.16556	-5.68367	1.32E-08	2.09E-07	6.844636	6.840944	7.025145	7.777648	7.966403	7.842845
AT5G16380	72.01922	-0.937610914	0.23143	-4.05138	5.09E-05	4.34E-04	5.58235	5.641983	5.675034	6.750572	6.460523	6.545826
AT5G62720	522.601	-0.937524759	0.17744	-5.28363	1.27E-07	1.76E-06	8.467465	8.229049	8.689544	9.119978	9.642251	9.490273
AT2G28200	238.1084	-0.937216041	0.17191	-5.45178	4.99E-08	7.32E-07	7.49852	7.212852	7.298852	8.09603	8.525969	8.256311
AT3G60690	135.6246	-0.936948059	0.183946	-5.0936	3.51E-07	4.52E-06	6.470584	6.53744	6.61607	7.661791	7.370331	7.427286
AT2G42890	1002.663	-0.935798843	0.094258	-9.92805	3.14E-23	2.04E-21	9.462003	9.41653	9.394948	10.29625	10.40961	10.3907
AT4G26080	855.1852	-0.935745189	0.151277	-6.18564	6.19E-10	1.19E-08	8.995323	9.379322	9.171808	9.92873	10.13649	10.32619
AT4G35790	1163.391	-0.934900854	0.094779	-9.86401	5.96E-23	3.75E-21	9.704365	9.651195	9.561243	10.62648	10.54888	10.56378
AT3G53460	2835.861	-0.934502175	0.125042	-7.47352	7.81E-14	2.35E-12	11.10241	10.72311	10.91289	11.88045	11.96098	11.7512
AT1G21110	103.7141	-0.932797589	0.229561	-4.0634	4.84E-05	4.14E-04	6.386406	5.846675	6.159877	7.26112	7.142352	6.917792
AT1G08980	1480.943	-0.93207995	0.126503	-7.36806	1.73E-13	5.03E-12	10.12884	9.769158	10.03511	10.87694	11.04134	10.86272
AT1G75800	1930.814	-0.931687049	0.132334	-7.04043	1.92E-12	5.03E-11	10.45583	10.11519	10.50365	11.41321	11.31361	11.20028
AT2G40960	169.9981	-0.928747696	0.269083	-3.45153	0.000557	0.0036643	6.80915	6.827194	6.911161	8.415115	7.432247	7.373965
AT4G21980	392.7707	-0.928632799	0.156878	-5.91947	3.23E-09	5.61E-08	8.217028	7.905054	8.076891	8.779925	9.183496	9.061642
AT1G12010	120.4412	-0.927112464	0.246728	-3.75762	1.72E-04	1.28E-03	6.609086	5.832979	6.522122	7.228912	7.491614	7.249225
AT1G71030	1605.056	-0.926903676	0.145708	-6.3614	2.00E-10	4.11E-09	10.19632	9.897246	10.19283	10.89373	11.28593	10.9215
AT3G16380	88.05758	-0.926701346	0.230173	-4.02611	5.67E-05	4.77E-04	5.977105	6.013812	5.743245	6.582321	7.09209	6.917792
AT1G21100	208.3591	-0.926664957	0.229067	-4.04538	5.22E-05	0.0004424	7.377757	6.927264	7.11799	8.531918	7.850244	7.834975
AT3G63010	69.10715	-0.926408007	0.245707	-3.77038	0.000163	0.0012218	5.710973	5.457928	5.584968	6.503738	6.292644	6.743763
AT5G26865	52.84967	-0.926401195	0.282385	-3.28063	1.04E-03	6.24E-03	5.477749	5.071394	4.996442	6.238498	5.883728	6.293554
AT4G16000	135.4882	-0.924243028	0.179591	-5.14636	2.66E-07	3.50E-06	6.609086	6.468249	6.569861	7.383209	7.491614	7.576354
AT3G61460	1098.801	-0.922927464	0.158731	-5.81442	6.08E-09	1.01E-07	9.749994	9.206868	9.659391	10.3939	10.62818	10.46142

AT4G24220	1973.557	-0.920423224	0.106944	-8.60658	7.53E-18	3.23E-16	10.41214	10.32542	10.48696	11.18881	11.44832	11.36511
AT1G20840	508.8093	-0.918553174	0.112567	-8.16004	3.35E-16	1.24E-14	8.446832	8.519345	8.411572	9.350757	9.347801	9.447037
AT1G08570	938.1818	-0.918261217	0.146996	-6.24685	4.19E-10	8.19E-09	9.493642	9.259011	9.238316	9.987665	10.38373	10.39472
AT3G07280	278.2325	-0.918204301	0.198104	-4.63497	3.57E-06	3.88E-05	7.542402	7.806883	7.348966	8.148628	8.644055	8.708006
AT2G39725	51.79959	-0.917991009	0.271695	-3.37876	7.28E-04	4.60E-03	5.108917	5.307588	5.051286	5.913254	6.336481	6.096967
AT5G39570	2092.233	-0.917527526	0.193303	-4.74657	2.07E-06	2.35E-05	10.2575	10.80956	10.31833	11.062	11.71022	11.43836
AT3G54260	81.94353	-0.917496114	0.246401	-3.72359	0.000196	0.0014478	5.599069	6.013812	5.82421	7.056115	6.646151	6.564985
AT4G37870	3250.135	-0.915733693	0.102894	-8.89979	5.60E-19	2.64E-17	11.18778	11.10602	11.10458	11.90232	12.06806	12.18186
AT1G62510	478.4582	-0.91361063	0.238265	-3.83443	0.000126	0.0009706	8.321467	8.159423	8.536968	8.962399	9.845547	8.908815
AT5G65110	958.8736	-0.913354039	0.157987	-5.78119	7.42E-09	1.22E-07	9.373668	9.363928	9.362786	9.93623	10.56279	10.329
AT1G09027	364.8687	-0.909927953	0.195158	-4.66251	3.12E-06	3.43E-05	8.24651	7.86187	7.760419	9.158962	8.920958	8.591379
AT4G32480	994.7991	-0.909498634	0.16793	-5.41596	6.10E-08	8.84E-07	9.66719	9.296918	9.260306	10.30013	10.60597	10.10352
AT4G05065	85.01486	-0.909194772	0.243883	-3.728	0.000193	0.0014252	6.039683	5.577738	5.959397	6.632428	7.09209	6.709736
AT5G41700	2070.084	-0.908842765	0.196708	-4.62026	3.83E-06	4.13E-05	10.8244	9.97413	10.49934	11.506	11.49862	11.20334
AT4G34139	230.0408	-0.90795747	0.207405	-4.3777	1.20E-05	0.0001174	7.277457	7.516861	7.078929	8.078063	8.599774	7.991862
AT2G40420	472.0688	-0.907561213	0.160992	-5.63731	1.73E-08	2.69E-07	8.512288	8.148407	8.366328	9.020001	9.456087	9.317464
AT3G15500	226.2323	-0.907325464	0.26714	-3.39645	6.83E-04	0.0043539	7.678415	7.329615	6.61607	8.55808	8.086794	7.970467
AT1G11530	421.3032	-0.906458942	0.152382	-5.94858	2.70E-09	4.75E-08	8.275402	8.117671	8.163456	8.839799	9.320774	9.133989
AT2G29660	60.16937	-0.906034093	0.249619	-3.62966	0.000284	0.0020099	5.530997	5.32726	5.298008	6.270495	6.420349	6.293554
AT2G01170	153.369	-0.905252933	0.212602	-4.25798	2.06E-05	0.0001918	6.609086	6.50326	7.018279	7.788738	7.756543	7.416778
AT3G06500	311.7535	-0.905111947	0.196543	-4.60516	4.12E-06	4.41E-05	7.896987	7.820827	7.504459	9.015287	8.535404	8.417829
AT4G00355	893.9744	-0.902222167	0.130843	-6.89545	5.37E-12	1.35E-10	9.322989	9.144477	9.357355	9.975584	10.31417	10.2616
AT5G58800	312.6711	-0.901502076	0.150492	-5.99036	2.09E-09	3.77E-08	7.697949	7.608139	7.96351	8.652165	8.736939	8.646381
AT2G47890	118.3714	-0.899766067	0.225775	-3.98524	6.74E-05	5.59E-04	6.255428	6.414087	6.421627	7.661791	7.013254	7.09959
AT5G42200	89.84259	-0.898544564	0.28089	-3.19892	1.38E-03	8.01E-03	6.15724	5.493187	6.121948	7.26112	6.841348	6.506726
AT5G38860	168.7717	-0.89775556	0.171548	-5.23327	1.67E-07	2.26E-06	6.90639	6.7122	7.004448	7.732415	7.865285	7.778644
AT5G06860	277.9821	-0.897748323	0.145631	-6.16454	7.07E-10	1.35E-08	7.751277	7.468962	7.562248	8.498535	8.525969	8.499764
AT4G33540	314.8092	-0.897354377	0.153039	-5.86358	4.53E-09	7.68E-08	7.551021	7.858494	7.893946	8.751866	8.72049	8.5867
AT1G75820	1273.748	-0.896401707	0.119104	-7.5262	5.22E-14	1.59E-12	9.892441	9.581237	9.886564	10.73366	10.68862	10.67267
AT1G56700	392.8669	-0.894386999	0.137015	-6.52765	6.68E-11	1.47E-09	8.195201	7.993895	8.099838	8.834457	9.103296	9.061642
AT5G59220	86.31464	-0.894223473	0.256298	-3.489	4.85E-04	3.23E-03	5.664073	6.299253	5.675034	6.750572	6.810594	6.947296
AT5G57887	168.8141	-0.893806579	0.245867	-3.63533	0.000278	0.0019712	7.186351	6.511881	6.827223	7.323456	7.994038	7.984766
AT1G12080	358.5259	-0.893176089	0.261774	-3.41201	6.45E-04	4.15E-03	8.056815	7.956488	7.80099	8.078063	9.379578	8.916294
AT3G27210	68.4558	-0.891611603	0.264865	-3.36629	7.62E-04	4.78E-03	5.513465	5.641983	5.639679	6.476563	6.050936	6.85691

AT5G61440	151.2936	-0.89085766	0.226033	-3.94127	8.11E-05	0.0006597	6.872409	6.704699	6.522122	7.397768	8.047769	7.384788
AT5G23575	376.179	-0.890389734	0.124841	-7.13216	9.88E-13	2.69E-11	8.068868	8.018304	8.036685	8.823715	8.984333	9.003142
AT4G28270	526.1823	-0.89015477	0.16356	-5.44238	5.26E-08	7.69E-07	8.42824	8.428918	8.676519	9.154682	9.693718	9.381005
AT2G27150	250.9807	-0.889134611	0.149737	-5.93799	2.89E-09	5.06E-08	7.444036	7.468962	7.449371	8.174225	8.516472	8.36961
AT1G23880	65.93093	-0.889105822	0.256061	-3.47224	5.16E-04	3.42E-03	5.441126	5.791094	5.320509	6.301797	6.499609	6.545826
AT1G09490	302.594	-0.888867549	0.159844	-5.56084	2.68E-08	4.09E-07	7.502969	7.810381	7.832646	8.464361	8.769286	8.637358
AT2G29310	256.8626	-0.887455638	0.234127	-3.79048	1.50E-04	1.14E-03	7.065234	7.583812	7.701631	7.915566	8.669986	8.509687
AT5G03235	71.80481	-0.886796822	0.263955	-3.35965	0.00078	0.0048759	5.91169	5.40337	5.56626	6.727704	6.714193	6.246861
AT3G61490	71.40247	-0.885132608	0.235596	-3.757	1.72E-04	1.28E-03	5.814819	5.626188	5.508634	6.632428	6.460523	6.583893
AT1G31812	4655.741	-0.884791668	0.177413	-4.98719	6.13E-07	7.60E-06	11.67673	11.50217	11.79265	12.18168	12.92087	12.50628
AT5G03150	70.42788	-0.884764857	0.260225	-3.4	0.000674	0.0043053	5.814819	5.365817	5.675034	6.838581	6.336481	6.404074
AT5G58650	119.3605	-0.88464867	0.246032	-3.59566	0.000324	0.0022581	6.524093	6.084872	6.462669	6.941541	7.707307	7.151212
AT3G26510	1363.7	-0.884596113	0.142163	-6.22241	4.90E-10	9.52E-09	10.03673	9.723495	9.913643	10.53724	10.91331	10.90186
AT4G01670	81.16262	-0.884119374	0.245586	-3.60003	3.18E-04	2.23E-03	5.726274	6.096383	5.603436	6.859769	6.841348	6.526408
AT3G14020	80.22124	-0.884014803	0.242594	-3.64401	2.68E-04	1.91E-03	5.925012	5.762479	5.792368	6.980752	6.292644	6.809501
AT5G11150	115.767	-0.882802142	0.215892	-4.0891	4.33E-05	3.75E-04	6.286726	6.25885	6.492702	6.941541	7.26084	7.488773
AT1G62515	311.9648	-0.882599638	0.25496	-3.46172	5.37E-04	0.0035437	7.709544	7.516861	7.981272	8.264377	9.242291	8.319723
AT4G04620	220.3367	-0.88199864	0.170556	-5.17132	2.32E-07	3.08E-06	7.175244	7.244235	7.386775	7.905412	8.323029	8.250405
AT3G56140	751.4535	-0.881851075	0.117125	-7.52914	5.11E-14	1.56E-12	8.974651	9.033594	9.108564	9.905994	10.07959	9.804262
AT2G01450	1482.997	-0.881098961	0.139614	-6.31096	2.77E-10	5.61E-09	10.22891	9.750156	10.0419	10.96023	10.95609	10.82149
AT3G26220	527.4624	-0.880428807	0.197303	-4.46233	8.11E-06	8.18E-05	8.8666	8.428918	8.194244	9.680454	9.259472	9.306122
AT1G09430	1682.27	-0.877093892	0.110638	-7.92757	2.23E-15	7.77E-14	10.31555	10.07938	10.21852	10.96635	11.17712	11.12643
AT1G01650	335.5979	-0.876005854	0.137486	-6.37159	1.87E-10	3.87E-09	7.788196	7.987727	7.882648	8.866214	8.635307	8.795773
AT2G19340	123.4928	-0.874340137	0.214971	-4.06726	4.76E-05	4.08E-04	6.386406	6.163581	6.721284	7.228912	7.432247	7.352073
AT2G40000	4016.442	-0.873320603	0.146023	-5.98069	2.22E-09	3.98E-08	11.67992	11.49023	11.16761	12.39763	12.44431	12.19152
AT4G24690	2209.171	-0.871704623	0.090279	-9.65573	4.65E-22	2.73E-20	10.60503	10.60317	10.61422	11.44334	11.40651	11.58927
AT2G22122	72.84555	-0.871325348	0.247066	-3.52669	0.000421	0.0028514	5.477749	5.832979	5.726492	6.301797	6.714193	6.709736
AT5G06865	285.7828	-0.870917888	0.145927	-5.96818	2.40E-09	4.26E-08	7.820635	7.529654	7.60412	8.545058	8.535404	8.534198
AT5G13730	387.5459	-0.870910696	0.152062	-5.72734	1.02E-08	1.64E-07	7.913791	8.1649	8.17585	8.807449	9.177483	8.92744
AT2G15695	514.7468	-0.870075579	0.111193	-7.8249	5.08E-15	1.72E-13	8.467465	8.489101	8.563151	9.32051	9.41067	9.415719
AT5G13870	87.31535	-0.869770774	0.227579	-3.82184	1.32E-04	1.02E-03	5.964257	5.733286	6.147344	6.859769	6.680573	6.961824
AT1G22070	175.3523	-0.867776245	0.212568	-4.08235	4.46E-05	3.84E-04	6.865515	6.834086	7.156022	7.853545	7.432247	8.13335
AT4G27790	134.2683	-0.865451997	0.181658	-4.76417	1.90E-06	2.16E-05	6.68935	6.520451	6.502575	7.548916	7.411902	7.395531
AT3G22121	478.1868	-0.865026786	0.182417	-4.74203	2.12E-06	2.40E-05	8.472011	8.257524	8.437538	9.513351	9.395208	8.855347

AT5G63620	485.1391	-0.863901177	0.126795	-6.81338	9.53E-12	2.32E-10	8.472011	8.344559	8.447796	9.13309	9.415787	9.328718
AT3G22120	478.3729	-0.863432908	0.182563	-4.7295	2.25E-06	2.54E-05	8.476542	8.257524	8.437538	9.513351	9.395208	8.855347
AT1G16110	105.4843	-0.860083259	0.240833	-3.57129	3.55E-04	2.45E-03	5.829063	6.269058	6.48276	7.26112	7.166841	6.887672
AT5G64860	487.7936	-0.859191221	0.181756	-4.72718	2.28E-06	2.56E-05	8.071865	8.548969	8.611855	9.124362	9.242291	9.520031
AT1G67070	56.86042	-0.858581292	0.257764	-3.33089	8.66E-04	5.33E-03	5.325325	5.40337	5.298008	5.99213	6.336481	6.338783
AT5G57910	118.482	-0.858012109	0.251803	-3.40747	6.56E-04	4.21E-03	6.424426	6.520451	6.18462	7.709256	7.142352	6.85691
AT5G02540	1338.295	-0.857236481	0.169145	-5.06806	4.02E-07	5.13E-06	9.75746	9.746507	10.11452	10.49385	11.04959	10.68038
AT1G11950	77.99746	-0.854539449	0.244881	-3.48962	4.84E-04	3.23E-03	5.98984	5.510498	5.870694	6.607592	6.537663	6.85691
AT1G19660	2356.081	-0.853734814	0.099569	-8.57434	9.97E-18	4.22E-16	10.66581	10.74841	10.71214	11.43189	11.68713	11.5793
AT5G47610	141.4112	-0.853412398	0.247784	-3.44418	0.000573	0.0037432	6.997486	6.073269	6.712804	7.482168	7.529878	7.576354
AT1G54740	1740.802	-0.850716453	0.143444	-5.93066	3.02E-09	5.26E-08	10.46725	10.12926	10.18978	11.12571	11.32725	10.92429
AT2G18170	301.5111	-0.850142573	0.135813	-6.25967	3.86E-10	7.60E-09	7.820635	7.65948	7.752166	8.545058	8.703851	8.57257
AT1G74520	297.5231	-0.850100427	0.144908	-5.86649	4.45E-09	7.55E-08	7.791837	7.663355	7.731324	8.407941	8.626506	8.712309
AT4G25970	902.8567	-0.849597934	0.117739	-7.21597	5.36E-13	1.48E-11	9.465426	9.20288	9.308943	10.10987	10.15803	10.28201
AT1G54290	95.57247	-0.849436867	0.217566	-3.90427	9.45E-05	0.0007542	6.145906	5.976925	6.172301	6.773083	6.900955	7.176347
AT1G71695	1082.629	-0.848177396	0.150983	-5.6177	1.94E-08	3.00E-07	9.695647	9.414232	9.630416	10.30594	10.71999	10.28201
AT1G50480	2858.458	-0.848104283	0.097567	-8.69254	3.54E-18	1.56E-16	11.06904	10.88989	11.01429	11.937	11.75634	11.83842
AT1G03290	495.6709	-0.84650459	0.148181	-5.71263	1.11E-08	1.78E-07	8.476542	8.523615	8.398412	9.106746	9.259472	9.558776
AT2G16365	275.0794	-0.845353367	0.140265	-6.02682	1.67E-09	3.04E-08	7.666567	7.639949	7.548016	8.349224	8.525969	8.53905
AT3G10120	118.198	-0.845194646	0.251914	-3.35509	7.93E-04	4.95E-03	6.600809	6.404859	6.10908	6.750572	7.585434	7.363061
AT3G02550	119.6401	-0.844986728	0.217205	-3.89028	0.0001	0.0007929	6.58411	6.289258	6.335866	7.482168	7.370331	6.961824
AT1G10140	279.9053	-0.843998996	0.137809	-6.1244	9.10E-10	1.70E-08	7.630427	7.742408	7.557519	8.457428	8.506912	8.529329
AT2G17880	545.2796	-0.840217403	0.231774	-3.62516	2.89E-04	2.04E-03	8.557899	8.221185	8.905867	9.128733	9.908619	9.23314
AT4G02610	242.1425	-0.839258552	0.141794	-5.91885	3.24E-09	5.63E-08	7.457851	7.410223	7.464605	8.318945	8.185975	8.347651
AT2G02760	1122.034	-0.839089224	0.123161	-6.81293	9.56E-12	2.32E-10	9.669171	9.458388	9.795127	10.40835	10.58113	10.49303
AT5G02150	446.8339	-0.838720383	0.151332	-5.54224	2.99E-08	4.52E-07	8.501212	8.060052	8.358194	9.175955	9.242291	9.091679
AT1G55110	188.5546	-0.837725548	0.18373	-4.55955	5.13E-06	5.37E-05	7.292953	7.008709	6.918555	8.041442	7.707307	8.012944
AT5G21020	2504.86	-0.837426831	0.168854	-4.95949	7.07E-07	8.69E-06	11.12315	10.41777	10.77815	11.63142	11.58974	11.74228
AT4G31650	123.1937	-0.837407873	0.243723	-3.4359	0.000591	0.0038429	6.168486	6.689579	6.462669	6.859769	7.529878	7.498771
AT2G02390	531.6456	-0.834196044	0.108178	-7.71136	1.24E-14	4.03E-13	8.545013	8.596353	8.591183	9.47267	9.37433	9.399801
AT1G21410	241.0115	-0.833428434	0.171617	-4.85634	1.20E-06	1.41E-05	7.60582	7.264784	7.428806	8.240343	8.099571	8.469581
AT1G10200	787.1613	-0.833383987	0.120557	-6.91275	4.75E-12	1.20E-10	9.097404	9.055873	9.267562	9.872507	9.955057	10.10679
AT5G64430	601.5142	-0.832652918	0.132524	-6.28305	3.32E-10	6.60E-09	8.64903	8.669457	8.931676	9.680454	9.465988	9.626455
AT1G33050	2196.911	-0.832482553	0.122565	-6.79219	1.10E-11	2.66E-10	10.78659	10.49427	10.56677	11.60813	11.33942	11.42466

AT5G63860	958.9135	-0.83204194	0.099531	-8.35966	6.29E-17	2.48E-15	9.372452	9.435913	9.472349	10.33649	10.27247	10.18473
AT1G14900	123.3213	-0.831670422	0.205564	-4.04579	5.21E-05	0.0004418	6.424426	6.494586	6.462669	7.03764	7.621318	7.261021
AT3G16450	239.4446	-0.831223859	0.213722	-3.88927	1.01E-04	7.95E-04	7.529377	7.525402	7.149752	8.478128	8.448184	7.819103
AT4G22490	332.4527	-0.831005306	0.242202	-3.43104	6.01E-04	0.0039029	8.404658	7.647793	7.407944	8.834457	8.590752	8.811901
AT5G52450	129.2159	-0.830333095	0.205751	-4.03562	5.45E-05	4.60E-04	6.757957	6.414087	6.411182	7.58753	7.305638	7.249225
AT3G23150	83.2721	-0.829717255	0.245633	-3.37788	0.00073	0.0046085	5.938213	6.03789	5.709543	6.362435	6.958197	6.90281
AT1G56220	8851.799	-0.829694674	0.141722	-5.8544	4.79E-09	8.09E-08	12.76147	12.40604	12.6988	13.29233	13.69261	13.40213
AT3G23490	1062.84	-0.829266051	0.117353	-7.06641	1.59E-12	4.22E-11	9.647232	9.461729	9.608868	10.304	10.57199	10.354
AT4G27410	283.003	-0.828237741	0.176927	-4.68124	2.85E-06	3.17E-05	7.751277	7.78926	7.418413	8.264377	8.72049	8.519541
AT1G09530	289.2081	-0.827585064	0.137928	-6.00011	1.97E-09	3.57E-08	7.769855	7.600076	7.72712	8.471261	8.626506	8.519541
AT1G53320	607.466	-0.825856888	0.116537	-7.08663	1.37E-12	3.68E-11	8.757265	8.709359	8.841197	9.54966	9.739336	9.524931
AT3G04965	67.67228	-0.823890687	0.235052	-3.50515	4.56E-04	3.07E-03	5.664073	5.54451	5.657465	6.530411	6.336481	6.506726
AT3G20810	63.34626	-0.823806171	0.261209	-3.15382	0.001611	0.0091087	5.548318	5.226103	5.726492	6.205775	6.537663	6.360876
AT1G66890	171.9358	-0.822980556	0.197343	-4.1703	3.04E-05	0.0002725	7.016281	6.854565	6.947758	7.732415	8.112236	7.488773
AT5G48412	489.8553	-0.822439004	0.127895	-6.43057	1.27E-10	2.70E-09	8.498986	8.325123	8.567861	9.233904	9.248041	9.394455
AT3G18830	463.3977	-0.821414875	0.164133	-5.00458	5.60E-07	6.98E-06	8.338991	8.307899	8.490592	9.510004	9.103296	8.985475
AT2G27200	175.4275	-0.821059787	0.162963	-5.03832	4.70E-07	5.93E-06	6.892893	7.03885	7.031979	7.915566	7.740317	7.794964
AT4G19860	1548.659	-0.820001428	0.150847	-5.43597	5.45E-08	7.95E-07	10.11292	10.0232	10.22301	10.61406	11.05288	11.14406
AT5G22580	1000.083	-0.819880435	0.167048	-4.90805	9.20E-07	1.11E-05	9.543081	9.248732	9.643862	10.04197	10.59021	10.29498
AT5G18170	2051.507	-0.81969979	0.13377	-6.12769	8.92E-10	1.67E-08	10.73031	10.34425	10.4882	11.50264	11.22309	11.33752
AT1G69830	1049.063	-0.819487759	0.178271	-4.59688	4.29E-06	4.57E-05	9.254724	9.732744	9.639394	10.04197	10.57885	10.50919
AT5G53160	1093.975	-0.817565958	0.176661	-4.62789	3.69E-06	4.00E-05	9.995488	9.30686	9.462265	10.46303	10.41217	10.48802
AT1G69890	332.1062	-0.816057511	0.219834	-3.71215	2.06E-04	1.51E-03	8.095624	7.827749	7.748022	9.192751	8.417898	8.474656
AT5G19120	3393.144	-0.816027807	0.137203	-5.9476	2.72E-09	4.78E-08	11.4533	11.03303	11.25234	11.94507	12.24381	12.04741
AT1G22500	222.83	-0.815014624	0.195064	-4.17818	2.94E-05	2.64E-04	7.538073	7.020841	7.376074	7.915566	8.333883	8.214456
AT2G29340	785.5664	-0.814411005	0.162176	-5.02177	5.12E-07	6.42E-06	9.413203	8.84177	9.124637	10.07179	10.00005	9.845978
AT1G03610	1042.527	-0.813597298	0.110344	-7.37329	1.66E-13	4.84E-12	9.622913	9.405005	9.642746	10.41731	10.32237	10.39606
AT2G34430	73076.62	-0.813553662	0.183916	-4.42352	9.71E-06	9.66E-05	15.76391	15.35132	15.87539	16.37044	16.85672	16.24201
AT5G63030	423.0534	-0.813471647	0.13139	-6.1913	5.97E-10	1.15E-08	8.290921	8.242062	8.230342	9.141766	9.195448	8.897523
AT3G03970	61.37856	-0.811830009	0.251596	-3.22672	0.001252	0.0073629	5.384387	5.610218	5.428035	6.138016	6.499609	6.316346
AT5G16160	492.8424	-0.811267563	0.115108	-7.04791	1.82E-12	4.79E-11	8.437566	8.499975	8.507852	9.19692	9.342436	9.353722
AT2G16600	2707.623	-0.810599991	0.081231	-9.9789	1.89E-23	1.25E-21	10.96001	10.89896	10.96068	11.80804	11.69346	11.75641
AT3G43670	223.1839	-0.808668634	0.152289	-5.31009	1.10E-07	1.53E-06	7.235299	7.32473	7.464605	8.215903	8.086794	8.171351
AT1G71080	501.697	-0.808527562	0.125904	-6.4218	1.35E-10	2.84E-09	8.64098	8.437994	8.429798	9.376713	9.369063	9.227139

AT3G44880	590.6367	-0.807671936	0.160851	-5.02124	5.13E-07	6.44E-06	8.503434	8.677143	8.999486	9.41658	9.55217	9.699794
AT5G63260	220.453	-0.806525172	0.152912	-5.27443	1.33E-07	1.84E-06	7.358247	7.300054	7.298852	8.041442	8.312092	8.067702
AT1G73680	322.9568	-0.806104804	0.132282	-6.09383	1.10E-09	2.05E-08	7.831287	7.937414	7.84434	8.694056	8.785191	8.591379
AT2G28910	963.4845	-0.805725068	0.175461	-4.59203	4.39E-06	4.66E-05	9.329266	9.799404	9.113405	10.24685	10.34401	10.20929
AT1G21610	436.1736	-0.805589562	0.127295	-6.32853	2.48E-10	5.04E-09	8.409405	8.290468	8.221402	9.005814	9.153175	9.19982
AT5G23210	438.4258	-0.803701594	0.184043	-4.36692	1.26E-05	0.0001227	8.38551	8.07758	8.429798	8.912575	9.470914	8.953118
AT5G59960	494.7264	-0.80212685	0.159629	-5.02495	5.04E-07	6.33E-06	8.600037	8.408286	8.450348	8.97216	9.425968	9.464998
AT5G66040	1224.768	-0.801789795	0.20898	-3.83668	0.000125	0.0009632	10.20658	9.354372	9.665996	10.65403	10.80045	10.35675
AT1G01770	242.5343	-0.801681428	0.143698	-5.57894	2.42E-08	3.70E-07	7.555311	7.414827	7.433975	8.311275	8.197907	8.314072
AT3G22440	2655.904	-0.801329671	0.100319	-7.98782	1.37E-15	4.85E-14	11.04941	10.81784	10.87206	11.78815	11.68395	11.69524
AT5G54520	87.72859	-0.800454809	0.234352	-3.41561	6.36E-04	4.10E-03	6.168486	5.657607	6.121948	6.795248	6.871459	6.809501
AT1G69850	391.7619	-0.800446792	0.164867	-4.8551	1.20E-06	1.42E-05	8.353844	7.908323	8.157219	9.088912	9.025087	8.775355
AT5G11900	218.3317	-0.800399022	0.150497	-5.31839	1.05E-07	1.47E-06	7.267032	7.280005	7.407944	8.078063	8.073902	8.214456
AT4G34230	138.6779	-0.799979815	0.229089	-3.492	4.79E-04	3.20E-03	6.541497	6.868059	6.560439	7.292624	7.190921	7.850673
AT4G28720	370.8752	-0.799187947	0.181715	-4.39802	1.09E-05	0.0001076	8.139144	7.799859	8.23331	8.682211	9.171444	8.775355
AT3G07525	86.67915	-0.798914685	0.234751	-3.40324	6.66E-04	4.26E-03	6.201706	5.805191	5.885862	6.795248	7.013254	6.602557
AT1G08230	139.2855	-0.797823038	0.222456	-3.58643	3.35E-04	0.0023294	7.022492	6.485861	6.411182	7.535811	7.349087	7.557346
AT3G04550	286.823	-0.797218534	0.215101	-3.70625	2.10E-04	1.54E-03	7.439402	7.838071	7.788938	8.013353	8.72049	8.72514
AT1G76180	3784.634	-0.79698836	0.128554	-6.19962	5.66E-10	1.09E-08	11.46674	11.46298	11.3499	11.96476	12.30921	12.39291
AT3G48170	838.5523	-0.796889122	0.102578	-7.76864	7.93E-15	2.61E-13	9.302719	9.174653	9.291964	9.987665	10.08923	10.0986
AT1G80310	136.8454	-0.796355695	0.238669	-3.33666	8.48E-04	5.24E-03	6.286726	6.57083	6.96214	7.685719	7.491614	7.163834
AT1G27090	1195.227	-0.79593973	0.173588	-4.58522	4.54E-06	4.80E-05	9.452834	10.11942	9.623646	10.58732	10.65006	10.48173
AT5G05080	841.2414	-0.795897535	0.121062	-6.57431	4.89E-11	1.09E-09	9.283453	9.13753	9.350538	9.916143	10.19124	10.0754
AT5G65207	449.4412	-0.795846856	0.156309	-5.09149	3.55E-07	4.56E-06	8.481059	8.114844	8.432382	9.061741	9.358471	9.054882
AT5G51980	314.1984	-0.795818839	0.165728	-4.80197	1.57E-06	1.81E-05	7.813489	7.865238	7.852083	8.386201	8.590752	8.912559
AT1G15350	345.4741	-0.795737085	0.153457	-5.18542	2.16E-07	2.88E-06	8.101503	7.746067	8.050212	8.740488	8.913741	8.69502
AT4G14430	383.5406	-0.795189023	0.135728	-5.85871	4.66E-09	7.89E-08	8.251806	8.036344	8.080192	8.937702	9.04504	8.815906
AT3G01035	94.1124	-0.794558948	0.235876	-3.36854	7.56E-04	4.75E-03	5.977105	6.152596	6.134702	6.656843	7.305638	6.743763
AT4G08825	64.85487	-0.793370822	0.244558	-3.24411	0.001178	0.0069828	5.495717	5.561219	5.639679	6.238498	6.610887	6.316346
AT1G47710	172.7747	-0.793281024	0.178335	-4.44825	8.66E-06	8.69E-05	7.118391	6.770842	7.04555	7.732415	7.723906	7.89677
AT2G41530	806.6588	-0.792728437	0.104364	-7.59582	3.06E-14	9.51E-13	9.19136	9.14309	9.269009	9.982845	10.08923	9.933349
AT3G18080	1538.2	-0.792439247	0.115559	-6.85743	7.01E-12	1.73E-10	10.10123	10.14943	10.15029	10.72791	10.98047	11.06887
AT3G13445	227.5334	-0.792247181	0.156125	-5.07444	3.89E-07	4.98E-06	7.444036	7.269875	7.402681	8.078063	8.35535	8.107448
AT3G17020	1336.607	-0.791371931	0.105105	-7.52938	5.10E-14	1.56E-12	9.853778	9.932994	10.0033	10.61562	10.84666	10.71938

AT4G20890	689.479	-0.790846438	0.174298	-4.53734	5.70E-06	5.92E-05	8.81744	9.039569	9.042421	9.376713	10.03705	9.849889
AT4G16330	350.5053	-0.790422873	0.132198	-5.9791	2.24E-09	4.01E-08	8.104434	7.885283	8.019596	8.779925	8.761267	8.859233
AT2G39310	484.8165	-0.787469778	0.238168	-3.30636	9.45E-04	5.75E-03	8.784929	8.399021	8.106327	9.738801	8.949469	8.999626
AT3G20550	257.3778	-0.786480416	0.170068	-4.62451	3.75E-06	4.06E-05	7.457851	7.796335	7.392097	8.25641	8.333883	8.474656
AT5G57565	183.8819	-0.78513743	0.183991	-4.26726	1.98E-05	0.0001843	7.124178	7.008709	7.11799	8.050685	7.510873	7.991862
AT1G31850	338.3238	-0.783887143	0.168125	-4.66252	3.12E-06	3.43E-05	7.869688	8.10633	7.878863	8.491765	8.728738	8.985475
AT4G31420	512.0025	-0.783113288	0.122121	-6.41258	1.43E-10	3.01E-09	8.634912	8.523615	8.512746	9.40221	9.189485	9.423612
AT2G37480	802.659	-0.781393674	0.109351	-7.14571	8.95E-13	2.45E-11	9.248114	9.110821	9.255935	9.994865	9.890353	10.07707
AT1G66410	974.5885	-0.779070002	0.114036	-6.8318	8.39E-12	2.06E-10	9.466565	9.475018	9.507096	10.09207	10.40961	10.29354
AT3G03870	824.7123	-0.778961981	0.146019	-5.33467	9.57E-08	1.35E-06	9.490285	9.020059	9.168711	9.987665	10.11777	9.985829
AT1G31830	162.7463	-0.778787713	0.16643	-4.67937	2.88E-06	3.19E-05	6.879269	7.014788	6.819344	7.697535	7.690514	7.702828
AT2G46680	295.1719	-0.778637836	0.218718	-3.56	3.71E-04	2.55E-03	7.448656	8.10348	7.640345	8.615264	8.185975	8.795773
AT2G25730	611.1337	-0.776202842	0.157768	-4.91991	8.66E-07	1.05E-05	8.555759	9.073452	8.755025	9.692313	9.495291	9.601156
AT4G27990	216.3299	-0.776004956	0.168631	-4.6018	4.19E-06	4.47E-05	7.129942	7.269875	7.528819	8.113777	8.047769	8.146128
AT4G36380	171.1488	-0.775265456	0.198311	-3.90934	9.25E-05	0.0007415	7.256532	6.847771	6.762956	7.661791	7.923931	7.720024
AT4G08290	231.0723	-0.774758076	0.191949	-4.03627	5.43E-05	4.59E-04	7.147097	7.639949	7.392097	7.935661	8.333883	8.302704
AT5G65495	98.81097	-0.77462265	0.210079	-3.6873	0.000227	0.0016512	6.201706	6.04978	6.290994	7.018926	7.11744	6.809501
AT5G51570	185.95	-0.77432977	0.157242	-4.92446	8.46E-07	1.02E-05	7.191872	7.032872	7.072314	7.853545	7.923931	7.881567
AT1G15910	392.7692	-0.773149952	0.120747	-6.40304	1.52E-10	3.19E-09	8.243855	8.186602	8.106327	8.986679	8.928139	8.960371
AT3G48990	3699.306	-0.772696403	0.100438	-7.69325	1.43E-14	4.61E-13	11.4795	11.2566	11.49049	12.14655	12.17832	12.24484
AT3G13672	259.9249	-0.771816688	0.14116	-5.46766	4.56E-08	6.73E-07	7.622271	7.563222	7.58566	8.364129	8.244671	8.469581
AT1G15670	446.9606	-0.771800772	0.143616	-5.37405	7.70E-08	1.10E-06	8.555759	8.215918	8.291418	9.205221	9.201388	9.027518
AT4G27870	1111.237	-0.771150713	0.095079	-8.11062	5.04E-16	1.85E-14	9.730209	9.635501	9.67257	10.53064	10.42746	10.40539
AT4G15490	198.7841	-0.770905234	0.181593	-4.24523	2.18E-05	0.0002018	6.952657	7.23385	7.386775	8.050685	7.804154	8.067702
AT5G52170	389.3502	-0.770815051	0.190088	-4.05505	5.01E-05	4.27E-04	8.208881	8.04528	8.206377	8.570985	9.320774	8.866973
AT4G24060	159.0469	-0.770771034	0.194215	-3.96865	7.23E-05	0.0005963	6.78012	6.907798	6.954967	7.426454	7.567152	7.934089
AT3G53530	232.6525	-0.770401354	0.163925	-4.69972	2.61E-06	2.91E-05	7.533732	7.280005	7.428806	8.165743	8.397349	8.04741
AT1G22190	547.3391	-0.769828807	0.187252	-4.1112	3.94E-05	0.0003433	8.981043	8.325123	8.570211	9.632015	9.37433	9.291818
AT1G33970	440.5993	-0.769719669	0.141684	-5.43263	5.55E-08	8.08E-07	8.124783	8.435731	8.455441	9.057163	9.109626	9.19982
AT3G07890	379.8064	-0.769688649	0.129377	-5.94918	2.69E-09	4.75E-08	8.127666	8.080481	8.182008	8.768767	9.025087	8.92744
AT5G41600	327.7096	-0.768933268	0.160519	-4.79031	1.67E-06	1.91E-05	7.956585	7.86187	7.916278	8.450462	8.970488	8.637358
AT1G02660	1028	-0.767551188	0.175885	-4.36393	1.28E-05	1.24E-04	9.635124	9.388714	9.642746	9.982845	10.67588	10.3007
AT1G73980	431.6388	-0.767209423	0.126695	-6.05555	1.40E-09	2.57E-08	8.283183	8.270284	8.406322	8.991487	9.064722	9.208984
AT1G80480	380.9665	-0.765944686	0.120256	-6.36928	1.90E-10	3.92E-09	8.083793	8.148407	8.185076	8.937702	8.920958	8.878505

AT2G27830	575.9637	-0.76454027	0.18594	-4.11177	3.93E-05	3.43E-04	8.985819	8.317767	8.805749	9.354494	9.480714	9.677961
AT5G56520	148.5252	-0.764454973	0.205349	-3.72271	1.97E-04	1.45E-03	6.823449	6.785138	6.704273	7.195969	7.880172	7.547747
AT5G04750	519.0926	-0.763973162	0.114859	-6.65139	2.90E-11	6.67E-10	8.63896	8.563555	8.546545	9.350757	9.451111	9.268632
AT4G37520	361.7286	-0.763170657	0.210771	-3.62086	0.000294	0.0020712	8.313891	7.984634	7.824797	9.217584	8.695459	8.524443
AT1G18330	475.3104	-0.762609479	0.184142	-4.14143	3.45E-05	0.0003045	8.721446	8.223811	8.36091	8.952571	9.475822	9.230143
AT1G12050	446.0489	-0.761981648	0.126063	-6.04445	1.50E-09	2.74E-08	8.280594	8.334874	8.478136	9.061741	9.242291	9.108099
AT5G58490	398.1177	-0.761618008	0.120814	-6.30407	2.90E-10	5.85E-09	8.195201	8.178502	8.24512	8.881834	9.01163	9.024061
AT1G20630	731.1889	-0.76139197	0.186653	-4.07918	4.52E-05	0.0003888	8.813864	9.02609	9.32989	9.523344	10.13028	9.847935
AT5G24680	98.27927	-0.757104286	0.202287	-3.74273	0.000182	0.0013492	6.212611	6.084872	6.279555	6.96128	7.013254	6.932619
AT2G46220	4242.351	-0.756042188	0.102503	-7.37578	1.63E-13	4.77E-12	11.70572	11.48038	11.66411	12.34695	12.48261	12.31194
AT1G35720	1362.652	-0.755316591	0.123782	-6.10199	1.05E-09	1.95E-08	9.852036	10.07065	10.01972	10.70614	10.59021	10.91592
AT3G10770	1625.693	-0.754367114	0.115745	-6.51749	7.15E-11	1.57E-09	10.38167	10.03969	10.27001	11.00252	11.01291	10.98067
AT1G78670	648.2325	-0.754255121	0.100722	-7.48848	6.97E-14	2.10E-12	8.917477	8.916724	8.904006	9.692313	9.697925	9.628733
AT1G15230	205.9603	-0.754240808	0.174296	-4.32735	1.51E-05	1.45E-04	7.05315	7.396321	7.298852	7.842944	8.161812	8.06097
AT4G25670	364.0717	-0.75418232	0.131469	-5.73656	9.66E-09	1.56E-07	8.035477	8.156677	8.053574	8.717459	8.854671	8.945828
AT2G02950	605.0033	-0.753617312	0.156029	-4.82999	1.37E-06	1.60E-05	9.052587	8.588223	8.744662	9.444897	9.722914	9.549187
AT4G37250	157.7284	-0.750922513	0.189982	-3.9526	7.73E-05	6.32E-04	7.047071	6.595375	6.954967	7.63746	7.621318	7.66781
AT4G30550	627.6077	-0.750704571	0.120272	-6.2417	4.33E-10	8.45E-09	8.971444	8.713102	8.904006	9.597745	9.689499	9.589508
AT4G02370	360.3203	-0.750371544	0.144398	-5.19653	2.03E-07	2.73E-06	8.197947	7.85511	8.125623	8.801986	8.877107	8.80386
AT5G11600	138.1788	-0.750160845	0.209234	-3.58527	3.37E-04	2.34E-03	6.90639	6.45042	6.643102	7.548916	7.585434	7.213246
AT3G16460	809.8257	-0.749540301	0.139924	-5.35678	8.47E-08	1.20E-06	9.4238	9.266673	8.973099	9.960952	10.05356	9.966147
AT1G74920	1718.027	-0.749406418	0.091083	-8.22772	1.91E-16	7.25E-15	10.36037	10.22675	10.37384	11.107	11.08053	11.03835
AT5G27320	286.1124	-0.749342243	0.139565	-5.36914	7.91E-08	1.13E-06	7.795468	7.604113	7.804986	8.531918	8.487601	8.464489
AT1G13448	75.3432	-0.749080864	0.23865	-3.13882	1.70E-03	0.0095315	5.925012	5.54451	5.959397	6.582321	6.537663	6.639175
AT3G09260	2359.506	-0.747579093	0.110786	-6.74794	1.50E-11	3.54E-10	10.88743	10.82305	10.6108	11.45122	11.62552	11.51856
AT1G72160	1725.86	-0.746831824	0.121654	-6.13898	8.31E-10	1.56E-08	10.18044	10.39523	10.39785	10.8963	11.14666	11.1872
AT5G37780	675.8693	-0.746521814	0.118276	-6.31167	2.76E-10	5.59E-09	8.864873	8.98952	9.064252	9.659462	9.85688	9.671346
AT2G40940	784.2776	-0.746046908	0.099943	-7.46469	8.35E-14	2.51E-12	9.238808	9.169213	9.173354	10.01389	9.894025	9.92039
AT2G16740	93.49665	-0.745906624	0.216209	-3.44994	0.000561	0.0036802	5.98984	6.217283	6.159877	6.680852	7.066287	6.917792
AT5G16040	162.3929	-0.744215955	0.182412	-4.07987	4.51E-05	0.0003879	6.959147	6.996475	6.819344	7.45458	7.690514	7.866203
AT2G02060	87.07263	-0.744079444	0.223373	-3.33111	0.000865	0.0053227	6.099659	6.001621	6.01564	6.96128	6.460523	6.887672
AT3G26910	120.772	-0.742948345	0.185392	-4.00745	6.14E-05	0.0005136	6.424426	6.545861	6.531797	7.338627	7.214606	7.213246
AT1G11545	282.3731	-0.742841318	0.230583	-3.22158	1.27E-03	7.49E-03	7.420713	7.806883	7.863621	7.895187	8.695459	8.712309
AT3G29320	2339.013	-0.742774166	0.174791	-4.24949	2.14E-05	0.0001984	10.37622	10.91651	10.93083	11.24927	11.62773	11.65613

AT1G04990	129.8301	-0.742293846	0.192545	-3.85516	0.000116	0.0009025	6.558693	6.603464	6.678374	7.323456	7.142352	7.566881
AT1G23870	1241.424	-0.741674086	0.122161	-6.07129	1.27E-09	2.35E-08	9.982803	9.863119	9.709271	10.44561	10.66947	10.68914
AT2G45740	1022.813	-0.741494802	0.111697	-6.63843	3.17E-11	7.25E-10	9.611628	9.488184	9.625906	10.19361	10.45754	10.31633
AT3G20290	669.1179	-0.741297452	0.108957	-6.8036	1.02E-11	2.47E-10	8.987407	8.863826	9.045801	9.674487	9.727037	9.738279
AT1G23020	661.738	-0.740914464	0.119589	-6.19552	5.81E-10	1.12E-08	9.092985	8.808914	8.937147	9.712836	9.681022	9.699794
AT4G25690	354.6128	-0.740874393	0.142906	-5.18435	2.17E-07	2.89E-06	8.156191	7.978426	7.998818	8.887003	8.891872	8.614552
AT4G28650	321.1923	-0.740232267	0.143418	-5.16137	2.45E-07	3.24E-06	7.859316	7.918085	7.956343	8.845121	8.497289	8.605328
AT1G21780	917.0896	-0.739828211	0.134215	-5.51225	3.54E-08	5.31E-07	9.563457	9.23708	9.438031	10.2982	10.18825	10.007
AT5G02810	1493.148	-0.739297293	0.112105	-6.59466	4.26E-11	9.58E-10	10.09166	10.2527	10.01542	10.77746	10.98908	10.83832
AT1G73750	176.2425	-0.739029411	0.18774	-3.93645	8.27E-05	0.0006718	7.047071	6.834086	7.211253	7.612711	8.007659	7.753812
AT1G34370	1015.821	-0.738769614	0.10155	-7.27491	3.47E-13	9.75E-12	9.611628	9.510941	9.586993	10.41552	10.28371	10.23792
AT1G10210	187.1753	-0.738012394	0.192915	-3.82557	1.30E-04	1.00E-03	7.397007	6.874758	7.078929	7.984706	7.740317	7.911813
AT1G51610	128.8707	-0.737844715	0.189391	-3.89588	9.78E-05	0.0007762	6.6255	6.468249	6.678374	7.179211	7.491614	7.363061
AT5G47860	464.1028	-0.737451928	0.119412	-6.17571	6.59E-10	1.26E-08	8.437566	8.387355	8.493071	9.088912	9.293232	9.171974
AT3G10740	1390.576	-0.737075786	0.097225	-7.58117	3.42E-14	1.06E-12	9.955473	10.07284	10.04105	10.6646	10.83715	10.79232
AT5G08520	669.029	-0.736618934	0.126406	-5.8274	5.63E-09	9.45E-08	9.15366	8.855383	8.879589	9.718646	9.672496	9.744595
AT1G79970	283.1973	-0.733858381	0.139781	-5.25007	1.52E-07	2.08E-06	7.802703	7.678751	7.705911	8.564547	8.497289	8.358672
AT3G02340	355.3923	-0.733811146	0.145909	-5.02924	4.92E-07	6.20E-06	8.235859	7.996969	7.91258	8.768767	8.906489	8.729392
AT5G16120	758.9131	-0.733015455	0.108948	-6.72814	1.72E-11	4.04E-10	9.171935	9.08794	9.191779	9.923709	9.975995	9.773708
AT3G10113	257.1081	-0.732646909	0.192397	-3.80799	1.40E-04	0.0010686	7.79909	7.423992	7.504459	7.994319	8.581674	8.385863
AT3G25910	1155.186	-0.731186646	0.099827	-7.32454	2.40E-13	6.86E-12	9.872809	9.718848	9.683462	10.47681	10.50393	10.50919
AT5G65210	143.6931	-0.730528979	0.188345	-3.87867	0.000105	0.0008266	6.919761	6.727085	6.606946	7.661791	7.411902	7.416778
AT4G37610	657.0335	-0.730369249	0.214253	-3.40892	6.52E-04	4.19E-03	9.350404	8.680971	8.65676	9.520021	9.989787	9.512649
AT4G28390	147.0745	-0.730196979	0.199373	-3.66247	2.50E-04	1.80E-03	6.9264	6.704699	6.729716	7.821505	7.349087	7.384788
AT2G26430	883.829	-0.729907947	0.103216	-7.07166	1.53E-12	4.06E-11	9.467704	9.288162	9.362786	10.07632	10.15803	10.09695
AT5G03730	752.3486	-0.729770802	0.107476	-6.7901	1.12E-11	2.70E-10	9.10474	9.234478	9.085757	9.797627	9.912245	9.922248
AT2G01140	802.0427	-0.729705289	0.120506	-6.05534	1.40E-09	2.57E-08	9.232125	9.113656	9.342314	9.999646	10.07312	9.834182
AT1G04503	874.9949	-0.729687561	0.171379	-4.25775	2.06E-05	1.92E-04	9.092985	9.373421	9.555326	9.859417	10.37324	10.02094
AT1G28960	463.9072	-0.729637439	0.118328	-6.16621	6.99E-10	1.33E-08	8.425899	8.491282	8.411572	9.217584	9.253768	9.075069
AT1G12790	120.3652	-0.729034133	0.200172	-3.64203	2.70E-04	1.93E-03	6.6255	6.404859	6.492702	7.426454	6.985988	7.284329
AT4G05070	1842.286	-0.728962839	0.141402	-5.15526	2.53E-07	3.35E-06	10.61688	10.25398	10.39653	11.15063	11.36081	10.97799
AT4G05060	293.4708	-0.728938191	0.142533	-5.11418	3.15E-07	4.09E-06	7.855842	7.65948	7.836554	8.633832	8.487601	8.443937
AT3G21870	169.6131	-0.728608408	0.186243	-3.91215	9.15E-05	0.0007334	7.083171	6.914316	6.969278	7.440585	7.966403	7.770414
AT3G20410	499.6462	-0.728407242	0.119908	-6.07469	1.24E-09	2.30E-08	8.672917	8.451502	8.529744	9.266002	9.293232	9.306122

AT1G06110	616.8642	-0.727579703	0.124766	-5.83155	5.49E-09	9.22E-08	8.907444	8.711231	8.940783	9.692313	9.597913	9.480216
AT5G10450	5537.6	-0.726933965	0.118631	-6.1277	8.92E-10	1.67E-08	12.13075	11.82568	12.08979	12.70329	12.88948	12.66647
AT4G17900	391.5906	-0.726883375	0.146124	-4.97442	6.54E-07	8.08E-06	8.353844	8.178502	8.070268	9.057163	8.745094	8.989026
AT3G03105	3230.212	-0.726732945	0.141183	-5.14744	2.64E-07	3.48E-06	11.43071	11.07399	11.19868	11.7833	12.18582	11.94281
AT4G11360	298.1349	-0.726653013	0.165753	-4.38396	1.17E-05	1.14E-04	8.02314	7.559068	7.80099	8.609021	8.581674	8.449103
AT1G01470	415.8674	-0.726497541	0.185391	-3.91873	8.90E-05	0.0007184	8.39272	8.244651	8.215411	8.615264	9.051631	9.325912
AT5G66880	521.4886	-0.726485317	0.112169	-6.4767	9.37E-11	2.02E-09	8.698355	8.573885	8.572556	9.347011	9.384807	9.317464
AT3G22430	118.0792	-0.725912316	0.211324	-3.43507	5.92E-04	3.85E-03	6.592483	6.579058	6.2564	7.338627	6.92986	7.341001
AT4G28652	313.2044	-0.725279938	0.141573	-5.12303	3.01E-07	3.92E-06	7.827745	7.885283	7.941902	8.779925	8.45814	8.5867
AT4G37300	3573.399	-0.725268308	0.098804	-7.34047	2.13E-13	6.12E-12	11.42633	11.26523	11.47678	12.15139	12.18132	12.03243
AT5G27520	157.8331	-0.724056482	0.178195	-4.06327	4.84E-05	4.14E-04	6.9264	6.996475	6.779293	7.63746	7.452309	7.778644
AT3G12290	1026.657	-0.72362914	0.105122	-6.88373	5.83E-12	1.45E-10	9.635124	9.47942	9.660494	10.30594	10.40189	10.26307
AT4G39660	381.8386	-0.722866404	0.152859	-4.72897	2.26E-06	2.54E-05	8.259715	8.012241	8.218409	8.658224	9.01163	9.00665
AT2G42610	165.6936	-0.721856147	0.195987	-3.68318	2.30E-04	1.67E-03	7.169658	6.778008	6.918555	7.440585	7.819681	7.819103
AT3G23710	139.8129	-0.721791012	0.184471	-3.91277	9.12E-05	7.32E-04	6.899657	6.658856	6.597764	7.412182	7.452309	7.5087
AT5G16110	2263.788	-0.720294293	0.170379	-4.22759	2.36E-05	0.0002167	10.99518	10.491	10.66015	11.73252	11.3884	11.23284
AT1G76150	600.8042	-0.720058389	0.114876	-6.26816	3.65E-10	7.24E-09	8.929095	8.724273	8.809731	9.546397	9.606891	9.500261
AT1G15800	144.0288	-0.719695291	0.19053	-3.77734	0.000159	0.0011924	6.858589	6.635377	6.779293	7.276958	7.723906	7.478706
AT3G15840	1386.936	-0.719693518	0.147792	-4.86963	1.12E-06	1.32E-05	9.874527	9.918477	10.25548	10.60625	10.9596	10.68695
AT5G40720	192.641	-0.719553548	0.170557	-4.21884	2.46E-05	2.25E-04	7.169658	7.186169	7.229204	7.905412	7.673524	8.120457
AT1G62310	530.9986	-0.719086179	0.146186	-4.91897	8.70E-07	1.05E-05	8.71187	8.688596	8.541764	9.520021	9.084138	9.472627
AT5G06870	235.0027	-0.718917474	0.159482	-4.50783	6.55E-06	6.73E-05	7.392218	7.624132	7.376074	8.295811	8.244671	8.06097
AT2G31800	807.4697	-0.718851844	0.135305	-5.31281	1.08E-07	1.51E-06	9.376095	9.16512	9.194827	9.753026	10.13649	10.01921
AT4G19190	456.0632	-0.718706266	0.130769	-5.49602	3.88E-08	5.78E-07	8.402278	8.565627	8.311216	9.213474	9.03842	9.19982
AT2G45380	671.7502	-0.717852415	0.109255	-6.57043	5.02E-11	1.12E-09	9.048027	8.963043	8.958825	9.712836	9.606891	9.800225
AT1G25560	2159.957	-0.717324058	0.129598	-5.53498	3.11E-08	4.70E-07	10.79975	10.4172	10.75974	11.38334	11.49137	11.30584
AT1G01160	631.5375	-0.717052023	0.121764	-5.88889	3.89E-09	6.66E-08	8.985819	8.735359	8.962407	9.552916	9.637878	9.671346
AT1G25320	91.23821	-0.716342315	0.211329	-3.38971	0.0007	0.0044447	6.234178	6.096383	6.01564	6.773083	6.810594	6.947296
AT3G11600	236.3904	-0.716193138	0.212961	-3.36303	7.71E-04	4.83E-03	7.813489	7.16446	7.354429	8.232242	8.417898	7.963265
AT2G01940	168.5534	-0.715833693	0.225645	-3.17239	1.51E-03	8.64E-03	7.303193	7.014788	6.569861	7.743857	7.510873	7.89677
AT1G02610	389.3425	-0.715285963	0.165033	-4.3342	1.46E-05	1.41E-04	8.43524	8.039329	8.076891	8.927704	9.090552	8.750466
AT4G12570	505.4879	-0.714797256	0.109194	-6.54614	5.90E-11	1.31E-09	8.591707	8.577996	8.567861	9.347011	9.304312	9.248035
AT4G23680	351.7909	-0.714406071	0.155374	-4.59797	4.27E-06	4.55E-05	8.124783	8.14564	7.875068	8.97216	8.728738	8.619142
AT4G14930	465.4615	-0.714103165	0.142931	-4.99612	5.85E-07	7.28E-06	8.587524	8.334874	8.432382	9.262029	9.293232	8.978347

AT5G48160	1990.03	-0.712151587	0.096537	-7.37702	1.62E-13	4.73E-12	10.66134	10.55027	10.45299	11.31307	11.21871	11.28649
AT1G48420	746.5606	-0.711851544	0.10424	-6.82899	8.55E-12	2.09E-10	9.135151	9.069078	9.221974	9.811286	9.901341	9.867357
AT1G47410	183.2334	-0.711684937	0.170743	-4.16817	3.07E-05	0.0002747	7.095007	7.186169	7.04555	7.832265	8.034523	7.649977
AT5G24460	148.7236	-0.710875373	0.195763	-3.6313	2.82E-04	0.0019978	6.727867	6.619509	7.058994	7.535811	7.656331	7.437718
AT5G56180	346.7716	-0.710119908	0.132417	-5.36277	8.20E-08	1.16E-06	8.127666	8.024343	7.956343	8.64608	8.831881	8.78764
AT1G03760	155.5329	-0.709717422	0.185859	-3.81858	1.34E-04	1.03E-03	7.106746	6.785138	6.738098	7.625138	7.529878	7.658921
AT5G42850	462.0042	-0.70752043	0.147893	-4.78399	1.72E-06	1.97E-05	8.540693	8.28796	8.507852	9.324326	9.207302	8.956749
AT2G26355	294.9224	-0.707216899	0.185211	-3.81844	1.34E-04	1.03E-03	7.963058	7.495286	7.901428	8.717459	8.535404	8.296986
AT3G57750	99.31985	-0.707212207	0.20123	-3.51444	4.41E-04	2.97E-03	6.327428	6.24857	6.147344	6.901235	6.985988	7.004552
AT1G12230	885.4575	-0.707174578	0.160294	-4.41173	1.03E-05	1.02E-04	9.315421	9.262847	9.565959	9.775499	10.23821	10.25866
AT4G34000	565.0327	-0.70715715	0.153226	-4.61513	3.93E-06	4.23E-05	8.744172	8.906952	8.558426	9.285706	9.395208	9.666919
AT1G78890	331.6209	-0.706522304	0.156505	-4.51437	6.35E-06	6.55E-05	8.04466	7.810381	8.046842	8.484963	8.891872	8.677522
AT4G12460	291.5351	-0.706282029	0.139356	-5.06817	4.02E-07	5.13E-06	7.784546	7.844911	7.752166	8.386201	8.487601	8.628279
AT1G30360	2306.142	-0.706236475	0.119099	-5.92981	3.03E-09	5.28E-08	10.77743	10.67833	10.85193	11.28691	11.64308	11.50374
AT2G34590	1424.39	-0.705686321	0.14257	-4.94977	7.43E-07	9.07E-06	10.15946	9.854672	10.18672	10.57456	10.9596	10.8145
AT1G04310	199.3585	-0.705486042	0.170803	-4.1304	3.62E-05	0.0003187	7.059205	7.244235	7.413188	8.078063	7.865285	7.926702
AT1G69870	297.9632	-0.705288934	0.19337	-3.64736	0.000265	0.0018912	7.618176	7.838071	7.977737	8.881834	8.333883	8.330959
AT3G14990	2011.175	-0.704974063	0.137096	-5.14219	2.72E-07	3.57E-06	10.64482	10.59913	10.47447	11.50768	11.02301	11.29081
AT5G59440	325.6814	-0.704817367	0.135942	-5.18469	2.16E-07	2.89E-06	8.056815	7.841495	7.941902	8.62148	8.753203	8.619142
AT5G61530	519.0487	-0.704780267	0.137326	-5.13218	2.86E-07	3.75E-06	8.670942	8.594325	8.609573	9.536562	9.109626	9.323102
AT4G02380	4282.736	-0.704066678	0.177063	-3.97636	7.00E-05	0.0005786	11.95601	11.3221	11.63186	12.60084	12.37374	12.12812
AT2G34410	5315.991	-0.703961	0.106897	-6.58542	4.54E-11	1.02E-09	12.02742	11.80369	12.08836	12.61103	12.75462	12.69502
AT2G22540	898.1167	-0.70219713	0.120776	-5.81407	6.10E-09	1.01E-07	9.453983	9.22271	9.548194	10.101	10.15803	10.11169
AT3G60300	1066.957	-0.700565012	0.114732	-6.1061	1.02E-09	1.91E-08	9.819412	9.560609	9.596244	10.39753	10.41983	10.29498
AT1G01780	88.15539	-0.699450897	0.209501	-3.33866	0.000842	0.005205	6.014976	6.084872	6.121948	6.727704	6.871459	6.777007
AT3G47860	168.0773	-0.69921431	0.205391	-3.40431	6.63E-04	4.25E-03	7.071238	6.854565	7.03878	7.353641	8.047769	7.676644
AT5G14780	4971.196	-0.698989308	0.108261	-6.45651	1.07E-10	2.29E-09	11.90334	11.8358	11.91252	12.40395	12.73479	12.6111
AT5G19530	336.4604	-0.697856058	0.152212	-4.58477	4.54E-06	4.81E-05	7.866239	8.097765	8.026456	8.892154	8.687018	8.529329
AT1G75170	100.2702	-0.697559875	0.208343	-3.34813	8.14E-04	5.07E-03	6.168486	6.45042	6.147344	6.96128	6.958197	7.004552
AT5G14240	745.4692	-0.697128816	0.129032	-5.40276	6.56E-08	9.47E-07	9.192737	9.00487	9.232395	9.715744	10.0237	9.808287
AT1G78600	207.4829	-0.696464084	0.181326	-3.84095	1.23E-04	9.49E-04	7.065234	7.533893	7.270239	8.087074	8.060895	7.89677
AT5G09220	598.5536	-0.695903798	0.170217	-4.08834	4.34E-05	3.76E-04	9.048027	8.652013	8.752958	9.246025	9.767632	9.561163
AT1G62480	2708.584	-0.695462179	0.175076	-3.97234	7.12E-05	0.0005877	11.31055	10.81175	10.83396	11.4451	11.95923	11.69903
AT2G37640	411.5445	-0.695423703	0.162312	-4.28448	1.83E-05	1.72E-04	8.411773	8.137306	8.311216	8.694056	9.140866	9.121102

AT2G18440	2098.2	-0.695058743	0.120764	-5.7555	8.64E-09	1.41E-07	10.7811	10.4456	10.68258	11.37692	11.22747	11.42006
AT1G68945	620.1745	-0.694352604	0.177639	-3.90877	9.28E-05	7.42E-04	9.078154	8.703726	8.829478	9.835552	9.637878	9.239116
AT3G22960	3428.687	-0.694209913	0.107786	-6.44062	1.19E-10	2.53E-09	11.40687	11.2232	11.41572	11.94755	12.18807	12.00988
AT1G29760	340.2735	-0.694187793	0.133052	-5.21742	1.81E-07	2.46E-06	8.101503	7.950158	8.002302	8.796502	8.761267	8.609947
AT4G38932	671.4345	-0.694039018	0.158433	-4.38066	1.18E-05	1.16E-04	9.237474	8.74636	8.964194	9.843551	9.706303	9.546779
AT4G27900	155.6342	-0.693949203	0.179061	-3.87549	1.06E-04	8.36E-04	6.787432	6.813311	7.06567	7.495767	7.690514	7.613635
AT4G14716	419.7073	-0.693055155	0.125269	-5.53252	3.16E-08	4.76E-07	8.308818	8.315306	8.355472	8.887003	9.03842	9.133989
AT5G60870	111.9893	-0.691958913	0.201967	-3.42611	0.000612	0.0039671	6.515311	6.404859	6.313604	7.110162	7.327526	6.947296
AT4G34020	414.5163	-0.691710378	0.117472	-5.88828	3.90E-09	6.68E-08	8.370981	8.272822	8.277109	9.024699	9.018374	8.97477
AT1G07420	171.6615	-0.691678396	0.208596	-3.31588	9.14E-04	5.59E-03	6.68935	7.080015	7.276007	7.535811	7.788457	7.881567
AT1G21000	776.2678	-0.691270215	0.103221	-6.69701	2.13E-11	4.95E-10	9.29119	9.180072	9.168711	9.943691	9.837942	9.938867
AT5G24060	267.7524	-0.690419189	0.158836	-4.34675	1.38E-05	1.34E-04	7.546718	7.895203	7.562248	8.378881	8.376502	8.380466
AT1G49032	629.6617	-0.687799014	0.177208	-3.88131	0.000104	0.0008189	9.254724	8.582096	8.801756	9.619647	9.57979	9.628733
AT3G08947	128.8893	-0.687651152	0.18313	-3.75499	1.73E-04	1.29E-03	6.750493	6.562555	6.560439	7.323456	7.370331	7.295843
AT2G15090	1203.667	-0.687595595	0.13633	-5.04363	4.57E-07	5.78E-06	9.979615	9.708572	9.825927	10.47165	10.75474	10.37314
AT3G58680	2069.491	-0.686790383	0.148309	-4.63081	3.64E-06	3.95E-05	10.63066	10.49318	10.73953	11.00846	11.53318	11.3787
AT3G22200	1124.549	-0.686117293	0.150732	-4.55192	5.32E-06	5.55E-05	9.523508	9.761949	9.926535	10.20607	10.49671	10.5979
AT1G01820	619.0218	-0.685244246	0.111651	-6.13738	8.39E-10	1.58E-08	8.979448	8.831475	8.852822	9.499917	9.633492	9.605789
AT1G16240	1230.905	-0.685048823	0.111006	-6.17125	6.78E-10	1.29E-08	9.980412	9.720709	9.925618	10.51069	10.63697	10.56378
AT2G46260	855.3856	-0.684720557	0.11771	-5.81703	5.99E-09	9.97E-08	9.468841	9.22926	9.362786	10.15556	9.944472	10.02959
AT3G07350	1044.841	-0.684433375	0.130121	-5.25998	1.44E-07	1.98E-06	9.72926	9.443819	9.73253	10.29625	10.48215	10.21839
AT5G44750	531.243	-0.684414868	0.163215	-4.19333	2.75E-05	0.0002487	8.821008	8.716835	8.44011	9.158962	9.298783	9.591845
AT5G57340	393.1041	-0.684366316	0.17521	-3.90598	9.38E-05	7.50E-04	8.192449	8.337301	8.163456	9.262029	8.669986	8.783557
AT5G40190	195.0231	-0.684197799	0.205516	-3.32918	8.71E-04	5.35E-03	7.141402	6.888065	7.552776	7.895187	8.007659	7.866203
AT5G04105	7974.471	-0.682232295	0.152233	-4.48149	7.41E-06	7.56E-05	12.55818	12.63798	12.51941	13.26728	13.54454	12.9235
AT4G32940	1003.356	-0.68182798	0.130978	-5.20565	1.93E-07	2.60E-06	9.778714	9.413082	9.547001	10.35712	10.15803	10.30356
AT3G54400	630.0561	-0.681380067	0.196594	-3.46593	5.28E-04	3.49E-03	8.682754	8.931259	9.08247	9.137435	9.890353	9.701959
AT5G66160	259.6467	-0.681377154	0.148841	-4.57789	4.70E-06	4.96E-05	7.670527	7.495286	7.739697	8.232242	8.417898	8.336545
AT3G56720	381.8956	-0.680772631	0.157737	-4.31586	1.59E-05	0.0001517	7.998147	8.292971	8.27423	8.652165	8.956509	9.020596
AT1G21760	444.3337	-0.680427291	0.134564	-5.05653	4.27E-07	5.43E-06	8.587524	8.28796	8.350014	9.047962	9.115929	9.14039
AT4G02715	360.5046	-0.6803256	0.145477	-4.67652	2.92E-06	3.24E-05	8.275402	8.112011	7.93827	8.839799	8.687018	8.866973
AT4G14620	309.4259	-0.680310444	0.143991	-4.72468	2.30E-06	2.59E-05	8.010698	7.858494	7.796984	8.429358	8.678527	8.628279
AT3G24518	101.4673	-0.680103724	0.214714	-3.16749	1.54E-03	0.0087631	6.223435	6.494586	6.121948	7.127736	6.900955	6.917792
AT2G36900	489.2688	-0.679224439	0.119113	-5.70237	1.18E-08	1.89E-07	8.620654	8.435731	8.604997	9.221681	9.213193	9.280272

AT5G66790	152.5557	-0.679140171	0.215101	-3.15732	0.001592	0.0090242	7.124178	6.477082	6.947758	7.649677	7.452309	7.595115
AT2G01490	923.8124	-0.678983495	0.130675	-5.19599	2.04E-07	2.73E-06	9.637149	9.333854	9.426408	10.17682	9.996634	10.27621
AT3G50440	356.5988	-0.678879653	0.132967	-5.10562	3.30E-07	4.26E-06	8.095624	8.036344	8.166565	8.64608	8.808725	8.886142
AT4G38440	264.4193	-0.678826357	0.149037	-4.55474	5.25E-06	5.49E-05	7.658614	7.705304	7.658123	8.471261	8.12479	8.433551
AT4G01000	1208.309	-0.677781931	0.113863	-5.9526	2.64E-09	4.66E-08	9.894982	9.921715	9.747138	10.39571	10.66947	10.54943
AT3G11570	203.2527	-0.677723508	0.154518	-4.38606	1.15E-05	1.13E-04	7.363149	7.319828	7.193077	8.013353	7.880172	8.02683
AT1G78700	311.708	-0.677576343	0.160277	-4.22754	2.36E-05	2.17E-04	7.890211	7.731377	8.063613	8.729019	8.635307	8.396597
AT3G05165	198.5071	-0.677080359	0.188543	-3.59111	0.000329	0.0022944	7.208311	7.482184	7.031979	7.732415	8.021154	8.06097
AT1G33700	153.328	-0.676155336	0.186069	-3.63389	0.000279	0.0019795	6.830546	6.97169	6.873611	7.732415	7.26084	7.66781
AT4G16150	1093.32	-0.674255857	0.096788	-6.96632	3.25E-12	8.33E-11	9.701465	9.801164	9.642746	10.42266	10.40189	10.36223
AT5G65020	279.7008	-0.674243734	0.138835	-4.85643	1.20E-06	1.41E-05	7.682343	7.731377	7.828726	8.407941	8.535404	8.358672
AT3G54880	215.7059	-0.673995612	0.167794	-4.0168	5.90E-05	4.95E-04	7.563853	7.37285	7.16848	8.113777	8.007659	8.06097
AT5G14270	623.2664	-0.673076607	0.118452	-5.68228	1.33E-08	2.11E-07	8.868326	8.978676	8.879589	9.496539	9.519264	9.725565
AT5G10650	205.8235	-0.672174209	0.169533	-3.96485	7.34E-05	6.05E-04	7.444036	7.269875	7.229204	8.104931	7.740317	8.09432
AT2G45170	1024.847	-0.671961901	0.14454	-4.64898	3.34E-06	3.65E-05	9.691755	9.507712	9.652756	10.07858	10.56509	10.22744
AT1G29400	2418.888	-0.671227722	0.093001	-7.21742	5.30E-13	1.47E-11	10.85387	10.88782	10.85338	11.6237	11.40908	11.57399
AT5G44260	1492.01	-0.670775095	0.165388	-4.05576	5.00E-05	4.26E-04	10.31049	9.822112	10.29792	10.78163	11.0627	10.66603
AT1G02305	1465.448	-0.668646071	0.103505	-6.46002	1.05E-10	2.24E-09	10.14458	10.0486	10.22748	10.70321	10.89327	10.84519
AT3G42150	182.5314	-0.668230066	0.19414	-3.442	0.000577	0.0037716	7.022492	7.020841	7.326908	7.884889	8.021154	7.528356
AT1G13280	209.1222	-0.668131951	0.17732	-3.76794	1.65E-04	0.0012335	7.31336	7.142421	7.528819	7.832265	8.060895	8.13335
AT3G26730	788.1985	-0.666799031	0.097172	-6.86204	6.79E-12	1.68E-10	9.218664	9.252596	9.283398	9.905994	9.886672	9.966147
AT3G18035	1091.097	-0.665136622	0.104759	-6.34919	2.16E-10	4.44E-09	9.709186	9.606613	9.835723	10.36455	10.37587	10.42519
AT3G04560	254.9849	-0.664097773	0.158897	-4.17943	2.92E-05	2.63E-04	7.634487	7.47779	7.752166	8.122569	8.333883	8.417829
AT5G05690	4716.862	-0.664077708	0.090018	-7.37716	1.62E-13	4.73E-12	11.82	11.86217	11.81164	12.60516	12.49475	12.39124
AT2G13360	13911.08	-0.663400794	0.124522	-5.32759	9.95E-08	1.40E-06	13.37936	13.26639	13.51312	13.88389	14.2563	14.01768
AT4G36050	335.7516	-0.662832939	0.137579	-4.81785	1.45E-06	1.68E-05	8.167445	7.927782	7.959931	8.751866	8.652751	8.668693
AT1G78070	255.4042	-0.662642362	0.206924	-3.20235	1.36E-03	7.93E-03	7.563853	7.643877	7.680041	8.113777	7.952384	8.712309
AT1G50570	418.6643	-0.661136859	0.129553	-5.1032	3.34E-07	4.31E-06	8.43524	8.202666	8.382459	8.986679	8.949469	9.081736
AT1G60710	940.6829	-0.660724996	0.092647	-7.13167	9.92E-13	2.69E-11	9.494759	9.478321	9.551764	10.18524	10.19124	10.1439
AT1G75240	263.0837	-0.659987544	0.204525	-3.22693	0.001251	0.0073593	8.02004	7.405604	7.479679	8.288016	8.535404	8.19614
AT2G44130	869.8319	-0.659959543	0.145827	-4.52562	6.02E-06	6.23E-05	9.625976	9.208195	9.307536	9.926222	10.19422	10.05691
AT5G03470	456.9182	-0.659872643	0.124361	-5.3061	1.12E-07	1.56E-06	8.446832	8.510769	8.455441	9.254049	8.970488	9.156268
AT4G01130	178.5116	-0.659770152	0.209891	-3.14339	1.67E-03	9.40E-03	7.11258	7.002605	7.193077	7.323456	8.086794	7.858459
AT1G22640	452.496	-0.659318204	0.168442	-3.91422	9.07E-05	7.29E-04	8.740409	8.221185	8.333514	8.996279	9.213193	9.149938

AT5G10960	1951.216	-0.65926378	0.117162	-5.62692	1.83E-08	2.86E-07	10.71697	10.42292	10.52621	11.11583	11.22163	11.33054
AT2G31585	155.3232	-0.659008257	0.17604	-3.74351	0.000181	0.0013457	6.933009	6.74913	7.04555	7.612711	7.603488	7.538084
AT3G16770	3322.725	-0.658982624	0.13854	-4.75663	1.97E-06	2.24E-05	11.54514	11.07726	11.31706	11.97875	12.1168	11.87822
AT4G30630	105.764	-0.658662803	0.201527	-3.26835	1.08E-03	0.0064779	6.405541	6.206701	6.452518	7.018926	7.142352	6.947296
AT4G31510	258.4427	-0.658182515	0.152502	-4.31589	1.59E-05	0.0001517	7.802703	7.516861	7.599527	8.341714	8.376502	8.226539
AT1G01170	1055.982	-0.657906939	0.134591	-4.88819	1.02E-06	1.21E-05	9.699528	9.472811	9.832791	10.21637	10.30868	10.48173
AT2G26110	357.6487	-0.657248677	0.14036	-4.68259	2.83E-06	3.15E-05	8.206155	7.934211	8.191195	8.699942	8.831881	8.807886
AT1G61970	137.1009	-0.655536888	0.196004	-3.3445	8.24E-04	5.12E-03	6.657778	6.888065	6.678374	7.353641	7.190921	7.622806
AT3G53540	765.6665	-0.655209395	0.11933	-5.49072	4.00E-08	5.94E-07	9.372452	9.148629	9.103707	9.898335	9.912245	9.816304
AT4G02725	835.6585	-0.65515996	0.10906	-6.00734	1.89E-09	3.42E-08	9.357791	9.273026	9.388306	9.867285	10.07959	10.04843
AT5G51150	407.5076	-0.654950865	0.144104	-4.54499	5.49E-06	5.72E-05	8.442206	8.175792	8.294263	8.855706	8.891872	9.133989
AT2G45050	120.5036	-0.654702147	0.192332	-3.40403	6.64E-04	4.25E-03	6.67365	6.395572	6.579222	7.145099	7.237908	7.272722
AT5G51070	2239.99	-0.654630821	0.09009	-7.26639	3.69E-13	1.03E-11	10.76684	10.77148	10.75304	11.31018	11.45081	11.49752
AT5G03560	308.1343	-0.654411277	0.165401	-3.9565	7.61E-05	6.24E-04	8.047708	7.655595	7.974194	8.734765	8.468027	8.479713
AT5G12390	195.6638	-0.654183492	0.200045	-3.27018	1.07E-03	6.44E-03	7.287806	7.074206	7.332454	7.821505	8.256129	7.613635
AT4G27000	1720.863	-0.653998679	0.119274	-5.48316	4.18E-08	6.19E-07	10.45468	10.22675	10.45172	11.17197	11.04794	10.89525
AT2G45980	1224.574	-0.652559577	0.127215	-5.12958	2.90E-07	3.80E-06	10.05199	9.672499	9.923782	10.50398	10.63478	10.51413
AT4G16760	1144.929	-0.652468592	0.112132	-5.81873	5.93E-09	9.89E-08	9.807772	9.894776	9.680203	10.56974	10.34937	10.43303
AT1G24160	1136.178	-0.652054484	0.123199	-5.2927	1.21E-07	1.68E-06	9.703399	9.924947	9.709271	10.30207	10.59473	10.42125
AT2G37150	169.2117	-0.650920951	0.184338	-3.53113	4.14E-04	2.81E-03	7.083171	7.147962	6.903729	7.864069	7.411902	7.786827
AT5G57660	5880.999	-0.650372843	0.095093	-6.8393	7.96E-12	1.96E-10	12.25842	12.01156	12.19286	12.81633	12.82218	12.79906
AT2G38540	7800.831	-0.650301887	0.135201	-4.8099	1.51E-06	1.75E-05	12.67712	12.35049	12.63837	13.16273	13.42178	13.05657
AT4G21560	262.7017	-0.650194751	0.14517	-4.47886	7.50E-06	7.64E-05	7.740014	7.643877	7.662533	8.371524	8.161812	8.443937
AT4G21450	1107.115	-0.649480092	0.112943	-5.75052	8.90E-09	1.45E-07	9.904261	9.617639	9.710336	10.38844	10.44007	10.38532
AT3G46000	724.4008	-0.648739306	0.130137	-4.98505	6.19E-07	7.67E-06	9.166336	8.95832	9.271898	9.680454	9.841749	9.85184
AT4G30910	248.0944	-0.648036872	0.163473	-3.96418	7.36E-05	6.06E-04	7.762452	7.460079	7.548016	8.422254	8.12479	8.189982
AT2G39000	636.4742	-0.647787211	0.118541	-5.46467	4.64E-08	6.83E-07	8.963396	8.895468	8.992497	9.448398	9.735248	9.624173
AT5G56550	1180.967	-0.647323387	0.16327	-3.96475	7.35E-05	6.05E-04	10.17487	9.667685	9.606581	10.57456	10.47726	10.44342
AT5G01750	2530.478	-0.647098013	0.141378	-4.57707	4.72E-06	4.98E-05	10.98089	10.81654	11.01386	11.3575	11.85289	11.53808
AT2G40840	685.2518	-0.646391124	0.19015	-3.39937	6.75E-04	4.31E-03	8.770241	9.117898	9.232395	9.297401	9.879281	9.89601
AT3G51500	251.0287	-0.645999845	0.158049	-4.08735	4.36E-05	3.77E-04	7.686261	7.428552	7.697339	8.318945	8.35535	8.126918
AT5G11670	881.9399	-0.64503424	0.121317	-5.31692	1.06E-07	1.48E-06	9.550621	9.386372	9.334043	10.08309	9.926657	10.19859
AT4G33480	219.8026	-0.645008848	0.165712	-3.89234	9.93E-05	0.0007869	7.261791	7.382284	7.613262	8.104931	7.966403	8.146128
AT5G42825	532.281	-0.644569343	0.138723	-4.64644	3.38E-06	3.69E-05	8.857944	8.469318	8.736318	9.335713	9.32622	9.381005

AT1G64795	233.7409	-0.644421091	0.182227	-3.53637	4.06E-04	2.76E-03	7.717222	7.34899	7.433975	8.400731	8.060895	8.005951
AT1G55280	122.2664	-0.64419947	0.204257	-3.15386	1.61E-03	9.11E-03	6.712583	6.494586	6.550955	7.162256	7.013254	7.478706
AT2G24240	197.3763	-0.643849983	0.170062	-3.78598	0.000153	0.0011554	7.453261	7.21813	7.111553	8.022777	7.865285	7.873905
AT2G46550	891.5794	-0.643639477	0.108198	-5.94874	2.70E-09	4.75E-08	9.302719	9.50879	9.505869	10.09431	10.06011	10.11331
AT3G20680	275.336	-0.642757895	0.157723	-4.07523	4.60E-05	3.95E-04	7.758737	7.705304	7.772711	8.122569	8.55409	8.489773
AT2G46600	1273.523	-0.64249566	0.144112	-4.45831	8.26E-06	8.32E-05	10.13745	9.669612	10.01283	10.71779	10.58794	10.50175
AT5G40760	192.4047	-0.641814208	0.154396	-4.15694	3.23E-05	2.87E-04	7.175244	7.285044	7.235138	7.810665	7.952384	7.889188
AT5G45490	897.1522	-0.641794033	0.132915	-4.82861	1.37E-06	1.61E-05	9.583549	9.283134	9.464793	10.24685	10.09562	9.940702
AT1G70830	1977.965	-0.641734223	0.179196	-3.5812	3.42E-04	2.37E-03	10.93759	10.48116	10.24817	11.37876	11.28173	11.04604
AT4G36760	832.839	-0.64127782	0.097976	-6.54529	5.94E-11	1.31E-09	9.275675	9.382851	9.376274	10.01861	9.969049	9.982271
AT3G03150	2000.029	-0.641231143	0.165698	-3.86987	0.000109	0.0008526	10.64784	10.4309	10.71107	11.0049	11.59313	11.10368
AT2G26210	441.2639	-0.641160621	0.117414	-5.46067	4.74E-08	6.98E-07	8.474278	8.377954	8.427208	9.066306	9.153175	9.010149
AT5G21940	3413.891	-0.640776648	0.129593	-4.94455	7.63E-07	9.31E-06	11.61744	11.18125	11.29663	12.06338	12.03851	11.97405
AT1G08460	378.4794	-0.64018913	0.147475	-4.34099	1.42E-05	1.37E-04	8.192449	8.042308	8.36091	8.912575	8.949469	8.699362
AT5G63195	2815.66	-0.640126057	0.083999	-7.62063	2.52E-14	7.93E-13	11.09911	11.04977	11.15944	11.76447	11.6797	11.78937
AT1G31820	99.81014	-0.63936383	0.202522	-3.15701	0.001594	0.0090316	6.265936	6.30918	6.324778	7.03764	6.747048	7.018519
AT1G60140	1659.016	-0.639298872	0.103024	-6.20536	5.46E-10	1.06E-08	10.34062	10.29327	10.38389	10.83342	11.02468	11.07974
AT5G22290	277.2445	-0.639153836	0.140453	-4.55066	5.35E-06	5.58E-05	7.85236	7.686387	7.743865	8.505274	8.333883	8.375048
AT5G62200	164.6303	-0.638718473	0.169351	-3.77157	0.000162	0.0012171	6.978444	6.920804	7.130779	7.561902	7.690514	7.720024
AT5G05750	596.7968	-0.638412239	0.136374	-4.68134	2.85E-06	3.17E-05	9.049549	8.661731	8.854751	9.462317	9.561435	9.507706
AT2G45820	1584.103	-0.638231825	0.109423	-5.83268	5.45E-09	9.17E-08	10.39967	10.18356	10.22375	10.81727	11.02468	10.90186
AT5G16880	1663.855	-0.637617281	0.107053	-5.95607	2.58E-09	4.58E-08	10.4932	10.25398	10.27434	11.05168	10.94197	10.96452
AT2G24270	3783.261	-0.637495886	0.096782	-6.58692	4.49E-11	1.01E-09	11.6043	11.42039	11.55596	12.16103	12.26239	12.08605
AT3G55770	2143.987	-0.636888746	0.110409	-5.76843	8.00E-09	1.31E-07	10.8137	10.53977	10.76077	11.36956	11.41931	11.26541
AT1G03080	880.9851	-0.636007168	0.165389	-3.84552	1.20E-04	9.34E-04	9.4191	9.637472	9.188724	10.13832	9.77959	10.25424
AT3G32980	263.2538	-0.634918499	0.193125	-3.28761	1.01E-03	6.10E-03	7.994992	7.47779	7.509364	8.429358	8.428064	8.13335
AT4G29950	1387.603	-0.634789103	0.102477	-6.19443	5.85E-10	1.13E-08	10.18875	10.0382	10.02573	10.76207	10.61934	10.78212
AT2G18280	1820.52	-0.634731738	0.112409	-5.64663	1.64E-08	2.57E-07	10.59151	10.29639	10.52075	11.09029	11.19062	11.06467
AT1G54010	203.25	-0.634202187	0.180532	-3.51297	0.000443	0.0029862	7.448656	7.026869	7.433975	7.864069	7.994038	8.019904
AT1G75540	377.251	-0.632644521	0.151179	-4.18474	2.85E-05	2.58E-04	8.259715	7.959642	8.374416	8.812891	8.839518	8.889946
AT4G38090	275.2394	-0.631557778	0.141149	-4.47442	7.66E-06	7.77E-05	7.827745	7.663355	7.772711	8.498535	8.333883	8.342109
AT3G16190	331.5723	-0.631347304	0.145541	-4.33792	1.44E-05	0.0001386	7.99183	7.881962	8.172762	8.723251	8.72049	8.53905
AT1G05277	134.6724	-0.631271687	0.179668	-3.51355	4.42E-04	2.98E-03	6.742991	6.697159	6.729716	7.228912	7.510873	7.363061
AT5G43440	418.2542	-0.630483496	0.154291	-4.08632	4.38E-05	3.78E-04	8.275402	8.257524	8.522484	8.751866	9.084138	9.124335

AT1G59900	2089.49	-0.629940936	0.115481	-5.45493	4.90E-08	7.20E-07	10.68747	10.55705	10.77459	11.3312	11.44331	11.1512
AT3G11560	1765.374	-0.629606578	0.083915	-7.50291	6.24E-14	1.89E-12	10.44257	10.47676	10.3879	11.03431	11.06596	11.10368
AT5G04325	442.3034	-0.629068051	0.157221	-4.00117	6.30E-05	5.26E-04	8.555759	8.415197	8.314022	9.066306	9.30982	8.823881
AT5G04315	442.3034	-0.629068051	0.157221	-4.00117	6.30E-05	0.0005257	8.555759	8.415197	8.314022	9.066306	9.30982	8.823881
AT3G52850	1419.201	-0.629007831	0.100211	-6.27686	3.45E-10	6.86E-09	10.12235	10.19699	10.04105	10.67061	10.74053	10.84421
AT4G14615	346.8726	-0.62878038	0.128271	-4.90195	9.49E-07	1.14E-05	8.121893	8.068843	8.066944	8.682211	8.847114	8.646381
AT2G21240	569.7115	-0.628664647	0.118507	-5.30488	1.13E-07	1.57E-06	8.905765	8.684788	8.821612	9.47953	9.363777	9.467545
AT1G63800	779.309	-0.628359476	0.128672	-4.88342	1.04E-06	1.24E-05	9.233464	9.194872	9.328503	9.692313	10.07959	9.878887
AT1G79700	790.4103	-0.627209938	0.118426	-5.29621	1.18E-07	1.65E-06	9.320471	9.148629	9.347802	9.87511	10.04036	9.810295
AT4G13270	204.8988	-0.627047883	0.149448	-4.19576	2.72E-05	0.0002464	7.397007	7.290065	7.31014	7.984706	7.980286	7.941438
AT5G63190	4347.639	-0.626675511	0.077328	-8.10417	5.31E-16	1.95E-14	11.73036	11.69503	11.78761	12.37757	12.32016	12.40093
AT3G53990	1305.47	-0.62628793	0.120683	-5.18952	2.11E-07	2.82E-06	10.00022	9.905449	10.08607	10.48535	10.81602	10.58153
AT1G78490	237.5436	-0.625808065	0.143386	-4.3645	1.27E-05	1.24E-04	7.476068	7.554903	7.613262	8.232242	8.12479	8.177588
AT2G38230	926.5171	-0.624546396	0.169293	-3.68915	0.000225	0.0016402	9.310354	9.679212	9.489827	9.958499	10.45754	9.951662
AT3G48115	249.9889	-0.624342138	0.155121	-4.02487	5.70E-05	4.80E-04	7.784546	7.451141	7.60412	8.25641	8.278775	8.226539
AT1G80180	2155.886	-0.623792419	0.125104	-4.9862	6.16E-07	7.63E-06	10.85126	10.53871	10.76488	11.29471	11.51302	11.24774
AT2G26690	922.9352	-0.623650137	0.137961	-4.52048	6.17E-06	6.37E-05	9.311622	9.679212	9.489827	9.960952	10.20312	10.22141
AT1G09910	401.6344	-0.62348922	0.118347	-5.26832	1.38E-07	1.90E-06	8.254447	8.297964	8.358194	8.922678	8.970488	8.908815
AT2G47850	141.834	-0.622921799	0.177017	-3.519	0.000433	0.0029269	6.830546	6.778008	6.835058	7.482168	7.26084	7.547747
AT1G69510	878.6051	-0.622423191	0.103628	-6.00632	1.90E-09	3.44E-08	9.494759	9.32532	9.472349	10.05351	10.10516	10.02267
AT2G16780	138.2834	-0.622243574	0.192048	-3.24003	1.20E-03	7.07E-03	6.858589	6.635377	6.811422	7.625138	7.26084	7.295843
AT3G13710	153.104	-0.621408431	0.172791	-3.5963	0.000323	0.0022538	7.034833	6.854565	6.850603	7.46844	7.621318	7.557346
AT4G23670	6509.965	-0.621157348	0.108203	-5.74064	9.43E-09	1.53E-07	12.36979	12.28504	12.30748	12.84417	13.13304	12.84876
AT2G37678	187.3236	-0.620733783	0.162707	-3.81504	1.36E-04	0.0010413	7.282641	7.212852	7.11799	7.685719	7.938228	7.881567
AT1G02816	250.9086	-0.620448218	0.173757	-3.57077	0.000356	0.0024575	7.525009	7.575611	7.748022	8.068994	8.544777	8.126918
AT1G78080	852.2236	-0.619725025	0.183157	-3.38358	7.15E-04	4.53E-03	9.658243	9.089381	9.353268	10.29625	9.795383	9.916666
AT1G60430	155.0215	-0.619140241	0.188871	-3.27812	1.05E-03	6.28E-03	7.089101	6.834086	6.858314	7.323456	7.740317	7.613635
AT4G05000	150.8273	-0.618754847	0.190512	-3.24786	1.16E-03	6.90E-03	7.077217	6.658856	6.918555	7.612711	7.529878	7.437718
AT3G58530	183.5415	-0.618304046	0.172696	-3.58031	3.43E-04	2.38E-03	7.308286	7.050731	7.149752	7.685719	7.994038	7.737017
AT3G48530	1261.398	-0.616030044	0.154994	-3.97454	7.05E-05	0.0005824	10.06934	9.865644	9.923782	10.2408	10.63916	10.80648
AT5G18640	533.4977	-0.616021496	0.119484	-5.15566	2.53E-07	3.34E-06	8.792217	8.644192	8.710993	9.213474	9.425968	9.372874
AT1G11910	5225.515	-0.615771454	0.10765	-5.72011	1.06E-08	1.71E-07	11.92609	12.06618	12.02875	12.45445	12.6629	12.75308
AT5G27710	147.9963	-0.615239406	0.178568	-3.44541	5.70E-04	3.73E-03	6.823449	6.813311	6.954967	7.45458	7.673524	7.363061
AT3G50660	193.8965	-0.615220683	0.164639	-3.7368	1.86E-04	0.0013798	7.411277	7.125667	7.223245	7.832265	7.966403	7.858459

AT1G21600	812.5109	-0.61497343	0.112283	-5.47701	4.33E-08	6.40E-07	9.392972	9.29941	9.270454	9.895773	10.08602	9.847935
AT1G27300	349.6116	-0.614796398	0.135106	-4.55048	5.35E-06	5.58E-05	8.041606	8.060052	8.224388	8.845121	8.661394	8.677522
AT1G03140	736.3279	-0.614590618	0.105187	-5.84283	5.13E-09	8.64E-08	9.211886	9.138922	9.191779	9.764306	9.908619	9.734054
AT1G11480	795.0085	-0.613799002	0.113203	-5.42211	5.89E-08	8.55E-07	9.300165	9.345959	9.229425	10.02566	9.930238	9.775765
AT5G42520	452.1947	-0.613392432	0.118565	-5.17349	2.30E-07	3.05E-06	8.472011	8.467103	8.505399	9.209354	9.064722	9.020596
AT3G13700	153.082	-0.613361126	0.173575	-3.53369	4.10E-04	2.79E-03	7.047071	6.854565	6.850603	7.46844	7.621318	7.547747
AT5G58740	317.0556	-0.61331502	0.142835	-4.29387	1.76E-05	1.66E-04	7.959825	7.841495	8.08677	8.538503	8.72049	8.509687
AT1G53400	484.2264	-0.612911166	0.123651	-4.9568	7.17E-07	8.79E-06	8.670942	8.437994	8.620949	9.150389	9.236518	9.208984
AT5G58640	186.7066	-0.612826321	0.167798	-3.65217	2.60E-04	1.86E-03	7.186351	7.120039	7.31014	7.63746	7.923931	7.911813
AT3G63000	421.0194	-0.612070017	0.1263	-4.84616	1.26E-06	1.48E-05	8.451442	8.272822	8.416803	8.957493	8.935284	9.088372
AT2G44410	586.2009	-0.611688857	0.134954	-4.53258	5.83E-06	6.04E-05	8.905765	8.696181	8.951635	9.635091	9.415787	9.356473
AT3G26280	1008.063	-0.611360519	0.182681	-3.34659	8.18E-04	0.0050901	9.713991	9.54809	9.62929	9.761494	10.42238	10.4855
AT4G26130	353.5708	-0.610737954	0.150863	-4.0483	5.16E-05	4.38E-04	8.121893	7.937414	8.294263	8.639969	8.877107	8.716599
AT2G41430	7796.318	-0.610697719	0.142324	-4.2909	1.78E-05	0.0001677	12.7416	12.35484	12.63374	12.97463	13.37196	13.24247
AT1G04400	2267.072	-0.610369191	0.108198	-5.64125	1.69E-08	2.64E-07	10.7543	10.8377	10.83004	11.27217	11.40007	11.57694
AT3G52240	890.7351	-0.609935807	0.119087	-5.12178	3.03E-07	3.94E-06	9.56771	9.417677	9.390966	10.22864	9.96207	10.02094
AT1G56280	6077.815	-0.609812413	0.142622	-4.27572	1.91E-05	0.0001787	12.48713	11.95479	12.19019	12.9365	12.82603	12.77072
AT5G22000	1089.903	-0.60965278	0.106053	-5.74858	9.00E-09	1.46E-07	9.879668	9.64435	9.71989	10.37195	10.38894	10.33738
AT3G26230	223.4831	-0.60959235	0.176771	-3.44849	5.64E-04	3.69E-03	7.580786	7.344171	7.474672	8.349224	7.865285	7.998924
AT3G59820	182.9723	-0.60925915	0.17364	-3.50874	4.50E-04	3.03E-03	7.135683	7.285044	7.111553	7.561902	7.909492	7.904311
AT1G07230	578.0039	-0.608816067	0.12564	-4.84572	1.26E-06	1.48E-05	8.812072	8.793086	8.909583	9.281787	9.465988	9.589508
AT2G46690	181.9123	-0.608796614	0.188287	-3.23334	0.001224	0.0072126	7.387414	6.933695	7.156022	7.625138	7.865285	7.873905
AT3G14050	488.6874	-0.608720973	0.131323	-4.63529	3.56E-06	3.87E-05	8.757265	8.559403	8.455441	9.158962	9.201388	9.268632
AT3G13224	260.0947	-0.608050244	0.162293	-3.74663	1.79E-04	1.33E-03	7.732457	7.799859	7.504459	8.464361	8.289966	8.146128
AT4G25180	163.3737	-0.607471605	0.18778	-3.23502	1.22E-03	7.18E-03	7.077217	6.799293	7.162264	7.810665	7.491614	7.585765
AT1G28580	282.6212	-0.606882031	0.160731	-3.77576	1.60E-04	1.20E-03	7.900364	7.810381	7.697339	8.639969	8.376502	8.226539
AT4G16710	185.5475	-0.605832909	0.187387	-3.23306	1.22E-03	7.22E-03	7.343439	7.300054	6.918555	7.649677	7.909492	7.889188
AT1G80780	788.7936	-0.605496664	0.139834	-4.33011	1.49E-05	1.43E-04	9.433154	9.049966	9.343688	9.808565	10.03705	9.845978
AT5G39590	1258.424	-0.605222043	0.090097	-6.71741	1.85E-11	4.33E-10	9.963565	9.992727	9.927452	10.51236	10.63478	10.56497
AT5G43460	756.7952	-0.604376084	0.135572	-4.45796	8.27E-06	8.33E-05	9.407282	9.02609	9.226449	9.94617	9.759603	9.804262
AT5G67570	351.9504	-0.603855949	0.148554	-4.0649	4.81E-05	0.0004115	8.035477	8.322675	8.002302	8.774357	8.626506	8.799822
AT1G62040	409.3402	-0.603167954	0.13466	-4.47919	7.49E-06	7.63E-05	8.418853	8.159423	8.44011	8.962399	8.928139	8.967588
AT4G21620	377.6961	-0.60291878	0.166532	-3.62045	0.000294	0.0020738	8.437566	8.153926	8.043464	8.927704	8.970488	8.600693
AT3G15970	299.7604	-0.602807911	0.134825	-4.47103	7.78E-06	7.88E-05	7.85236	7.858494	7.984799	8.498535	8.428064	8.577295

AT3G07780	766.1866	-0.60275102	0.129748	-4.64555	3.39E-06	3.71E-05	9.340495	9.338708	9.037336	9.941208	9.860637	9.763378
AT1G77710	745.6979	-0.602609129	0.142888	-4.21735	2.47E-05	2.26E-04	9.200975	8.983333	9.402877	9.704076	9.940927	9.800225
AT5G18110	1160.536	-0.602453371	0.100885	-5.97166	2.35E-09	4.18E-08	9.90762	9.722567	9.9025	10.46129	10.4699	10.42911
AT1G16890	1354.979	-0.602363113	0.106781	-5.6411	1.69E-08	2.64E-07	10.10196	10.02772	10.07371	10.55521	10.83142	10.6335
AT4G21510	160.4851	-0.602232344	0.168043	-3.58379	3.39E-04	2.35E-03	7.047071	6.881427	7.058994	7.661791	7.585434	7.576354
AT2G45560	182.1811	-0.601767637	0.175068	-3.43734	0.000587	0.0038246	7.106746	7.097304	7.332454	7.625138	7.772588	7.948751
AT4G16500	528.118	-0.601542746	0.153024	-3.93103	8.46E-05	0.0006856	8.792217	8.523615	8.787693	9.13309	9.55681	9.245068
AT5G58960	328.6893	-0.601290862	0.151775	-3.96172	7.44E-05	6.12E-04	7.900364	8.178502	7.977737	8.505274	8.816485	8.582005
AT5G59950	523.4607	-0.600873744	0.1098	-5.47245	4.44E-08	6.55E-07	8.659031	8.684788	8.752958	9.324326	9.337051	9.256898
AT3G02455	173.1748	-0.60084969	0.169614	-3.54246	3.96E-04	0.0027033	7.077217	7.23385	7.004448	7.842944	7.621318	7.676644
AT3G45770	706.7299	-0.600630557	0.120365	-4.99008	6.04E-07	7.50E-06	9.138014	9.012485	9.233877	9.619647	9.853112	9.736168
AT4G33670	747.8253	-0.599596191	0.140501	-4.26756	1.98E-05	0.0001842	9.234802	9.018548	9.36143	9.644278	9.969049	9.832206
AT1G07570	226.5068	-0.59875373	0.170054	-3.52095	0.00043	0.0029073	7.682343	7.31491	7.444257	8.041442	8.256129	8.005951
AT1G67530	258.2768	-0.598117794	0.142089	-4.20947	2.56E-05	0.000233	7.751277	7.663355	7.622346	8.264377	8.397349	8.208377
AT3G62130	340.3569	-0.596226824	0.12492	-4.77287	1.82E-06	2.07E-05	8.139144	8.07758	8.033283	8.717459	8.652751	8.681917
AT5G26751	816.1398	-0.595713323	0.107446	-5.54429	2.95E-08	4.47E-07	9.417922	9.242271	9.365493	9.864667	9.969049	9.99292
AT1G22930	1510.606	-0.595296618	0.135272	-4.40074	1.08E-05	0.0001064	10.10562	10.47014	10.07949	10.79407	10.7688	10.92707
AT4G15780	870.16	-0.593153583	0.103165	-5.74956	8.95E-09	1.46E-07	9.500333	9.343546	9.461	10.06268	10.06988	9.971541
AT2G34420	124319.1	-0.591636239	0.165492	-3.57502	0.00035	0.0024217	16.52764	16.3142	16.87379	17.16545	17.41588	16.96587
AT2G08665	124319.2	-0.591633785	0.165491	-3.57502	3.50E-04	0.0024217	16.52764	16.31421	16.87379	17.16545	17.41588	16.96587
AT4G12300	160.277	-0.59147924	0.182593	-3.23934	0.001198	0.0070854	7.158422	6.914316	6.918555	7.788738	7.491614	7.5087
AT3G58170	448.8162	-0.591450298	0.129552	-4.56535	4.99E-06	5.24E-05	8.338991	8.561481	8.532156	9.043339	9.189485	9.010149
AT5G24670	580.8253	-0.59104153	0.112464	-5.25538	1.48E-07	2.02E-06	8.893958	8.857075	8.821612	9.559406	9.347801	9.436673
AT3G14100	1299.309	-0.590924199	0.129227	-4.57277	4.81E-06	5.07E-05	10.2336	9.887341	9.897831	10.69144	10.54188	10.60023
AT5G63880	170.6299	-0.590824602	0.168526	-3.50583	0.000455	0.0030635	7.034833	6.990318	7.235138	7.661791	7.756543	7.658921
AT1G49820	322.7905	-0.590390887	0.148204	-3.98364	6.79E-05	0.0005626	8.098567	7.792802	8.116007	8.64608	8.581674	8.591379
AT5G63200	255.5831	-0.590326017	0.142194	-4.15155	3.30E-05	0.000293	7.713388	7.587895	7.722903	8.356696	8.197907	8.250405
AT1G65370	482.0677	-0.590318397	0.127295	-4.63741	3.53E-06	3.84E-05	8.708021	8.515063	8.541764	9.229841	9.04504	9.259841
AT1G02860	288.3728	-0.590208876	0.145301	-4.06198	4.87E-05	4.16E-04	7.879986	7.831198	7.836554	8.25641	8.468027	8.5867
AT5G03200	125.1455	-0.590013247	0.18199	-3.24201	1.19E-03	7.03E-03	6.727867	6.611509	6.597764	7.212535	7.283413	7.249225
AT4G03030	543.2363	-0.590007876	0.155304	-3.79905	1.45E-04	1.10E-03	8.937336	8.663666	8.638965	9.316684	9.57979	9.149938
AT3G48000	1358.927	-0.589824133	0.11064	-5.33102	9.77E-08	1.37E-06	9.952223	10.16318	10.11693	10.58255	10.79653	10.64368
AT1G03380	621.5476	-0.589414236	0.129171	-4.56305	5.04E-06	5.29E-05	8.920806	9.041059	8.907726	9.591425	9.337051	9.688919
AT5G62270	350.5776	-0.589202153	0.165008	-3.57075	0.000356	0.0024575	8.068868	8.197331	8.109561	8.378881	8.877107	8.870827

AT5G03340	520.8211	-0.589072659	0.119236	-4.9404	7.80E-07	9.50E-06	8.618605	8.768114	8.702452	9.361938	9.337051	9.181316
AT4G39100	659.1875	-0.588969841	0.122999	-4.7884	1.68E-06	1.93E-05	9.136583	8.858766	9.105328	9.585078	9.672496	9.642326
AT3G29240	3504.671	-0.588930882	0.139831	-4.21173	2.53E-05	2.31E-04	11.61076	11.3008	11.40819	11.80599	12.24381	12.04826
AT5G17900	408.8828	-0.588896652	0.161663	-3.64275	0.00027	0.0019216	8.383099	8.553151	8.076891	8.996279	8.800923	9.030967
AT4G36195	542.7012	-0.588783948	0.130469	-4.51282	6.40E-06	6.59E-05	8.630852	8.784217	8.856677	9.209354	9.358471	9.477691
AT5G30495	147.4787	-0.588444723	0.181653	-3.23939	0.001198	0.0070854	6.90639	6.799293	6.93323	7.26112	7.638931	7.528356
AT1G73380	251.2983	-0.588210748	0.176476	-3.3331	8.59E-04	5.29E-03	7.824194	7.529654	7.581008	8.498535	8.034523	8.165086
AT4G38495	177.358	-0.588167084	0.179401	-3.2785	0.001044	0.0062758	7.118391	6.946471	7.348966	7.799743	7.772588	7.66781
AT5G05140	343.0101	-0.588143472	0.130818	-4.49589	6.93E-06	7.11E-05	8.214317	8.05711	8.016154	8.694056	8.703851	8.681917
AT3G50430	187.4045	-0.587954806	0.161096	-3.64971	0.000263	0.001876	7.277457	7.254546	7.156022	7.685719	7.850244	7.926702
AT5G58575	196.6948	-0.587156037	0.186619	-3.14628	0.001654	0.0093266	7.457851	7.032872	7.348966	7.905412	8.060895	7.711452
AT5G20620	638.6273	-0.586855538	0.129462	-4.53302	5.81E-06	6.03E-05	8.794034	9.086498	9.084114	9.50665	9.611358	9.640069
AT3G53030	330.1283	-0.586206709	0.129898	-4.51282	6.40E-06	6.59E-05	8.089721	8.021327	8.029874	8.545058	8.626506	8.729392
AT1G74900	263.6793	-0.585353023	0.141015	-4.15101	3.31E-05	2.94E-04	7.817066	7.70154	7.649262	8.364129	8.233121	8.336545
AT4G02510	5200.117	-0.584961938	0.172351	-3.39401	6.89E-04	0.0043842	12.10061	12.1636	11.74744	12.92304	12.31332	12.5345
AT1G22040	191.4982	-0.584872349	0.163613	-3.57472	0.000351	0.0024237	7.323457	7.169918	7.304507	7.777648	7.756543	8.005951
AT5G52240	863.5776	-0.583925974	0.123517	-4.7275	2.27E-06	2.56E-05	9.483548	9.319193	9.477364	9.877709	10.19719	9.973335
AT1G75370	739.9591	-0.583580717	0.121767	-4.79261	1.65E-06	1.89E-05	9.064675	9.23708	9.315959	9.683428	9.814884	9.886522
AT5G46250	935.8136	-0.582887332	0.117583	-4.95724	7.15E-07	8.78E-06	9.699528	9.534403	9.393622	10.09431	10.13339	10.17852
AT3G12120	4154.156	-0.582645813	0.151739	-3.8398	1.23E-04	9.52E-04	11.89786	11.42582	11.71966	12.4527	12.32016	12.06054
AT1G32090	197.4161	-0.582146502	0.159929	-3.64002	2.73E-04	1.94E-03	7.240636	7.285044	7.397399	7.755209	7.923931	7.998924
AT1G14400	1529.111	-0.580497876	0.13564	-4.27969	1.87E-05	0.0001758	10.33502	10.02245	10.38055	10.75925	11.00784	10.75109
AT5G52920	3423.033	-0.580086066	0.127403	-4.55314	5.29E-06	5.52E-05	11.45589	11.35242	11.44321	11.75531	12.20221	12.02466
AT1G64950	312.1073	-0.580034993	0.162711	-3.56482	3.64E-04	2.51E-03	7.780888	8.151169	7.934627	8.364129	8.695459	8.591379
AT3G51840	3496.349	-0.579445019	0.076511	-7.57336	3.64E-14	1.12E-12	11.40806	11.46075	11.48521	12.04557	12.06073	11.99495
AT4G26620	380.9206	-0.579262979	0.126674	-4.57288	4.81E-06	5.07E-05	8.326495	8.205326	8.23331	8.947631	8.77726	8.78764
AT4G14030	1380.036	-0.578905813	0.126894	-4.56212	5.06E-06	5.31E-05	10.18321	10.05378	10.08278	10.49554	10.90423	10.65825
AT5G56795	233.5833	-0.578568231	0.175617	-3.2945	0.000986	0.0059712	7.642574	7.693983	7.264448	8.215903	8.185975	8.012944
AT5G51400	624.5591	-0.578547605	0.146115	-3.95954	7.51E-05	0.0006165	9.138014	8.731673	8.992497	9.503287	9.697925	9.449617
AT1G60870	549.8613	-0.578235493	0.131504	-4.39711	1.10E-05	0.000108	8.8666	8.665599	8.801756	9.246025	9.547515	9.300417
AT2G19790	503.4669	-0.576828888	0.146449	-3.93877	8.19E-05	0.0006656	8.762841	8.504302	8.683046	9.029381	9.436078	9.236131
AT3G24315	274.0057	-0.576576235	0.158564	-3.63623	0.000277	0.0019654	7.806308	7.604113	7.91258	8.191041	8.487601	8.407252
AT4G32250	361.7722	-0.576053595	0.127197	-4.52882	5.93E-06	6.14E-05	8.104434	8.175792	8.271346	8.807449	8.695459	8.783557
AT1G29120	240.8199	-0.575984496	0.183136	-3.14512	1.66E-03	9.36E-03	7.516233	7.464527	7.772711	7.874516	8.40766	8.22051

AT1G64040	817.8547	-0.574575394	0.106119	-5.41444	6.15E-08	8.91E-07	9.463145	9.324097	9.28483	9.992469	9.908619	9.911062
AT1G80440	2639.645	-0.574179623	0.12792	-4.48858	7.17E-06	7.33E-05	11.13466	10.85304	11.13346	11.6237	11.78233	11.46219
AT1G75380	4484.673	-0.574051638	0.115321	-4.97786	6.43E-07	7.95E-06	11.70572	11.78313	11.93821	12.21546	12.50559	12.4394
AT2G07180	802.2352	-0.573783195	0.11247	-5.10164	3.37E-07	4.34E-06	9.384558	9.301898	9.301893	10.02096	9.93381	9.767519
AT3G16740	233.7948	-0.573692874	0.157801	-3.63554	2.77E-04	1.97E-03	7.626355	7.529654	7.489642	7.95548	8.333883	8.107448
AT1G21450	730.8746	-0.573425112	0.103219	-5.55543	2.77E-08	4.20E-07	9.203711	9.226643	9.160938	9.854148	9.767632	9.701959
AT1G45976	411.1887	-0.572371098	0.16598	-3.44844	5.64E-04	3.69E-03	8.532012	8.226433	8.314022	9.128733	9.025087	8.659809
AT2G46530	413.3013	-0.571190264	0.161555	-3.53558	4.07E-04	2.77E-03	8.458331	8.249814	8.40369	8.633832	9.147034	9.041265
AT1G10660	338.8639	-0.570586286	0.133354	-4.27875	1.88E-05	1.76E-04	8.167445	8.04528	8.050212	8.55808	8.793078	8.650871
AT1G08550	455.9522	-0.570463572	0.15779	-3.61534	0.0003	0.0021105	8.451442	8.559403	8.524908	8.823715	9.379578	9.041265
AT2G39950	368.8616	-0.568413276	0.12683	-4.4817	7.41E-06	7.55E-05	8.150531	8.170356	8.316823	8.790998	8.831881	8.746276
AT4G13830	911.996	-0.567218391	0.136431	-4.15754	3.22E-05	2.86E-04	9.539837	9.296918	9.691037	9.973155	10.19719	10.10188
AT1G03970	285.643	-0.566512058	0.130963	-4.32575	1.52E-05	1.46E-04	7.820635	7.85511	7.871262	8.457428	8.40766	8.396597
AT5G13800	844.4564	-0.56606987	0.097679	-5.79521	6.82E-09	1.13E-07	9.344219	9.447194	9.436744	9.960952	10.02035	9.960732
AT5G04760	213.7148	-0.565299535	0.178371	-3.16924	1.53E-03	8.72E-03	7.502969	7.563222	7.217262	8.087074	7.756543	8.13335
AT3G59950	629.8896	-0.565134367	0.141267	-4.00047	6.32E-05	5.27E-04	9.19136	8.953581	8.785673	9.455375	9.597913	9.619599
AT2G02070	572.6877	-0.565051837	0.122948	-4.59587	4.31E-06	4.58E-05	8.824567	8.729827	8.988989	9.350757	9.436078	9.472627
AT3G23050	4101.112	-0.563763723	0.10961	-5.14335	2.70E-07	3.55E-06	11.74733	11.58187	11.73336	12.13302	12.41777	12.21104
AT1G29390	410.5575	-0.562919757	0.16465	-3.41889	6.29E-04	0.0040575	8.241194	8.332442	8.520055	8.627669	9.109626	9.048089
AT2G42620	638.9057	-0.562833304	0.125828	-4.47302	7.71E-06	7.81E-05	9.114948	9.069078	8.82555	9.503287	9.633492	9.596508
AT5G44250	676.4287	-0.561910493	0.131987	-4.25732	2.07E-05	1.92E-04	9.242804	8.892169	9.124637	9.650371	9.584342	9.738279
AT3G49800	520.9517	-0.561815478	0.124457	-4.51413	6.36E-06	6.56E-05	8.792217	8.565627	8.779595	9.205221	9.337051	9.306122
AT4G01410	239.6236	-0.561558713	0.177146	-3.17003	1.52E-03	8.70E-03	7.686261	7.309975	7.752166	8.288016	8.161812	8.04741
AT1G69690	322.9586	-0.56126235	0.134556	-4.17122	3.03E-05	2.72E-04	7.998147	7.953326	8.125623	8.498535	8.669986	8.614552
AT5G49650	659.4372	-0.561158596	0.108938	-5.15117	2.59E-07	3.42E-06	8.976251	9.080714	9.113405	9.546397	9.633492	9.682354
AT1G26270	604.327	-0.558457855	0.136984	-4.07682	4.57E-05	0.0003926	9.097404	8.96147	8.710993	9.451891	9.470914	9.563547
AT3G54850	347.5793	-0.55819155	0.150673	-3.70464	2.12E-04	1.55E-03	8.313891	8.071761	8.005778	8.839799	8.563343	8.681917
AT4G16060	183.1304	-0.557912405	0.175307	-3.18249	1.46E-03	0.0083918	7.197372	7.080015	7.365292	7.673804	7.690514	7.948751
AT5G03230	1046.229	-0.557404543	0.108504	-5.1372	2.79E-07	3.66E-06	9.844168	9.640424	9.682376	10.20813	10.28371	10.36086
AT3G01910	1028.313	-0.556711902	0.135384	-4.1121	3.92E-05	3.42E-04	9.715908	9.687798	9.683462	10.03733	10.50873	10.20471
AT4G17140	1251.421	-0.55670025	0.163612	-3.40256	6.68E-04	4.27E-03	9.830959	10.2744	9.783025	10.64185	10.32237	10.6493
AT1G26920	776.8844	-0.556600444	0.170401	-3.26641	0.001089	0.0065164	9.310354	9.112239	9.426408	9.943691	10.08602	9.490273
AT1G64230	3349.034	-0.556550824	0.136713	-4.07095	4.68E-05	4.02E-04	11.53377	11.12581	11.50591	11.90232	12.10417	11.87726
AT1G05840	656.3938	-0.555695505	0.113001	-4.9176	8.76E-07	1.06E-05	9.135151	9.015519	9.01509	9.585078	9.524011	9.719165

AT4G03520	1693.107	-0.555310061	0.147431	-3.76657	1.66E-04	0.0012392	10.2575	10.25975	10.68854	10.85469	11.10767	10.96992
AT1G74840	761.0835	-0.555098392	0.151461	-3.66495	2.47E-04	1.79E-03	9.528972	9.110821	9.106947	9.775499	9.951537	9.752973
AT1G65720	732.1748	-0.554293059	0.151678	-3.65441	0.000258	0.0018481	9.340495	8.9151	9.32989	9.644278	9.897687	9.76545
AT5G12250	2444.846	-0.554001863	0.151307	-3.66145	2.51E-04	0.0018066	10.71744	10.91733	11.17032	11.27611	11.66581	11.56212
AT2G45200	322.5995	-0.553863079	0.136328	-4.06272	4.85E-05	0.0004148	8.038544	7.905054	8.141508	8.538503	8.61765	8.614552
AT4G34250	638.4181	-0.553281954	0.133037	-4.15887	3.20E-05	2.85E-04	8.930747	8.937672	9.153124	9.610301	9.70212	9.39713
AT5G03740	340.077	-0.55265402	0.155779	-3.54768	0.000389	0.0026589	8.303727	8.018304	7.977737	8.511981	8.687018	8.78764
AT5G65760	376.0235	-0.552345523	0.131387	-4.20396	2.62E-05	0.0002383	8.262341	8.14564	8.339035	8.705805	8.906489	8.815906
AT1G28150	350.8365	-0.552152689	0.142394	-3.87763	0.000105	0.0008291	8.068868	8.033353	8.336277	8.688146	8.736939	8.708006
AT1G28200	264.1911	-0.552106641	0.169401	-3.25916	1.12E-03	0.0066635	7.717222	7.499627	7.970641	8.25641	8.376502	8.279694
AT4G28300	479.4573	-0.552061307	0.130375	-4.23442	2.29E-05	2.11E-04	8.71187	8.449259	8.627731	9.19692	9.224903	9.058266
AT5G26740	425.2837	-0.552035426	0.135605	-4.07091	4.68E-05	0.0004019	8.579122	8.332442	8.358194	9.061741	9.025087	8.874671
AT3G52072	213.6201	-0.55112386	0.156098	-3.53063	4.15E-04	2.81E-03	7.425408	7.339335	7.543241	7.874516	8.086794	8.02683
AT1G32150	154.0189	-0.550619004	0.169468	-3.2491	1.16E-03	6.87E-03	7.047071	6.977926	6.873611	7.561902	7.548635	7.478706
AT4G26400	684.7931	-0.550264913	0.119904	-4.58922	4.45E-06	4.72E-05	9.241473	9.003343	9.093944	9.750192	9.689499	9.575405
AT3G42170	442.5754	-0.549905865	0.141761	-3.8791	1.05E-04	8.26E-04	8.399895	8.410594	8.629985	8.942675	9.230722	8.949478
AT1G16840	376.5856	-0.549793308	0.173135	-3.17552	1.50E-03	8.56E-03	8.303727	7.975313	8.447796	8.615264	8.808725	8.989026
AT3G15353	6413.371	-0.549662933	0.14379	-3.82268	1.32E-04	1.02E-03	12.51382	12.14051	12.34466	12.68449	13.11546	12.86576
AT3G30390	1656.685	-0.549522582	0.0939	-5.85222	4.85E-09	8.19E-08	10.46041	10.28135	10.42989	10.93923	10.93307	10.96182
AT3G51830	331.9261	-0.549347743	0.144115	-3.81187	1.38E-04	1.05E-03	8.136283	8.05711	8.033283	8.429358	8.652751	8.783557
AT5G49640	319.7849	-0.549120346	0.149783	-3.66612	0.000246	0.0017785	8.164639	7.895203	8.002302	8.676251	8.386963	8.641876
AT4G01120	460.3963	-0.548993004	0.164554	-3.33625	8.49E-04	5.24E-03	8.768395	8.337301	8.483132	8.912575	9.298783	9.075069
AT2G37410	891.7284	-0.548706397	0.110015	-4.98758	6.11E-07	7.59E-06	9.53224	9.419969	9.535025	10.07179	10.15803	9.922248
AT3G60600	1135.047	-0.547665347	0.127692	-4.28895	1.80E-05	1.69E-04	9.980412	9.64533	9.891269	10.49385	10.42238	10.27766
AT3G59940	5525.225	-0.547210854	0.112685	-4.85609	1.20E-06	1.41E-05	12.23555	11.9666	12.17233	12.62657	12.8159	12.594
AT3G56130	876.7812	-0.547150926	0.129901	-4.21206	2.53E-05	2.31E-04	9.492524	9.333854	9.585833	9.830195	10.12716	10.10842
AT1G75220	775.6365	-0.546696035	0.114284	-4.78366	1.72E-06	1.97E-05	9.360245	9.365118	9.164052	9.764306	9.868124	9.9148
AT3G09250	265.1701	-0.545492951	0.152024	-3.5882	0.000333	0.0023143	7.670527	7.888597	7.697339	8.174225	8.301072	8.428329
AT2G44140	1228.155	-0.545464726	0.12006	-4.54325	5.54E-06	5.76E-05	10.12884	9.809055	9.926535	10.46303	10.60372	10.46907
AT5G44580	640.5911	-0.54545439	0.149663	-3.64455	2.68E-04	1.91E-03	9.14799	8.794854	9.10046	9.365646	9.642251	9.697625
AT2G38000	311.8498	-0.545176974	0.130729	-4.17027	3.04E-05	0.0002725	8.00757	7.901778	8.053574	8.538503	8.525969	8.548707
AT3G02520	567.2046	-0.545018499	0.127684	-4.26849	1.97E-05	1.84E-04	8.792217	9.00487	8.727926	9.354494	9.490449	9.353722
AT5G11390	301.5895	-0.545000624	0.158548	-3.43745	5.87E-04	3.82E-03	8.062854	7.975313	7.768625	8.326574	8.45814	8.664258
AT4G15540	314.6022	-0.544150599	0.161268	-3.37419	7.40E-04	4.67E-03	7.988661	7.959642	8.070268	8.538503	8.267496	8.78764

AT5G24610	371.0593	-0.543179474	0.169102	-3.21215	1.32E-03	7.70E-03	8.329003	8.14564	8.239227	9.075391	8.477847	8.746276
AT1G80420	506.7603	-0.543178111	0.122796	-4.4234	9.72E-06	9.66E-05	8.729061	8.630402	8.691703	9.128733	9.395208	9.178208
AT1G55520	281.1301	-0.54308543	0.145975	-3.7204	0.000199	0.0014641	7.896987	7.77142	7.84434	8.199376	8.487601	8.464489
AT3G05120	463.9913	-0.542393472	0.123529	-4.3908	1.13E-05	1.11E-04	8.659031	8.484728	8.539368	9.015287	9.109626	9.193678
AT2G19270	368.2897	-0.542361243	0.137206	-3.9529	7.72E-05	6.32E-04	8.206155	8.134517	8.325192	8.717459	8.935284	8.673114
AT5G12120	966.212	-0.542012353	0.096569	-5.61267	1.99E-08	3.08E-07	9.608535	9.653145	9.601996	10.14049	10.10198	10.24388
AT1G06890	484.9263	-0.541953447	0.126523	-4.28344	1.84E-05	0.0001731	8.70223	8.571825	8.595803	9.316684	9.134672	9.054882
AT3G17820	462.2631	-0.540299776	0.118265	-4.56854	4.91E-06	5.16E-05	8.612442	8.451502	8.602704	9.084419	9.128451	9.094978
AT4G25620	423.5897	-0.540070928	0.136838	-3.9468	7.92E-05	0.0006459	8.600037	8.277886	8.393114	8.962399	9.025087	8.945828
AT3G16420	1645.123	-0.54003219	0.107396	-5.0284	4.95E-07	6.22E-06	10.52745	10.31437	10.30781	10.84277	10.98736	10.96091
AT3G17800	1062.801	-0.539087939	0.111082	-4.85308	1.22E-06	1.43E-05	9.732105	9.671537	9.861847	10.17259	10.38634	10.33738
AT3G13740	355.9983	-0.539030141	0.136825	-3.93956	8.16E-05	6.64E-04	8.270192	8.109173	8.17585	8.801986	8.544777	8.807886
AT1G26580	1445.497	-0.538944039	0.102284	-5.26909	1.37E-07	1.89E-06	10.23828	10.23523	10.1345	10.79131	10.60372	10.82448
AT5G04040	927.4856	-0.538750658	0.102863	-5.23754	1.63E-07	2.22E-06	9.607503	9.547042	9.522948	10.09431	10.20903	10.00875
AT1G35670	268.2044	-0.538525431	0.166871	-3.2272	1.25E-03	7.35E-03	7.855842	7.686387	7.752166	8.087074	8.590752	8.250405
AT3G02860	218.1264	-0.538492296	0.171508	-3.13976	1.69E-03	9.51E-03	7.382594	7.542334	7.52398	8.078063	7.723906	8.208377
AT1G58030	1327.843	-0.538308989	0.093328	-5.76791	8.03E-09	1.32E-07	10.15663	10.01943	10.06207	10.67211	10.57885	10.61064
AT3G49490	765.48	-0.537926502	0.106368	-5.0572	4.25E-07	5.41E-06	9.271769	9.26157	9.328503	9.730198	9.814884	9.927809
AT4G13520	580.8451	-0.537515436	0.136344	-3.94234	8.07E-05	6.57E-04	8.897341	8.744533	8.997742	9.339489	9.620253	9.320286
AT1G70160	1076.853	-0.537350814	0.087925	-6.11143	9.87E-10	1.85E-08	9.763035	9.771853	9.802139	10.30013	10.32237	10.33319
AT4G38250	471.6288	-0.536075173	0.131645	-4.07212	4.66E-05	4.00E-04	8.688624	8.424359	8.632235	9.070855	9.236518	9.081736
AT3G13670	890.5159	-0.53580497	0.105709	-5.06867	4.01E-07	5.12E-06	9.552769	9.532286	9.434167	10.00441	9.969049	10.15025
AT4G37470	484.8373	-0.535688776	0.156036	-3.43311	5.97E-04	3.88E-03	8.766546	8.401343	8.678698	9.001054	9.37433	9.117863
AT3G20310	279.8613	-0.53511652	0.156276	-3.42418	6.17E-04	3.99E-03	7.890211	7.624132	7.96708	8.400731	8.45814	8.279694
AT1G33680	674.7309	-0.534905067	0.159548	-3.35263	8.00E-04	0.0049924	9.160716	9.152769	8.994247	9.93623	9.353146	9.587167
AT3G19930	270.0849	-0.534783419	0.161638	-3.30852	9.38E-04	5.71E-03	7.950083	7.616158	7.756298	8.341714	8.468027	8.171351
AT1G43130	359.0491	-0.534245196	0.146525	-3.64609	0.000266	0.0018994	8.351379	8.024343	8.188139	8.812891	8.77726	8.619142
AT4G09695	466.3626	-0.533709443	0.131804	-4.04927	5.14E-05	0.0004368	8.664999	8.415197	8.620949	9.047962	9.230722	9.058266
AT1G49240	2295.244	-0.533038477	0.121772	-4.37735	1.20E-05	0.0001176	11.04178	10.66969	10.87539	11.35843	11.49983	11.36647
AT1G13260	1017.645	-0.532694928	0.156439	-3.40513	6.61E-04	0.0042399	9.901736	9.450561	9.696424	10.06268	10.45257	10.17385
AT1G73840	215.2995	-0.531830733	0.168576	-3.15484	1.61E-03	9.09E-03	7.494057	7.23385	7.626867	8.022777	8.034523	7.948751
AT5G32440	720.1835	-0.531725904	0.118485	-4.4877	7.20E-06	7.36E-05	9.253404	9.06469	9.280532	9.835552	9.642251	9.725565
AT4G35830	4519.472	-0.531424385	0.079696	-6.66815	2.59E-11	5.98E-10	11.85457	11.88288	11.81461	12.45792	12.33914	12.35351
AT2G17380	365.8347	-0.53085509	0.139104	-3.81625	1.35E-04	1.04E-03	8.301175	8.086266	8.268455	8.688146	8.913741	8.681917

AT5G49645	348.3558	-0.530406946	0.147643	-3.59249	3.28E-04	2.28E-03	8.311357	8.042308	8.109561	8.790998	8.506912	8.754644
AT5G10480	453.3443	-0.530131329	0.1298	-4.08421	4.42E-05	0.0003814	8.608319	8.405976	8.574898	8.97216	9.201388	9.034408
AT4G28240	2260.752	-0.530058609	0.152724	-3.4707	5.19E-04	3.43E-03	11.02796	10.51853	10.94764	11.35564	11.53788	11.2566
AT4G29010	1667.437	-0.529712803	0.101502	-5.21872	1.80E-07	2.44E-06	10.31049	10.47731	10.44725	10.8441	10.96135	11.02717
AT5G39610	312.8259	-0.528860048	0.153679	-3.44134	5.79E-04	3.78E-03	8.159013	7.86187	7.981272	8.564547	8.35535	8.664258
AT2G34690	532.5475	-0.528740814	0.128913	-4.10155	4.10E-05	0.0003565	8.904084	8.598378	8.785673	9.358221	9.270813	9.274464
AT4G13360	317.7541	-0.528686782	0.13097	-4.0367	5.42E-05	0.0004581	7.975916	8.030356	8.056928	8.491765	8.669986	8.514622
AT5G43330	238.4156	-0.52825404	0.16094	-3.2823	0.00103	0.0062079	7.551021	7.521138	7.760419	8.013353	8.099571	8.302704
AT2G20120	567.3503	-0.52712684	0.135791	-3.88189	0.000104	0.0008175	8.96662	8.671383	8.924349	9.52666	9.32622	9.314637
AT5G07285	177.6582	-0.527116254	0.168499	-3.1283	0.001758	0.0098448	7.164051	7.050731	7.343483	7.755209	7.673524	7.737017
AT5G07470	563.4851	-0.526442682	0.126088	-4.17522	2.98E-05	0.0002677	8.91581	8.682881	8.937147	9.441388	9.40038	9.303273
AT3G57340	739.7208	-0.52491932	0.122974	-4.26852	1.97E-05	1.84E-04	9.389372	9.083609	9.244213	9.718646	9.759603	9.838124
AT3G20250	515.9372	-0.524839732	0.134855	-3.89188	9.95E-05	0.0007879	8.904084	8.657851	8.595803	9.331927	9.159291	9.265708
AT2G32520	397.0067	-0.524060544	0.121956	-4.29712	1.73E-05	1.64E-04	8.375841	8.327567	8.347277	8.790998	8.862188	8.967588
AT3G09275	5279.387	-0.523853076	0.115947	-4.51802	6.24E-06	6.44E-05	12.17341	11.89961	12.14402	12.55408	12.74499	12.51123
AT1G09770	1740.499	-0.523775284	0.125301	-4.18013	2.91E-05	2.62E-04	10.39609	10.6874	10.31972	11.05053	11.03968	10.92799
AT3G19960	560.3164	-0.523456532	0.144618	-3.61957	0.000295	0.0020803	8.727161	9.069078	8.704592	9.277857	9.379578	9.457328
AT2G38550	324.0156	-0.523021536	0.137391	-3.80682	1.41E-04	1.07E-03	8.00757	8.018304	8.150955	8.525303	8.497289	8.712309
AT4G32210	409.3161	-0.523015964	0.133599	-3.91481	9.05E-05	0.0007277	8.442206	8.221185	8.502942	8.942675	8.877107	8.938501
AT5G53130	575.5309	-0.522769477	0.129076	-4.0501	5.12E-05	4.36E-04	8.937336	8.727978	8.974873	9.539848	9.32622	9.356473
AT4G25640	365.7319	-0.522728354	0.152576	-3.42601	0.000613	0.0039676	8.331507	8.262642	8.093319	8.947631	8.525969	8.767106
AT3G21330	670.9732	-0.522071666	0.13527	-3.85948	1.14E-04	0.0008872	9.174726	9.001813	9.126234	9.394971	9.783555	9.693279
AT5G25280	3466.171	-0.521905251	0.103602	-5.0376	4.71E-07	5.95E-06	11.57924	11.32179	11.50407	12.01107	12.06155	11.92067
AT4G02340	267.1454	-0.52136652	0.148737	-3.50529	4.56E-04	3.07E-03	7.866239	7.632062	7.832646	8.207663	8.312092	8.39124
AT3G26890	909.4786	-0.520720796	0.096583	-5.39144	6.99E-08	1.01E-06	9.609567	9.50015	9.52416	10.03267	10.06988	10.10188
AT1G43700	726.7047	-0.520330181	0.134279	-3.87499	1.07E-04	0.0008366	9.433154	9.054399	9.145267	9.792126	9.722914	9.723435
AT4G12040	650.9597	-0.519508355	0.139507	-3.7239	0.000196	0.0014465	9.222716	8.819371	9.111793	9.51669	9.655291	9.589508
AT3G23640	1416.394	-0.519499013	0.092587	-5.61094	2.01E-08	3.11E-07	10.17348	10.17068	10.21402	10.73939	10.60148	10.76979
AT1G51200	2498.755	-0.519382669	0.121022	-4.29162	1.77E-05	0.0001673	11.16586	10.7773	11.034	11.50095	11.5738	11.50312
AT3G49590	492.5871	-0.518601819	0.140382	-3.69423	0.000221	0.001611	8.659031	8.534233	8.791725	9.237956	8.977427	9.311804
AT4G33910	251.7399	-0.518151798	0.151057	-3.43018	6.03E-04	3.91E-03	7.697949	7.65948	7.748022	8.174225	8.060895	8.39124
AT1G07440	427.5346	-0.517715951	0.126688	-4.08655	4.38E-05	3.78E-04	8.34891	8.437994	8.579569	8.922678	9.01163	9.00665
AT4G21580	503.8421	-0.517384328	0.133275	-3.88207	1.04E-04	8.17E-04	8.545013	8.798382	8.736318	9.13309	9.153175	9.350965
AT5G16840	358.0448	-0.517234345	0.14054	-3.68033	2.33E-04	1.69E-03	8.195201	8.126118	8.279982	8.525303	8.839518	8.795773

AT1G19400	1377.052	-0.517049253	0.091734	-5.63637	1.74E-08	2.71E-07	10.20658	10.10596	10.11854	10.60311	10.72619	10.66492
AT2G44065	273.2689	-0.516901461	0.146978	-3.51687	4.37E-04	2.95E-03	7.813489	7.742408	7.882648	8.498535	8.278775	8.22051
AT4G27800	1345.252	-0.516778212	0.109001	-4.74105	2.13E-06	2.40E-05	10.07009	9.994266	10.25402	10.63572	10.68439	10.57446
AT3G22970	1049.748	-0.516700546	0.115607	-4.46947	7.84E-06	7.93E-05	9.860728	9.710446	9.686713	10.39934	10.12404	10.28346
AT1G71730	355.0403	-0.516617788	0.154593	-3.34179	8.32E-04	5.16E-03	8.298618	7.937414	8.299936	8.757522	8.769286	8.614552
AT5G53300	4980.341	-0.516490233	0.13584	-3.8022	0.000143	0.00109	12.20993	11.73286	12.00832	12.55895	12.58837	12.40791
AT2G03890	2435.358	-0.516194685	0.132968	-3.8821	1.04E-04	8.17E-04	11.12639	10.93105	10.82366	11.69248	11.35282	11.39486
AT5G09600	406.251	-0.515875657	0.132254	-3.90065	9.59E-05	7.64E-04	8.430577	8.231661	8.48811	8.962399	8.839518	8.912559
AT1G13270	261.8782	-0.515762183	0.146186	-3.52813	0.000418	0.0028367	7.705689	7.727681	7.80897	8.191041	8.448184	8.183799
AT4G27750	263.7238	-0.515486574	0.158478	-3.25272	1.14E-03	6.79E-03	7.827745	7.533893	7.905155	8.303563	8.267496	8.285481
AT5G67330	392.0967	-0.515330927	0.150811	-3.41706	0.000633	0.0040787	8.363662	8.167631	8.460515	9.047962	8.712195	8.783557
AT2G23090	1676.449	-0.515228358	0.113773	-4.52858	5.94E-06	6.15E-05	10.52526	10.28764	10.45934	10.89886	11.08375	10.85593
AT2G36580	475.1219	-0.51464831	0.111205	-4.62792	3.69E-06	4.00E-05	8.587524	8.624451	8.632235	9.128733	9.084138	9.175095
AT1G77480	279.9924	-0.514639225	0.135756	-3.79092	0.00015	0.0011343	7.9337	7.813872	7.796984	8.371524	8.40766	8.336545
AT4G29905	1172.139	-0.51449494	0.13993	-3.67681	0.000236	0.0017124	10.17138	9.754703	9.770821	10.48535	10.38634	10.4265
AT3G60340	790.7249	-0.514288868	0.131804	-3.90191	9.54E-05	7.60E-04	9.324247	9.20288	9.491067	9.701145	10.00345	9.878887
AT5G45360	609.8339	-0.514128027	0.114222	-4.50112	6.76E-06	6.94E-05	9.070681	8.875564	8.962407	9.546397	9.490449	9.43407
AT3G61600	1365.453	-0.513283439	0.104434	-4.91491	8.88E-07	1.07E-05	10.09092	10.04564	10.26131	10.60625	10.62597	10.71831
AT5G44340	3831.372	-0.513089966	0.123034	-4.17031	3.04E-05	2.72E-04	11.68213	11.45014	11.71622	12.03106	12.31812	12.05463
AT3G15580	385.7334	-0.511435988	0.151577	-3.37409	7.41E-04	4.67E-03	8.370981	8.074674	8.460515	8.734765	8.935284	8.819899
AT1G11660	405.7118	-0.51018675	0.121696	-4.19229	2.76E-05	0.0002497	8.358761	8.370863	8.44011	8.834457	8.869667	8.992568
AT2G20230	1124.668	-0.509430995	0.140459	-3.62689	2.87E-04	2.03E-03	9.892441	9.659948	9.99108	10.1641	10.54188	10.38667
AT3G62580	311.052	-0.509112624	0.132523	-3.84169	1.22E-04	9.46E-04	7.9337	8.042308	8.029874	8.551584	8.581674	8.428329
AT5G03455	414.6763	-0.508091142	0.12886	-3.94297	8.05E-05	0.0006557	8.467465	8.275356	8.502942	8.937702	8.984333	8.878505
AT5G11580	679.7577	-0.508055633	0.132116	-3.84554	1.20E-04	9.34E-04	9.035798	9.120719	9.220479	9.552916	9.853112	9.512649
AT1G06400	800.8116	-0.506891523	0.151978	-3.33529	8.52E-04	5.26E-03	9.494759	9.181424	9.397596	9.893206	10.09243	9.628733
AT1G13960	432.8982	-0.506869391	0.132811	-3.81648	1.35E-04	0.0010362	8.632884	8.424359	8.369029	9.001054	9.064722	8.920019
AT3G15290	321.8143	-0.506595445	0.157749	-3.2114	1.32E-03	0.0077105	7.988661	7.885283	8.268455	8.682211	8.468027	8.543887
AT3G02360	956.8212	-0.506306912	0.091079	-5.55897	2.71E-08	4.13E-07	9.615742	9.628581	9.638275	10.10544	10.12716	10.17229
AT2G27190	334.8879	-0.506207688	0.134059	-3.776	1.59E-04	1.20E-03	8.095624	8.148407	8.090048	8.464361	8.736939	8.664258
AT1G29350	817.4092	-0.505874589	0.101034	-5.00699	5.53E-07	6.90E-06	9.448227	9.339919	9.405511	9.951114	9.937373	9.838124
AT4G34120	356.2215	-0.505227422	0.126767	-3.98547	6.73E-05	5.59E-04	8.206155	8.123308	8.271346	8.729019	8.761267	8.650871
AT3G47610	433.3673	-0.505021104	0.137065	-3.68455	2.29E-04	1.67E-03	8.532012	8.464884	8.44011	8.860969	9.213193	8.897523
AT5G60360	4567.568	-0.504990916	0.137574	-3.67068	0.000242	0.00175	11.64211	12.11651	11.83795	12.45966	12.44055	12.26512

AT4G32150	1300.883	-0.504599328	0.147195	-3.42809	6.08E-04	3.94E-03	10.2542	9.791459	10.11532	10.53724	10.7344	10.4563
AT4G17230	319.3714	-0.504583793	0.137755	-3.6629	2.49E-04	1.80E-03	8.035477	8.159423	7.93827	8.639969	8.477847	8.543887
AT4G36730	1351.656	-0.504117349	0.129376	-3.89652	9.76E-05	7.75E-04	10.31682	9.947366	10.08031	10.60625	10.74867	10.54219
AT4G36648	990.2147	-0.503810588	0.154673	-3.25726	1.12E-03	6.70E-03	9.809569	9.552275	9.652756	10.46648	9.944472	10.09531
AT5G01090	289.6389	-0.503677044	0.150836	-3.33923	8.40E-04	5.20E-03	7.903732	8.033353	7.772711	8.280179	8.40766	8.543887
AT3G09770	270.6311	-0.503492713	0.144601	-3.48196	4.98E-04	3.31E-03	7.769855	7.697767	7.945526	8.334164	8.333883	8.285481
AT1G48840	241.372	-0.503452528	0.155624	-3.23506	1.22E-03	7.18E-03	7.747532	7.499627	7.680041	8.041442	8.137237	8.26805
AT1G49510	390.734	-0.503263872	0.122125	-4.1209	3.77E-05	0.0003306	8.334006	8.312842	8.358194	8.757522	8.949469	8.827852
AT2G20060	275.4652	-0.503130359	0.146401	-3.43665	0.000589	0.0038332	7.99183	7.742408	7.756298	8.356696	8.312092	8.358672
AT1G29370	844.4214	-0.503118329	0.100719	-4.99526	5.88E-07	7.31E-06	9.505884	9.384026	9.452111	10.01861	9.93381	9.909189
AT4G12340	350.4802	-0.502822147	0.13208	-3.80695	0.000141	0.0010721	8.184162	8.060052	8.288568	8.699942	8.712195	8.655347
AT1G60200	759.3418	-0.502793986	0.131529	-3.82267	1.32E-04	1.02E-03	9.297607	9.473915	9.084114	9.832876	9.771629	9.802245
AT4G36850	2540.737	-0.502792125	0.130965	-3.83912	1.23E-04	9.54E-04	11.22124	10.88658	10.97272	11.57649	11.67438	11.36511
AT4G34890	783.9323	-0.502786422	0.112814	-4.45679	8.32E-06	8.37E-05	9.214601	9.421114	9.382971	9.797627	9.811005	9.927809
AT1G56460	454.3607	-0.501711766	0.127767	-3.92679	8.61E-05	6.97E-04	8.496758	8.653962	8.520055	8.991487	8.984333	9.193678
AT1G27290	773.8156	-0.501585444	0.146151	-3.43198	0.000599	0.003892	9.450532	9.052923	9.421212	9.811286	9.96207	9.708435
AT4G19530	2371.196	-0.501371999	0.093493	-5.36269	8.20E-08	1.16E-06	10.96365	10.86529	10.97936	11.37048	11.54723	11.40486
AT5G16030	7068.653	-0.501362911	0.080085	-6.26038	3.84E-10	7.57E-09	12.47189	12.46619	12.59893	13.01149	13.04537	12.995
AT3G04610	501.7944	-0.501195914	0.114719	-4.36889	1.25E-05	0.0001219	8.698355	8.690496	8.700308	9.115581	9.320774	9.175095
AT1G04410	5291.492	-0.501162017	0.131146	-3.8214	1.33E-04	1.02E-03	11.96993	12.06416	12.23474	12.3535	12.71417	12.70746
AT5G11770	694.337	-0.500972021	0.120211	-4.16745	3.08E-05	2.75E-04	9.120749	9.166486	9.209972	9.835552	9.629092	9.53711
AT4G36400	233.7307	-0.500258087	0.147497	-3.39164	6.95E-04	4.42E-03	7.601678	7.604113	7.58566	7.95548	8.221479	8.13335
AT4G32760	1205.748	-0.49998348	0.111761	-4.47369	7.69E-06	7.80E-05	10.07607	9.858057	9.944756	10.57456	10.45506	10.36497
AT5G39600	235.0947	-0.499132999	0.143631	-3.47511	0.000511	0.0033862	7.658614	7.559068	7.60412	8.03214	8.173944	8.13335
AT2G03680	739.6607	-0.499036606	0.145987	-3.41836	0.00063	0.0040624	9.273072	9.083609	9.392295	9.569087	9.979456	9.717026
AT1G14740	1518.365	-0.498785823	0.122928	-4.05753	4.96E-05	0.0004234	10.4904	10.22675	10.14872	10.80093	10.71792	10.87333
AT5G65910	540.9957	-0.498671763	0.134718	-3.70159	0.000214	0.0015667	8.760985	8.675226	8.971322	9.175955	9.384807	9.367428
AT5G14590	374.1894	-0.498314753	0.156697	-3.18011	1.47E-03	0.0084535	8.211602	8.223811	8.393114	8.518658	8.808725	8.978347
AT3G19290	500.9815	-0.498313164	0.118999	-4.18755	2.82E-05	2.55E-04	8.790399	8.703726	8.595803	9.141766	9.207302	9.250995
AT2G04940	286.2056	-0.498171873	0.136277	-3.65558	2.57E-04	1.84E-03	7.876561	7.806883	7.988316	8.393484	8.40766	8.385863
AT1G67325	333.0914	-0.49732444	0.158194	-3.14376	0.001668	0.0093965	8.203425	7.969065	8.144664	8.839799	8.535404	8.438754
AT5G62930	336.7187	-0.496133982	0.146373	-3.38952	0.0007	0.0044466	8.296057	7.975313	8.083485	8.670267	8.678527	8.53905
AT5G06140	884.8772	-0.495556928	0.095201	-5.20537	1.94E-07	2.61E-06	9.561326	9.533345	9.466055	9.999646	10.01023	10.04502
AT5G21160	1113.521	-0.495447485	0.109452	-4.52661	5.99E-06	6.20E-05	9.962758	9.829028	9.753354	10.45435	10.30317	10.28779

AT3G51880	1161.566	-0.495414316	0.10587	-4.67946	2.88E-06	3.19E-05	9.935041	9.957658	9.833769	10.34026	10.54655	10.34155
AT2G20670	8291.558	-0.495207967	0.110624	-4.47651	7.59E-06	7.71E-05	12.90935	12.62723	12.68613	13.29476	13.30734	13.12917
AT3G14010	737.2643	-0.49470261	0.126546	-3.90927	9.26E-05	7.42E-04	9.238808	9.332638	9.199387	9.943691	9.58888	9.710588
AT1G67310	525.4323	-0.494400105	0.111133	-4.44871	8.64E-06	8.67E-05	8.788578	8.71497	8.809731	9.32051	9.195448	9.280272
AT1G32700	789.6186	-0.494373692	0.131866	-3.74906	0.000177	0.0013201	9.549547	9.23578	9.25885	9.965846	9.803215	9.790084
AT1G49300	448.5699	-0.493635812	0.147007	-3.35791	0.000785	0.0049041	8.587524	8.392033	8.620949	8.855706	9.253768	8.992568
AT1G75330	535.3686	-0.493578387	0.134839	-3.66051	2.52E-04	1.81E-03	8.864873	8.63238	8.883372	9.434343	9.159291	9.274464
AT5G01155	5625.244	-0.493261902	0.147665	-3.34042	0.000837	0.0051796	12.38839	11.98181	12.15736	12.85311	12.74347	12.43224
AT1G65980	1011.016	-0.492677192	0.114747	-4.2936	1.76E-05	0.000166	9.777796	9.628581	9.728329	10.35898	10.17019	10.0887
AT2G29300	216.1798	-0.492121424	0.152084	-3.23586	1.21E-03	7.16E-03	7.387414	7.546535	7.533642	7.895187	8.060895	8.012944
AT1G51160	393.6408	-0.491271683	0.151028	-3.25285	1.14E-03	0.0067897	8.373413	8.213278	8.463046	8.609021	8.963516	8.956749
AT3G21175	220.5888	-0.490217298	0.14633	-3.35009	0.000808	0.0050349	7.593357	7.460079	7.514252	8.03214	7.994038	8.02683
AT3G48890	526.6016	-0.490069245	0.11771	-4.16335	3.14E-05	2.80E-04	8.803081	8.655908	8.856677	9.285706	9.298783	9.224129
AT4G34370	295.0241	-0.48999461	0.139907	-3.50229	4.61E-04	0.0031012	8.029322	7.924557	7.875068	8.525303	8.289966	8.474656
AT5G06395	608.3536	-0.489646576	0.12492	-3.91969	8.87E-05	0.0007164	9.103276	8.955162	8.871992	9.387695	9.611358	9.428851
AT2G21620	547.7878	-0.489523856	0.121831	-4.01806	5.87E-05	4.93E-04	8.783101	8.86719	8.841197	9.469227	9.304312	9.193678
AT4G21860	855.7789	-0.489196319	0.127165	-3.84693	1.20E-04	9.30E-04	9.363918	9.378144	9.661596	9.867285	10.0237	10.0105
AT2G45620	1069.438	-0.488063749	0.113978	-4.28208	1.85E-05	1.74E-04	9.841536	9.815163	9.72938	10.44035	10.26682	10.14708
AT4G36720	356.3987	-0.487654939	0.154268	-3.1611	0.001572	0.0089275	8.407033	8.04528	8.163456	8.55808	8.753203	8.799822
AT1G18450	616.9834	-0.486387937	0.121439	-4.00522	6.20E-05	0.0005178	9.122196	8.898758	8.994247	9.529968	9.37433	9.570674
AT5G37710	257.7451	-0.485580571	0.150952	-3.21678	0.001296	0.0075949	7.795468	7.749716	7.680041	8.09603	8.438159	8.177588
AT2G28400	537.3677	-0.485299106	0.132343	-3.66697	2.45E-04	1.77E-03	8.976251	8.679058	8.752958	9.262029	9.230722	9.38909
AT1G59700	282.8324	-0.485145822	0.137204	-3.53595	4.06E-04	2.76E-03	7.84537	7.831198	7.96708	8.443461	8.365965	8.308399
AT2G25910	482.5146	-0.484807937	0.121276	-3.99756	6.40E-05	5.33E-04	8.708021	8.555238	8.680874	9.070855	9.253768	9.098269
AT1G16810	474.022	-0.484699456	0.12681	-3.82224	1.32E-04	0.0010165	8.682754	8.532116	8.652331	8.981856	9.253768	9.10483
AT1G07280	1127.749	-0.484500918	0.124905	-3.87896	1.05E-04	8.26E-04	9.694675	9.857211	10.05202	10.28063	10.38894	10.41993
AT3G50700	407.1338	-0.484055748	0.134177	-3.6076	3.09E-04	2.17E-03	8.498986	8.252389	8.460515	8.796502	8.935284	8.953118
AT2G01100	629.4153	-0.483914121	0.126516	-3.82492	1.31E-04	1.01E-03	9.140871	9.067617	8.877693	9.591425	9.575223	9.402466
AT3G22480	267.6751	-0.483790617	0.145112	-3.33392	8.56E-04	5.28E-03	7.917128	7.756987	7.718673	8.2723	8.397349	8.214456
AT4G09830	542.319	-0.483619761	0.147215	-3.28512	1.02E-03	0.0061503	8.969838	8.548969	8.904006	9.358221	9.331646	9.239116
AT1G78420	291.3368	-0.483481581	0.141422	-3.41871	0.000629	0.0040591	7.998147	7.969065	7.792967	8.356696	8.516472	8.375048
AT2G08865	212.7494	-0.483080809	0.154412	-3.12852	1.76E-03	9.84E-03	7.453261	7.512572	7.444257	8.068994	8.007659	7.811102
AT5G10780	548.0185	-0.483060062	0.11645	-4.14821	3.35E-05	2.96E-04	8.727161	8.848592	8.926184	9.269965	9.369063	9.331517
AT3G20390	853.6406	-0.482851613	0.106276	-4.54335	5.54E-06	5.76E-05	9.468841	9.469496	9.482362	10.04429	10.01023	9.826263

AT3G11730	331.9688	-0.482733476	0.146872	-3.28676	1.01E-03	6.12E-03	8.211602	7.959642	8.163456	8.450462	8.626506	8.716599
AT5G42820	1248.215	-0.482354335	0.106161	-4.54359	5.53E-06	5.76E-05	10.14601	9.915231	9.998076	10.44386	10.56509	10.51782
AT5G23080	407.6967	-0.482276261	0.135645	-3.55543	0.000377	0.002592	8.375841	8.553151	8.299936	8.917636	8.77726	8.985475
AT2G27860	2390.144	-0.482254269	0.109895	-4.3883	1.14E-05	1.12E-04	10.98089	10.8424	11.04629	11.34441	11.592	11.39218
AT5G42920	319.3092	-0.482112143	0.14182	-3.39946	6.75E-04	4.31E-03	8.124783	7.978426	8.070268	8.364129	8.61765	8.641876
AT1G27650	713.4968	-0.481615444	0.136176	-3.53672	4.05E-04	2.76E-03	9.390573	9.117898	9.115015	9.718646	9.837942	9.546779
AT5G60980	513.1889	-0.481444913	0.121568	-3.9603	7.49E-05	0.0006149	8.729061	8.784217	8.704592	9.312848	9.293232	9.075069
AT2G40640	245.7316	-0.480974067	0.147922	-3.25154	1.15E-03	6.82E-03	7.654621	7.612154	7.780847	8.050685	8.197907	8.250405
AT5G13570	374.0891	-0.480765743	0.131263	-3.66262	2.50E-04	1.80E-03	8.416497	8.210632	8.221402	8.757522	8.831881	8.733632
AT5G18630	792.5988	-0.480421314	0.143509	-3.34767	8.15E-04	5.07E-03	9.367582	9.405005	9.325725	9.552916	10.06337	9.907313
AT2G20960	879.1206	-0.480315867	0.098036	-4.89937	9.61E-07	1.15E-05	9.579342	9.471707	9.503413	9.956041	10.05684	9.996453
AT3G03535	396.2063	-0.48025275	0.140207	-3.42532	6.14E-04	3.98E-03	8.280594	8.297964	8.522484	8.711644	8.899199	8.945828
AT1G60420	440.4697	-0.479837353	0.115416	-4.15748	3.22E-05	2.86E-04	8.557899	8.551062	8.45798	9.015287	9.031769	8.978347
AT5G01800	544.6666	-0.479542085	0.140514	-3.41276	6.43E-04	4.14E-03	8.873489	8.60242	8.980183	9.324326	9.390016	9.227139
AT2G18270	315.9343	-0.479303943	0.14691	-3.26256	1.10E-03	6.59E-03	8.071865	7.875296	8.163456	8.531918	8.635307	8.423089
AT1G69490	607.3467	-0.478094225	0.143953	-3.32118	0.000896	0.0054907	9.085588	8.98952	8.868178	9.221681	9.663919	9.49777
AT4G16520	2211.349	-0.477956764	0.112434	-4.25102	2.13E-05	0.0001973	10.80921	10.79374	10.94085	11.16666	11.48042	11.33473
AT3G08943	319.0073	-0.477684775	0.143731	-3.32346	8.89E-04	5.45E-03	8.116097	8.080481	7.995326	8.682211	8.323029	8.591379
AT3G06430	669.1484	-0.477462465	0.113862	-4.19335	2.75E-05	2.49E-04	9.097404	9.097995	9.182596	9.465776	9.710474	9.642326
AT5G18525	329.87	-0.477456004	0.133581	-3.57428	0.000351	0.0024271	8.038544	8.210632	8.080192	8.564547	8.525969	8.673114
AT3G06190	597.3541	-0.477103126	0.143166	-3.33252	8.61E-04	5.30E-03	9.195489	8.808914	8.854751	9.513351	9.379578	9.441865
AT5G58220	547.3995	-0.476732398	0.145139	-3.28465	0.001021	0.0061591	8.888868	8.604437	8.992497	9.444897	9.265154	9.242095
AT4G31430	471.8947	-0.475649417	0.14431	-3.29603	9.81E-04	5.94E-03	8.753536	8.59026	8.524908	9.309002	8.928139	9.05149
AT5G54390	478.0398	-0.475518491	0.124012	-3.83444	1.26E-04	9.71E-04	8.57067	8.727978	8.625474	9.233904	9.115929	9.017122
AT3G20920	371.0999	-0.474594474	0.130021	-3.65014	2.62E-04	1.87E-03	8.329003	8.202666	8.297103	8.834457	8.808725	8.632826
AT1G64810	255.8115	-0.474563664	0.14205	-3.34083	8.35E-04	5.17E-03	7.709544	7.712801	7.796984	8.191041	8.344656	8.139753
AT2G41220	396.5274	-0.47429131	0.138267	-3.43025	6.03E-04	3.91E-03	8.321467	8.517206	8.285712	8.937702	8.695459	8.912559
AT3G61430	3817.341	-0.474263861	0.147021	-3.22582	1.26E-03	0.0073828	11.52423	11.56307	11.80766	11.93513	12.38878	11.99274
AT2G07741	3303.105	-0.474139465	0.149777	-3.16564	1.55E-03	8.81E-03	11.44379	11.56979	11.2589	11.7318	11.76639	12.18728
AT5G12410	226.1747	-0.474077478	0.151182	-3.13581	1.71E-03	9.62E-03	7.525009	7.674917	7.499537	7.95548	8.060895	8.120457
AT4G29840	644.0432	-0.473882789	0.124478	-3.80697	1.41E-04	1.07E-03	9.076662	8.945648	9.191779	9.677473	9.48559	9.485253
AT4G33380	593.6199	-0.473261694	0.109128	-4.33677	1.45E-05	0.0001392	8.958545	9.024585	8.88526	9.380383	9.470914	9.452192
AT1G04300	1002.22	-0.473008394	0.11949	-3.95855	7.54E-05	0.0006186	9.809569	9.711382	9.61001	10.34965	10.07636	10.12627
AT3G16400	1228.851	-0.472733954	0.122875	-3.84727	0.000119	0.0009295	10.02285	10.11448	9.865678	10.61094	10.50634	10.32057

AT5G65670	1536.089	-0.472730652	0.086983	-5.43473	5.49E-08	8.00E-07	10.26341	10.33759	10.38389	10.78993	10.80826	10.8135
AT3G23920	702.4139	-0.471634397	0.130466	-3.615	3.00E-04	0.0021121	9.230784	9.22926	9.131016	9.45885	9.860637	9.688919
AT1G08820	481.0976	-0.471169424	0.111196	-4.23729	2.26E-05	0.0002084	8.682754	8.671383	8.616409	9.093392	9.128451	9.168846
AT4G32390	432.7312	-0.471042111	0.114504	-4.11374	3.89E-05	3.40E-04	8.540693	8.482536	8.493071	8.981856	8.935284	9.01364
AT1G12440	2032.007	-0.471001021	0.128153	-3.6753	0.000238	0.001722	10.88232	10.51371	10.77255	11.28005	11.26623	11.06971
AT3G07220	444.8127	-0.470912156	0.1324	-3.55674	3.75E-04	2.58E-03	8.564299	8.435731	8.609573	8.986679	9.177483	8.886142
AT3G15660	445.2747	-0.470821979	0.131981	-3.56734	3.61E-04	2.49E-03	8.545013	8.442511	8.634482	8.860969	9.140866	9.048089
AT4G37830	1199.879	-0.469284485	0.132209	-3.54957	3.86E-04	2.64E-03	9.988367	9.809055	10.10077	10.3793	10.63697	10.31067
AT1G27100	1063.114	-0.466982	0.099137	-4.71047	2.47E-06	2.77E-05	9.883085	9.729052	9.785049	10.22047	10.33323	10.26013
AT3G20770	2982.393	-0.466666701	0.095309	-4.89636	9.76E-07	1.17E-05	11.39106	11.23398	11.23652	11.84835	11.70292	11.71948
AT5G19540	471.3859	-0.466493589	0.134928	-3.45734	5.46E-04	3.59E-03	8.487808	8.59026	8.797752	9.066306	9.04504	9.181316
AT1G17290	1270.524	-0.466319458	0.088411	-5.27442	1.33E-07	1.84E-06	10.06784	10.0164	10.09262	10.57456	10.50393	10.50299
AT5G41370	531.2508	-0.466153209	0.137735	-3.38442	7.13E-04	4.52E-03	8.738524	8.944057	8.715245	9.128733	9.230722	9.436673
AT4G19600	365.9166	-0.465487902	0.133597	-3.48426	4.94E-04	3.28E-03	8.270192	8.377954	8.144664	8.740488	8.652751	8.80386
AT4G15545	2851.212	-0.464498667	0.127011	-3.65716	0.000255	0.0018335	11.24401	11.06706	11.34477	11.54985	11.88553	11.62566
AT3G03950	287.1686	-0.464316094	0.134778	-3.44505	5.71E-04	3.73E-03	7.99183	7.891904	7.859785	8.422254	8.40766	8.330959
AT3G09820	849.2781	-0.463279274	0.131256	-3.52958	4.16E-04	2.82E-03	9.286037	9.591441	9.537428	10.06496	9.955057	9.812301
AT4G35300	298.1454	-0.462760597	0.144466	-3.20325	0.001359	0.0079089	7.802703	8.060052	8.036685	8.371524	8.487601	8.459378
AT1G06700	1140.281	-0.462442886	0.09614	-4.8101	1.51E-06	1.75E-05	9.926786	9.822979	9.959168	10.34965	10.42999	10.3318
AT5G54810	731.6899	-0.462312649	0.133858	-3.45375	5.53E-04	3.64E-03	9.345458	9.30686	9.131016	9.92873	9.646611	9.598834
AT1G68020	1231.808	-0.46195006	0.096732	-4.77555	1.79E-06	2.05E-05	9.961142	10.11377	9.970774	10.44561	10.48215	10.51536
AT4G21960	11064.21	-0.461657842	0.136434	-3.38374	7.15E-04	4.53E-03	13.38253	13.09964	13.03603	13.79181	13.70076	13.43016
AT4G10030	491.4995	-0.461045231	0.125475	-3.67441	2.38E-04	0.0017266	8.770241	8.582096	8.713121	9.038701	9.270813	9.159423
AT3G10300	298.5124	-0.461014148	0.146974	-3.1367	1.71E-03	9.60E-03	8.02314	7.792802	8.093319	8.386201	8.448184	8.484752
AT5G25340	368.1177	-0.460376083	0.120843	-3.8097	0.000139	0.0010625	8.306275	8.282932	8.248058	8.751866	8.687018	8.779462
AT5G16150	3761.104	-0.460277992	0.114655	-4.01447	5.96E-05	0.0004997	11.53377	11.51804	11.80866	11.989	12.14018	12.13855
AT5G64370	321.4265	-0.459988736	0.134553	-3.41864	0.000629	0.0040592	8.101503	7.990814	8.154091	8.457428	8.544777	8.628279
AT3G58600	251.5512	-0.459880573	0.141624	-3.24719	1.17E-03	6.92E-03	7.817066	7.70154	7.671314	8.182658	8.149577	8.244476
AT4G05160	416.1879	-0.459876435	0.140822	-3.26565	1.09E-03	6.53E-03	8.3365	8.417493	8.600407	8.790998	8.891872	9.054882
AT4G34870	2079.509	-0.459827324	0.107745	-4.26774	1.97E-05	1.84E-04	10.72508	10.69357	10.89145	11.10367	11.2747	11.32
AT1G53590	297.9142	-0.459612995	0.136791	-3.35997	7.80E-04	4.87E-03	7.982303	8.048247	7.893946	8.429358	8.344656	8.524443
AT3G09850	698.5416	-0.459504618	0.129429	-3.55026	3.85E-04	2.64E-03	9.070681	9.396883	9.106947	9.692313	9.659612	9.640069
AT1G78680	388.9596	-0.459414452	0.12506	-3.67355	0.000239	0.0017309	8.285767	8.428918	8.36091	8.796502	8.753203	8.901297
AT3G06380	345.7327	-0.459105441	0.140845	-3.25965	1.12E-03	0.0066551	8.329003	8.030356	8.185076	8.711644	8.635307	8.605328

AT4G32610	307.2395	-0.458958681	0.129157	-3.55349	0.00038	0.0026077	8.065864	7.972192	8.009245	8.484963	8.516472	8.443937
AT3G19860	801.2896	-0.458865507	0.122457	-3.74715	0.000179	0.0013295	9.526789	9.221396	9.426408	9.911077	9.882981	9.788047
AT3G01520	968.3092	-0.458523321	0.132989	-3.44783	0.000565	0.0036998	9.835375	9.468389	9.679115	10.05581	10.26682	10.07373
AT4G13640	341.1923	-0.457722839	0.139712	-3.27619	1.05E-03	6.32E-03	8.164639	8.027353	8.294263	8.62148	8.72049	8.553511
AT4G28910	506.2248	-0.455382829	0.124685	-3.65227	2.60E-04	0.00186	8.861413	8.684788	8.665575	9.293513	9.096938	9.193678
AT5G12200	425.1044	-0.454955374	0.119041	-3.82184	1.32E-04	0.0010173	8.525467	8.506461	8.437538	8.927704	8.877107	9.024061
AT3G15095	549.3816	-0.454903825	0.1292	-3.52092	0.00043	0.0029073	8.790399	9.027594	8.732128	9.262029	9.342436	9.342662
AT5G36160	616.9632	-0.454812835	0.113708	-3.99984	6.34E-05	0.0005283	9.011025	8.918346	9.135782	9.482948	9.480714	9.482737
AT1G08110	1070.827	-0.453204764	0.106187	-4.26799	1.97E-05	1.84E-04	9.779631	9.750156	9.918261	10.25487	10.37587	10.19552
AT5G62910	304.0855	-0.453109213	0.13138	-3.44885	0.000563	0.0036904	7.959825	8.021327	8.026456	8.443461	8.55409	8.396597
AT5G56220	319.666	-0.452526105	0.140496	-3.22092	1.28E-03	7.50E-03	8.116097	8.04528	8.080192	8.371524	8.525969	8.686298
AT1G68490	512.7857	-0.452089168	0.126648	-3.56966	3.57E-04	2.47E-03	8.868326	8.764512	8.632235	9.097857	9.270813	9.271551
AT3G57050	796.7833	-0.451640327	0.105382	-4.28573	1.82E-05	1.71E-04	9.424972	9.29567	9.461	9.900892	9.799304	9.844019
AT5G28300	1496.382	-0.451577152	0.087062	-5.18685	2.14E-07	2.86E-06	10.33127	10.33395	10.24449	10.72358	10.75474	10.79232
AT5G19500	1109.352	-0.451390886	0.106712	-4.22998	2.34E-05	2.15E-04	9.90005	9.816904	9.893147	10.44211	10.33593	10.19245
AT1G52190	1472.283	-0.451367915	0.11122	-4.05834	4.94E-05	4.22E-04	10.35238	10.2655	10.21552	10.55845	10.82759	10.80345
AT3G06810	460.6652	-0.45092904	0.115862	-3.89197	9.94E-05	7.88E-04	8.610382	8.673305	8.532156	9.047962	9.03842	9.091679
AT5G03630	520.1411	-0.450720273	0.143062	-3.15052	1.63E-03	0.0092044	8.8666	8.840059	8.623213	9.430808	9.064722	9.184416
AT1G69410	804.0144	-0.450547402	0.11516	-3.91235	9.14E-05	7.33E-04	9.528972	9.344753	9.34506	9.965846	9.767632	9.844019
AT1G05010	3841.519	-0.450119785	0.143373	-3.13951	0.001692	0.0095114	11.73745	11.4779	11.75132	11.87526	12.34988	12.09143
AT5G43850	488.9117	-0.44929389	0.140927	-3.18814	1.43E-03	8.27E-03	8.630852	8.608462	8.813702	8.996279	9.353146	9.071724
AT5G66180	475.4665	-0.44897445	0.12033	-3.7312	0.000191	0.0014092	8.64903	8.628421	8.665575	9.061741	9.248041	9.003142
AT2G42680	959.5167	-0.448871229	0.106507	-4.21446	2.50E-05	2.28E-04	9.688829	9.600563	9.698573	9.980428	10.20016	10.15974
AT2G33340	1319.647	-0.448627679	0.11473	-3.91028	9.22E-05	7.39E-04	10.27451	10.00117	10.07867	10.6631	10.56048	10.49926
AT5G20520	749.6725	-0.448055934	0.126383	-3.54521	0.000392	0.0026788	9.420276	9.117898	9.368196	9.848859	9.731148	9.697625
AT5G63510	436.5805	-0.447373752	0.117946	-3.79304	0.000149	0.0011257	8.478802	8.504302	8.604997	8.927704	9.01163	9.003142
AT2G43745	321.2502	-0.44732097	0.135368	-3.30447	9.52E-04	5.78E-03	8.029322	8.030356	8.206377	8.505274	8.497289	8.609947
AT5G38650	521.1852	-0.44703456	0.112455	-3.97522	7.03E-05	0.000581	8.819225	8.696181	8.839251	9.246025	9.224903	9.236131
AT2G14910	1937.963	-0.446983065	0.12447	-3.59108	0.000329	0.0022944	10.81638	10.44278	10.74734	11.05628	11.14512	11.18333
AT5G03280	1263.645	-0.446458493	0.096679	-4.61794	3.88E-06	4.18E-05	9.984395	10.15632	10.04274	10.48365	10.50153	10.54822
AT5G22080	624.0062	-0.445418382	0.110543	-4.02938	5.59E-05	4.72E-04	9.085588	9.083609	8.964194	9.405816	9.538159	9.53711
AT5G47560	1647.732	-0.445274668	0.10686	-4.16688	3.09E-05	2.76E-04	10.50045	10.4126	10.41559	10.8294	11.05452	10.78825
AT5G49460	742.2424	-0.44399599	0.139436	-3.18424	1.45E-03	0.0083545	9.213244	9.224022	9.438031	9.50665	9.834125	9.867357
AT3G15070	381.1874	-0.443627319	0.13992	-3.17057	1.52E-03	8.69E-03	8.425899	8.247235	8.319618	8.615264	8.956509	8.771237

AT1G72710	1346.609	-0.440007816	0.10709	-4.10875	3.98E-05	0.0003467	10.2621	10.02847	10.17288	10.68404	10.56279	10.55183
AT3G13200	1230.108	-0.439965609	0.092767	-4.74272	2.11E-06	2.39E-05	10.06934	9.958447	10.05286	10.52402	10.45008	10.43433
AT3G61440	3239.823	-0.439706515	0.094593	-4.64843	3.34E-06	3.66E-05	11.47498	11.45517	11.33582	11.78676	11.97145	11.83645
AT1G48430	361.1619	-0.439400168	0.134149	-3.27545	0.001055	0.0063306	8.363662	8.142867	8.268455	8.796502	8.678527	8.637358
AT2G43010	1533.605	-0.439380096	0.12565	-3.49686	4.71E-04	3.15E-03	10.24825	10.5196	10.24449	10.85996	10.86548	10.63123
AT5G12110	388.8758	-0.438969444	0.129856	-3.38043	7.24E-04	4.57E-03	8.3365	8.349377	8.406322	8.682211	8.970488	8.775355
AT3G06860	627.1273	-0.438410977	0.133774	-3.27725	1.05E-03	6.30E-03	8.902402	9.224022	9.027111	9.394971	9.48559	9.6081
AT1G77180	1748.015	-0.438384074	0.084587	-5.18266	2.19E-07	2.91E-06	10.58313	10.5445	10.47573	10.99775	10.97528	10.95457
AT4G32910	506.2152	-0.4364196	0.116087	-3.75943	0.00017	0.0012717	8.838714	8.66753	8.742581	9.217584	9.134672	9.212026
AT1G71980	375.8341	-0.436307181	0.134493	-3.24408	1.18E-03	6.98E-03	8.353844	8.202666	8.40895	8.723251	8.669986	8.874671
AT4G33090	1491.717	-0.43595781	0.113666	-3.83543	0.000125	0.0009675	10.18321	10.37352	10.35897	10.60625	10.75474	10.86658
AT1G50010	1305.16	-0.435338414	0.126263	-3.44787	0.000565	0.0036998	10.05956	10.02772	10.24376	10.35526	10.70125	10.5874
AT5G20350	1000.396	-0.434503133	0.118802	-3.65738	2.55E-04	1.83E-03	9.889046	9.602583	9.686713	10.23676	10.18526	10.08705
AT5G54680	806.0329	-0.433893848	0.124347	-3.48937	4.84E-04	3.23E-03	9.500333	9.221396	9.52416	9.816714	9.841749	9.9148
AT4G38130	822.5786	-0.432583871	0.113556	-3.80944	1.39E-04	1.06E-03	9.528972	9.504476	9.304717	9.872507	9.958568	9.832206
AT4G16180	584.7446	-0.430341431	0.110801	-3.8839	1.03E-04	8.12E-04	9.04498	8.918346	8.918829	9.343255	9.390016	9.447037
AT3G07310	847.6814	-0.430309583	0.10863	-3.96123	7.46E-05	6.13E-04	9.46086	9.517378	9.509546	9.781063	9.986352	10.0105
AT2G26300	682.297	-0.429770166	0.110869	-3.87639	1.06E-04	8.33E-04	9.206441	9.200216	9.140532	9.733071	9.597913	9.512649
AT3G13772	598.577	-0.429511117	0.113399	-3.78762	1.52E-04	1.15E-03	9.075169	8.924817	8.974873	9.365646	9.524011	9.394455
AT5G58290	1042.886	-0.42928283	0.101446	-4.23162	2.32E-05	0.0002132	9.731157	9.750156	9.8997	10.17471	10.25544	10.25129
AT4G02080	799.7034	-0.428694311	0.119382	-3.59094	3.29E-04	2.29E-03	9.462003	9.278089	9.486099	9.712836	9.948009	9.867357
AT2G47650	352.7945	-0.428295938	0.132565	-3.23083	1.23E-03	7.27E-03	8.264963	8.131723	8.305587	8.570985	8.669986	8.750466
AT5G25350	1903.26	-0.428288416	0.101136	-4.23477	2.29E-05	0.0002106	10.67766	10.68597	10.62443	11.23007	11.06596	10.97709
AT5G58200	473.7838	-0.428136393	0.110589	-3.87141	1.08E-04	8.48E-04	8.636937	8.694288	8.64789	9.11117	9.071223	9.088372
AT1G23960	826.2811	-0.427834837	0.114702	-3.72998	0.000191	0.0014152	9.565585	9.345959	9.461	9.781063	9.944472	9.944364
AT4G25170	744.332	-0.427118096	0.135169	-3.15988	0.001578	0.008957	9.421452	9.206868	9.281966	9.607172	9.958568	9.640069
AT5G44790	801.204	-0.426902517	0.128594	-3.31976	9.01E-04	5.51E-03	9.451683	9.505556	9.28483	10.02096	9.697925	9.802245
AT3G47810	435.4321	-0.426738268	0.121956	-3.49912	0.000467	0.0031335	8.481059	8.512917	8.609573	8.991487	9.04504	8.870827
AT3G09840	4364.842	-0.426388788	0.072653	-5.86885	4.39E-09	7.45E-08	11.82289	11.89034	11.87239	12.30363	12.29678	12.27024
AT4G25570	1242.155	-0.425333616	0.122099	-3.48352	4.95E-04	3.29E-03	10.1274	9.886513	10.11532	10.43507	10.62818	10.3636
AT1G80300	533.484	-0.42463105	0.117907	-3.6014	3.17E-04	2.21E-03	8.71379	8.916724	8.862439	9.254049	9.21906	9.303273
AT4G37260	684.6704	-0.424065621	0.113057	-3.75089	1.76E-04	1.31E-03	9.138014	9.218766	9.206955	9.591425	9.755573	9.507706
AT5G36230	1198.921	-0.423062808	0.095335	-4.43763	9.10E-06	9.08E-05	10.08648	9.920906	9.989325	10.4191	10.42238	10.43563
AT2G20050	896.1395	-0.422962947	0.123186	-3.43353	0.000596	0.0038738	9.549547	9.672499	9.51077	9.805838	10.10516	10.09201

AT1G65660	619.277	-0.42267342	0.131428	-3.21601	0.0013	0.0076101	9.012586	9.225333	8.887146	9.465776	9.41067	9.541953
AT1G04850	712.8506	-0.422612917	0.108115	-3.90893	9.27E-05	0.0007421	9.357791	9.217449	9.17026	9.680454	9.681022	9.669134
AT4G28260	667.2378	-0.422447271	0.101561	-4.15955	3.19E-05	2.84E-04	9.166336	9.169213	9.12783	9.536562	9.642251	9.565926
AT5G08570	533.8238	-0.422377141	0.11912	-3.54583	0.000391	0.0026733	8.729061	8.875564	8.900276	9.312848	9.153175	9.303273
AT3G51370	829.0599	-0.421765389	0.114298	-3.69005	2.24E-04	1.64E-03	9.498106	9.497981	9.402877	10.01153	9.919469	9.742493
AT5G28840	2535.023	-0.419878596	0.09813	-4.2788	1.88E-05	0.0001764	11.21955	11.00887	11.00521	11.50348	11.52017	11.48939
AT5G27860	699.8747	-0.419630166	0.122783	-3.41764	6.32E-04	0.004071	9.311622	9.076362	9.271898	9.686396	9.731148	9.527375
AT5G16650	448.707	-0.417746937	0.117514	-3.55487	3.78E-04	2.60E-03	8.57067	8.540567	8.643434	8.932711	9.084138	9.00665
AT1G53910	897.9374	-0.417396139	0.124005	-3.36596	0.000763	0.0047858	9.535501	9.433646	9.770821	10.00679	9.965564	10.04673
AT1G65820	491.6898	-0.416770064	0.118331	-3.52207	0.000428	0.0028969	8.736636	8.616479	8.799756	9.084419	9.159291	9.171974
AT1G11840	1098.014	-0.416439606	0.132004	-3.15476	1.61E-03	9.09E-03	9.683939	10.06261	9.859928	10.24484	10.2237	10.41334
AT5G62700	1862.261	-0.415999218	0.102501	-4.05849	4.94E-05	0.0004219	10.49989	10.66196	10.74318	11.07791	11.09336	11.00191
AT5G55190	1162.723	-0.415995068	0.132077	-3.14964	1.63E-03	0.0092278	10.08945	9.699166	10.05622	10.42444	10.37324	10.34016
AT5G42090	814.4794	-0.414591699	0.113268	-3.66029	2.52E-04	1.81E-03	9.500333	9.362737	9.467316	9.794879	10.01023	9.786007
AT5G66030	980.448	-0.414419488	0.115477	-3.58875	0.000332	0.0023113	9.685897	9.810803	9.641629	9.973155	10.19124	10.22141
AT1G75950	1451.382	-0.414030924	0.11646	-3.55514	0.000378	0.0025935	10.31302	10.10311	10.41101	10.68996	10.62377	10.77391
AT2G15900	463.7386	-0.413460886	0.130197	-3.17565	0.001495	0.0085559	8.655039	8.682881	8.584226	9.088912	8.854671	9.18751
AT4G21110	537.1755	-0.413223428	0.12342	-3.34811	0.000814	0.005066	8.948795	8.727978	8.8586	9.361938	9.242291	9.18751
AT1G18700	434.7706	-0.412985876	0.125405	-3.29321	9.91E-04	6.00E-03	8.67489	8.480342	8.465572	8.967288	8.970488	8.945828
AT3G56310	915.029	-0.41288219	0.095762	-4.31157	1.62E-05	0.0001544	9.655249	9.575079	9.61457	9.999646	10.09562	10.00174
AT5G06260	313.7347	-0.412609221	0.130295	-3.16673	1.54E-03	0.0087797	8.092675	8.04528	8.080192	8.564547	8.506912	8.401935
AT3G03070	524.7635	-0.412248541	0.123919	-3.32677	8.79E-04	5.40E-03	8.847487	8.671383	8.911437	9.241996	9.293232	9.159423
AT4G25100	8059.956	-0.411429612	0.124245	-3.31145	0.000928	0.0056583	12.80973	12.55972	12.87244	13.09901	13.33698	13.05805
AT4G23890	1378.165	-0.411262305	0.104364	-3.94064	8.13E-05	0.0006612	10.16718	10.14597	10.30216	10.51236	10.72206	10.62554
AT3G23280	740.2043	-0.410997863	0.131262	-3.13112	0.001741	0.0097617	9.253404	9.200216	9.464793	9.552916	9.818753	9.794149
AT1G67480	389.3353	-0.410683368	0.12369	-3.32025	8.99E-04	5.51E-03	8.353844	8.412897	8.38513	8.694056	8.906489	8.799822
AT5G58720	640.5513	-0.409118497	0.123721	-3.30678	0.000944	0.0057465	9.207804	9.163753	8.926184	9.565867	9.470914	9.512649
AT1G21750	718.641	-0.408662895	0.113097	-3.61338	3.02E-04	2.12E-03	9.211886	9.365118	9.230911	9.572299	9.706303	9.761303
AT5G54430	499.4483	-0.405663393	0.120521	-3.36593	0.000763	0.0047858	8.792217	8.77887	8.680874	9.128733	9.051631	9.274464
AT1G59580	424.1261	-0.405649195	0.122358	-3.31526	9.16E-04	5.59E-03	8.616554	8.435731	8.480636	8.952571	8.899199	8.912559
AT2G30440	1393.289	-0.405553004	0.114763	-3.53384	4.10E-04	2.78E-03	10.39609	10.10026	10.16668	10.68996	10.5834	10.6301
AT2G03690	369.4681	-0.405116187	0.127555	-3.17601	1.49E-03	8.55E-03	8.380684	8.226433	8.333514	8.639969	8.745094	8.779462
AT2G37220	2509.012	-0.404702735	0.124756	-3.24395	0.001179	0.0069844	10.96768	10.98507	11.25087	11.35377	11.63652	11.44742
AT3G50000	1150.305	-0.40395847	0.112424	-3.59316	0.000327	0.0022787	10.07607	9.816034	9.945661	10.37563	10.42492	10.2733

AT1G67280	1423.418	-0.403309644	0.092574	-4.35663	1.32E-05	1.28E-04	10.23628	10.20299	10.33223	10.65706	10.72206	10.61524
AT1G06457	1472.794	-0.403261751	0.122958	-3.27967	0.001039	0.0062545	10.38227	10.30013	10.2386	10.52236	10.69916	10.8962
AT3G19910	487.3099	-0.402877302	0.119183	-3.38034	7.24E-04	0.0045719	8.64903	8.755465	8.727926	9.180173	9.177483	9.003142
AT5G42940	372.212	-0.402849932	0.124062	-3.24716	1.17E-03	6.92E-03	8.380684	8.280411	8.314022	8.807449	8.72049	8.668693
AT1G26830	870.1561	-0.401488707	0.10575	-3.79658	1.47E-04	1.11E-03	9.530062	9.575079	9.55414	10.03034	9.807115	10.0105
AT3G48750	600.0056	-0.40082223	0.111466	-3.59593	3.23E-04	2.26E-03	9.081132	8.936071	9.02882	9.48976	9.347801	9.413078
AT2G43970	2669.699	-0.398825126	0.102982	-3.87276	1.08E-04	8.43E-04	11.2946	11.14222	11.05596	11.62525	11.59989	11.48185
AT2G41760	365.662	-0.398068433	0.123013	-3.23599	0.001212	0.0071573	8.262341	8.290468	8.350014	8.676251	8.77726	8.664258
AT1G04440	772.0101	-0.397705802	0.10182	-3.90595	9.39E-05	7.50E-04	9.443606	9.362737	9.336806	9.827509	9.710474	9.798202
AT1G55680	806.9416	-0.397695828	0.110696	-3.59269	3.27E-04	2.28E-03	9.488043	9.510941	9.331276	9.885479	9.743412	9.89601
AT1G47128	4234.294	-0.397530392	0.10509	-3.78276	1.55E-04	1.17E-03	11.75271	11.96208	11.77647	12.15836	12.35588	12.18302
AT1G12910	602.3578	-0.397413375	0.116584	-3.40881	6.52E-04	4.19E-03	9.103276	8.936071	9.01509	9.380383	9.538159	9.350965
AT5G61230	509.8385	-0.396294412	0.119183	-3.3251	0.000884	0.0054218	8.826343	8.657851	8.856677	9.217584	9.183496	9.146762
AT1G02840	1364.333	-0.395775148	0.089757	-4.40942	1.04E-05	0.0001026	10.25024	10.16181	10.19283	10.65554	10.59473	10.54943
AT5G24890	967.6055	-0.395483277	0.118284	-3.34351	8.27E-04	0.0051383	9.855519	9.658008	9.588153	10.02566	10.20312	10.08539
AT3G19820	5763.17	-0.39514781	0.08642	-4.57243	4.82E-06	5.08E-05	12.31486	12.24278	12.28173	12.67257	12.77268	12.58347
AT2G40060	1051.477	-0.394516714	0.116204	-3.39504	6.86E-04	4.37E-03	9.877956	9.777226	9.817052	10.07179	10.39931	10.18936
AT1G52740	1658.02	-0.393839612	0.124763	-3.15671	1.60E-03	9.04E-03	10.58628	10.35806	10.49192	10.92171	11.02468	10.68038
AT4G38800	1141.178	-0.393293019	0.122719	-3.20483	0.001351	0.007871	9.965984	9.746507	10.09996	10.31747	10.40447	10.30498
AT1G54080	614.9592	-0.391452897	0.12496	-3.13263	1.73E-03	9.72E-03	9.14799	9.070537	8.935326	9.569087	9.456087	9.320286
AT3G14290	422.2333	-0.391080753	0.117817	-3.31939	9.02E-04	5.52E-03	8.46062	8.495635	8.584226	8.887003	8.935284	8.905061
AT3G51420	489.2655	-0.390119791	0.116428	-3.35074	0.000806	0.0050243	8.680792	8.690496	8.807742	9.043339	9.159291	9.156268
AT4G10610	763.7783	-0.389694964	0.111346	-3.49986	4.65E-04	0.0031272	9.377307	9.330203	9.409452	9.767112	9.620253	9.878887
AT3G20230	435.9157	-0.388762033	0.123636	-3.14441	1.66E-03	9.38E-03	8.581227	8.447013	8.645664	8.957493	9.004854	8.901297
AT5G23670	688.535	-0.388679916	0.121504	-3.19892	1.38E-03	8.01E-03	9.300165	9.180072	9.17026	9.444897	9.75153	9.628733
AT1G05890	1016.022	-0.3886524	0.098292	-3.95406	7.68E-05	0.0006294	9.873668	9.752886	9.713528	10.18314	10.15497	10.17852
AT1G32130	703.8632	-0.388560922	0.111787	-3.47592	0.000509	0.0033769	9.210527	9.347164	9.191779	9.543126	9.70212	9.682354
AT5G05930	360.7945	-0.387389343	0.124022	-3.12355	1.79E-03	9.99E-03	8.326495	8.223811	8.325192	8.694056	8.626506	8.716599
AT3G62700	807.7836	-0.385852879	0.111371	-3.46458	0.000531	0.003509	9.371236	9.553319	9.422513	9.923709	9.845547	9.750883
AT4G14040	1249.488	-0.385484176	0.104928	-3.6738	0.000239	0.0017302	9.959524	10.19966	10.07205	10.46821	10.50634	10.43433
AT5G57020	691.9082	-0.384987158	0.108165	-3.55925	0.000372	0.0025574	9.256042	9.144477	9.279097	9.625844	9.685267	9.541953
AT2G34170	559.3101	-0.384870654	0.115521	-3.33161	8.63E-04	0.0053194	8.909121	8.838346	9.020254	9.373033	9.248041	9.306122
AT3G55460	648.5875	-0.383249369	0.110425	-3.47068	5.19E-04	3.43E-03	9.192737	9.054399	9.15469	9.493153	9.615812	9.462445
AT2G36460	1282.362	-0.382526548	0.098213	-3.89488	9.82E-05	0.0007789	10.15875	10.07502	10.12574	10.38662	10.56279	10.56021

AT5G14390	519.3645	-0.381458644	0.120059	-3.17726	0.001487	0.0085199	8.890566	8.696181	8.854751	9.171726	9.282066	9.156268
AT4G02620	512.9818	-0.380779876	0.115348	-3.30114	0.000963	0.0058439	8.875205	8.733518	8.785673	9.119978	9.259472	9.175095
AT2G18750	800.0036	-0.380544067	0.121371	-3.13538	1.72E-03	9.64E-03	9.477909	9.560609	9.263213	9.792126	9.897687	9.786007
AT1G15690	5499.364	-0.380357757	0.091644	-4.15038	3.32E-05	0.0002941	12.23321	12.12952	12.29528	12.61142	12.68002	12.51677
AT3G01770	815.9748	-0.378617995	0.116906	-3.23866	1.20E-03	7.10E-03	9.542001	9.347164	9.513214	9.965846	9.837942	9.746694
AT2G46900	848.5741	-0.377819276	0.107289	-3.52152	4.29E-04	2.90E-03	9.636137	9.444945	9.496018	9.968286	9.875572	9.878887
AT5G01020	829.0968	-0.376457809	0.114536	-3.28682	1.01E-03	6.12E-03	9.60647	9.349571	9.515653	9.921191	9.879281	9.822288
AT3G63500	1112.458	-0.376383712	0.114031	-3.3007	9.64E-04	5.85E-03	9.914315	10.04638	9.785049	10.38662	10.21198	10.28923
AT3G57410	1852.636	-0.376209163	0.089309	-4.21246	2.53E-05	2.30E-04	10.70108	10.67833	10.57966	11.07452	11.05288	10.97082
AT1G60170	687.9507	-0.375779865	0.11673	-3.21923	1.29E-03	7.54E-03	9.138014	9.239678	9.294808	9.462317	9.693718	9.651318
AT1G14570	545.6406	-0.375584233	0.112964	-3.32482	0.000885	0.0054256	8.857944	8.834915	8.980183	9.217584	9.315308	9.280272
AT4G33510	1324.813	-0.375456177	0.101728	-3.69079	2.24E-04	1.63E-03	10.10562	10.10667	10.29366	10.59524	10.53719	10.51413
AT5G60160	563.4086	-0.375387099	0.117697	-3.18945	1.43E-03	8.24E-03	8.882053	8.892169	9.032232	9.225767	9.420887	9.303273
AT5G62690	2143.567	-0.374800215	0.09399	-3.98768	6.67E-05	0.000554	10.79159	10.86823	10.93083	11.26524	11.31224	11.14803
AT5G49980	641.9339	-0.374584769	0.114265	-3.27821	1.04E-03	6.28E-03	9.185837	9.182774	9.001228	9.536562	9.538159	9.441865
AT4G05320	17301.59	-0.373549286	0.087014	-4.29296	1.76E-05	0.0001664	13.80705	13.82235	13.99883	14.26596	14.25719	14.2396
AT3G27260	921.104	-0.372689195	0.109913	-3.39075	6.97E-04	4.43E-03	9.621891	9.724423	9.60429	10.101	9.871853	10.08373
AT3G28710	1610.061	-0.371002882	0.094034	-3.9454	7.97E-05	6.49E-04	10.49878	10.35985	10.50119	10.76909	10.88037	10.83438
AT2G01470	497.0871	-0.370841614	0.111474	-3.32671	8.79E-04	5.40E-03	8.790399	8.764512	8.736318	9.150389	9.058191	9.18751
AT4G02880	736.9025	-0.37076349	0.099374	-3.731	1.91E-04	1.41E-03	9.289904	9.317964	9.37493	9.718646	9.722914	9.664701
AT5G42092	771.6138	-0.3702962	0.111281	-3.32759	8.76E-04	0.0053814	9.444763	9.311806	9.417303	9.695263	9.897687	9.708435
AT2G02040	430.0235	-0.370137065	0.117739	-3.14371	0.001668	0.0093965	8.560035	8.519345	8.577235	8.834457	8.984333	8.956749
AT1G72180	1136.992	-0.369984247	0.102574	-3.60698	3.10E-04	0.0021731	9.825641	10.00652	10.0223	10.35526	10.31143	10.31209
AT3G27090	2486.602	-0.369532582	0.096842	-3.81581	1.36E-04	1.04E-03	11.17703	10.97341	11.09155	11.39608	11.4408	11.52285
AT1G74910	2461.938	-0.369068276	0.093005	-3.96826	7.24E-05	5.97E-04	11.091	10.9843	11.12709	11.34629	11.4743	11.4944
AT5G02515	685.0173	-0.368219083	0.110421	-3.33469	0.000854	0.0052673	9.166336	9.284393	9.212982	9.628933	9.672496	9.485253
AT1G76030	1821.758	-0.368206491	0.082399	-4.4686	7.87E-06	7.96E-05	10.65984	10.58183	10.66181	11.01556	11.0146	10.98512
AT1G16180	1057.36	-0.368123497	0.101423	-3.6296	2.84E-04	2.01E-03	9.797849	9.804676	9.942944	10.14481	10.2781	10.23792
AT3G15000	582.9324	-0.366915837	0.117411	-3.12505	1.78E-03	9.94E-03	8.929095	8.959896	9.080823	9.250042	9.461046	9.372874
AT1G72650	532.0173	-0.366235782	0.115521	-3.1703	1.52E-03	8.70E-03	8.843984	8.918346	8.829478	9.309002	9.103296	9.265708
AT5G24690	1724.98	-0.36547629	0.093572	-3.90582	9.39E-05	0.0007505	10.60192	10.52972	10.53886	11.01909	10.91872	10.83339
AT3G60820	918.7687	-0.364314011	0.116281	-3.13306	0.00173	0.0097038	9.603365	9.523786	9.809118	9.997258	10.00005	10.05183
AT1G70290	4292.379	-0.363797467	0.091744	-3.96533	7.33E-05	0.0006042	11.93208	11.9133	11.76907	12.31949	12.20886	12.18612
AT5G11500	1111.702	-0.362575983	0.093827	-3.8643	1.11E-04	8.71E-04	9.945701	9.906266	9.920104	10.26483	10.37849	10.22894

AT1G54580	654.4169	-0.362356148	0.111041	-3.26326	1.10E-03	0.0065817	9.092985	9.116485	9.273341	9.503287	9.514501	9.561163
AT1G32790	1201.815	-0.36230058	0.105671	-3.42858	6.07E-04	3.93E-03	10.07607	9.911977	10.12254	10.41373	10.32781	10.46142
AT3G27020	1215.784	-0.359841543	0.099685	-3.60979	3.06E-04	2.15E-03	10.00728	10.01033	10.14636	10.3346	10.47481	10.44342
AT3G28900	722.157	-0.359220544	0.105625	-3.4009	0.000672	0.0042942	9.383352	9.244859	9.281966	9.692313	9.706303	9.605789
AT5G11490	708.4252	-0.357774227	0.103701	-3.45004	5.61E-04	3.68E-03	9.315421	9.27556	9.239792	9.677473	9.685267	9.556385
AT3G15730	3126.801	-0.35733485	0.080505	-4.43868	9.05E-06	9.04E-05	11.43944	11.4181	11.40293	11.70131	11.81755	11.8161
AT5G23540	704.8472	-0.356800382	0.109409	-3.26115	1.11E-03	0.0066246	9.329266	9.188163	9.293386	9.536562	9.70212	9.653557
AT3G08930	629.2966	-0.355395841	0.111564	-3.18559	0.001445	0.0083248	9.192737	9.00487	9.124637	9.503287	9.465988	9.43407
AT5G16470	1435.504	-0.353370342	0.109754	-3.21965	1.28E-03	7.53E-03	10.38649	10.15563	10.34258	10.57456	10.75474	10.6335
AT5G43940	1707.923	-0.353293331	0.08588	-4.1138	3.89E-05	0.00034	10.50655	10.53449	10.60966	10.87043	10.90423	10.93997
AT1G12760	504.604	-0.351624103	0.111138	-3.16385	1.56E-03	8.85E-03	8.856206	8.771708	8.748816	9.150389	9.171444	9.124335
AT4G24520	1730.539	-0.351622513	0.100829	-3.48732	4.88E-04	3.25E-03	10.6332	10.57362	10.50057	11.04822	10.8561	10.85884
AT5G19330	1286.239	-0.351298969	0.091414	-3.84292	1.22E-04	0.0009424	10.1531	10.11235	10.15734	10.50398	10.56509	10.41862
AT4G12560	628.0257	-0.349254973	0.103945	-3.35999	7.79E-04	4.87E-03	9.140871	9.095129	9.10046	9.50665	9.390016	9.482737
AT4G05050	2492.395	-0.348556919	0.109376	-3.18677	1.44E-03	8.30E-03	11.09616	10.94902	11.23171	11.40872	11.54256	11.39218
AT4G30920	908.3897	-0.34533784	0.095065	-3.63264	0.000281	0.0019881	9.613687	9.612637	9.707137	9.990069	9.982908	10.00174
AT1G15500	1433.471	-0.342150406	0.103275	-3.313	9.23E-04	5.64E-03	10.36588	10.24496	10.29508	10.75219	10.66303	10.52273
AT5G25270	903.5038	-0.340123546	0.097962	-3.47198	5.17E-04	3.42E-03	9.713991	9.593473	9.607725	10.01153	9.982908	9.951662
AT2G17200	981.2124	-0.338353786	0.10137	-3.3378	8.44E-04	5.22E-03	9.754665	9.68208	9.842542	10.11648	10.03039	10.14708
AT1G13440	6681.832	-0.335466554	0.074949	-4.47591	7.61E-06	7.72E-05	12.53787	12.48847	12.55617	12.82444	12.92042	12.84852
AT1G30230	4716.68	-0.335423278	0.106373	-3.15326	1.61E-03	0.0091222	12.06927	11.87392	12.11917	12.4241	12.41266	12.24633
AT4G10920	629.0897	-0.334894302	0.10501	-3.18917	1.43E-03	0.0082465	9.152245	9.159644	9.047488	9.451891	9.465988	9.457328
AT3G01090	575.4276	-0.330527409	0.103657	-3.18867	0.001429	0.0082551	9.020365	8.98643	8.978415	9.328131	9.32622	9.328718
AT4G29380	879.1596	-0.327256716	0.10015	-3.26767	0.001084	0.0064922	9.680016	9.594488	9.544614	9.990069	9.915862	9.903555
AT5G63400	966.9449	-0.323633796	0.103599	-3.12391	0.001785	0.0099757	9.805073	9.685894	9.739853	10.08084	10.16412	9.971541
AT5G54540	1174.516	-0.306666819	0.096266	-3.18561	1.44E-03	8.32E-03	10.01197	10.01412	10.08853	10.35152	10.25259	10.42125
AT1G01490	938.4492	-0.304504006	0.095732	-3.1808	1.47E-03	8.44E-03	9.745308	9.74285	9.651647	9.975584	10.05356	10.03303
AT4G34450	2748.015	-0.302269055	0.091306	-3.3105	0.000931	0.0056748	11.17947	11.3221	11.28988	11.49589	11.56347	11.6421
AT4G02480	2965.301	-0.287304505	0.087128	-3.29751	9.75E-04	5.91E-03	11.32852	11.46298	11.35536	11.68062	11.60886	11.72162
AT5G64270	1991.579	-0.282411589	0.082565	-3.42048	0.000625	0.0040388	10.80021	10.81915	10.81872	11.11913	11.02468	11.13687
AT2G18300	2347.185	0.295316351	0.085626	3.448917	0.000563	0.0036904	11.36258	11.27318	11.37431	11.00846	11.1045	11.01154
AT1G07920	4462.73	0.296133567	0.093211	3.177035	1.49E-03	8.52E-03	12.15586	12.25903	12.37286	12.01521	11.95485	11.93086
AT3G53430	1351.275	0.298828308	0.094855	3.150384	1.63E-03	9.21E-03	10.49655	10.51479	10.61308	10.22456	10.31691	10.18936
AT2G39460	1364.959	0.303727772	0.088312	3.439272	0.000583	0.0038021	10.57787	10.55184	10.55259	10.31938	10.20016	10.2409

AT3G59970	1394.115	0.308978756	0.098864	3.125276	0.001776	0.0099382	10.65135	10.52653	10.59762	10.17471	10.35471	10.3135
AT1G67900	4355.838	0.31840764	0.097733	3.257924	1.12E-03	6.69E-03	12.23905	12.19707	12.28227	12.00336	11.97925	11.76783
AT2G35370	1538.971	0.318670659	0.101697	3.133533	1.73E-03	9.69E-03	10.68502	10.70818	10.81922	10.3627	10.54188	10.35124
AT4G34350	2880.258	0.337724944	0.091139	3.705594	0.000211	0.0015457	11.64311	11.61276	11.69727	11.35936	11.37664	11.19875
AT2G07050	1064.139	0.340655513	0.102079	3.337187	8.46E-04	5.23E-03	10.18805	10.25655	10.20498	9.755854	9.979456	9.886522
AT1G19835	1472.893	0.341960617	0.092526	3.695817	0.000219	0.0016014	10.65235	10.7443	10.66181	10.41552	10.33053	10.28201
AT4G10320	1086.124	0.342455876	0.101201	3.38391	0.000715	0.0045251	10.16718	10.33213	10.24302	9.960952	9.845547	9.899787
AT3G11130	2787.278	0.346324855	0.10764	3.217422	0.001293	0.0075832	11.4913	11.6841	11.6478	11.31882	11.10767	11.33891
AT3G02170	1632.197	0.351902237	0.087015	4.044167	5.25E-05	4.44E-04	10.83679	10.81349	10.86443	10.44211	10.56048	10.4563
AT1G07940	4086.44	0.36139717	0.096495	3.745252	1.80E-04	1.34E-03	12.03991	12.16241	12.28941	11.82025	11.78924	11.80103
AT3G14930	1010.964	0.363497301	0.109111	3.331446	0.000864	0.00532	10.14173	10.12224	10.19054	9.805838	9.915862	9.640069
AT1G55360	865.0212	0.367519364	0.11401	3.223577	1.27E-03	7.44E-03	9.877956	9.966309	9.944756	9.394971	9.693718	9.584822
AT3G48930	1147.132	0.369173973	0.109424	3.373779	7.41E-04	4.67E-03	10.34868	10.25847	10.40181	10.04429	10.03039	9.822288
AT4G09010	1862.064	0.374258225	0.115481	3.240876	1.19E-03	7.05E-03	10.96567	10.97692	11.1645	10.59366	10.82181	10.56259
AT4G17390	1924.214	0.378355415	0.117653	3.215853	0.001301	0.0076125	11.11008	10.96402	11.18189	10.79956	10.78274	10.52762
AT2G31410	383.7896	0.383444335	0.12038	3.185288	1.45E-03	8.33E-03	8.784929	8.768114	8.752958	8.386201	8.365965	8.39124
AT5G58140	1653.917	0.385434313	0.110492	3.488341	4.86E-04	0.0032397	10.84207	10.92016	10.86107	10.64795	10.32237	10.4627
AT1G23310	16815.57	0.389927224	0.092139	4.23194	2.32E-05	0.000213	14.18465	14.15685	14.3146	13.89996	13.85542	13.7237
AT2G24020	684.5289	0.391428971	0.108225	3.616809	0.000298	0.0021009	9.559191	9.595502	9.659391	9.119978	9.230722	9.274464
AT3G54660	800.7548	0.392284727	0.100486	3.903883	9.47E-05	7.55E-04	9.808671	9.794996	9.888448	9.47953	9.405534	9.420986
AT1G33240	1135.555	0.396190782	0.113952	3.476827	5.07E-04	3.37E-03	10.26341	10.27123	10.46124	9.862045	10.06337	9.882709
AT3G16000	1046.784	0.397453702	0.105928	3.7521	0.000175	0.0013061	10.17348	10.3156	10.16668	9.738801	9.807115	9.903555
AT4G02920	1171.746	0.398519843	0.105224	3.787335	1.52E-04	0.0011498	10.44777	10.27503	10.4182	10.06951	9.955057	9.9148
AT3G04840	1640.369	0.399622288	0.103042	3.87824	1.05E-04	8.28E-04	10.7557	10.89484	10.9499	10.50734	10.36268	10.51659
AT3G28180	791.8603	0.401242574	0.126645	3.168235	1.53E-03	8.74E-03	9.68296	9.752886	10.00156	9.384044	9.456087	9.402466
AT3G53580	549.9335	0.406757363	0.124147	3.276409	0.001051	0.0063151	9.246788	9.208195	9.426408	8.807449	8.906489	8.938501
AT5G28020	847.593	0.407164431	0.107777	3.77783	1.58E-04	1.19E-03	9.885643	9.861434	10.00677	9.413001	9.55681	9.553989
AT1G22060	1239.253	0.409019887	0.127501	3.207983	1.34E-03	7.79E-03	10.31619	10.61822	10.45998	10.11868	9.879281	10.14549
AT3G57610	517.3779	0.410391776	0.12817	3.201932	0.001365	0.0079379	9.087071	9.193532	9.343688	8.757522	8.753203	8.866973
AT1G08520	1430.845	0.413649919	0.115571	3.579186	0.000345	0.0023866	10.49878	10.70254	10.81179	10.24886	10.30868	10.22292
AT1G71500	1057.892	0.41428592	0.123301	3.359959	7.80E-04	4.87E-03	10.11583	10.18018	10.41167	9.735939	9.923068	9.810295
AT4G03280	5025.365	0.414789858	0.122178	3.394958	0.000686	0.0043722	12.37207	12.39719	12.67826	12.05422	12.19924	11.94875
AT4G15560	6888.779	0.415010252	0.072724	5.706683	1.15E-08	1.84E-07	12.90568	12.9555	12.96807	12.4931	12.56783	12.51985
AT2G23930	440.7001	0.41544254	0.12749	3.25862	1.12E-03	0.0066716	8.909121	8.99568	9.0254	8.478128	8.72049	8.489773

AT5G67220	486.8727	0.418791369	0.130309	3.213839	0.00131	0.007661	9.217311	8.98952	9.156255	8.751866	8.769286	8.591379
AT2G39390	428.8698	0.42159868	0.123138	3.423788	0.000618	0.0039941	8.8666	9.009444	8.958825	8.577394	8.448184	8.524443
AT5G42390	1236.442	0.422517923	0.134768	3.135142	1.72E-03	9.64E-03	10.21948	10.63854	10.5256	10.00679	10.00345	10.11494
AT3G58140	574.4579	0.42371335	0.128968	3.285407	1.02E-03	6.15E-03	9.206441	9.411931	9.469835	8.839799	8.970488	8.999626
AT1G22530	1626.316	0.424477005	0.124469	3.410291	6.49E-04	0.0041719	10.78339	10.84539	10.96068	10.44386	10.61712	10.23493
AT3G19720	787.8469	0.424748559	0.10797	3.933968	8.36E-05	6.78E-04	9.730209	9.879867	9.85512	9.441388	9.30982	9.420986
AT5G02870	1880.761	0.424840683	0.107637	3.946976	7.91E-05	6.46E-04	10.9519	11.08451	11.18495	10.73509	10.54188	10.65713
AT3G51800	629.7764	0.425757347	0.119079	3.575427	0.00035	0.0024198	9.455131	9.560609	9.484855	8.922678	9.084138	9.18751
AT3G49260	387.8246	0.430531394	0.130286	3.304509	9.51E-04	0.0057818	8.768395	8.777083	8.854751	8.436427	8.448184	8.226539
AT4G20362	5321.72	0.431701118	0.130208	3.31548	9.15E-04	5.59E-03	12.3446	12.63058	12.73615	12.00752	12.26877	12.14174
AT4G20360	5311.552	0.432668384	0.130089	3.325939	0.000881	0.0054068	12.34181	12.62847	12.73393	12.00514	12.26381	12.13935
AT1G55490	2136.521	0.432943228	0.078438	5.519527	3.40E-08	5.11E-07	11.24534	11.27064	11.27195	10.85469	10.81989	10.80949
AT2G08770	20248.92	0.43322636	0.107218	4.040595	5.33E-05	4.51E-04	14.58904	14.30318	14.61054	14.09405	14.07227	14.04555
AT5G42650	491.682	0.433581513	0.131124	3.306648	9.44E-04	5.75E-03	9.040396	9.120719	9.271898	8.583775	8.745094	8.78764
AT3G53890	599.7942	0.434121958	0.137341	3.160917	1.57E-03	8.93E-03	9.295044	9.394554	9.588153	9.024699	9.115929	8.8397
AT5G30510	2701.16	0.434700267	0.135434	3.209684	0.001329	0.0077497	11.41987	11.55944	11.80318	10.99176	11.30536	11.17634
AT3G13580	762.4514	0.434723499	0.103944	4.182301	2.89E-05	2.60E-04	9.839778	9.718848	9.771842	9.373033	9.363777	9.28894
AT1G66330	482.0386	0.437354731	0.136299	3.208784	0.001333	0.0077722	8.929095	9.201549	9.212982	8.615264	8.703851	8.712309
AT3G02690	545.9206	0.437482706	0.136421	3.206848	0.001342	0.0078209	9.342978	9.106558	9.426408	8.902401	8.906489	8.762964
AT1G19720	915.416	0.439404974	0.132682	3.311722	9.27E-04	5.66E-03	10.03903	10.2083	9.867589	9.735939	9.538159	9.517574
AT4G10480	808.146	0.439613336	0.119668	3.673602	2.39E-04	1.73E-03	9.843291	9.857211	9.889389	9.221681	9.575223	9.452192
AT3G46780	4798.255	0.439963752	0.110533	3.980391	6.88E-05	0.0005698	12.4287	12.30176	12.5572	12.00573	12.09782	11.85748
AT4G26540	722.2198	0.440541631	0.134322	3.279745	1.04E-03	6.25E-03	9.566648	9.613639	9.90901	9.205221	9.259472	9.306122
AT5G18410	437.721	0.440953901	0.119027	3.704646	2.12E-04	1.55E-03	8.935691	8.983333	9.030527	8.498535	8.516472	8.591379
AT5G22440	541.1229	0.441251047	0.129999	3.394257	0.000688	0.0043823	9.15366	9.354372	9.34917	8.981856	8.761267	8.771237
AT3G55750	674.6838	0.445090398	0.112749	3.94763	7.89E-05	6.44E-04	9.549547	9.649242	9.615708	9.250042	9.183496	9.041265
AT3G55280	367.323	0.447554533	0.141931	3.153332	0.001614	0.0091221	8.753536	8.655908	8.78365	8.457428	8.256129	8.113967
AT4G00300	696.0598	0.44864555	0.1285	3.491395	4.81E-04	3.21E-03	9.783293	9.622623	9.541026	9.316684	9.224903	9.054882
AT2G31750	568.1285	0.449171768	0.115857	3.876956	1.06E-04	8.31E-04	9.368801	9.273026	9.436744	8.839799	8.949469	8.934823
AT5G13280	436.393	0.454413219	0.125499	3.620853	2.94E-04	2.07E-03	8.961781	9.02609	8.960617	8.658224	8.487601	8.423089
AT1G65930	3106.593	0.45573051	0.105496	4.319883	1.56E-05	1.49E-04	11.97013	11.72916	11.72626	11.41321	11.31086	11.33473
AT3G44010	872.0837	0.457630164	0.131501	3.48006	0.000501	0.0033285	10.06709	9.794996	10.06374	9.510004	9.659612	9.3864
AT3G05590	1809.015	0.46149867	0.082492	5.594476	2.21E-08	3.41E-07	11.02834	11.00925	11.06764	10.60468	10.54422	10.56378
AT5G06970	750.4109	0.462681213	0.109469	4.226609	2.37E-05	0.0002175	9.744369	9.777226	9.780998	9.158962	9.363777	9.372874

AT2G37110	525.9599	0.463571534	0.145927	3.176745	1.49E-03	8.53E-03	9.261304	9.109402	9.380296	8.902401	8.884509	8.563072
AT1G69740	1219.632	0.464181605	0.113021	4.107028	4.01E-05	3.49E-04	10.37622	10.42578	10.59185	9.890635	10.10832	9.998216
AT1G09590	770.2613	0.464548859	0.123325	3.766867	1.65E-04	1.24E-03	9.693702	9.837627	9.873308	9.339489	9.48559	9.184416
AT5G64170	562.0495	0.464792284	0.144534	3.215793	0.001301	0.0076125	9.396562	9.478321	9.171808	9.061741	8.728738	8.831812
AT2G35750	20604.05	0.467625705	0.101647	4.600481	4.22E-06	4.50E-05	14.62299	14.36449	14.63782	14.09907	14.07753	14.05108
AT2G31751	600.7184	0.470641585	0.111011	4.239583	2.24E-05	2.07E-04	9.463145	9.376964	9.51077	8.937702	8.970488	9.017122
AT5G66190	4203.308	0.471527246	0.116505	4.047283	5.18E-05	0.0004399	12.09767	12.27827	12.37739	11.86219	11.84437	11.62337
AT2G35040	607.8967	0.471815912	0.114851	4.108066	3.99E-05	3.48E-04	9.416744	9.497981	9.492307	9.115581	8.891872	8.956749
AT3G23400	654.3513	0.47211281	0.126008	3.746694	1.79E-04	1.33E-03	9.444763	9.530166	9.73253	9.057163	9.153175	9.085058
AT5G24300	711.0403	0.472894584	0.135393	3.492748	0.000478	0.0031958	9.516925	9.717917	9.832791	9.057163	9.298783	9.283167
AT4G31700	1602.458	0.473623014	0.105399	4.493633	7.00E-06	7.17E-05	10.71265	10.94066	10.93266	10.35526	10.41217	10.39873
AT4G15770	259.0701	0.47460588	0.148481	3.196408	1.39E-03	8.07E-03	8.278	8.186602	8.277109	7.864069	7.567152	7.819103
AT5G57290	669.2557	0.477004638	0.127602	3.738213	0.000185	0.0013724	9.619844	9.440436	9.754387	9.11117	9.090552	9.175095
AT4G29060	2134.352	0.477729655	0.149716	3.1909	1.42E-03	8.21E-03	11.00148	11.50569	11.29593	10.62493	10.90968	10.84323
AT5G14570	294.914	0.479450037	0.150213	3.191811	1.41E-03	0.0081861	8.516694	8.417493	8.339035	7.821505	8.149577	7.866203
AT5G08130	1082.874	0.481384273	0.143148	3.362834	0.000771	0.0048315	10.2456	10.28638	10.3879	10.00441	9.490449	9.8979
AT5G60600	2563.745	0.481919646	0.119076	4.047167	5.18E-05	0.0004399	11.32382	11.64716	11.6478	11.00133	11.09656	11.08306
AT2G28470	655.9532	0.482840967	0.153781	3.139803	1.69E-03	9.51E-03	9.562392	9.649242	9.52416	9.038701	9.384807	8.819899
AT5G40450	8109.909	0.483153533	0.142653	3.386909	0.000707	0.0044847	13.20731	13.44575	12.9321	12.66318	12.63684	12.84925
AT4G25960	832.3889	0.483242352	0.103369	4.674921	2.94E-06	3.26E-05	10.01041	9.8807	9.879951	9.42015	9.470914	9.428851
AT4G33220	510.2295	0.483603894	0.116456	4.152674	3.29E-05	0.0002917	9.22541	9.166486	9.276222	8.779925	8.635307	8.771237
AT2G27710	867.8318	0.483959709	0.10077	4.802594	1.57E-06	1.81E-05	9.925958	9.97413	10.05705	9.499917	9.470914	9.522483
AT1G63660	240.8179	0.48469438	0.149461	3.242942	1.18E-03	7.01E-03	8.056815	8.194656	8.163456	7.697535	7.707307	7.557346
AT3G12345	1087.434	0.484858993	0.130485	3.715832	2.03E-04	1.49E-03	10.24693	10.23913	10.4357	9.707002	10.03705	9.70628
AT5G02840	1819.397	0.485046275	0.14244	3.40526	6.61E-04	4.24E-03	11.25394	10.84496	11.0357	10.70468	10.59473	10.37314
AT2G47240	317.5611	0.485922755	0.139429	3.485083	0.000492	0.0032751	8.653039	8.517206	8.445238	8.013353	8.034523	8.09432
AT2G17840	890.6471	0.485935653	0.141042	3.445326	0.00057	0.0037312	10.17348	10.09309	9.784037	9.423711	9.509722	9.653557
AT1G63090	317.5325	0.486134724	0.135416	3.589947	3.31E-04	0.0023026	8.585428	8.451502	8.584226	8.09603	7.980286	8.06097
AT3G55800	3153.031	0.486897524	0.141003	3.453112	0.000554	0.0036437	11.74897	11.81614	11.97081	11.07452	11.57609	11.37328
AT4G35800	1246.222	0.487141664	0.139253	3.498245	4.68E-04	3.14E-03	10.32312	10.64296	10.55021	10.18943	9.803215	10.02614
AT5G16010	435.6017	0.488569762	0.125309	3.898926	9.66E-05	0.0007682	9.064675	8.931259	8.981948	8.400731	8.60874	8.499764
AT4G27720	257.3126	0.488899304	0.155232	3.149485	1.64E-03	9.23E-03	8.189692	8.234269	8.285712	7.509239	7.909492	7.794964
AT3G44480	594.1986	0.489325096	0.115446	4.238556	2.25E-05	2.07E-04	9.452834	9.515236	9.358714	9.029381	8.877107	8.934823
AT2G26250	2454.437	0.490522973	0.120247	4.079288	4.52E-05	3.89E-04	11.65366	11.30918	11.48022	11.05513	11.0347	10.88481

AT5G08650	623.2443	0.490844124	0.110485	4.442625	8.89E-06	8.89E-05	9.462003	9.494723	9.577683	8.942675	9.04504	9.061642
AT1G08930	1152.971	0.490943834	0.143339	3.425065	0.000615	0.0039784	10.5367	10.20963	10.43441	10.11868	9.811005	9.74879
AT3G55620	435.5	0.494378738	0.132309	3.73654	1.87E-04	1.38E-03	8.988993	8.908585	9.095576	8.371524	8.497289	8.609947
AT4G39350	1198.873	0.49473906	0.113456	4.360627	1.30E-05	1.26E-04	10.30476	10.54974	10.50119	9.882893	10.04698	9.942534
AT2G43410	304.0104	0.495122023	0.148608	3.331726	8.63E-04	5.32E-03	8.42824	8.484728	8.548929	7.984706	7.756543	8.146128
AT2G33850	658.238	0.495906086	0.138334	3.584847	3.37E-04	2.34E-03	9.673124	9.67346	9.410764	8.986679	9.276451	9.00665
AT3G62310	478.503	0.496011511	0.114079	4.347983	1.37E-05	1.33E-04	9.169138	9.112239	9.123038	8.658224	8.581674	8.655347
AT1G18730	687.4452	0.497623287	0.139755	3.560675	3.70E-04	2.55E-03	9.462003	9.695387	9.793117	8.981856	9.253768	9.212026
AT5G08280	1235.435	0.49783765	0.144034	3.456393	5.47E-04	0.0036052	10.27711	10.46515	10.728	9.859417	10.10516	10.01921
AT3G53870	1631.561	0.498587506	0.089727	5.556722	2.75E-08	4.18E-07	10.82795	10.92984	10.94312	10.40655	10.43504	10.36223
AT4G34190	804.7596	0.498867142	0.153639	3.247002	1.17E-03	0.006918	9.789679	9.783471	10.05789	9.192751	9.646611	9.265708
AT2G16500	329.0862	0.50018059	0.150214	3.329794	8.69E-04	5.34E-03	8.545013	8.711231	8.541764	8.264377	7.865285	8.100899
AT2G24050	266.3763	0.500245533	0.156221	3.202162	1.36E-03	7.93E-03	8.136283	8.389696	8.344535	7.915566	7.723906	7.711452
AT5G65460	239.8091	0.501428449	0.158763	3.15834	0.001587	0.0090004	8.004436	8.215918	8.197287	7.482168	7.740317	7.676644
AT2G36170	575.342	0.50244821	0.125389	4.007127	6.15E-05	0.0005139	9.456279	9.310571	9.436744	9.052569	8.808725	8.807886
AT5G53580	348.3518	0.503804947	0.126373	3.986653	6.70E-05	5.56E-04	8.657037	8.677143	8.710993	8.157211	8.137237	8.214456
AT4G33110	239.5831	0.505867895	0.146572	3.451331	5.58E-04	3.67E-03	8.127666	8.159423	8.138345	7.522586	7.723906	7.649977
AT4G27430	1027.712	0.506642496	0.153539	3.299768	0.000968	0.0058698	9.952223	10.42635	10.30216	9.556165	9.85688	9.752973
AT1G29850	414.7315	0.506793225	0.129296	3.919637	8.87E-05	7.16E-04	8.940619	8.9151	8.946219	8.25641	8.448184	8.53905
AT4G07410	542.1997	0.5068349	0.144812	3.499941	4.65E-04	3.13E-03	9.415564	9.293172	9.255935	8.981856	8.506912	8.870827
AT2G44210	852.6295	0.507104913	0.121155	4.185597	2.84E-05	0.0002568	9.954661	9.916854	10.03426	9.262029	9.606891	9.492776
AT1G12500	210.9386	0.507634673	0.157102	3.231244	1.23E-03	7.26E-03	7.966283	7.888597	8.016154	7.368501	7.585434	7.395531
AT4G08520	265.571	0.508132336	0.161802	3.14046	0.001687	0.0094867	8.116097	8.392033	8.352746	7.600176	7.894906	7.827061
AT1G79790	417.1042	0.510534418	0.130266	3.919159	8.89E-05	0.0007176	8.822788	8.967751	9.030527	8.498535	8.386963	8.39124
AT4G17270	328.2421	0.511269319	0.150196	3.404025	0.000664	0.0042507	8.624742	8.515063	8.652331	8.295811	7.994038	7.926702
AT3G63130	384.1053	0.511414616	0.149283	3.425816	6.13E-04	0.0039694	8.690576	8.798382	8.969543	8.113777	8.428064	8.364152
AT1G48355	528.568	0.51195719	0.15938	3.212185	0.001317	0.0076975	9.095932	9.21613	9.504641	8.740488	8.949469	8.596044
AT3G05560	811.0274	0.511994442	0.100622	5.088317	3.61E-07	4.64E-06	9.874527	9.887341	9.933851	9.427264	9.415787	9.314637
AT5G59850	546.7141	0.512142147	0.115634	4.428995	9.47E-06	9.44E-05	9.306541	9.359158	9.321548	8.740488	8.949469	8.762964
AT4G34530	536.5038	0.512283706	0.152397	3.361505	0.000775	0.0048524	9.098874	9.376964	9.415997	8.609021	8.998046	8.737859
AT3G22320	512.5219	0.513274548	0.130997	3.91821	8.92E-05	0.0007195	9.334267	9.159644	9.230911	8.615264	8.644055	8.878505
AT5G61790	763.8155	0.515863905	0.160879	3.206543	0.001343	0.0078259	9.811364	10.01488	9.605436	9.409413	8.998046	9.420986
AT4G01460	280.7136	0.516412185	0.146566	3.523412	0.000426	0.0028839	8.397507	8.334874	8.398412	8.03214	7.723906	7.778644
AT2G23120	941.6018	0.516540939	0.153217	3.371294	0.000748	0.0047085	10.20658	9.96866	10.16978	9.262029	9.75153	9.721301

AT3G50820	7749.044		0.516667509	0.162644	3.176682	1.49E-03	8.53E-03	12.88036	13.11369	13.43035	12.55042	12.85975	12.45777
AT5G42020	621.0793		0.517132525	0.157047	3.292859	9.92E-04	0.0060018	9.562392	9.67538	9.307536	8.981856	8.761267	9.193678
AT2G43375	1245.013		0.521027221	0.140267	3.714527	2.04E-04	1.49E-03	10.49878	10.61172	10.45617	9.709922	10.03372	10.19706
AT5G02565	7014.663		0.521054796	0.134867	3.863474	0.000112	0.0008739	13.07126	12.84181	13.11981	12.58304	12.62031	12.23254
AT1G17980	380.7413		0.52106474	0.127826	4.076347	4.57E-05	0.0003933	8.808483	8.833196	8.813702	8.207663	8.233121	8.407252
AT1G04240	660.0698		0.521530889	0.145451	3.585604	3.36E-04	2.34E-03	9.720691	9.480518	9.607725	8.850423	9.30982	9.054882
AT4G13180	298.4838		0.521569518	0.15075	3.459822	0.000541	0.0035623	8.451442	8.504302	8.429798	8.087074	8.007659	7.711452
AT4G36360	443.2176		0.522143476	0.125911	4.146919	3.37E-05	2.98E-04	9.014145	9.093694	8.990744	8.364129	8.599774	8.553511
AT3G16780	334.2666		0.522196893	0.153428	3.403526	0.000665	0.0042574	8.501212	8.798382	8.560791	8.068994	8.244671	7.991862
AT1G58440	371.1618		0.522300441	0.158068	3.304274	0.000952	0.0057845	8.721446	8.75183	8.850891	7.994319	8.535404	8.189982
AT4G26095	221.6056		0.522571192	0.154067	3.39185	0.000694	0.0044177	8.041606	7.956488	8.112788	7.58753	7.491614	7.448074
AT1G05137	1602.71		0.524078947	0.141498	3.703802	0.000212	0.0015544	11.07652	10.70865	10.86011	10.23271	10.25544	10.55543
AT3G21250	533.6333		0.524341722	0.160112	3.274837	0.001057	0.0063429	9.236139	9.550184	9.095576	8.823715	8.599774	8.866973
AT4G08470	299.6838		0.524415266	0.165845	3.162071	1.57E-03	8.90E-03	8.562168	8.349377	8.512746	8.191041	7.673524	7.89677
AT4G01690	972.9604		0.524947186	0.137434	3.819642	1.34E-04	1.03E-03	10.04209	10.16523	10.28367	9.741657	9.759603	9.391775
AT5G49030	328.4769		0.524984439	0.164725	3.187041	1.44E-03	8.29E-03	8.395116	8.773502	8.634482	7.95548	7.994038	8.238522
AT1G41880	702.8855		0.525215965	0.118952	4.415378	1.01E-05	1.00E-04	9.635124	9.809929	9.643862	9.052569	9.270813	9.184416
AT3G09200	4260.872		0.525896213	0.126402	4.160498	3.18E-05	0.0002828	12.18141	12.26015	12.44264	11.96721	11.66474	11.64548
AT5G40950	1710.892		0.526340557	0.127631	4.123913	3.72E-05	3.27E-04	10.81593	10.93025	11.17688	10.38113	10.56969	10.3974
AT2G09735	245.3561		0.526437371	0.162456	3.240483	1.19E-03	7.06E-03	8.346437	8.024343	8.166565	7.600176	7.740317	7.622806
AT3G16800	561.4474		0.527992437	0.107532	4.91011	9.10E-07	1.10E-05	9.398951	9.380499	9.350538	8.850423	8.847114	8.8397
AT1G76100	2244.934		0.528294902	0.163179	3.237514	1.21E-03	7.13E-03	11.27718	11.2735	11.55863	10.75643	11.14666	10.55543
AT3G15010	436.416		0.530530058	0.133883	3.962637	7.41E-05	0.0006101	9.018812	9.079265	8.955235	8.326574	8.448184	8.637358
AT2G29650	606.2264		0.532527276	0.127672	4.17106	3.03E-05	2.72E-04	9.362695	9.457272	9.634912	8.866214	9.025087	8.963984
AT1G68830	846.4512		0.532895343	0.110066	4.841619	1.29E-06	1.51E-05	9.934217	9.963955	10.00417	9.358221	9.58888	9.353722
AT5G14210	245.3317		0.533325271	0.170681	3.124683	1.78E-03	9.95E-03	8.375841	8.068843	8.106327	7.821505	7.567152	7.538084
AT2G21330	5321.936		0.536048278	0.135412	3.958633	7.54E-05	0.0006186	12.56976	12.53098	12.76015	12.24998	12.14712	11.8121
AT2G26910	1320.917		0.537600546	0.117142	4.589313	4.45E-06	4.72E-05	10.66729	10.65322	10.52136	10.23473	9.904985	10.05183
AT4G20070	476.6268		0.537724704	0.122402	4.393105	1.12E-05	0.0001099	9.081132	9.106558	9.247152	8.652165	8.544777	8.605328
AT5G53370	342.1428		0.538318701	0.155543	3.460908	5.38E-04	0.003549	8.684714	8.63238	8.680874	8.318945	8.185975	7.842845
AT3G04350	350.7054		0.538349842	0.136398	3.946901	7.92E-05	0.0006458	8.808483	8.624451	8.674337	8.059868	8.209741	8.208377
AT1G12900	5302.241		0.538510769	0.138859	3.878116	1.05E-04	8.28E-04	12.44599	12.60108	12.79069	11.96415	12.29955	11.93086
AT3G24170	499.9461		0.539458206	0.154212	3.498149	4.68E-04	3.14E-03	9.037333	9.451682	9.131016	8.757522	8.55409	8.681917
AT3G56060	317.096		0.540132976	0.135389	3.989503	6.62E-05	0.0005499	8.518892	8.546873	8.609573	8.104931	8.021154	7.919277

AT1G78850	941.2736	0.54263566	0.09909	5.476172	4.35E-08	6.43E-07	10.14601	10.05304	10.17828	9.559406	9.615812	9.570674
AT1G17220	1454.637	0.544202824	0.116472	4.672407	2.98E-06	3.29E-05	10.60606	10.91854	10.72484	10.24685	10.2237	10.15025
AT2G06520	14961.28	0.544954589	0.166228	3.278349	1.04E-03	6.28E-03	14.00253	13.94331	14.37589	13.44142	13.85589	13.34387
AT3G23940	354.0612	0.545827273	0.134841	4.047926	5.17E-05	4.39E-04	8.694471	8.768114	8.715245	8.224096	7.980286	8.26805
AT3G52960	574.6838	0.548034937	0.173214	3.163925	1.56E-03	8.85E-03	9.256042	9.29567	9.668191	8.676251	9.134672	8.746276
AT4G30620	180.9157	0.548538616	0.174824	3.137663	0.001703	0.0095672	7.869688	7.709057	7.671314	7.162256	7.391266	7.059624
AT1G63360	263.8891	0.549220944	0.16323	3.364705	7.66E-04	4.80E-03	8.331507	8.399021	8.178932	7.915566	7.472095	7.786827
AT3G26710	475.1931	0.550234555	0.130088	4.2297	2.34E-05	2.15E-04	9.106203	9.218766	9.119834	8.450462	8.55409	8.742073
AT1G05057	2114.555	0.550316356	0.166531	3.304582	9.51E-04	5.78E-03	11.17599	11.21266	11.49297	10.40475	10.72412	11.02025
AT1G05063	2114.555	0.550316356	0.166531	3.304582	9.51E-04	0.0057818	11.17599	11.21266	11.49297	10.40475	10.72412	11.02025
AT2G02800	244.0477	0.550558194	0.164723	3.342319	8.31E-04	5.16E-03	8.083793	8.202666	8.294263	7.574773	7.432247	7.827061
AT5G35170	914.5126	0.550711775	0.127251	4.327746	1.51E-05	1.45E-04	9.912645	10.13764	10.20422	9.398595	9.584342	9.6081
AT3G16080	556.7783	0.552295866	0.135913	4.063587	4.83E-05	4.14E-04	9.334267	9.367495	9.415997	8.967288	8.891872	8.577295
AT5G22060	425.1951	0.552363387	0.171935	3.212636	1.32E-03	0.0076879	9.22541	8.947238	8.765313	8.609021	8.197907	8.433551
AT2G16280	795.8472	0.554165368	0.109348	5.0679	4.02E-07	5.13E-06	9.926786	9.868164	9.866634	9.339489	9.451111	9.208984
AT5G58870	523.7474	0.55458439	0.135736	4.08575	4.39E-05	3.79E-04	9.145147	9.431375	9.280532	8.633832	8.695459	8.8397
AT3G13230	239.4039	0.555392865	0.156991	3.537741	4.04E-04	0.0027478	8.175828	8.140089	8.194244	7.709256	7.327526	7.702828
AT1G78100	191.3075	0.556638329	0.171034	3.25454	1.14E-03	6.75E-03	7.866239	7.731377	7.905155	7.179211	7.491614	7.163834
AT1G22140	153.1567	0.557478514	0.174054	3.202914	1.36E-03	7.92E-03	7.476068	7.521138	7.571658	7.03764	6.92986	6.90281
AT1G07510	341.7455	0.558047726	0.141302	3.94934	7.84E-05	6.40E-04	8.577014	8.77887	8.672152	8.224096	7.966403	8.120457
AT3G01480	990.7534	0.558146814	0.124411	4.486307	7.25E-06	7.40E-05	10.07009	10.16796	10.36845	9.539848	9.743412	9.646829
AT3G47650	623.5311	0.558681518	0.143087	3.904487	9.44E-05	7.54E-04	9.398951	9.447194	9.749213	8.922678	9.103296	8.901297
AT3G13330	441.178	0.558693506	0.160431	3.482448	4.97E-04	3.30E-03	8.892263	9.16785	9.070903	8.694056	8.173944	8.499764
AT1G21270	508.7543	0.558870533	0.161025	3.470712	0.000519	0.0034336	9.178902	9.210845	9.360073	8.977016	8.40766	8.591379
AT3G01810	207.5402	0.558990695	0.171499	3.259449	0.001116	0.0066583	7.84537	8.015276	8.02303	7.179211	7.391266	7.566881
AT1G62750	1900.085	0.559142114	0.108181	5.168557	2.36E-07	3.13E-06	11.0769	11.11807	11.23726	10.49554	10.73645	10.51166
AT1G05560	585.5866	0.559395767	0.157748	3.546143	3.91E-04	2.67E-03	9.265237	9.624611	9.443167	8.615264	9.031769	8.97477
AT1G05562	585.5866	0.559395767	0.157748	3.546143	3.91E-04	0.0026708	9.265237	9.624611	9.443167	8.615264	9.031769	8.97477
AT5G02010	174.0472	0.56027698	0.167679	3.341373	8.34E-04	5.17E-03	7.758737	7.686387	7.671314	7.245106	7.142352	7.032351
AT2G20410	4966.068	0.56072631	0.137019	4.09232	4.27E-05	0.0003696	12.55564	12.42542	12.61737	11.76587	11.8651	12.22428
AT5G45775	750.0573	0.561653517	0.099585	5.639932	1.70E-08	2.66E-07	9.835375	9.783471	9.803138	9.266002	9.242291	9.221112
AT1G51590	193.0561	0.561787556	0.161208	3.48486	4.92E-04	3.28E-03	7.813489	7.946983	7.80897	7.245106	7.283413	7.329843
AT1G62960	417.0073	0.562208969	0.160553	3.501711	4.62E-04	3.11E-03	8.948795	8.755465	9.153124	8.386201	8.563343	8.226539
AT3G62530	1007.848	0.562769094	0.107224	5.24852	1.53E-07	2.09E-06	10.12596	10.24237	10.32599	9.600894	9.722914	9.677961

AT3G15210	300.3187	0.562931344	0.174795	3.220532	1.28E-03	0.0075083	8.696414	8.272822	8.485623	8.113777	7.756543	7.858459
AT1G28380	293.5991	0.563111176	0.177645	3.169864	1.53E-03	0.0087067	8.632884	8.305422	8.422015	8.139994	7.835043	7.649977
AT5G13370	197.9895	0.563114424	0.172856	3.257709	0.001123	0.00669	7.784546	8.05711	7.824797	7.440585	7.283413	7.237331
AT1G33811	306.0267	0.563940489	0.178645	3.156769	0.001595	0.0090368	8.678827	8.280411	8.565508	7.709256	8.185975	7.919277
AT1G50460	189.1457	0.56548484	0.167355	3.378954	7.28E-04	4.59E-03	7.740014	7.868599	7.871262	7.397768	7.237908	7.125632
AT1G19130	236.3708	0.566313436	0.157295	3.60032	3.18E-04	2.22E-03	8.285767	8.027353	8.125623	7.561902	7.567152	7.595115
AT3G58670	177.9307	0.567100801	0.179419	3.160769	1.57E-03	8.93E-03	7.869688	7.667219	7.697339	7.353641	6.92986	7.163834
AT2G04955	461.0309	0.56840279	0.13775	4.126345	3.69E-05	3.23E-04	8.96662	9.126344	9.227938	8.670267	8.477847	8.449103
AT3G27925	406.588	0.568685099	0.168014	3.384757	0.000712	0.0045134	8.744172	8.793086	9.211477	8.422254	8.323029	8.308399
AT3G16370	3414.055	0.56869101	0.149539	3.802964	1.43E-04	1.09E-03	11.86411	11.82719	12.26025	11.24122	11.58861	11.41083
AT2G29450	622.8322	0.568731446	0.140987	4.033918	5.49E-05	4.63E-04	9.604401	9.621627	9.394948	9.171726	8.899199	8.811901
AT3G17040	1064.151	0.570105452	0.104306	5.465689	4.61E-08	6.80E-07	10.28424	10.35507	10.30781	9.872507	9.655291	9.684546
AT5G66590	675.419	0.572338647	0.156546	3.65603	0.000256	0.0018386	9.639171	9.497981	9.825927	9.171726	9.248041	8.799822
AT1G32470	1561.721	0.574262481	0.12065	4.759739	1.94E-06	2.21E-05	10.76637	10.78176	11.04417	10.25487	10.40447	10.21081
AT4G21210	400.81	0.577132011	0.17285	3.338916	8.41E-04	0.0052014	8.587524	9.015519	9.089038	8.199376	8.438159	8.336545
AT2G18730	270.6523	0.577421387	0.14055	4.108295	3.99E-05	3.47E-04	8.338991	8.320223	8.374416	7.743857	7.865285	7.694153
AT1G51805	307.6723	0.578042533	0.165628	3.49001	4.83E-04	3.22E-03	8.666982	8.471529	8.434963	8.013353	8.137237	7.676644
AT3G06700	1068.734	0.57888771	0.099042	5.844848	5.07E-09	8.54E-08	10.30476	10.27566	10.38989	9.797627	9.759603	9.671346
AT1G04350	316.1972	0.579755891	0.139898	4.144138	3.41E-05	0.0003011	8.595878	8.486916	8.629985	8.104931	7.952384	7.89677
AT3G05350	462.0434	0.579944323	0.15747	3.682889	0.000231	0.0016753	8.950425	9.20554	9.197869	8.326574	8.45814	8.75881
AT2G15290	286.577	0.580055879	0.163804	3.541159	3.98E-04	0.0027153	8.262341	8.394366	8.627731	7.832265	7.723906	7.948751
AT4G09900	175.0205	0.580467144	0.165872	3.49948	4.66E-04	0.0031308	7.666567	7.716535	7.788938	7.195969	7.09209	7.112669
AT1G68000	190.2598	0.581927872	0.159671	3.644537	2.68E-04	1.91E-03	7.869688	7.817354	7.855939	7.292624	7.142352	7.307265
AT5G59730	273.4208	0.582505378	0.14707	3.960725	7.47E-05	6.14E-04	8.373413	8.29547	8.437538	7.821505	7.603488	7.866203
AT4G28740	167.4783	0.583090994	0.173763	3.355665	7.92E-04	4.94E-03	7.662596	7.596027	7.735517	6.941541	7.040014	7.201051
AT4G27080	154.617	0.584453727	0.181705	3.216491	1.30E-03	0.0076009	7.630427	7.414827	7.60412	7.018926	6.810594	7.004552
AT4G19710	300.6135	0.584771395	0.159923	3.656583	2.56E-04	1.84E-03	8.521087	8.484728	8.517623	7.685719	7.819681	8.158795
AT4G17300	235.0651	0.585582937	0.154289	3.795361	1.47E-04	1.12E-03	8.02314	8.175792	8.239227	7.574773	7.567152	7.528356
AT1G21350	213.1859	0.585695079	0.183582	3.190377	1.42E-03	8.22E-03	7.99183	7.838071	8.169667	7.179211	7.656331	7.384788
AT4G28750	15218.81	0.586062036	0.161127	3.63726	2.76E-04	1.96E-03	14.02019	13.95876	14.45358	13.39205	13.79066	13.47974
AT1G27330	178.6453	0.586855991	0.179279	3.273414	1.06E-03	6.37E-03	7.903732	7.671073	7.693034	6.999965	7.142352	7.307265
AT5G59732	273.1108	0.587801229	0.148023	3.971011	7.16E-05	0.0005908	8.375841	8.29547	8.437538	7.821505	7.585434	7.866203
AT5G46800	409.6007	0.589146593	0.147583	3.991971	6.55E-05	0.0005447	8.938978	8.757278	9.12783	8.311275	8.386963	8.358672
AT5G57800	562.9071	0.589380373	0.108698	5.422184	5.89E-08	8.55E-07	9.410837	9.424543	9.378956	8.768767	8.854671	8.815906

AT4G25700	232.8258	0.589433837	0.180984	3.256835	1.13E-03	6.71E-03	8.05075	7.950158	8.377102	7.412182	7.638931	7.557346
AT5G08720	218.0971	0.589502431	0.181187	3.253556	1.14E-03	0.0067744	8.047708	8.074674	8.040079	7.397768	7.09209	7.728545
AT2G29180	215.6046	0.590596089	0.163184	3.619213	2.96E-04	2.08E-03	7.988661	8.003097	8.08677	7.195969	7.548635	7.518562
AT5G47070	221.5114	0.591535272	0.180596	3.275459	0.001055	0.0063306	7.99183	8.210632	7.988316	7.732415	7.349087	7.272722
AT5G26030	252.3174	0.591632628	0.15358	3.852287	1.17E-04	0.0009123	8.251806	8.14564	8.363621	7.743857	7.529878	7.66781
AT4G02970	9091.69	0.592027292	0.162205	3.649875	2.62E-04	0.0018753	13.54039	13.19265	13.50408	12.67144	13.12915	12.59313
AT2G34357	392.9927	0.592554825	0.169394	3.498085	0.000469	0.0031423	8.838714	9.073452	8.761207	8.207663	8.047769	8.543887
AT1G30690	636.9085	0.592941044	0.181949	3.258837	1.12E-03	0.0066681	9.244133	9.75833	9.70607	8.711644	9.207302	8.992568
AT5G10470	352.991	0.592958732	0.171166	3.464238	5.32E-04	3.51E-03	8.645011	8.9151	8.654547	8.078063	7.865285	8.385863
AT1G29040	185.8892	0.593617493	0.186785	3.178078	0.001483	0.0085016	7.824194	7.583812	8.012703	7.276958	7.190921	7.163834
AT4G00430	1374.241	0.5938793	0.113027	5.254299	1.49E-07	2.03E-06	10.56994	10.70818	10.7913	10.13615	10.18226	9.964344
AT5G03120	268.5792	0.593886302	0.186395	3.186172	0.001442	0.0083118	8.3365	8.285448	8.390457	7.495767	8.137237	7.528356
AT5G15750	179.8269	0.59585711	0.170766	3.489322	0.000484	0.0032303	7.777219	7.778582	7.772711	7.074357	7.013254	7.352073
AT1G47500	244.5993	0.596792024	0.14611	4.084537	4.42E-05	3.81E-04	8.211602	8.257524	8.154091	7.625138	7.656331	7.547747
AT2G34070	361.9485	0.596866009	0.138527	4.308656	1.64E-05	0.0001562	8.790399	8.63238	8.887146	8.174225	8.185975	8.152475
AT1G64680	382.1022	0.598120648	0.130513	4.582839	4.59E-06	4.85E-05	8.804884	8.87222	8.871992	8.356696	8.267496	8.120457
AT1G48630	275.0651	0.598616087	0.155482	3.850055	1.18E-04	9.20E-04	8.203425	8.435731	8.490592	7.842944	7.707307	7.762137
AT2G24090	1139.864	0.598769465	0.135353	4.42375	9.70E-06	9.65E-05	10.29646	10.34425	10.61763	9.659462	9.96207	9.830228
AT1G26880	653.8483	0.599160863	0.114903	5.214482	1.84E-07	2.49E-06	9.547395	9.629571	9.693194	9.11117	9.025087	8.92744
AT3G44620	237.0564	0.600800468	0.186552	3.220548	1.28E-03	0.0075083	7.985486	8.148407	8.355472	7.26112	7.621318	7.737017
AT5G44870	266.286	0.600850347	0.190459	3.154755	1.61E-03	9.09E-03	8.566426	8.275356	8.144664	7.984706	7.452309	7.66781
AT2G38170	2802.361	0.602457883	0.107296	5.614916	1.97E-08	3.05E-07	11.72917	11.59917	11.83625	11.22906	11.08696	11.02976
AT3G11710	667.4992	0.602462333	0.132477	4.547669	5.42E-06	5.65E-05	9.467704	9.817773	9.667094	9.075391	9.01163	9.054882
AT2G26710	305.4326	0.602507603	0.135313	4.4527	8.48E-06	8.52E-05	8.581227	8.510769	8.495545	7.864069	8.007659	7.904311
AT2G47450	2225.601	0.602585966	0.160064	3.764652	0.000167	0.0012476	11.19123	11.60144	11.36046	10.93799	10.89876	10.46525
AT5G50900	296.3137	0.6043702	0.148077	4.081464	4.48E-05	3.86E-04	8.49005	8.553151	8.40895	7.97503	7.966403	7.694153
AT5G49730	2280.293	0.604453953	0.127087	4.75623	1.97E-06	2.24E-05	11.41102	11.40457	11.47177	11.06086	10.66518	10.69132
AT4G25370	530.4023	0.604737627	0.135989	4.44696	8.71E-06	8.73E-05	9.195489	9.291921	9.474859	8.602751	8.847114	8.69502
AT3G11670	679.0302	0.606112663	0.111684	5.427039	5.73E-08	8.33E-07	9.643208	9.743765	9.664897	9.001054	9.025087	9.175095
AT4G24090	148.417	0.606363328	0.191184	3.171627	0.001516	0.0086637	7.363149	7.468962	7.635867	6.901235	7.040014	6.709736
AT5G24120	209.4814	0.606556213	0.189474	3.201266	1.37E-03	7.95E-03	8.130544	7.927782	7.916278	6.999965	7.491614	7.566881
AT3G57660	310.8823	0.607837443	0.183546	3.311633	9.28E-04	5.66E-03	8.523279	8.801901	8.336277	8.022777	7.656331	8.087712
AT3G56650	308.9399	0.608390512	0.176153	3.453755	5.53E-04	3.64E-03	8.416497	8.433463	8.767362	7.777648	8.185975	7.819103
AT5G64050	445.6573	0.608475901	0.122047	4.985605	6.18E-07	7.65E-06	9.038865	9.138922	9.049173	8.371524	8.535404	8.484752

AT1G30380	12220.67	0.608507987	0.165925	3.667375	2.45E-04	1.77E-03	13.70165	13.67726	14.13627	13.03961	13.50066	13.11786
AT5G65470	399.8237	0.608725324	0.167992	3.62353	0.000291	0.0020521	8.856206	8.703726	9.177982	8.280179	8.185975	8.423089
AT3G57000	228.0356	0.609408281	0.171624	3.550827	0.000384	0.0026328	8.041606	8.260085	8.026456	7.26112	7.673524	7.538084
AT3G55330	399.9909	0.609771847	0.158498	3.847184	1.19E-04	9.30E-04	8.81744	8.900401	9.035637	8.03214	8.55409	8.296986
AT2G36620	673.2587	0.611548376	0.122857	4.977736	6.43E-07	7.95E-06	9.531151	9.689699	9.782012	9.047962	9.140866	8.981915
AT3G58790	283.2406	0.611703379	0.177836	3.439701	5.82E-04	0.0037971	8.334006	8.249814	8.658968	7.810665	7.980286	7.622806
AT1G49700	1597.957	0.611956748	0.191664	3.192862	1.41E-03	8.16E-03	11.17738	10.49427	11.0104	10.28063	10.53719	10.02614
AT4G32190	331.0728	0.612772308	0.152602	4.015498	5.93E-05	0.0004978	8.680792	8.7609	8.517623	7.925648	7.923931	8.208377
AT2G25830	146.587	0.612961697	0.182036	3.367253	0.000759	0.0047675	7.382594	7.516861	7.53845	6.941541	6.900955	6.743763
AT3G47560	255.2872	0.61353851	0.149586	4.101577	4.10E-05	3.57E-04	8.306275	8.1649	8.352746	7.661791	7.723906	7.595115
AT1G68660	360.7301	0.613938864	0.128464	4.779092	1.76E-06	2.01E-05	8.727161	8.771708	8.829478	8.09603	8.149577	8.214456
AT3G44890	1007.252	0.614512754	0.128775	4.771986	1.82E-06	2.08E-05	10.12307	10.21294	10.40839	9.610301	9.783555	9.502747
AT3G02830	224.3416	0.61560334	0.176305	3.491705	0.00048	0.0032066	8.219733	7.908323	8.144664	7.673804	7.327526	7.373965
AT3G53260	483.1619	0.61658057	0.134141	4.596525	4.30E-06	4.57E-05	9.246788	9.196209	9.142112	8.746188	8.572538	8.39124
AT5G44520	240.6151	0.616933287	0.15219	4.053708	5.04E-05	4.30E-04	8.136283	8.1649	8.288568	7.495767	7.529878	7.66781
AT4G03550	513.2903	0.619427841	0.147704	4.193699	2.74E-05	2.49E-04	9.262616	9.418824	9.167159	8.881834	8.544777	8.519541
AT4G17740	213.3664	0.620841211	0.168738	3.679314	2.34E-04	1.70E-03	7.859316	8.042308	8.147813	7.353641	7.529878	7.307265
AT5G06980	966.8743	0.621150303	0.133818	4.64174	3.45E-06	3.77E-05	10.41274	10.06261	10.09181	9.546397	9.650957	9.515113
AT5G38520	435.4444	0.621511621	0.139418	4.457898	8.28E-06	8.33E-05	8.890566	9.042547	9.197869	8.464361	8.448184	8.353172
AT3G02570	383.6306	0.622915573	0.16318	3.817345	0.000135	0.0010332	8.907444	8.727978	8.957031	8.471261	8.233121	7.970467
AT4G13840	309.3585	0.622956169	0.16498	3.775949	1.59E-04	1.20E-03	8.503434	8.780654	8.363621	7.994319	7.894906	7.889188
AT5G40160	193.0103	0.624419974	0.192239	3.24815	1.16E-03	0.0068965	7.622271	7.905054	8.08677	7.09237	7.327526	7.307265
AT5G02760	3890.647	0.625300577	0.14527	4.304401	1.67E-05	1.59E-04	12.07189	12.12162	12.40809	11.43366	11.81755	11.43966
AT4G28400	248.3162	0.626305334	0.161805	3.870741	0.000109	0.0008498	8.395116	8.092027	8.239227	7.685719	7.491614	7.63192
AT1G14580	190.2185	0.628380173	0.185597	3.385715	0.00071	0.0044998	7.60995	7.953326	7.991826	7.245106	7.305638	7.125632
AT3G29370	357.5328	0.629054323	0.200269	3.141046	0.001683	0.0094698	8.600037	8.777083	8.918829	8.478128	8.099571	7.711452
AT2G37990	226.7347	0.629363716	0.150607	4.178838	2.93E-05	2.64E-04	8.144849	8.05711	8.135175	7.522586	7.510873	7.406194
AT5G48300	1227.405	0.629459086	0.171783	3.664262	0.000248	0.0017899	10.2509	10.66824	10.68584	9.578703	10.01023	10.07874
AT4G22890	2019.287	0.630713823	0.112481	5.607302	2.06E-08	3.18E-07	11.12278	11.21925	11.43066	10.60625	10.67161	10.60486
AT4G38950	252.5761	0.631090656	0.200662	3.145046	1.66E-03	0.0093618	8.474278	8.292971	8.040079	7.810665	7.190921	7.770414
AT1G67740	4530.674	0.631156573	0.176256	3.580907	0.000342	0.0023728	12.22448	12.26494	12.7471	11.69617	12.05828	11.56271
AT4G35600	253.4277	0.631222626	0.183542	3.439121	5.84E-04	3.80E-03	8.449139	8.021327	8.327972	7.832265	7.567152	7.478706
AT1G29920	76702.81	0.632848596	0.154995	4.083027	4.45E-05	0.0003832	16.46648	16.33593	16.71213	16.0507	15.96272	15.54605
AT2G21280	216.0168	0.633026036	0.175142	3.614366	0.000301	0.0021161	7.88681	8.015276	8.212406	7.26112	7.585434	7.363061

AT1G72970	299.5841	0.633133988	0.149744	4.228119	2.36E-05	2.16E-04	8.43291	8.548969	8.570211	8.068994	7.723906	7.803055
AT1G63260	235.6053	0.635316817	0.202252	3.141222	1.68E-03	9.47E-03	8.144849	8.083376	8.265559	7.482168	7.909492	7.112669
AT4G28755	5797.285	0.635895133	0.170561	3.728257	1.93E-04	1.42E-03	12.67786	12.56633	13.07388	12.02227	12.41777	11.93593
AT1G10900	208.6096	0.638134241	0.177133	3.602575	0.000315	0.0022061	7.806308	8.191976	7.970641	7.338627	7.411902	7.307265
AT1G76990	860.9336	0.63887507	0.187101	3.414608	6.39E-04	4.11E-03	10.43269	9.78258	9.815073	9.496539	9.282066	9.372874
AT5G25190	519.9577	0.641541241	0.152986	4.193455	2.75E-05	2.49E-04	9.085588	9.322872	9.508321	8.570985	8.61765	8.783557
AT1G11410	155.4195	0.641617477	0.192658	3.330351	0.000867	0.0053333	7.476068	7.636011	7.64481	6.773083	6.747048	7.176347
AT2G34660	464.1118	0.642805689	0.2057	3.124967	0.001778	0.0099443	8.885464	9.526979	8.971322	8.670267	8.185975	8.567829
AT1G22750	134.3541	0.643342282	0.194168	3.313334	9.22E-04	5.63E-03	7.308286	7.31491	7.504459	6.750572	6.499609	6.841279
AT3G11945	330.5193	0.643412541	0.164061	3.921794	8.79E-05	0.0007111	8.523279	8.529995	8.902142	7.945604	8.060895	8.019904
AT3G56825	4074.913	0.644561443	0.138795	4.643994	3.42E-06	3.73E-05	12.24803	12.28473	12.31713	11.40782	11.52017	11.90853
AT4G39330	757.1042	0.645299342	0.138194	4.669525	3.02E-06	3.33E-05	9.887345	9.780798	9.896895	8.917636	9.32622	9.328718
AT5G67510	355.7764	0.645371193	0.165592	3.897362	9.72E-05	0.0007724	8.725258	8.729827	8.845083	8.378881	8.099571	7.819103
AT5G01220	271.2386	0.646257291	0.163113	3.962019	7.43E-05	6.11E-04	8.308818	8.317767	8.505399	7.46844	7.819681	7.850673
AT1G69070	233.7719	0.646319355	0.167753	3.852803	1.17E-04	0.0009107	8.153364	8.307899	8.012703	7.612711	7.567152	7.352073
AT2G31141	44329.01	0.646687331	0.169957	3.804996	1.42E-04	1.08E-03	15.85897	15.36281	15.90452	14.98621	15.3171	14.8626
AT4G34260	810.1863	0.648120921	0.138242	4.688315	2.75E-06	3.07E-05	9.771357	9.971788	10.0967	9.205221	9.480714	9.202881
AT1G76110	384.5512	0.648641474	0.172399	3.762433	1.68E-04	1.26E-03	8.909121	8.878901	8.843141	8.2723	8.506912	7.842845
AT1G11280	226.0878	0.64886396	0.166819	3.889633	1.00E-04	7.95E-04	8.010698	8.277886	8.046842	7.338627	7.585434	7.458357
AT1G10960	487.4244	0.652629924	0.185601	3.516309	0.000438	0.0029528	9.263927	9.014003	9.370894	8.240343	8.913741	8.459378
AT5G51120	287.917	0.652826084	0.144663	4.51274	6.40E-06	6.59E-05	8.387918	8.437994	8.560791	7.799743	7.909492	7.720024
AT1G29690	204.5673	0.653280253	0.188336	3.468697	5.23E-04	3.46E-03	7.92378	8.051207	7.96351	7.649677	7.066287	7.138479
AT1G48350	1413.748	0.653428749	0.128782	5.073929	3.90E-07	4.99E-06	10.63726	10.64247	10.97005	10.06268	10.18825	10.04331
AT2G41680	457.7931	0.65370167	0.137127	4.767115	1.87E-06	2.13E-05	9.040396	9.169213	9.18106	8.596453	8.544777	8.273883
AT1G23280	224.5952	0.653861683	0.203116	3.219156	1.29E-03	7.54E-03	8.071865	8.265194	8.019596	7.612711	6.900955	7.658921
AT5G66900	251.9465	0.653894429	0.145622	4.49035	7.11E-06	7.28E-05	8.301175	8.262642	8.27423	7.685719	7.472095	7.658921
AT1G23030	462.4134	0.654139874	0.156135	4.189589	2.79E-05	2.52E-04	9.184452	9.007921	9.233877	8.443461	8.745094	8.250405
AT3G45640	660.9859	0.654745624	0.143552	4.561035	5.09E-06	5.34E-05	9.842414	9.493635	9.640512	9.154682	8.847114	8.978347
AT5G61770	140.7263	0.65484318	0.194465	3.367405	0.000759	0.004767	7.546718	7.516861	7.241048	6.838581	6.779171	6.709736
AT4G37280	205.8241	0.655223017	0.164199	3.99041	6.60E-05	5.48E-04	8.053786	7.868599	8.02303	7.195969	7.452309	7.318598
AT5G36940	129.0493	0.655286974	0.204813	3.199439	1.38E-03	0.0079986	7.164051	7.300054	7.474672	6.420628	6.779171	6.72685
AT4G01090	271.7192	0.656350509	0.19819	3.311731	9.27E-04	5.66E-03	8.423554	8.561481	8.144664	8.013353	7.673524	7.406194
AT4G09890	295.863	0.657057467	0.146833	4.474864	7.65E-06	7.76E-05	8.6145	8.42664	8.485623	7.755209	7.788457	7.963265
AT4G16410	683.3284	0.657204278	0.144009	4.563634	5.03E-06	5.28E-05	9.713031	9.556448	9.847392	9.088912	9.213193	8.827852

AT5G65010	1385.532	0.657274043	0.12303	5.342406	9.17E-08	1.29E-06	10.71744	10.76878	10.71373	9.973155	9.908619	10.28201
AT1G49430	234.5258	0.657861879	0.166028	3.962354	7.42E-05	0.0006106	8.098567	8.123308	8.288568	7.276958	7.690514	7.538084
AT5G54630	416.8931	0.658212487	0.152387	4.319355	1.56E-05	0.0001495	9.025012	9.089381	8.868178	8.334164	8.55409	8.107448
AT3G51820	657.3927	0.661550759	0.115522	5.726631	1.02E-08	1.65E-07	9.735889	9.541789	9.686713	9.005814	9.025087	8.945828
AT1G44790	123.4025	0.662093667	0.20907	3.166857	0.001541	0.0087778	7.016281	7.32473	7.402681	6.607592	6.610887	6.526408
AT2G32560	365.222	0.662507525	0.159201	4.161459	3.16E-05	0.0002819	8.821008	8.777083	8.82555	7.915566	8.45814	8.019904
AT5G22880	254.7256	0.663742198	0.166047	3.997321	6.41E-05	5.33E-04	8.173039	8.392033	8.297103	7.482168	7.880172	7.498771
AT1G32540	244.7697	0.664215636	0.145801	4.555627	5.22E-06	5.47E-05	8.227818	8.242062	8.236271	7.495767	7.673524	7.538084
AT4G18480	1917.538	0.664513612	0.126352	5.25924	1.45E-07	1.99E-06	11.00344	11.27729	11.30759	10.38113	10.65006	10.55183
AT5G62570	373.526	0.666734156	0.126506	5.270392	1.36E-07	1.88E-06	8.882053	8.870545	8.789711	8.191041	8.112236	8.208377
AT5G51460	171.2084	0.668611225	0.185787	3.598813	3.20E-04	2.24E-03	7.879986	7.678751	7.608698	7.228912	7.013254	6.887672
AT3G18750	104.154	0.668759819	0.212846	3.141989	1.68E-03	9.45E-03	7.022492	7.050731	6.997482	6.102904	6.292644	6.545826
AT3G61970	110.2924	0.669085229	0.211175	3.168388	1.53E-03	8.74E-03	7.016281	7.254546	7.004448	6.270495	6.574739	6.404074
AT1G73490	281.1362	0.66918924	0.140611	4.759154	1.94E-06	2.21E-05	8.49005	8.419785	8.416803	7.766472	7.656331	7.842845
AT5G05370	109.1033	0.67036863	0.205909	3.255649	0.001131	0.0067293	7.010043	7.03885	7.211253	6.391825	6.292644	6.486772
AT1G64390	193.6854	0.670675089	0.170384	3.936256	8.28E-05	6.72E-04	7.862782	7.965931	7.908872	7.179211	7.013254	7.416778
AT1G27950	598.7185	0.670877544	0.154546	4.340963	1.42E-05	0.0001368	9.558123	9.289416	9.701791	8.807449	9.025087	8.70369
AT1G10170	1166.185	0.671027117	0.15624	4.294861	1.75E-05	1.65E-04	10.353	10.55705	10.55259	10.11648	9.593404	9.651318
AT5G23920	151.8057	0.671458959	0.17871	3.757258	0.000172	0.0012817	7.626355	7.455617	7.590297	6.880651	6.810594	6.917792
AT4G25890	257.0975	0.671549707	0.148905	4.509923	6.49E-06	6.67E-05	8.318946	8.213278	8.40369	7.649677	7.567152	7.66781
AT1G43560	205.2877	0.67234969	0.211144	3.184319	1.45E-03	8.35E-03	7.713388	7.898494	8.308404	7.110162	7.305638	7.458357
AT5G47640	217.2213	0.673711936	0.162325	4.150391	3.32E-05	2.94E-04	8.178611	8.05711	7.974194	7.522586	7.283413	7.341001
AT1G37130	12219.58	0.674980464	0.122725	5.499948	3.80E-08	5.66E-07	13.93098	13.82749	13.87424	13.42263	13.12056	13.01277
AT4G33865	828.8829	0.675426869	0.144512	4.673832	2.96E-06	3.27E-05	10.07159	9.770955	10.12334	9.316684	9.470914	9.146762
AT1G22430	388.1571	0.676173677	0.134173	5.03958	4.67E-07	5.89E-06	8.935691	8.863826	8.926184	8.371524	8.034523	8.226539
AT1G70610	177.0667	0.67798209	0.164485	4.121857	3.76E-05	3.29E-04	7.758737	7.78215	7.804986	7.056115	7.040014	7.163834
AT1G09533	327.514	0.678103481	0.203841	3.326625	8.79E-04	5.40E-03	8.574902	8.90368	8.50048	7.649677	7.835043	8.319723
AT5G14550	465.7974	0.678166073	0.185677	3.652404	2.60E-04	1.86E-03	9.445919	9.020059	9.006442	8.757522	8.344656	8.285481
AT3G57530	526.6757	0.678174719	0.132851	5.104782	3.31E-07	4.27E-06	9.325503	9.40153	9.304717	8.845121	8.635307	8.484752
AT4G23740	271.6968	0.678503782	0.151464	4.479643	7.48E-06	7.62E-05	8.323983	8.447013	8.401053	7.509239	7.865285	7.737017
AT5G65890	128.319	0.678899467	0.214921	3.15884	0.001584	0.008987	7.047071	7.499627	7.386775	6.750572	6.499609	6.602557
AT1G20340	18869.66	0.679128879	0.158642	4.280892	1.86E-05	1.75E-04	14.37384	14.42515	14.70426	13.5409	14.10531	13.74787
AT4G20830	458.0936	0.680748816	0.191848	3.54837	3.88E-04	2.65E-03	9.185837	9.181424	9.079174	8.866214	8.256129	8.120457
AT2G35260	1545.864	0.680764554	0.12681	5.368382	7.94E-08	1.13E-06	10.76406	11.03975	10.87444	10.28063	10.31691	10.02267

AT1G80750	357.9158	0.681315848	0.152885	4.456393	8.34E-06	8.38E-05	8.706093	8.750009	8.924349	8.248399	7.835043	8.165086
AT5G50950	272.6682	0.68176866	0.154143	4.422973	9.74E-06	9.68E-05	8.293491	8.544774	8.352746	7.612711	7.740317	7.770414
AT4G17810	171.2231	0.681841396	0.193215	3.528934	0.000417	0.0028289	7.60582	7.596027	7.96351	6.96128	7.166841	6.99045
AT2G37600	319.7459	0.683389143	0.147272	4.64033	3.48E-06	3.79E-05	8.581227	8.527871	8.767362	8.03214	7.938228	7.842845
AT4G36540	1153.89	0.683730624	0.101947	6.706699	1.99E-11	4.65E-10	10.53724	10.45233	10.43506	9.692313	9.897687	9.775765
AT1G68780	195.3211	0.684037609	0.186706	3.663721	0.000249	0.0017927	8.029322	7.911584	7.80897	6.901235	7.491614	7.249225
AT3G14415	3500.865	0.685367521	0.110066	6.226886	4.76E-10	9.26E-09	11.96329	12.018	12.23826	11.31211	11.47675	11.36988
AT4G19520	1247.111	0.685463611	0.180369	3.800349	1.44E-04	0.0010969	10.40563	10.60518	10.752	10.26483	9.633492	9.688919
AT1G73720	261.2324	0.685839296	0.155282	4.41672	1.00E-05	9.94E-05	8.430577	8.249814	8.352746	7.625138	7.472095	7.794964
AT2G28605	135.4883	0.685903668	0.19287	3.556308	3.76E-04	2.58E-03	7.256532	7.405604	7.533642	6.680852	6.646151	6.760481
AT1G61190	456.0155	0.686744816	0.130093	5.278873	1.30E-07	1.80E-06	9.169138	9.214811	9.045801	8.491765	8.289966	8.534198
AT3G02790	135.026	0.686940137	0.192937	3.560445	3.70E-04	2.55E-03	7.382594	7.264784	7.533642	6.632428	6.680573	6.760481
AT3G47070	804.5597	0.687540555	0.165399	4.156862	3.23E-05	2.87E-04	9.831843	9.839341	10.18289	8.957493	9.470914	9.314637
AT3G06070	351.7966	0.68782213	0.153984	4.466852	7.94E-06	8.02E-05	8.883759	8.557322	8.839251	8.050685	8.185975	7.984766
AT5G12470	253.3679	0.689089895	0.148366	4.644542	3.41E-06	3.72E-05	8.341477	8.244651	8.299936	7.709256	7.603488	7.488773
AT1G75750	2696.802	0.689524316	0.177478	3.885115	0.000102	0.0008089	11.98114	11.36794	11.70771	11.17514	11.08375	10.70865
AT4G01560	231.1698	0.689679813	0.155797	4.426786	9.56E-06	9.53E-05	8.059837	8.272822	8.157219	7.495767	7.472095	7.437718
AT3G24550	422.4985	0.689878379	0.1427	4.834479	1.33E-06	1.56E-05	9.003195	8.877233	9.203933	8.378881	8.289966	8.330959
AT5G54190	698.3334	0.691208706	0.207587	3.329736	8.69E-04	5.34E-03	10.03596	9.484904	9.713528	9.441388	8.862188	8.746276
AT3G62720	382.1098	0.692485615	0.141349	4.899129	9.63E-07	1.15E-05	8.836953	8.905317	8.909583	8.224096	8.365965	7.977634
AT4G18970	647.3643	0.692867352	0.211676	3.273237	1.06E-03	6.37E-03	9.278272	9.607619	9.988447	8.538503	9.134672	9.075069
AT1G54780	1300.694	0.693384532	0.18182	3.813582	1.37E-04	0.0010465	10.3344	10.59355	10.9705	9.867285	10.18825	9.759225
AT4G18570	277.6811	0.693508904	0.163006	4.254487	2.10E-05	1.94E-04	8.437566	8.282932	8.567861	7.915566	7.567152	7.66781
AT1G14840	293.0393	0.694149769	0.147392	4.709555	2.48E-06	2.78E-05	8.451442	8.584141	8.478136	7.63746	7.966403	7.811102
AT3G61870	555.6955	0.69990275	0.162553	4.305681	1.66E-05	1.58E-04	9.305268	9.332638	9.630416	8.498535	8.977427	8.659809
AT4G23940	198.6972	0.70103499	0.202526	3.46145	5.37E-04	3.55E-03	7.646601	8.151169	8.040079	7.323456	6.985988	7.352073
AT1G70410	811.9241	0.701121611	0.120228	5.83159	5.49E-09	9.22E-08	9.965984	9.92978	10.02573	9.229841	9.456087	9.121102
AT5G09065	539.9699	0.70382221	0.153132	4.596181	4.30E-06	4.58E-05	9.246788	9.514163	9.413383	8.478128	8.599774	8.908815
AT3G15520	288.1652	0.704564862	0.168784	4.174367	2.99E-05	0.0002686	8.288346	8.504302	8.645664	7.600176	7.966403	7.745439
AT2G38740	116.4569	0.706124776	0.222117	3.179074	0.001477	0.0084781	7.282641	7.180772	7.143456	6.750572	5.941644	6.545826
AT4G21280	5957.664	0.706837581	0.150627	4.692644	2.70E-06	3.01E-05	12.74946	12.76399	13.03433	11.92134	12.41649	12.02769
AT3G10050	155.8902	0.707067836	0.18592	3.803068	1.43E-04	1.09E-03	7.724859	7.628102	7.469647	7.03764	6.747048	6.85691
AT1G66130	349.9848	0.707125585	0.138976	5.088098	3.62E-07	4.64E-06	8.836953	8.677143	8.775529	8.003867	8.209741	7.956026
AT1G29965	113.604	0.707825038	0.216093	3.275563	0.001055	0.0063306	7.047071	7.223389	7.16848	6.205775	6.779171	6.293554

AT5G13180	473.9911	0.707984946	0.143613	4.929818	8.23E-07	9.99E-06	9.395366	9.042547	9.15469	8.491765	8.535404	8.443937
AT1G52827	115.7994	0.708623266	0.223249	3.174141	1.50E-03	8.59E-03	6.959147	7.223389	7.332454	6.582321	6.610887	6.17386
AT3G18980	325.8281	0.708752718	0.180758	3.921009	8.82E-05	0.000713	8.704163	8.731673	8.544156	7.994319	8.233121	7.566881
AT3G60965	131.2614	0.710422615	0.206245	3.444553	5.72E-04	3.74E-03	7.191872	7.495286	7.402681	6.859769	6.420349	6.583893
AT3G21110	110.3725	0.711754137	0.22126	3.216822	1.30E-03	0.0075949	7.083171	7.212852	7.092067	6.556599	5.883728	6.564985
AT1G50250	1104.854	0.712540835	0.125678	5.669588	1.43E-08	2.26E-07	10.22488	10.518	10.51589	9.607172	9.783555	9.725565
AT4G33010	2577.936	0.713140994	0.177348	4.021144	5.79E-05	4.87E-04	11.24135	11.68934	11.93935	10.73222	11.07245	10.94272
AT5G43870	123.708	0.713346879	0.218428	3.265829	0.001091	0.0065253	7.224564	7.228629	7.354429	6.362435	6.958197	6.293554
AT1G72330	237.7704	0.713588118	0.179483	3.9758	7.01E-05	5.80E-04	8.104434	8.173076	8.382459	7.426454	7.237908	7.728545
AT3G48500	189.4677	0.713971053	0.196801	3.627876	2.86E-04	2.02E-03	7.988661	7.993895	7.701631	7.228912	6.779171	7.373965
AT4G39640	594.2747	0.714585157	0.174389	4.097649	4.17E-05	0.0003623	9.702432	9.493635	9.398918	9.141766	8.572538	8.632826
AT2G38470	395.8634	0.714923736	0.224827	3.179889	1.47E-03	8.46E-03	9.079644	9.09082	8.665575	8.639969	7.690514	8.146128
AT4G18010	451.7458	0.715348677	0.185211	3.862338	1.12E-04	8.77E-04	8.990578	9.22271	9.203933	8.785472	8.209741	8.146128
AT2G31070	190.9983	0.715376507	0.178929	3.998111	6.39E-05	5.32E-04	7.982303	7.778582	7.930976	7.353641	7.214606	6.947296
AT1G47580	111.2415	0.715857958	0.221222	3.235929	1.21E-03	0.0071573	7.147097	7.032872	7.174668	6.238498	6.779171	6.148682
AT3G47960	117.1736	0.716406035	0.223403	3.206794	1.34E-03	7.82E-03	6.991167	7.153483	7.423619	6.332435	6.714193	6.360876
AT5G16715	310.0865	0.716568493	0.178707	4.009736	6.08E-05	0.0005091	8.43524	8.785996	8.577235	7.832265	7.585434	8.113967
AT5G03555	179.9472	0.717765507	0.172309	4.165565	3.11E-05	2.77E-04	7.834821	7.69019	7.927316	7.127736	7.013254	7.112669
AT3G23530	384.4842	0.717794462	0.146423	4.902212	9.48E-07	1.14E-05	8.934045	8.970881	8.817662	8.400731	8.060895	8.054206
AT2G46340	578.8129	0.718440279	0.122224	5.878085	4.15E-09	7.07E-08	9.474515	9.600563	9.415997	8.740488	8.687018	8.870827
AT5G52780	338.7415	0.718639151	0.155098	4.63345	3.60E-06	3.90E-05	8.783101	8.544774	8.831438	7.832265	8.149577	8.005951
AT3G54460	428.2851	0.719957533	0.137976	5.218	1.81E-07	2.45E-06	8.987407	9.115071	9.092311	8.139994	8.35535	8.484752
AT1G49380	253.367	0.72033417	0.14437	4.989495	6.05E-07	7.52E-06	8.311357	8.282932	8.333514	7.495767	7.621318	7.622806
AT3G46540	170.238	0.722199246	0.194989	3.703804	2.12E-04	1.55E-03	7.900364	7.682574	7.649262	7.228912	6.646151	7.046052
AT2G03750	364.3376	0.722245222	0.13971	5.169595	2.35E-07	3.11E-06	8.953678	8.780654	8.746741	8.022777	8.221479	8.067702
AT3G13470	454.4068	0.722890563	0.129119	5.598659	2.16E-08	3.33E-07	9.123641	9.192192	9.123038	8.280179	8.599774	8.380466
AT3G58660	202.8548	0.723246814	0.185384	3.901341	9.57E-05	7.62E-04	7.841862	8.213278	7.905155	7.397768	7.09209	7.249225
AT5G49910	546.7581	0.723745435	0.123661	5.852648	4.84E-09	8.17E-08	9.412021	9.50879	9.328503	8.596453	8.661394	8.78764
AT3G24430	551.2596	0.724052582	0.170748	4.240483	2.23E-05	2.06E-04	9.140871	9.424543	9.673663	8.676251	8.862188	8.534198
AT3G46530	550.8191	0.724412254	0.175156	4.135814	3.54E-05	0.0003116	9.325503	9.622623	9.32989	8.996279	8.376502	8.623718
AT5G61270	95.87494	0.724430684	0.212755	3.405005	0.000662	0.0042408	6.865515	6.933695	6.96214	6.066916	6.247434	6.222936
AT1G79150	144.6642	0.724886284	0.214899	3.373141	7.43E-04	4.68E-03	7.397007	7.632062	7.52398	6.556599	6.499609	7.112669
AT2G42220	2269.735	0.726737477	0.118345	6.14084	8.21E-10	1.55E-08	11.44697	11.36971	11.58064	10.73079	10.8951	10.56972
AT3G02880	349.3124	0.727680925	0.141977	5.125335	2.97E-07	3.88E-06	8.709947	8.7609	8.833395	7.945604	8.244671	7.926702

AT4G10450	268.6628	0.729929429	0.150268	4.85751	1.19E-06	1.40E-05	8.411773	8.484728	8.297103	7.766472	7.529878	7.658921
AT1G77450	131.4902	0.730964804	0.198091	3.690037	2.24E-04	1.64E-03	7.471535	7.31491	7.31014	6.680852	6.810594	6.404074
AT1G78580	175.9287	0.731122245	0.198364	3.685763	0.000228	0.0016598	7.642574	8.033353	7.680041	6.901235	6.985988	7.213246
AT4G17870	362.1845	0.731573779	0.155895	4.692723	2.70E-06	3.01E-05	9.020365	8.644192	8.789711	8.050685	8.185975	8.02683
AT1G21910	265.8291	0.733169486	0.187319	3.914022	9.08E-05	7.29E-04	8.456039	8.074674	8.572556	7.561902	7.819681	7.528356
AT1G26220	131.2544	0.734179052	0.204069	3.597699	3.21E-04	2.24E-03	7.489581	7.259674	7.343483	6.391825	6.900955	6.564985
AT1G74670	6241.163	0.734539293	0.128929	5.697238	1.22E-08	1.94E-07	12.73074	12.944	13.09769	12.03222	12.33104	12.18882
AT5G11680	259.3668	0.735204572	0.15193	4.839095	1.30E-06	1.53E-05	8.409405	8.28796	8.350014	7.426454	7.656331	7.711452
AT5G13930	177.6488	0.735271732	0.207593	3.54189	0.000397	0.0027085	7.511825	7.914839	7.952747	7.245106	7.09209	6.809501
AT5G48930	589.0898	0.735668855	0.193403	3.803823	1.42E-04	0.0010841	9.906781	9.193532	9.404195	8.907497	8.736939	8.681917
AT5G62790	789.9163	0.736646468	0.17333	4.249967	2.14E-05	0.0001981	9.574067	10.10738	10.11934	9.024699	9.270813	9.297557
AT5G63790	734.0926	0.737115174	0.225635	3.266849	0.001088	0.0065094	9.814946	10.07938	9.634912	9.591425	8.703851	8.795773
AT5G43450	592.4114	0.737239112	0.184009	4.006533	6.16E-05	0.0005151	9.349169	9.482713	9.764681	9.115581	8.506912	8.659809
AT5G08170	114.7457	0.737298871	0.213706	3.450055	0.00056	0.0036798	7.05315	7.147962	7.354429	6.556599	6.152528	6.506726
AT1G30520	189.3537	0.737335081	0.167563	4.400353	1.08E-05	1.06E-04	7.959825	7.77142	7.956343	7.195969	7.142352	7.112669
AT3G44735	81.44592	0.738508714	0.233077	3.168515	1.53E-03	8.74E-03	6.649776	6.595375	6.827223	6.030007	5.941644	5.838142
AT5G48790	572.0828	0.738616779	0.13712	5.386626	7.18E-08	1.03E-06	9.501445	9.348368	9.596244	8.602751	8.928139	8.686298
AT2G09690	206.7759	0.739710915	0.189248	3.908683	9.28E-05	7.42E-04	8.141999	8.068843	7.84434	7.495767	7.305638	6.99045
AT5G24590	419.3001	0.740236626	0.13677	5.412289	6.22E-08	9.00E-07	9.001624	9.130549	8.985473	8.457428	8.244671	8.165086
AT4G16400	654.754	0.741357919	0.147192	5.036668	4.74E-07	5.97E-06	9.665207	9.536517	9.826909	8.922678	9.140866	8.720876
AT2G24860	204.0694	0.741700016	0.172701	4.294703	1.75E-05	1.65E-04	7.838346	8.062988	8.099838	7.127736	7.411902	7.225339
AT1G72430	783.7339	0.74201191	0.177018	4.191721	2.77E-05	0.0002502	10.02904	9.65217	10.10887	9.192751	9.441106	8.889946
AT4G09320	1629.41	0.743561414	0.115883	6.416509	1.39E-10	2.93E-09	10.938	10.86192	11.17573	10.23878	10.23821	10.26454
AT3G10520	389.5301	0.743927279	0.15307	4.860051	1.17E-06	1.39E-05	8.725258	8.966183	9.093944	8.248399	8.221479	8.081073
AT4G13560	278.2861	0.744046043	0.203593	3.654576	0.000258	0.0018475	8.501212	8.720559	8.093319	7.788738	7.432247	7.811102
AT1G23950	188.2039	0.744295614	0.172782	4.307717	1.65E-05	0.0001568	7.859316	7.891904	7.93827	7.353641	6.900955	7.09959
AT5G55790	132.1808	0.745406575	0.192084	3.880636	1.04E-04	8.21E-04	7.448656	7.353794	7.381434	6.607592	6.420349	6.793345
AT4G19660	216.3973	0.746391794	0.205181	3.637723	2.75E-04	1.96E-03	8.222433	8.086266	7.991826	7.661791	6.810594	7.363061
AT1G70230	143.6447	0.746402276	0.189695	3.934754	8.33E-05	6.76E-04	7.33847	7.579718	7.58566	6.773083	6.779171	6.692417
AT1G77090	213.6512	0.746737973	0.158581	4.708886	2.49E-06	2.79E-05	7.966283	8.117671	8.131998	7.338627	7.349087	7.272722
AT1G50732	153.1073	0.747470196	0.1998	3.741099	1.83E-04	1.36E-03	7.589179	7.473382	7.714432	6.503738	7.09209	6.872373
AT1G29700	155.2826	0.747725704	0.185909	4.022	5.77E-05	4.85E-04	7.572344	7.554903	7.735517	7.03764	6.646151	6.841279
AT1G80840	126.8917	0.748091898	0.196646	3.804259	0.000142	0.0010826	7.358247	7.269875	7.348966	6.727704	6.610887	6.360876
AT4G26860	237.3087	0.748693329	0.153335	4.882734	1.05E-06	1.24E-05	8.272799	8.137306	8.268455	7.574773	7.391266	7.427286

AT5G53900	178.1087	0.749409527	0.180609	4.149356	3.33E-05	0.0002951	7.873129	7.891904	7.697339	7.110162	6.779171	7.201051
AT1G75690	683.5588	0.750441689	0.150729	4.978759	6.40E-07	7.91E-06	9.638161	9.632539	9.948373	8.912575	9.201388	8.8397
AT3G05500	250.7092	0.750970423	0.190261	3.94706	7.91E-05	6.46E-04	8.383099	8.451502	8.08677	7.625138	7.166841	7.737017
AT5G64770	499.5753	0.751393064	0.142955	5.256143	1.47E-07	2.01E-06	9.196862	9.336283	9.357355	8.280179	8.626506	8.673114
AT1G07520	107.0861	0.751848317	0.234101	3.211639	1.32E-03	7.71E-03	6.90639	7.202238	7.137131	6.607592	6.336481	5.958939
AT1G12800	782.6191	0.753763259	0.112828	6.680655	2.38E-11	5.51E-10	9.827416	9.973349	10.0223	9.137435	9.253768	9.165712
AT4G20835	275.3892	0.753798352	0.200405	3.761372	1.69E-04	0.0012623	8.527652	8.453741	8.352746	8.087074	7.432247	7.384788
AT5G63180	478.3707	0.754738984	0.164809	4.579484	4.66E-06	4.93E-05	9.403718	9.069078	9.230911	8.232242	8.417898	8.708006
AT1G02270	202.8136	0.757128803	0.19832	3.81772	0.000135	0.0010319	7.758737	7.918085	8.294263	7.179211	7.237908	7.272722
AT1G26218	157.5662	0.757472909	0.183482	4.128314	3.65E-05	3.21E-04	7.709544	7.538119	7.658123	6.773083	7.09209	6.760481
AT1G13080	109.9475	0.757622918	0.215342	3.518225	4.34E-04	2.93E-03	7.124178	7.23385	7.004448	6.503738	6.460523	6.096967
AT1G72645	5111.542	0.757906785	0.152736	4.962209	6.97E-07	8.57E-06	12.57241	12.82883	12.54487	11.56605	11.92554	12.10704
AT3G55000	171.3799	0.760107859	0.175015	4.343096	1.40E-05	0.0001358	7.751277	7.716535	7.812943	7.127736	7.040014	6.809501
AT1G49975	265.9239	0.760312229	0.17994	4.225354	2.39E-05	2.19E-04	8.366106	8.267741	8.522484	7.46844	7.952384	7.416778
AT5G65860	114.1787	0.760510142	0.197891	3.843067	0.000122	0.0009421	7.180808	7.158982	7.199161	6.362435	6.537663	6.338783
AT1G58290	3249.152	0.761614454	0.112441	6.773486	1.26E-11	3.00E-10	12.03742	11.94627	12.01243	11.35936	11.29151	11.02631
AT1G03630	743.5308	0.761970122	0.153231	4.972675	6.60E-07	8.15E-06	9.740606	9.871518	9.995456	8.876646	9.384807	9.010149
AT1G51700	117.5186	0.763156491	0.230532	3.310413	9.32E-04	5.68E-03	7.164051	7.228629	7.304507	6.859769	6.050936	6.270397
AT4G12980	272.8286	0.764709029	0.196595	3.88976	1.00E-04	7.94E-04	8.241194	8.515063	8.515186	7.383209	8.047769	7.468567
AT1G63860	248.3841	0.764965604	0.17601	4.346157	1.39E-05	1.34E-04	8.306275	8.167631	8.411572	7.777648	7.452309	7.295843
AT5G59430	419.0979	0.766429381	0.17985	4.261499	2.03E-05	1.89E-04	9.004764	9.23708	8.915138	8.25641	7.909492	8.548707
AT1G75670	96.27683	0.767096002	0.224989	3.409486	6.51E-04	4.18E-03	6.919761	6.996475	6.940512	6.420628	5.760417	6.17386
AT3G06530	318.7055	0.767262305	0.166576	4.60608	4.10E-06	4.39E-05	8.49005	8.870545	8.598107	7.95548	7.690514	7.956026
AT2G41110	313.1828	0.767437665	0.176364	4.351453	1.35E-05	1.31E-04	8.585428	8.480342	8.801756	7.905412	8.073902	7.557346
AT5G45430	803.2089	0.768783714	0.124467	6.176615	6.55E-10	1.25E-08	9.849418	9.989644	10.11854	9.192751	9.109626	9.314637
AT2G43290	188.4062	0.769368212	0.177559	4.333022	1.47E-05	0.0001414	7.824194	7.834638	8.029874	7.212535	7.237908	6.917792
AT1G70750	109.0946	0.770816556	0.224028	3.440712	5.80E-04	3.78E-03	7.152771	7.274949	6.940512	6.582321	6.050936	6.293554
AT3G02150	133.3306	0.771045468	0.209429	3.681651	2.32E-04	1.68E-03	7.382594	7.583812	7.223245	6.656843	6.810594	6.404074
AT5G20140	210.8687	0.771335455	0.168995	4.564254	5.01E-06	5.27E-05	7.956585	8.024343	8.215411	7.212535	7.214606	7.395531
AT3G23390	480.2967	0.771567171	0.124908	6.177093	6.53E-10	1.25E-08	9.176119	9.269218	9.286259	8.478128	8.590752	8.347651
AT3G48730	862.5141	0.771807823	0.185944	4.150755	3.31E-05	0.0002938	9.810467	10.12926	10.30428	8.907497	9.490449	9.449617
AT3G26980	234.4781	0.772265584	0.172025	4.489266	7.15E-06	7.31E-05	8.366106	8.039329	8.221402	7.46844	7.567152	7.272722
AT2G04030	1054.988	0.773081775	0.148432	5.20833	1.91E-07	2.57E-06	10.15946	10.53184	10.44149	9.597745	9.384807	9.777819
AT5G17170	337.9006	0.773142293	0.171998	4.495075	6.95E-06	7.13E-05	8.510079	8.726127	8.969543	7.832265	7.923931	8.09432

AT2G04110	120.2572	0.773922294	0.203615	3.800916	1.44E-04	1.09E-03	7.224564	7.249399	7.31014	6.704469	6.420349	6.270397
AT5G28750	590.9781	0.773987485	0.159404	4.855514	1.20E-06	1.41E-05	9.360245	9.534403	9.72306	8.583775	9.018374	8.664258
AT2G35860	91.04652	0.774276434	0.233182	3.320485	0.000899	0.0055017	6.801947	6.820269	6.969278	5.694846	6.292644	6.17386
AT5G57345	928.5767	0.774798498	0.14751	5.252516	1.50E-07	2.05E-06	10.06107	10.20963	10.31483	9.591425	9.490449	9.130778
AT4G01070	151.0001	0.776890948	0.21422	3.6266	2.87E-04	2.03E-03	7.348392	7.838071	7.566961	6.773083	6.574739	6.976208
AT3G44750	338.0168	0.776907489	0.2054	3.782415	1.55E-04	1.17E-03	8.566426	8.836631	8.847022	8.215903	7.370331	8.100899
AT2G33180	106.0425	0.777088659	0.214863	3.616669	0.000298	0.0021014	7.219167	6.920804	7.098592	6.172293	6.379024	6.316346
AT1G51110	345.774	0.778579412	0.16169	4.815269	1.47E-06	1.71E-05	8.534187	8.918346	8.856677	7.874516	8.060895	8.02683
AT1G21500	1202.079	0.77953809	0.169015	4.61223	3.98E-06	4.28E-05	10.38769	10.42919	10.85819	9.692313	10.0237	9.598834
AT1G09333	4945.587	0.77990256	0.156189	4.993329	5.93E-07	7.38E-06	12.52562	12.79986	12.50106	11.49589	11.86041	12.05293
AT1G61380	171.9343	0.780920614	0.197729	3.949448	7.83E-05	6.40E-04	7.674477	7.891904	7.780847	7.26112	6.574739	6.99045
AT5G42070	155.706	0.780934635	0.220365	3.54383	3.94E-04	2.69E-03	7.670527	7.37285	7.812943	6.680852	7.190921	6.602557
AT5G12860	442.2721	0.783228256	0.21437	3.653629	2.59E-04	1.85E-03	8.694471	9.180072	9.448285	8.602751	8.233121	8.113967
AT3G46520	162.0023	0.784420448	0.2168	3.618175	2.97E-04	2.09E-03	7.568105	7.888597	7.635867	7.162256	6.379024	6.976208
AT2G05620	1112.193	0.784699481	0.12031	6.522333	6.92E-11	1.52E-09	10.52417	10.35507	10.49811	9.755854	9.763623	9.485253
AT4G31390	223.1617	0.784827894	0.152391	5.150078	2.60E-07	3.43E-06	8.121893	8.197331	8.141508	7.323456	7.305638	7.427286
AT5G59870	119.6728	0.785650479	0.212218	3.702094	0.000214	0.0015641	7.135683	7.158982	7.469647	6.332435	6.499609	6.526408
AT1G74750	75.79178	0.786088487	0.250744	3.135022	1.72E-03	9.64E-03	6.6255	6.778008	6.442294	5.872138	5.476813	5.958939
AT5G46690	144.4931	0.786419404	0.20819	3.777415	0.000158	0.0011924	7.60995	7.563222	7.392097	6.921529	6.871459	6.360876
AT4G14910	234.2404	0.787720617	0.160612	4.904494	9.37E-07	1.12E-05	8.170244	8.368491	8.116007	7.383209	7.452309	7.437718
AT1G31660	229.9935	0.787792501	0.165091	4.771883	1.83E-06	2.08E-05	8.164639	8.297964	8.141508	7.509239	7.11744	7.498771
AT5G45680	425.2661	0.787971181	0.179874	4.380694	1.18E-05	1.16E-04	8.963396	9.021569	9.245683	7.864069	8.45814	8.438754
AT5G02120	532.2251	0.788358367	0.205854	3.829698	0.000128	0.0009888	9.303994	9.246151	9.632666	8.232242	9.025087	8.428329
AT3G21690	269.9358	0.788643857	0.184735	4.269051	1.96E-05	0.0001834	8.262341	8.536347	8.473124	7.935661	7.491614	7.384788
AT3G23805	134.5644	0.789547611	0.223659	3.530138	4.15E-04	2.82E-03	7.551021	7.207555	7.499537	6.859769	6.714193	6.246861
AT5G56730	118.1478	0.792155973	0.211041	3.75357	0.000174	0.0012988	7.323457	7.358582	7.04555	6.332435	6.379024	6.564985
AT5G09870	403.9922	0.792197613	0.123871	6.395326	1.60E-10	3.34E-09	9.003195	9.051445	8.969543	8.199376	8.149577	8.262192
AT2G23672	178.6462	0.792214176	0.187817	4.21801	2.46E-05	0.0002253	7.903732	7.682574	7.893946	7.110162	7.214606	6.760481
AT1G05207	3408.786	0.794011606	0.168266	4.718797	2.37E-06	2.66E-05	11.893	11.95598	12.35747	11.09811	11.55421	11.12643
AT3G07230	496.8262	0.794360319	0.149098	5.32778	9.94E-08	1.40E-06	9.309084	9.188163	9.405511	8.264377	8.736939	8.479713
AT1G18060	314.9957	0.795562002	0.168	4.735481	2.19E-06	2.47E-05	8.549321	8.614479	8.777564	7.522586	8.047769	7.919277
AT1G30680	126.1227	0.796084638	0.214919	3.704123	0.000212	0.0015534	7.11258	7.419417	7.484669	6.656843	6.247434	6.602557
AT1G10830	132.6407	0.796655234	0.205147	3.883343	1.03E-04	8.14E-04	7.363149	7.290065	7.58566	6.773083	6.336481	6.620982
AT1G08580	131.9392	0.797303336	0.218911	3.642134	2.70E-04	1.93E-03	7.420713	7.473382	7.359871	6.448866	6.200761	6.947296

AT1G64150	139.2601	0.797376123	0.214929	3.709955	2.07E-04	1.52E-03	7.318417	7.405604	7.710177	6.420628	6.646151	6.872373
AT1G72755	139.6988	0.798155874	0.185613	4.300113	1.71E-05	1.62E-04	7.533732	7.43763	7.469647	6.680852	6.810594	6.545826
AT3G62410	896.469	0.798579709	0.163286	4.890679	1.00E-06	1.20E-05	10.00571	10.09237	10.35557	9.597745	9.32622	9.071724
AT4G27700	702.8578	0.799895937	0.149231	5.360112	8.32E-08	1.18E-06	9.648237	9.856365	9.903431	8.729019	9.207302	9.024061
AT5G55920	231.0617	0.802948421	0.181668	4.41986	9.88E-06	9.80E-05	8.121893	8.382662	8.131998	7.353641	7.11744	7.622806
AT1G15250	151.7518	0.80350322	0.180672	4.447309	8.70E-06	8.72E-05	7.593357	7.533893	7.680041	6.859769	6.841348	6.674887
AT3G13000	177.6591	0.804215156	0.166831	4.820525	1.43E-06	1.67E-05	7.762452	7.871951	7.855939	7.03764	7.040014	6.976208
AT1G80030	274.1544	0.805120898	0.154231	5.220228	1.79E-07	2.42E-06	8.321467	8.553151	8.478136	7.755209	7.621318	7.538084
AT4G36500	209.7495	0.805368714	0.205551	3.918098	8.93E-05	7.20E-04	8.275402	8.006151	7.934627	7.548916	6.810594	7.261021
AT5G65610	228.526	0.806102697	0.168468	4.784905	1.71E-06	1.96E-05	8.11319	8.089149	8.355472	7.308122	7.529878	7.295843
AT4G22010	220.1578	0.806302021	0.166749	4.835416	1.33E-06	1.56E-05	7.998147	8.215918	8.191195	7.162256	7.472095	7.329843
AT4G30150	167.5678	0.807409425	0.227554	3.548214	3.88E-04	2.65E-03	7.392218	8.027353	7.788938	7.09237	6.610887	6.99045
AT2G44740	134.9965	0.8074256	0.219734	3.674552	0.000238	0.0017266	7.267032	7.339335	7.680041	6.332435	6.841348	6.639175
AT1G15410	211.8738	0.807460492	0.186919	4.319832	1.56E-05	0.0001493	7.817066	8.260085	8.150955	7.368501	7.214606	7.201051
AT2G42130	231.425	0.807511125	0.170797	4.727909	2.27E-06	2.56E-05	8.029322	8.207982	8.374416	7.397768	7.491614	7.295843
AT5G18400	221.4868	0.807826887	0.175178	4.61145	4.00E-06	4.29E-05	8.139144	8.221185	8.116007	7.383209	6.958197	7.538084
AT5G27030	151.2008	0.808052	0.183226	4.410127	1.03E-05	0.0001023	7.634487	7.636011	7.52398	6.607592	6.958197	6.777007
AT2G30695	146.1651	0.809219875	0.20847	3.881705	1.04E-04	8.18E-04	7.502969	7.363353	7.780847	6.530411	6.747048	6.872373
AT1G15810	1298.491	0.809582425	0.145704	5.556333	2.76E-08	4.18E-07	10.65735	10.87785	10.52863	9.692313	10.09881	9.812301
AT1G48300	2332.127	0.810098925	0.180636	4.484704	7.30E-06	7.45E-05	11.79663	11.57417	11.20248	10.76207	10.39414	10.93169
AT5G62220	155.1004	0.811763133	0.198325	4.093098	4.26E-05	0.0003686	7.466988	7.616158	7.80897	6.632428	7.040014	6.760481
AT3G09050	122.8897	0.812018944	0.212578	3.819868	0.000134	0.0010245	7.213749	7.363353	7.370693	6.238498	6.336481	6.760481
AT3G02730	725.7831	0.812330518	0.182344	4.454928	8.39E-06	8.44E-05	9.591926	9.743765	10.17905	8.876646	9.259472	8.934823
AT5G55740	94.70399	0.812527476	0.230134	3.530678	4.14E-04	2.81E-03	6.816318	7.108716	6.888749	5.913254	6.050936	6.293554
AT5G20160	385.8441	0.813609508	0.139855	5.817527	5.97E-09	9.95E-08	8.914141	8.898758	9.042421	8.248399	7.909492	8.177588
AT5G46790	94.85087	0.813764217	0.222088	3.664157	2.48E-04	1.79E-03	7.034833	6.827194	6.940512	6.238498	6.152528	5.929675
AT4G02530	720.9736	0.814192197	0.15893	5.122961	3.01E-07	3.92E-06	9.609567	9.839341	10.06457	8.839799	9.207302	9.00665
AT2G26080	1749.266	0.814293017	0.086699	9.392224	5.87E-21	3.19E-19	11.07466	11.15156	11.1469	10.34214	10.31691	10.26307
AT2G17480	276.1175	0.81541905	0.155751	5.235387	1.65E-07	2.24E-06	8.423554	8.415197	8.556058	7.799743	7.673524	7.448074
AT1G08300	80.24099	0.815830198	0.255124	3.197779	1.38E-03	8.03E-03	6.952657	6.423257	6.695692	5.913254	5.82339	5.838142
AT2G33110	84.64623	0.815854993	0.254914	3.200507	1.37E-03	7.97E-03	6.794708	6.977926	6.569861	6.205775	5.476813	5.987621
AT5G61455	2770.033	0.816148254	0.176889	4.613901	3.95E-06	4.25E-05	11.70403	11.80105	11.87406	10.57296	10.86735	11.33124
AT2G23670	176.7843	0.816461099	0.189543	4.307519	1.65E-05	0.0001569	7.903732	7.671073	7.886424	7.074357	7.190921	6.72685
AT2G30520	3686.513	0.817018261	0.136277	5.995276	2.03E-09	3.67E-08	12.40957	12.02818	12.14619	11.53428	11.3661	11.2102

AT1G11790	73.61893	0.817641393	0.24897	3.284097	0.001023	0.0061698	6.479641	6.477082	6.779293	5.694846	5.69457	5.806294
AT4G39950	216.2199	0.817689098	0.25898	3.157346	1.59E-03	9.02E-03	7.88681	8.428918	8.033283	7.625138	6.499609	7.427286
AT5G00875	211.3206	0.81800501	0.169654	4.821617	1.42E-06	1.66E-05	8.053786	8.086266	8.099838	7.03764	7.529878	7.188751
AT1G56500	557.2747	0.818338519	0.155599	5.259276	1.45E-07	1.99E-06	9.351637	9.493635	9.584671	8.334164	8.854671	8.720876
AT2G04375	2631.268	0.818438899	0.123355	6.634827	3.25E-11	7.41E-10	11.65366	11.69834	11.79842	10.72647	10.80826	11.10286
AT3G07315	417.9691	0.818911221	0.19199	4.265385	2.00E-05	0.0001857	8.902402	8.99568	9.287687	7.821505	8.301072	8.484752
AT5G18010	203.9004	0.81919545	0.175727	4.661742	3.14E-06	3.44E-05	8.150531	7.85511	8.073583	7.195969	7.349087	7.073069
AT3G11170	1034.547	0.819619823	0.110157	7.440481	1.00E-13	3.00E-12	10.3895	10.26678	10.44725	9.619647	9.575223	9.439271
AT3G10610	190.3555	0.820233196	0.170894	4.799648	1.59E-06	1.83E-05	8.001295	7.820827	7.988316	7.245106	6.985988	7.059624
AT5G20270	151.5191	0.820440001	0.210341	3.900522	9.60E-05	0.0007638	7.820635	7.339335	7.626867	6.817077	6.900955	6.602557
AT5G56380	108.7613	0.820480168	0.236144	3.474493	5.12E-04	3.39E-03	6.959147	7.175355	7.298852	6.503738	5.760417	6.446015
AT3G54050	2773.357	0.82124951	0.117223	7.005854	2.45E-12	6.38E-11	11.633	11.76093	11.96613	10.97487	11.03802	10.87908
AT1G29810	62.86093	0.821338078	0.258726	3.174548	1.50E-03	0.0085846	6.297009	6.395572	6.411182	5.437371	5.310931	5.706299
AT5G08920	168.8326	0.823199486	0.189177	4.351489	1.35E-05	0.0001311	7.601678	7.803375	7.901428	6.980752	6.680573	7.059624
AT4G08040	124.1162	0.823352918	0.219464	3.751648	0.000176	0.0013077	7.462427	7.16446	7.315751	6.270495	6.841348	6.316346
AT5G59450	252.6081	0.824805383	0.179026	4.60717	4.08E-06	4.37E-05	8.153364	8.401343	8.483132	7.600176	7.190921	7.649977
AT3G21670	720.5981	0.825131367	0.177503	4.648543	3.34E-06	3.66E-05	9.44938	10.10881	9.933851	8.996279	8.949469	9.085058
AT4G00955	341.552	0.826822707	0.136705	6.048222	1.46E-09	2.68E-08	8.692525	8.803658	8.833395	7.832265	8.007659	7.984766
AT1G15000	134.3799	0.827526884	0.211949	3.904362	9.45E-05	7.54E-04	7.31336	7.329615	7.671314	6.773083	6.379024	6.583893
AT4G19985	82.74298	0.828016546	0.228135	3.629508	0.000284	0.0020101	6.765383	6.792233	6.695692	6.030007	5.82339	5.838142
AT3G56070	232.8809	0.828052768	0.193681	4.275337	1.91E-05	1.79E-04	8.238529	8.422074	8.016154	7.649677	7.09209	7.341001
AT2G37240	116.9256	0.828233257	0.21151	3.915807	9.01E-05	0.0007253	7.106746	7.244235	7.354429	6.172293	6.646151	6.360876
AT3G56705	2787.032	0.828559633	0.136043	6.090432	1.13E-09	2.09E-08	11.6958	11.77129	11.93867	10.84542	10.80631	11.2041
AT5G65613	241.4675	0.829038088	0.191776	4.322951	1.54E-05	0.0001474	8.161829	8.140089	8.507852	7.179211	7.707307	7.395531
AT3G20362	94.79182	0.831959688	0.225637	3.687165	0.000227	0.0016512	6.90639	6.888065	7.06567	5.99213	5.883728	6.316346
AT3G09440	741.229	0.832300148	0.168057	4.952485	7.33E-07	8.97E-06	10.01587	9.975688	9.68563	9.343255	8.77726	8.963984
AT2G46310	68.20987	0.832480529	0.252155	3.301469	9.62E-04	5.84E-03	6.541497	6.50326	6.411182	5.829815	5.310931	5.635566
AT2G28080	92.10001	0.834012359	0.235096	3.547541	3.89E-04	2.66E-03	7.010043	6.704699	7.01138	6.102904	5.760417	6.198607
AT3G55510	119.8265	0.834491495	0.201947	4.13222	3.59E-05	3.16E-04	7.152771	7.382284	7.31014	6.476563	6.292644	6.486772
AT3G48100	59.89823	0.834850056	0.263661	3.166382	1.54E-03	8.79E-03	6.424426	6.269058	6.18462	5.379864	5.476813	5.441888
AT5G41400	88.5653	0.835013113	0.225464	3.703531	2.13E-04	1.56E-03	6.872409	6.727085	6.93323	5.99213	6.102626	5.899804
AT3G54720	226.0531	0.835091743	0.192688	4.333906	1.46E-05	0.0001409	8.124783	8.236871	8.236271	7.179211	7.013254	7.685425
AT5G46520	136.5492	0.837173701	0.192895	4.340054	1.42E-05	1.37E-04	7.353328	7.563222	7.489642	6.750572	6.460523	6.602557
AT5G22800	600.734	0.837422232	0.163857	5.110678	3.21E-07	4.16E-06	9.257359	9.791459	9.689957	8.768767	8.712195	8.750466

AT4G18440	160.5852	0.838524804	0.212739	3.941563	8.10E-05	6.59E-04	7.507404	7.858494	7.72712	7.179211	6.610887	6.657142
AT3G55040	229.5288	0.839400042	0.171919	4.882528	1.05E-06	1.24E-05	8.116097	8.097765	8.401053	7.440585	7.391266	7.249225
AT1G72416	106.8218	0.840224693	0.264316	3.178863	1.48E-03	0.0084824	7.392218	6.827194	7.130779	6.530411	5.625572	6.382637
AT5G46510	195.8782	0.84045724	0.171137	4.911011	9.06E-07	1.09E-05	7.936991	8.117671	7.901428	7.245106	6.985988	7.138479
AT5G44130	224.5018	0.840891163	0.255402	3.292418	9.93E-04	6.01E-03	8.18693	7.778582	8.498015	7.212535	7.756543	6.85691
AT5G54585	92.98611	0.840908737	0.249903	3.364938	0.000766	0.0048006	7.147097	6.741819	6.835058	5.694846	6.379024	6.04333
AT5G27120	406.204	0.84242835	0.161636	5.211901	1.87E-07	2.53E-06	8.83519	9.201549	9.05757	8.199376	7.952384	8.336545
AT1G07010	217.2843	0.842490204	0.166751	5.052391	4.36E-07	5.53E-06	8.118998	8.167631	8.112788	7.495767	7.237908	7.08639
AT1G15002	2514.996	0.842931806	0.119777	7.037487	1.96E-12	5.13E-11	11.79187	11.49947	11.67807	10.73222	10.72824	10.95184
AT4G38860	1052.982	0.843733911	0.121513	6.943574	3.82E-12	9.75E-11	10.43036	10.30943	10.46503	9.585078	9.70212	9.367428
AT4G10060	509.9961	0.84420309	0.134604	6.271753	3.57E-10	7.08E-09	9.211886	9.473915	9.389637	8.400731	8.516472	8.596044
AT3G09905	265.6674	0.84567591	0.183636	4.605179	4.12E-06	4.41E-05	8.262341	8.28796	8.691703	7.412182	7.603488	7.658921
AT5G64840	1946.807	0.845873773	0.108208	7.817079	5.41E-15	1.82E-13	11.22596	11.39877	11.24022	10.42266	10.5674	10.32479
AT2G33620	167.7874	0.846342216	0.196421	4.308824	1.64E-05	0.0001561	7.529377	7.878633	7.867447	7.074357	6.841348	6.777007
AT1G62780	234.9701	0.846819464	0.178463	4.745079	2.08E-06	2.36E-05	8.035477	8.310373	8.395765	7.353641	7.214606	7.547747
AT1G64770	312.8791	0.849977209	0.172892	4.916221	8.82E-07	1.06E-05	8.414137	8.817633	8.721599	7.600176	7.952384	7.827061
AT1G66930	75.38991	0.850595874	0.252601	3.367352	7.59E-04	0.004767	6.823449	6.50326	6.560439	5.597298	5.625572	5.958939
AT1G31190	130.6586	0.851043261	0.214926	3.959704	7.50E-05	6.16E-04	7.180808	7.396321	7.613262	6.582321	6.680573	6.360876
AT4G22830	101.1173	0.852283781	0.247075	3.4495	5.62E-04	3.68E-03	6.823449	6.990318	7.281752	5.786214	6.460523	6.198607
AT5G13770	872.3594	0.853482713	0.117117	7.287419	3.16E-13	8.91E-12	10.05047	10.23653	10.11371	9.171726	9.415787	9.242095
AT1G66940	395.1491	0.853578211	0.169191	5.045046	4.53E-07	5.74E-06	9.194114	8.737199	9.032232	8.295811	8.060895	8.019904
AT3G03840	77.10252	0.853779292	0.23864	3.577682	3.47E-04	2.40E-03	6.78012	6.57083	6.61607	5.694846	5.883728	5.773726
AT3G08225	77.67383	0.854461786	0.23355	3.658575	2.54E-04	1.82E-03	6.735449	6.611509	6.660846	5.786214	5.82339	5.773726
AT1G20691	77.56758	0.85465472	0.240777	3.549564	3.86E-04	2.64E-03	6.816318	6.528971	6.652001	5.829815	5.760417	5.773726
AT2G04530	66.89428	0.854677545	0.259587	3.292449	9.93E-04	6.01E-03	6.337426	6.376816	6.660846	5.545934	5.476813	5.671366
AT1G52240	1529.628	0.855582863	0.143771	5.951014	2.66E-09	4.70E-08	10.83326	10.8278	11.15944	9.885479	10.26398	10.0754
AT1G77940	694.4834	0.855980857	0.104875	8.161928	3.30E-16	1.22E-14	9.776878	9.794112	9.85512	8.912575	8.970488	8.960371
AT5G55400	94.07332	0.856927632	0.21581	3.970754	7.16E-05	0.0005912	7.010043	6.920804	6.896258	6.030007	6.102626	6.070398
AT2G26220	95.51603	0.857804858	0.219067	3.915728	9.01E-05	0.0007253	7.034833	6.959136	6.8812	6.102904	6.247434	5.929675
AT5G13630	9763.969	0.859581156	0.100016	8.59442	8.37E-18	3.57E-16	13.50319	13.66879	13.68758	12.86691	12.73939	12.6559
AT1G71810	86.68616	0.85978658	0.231596	3.712448	0.000205	0.0015061	6.67365	6.820269	6.976381	5.913254	5.997325	5.929675
AT3G61620	142.835	0.860957163	0.250802	3.432817	0.000597	0.003882	7.49852	7.525402	7.631374	7.179211	6.050936	6.466537
AT1G56110	488.125	0.861410046	0.183162	4.702987	2.56E-06	2.87E-05	9.146569	9.553319	9.202419	8.356696	8.137237	8.708006
AT5G09585	2920.139	0.863205624	0.109141	7.909106	2.59E-15	8.93E-14	11.97013	11.87434	11.80043	10.87043	10.99593	11.15437

AT2G13790	211.9392	0.863882536	0.192058	4.498028	6.86E-06	7.04E-05	8.217028	7.993895	8.109561	7.561902	7.040014	7.004552
AT3G49530	312.8422	0.864031828	0.172001	5.02342	5.08E-07	6.37E-06	8.574902	8.630402	8.791725	8.087074	7.603488	7.613635
AT1G65190	225.2646	0.86403236	0.236272	3.656942	2.55E-04	1.83E-03	8.553616	8.128923	7.832646	7.63746	7.09209	7.112669
AT1G51660	141.3673	0.865253556	0.206394	4.192249	2.76E-05	2.50E-04	7.392218	7.499627	7.701631	6.817077	6.292644	6.72685
AT2G34860	449.4416	0.866221457	0.173773	4.984786	6.20E-07	7.68E-06	8.937336	9.112239	9.464793	8.240343	8.45814	8.22051
AT2G05070	23712.23	0.867333668	0.151598	5.721277	1.06E-08	1.70E-07	14.85817	14.75377	15.09086	13.89788	14.3071	13.82577
AT4G39710	226.7744	0.868330269	0.198792	4.368045	1.25E-05	0.0001223	7.99183	8.065919	8.517623	7.26112	7.26084	7.416778
AT1G20690	77.07387	0.868547501	0.242368	3.583596	0.000339	0.0023505	6.816318	6.520451	6.652001	5.829815	5.69457	5.773726
AT2G44290	155.3783	0.869185026	0.278084	3.125622	1.77E-03	9.93E-03	7.766158	7.391657	7.816905	7.195969	6.871459	5.987621
AT5G02905	248.8327	0.869885774	0.192219	4.525484	6.03E-06	6.23E-05	8.476542	8.460437	8.053574	7.195969	7.510873	7.613635
AT5G28770	568.7623	0.869994257	0.126106	6.898888	5.24E-12	1.32E-10	9.560259	9.392221	9.621383	8.564547	8.678527	8.699362
AT5G27240	124.2845	0.871963375	0.262132	3.326428	0.00088	0.0053986	7.328478	7.533893	7.211253	6.030007	6.102626	6.947296
AT4G04955	114.0498	0.872153137	0.209327	4.166466	3.09E-05	2.76E-04	7.292953	7.180772	7.180831	6.066916	6.574739	6.338783
AT3G25120	94.30056	0.873963977	0.259502	3.367846	7.58E-04	4.76E-03	7.016281	6.651072	7.143456	6.270495	6.200761	5.635566
AT4G34630	105.4835	0.873991242	0.206333	4.235836	2.28E-05	2.10E-04	7.095007	7.068373	7.16848	6.270495	6.247434	6.148682
AT1G68890	228.4911	0.875397498	0.185465	4.720017	2.36E-06	2.65E-05	8.05075	8.467103	8.109561	7.195969	7.370331	7.406194
AT5G49740	852.9011	0.875470391	0.12705	6.890764	5.55E-12	1.39E-10	10.07682	10.09811	10.16668	9.444897	9.071223	9.137193
AT2G28950	821.4786	0.875736323	0.133648	6.552562	5.66E-11	1.25E-09	10.0244	9.942591	10.1974	8.977016	9.320774	9.208984
AT5G18255	855.8344	0.875830575	0.187531	4.670334	3.01E-06	3.32E-05	10.29774	9.777226	10.24229	9.52666	8.970488	9.114615
AT5G25120	79.34292	0.879301931	0.238235	3.690897	0.000223	0.0016313	6.735449	6.799293	6.61607	5.829815	5.553109	5.958939
AT2G47490	125.0364	0.880067556	0.193498	4.54821	5.41E-06	5.64E-05	7.318417	7.32473	7.418413	6.391825	6.574739	6.425197
AT1G31970	426.3967	0.880592399	0.164156	5.364361	8.12E-08	1.16E-06	9.119301	9.332638	8.875795	8.215903	8.086794	8.342109
AT1G77500	92.81112	0.881392991	0.263412	3.346064	8.20E-04	0.0050986	6.787432	7.080015	6.947758	6.503738	5.553109	5.806294
AT1G36310	160.844	0.882757474	0.180923	4.879176	1.07E-06	1.26E-05	7.638536	7.746067	7.776785	6.656843	6.810594	6.961824
AT2G40520	89.41719	0.884289516	0.236096	3.745468	0.00018	0.0013368	7.071238	6.827194	6.712804	5.99213	6.102626	5.838142
AT5G35630	8686.066	0.885021041	0.174059	5.084616	3.68E-07	4.72E-06	13.15897	13.55908	13.6391	12.20774	12.7915	12.62771
AT3G12270	104.6418	0.88944901	0.218569	4.069416	4.71E-05	4.04E-04	7.191872	7.196901	6.911161	6.102904	6.292644	6.198607
AT5G18050	525.4091	0.889589481	0.152898	5.818204	5.95E-09	9.91E-08	9.542001	9.177365	9.511992	8.538503	8.644055	8.375048
AT1G65295	131.8165	0.890695806	0.220556	4.038407	5.38E-05	4.55E-04	7.642574	7.300054	7.337979	6.750572	6.574739	6.222936
AT5G08050	503.3467	0.89220726	0.171607	5.199135	2.00E-07	2.69E-06	9.274374	9.22271	9.555326	8.443461	8.72049	8.165086
AT2G19620	68.16287	0.893311809	0.267376	3.341034	8.35E-04	5.17E-03	6.396005	6.30918	6.762956	5.492674	5.625572	5.598855
AT1G35320	72.62236	0.894789647	0.267936	3.339568	8.39E-04	5.19E-03	6.497586	6.719662	6.579222	6.066916	5.123469	5.598855
AT4G09650	3841.045	0.895062588	0.182523	4.903838	9.40E-07	1.13E-05	12.04469	12.30719	12.4952	11.00846	11.70918	11.33194
AT2G44500	327.1196	0.895244878	0.15165	5.903353	3.56E-09	6.13E-08	8.68667	8.622462	8.904006	7.743857	7.835043	7.911813

AT2G30250	628.7935	0.896489994	0.217142	4.12858	3.65E-05	3.21E-04	9.587744	9.749244	9.726224	9.273916	8.438159	8.39124
AT1G68870	54.74522	0.896545032	0.286597	3.128244	0.001759	0.0098448	6.357218	6.061572	6.159877	4.896561	5.310931	5.482759
AT5G02890	126.6933	0.897936305	0.218672	4.106315	4.02E-05	0.0003498	7.328478	7.40097	7.386775	6.362435	6.871459	6.123056
AT2G27402	78.48609	0.898410138	0.264918	3.391275	6.96E-04	4.42E-03	6.865515	6.395572	6.803457	5.437371	5.997325	5.838142
AT3G06170	326.6899	0.898466874	0.139612	6.435462	1.23E-10	2.61E-09	8.757265	8.644192	8.819638	7.810665	7.772588	7.89677
AT5G08180	316.0367	0.899513065	0.150543	5.975112	2.30E-09	4.10E-08	8.618605	8.735359	8.740496	7.755209	7.567152	7.963265
AT3G18890	678.2502	0.901140308	0.140015	6.436025	1.23E-10	2.61E-09	9.563457	9.847027	9.94113	8.812891	8.942394	8.886142
AT3G06680	187.3532	0.901765937	0.163516	5.514838	3.49E-08	5.24E-07	7.930401	7.937414	7.956343	6.941541	7.11744	7.032351
AT3G06900	6903.184	0.902015408	0.1716	5.256506	1.47E-07	2.01E-06	13.18187	12.86345	13.34066	12.07475	11.99386	12.52994
AT1G04180	66.93919	0.90203741	0.277055	3.255811	1.13E-03	6.73E-03	6.541497	6.386224	6.531797	5.99213	5.019734	5.399825
AT3G06895	6903.287	0.902046029	0.171607	5.25647	1.47E-07	2.01E-06	13.18187	12.86345	13.34074	12.07475	11.99386	12.52994
AT1G68570	144.8804	0.902108312	0.206562	4.367252	1.26E-05	1.23E-04	7.372904	7.731377	7.594919	6.817077	6.714193	6.425197
AT3G57490	133.208	0.902293284	0.190898	4.726572	2.28E-06	2.57E-05	7.439402	7.377575	7.543241	6.607592	6.537663	6.466537
AT3G21300	153.1887	0.902531134	0.190103	4.747598	2.06E-06	2.34E-05	7.713388	7.723975	7.528819	6.941541	6.574739	6.657142
AT1G68410	277.4819	0.903058521	0.184127	4.904539	9.36E-07	1.12E-05	8.358761	8.538459	8.614134	7.874516	7.567152	7.272722
AT4G18280	61.29766	0.903167732	0.283099	3.190285	1.42E-03	8.22E-03	6.255428	6.376816	6.40066	4.896561	5.883728	5.356498
AT2G34510	654.0916	0.903444355	0.158442	5.702047	1.18E-08	1.89E-07	9.788769	9.617639	9.806131	8.991487	8.963516	8.479713
AT3G11120	417.2575	0.904619841	0.132784	6.812732	9.58E-12	2.32E-10	8.965009	9.136136	9.168711	8.148628	8.244671	8.152475
AT5G39660	315.5871	0.905129029	0.19779	4.576214	4.73E-06	5.00E-05	8.996901	8.489101	8.560791	7.984706	7.472095	7.786827
AT3G18130	321.1587	0.905289941	0.152037	5.954395	2.61E-09	4.62E-08	8.551471	8.773502	8.82555	7.799743	7.690514	7.889188
AT5G17300	235.073	0.905319133	0.275103	3.290835	9.99E-04	6.04E-03	8.723353	8.074674	7.923646	7.821505	6.985988	7.004552
AT3G48200	403.4834	0.905384503	0.151834	5.963	2.48E-09	4.39E-08	8.885464	9.217449	9.02882	8.157211	7.966403	8.232543
AT2G09225	165.1296	0.905653431	0.240985	3.758138	1.71E-04	1.28E-03	7.721046	7.639949	7.91258	7.056115	7.11744	6.198607
AT4G17730	549.0862	0.906311055	0.134253	6.750788	1.47E-11	3.48E-10	9.645221	9.341129	9.466055	8.62148	8.581674	8.519541
AT3G19170	1305.191	0.906482899	0.156509	5.791893	6.96E-09	1.15E-07	10.39669	10.87409	10.89754	9.900892	9.685267	9.853789
AT1G52340	112.187	0.90703687	0.225178	4.028086	5.62E-05	0.0004741	6.98482	7.396321	7.246933	6.448866	6.050936	6.293554
AT1G03930	349.4535	0.90967963	0.145819	6.238419	4.42E-10	8.62E-09	8.725258	8.850293	8.949832	8.022777	7.740317	7.963265
AT1G16720	1347.606	0.91032786	0.127004	7.167704	7.63E-13	2.10E-11	10.63877	10.75341	10.94312	9.913613	9.965564	9.710588
AT3G54490	71.41788	0.910420663	0.284636	3.198539	0.001381	0.0080155	6.70488	6.806319	6.159877	5.492674	5.476813	5.838142
AT1G10360	260.9981	0.9107367	0.186033	4.895555	9.80E-07	1.17E-05	8.664999	8.189292	8.390457	7.612711	7.283413	7.547747
AT2G43320	123.483	0.910862833	0.205651	4.429168	9.46E-06	9.43E-05	7.197372	7.419417	7.444257	6.476563	6.200761	6.526408
AT4G04570	469.1543	0.910959873	0.205033	4.442983	8.87E-06	8.88E-05	9.66719	8.978676	9.060915	8.436427	8.40766	8.146128
AT5G55220	674.0154	0.91134663	0.148099	6.153633	7.57E-10	1.44E-08	9.595055	9.869842	9.882789	8.627669	8.935284	8.999626
AT4G17600	459.6943	0.912585039	0.159211	5.731935	9.93E-09	1.60E-07	9.113495	9.23838	9.343688	8.022777	8.572538	8.302704

AT4G38940	70.54879	0.912867033	0.262138	3.482391	4.97E-04	3.30E-03	6.697136	6.511881	6.48276	5.913254	5.123469	5.635566
AT1G33110	263.8666	0.912900651	0.160272	5.69594	1.23E-08	1.95E-07	8.3365	8.49346	8.478136	7.709256	7.472095	7.341001
AT3G27960	139.1014	0.913190378	0.205713	4.439154	9.03E-06	9.03E-05	7.323457	7.693983	7.533642	6.420628	6.610887	6.709736
AT5G46910	114.1588	0.914688907	0.256699	3.563277	0.000366	0.0025225	7.047071	7.50827	7.149752	6.727704	5.941644	6.070398
AT3G02795	227.5885	0.914928065	0.260721	3.509218	4.49E-04	3.03E-03	8.275402	8.471529	7.93827	6.96128	6.810594	7.811102
AT2G41880	99.52661	0.915797936	0.226357	4.045815	5.21E-05	4.42E-04	7.219167	6.868059	7.018279	6.066916	6.247434	6.015745
AT3G44190	213.5635	0.917002694	0.180589	5.077835	3.82E-07	4.89E-06	8.071865	8.310373	8.026456	7.397768	6.958197	7.201051
AT1G76080	975.3942	0.918800559	0.151729	6.055523	1.40E-09	2.57E-08	10.28424	10.18693	10.4776	9.209354	9.668214	9.262777
AT5G62440	83.94307	0.91920279	0.260938	3.522685	0.000427	0.002891	6.757957	7.056636	6.606946	5.913254	5.396255	6.096967
AT4G10300	418.0579	0.919641447	0.178813	5.143037	2.70E-07	3.55E-06	8.89903	9.150011	9.232395	7.945604	8.506912	8.005951
AT2G40140	529.297	0.919862714	0.235618	3.90404	9.46E-05	0.0007547	9.536586	9.525916	9.28483	8.991487	7.819681	8.423089
AT1G04680	333.7744	0.921101877	0.156147	5.898949	3.66E-09	6.28E-08	8.70223	8.731673	8.892788	7.58753	8.021154	7.904311
AT3G50340	181.6315	0.921577853	0.215952	4.267508	1.98E-05	1.84E-04	7.601678	7.865238	8.206377	7.127736	6.810594	6.917792
AT1G72450	625.9186	0.924506896	0.189495	4.878795	1.07E-06	1.27E-05	9.676082	9.692546	9.700719	9.171726	8.497289	8.433551
AT1G29465	409.3621	0.926496963	0.165409	5.601262	2.13E-08	3.28E-07	8.973048	8.981783	9.24862	8.311275	8.209741	7.858459
AT3G22660	168.1502	0.926696047	0.22013	4.209776	2.56E-05	2.33E-04	7.705689	8.089149	7.571658	7.074357	6.537663	6.85691
AT4G33666	201.3876	0.92895958	0.174286	5.330077	9.82E-08	1.38E-06	8.092675	7.965931	8.08677	7.056115	7.370331	6.917792
AT4G03905	680.1804	0.931343274	0.241851	3.850892	1.18E-04	9.17E-04	9.637149	9.830752	9.975212	8.682211	8.209741	9.381005
AT4G15760	2567.225	0.931838585	0.104484	8.918507	4.73E-19	2.26E-17	11.71845	11.68004	11.7619	10.92799	10.7668	10.63917
AT2G40205	288.6297	0.932886389	0.187667	4.970958	6.66E-07	8.21E-06	8.46974	8.387355	8.823582	7.522586	7.880172	7.458357
AT3G06880	207.4086	0.93450238	0.237697	3.931486	8.44E-05	0.0006845	7.755012	8.167631	8.327972	7.368501	7.327526	6.639175
AT1G74070	169.3175	0.936130551	0.197765	4.733544	2.21E-06	2.49E-05	7.780888	7.73874	7.897692	6.476563	7.166841	6.872373
AT3G60960	88.27382	0.936230108	0.243771	3.840612	0.000123	0.0009496	6.965608	6.861328	6.858314	5.694846	5.625572	6.270397
AT3G23580	112.0712	0.937005421	0.240355	3.89843	9.68E-05	7.69E-04	6.946137	7.305023	7.413188	6.391825	5.82339	6.425197
AT4G32590	362.6795	0.937123801	0.155144	6.040335	1.54E-09	2.80E-08	8.747925	8.840059	9.106947	7.965288	7.894906	7.991862
AT1G18740	500.7442	0.937697102	0.282287	3.321788	8.94E-04	5.48E-03	9.63411	9.450561	8.999486	9.043339	7.819681	7.970467
AT4G04850	249.4181	0.937782067	0.167937	5.584122	2.35E-08	3.61E-07	8.238529	8.50214	8.366328	7.46844	7.166841	7.547747
AT3G50800	75.86812	0.939530472	0.242076	3.881133	1.04E-04	8.19E-04	6.633638	6.6743	6.669636	5.694846	5.941644	5.482759
AT5G39850	185.6364	0.939760158	0.223219	4.210036	2.55E-05	2.33E-04	7.824194	8.048247	7.995326	6.838581	6.499609	7.395531
AT1G09070	1983.039	0.940432603	0.181986	5.167611	2.37E-07	3.14E-06	11.42691	11.29301	11.34751	10.8064	10.20608	10.05522
AT3G27700	1981.551	0.942269466	0.131708	7.154241	8.41E-13	2.30E-11	11.26969	11.26396	11.50653	10.30979	10.61044	10.25866
AT3G28930	99.81153	0.943992374	0.286576	3.294043	0.000988	0.0059794	7.169658	7.285044	6.729716	6.332435	5.220243	6.338783
AT1G30410	308.6948	0.94434704	0.207729	4.546062	5.47E-06	5.70E-05	8.49005	8.975563	8.534564	7.97503	7.305638	7.745439
AT1G04013	152.6077	0.945305078	0.220671	4.283768	1.84E-05	1.73E-04	7.614069	7.43763	7.927316	6.476563	6.610887	6.932619

AT3G26700	84.04761	0.945396268	0.263019	3.594403	0.000325	0.0022684	6.844636	6.74913	6.850603	6.270495	5.760417	5.356498
AT5G15350	551.4135	0.946793523	0.194912	4.857556	1.19E-06	1.40E-05	9.336761	9.288162	9.844484	8.422254	8.824203	8.353172
AT1G18390	88.27926	0.947773727	0.249005	3.806238	0.000141	0.0010746	6.90639	6.952817	6.771147	6.138016	6.102626	5.441888
AT4G08815	141.1006	0.947907087	0.192624	4.921016	8.61E-07	1.04E-05	7.568105	7.538119	7.533642	6.656843	6.779171	6.338783
AT1G29460	290.6013	0.948084336	0.169538	5.592172	2.24E-08	3.45E-07	8.670942	8.368491	8.698162	7.574773	7.819681	7.488773
AT1G08747	121.2164	0.948805451	0.214603	4.421204	9.82E-06	9.75E-05	7.235299	7.249399	7.514252	6.102904	6.499609	6.466537
AT5G11970	135.8404	0.949129394	0.206916	4.587027	4.50E-06	4.76E-05	7.576572	7.353794	7.581008	6.773083	6.200761	6.526408
AT3G23810	504.1992	0.950021701	0.149662	6.3478	2.18E-10	4.48E-09	9.316685	9.512016	9.321548	8.224096	8.344656	8.641876
AT1G04107	159.0274	0.950103741	0.215681	4.405131	1.06E-05	0.0001046	7.927094	7.386978	7.828726	6.727704	6.646151	6.85691
AT1G09513	130.1686	0.951875594	0.206743	4.604143	4.14E-06	4.43E-05	7.387414	7.290065	7.635867	6.530411	6.336481	6.506726
AT4G13570	146.3956	0.951974234	0.240821	3.953042	7.72E-05	0.0006317	7.747532	7.77142	7.258633	6.941541	6.610887	6.270397
AT5G08600	68.93916	0.951979276	0.27911	3.410773	0.000648	0.0041656	6.386406	6.799293	6.40066	5.123743	5.69457	5.740407
AT3G62260	127.6544	0.952553784	0.228905	4.161343	3.16E-05	2.82E-04	7.251253	7.499627	7.509364	6.750572	5.883728	6.486772
AT5G44930	119.6625	0.954643318	0.211936	4.504403	6.66E-06	6.84E-05	7.141402	7.391657	7.423619	6.530411	6.200761	6.270397
AT1G78230	144.5151	0.954716672	0.213444	4.472917	7.72E-06	7.82E-05	7.328478	7.70154	7.731324	6.476563	6.499609	6.809501
AT3G47420	273.1941	0.955487126	0.181333	5.269239	1.37E-07	1.89E-06	8.390321	8.69807	8.422015	7.743857	7.190921	7.576354
AT1G26945	316.2562	0.955622424	0.198346	4.817946	1.45E-06	1.68E-05	8.326495	8.75909	8.994247	7.649677	7.723906	7.827061
AT1G14150	212.0949	0.955727347	0.218318	4.377678	1.20E-05	0.0001174	7.998147	8.012241	8.374416	6.795248	7.567152	7.046052
AT3G48720	542.118	0.956823149	0.164666	5.810704	6.22E-09	1.03E-07	9.582499	9.181424	9.661596	8.570985	8.599774	8.380466
AT1G09390	121.7901	0.957440034	0.205192	4.666074	3.07E-06	3.38E-05	7.333483	7.329615	7.397399	6.607592	6.050936	6.360876
AT1G06467	287.0036	0.958440117	0.178941	5.356181	8.50E-08	1.20E-06	8.68667	8.650062	8.393114	7.426454	7.432247	7.866203
AT3G62030	2556.495	0.958930506	0.176781	5.424396	5.82E-08	8.45E-07	11.32852	11.74479	12.02875	10.59681	10.92053	10.7108
AT5G66600	49.77257	0.959476761	0.300747	3.190311	0.001421	0.0082221	5.829063	6.25885	6.134702	5.319969	4.907958	4.953151
AT3G46670	75.42545	0.959784278	0.298637	3.213887	1.31E-03	7.66E-03	6.276368	6.977926	6.721284	5.872138	5.019734	5.869303
AT5G18060	529.8596	0.960037438	0.152187	6.308268	2.82E-10	5.70E-09	9.545239	9.259011	9.551764	8.682211	8.417898	8.330959
AT4G27600	741.0325	0.960477999	0.11321	8.484022	2.18E-17	8.94E-16	9.93915	9.882363	9.994582	9.093392	8.928139	8.886142
AT3G24480	192.6002	0.961430242	0.194273	4.948869	7.46E-07	9.11E-06	7.795468	7.959642	8.230342	7.179211	6.958197	6.917792
AT4G04800	99.5097	0.961897889	0.236854	4.061151	4.88E-05	4.17E-04	7.106746	6.965426	7.078929	6.172293	6.379024	5.635566
AT1G15820	21231.39	0.962804934	0.162645	5.919684	3.23E-09	5.60E-08	14.57791	14.65427	15.0668	13.63589	14.04941	13.68249
AT5G01810	252.2765	0.962936076	0.151717	6.346938	2.20E-10	4.50E-09	8.321467	8.419785	8.427208	7.535811	7.391266	7.318598
AT1G12020	87.32445	0.962998993	0.256354	3.756516	0.000172	0.0012848	6.965608	6.734471	6.911161	5.319969	6.247434	5.958939
AT1G57990	658.6617	0.96418075	0.250232	3.853154	1.17E-04	9.10E-04	9.898363	9.813421	9.623646	9.32051	8.669986	8.067702
AT5G42250	103.9229	0.965449062	0.280017	3.447821	5.65E-04	3.70E-03	6.681522	7.153483	7.439125	6.066916	6.499609	5.740407
AT1G64100	56.21104	0.966505591	0.274861	3.516345	0.000438	0.0029528	6.179644	6.414087	6.159877	5.25748	5.220243	5.265734

AT3G55010	133.9074	0.966929855	0.217384	4.44803	8.67E-06	8.70E-05	7.303193	7.563222	7.608698	6.391825	6.200761	6.777007
AT5G25440	122.643	0.967278399	0.289193	3.344751	8.24E-04	5.12E-03	7.721046	6.977926	7.332454	6.859769	5.883728	6.096967
AT4G11900	116.5194	0.967329783	0.233613	4.140736	3.46E-05	3.05E-04	7.287806	7.32473	7.276007	6.727704	5.82339	6.148682
AT4G30610	57.64327	0.970362824	0.283386	3.424174	6.17E-04	3.99E-03	6.286726	6.130373	6.431998	5.545934	5.310931	4.953151
AT3G59670	71.26252	0.971163138	0.252447	3.847	1.20E-04	9.30E-04	6.567215	6.7122	6.47275	5.741253	5.625572	5.399825
AT1G19670	146.1661	0.971456701	0.261022	3.721737	1.98E-04	1.46E-03	7.622271	7.731377	7.499537	7.162256	6.336481	6.096967
AT5G67370	171.4992	0.971856214	0.176648	5.501642	3.76E-08	5.61E-07	7.9337	7.838071	7.739697	6.750572	6.958197	6.85691
AT3G12110	80.42162	0.973946179	0.237435	4.101941	4.10E-05	3.56E-04	6.6255	6.827194	6.827223	5.646896	5.82339	5.806294
AT4G34950	106.1288	0.973984814	0.267929	3.635235	2.78E-04	0.0019712	6.697136	7.212852	7.489642	6.270495	5.883728	6.222936
AT5G62430	317.084	0.975146013	0.144241	6.760516	1.38E-11	3.27E-10	8.74605	8.696181	8.715245	7.821505	7.850244	7.547747
AT1G44575	3900.389	0.976360317	0.157659	6.192865	5.91E-10	1.14E-08	12.11176	12.26207	12.60811	11.51521	11.3871	11.11754
AT4G06805	1407.945	0.976748549	0.170263	5.736698	9.65E-09	1.56E-07	10.77881	10.71005	11.10011	10.05121	10.01023	9.520031
AT4G06810	1408.036	0.976894589	0.170231	5.738627	9.54E-09	1.55E-07	10.77881	10.71052	11.10011	10.05121	10.01023	9.520031
AT1G71140	80.59047	0.976973425	0.309521	3.156402	0.001597	0.0090445	6.735449	6.914316	6.695692	6.391825	4.907958	5.482759
AT1G74730	455.0978	0.97725284	0.156045	6.262623	3.79E-10	7.48E-09	9.18168	9.126344	9.412074	8.471261	8.173944	8.087712
AT3G03830	75.44481	0.978776325	0.243919	4.012714	6.00E-05	5.03E-04	6.78012	6.562555	6.660846	5.545934	5.82339	5.635566
AT1G23740	503.4931	0.979940215	0.153237	6.394932	1.61E-10	3.35E-09	9.306541	9.363928	9.494782	8.639969	8.365965	8.152475
AT1G80610	52.48485	0.980322008	0.295342	3.319281	9.02E-04	5.52E-03	6.201706	5.989326	6.302344	5.492674	4.786789	5.01018
AT5G46470	404.6015	0.983447529	0.151934	6.472857	9.62E-11	2.07E-09	8.929095	9.141702	9.157818	8.288016	7.894906	8.019904
AT2G24150	206.7239	0.98366026	0.16577	5.933883	2.96E-09	5.18E-08	8.150531	8.021327	8.17585	7.195969	6.92986	7.176347
AT1G06263	126.1461	0.985068119	0.220199	4.473526	7.69E-06	7.80E-05	7.256532	7.363353	7.581008	6.656843	6.379024	6.123056
AT4G10770	188.4061	0.985206991	0.192997	5.104792	3.31E-07	4.27E-06	7.713388	8.05711	8.141508	6.880651	7.040014	7.004552
AT4G04940	100.8651	0.986550318	0.24962	3.952206	7.74E-05	0.0006333	6.919761	7.32473	7.03878	6.138016	5.553109	6.338783
AT4G37910	948.7153	0.99089198	0.118487	8.362859	6.12E-17	2.42E-15	10.15805	10.37411	10.38055	9.347011	9.195448	9.361961
AT1G32920	397.3929	0.99090439	0.235773	4.202795	2.64E-05	2.39E-04	9.070681	8.913474	9.196349	8.577394	7.548635	7.745439
AT1G27770	792.2051	0.991332434	0.139404	7.111194	1.15E-12	3.12E-11	9.940791	10.0895	10.10483	9.262029	9.018374	8.823881
AT2G39470	775.8859	0.991753564	0.140225	7.07259	1.52E-12	4.04E-11	9.83361	10.00881	10.18442	9.015287	9.153175	8.870827
AT4G24380	60.52711	0.993016849	0.296848	3.345202	0.000822	0.005112	6.317359	6.414087	6.40066	5.872138	4.786789	5.065041
AT1G63750	188.0072	0.993664456	0.165061	6.02	1.74E-09	3.17E-08	7.943552	7.990814	8.002302	7.056115	6.958197	6.90281
AT1G04530	175.9828	0.994292134	0.182383	5.451662	4.99E-08	7.32E-07	8.013818	7.810381	7.84434	6.999965	6.610887	6.947296
AT4G38970	8254.563	0.994367244	0.148162	6.71133	1.93E-11	4.51E-10	13.2394	13.34644	13.66721	12.48374	12.56553	12.18263
AT4G37080	370.4633	0.994393052	0.140251	7.090096	1.34E-12	3.59E-11	8.847487	9.018548	9.001228	7.945604	7.804154	8.06097
AT1G09340	2507.945	0.995079851	0.142486	6.983721	2.87E-12	7.40E-11	11.47129	11.67093	11.94908	10.67957	10.82566	10.59324
AT2G09700	176.1451	0.995829153	0.297124	3.351563	8.04E-04	5.01E-03	7.224564	8.167631	8.138345	7.323456	6.537663	6.506726

AT4G15800	307.6987	0.996078225	0.19121	5.209333	1.90E-07	2.56E-06	8.585428	8.469318	8.962407	7.743857	7.835043	7.427286
AT2G41105	1050.629	0.997702489	0.238419	4.184668	2.86E-05	2.58E-04	10.56835	10.25655	10.55377	9.965846	9.253768	8.827852
AT2G36870	105.1939	0.998130804	0.227561	4.386208	1.15E-05	1.13E-04	7.256532	7.131273	7.052287	5.741253	6.420349	6.17386
AT4G39280	250.1792	0.999286428	0.156294	6.393617	1.62E-10	3.38E-09	8.368546	8.475942	8.322408	7.212535	7.491614	7.427286
AT4G13340	424.5288	0.999656561	0.223331	4.476126	7.60E-06	7.72E-05	8.979448	9.032096	9.41991	8.478128	8.185975	7.585765
AT5G27360	87.60945	0.999666046	0.233689	4.277771	1.89E-05	1.77E-04	6.899657	6.984135	6.795447	5.597298	5.941644	6.015745
AT2G26215	49.99984	1.002054836	0.283628	3.53299	0.000411	0.0027924	6.168486	6.084872	6.042961	5.051918	5.123469	5.01018
AT1G64860	993.6051	1.002276781	0.146995	6.818433	9.20E-12	2.25E-10	10.19975	10.63114	10.26494	9.43787	9.379578	9.265708
AT4G25290	107.7133	1.002974632	0.216182	4.639486	3.49E-06	3.80E-05	7.197372	7.280005	7.06567	5.953231	6.379024	6.148682
AT1G17360	413.533	1.003015841	0.155082	6.467631	9.96E-11	2.13E-09	9.009462	9.270488	9.065918	8.2723	7.835043	8.13335
AT3G19360	54.26389	1.003860013	0.28349	3.541077	3.98E-04	2.72E-03	6.15724	6.279193	6.2564	5.319969	4.654502	5.356498
AT1G60590	76.76581	1.00523313	0.249561	4.028013	5.63E-05	4.74E-04	6.541497	6.874758	6.678374	5.741253	5.760417	5.522505
AT1G49245	7319.137	1.005344912	0.17005	5.912058	3.38E-09	5.84E-08	13.27674	12.9963	13.47327	12.08884	12.0151	12.54056
AT5G62520	203.1621	1.005765582	0.181249	5.549069	2.87E-08	4.35E-07	8.161829	8.054161	8.080192	7.368501	6.810594	6.961824
AT1G74640	87.83246	1.006010434	0.242625	4.146367	3.38E-05	2.98E-04	6.712583	6.940097	7.025145	6.102904	5.82339	5.635566
AT3G07860	272.2767	1.006023423	0.209356	4.805332	1.54E-06	1.79E-05	8.49005	8.478144	8.602704	6.980752	7.452309	7.866203
AT4G25340	131.1486	1.007173327	0.220978	4.5578	5.17E-06	5.41E-05	7.31336	7.69019	7.392097	6.138016	6.499609	6.620982
AT1G08633	50.83138	1.007193142	0.307109	3.2796	0.00104	0.0062545	6.002463	5.989326	6.346869	5.319969	5.220243	4.62832
AT3G26570	386.236	1.007568378	0.156837	6.424284	1.32E-10	2.80E-09	8.930747	8.888864	9.211477	8.087074	8.060895	7.842845
AT1G09800	1112.321	1.008298079	0.244784	4.119142	3.80E-05	3.33E-04	10.2797	10.66535	10.68421	8.845121	9.379578	10.02441
AT5G60280	129.9066	1.010495616	0.229483	4.403352	1.07E-05	1.05E-04	7.303193	7.608139	7.479679	6.750572	5.883728	6.446015
AT1G65230	307.2446	1.0105903	0.177239	5.701845	1.19E-08	1.89E-07	8.547169	8.630402	8.868178	7.426454	7.952384	7.585765
AT5G59750	212.9166	1.010663114	0.159432	6.339153	2.31E-10	4.72E-09	8.121893	8.189292	8.178932	7.26112	7.09209	7.059624
AT2G43560	561.1284	1.012730043	0.162207	6.243436	4.28E-10	8.36E-09	9.35287	9.550184	9.74298	8.311275	8.761267	8.494778
AT3G45140	916.1799	1.013314164	0.270224	3.749902	1.77E-04	1.32E-03	9.962758	10.69924	10.06374	9.755854	8.55409	9.054882
AT5G14060	1321.401	1.013899299	0.138975	7.295574	2.97E-13	8.43E-12	10.72222	10.83039	10.82415	10.035	9.637878	9.580122
AT5G44530	161.6797	1.014022836	0.20711	4.896051	9.78E-07	1.17E-05	7.917128	7.878633	7.499537	6.582321	6.680573	6.90281
AT5G02915	218.3662	1.015271085	0.186206	5.452395	4.97E-08	7.30E-07	8.323983	8.310373	7.949141	6.999965	7.214606	7.272722
AT1G05253	231.9215	1.016157238	0.18139	5.602064	2.12E-08	3.27E-07	8.136283	8.200001	8.498015	7.292624	7.391266	7.08639
AT5G67350	59.49788	1.017969711	0.303	3.359632	0.00078	0.0048759	6.255428	6.511881	6.324778	5.786214	4.50885	5.218117
AT3G61220	82.46339	1.018474423	0.290566	3.505137	0.000456	0.0030691	6.376742	6.984135	7.04555	5.597298	5.396255	6.096967
AT1G61300	184.3079	1.018621779	0.167988	6.063674	1.33E-09	2.45E-08	7.92378	8.030356	7.919966	6.980752	6.92986	6.872373
AT5G00590	86.79424	1.019133539	0.254959	3.997247	6.41E-05	5.33E-04	6.851629	6.627465	7.156022	5.953231	5.625572	5.869303
AT4G28220	576.4326	1.019553416	0.13567	7.514927	5.69E-14	1.73E-12	9.472248	9.762852	9.547001	8.471261	8.581674	8.641876

AT3G15540	264.489	1.020636076	0.24757	4.122622	3.75E-05	0.0003284	7.920458	8.784217	8.614134	7.212535	7.756543	7.261021
AT1G07993	92.26293	1.021636168	0.263023	3.884213	1.03E-04	0.0008117	6.681522	6.977926	7.211253	6.138016	6.050936	5.522505
AT3G02370	120.8763	1.021796241	0.237311	4.305728	1.66E-05	1.58E-04	7.60995	7.228629	7.246933	6.391825	5.82339	6.545826
AT1G02380	136.0454	1.022072363	0.224491	4.552837	5.29E-06	5.53E-05	7.580786	7.43763	7.576341	6.901235	6.152528	6.246861
AT3G04770	52.24857	1.022365968	0.305965	3.341449	8.33E-04	5.17E-03	6.244842	6.338556	5.915727	5.492674	4.786789	4.893774
AT1G28600	127.299	1.024637814	0.223859	4.577166	4.71E-06	4.98E-05	7.406536	7.196901	7.662533	6.556599	6.379024	6.198607
AT4G10040	114.9397	1.026218014	0.213274	4.811741	1.50E-06	1.73E-05	7.303193	7.339335	7.241048	6.503738	5.883728	6.222936
AT1G49750	306.7309	1.026826553	0.219082	4.686949	2.77E-06	3.09E-05	8.804884	8.606451	8.687381	8.131308	7.26084	7.352073
AT5G50100	152.4797	1.027622876	0.188712	5.445463	5.17E-08	7.57E-07	7.642574	7.746067	7.662533	6.582321	6.900955	6.466537
AT1G61580	250.2663	1.027650175	0.199855	5.141979	2.72E-07	3.57E-06	8.074856	8.592294	8.505399	7.561902	7.142352	7.307265
AT1G19380	103.764	1.028296816	0.256468	4.009449	6.09E-05	5.10E-04	7.353328	6.704699	7.304507	6.138016	6.050936	6.04333
AT3G13940	113.1717	1.02840792	0.220973	4.654007	3.26E-06	3.57E-05	7.186351	7.391657	7.246933	6.420628	5.760417	6.316346
AT5G24810	211.3811	1.029075999	0.163769	6.283686	3.31E-10	6.58E-09	8.241194	8.134517	8.090048	7.179211	7.190921	6.99045
AT1G62630	105.7143	1.030704721	0.214988	4.794235	1.63E-06	1.88E-05	7.118391	7.254546	7.156022	6.270495	5.82339	6.17386
AT2G27840	139.6789	1.032977464	0.198228	5.211059	1.88E-07	2.54E-06	7.411277	7.686387	7.608698	6.448866	6.420349	6.639175
AT4G39363	490.0118	1.033372038	0.179908	5.743889	9.25E-09	1.50E-07	9.163529	9.569928	9.368196	8.157211	8.099571	8.614552
AT1G24170	380.8943	1.034054297	0.143949	7.183452	6.80E-13	1.88E-11	8.979448	8.921585	9.103707	8.022777	8.086794	7.778644
AT2G42770	131.733	1.035320106	0.202738	5.106688	3.28E-07	4.24E-06	7.328478	7.495286	7.631374	6.391825	6.336481	6.526408
AT1G70000	42.04894	1.037297565	0.326426	3.17774	1.48E-03	8.51E-03	5.679876	6.084872	5.839872	4.29471	4.907958	5.01018
AT3G19450	152.6115	1.038336485	0.200205	5.186377	2.14E-07	2.87E-06	7.848869	7.612154	7.608698	6.362435	6.871459	6.657142
AT1G06273	44.59313	1.039912088	0.314583	3.305688	9.47E-04	5.76E-03	5.741414	6.061572	6.01564	4.97633	5.123469	4.474702
AT3G32940	107.5446	1.040855262	0.24759	4.203948	2.62E-05	2.38E-04	7.245954	7.026869	7.32134	6.556599	5.883728	5.806294
AT4G12320	74.1505	1.041810113	0.286299	3.638884	2.74E-04	1.95E-03	6.532821	6.57083	6.858314	5.492674	6.102626	5.065041
AT3G43720	217.9478	1.042572937	0.20302	5.13532	2.82E-07	3.69E-06	8.068868	8.04528	8.465572	7.03764	7.432247	6.947296
AT3G59410	1164.399	1.042711608	0.111577	9.345228	9.17E-21	4.87E-19	10.59099	10.52653	10.7259	9.552916	9.676766	9.475161
AT1G11870	971.8346	1.045171758	0.175648	5.95038	2.68E-09	4.71E-08	10.26734	10.20697	10.58953	8.97216	9.265154	9.577765
AT1G04733	1072.219	1.047377403	0.253756	4.127503	3.67E-05	3.22E-04	10.2316	10.63706	10.64296	8.723251	9.276451	9.969746
AT2G23600	311.6123	1.048323866	0.231708	4.524332	6.06E-06	6.26E-05	9.112039	8.358966	8.627731	7.95548	7.327526	7.566881
AT3G62150	299.8416	1.049056058	0.212623	4.933882	8.06E-07	9.80E-06	8.826343	8.655908	8.527328	8.041442	7.305638	7.295843
AT3G51860	141.3203	1.049666364	0.238323	4.404386	1.06E-05	1.05E-04	7.287806	7.746067	7.718673	6.880651	6.200761	6.338783
AT5G04190	480.6501	1.050218375	0.19299	5.441816	5.27E-08	7.70E-07	9.267854	9.239678	9.520521	7.884889	8.669986	8.183799
AT1G64105	52.35098	1.050373265	0.284814	3.687924	2.26E-04	1.65E-03	6.087861	6.32883	6.10908	5.051918	5.220243	5.01018
AT1G02205	354.2627	1.051526998	0.243331	4.321393	1.55E-05	1.48E-04	8.383099	9.127747	9.135782	8.059868	7.327526	7.956026
AT4G28660	288.1418	1.051908127	0.21951	4.792069	1.65E-06	1.90E-05	8.43524	8.548969	8.827515	7.09237	7.980286	7.416778

AT3G07215	271.2349	1.053339099	0.18425	5.71689	1.08E-08	1.74E-07	8.399895	8.458209	8.72582	7.46844	7.11744	7.676644
AT1G69840	862.7517	1.054349803	0.12376	8.519316	1.60E-17	6.70E-16	10.10709	10.13136	10.3239	9.262029	9.071223	9.034408
AT3G16810	186.3235	1.057600719	0.174974	6.044326	1.50E-09	2.74E-08	8.029322	8.024343	7.930976	6.859769	6.714193	7.09959
AT1G52230	5692.438	1.05792531	0.163061	6.487922	8.70E-11	1.88E-09	12.79496	12.80666	13.12041	11.59714	12.15096	11.70498
AT5G59550	295.0541	1.058631309	0.165474	6.397551	1.58E-10	3.30E-09	8.698355	8.598378	8.64789	7.853545	7.472095	7.352073
AT1G30290	65.203	1.059139789	0.268072	3.950957	7.78E-05	0.0006362	6.286726	6.68196	6.492702	5.437371	5.396255	5.356498
AT5G43630	110.9552	1.059763025	0.234824	4.513005	6.39E-06	6.59E-05	7.328478	7.319828	7.06567	6.332435	6.379024	5.740407
AT5G08865	824.2449	1.059926542	0.168298	6.297903	3.02E-10	6.07E-09	10.20317	10.2136	9.969884	8.937702	8.753203	9.367428
AT1G73080	497.6398	1.060520047	0.287934	3.683207	0.00023	0.0016743	9.376095	9.58226	9.28483	9.005814	7.567152	7.866203
AT4G36010	64.18525	1.061372699	0.280493	3.783954	0.000154	0.0011645	6.30722	6.348216	6.754717	5.379864	5.123469	5.522505
AT4G32770	97.66152	1.06234413	0.252577	4.206016	2.60E-05	2.36E-04	6.742991	7.175355	7.270239	5.953231	5.69457	6.17386
AT5G23940	242.9962	1.063829829	0.169975	6.258747	3.88E-10	7.64E-09	8.195201	8.435731	8.465572	7.426454	7.305638	7.138479
AT5G05580	54.16191	1.064130959	0.289112	3.680692	2.33E-04	1.69E-03	6.276368	6.24857	6.220956	4.812122	4.907958	5.482759
AT3G55630	85.22558	1.064488104	0.237699	4.4783	7.52E-06	7.65E-05	6.919761	6.719662	6.96214	5.646896	5.997325	5.706299
AT3G44635	54.36832	1.064848181	0.296709	3.58887	3.32E-04	2.31E-03	6.30722	6.196041	6.232868	5.597298	4.786789	4.831847
AT5G13190	389.4262	1.065922554	0.19735	5.401168	6.62E-08	9.54E-07	9.000051	9.107981	9.045801	8.318945	7.980286	7.478706
AT4G00975	116.2536	1.070869102	0.232094	4.613938	3.95E-06	4.25E-05	7.511825	7.131273	7.32134	5.99213	6.050936	6.506726
AT3G04790	398.0842	1.071138627	0.181803	5.891757	3.82E-09	6.55E-08	8.81744	9.086498	9.314559	7.732415	8.060895	8.146128
AT4G21400	78.76418	1.073003988	0.276391	3.882199	1.04E-04	8.17E-04	6.727867	6.946471	6.634148	6.138016	5.220243	5.399825
AT2G22500	71.07103	1.07303512	0.327007	3.281379	1.03E-03	0.0062266	6.757957	6.269058	6.858314	5.741253	4.346824	5.838142
AT4G39940	138.3557	1.073614136	0.229303	4.682086	2.84E-06	3.16E-05	7.453261	7.85511	7.381434	6.632428	6.102626	6.564985
AT4G03935	41.52848	1.075509218	0.328835	3.270665	0.001073	0.0064309	5.98984	5.703489	5.915727	5.19216	4.346824	4.474702
AT4G38840	406.7947	1.077565951	0.154374	6.980236	2.95E-12	7.58E-11	9.305268	8.975563	9.040728	8.03214	8.149577	7.89677
AT4G36090	84.85617	1.078022854	0.235815	4.571483	4.84E-06	5.10E-05	6.952657	6.840944	6.819344	5.492674	5.941644	5.838142
AT3G54500	2879.533	1.07906027	0.148969	7.243499	4.37E-13	1.22E-11	12.18954	11.86575	11.71993	11.02379	10.7303	10.75318
AT3G55710	53.18866	1.079705358	0.325776	3.314261	9.19E-04	0.0056123	5.771226	6.432368	6.411182	5.19216	4.50885	5.31183
AT5G17230	1365.625	1.080104024	0.097792	11.04493	2.32E-28	2.01E-26	10.86255	10.82478	10.8938	9.680454	9.759603	9.867357
AT3G16870	45.80071	1.080153023	0.302533	3.570364	3.56E-04	2.46E-03	6.027383	6.04978	5.944987	5.19216	4.654502	4.699401
AT1G29820	46.44414	1.080211228	0.324329	3.3306	0.000867	0.0053298	6.099659	6.03789	6.001784	4.414102	4.50885	5.399825
AT1G52550	46.16651	1.081691659	0.318492	3.396296	6.83E-04	4.35E-03	5.964257	5.762479	6.268024	4.626795	5.220243	4.767144
AT5G15850	806.1424	1.082812649	0.202536	5.346278	8.98E-08	1.27E-06	10.27516	10.01109	10.02744	9.448398	8.644055	8.746276
AT2G09805	231.7037	1.082865583	0.188657	5.739869	9.47E-09	1.54E-07	8.214317	8.128923	8.565508	7.245106	7.040014	7.295843
AT4G02630	80.10421	1.085414996	0.285668	3.799563	1.45E-04	1.10E-03	6.488642	7.120039	6.746431	5.319969	5.553109	5.987621
AT4G19020	117.3304	1.086118457	0.211979	5.123707	3.00E-07	3.91E-06	7.358247	7.468962	7.186967	6.172293	6.102626	6.360876

AT5G15845	816.3106	1.087406269	0.200925	5.412002	6.23E-08	9.01E-07	10.29261	10.03521	10.04444	9.45885	8.661394	8.767106
AT5G47910	268.3782	1.088656381	0.279029	3.901586	9.56E-05	7.61E-04	8.666982	8.652013	8.265559	8.022777	6.680573	7.09959
AT5G58760	51.5366	1.089512312	0.320018	3.404534	6.63E-04	4.25E-03	5.951294	6.025901	6.502575	4.524365	5.220243	5.218117
AT5G64940	2089.688	1.091684864	0.161795	6.747344	1.51E-11	3.56E-10	11.53703	11.55762	11.3403	10.70614	10.12404	10.20319
AT2G05100	24707.96	1.092414732	0.143449	7.615362	2.63E-14	8.24E-13	14.96426	14.88392	15.2542	13.83858	14.1618	13.77242
AT2G01590	116.5804	1.093923298	0.256056	4.272201	1.94E-05	1.81E-04	7.392218	7.032872	7.528819	6.530411	6.247434	5.740407
AT2G40610	889.313	1.094655127	0.204226	5.36002	8.32E-08	1.18E-06	9.94815	10.20631	10.54305	8.723251	9.456087	9.117863
AT5G37770	202.1528	1.095747131	0.216827	5.05355	4.34E-07	5.50E-06	8.326495	7.824292	8.154091	7.018926	7.283413	6.657142
AT1G17050	97.22746	1.096975457	0.234586	4.676215	2.92E-06	3.24E-05	6.872409	7.050731	7.281752	5.913254	5.883728	6.015745
AT3G62070	242.4678	1.097060251	0.183461	5.979815	2.23E-09	3.99E-08	8.579122	8.356575	8.172762	7.412182	7.283413	7.08639
AT4G03110	428.9722	1.09723894	0.143988	7.620351	2.53E-14	7.94E-13	9.205077	9.083609	9.293386	8.215903	8.149577	7.89677
AT1G74450	399.2455	1.09735576	0.189562	5.788903	7.08E-09	1.17E-07	9.123641	9.224022	8.928017	8.2723	8.021154	7.566881
AT1G08477	165.472	1.099450548	0.188056	5.846398	5.02E-09	8.47E-08	7.705689	7.990814	7.792967	6.817077	6.610887	6.692417
AT5G10750	143.3818	1.100520079	0.242922	4.530343	5.89E-06	6.10E-05	7.638536	7.264784	7.91258	6.607592	6.680573	6.17386
AT3G53830	52.07647	1.101621851	0.320376	3.43853	5.85E-04	3.81E-03	6.190717	6.414087	5.944987	5.492674	4.907958	4.553554
AT4G25050	1694.194	1.102276014	0.107954	10.21065	1.78E-24	1.25E-22	11.20563	11.03527	11.28094	10.06039	10.13339	10.0105
AT3G46550	43.45939	1.10428651	0.334884	3.297524	0.000975	0.0059145	5.91169	5.673064	6.208946	5.19216	4.50885	4.474702
AT4G24350	65.71167	1.104732066	0.285978	3.863001	1.12E-04	8.75E-04	6.575687	6.68196	6.244682	5.319969	5.760417	5.01018
AT2G41170	130.1746	1.107261672	0.205446	5.389556	7.06E-08	1.02E-06	7.416003	7.587895	7.444257	6.362435	6.610887	6.123056
AT1G76040	65.39156	1.108491112	0.280052	3.958158	7.55E-05	6.19E-04	6.286726	6.50326	6.754717	5.25748	5.123469	5.598855
AT4G21850	73.63256	1.109143476	0.290253	3.821299	1.33E-04	1.02E-03	6.681522	6.279193	7.01138	5.694846	5.553109	5.31183
AT3G01500	6692.217	1.109694941	0.162493	6.829182	8.54E-12	2.09E-10	12.85067	13.1516	13.43576	11.95802	12.23804	11.88538
AT4G08870	197.3525	1.110522886	0.264362	4.200768	2.66E-05	2.41E-04	7.690167	8.453741	8.050212	7.353641	6.841348	6.486772
AT2G00850	306.0634	1.111116023	0.197815	5.616953	1.94E-08	3.01E-07	8.757265	8.484728	8.879589	7.600176	7.894906	7.213246
AT5G57280	111.7288	1.111685771	0.21144	5.257698	1.46E-07	2.00E-06	7.147097	7.37285	7.298852	6.238498	6.102626	6.070398
AT1G79075	183.022	1.113773324	0.18107	6.151069	7.70E-10	1.46E-08	7.855842	7.993895	8.090048	6.656843	6.900955	6.961824
AT3G44720	367.796	1.114122926	0.193193	5.766876	8.08E-09	1.32E-07	9.021916	8.838346	9.095576	8.240343	7.510873	7.66781
AT1G52510	469.7143	1.114160823	0.148788	7.488262	6.98E-14	2.10E-12	9.215957	9.243565	9.52416	8.068994	8.312092	8.232543
AT1G14170	58.52354	1.114447649	0.316573	3.520348	4.31E-04	2.91E-03	6.633638	6.196041	6.232868	4.414102	5.476813	5.441888
AT3G02410	65.24926	1.11605685	0.341379	3.269261	1.08E-03	6.46E-03	5.91169	6.587239	6.940512	5.597298	4.654502	5.482759
AT1G74310	99.79813	1.116573655	0.27721	4.027894	5.63E-05	0.0004741	7.267032	7.40097	6.597764	5.872138	5.82339	6.123056
AT1G11670	33.00602	1.11866851	0.344855	3.243885	1.18E-03	6.98E-03	5.565434	5.384715	5.709543	4.414102	4.50885	4.208431
AT4G01883	69.66676	1.119658374	0.263961	4.241753	2.22E-05	2.05E-04	6.424426	6.658856	6.704273	5.492674	5.69457	5.168875
AT1G52830	51.1586	1.119851012	0.341461	3.27959	1.04E-03	6.25E-03	5.631937	6.269058	6.492702	4.812122	5.396255	4.699401

AT5G08855	871.6601	1.123360444	0.170678	6.581751	4.65E-11	1.04E-09	10.29966	10.35387	10.02829	8.986679	8.793078	9.391775
AT3G03820	216.5649	1.123704317	0.19303	5.821394	5.84E-09	9.76E-08	8.147693	8.236871	8.271346	6.817077	7.491614	6.887672
AT1G44446	2128.026	1.128260168	0.085563	13.18623	1.05E-39	1.70E-37	11.55374	11.50784	11.47929	10.43507	10.37324	10.33319
AT5G39785	47.1163	1.128914929	0.348731	3.237204	1.21E-03	7.13E-03	6.063973	6.206701	5.959397	5.545934	3.955229	4.553554
AT1G72470	102.3331	1.130234473	0.232496	4.861308	1.17E-06	1.38E-05	6.919761	7.191545	7.343483	6.030007	5.883728	6.04333
AT2G01180	118.9652	1.130977359	0.236797	4.776152	1.79E-06	2.04E-05	7.471535	7.423992	7.229204	6.632428	5.82339	6.015745
AT1G69530	1889.136	1.132400251	0.146237	7.743615	9.66E-15	3.16E-13	11.03566	11.48257	11.48147	10.15127	10.12404	10.30783
AT5G66580	737.1959	1.132499187	0.173072	6.543503	6.01E-11	1.32E-09	10.20249	9.81951	9.926535	9.106746	8.816485	8.53905
AT2G20721	40.50379	1.133598002	0.345482	3.281203	1.03E-03	6.23E-03	5.58235	5.860242	6.042961	4.414102	5.220243	4.208431
AT5G33290	43.64132	1.134500075	0.314126	3.611605	3.04E-04	2.14E-03	5.925012	5.976925	6.001784	5.123743	4.164273	4.767144
AT1G29500	324.8121	1.13666017	0.179751	6.32352	2.56E-10	5.19E-09	9.052587	8.628421	8.715245	7.522586	7.880172	7.557346
AT4G04335	143.234	1.136786116	0.198933	5.714407	1.10E-08	1.76E-07	7.476068	7.639949	7.784898	6.362435	6.537663	6.526408
AT3G44630	544.0434	1.137014379	0.179725	6.326396	2.51E-10	5.11E-09	9.510311	9.590424	9.571833	8.790998	8.137237	8.152475
AT4G24780	347.5886	1.137302131	0.145918	7.794134	6.49E-15	2.16E-13	8.766546	8.991062	8.96598	7.720882	7.690514	7.842845
AT5G53905	8163.678	1.137481633	0.357231	3.184162	1.45E-03	8.35E-03	13.79037	13.18112	13.44387	12.02638	11.61443	12.78993
AT4G26850	607.9248	1.137793098	0.143523	7.927601	2.23E-15	7.77E-14	9.869368	9.563722	9.699646	8.711644	8.365965	8.57257
AT3G59780	1268.214	1.139394908	0.114034	9.991741	1.66E-23	1.10E-21	10.622	10.85981	10.82366	9.536562	9.697925	9.635546
AT5G18080	272.7661	1.140027524	0.164664	6.923368	4.41E-12	1.12E-10	8.663012	8.455977	8.546545	7.212535	7.656331	7.341001
AT1G22400	280.9146	1.140605288	0.185427	6.151236	7.69E-10	1.46E-08	8.418853	8.661731	8.721599	7.743857	7.327526	7.201051
AT3G05490	247.3123	1.140681703	0.213082	5.353259	8.64E-08	1.22E-06	8.485562	8.033353	8.678698	7.308122	7.411902	7.032351
AT3G25740	67.72531	1.14143096	0.268187	4.256103	2.08E-05	1.93E-04	6.575687	6.414087	6.746431	5.597298	5.019734	5.441888
AT1G27480	72.57606	1.142953735	0.257645	4.436164	9.16E-06	9.14E-05	6.617317	6.579058	6.803457	5.694846	5.553109	5.218117
AT2G32540	169.3561	1.146491179	0.188195	6.092044	1.11E-09	2.07E-08	7.838346	8.024343	7.780847	6.880651	6.499609	6.709736
AT2G06950	558.6193	1.148478883	0.159062	7.220334	5.19E-13	1.44E-11	9.536586	9.600563	9.657183	8.740488	8.137237	8.330959
AT1G04250	145.6369	1.148487875	0.231495	4.961172	7.01E-07	8.61E-06	7.392218	7.78926	7.792967	6.030007	6.680573	6.657142
AT2G37690	103.118	1.148880254	0.239213	4.802741	1.57E-06	1.81E-05	6.90639	7.180772	7.397399	6.102904	5.997325	5.869303
AT3G63140	1438.88	1.149239549	0.165364	6.94976	3.66E-12	9.35E-11	10.71361	10.86781	11.24831	9.588255	9.965564	9.796177
AT1G02350	97.2468	1.150750502	0.252881	4.550553	5.35E-06	5.58E-05	7.059205	7.002605	7.174668	5.694846	6.420349	5.522505
AT4G23010	186.1384	1.151048447	0.173047	6.651651	2.90E-11	6.67E-10	8.029322	7.965931	8.070268	6.773083	6.714193	7.004552
AT1G03935	35.57387	1.153905966	0.343316	3.36106	7.76E-04	4.86E-03	5.771226	5.762479	5.547306	4.626795	3.710678	4.699401
AT1G61667	116.6932	1.153972262	0.229619	5.025596	5.02E-07	6.31E-06	7.298082	7.114389	7.60412	6.102904	6.247434	6.148682
AT3G05655	84.06512	1.155404559	0.315969	3.656707	2.55E-04	1.84E-03	6.892893	7.056636	6.762956	4.812122	5.476813	6.270397
AT1G03940	143.363	1.157891757	0.210193	5.508714	3.61E-08	5.41E-07	7.502969	7.86187	7.571658	6.530411	6.200761	6.583893
AT5G07015	68.90899	1.159164007	0.255917	4.529446	5.91E-06	6.13E-05	6.657778	6.511881	6.652001	5.597298	5.220243	5.356498

AT4G23250	105.3804	1.159658115	0.230659	5.027587	4.97E-07	6.25E-06	7.197372	7.319828	7.137131	6.238498	5.476813	6.148682
AT2G41250	492.1124	1.160467218	0.248271	4.6742	2.95E-06	3.27E-05	9.604401	9.417677	9.244213	8.801986	7.707307	7.873905
AT5G18020	303.7143	1.162930288	0.151334	7.68453	1.54E-14	4.91E-13	8.657037	8.703726	8.789711	7.495767	7.756543	7.395531
AT4G27030	96.57261	1.163014782	0.230211	5.05196	4.37E-07	5.54E-06	6.9264	7.062516	7.241048	5.913254	5.997325	5.773726
AT5G20480	63.17741	1.163376281	0.305029	3.813983	1.37E-04	0.0010451	6.168486	6.734471	6.531797	5.694846	4.786789	5.117892
AT3G03773	48.2334	1.164026043	0.298214	3.903328	9.49E-05	0.0007564	6.122968	5.989326	6.208946	4.626795	4.907958	5.065041
AT5G44510	171.6036	1.166418135	0.205823	5.667081	1.45E-08	2.29E-07	7.893603	8.060052	7.784898	6.607592	6.379024	7.018519
AT1G04360	27.31726	1.167998249	0.370165	3.155347	1.60E-03	9.08E-03	5.441126	5.226103	5.320509	4.021447	3.710678	4.302755
AT1G79520	2223.168	1.168844248	0.177072	6.600947	4.09E-11	9.21E-10	11.45215	11.70869	11.62001	10.1641	10.14884	10.78621
AT1G01120	439.9338	1.169177754	0.177179	6.598852	4.14E-11	9.32E-10	9.210527	9.012485	9.514434	7.842944	8.185975	8.152475
AT1G34750	115.7753	1.170678018	0.27109	4.318403	1.57E-05	0.0001501	7.563853	7.239052	7.241048	6.680852	5.69457	5.773726
AT3G08770	113.2527	1.171664192	0.247921	4.725962	2.29E-06	2.58E-05	6.933009	7.512572	7.433975	6.172293	6.247434	5.899804
AT4G23015	49.21032	1.172954907	0.292194	4.014302	5.96E-05	0.0004999	6.051879	6.174482	6.196834	5.051918	4.654502	4.953151
AT5G64780	54.24353	1.174489587	0.310955	3.777044	0.000159	0.0011934	5.977105	6.217283	6.579222	5.319969	4.907958	4.831847
AT5G20410	44.34577	1.175450947	0.339508	3.46222	5.36E-04	3.54E-03	5.679876	6.04978	6.172301	4.524365	5.310931	4.302755
AT3G59068	918.7052	1.176576314	0.180894	6.504225	7.81E-11	1.70E-09	10.37076	10.00194	10.54723	9.354494	9.128451	8.8397
AT1G76520	331.0705	1.178626323	0.13935	8.45801	2.72E-17	1.10E-15	8.801276	8.855383	8.894664	7.777648	7.621318	7.576354
AT3G25600	84.65997	1.179022062	0.28272	4.170284	3.04E-05	0.0002725	7.141402	6.68196	6.858314	6.102904	5.625572	5.168875
AT1G29510	248.9326	1.179710402	0.156254	7.549967	4.35E-14	1.34E-12	8.545013	8.37323	8.40369	7.323456	7.214606	7.201051
AT5G38420	11346.08	1.181589278	0.187109	6.314977	2.70E-10	5.48E-09	13.72891	13.798	14.26828	12.63849	13.06342	12.44942
AT1G48480	191.0044	1.182042647	0.179582	6.582171	4.64E-11	1.04E-09	8.04466	8.162164	8.002302	6.980752	6.537663	6.976208
AT3G10060	296.1965	1.183431985	0.18011	6.570602	5.01E-11	1.12E-09	8.416497	8.757278	8.868178	7.482168	7.638931	7.352073
AT5G62740	189.0153	1.183890937	0.170329	6.950607	3.64E-12	9.30E-11	7.966283	8.04528	8.131998	6.795248	6.871459	6.872373
AT1G09797	917.1752	1.185779021	0.162117	7.314322	2.59E-13	7.38E-12	10.15663	10.25141	10.52983	9.221681	9.282066	8.815906
AT2G32340	33.21354	1.186997507	0.369366	3.213607	1.31E-03	0.0076655	5.364967	5.776858	5.692391	4.29471	3.416038	4.767144
AT4G20030	70.85688	1.188461005	0.293213	4.053237	5.05E-05	0.0004304	6.77277	6.595375	6.660846	5.646896	4.346824	5.740407
AT3G01505	139.1336	1.189512135	0.22907	5.192787	2.07E-07	2.77E-06	7.318417	7.643877	7.848216	6.138016	6.420349	6.564985
AT5G07325	2903.441	1.189846398	0.14205	8.376239	5.46E-17	2.17E-15	11.85544	11.81418	12.24489	10.64185	10.81602	10.86562
AT2G40460	64.85851	1.192416604	0.285049	4.183203	2.87E-05	2.59E-04	6.234178	6.7122	6.634148	5.123743	5.123469	5.522505
AT5G06835	89.45954	1.192483992	0.316764	3.764586	1.67E-04	1.25E-03	6.952657	7.103022	6.990483	5.694846	4.50885	6.316346
AT1G62180	296.1027	1.192499301	0.161901	7.365585	1.76E-13	5.12E-12	8.577014	8.877233	8.625474	7.495767	7.370331	7.566881
AT3G59070	907.3393	1.193417078	0.181711	6.567669	5.11E-11	1.14E-09	10.35485	9.989644	10.53706	9.324326	9.103296	8.80386
AT2G44230	231.4458	1.193700125	0.165477	7.213701	5.45E-13	1.51E-11	8.278	8.260085	8.473124	7.056115	7.26084	7.08639
AT2G41100	3195.095	1.195446734	0.238452	5.013361	5.35E-07	6.70E-06	12.2694	11.91492	12.19667	11.41321	10.78274	10.22292

AT2G08915	65.83908	1.195592981	0.308513	3.875344	1.06E-04	8.36E-04	6.558693	6.834086	6.220956	5.741253	5.123469	4.893774
AT3G08405	108.3596	1.196781197	0.263738	4.537758	5.69E-06	5.91E-05	7.60995	7.169918	6.940512	6.301797	5.82339	5.869303
AT3G26310	68.04665	1.197760236	0.256512	4.669418	3.02E-06	3.33E-05	6.497586	6.666599	6.643102	5.379864	5.220243	5.441888
AT3G56360	511.4366	1.198470028	0.243367	4.924536	8.46E-07	1.02E-05	9.748121	9.752886	8.795746	8.295811	7.994038	8.401935
AT5G11975	115.4692	1.198751742	0.217729	5.505703	3.68E-08	5.50E-07	7.387414	7.169918	7.484669	6.270495	5.997325	6.070398
AT5G55260	61.47578	1.198792625	0.286883	4.178687	2.93E-05	0.0002638	6.58411	6.357813	6.442294	5.646896	4.786789	5.01018
AT5G54075	9378.948	1.201912318	0.359571	3.342628	8.30E-04	0.0051509	14.01017	13.38251	13.68405	12.17271	11.75735	12.94033
AT3G02870	128.9833	1.202640696	0.22162	5.426597	5.74E-08	8.35E-07	7.261791	7.616158	7.640345	6.102904	6.200761	6.466537
AT1G66500	115.303	1.205584456	0.361246	3.337292	8.46E-04	0.0052269	7.213749	7.542334	7.376074	6.901235	4.786789	5.635566
AT5G01195	97.5767	1.206214807	0.283983	4.247484	2.16E-05	0.0002	7.152771	7.249399	6.954967	6.448866	5.396255	5.441888
AT1G08470	80.06816	1.207922202	0.242794	4.975097	6.52E-07	8.05E-06	6.735449	6.940097	6.803457	5.646896	5.69457	5.441888
AT4G39800	1758.387	1.2087889	0.138348	8.737298	2.39E-18	1.06E-16	10.99478	11.3337	11.43711	9.95358	10.05356	10.11331
AT3G10570	39.6646	1.209494249	0.337057	3.588397	0.000333	0.0023132	5.843167	5.951799	5.692391	4.29471	5.123469	4.208431
AT4G37930	6666.589	1.209520991	0.14616	8.275345	1.28E-16	4.93E-15	12.91603	13.22168	13.39252	11.78191	12.11837	11.96687
AT1G57770	116.329	1.209795812	0.261139	4.632771	3.61E-06	3.91E-05	6.919761	7.521138	7.58566	6.362435	5.997325	5.929675
AT1G26560	96.01306	1.211837825	0.331662	3.653836	2.58E-04	1.85E-03	6.524093	7.410223	7.281752	6.066916	4.786789	6.123056
AT4G28900	124.194	1.212300844	0.204327	5.933141	2.97E-09	5.20E-08	7.420713	7.37285	7.571658	6.332435	6.200761	6.123056
AT5G53902	10503.35	1.215811053	0.353282	3.441473	5.79E-04	3.78E-03	14.16588	13.54228	13.86787	12.32997	11.93627	13.08651
AT1G01600	33.16846	1.216642063	0.35351	3.441605	5.78E-04	3.78E-03	5.58235	5.819152	5.385981	4.021447	4.346824	4.474702
AT2G37250	947.818	1.21669647	0.1272	9.565212	1.12E-21	6.39E-20	10.3206	10.44672	10.3698	9.301278	8.891872	9.202881
AT5G43620	86.98849	1.221899135	0.320962	3.806988	0.000141	0.0010721	7.003778	7.062516	6.842852	6.391825	5.019734	5.117892
AT2G07774	98.56316	1.222941942	0.238457	5.128569	2.92E-07	3.81E-06	7.164051	6.965426	7.235138	5.99213	6.102626	5.522505
AT5G09995	73.03668	1.222951987	0.257033	4.757952	1.96E-06	2.22E-05	6.727867	6.545861	6.842852	5.25748	5.476813	5.561185
AT1G74330	140.818	1.223950527	0.194088	6.306147	2.86E-10	5.77E-09	7.618176	7.697767	7.617811	6.582321	6.152528	6.382637
AT3G03850	94.56001	1.226170074	0.263755	4.648904	3.34E-06	3.65E-05	7.328478	6.868059	6.969278	5.741253	6.200761	5.441888
AT2G21140	163.073	1.227662333	0.191539	6.409472	1.46E-10	3.06E-09	7.88681	7.756987	7.886424	6.632428	6.841348	6.338783
AT4G28085	26.59822	1.228440364	0.390983	3.141928	1.68E-03	9.45E-03	5.384387	5.047874	5.407161	3.862584	4.50885	3.458449
AT1G10550	130.6008	1.229780723	0.269061	4.570641	4.86E-06	5.12E-05	7.298082	7.259674	7.959931	5.872138	6.499609	6.338783
AT5G18030	421.5051	1.233793492	0.140007	8.812368	1.23E-18	5.61E-17	9.279569	9.066154	9.287687	8.03214	7.994038	7.881567
AT1G26770	140.8364	1.234085392	0.2304	5.356267	8.50E-08	1.20E-06	7.614069	7.731377	7.60412	6.838581	5.997325	6.123056
AT5G25130	98.33321	1.236181014	0.235111	5.25785	1.46E-07	2.00E-06	6.946137	7.23385	7.211253	6.102904	5.69457	5.740407
AT2G20724	142.7957	1.237670294	0.230373	5.372463	7.77E-08	1.11E-06	7.662596	7.391657	7.89019	6.391825	6.680573	6.096967
AT2G46830	686.0554	1.240066575	0.257688	4.812285	1.49E-06	1.73E-05	10.29902	9.78525	9.631541	9.171726	8.073902	8.412551
AT3G01060	174.0399	1.241380006	0.177623	6.988833	2.77E-12	7.14E-11	8.047708	7.871951	7.927316	6.704469	6.610887	6.72685

AT2G17040	137.2485	1.246891633	0.318663	3.912879	9.12E-05	0.0007321	7.809903	7.600076	7.474672	7.03764	5.396255	5.899804
AT4G12390	120.7421	1.248561339	0.241354	5.173156	2.30E-07	3.06E-06	7.448656	7.21813	7.58566	6.332435	6.379024	5.671366
AT3G01550	46.80547	1.248644641	0.318496	3.920446	8.84E-05	0.0007144	6.027383	6.04978	6.196834	5.25748	4.50885	4.391288
AT2G42380	227.5782	1.250232252	0.159043	7.86099	3.81E-15	1.30E-13	8.375841	8.252389	8.369029	7.09237	7.11744	7.004552
AT5G27660	254.3999	1.251347273	0.22487	5.564746	2.63E-08	4.01E-07	8.233184	8.489101	8.742581	6.680852	7.391266	7.437718
AT4G00880	46.31113	1.251410992	0.308559	4.05566	5.00E-05	0.0004265	5.98984	6.25885	5.959397	4.524365	4.907958	4.831847
AT4G27940	99.04847	1.25168272	0.237139	5.278263	1.30E-07	1.81E-06	7.095007	7.136858	7.223245	6.238498	5.553109	5.671366
AT3G02045	197.9698	1.251728062	0.286388	4.370742	1.24E-05	1.21E-04	8.095624	8.120492	8.248058	5.872138	6.810594	7.384788
AT5G59920	154.2378	1.252732725	0.200352	6.252652	4.04E-10	7.90E-09	7.701824	7.767825	7.852083	6.704469	6.610887	6.17386
AT1G09793	790.132	1.253453354	0.170534	7.350188	1.98E-13	5.71E-12	9.96195	10.06187	10.33293	8.991487	9.018374	8.504734
AT3G51450	31.38197	1.254652368	0.394225	3.18258	0.00146	0.0083914	5.345282	5.762479	5.56626	4.722432	2.545235	4.302755
AT3G04175	32.07119	1.256160323	0.354014	3.548339	3.88E-04	2.65E-03	5.58235	5.657607	5.508634	4.164539	3.710678	4.553554
AT2G33570	176.4541	1.258541491	0.260594	4.829511	1.37E-06	1.60E-05	8.029322	7.596027	8.236271	6.205775	7.166841	6.506726
AT1G44000	318.2273	1.26190062	0.165238	7.636887	2.23E-14	7.03E-13	8.773927	8.789545	8.896537	7.821505	7.432247	7.318598
AT2G37970	120.1255	1.263743669	0.257878	4.900539	9.56E-07	1.14E-05	7.516233	7.567364	7.193077	6.607592	5.760417	5.806294
AT2G29120	76.79375	1.265415528	0.390993	3.236413	1.21E-03	7.15E-03	7.040965	6.627465	6.787392	6.332435	4.164273	4.767144
AT3G45860	182.3926	1.265675246	0.328717	3.850351	1.18E-04	0.000919	8.24651	7.796335	8.063613	7.440585	6.292644	5.773726
AT4G38850	77.47711	1.265701201	0.25609	4.942405	7.72E-07	9.41E-06	6.633638	6.888065	6.858314	5.25748	5.82339	5.399825
AT1G55480	564.3726	1.268758593	0.146161	8.680543	3.94E-18	1.72E-16	9.436647	9.701995	9.774901	8.341714	8.497289	8.250405
AT2G47750	46.41091	1.27018304	0.348161	3.648268	2.64E-04	0.0018855	6.027383	6.376816	5.839872	5.319969	4.164273	4.391288
AT1G61795	30.84525	1.272759544	0.354662	3.588655	3.32E-04	2.31E-03	5.477749	5.54451	5.56626	4.29471	3.710678	4.302755
AT4G03320	46.15375	1.273121251	0.369858	3.442193	5.77E-04	0.0037699	6.087861	6.152596	6.01564	5.379864	4.654502	3.613718
AT1G72600	5559.231	1.276569071	0.20028	6.373922	1.84E-10	3.81E-09	13.1106	12.59285	13.0982	11.80326	11.86229	11.15041
AT2G21910	126.2139	1.278147267	0.225863	5.658938	1.52E-08	2.40E-07	7.593357	7.249399	7.631374	6.391825	6.200761	5.958939
AT5G16000	338.4049	1.279265452	0.163769	7.811412	5.66E-15	1.90E-13	8.897341	8.932865	8.918829	7.925648	7.391266	7.458357
AT1G72610	5602.732	1.279369504	0.20121	6.35839	2.04E-10	4.19E-09	13.12347	12.60235	13.11157	11.80804	11.8772	11.15595
AT4G00490	123.7446	1.280779618	0.225574	5.677866	1.36E-08	2.16E-07	7.666567	7.490932	7.264448	6.138016	5.883728	6.360876
AT5G58260	182.688	1.283726697	0.214797	5.976469	2.28E-09	4.07E-08	7.841862	7.895203	8.308404	6.530411	6.985988	6.620982
AT1G08643	79.48135	1.284063783	0.245852	5.222913	1.76E-07	2.39E-06	6.742991	6.959136	6.811422	5.492674	5.69457	5.399825
AT5G22690	225.5072	1.284367626	0.24821	5.174514	2.29E-07	3.04E-06	8.222433	8.582096	8.194244	7.535811	6.610887	6.657142
AT4G17260	49.45282	1.285005789	0.314631	4.084162	4.42E-05	3.81E-04	6.122968	5.951799	6.431998	5.123743	4.654502	4.62832
AT5G04140	6453.836	1.286661651	0.181771	7.078477	1.46E-12	3.89E-11	12.6568	13.41189	13.32857	11.95187	11.7922	11.80456
AT1G04877	27.44559	1.287132454	0.403627	3.188918	1.43E-03	8.25E-03	5.726274	5.023964	5.320509	3.480233	3.710678	4.391288
AT1G50320	477.204	1.28869838	0.172977	7.450129	9.32E-14	2.80E-12	9.220016	9.308098	9.673663	7.884889	8.161812	8.226539

AT3G51238	26.40717	1.289813926	0.386441	3.33767	8.45E-04	0.005221	5.037115	5.307588	5.547306	4.021447	3.955229	3.753887
AT5G00660	96.8793	1.290362726	0.315099	4.095099	4.22E-05	0.0003658	6.933009	7.521138	6.8812	6.332435	5.553109	5.168875
AT5G13400	141.7313	1.294814084	0.202585	6.391465	1.64E-10	3.42E-09	7.60995	7.612154	7.796984	6.582321	6.102626	6.293554
AT5G61910	582.818	1.295668894	0.16575	7.816985	5.41E-15	1.82E-13	9.569832	9.494723	9.993707	8.415115	8.376502	8.358672
AT3G61198	1196.794	1.2983903	0.155853	8.330891	8.02E-17	3.12E-15	10.92935	10.55184	10.72062	9.358221	9.189485	9.658025
AT4G17670	27.28495	1.299421616	0.386017	3.366226	7.62E-04	4.78E-03	5.242615	5.117315	5.675034	3.862584	3.955229	3.998987
AT4G23260	179.5264	1.299449971	0.296643	4.380519	1.18E-05	1.16E-04	7.88681	8.039329	8.154091	7.353641	5.883728	6.246861
AT4G19865	31.66424	1.299541993	0.408246	3.183235	0.001456	0.0083778	5.785904	5.610218	5.298008	4.896561	3.710678	3.284433
AT4G04020	528.4118	1.299978785	0.196707	6.608719	3.88E-11	8.76E-10	9.2281	9.536517	9.863764	7.925648	8.386963	8.342109
AT5G48570	48.4694	1.300908257	0.342134	3.802338	0.000143	0.0010897	6.051879	6.562555	5.776179	4.414102	4.786789	5.01018
AT5G64660	64.19171	1.301125468	0.272179	4.780398	1.75E-06	2.00E-05	6.524093	6.635377	6.522122	5.379864	4.654502	5.356498
AT4G37800	526.1008	1.301321154	0.29562	4.401999	1.07E-05	1.06E-04	9.22541	9.565794	9.861847	7.162256	8.736939	8.256311
AT4G24160	241.9705	1.301955411	0.176249	7.387014	1.50E-13	4.41E-12	8.241194	8.49346	8.558426	7.212535	7.190921	6.947296
AT3G57040	214.4642	1.302041705	0.176159	7.391273	1.45E-13	4.29E-12	8.235859	8.337301	8.203353	7.018926	7.142352	6.674887
AT3G17890	208.3745	1.303195492	0.217118	6.002243	1.95E-09	3.52E-08	8.214317	8.229049	8.265559	6.420628	6.747048	7.307265
AT5G61010	140.9146	1.303717029	0.230915	5.645886	1.64E-08	2.57E-07	7.551021	7.686387	7.772711	6.773083	5.941644	6.096967
AT5G52810	30.42066	1.304844596	0.36268	3.597784	3.21E-04	0.0022428	5.441126	5.561219	5.56626	4.414102	3.416038	4.208431
AT1G24530	146.1964	1.30567008	0.328184	3.978466	6.94E-05	0.0005738	7.471535	7.008709	8.387796	6.066916	6.610887	6.270397
AT4G38960	40.43527	1.306263539	0.344201	3.795063	1.48E-04	1.12E-03	6.15724	5.873683	5.709543	4.626795	3.710678	4.831847
AT5G16170	29.40941	1.30855665	0.380428	3.439697	5.82E-04	3.80E-03	5.345282	5.384715	5.657465	3.242825	4.346824	4.302755
AT3G05715	993.2029	1.309490719	0.165369	7.918579	2.40E-15	8.31E-14	10.59829	10.23198	10.56677	9.398595	9.103296	8.878505
AT3G59010	47.06753	1.309547069	0.355574	3.682915	2.31E-04	1.68E-03	6.039683	6.367346	5.987793	5.319969	3.416038	4.699401
AT1G09185	44.54953	1.311844255	0.331711	3.954786	7.66E-05	0.0006277	6.297009	5.819152	5.944987	4.812122	4.907958	4.208431
AT4G13493	67.92143	1.314824634	0.301056	4.367372	1.26E-05	1.23E-04	6.787432	6.799293	6.313604	5.597298	4.50885	5.356498
AT2G34920	32.25882	1.316254525	0.385454	3.414815	6.38E-04	0.0041094	5.132078	5.626188	5.930431	3.684038	4.50885	4.208431
AT5G08760	116.1763	1.316315824	0.253406	5.194493	2.05E-07	2.75E-06	7.713388	7.026869	7.386775	6.138016	5.883728	6.04333
AT3G51240	26.79947	1.316586185	0.385067	3.419111	0.000628	0.0040562	5.085378	5.307588	5.584968	4.021447	3.955229	3.753887
AT5G22390	77.61111	1.31685893	0.270991	4.859426	1.18E-06	1.39E-05	6.865515	6.666599	6.911161	5.786214	5.553109	4.953151
AT5G35970	719.6972	1.317223945	0.140593	9.369044	7.32E-21	3.93E-19	9.823864	10.17272	10.01024	8.664258	8.808725	8.563072
AT5G36700	701.8896	1.317419772	0.139095	9.471371	2.76E-21	1.53E-19	9.773199	9.977246	10.1495	8.676251	8.652751	8.596044
AT5G36790	703.3015	1.318541406	0.139232	9.470133	2.79E-21	1.54E-19	9.77504	9.982683	10.15186	8.682211	8.652751	8.596044
AT5G21430	293.3122	1.320342618	0.169292	7.799204	6.23E-15	2.08E-13	8.591707	8.669457	8.873895	7.308122	7.621318	7.213246
AT3G55090	25.21536	1.321836741	0.388281	3.404328	6.63E-04	0.0042484	5.263741	5.094538	5.428035	4.021447	3.416038	3.881636
AT4G26120	38.67977	1.323714061	0.389502	3.398474	6.78E-04	4.32E-03	5.800434	5.747956	6.056429	5.19216	3.045364	4.107507

AT4G07145	55.62845	1.325023108	0.327502	4.045853	5.21E-05	0.0004418	6.68935	6.084872	6.220956	5.25748	5.123469	4.391288
AT1G75100	718.2582	1.325874849	0.133019	9.967593	2.11E-23	1.39E-21	10.08277	9.965525	9.977869	8.881834	8.60874	8.494778
AT5G54610	50.43364	1.326445628	0.373946	3.54716	0.000389	0.0026627	6.405541	5.976925	6.335866	5.437371	3.045364	4.831847
AT5G67340	90.73986	1.32796238	0.399908	3.320671	0.000898	0.0054994	6.892893	7.451141	6.835058	6.530411	4.50885	4.767144
AT5G05860	97.1034	1.328155835	0.226637	5.860267	4.62E-09	7.83E-08	7.261791	7.056636	7.111553	5.741253	5.69457	5.869303
AT5G38430	5424.815	1.329982736	0.181531	7.326494	2.36E-13	6.77E-12	12.75542	12.74971	13.23339	11.52602	11.86135	11.26028
AT4G06255	178.383	1.330947499	0.316926	4.199547	2.67E-05	2.43E-04	8.272799	7.72026	8.076891	6.270495	5.69457	7.295843
AT5G63060	25.43674	1.332030306	0.395525	3.367755	0.000758	0.0047635	4.961552	5.32726	5.468897	3.684038	4.164273	3.613718
AT5G06000	29.73927	1.332413911	0.366638	3.634137	2.79E-04	1.98E-03	5.441126	5.43997	5.621672	4.164539	3.416038	4.302755
AT3G44400	135.4138	1.332414737	0.280199	4.755251	1.98E-06	2.25E-05	7.453261	7.643877	7.788938	6.817077	5.396255	6.070398
AT1G24145	35.01119	1.332782557	0.388101	3.434115	0.000594	0.0038664	5.938213	5.475665	5.792368	4.524365	2.545235	4.699401
AT4G06305	180.8649	1.333155617	0.307013	4.34234	1.41E-05	0.0001361	8.293491	7.749716	8.083485	6.270495	5.82339	7.295843
AT5G24030	177.5654	1.334005135	0.224476	5.942749	2.80E-09	4.92E-08	7.927094	7.806883	8.24512	6.859769	6.779171	6.222936
AT3G23410	94.75566	1.334046793	0.246342	5.415427	6.11E-08	8.86E-07	7.348392	7.008709	6.947758	5.913254	5.625572	5.635566
AT5G21100	153.6482	1.335457014	0.202217	6.604081	4.00E-11	9.02E-10	7.869688	7.911584	7.599527	6.607592	6.336481	6.338783
AT3G03770	79.34145	1.335648094	0.257041	5.196246	2.03E-07	2.73E-06	6.697136	7.026869	6.858314	5.319969	5.310931	5.706299
AT5G05990	55.92292	1.335648971	0.282615	4.726032	2.29E-06	2.58E-05	6.443067	6.24857	6.40066	4.812122	5.019734	5.065041
AT5G03355	66.78502	1.336656025	0.318044	4.202743	2.64E-05	0.0002394	6.67365	6.163581	6.947758	5.597298	4.786789	5.065041
AT1G72890	91.71258	1.33774047	0.278359	4.80581	1.54E-06	1.78E-05	6.816318	7.175355	7.193077	6.172293	5.396255	5.265734
AT3G42806	644.6375	1.340378381	0.220378	6.082169	1.19E-09	2.20E-08	9.925958	9.874028	9.801139	9.010559	8.149577	8.081073
AT1G48610	84.55367	1.342202322	0.242667	5.531036	3.18E-08	4.80E-07	6.851629	7.091564	6.888749	5.646896	5.625572	5.441888
AT3G59400	897.4382	1.342499001	0.150263	8.93431	4.10E-19	1.97E-17	10.32563	10.17408	10.48758	8.942675	9.213193	8.750466
AT5G02230	66.326	1.343714816	0.293871	4.572458	4.82E-06	5.08E-05	6.317359	6.53744	6.925911	4.97633	5.220243	5.356498
AT4G07135	56.1189	1.343805984	0.324607	4.139788	3.48E-05	0.0003063	6.68935	6.107802	6.2564	5.25748	5.123469	4.391288
AT1G10370	49.55115	1.349412702	0.317603	4.248734	2.15E-05	1.99E-04	5.91169	6.196041	6.462669	4.524365	4.786789	4.953151
AT2G34620	353.3033	1.350139406	0.142014	9.507072	1.96E-21	1.09E-19	9.035798	8.948827	8.990744	7.482168	7.819681	7.595115
AT1G16820	2304.508	1.350973927	0.201715	6.697441	2.12E-11	4.94E-10	11.65316	11.74502	11.71781	9.827509	10.17624	10.76979
AT1G23710	389.2913	1.352576236	0.182143	7.425895	1.12E-13	3.32E-12	9.171935	9.255165	8.978415	8.104931	7.585434	7.5087
AT5G25140	107.035	1.353829427	0.239345	5.656386	1.55E-08	2.43E-07	7.457851	7.21813	7.162264	6.138016	5.997325	5.522505
AT1G29660	1323.133	1.354931035	0.120119	11.27988	1.65E-29	1.51E-27	10.91148	10.81958	10.96515	9.394971	9.451111	9.712737
AT1G06553	102.1718	1.357126127	0.330388	4.107669	4.00E-05	0.000348	7.022492	7.091564	7.58566	5.437371	4.907958	6.446015
AT1G07120	22.64874	1.357493413	0.427437	3.175891	1.49E-03	8.55E-03	4.797403	5.023964	5.528099	3.480233	3.045364	3.998987
AT1G26730	48.74526	1.358956995	0.319951	4.247395	2.16E-05	2.00E-04	6.063973	5.951799	6.492702	4.626795	4.654502	4.893774
AT1G74360	115.9257	1.36031022	0.268749	5.061646	4.16E-07	5.29E-06	7.33847	7.563222	7.343483	6.530411	5.220243	5.869303

AT2G30010	179.2208	1.362083711	0.232116	5.868127	4.41E-09	7.48E-08	8.098567	7.888597	8.070268	6.332435	7.166841	6.270397
AT1G04223	52.50628	1.362809499	0.295161	4.617181	3.89E-06	4.19E-05	6.276368	6.24857	6.368627	4.896561	4.346824	5.117892
AT1G79470	37.49078	1.362912197	0.337769	4.035046	5.46E-05	0.0004608	5.695509	5.762479	6.001784	4.524365	3.955229	4.474702
AT2G21320	51.64778	1.362963761	0.295602	4.610803	4.01E-06	4.30E-05	6.179644	6.348216	6.268024	5.123743	4.50885	4.767144
AT4G08735	43.75899	1.366372887	0.332308	4.111766	3.93E-05	0.0003426	5.870968	6.24857	5.973665	4.021447	4.654502	4.893774
AT5G58670	239.6578	1.366978867	0.211816	6.453618	1.09E-10	2.33E-09	8.251806	8.435731	8.645664	7.397768	6.574739	7.004552
AT5G38410	16830.17	1.369386841	0.183173	7.475918	7.67E-14	2.31E-12	14.29097	14.44234	14.91875	13.10789	13.43727	12.92651
AT2G41640	572.9265	1.371082944	0.16171	8.47865	2.28E-17	9.35E-16	9.559191	9.757424	9.775919	8.609021	8.073902	8.165086
AT4G25420	27.86979	1.37216604	0.379465	3.616053	0.000299	0.0021059	5.199411	5.475665	5.56626	4.164539	3.416038	3.998987
AT4G37900	32.66599	1.373457427	0.434397	3.161753	1.57E-03	0.0089094	5.284563	5.951799	5.657465	4.97633	3.416038	3.08651
AT3G27690	8712.084	1.374198469	0.181024	7.591263	3.17E-14	9.83E-13	13.51583	13.48007	13.85529	12.02521	12.59459	11.9533
AT5G14545	87.73397	1.374633835	0.301459	4.559941	5.12E-06	5.36E-05	7.303193	6.666599	7.025145	6.066916	5.396255	5.065041
AT5G00765	66.64033	1.375769875	0.29982	4.588649	4.46E-06	4.73E-05	6.30722	6.763641	6.803457	4.97633	4.786789	5.561185
AT1G10657	20.12327	1.384675313	0.423895	3.266554	1.09E-03	6.51E-03	5.132078	4.949759	4.849451	2.958503	3.416038	3.753887
AT4G28780	183.2175	1.386757557	0.188346	7.362822	1.80E-13	5.22E-12	7.950083	8.048247	8.194244	6.420628	6.680573	6.809501
AT5G44050	26.34445	1.387219065	0.432171	3.209888	1.33E-03	7.75E-03	4.961552	5.139739	5.855366	4.164539	2.545235	3.998987
AT2G31380	202.5332	1.391516007	0.202374	6.875974	6.16E-12	1.53E-10	8.334006	8.181207	8.135175	7.09237	6.247434	6.841279
AT1G14345	176.092	1.39154108	0.201559	6.903902	5.06E-12	1.27E-10	7.963058	7.834638	8.197287	6.632428	6.779171	6.360876
AT5G44680	1111.137	1.392425772	0.118822	11.7186	1.02E-31	1.09E-29	10.50212	10.66631	10.78676	9.273916	9.28766	9.196752
AT2G21595	26.20701	1.393035282	0.42725	3.26047	0.001112	0.006639	5.345282	4.701066	5.776179	3.684038	4.164273	3.458449
AT1G42970	6166.447	1.393119824	0.151483	9.196515	3.70E-20	1.89E-18	12.85339	13.138	13.35801	11.76657	11.85289	11.5056
AT4G34610	68.81066	1.394658585	0.326474	4.27188	1.94E-05	1.81E-04	6.844636	6.520451	6.669636	4.021447	5.625572	5.482759
AT3G29810	28.66466	1.39549138	0.37011	3.770474	0.000163	0.0012217	5.459553	5.421786	5.488902	4.29471	3.710678	3.753887
AT1G73870	380.9552	1.395520856	0.159806	8.732614	2.49E-18	1.11E-16	8.935691	9.32042	9.072561	7.649677	7.723906	7.728545
AT1G31173	51.33931	1.397041718	0.300977	4.641697	3.46E-06	3.77E-05	6.122968	6.348216	6.279555	4.812122	5.123469	4.474702
AT1G05273	2240.254	1.401152815	0.203561	6.883197	5.85E-12	1.46E-10	11.62128	11.72754	11.68729	9.738801	10.09881	10.69458
AT3G57765	2259.348	1.403334788	0.199166	7.046057	1.84E-12	4.84E-11	11.60042	11.73929	11.73126	9.786605	10.09881	10.69567
AT3G11020	138.7616	1.403827323	0.248632	5.646195	1.64E-08	2.57E-07	7.485091	7.905054	7.622346	6.680852	5.82339	5.987621
AT4G36580	35.17899	1.404009458	0.352876	3.978758	6.93E-05	5.73E-04	5.785904	5.846675	5.621672	4.626795	3.416038	4.302755
AT1G29071	21.11788	1.404155131	0.435192	3.226517	1.25E-03	0.0073665	4.738256	5.307588	5.104121	2.958503	3.045364	3.998987
AT2G32645	155.9047	1.405043283	0.190032	7.393723	1.43E-13	4.21E-12	7.79909	7.775006	7.941902	6.270495	6.420349	6.506726
AT5G64310	145.9896	1.405363127	0.342901	4.098451	4.16E-05	0.0003612	7.705689	7.442148	8.096582	7.03764	5.553109	5.561185
AT4G23290	178.4317	1.406276461	0.221765	6.341283	2.28E-10	4.66E-09	7.831287	8.006151	8.221402	6.420628	7.013254	6.293554
AT4G04465	59.36602	1.409227223	0.304053	4.634801	3.57E-06	3.88E-05	6.415014	6.348216	6.687059	4.896561	4.346824	5.399825

AT2G39730	30756.04	1.409301949	0.140496	10.0309	1.12E-23	7.54E-22	15.2571	15.4638	15.6161	14.1771	14.09379	13.76868
AT3G19150	44.50045	1.409544564	0.385608	3.655377	2.57E-04	1.84E-03	6.051879	5.805191	6.379384	5.25748	3.045364	4.391288
AT2G48120	102.3233	1.414265495	0.223609	6.324719	2.54E-10	5.16E-09	7.124178	7.244235	7.348966	5.829815	5.760417	5.773726
AT5G07200	22.16783	1.416303792	0.407007	3.479803	5.02E-04	0.0033309	5.108917	5.117315	5.15509	3.480233	3.045364	3.881636
AT1G12570	32.27948	1.416439685	0.36346	3.8971	9.74E-05	7.73E-04	5.477749	5.819152	5.603436	4.29471	3.416038	4.302755
AT3G42640	75.95629	1.418324364	0.268991	5.272766	1.34E-07	1.86E-06	7.040965	6.763641	6.660846	5.545934	4.907958	5.441888
AT2G32650	205.9142	1.418674559	0.176098	8.056158	7.87E-16	2.83E-14	8.206155	8.178502	8.341788	6.607592	6.779171	6.961824
AT1G52290	76.31766	1.419892904	0.264103	5.376283	7.60E-08	1.09E-06	7.040965	6.689579	6.729716	5.123743	5.476813	5.441888
AT1G16070	23.57837	1.421194208	0.394461	3.602875	3.15E-04	2.20E-03	5.242615	5.183567	5.179914	3.684038	3.416038	3.753887
AT3G29034	25.9275	1.421289161	0.399779	3.555186	3.78E-04	2.59E-03	5.108917	5.475665	5.407161	4.29471	3.045364	3.613718
AT1G06087	93.87061	1.422360555	0.253179	5.618007	1.93E-08	3.00E-07	7.065234	7.382284	6.896258	5.646896	5.476813	5.773726
AT4G29740	20.66338	1.422466542	0.426429	3.335761	8.51E-04	5.25E-03	5.242615	4.788814	5.024124	2.958503	3.416038	3.753887
AT5G43750	272.0323	1.423224519	0.19873	7.161599	7.97E-13	2.19E-11	8.418853	8.620471	8.847022	7.127736	7.510873	6.90281
AT5G17000	177.8992	1.423771015	0.284256	5.008765	5.48E-07	6.85E-06	7.678415	8.170356	8.24512	7.110162	5.760417	6.404074
AT4G39510	125.946	1.424106722	0.216536	6.576761	4.81E-11	1.07E-09	7.33847	7.674917	7.594919	5.99213	6.050936	6.17386
AT2G35960	30.26758	1.425974027	0.365434	3.902139	9.53E-05	0.0007599	5.495717	5.561219	5.603436	4.021447	3.416038	4.302755
AT2G04790	42.18185	1.426380872	0.367236	3.884098	1.03E-04	0.0008118	6.063973	5.964416	6.042961	3.480233	4.164273	5.117892
AT1G24095	23.34605	1.428380889	0.413973	3.450422	0.00056	0.0036765	5.154874	5.047874	5.298008	3.684038	4.164273	2.85705
AT2G32530	60.59607	1.430059536	0.320805	4.457727	8.28E-06	8.34E-05	6.168486	6.651072	6.695692	5.437371	4.164273	5.01018
AT3G07735	33.08954	1.433177588	0.357904	4.004368	6.22E-05	5.19E-04	5.548318	5.860242	5.584968	4.414102	3.710678	4.107507
AT5G46490	293.5476	1.433753315	0.21365	6.710752	1.94E-11	4.53E-10	8.684714	8.829752	8.765313	7.766472	6.900955	7.004552
AT2G20723	20.8335	1.437544295	0.424258	3.388369	7.03E-04	4.46E-03	5.177314	4.816916	5.051286	3.242825	3.955229	3.08651
AT1G04897	767.6223	1.438099553	0.220748	6.514667	7.29E-11	1.59E-09	10.04285	9.96081	10.40247	8.174225	8.525969	9.101553
AT4G03230	80.28816	1.44154937	0.251034	5.742457	9.33E-09	1.51E-07	6.90639	6.763641	7.018279	5.379864	5.625572	5.265734
AT5G54070	8320.433	1.441635402	0.354413	4.067674	4.75E-05	0.000407	13.89985	13.27298	13.59421	11.79984	11.42058	12.56783
AT1G72790	210.4858	1.446637481	0.220437	6.562604	5.29E-11	1.18E-09	8.243855	7.978426	8.570211	7.03764	6.747048	6.564985
AT1G65486	25.2069	1.44681336	0.430347	3.361972	7.74E-04	4.85E-03	5.58235	4.670589	5.547306	3.684038	3.045364	3.998987
AT1G03506	20.81541	1.448814092	0.434371	3.335433	0.000852	0.0052556	5.132078	5.204992	4.720024	2.604042	3.955229	3.458449
AT5G44568	26.21258	1.449061666	0.417039	3.474644	5.12E-04	3.39E-03	5.695509	4.97492	5.320509	4.29471	3.416038	3.284433
AT4G19200	725.9162	1.450585325	0.167052	8.68344	3.84E-18	1.68E-16	10.31239	9.963955	9.868544	8.688146	8.712195	8.342109
AT5G63980	101.0342	1.450812327	0.227173	6.38638	1.70E-10	3.53E-09	7.124178	7.319828	7.264448	5.646896	5.625572	5.899804
AT1G48745	49.23424	1.450987647	0.341093	4.253937	2.10E-05	0.0001948	6.190717	6.04978	6.411182	5.25748	4.50885	3.998987
AT3G52450	36.1673	1.454681304	0.384991	3.778484	1.58E-04	0.0011879	6.134483	5.346667	5.82421	4.722432	3.955229	3.753887
AT5G17670	231.7073	1.455849396	0.205015	7.10119	1.24E-12	3.33E-11	8.437566	8.200001	8.607287	7.26112	6.747048	6.709736

AT1G35180	33.7139	1.457262246	0.372019	3.917168	8.96E-05	0.0007217	5.741414	5.832979	5.448611	4.164539	4.654502	3.458449
AT3G48090	257.0892	1.457711021	0.170294	8.559988	1.13E-17	4.77E-16	8.485562	8.561481	8.674337	7.245106	6.747048	7.176347
AT4G37925	299.7244	1.459522018	0.165719	8.80721	1.28E-18	5.87E-17	8.762841	8.769913	8.819638	7.353641	7.567152	7.004552
AT1G23410	39.26877	1.460591681	0.336357	4.342379	1.41E-05	0.0001361	5.741414	6.130373	5.82421	4.414102	4.346824	4.302755
AT1G04227	94.5832	1.462432919	0.267113	5.474956	4.38E-08	6.47E-07	6.879269	7.391657	7.156022	5.437371	5.310931	5.958939
AT2G40670	19.01438	1.463366506	0.431499	3.391357	6.95E-04	4.42E-03	4.738256	4.949759	5.077945	3.242825	3.045364	3.458449
AT5G26920	389.4514	1.463368049	0.295241	4.956526	7.18E-07	8.80E-06	9.001624	9.163753	9.378956	8.356696	6.871459	7.188751
AT5G09495	24.28072	1.464476266	0.406412	3.603426	0.000314	0.0022002	4.935458	5.384715	5.364486	3.684038	3.955229	3.284433
AT5G04305	760.7162	1.464809052	0.241157	6.074101	1.25E-09	2.31E-08	10.03596	9.969442	10.39586	8.182658	8.256129	9.165712
AT5G41471	54.18909	1.465290044	0.345182	4.244983	2.19E-05	0.000202	6.633638	6.319038	6.159877	4.164539	4.346824	5.399825
AT5G08155	3208.117	1.467149195	0.364524	4.024838	5.70E-05	0.0004797	12.57452	11.82999	12.24102	10.31363	10.09881	11.1701
AT1G10522	2749.602	1.46716357	0.189686	7.734687	1.04E-14	3.38E-13	12.24819	11.67574	11.9933	10.47852	10.11148	10.77905
AT2G31730	46.7612	1.467966436	0.313912	4.676362	2.92E-06	3.24E-05	6.244842	5.989326	6.18462	4.626795	4.907958	4.302755
AT4G08755	42.17441	1.470877073	0.340595	4.318552	1.57E-05	0.00015	5.843167	6.238216	5.944987	3.862584	4.50885	4.767144
AT3G12965	145.0503	1.472301442	0.204015	7.216648	5.33E-13	1.48E-11	7.572344	7.803375	7.863621	6.238498	6.379024	6.148682
AT1G10000	19.6835	1.472532681	0.439783	3.34832	0.000813	0.0050659	4.797403	5.094538	5.077945	2.958503	2.545235	3.881636
AT3G01145	80.49992	1.472643323	0.29855	4.932652	8.11E-07	9.86E-06	6.913091	7.223389	6.606946	5.051918	5.019734	5.806294
AT1G15550	35.13429	1.473723758	0.406833	3.622431	2.92E-04	2.06E-03	5.132078	5.926227	6.082995	3.242825	4.654502	4.208431
AT3G14450	50.24215	1.474114021	0.330287	4.463135	8.08E-06	8.15E-05	6.11136	6.25885	6.442294	4.29471	4.164273	5.218117
AT3G07715	1107.51	1.475044901	0.155039	9.514041	1.83E-21	1.03E-19	10.86212	10.76112	10.35897	9.158962	9.28766	9.088372
AT3G07610	195.9097	1.477021692	0.209101	7.063666	1.62E-12	4.29E-11	7.975916	8.42664	8.109561	6.556599	6.958197	6.506726
AT4G13572	126.2479	1.478593107	0.215101	6.873963	6.24E-12	1.55E-10	7.697949	7.50827	7.479679	6.205775	5.69457	6.123056
AT1G67470	59.50197	1.479712537	0.307042	4.819245	1.44E-06	1.67E-05	6.742991	6.185302	6.512382	5.25748	4.786789	4.699401
AT1G56520	273.0087	1.480035564	0.230441	6.422618	1.34E-10	2.83E-09	8.523279	8.653962	8.813702	7.612711	7.013254	6.620982
AT5G54100	64.58513	1.482347674	0.289891	5.113466	3.16E-07	4.10E-06	6.424426	6.485861	6.865983	5.051918	5.310931	4.831847
AT5G34871	43.38171	1.484430471	0.335634	4.422767	9.74E-06	9.68E-05	5.898243	6.196041	6.029365	4.812122	4.654502	3.881636
AT1G03457	104.7784	1.487120689	0.282515	5.26386	1.41E-07	1.94E-06	7.425408	6.74913	7.60412	5.694846	5.82339	5.706299
AT4G13494	105.1451	1.488476558	0.252989	5.883552	4.02E-09	6.86E-08	7.502969	7.377575	7.004448	5.545934	5.625572	6.015745
AT1G32520	87.64705	1.488584331	0.238876	6.23161	4.62E-10	8.99E-09	6.98482	7.085801	7.031979	5.437371	5.760417	5.356498
AT5G56985	80.09329	1.488592885	0.31849	4.673911	2.96E-06	3.27E-05	6.830546	6.404859	7.370693	5.437371	5.553109	5.01018
AT1G29450	324.0896	1.494127982	0.183032	8.163196	3.26E-16	1.21E-14	8.973048	8.748186	8.990744	7.26112	7.756543	7.125632
AT4G15430	64.47514	1.495035806	0.333052	4.488895	7.16E-06	7.32E-05	6.794708	6.635377	6.47275	4.626795	4.164273	5.671366
AT5G58770	23.74282	1.495075283	0.412717	3.622523	2.92E-04	2.06E-03	5.530997	5.139739	4.996442	3.862584	3.045364	3.613718
AT4G05020	134.9867	1.500019555	0.221197	6.781389	1.19E-11	2.85E-10	7.529377	7.841495	7.608698	6.391825	5.760417	6.096967

AT4G36280	21.97157	1.500610341	0.409086	3.668201	2.44E-04	1.77E-03	5.177314	5.047874	5.129831	3.242825	3.710678	3.458449
AT2G08765	61.84222	1.501790735	0.284936	5.270625	1.36E-07	1.88E-06	6.506476	6.627465	6.502575	5.25748	5.123469	4.553554
AT4G21870	41.19876	1.501930238	0.392648	3.825128	1.31E-04	1.01E-03	6.506476	5.43997	5.870694	3.684038	4.50885	4.62832
AT2G31865	328.2994	1.50282206	0.455921	3.296235	0.00098	0.0059372	8.89903	9.030597	9.08247	8.050685	6.247434	6.526408
AT4G25630	125.4532	1.503790805	0.258196	5.824212	5.74E-09	9.61E-08	7.202852	7.86187	7.557519	6.301797	5.69457	5.899804
AT3G03585	163.7578	1.504101553	0.281384	5.345371	9.02E-08	1.27E-06	7.943552	7.978426	7.949141	5.597298	6.102626	6.976208
AT2G13550	20.58324	1.504167643	0.44846	3.354076	7.96E-04	4.97E-03	5.325325	5.117315	4.685768	3.862584	1.77337	3.458449
AT5G56980	232.7321	1.505069275	0.262706	5.729111	1.01E-08	1.63E-07	8.329003	7.981533	8.887146	6.921529	7.166841	6.486772
AT2G30020	84.62027	1.506286233	0.274368	5.490019	4.02E-08	5.96E-07	7.186351	7.114389	6.669636	5.694846	5.019734	5.441888
AT2G34925	24.96266	1.506709588	0.396813	3.797029	0.000146	0.00111	5.221175	5.204992	5.468897	3.242825	3.710678	3.881636
AT1G17600	71.9033	1.507399019	0.327951	4.596414	4.30E-06	4.58E-05	6.78012	6.827194	6.746431	5.913254	4.346824	4.699401
AT1G06537	30.97928	1.507422488	0.392188	3.843619	1.21E-04	9.41E-04	5.495717	5.776858	5.488902	4.524365	3.955229	3.08651
AT5G06825	2096.59	1.507937005	0.154991	9.729169	2.26E-22	1.37E-20	11.4048	11.64985	11.74666	9.87511	9.940927	10.3471
AT2G35710	20.8836	1.508807424	0.445506	3.386724	7.07E-04	4.49E-03	5.154874	4.607633	5.320509	2.604042	3.955229	3.284433
AT5G50740	70.12367	1.510496933	0.32856	4.597329	4.28E-06	4.56E-05	6.122968	7.136858	6.803457	5.319969	5.019734	5.01018
AT1G69730	159.1793	1.51060358	0.236679	6.382496	1.74E-10	3.62E-09	7.975916	7.85511	7.905155	6.680852	5.476813	6.506726
AT1G22590	40.33671	1.512773363	0.344426	4.392159	1.12E-05	0.0001104	5.964257	5.657607	6.220956	4.164539	4.346824	4.474702
AT3G27170	93.73736	1.512984922	0.244901	6.177942	6.49E-10	1.24E-08	7.202852	7.074206	7.124399	5.694846	5.883728	5.168875
AT3G47342	72.15092	1.513035755	0.343626	4.403149	1.07E-05	1.05E-04	6.865515	7.212852	6.056429	5.379864	5.220243	4.831847
AT1G05563	30.00618	1.513490491	0.376205	4.023051	5.74E-05	4.83E-04	5.695509	5.54451	5.448611	4.29471	3.045364	3.998987
AT2G37100	40.36606	1.514713864	0.380404	3.981855	6.84E-05	5.66E-04	5.679876	5.577738	6.47275	4.164539	4.654502	4.107507
AT1G61260	45.75662	1.516919812	0.361227	4.199358	2.68E-05	0.0002427	5.695509	6.163581	6.502575	4.896561	3.710678	4.553554
AT1G05427	29.80347	1.5195469	0.375211	4.049847	5.13E-05	4.36E-04	5.648094	5.43997	5.584968	3.684038	3.416038	4.302755
AT1G04277	93.35052	1.519873465	0.241658	6.289362	3.19E-10	6.37E-09	7.028676	7.212852	7.199161	5.437371	5.310931	5.838142
AT5G04425	60.71352	1.52035564	0.306085	4.967105	6.80E-07	8.37E-06	6.617317	6.603464	6.431998	4.626795	4.346824	5.441888
AT1G28370	39.62307	1.520583168	0.373363	4.072665	4.65E-05	3.99E-04	6.051879	5.40337	6.2564	4.626795	4.164273	3.998987
AT1G61360	78.77038	1.521648043	0.328171	4.636748	3.54E-06	3.85E-05	7.147097	7.103022	6.390061	5.829815	4.654502	5.065041
AT5G51190	42.63612	1.522077783	0.355084	4.286527	1.81E-05	0.000171	6.11136	5.610218	6.302344	4.414102	4.786789	3.998987
AT4G19810	30.30424	1.522714992	0.452044	3.36851	7.56E-04	4.75E-03	5.403549	5.047874	6.147344	4.626795	2.545235	3.458449
AT3G44860	19.37587	1.528692307	0.475836	3.212646	1.32E-03	7.69E-03	4.644725	5.457928	4.685768	3.862584	3.045364	2.247075
AT2G38320	18.1337	1.531976821	0.480424	3.1888	0.001429	0.0082533	4.321109	4.788814	5.364486	2.133098	3.710678	3.08651
AT1G21520	98.19783	1.533432447	0.300121	5.109387	3.23E-07	4.18E-06	7.083171	6.977926	7.566961	6.138016	4.907958	5.441888
AT1G63240	170.1374	1.536214718	0.239106	6.424828	1.32E-10	2.79E-09	7.666567	8.191976	8.08677	6.102904	6.841348	6.246861
AT5G03520	660.3643	1.537741667	0.150429	10.22238	1.57E-24	1.12E-22	9.919317	9.846175	10.06123	8.157211	8.669986	8.314072

AT1G08103	40.14606	1.540905325	0.32908	4.68247	2.83E-06	3.15E-05	6.027383	6.001621	5.870694	4.414102	3.955229	4.474702
AT5G48490	662.598	1.54289978	0.228801	6.743408	1.55E-11	3.64E-10	10.12379	9.703877	10.02144	8.525303	8.703851	7.694153
AT3G55980	763.6298	1.543096087	0.17355	8.891376	6.04E-19	2.84E-17	10.14672	10.18491	10.15342	8.977016	8.312092	8.364152
AT2G29490	110.6736	1.544120401	0.316212	4.883175	1.04E-06	1.24E-05	6.787432	7.908323	7.293174	5.99213	5.82339	5.399825
AT4G34390	688.2917	1.545319839	0.196231	7.875023	3.41E-15	1.16E-13	9.90342	10.13625	9.995456	8.871439	8.149577	8.126918
AT3G25510	25.03475	1.547373821	0.457477	3.38241	7.19E-04	4.54E-03	5.459553	5.475665	5.104121	4.414102	0	3.458449
AT5G40690	98.11062	1.550737748	0.287664	5.390795	7.01E-08	1.01E-06	7.191872	7.396321	7.092067	6.205775	4.907958	5.31183
AT2G40100	41.54862	1.551167244	0.368904	4.204801	2.61E-05	0.0002377	5.565434	6.141527	6.302344	4.524365	3.416038	4.62832
AT4G18253	25.05407	1.552669907	0.434957	3.56971	3.57E-04	2.47E-03	5.364967	5.183567	5.468897	4.414102	1.77337	3.284433
AT3G46620	370.7975	1.553077897	0.206979	7.503551	6.21E-14	1.89E-12	9.063169	9.289416	9.008176	7.945604	7.411902	7.112669
AT2G31870	64.75974	1.553118774	0.289959	5.356336	8.49E-08	1.20E-06	6.396005	6.587239	6.8812	5.19216	4.907958	4.893774
AT1G71200	20.32656	1.55490508	0.430303	3.613508	3.02E-04	2.12E-03	5.305088	4.89808	4.880072	3.480233	3.045364	3.284433
AT3G09162	13.59339	1.557072357	0.489769	3.179196	0.001477	0.0084764	4.194811	4.575094	4.753486	2.958503	1.77337	2.85705
AT1G65490	171.0796	1.558040956	0.274715	5.671473	1.42E-08	2.24E-07	8.397507	7.647793	7.916278	6.704469	6.537663	5.806294
AT3G09600	176.9403	1.559247802	0.337939	4.613989	3.95E-06	4.25E-05	8.507867	7.965931	7.684385	6.901235	5.019734	6.583893
AT1G06233	51.68433	1.56386615	0.310226	5.041047	4.63E-07	5.85E-06	6.201706	6.528971	6.232868	4.896561	4.164273	4.767144
AT1G09883	28.10634	1.56435388	0.38633	4.049271	5.14E-05	4.37E-04	5.364967	5.626188	5.385981	3.862584	4.164273	3.284433
AT1G06620	56.37822	1.564511441	0.413104	3.787208	0.000152	0.0011501	6.396005	6.468249	6.560439	5.694846	3.710678	3.613718
AT4G13495	1685.793	1.564836546	0.11916	13.13222	2.15E-39	3.44E-37	11.42134	11.28674	11.18993	9.783836	9.826459	9.558776
AT5G01505	91.36026	1.570771069	0.255952	6.136982	8.41E-10	1.58E-08	6.939588	7.259674	7.186967	5.379864	5.123469	5.806294
AT3G14170	32.24291	1.571111026	0.364585	4.309312	1.64E-05	1.56E-04	5.648094	5.762479	5.603436	3.480233	3.955229	4.302755
AT1G32870	208.0889	1.572036327	0.278413	5.646418	1.64E-08	2.57E-07	7.950083	8.573885	8.344535	7.245106	6.247434	6.198607
AT4G06115	23.2642	1.576995548	0.410788	3.838948	1.24E-04	9.55E-04	5.384387	5.267421	4.968217	3.480233	3.710678	3.284433
AT4G34150	399.3373	1.583060735	0.199025	7.954085	1.80E-15	6.33E-14	9.29376	8.972443	9.417303	7.965288	7.472095	7.329843
AT1G34180	37.26261	1.583974822	0.468525	3.380766	7.23E-04	4.57E-03	5.530997	6.217283	5.930431	5.123743	2.545235	2.85705
AT3G14870	74.93919	1.588894636	0.281398	5.646437	1.64E-08	2.57E-07	6.697136	6.7122	7.11799	4.896561	5.123469	5.441888
AT4G09030	59.99582	1.593086443	0.324855	4.903999	9.39E-07	1.13E-05	6.46147	6.289258	6.835058	5.379864	4.346824	4.553554
AT1G31540	414.287	1.593193354	0.26857	5.932141	2.99E-09	5.22E-08	9.287327	9.294422	9.339562	8.303563	6.985988	7.213246
AT1G14870	103.042	1.594213172	0.368664	4.324294	1.53E-05	1.47E-04	7.251253	7.300054	7.433975	6.476563	4.786789	4.62832
AT5G09570	17.81985	1.594438187	0.467549	3.410202	6.49E-04	4.17E-03	4.707748	4.949759	4.996442	3.862584	1.77337	2.584062
AT1G78860	55.53245	1.596122883	0.296581	5.381745	7.38E-08	1.06E-06	6.609086	6.376816	6.279555	4.626795	4.786789	4.831847
AT1G49900	16.75643	1.598118357	0.487092	3.280938	0.001035	0.0062335	4.797403	5.24691	4.242928	3.480233	1.77337	2.85705
AT5G56850	226.3206	1.599037227	0.197897	8.080145	6.47E-16	2.35E-14	8.293491	8.671383	8.27423	6.96128	6.779171	6.620982
AT3G62960	15.24898	1.60193768	0.486553	3.292425	0.000993	0.006007	4.321109	4.607633	5.051286	2.133098	2.545235	3.284433

AT1G32220	230.9885	1.60352835	0.219813	7.294971	2.99E-13	8.44E-12	8.147693	8.428918	8.732128	6.530411	7.11744	6.743763
AT4G00883	34.76275	1.606459503	0.354424	4.532587	5.83E-06	6.04E-05	5.615596	5.964416	5.726492	4.021447	4.164273	3.998987
AT2G30040	149.5652	1.607683931	0.215467	7.461395	8.56E-14	2.57E-12	7.682343	7.868599	7.923646	6.362435	6.379024	5.806294
AT4G00165	41.57807	1.609546756	0.346807	4.641039	3.47E-06	3.78E-05	5.785904	6.025901	6.279555	4.29471	3.710678	4.62832
AT4G06795	17.25089	1.609600752	0.474078	3.395221	6.86E-04	4.37E-03	4.61215	5.047874	4.910056	3.480233	0	3.284433
AT1G14780	15.04381	1.61093222	0.498246	3.233209	1.22E-03	7.21E-03	4.85422	4.639454	4.501109	3.684038	1.77337	1.806583
AT1G07620	18.36401	1.612299297	0.488317	3.301745	9.61E-04	0.0058341	4.88181	5.139739	4.818167	4.021447	0	2.584062
AT2G18328	81.42554	1.613466949	0.247042	6.531132	6.53E-11	1.43E-09	7.034833	6.907798	6.954967	5.319969	5.396255	5.218117
AT2G06835	47.74536	1.614390739	0.313163	5.155108	2.53E-07	3.35E-06	6.297009	6.152596	6.196834	4.812122	4.50885	4.208431
AT3G46490	15.97443	1.616199613	0.488879	3.305931	0.000947	0.0057597	5.085378	4.113676	4.880072	2.133098	3.045364	3.08651
AT1G20510	326.716	1.618770236	0.214247	7.555625	4.17E-14	1.28E-12	8.672917	8.981783	9.193304	7.63746	6.841348	7.261021
AT3G15354	141.3054	1.618834674	0.202974	7.975575	1.52E-15	5.33E-14	7.717222	7.851718	7.710177	6.138016	5.82339	6.246861
AT4G09035	54.20157	1.618864399	0.335375	4.827025	1.39E-06	1.62E-05	6.223435	6.163581	6.762956	5.123743	4.346824	4.391288
AT1G19150	374.023	1.619882953	0.214756	7.542899	4.60E-14	1.41E-12	8.803081	9.129149	9.455927	7.625138	7.673524	7.125632
AT5G23060	3142.385	1.620217523	0.126461	12.81198	1.41E-37	1.98E-35	12.07675	12.16001	12.39174	10.66911	10.64789	10.40672
AT1G78340	28.91179	1.623614663	0.456763	3.554612	3.79E-04	2.60E-03	5.495717	6.03789	4.968217	4.524365	2.545235	3.08651
AT1G73177	26.09084	1.625187567	0.396982	4.093861	4.24E-05	3.68E-04	5.384387	5.24691	5.528099	4.021447	3.416038	3.284433
AT4G06800	17.4346	1.626924799	0.472761	3.441323	0.000579	0.0037773	4.644725	5.071394	4.910056	3.480233	0	3.284433
AT2G20750	26.29291	1.628151385	0.419316	3.882877	1.03E-04	0.0008152	5.284563	5.204992	5.657465	2.604042	4.346824	3.458449
AT5G64750	61.93705	1.630378371	0.466759	3.492978	0.000478	0.0031939	6.297009	6.868059	6.729716	5.829815	3.416038	3.08651
AT2G43110	82.15733	1.63247912	0.253617	6.43679	1.22E-10	2.60E-09	6.98482	6.965426	7.04555	5.379864	4.786789	5.522505
AT2G33330	91.28033	1.633011476	0.247399	6.600724	4.09E-11	9.21E-10	7.11258	7.114389	7.211253	5.19216	5.220243	5.773726
AT5G51720	25.92156	1.634394524	0.397068	4.116154	3.85E-05	3.37E-04	5.263741	5.287644	5.584968	3.242825	3.710678	3.753887
AT1G01790	475.677	1.63558264	0.191503	8.540769	1.33E-17	5.60E-16	9.26916	9.541789	9.669287	7.383209	8.073902	7.948751
AT5G46500	36.97643	1.636520436	0.42253	3.873148	1.07E-04	0.0008425	5.898243	6.130373	5.639679	4.97633	2.545235	3.458449
AT2G34760	79.2176	1.63964249	0.316857	5.174714	2.28E-07	3.04E-06	6.77277	6.734471	7.252795	5.492674	5.553109	4.391288
AT3G44205	19.63127	1.643919709	0.476607	3.449213	5.62E-04	3.69E-03	4.88181	5.267421	4.968217	4.021447	0	2.85705
AT1G17610	39.42972	1.644399882	0.436489	3.767332	1.65E-04	1.24E-03	5.785904	5.976925	6.208946	5.123743	2.545235	3.284433
AT3G57640	32.06211	1.645103924	0.385171	4.271101	1.95E-05	0.0001819	5.898243	5.688357	5.488902	4.164539	2.545235	4.208431
AT3G47347	119.1673	1.645491316	0.238997	6.884994	5.78E-12	1.44E-10	7.572344	7.612154	7.392097	6.205775	5.220243	5.806294
AT1G72920	37.75636	1.646724624	0.343546	4.793318	1.64E-06	1.89E-05	5.800434	5.805191	6.082995	4.164539	4.164273	4.107507
AT5G52750	111.4161	1.647885354	0.390445	4.220531	2.44E-05	0.000223	7.614069	7.169918	7.581008	6.391825	3.416038	5.598855
AT1G31290	63.90087	1.651029865	0.30112	5.482971	4.18E-08	6.19E-07	6.46147	6.734471	6.738098	5.379864	4.164273	4.831847
AT4G00163	38.17759	1.652459605	0.355166	4.652636	3.28E-06	3.59E-05	5.679876	5.93907	6.147344	4.021447	3.710678	4.474702

AT5G09935	1660.073	1.654438197	0.217764	7.597406	3.02E-14	9.41E-13	11.21244	11.43009	11.29344	9.047962	9.461046	10.09036
AT5G12340	14.01783	1.655248642	0.520945	3.177397	1.49E-03	0.0085178	3.956125	5.071394	4.614715	3.242825	0	2.584062
AT3G19970	238.828	1.658259474	0.266427	6.224066	4.84E-10	9.42E-09	8.402278	8.464884	8.698162	7.383209	6.574739	6.123056
AT3G01290	1100.179	1.659538908	0.188581	8.800149	1.37E-18	6.20E-17	10.63422	10.6721	10.8403	9.444897	8.769286	8.712309
AT2G09395	98.2803	1.661294592	0.257066	6.462523	1.03E-10	2.20E-09	7.533732	7.044803	7.137131	5.437371	5.476813	5.635566
AT4G12830	23.93451	1.663301483	0.417667	3.982358	6.82E-05	5.65E-04	5.221175	5.24691	5.342665	2.604042	4.164273	3.284433
AT2G32160	71.97206	1.665360529	0.346656	4.804075	1.55E-06	1.80E-05	6.77277	7.014788	6.695692	5.786214	3.710678	4.767144
AT3G01440	49.58704	1.668639114	0.322956	5.16677	2.38E-07	3.15E-06	6.063973	6.30918	6.452518	4.021447	4.907958	4.553554
AT1G54820	186.6005	1.670656921	0.181016	9.229321	2.72E-20	1.40E-18	8.136283	8.207982	8.160341	6.632428	6.200761	6.486772
AT5G18660	225.6261	1.671779625	0.194856	8.579565	9.52E-18	4.04E-16	8.170244	8.544774	8.563151	6.582321	6.900955	6.709736
AT1G42990	818.4943	1.672485511	0.214807	7.785988	6.92E-15	2.30E-13	10.28035	10.31868	10.30075	9.029381	7.923931	8.529329
AT3G25882	30.3212	1.675085803	0.396479	4.224909	2.39E-05	2.19E-04	5.857134	5.365817	5.639679	3.862584	2.545235	4.208431
AT1G66160	114.5945	1.675171606	0.285894	5.859412	4.65E-09	7.86E-08	7.363149	7.529654	7.557519	6.362435	4.907958	5.441888
AT1G76600	524.7354	1.675235235	0.508224	3.296254	9.80E-04	0.0059372	9.599216	9.82471	9.834747	8.545058	6.460523	6.602557
AT2G04435	1405.195	1.675407626	0.182447	9.182964	4.19E-20	2.13E-18	10.96728	11.16289	11.08869	8.937702	9.28766	9.729815
AT2G04455	1405.195	1.675407626	0.182447	9.182964	4.19E-20	2.13E-18	10.96728	11.16289	11.08869	8.937702	9.28766	9.729815
AT2G23100	65.78155	1.67655611	0.288091	5.819541	5.90E-09	9.85E-08	6.633638	6.587239	6.811422	5.25748	5.019734	4.474702
AT2G07739	1357.562	1.677500488	0.13058	12.8465	9.00E-38	1.30E-35	10.85256	10.99893	11.19107	9.233904	9.270813	9.447037
AT3G47480	86.12641	1.681282661	0.288613	5.825395	5.70E-09	9.56E-08	7.047071	7.002605	7.199161	5.786214	4.164273	5.399825
AT1G35560	60.32933	1.684763625	0.293636	5.737592	9.60E-09	1.55E-07	6.567215	6.603464	6.569861	5.051918	3.955229	5.01018
AT2G18120	9.924615	1.685152351	0.533714	3.157406	1.59E-03	9.02E-03	4.194811	3.970903	4.33422	2.133098	0	2.584062
AT2G44370	14.61173	1.685733582	0.49584	3.399754	0.000674	0.004308	4.961552	4.575094	4.420076	2.958503	0	3.08651
AT1G32583	39.06267	1.686067251	0.344816	4.889761	1.01E-06	1.20E-05	5.756397	6.025901	6.069773	4.414102	4.164273	3.881636
AT5G49480	360.1116	1.686180863	0.252774	6.670707	2.55E-11	5.88E-10	8.868326	9.328984	9.138951	7.935661	7.013254	6.887672
AT2G08860	689.3208	1.688788309	0.125159	13.49309	1.72E-41	2.96E-39	9.962758	10.09596	10.08196	8.349224	8.161812	8.464489
AT1G19180	291.8292	1.694777107	0.320389	5.289756	1.22E-07	1.70E-06	8.634912	8.953581	8.913289	7.661791	5.625572	7.018519
AT5G49520	68.53049	1.695851337	0.315578	5.373799	7.71E-08	1.10E-06	6.633638	6.834086	6.787392	5.597298	4.654502	4.302755
AT3G16510	59.29932	1.697047775	0.314043	5.403868	6.52E-08	9.43E-07	6.479641	6.494586	6.660846	5.319969	4.50885	4.208431
AT5G46295	13.13839	1.698757955	0.521264	3.258923	1.12E-03	6.67E-03	4.707748	4.788814	3.985697	3.242825	0	2.247075
AT2G45680	88.91113	1.699838542	0.288183	5.898462	3.67E-09	6.30E-08	6.851629	6.990318	7.444257	5.597298	5.476813	4.893774
AT1G61890	565.4079	1.70018003	0.199022	8.542675	1.31E-17	5.52E-16	9.583549	9.514163	10.13291	8.199376	7.772588	8.04741
AT3G62760	10.26886	1.705919103	0.538028	3.170686	0.001521	0.0086899	3.903255	4.067637	4.614715	2.133098	0	2.584062
AT5G14565	21.05813	1.706190583	0.435902	3.91416	9.07E-05	0.0007292	5.061448	5.094538	5.228318	2.133098	3.416038	3.613718
AT5G08795	99.42746	1.706683716	0.391976	4.354048	1.34E-05	0.0001297	7.959825	7.32473	6.082995	5.741253	5.220243	5.117892

AT2G45685	89.49713	1.711082189	0.288334	5.93438	2.95E-09	5.16E-08	6.865515	6.996475	7.459545	5.597298	5.476813	4.893774
AT1G35140	658.3554	1.711374453	0.213503	8.015675	1.10E-15	3.90E-14	9.991536	9.736427	10.20724	8.491765	8.40766	7.66781
AT3G48520	14.05604	1.711445733	0.495172	3.456265	5.48E-04	0.003606	4.644725	4.89808	4.195021	2.958503	1.77337	2.584062
AT1G69790	43.94721	1.713255881	0.370324	4.626376	3.72E-06	4.02E-05	5.631937	6.395572	6.313604	4.626795	3.416038	4.391288
AT2G23110	22.03342	1.718948701	0.519079	3.311539	9.28E-04	5.66E-03	4.85422	5.457928	5.364486	4.29471	0	1.806583
AT5G23750	157.772	1.723650508	0.218437	7.890818	3.00E-15	1.03E-13	7.701824	7.914839	8.157219	6.172293	6.336481	6.015745
AT4G13900	105.1842	1.725445962	0.332949	5.182316	2.19E-07	2.92E-06	7.261791	7.358582	7.528819	6.270495	4.164273	5.31183
AT2G19650	31.42615	1.725970676	0.371551	4.645317	3.40E-06	3.71E-05	5.599069	5.718464	5.726492	4.021447	3.045364	3.998987
AT2G38340	57.4517	1.726278814	0.387621	4.453522	8.45E-06	8.49E-05	6.122968	6.990318	6.368627	5.25748	3.045364	4.62832
AT2G38780	404.3053	1.729781776	0.145684	11.8735	1.63E-32	1.82E-30	9.258675	9.414232	9.182596	7.625138	7.452309	7.518562
AT5G17760	88.72049	1.731204355	0.274693	6.302326	2.93E-10	5.91E-09	7.106746	7.290065	6.997482	4.97633	4.907958	5.773726
AT3G55850	1022.32	1.73140233	0.183803	9.419871	4.52E-21	2.46E-19	10.39729	10.4022	11.00129	8.746188	8.831881	8.97477
AT5G43890	22.18475	1.736462226	0.426597	4.070499	4.69E-05	0.0004025	5.305088	4.97492	5.342665	3.242825	2.545235	3.613718
AT3G56400	556.249	1.737075426	0.469903	3.696669	0.000218	0.0015965	9.68296	9.746507	10.03766	8.551584	6.574739	7.138479
AT5G17330	93.12929	1.737178868	0.27071	6.417127	1.39E-10	2.92E-09	7.169658	6.977926	7.418413	5.786214	4.907958	5.265734
AT2G24600	100.4306	1.737728496	0.317694	5.469819	4.50E-08	6.65E-07	7.529377	7.002605	7.359871	6.138016	5.019734	4.831847
AT1G29430	166.2318	1.741855218	0.20246	8.603464	7.73E-18	3.31E-16	8.092675	7.810381	8.122425	6.238498	6.336481	6.148682
AT3G58150	14.77001	1.742987512	0.496075	3.513558	0.000442	0.0029806	4.61215	4.158291	5.104121	2.604042	2.545235	2.584062
AT5G51465	12.76086	1.743744455	0.510192	3.417821	6.31E-04	4.07E-03	4.61215	4.670589	4.195021	2.604042	0	2.85705
AT3G09525	12.84157	1.745547278	0.504287	3.461419	0.000537	0.0035458	4.399556	4.541804	4.577832	2.958503	0	2.584062
AT3G55950	54.78127	1.747655175	0.329369	5.306077	1.12E-07	1.56E-06	6.541497	6.130373	6.652001	5.051918	3.955229	4.474702
AT1G29440	358.1343	1.750837347	0.157724	11.10062	1.25E-28	1.11E-26	9.14941	8.95832	9.226449	7.308122	7.510873	7.225339
AT4G01540	26.04648	1.752611123	0.400784	4.372961	1.23E-05	1.20E-04	5.495717	5.457928	5.342665	3.862584	2.545235	3.613718
AT3G47780	115.6956	1.755979597	0.404643	4.33958	1.43E-05	0.0001376	7.494057	7.62015	7.528819	6.582321	4.164273	4.62832
AT1G15990	55.72591	1.759435006	0.323317	5.441835	5.27E-08	7.70E-07	6.11136	6.511881	6.738098	4.29471	4.786789	4.699401
AT5G52740	14.74971	1.759704893	0.552055	3.187554	1.43E-03	8.28E-03	4.826091	4.639454	4.720024	3.684038	0	0
AT1G72540	24.86693	1.764374081	0.45828	3.849994	1.18E-04	0.0009198	5.108917	5.641983	5.428035	3.242825	0	4.107507
AT4G12917	35.45327	1.765768166	0.359614	4.910173	9.10E-07	1.10E-05	5.857134	5.626188	6.029365	3.862584	3.955229	3.998987
AT2G05940	270.5798	1.766442545	0.523742	3.372732	0.000744	0.0046851	8.688624	9.057347	8.736318	7.368501	4.654502	6.070398
AT1G11735	16.85654	1.767236847	0.459187	3.848625	0.000119	0.0009246	4.797403	4.730912	4.996442	2.958503	2.545235	2.85705
AT4G39364	308.3791	1.769571057	0.170802	10.36034	3.76E-25	2.74E-23	8.700294	9.023078	8.990744	7.127736	6.958197	7.201051
AT5G16360	56.97742	1.772002977	0.317495	5.581191	2.39E-08	3.66E-07	6.600809	6.643246	6.268024	5.123743	4.164273	4.391288
AT3G25655	8.393597	1.77275502	0.554742	3.195639	0.001395	0.0080856	3.903255	3.696207	4.289296	2.133098	0	1.806583
AT5G08845	93.97232	1.773664211	0.4129	4.295631	1.74E-05	1.65E-04	7.9337	7.244235	5.944987	5.646896	5.220243	4.62832

AT1G10340	217.1943	1.773826022	0.512533	3.460902	5.38E-04	0.003549	8.483312	8.495635	8.558426	7.179211	5.019734	5.168875
AT1G29435	80.96671	1.77394394	0.276234	6.421888	1.35E-10	2.84E-09	7.034833	6.697159	7.229204	5.123743	5.220243	5.117892
AT3G54150	51.07371	1.775255342	0.329921	5.380851	7.41E-08	1.06E-06	6.244842	6.338556	6.512382	4.97633	3.416038	4.474702
AT5G27780	45.59659	1.777888055	0.350972	5.065618	4.07E-07	5.19E-06	6.265936	5.762479	6.48276	4.414102	4.346824	4.107507
AT3G48420	398.6965	1.780865142	0.208771	8.530242	1.46E-17	6.11E-16	8.94553	9.247442	9.588153	7.145099	7.707307	7.468567
AT1G69900	54.1374	1.781986338	0.311509	5.720491	1.06E-08	1.70E-07	6.201706	6.441423	6.625137	4.524365	4.786789	4.391288
AT3G05325	26.63436	1.782905974	0.427371	4.171798	3.02E-05	2.71E-04	5.548318	5.703489	5.024124	3.684038	3.955229	2.584062
AT3G02725	68.69996	1.783007222	0.312698	5.702014	1.18E-08	1.89E-07	6.337426	7.085801	6.811422	5.051918	4.907958	4.699401
AT1G61340	38.36672	1.786223167	0.362669	4.925215	8.43E-07	1.02E-05	5.938213	6.141527	5.839872	3.684038	3.416038	4.553554
AT1G06100	8.921386	1.786595378	0.556603	3.209822	0.001328	0.0077477	3.605233	4.607633	3.740762	1.429371	1.77337	1.806583
AT4G22570	337.819	1.789374114	0.156587	11.42733	3.05E-30	2.95E-28	8.887167	9.09082	9.138951	7.308122	7.214606	7.163834
AT2G32200	22.5741	1.789545985	0.50757	3.525715	4.22E-04	2.86E-03	5.305088	5.287644	5.27515	4.29471	0	1.806583
AT1G36622	14.66665	1.790301429	0.495233	3.615067	3.00E-04	2.11E-03	4.61215	4.89808	4.53998	3.242825	0	2.584062
AT5G39860	175.4684	1.79047928	0.201265	8.896119	5.78E-19	2.72E-17	8.107358	8.012241	8.191195	5.872138	6.460523	6.425197
AT5G25920	10.22561	1.793032006	0.572545	3.131688	1.74E-03	0.0097449	3.046206	4.97492	4.195021	2.604042	0	1.169325
AT3G46900	53.57749	1.796536952	0.320021	5.613808	1.98E-08	3.06E-07	6.337426	6.30918	6.634148	4.97633	3.955229	4.391288
AT5G07165	9.478652	1.796828998	0.563848	3.186728	1.44E-03	8.30E-03	3.391538	4.113676	4.685768	2.604042	0	1.169325
AT5G35580	36.05704	1.796879177	0.384897	4.668463	3.03E-06	3.34E-05	5.756397	5.791094	6.082995	4.626795	3.416038	3.284433
AT4G25380	11.54173	1.79787731	0.534893	3.361192	0.000776	0.0048568	3.903255	4.437061	4.720024	2.958503	0	1.806583
AT4G28460	103.7109	1.801745556	0.571252	3.154032	1.61E-03	9.11E-03	7.638536	7.6517	7.252795	5.741253	1.77337	4.208431
AT1G05575	51.37992	1.801945028	0.390763	4.611347	4.00E-06	4.29E-05	6.697136	6.299253	6.147344	5.19216	2.545235	4.208431
AT5G60010	8.237739	1.802726436	0.5722	3.15052	0.00163	0.0092044	4.360866	3.812433	3.67243	2.604042	0	0
AT4G04700	22.97313	1.803614381	0.43887	4.109677	3.96E-05	3.45E-04	5.345282	5.307588	5.228318	3.684038	0	3.613718
AT1G08507	8.865726	1.803821227	0.553795	3.257199	0.001125	0.0066989	4.280226	3.867214	3.985697	2.604042	0	1.169325
AT5G12880	10.05177	1.804187212	0.535741	3.36765	7.58E-04	0.0047641	4.194811	3.812433	4.501109	2.133098	1.77337	1.806583
AT1G71280	14.94016	1.806060531	0.526488	3.430395	6.03E-04	3.91E-03	4.150129	5.139739	4.753486	3.480233	0	1.806583
AT4G03295	26.8652	1.807120262	0.413862	4.366477	1.26E-05	1.23E-04	5.263741	5.641983	5.56626	3.862584	1.77337	3.753887
AT3G48280	10.03035	1.809793906	0.545445	3.318014	9.07E-04	5.54E-03	3.848373	4.362754	4.377786	0	1.77337	2.584062
AT2G10735	19.65002	1.811087954	0.442988	4.088342	4.34E-05	3.76E-04	5.177314	4.844481	5.15509	2.958503	2.545235	3.284433
AT1G27960	105.1125	1.811458428	0.305399	5.931444	3.00E-09	5.24E-08	6.991167	7.114389	7.897692	5.25748	5.553109	5.561185
AT5G64905	10.22572	1.812999551	0.560162	3.236566	0.00121	0.0071463	4.676581	4.113676	3.928251	0	0	2.85705
AT2G33530	85.14893	1.814394815	0.259198	7.000041	2.56E-12	6.64E-11	7.071238	7.074206	7.130779	5.492674	4.50885	5.31183
AT5G02490	227.5202	1.815855302	0.529315	3.430579	6.02E-04	3.91E-03	8.534187	8.684788	8.586549	7.056115	4.164273	5.740407
AT1G67800	137.7516	1.81665306	0.256269	7.088849	1.35E-12	3.62E-11	7.701824	7.663355	7.945526	6.420628	5.310931	5.635566

AT3G12320	741.4432	1.819645571	0.17326	10.50239	8.42E-26	6.40E-24	10.38227	10.11023	10.04359	8.615264	7.966403	8.308399
AT2G13940	8.643072	1.820338725	0.574344	3.169425	1.53E-03	8.72E-03	3.391538	4.472827	4.094152	2.604042	0	0
AT3G61630	336.3544	1.822104008	0.214643	8.488987	2.08E-17	8.61E-16	8.950425	8.897114	9.289114	7.426454	6.537663	7.341001
AT2G37540	124.293	1.827400406	0.226541	8.066527	7.23E-16	2.61E-14	7.546718	7.587895	7.722903	6.066916	5.625572	5.482759
AT5G34851	15.3664	1.827437118	0.528018	3.460939	0.000538	0.003549	4.104018	5.527604	4.242928	2.958503	1.77337	2.247075
AT5G58120	182.3609	1.827913825	0.231659	7.890537	3.01E-15	1.03E-13	8.121893	8.117671	8.28285	6.795248	5.82339	6.015745
AT1G13330	75.26019	1.82860792	0.347966	5.255132	1.48E-07	2.02E-06	6.899657	7.239052	6.606946	5.545934	3.416038	5.01018
AT3G45090	60.22633	1.829316156	0.30056	6.086362	1.16E-09	2.14E-08	6.567215	6.689579	6.579222	4.626795	3.955229	5.065041
AT5G50915	91.25886	1.829420371	0.270632	6.759815	1.38E-11	3.28E-10	7.129942	7.191545	7.229204	5.786214	5.123469	4.767144
AT3G56290	87.35716	1.833035222	0.298137	6.148303	7.83E-10	1.48E-08	7.175244	6.984135	7.193077	5.597298	5.476813	4.302755
AT4G09845	186.0464	1.836484108	0.190788	9.625762	6.22E-22	3.62E-20	8.130544	8.339725	8.122425	6.205775	6.292644	6.446015
AT5G08525	41.76358	1.837511446	0.44284	4.14938	3.33E-05	2.95E-04	6.201706	6.7122	4.996442	3.862584	3.416038	4.391288
AT5G04465	156.6784	1.840110779	0.278126	6.6161	3.69E-11	8.35E-10	7.903732	7.775006	8.209395	5.492674	5.625572	6.602557
AT5G04485	156.6784	1.840110779	0.278126	6.6161	3.69E-11	8.35E-10	7.903732	7.775006	8.209395	5.492674	5.625572	6.602557
AT1G15980	552.0887	1.841050101	0.18868	9.757528	1.71E-22	1.05E-20	9.346696	9.886513	9.98581	7.810665	7.923931	7.904311
AT5G45340	28.38093	1.843046791	0.396228	4.651485	3.30E-06	3.61E-05	5.58235	5.657607	5.488902	3.480233	2.545235	3.998987
AT5G41765	8.43368	1.844642504	0.573526	3.216319	1.30E-03	0.0076038	3.140592	4.639454	3.928251	2.133098	0	1.169325
AT1G05894	9.518443	1.846993659	0.546972	3.376761	0.000733	0.0046261	3.956125	4.472827	3.868421	1.429371	2.545235	1.169325
AT3G46370	22.30156	1.850872693	0.463607	3.992331	6.54E-05	0.0005441	4.676581	5.626188	5.320509	2.133098	3.045364	3.613718
AT3G15310	13.40893	1.852320008	0.507407	3.650561	2.62E-04	1.87E-03	4.150129	4.575094	4.880072	2.604042	2.545235	1.806583
AT3G48290	10.1517	1.853297705	0.550645	3.365684	7.64E-04	4.79E-03	3.903255	4.020081	4.614715	2.133098	2.545235	0
AT3G22930	33.71976	1.855204071	0.414338	4.477518	7.55E-06	7.68E-05	5.459553	5.493187	6.290994	3.862584	4.164273	3.08651
AT3G13210	13.25474	1.855950037	0.524327	3.539683	4.01E-04	2.73E-03	4.194811	4.760154	4.614715	1.429371	3.416038	1.169325
AT1G19050	70.20852	1.856493231	0.286481	6.480344	9.15E-11	1.98E-09	6.830546	6.799293	6.835058	5.379864	4.50885	4.553554
AT1G53620	15.82156	1.85774132	0.50435	3.683434	2.30E-04	1.67E-03	4.321109	5.204992	4.786189	3.242825	0	2.584062
AT1G63820	5.275335	1.859873929	0.588209	3.161929	1.57E-03	0.008906	3.466342	3.194694	3.67243	1.429371	0	0
AT5G66630	35.27238	1.86121369	0.441352	4.217074	2.47E-05	0.0002261	5.898243	5.762479	6.029365	4.722432	0	3.458449
AT3G04155	38.32853	1.861834626	0.376397	4.946471	7.56E-07	9.22E-06	5.964257	6.174482	5.743245	4.414102	4.164273	3.08651
AT1G70185	1005.287	1.862398727	0.197075	9.450222	3.38E-21	1.85E-19	10.42861	10.35207	11.02888	8.564547	8.644055	8.920019
AT4G13575	1029.786	1.863933835	0.152049	12.25875	1.51E-34	1.83E-32	10.86039	10.66583	10.43956	8.957493	8.703851	8.637358
AT1G55775	25.54311	1.863958973	0.491442	3.792833	1.49E-04	1.13E-03	5.771226	5.703489	4.720024	4.164539	0	2.85705
AT1G72930	187.4393	1.86479373	0.301356	6.188018	6.09E-10	1.17E-08	8.395116	8.009199	8.28285	6.941541	5.220243	6.015745
AT3G26830	127.4752	1.865389477	0.346313	5.386425	7.19E-08	1.03E-06	7.678415	8.015276	7.326908	5.913254	3.955229	6.148682
AT2G24285	5.141653	1.865802506	0.589107	3.167173	0.001539	0.0087702	3.140592	3.75549	3.361256	0	0	1.169325

AT3G07385	8.895565	1.865983389	0.553814	3.369335	7.53E-04	4.74E-03	4.056385	3.75549	4.377786	1.429371	0	2.247075
AT5G04415	168.7856	1.86958383	0.264209	7.07616	1.48E-12	3.95E-11	7.773542	7.975313	8.401053	5.492674	6.499609	6.222936
AT3G23170	149.2369	1.869695172	0.249392	7.497024	6.53E-14	1.98E-12	7.848869	7.538119	8.230342	6.066916	5.760417	5.987621
AT2G26390	3.936649	1.870042639	0.592163	3.157987	1.59E-03	9.01E-03	3.140592	2.911631	3.361256	0	0	0
AT4G35180	12.74245	1.874768486	0.536262	3.495997	4.72E-04	0.0031604	4.738256	4.113676	4.685768	3.242825	0	1.169325
AT4G31950	4.63874	1.874852838	0.591664	3.168781	1.53E-03	0.0087334	4.150129	2.559128	2.844834	0	0	0
AT4G33070	14.83313	1.877127994	0.515502	3.641362	2.71E-04	0.0019304	4.61215	4.067637	5.27515	2.133098	1.77337	2.85705
AT5G08805	6.393706	1.879268077	0.579154	3.244853	1.18E-03	6.97E-03	3.791321	3.75549	3.525217	0	0	1.806583
AT3G29000	66.07812	1.879407213	0.365496	5.142079	2.72E-07	3.57E-06	6.742991	6.6743	6.873611	5.545934	3.045364	4.391288
AT1G24147	23.91594	1.87946131	0.45558	4.125427	3.70E-05	3.25E-04	5.599069	5.117315	5.342665	4.021447	0	3.08651
AT1G35230	9.536555	1.880072077	0.552866	3.400594	6.72E-04	0.0042969	4.104018	3.812433	4.501109	2.604042	0	1.169325
AT5G24640	124.7014	1.881826822	0.548284	3.432209	5.99E-04	3.89E-03	7.701824	7.746067	7.863621	6.138016	3.045364	4.302755
AT5G27940	6.7614	1.8844554	0.581922	3.238328	1.20E-03	7.11E-03	3.22918	4.243584	3.67243	0	0	1.806583
AT3G46090	587.4069	1.885287438	0.592327	3.182851	1.46E-03	8.39E-03	9.986779	10.27756	10.0654	7.179211	1.77337	5.773726
AT3G03435	617.2252	1.887071794	0.212728	8.870835	7.26E-19	3.37E-17	9.883938	10.17	9.741938	7.945604	7.452309	8.385863
AT2G30540	30.92895	1.889026299	0.396125	4.768758	1.85E-06	2.11E-05	5.615596	5.791094	5.709543	4.164539	1.77337	3.753887
AT3G57770	1013.657	1.88975406	0.206932	9.132236	6.71E-20	3.35E-18	10.34311	10.82262	10.75459	8.207663	8.745094	9.037841
AT2G44220	5.04466	1.889895873	0.591545	3.194845	1.40E-03	8.11E-03	2.719161	2.559128	4.377786	0	0	0
AT4G08985	152.2975	1.891789587	0.59239	3.193488	1.41E-03	0.0081424	8.11319	8.415197	7.977737	5.319969	0	3.08651
AT1G66870	4.120496	1.893087845	0.59222	3.196596	1.39E-03	0.0080643	2.719161	3.356609	3.445564	0	0	0
AT5G05995	64.07968	1.895740925	0.378913	5.003101	5.64E-07	7.03E-06	6.765383	6.763641	6.660846	5.492674	2.545235	4.391288
AT5G52720	12.11721	1.898243025	0.550236	3.44987	0.000561	0.0036802	4.509766	4.362754	4.577832	3.242825	0	0
AT2G43960	5.925744	1.900252738	0.589182	3.225239	1.26E-03	7.40E-03	3.046206	4.243584	3.271714	1.429371	0	0
AT4G33050	583.238	1.903043063	0.24699	7.704951	1.31E-14	4.23E-13	9.840657	9.941794	9.817052	8.491765	7.26084	7.566881
AT5G45650	83.19698	1.905839192	0.346885	5.49415	3.93E-08	5.83E-07	6.757957	7.091564	7.370693	5.741253	3.710678	4.831847
AT5G40040	13.07611	1.906137123	0.559699	3.405646	0.00066	0.004234	3.903255	4.89808	4.818167	3.242825	0	0
AT5G04085	604.331	1.909565639	0.174918	10.91692	9.57E-28	7.98E-26	9.779631	9.746507	10.16823	7.777648	7.865285	8.183799
AT3G54530	14.36787	1.909702117	0.502927	3.797176	1.46E-04	1.11E-03	4.360866	4.924151	4.753486	2.604042	0	2.85705
AT5G51440	46.23488	1.911298991	0.343817	5.559053	2.71E-08	4.13E-07	6.424426	6.227787	6.069773	4.722432	3.955229	3.753887
AT2G43141	5.650412	1.912642702	0.587729	3.254294	1.14E-03	6.76E-03	3.046206	3.696207	3.806003	1.429371	0	0
AT4G25200	39.60309	1.913058997	0.421924	4.534127	5.78E-06	6.01E-05	6.027383	6.269058	5.885862	4.722432	0	3.881636
AT2G32640	65.80388	1.913510162	0.294867	6.489394	8.62E-11	1.87E-09	6.532821	6.888065	6.754717	4.626795	5.123469	4.474702
AT5G07725	1112.447	1.913806538	0.145766	13.12931	2.24E-39	3.55E-37	10.53941	10.99702	10.78979	8.807449	8.808725	8.912559
AT3G03385	17.78734	1.913885665	0.466493	4.102713	4.08E-05	3.55E-04	4.935458	4.639454	5.204319	2.604042	3.045364	2.584062

AT1G02450	29.468	1.914951268	0.425944	4.495782	6.93E-06	7.11E-05	5.664073	5.626188	5.675034	4.29471	0	3.458449
AT2G18193	1370.791	1.915348995	0.5871	3.262389	1.10E-03	0.0065973	11.03143	11.50001	11.33548	8.902401	4.50885	7.284329
AT3G12860	40.04632	1.921016757	0.41696	4.607196	4.08E-06	4.37E-05	5.615596	6.50326	6.001784	3.242825	3.045364	4.62832
AT2G46192	3293.961	1.9223482	0.1352	14.21856	7.03E-46	1.41E-43	12.39618	12.1188	12.5185	10.40114	10.25829	10.53856
AT3G03445	608.3681	1.922368298	0.219712	8.749496	2.14E-18	9.56E-17	9.869368	10.16249	9.72517	7.874516	7.370331	8.358672
AT4G26200	160.8186	1.922605453	0.59236	3.245673	1.17E-03	6.95E-03	8.331507	8.280411	8.157219	5.379864	0	3.284433
AT5G53250	29.08671	1.925061467	0.490958	3.921033	8.82E-05	0.000713	5.345282	4.541804	6.452518	2.604042	3.416038	3.613718
AT1G06227	347.8828	1.926718582	0.205287	9.3855	6.26E-21	3.38E-19	8.929095	9.456156	8.904006	7.308122	7.142352	6.961824
AT4G13420	7.460576	1.929564239	0.574948	3.356068	7.91E-04	4.94E-03	4.194811	3.970903	3.271714	1.429371	1.77337	0
AT1G16060	13.29575	1.93475968	0.509014	3.800998	1.44E-04	1.09E-03	4.437236	4.472827	4.849451	2.604042	0	2.584062
AT5G10140	8.250489	1.937231095	0.560114	3.458634	5.43E-04	3.58E-03	4.194811	3.812433	3.928251	0	1.77337	1.806583
AT5G55770	4.989648	1.938867472	0.59211	3.274508	0.001058	0.0063488	3.466342	2.264856	4.094152	0	0	0
AT1G43605	7.903109	1.940914646	0.573983	3.381483	7.21E-04	0.0045574	4.194811	3.91999	3.6007	0	2.545235	0
AT5G06845	30.75992	1.941226991	0.379258	5.118482	3.08E-07	4.00E-06	5.599069	5.733286	5.743245	3.480233	3.416038	3.753887
AT5G60800	85.1155	1.942435421	0.308296	6.300562	2.97E-10	5.97E-09	6.641729	7.433099	7.162264	5.319969	5.123469	4.699401
AT1G04263	929.475	1.942911208	0.200435	9.693452	3.21E-22	1.93E-20	10.30858	10.25463	10.94945	8.371524	8.525969	8.699362
AT1G19210	8.526611	1.94327623	0.559237	3.474869	5.11E-04	0.0033884	4.280226	4.158291	3.525217	1.429371	1.77337	1.169325
AT1G07135	108.0836	1.943358594	0.359642	5.403594	6.53E-08	9.43E-07	7.047071	7.972192	7.246933	6.030007	4.164273	5.168875
AT2G39320	10.49285	1.94339169	0.557096	3.488434	4.86E-04	3.24E-03	3.537458	4.437061	4.720024	2.604042	0	1.169325
AT1G50400	14.94055	1.945643773	0.506222	3.843462	0.000121	0.0009409	4.280226	4.844481	5.051286	2.604042	0	2.85705
AT4G11070	62.96743	1.94726779	0.324068	6.008832	1.87E-09	3.39E-08	6.787432	6.927264	6.324778	4.414102	4.164273	5.01018
AT1G61470	64.87632	1.94727234	0.446277	4.363376	1.28E-05	1.25E-04	6.830546	6.813311	6.695692	5.597298	1.77337	3.753887
AT3G48650	387.3171	1.948840662	0.575716	3.385074	7.12E-04	4.51E-03	9.537671	9.552275	9.254475	7.308122	3.416038	5.806294
AT1G74010	15.00356	1.949347328	0.534506	3.647008	0.000265	0.0018932	4.826091	5.32726	3.806003	2.958503	0	2.247075
AT3G09450	7.301432	1.951085896	0.578149	3.374714	7.39E-04	0.0046583	3.466342	3.431225	4.461161	1.429371	0	1.169325
AT4G14368	19.32455	1.954244344	0.524744	3.724188	1.96E-04	0.0014456	4.797403	5.493187	4.968217	3.862584	0	1.169325
AT3G04165	453.647	1.955637352	0.197034	9.925358	3.23E-23	2.09E-21	9.226756	9.753794	9.484855	7.535811	7.772588	7.176347
AT2G15030	10.29293	1.95843124	0.552668	3.543592	3.95E-04	2.69E-03	4.676581	4.284411	3.740762	2.604042	0	1.169325
AT3G26470	71.51331	1.964744882	0.312056	6.296132	3.05E-10	6.13E-09	6.532821	6.874758	7.16848	5.123743	4.164273	4.831847
AT5G53700	7.306558	1.96554558	0.574202	3.42309	0.000619	0.0040034	3.848373	3.970903	3.740762	2.133098	0	0
AT4G23280	14.98503	1.966413458	0.519722	3.783591	1.55E-04	1.17E-03	4.676581	4.760154	4.849451	3.480233	0	1.169325
AT4G09100	13.436	1.968302896	0.529426	3.717806	2.01E-04	1.48E-03	4.768132	4.760154	4.289296	3.242825	0	1.169325
AT5G01050	6.062977	1.968544706	0.585028	3.364874	7.66E-04	4.80E-03	3.312641	3.634384	3.868421	0	1.77337	0
AT2G25297	11.12916	1.971653702	0.530118	3.719273	0.0002	0.0014698	4.509766	4.541804	3.985697	1.429371	1.77337	2.247075

AT2G32130	7.159561	1.973798598	0.575276	3.431047	6.01E-04	3.90E-03	3.312641	4.158291	3.928251	1.429371	0	1.169325
AT1G12740	12.24876	1.974445963	0.553044	3.570142	0.000357	0.0024619	3.466342	4.541804	5.129831	2.133098	0	2.247075
AT1G73550	25.29369	1.974791619	0.458973	4.302632	1.69E-05	0.00016	5.345282	4.924151	5.915727	3.862584	2.545235	2.584062
AT5G59090	18.44937	1.976210196	0.525051	3.763842	1.67E-04	1.25E-03	5.648094	5.023964	4.040944	3.242825	2.545235	1.169325
AT2G08370	6.264196	1.976398534	0.585528	3.375413	7.37E-04	4.65E-03	3.140592	3.75549	3.985697	0	1.77337	0
AT3G52430	122.4707	1.977362081	0.305631	6.469777	9.81E-11	2.11E-09	7.614069	7.596027	7.748022	6.205775	4.164273	5.482759
AT2G36440	17.1779	1.978319453	0.504811	3.91893	8.89E-05	7.18E-04	4.987182	5.384715	4.289296	2.958503	0	2.85705
AT3G08565	892.3691	1.979308209	0.206307	9.593989	8.47E-22	4.89E-20	10.25024	10.21161	10.89988	8.248399	8.386963	8.668693
AT2G08960	18.44537	1.980810304	0.489744	4.044584	5.24E-05	0.0004438	4.644725	5.475665	4.880072	3.242825	0	2.85705
AT2G32180	123.174	1.981409689	0.234205	8.460155	2.67E-17	1.09E-15	7.650616	7.521138	7.772711	5.597298	5.123469	5.869303
AT3G20200	11.15898	1.986765586	0.533826	3.721749	1.98E-04	1.46E-03	4.056385	4.701066	4.33422	2.133098	0	2.247075
AT5G10250	32.3457	1.98945362	0.383153	5.192323	2.08E-07	2.78E-06	6.002463	5.577738	5.692391	3.480233	3.710678	3.613718
AT1G22220	11.3373	1.989700942	0.546997	3.637501	0.000275	0.0019568	3.791321	4.575094	4.650679	2.133098	2.545235	0
AT4G31000	143.1547	1.989802513	0.245292	8.111972	4.98E-16	1.83E-14	7.485091	8.089149	7.949141	5.872138	5.69457	5.806294
AT1G61490	102.8149	1.990883314	0.246491	8.076891	6.64E-16	2.40E-14	7.411277	7.368109	7.392097	5.741253	4.907958	5.168875
AT3G04210	287.2176	1.994886608	0.280565	7.110245	1.16E-12	3.13E-11	8.900717	8.932865	8.779595	7.46844	5.997325	6.360876
AT2G29110	17.33848	1.995785853	0.540958	3.689352	2.25E-04	1.64E-03	4.738256	5.117315	5.077945	3.684038	0	0
AT3G02832	193.6654	1.996491825	0.221394	9.017816	1.92E-19	9.41E-18	8.11319	8.510769	8.24512	5.953231	6.050936	6.564985
AT1G51270	46.93737	1.996642343	0.407793	4.89622	9.77E-07	1.17E-05	6.337426	6.24857	6.324778	4.97633	3.710678	2.584062
AT5G66562	13.00375	1.99845193	0.512474	3.899617	9.63E-05	0.0007665	4.321109	4.844481	4.461161	1.429371	2.545235	2.247075
AT5G40400	95.64877	2.001205579	0.294607	6.79279	1.10E-11	2.65E-10	7.272254	6.933695	7.622346	5.123743	4.654502	5.522505
AT5G62970	7.532032	2.00601505	0.57582	3.483755	0.000494	0.0032889	3.22918	4.324114	3.985697	1.429371	0	1.169325
AT2G07615	15.21989	2.006682007	0.501248	4.003372	6.24E-05	0.0005213	4.578823	5.139739	4.461161	2.133098	3.045364	1.806583
AT1G16130	127.8893	2.006888498	0.294527	6.813933	9.50E-12	2.31E-10	7.542402	7.865238	7.701631	6.205775	5.476813	4.767144
AT3G24612	30.32519	2.007069195	0.430421	4.663032	3.12E-06	3.43E-05	5.771226	6.107802	5.051286	3.242825	3.045364	3.753887
AT3G09160	11.14046	2.007302859	0.528739	3.796394	0.000147	0.0011122	4.23815	4.362754	4.53998	2.133098	0	2.247075
AT2G09695	2930.148	2.007882047	0.149242	13.45386	2.92E-41	4.97E-39	12.2694	11.92444	12.37957	10.09431	10.02035	10.3471
AT1G05853	878.2986	2.009170229	0.207564	9.679757	3.68E-22	2.19E-20	10.23494	10.19699	10.88061	8.165743	8.365965	8.623718
AT3G27540	76.8625	2.010563263	0.300602	6.68846	2.26E-11	5.24E-10	6.823449	6.881427	7.241048	5.25748	3.955229	4.953151
AT1G16160	8.891416	2.016763125	0.559463	3.604821	3.12E-04	0.0021901	3.956125	4.472827	3.806003	2.133098	0	1.169325
AT1G01560	97.58553	2.020801479	0.381193	5.301257	1.15E-07	1.60E-06	7.406536	7.353794	7.31014	5.913254	2.545235	5.065041
AT1G72940	106.1704	2.021160544	0.295542	6.838818	7.98E-12	1.97E-10	7.444036	6.946471	7.820856	5.492674	5.310931	5.117892
AT3G57450	148.0783	2.021892786	0.288919	6.998122	2.59E-12	6.72E-11	8.208881	7.65948	7.875068	6.238498	4.654502	5.929675
AT3G59320	41.62682	2.02355613	0.348154	5.812239	6.16E-09	1.02E-07	6.190717	6.119132	6.056429	4.414102	3.710678	3.613718

AT5G01775	340.8763	2.024936622	0.242421	8.352959	6.66E-17	2.61E-15	9.021916	9.402689	8.889029	6.704469	6.574739	7.498771
AT1G80570	39.57356	2.025244726	0.372516	5.43667	5.43E-08	7.92E-07	6.317359	5.718464	6.10908	3.684038	3.416038	4.208431
AT3G29798	8.666104	2.026468602	0.559232	3.623665	0.00029	0.0020516	3.791321	4.400386	3.985697	1.429371	0	1.806583
AT3G03670	9.733852	2.029278718	0.563396	3.60187	3.16E-04	2.21E-03	4.544707	3.812433	4.242928	2.604042	0	0
AT4G04410	53.55164	2.030081839	0.390762	5.19519	2.05E-07	2.74E-06	7.010043	6.061572	6.244682	3.242825	4.346824	4.699401
AT2G32179	126.7914	2.031385296	0.232625	8.732452	2.49E-18	1.11E-16	7.709544	7.571493	7.816905	5.597298	5.123469	5.869303
AT1G19450	184.1556	2.03258184	0.229568	8.853944	8.45E-19	3.90E-17	7.879986	8.377954	8.387796	6.030007	5.941644	6.338783
AT4G35590	6.314861	2.033054403	0.583156	3.486295	4.90E-04	3.26E-03	3.466342	3.812433	3.740762	0	1.77337	0
AT1G60840	5.906139	2.033661901	0.585514	3.473292	0.000514	0.0034057	3.903255	3.502171	3.445564	0	0	1.169325
AT5G37500	225.9278	2.035056407	0.262286	7.758922	8.57E-15	2.81E-13	8.321467	8.394366	8.833395	6.859769	5.553109	6.425197
AT5G27330	15.65224	2.036310048	0.525801	3.87278	0.000108	0.0008433	4.399556	5.475665	4.377786	2.958503	0	2.247075
AT4G21490	13.92922	2.036561393	0.526817	3.865788	0.000111	0.0008664	4.056385	4.437061	5.27515	2.133098	1.77337	2.247075
AT1G07180	267.156	2.0390145	0.206373	9.880226	5.07E-23	3.24E-21	8.568549	8.84689	8.860521	7.074357	6.336481	6.466537
AT5G02865	35.6759	2.041493783	0.369027	5.532099	3.16E-08	4.77E-07	5.843167	5.846675	6.056429	3.242825	3.710678	3.998987
AT3G59710	55.0123	2.041739179	0.340957	5.988256	2.12E-09	3.82E-08	6.297009	6.627465	6.61607	4.97633	3.955229	3.753887
AT1G30350	6.12827	2.041772665	0.58413	3.495406	0.000473	0.0031666	3.466342	3.634384	3.868421	1.429371	0	0
AT4G08115	6.545533	2.043782626	0.592227	3.451011	5.58E-04	0.0036694	3.605233	4.701066	2.032847	0	0	0
AT2G04039	206.7306	2.046215073	0.196018	10.4389	1.65E-25	1.23E-23	8.31642	8.252389	8.588868	6.270495	6.379024	6.270397
AT2G39200	52.93685	2.050181723	0.356626	5.748827	8.99E-09	1.46E-07	6.179644	6.658856	6.579222	4.722432	2.545235	4.474702
AT3G53150	51.51546	2.050675056	0.470806	4.355669	1.33E-05	0.0001288	6.488642	6.914316	5.855366	5.123743	1.77337	3.08651
AT4G03925	446.3859	2.053097574	0.232281	8.838836	9.67E-19	4.45E-17	9.339251	9.484904	9.694272	7.127736	6.841348	7.889188
AT3G45851	9.72771	2.053727138	0.564193	3.640114	2.73E-04	1.94E-03	3.391538	4.400386	4.650679	2.133098	0	1.169325
AT5G19240	94.97208	2.05702462	0.303462	6.778528	1.21E-11	2.90E-10	7.489581	6.813311	7.504459	5.437371	4.50885	5.168875
AT3G51570	7.905431	2.06257614	0.567441	3.634872	0.000278	0.0019731	3.848373	4.113676	3.868421	1.429371	1.77337	0
AT1G35210	45.28888	2.063775075	0.339975	6.070365	1.28E-09	2.36E-08	6.286726	6.084872	6.368627	4.414102	3.710678	3.881636
AT2G18180	12.23637	2.06413544	0.532664	3.87512	0.000107	0.0008364	4.007126	4.788814	4.650679	2.604042	0	1.806583
AT1G61270	7.571831	2.066413304	0.586313	3.524417	4.24E-04	2.87E-03	2.836608	4.020081	4.501109	0	1.77337	0
AT4G00700	87.92577	2.067834406	0.422353	4.895987	9.78E-07	1.17E-05	6.80915	7.212852	7.562248	5.872138	3.045364	4.107507
AT1G73805	272.039	2.068028584	0.296582	6.972862	3.11E-12	7.96E-11	8.764694	8.557322	9.070903	7.26112	5.553109	6.466537
AT5G58080	7.734557	2.068442641	0.572061	3.615775	2.99E-04	2.11E-03	3.605233	3.75549	4.377786	1.429371	0	1.169325
AT2G04050	650.3254	2.06977117	0.588612	3.516358	4.38E-04	2.95E-03	9.848544	10.17	10.63905	7.482168	3.045364	5.706299
AT4G00970	73.27848	2.070173556	0.288304	7.18053	6.94E-13	1.92E-11	6.899657	6.806319	7.06567	5.19216	4.346824	4.553554
AT2G35820	258.8268	2.070846629	0.211429	9.794539	1.19E-22	7.38E-21	8.870049	8.588223	8.706729	7.03764	6.200761	6.382637
AT3G02590	6.577902	2.072053757	0.58469	3.543849	0.000394	0.0026908	3.466342	3.431225	4.242928	1.429371	0	0

AT1G77640	68.87447	2.072534214	0.319338	6.490096	8.58E-11	1.86E-09	6.851629	6.414087	7.16848	4.626795	4.654502	4.62832
AT1G10470	248.8868	2.074465503	0.224311	9.248162	2.28E-20	1.18E-18	8.538527	8.624451	8.848958	6.880651	5.760417	6.639175
AT3G00800	46.18051	2.076475272	0.352867	5.884581	3.99E-09	6.82E-08	6.087861	6.130373	6.550955	4.021447	4.50885	3.613718
AT1G17420	87.9876	2.077216866	0.304855	6.81379	9.51E-12	2.31E-10	6.952657	7.142421	7.459545	5.492674	3.955229	5.01018
AT1G08123	26.23733	2.079300148	0.40997	5.071835	3.94E-07	5.03E-06	5.495717	5.610218	5.407161	2.958503	3.045364	3.458449
AT3G05015	15.47671	2.081028118	0.49985	4.163303	3.14E-05	0.00028	4.437236	4.844481	5.077945	2.958503	1.77337	1.806583
AT1G14540	107.014	2.08453348	0.578415	3.603869	3.14E-04	0.0021975	7.982303	7.512572	7.326908	4.414102	0	4.893774
AT4G26470	24.5449	2.086019484	0.437368	4.769488	1.85E-06	2.11E-05	4.987182	5.594069	5.584968	2.604042	3.416038	3.08651
AT3G21150	11.29223	2.092590687	0.545586	3.835491	1.25E-04	9.68E-04	4.104018	4.201568	4.849451	2.604042	0	1.169325
AT3G25180	14.05058	2.093013059	0.547495	3.822892	1.32E-04	1.01E-03	4.056385	5.365817	4.420076	2.958503	0	1.169325
AT4G26890	21.92465	2.093887291	0.505338	4.143539	3.42E-05	0.0003018	4.644725	4.760154	6.056429	3.242825	1.77337	2.584062
AT3G48390	98.08009	2.094762518	0.289992	7.223523	5.07E-13	1.41E-11	7.724859	7.142421	7.072314	4.97633	5.396255	5.065041
AT1G17170	74.52834	2.096073292	0.340229	6.160778	7.24E-10	1.38E-08	6.405541	7.31491	7.058994	4.812122	3.955229	5.01018
AT5G54035	5.904978	2.099661471	0.592339	3.544699	0.000393	0.0026833	3.046206	2.911631	4.501109	0	0	0
AT5G02315	24.96079	2.100679162	0.489131	4.294719	1.75E-05	1.65E-04	5.477749	6.061572	4.461161	2.133098	3.045364	3.284433
AT4G27280	759.8368	2.101782488	0.186177	11.28918	1.48E-29	1.37E-27	10.32563	9.956869	10.49996	8.386201	8.086794	7.866203
AT3G09545	17.51439	2.101977614	0.504127	4.169536	3.05E-05	2.73E-04	4.399556	5.267421	5.179914	3.242825	0	2.247075
AT5G00760	15.81949	2.102243847	0.489218	4.297154	1.73E-05	1.64E-04	4.987182	4.670589	4.786189	2.133098	3.045364	1.806583
AT2G31690	9.75769	2.104548782	0.566186	3.717064	2.02E-04	1.48E-03	4.578823	3.569791	4.461161	0	0	2.247075
AT5G08860	44.87176	2.10509329	0.430217	4.8931	9.93E-07	1.18E-05	6.223435	6.881427	5.298008	4.164539	3.710678	3.613718
AT2G21640	85.14276	2.108867083	0.283628	7.435327	1.04E-13	3.11E-12	7.11258	7.091564	7.246933	5.492674	4.164273	4.767144
AT1G09125	40.58165	2.10931415	0.362262	5.822622	5.79E-09	9.69E-08	5.98984	6.206701	6.134702	4.414102	3.045364	3.613718
AT1G72910	99.64598	2.109987984	0.402722	5.239313	1.61E-07	2.20E-06	7.401779	7.31491	7.499537	6.030007	3.045364	4.391288
AT2G09685	45.91622	2.110165515	0.33128	6.36973	1.89E-10	3.91E-09	6.234178	6.299253	6.302344	3.862584	3.955229	4.208431
AT1G03660	34.72166	2.113971411	0.428781	4.930191	8.21E-07	9.98E-06	5.631937	5.762479	6.302344	3.862584	0	3.998987
AT3G22370	908.6313	2.113980421	0.212034	9.970027	2.06E-23	1.36E-21	10.41392	10.57362	10.63625	8.694056	7.638931	8.509687
AT1G33260	13.55494	2.115254221	0.507299	4.169636	3.05E-05	2.73E-04	4.676581	4.541804	4.720024	1.429371	1.77337	2.584062
AT2G27505	8.656174	2.116129866	0.572325	3.697428	2.18E-04	1.59E-03	4.280226	3.502171	4.377786	2.133098	0	0
AT3G04420	10.21005	2.116326786	0.562098	3.76505	1.67E-04	1.25E-03	3.669966	4.89808	4.094152	2.133098	0	1.169325
AT1G72900	361.9209	2.116452332	0.290108	7.295403	2.98E-13	8.43E-12	8.960164	9.417677	9.273341	7.697535	6.379024	6.425197
AT5G13210	496.249	2.116836302	0.566688	3.73545	1.87E-04	1.39E-03	9.545239	9.947366	9.938405	7.522586	3.955229	6.096967
AT3G60470	6.593823	2.123752466	0.582205	3.647775	2.65E-04	1.89E-03	3.466342	3.867214	3.928251	1.429371	0	0
AT5G61900	409.8549	2.133557819	0.207274	10.29341	7.55E-25	5.45E-23	9.211886	9.141702	9.760572	7.353641	7.013254	7.201051
AT1G09867	249.6025	2.138468962	0.204414	10.46147	1.30E-25	9.78E-24	8.668963	8.690496	8.715245	6.270495	6.050936	6.90281

AT4G03995	1151.271	2.145220923	0.14113	15.20028	3.52E-52	1.04E-49	10.8447	11.01039	10.78271	8.471261	8.687018	8.916294
AT5G14730	198.5842	2.14953884	0.249037	8.631394	6.06E-18	2.60E-16	8.283183	8.267741	8.544156	6.530411	5.123469	6.270397
AT3G55920	63.6196	2.152444037	0.306969	7.011916	2.35E-12	6.13E-11	6.600809	6.874758	6.704273	4.524365	4.907958	3.998987
AT3G28210	43.43654	2.153580424	0.457231	4.710054	2.48E-06	2.78E-05	6.099659	6.562555	6.042961	4.812122	0	3.08651
AT3G04775	226.8235	2.15514179	0.221395	9.734384	2.15E-22	1.31E-20	8.303727	8.855383	8.445238	6.503738	6.050936	6.360876
AT2G32030	34.22559	2.155179471	0.395864	5.444239	5.20E-08	7.62E-07	5.829063	5.641983	6.159877	4.021447	2.545235	3.458449
AT5G01785	303.0149	2.16024627	0.245554	8.797456	1.40E-18	6.34E-17	8.892263	9.278089	8.700308	6.332435	6.420349	7.188751
AT1G56510	430.9254	2.162870098	0.236177	9.157846	5.29E-20	2.67E-18	9.394169	9.415381	9.623646	7.777648	6.537663	7.073069
AT2G39110	32.263	2.165731906	0.463598	4.671574	2.99E-06	3.30E-05	5.441126	5.733286	6.208946	4.29471	2.545235	1.806583
AT1G06733	7.421707	2.166720255	0.581366	3.726949	1.94E-04	0.0014307	4.399556	3.569791	3.6007	0	1.77337	0
AT2G25735	114.863	2.167562483	0.278098	7.79424	6.48E-15	2.16E-13	7.717222	7.47779	7.53845	5.872138	5.220243	4.62832
AT1G72210	21.61994	2.167887139	0.462097	4.691411	2.71E-06	3.03E-05	5.012364	5.510498	5.298008	2.958503	0	3.284433
AT1G04217	65.96742	2.171202557	0.333403	6.51225	7.40E-11	1.62E-09	6.823449	6.57083	6.969278	5.051918	4.50885	3.613718
AT5G59680	8.642418	2.179637127	0.566782	3.845638	0.00012	0.0009339	3.956125	4.284411	4.040944	2.133098	0	0
AT2G38790	287.4532	2.18364587	0.197397	11.06218	1.91E-28	1.67E-26	8.878633	9.048485	8.748816	7.018926	6.247434	6.564985
AT3G02380	337.7907	2.184503822	0.179394	12.17711	4.12E-34	4.90E-32	9.328012	8.939271	9.084114	7.056115	6.747048	6.85691
AT1G65450	21.56366	2.190589252	0.467043	4.690342	2.73E-06	3.04E-05	4.908884	5.54451	5.298008	3.480233	1.77337	2.247075
AT2G09775	6.759219	2.193681236	0.581418	3.772987	1.61E-04	1.21E-03	3.605233	4.067637	3.740762	0	0	1.169325
AT1G14480	60.87902	2.195072135	0.399407	5.495824	3.89E-08	5.78E-07	6.11136	6.45042	7.32134	4.524365	3.045364	4.553554
AT4G37030	68.71002	2.196411865	0.413519	5.311514	1.09E-07	1.52E-06	6.657778	7.062516	6.93323	5.437371	2.545235	3.613718
AT1G32080	450.5222	2.198153801	0.190201	11.55701	6.80E-31	6.75E-29	9.203711	9.544943	9.816063	7.26112	7.349087	7.272722
AT3G50060	191.8083	2.201096602	0.253761	8.673899	4.18E-18	1.82E-16	8.214317	8.018304	8.661174	6.420628	5.82339	5.740407
AT3G55860	122.2122	2.203755474	0.265458	8.301716	1.03E-16	3.97E-15	7.507404	7.433099	8.019596	5.25748	5.476813	5.399825
AT1G72760	18.58295	2.205124744	0.494323	4.460901	8.16E-06	8.23E-05	4.399556	5.43997	5.228318	2.604042	1.77337	2.584062
AT2G21650	10.64186	2.206171194	0.573976	3.843662	1.21E-04	9.41E-04	5.177314	3.502171	3.928251	1.429371	1.77337	0
AT4G36000	22.31853	2.208416262	0.473393	4.665083	3.08E-06	3.39E-05	5.345282	4.670589	5.759806	2.958503	2.545235	2.584062
AT4G27140	15.22808	2.209344032	0.562047	3.930888	8.46E-05	0.0006858	4.23815	4.284411	5.621672	2.958503	0	0
AT4G08112	12.22762	2.209636592	0.552705	3.997862	6.39E-05	5.32E-04	4.280226	5.071394	4.195021	0	0	2.584062
AT3G09225	51.35566	2.209931161	0.383156	5.767704	8.04E-09	1.32E-07	6.223435	6.595375	6.550955	4.896561	3.416038	3.08651
AT5G01490	13.2911	2.21015667	0.521326	4.239487	2.24E-05	0.0002066	4.509766	4.924151	4.461161	2.604042	0	1.806583
AT4G23210	151.2758	2.21083976	0.250693	8.818923	1.16E-18	5.30E-17	7.841862	7.956488	8.147813	6.205775	5.019734	5.522505
AT4G38420	182.5146	2.212560149	0.314646	7.031899	2.04E-12	5.33E-11	7.969501	7.647793	8.879589	5.694846	6.152528	5.806294
AT3G63360	6.136739	2.2132608	0.591521	3.741647	1.83E-04	1.35E-03	3.731919	2.803425	4.289296	0	0	0
AT1G77870	17.70693	2.215799751	0.486627	4.553384	5.28E-06	5.52E-05	4.88181	5.307588	4.849451	2.604042	0	2.85705

AT3G45410	28.50959	2.217192735	0.447903	4.950163	7.42E-07	9.06E-06	5.530997	5.577738	5.855366	0	3.416038	3.753887
AT1G08173	6.159323	2.217339533	0.591482	3.748785	1.78E-04	1.32E-03	3.312641	3.10637	4.420076	0	0	0
AT2G04965	8366.71	2.217508867	0.178065	12.45336	1.34E-35	1.78E-33	13.64101	13.61197	13.99085	11.65054	11.68183	11.05708
AT1G08347	11.68135	2.218069755	0.534525	4.14961	3.33E-05	0.000295	4.644725	4.400386	4.377786	0	1.77337	2.247075
AT1G61275	2540.391	2.223570329	0.19127	11.62528	3.07E-31	3.14E-29	12.20959	11.98781	11.90858	9.951114	9.993215	9.221112
AT2G39650	74.56407	2.224402241	0.348708	6.378977	1.78E-10	3.70E-09	6.742991	7.068373	7.162264	5.25748	2.545235	4.62832
AT3G11370	8.51612	2.226432033	0.584577	3.80862	1.40E-04	0.0010665	3.903255	3.10637	4.849451	1.429371	0	0
AT1G04640	119.7805	2.226721724	0.249095	8.939237	3.92E-19	1.89E-17	7.642574	7.414827	7.867447	5.319969	5.220243	5.441888
AT1G51620	37.59717	2.227871146	0.396425	5.61991	1.91E-08	2.97E-07	5.98984	5.846675	6.2564	4.29471	1.77337	3.458449
AT1G06830	42.04055	2.228456025	0.354118	6.292972	3.11E-10	6.24E-09	6.002463	6.174482	6.335866	3.862584	3.710678	3.753887
AT5G06775	7.339427	2.2304767	0.582145	3.831478	1.27E-04	0.000982	3.312641	3.970903	4.33422	0	0	1.169325
AT2G46400	507.8709	2.231052345	0.580016	3.846538	1.20E-04	9.31E-04	9.809569	9.795878	10.0629	6.704469	2.545235	6.446015
AT1G20015	28.70881	2.231258314	0.431265	5.173753	2.29E-07	3.05E-06	5.441126	5.832979	5.743245	2.604042	1.77337	3.881636
AT1G08117	31.57814	2.23210804	0.396425	5.630597	1.80E-08	2.80E-07	5.615596	5.805191	5.900872	3.684038	3.416038	2.85705
AT3G09405	45.70626	2.232588373	0.404719	5.516393	3.46E-08	5.19E-07	6.201706	6.084872	6.597764	4.722432	3.045364	2.85705
AT1G12950	16.45798	2.233459871	0.513861	4.346425	1.38E-05	0.000134	4.61215	4.437061	5.488902	2.133098	2.545235	1.806583
AT2G05565	17.99596	2.239636646	0.483735	4.629881	3.66E-06	3.96E-05	5.154874	4.844481	5.129831	1.429371	1.77337	3.08651
AT5G56960	7.139863	2.24047287	0.578355	3.873872	1.07E-04	0.0008402	3.848373	3.91999	3.868421	1.429371	0	0
AT5G37420	5.854592	2.242233511	0.590827	3.795075	1.48E-04	1.12E-03	3.22918	4.158291	3.445564	0	0	0
AT2G25440	11.91158	2.24235974	0.562561	3.985982	6.72E-05	0.0005576	3.391538	4.87153	4.910056	2.133098	0	1.169325
AT1G09087	39.58542	2.250419576	0.386603	5.821015	5.85E-09	9.77E-08	5.800434	6.001621	6.452518	3.242825	3.416038	3.998987
AT4G12720	690.3344	2.255149675	0.164991	13.66831	1.57E-42	2.90E-40	10.11292	9.962383	10.39321	8.03214	7.638931	7.889188
AT2G20142	296.0387	2.256740199	0.5759	3.918631	8.91E-05	7.18E-04	8.864873	8.831475	9.528999	6.530411	3.045364	4.391288
AT5G05640	22.67629	2.25878865	0.459899	4.911489	9.04E-07	1.09E-05	4.987182	5.688357	5.27515	3.242825	2.545235	2.247075
AT3G27865	183.6165	2.259493279	0.201145	11.23314	2.80E-29	2.54E-27	8.18693	8.396696	8.188139	6.030007	6.050936	5.806294
AT3G46890	9.301742	2.260339608	0.564055	4.007305	6.14E-05	5.14E-04	3.903255	3.970903	4.614715	0	1.77337	1.169325
AT3G00700	13.63505	2.26781724	0.514909	4.404307	1.06E-05	1.05E-04	4.644725	4.760154	4.650679	2.604042	0	1.806583
AT3G29340	6.310453	2.268850752	0.590838	3.840054	0.000123	0.0009512	3.848373	2.911631	4.242928	0	0	0
AT2G40330	11.63906	2.269527924	0.554014	4.096516	4.19E-05	3.64E-04	4.88181	4.362754	4.145468	2.604042	0	0
AT2G30766	61.95146	2.277267696	0.344806	6.604488	3.99E-11	9.01E-10	6.9264	6.868059	6.390061	4.021447	3.416038	4.831847
AT2G04495	36.67388	2.278346035	0.464975	4.899929	9.59E-07	1.15E-05	6.075967	6.395572	5.488902	4.414102	1.77337	2.247075
AT2G03760	793.0284	2.280313175	0.244153	9.339678	9.66E-21	5.11E-19	10.12812	10.46626	10.51832	8.564547	7.349087	7.794964
AT5G44140	13.68232	2.282871151	0.544086	4.19579	2.72E-05	2.46E-04	4.056385	4.324114	5.385981	1.429371	1.77337	1.806583
AT2G07754	48.1959	2.283739395	0.334587	6.825544	8.76E-12	2.14E-10	6.470584	6.25885	6.390061	3.862584	3.955229	3.998987

AT5G14490	9.206347	2.284938331	0.564627	4.046813	5.19E-05	4.40E-04	4.509766	3.696207	4.289296	1.429371	0	1.169325
AT1G07393	18.50996	2.287851656	0.494877	4.623068	3.78E-06	4.08E-05	4.908884	5.510498	4.685768	1.429371	3.045364	2.247075
AT1G07487	14.20259	2.29021427	0.513234	4.46232	8.11E-06	8.18E-05	4.644725	4.788814	4.753486	0	2.545235	2.247075
AT5G59860	14.00246	2.304260756	0.513358	4.488607	7.17E-06	7.33E-05	4.707748	4.816916	4.650679	2.604042	0	1.806583
AT4G20000	26.14762	2.304596081	0.437664	5.265672	1.40E-07	1.92E-06	5.513465	5.747956	5.364486	3.684038	1.77337	2.584062
AT2G35570	25.20998	2.305433522	0.508833	4.530822	5.88E-06	6.09E-05	4.707748	5.54451	6.042961	3.684038	1.77337	1.169325
AT1G05880	142.4585	2.310520084	0.588482	3.926239	8.63E-05	6.99E-04	8.101503	8.009199	8.182008	4.896561	0	2.85705
AT1G06453	3653.028	2.313499603	0.10148	22.7976	4.84E-115	8.89E-112	12.47543	12.60914	12.6315	10.11868	10.27529	10.3276
AT2G20150	7.857955	2.314627437	0.591389	3.913883	9.08E-05	0.0007295	2.450992	4.243584	4.685768	0	0	0
AT3G59330	31.8754	2.317159449	0.413866	5.598815	2.16E-08	3.33E-07	6.002463	5.641983	5.839872	2.958503	1.77337	3.881636
AT4G13577	121.7773	2.318113852	0.239756	9.66862	4.10E-22	2.43E-20	7.806308	7.538119	7.710177	5.545934	5.123469	5.168875
AT1G16120	13.52575	2.324068241	0.537176	4.326457	1.52E-05	1.45E-04	4.321109	4.472827	5.15509	2.604042	0	1.169325
AT4G04540	56.40371	2.329983024	0.337424	6.905213	5.01E-12	1.26E-10	6.757957	6.395572	6.669636	4.626795	3.045364	4.107507
AT1G22830	96.19372	2.331089606	0.30392	7.670072	1.72E-14	5.48E-13	6.991167	7.295068	7.718673	5.25748	4.50885	4.767144
AT1G76470	7.533967	2.334853569	0.590891	3.951413	7.77E-05	6.35E-04	3.466342	4.844481	3.074005	0	0	0
AT4G29780	408.6392	2.339368211	0.434333	5.386119	7.20E-08	1.03E-06	9.378518	9.568896	9.493545	7.535811	5.82339	6.123056
AT4G06130	56.13496	2.343135771	0.331764	7.062656	1.63E-12	4.31E-11	6.794708	6.619509	6.390061	4.164539	3.416038	4.391288
AT3G01165	25.18762	2.344838299	0.485144	4.833281	1.34E-06	1.57E-05	5.870968	5.641983	4.910056	3.684038	0	2.247075
AT1G09855	21.69213	2.347571356	0.456401	5.143656	2.69E-07	3.55E-06	5.284563	5.493187	5.077945	2.958503	2.545235	2.247075
AT2G09885	42.0454	2.35017223	0.368887	6.370988	1.88E-10	3.88E-09	6.376742	5.926227	6.268024	3.242825	3.710678	3.881636
AT3G44260	358.5008	2.360376965	0.203639	11.59101	4.58E-31	4.63E-29	9.261304	8.997215	9.438031	6.362435	6.985988	7.018519
AT1G72950	44.65817	2.363063459	0.396018	5.967056	2.42E-09	4.29E-08	6.286726	6.404859	6.196834	4.626795	2.545235	2.85705
AT5G25260	271.4282	2.363876836	0.572701	4.127596	3.67E-05	0.000322	8.799469	9.012485	9.151556	5.953231	1.77337	5.265734
AT4G04710	6.100399	2.364476566	0.588398	4.018496	5.86E-05	4.92E-04	3.669966	3.75549	3.740762	0	0	0
AT2G18720	14.93371	2.364664954	0.515324	4.588694	4.46E-06	4.73E-05	4.544707	5.117315	4.753486	2.604042	0	1.806583
AT2G26480	17.58778	2.366070204	0.511935	4.621821	3.80E-06	4.11E-05	4.280226	5.139739	5.468897	2.133098	1.77337	2.247075
AT1G35513	6.235753	2.36644774	0.588581	4.020596	5.81E-05	4.88E-04	3.848373	3.431225	3.928251	0	0	0
AT1G76420	12.79231	2.367357584	0.551471	4.292808	1.76E-05	0.0001664	4.321109	3.970903	5.27515	1.429371	1.77337	1.169325
AT3G61340	19.39448	2.371545472	0.50429	4.702745	2.57E-06	2.87E-05	4.578823	5.641983	5.129831	2.958503	0	2.247075
AT5G18270	146.2777	2.374108988	0.556529	4.265918	1.99E-05	1.85E-04	7.678415	8.215918	8.291418	5.646896	2.545235	3.613718
AT5G03195	118.8794	2.374195784	0.280578	8.461803	2.63E-17	1.07E-15	7.601678	7.34899	7.998818	4.97633	4.907958	5.482759
AT1G05767	45.69662	2.378118517	0.442572	5.373403	7.73E-08	1.10E-06	6.470584	6.404859	6.172301	4.722432	0	2.85705
AT1G65483	6.35634	2.379183524	0.588442	4.043189	5.27E-05	0.0004461	3.669966	3.502171	4.094152	0	0	0
AT1G26761	62.10245	2.381751609	0.327312	7.276692	3.42E-13	9.63E-12	6.641729	7.026869	6.550955	4.524365	3.710678	4.208431

AT5G54820	6.515863	2.383535418	0.588635	4.049262	5.14E-05	0.0004368	4.194811	3.75549	3.361256	0	0	0
AT2G25770	68.61318	2.38367832	0.384431	6.200535	5.63E-10	1.09E-08	6.286726	6.834086	7.392097	4.896561	3.955229	3.613718
AT5G64120	168.5296	2.38663649	0.244514	9.760748	1.66E-22	1.02E-20	7.99183	7.946983	8.493071	5.545934	5.82339	5.706299
AT1G06135	13.23545	2.386701709	0.536589	4.447911	8.67E-06	8.70E-05	4.826091	4.788814	4.420076	0	0	2.584062
AT1G72960	13.22661	2.390576801	0.526883	4.537202	5.70E-06	5.92E-05	4.360866	4.760154	4.849451	2.133098	0	1.806583
AT1G08937	141.7864	2.390604236	0.252567	9.465246	2.93E-21	1.61E-19	7.866239	7.716535	8.157219	5.051918	5.310931	5.773726
AT3G28160	254.5848	2.391159267	0.216675	11.03568	2.57E-28	2.22E-26	8.397507	8.821106	8.990744	6.270495	6.292644	6.316346
AT4G04195	36.28296	2.395213865	0.413396	5.793994	6.87E-09	1.14E-07	5.938213	5.776858	6.302344	2.133098	3.045364	3.998987
AT4G04185	36.28296	2.395213865	0.413396	5.793994	6.87E-09	1.14E-07	5.938213	5.776858	6.302344	2.133098	3.045364	3.998987
AT1G58420	30.93921	2.397871677	0.496362	4.830892	1.36E-06	1.59E-05	6.087861	5.733286	5.621672	4.164539	0	1.169325
AT1G52315	40.91341	2.398599848	0.403057	5.951025	2.66E-09	4.70E-08	5.898243	6.611509	5.930431	4.021447	2.545235	3.458449
AT4G06235	90.49235	2.400400958	0.298988	8.028419	9.87E-16	3.53E-14	7.022492	7.468962	7.354429	5.19216	3.710678	4.831847
AT2G09705	327.9174	2.407798734	0.182874	13.16646	1.37E-39	2.20E-37	8.942258	9.119309	9.280532	6.391825	6.747048	6.809501
AT1G29290	20.50596	2.408672525	0.464687	5.183432	2.18E-07	2.90E-06	5.221175	5.047874	5.407161	2.133098	2.545235	2.584062
AT4G39366	441.5255	2.413684266	0.161724	14.92471	2.28E-50	6.09E-48	9.447073	9.642388	9.55414	6.859769	7.013254	7.329843
AT2G35720	74.85398	2.415213831	0.28909	8.354528	6.57E-17	2.58E-15	6.978444	6.97169	7.105087	4.812122	3.955229	4.474702
AT4G09350	40.03175	2.418632177	0.406403	5.951309	2.66E-09	4.70E-08	5.615596	6.376816	6.346869	2.604042	3.710678	3.753887
AT2G34600	65.84543	2.423136495	0.41833	5.792408	6.94E-09	1.15E-07	6.30722	6.511881	7.494598	4.896561	3.045364	3.613718
AT5G64870	348.4073	2.423460202	0.510696	4.745411	2.08E-06	2.36E-05	9.189981	9.109402	9.565959	7.074357	4.786789	5.31183
AT4G11730	8.906973	2.425901493	0.575472	4.215499	2.49E-05	2.28E-04	3.903255	3.812433	4.685768	1.429371	0	0
AT5G07105	810.5576	2.432181799	0.150418	16.16944	8.29E-59	3.13E-56	10.44892	10.60317	10.19207	7.895187	8.007659	7.970467
AT2G04450	48.06637	2.432352382	0.490738	4.956522	7.18E-07	8.80E-06	5.771226	6.32883	6.997482	4.626795	1.77337	1.806583
AT3G10815	98.66404	2.433479707	0.31806	7.651001	1.99E-14	6.34E-13	7.124178	7.37285	7.705911	5.492674	4.164273	4.391288
AT5G11140	27.41992	2.45016924	0.488928	5.011305	5.41E-07	6.77E-06	5.870968	6.04978	4.786189	3.480233	0	2.584062
AT1G12013	1278.11	2.451847839	0.189447	12.94212	2.60E-38	3.89E-36	11.13466	10.77685	11.30829	8.429358	8.289966	8.901297
AT3G28170	21.826	2.451876156	0.492353	4.979916	6.36E-07	7.87E-06	5.154874	5.805191	4.849451	2.604042	3.045364	1.169325
AT4G00130	23.70858	2.452290707	0.453885	5.402888	6.56E-08	9.47E-07	5.284563	5.365817	5.621672	3.242825	2.545235	1.806583
AT5G33370	56.4465	2.454720454	0.388738	6.314594	2.71E-10	5.49E-09	5.938213	6.907798	6.8812	4.414102	3.710678	3.458449
AT2G20800	194.3997	2.469051433	0.591621	4.173364	3.00E-05	0.0002697	8.178611	8.504302	8.942597	4.524365	0	1.806583
AT1G05680	517.5495	2.470543366	0.54906	4.49959	6.81E-06	6.99E-05	9.342978	10.14251	10.10564	7.26112	4.164273	5.869303
AT1G31835	6452.115	2.470914926	0.100835	24.50462	1.32E-132	3.77E-129	13.3426	13.43821	13.47437	10.8064	10.90968	11.06046
AT4G01950	374.3866	2.473187584	0.201768	12.25756	1.53E-34	1.85E-32	9.130845	9.126344	9.649427	6.750572	6.747048	6.841279
AT3G60415	216.7402	2.474423885	0.312147	7.927117	2.24E-15	7.78E-14	8.288346	8.370863	8.922512	6.632428	5.123469	5.441888
AT3G20760	102.1309	2.474488981	0.259096	9.550471	1.29E-21	7.28E-20	7.358247	7.533893	7.474672	5.051918	5.123469	4.553554

AT3G24615	183.4501	2.478019144	0.245232	10.1048	5.26E-24	3.61E-22	7.975916	8.592294	8.28285	5.953231	5.396255	5.740407
AT1G26970	7.467822	2.479798702	0.587481	4.221072	2.43E-05	2.22E-04	3.391538	3.812433	4.53998	0	0	0
AT3G02840	122.8974	2.484371483	0.534332	4.649493	3.33E-06	3.65E-05	8.164639	7.924557	7.252795	5.379864	2.545235	3.753887
AT1G74870	49.66723	2.487317957	0.355815	6.99049	2.74E-12	7.07E-11	6.532821	6.595375	6.220956	4.29471	3.045364	3.613718
AT5G64850	99.46245	2.495255072	0.288593	8.64628	5.32E-18	2.30E-16	7.372904	7.191545	7.684385	5.19216	4.786789	4.391288
AT2G41730	275.9481	2.497051838	0.467335	5.343175	9.13E-08	1.29E-06	8.87692	8.75183	9.196349	6.607592	4.346824	5.806294
AT3G23250	181.0521	2.497329362	0.584795	4.270433	1.95E-05	0.0001824	8.414137	8.272822	8.638965	1.429371	2.545235	5.117892
AT5G52730	16.29186	2.497826629	0.559894	4.461253	8.15E-06	8.22E-05	4.056385	4.670589	5.743245	2.604042	0	0
AT5G09876	15.84029	2.500453758	0.504562	4.955693	7.21E-07	8.83E-06	4.85422	5.023964	4.880072	2.133098	0	2.247075
AT2G25460	71.05939	2.503553978	0.351542	7.121635	1.07E-12	2.89E-11	6.830546	7.212852	6.819344	5.051918	3.710678	3.458449
AT4G31800	218.3683	2.503573765	0.31002	8.075532	6.72E-16	2.43E-14	8.308818	8.486916	8.866268	6.530411	4.50885	5.869303
AT3G05335	90.37579	2.515795421	0.273371	9.202851	3.49E-20	1.78E-18	7.240636	7.433099	7.223245	4.626795	4.346824	4.893774
AT3G03595	120.6076	2.520843269	0.2497	10.09548	5.78E-24	3.94E-22	7.690167	7.705304	7.722903	4.414102	5.396255	5.31183
AT1G69930	43.88752	2.520914153	0.445414	5.659711	1.52E-08	2.39E-07	6.051879	6.545861	6.313604	4.524365	0	2.584062
AT1G33720	197.6012	2.523362541	0.332835	7.581417	3.42E-14	1.06E-12	8.192449	8.320223	8.734225	6.530411	4.654502	5.168875
AT4G39030	273.6344	2.525423793	0.236799	10.66485	1.49E-26	1.16E-24	8.606253	8.793086	9.194827	6.656843	5.82339	6.148682
AT3G01155	102.5507	2.526210962	0.278995	9.054675	1.37E-19	6.78E-18	7.169658	7.6517	7.562248	4.722432	5.123469	4.699401
AT1G42980	14.40198	2.527846059	0.541777	4.665845	3.07E-06	3.38E-05	4.23815	4.701066	5.27515	2.133098	1.77337	0
AT1G13470	56.46087	2.534625219	0.385354	6.577386	4.79E-11	1.07E-09	6.488642	6.7122	6.771147	4.722432	0	3.753887
AT4G22590	120.3371	2.535735923	0.379975	6.673424	2.50E-11	5.78E-10	7.694063	7.495286	7.981272	5.872138	3.045364	4.391288
AT2G27660	53.82416	2.535964241	0.401833	6.310989	2.77E-10	5.61E-09	6.67365	6.395572	6.704273	4.722432	0	3.458449
AT5G13320	812.5962	2.536724107	0.566298	4.47949	7.48E-06	7.62E-05	10.55502	10.46182	10.72062	7.412182	3.710678	6.583893
AT1G67855	44.64722	2.548890706	0.399133	6.386075	1.70E-10	3.54E-09	6.11136	6.073269	6.721284	4.021447	1.77337	3.613718
AT2G15020	17.31166	2.549701912	0.522747	4.877509	1.07E-06	1.27E-05	4.644725	5.204992	5.27515	0	0	2.85705
AT2G44070	26.30665	2.550744945	0.448014	5.693443	1.25E-08	1.98E-07	5.679876	5.762479	5.342665	3.242825	0	2.85705
AT4G11250	7.244885	2.556009438	0.58455	4.372612	1.23E-05	1.20E-04	4.104018	3.812433	3.928251	0	0	0
AT2G23270	40.56418	2.559245933	0.473754	5.402056	6.59E-08	9.50E-07	6.075967	6.185302	6.421627	1.429371	0	4.302755
AT4G38560	123.6681	2.561696699	0.300404	8.527493	1.50E-17	6.25E-16	7.630427	7.616158	7.995326	5.741253	4.50885	4.474702
AT5G56970	17.34139	2.564109355	0.507207	5.05535	4.30E-07	5.46E-06	4.85422	4.844481	5.385981	2.604042	0	1.806583
AT1G30370	62.9448	2.567707051	0.383956	6.687496	2.27E-11	5.27E-10	6.479641	6.820269	7.105087	4.524365	0	4.302755
AT1G08860	18.31202	2.567995031	0.512716	5.008606	5.48E-07	6.85E-06	4.544707	4.99965	5.621672	2.133098	1.77337	1.806583
AT1G67856	52.3631	2.571485439	0.395299	6.50517	7.76E-11	1.69E-09	6.327428	6.289258	6.96214	4.414102	1.77337	3.613718
AT3G03175	21.56755	2.572786152	0.471079	5.461479	4.72E-08	6.95E-07	5.037115	5.421786	5.468897	2.604042	2.545235	1.806583
AT1G08353	31.72908	2.581537117	0.436946	5.908143	3.46E-09	5.96E-08	5.857134	5.510498	6.147344	1.429371	3.045364	3.458449

AT1G20310	18.86933	2.583215164	0.512552	5.039904	4.66E-07	5.88E-06	5.154874	5.139739	5.204319	3.242825	0	0
AT3G16860	102.5916	2.586518617	0.269999	9.579749	9.73E-22	5.59E-20	7.476068	7.40097	7.599527	5.19216	3.955229	4.831847
AT4G06010	172.4846	2.59450948	0.25577	10.14391	3.53E-24	2.46E-22	7.879986	8.544774	8.194244	5.741253	5.476813	5.356498
AT1G76430	16.97418	2.598031953	0.525901	4.940151	7.81E-07	9.51E-06	5.284563	4.730912	5.024124	2.958503	0	0
AT5G39580	104.3411	2.599660144	0.355295	7.316901	2.54E-13	7.25E-12	7.755012	7.643877	7.130779	5.545934	3.416038	4.208431
AT1G07897	586.6485	2.603847845	0.220816	11.79193	4.30E-32	4.70E-30	9.477909	10.18356	10.19892	7.03764	7.548635	7.284329
AT1G17380	58.48942	2.605026251	0.341227	7.634281	2.27E-14	7.17E-13	6.506476	6.834086	6.771147	4.29471	2.545235	4.107507
AT4G15258	81.05739	2.605063008	0.303436	8.585222	9.07E-18	3.86E-16	6.865515	7.339335	7.217262	4.414102	4.654502	4.208431
AT5G58610	18.13549	2.607943255	0.512175	5.091902	3.54E-07	4.55E-06	4.578823	5.139739	5.508634	2.133098	0	2.247075
AT3G05320	81.68409	2.60864553	0.32216	8.097349	5.62E-16	2.06E-14	7.100888	6.97169	7.418413	5.051918	3.710678	3.998987
AT1G51850	116.0955	2.61073402	0.572282	4.561969	5.07E-06	5.31E-05	7.654621	7.43763	8.197287	4.626795	0	3.08651
AT3G13100	55.05717	2.612473331	0.393777	6.634396	3.26E-11	7.43E-10	6.051879	6.611509	7.058994	4.29471	3.045364	3.458449
AT3G57460	35.3446	2.621200845	0.452402	5.793966	6.87E-09	1.14E-07	5.384387	6.084872	6.442294	3.242825	0	3.458449
AT1G55390	13.56513	2.621425176	0.533158	4.91679	8.80E-07	1.06E-05	4.578823	4.607633	4.996442	1.429371	0	1.806583
AT1G74456	88.72941	2.623642992	0.274138	9.570531	1.06E-21	6.09E-20	7.282641	7.264784	7.293174	4.626795	4.786789	4.302755
AT1G13490	11.53918	2.625078526	0.548091	4.789495	1.67E-06	1.92E-05	4.399556	4.639454	4.53998	1.429371	0	1.169325
AT5G40395	74.30972	2.626586732	0.353381	7.432737	1.06E-13	3.16E-12	7.028676	6.666599	7.392097	4.021447	3.045364	4.767144
AT3G47348	326.141	2.631901749	0.18289	14.39063	5.93E-47	1.29E-44	9.097404	9.264123	9.054217	6.138016	6.810594	6.425197
AT3G07195	119.2622	2.635947743	0.543901	4.846375	1.26E-06	1.48E-05	7.963058	7.749716	7.705911	5.123743	1.77337	3.284433
AT3G27150	12.15375	2.637236802	0.545113	4.837966	1.31E-06	1.54E-05	4.509766	4.541804	4.685768	1.429371	1.77337	0
AT4G39361	279.0672	2.638536863	0.209476	12.59592	2.22E-36	3.06E-34	8.626782	9.152769	8.949832	6.238498	6.102626	6.270397
AT2G20350	17.89711	2.652202672	0.529401	5.009815	5.45E-07	6.82E-06	4.707748	5.688357	4.720024	2.604042	0	1.169325
AT3G55150	17.32364	2.65395901	0.520442	5.09943	3.41E-07	4.39E-06	4.961552	4.949759	5.251924	2.958503	0	0
AT1G75163	51.05374	2.654330914	0.382691	6.935965	4.03E-12	1.02E-10	6.297009	6.927264	6.232868	2.958503	3.955229	3.753887
AT5G54450	8.722906	2.680724176	0.582935	4.598664	4.25E-06	4.54E-05	3.791321	3.91999	4.720024	0	0	0
AT5G66620	78.80702	2.680791384	0.367571	7.293268	3.03E-13	8.54E-12	6.952657	7.223389	7.252795	5.123743	1.77337	3.881636
AT1G44130	20.55873	2.685495329	0.485096	5.536004	3.09E-08	4.68E-07	5.085378	5.226103	5.528099	2.604042	0	2.247075
AT1G61800	69.44008	2.685835027	0.42894	6.261564	3.81E-10	7.53E-09	7.152771	6.119132	7.407944	3.684038	1.77337	4.62832
AT2G32020	52.53943	2.696036325	0.376285	7.164888	7.79E-13	2.14E-11	6.276368	6.45042	6.925911	3.684038	2.545235	3.998987
AT4G16250	95.0387	2.697492529	0.275298	9.798447	1.14E-22	7.11E-21	7.287806	7.451141	7.454467	4.812122	3.955229	4.699401
AT2G08725	75.55302	2.697706209	0.31962	8.440365	3.16E-17	1.28E-15	6.787432	7.175355	7.211253	4.626795	4.164273	3.753887
AT4G06225	77.89085	2.703261124	0.325857	8.295852	1.08E-16	4.17E-15	6.80915	7.32473	7.199161	4.626795	3.045364	4.391288
AT1G09787	206.9852	2.717365309	0.237666	11.43356	2.84E-30	2.76E-28	8.214317	8.682881	8.572556	5.829815	5.997325	5.265734
AT2G07759	43.98699	2.71814011	0.368379	7.378642	1.60E-13	4.68E-12	6.376742	6.217283	6.357789	3.480233	3.045364	3.458449

AT5G45630	32.88955	2.731748773	0.435836	6.267838	3.66E-10	7.25E-09	5.91169	5.493187	6.279555	2.604042	2.545235	3.08651
AT5G66640	90.33773	2.736783793	0.406116	6.738922	1.60E-11	3.75E-10	6.939588	7.339335	7.701631	5.25748	1.77337	3.753887
AT1G07593	28.6164	2.749680906	0.453447	6.063954	1.33E-09	2.45E-08	5.477749	5.527604	6.134702	2.604042	1.77337	2.85705
AT5G24080	13.98696	2.753350104	0.551149	4.995659	5.86E-07	7.30E-06	4.437236	4.400386	5.364486	1.429371	0	1.169325
AT4G04510	18.12962	2.758811326	0.522172	5.283343	1.27E-07	1.76E-06	4.544707	5.527604	5.204319	1.429371	0	2.247075
AT2G07807	10.99168	2.761005735	0.561749	4.915014	8.88E-07	1.07E-05	4.676581	4.507728	4.242928	1.429371	0	0
AT1G53541	572.4654	2.762181272	0.223035	12.38454	3.17E-35	4.05E-33	9.457425	10.16864	10.18978	6.859769	7.349087	7.125632
AT3G18610	47.29103	2.766682983	0.398507	6.942614	3.85E-12	9.79E-11	6.276368	6.587239	6.442294	4.29471	0	3.08651
AT2G36792	982.8152	2.769123238	0.561633	4.930489	8.20E-07	9.97E-06	10.49934	10.84795	11.1884	7.697535	4.907958	5.356498
AT4G27652	60.46211	2.771894726	0.373712	7.417199	1.20E-13	3.54E-12	6.750493	6.459362	7.058994	4.524365	3.045364	3.08651
AT1G17960	63.33199	2.772214712	0.375117	7.390264	1.47E-13	4.31E-12	6.443067	6.861328	7.162264	4.414102	1.77337	3.881636
AT5G47850	41.32965	2.777573796	0.412381	6.735458	1.63E-11	3.84E-10	5.951294	6.441423	6.313604	4.021447	1.77337	2.584062
AT2G36790	980.0984	2.783866682	0.561464	4.958225	7.11E-07	8.74E-06	10.49488	10.84667	11.18495	7.673804	4.907958	5.31183
AT1G02520	59.71548	2.783904615	0.360509	7.722143	1.14E-14	3.72E-13	6.396005	6.946471	6.896258	3.862584	2.545235	4.107507
AT3G13600	82.23187	2.787312942	0.303662	9.178994	4.35E-20	2.21E-18	7.065234	7.259674	7.252795	4.626795	4.50885	3.613718
AT1G65610	24.46579	2.800766342	0.472734	5.924615	3.13E-09	5.45E-08	5.325325	5.43997	5.82421	2.133098	0	2.85705
AT3G10930	72.61932	2.809824505	0.333537	8.424316	3.63E-17	1.46E-15	7.034833	6.881427	7.186967	4.722432	2.545235	3.881636
AT3G50770	76.3482	2.81603224	0.357742	7.871686	3.50E-15	1.19E-13	6.697136	7.31491	7.252795	4.812122	3.045364	3.613718
AT2G36800	511.2186	2.822011587	0.218041	12.94255	2.59E-38	3.89E-36	9.442449	9.921715	10.03936	7.179211	6.379024	6.99045
AT2G39490	30.72113	2.826242406	0.444951	6.351806	2.13E-10	4.37E-09	5.756397	5.673064	6.069773	3.480233	1.77337	1.806583
AT3G44350	55.20153	2.831427114	0.404206	7.004907	2.47E-12	6.41E-11	6.965608	6.719662	6.232868	4.29471	0	3.458449
AT5G57010	26.81918	2.834448122	0.455538	6.222199	4.90E-10	9.52E-09	5.785904	5.657607	5.56626	2.133098	0	3.08651
AT1G32910	13.44612	2.838225066	0.565918	5.015262	5.30E-07	6.64E-06	4.509766	4.067637	5.407161	1.429371	0	0
AT2G35290	92.49392	2.842653359	0.365194	7.783949	7.03E-15	2.33E-13	7.040965	6.868059	7.995326	4.29471	4.164273	4.302755
AT3G50825	157.0445	2.844091367	0.225932	12.58827	2.45E-36	3.35E-34	8.068868	8.210632	8.099838	5.25748	5.019734	5.265734
AT1G03445	16.94786	2.868191299	0.557675	5.143126	2.70E-07	3.55E-06	4.321109	4.639454	5.839872	0	0	1.806583
AT2G14661	12.42331	2.871912532	0.562056	5.109652	3.23E-07	4.18E-06	4.104018	4.730912	5.024124	0	0	1.169325
AT4G11000	83.12398	2.877310357	0.369978	7.776984	7.43E-15	2.46E-13	6.919761	7.212852	7.528819	4.97633	3.416038	3.08651
AT3G09855	181.8326	2.887109567	0.216317	13.34666	1.24E-40	2.05E-38	8.272799	8.422074	8.330745	5.25748	5.220243	5.561185
AT4G24570	111.6137	2.893124774	0.271576	10.65309	1.69E-26	1.32E-24	7.758737	7.460079	7.710177	4.896561	4.346824	4.553554
AT4G08285	17.54252	2.909222283	0.523181	5.560643	2.69E-08	4.09E-07	4.935458	4.844481	5.468897	2.133098	0	1.169325
AT5G54710	317.2143	2.91830967	0.540768	5.396607	6.79E-08	9.78E-07	9.003195	9.071996	9.57066	6.066916	3.045364	4.474702
AT4G34410	294.0708	2.927365822	0.279021	10.49158	9.44E-26	7.16E-24	9.003195	9.305621	8.78365	6.632428	5.123469	5.635566
AT5G02645	190.29	2.928838311	0.223983	13.07619	4.50E-39	7.02E-37	8.358761	8.536347	8.350014	5.437371	4.786789	5.671366

AT4G06120	70.24826	2.931276997	0.33769	8.680388	3.94E-18	1.72E-16	7.152771	7.026869	6.819344	2.958503	3.710678	4.391288
AT4G08555	453.2893	2.93331658	0.557356	5.262911	1.42E-07	1.95E-06	9.451683	9.809929	9.98581	6.172293	2.545235	5.168875
AT3G09865	179.7822	2.94658867	0.217896	13.52294	1.15E-41	2.00E-39	8.257083	8.410594	8.325192	5.25748	5.220243	5.399825
AT1G01060	421.8555	2.947925538	0.435079	6.77561	1.24E-11	2.96E-10	10.07009	9.271758	9.318756	6.817077	5.760417	5.265734
AT1G06243	214.1252	2.948899109	0.271845	10.84773	2.04E-27	1.66E-25	8.34891	9.000282	8.297103	5.545934	5.82339	5.117892
AT1G30180	10.70744	2.950244534	0.575388	5.127403	2.94E-07	3.84E-06	4.056385	4.89808	4.377786	0	0	0
AT3G45960	37.73716	2.955114566	0.469418	6.295273	3.07E-10	6.16E-09	6.039683	5.40337	6.721284	3.242825	2.545235	1.806583
AT4G27654	39.75652	2.965474185	0.435484	6.809598	9.79E-12	2.37E-10	5.964257	6.152596	6.502575	3.862584	0	2.247075
AT4G09715	20.36168	2.970138209	0.499285	5.948787	2.70E-09	4.75E-08	5.284563	5.226103	5.407161	2.604042	0	1.169325
AT3G29725	12.91122	2.97284941	0.553667	5.369379	7.90E-08	1.13E-06	4.61215	4.89808	4.614715	1.429371	0	0
AT1G08795	13.15074	2.97633201	0.578669	5.143412	2.70E-07	3.55E-06	4.150129	4.067637	5.56626	0	0	0
AT2G27080	178.9118	2.988879615	0.320104	9.33721	9.89E-21	5.21E-19	8.211602	7.959642	8.750889	5.829815	4.164273	4.831847
AT1G47590	10.52504	2.998700306	0.572412	5.238708	1.62E-07	2.20E-06	4.321109	4.437061	4.614715	0	0	0
AT3G13080	2455.457	2.999607444	0.447896	6.697102	2.13E-11	4.95E-10	11.67525	12.329	12.35134	9.241996	7.432247	8.319723
AT5G22680	23.38055	3.01395056	0.482386	6.248004	4.16E-10	8.14E-09	5.325325	5.610218	5.547306	2.604042	0	1.806583
AT5G52050	153.8491	3.015656808	0.324674	9.288264	1.57E-20	8.19E-19	8.259715	8.074674	8.050212	5.786214	3.955229	4.107507
AT4G06310	84.48376	3.019073998	0.326259	9.253607	2.17E-20	1.13E-18	7.065234	7.391657	7.304507	3.242825	4.907958	3.753887
AT3G01055	54.39095	3.022077344	0.373897	8.082652	6.34E-16	2.31E-14	6.617317	6.704699	6.660846	4.164539	0	3.284433
AT5G37490	45.76507	3.024021674	0.411392	7.350697	1.97E-13	5.69E-12	6.255428	6.619509	6.368627	4.021447	0	2.584062
AT1G57650	24.79155	3.03993756	0.489598	6.209051	5.33E-10	1.03E-08	5.42246	5.287644	5.959397	2.604042	0	1.806583
AT1G43910	394.4243	3.041107271	0.546638	5.563289	2.65E-08	4.03E-07	9.159308	9.073452	10.18212	4.896561	3.045364	5.958939
AT2G35935	34.03149	3.046796943	0.429098	7.100467	1.24E-12	3.35E-11	6.099659	5.819152	6.096096	2.604042	1.77337	2.85705
AT3G56380	14.09531	3.055770317	0.554255	5.513289	3.52E-08	5.28E-07	4.768132	4.472827	5.204319	0	0	1.169325
AT3G61190	134.7409	3.063202057	0.342866	8.934112	4.10E-19	1.97E-17	7.979113	7.455617	8.297103	5.379864	3.416038	4.302755
AT2G44840	26.56838	3.063932879	0.505641	6.0595	1.37E-09	2.51E-08	5.477749	5.117315	6.2564	1.429371	0	2.584062
AT5G09505	95.93834	3.065298874	0.389841	7.862954	3.75E-15	1.28E-13	7.397007	8.051207	6.606946	3.684038	4.50885	3.881636
AT3G28580	54.00222	3.078237266	0.396857	7.756548	8.73E-15	2.86E-13	6.327428	6.894673	6.721284	3.242825	0	3.881636
AT3G12900	23.92255	3.08509547	0.536065	5.755074	8.66E-09	1.41E-07	4.85422	5.023964	6.324778	0	1.77337	1.806583
AT5G08515	95.51864	3.085900067	0.345074	8.94273	3.80E-19	1.83E-17	7.387414	7.871951	6.96214	4.414102	4.164273	3.753887
AT2G40750	75.17003	3.089316235	0.37401	8.259984	1.46E-16	5.59E-15	7.158422	6.734471	7.418413	4.29471	0	3.998987
AT5G24150	22.58289	3.09099771	0.523732	5.901866	3.59E-09	6.18E-08	4.707748	5.886999	5.56626	2.133098	1.77337	0
AT5G02055	88.60029	3.099230737	0.391418	7.917951	2.41E-15	8.34E-14	7.348392	7.881962	6.541408	4.164539	4.164273	3.284433
AT2G08375	28.28704	3.108884964	0.475207	6.542174	6.06E-11	1.33E-09	5.726274	6.107802	5.407161	2.133098	0	2.584062
AT2G32810	285.3039	3.109293374	0.214249	14.51254	1.01E-47	2.42E-45	8.87692	9.173295	8.962407	5.25748	6.200761	5.899804

AT2G22470	200.0311	3.113374023	0.311489	9.99513	1.60E-23	1.07E-21	8.444521	8.153926	8.866268	5.872138	3.955229	5.01018
AT3G02445	119.8443	3.113529736	0.354423	8.784774	1.57E-18	7.03E-17	7.555311	8.334874	7.258633	3.862584	4.164273	4.831847
AT4G06240	72.39787	3.115878065	0.346395	8.995167	2.36E-19	1.15E-17	6.727867	7.264784	7.156022	4.021447	3.045364	3.753887
AT5G01380	19.55855	3.117262195	0.511296	6.09679	1.08E-09	2.02E-08	5.085378	5.421786	5.298008	1.429371	0	1.806583
AT2G08900	31.5145	3.118112023	0.46749	6.669897	2.56E-11	5.91E-10	5.771226	6.319038	5.584968	2.133098	0	2.85705
AT3G17609	24.89941	3.122319303	0.480834	6.493555	8.38E-11	1.82E-09	5.695509	5.43997	5.657465	1.429371	0	2.584062
AT4G21680	117.3354	3.126205488	0.340613	9.178161	4.39E-20	2.22E-18	7.202852	7.624132	8.250989	4.524365	3.955229	4.474702
AT4G23215	50.94987	3.142161101	0.388326	8.091556	5.89E-16	2.15E-14	6.609086	6.554232	6.579222	4.021447	0	2.85705
AT2G06855	20.9093	3.143311868	0.508188	6.185332	6.20E-10	1.19E-08	5.548318	5.307588	5.179914	2.133098	1.77337	0
AT4G06250	83.81185	3.155450084	0.317513	9.938031	2.84E-23	1.85E-21	7.095007	7.382284	7.298852	3.684038	4.50885	3.613718
AT5G09515	93.52915	3.194951386	0.395325	8.081842	6.38E-16	2.32E-14	7.368035	8.036344	6.579222	3.480233	4.164273	3.881636
AT2G35744	413.4492	3.197133397	0.240743	13.28026	3.01E-40	4.93E-38	9.098874	9.92656	9.525372	6.238498	6.460523	6.015745
AT4G23070	16.04959	3.202106202	0.549741	5.824757	5.72E-09	9.59E-08	4.826091	4.701066	5.448611	0	0	1.169325
AT1G13310	49.92424	3.205134777	0.394065	8.13351	4.17E-16	1.54E-14	6.357218	6.635377	6.669636	3.684038	0	3.08651
AT3G62210	15.66843	3.207685238	0.572075	5.607107	2.06E-08	3.18E-07	4.544707	4.284411	5.759806	0	0	0
AT2G02680	28.5738	3.21479849	0.482462	6.663321	2.68E-11	6.17E-10	5.599069	5.594069	6.121948	2.958503	0	1.169325
AT2G39530	21.48626	3.21576993	0.507316	6.338792	2.32E-10	4.73E-09	5.154874	5.527604	5.508634	2.133098	0	1.169325
AT5G22520	33.53023	3.217572715	0.45031	7.145239	8.98E-13	2.46E-11	5.98984	5.846675	6.172301	3.242825	0	1.806583
AT1G20515	36.49004	3.219827901	0.471246	6.832588	8.34E-12	2.05E-10	5.938213	5.688357	6.61607	3.242825	0	1.806583
AT3G01760	25.02919	3.225284501	0.501596	6.430038	1.28E-10	2.70E-09	5.263741	5.626188	5.915727	0	0	2.584062
AT2G04070	179.6805	3.238491446	0.54066	5.989882	2.10E-09	3.78E-08	8.249161	8.151169	8.831438	4.524365	0	3.998987
AT2G47000	981.7918	3.243590978	0.218042	14.87598	4.72E-50	1.25E-47	10.3671	10.97068	10.99999	7.788738	6.985988	7.437718
AT4G30430	33.13238	3.278773186	0.476228	6.884881	5.78E-12	1.44E-10	5.741414	5.657607	6.48276	2.133098	0	2.584062
AT2G04040	238.7008	3.285291004	0.272966	12.03554	2.31E-33	2.65E-31	8.272799	8.84177	9.105328	5.597298	5.019734	5.265734
AT1G04517	25.2941	3.293434701	0.491257	6.704097	2.03E-11	4.73E-10	5.565434	5.610218	5.743245	0	0	2.584062
AT2G35747	335.3811	3.301216194	0.263048	12.54989	3.98E-36	5.41E-34	9.106203	9.732744	8.807742	5.829815	5.82339	5.773726
AT3G09870	27.54803	3.304197803	0.525932	6.282556	3.33E-10	6.62E-09	5.771226	4.788814	6.346869	2.133098	0	1.169325
AT5G17350	50.83073	3.30584481	0.396111	8.345763	7.08E-17	2.77E-15	6.681522	6.32883	6.729716	3.480233	1.77337	2.85705
AT1G08875	25.58333	3.309261316	0.490518	6.74646	1.51E-11	3.57E-10	5.599069	5.610218	5.759806	0	0	2.584062
AT4G30280	775.46	3.328055775	0.518892	6.413778	1.42E-10	2.99E-09	10.31112	10.27503	10.93585	6.999965	4.786789	5.117892
AT5G64810	97.06413	3.331983388	0.312827	10.65119	1.72E-26	1.34E-24	7.411277	7.559068	7.53845	4.524365	1.77337	4.107507
AT1G73340	18.35498	3.332039544	0.542309	6.14417	8.04E-10	1.52E-08	5.012364	4.89808	5.603436	0	1.77337	0
AT1G04527	27.03232	3.332539494	0.493638	6.750985	1.47E-11	3.48E-10	6.002463	5.307588	5.808377	1.429371	0	2.247075
AT4G09215	58.47878	3.337670531	0.383505	8.703072	3.23E-18	1.43E-16	6.649776	6.834086	6.8812	4.021447	0	2.85705

AT1G30190	80.6578	3.33861717	0.425563	7.845176	4.32E-15	1.46E-13	6.952657	6.920804	7.739697	4.524365	1.77337	2.247075
AT2G35930	93.08535	3.343311505	0.325165	10.2819	8.50E-25	6.10E-23	7.372904	7.274949	7.658123	4.414102	2.545235	3.881636
AT4G02160	21.19472	3.348455911	0.572629	5.847518	4.99E-09	8.42E-08	5.177314	3.970903	6.290994	0	0	0
AT1G06137	17.77101	3.373150534	0.539101	6.256995	3.92E-10	7.71E-09	5.384387	5.071394	5.024124	1.429371	0	0
AT1G15580	35.15185	3.379716437	0.462649	7.305141	2.77E-13	7.86E-12	5.615596	6.196041	6.357789	2.133098	1.77337	2.247075
AT1G08757	16.70058	3.385556888	0.564489	5.997558	2.00E-09	3.62E-08	5.061448	4.362754	5.621672	0	0	0
AT1G13650	28.45977	3.386574527	0.48239	7.020403	2.21E-12	5.77E-11	5.756397	6.025901	5.547306	1.429371	2.545235	1.169325
AT5G22530	15.0927	3.390562178	0.560998	6.043805	1.51E-09	2.75E-08	5.085378	5.183567	4.53998	0	0	0
AT5G13225	84.79225	3.39105914	0.407479	8.322055	8.65E-17	3.36E-15	7.31336	7.865238	6.47275	3.862584	3.416038	3.08651
AT1G22240	18.83979	3.399326976	0.540588	6.288197	3.21E-10	6.41E-09	4.797403	5.421786	5.448611	1.429371	0	0
AT5G02665	66.03458	3.401893281	0.379395	8.966632	3.06E-19	1.48E-17	6.830546	7.309975	6.669636	3.480233	3.045364	3.08651
AT5G52760	227.3983	3.408524221	0.252809	13.48262	1.98E-41	3.39E-39	8.751668	8.561481	8.841197	5.646896	3.710678	5.31183
AT1G20520	31.40585	3.41382565	0.498958	6.841911	7.81E-12	1.93E-10	5.741414	5.43997	6.462669	2.604042	0	1.169325
AT5G66564	137.2616	3.421638188	0.266602	12.83425	1.05E-37	1.50E-35	7.927094	8.100625	7.941902	4.29471	4.654502	4.391288
AT3G13857	289.6924	3.423183109	0.214491	15.95956	2.44E-57	8.37E-55	9.069182	9.021569	9.113405	5.319969	4.907958	5.987621
AT1G21850	34.03105	3.423515795	0.481537	7.109556	1.16E-12	3.15E-11	5.695509	5.747956	6.550955	1.429371	1.77337	2.247075
AT1G74710	615.4413	3.425983055	0.2378	14.40701	4.68E-47	1.03E-44	9.902578	10.10382	10.40905	6.859769	5.625572	6.85691
AT5G09115	289.1283	3.436001979	0.231615	14.83499	8.70E-50	2.24E-47	9.072179	9.018548	9.115015	5.25748	4.654502	6.04333
AT5G46315	277.8219	3.446201075	0.228531	15.07981	2.20E-51	5.95E-49	9.026558	8.951998	9.055894	5.19216	4.654502	5.958939
AT3G14735	288.3072	3.467882843	0.2263	15.32427	5.26E-53	1.57E-50	9.069182	9.020059	9.11018	5.25748	4.654502	5.987621
AT3G13855	288.3978	3.468401429	0.226236	15.33088	4.76E-53	1.45E-50	9.069182	9.021569	9.11018	5.25748	4.654502	5.987621
AT4G07875	288.6895	3.469848503	0.226321	15.33156	4.71E-53	1.45E-50	9.073675	9.020059	9.111793	5.25748	4.654502	5.987621
AT4G04615	288.7095	3.469871349	0.226421	15.32484	5.22E-53	1.57E-50	9.070681	9.020059	9.115015	5.25748	4.654502	5.987621
AT5G00750	367.9334	3.483931331	0.237047	14.69719	6.72E-49	1.71E-46	9.380937	9.180072	9.654971	5.741253	5.019734	6.222936
AT1G65481	16.23705	3.501551686	0.556533	6.291721	3.14E-10	6.29E-09	5.012364	4.816916	5.320509	0	0	0
AT5G02655	72.83686	3.50306968	0.359862	9.73447	2.15E-22	1.31E-20	7.287806	7.153483	6.842852	3.684038	3.045364	3.08651
AT5G51174	242.4117	3.503978091	0.274734	12.75406	2.96E-37	4.13E-35	8.353844	9.12494	8.866268	5.19216	5.476813	4.767144
AT3G48640	39.3615	3.511199299	0.456536	7.690952	1.46E-14	4.69E-13	5.843167	6.45042	6.390061	2.958503	1.77337	1.169325
AT1G16635	271.5123	3.518504665	0.285428	12.3271	6.47E-35	8.15E-33	8.49229	9.452802	8.839251	5.051918	5.476813	5.265734
AT5G36925	52.72612	3.521933475	0.400134	8.801885	1.35E-18	6.13E-17	6.727867	6.485861	6.721284	3.242825	3.045364	1.806583
AT4G07625	38.76611	3.553262539	0.449967	7.896719	2.86E-15	9.84E-14	5.925012	6.432368	6.302344	2.133098	1.77337	2.247075
AT2G27690	132.6377	3.564015813	0.34202	10.42049	2.00E-25	1.48E-23	7.694063	7.567364	8.465572	4.414102	3.955229	3.881636
AT1G68765	65.97922	3.592629122	0.383222	9.374805	6.93E-21	3.73E-19	7.169658	6.940097	6.811422	3.862584	0	2.85705
AT5G67450	47.39256	3.592800662	0.428839	8.37797	5.38E-17	2.14E-15	6.524093	6.227787	6.762956	2.958503	0	2.584062

AT5G41730	32.13856	3.617690398	0.472863	7.650607	2.00E-14	6.35E-13	5.898243	5.951799	6.096096	2.133098	0	1.806583
AT1G72240	61.98116	3.633157104	0.435903	8.334786	7.76E-17	3.03E-15	6.765383	6.269058	7.439125	2.958503	1.77337	2.85705
AT2G42065	28.97269	3.634022154	0.491313	7.396546	1.40E-13	4.13E-12	5.756397	5.718464	6.042961	1.429371	0	1.806583
AT5G38700	33.54536	3.637127063	0.477265	7.620764	2.52E-14	7.93E-13	6.051879	5.747956	6.290994	2.133098	0	1.806583
AT3G06890	51.59412	3.665209796	0.433109	8.462561	2.62E-17	1.07E-15	6.46147	6.319038	7.04555	2.604042	1.77337	2.584062
AT2G35387	89.67194	3.690752008	0.335367	11.00513	3.61E-28	3.09E-26	7.282641	7.554903	7.381434	3.242825	3.710678	3.458449
AT5G51000	22.13262	3.746536459	0.552447	6.781716	1.19E-11	2.85E-10	4.85422	5.43997	5.987793	0	0	0
AT3G29156	53.43487	3.748345002	0.449898	8.331535	7.98E-17	3.11E-15	6.201706	6.959136	6.835058	3.480233	0	1.169325
AT1G09943	1353.789	3.756851483	0.191412	19.62706	9.08E-86	1.01E-82	11.13215	11.08775	11.63804	7.548916	7.11744	7.604405
AT1G09937	1353.506	3.761543379	0.191268	19.66639	4.19E-86	4.89E-83	11.13215	11.08775	11.63804	7.535811	7.11744	7.604405
AT2G08910	99.53982	3.765497572	0.333439	11.29291	1.42E-29	1.32E-27	7.559588	7.756987	7.348966	3.480233	3.710678	3.458449
AT4G09135	206.4111	3.77317129	0.248623	15.17628	5.08E-52	1.48E-49	8.485562	8.753648	8.558426	4.414102	4.786789	4.831847
AT1G05247	259.6792	3.807905427	0.305305	12.47246	1.06E-35	1.40E-33	8.421205	9.425684	8.791725	4.626795	5.310931	4.699401
AT5G54030	30.08113	3.817521017	0.534694	7.139643	9.36E-13	2.55E-11	5.710973	5.204992	6.531797	1.429371	0	0
AT3G10986	33.47166	3.853168454	0.510412	7.549132	4.38E-14	1.35E-12	5.800434	5.688357	6.541408	2.133098	0	0
AT1G09932	148.0076	3.853735542	0.347463	11.09107	1.39E-28	1.23E-26	8.056815	7.834638	8.468093	4.896561	2.545235	3.284433
AT2G36750	36.42553	3.876054014	0.52066	7.444504	9.73E-14	2.91E-12	5.242615	6.376816	6.61607	0	0	1.806583
AT4G07635	131.8273	3.906600459	0.318662	12.2594	1.50E-34	1.82E-32	7.956585	8.083376	7.867447	4.626795	3.710678	2.584062
AT4G16820	27.77998	3.964851424	0.516171	7.681268	1.58E-14	5.03E-13	5.800434	5.747956	5.870694	1.429371	0	0
AT4G02170	64.83101	3.978809484	0.478332	8.318088	8.94E-17	3.47E-15	6.787432	6.073269	7.680041	1.429371	1.77337	2.584062
AT1G65390	74.17779	3.998164215	0.395306	10.11409	4.78E-24	3.30E-22	7.095007	7.074206	7.326908	3.862584	0	1.806583
AT3G29250	100.0304	4.008993665	0.4037	9.930627	3.06E-23	1.99E-21	7.328478	7.062516	8.160341	2.958503	3.416038	2.85705
AT3G45700	37.28339	4.09896825	0.527072	7.776865	7.43E-15	2.46E-13	6.087861	5.475665	6.819344	0	0	1.169325
AT1G09950	59.6729	4.100685521	0.46657	8.789	1.51E-18	6.80E-17	6.6255	6.319038	7.444257	2.133098	1.77337	1.806583
AT5G22380	85.45932	4.140996733	0.382015	10.83987	2.23E-27	1.81E-25	7.261791	7.23385	7.613262	3.242825	0	3.08651
AT5G58750	35.85393	4.145916297	0.518206	8.000516	1.24E-15	4.39E-14	5.741414	5.976925	6.643102	1.429371	0	0
AT5G41550	68.92581	4.164811296	0.422081	9.867323	5.77E-23	3.64E-21	6.712583	7.136858	7.304507	2.958503	2.545235	1.169325
AT2G00370	60.07113	4.203650306	0.480223	8.753539	2.07E-18	9.24E-17	6.190717	6.763641	7.454467	2.604042	0	1.169325
AT1G22470	88.57241	4.211363384	0.421204	9.998397	1.55E-23	1.04E-21	7.169658	7.050731	7.923646	3.480233	1.77337	1.806583
AT2G07605	366.1769	4.228808995	0.231082	18.30001	8.27E-75	5.45E-72	9.209166	9.578161	9.537428	4.812122	5.396255	5.065041
AT5G54720	124.5492	4.251240816	0.348551	12.19691	3.23E-34	3.86E-32	7.841862	7.686387	8.172762	4.021447	2.545235	2.85705
AT2G07335	204.7129	4.25949672	0.307963	13.83119	1.65E-43	3.10E-41	8.346437	8.638298	8.854751	3.242825	3.416038	4.767144
AT1G33760	146.8185	4.268422494	0.364996	11.69443	1.36E-31	1.44E-29	8.032403	7.796335	8.534564	4.414102	1.77337	2.85705
AT2G13810	48.31647	4.353286561	0.52059	8.362216	6.16E-17	2.43E-15	5.710973	6.511881	7.20522	1.429371	0	0

AT1G05913	550.2055	4.367499918	0.232464	18.78786	9.49E-79	7.87E-76	9.915985	10.34364	9.81309	5.597298	5.883728	5.117892
AT3G21805	794.1069	4.391643822	0.271739	16.16129	9.46E-59	3.52E-56	10.08426	10.97302	10.54125	6.503738	5.625572	5.482759
AT2G21900	77.13376	4.397807122	0.410857	10.70398	9.75E-27	7.73E-25	7.158422	7.103022	7.454467	2.604042	0	2.584062
AT1G01680	220.7745	4.44029318	0.305866	14.51711	9.44E-48	2.29E-45	8.446832	8.7609	8.973099	4.626795	1.77337	4.107507
AT2G20720	266.7597	4.505572761	0.284529	15.83522	1.78E-56	6.01E-54	8.775767	9.058818	9.18413	3.684038	3.416038	4.893774
AT1G05917	544.9537	4.51851747	0.243039	18.59172	3.75E-77	2.92E-74	9.905941	10.33941	9.804136	5.379864	5.82339	4.831847
AT3G21080	109.9014	4.683714386	0.406187	11.53093	9.21E-31	9.07E-29	7.525009	7.495286	8.154091	2.958503	1.77337	2.247075
AT5G62480	155.575	4.743709873	0.347201	13.66272	1.70E-42	3.11E-40	8.159013	8.100625	8.475632	3.480233	0	3.458449
AT3G56891	137.1618	4.778637782	0.392602	12.17172	4.40E-34	5.21E-32	8.192449	7.600076	8.339035	3.684038	0	2.247075
AT5G34853	153.5389	5.968867259	0.437787	13.63417	2.51E-42	4.57E-40	8.264963	8.322675	8.197287	1.429371	0	1.169325
AT5G34850	944.328	7.955572999	0.364358	21.83452	1.09E-105	1.75E-102	10.86385	10.87827	10.90454	0	0	2.85705
