# Supplementary material for: Induction of epigenetic variation in Arabidopsis by over-expression of DNA METHYLTRANSFERASE1 (MET1)
Source: PLoS One. 2018 Feb 21;13(2):e0192170. doi: 10.1371/journal.pone.0192170 (PMC5821449; doi:10.1371/journal.pone.0192170)
Supplement: S2 Table — (PDF) [file pone.0192170.s007.pdf]

S2 Table: List of genes with altered transcript levels in line A1-

	baseMean	log2FoldCh	lfcSE	stat	pvalue	padj	control 1	control 2	control 3	A1-4	A1-5	A1-6
AT2G11780	2722.871	-10.3706	0.308625	-33.6026	1.54E-247	3.95E-243	0.60238	1.006035	1.442988	12.49783	12.51056	12.20393
AT4G07942	1858.625	-10.027	0.364325	-27.5221	9.56E-167	6.14E-163	0	0	0	11.58755	12.36911	11.4445
AT3G06465	1813.534	-9.7805	0.318758	-30.6831	9.56E-207	1.23E-202	1.026049	1.329167	0.653468	11.97388	11.9454	11.50913
AT4G06506	2193.093	-9.6911	0.366074	-26.4731	1.98E-154	7.27E-151	1.026049	0	0.653468	11.69192	12.71583	11.60924
AT1G42050	1711.461	-9.59963	0.364805	-26.3144	1.31E-152	3.74E-149	0.60238	0	0.653468	11.1121	12.26647	11.61207
AT3G42716	1225.922	-9.39778	0.356392	-26.3692	3.09E-153	9.92E-150	0.60238	0.588989	0	11.23829	11.6299	10.79158
AT4G06720	992.0394	-9.23309	0.364092	-25.3593	7.10E-142	1.52E-138	0	0.588989	0	10.78723	11.36062	10.60545
AT3G30620	1296.299	-9.19948	0.325159	-28.2922	4.31E-176	3.69E-172	1.35313	1.006035	1.101576	11.32918	11.6396	10.97558
AT2G09187	931.4021	-9.19793	0.362285	-25.3886	3.37E-142	7.86E-139	0.60238	0	0	10.63575	11.2447	10.61958
AT2G11778	606.4654	-8.98106	0.36327	-24.7228	6.08E-135	1.12E-131	0	0	0	10.29395	10.15353	10.2847
AT3G32880	677.2519	-8.75065	0.382378	-22.8848	6.58E-116	9.39E-113	0	0	0	10.13354	10.94967	9.915018
AT4G06485	775.4451	-8.65258	0.342374	-25.2723	6.44E-141	1.27E-137	0.60238	1.006035	1.101576	10.38536	10.97286	10.35053
AT3G42719	545.3891	-8.60446	0.379333	-22.6831	6.58E-114	8.89E-111	0	0	0	10.01076	10.5007	9.634467
AT3G43862	532.7758	-8.44571	0.362478	-23.2999	4.44E-120	6.71E-117	0.60238	0	0.653468	9.811164	10.41297	9.871625
AT3G30749	382.1356	-8.34583	0.375108	-22.2491	1.15E-109	1.41E-106	0	0	0	9.554553	9.759072	9.403699
AT4G06718	510.7634	-8.3172	0.343937	-24.1823	3.42E-129	5.48E-126	1.026049	0.588989	0.653468	10.09481	10.13309	9.729548
AT5G35057	379.2385	-8.23078	0.381585	-21.57	3.44E-103	3.84E-100	0	0	0	9.316796	9.934731	9.370683
AT4G06736	397.5226	-8.11142	0.391385	-20.7249	2.07E-95	1.97E-92	0	0	0	9.531537	10.13309	9.036445
AT4G07605	325.0349	-8.05304	0.383978	-20.9727	1.17E-97	1.15E-94	0	0	0	9.30055	9.657748	9.0081
AT5G33389	356.088	-8.01635	0.364782	-21.9757	4.92E-107	5.74E-104	0	0.588989	0.653468	9.181402	9.538439	9.669278
AT4G03790	465.2503	-8.01004	0.372505	-21.5032	1.45E-102	1.56E-99	0	1.329167	0	9.643111	10.3553	9.416697
AT2G06590	278.7165	-7.90865	0.384255	-20.5818	4.00E-94	3.31E-91	0	0	0	8.919455	9.396978	9.013813
AT2G10180	326.9346	-7.81933	0.385186	-20.3001	1.28E-91	9.99E-89	0	0	0.653468	9.077177	9.820033	9.022342
AT4G05587	289.2598	-7.79295	0.379147	-20.5539	7.10E-94	5.70E-91	0	0	0.653468	8.915905	9.517558	9.028
AT4G03860	357.689	-7.73493	0.384256	-20.1296	4.06E-90	3.07E-87	0.60238	0	0.653468	9.117922	10.02627	9.102286
AT4G06517	267.0821	-7.71273	0.393874	-19.5817	2.21E-85	1.39E-82	0	0	0	8.734308	9.528036	8.785697
AT2G14180	402.5403	-7.69895	0.373064	-20.6371	1.28E-94	1.09E-91	1.35313	0	0.653468	9.308696	10.19358	9.254831
AT4G06531	292.917	-7.69405	0.386905	-19.8862	5.36E-88	3.72E-85	0.60238	0	0	9.102387	9.619065	8.727774
AT4G28970	226.5839	-7.66527	0.389249	-19.6925	2.50E-86	1.69E-83	0	0	0	9.061195	8.778398	8.604447
AT4G06615	237.5282	-7.66382	0.390603	-19.6205	1.03E-85	6.64E-83	0	0	0	8.820385	9.214403	8.577693
AT3G30836	229.9641	-7.60687	0.392472	-19.382	1.10E-83	6.48E-81	0	0	0	8.831735	9.161545	8.469584

AT2G06670	204.5466	-7.57145	0.388488	-19.4895	1.35E-84	8.24E-82	0	0	0	8.576848	8.830121	8.619516
AT1G67105	935.6621	-7.52575	0.282105	-26.6771	8.67E-157	3.71E-153	3.439114	2.331572	2.324987	11.27686	10.70844	10.49643
AT4G06530	254.594	-7.50299	0.403848	-18.5787	4.78E-77	2.45E-74	0	0	0	8.607986	9.599327	8.510575
AT2G03965	239.9279	-7.47149	0.360698	-20.714	2.59E-95	2.30E-92	0.60238	0.588989	0.653468	8.785791	9.035019	8.891901
AT3G30610	229.63	-7.46281	0.387048	-19.2814	7.70E-83	4.30E-80	0	0.588989	0	8.781895	9.201369	8.457056
AT3G43863	275.3359	-7.44975	0.373245	-19.9594	1.24E-88	9.11E-86	0.60238	0	1.101576	8.705807	9.528036	8.958595
AT3G30695	251.9493	-7.38815	0.371056	-19.9111	3.26E-88	2.33E-85	0	1.006035	0.653468	8.685098	9.362265	8.792361
AT4G06573	198.1766	-7.37027	0.398964	-18.4735	3.37E-76	1.67E-73	0	0	0	8.572344	9.02025	8.190645
AT5G32495	213.1365	-7.36578	0.38866	-18.9518	4.27E-80	2.29E-77	0.60238	0	0	8.531166	9.134371	8.448643
AT3G32010	190.0416	-7.3489	0.397952	-18.4668	3.82E-76	1.85E-73	0	0	0	8.469531	8.944051	8.210684
AT5G32404	179.9217	-7.33095	0.396104	-18.5077	1.79E-76	9.02E-74	0	0	0	8.26147	8.813085	8.348197
AT1G23915	183.4003	-7.29536	0.400921	-18.1965	5.50E-74	2.40E-71	0	0	0	8.865258	8.508985	8.091462
AT2G06760	159.2532	-7.2713	0.394516	-18.4309	7.42E-76	3.53E-73	0	0	0	8.39498	8.328941	8.230449
AT1G40101	179.9324	-7.21863	0.403061	-17.9095	9.94E-72	4.05E-69	0	0	0	8.051003	8.944051	8.343667
AT3G32240	163.9094	-7.19152	0.399923	-17.9822	2.68E-72	1.13E-69	0	0	0	8.024871	8.687846	8.292876
AT5G33050	182.3569	-7.17228	0.392154	-18.2895	1.00E-74	4.61E-72	0.60238	0	0	8.114333	8.880053	8.444419
AT5G32624	144.9348	-7.12589	0.399585	-17.8332	3.90E-71	1.54E-68	0	0	0	8.063893	8.037767	8.418806
AT3G43867	164.2421	-7.10999	0.38966	-18.2467	2.20E-74	9.74E-72	0	0	0.653468	8.316365	8.630654	8.091462
AT5G31719	176.4184	-7.05125	0.383543	-18.3845	1.75E-75	8.17E-73	0	0.588989	0.653468	8.132803	8.813085	8.366176
AT5G32475	170.3183	-7.03174	0.39703	-17.7108	3.46E-70	1.33E-67	0	0	0.653468	8.204403	8.8636	8.041989
AT3G42720	190.4478	-7.02889	0.391552	-17.9513	4.68E-72	1.94E-69	0.60238	0.588989	0	7.950468	8.959616	8.641827
AT1G40121	137.5629	-7.00669	0.402299	-17.4166	6.17E-68	2.06E-65	0	0	0	7.842399	8.376102	8.058669
AT5G29975	169.6044	-7.00615	0.383943	-18.2479	2.15E-74	9.69E-72	0	0.588989	0.653468	8.120516	8.760736	8.264398
AT5G32306	138.797	-6.98521	0.403822	-17.2978	4.89E-67	1.57E-64	0	0	0	7.857284	8.466036	7.967393
AT4G06686	123.189	-6.94963	0.401538	-17.3075	4.13E-67	1.34E-64	0	0	0	7.908207	7.915398	8.025114
AT2G10310	136.8064	-6.94877	0.405301	-17.1447	6.89E-66	2.16E-63	0	0	0	7.957392	8.466036	7.792291
AT1G41720	123.9197	-6.92597	0.402544	-17.2055	2.41E-66	7.66E-64	0	0	0	7.998257	8.066803	7.798924
AT4G06484	170.5915	-6.92342	0.404605	-17.1115	1.22E-65	3.72E-63	0.60238	0	0	8.018263	8.975015	8.041989
AT2G15555	176.0742	-6.87679	0.355386	-19.3502	2.03E-83	1.16E-80	1.35313	1.006035	0	8.316365	8.591227	8.461244
AT3G30837	152.964	-6.87462	0.386512	-17.7863	9.03E-71	3.51E-68	0	1.006035	0	8.089333	8.611075	8.002302
AT4G08050	641.2551	-6.87256	0.266161	-25.8211	5.14E-147	1.32E-143	2.501815	3.766043	2.324987	10.28984	10.5834	10.02662
AT2G06330	140.0059	-6.86766	0.396012	-17.342	2.27E-67	7.46E-65	0	0	0.653468	7.957392	8.48767	7.876237
AT4G06704	139.6741	-6.84043	0.412867	-16.5681	1.18E-61	3.11E-59	0	0	0	7.757664	8.669033	7.765446

AT3G01345	176.2633	-6.8286	0.34752	-19.6495	5.84E-86	3.85E-83	1.35313	1.329167	0	8.454926	8.42177	8.506528
AT4G05632	115.5434	-6.82403	0.40619	-16.8001	2.44E-63	7.03E-61	0	0	0	7.650652	7.850087	8.047571
AT2G14730	112.496	-6.80468	0.405644	-16.775	3.72E-63	1.06E-60	0	0	0	7.936518	7.850087	7.660083
AT5G32197	114.4267	-6.79466	0.40614	-16.7299	7.94E-63	2.19E-60	0	0	0	7.642081	8.066803	7.792291
AT4G08080	139.2227	-6.79297	0.400921	-16.9434	2.15E-64	6.35E-62	0.60238	0	0	7.804499	8.571102	7.869951
AT3G32020	121.6989	-6.79039	0.409407	-16.5859	8.81E-62	2.36E-59	0	0	0	7.929492	8.255176	7.522564
AT5G32107	136.3583	-6.78307	0.385305	-17.6044	2.28E-69	8.61E-67	0	0.588989	0.653468	7.857284	8.328941	8.058669
AT4G07586	132.6205	-6.76714	0.384742	-17.5888	3.00E-69	1.12E-66	0	1.006035	0	7.977969	8.255176	7.907267
AT3G33072	406.8639	-6.7485	0.452824	-14.9031	3.14E-50	5.85E-48	0	0	0	8.957944	10.28907	9.442346
AT5G32228	108.7281	-6.74519	0.407088	-16.5694	1.16E-61	3.07E-59	0	0	0	7.571623	7.915398	7.805528
AT2G04655	111.023	-6.7403	0.407791	-16.5288	2.28E-61	5.85E-59	0	0	0	7.589563	8.066803	7.703156
AT2G07400	106.3706	-6.72086	0.4072	-16.5051	3.37E-61	8.58E-59	0	0	0	7.63346	7.915398	7.652777
AT4G06622	106.3559	-6.63546	0.411887	-16.1099	2.17E-58	5.27E-56	0	0	0	7.439382	8.095267	7.600575
AT4G05633	108.8662	-6.62027	0.413797	-15.9988	1.30E-57	3.07E-55	0	0	0	7.488014	8.203818	7.506442
AT3G43304	106.6829	-6.60238	0.398551	-16.5659	1.23E-61	3.19E-59	0	0.588989	0	7.59845	7.915398	7.696066
AT5G33255	105.4468	-6.5797	0.402275	-16.3562	3.93E-60	9.89E-58	0	0.588989	0	7.624787	7.47011	8.025114
AT4G05591	124.2991	-6.57657	0.422825	-15.5539	1.50E-54	3.15E-52	0	0	0	7.459032	8.611075	7.506442
AT2G06470	118.9656	-6.57288	0.407236	-16.1402	1.33E-58	3.26E-56	0	0.588989	0	7.326244	8.255176	7.967393
AT3G44042	92.9052	-6.56537	0.412363	-15.9213	4.51E-57	1.05E-54	0	0	0	7.488014	7.33342	7.778931
AT3G42431	91.37902	-6.54544	0.411139	-15.9203	4.58E-57	1.06E-54	0	0	0	7.525773	7.594963	7.440091
AT3G42060	108.1823	-6.5237	0.406243	-16.0586	4.98E-58	1.18E-55	0	0	0.653468	8.089333	7.74622	7.361607
AT1G40310	95.65134	-6.51647	0.41443	-15.7239	1.04E-55	2.32E-53	0	0	0	7.607282	7.850087	7.240127
AT2G13547	99.24463	-6.50902	0.402468	-16.1728	7.85E-59	1.94E-56	0	0	0.653468	7.879329	7.51294	7.490138
AT5G35052	94.53188	-6.50749	0.414016	-15.7179	1.14E-55	2.52E-53	0	0	0	7.44924	7.883112	7.316087
AT5G33391	87.36299	-6.49421	0.413586	-15.7022	1.46E-55	3.21E-53	0	0	0	7.409395	7.284819	7.652777
AT5G32511	105.4972	-6.47608	0.420941	-15.3848	2.07E-53	4.22E-51	0	0	0	7.336904	8.280186	7.352617
AT3G42718	87.62789	-6.42225	0.415739	-15.4478	7.81E-54	1.62E-51	0	0	0	7.215076	7.74622	7.370542
AT4G03865	103.2122	-6.3906	0.424979	-15.0374	4.17E-51	8.12E-49	0	0	0	7.226586	8.304769	7.288068
AT1G42040	271.1499	-6.34577	0.458356	-13.8446	1.37E-43	2.05E-41	0	0	0	8.384742	9.723045	8.815446
AT3G32970	91.48207	-6.30457	0.4244	-14.8552	6.43E-50	1.17E-47	0	0	0	7.168094	8.066803	7.128689
AT1G36520	102.0057	-6.29717	0.40037	-15.7284	9.67E-56	2.18E-53	0	0	1.101576	7.459032	8.095267	7.361607
AT3G47320	81.96596	-6.28838	0.423351	-14.8538	6.57E-50	1.19E-47	0	0	0	7.773445	7.182411	7.030642
AT5G37125	83.22593	-6.28097	0.408063	-15.3921	1.85E-53	3.80E-51	0.60238	0	0	7.226586	7.284819	7.615684

AT4G06606	75.21667	-6.2763	0.418122	-15.0107	6.25E-51	1.21E-48	0	0	0	7.156105	7.380437	7.180412
AT3G33127	76.90897	-6.24964	0.420198	-14.8731	4.93E-50	9.04E-48	0	0	0	7.003888	7.554534	7.210579
AT2G04290	84.33872	-6.21522	0.426856	-14.5605	5.01E-48	8.82E-46	0	0	0	7.497547	7.78168	6.758512
AT5G30762	88.27103	-6.21506	0.428231	-14.5134	9.97E-48	1.74E-45	0	0	0	6.878163	8.037767	7.249844
AT2G00430	136.5028	-6.20039	0.346557	-17.8914	1.38E-71	5.52E-69	1.35313	1.329167	1.442988	8.38987	7.816289	8.002302
AT2G20460	77.60996	-6.19834	0.407947	-15.194	3.88E-52	7.66E-50	0	0	0.653468	7.191776	7.380437	7.278607
AT3G32043	77.49596	-6.18278	0.408765	-15.1255	1.10E-51	2.16E-49	0.60238	0	0	7.27173	7.42597	7.139183
AT2G05914	130.9794	-6.15855	0.352696	-17.4613	2.82E-68	9.80E-66	1.61959	1.329167	0.653468	7.86467	8.352714	7.812101
AT4G06735	74.1351	-6.10753	0.426571	-14.3177	1.70E-46	2.74E-44	0	0	0	6.833696	7.672573	7.019324
AT4G06628	72.22302	-6.07828	0.427088	-14.2319	5.81E-46	9.15E-44	0	0	0	6.833696	7.63429	6.949491
AT4G06607	85.2319	-6.06209	0.405771	-14.9397	1.82E-50	3.43E-48	0	0.588989	0.653468	7.082003	7.850087	7.200593
AT5G15360	73.34113	-6.02114	0.401517	-14.996	7.80E-51	1.50E-48	0.60238	0	0.653468	7.144016	7.182411	7.278607
AT3G33058	73.01778	-6.01565	0.401721	-14.9747	1.07E-50	2.04E-48	1.026049	0	0	7.226586	7.182411	7.180412
AT3G33157	75.92756	-5.99952	0.418841	-14.3241	1.55E-46	2.52E-44	0	0.588989	0	7.069274	7.709866	6.837962
AT2G01840	96.60589	-5.99836	0.406455	-14.7577	2.74E-49	4.93E-47	0.60238	1.006035	0	7.580621	8.066803	6.925441
AT2G07395	68.05623	-5.99204	0.413989	-14.4739	1.77E-47	3.05E-45	0	0	0.653468	7.069274	7.284819	6.913264
AT3G44265	66.43804	-5.86555	0.40576	-14.4557	2.31E-47	3.95E-45	0.60238	0.588989	0	6.949358	7.182411	7.041871
AT2G23720	57.50325	-5.86107	0.432876	-13.5398	9.10E-42	1.28E-39	0	0	0	7.191776	6.752923	6.55411
AT4G05588	63.10548	-5.85523	0.434563	-13.4738	2.23E-41	3.02E-39	0	0	0	6.457472	7.47011	6.863502
AT3G33073	77.28539	-5.85515	0.442929	-13.2192	6.80E-40	8.56E-38	0	0	0	6.4378	8.008134	6.937516
AT1G38450	83.15141	-5.85384	0.433378	-13.5075	1.41E-41	1.93E-39	0.60238	0	0	6.605875	8.066803	7.085933
AT2G06250	62.27868	-5.8497	0.433989	-13.4789	2.08E-41	2.83E-39	0	0	0	6.691428	7.47011	6.585144
AT1G38185	67.41655	-5.84259	0.42225	-13.8368	1.53E-43	2.27E-41	0	0	0.653468	6.803271	7.554534	6.744835
AT3G30170	63.28878	-5.83335	0.407021	-14.3318	1.38E-46	2.26E-44	0.60238	0	0.653468	7.094621	6.822676	7.041871
AT5G30450	63.36628	-5.83286	0.42338	-13.7769	3.51E-43	5.18E-41	0.60238	0	0	6.605875	6.952811	7.334468
AT2G13400	55.76473	-5.80949	0.432259	-13.4398	3.53E-41	4.75E-39	0	0	0	6.514927	7.128345	6.731026
AT4G29200	85.43925	-5.79169	0.425747	-13.6036	3.81E-42	5.47E-40	0	0.588989	0.653468	6.674719	8.095267	7.118118
AT4G05715	53.42006	-5.78597	0.436364	-13.2595	3.97E-40	5.11E-38	0	0	0	7.030399	6.024848	6.99642
AT4G03005	66.93282	-5.75495	0.402435	-14.3003	2.18E-46	3.47E-44	1.026049	0	0.653468	7.378772	6.752923	7.007918
AT4G08691	65.54856	-5.72135	0.416435	-13.7389	5.94E-43	8.62E-41	0.60238	0.588989	0	6.514927	7.234523	7.259495
AT2G10680	55.7803	-5.72071	0.42148	-13.5729	5.80E-42	8.27E-40	0	0	0.653468	6.623396	7.013727	6.772062
AT2G13300	52.93078	-5.6965	0.4239	-13.4383	3.61E-41	4.82E-39	0.60238	0	0	6.740426	6.434336	6.98483
AT2G05660	52.46651	-5.68228	0.423204	-13.4268	4.21E-41	5.58E-39	0.60238	0	0	6.907064	6.602406	6.645278

AT5G37385	49.96647	-5.66666	0.438298	-12.9288	3.10E-38	3.67E-36	0	0	0	6.976881	6.602406	6.315891
AT2G10660	67.57417	-5.6549	0.43503	-12.9989	1.24E-38	1.50E-36	0	0.588989	0	6.691428	7.74622	6.489965
AT5G33254	47.55553	-5.65272	0.435555	-12.9782	1.63E-38	1.94E-36	0	0	0	6.533581	6.752923	6.45679
AT1G36085	49.62593	-5.64682	0.439173	-12.8578	7.77E-38	8.95E-36	0	0	0	6.976881	6.602406	6.278406
AT3G44070	55.27904	-5.61658	0.442475	-12.6936	6.42E-37	7.17E-35	0	0	0	6.514927	7.380437	6.259292
AT3G42253	67.22791	-5.61413	0.40784	-13.7655	4.11E-43	6.03E-41	0	0	1.442988	6.803271	7.51294	6.785485
AT1G20400	57.89246	-5.60267	0.43361	-12.921	3.43E-38	4.04E-36	0.60238	0	0	7.282799	6.88921	6.239921
AT4G06538	110.9321	-5.59762	0.435264	-12.8603	7.53E-38	8.71E-36	0	1.329167	0.653468	6.224753	8.376102	8.019445
AT3G29612	51.27459	-5.58748	0.42583	-13.1214	2.48E-39	3.07E-37	0	0.588989	0	6.605875	6.952811	6.473473
AT2G05700	52.11013	-5.5825	0.428163	-13.0383	7.41E-39	9.02E-37	0	0	0.653468	6.9213	6.822676	6.334274
AT4G09380	56.7942	-5.57902	0.419306	-13.3054	2.15E-40	2.79E-38	0.60238	0.588989	0	7.179983	6.752923	6.489965
AT3G30825	44.52833	-5.57678	0.437746	-12.7397	3.56E-37	4.01E-35	0	0	0	6.377123	6.602406	6.489965
AT4G08093	53.206	-5.57055	0.41724	-13.351	1.17E-40	1.54E-38	0	0.588989	0.653468	7.030399	6.342338	6.772062
AT2G10280	65.31672	-5.56901	0.45322	-12.2877	1.06E-34	1.07E-32	0	0	0	7.39926	7.42597	5.758224
AT4G06738	53.81985	-5.55928	0.444858	-12.4967	7.78E-36	8.29E-34	0	0	0	6.588138	7.33342	6.096513
AT2G06720	59.97108	-5.50878	0.438624	-12.5592	3.54E-36	3.82E-34	0	0	0.653468	6.05437	7.47011	6.888598
AT1G38360	53.55002	-5.49473	0.417834	-13.1505	1.69E-39	2.10E-37	0.60238	0	0.653468	6.640707	7.072174	6.473473
AT2G02205	43.03735	-5.4789	0.445895	-12.2874	1.06E-34	1.07E-32	0	0	0	6.640707	5.617177	6.811962
AT2G10000	50.61162	-5.47257	0.4315	-12.6827	7.38E-37	8.21E-35	0	0	0.653468	6.417857	7.128345	6.334274
AT5G31963	45.83491	-5.46948	0.444	-12.3187	7.19E-35	7.33E-33	0	0	0	6.028299	6.952811	6.473473
AT4G08598	45.56745	-5.4681	0.447419	-12.2214	2.39E-34	2.33E-32	0	0	0	6.105138	6.244072	7.041871
AT3G30668	40.42157	-5.4524	0.441381	-12.3531	4.69E-35	4.84E-33	0	0	0	6.292034	6.434336	6.334274
AT4G04170	1209.675	-5.43491	0.418216	-12.9955	1.30E-38	1.57E-36	4.57299	4.655421	3.173324	10.57514	11.2447	11.66795
AT2G09930	42.53866	-5.43469	0.443282	-12.2601	1.48E-34	1.48E-32	0	0	0	6.079977	6.752923	6.370353
AT3G31540	46.89425	-5.42176	0.449146	-12.0713	1.50E-33	1.41E-31	0	0	0	5.800813	6.822676	6.850789
AT5G26270	131.7881	-5.41124	0.320243	-16.8973	4.71E-64	1.38E-61	2.363383	2.179302	1.950366	7.553458	8.328941	8.096855
AT4G18150	50.77746	-5.38825	0.422873	-12.742	3.45E-37	3.91E-35	1.026049	0	0	6.640707	7.013727	6.278406
AT4G06835	42.17239	-5.3755	0.433468	-12.4011	2.58E-35	2.68E-33	0.60238	0	0	6.691428	6.13862	6.352426
AT5G32423	42.1718	-5.37418	0.446765	-12.0291	2.50E-33	2.34E-31	0	0	0	5.890376	6.752923	6.473473
AT2G11650	40.55333	-5.34077	0.446532	-11.9606	5.72E-33	5.34E-31	0	0	0	5.974702	6.752923	6.239921
AT3G31310	36.35997	-5.32665	0.445419	-11.9587	5.84E-33	5.44E-31	0	0	0	6.269954	6.13862	6.200381
AT5G35145	37.36019	-5.30686	0.446363	-11.8891	1.35E-32	1.24E-30	0	0	0	6.001749	6.434336	6.259292
AT4G04165	49.38939	-5.26382	0.448101	-11.7469	7.32E-32	6.51E-30	0.60238	0	0	5.769679	6.602406	7.200593

AT2G09920	41.43767	-5.26221	0.451237	-11.6617	2.00E-31	1.75E-29	0	0	0	5.890376	6.952811	6.096513
AT4G25580	69.81524	-5.25574	0.387519	-13.5625	6.68E-42	9.48E-40	1.026049	1.006035	1.442988	6.756396	7.63429	6.798784
AT3G30820	39.64125	-5.25049	0.435705	-12.0505	1.93E-33	1.81E-31	0.60238	0	0	6.105138	6.520817	6.315891
AT3G06955	62.63781	-5.23876	0.371641	-14.0963	4.00E-45	6.20E-43	1.35313	1.329167	1.101576	6.963185	6.752923	7.149602
AT2G06490	43.20736	-5.23718	0.429643	-12.1896	3.53E-34	3.42E-32	0.60238	0.588989	0	6.863493	6.024848	6.315891
AT4G06698	69.63737	-5.23185	0.45161	-11.5849	4.92E-31	4.24E-29	1.026049	0	0	5.70532	7.850087	7.096741
AT3G54730	65.51468	-5.22157	0.385839	-13.533	9.98E-42	1.39E-39	0.60238	1.329167	1.442988	7.030399	7.380437	6.569711
AT4G06624	37.16673	-5.20175	0.450909	-11.5361	8.67E-31	7.38E-29	0	0	0	5.861135	6.679627	6.030403
AT4G15242	40.07671	-5.19875	0.426891	-12.1782	4.06E-34	3.90E-32	0.60238	0	0.653468	6.397634	6.024848	6.538339
AT2G13390	47.27083	-5.1958	0.41756	-12.4432	1.52E-35	1.61E-33	1.026049	0.588989	0	6.20161	6.88921	6.538339
AT2G10190	40.42271	-5.18524	0.440477	-11.7719	5.45E-32	4.86E-30	0	0.588989	0	6.397634	6.679627	5.863234
AT4G06643	37.85113	-5.185	0.439575	-11.7955	4.12E-32	3.73E-30	0.60238	0	0	5.974702	6.244072	6.50627
AT2G11120	40.92944	-5.17859	0.444635	-11.6469	2.38E-31	2.08E-29	0	0.588989	0	6.833696	6.244072	5.863234
AT5G44416	34.58656	-5.16456	0.453342	-11.3922	4.57E-30	3.78E-28	0	0	0	6.496028	6.024848	5.785202
AT2G09830	38.18895	-5.15834	0.439752	-11.7301	8.93E-32	7.91E-30	0	0.588989	0	6.313781	6.520817	5.913005
AT3G30770	36.67141	-5.14909	0.442656	-11.6323	2.83E-31	2.44E-29	0.60238	0	0	6.129868	5.766238	6.615524
AT4G08078	34.82985	-5.10606	0.453865	-11.2502	2.31E-29	1.86E-27	0	0	0	5.769679	6.602406	5.913005
AT2G10010	31.79599	-5.1034	0.452454	-11.2794	1.66E-29	1.35E-27	0	0	0	5.831289	6.13862	6.052778
AT5G35146	32.40029	-5.10264	0.452692	-11.2718	1.81E-29	1.47E-27	0	0	0	6.05437	6.244072	5.785202
AT2G15940	45.14248	-5.09754	0.407858	-12.4983	7.62E-36	8.16E-34	0.60238	0.588989	1.101576	6.551997	6.602406	6.334274
AT2G06480	32.32566	-5.09375	0.454619	-11.2044	3.88E-29	3.10E-27	0	0	0	5.70532	6.024848	6.315891
AT1G41775	35.98581	-5.07868	0.456187	-11.1329	8.68E-29	6.84E-27	0	0	0	5.831289	6.752923	5.758224
AT2G23490	31.11747	-5.07518	0.454332	-11.1706	5.68E-29	4.50E-27	0	0	0	6.224753	5.90133	5.785202
AT2G04885	33.49976	-5.06998	0.442341	-11.4617	2.05E-30	1.72E-28	0	0	0.653468	6.313781	5.766238	6.117894
AT2G19840	59.4956	-5.06274	0.390517	-12.9642	1.95E-38	2.32E-36	1.026049	1.006035	1.442988	6.756396	7.33342	6.439912
AT1G42370	31.93208	-5.04903	0.454655	-11.1052	1.18E-28	9.19E-27	0	0	0	5.70532	6.342338	5.937262
AT3G38525	40.87969	-5.04018	0.448665	-11.2337	2.78E-29	2.24E-27	0	0.588989	0	5.890376	7.013727	5.888334
AT1G42360	31.73372	-5.0344	0.45524	-11.0588	1.99E-28	1.52E-26	0	0	0	5.672031	6.342338	5.937262
AT3G43307	40.42915	-5.0057	0.450228	-11.1181	1.02E-28	7.99E-27	0.60238	0	0	5.769679	7.013727	5.937262
AT5G04935	29.95108	-5.00318	0.455476	-10.9845	4.54E-28	3.38E-26	0	0	0	5.861135	6.13862	5.758224
AT2G06800	40.16641	-4.99573	0.46414	-10.7634	5.12E-27	3.63E-25	0	0	0	5.672031	7.128345	5.730732
AT1G37603	34.67878	-4.9866	0.445396	-11.1959	4.27E-29	3.41E-27	0.60238	0	0	5.737858	6.520817	6.030403
AT4G04030	46.39197	-4.97603	0.408536	-12.1801	3.97E-34	3.83E-32	0.60238	1.593003	0	6.756396	6.602406	6.200381

AT2G06845	28.99523	-4.97052	0.457439	-10.866	1.67E-27	1.21E-25	0	0	0	5.637956	5.90133	6.074811
AT1G40119	33.676	-4.93832	0.447725	-11.0298	2.74E-28	2.06E-26	0.60238	0	0	5.603056	6.434336	6.117894
AT2G12650	34.84964	-4.91091	0.436351	-11.2545	2.20E-29	1.78E-27	0.60238	0.588989	0	6.224753	6.342338	5.785202
AT1G10070	2333.689	-4.90686	0.179498	-27.3366	1.56E-164	8.00E-161	7.299229	7.104714	6.809303	12.11449	12.42063	11.84706
AT3G33225	29.30907	-4.90367	0.459687	-10.6674	1.45E-26	1.01E-24	0	0	0	5.861135	6.244072	5.489617
AT2G07460	31.61634	-4.89981	0.447266	-10.955	6.29E-28	4.65E-26	0	0	0.653468	6.028299	6.244072	5.674125
AT3G24542	31.85273	-4.88911	0.462337	-10.5748	3.90E-26	2.63E-24	0	0	0	5.454347	6.602406	5.730732
AT4G07355	27.19726	-4.88652	0.46016	-10.6192	2.43E-26	1.66E-24	0	0	0	5.567291	5.766238	6.007675
AT5G32515	26.2208	-4.86742	0.46032	-10.574	3.93E-26	2.65E-24	0	0	0	5.672031	5.617177	5.913005
AT5G32726	26.80054	-4.85199	0.461537	-10.5127	7.55E-26	5.03E-24	0	0	0	5.492986	5.766238	6.007675
AT4G08593	26.17562	-4.84315	0.460927	-10.5074	7.99E-26	5.30E-24	0	0	0	5.567291	5.766238	5.863234
AT4G05593	27.64581	-4.84197	0.462141	-10.4773	1.10E-25	7.24E-24	0	0	0	5.373819	6.024848	5.961117
AT2G13175	33.59712	-4.8378	0.437895	-11.0479	2.24E-28	1.70E-26	0	0.588989	0.653468	5.800813	6.434336	5.937262
AT4G08091	39.6691	-4.83724	0.447793	-10.8024	3.35E-27	2.40E-25	0	1.006035	0	5.454347	6.88921	6.278406
AT3G31996	27.41408	-4.83014	0.462226	-10.4497	1.47E-25	9.56E-24	0	0	0	5.919035	6.024848	5.387687
AT4G05205	29.71752	-4.81915	0.451259	-10.6794	1.27E-26	8.88E-25	0.60238	0	0	5.530617	6.024848	6.117894
AT3G30827	32.06676	-4.8041	0.452791	-10.61	2.68E-26	1.82E-24	0.60238	0	0	5.861135	6.520817	5.489617
AT3G29650	38.29694	-4.80201	0.472312	-10.167	2.78E-24	1.69E-22	0	0	0	5.197878	7.128345	5.811684
AT5G32408	25.11491	-4.79884	0.462428	-10.3775	3.14E-25	1.99E-23	0	0	0	5.567291	5.617177	5.83769
AT2G06460	28.35956	-4.79684	0.45217	-10.6085	2.72E-26	1.85E-24	0	0	0.653468	5.672031	5.617177	6.180197
AT3G29739	57.84837	-4.78961	0.390425	-12.2677	1.35E-34	1.36E-32	1.35313	1.329167	1.442988	6.417857	7.42597	6.45679
AT2G05915	38.62819	-4.76897	0.412015	-11.5747	5.53E-31	4.75E-29	1.35313	0.588989	0.653468	6.05437	6.244072	6.489965
AT1G41726	27.70688	-4.74247	0.465846	-10.1803	2.43E-24	1.48E-22	0	0	0	5.288529	6.342338	5.615206
AT3G29734	85.491	-4.71669	0.32562	-14.4853	1.50E-47	2.61E-45	1.61959	2.710638	2.324987	7.030399	7.672573	7.405736
AT3G43154	27.55218	-4.69358	0.453614	-10.3471	4.31E-25	2.72E-23	0	0	0.653468	5.530617	6.13862	5.674125
AT4G06542	29.47478	-4.68922	0.456059	-10.2821	8.49E-25	5.29E-23	0	0.588989	0	5.454347	6.434336	5.615206
AT3G42622	30.12547	-4.68846	0.445283	-10.5292	6.34E-26	4.24E-24	0.60238	0	0.653468	5.492986	6.024848	6.180197
AT3G29736	66.0799	-4.68729	0.346655	-13.5215	1.17E-41	1.60E-39	1.35313	2.469294	1.718868	6.740426	7.182411	7.118118
AT3G33081	28.49421	-4.68237	0.469311	-9.97713	1.92E-23	1.12E-21	0	0	0	5.331804	6.520817	5.387687
AT4G07965	28.12576	-4.68082	0.459646	-10.1835	2.35E-24	1.44E-22	0.60238	0	0	5.373819	5.617177	6.334274
AT3G33118	38.3686	-4.67469	0.469012	-9.9671	2.12E-23	1.23E-21	0	0.588989	0	4.826463	6.88921	6.422834
AT3G42658	56.47024	-4.66641	0.352753	-13.2285	6.00E-40	7.63E-38	2.038899	1.593003	1.718868	6.990448	6.520817	6.850789
AT4G33980	112.5413	-4.64721	0.303179	-15.3283	4.95E-53	1.00E-50	2.210244	2.817685	3.173324	7.749708	8.12318	7.379421

AT1G37160	24.07009	-4.627	0.467947	-9.88787	4.70E-23	2.66E-21	0	0	0	5.373819	6.024848	5.352044
AT5G04965	22.85619	-4.62097	0.467617	-9.88196	4.98E-23	2.82E-21	0	0	0	5.454347	5.766238	5.387687
AT3G32070	22.91236	-4.61816	0.467868	-9.87065	5.58E-23	3.14E-21	0	0	0	5.530617	5.766238	5.315499
AT1G67240	31.61531	-4.60297	0.451819	-10.1876	2.25E-24	1.38E-22	0	0.588989	0.653468	5.197878	6.434336	6.096513
AT5G05005	21.76739	-4.57281	0.469111	-9.74782	1.88E-22	1.03E-20	0	0	0	5.414645	5.617177	5.387687
AT2G05950	37.81882	-4.57076	0.409015	-11.175	5.40E-29	4.30E-27	1.35313	1.006035	0.653468	6.028299	6.602406	6.007675
AT2G19850	33.81868	-4.56684	0.417073	-10.9497	6.66E-28	4.91E-26	1.026049	1.329167	0	6.028299	6.244072	5.961117
AT4G05275	31.92518	-4.5658	0.479211	-9.52773	1.61E-21	8.29E-20	0	0	0	4.561494	6.679627	6.096513
AT2G06340	21.96751	-4.56512	0.470046	-9.71206	2.68E-22	1.45E-20	0	0	0	5.197878	5.617177	5.615206
AT3G33085	21.89249	-4.54209	0.470222	-9.65946	4.48E-22	2.39E-20	0	0	0	5.288529	5.766238	5.352044
AT2G13310	27.12572	-4.53744	0.462591	-9.80875	1.03E-22	5.75E-21	0.60238	0	0	5.800813	6.244072	5.074348
AT2G04320	23.00483	-4.53707	0.458845	-9.88804	4.69E-23	2.66E-21	0.60238	0	0	5.637956	5.450922	5.553778
AT5G24240	44.03725	-4.52354	0.445929	-10.1441	3.52E-24	2.12E-22	0.60238	1.329167	0.653468	5.197878	6.602406	7.030642
AT1G40115	23.79671	-4.5161	0.472886	-9.55007	1.30E-21	6.73E-20	0	0	0	5.414645	6.13862	5.029925
AT3G29700	20.11594	-4.494	0.472835	-9.50438	2.01E-21	1.03E-19	0	0	0	5.637956	5.046837	5.352044
AT4G05592	22.87071	-4.47739	0.475042	-9.42525	4.29E-21	2.16E-19	0	0	0	4.826463	5.90133	5.702706
AT3G28193	23.23429	-4.47699	0.460796	-9.71577	2.58E-22	1.41E-20	0	0	0.653468	5.603056	5.766238	5.278004
AT3G43571	24.50436	-4.47529	0.461955	-9.68771	3.40E-22	1.83E-20	0	0	0.653468	5.603056	6.024848	5.15929
AT2G06904	28.65092	-4.45709	0.480874	-9.26873	1.88E-20	9.17E-19	0	0	0	4.561494	6.602406	5.758224
AT3G32377	19.38044	-4.44933	0.473967	-9.38744	6.15E-21	3.07E-19	0	0	0	5.331804	5.046837	5.522054
AT1G39110	24.92299	-4.4474	0.465644	-9.55106	1.28E-21	6.68E-20	0	0.588989	0	4.942684	6.024848	5.811684
AT3G62475	24.12273	-4.42255	0.479042	-9.23206	2.65E-20	1.27E-18	0	0	0	5.861135	6.024848	4.614569
AT2G05000	20.59081	-4.42058	0.475967	-9.28758	1.58E-20	7.74E-19	0	0	0	5.70532	5.450922	4.936752
AT4G03760	37.83785	-4.41538	0.482752	-9.14626	5.89E-20	2.74E-18	0	0	0.653468	4.561494	5.90133	7.190538
AT2G11775	23.09106	-4.41164	0.477414	-9.24069	2.45E-20	1.18E-18	0	0	0	4.764642	6.13862	5.456434
AT5G29562	21.74142	-4.40719	0.463201	-9.51463	1.82E-21	9.37E-20	0.60238	0	0	5.243916	5.617177	5.522054
AT4G06544	21.33826	-4.39207	0.476667	-9.21413	3.14E-20	1.50E-18	0	0	0	4.826463	5.90133	5.42247
AT5G04985	18.99296	-4.38776	0.47556	-9.22651	2.80E-20	1.34E-18	0	0	0	5.454347	5.262981	5.117444
AT4G06575	23.04412	-4.37382	0.478482	-9.14104	6.19E-20	2.87E-18	0	0	0	4.942684	6.244072	5.117444
AT4G06702	21.13637	-4.37323	0.477099	-9.1663	4.90E-20	2.31E-18	0	0	0	5.373819	5.90133	4.837144
AT2G05695	18.10428	-4.34751	0.476927	-9.11567	7.82E-20	3.61E-18	0	0	0	5.197878	5.046837	5.387687
AT4G06586	20.73966	-4.34425	0.477852	-9.0912	9.80E-20	4.49E-18	0	0	0	5.288529	5.90133	4.837144
AT5G42900	81.18587	-4.34403	0.3258	-13.3334	1.48E-40	1.94E-38	2.74425	2.817685	1.950366	7.304685	7.672573	6.825021

AT5G15420	20.36591	-4.33791	0.465303	-9.32277	1.13E-20	5.60E-19	0	0.588989	0	5.288529	5.450922	5.387687
AT2G12910	28.40033	-4.32629	0.479988	-9.01333	2.00E-19	8.99E-18	0	0.588989	0	4.700053	5.450922	6.688789
AT2G13463	21.98262	-4.32261	0.473625	-9.12666	7.06E-20	3.27E-18	0.60238	0	0	6.028299	4.089834	5.702706
AT2G11410	17.63863	-4.32053	0.47826	-9.03385	1.66E-19	7.51E-18	0	0	0	5.331804	4.792505	5.352044
AT2G10780	19.84175	-4.31344	0.479607	-8.9937	2.39E-19	1.07E-17	0	0	0	4.700053	5.617177	5.553778
AT2G11115	18.35271	-4.3055	0.477724	-9.01254	2.01E-19	9.04E-18	0	0	0	5.197878	5.450922	5.029925
AT1G08630	510.0416	-4.29614	0.258409	-16.6254	4.57E-62	1.23E-59	5.73715	5.333673	4.803662	9.630169	10.44096	9.573871
AT4G06540	20.36028	-4.29246	0.466512	-9.20117	3.54E-20	1.68E-18	0	0	0.653468	5.197878	5.617177	5.278004
AT2G13431	54.83296	-4.2908	0.350277	-12.2497	1.69E-34	1.67E-32	2.74425	1.329167	1.950366	6.907064	6.434336	6.850789
AT3G00620	69.94198	-4.28644	0.324198	-13.2217	6.58E-40	8.32E-38	2.363383	2.710638	2.149802	6.803271	7.47011	6.913264
AT5G30480	23.95814	-4.27301	0.487077	-8.77275	1.74E-18	7.42E-17	0	0	0	4.324977	5.617177	6.278406
AT5G30852	20.37456	-4.25553	0.481951	-8.82979	1.05E-18	4.52E-17	0	0	0	4.561494	5.90133	5.387687
AT2G00560	24.45149	-4.25358	0.462635	-9.19426	3.78E-20	1.79E-18	0	0.588989	0.653468	4.826463	6.024848	5.785202
AT4G15245	17.00272	-4.25258	0.48126	-8.83636	9.89E-19	4.28E-17	0	0	0	5.454347	4.483533	5.278004
AT3G33160	16.8562	-4.23937	0.480029	-8.83148	1.03E-18	4.46E-17	0	0	0	5.101148	5.046837	5.199956
AT2G12040	20.537	-4.23633	0.457898	-9.25168	2.21E-20	1.07E-18	0.60238	0	0.653468	5.454347	5.262981	5.42247
AT2G11340	17.53119	-4.22449	0.480279	-8.79591	1.42E-18	6.07E-17	0	0	0	4.942684	5.450922	5.074348
AT3G46487	43.00874	-4.22241	0.461252	-9.15423	5.47E-20	2.56E-18	1.35313	0	1.101576	4.997462	7.284819	6.138962
AT5G01080	22.36107	-4.21792	0.448412	-9.40635	5.14E-21	2.57E-19	0.60238	0.588989	0.653468	5.672031	5.450922	5.352044
AT1G38167	17.41709	-4.21117	0.480681	-8.76083	1.94E-18	8.23E-17	0	0	0	5.050236	5.450922	4.936752
AT3G45270	23.04108	-4.2095	0.460522	-9.14073	6.20E-20	2.88E-18	0	0	1.101576	5.331804	6.024848	5.117444
AT5G04995	16.57182	-4.208	0.481002	-8.74841	2.16E-18	9.14E-17	0	0	0	5.197878	5.046837	5.029925
AT2G10490	16.89528	-4.20634	0.480801	-8.74859	2.16E-18	9.14E-17	0	0	0	5.101148	5.262981	4.984091
AT3G31356	15.96399	-4.13833	0.484105	-8.54842	1.25E-17	5.09E-16	0	0	0	5.373819	4.792505	4.887807
AT4G08720	19.03769	-4.12016	0.46358	-8.8877	6.24E-19	2.73E-17	0	1.006035	0	5.331804	5.046837	5.42247
AT2G10285	17.54476	-4.11774	0.4863	-8.46749	2.51E-17	1.00E-15	0	0	0	5.567291	5.262981	4.488922
AT4G08710	18.24248	-4.1122	0.473717	-8.68071	3.93E-18	1.65E-16	0.60238	0	0	4.826463	5.450922	5.315499
AT2G05752	15.43785	-4.09494	0.484311	-8.45518	2.79E-17	1.10E-15	0	0	0	4.997462	5.046837	4.936752
AT3G32295	16.61443	-4.08116	0.486583	-8.3874	4.97E-17	1.96E-15	0	0	0	4.486882	5.262981	5.387687
AT3G42256	36.48872	-4.06956	0.39205	-10.3802	3.05E-25	1.94E-23	1.61959	1.329167	1.718868	5.947137	6.602406	5.83769
AT5G32517	18.96544	-4.05751	0.487966	-8.31514	9.16E-17	3.51E-15	0	0	0	4.764642	6.024848	4.614569
AT5G35048	17.16725	-4.05477	0.476444	-8.5105	1.73E-17	7.01E-16	0.60238	0	0	5.454347	5.046837	4.837144
AT5G33240	14.53641	-4.04131	0.486936	-8.29947	1.05E-16	3.99E-15	0	0	0	5.050236	4.483533	5.117444

AT4G05025	14.49124	-4.02323	0.48681	-8.26446	1.40E-16	5.32E-15	0	0	0	4.885743	4.792505	5.029925
AT5G28235	15.78096	-4.01915	0.489849	-8.20488	2.31E-16	8.56E-15	0	0	0	5.637956	4.483533	4.673514
AT3G29648	20.27123	-4.01229	0.480777	-8.34544	7.09E-17	2.74E-15	0	0.588989	0	4.408199	6.024848	5.239508
AT1G05147	55.60295	-3.98883	0.381352	-10.4597	1.32E-25	8.65E-24	2.628119	2.331572	1.101576	6.623396	7.33342	6.052778
AT5G32473	17.04413	-3.98698	0.489181	-8.15031	3.63E-16	1.33E-14	0	0	0	4.408199	5.766238	4.887807
AT3G14670	14.48947	-3.98536	0.488477	-8.15876	3.38E-16	1.24E-14	0	0	0	5.197878	4.792505	4.673514
AT2G11110	17.07045	-3.9852	0.489207	-8.14624	3.75E-16	1.37E-14	0	0	0	4.885743	5.766238	4.421742
AT2G14570	14.41308	-3.96786	0.490757	-8.08518	6.21E-16	2.23E-14	0	0	0	4.885743	4.089834	5.42247
AT3G43128	15.40367	-3.96692	0.488408	-8.12214	4.58E-16	1.66E-14	0	0	0	4.764642	5.450922	4.614569
AT3G24517	14.38813	-3.96366	0.488359	-8.11629	4.81E-16	1.74E-14	0	0	0	4.942684	5.046837	4.673514
AT5G32490	13.77107	-3.95918	0.489262	-8.09215	5.86E-16	2.11E-14	0	0	0	4.885743	4.483533	5.074348
AT2G06930	15.45984	-3.95678	0.488991	-8.09172	5.88E-16	2.11E-14	0	0	0	4.885743	5.450922	4.488922
AT3G30720	253.0322	-3.95131	0.190724	-20.7174	2.41E-95	2.21E-92	4.804643	4.869612	4.917441	9.089837	8.795846	8.805598
AT2G21460	28.72487	-3.94649	0.44643	-8.84011	9.56E-19	4.15E-17	1.026049	1.593003	0.653468	5.414645	5.046837	6.585144
AT4G06566	35.97933	-3.94205	0.433315	-9.09743	9.25E-20	4.25E-18	1.026049	2.009043	0.653468	5.197878	6.822676	6.007675
AT4G06748	15.61679	-3.93857	0.493876	-7.97482	1.53E-15	5.32E-14	0	0	0	5.737858	3.546624	4.984091
AT4G02865	16.11309	-3.9383	0.478878	-8.22401	1.97E-16	7.35E-15	0	0	0.653468	4.700053	5.262981	5.117444
AT5G31302	14.21786	-3.93801	0.489184	-8.05015	8.27E-16	2.95E-14	0	0	0	4.942684	5.046837	4.614569
AT4G05556	14.10552	-3.93779	0.489017	-8.05245	8.12E-16	2.90E-14	0	0	0	4.700053	5.046837	4.837144
AT3G42083	23.06496	-3.93021	0.472143	-8.3242	8.49E-17	3.27E-15	0.60238	0.588989	0.653468	6.20161	5.450922	4.488922
AT2G06002	66.05134	-3.92201	0.306529	-12.7949	1.75E-37	2.00E-35	2.951747	3.010545	2.622111	7.144016	6.520817	7.200593
AT3G02515	111.4939	-3.92182	0.242098	-16.1993	5.10E-59	1.27E-56	3.632365	3.534563	3.825555	7.725575	7.74622	7.717232
AT2G15750	18.45266	-3.91664	0.461208	-8.49213	2.03E-17	8.14E-16	0.60238	0.588989	0.653468	5.567291	5.046837	4.984091
AT2G06965	13.58804	-3.91306	0.491656	-7.95893	1.74E-15	6.02E-14	0	0	0	5.243916	4.089834	4.887807
AT4G02795	170.0831	-3.89714	0.208144	-18.7233	3.20E-78	1.68E-75	4.406711	4.181303	4.434696	8.39498	8.203818	8.383934
AT2G13870	20.2952	-3.89557	0.495855	-7.85627	3.96E-15	1.33E-13	0	0	0	3.933736	6.244072	5.074348
AT5G32566	17.12701	-3.89184	0.495343	-7.85687	3.94E-15	1.32E-13	0	0	0	3.933736	5.262981	5.702706
AT2G12320	14.83668	-3.88991	0.49076	-7.9263	2.26E-15	7.74E-14	0	0	0	4.632436	5.450922	4.553113
AT2G11640	13.14615	-3.8875	0.491196	-7.91436	2.49E-15	8.50E-14	0	0	0	4.997462	4.483533	4.784636
AT3G30746	16.30511	-3.8831	0.492299	-7.88767	3.08E-15	1.05E-13	0	0	0	4.700053	5.766238	4.35128
AT2G01026	16.23818	-3.8732	0.481717	-8.04042	8.95E-16	3.18E-14	0	0	0.653468	4.486882	5.450922	5.074348
AT2G26630	16.58796	-3.8691	0.481458	-8.03623	9.26E-16	3.28E-14	0	0	0.653468	4.826463	5.617177	4.614569
AT4G25530	104.4545	-3.85788	0.508022	-7.59391	3.10E-14	9.74E-13	0	0	0	6.533581	8.611075	7.190538

AT3G32300	14.79937	-3.85488	0.493318	-7.81418	5.53E-15	1.83E-13	0	0	0	4.142579	5.262981	5.15929
AT1G42367	13.01672	-3.84619	0.491876	-7.81943	5.31E-15	1.76E-13	0	0	0	4.632436	4.792505	4.837144
AT4G06511	14.43997	-3.83202	0.492527	-7.78032	7.23E-15	2.37E-13	0	0	0	4.486882	5.450922	4.553113
AT4G07570	12.66518	-3.83158	0.492754	-7.77585	7.49E-15	2.45E-13	0	0	0	4.700053	4.483533	4.936752
AT2G04000	13.22607	-3.82215	0.492338	-7.76327	8.28E-15	2.71E-13	0	0	0	4.632436	5.046837	4.614569
AT1G36540	13.19799	-3.81763	0.492496	-7.75159	9.08E-15	2.95E-13	0	0	0	4.561494	5.046837	4.673514
AT5G32775	13.19799	-3.81763	0.492496	-7.75159	9.08E-15	2.95E-13	0	0	0	4.561494	5.046837	4.673514
AT5G32241	13.284	-3.79722	0.493645	-7.6922	1.45E-14	4.65E-13	0	0	0	4.324977	5.046837	4.887807
AT3G30823	13.54952	-3.79132	0.493383	-7.68434	1.54E-14	4.94E-13	0	0	0	4.486882	5.262981	4.553113
AT2G07240	19.20523	-3.78757	0.47771	-7.9286	2.22E-15	7.62E-14	0.60238	0	0.653468	4.486882	6.024848	4.887807
AT2G05015	13.05266	-3.77082	0.496612	-7.5931	3.12E-14	9.77E-13	0	0	0	4.486882	4.089834	5.387687
AT3G29615	14.90283	-3.76989	0.486622	-7.74705	9.40E-15	3.05E-13	0.60238	0	0	4.324977	5.046837	5.278004
AT1G23930	12.94603	-3.76599	0.495815	-7.59555	3.06E-14	9.62E-13	0	0	0	5.243916	4.483533	4.35128
AT3G06095	37.39887	-3.76294	0.361773	-10.4014	2.44E-25	1.55E-23	2.038899	2.179302	1.950366	6.17809	6.342338	6.007675
AT3G57520	5030.653	-3.74334	0.193142	-19.3813	1.11E-83	6.48E-81	9.395853	9.368478	9.174815	12.92353	13.64167	12.91581
AT3G60176	15.3414	-3.73465	0.496711	-7.51876	5.53E-14	1.69E-12	0	0	0	4.408199	5.766238	4.277199
AT4G14120	3226.741	-3.73234	0.153923	-24.2482	6.91E-130	1.18E-126	8.857266	8.412889	8.905113	12.68102	12.5445	12.43602
AT2G01037	24.97553	-3.71803	0.440845	-8.43387	3.34E-17	1.32E-15	0.60238	1.006035	1.718868	4.885743	6.13862	5.615206
AT2G09910	21.12784	-3.71715	0.485248	-7.66032	1.85E-14	5.92E-13	0.60238	0.588989	0	4.324977	6.342338	4.784636
AT5G35608	14.9926	-3.70958	0.491723	-7.54406	4.56E-14	1.40E-12	0	0.588989	0	4.324977	4.483533	5.644966
AT3G31910	16.35862	-3.70913	0.466993	-7.94258	1.98E-15	6.84E-14	1.026049	0.588989	0	4.885743	5.262981	4.984091
AT3G53910	12.98254	-3.70054	0.49604	-7.46016	8.64E-14	2.61E-12	0	0	0	4.324977	5.262981	4.488922
AT4G01980	26.78607	-3.7002	0.40324	-9.17618	4.47E-20	2.11E-18	1.61959	1.593003	1.442988	5.492986	5.90133	5.730732
AT4G07937	12.43264	-3.69817	0.495991	-7.45612	8.91E-14	2.67E-12	0	0	0	4.324977	5.046837	4.614569
AT3G43000	14.00139	-3.69768	0.490145	-7.54405	4.56E-14	1.40E-12	0.60238	0	0	4.408199	4.483533	5.42247
AT4G14130	8997.816	-3.69539	0.172399	-21.435	6.30E-102	6.47E-99	10.38922	9.824209	10.4082	14.11143	14.14938	13.82529
AT2G06335	11.55929	-3.68583	0.496655	-7.4213	1.16E-13	3.45E-12	0	0	0	4.486882	4.483533	4.784636
AT3G42100	18.02225	-3.66734	0.451208	-8.12781	4.37E-16	1.59E-14	0.60238	0.588989	1.442988	5.150324	5.046837	5.315499
AT4G09425	32.02076	-3.63948	0.385077	-9.4513	3.35E-21	1.70E-19	1.35313	2.331572	1.950366	5.737858	5.90133	6.200381
AT2G12830	11.07656	-3.62642	0.498017	-7.28172	3.30E-13	9.41E-12	0	0	0	4.561494	4.483533	4.553113
AT4G06592	15.68406	-3.6238	0.491399	-7.37446	1.65E-13	4.84E-12	0.60238	0	0	4.142579	5.766238	4.614569
AT4G03840	11.96876	-3.62289	0.499047	-7.25962	3.88E-13	1.10E-11	0	0	0	4.041933	4.792505	4.936752
AT4G07600	12.00608	-3.6211	0.498134	-7.26934	3.61E-13	1.03E-11	0	0	0	4.561494	5.046837	4.199107

AT2G12840	11.04847	-3.62107	0.498187	-7.26851	3.63E-13	1.03E-11	0	0	0	4.486882	4.483533	4.614569
AT3G31908	11.40001	-3.61939	0.497984	-7.26809	3.65E-13	1.03E-11	0	0	0	4.408199	4.792505	4.488922
AT3G25719	15.79431	-3.60667	0.481523	-7.49014	6.88E-14	2.08E-12	0	0.588989	0.653468	4.324977	5.617177	4.784636
AT4G04270	15.1132	-3.60248	0.501248	-7.18701	6.62E-13	1.84E-11	0	0	0	4.700053	5.766238	3.728968
AT1G80160	123.5302	-3.60117	0.264419	-13.6192	3.08E-42	4.44E-40	4.048462	4.098802	3.882709	7.86467	8.229725	7.370542
AT2G15520	13.559	-3.59686	0.489827	-7.34312	2.09E-13	6.06E-12	0	0	0.653468	4.700053	5.262981	4.277199
AT2G15810	235.6963	-3.5939	0.460319	-7.80741	5.84E-15	1.93E-13	3.632365	4.596507	2.622111	8.41524	9.408366	8.477876
AT5G29075	15.51814	-3.59011	0.471895	-7.60786	2.79E-14	8.79E-13	0.60238	0.588989	0.653468	4.486882	5.262981	5.074348
AT4G06507	11.69538	-3.57522	0.499355	-7.15968	8.09E-13	2.23E-11	0	0	0	4.142579	5.046837	4.488922
AT1G41680	10.7641	-3.56905	0.49957	-7.14425	9.05E-13	2.48E-11	0	0	0	4.632436	4.483533	4.35128
AT2G01028	14.87578	-3.55186	0.483162	-7.35128	1.96E-13	5.72E-12	0	0.588989	0.653468	4.236658	5.450922	4.837144
AT2G11950	12.15926	-3.54185	0.500529	-7.07621	1.48E-12	4.01E-11	0	0	0	4.408199	5.262981	4.028969
AT2G06245	10.88919	-3.5333	0.500216	-7.06354	1.62E-12	4.38E-11	0	0	0	4.408199	4.792505	4.277199
AT5G45570	14.76882	-3.48683	0.482126	-7.23219	4.75E-13	1.33E-11	0	0.588989	1.101576	5.567291	4.089834	4.673514
AT3G30700	10.14094	-3.47951	0.501681	-6.9357	4.04E-12	1.05E-10	0	0	0	4.324977	4.483533	4.421742
AT3G30582	26.62281	-3.47232	0.461453	-7.52476	5.28E-14	1.61E-12	1.026049	1.815969	0.653468	4.324977	6.434336	5.702706
AT4G06533	11.04238	-3.46518	0.502037	-6.90224	5.12E-12	1.32E-10	0	0	0	4.236658	5.046837	4.116545
AT1G61510	12.70073	-3.46353	0.505071	-6.85752	7.01E-12	1.78E-10	0	0	0	5.331804	4.792505	3.487532
AT2G15510	13.44246	-3.46242	0.477546	-7.25045	4.15E-13	1.17E-11	1.026049	0	0.653468	5.050236	4.483533	4.730144
AT1G68050	100.0817	-3.45475	0.267594	-12.9104	3.93E-38	4.59E-36	3.368482	4.055712	4.229687	7.804499	7.380437	7.414402
AT2G01034	15.86171	-3.44894	0.479273	-7.19619	6.19E-13	1.72E-11	0	0.588989	1.101576	4.236658	5.617177	4.837144
AT2G23480	10.99265	-3.44493	0.494298	-6.96934	3.18E-12	8.36E-11	0.60238	0	0	4.561494	4.483533	4.488922
AT3G31450	9.675296	-3.44029	0.503022	-6.83923	7.96E-12	2.01E-10	0	0	0	4.561494	4.089834	4.35128
AT2G05135	13.13373	-3.4326	0.495789	-6.92351	4.41E-12	1.14E-10	0	0.588989	0	4.408199	5.450922	4.028969
AT3G06715	11.57951	-3.43086	0.504105	-6.80584	1.00E-11	2.53E-10	0	0	0	4.764642	5.046837	3.613295
AT3G15440	39.88521	-3.40678	0.364874	-9.33685	9.92E-21	4.92E-19	3.045281	1.815969	2.324987	6.514927	6.244072	5.937262
AT2G10965	12.10133	-3.40574	0.505005	-6.74398	1.54E-11	3.81E-10	0	0	0	4.700053	5.262981	3.487532
AT2G13160	10.09576	-3.38937	0.503777	-6.72792	1.72E-11	4.22E-10	0	0	0	4.041933	4.792505	4.277199
AT3G43680	12.16433	-3.38845	0.501075	-6.76236	1.36E-11	3.38E-10	0.60238	0	0	4.236658	3.546624	5.489617
AT2G13430	26.26873	-3.37964	0.413929	-8.16477	3.22E-16	1.18E-14	2.501815	1.593003	1.101576	6.079977	5.046837	5.702706
AT3G44006	9.714375	-3.36251	0.50476	-6.6616	2.71E-11	6.54E-10	0	0	0	4.561494	4.483533	3.935733
AT3G42535	13.40828	-3.35623	0.480371	-6.98674	2.81E-12	7.44E-11	1.026049	0	0.653468	4.826463	5.046837	4.35128
AT4G00130	236.0499	-3.3549	0.200052	-16.7702	4.03E-63	1.14E-60	5.179468	5.259657	5.513797	8.642768	8.571102	9.028

AT2G06906	11.49726	-3.34902	0.498526	-6.71785	1.84E-11	4.50E-10	0	0	0.653468	3.816762	4.792505	4.887807
AT3G30690	9.289575	-3.31808	0.505436	-6.56479	5.21E-11	1.23E-09	0	0	0	4.324977	4.483533	4.028969
AT2G11522	8.938036	-3.31775	0.50568	-6.56097	5.35E-11	1.26E-09	0	0	0	4.408199	4.089834	4.199107
AT4G02960	14.94941	-3.31341	0.466718	-7.09939	1.25E-12	3.41E-11	0.60238	1.006035	1.101576	4.408199	5.046837	5.15929
AT4G08190	10.27703	-3.31241	0.50567	-6.55054	5.73E-11	1.34E-09	0	0	0	3.933736	5.046837	4.028969
AT2G00450	33.4114	-3.3105	0.361752	-9.1513	5.63E-20	2.63E-18	1.844425	2.710638	2.481185	5.919035	5.90133	6.138962
AT4G06682	10.54843	-3.30348	0.498142	-6.6316	3.32E-11	7.96E-10	0	0.588989	0	4.142579	4.792505	4.35128
AT4G09316	16.07142	-3.30218	0.476161	-6.935	4.06E-12	1.06E-10	0.60238	0.588989	1.101576	4.486882	5.766238	4.35128
AT2G06562	10.24895	-3.30057	0.506002	-6.52284	6.90E-11	1.59E-09	0	0	0	3.816762	5.046837	4.116545
AT1G49090	15.05004	-3.2843	0.487867	-6.73196	1.67E-11	4.11E-10	1.026049	0.588989	0	3.816762	5.617177	4.837144
AT4G33150	1266.716	-3.28257	0.19358	-16.9572	1.70E-64	5.09E-62	7.95229	7.766877	7.5529	11.03646	11.56269	10.8358
AT5G32488	9.006964	-3.26456	0.506611	-6.44391	1.16E-10	2.61E-09	0	0	0	3.933736	4.483533	4.277199
AT2G19800	2490.611	-3.26416	0.226695	-14.3989	5.26E-47	8.94E-45	8.031574	8.964762	8.969188	12.21036	12.3662	11.84104
AT3G43310	9.813151	-3.26355	0.507205	-6.43437	1.24E-10	2.77E-09	0	0	0	3.549829	4.792505	4.488922
AT5G31981	9.603799	-3.26009	0.507677	-6.42159	1.35E-10	3.00E-09	0	0	0	3.549829	4.483533	4.730144
AT3G33151	19.11477	-3.25342	0.495879	-6.56091	5.35E-11	1.26E-09	1.026049	0.588989	0	4.142579	6.342338	4.116545
AT4G04293	32.58354	-3.25082	0.400544	-8.11601	4.82E-16	1.74E-14	1.844425	2.710638	2.149802	6.588138	5.450922	5.553778
AT4G03950	11.74245	-3.24952	0.494367	-6.5731	4.93E-11	1.16E-09	0.60238	0.588989	0	3.816762	4.792505	4.936752
AT3G33595	26.74328	-3.24917	0.495393	-6.55876	5.43E-11	1.27E-09	0.60238	1.329167	0.653468	4.700053	6.88921	4.028969
AT5G32516	12.63452	-3.24693	0.509995	-6.3666	1.93E-10	4.23E-09	0	0	0	2.797163	5.046837	5.278004
AT5G35792	8.388139	-3.2423	0.507702	-6.38623	1.70E-10	3.75E-09	0	0	0	4.408199	3.546624	4.35128
AT4G03795	9.936485	-3.23479	0.507355	-6.37578	1.82E-10	3.99E-09	0	0	0	3.933736	5.046837	3.836051
AT5G36900	12.06334	-3.23407	0.498343	-6.48965	8.60E-11	1.96E-09	1.026049	0	0	4.041933	4.089834	5.352044
AT2G15800	58.32152	-3.2178	0.411457	-7.82051	5.26E-15	1.74E-13	3.133118	3.098096	1.950366	5.831289	7.709866	6.096513
AT1G30784	12.82598	-3.21544	0.477545	-6.73328	1.66E-11	4.09E-10	0.60238	1.006035	0.653468	4.942684	4.792505	4.277199
AT4G07850	48.61775	-3.21366	0.401967	-7.99483	1.30E-15	4.55E-14	2.210244	3.534563	1.718868	5.70532	6.952811	6.674431
AT2G06140	8.314879	-3.20406	0.50797	-6.30758	2.83E-10	6.15E-09	0	0	0	4.041933	4.089834	4.277199
AT4G33465	31.17081	-3.19749	0.376341	-8.49627	1.96E-17	7.87E-16	2.628119	2.331572	1.718868	5.800813	6.244072	5.553778
AT2G16670	11.88977	-3.19681	0.495739	-6.44858	1.13E-10	2.53E-09	0	1.006035	0	4.700053	5.046837	3.836051
AT3G42445	9.852231	-3.19373	0.508392	-6.28202	3.34E-10	7.20E-09	0	0	0	3.549829	5.046837	4.116545
AT3G32897	10.43021	-3.18561	0.50874	-6.26175	3.81E-10	8.14E-09	0	0	0	3.689461	5.262981	3.836051
AT2G07550	16.45917	-3.18261	0.471511	-6.74983	1.48E-11	3.67E-10	1.026049	0.588989	1.718868	5.050236	3.546624	5.730732
AT1G40077	8.228861	-3.17462	0.508568	-6.24228	4.31E-10	9.17E-09	0	0	0	4.324977	4.089834	3.935733

AT3G35707	9.822382	-3.16904	0.508915	-6.22705	4.75E-10	1.00E-08	0	0	0	4.142579	5.046837	3.487532
AT2G12210	10.6865	-3.16843	0.50957	-6.21786	5.04E-10	1.06E-08	0	0	0	3.222033	5.262981	4.277199
AT3G43575	8.247714	-3.16304	0.509518	-6.20791	5.37E-10	1.13E-08	0	0	0	3.933736	3.546624	4.673514
AT4G22415	10.2087	-3.15862	0.494199	-6.39139	1.64E-10	3.63E-09	0.60238	0	0.653468	4.486882	4.483533	4.199107
AT4G37220	215.8884	-3.14887	0.215542	-14.6091	2.46E-48	4.36E-46	5.179468	5.315522	5.420381	8.321742	9.02025	8.423107
AT1G38194	10.23186	-3.13089	0.50981	-6.1413	8.18E-10	1.70E-08	0	0	0	3.549829	5.262981	3.836051
AT4G06590	8.875771	-3.12993	0.50921	-6.14664	7.91E-10	1.65E-08	0	0	0	3.933736	4.792505	3.728968
AT1G36763	8.382043	-3.12542	0.50931	-6.13657	8.43E-10	1.75E-08	0	0	0	4.142579	4.483533	3.728968
AT4G06712	8.737915	-3.12537	0.510283	-6.12479	9.08E-10	1.88E-08	0	0	0	4.764642	4.089834	3.487532
AT4G04945	7.736897	-3.09772	0.510404	-6.06916	1.29E-09	2.62E-08	0	0	0	3.933736	3.546624	4.488922
AT4G06684	7.775976	-3.09708	0.509869	-6.07427	1.25E-09	2.54E-08	0	0	0	3.933736	4.089834	4.116545
AT3G42996	9.858456	-3.0859	0.497257	-6.20585	5.44E-10	1.14E-08	0.60238	0.588989	0	3.933736	4.483533	4.553113
AT1G36795	8.127516	-3.08559	0.509928	-6.05104	1.44E-09	2.92E-08	0	0	0	3.816762	4.483533	3.935733
AT2G06425	8.205108	-3.06739	0.511687	-5.99467	2.04E-09	4.08E-08	0	0	0	4.885743	2.665087	4.028969
AT5G32420	7.633789	-3.06712	0.510344	-6.00991	1.86E-09	3.73E-08	0	0	0	4.041933	4.089834	3.935733
AT4G33467	35.50267	-3.06525	0.356536	-8.59732	8.16E-18	3.35E-16	2.951747	2.817685	1.950366	6.001749	6.342338	5.785202
AT5G28810	7.424436	-3.06319	0.510779	-5.99708	2.01E-09	4.02E-08	0	0	0	4.041933	3.546624	4.277199
AT4G06578	7.577619	-3.04987	0.510674	-5.97224	2.34E-09	4.66E-08	0	0	0	3.816762	4.089834	4.116545
AT3G43290	7.310334	-3.04032	0.511101	-5.94858	2.70E-09	5.30E-08	0	0	0	4.236658	3.546624	4.028969
AT2G12810	7.282249	-3.03971	0.511092	-5.94748	2.72E-09	5.34E-08	0	0	0	4.142579	3.546624	4.116545
AT2G12240	8.735346	-3.03109	0.511316	-5.92801	3.07E-09	5.99E-08	0	0	0	3.222033	4.792505	4.199107
AT2G23500	8.463514	-3.03067	0.507439	-5.97248	2.34E-09	4.65E-08	0.60238	0	0	4.486882	2.665087	4.614569
AT5G33387	7.549534	-3.0291	0.511088	-5.92677	3.09E-09	6.02E-08	0	0	0	3.689461	4.089834	4.199107
AT3G29738	7.746128	-3.01118	0.51159	-5.88592	3.96E-09	7.63E-08	0	0	0	4.408199	4.089834	3.487532
AT5G30470	14.70566	-2.99926	0.511971	-5.85825	4.68E-09	8.94E-08	0	0.588989	0	2.192331	5.046837	5.730732
AT1G42110	7.083891	-2.99911	0.511679	-5.86131	4.59E-09	8.78E-08	0	0	0	4.041933	3.546624	4.116545
AT5G41080	238.0994	-2.99889	0.273804	-10.9527	6.45E-28	4.76E-26	5.442744	5.43803	5.601531	8.037996	9.373929	8.542549
AT2G16000	8.386617	-2.99624	0.507965	-5.89851	3.67E-09	7.10E-08	0	0	0.653468	4.700053	2.665087	4.35128
AT4G08013	14.92013	-2.99113	0.513955	-5.81983	5.89E-09	1.10E-07	0	0	0	1.757146	5.766238	5.117444
AT5G32483	7.237074	-2.97893	0.511697	-5.82167	5.83E-09	1.09E-07	0	0	0	3.816762	4.089834	3.935733
AT3G23085	14.09819	-2.97348	0.4626	-6.42774	1.30E-10	2.89E-09	0.60238	1.815969	1.101576	5.150324	4.483533	4.673514
AT5G38595	9.161342	-2.97187	0.513615	-5.78617	7.20E-09	1.34E-07	0	0	0	4.408199	0	5.15929
AT3G30780	7.493364	-2.96289	0.512289	-5.78364	7.31E-09	1.35E-07	0	0	0	3.395221	4.089834	4.35128

AT5G33251	10.15707	-2.94244	0.507741	-5.79516	6.83E-09	1.27E-07	0	0	0.653468	3.549829	5.262981	3.728968
AT4G03825	8.110426	-2.94031	0.512266	-5.73981	9.48E-09	1.73E-07	0	0	0	3.549829	4.792505	3.613295
AT1G41840	7.066801	-2.93926	0.512234	-5.73811	9.57E-09	1.75E-07	0	0	0	3.816762	4.089834	3.836051
AT4G07936	7.038716	-2.92863	0.512401	-5.71551	1.09E-08	1.98E-07	0	0	0	3.689461	4.089834	3.935733
AT1G24260	10.70713	-2.92776	0.483004	-6.06157	1.35E-09	2.74E-08	1.026049	0.588989	1.101576	4.826463	3.546624	4.614569
AT4G06588	9.398176	-2.91928	0.500757	-5.82972	5.55E-09	1.04E-07	0.60238	0	0.653468	3.933736	4.792505	3.935733
AT5G34834	10.54844	-2.91456	0.499026	-5.84049	5.20E-09	9.85E-08	0	1.006035	0.653468	3.549829	4.483533	4.936752
AT2G15550	10.09143	-2.90584	0.513674	-5.65696	1.54E-08	2.74E-07	0	0	0	2.526213	5.262981	4.277199
AT4G05510	9.312048	-2.89923	0.502976	-5.76415	8.21E-09	1.51E-07	0	0	1.101576	3.689461	4.483533	4.488922
AT3G42723	10.65187	-2.86952	0.49135	-5.84007	5.22E-09	9.87E-08	0	0.588989	1.442988	4.700053	4.483533	4.028969
AT1G15380	63.09437	-2.8676	0.362669	-7.90693	2.64E-15	9.00E-14	4.258344	3.332773	2.750488	6.129868	7.072174	7.170215
AT2G05705	8.144736	-2.85959	0.502858	-5.68667	1.30E-08	2.32E-07	0.60238	0.588989	0	4.041933	4.089834	4.116545
AT5G60100	73.01443	-2.85779	0.259826	-10.9988	3.87E-28	2.89E-26	4.092952	4.259341	3.703969	7.094621	7.072174	6.949491
AT2G12300	10.47106	-2.85751	0.514166	-5.55756	2.74E-08	4.73E-07	0	0	0	2.526213	5.450922	4.028969
AT1G36460	7.334086	-2.85442	0.513381	-5.56004	2.70E-08	4.67E-07	0	0	0	3.222033	4.483533	3.935733
AT5G28870	7.255927	-2.85417	0.513945	-5.55347	2.80E-08	4.83E-07	0	0	0	3.222033	3.546624	4.673514
AT2G10955	8.024408	-2.84975	0.513568	-5.54893	2.87E-08	4.95E-07	0	0	0	3.933736	4.792505	3.027056
AT3G44215	8.086692	-2.84477	0.504652	-5.63709	1.73E-08	3.05E-07	0	0.588989	0.653468	3.933736	3.546624	4.553113
AT5G32510	7.191899	-2.84412	0.513385	-5.53992	3.03E-08	5.18E-07	0	0	0	3.395221	4.483533	3.728968
AT4G28960	7.008868	-2.83148	0.51376	-5.51128	3.56E-08	6.04E-07	0	0	0	4.236658	4.089834	3.197407
AT2G15160	7.151297	-2.8257	0.509741	-5.5434	2.97E-08	5.09E-07	0	0	0.653468	3.933736	3.546624	4.199107
AT5G28165	9.358874	-2.82287	0.49842	-5.66364	1.48E-08	2.64E-07	0.60238	0.588989	0.653468	3.549829	4.483533	4.553113
AT3G59930	55.8357	-2.8179	0.305346	-9.22856	2.74E-20	1.31E-18	4.002555	3.534563	3.173324	6.787815	6.822676	6.334274
AT3G19390	61.8966	-2.81379	0.339972	-8.27654	1.27E-16	4.83E-15	3.294214	3.818543	3.639105	6.457472	7.51294	6.138962
AT1G40135	8.243734	-2.79441	0.511063	-5.46783	4.56E-08	7.65E-07	0.60238	0	0	3.025182	4.483533	4.421742
AT3G06115	6.499814	-2.7936	0.513885	-5.43623	5.44E-08	9.05E-07	0	0	0	3.549829	4.089834	3.728968
AT5G32434	6.232529	-2.79047	0.514062	-5.42828	5.69E-08	9.45E-07	0	0	0	4.041933	3.546624	3.613295
AT5G44440	27.09903	-2.78998	0.435918	-6.40024	1.55E-10	3.44E-09	3.215913	1.329167	1.442988	5.974702	5.766238	5.199956
AT5G32591	7.571523	-2.78159	0.514067	-5.41095	6.27E-08	1.04E-06	0	0	0	3.395221	4.792505	3.34975
AT5G32436	7.599608	-2.77952	0.514104	-5.40653	6.43E-08	1.06E-06	0	0	0	3.549829	4.792505	3.197407
AT3G30360	7.930922	-2.77732	0.514558	-5.39749	6.76E-08	1.11E-06	0	0	0	2.526213	4.483533	4.488922
AT1G21400	3360.147	-2.77084	0.14543	-19.0528	6.23E-81	3.41E-78	9.793885	9.644116	9.638111	12.45963	12.77641	12.29737
AT3G20340	136.9047	-2.77029	0.250181	-11.0732	1.69E-28	1.30E-26	5.442744	4.437751	4.917441	7.842399	8.12318	7.805528

AT5G33150	6.120189	-2.76338	0.514294	-5.37315	7.74E-08	1.26E-06	0	0	0	3.549829	3.546624	4.028969
AT2G07510	6.051261	-2.75801	0.51451	-5.36046	8.30E-08	1.35E-06	0	0	0	4.142579	2.665087	3.935733
AT2G22710	22.22554	-2.75717	0.393351	-7.00944	2.39E-12	6.36E-11	2.501815	2.469294	1.718868	5.331804	5.450922	5.352044
AT4G06664	6.515141	-2.7458	0.514589	-5.33591	9.51E-08	1.53E-06	0	0	0	4.408199	3.546624	3.197407
AT3G43303	6.443644	-2.74461	0.514381	-5.33575	9.51E-08	1.53E-06	0	0	0	3.222033	4.089834	3.935733
AT2G10820	6.823269	-2.73598	0.51438	-5.31898	1.04E-07	1.67E-06	0	0	0	3.222033	4.483533	3.613295
AT3G33528	10.37324	-2.73586	0.485237	-5.6382	1.72E-08	3.03E-07	1.026049	0.588989	1.101576	4.041933	4.792505	4.199107
AT2G11100	5.949917	-2.72735	0.514529	-5.30067	1.15E-07	1.84E-06	0	0	0	3.549829	3.546624	3.935733
AT2G13320	7.401251	-2.72384	0.514524	-5.2939	1.20E-07	1.90E-06	0	0	0	3.395221	4.792505	3.197407
AT4G09430	11.92829	-2.72058	0.468818	-5.80306	6.51E-09	1.21E-07	1.026049	1.329167	1.442988	4.885743	4.483533	4.199107
AT3G43546	6.600936	-2.71961	0.511576	-5.31614	1.06E-07	1.69E-06	0	0.588989	0	3.816762	3.546624	4.028969
AT3G30775	4479.675	-2.71853	0.211104	-12.8777	6.01E-38	6.99E-36	10.34679	9.695025	10.17213	12.67053	13.35507	12.68864
AT4G09355	6.135516	-2.71849	0.5148	-5.28067	1.29E-07	2.04E-06	0	0	0	4.408199	2.665087	3.613295
AT5G24470	201.0517	-2.71738	0.227529	-11.943	7.06E-33	6.55E-31	5.004194	5.942585	5.792589	8.503046	8.42177	8.482004
AT3G33178	17.70378	-2.71346	0.511882	-5.30094	1.15E-07	1.84E-06	0.60238	0.588989	0.653468	4.486882	6.342338	2.023222
AT5G38005	33.73131	-2.71304	0.367301	-7.38641	1.51E-13	4.45E-12	3.045281	2.917335	3.078658	5.861135	5.046837	6.538339
AT2G07213	61.6042	-2.71259	0.333356	-8.13721	4.04E-16	1.47E-14	3.748168	3.403219	4.091061	5.974702	7.128345	7.041871
AT4G39070	92.2453	-2.70869	0.266655	-10.158	3.05E-24	1.84E-22	4.72207	3.965467	4.74322	7.63346	7.128345	7.249844
AT1G17277	8.065251	-2.70682	0.514748	-5.25853	1.45E-07	2.28E-06	0	0	0	3.025182	5.046837	3.34975
AT3G33136	14.01609	-2.7037	0.51276	-5.27284	1.34E-07	2.12E-06	0	1.006035	0	4.408199	5.90133	2.346685
AT1G25430	51.79715	-2.7014	0.343175	-7.87178	3.50E-15	1.18E-13	3.133118	4.055712	3.345839	7.003888	6.434336	6.052778
AT2G06914	6.441881	-2.69619	0.514739	-5.23798	1.62E-07	2.53E-06	0	0	0	4.041933	4.089834	3.027056
AT4G07580	6.06402	-2.69282	0.514821	-5.23059	1.69E-07	2.62E-06	0	0	0	3.222033	3.546624	4.199107
AT5G31804	5.779644	-2.68839	0.514745	-5.22275	1.76E-07	2.72E-06	0	0	0	3.549829	3.546624	3.836051
AT3G33575	12.48739	-2.68642	0.514184	-5.22463	1.75E-07	2.70E-06	0	0.588989	0	3.395221	5.90133	2.833864
AT2G15420	7.571765	-2.68112	0.512156	-5.23497	1.65E-07	2.57E-06	0	0	0.653468	3.025182	4.483533	4.116545
AT1G76410	133.9113	-2.68031	0.217421	-12.3278	6.42E-35	6.57E-33	5.240069	4.844514	5.097223	7.849861	8.12318	7.638054
AT4G13540	69.99772	-2.67426	0.340807	-7.84686	4.27E-15	1.43E-13	3.95514	4.403791	3.173324	6.907064	7.51294	6.278406
AT4G05638	6.103099	-2.66786	0.51481	-5.18222	2.19E-07	3.34E-06	0	0	0	3.222033	4.089834	3.728968
AT2G11983	5.626462	-2.66267	0.514975	-5.17049	2.33E-07	3.54E-06	0	0	0	3.816762	2.665087	4.028969
AT2G06885	12.17541	-2.65881	0.507833	-5.23559	1.64E-07	2.56E-06	0	0.588989	1.101576	2.797163	5.617177	4.199107
AT2G33830	8691.835	-2.65841	0.169851	-15.6514	3.25E-55	7.01E-53	11.34135	10.82702	11.23049	14.10813	13.87092	13.63276
AT3G32195	23.02141	-2.65004	0.43126	-6.14489	8.00E-10	1.67E-08	1.35313	2.710638	2.149802	4.561494	6.024848	5.387687

AT2G11773	7.174809	-2.6481	0.514941	-5.14253	2.71E-07	4.07E-06	0	0	0	3.025182	4.792505	3.34975
AT5G56870	3255.329	-2.64619	0.226508	-11.6826	1.57E-31	1.38E-29	10.17118	9.265873	9.452377	12.29308	12.80793	12.25246
AT2G12066	5.609372	-2.64617	0.514931	-5.13888	2.76E-07	4.15E-06	0	0	0	3.549829	3.546624	3.728968
AT3G24516	6.510809	-2.643	0.514907	-5.13297	2.85E-07	4.27E-06	0	0	0	3.395221	4.483533	3.197407
AT5G20260	22.74582	-2.63763	0.422779	-6.2388	4.41E-10	9.37E-09	3.045281	1.815969	1.442988	5.530617	5.617177	5.029925
AT1G62580	25.50751	-2.63652	0.401543	-6.56598	5.17E-11	1.22E-09	1.61959	2.817685	2.868369	5.101148	5.617177	5.863234
AT5G32520	6.848218	-2.63037	0.515141	-5.10611	3.29E-07	4.88E-06	0	0	0	3.025182	2.665087	4.887807
AT3G45300	2673.607	-2.61836	0.178555	-14.6641	1.09E-48	1.95E-46	9.808133	9.279982	9.271607	12.21686	12.36327	11.92266
AT5G32405	6.558099	-2.61664	0.51271	-5.10355	3.33E-07	4.94E-06	0.60238	0	0	3.395221	4.089834	3.836051
AT1G36060	50.89952	-2.61465	0.310027	-8.43365	3.35E-17	1.32E-15	4.002555	3.010545	3.825555	6.335206	6.679627	6.489965
AT3G42712	8.291025	-2.61258	0.511429	-5.10839	3.25E-07	4.83E-06	1.026049	0	0	3.025182	4.089834	4.730144
AT5G57640	36.54749	-2.61245	0.345231	-7.56726	3.81E-14	1.18E-12	3.506448	2.817685	2.977342	6.105138	6.342338	5.615206
AT5G34780	129.7331	-2.60428	0.212135	-12.2765	1.21E-34	1.22E-32	5.072441	5.056075	5.144727	7.765576	8.095267	7.577611
AT4G06656	6.599173	-2.59836	0.513655	-5.05857	4.22E-07	6.16E-06	0	0.588989	0	4.408199	3.546624	3.197407
AT3G33377	13.0092	-2.58684	0.514965	-5.02334	5.08E-07	7.32E-06	0	0.588989	0	3.222033	6.024848	2.610742
AT4G06541	8.558978	-2.58285	0.515139	-5.01389	5.33E-07	7.68E-06	0	0	0	2.526213	5.262981	3.34975
AT5G39520	97.55839	-2.57855	0.233477	-11.0441	2.34E-28	1.77E-26	4.932558	4.403791	4.832959	7.336904	7.380437	7.514525
AT3G42090	6.481202	-2.57817	0.513046	-5.02521	5.03E-07	7.25E-06	0	0	0.653468	3.816762	4.089834	3.34975
AT5G32312	5.257832	-2.57181	0.51514	-4.99244	5.96E-07	8.52E-06	0	0	0	3.689461	2.665087	3.935733
AT2G10540	8.136302	-2.56709	0.51017	-5.03184	4.86E-07	7.02E-06	0	1.006035	0	3.689461	4.792505	3.34975
AT4G15530	2493.316	-2.56278	0.198385	-12.9182	3.55E-38	4.17E-36	9.489314	9.444569	9.26893	11.71509	12.52894	11.83823
AT5G36655	8.651573	-2.55883	0.505798	-5.05899	4.21E-07	6.15E-06	1.026049	0.588989	0	3.222033	4.792505	4.028969
AT3G33082	5.525117	-2.55325	0.515141	-4.95641	7.18E-07	1.01E-05	0	0	0	3.025182	3.546624	4.028969
AT5G23240	103.4181	-2.54394	0.273072	-9.316	1.21E-20	5.96E-19	4.77764	4.437751	5.097223	7.86467	7.47011	7.053013
AT4G06672	5.240742	-2.54027	0.515141	-4.93122	8.17E-07	1.14E-05	0	0	0	3.395221	3.546624	3.613295
AT3G29730	7.940524	-2.53999	0.505151	-5.02817	4.95E-07	7.15E-06	0.60238	0.588989	0.653468	3.222033	4.483533	4.116545
AT1G15040	137.1009	-2.53789	0.285301	-8.89549	5.82E-19	2.55E-17	5.354131	5.297139	4.580013	7.667641	8.466036	7.388246
AT5G29568	5.592281	-2.52567	0.515141	-4.90288	9.44E-07	1.31E-05	0	0	0	3.222033	4.089834	3.34975
AT3G33166	69.00813	-2.52531	0.512248	-4.92986	8.23E-07	1.15E-05	2.210244	2.817685	1.442988	6.15418	8.177436	5.489617
AT3G30585	9.012346	-2.52306	0.512672	-4.92139	8.59E-07	1.20E-05	0	0	1.101576	2.526213	4.792505	4.488922
AT4G04223	157.5628	-2.52073	0.206976	-12.1788	4.03E-34	3.88E-32	5.751099	5.161454	5.439554	8.083015	8.203818	7.979124
AT1G42070	6.018845	-2.51869	0.515099	-4.88972	1.01E-06	1.39E-05	0	0	0	2.526213	4.089834	4.028969
AT3G42252	6.114094	-2.51154	0.51513	-4.87555	1.09E-06	1.49E-05	0	0	0	3.025182	4.483533	3.197407

AT5G32702	6.708943	-2.50963	0.513964	-4.88289	1.05E-06	1.44E-05	0	0.588989	0	3.025182	4.483533	3.613295
AT5G14920	2058.137	-2.50623	0.225491	-11.1146	1.07E-28	8.29E-27	9.068346	8.775229	9.513593	11.64766	12.22038	11.38181
AT3G30396	5.564197	-2.50508	0.515124	-4.86307	1.16E-06	1.58E-05	0	0	0	3.025182	4.089834	3.487532
AT4G06621	5.564197	-2.50508	0.515124	-4.86307	1.16E-06	1.58E-05	0	0	0	3.025182	4.089834	3.487532
AT4G22450	6.327778	-2.50442	0.515029	-4.86268	1.16E-06	1.58E-05	0	0	0	4.236658	4.089834	2.346685
AT3G31410	5.824597	-2.50198	0.514556	-4.86239	1.16E-06	1.58E-05	0	0.588989	0	3.549829	2.665087	4.277199
AT5G03090	5.676536	-2.49923	0.515106	-4.85188	1.22E-06	1.66E-05	0	0	0	3.689461	4.089834	2.833864
AT4G15590	6.232881	-2.49819	0.514481	-4.85575	1.20E-06	1.63E-05	0.60238	0	0	4.324977	3.546624	3.027056
AT4G07250	4.945372	-2.49055	0.515041	-4.83563	1.33E-06	1.79E-05	0	0	0	3.816762	2.665087	3.613295
AT5G61380	444.9392	-2.4864	0.148605	-16.7316	7.71E-63	2.15E-60	6.933848	7.083418	7.052865	9.58617	9.723045	9.390583
AT3G34299	11.94849	-2.48471	0.515138	-4.82339	1.41E-06	1.90E-05	0	0.588989	0	3.222033	5.90133	2.346685
AT2G21660	1652.777	-2.48385	0.233123	-10.6547	1.66E-26	1.15E-24	8.221384	9.356322	8.756314	11.69659	11.34589	11.35223
AT5G32053	432.8308	-2.48128	0.514129	-4.82617	1.39E-06	1.87E-05	2.628119	0	0.653468	9.549979	9.988833	9.694401
AT3G30744	8.682554	-2.47628	0.514697	-4.81115	1.50E-06	2.00E-05	0	0	0.653468	2.797163	5.262981	3.197407
AT3G33193	25.34766	-2.47377	0.491265	-5.03551	4.77E-07	6.90E-06	1.026049	2.009043	2.324987	4.041933	6.822676	4.116545
AT2G11623	5.042384	-2.47317	0.515049	-4.80181	1.57E-06	2.08E-05	0	0	0	3.222033	3.546624	3.613295
AT1G52850	7.233094	-2.47224	0.514416	-4.80592	1.54E-06	2.05E-05	0.60238	0	0	2.797163	4.792505	3.487532
AT4G04530	6.019196	-2.46615	0.514249	-4.79564	1.62E-06	2.14E-05	0.60238	0	0	3.222033	4.089834	3.613295
AT1G41910	5.478179	-2.45024	0.514986	-4.75787	1.96E-06	2.55E-05	0	0	0	3.549829	4.089834	2.833864
AT4G06518	5.971906	-2.44813	0.514979	-4.75384	2.00E-06	2.60E-05	0	0	0	3.222033	4.483533	2.833864
AT2G43795	7.742175	-2.44705	0.50548	-4.84104	1.29E-06	1.75E-05	0	1.329167	0.653468	4.486882	3.546624	3.728968
AT5G23235	90.4939	-2.4411	0.282747	-8.6335	5.95E-18	2.47E-16	4.634483	4.296832	5.048102	7.667641	7.234523	6.876105
AT2G10620	6.834505	-2.44007	0.514707	-4.74069	2.13E-06	2.76E-05	0	0	0.653468	2.526213	4.483533	3.935733
AT3G29610	4.803184	-2.42697	0.514764	-4.71471	2.42E-06	3.11E-05	0	0	0	3.933736	2.665087	3.34975
AT2G11060	4.872112	-2.42476	0.514876	-4.70941	2.48E-06	3.18E-05	0	0	0	3.222033	3.546624	3.487532
AT4G26290	106.5725	-2.4237	0.277487	-8.73447	2.45E-18	1.03E-16	5.317104	4.655421	4.580013	7.525773	7.915398	7.030642
AT3G23725	14.48416	-2.41868	0.470236	-5.14356	2.70E-07	4.06E-06	1.026049	2.331572	1.101576	4.041933	5.450922	4.553113
AT3G42179	9.07331	-2.40624	0.512513	-4.69499	2.67E-06	3.39E-05	1.026049	0	0.653468	4.826463	4.483533	2.346685
AT4G05589	5.223651	-2.39863	0.514778	-4.65954	3.17E-06	4.00E-05	0	0	0	3.025182	4.089834	3.197407
AT5G35375	6.586777	-2.39768	0.508987	-4.71069	2.47E-06	3.16E-05	1.026049	0	0.653468	3.689461	3.546624	4.028969
AT5G00480	5.279821	-2.39597	0.514743	-4.65469	3.24E-06	4.08E-05	0	0	0	3.395221	4.089834	2.833864
AT3G33154	10.45233	-2.39585	0.514539	-4.65631	3.22E-06	4.05E-05	0.60238	0.588989	0	3.395221	5.617177	2.346685
AT5G28845	7.427369	-2.39475	0.508692	-4.70766	2.51E-06	3.20E-05	0	1.006035	0.653468	3.222033	4.483533	3.836051

AT3G42820	6.308256	-2.38805	0.513912	-4.64681	3.37E-06	4.23E-05	1.026049	0	0	4.408199	2.665087	3.613295
AT5G34623	5.171591	-2.38535	0.515006	-4.63169	3.63E-06	4.53E-05	0	0.588989	0	3.689461	2.665087	3.836051
AT2G07215	39.94901	-2.37814	0.363138	-6.54885	5.80E-11	1.35E-09	3.691428	3.098096	3.424931	5.243916	6.520817	6.38806
AT2G09900	4.729924	-2.37791	0.514615	-4.62075	3.82E-06	4.77E-05	0	0	0	3.395221	3.546624	3.197407
AT1G50850	5.337754	-2.37448	0.514523	-4.61492	3.93E-06	4.88E-05	0	0	0	2.526213	4.089834	3.613295
AT2G07100	6.794961	-2.37293	0.515106	-4.60669	4.09E-06	5.06E-05	0	0.588989	0	2.192331	4.483533	4.028969
AT5G33434	11.97407	-2.36855	0.479752	-4.93702	7.93E-07	1.11E-05	1.61959	1.006035	1.950366	3.816762	4.089834	5.239508
AT1G07040	943.0605	-2.3557	0.164178	-14.3484	1.09E-46	1.81E-44	8.106727	8.141285	8.368259	10.62926	10.88999	10.33806
AT3G06505	7.934678	-2.35241	0.500961	-4.69579	2.66E-06	3.38E-05	0	1.329167	1.101576	4.142579	4.089834	3.728968
AT2G10250	13.64743	-2.34603	0.444331	-5.27992	1.29E-07	2.04E-06	1.35313	2.331572	1.950366	4.885743	4.483533	4.614569
AT2G10640	6.776349	-2.34471	0.513119	-4.56953	4.89E-06	5.97E-05	0	0.588989	0.653468	2.797163	4.483533	3.728968
AT4G35770	22354.78	-2.34266	0.170189	-13.7651	4.13E-43	6.03E-41	12.92151	12.54793	12.87041	15.30887	15.3988	14.8291
AT3G30418	5.259947	-2.33387	0.51514	-4.53056	5.88E-06	7.10E-05	0.60238	0	0	3.222033	2.665087	4.199107
AT3G10985	1559.875	-2.33362	0.223747	-10.4297	1.81E-25	1.17E-23	9.314822	8.489769	8.807023	11.59312	11.51081	10.902
AT3G62950	400.6163	-2.33186	0.23276	-10.0183	1.27E-23	7.39E-22	7.460728	6.543296	6.653811	9.508148	9.528036	9.162683
AT1G38390	5.167482	-2.3301	0.514184	-4.53164	5.85E-06	7.07E-05	0	0	0	2.526213	4.089834	3.487532
AT3G05955	11.12044	-2.32213	0.495863	-4.68299	2.83E-06	3.59E-05	0	1.329167	1.718868	3.933736	5.262981	3.613295
AT5G35794	4.645669	-2.31459	0.514014	-4.50297	6.70E-06	8.00E-05	0	0	0	2.797163	3.546624	3.613295
AT5G31572	5.771117	-2.31059	0.514378	-4.492	7.06E-06	8.40E-05	1.026049	0	0	3.689461	2.665087	4.116545
AT4G09370	4.814179	-2.31011	0.513891	-4.49533	6.95E-06	8.28E-05	0	0	0	3.816762	3.546624	2.610742
AT4G02314	5.508379	-2.30965	0.51506	-4.48424	7.32E-06	8.68E-05	0.60238	0	0	3.222033	4.089834	3.197407
AT5G35050	5.536464	-2.30869	0.51507	-4.48229	7.38E-06	8.74E-05	0.60238	0	0	3.395221	4.089834	3.027056
AT4G30270	3873.71	-2.30724	0.238852	-9.65967	4.47E-22	2.39E-20	10.71472	9.807259	10.00039	12.25284	13.06158	12.60215
AT3G30465	4.945149	-2.30544	0.515141	-4.47537	7.63E-06	9.00E-05	0	0.588989	0	3.395221	2.665087	3.935733
AT3G15460	859.4645	-2.30515	0.188492	-12.2294	2.16E-34	2.13E-32	8.386869	8.003519	7.926923	10.80557	10.17369	10.43387
AT5G29032	5.575191	-2.30379	0.513971	-4.48233	7.38E-06	8.74E-05	0	0	0	2.797163	4.483533	2.833864
AT4G07939	5.477956	-2.29838	0.515098	-4.46201	8.12E-06	9.53E-05	0	0.588989	0	3.025182	4.089834	3.34975
AT5G31787	4.587736	-2.29673	0.513863	-4.46953	7.84E-06	9.22E-05	0	0	0	3.549829	3.546624	2.833864
AT5G52910	243.7572	-2.29647	0.183401	-12.5216	5.69E-36	6.11E-34	6.039521	6.446534	6.451453	8.839252	8.611075	8.566073
AT2G15890	4038.251	-2.29207	0.14231	-16.1061	2.31E-58	5.55E-56	10.35652	10.24745	10.52948	12.9035	12.72952	12.49619
AT1G37405	5.439228	-2.27952	0.513717	-4.4373	9.11E-06	0.000106	0.60238	0.588989	0	3.549829	3.546624	3.487532
AT3G31909	4.208112	-2.27616	0.513492	-4.4327	9.31E-06	0.000108	0	0	0	3.549829	2.665087	3.34975
AT5G09440	584.0816	-2.27565	0.234472	-9.70544	2.86E-22	1.55E-20	8.079347	7.045376	7.266564	9.858171	10.12621	9.826886

AT2G07660	7.143235	-2.27133	0.507831	-4.47261	7.73E-06	9.11E-05	0	1.006035	1.101576	3.222033	4.089834	4.116545
AT4G04560	5.721266	-2.26761	0.514391	-4.40835	1.04E-05	0.00012	0	1.006035	0	3.222033	3.546624	3.935733
AT2G08685	21.09227	-2.26394	0.382416	-5.92009	3.22E-09	6.25E-08	2.501815	2.710638	2.977342	5.414645	5.046837	5.199956
AT3G33163	4.777214	-2.263	0.515113	-4.39322	1.12E-05	0.000128	0.60238	0	0	3.395221	2.665087	3.836051
AT5G47240	65.64214	-2.25975	0.296575	-7.61948	2.55E-14	8.06E-13	4.21874	4.22085	4.680135	7.203473	6.752923	6.352426
AT4G06678	4.842263	-2.25623	0.51313	-4.397	1.10E-05	0.000126	0	0	0	3.933736	3.546624	2.346685
AT5G07440	4157.676	-2.25604	0.157603	-14.3147	1.77E-46	2.84E-44	10.60427	10.31067	10.39966	12.53331	13.04161	12.64719
AT3G42057	16.9285	-2.25568	0.512229	-4.40365	1.06E-05	0.000122	1.844425	0	1.101576	3.395221	6.342338	3.197407
AT1G36400	5.335768	-2.24943	0.515141	-4.36664	1.26E-05	0.000143	0	0.588989	0	3.222033	4.089834	3.027056
AT3G00600	5.267063	-2.2466	0.512727	-4.38167	1.18E-05	0.000134	0	0	0	4.236658	3.546624	2.023222
AT1G11810	5.303359	-2.24264	0.514751	-4.35674	1.32E-05	0.000149	0.60238	0	0	4.324977	0	3.728968
AT2G06400	4.333209	-2.24169	0.513264	-4.36753	1.26E-05	0.000143	0	0	0	3.025182	3.546624	3.197407
AT3G42110	5.319142	-2.23151	0.51514	-4.33184	1.48E-05	0.000165	0	0	0.653468	3.025182	4.089834	3.197407
AT4G06560	6.293617	-2.23007	0.514022	-4.33848	1.43E-05	0.000161	0	0.588989	0.653468	3.025182	4.483533	3.197407
AT1G41700	6.170634	-2.21992	0.511219	-4.3424	1.41E-05	0.000158	0.60238	0.588989	0.653468	3.025182	4.089834	3.728968
AT3G32210	4.292366	-2.21869	0.51255	-4.32872	1.50E-05	0.000167	0	0	0	3.933736	2.665087	2.833864
AT1G12720	5.376834	-2.21567	0.512826	-4.32051	1.56E-05	0.000173	0	0	0	2.526213	4.483533	2.833864
AT2G15815	5.291057	-2.20809	0.515113	-4.28661	1.81E-05	0.000199	0	0	0.653468	2.797163	4.089834	3.34975
AT4G07760	4.065924	-2.20128	0.512393	-4.29607	1.74E-05	0.000192	0	0	0	3.689461	2.665087	3.027056
AT1G03090	4836.612	-2.18234	0.142956	-15.2657	1.29E-52	2.60E-50	10.82845	10.68225	10.68267	12.96706	13.17926	12.69643
AT3G30600	5.137411	-2.18075	0.515039	-4.23414	2.29E-05	0.000247	0	0.588989	0	3.025182	4.089834	3.027056
AT5G34990	4.162937	-2.17845	0.512328	-4.25206	2.12E-05	0.000229	0	0	0	3.025182	3.546624	3.027056
AT4G06641	4.095772	-2.17528	0.511838	-4.24994	2.14E-05	0.000231	0	0	0	2.797163	2.665087	3.836051
AT1G40630	3.953585	-2.1751	0.511956	-4.24861	2.15E-05	0.000232	0	0	0	3.025182	2.665087	3.613295
AT5G32410	4.27704	-2.17302	0.512075	-4.24356	2.20E-05	0.000237	0	0	0	2.526213	3.546624	3.487532
AT5G14470	63.46404	-2.1674	0.285019	-7.60441	2.86E-14	9.01E-13	4.72207	4.181303	4.138765	6.657813	7.128345	6.38806
AT5G19230	76.24086	-2.16274	0.263284	-8.21448	2.13E-16	7.92E-15	5.072441	4.739512	4.229687	7.107129	6.952811	6.937516
AT3G30830	4.219106	-2.15984	0.511915	-4.21914	2.45E-05	0.000262	0	0	0	3.395221	3.546624	2.610742
AT2G15880	312.4446	-2.159	0.267432	-8.07309	6.85E-16	2.45E-14	6.36388	6.900687	6.725863	8.689264	9.657748	8.461244
AT1G05313	3.839482	-2.15488	0.511664	-4.21151	2.54E-05	0.000271	0	0	0	3.395221	2.665087	3.197407
AT1G40129	3.839482	-2.15488	0.511664	-4.21151	2.54E-05	0.000271	0	0	0	3.395221	2.665087	3.197407
AT1G40118	4.419227	-2.14803	0.51141	-4.20021	2.67E-05	0.000283	0	0	0	2.192331	3.546624	3.728968
AT3G24514	3.867567	-2.14747	0.511491	-4.19844	2.69E-05	0.000285	0	0	0	3.549829	2.665087	3.027056

AT5G60610	16.09277	-2.14696	0.42873	-5.00771	5.51E-07	7.92E-06	2.038899	1.815969	2.977342	4.942684	4.792505	4.837144
AT3G28915	8.853194	-2.14426	0.515022	-4.16344	3.13E-05	0.000327	0.60238	0.588989	0.653468	5.567291	0	2.610742
AT4G34030	2356.642	-2.13487	0.14865	-14.3617	9.00E-47	1.50E-44	9.771819	9.701155	9.716837	11.87996	12.17776	11.6419
AT4G06603	23.29911	-2.13378	0.392485	-5.43659	5.43E-08	9.04E-07	2.74425	3.403219	2.622111	5.050236	5.046837	5.811684
AT2G07750	24.06536	-2.12739	0.360618	-5.89929	3.65E-09	7.07E-08	2.951747	3.180636	2.977342	5.288529	5.450922	5.42247
AT1G67265	348.1845	-2.12568	0.194277	-10.9415	7.30E-28	5.33E-26	7.236162	6.838488	6.710159	9.14547	9.419665	8.888795
AT1G22990	29.13824	-2.1251	0.355255	-5.98191	2.21E-09	4.40E-08	3.632365	3.403219	2.750488	5.861135	5.617177	5.456434
AT3G33065	5.262731	-2.11223	0.510831	-4.13488	3.55E-05	0.000367	0	0	0	3.025182	4.483533	2.023222
AT4G15890	38.51124	-2.11022	0.321987	-6.55376	5.61E-11	1.31E-09	3.439114	4.181303	3.571186	5.974702	5.90133	6.200381
AT3G39230	8.862429	-2.10921	0.512545	-4.11517	3.87E-05	0.000397	0.60238	0.588989	1.101576	3.395221	5.262981	2.346685
AT4G06680	3.992664	-2.10812	0.511018	-4.12534	3.70E-05	0.000381	0	0	0	3.025182	3.546624	2.833864
AT5G48250	117.3038	-2.10281	0.216833	-9.69783	3.08E-22	1.66E-20	5.137605	5.655661	5.400949	7.676061	7.63429	7.481917
AT3G30665	3.964579	-2.09825	0.510803	-4.10774	4.00E-05	0.000409	0	0	0	2.797163	3.546624	3.027056
AT5G27927	4.083014	-2.096	0.509827	-4.1112	3.94E-05	0.000404	0	0	0	3.933736	0	3.487532
AT2G01029	25.66012	-2.09394	0.401693	-5.21279	1.86E-07	2.85E-06	2.74425	2.817685	3.173324	4.561494	6.244072	5.239508
AT3G13450	1519.294	-2.09227	0.140075	-14.9367	1.90E-50	3.56E-48	9.236423	9.01343	9.150294	11.25314	11.47335	11.06978
AT1G28330	5110.903	-2.08993	0.164972	-12.6684	8.85E-37	9.80E-35	11.06668	10.62629	10.91404	13.2866	12.94619	12.80009
AT5G33355	7.07889	-2.08637	0.498437	-4.18582	2.84E-05	0.000301	1.35313	1.006035	1.101576	3.549829	3.546624	4.199107
AT1G06570	3321.29	-2.08477	0.170505	-12.227	2.23E-34	2.19E-32	10.33179	10.12708	10.30156	12.41694	12.69854	12.01849
AT3G61060	513.357	-2.08127	0.250126	-8.32089	8.73E-17	3.36E-15	7.940164	7.099419	7.308884	9.292358	10.19358	9.544566
AT5G29560	5.491289	-2.08052	0.514425	-4.04436	5.25E-05	0.000526	0.60238	0	0	2.797163	4.483533	2.610742
AT2G39855	11.48647	-2.07706	0.45854	-4.52972	5.91E-06	7.12E-05	2.363383	2.009043	1.442988	4.632436	3.546624	4.730144
AT2G39350	130.2477	-2.07669	0.201477	-10.3073	6.53E-25	4.10E-23	5.442744	5.487509	5.807425	7.70103	7.850087	7.674584
AT2G02100	1098.703	-2.06858	0.186529	-11.0898	1.41E-28	1.09E-26	8.940271	8.315788	8.697799	11.07119	10.59342	10.71122
AT1G08533	4.830824	-2.05343	0.515057	-3.98681	6.70E-05	0.000656	0	1.006035	0	3.222033	2.665087	3.935733
AT3G23060	4.862881	-2.05324	0.508547	-4.03747	5.40E-05	0.000541	0	0	0	1.757146	2.665087	4.488922
AT1G09420	198.9863	-2.03597	0.191355	-10.6397	1.95E-26	1.34E-24	6.159424	6.231052	6.227084	8.300115	8.649971	8.030761
AT5G50450	183.4553	-2.03414	0.201099	-10.1151	4.73E-24	2.82E-22	6.240765	5.93071	6.06369	8.144986	8.550692	7.937644
AT1G62510	814.7071	-2.0322	0.210986	-9.63191	5.86E-22	3.11E-20	8.213849	8.050918	8.427078	10.2339	10.82349	9.92415
AT5G22500	3068.936	-2.03218	0.192782	-10.5413	5.57E-26	3.74E-24	9.815619	10.10809	10.51543	12.5167	12.29283	11.99196
AT2G11620	14.25307	-2.03205	0.429169	-4.73484	2.19E-06	2.84E-05	2.210244	2.331572	2.481185	4.408199	4.483533	5.029925
AT5G31980	4.692608	-2.03095	0.508028	-3.99772	6.40E-05	0.00063	0	0	0	1.757146	2.665087	4.421742
AT5G27345	7.315175	-2.02977	0.504955	-4.01971	5.83E-05	0.00058	1.844425	1.006035	0	3.933736	3.546624	4.028969

AT2G11405	3.794307	-2.02908	0.509208	-3.98478	6.75E-05	0.000661	0	0	0	2.797163	3.546624	2.833864
AT5G29574	3.794307	-2.02908	0.509208	-3.98478	6.75E-05	0.000661	0	0	0	2.797163	3.546624	2.833864
AT1G79360	175.3725	-2.02452	0.199296	-10.1583	3.04E-24	1.84E-22	6.220856	5.793186	6.06369	8.083015	8.42177	7.949617
AT1G17665	33.14177	-2.02344	0.346191	-5.84487	5.07E-09	9.62E-08	3.691428	3.332773	3.424931	5.243916	6.342338	5.702706
AT4G06564	3.470852	-2.02176	0.508732	-3.9741	7.06E-05	0.000689	0	0	0	3.222033	2.665087	3.027056
AT1G04157	138.9179	-2.01425	0.200167	-10.0629	8.06E-24	4.76E-22	5.764914	5.76671	5.601531	7.834898	8.037767	7.577611
AT3G47330	3.498937	-2.01418	0.508489	-3.96111	7.46E-05	0.000725	0	0	0	3.395221	2.665087	2.833864
AT1G62515	539.8793	-2.01168	0.233741	-8.60645	7.54E-18	3.11E-16	7.602111	7.408578	7.871527	9.515204	10.31351	9.327748
AT1G19530	63.18621	-2.01066	0.353204	-5.69265	1.25E-08	2.25E-07	4.371025	4.403791	4.315217	5.637956	7.51294	6.50627
AT1G03100	291.9116	-2.00868	0.186523	-10.7691	4.82E-27	3.42E-25	7.090168	6.670137	6.637299	9.05152	8.896321	8.689016
AT3G43573	4.303138	-2.00866	0.513633	-3.9107	9.20E-05	0.000879	0	0.588989	0	3.395221	3.546624	2.610742
AT3G42727	4.787987	-2.00715	0.515098	-3.89663	9.75E-05	0.000926	0.60238	0.588989	0	2.526213	3.546624	3.728968
AT5G34837	3.766222	-2.00673	0.50857	-3.94583	7.95E-05	0.000769	0	0	0	2.526213	3.546624	3.027056
AT4G03910	5.700289	-2.00425	0.507909	-3.94608	7.94E-05	0.000769	0	0	0	1.757146	4.792505	2.610742
AT2G10900	3.753464	-1.99609	0.507705	-3.93159	8.44E-05	0.000812	0	0	0	3.816762	2.665087	2.346685
AT3G33537	9.910868	-1.99026	0.514602	-3.86757	0.00011	0.001027	0	1.329167	0	2.526213	5.617177	2.610742
AT1G40095	3.414682	-1.98731	0.507736	-3.91406	9.08E-05	0.000868	0	0	0	2.797163	2.665087	3.34975
AT4G36670	726.4095	-1.98633	0.159989	-12.4154	2.16E-35	2.25E-33	8.304134	7.936808	8.173019	10.09009	10.43541	10.02237
AT2G43865	3.658215	-1.98594	0.507077	-3.91644	8.99E-05	0.00086	0	0	0	3.549829	0	3.613295
AT5G22505	396.9937	-1.9857	0.200468	-9.90535	3.95E-23	2.26E-21	7.017014	7.211635	7.521753	9.590631	9.350505	8.973333
AT2G06822	5.77134	-1.98396	0.515025	-3.85217	0.000117	0.001085	0	1.006035	0	3.025182	4.483533	2.610742
AT3G15635	242.0679	-1.98152	0.183069	-10.8239	2.65E-27	1.91E-25	6.806053	6.351747	6.488747	8.664088	8.724751	8.435932
AT2G00440	11.44058	-1.96961	0.468998	-4.19961	2.67E-05	0.000284	1.61959	2.331572	2.149802	5.101148	2.665087	4.421742
AT4G37390	156.9442	-1.96762	0.211367	-9.30901	1.29E-20	6.35E-19	6.298895	5.832011	5.668068	7.984764	7.883112	8.086048
AT5G35935	63.48014	-1.96707	0.278408	-7.06541	1.60E-12	4.33E-11	4.048462	4.844514	4.832959	6.787815	6.679627	6.645278
AT3G33066	3.738137	-1.95985	0.507097	-3.86484	0.000111	0.001037	0	0	0	2.192331	3.546624	3.197407
AT5G35061	4.866016	-1.95742	0.506924	-3.86138	0.000113	0.00105	0	0	0	2.526213	4.483533	2.023222
AT3G43563	4.2197	-1.95608	0.514993	-3.79827	0.000146	0.001316	0.60238	0	0.653468	3.549829	2.665087	3.197407
AT4G39675	24.1446	-1.95214	0.381096	-5.12244	3.02E-07	4.50E-06	2.951747	3.332773	2.868369	4.764642	5.90133	5.278004
AT3G42624	3.624034	-1.95158	0.507044	-3.84893	0.000119	0.001097	0	0	0	2.797163	3.546624	2.610742
AT5G07010	1128.975	-1.95096	0.190393	-10.247	1.22E-24	7.57E-23	8.777734	8.924909	8.683797	10.31836	11.04395	11.00528
AT3G21570	3.300579	-1.94879	0.506653	-3.84639	0.00012	0.001107	0	0	0	3.222033	2.665087	2.833864
AT1G33130	4.048611	-1.9453	0.512919	-3.79261	0.000149	0.001343	0	0.588989	0	2.797163	3.546624	3.027056

AT5G35065	4.145847	-1.94071	0.506693	-3.83016	0.000128	0.001176	0	0	0	2.526213	4.089834	2.346685
AT1G07887	124.7239	-1.93973	0.255343	-7.59655	3.04E-14	9.56E-13	5.896243	4.918535	5.807425	7.692755	7.74622	7.546413
AT4G28040	564.4971	-1.93951	0.146927	-13.2006	8.71E-40	1.09E-37	7.799592	7.687943	8.006222	9.901902	9.87031	9.669278
AT2G04036	3.595949	-1.93881	0.50663	-3.82688	0.00013	0.001189	0	0	0	2.526213	3.546624	2.833864
AT4G06732	3.595949	-1.93881	0.50663	-3.82688	0.00013	0.001189	0	0	0	2.526213	3.546624	2.833864
AT1G53490	125.1826	-1.93822	0.258223	-7.50598	6.10E-14	1.85E-12	5.870916	4.988944	5.731672	7.544288	8.008134	7.405736
AT4G19160	3222.357	-1.93745	0.160064	-12.1042	1.00E-33	9.51E-32	10.55989	10.22453	10.21519	12.4766	12.44018	12.03228
AT5G32678	4.259949	-1.93555	0.50626	-3.82324	0.000132	0.001205	0	0	0	1.757146	4.089834	3.027056
AT5G28430	23.19458	-1.93133	0.421259	-4.58465	4.55E-06	5.58E-05	3.045281	3.098096	2.622111	4.041933	5.90133	5.522054
AT4G16563	81.9437	-1.93132	0.253216	-7.62716	2.40E-14	7.60E-13	5.072441	4.844514	4.944537	6.691428	7.51294	6.888598
AT1G53480	125.5402	-1.93073	0.256799	-7.51843	5.54E-14	1.69E-12	5.908741	4.918535	5.836646	7.70103	7.74622	7.562097
AT5G37875	4.53333	-1.93024	0.505282	-3.82011	0.000133	0.001217	0	0	0	1.131026	3.546624	4.028969
AT5G54080	2232.661	-1.92835	0.188709	-10.2186	1.64E-24	1.01E-22	10.03315	9.579034	9.756558	11.79361	12.12178	11.40808
AT2G10960	3.697072	-1.92834	0.51219	-3.7649	0.000167	0.001486	0	0.588989	0	3.025182	2.665087	3.34975
AT3G33124	5.530016	-1.92651	0.505565	-3.8106	0.000139	0.00126	0	0	0	1.757146	4.792505	2.346685
AT4G06730	4.132866	-1.91935	0.512228	-3.74707	0.000179	0.001583	0	0.588989	0	3.395221	3.546624	2.346685
AT2G38990	3.459857	-1.91926	0.505039	-3.80022	0.000145	0.001307	0	0	0	3.395221	0	3.613295
AT1G77380	35.3904	-1.91896	0.328649	-5.83894	5.25E-09	9.93E-08	4.092952	3.654941	3.703969	5.672031	5.617177	6.239921
AT3G15630	948.821	-1.91732	0.175121	-10.9485	6.75E-28	4.96E-26	8.876482	8.333935	8.499668	10.70835	10.63763	10.31972
AT1G27670	32.6992	-1.91559	0.361547	-5.29832	1.17E-07	1.86E-06	4.178017	2.917335	3.424931	5.603056	6.024848	5.702706
AT3G06435	87.60764	-1.91541	0.253436	-7.55776	4.10E-14	1.27E-12	5.354131	4.655421	5.257064	6.9213	7.284819	7.230345
AT5G05130	99.36307	-1.91171	0.211562	-9.03617	1.62E-19	7.36E-18	5.493412	5.27852	5.320494	7.389052	7.182411	7.370542
AT1G54100	3781.879	-1.90485	0.125139	-15.2219	2.53E-52	5.04E-50	10.61861	10.62029	10.60147	12.54571	12.72952	12.35167
AT5G32630	4.757916	-1.89559	0.515002	-3.68074	0.000233	0.001993	0.60238	1.006035	0	3.025182	3.546624	3.34975
AT3G43850	151.979	-1.89066	0.257651	-7.33807	2.17E-13	6.28E-12	6.451914	5.386795	5.731672	8.037996	7.946977	7.818645
AT4G22810	4.316713	-1.88371	0.514851	-3.65874	0.000253	0.002151	0.60238	0	0.653468	2.797163	3.546624	3.197407
AT5G57650	8.520861	-1.88148	0.512871	-3.66853	0.000244	0.00208	1.61959	1.006035	0	3.689461	5.046837	2.346685
AT4G04265	4.202016	-1.87576	0.504193	-3.72033	0.000199	0.001739	0	0	0	3.025182	4.089834	1.605623
AT4G06631	3.102222	-1.87562	0.504246	-3.71964	0.0002	0.001742	0	0	0	3.025182	2.665087	2.833864
AT5G33270	3.102222	-1.87562	0.504246	-3.71964	0.0002	0.001742	0	0	0	3.025182	2.665087	2.833864
AT5G23050	639.2018	-1.8748	0.151472	-12.3772	3.47E-35	3.60E-33	8.057062	8.067282	8.028552	9.85632	10.26422	9.795717
AT5G03615	306.2076	-1.87171	0.240509	-7.78228	7.12E-15	2.34E-13	7.447939	6.527613	6.702243	9.057977	9.092627	8.619516
AT4G06587	3.526799	-1.86911	0.51113	-3.65682	0.000255	0.002165	0	0.588989	0	3.025182	2.665087	3.197407

AT1G37050	3.425677	-1.8626	0.504058	-3.69521	0.00022	0.001898	0	0	0	2.526213	3.546624	2.610742
AT3G00220	62.95949	-1.85982	0.270987	-6.86316	6.74E-12	1.71E-10	4.907866	4.333373	4.647527	6.514927	7.013727	6.45679
AT1G76590	239.9118	-1.85738	0.171741	-10.815	2.92E-27	2.10E-25	6.534884	6.558811	6.838487	8.425264	8.742856	8.538591
AT2G34130	3.850254	-1.85505	0.511279	-3.62826	0.000285	0.00238	0	0.588989	0	2.526213	3.546624	3.027056
AT4G16215	3.852592	-1.85238	0.511287	-3.62296	0.000291	0.002424	0.60238	0	0	2.526213	3.546624	3.027056
AT2G38465	35.43441	-1.84706	0.31903	-5.78963	7.05E-09	1.31E-07	3.95514	4.011295	3.499911	5.919035	6.024848	5.644966
AT3G61890	479.9361	-1.84508	0.201326	-9.16464	4.97E-20	2.34E-18	7.845823	7.663037	7.380081	9.706135	9.802877	9.123581
AT4G24230	1745.897	-1.84498	0.148561	-12.419	2.06E-35	2.16E-33	9.706208	9.477594	9.409263	11.48646	11.58544	11.17425
AT3G29510	3.947489	-1.84036	0.503165	-3.65756	0.000255	0.00216	0	0	0	2.192331	4.089834	2.346685
AT2G11380	3.158392	-1.83962	0.502755	-3.65908	0.000253	0.002149	0	0	0	3.395221	2.665087	2.346685
AT3G22121	752.6315	-1.83922	0.215136	-8.54913	1.24E-17	5.07E-16	8.364359	8.148993	8.32767	10.20219	10.65207	9.64371
AT3G22120	753.0026	-1.83811	0.215225	-8.54042	1.34E-17	5.44E-16	8.368889	8.148993	8.32767	10.20365	10.65207	9.64371
AT4G06486	3.481846	-1.83737	0.502945	-3.65321	0.000259	0.002193	0	0	0	3.025182	3.546624	2.023222
AT1G56300	272.0544	-1.83639	0.184973	-9.92789	3.15E-23	1.81E-21	6.673172	6.882307	7.04036	9.012157	8.550692	8.626991
AT3G33197	4.696337	-1.83432	0.514614	-3.56445	0.000365	0.002957	0.60238	0	0.653468	2.797163	4.089834	2.610742
AT3G43291	3.397592	-1.83378	0.502885	-3.64652	0.000266	0.002242	0	0	0	2.192331	3.546624	2.833864
AT1G39590	3.046052	-1.83358	0.502596	-3.64822	0.000264	0.002229	0	0	0	2.526213	2.665087	3.197407
AT3G33115	4.022512	-1.83352	0.502069	-3.65193	0.00026	0.002203	0	0	0	1.131026	3.546624	3.728968
AT1G65970	102.8005	-1.83248	0.264604	-6.92538	4.35E-12	1.13E-10	5.589703	5.454712	5.072871	6.787815	7.63429	7.554276
AT2G10611	4.136967	-1.83044	0.509756	-3.5908	0.00033	0.002706	0.60238	0	0	1.757146	3.546624	3.613295
AT2G06150	4.174525	-1.83037	0.514551	-3.55721	0.000375	0.003032	0.60238	0	0.653468	3.025182	3.546624	2.833864
AT5G39410	177.2064	-1.82989	0.197824	-9.25008	2.24E-20	1.08E-18	6.47731	5.942585	6.238077	8.204403	8.203818	7.984953
AT2G04770	3.53978	-1.82987	0.50246	-3.64183	0.000271	0.002275	0	0	0	1.757146	3.546624	3.197407
AT2G13115	3.53978	-1.82987	0.50246	-3.64183	0.000271	0.002275	0	0	0	1.757146	3.546624	3.197407
AT4G12490	20.43644	-1.82828	0.45508	-4.01749	5.88E-05	0.000584	2.210244	2.917335	2.750488	4.700053	6.13862	3.728968
AT4G03923	3.5007	-1.82565	0.501786	-3.63831	0.000274	0.002303	0	0	0	1.757146	2.665087	3.836051
AT2G44790	31.65502	-1.82556	0.383893	-4.75538	1.98E-06	2.58E-05	4.092952	3.258709	3.173324	4.885743	6.13862	5.913005
AT3G31440	4.035631	-1.82288	0.513454	-3.55022	0.000385	0.003107	0	1.006035	0	3.549829	2.665087	3.027056
AT3G42650	4.231865	-1.82255	0.501898	-3.63132	0.000282	0.002357	0	0	0	1.131026	4.089834	3.197407
AT2G14170	1640.07	-1.82041	0.1439	-12.6505	1.11E-36	1.22E-34	9.51815	9.366276	9.506792	11.26155	11.57538	11.11567
AT3G46640	474.38	-1.81187	0.136338	-13.2896	2.66E-40	3.44E-38	7.56311	7.698487	7.821247	9.570448	9.538439	9.502512
AT5G57655	8545.117	-1.80822	0.125706	-14.3845	6.47E-47	1.09E-44	12.03123	11.78539	11.77289	13.72152	13.81854	13.56348
AT5G28230	3.470629	-1.80397	0.509316	-3.54193	0.000397	0.003192	0	0.588989	0	2.526213	2.665087	3.487532

AT3G27473	6.510493	-1.79671	0.513258	-3.5006	0.000464	0.00367	1.026049	0.588989	1.718868	3.549829	0	4.673514
AT5G26970	5.073307	-1.79671	0.51386	-3.4965	0.000471	0.003719	1.35313	0.588989	0.653468	3.025182	2.665087	4.028969
AT5G37390	2.931949	-1.79499	0.501114	-3.58201	0.000341	0.002792	0	0	0	3.025182	2.665087	2.610742
AT1G32225	2.903865	-1.7932	0.501058	-3.57883	0.000345	0.002821	0	0	0	2.797163	2.665087	2.833864
AT3G33091	2.903865	-1.7932	0.501058	-3.57883	0.000345	0.002821	0	0	0	2.797163	2.665087	2.833864
AT2G07630	3.412696	-1.79049	0.509173	-3.51648	0.000437	0.003481	0	0.588989	0	3.395221	2.665087	2.610742
AT2G01422	3.186476	-1.79011	0.500615	-3.57582	0.000349	0.002848	0	0	0	3.549829	2.665087	2.023222
AT5G48180	955.4467	-1.78771	0.180694	-9.89357	4.44E-23	2.53E-21	8.406831	9.057782	8.546845	10.51304	10.68989	10.4296
AT3G33181	7.535134	-1.78731	0.510539	-3.50082	0.000464	0.003668	0	1.006035	0	2.526213	5.262981	1.605623
AT1G09421	58.31499	-1.78605	0.295988	-6.0342	1.60E-09	3.24E-08	4.406711	4.739512	4.47242	6.457472	7.072174	5.984584
AT1G20390	68.51504	-1.78555	0.347886	-5.13257	2.86E-07	4.28E-06	4.664273	5.315522	4.041726	7.191776	6.952811	6.030403
AT1G20440	3144.888	-1.78533	0.145626	-12.2596	1.49E-34	1.49E-32	10.53239	10.49232	10.27044	12.38069	12.37493	11.99232
AT4G04105	3.017967	-1.77994	0.50028	-3.55789	0.000374	0.003025	0	0	0	2.192331	2.665087	3.34975
AT1G73120	27.70764	-1.77831	0.380427	-4.67451	2.95E-06	3.73E-05	3.368482	3.180636	3.937685	6.05437	5.262981	5.029925
AT3G42360	5.331659	-1.77647	0.49976	-3.55466	0.000378	0.003058	0	0	0	1.131026	4.792505	2.346685
AT5G29571	3.255404	-1.77605	0.500588	-3.54793	0.000388	0.00313	0	0	0	2.526213	3.546624	2.346685
AT1G36080	2.960034	-1.77573	0.500213	-3.54995	0.000385	0.003108	0	0	0	3.222033	2.665087	2.346685
AT2G09953	2.960034	-1.77573	0.500213	-3.54995	0.000385	0.003108	0	0	0	3.222033	2.665087	2.346685
AT1G79440	607.3573	-1.77527	0.160672	-11.049	2.22E-28	1.68E-26	7.961317	8.136123	8.087478	10.03059	10.01886	9.577734
AT3G30713	3.369507	-1.77216	0.500151	-3.54325	0.000395	0.003178	0	0	0	1.757146	3.546624	3.027056
AT1G72060	301.4819	-1.76857	0.281689	-6.27845	3.42E-10	7.34E-09	7.547209	6.43817	6.67823	8.337752	9.277863	8.94968
AT3G47340	30753.02	-1.76728	0.140424	-12.5853	2.54E-36	2.76E-34	13.83601	13.64487	13.74166	15.60978	15.71908	15.26768
AT3G33130	8.866418	-1.76481	0.515141	-3.42588	0.000613	0.004701	1.35313	0.588989	0.653468	2.526213	5.450922	2.023222
AT3G02550	181.9678	-1.7646	0.192606	-9.16171	5.11E-20	2.40E-18	6.47731	6.181803	6.227084	8.070295	8.444073	7.931619
AT1G37040	5.092477	-1.76381	0.512078	-3.44442	0.000572	0.004415	0	0.588989	0.653468	1.131026	4.089834	3.728968
AT1G22770	817.58	-1.75974	0.153849	-11.4381	2.70E-30	2.26E-28	8.238813	8.587553	8.663555	10.31566	10.4465	10.16656
AT1G49080	3.227319	-1.75905	0.499804	-3.51948	0.000432	0.003447	0	0	0	2.192331	3.546624	2.610742
AT1G30720	122.7086	-1.75739	0.294342	-5.97058	2.36E-09	4.70E-08	5.526228	5.76671	5.731672	6.657813	7.915398	7.943643
AT3G30330	4.687458	-1.75496	0.514785	-3.40912	0.000652	0.004942	1.026049	0	0.653468	2.192331	3.546624	3.728968
AT3G31442	3.865358	-1.75065	0.51232	-3.41711	0.000633	0.004819	0	1.006035	0	3.549829	2.665087	2.833864
AT4G12480	117.6367	-1.74759	0.29476	-5.92887	3.05E-09	5.96E-08	5.66531	5.01167	5.961278	7.082003	8.12318	7.249844
AT5G20250	11266.18	-1.74535	0.151257	-11.539	8.39E-31	7.16E-29	12.4573	12.21125	12.23825	13.93707	14.36855	13.92636
AT4G26288	297.159	-1.74248	0.208565	-8.35463	6.56E-17	2.55E-15	7.270463	6.900687	6.85286	8.846731	9.201369	8.452856

AT2G12815	2.94904	-1.74157	0.498199	-3.49574	0.000473	0.003729	0	0	0	3.395221	0	3.197407
AT2G04310	3.41316	-1.73992	0.506683	-3.43395	0.000595	0.004578	0	0	0.653468	3.549829	0	3.34975
AT2G38400	833.9824	-1.73662	0.196736	-8.82719	1.07E-18	4.62E-17	8.720396	8.283469	8.555659	10.4296	10.62304	9.882596
AT3G26740	11336.52	-1.73599	0.152563	-11.3788	5.33E-30	4.38E-28	12.20692	12.14775	12.57622	14.16308	14.22338	13.88993
AT2G11450	3.460209	-1.73423	0.505946	-3.42769	0.000609	0.004674	0.60238	0	0	3.816762	0	3.027056
AT2G23710	2.988119	-1.73366	0.498195	-3.47988	0.000502	0.003929	0	0	0	3.395221	2.665087	2.023222
AT2G45210	72.41863	-1.73353	0.267531	-6.47974	9.19E-11	2.08E-09	5.259717	4.818971	5.097223	7.156105	6.13862	6.98483
AT2G03990	2.920955	-1.73299	0.497809	-3.48124	0.000499	0.00391	0	0	0	3.222033	0	3.34975
AT1G49130	1452.855	-1.73291	0.192857	-8.98548	2.58E-19	1.14E-17	9.553402	9.252816	9.203139	11.50666	11.06209	10.75196
AT1G21670	522.1792	-1.73179	0.148785	-11.6395	2.59E-31	2.26E-29	7.839308	7.773541	8.025383	9.69789	9.802877	9.465464
AT1G72070	314.3042	-1.73159	0.275012	-6.29643	3.05E-10	6.59E-09	7.617422	6.503765	6.85286	8.420261	9.314642	8.993715
AT2G23030	48.43805	-1.73156	0.308646	-5.61017	2.02E-08	3.55E-07	4.882744	4.098802	4.138765	6.514927	6.13862	6.138962
AT4G24050	210.947	-1.72781	0.203852	-8.47583	2.33E-17	9.33E-16	6.858537	6.351747	6.441977	8.554188	8.376102	8.123525
AT2G43400	894.282	-1.72294	0.157476	-10.9409	7.34E-28	5.34E-26	8.764041	8.623039	8.579623	10.33041	10.72675	10.20203
AT2G34790	44.39321	-1.71894	0.29615	-5.80429	6.46E-09	1.21E-07	4.296891	4.535085	4.273086	6.15418	5.617177	6.489965
AT5G16370	1901.435	-1.7188	0.16333	-10.5234	6.74E-26	4.49E-24	9.97491	9.561813	9.692069	11.51137	11.75113	11.26389
AT5G57630	502.5082	-1.71869	0.190272	-9.03279	1.67E-19	7.57E-18	8.128263	7.626693	7.646629	9.695821	9.785514	9.302294
AT5G63810	746.716	-1.71772	0.165834	-10.3581	3.84E-25	2.43E-23	8.170394	8.613785	8.388133	10.17274	10.37864	9.945234
AT1G03580	146.8418	-1.7151	0.213911	-8.01781	1.08E-15	3.79E-14	6.354774	5.793186	5.893371	7.789056	8.037767	7.68894
AT2G12770	4.829646	-1.71076	0.513917	-3.32886	0.000872	0.006409	1.026049	0	0.653468	1.757146	3.546624	3.935733
AT3G33142	5.006218	-1.70585	0.505617	-3.37379	0.000741	0.005537	0	0.588989	0	3.025182	4.483533	1.015523
AT3G27327	2.761677	-1.70426	0.496972	-3.42928	0.000605	0.00465	0	0	0	3.025182	2.665087	2.346685
AT3G31980	2.761677	-1.70426	0.496972	-3.42928	0.000605	0.00465	0	0	0	3.025182	2.665087	2.346685
AT2G41280	4.245576	-1.70335	0.515083	-3.30694	0.000943	0.00685	0.60238	1.006035	0.653468	3.689461	2.665087	2.833864
AT5G17460	38.23128	-1.70122	0.329099	-5.16934	2.35E-07	3.56E-06	3.95514	4.011295	4.273086	6.335206	5.90133	5.456434
AT3G07650	146.115	-1.70014	0.193761	-8.77443	1.72E-18	7.33E-17	6.250617	6.109902	5.851038	7.765576	7.78168	7.919495
AT3G30767	2.705507	-1.69964	0.496778	-3.42132	0.000623	0.004766	0	0	0	2.526213	2.665087	2.833864
AT3G42251	2.705507	-1.69964	0.496778	-3.42132	0.000623	0.004766	0	0	0	2.526213	2.665087	2.833864
AT5G32169	3.521168	-1.69812	0.507922	-3.34326	0.000828	0.006119	0	0	0.653468	2.526213	3.546624	2.610742
AT1G53885	34.93093	-1.69793	0.332444	-5.10743	3.27E-07	4.85E-06	4.136111	3.766043	3.703969	5.800813	6.244072	5.315499
AT1G53903	34.93093	-1.69793	0.332444	-5.10743	3.27E-07	4.85E-06	4.136111	3.766043	3.703969	5.800813	6.244072	5.315499
AT4G06652	3.489706	-1.69734	0.495916	-3.42263	0.00062	0.004752	0	0	0	2.192331	0	4.199107
AT4G17243	13.53838	-1.69654	0.451394	-3.75844	0.000171	0.001518	2.951747	1.593003	2.324987	4.324977	5.046837	4.199107

AT4G27260	2513.5	-1.69488	0.17048	-9.9418	2.74E-23	1.58E-21	10.39977	9.937587	10.14173	11.87632	12.18274	11.65155
AT1G55510	474.9933	-1.69338	0.162269	-10.4357	1.70E-25	1.10E-23	7.78949	7.715891	7.780474	9.498685	9.785514	9.225585
AT2G15960	4296.875	-1.69207	0.180458	-9.37652	6.82E-21	3.39E-19	11.26533	10.71508	10.79812	12.96685	12.53674	12.53047
AT2G43830	65.91965	-1.6918	0.251272	-6.73294	1.66E-11	4.09E-10	4.907866	4.869612	4.889826	6.772191	6.88921	6.405552
AT1G64660	2963.456	-1.68978	0.168455	-10.0311	1.11E-23	6.53E-22	10.1575	10.49736	10.55772	11.95324	12.45124	12.01066
AT3G06850	909.5196	-1.68142	0.168716	-9.96601	2.15E-23	1.24E-21	8.723936	8.604471	8.794047	10.26772	10.79775	10.22191
AT5G32400	3.085132	-1.67618	0.495801	-3.38075	0.000723	0.005417	0	0	0	2.526213	3.546624	2.023222
AT4G15610	135.1458	-1.67585	0.30279	-5.53468	3.12E-08	5.33E-07	5.9456	5.906661	5.747144	6.657813	8.150563	8.002302
AT1G19540	260.5356	-1.67573	0.231152	-7.24947	4.18E-13	1.18E-11	6.981957	6.924835	6.645579	8.327098	9.188216	8.306906
AT2G07620	36.71638	-1.67353	0.33397	-5.01102	5.41E-07	7.79E-06	4.21874	4.22085	3.345839	5.672031	6.244072	5.702706
AT1G42410	3.493083	-1.67117	0.50692	-3.29671	0.000978	0.007045	0	0	0.653468	2.192331	3.546624	2.833864
AT4G05636	2.750682	-1.66882	0.494727	-3.37323	0.000743	0.005546	0	0	0	3.222033	0	3.197407
AT2G04120	9.821294	-1.66316	0.470981	-3.53128	0.000414	0.00331	2.501815	1.593003	1.718868	3.933736	4.483533	3.935733
AT5G19097	5.591148	-1.65962	0.510189	-3.25296	0.001142	0.00806	1.35313	1.329167	0.653468	3.816762	3.546624	2.833864
AT3G51400	73.46785	-1.6578	0.241875	-6.85394	7.18E-12	1.82E-10	5.050049	5.056075	5.072871	6.818564	7.128345	6.55411
AT3G29633	12.11903	-1.65693	0.451249	-3.67187	0.000241	0.002058	2.210244	2.817685	1.950366	3.933736	4.483533	4.673514
AT2G14010	4.005774	-1.65556	0.505802	-3.27313	0.001064	0.007579	0.60238	0	0	1.757146	4.089834	2.610742
AT5G29034	3.018319	-1.64964	0.505319	-3.26455	0.001096	0.00778	0.60238	0	0	3.025182	2.665087	2.610742
AT5G61440	205.8623	-1.64713	0.182937	-9.00377	2.18E-19	9.78E-18	6.765398	6.596882	6.413169	8.405146	8.399116	8.096855
AT3G55450	222.6909	-1.6452	0.16862	-9.75685	1.72E-22	9.49E-21	6.864965	6.684352	6.718032	8.49355	8.150563	8.542549
AT5G34835	3.425918	-1.64306	0.503864	-3.26093	0.00111	0.007861	0	0	0.653468	1.757146	2.665087	3.728968
AT4G06525	3.113217	-1.64153	0.493809	-3.32423	0.000887	0.006506	0	0	0	2.797163	3.546624	1.605623
AT5G66170	68.61398	-1.63847	0.255463	-6.41371	1.42E-10	3.16E-09	5.004194	5.201541	4.74322	6.476879	6.88921	6.837962
AT4G04330	244.8439	-1.63783	0.221929	-7.37997	1.58E-13	4.66E-12	6.485677	6.86369	7.17229	8.850455	8.150563	8.566073
AT3G15620	133.6162	-1.63745	0.202838	-8.0727	6.88E-16	2.46E-14	6.148927	5.942585	5.777599	7.676061	7.883112	7.490138
AT5G28050	917.2523	-1.63563	0.148941	-10.9818	4.68E-28	3.47E-26	8.777734	8.691496	8.829001	10.39814	10.70382	10.21076
AT5G54165	25.3619	-1.63391	0.370785	-4.40662	1.05E-05	0.000121	3.95514	3.596007	2.868369	5.243916	5.262981	5.553778
AT4G05594	2.959812	-1.62871	0.504392	-3.22906	0.001242	0.008653	0	0.588989	0	2.526213	2.665087	3.027056
AT4G25000	26.05609	-1.62794	0.424966	-3.83075	0.000128	0.001174	3.045281	4.140642	2.324987	4.885743	6.024848	5.199956
AT1G58180	1403.017	-1.62716	0.152897	-10.6422	1.90E-26	1.31E-24	9.536387	9.40649	9.214314	11.15565	11.2028	10.78992
AT3G30587	2.96215	-1.62577	0.504402	-3.22317	0.001268	0.008812	0.60238	0	0	2.526213	2.665087	3.027056
AT5G59570	39.62033	-1.62431	0.317586	-5.11456	3.14E-07	4.69E-06	4.178017	4.535085	3.990643	5.800813	5.617177	6.352426
AT2G11090	2.563319	-1.62373	0.492777	-3.29506	0.000984	0.007077	0	0	0	2.797163	2.665087	2.346685

AT4G04635	2.563319	-1.62373	0.492777	-3.29506	0.000984	0.007077	0	0	0	2.797163	2.665087	2.346685
AT4G03880	3.341774	-1.62195	0.505124	-3.211	0.001323	0.009125	0.60238	0	0	2.526213	3.546624	2.346685
AT2G12050	2.535235	-1.62092	0.492633	-3.29032	0.001001	0.007183	0	0	0	2.526213	2.665087	2.610742
AT4G16146	118.192	-1.61988	0.296016	-5.47228	4.44E-08	7.48E-07	5.354131	5.581648	6.248986	8.037996	7.234523	7.096741
AT2G06105	3.11909	-1.6155	0.501454	-3.22164	0.001275	0.008845	0	0.588989	0	2.797163	0	3.728968
AT5G58660	9.701699	-1.61506	0.475912	-3.3936	0.00069	0.005201	2.038899	2.179302	1.442988	3.689461	4.792505	3.613295
AT1G07985	49.00917	-1.61463	0.271032	-5.95736	2.56E-09	5.07E-08	4.406711	4.684	4.614164	6.313781	6.13862	6.29727
AT1G13930	2238.041	-1.60866	0.16736	-9.61197	7.12E-22	3.75E-20	9.863756	10.06799	10.27845	12.01636	11.55759	11.56517
AT2G13940	34.2211	-1.60723	0.349115	-4.60373	4.15E-06	5.13E-05	3.294214	4.369011	3.990643	5.414645	6.024848	5.83769
AT4G08430	2.591404	-1.60045	0.491373	-3.25709	0.001126	0.007957	0	0	0	3.025182	2.665087	2.023222
AT2G24780	2.58041	-1.59873	0.490971	-3.25625	0.001129	0.007974	0	0	0	3.222033	0	3.027056
AT3G33175	4.270944	-1.59861	0.491106	-3.25512	0.001133	0.008003	0	0	0	1.131026	4.483533	2.023222
AT3G60140	37.24318	-1.59693	0.372181	-4.29073	1.78E-05	0.000196	3.748168	4.626265	3.571186	5.197878	6.024848	6.278406
AT5G67480	1851.097	-1.59608	0.129464	-12.3283	6.38E-35	6.55E-33	9.941846	9.677366	9.847292	11.54624	11.49754	11.29179
AT3G05870	227.4887	-1.59553	0.249848	-6.38601	1.70E-10	3.75E-09	6.723563	6.773485	6.867092	8.901615	7.554534	8.542549
AT2G04330	2.50715	-1.59177	0.490901	-3.24254	0.001185	0.008308	0	0	0	2.192331	2.665087	2.833864
AT3G37820	31.49118	-1.59059	0.493773	-3.2213	0.001276	0.008851	0	0	0.653468	4.885743	7.234523	3.487532
AT5G32161	2.552325	-1.58819	0.490368	-3.23878	0.0012	0.008405	0	0	0	3.025182	0	3.197407
AT5G16340	186.7591	-1.58362	0.18545	-8.53937	1.35E-17	5.49E-16	6.680479	6.351747	6.479514	8.255864	8.229725	7.949617
AT2G28630	892.1099	-1.57937	0.207059	-7.62764	2.39E-14	7.58E-13	9.226473	8.360736	8.526815	10.45306	10.42423	10.32892
AT3G48360	3632.777	-1.57928	0.175435	-9.00208	2.21E-19	9.91E-18	11.11669	10.597	10.60572	12.55316	12.52633	12.15661
AT3G59880	17.92803	-1.57861	0.408253	-3.86674	0.00011	0.00103	3.133118	2.595007	3.173324	4.997462	5.262981	4.277199
AT1G77210	1231.43	-1.57752	0.18824	-8.38034	5.28E-17	2.07E-15	9.45989	8.880842	9.286244	11.03646	10.98812	10.51467
AT1G47890	23.65481	-1.57467	0.43603	-3.61137	0.000305	0.002522	2.74425	3.180636	3.766043	3.816762	5.766238	5.644966
AT4G04180	86.16712	-1.57237	0.233793	-6.72546	1.75E-11	4.28E-10	5.509914	5.351599	5.341032	6.772191	7.072174	7.249844
AT4G39780	98.80064	-1.57213	0.236729	-6.64106	3.11E-11	7.49E-10	5.883635	5.27852	5.651719	7.439382	6.952811	7.259495
AT2G19450	130.305	-1.57074	0.199654	-7.8673	3.62E-15	1.22E-13	5.883635	6.077955	6.100294	7.827358	7.51294	7.514525
AT2G02930	134.9845	-1.57059	0.289988	-5.41604	6.09E-08	1.01E-06	6.468894	5.844724	5.400949	7.11953	8.037767	7.818645
AT3G28153	9.528597	-1.56588	0.47683	-3.28394	0.001024	0.007335	2.210244	1.593003	1.950366	3.549829	4.792505	3.613295
AT3G28923	2.52424	-1.56228	0.488842	-3.19589	0.001394	0.009547	0	0	0	2.797163	0	3.34975
AT3G12750	33.20484	-1.55987	0.338626	-4.60648	4.10E-06	5.07E-05	4.136111	4.333373	3.571186	5.637956	5.262981	6.074811
AT5G66053	185.2658	-1.55893	0.237915	-6.55246	5.66E-11	1.32E-09	6.896684	5.906661	6.507039	8.132803	8.229725	8.058669
AT1G18270	1575.901	-1.555	0.162243	-9.58439	9.30E-22	4.87E-20	9.651483	9.640475	9.52822	11.11752	11.53179	10.92037

AT1G53580	708.3506	-1.55281	0.153806	-10.0959	5.76E-24	3.42E-22	8.475654	8.429862	8.460313	10.06306	10.30134	9.767246
AT1G60740	24.55138	-1.54706	0.381739	-4.05267	5.06E-05	0.000509	3.506448	3.180636	3.499911	4.632436	6.024848	4.984091
AT5G23660	445.0762	-1.54686	0.192439	-8.03818	9.12E-16	3.23E-14	7.308691	7.927874	8.028552	9.428158	9.419665	9.32085
AT1G15125	349.1557	-1.54006	0.461244	-3.33893	0.000841	0.006203	7.896905	6.412782	6.03876	8.61676	9.638536	9.028
AT1G49000	22.00358	-1.535	0.411467	-3.73054	0.000191	0.001677	3.748168	2.179302	3.703969	5.331804	5.046837	5.074348
AT2G46610	191.0053	-1.53103	0.178118	-8.59562	8.28E-18	3.39E-16	6.723563	6.43817	6.534049	8.083015	8.376102	8.041989
AT2G23170	674.2569	-1.52596	0.194687	-7.83801	4.58E-15	1.52E-13	8.371149	8.427751	8.380712	10.19342	10.19358	9.4529
AT3G51325	16.03049	-1.52108	0.420873	-3.61411	0.000301	0.0025	3.133118	2.710638	2.750488	4.942684	5.046837	4.028969
AT4G36930	55.15178	-1.52065	0.260952	-5.82732	5.63E-09	1.06E-07	4.72207	4.844514	4.773757	6.476879	6.520817	6.200381
AT3G59350	1756.016	-1.51554	0.142237	-10.655	1.65E-26	1.15E-24	9.866161	9.571406	9.951978	11.3365	11.49487	11.22007
AT3G13310	369.5502	-1.51527	0.154894	-9.78259	1.34E-22	7.39E-21	7.741388	7.531616	7.40955	9.035249	9.049638	9.228045
AT5G66052	768.5829	-1.5123	0.206186	-7.33465	2.22E-13	6.43E-12	9.001359	8.064568	8.568779	10.15478	10.17369	10.18314
AT3G05845	23.82693	-1.51188	0.355883	-4.24826	2.15E-05	0.000233	3.57078	3.534563	3.766043	5.567291	4.792505	5.199956
AT3G11550	11.19903	-1.50218	0.463129	-3.24355	0.00118	0.008284	2.74425	1.593003	2.324987	4.041933	4.792505	3.836051
AT4G38470	1646.102	-1.49799	0.141467	-10.589	3.35E-26	2.27E-24	9.767537	9.683571	9.712902	11.16615	11.51345	11.05531
AT5G02580	78.52571	-1.49262	0.312081	-4.78281	1.73E-06	2.27E-05	5.778598	4.988944	5.097223	7.336904	5.90133	7.075043
AT3G29631	23.92242	-1.49106	0.356163	-4.18645	2.83E-05	0.0003	3.57078	3.534563	3.825555	5.567291	4.792505	5.199956
AT2G30615	424.4251	-1.4891	0.147542	-10.0927	5.95E-24	3.52E-22	7.709596	7.753456	7.821247	9.16055	9.538439	9.193218
AT5G64260	1503.085	-1.48501	0.166927	-8.89616	5.78E-19	2.53E-17	9.793885	9.229982	9.703019	11.08073	11.28232	10.99881
AT3G51730	2548.771	-1.46952	0.145269	-10.1158	4.70E-24	2.80E-22	10.48466	10.20925	10.41971	11.84725	12.09737	11.66432
AT2G39980	231.7277	-1.46689	0.185024	-7.9281	2.23E-15	7.64E-14	7.084671	6.838488	6.787021	8.590275	8.508985	8.160054
AT2G24195	18.99877	-1.46245	0.382179	-3.8266	0.00013	0.00119	3.133118	3.332773	3.173324	4.764642	5.262981	4.730144
AT1G11210	180.097	-1.46222	0.207645	-7.0419	1.90E-12	5.08E-11	6.298895	6.611834	6.710159	8.310969	8.066803	7.778931
AT5G13220	48.76435	-1.46145	0.289941	-5.04051	4.64E-07	6.72E-06	4.693461	4.503365	4.580013	5.737858	6.602406	6.239921
AT5G13330	50.69581	-1.45842	0.324981	-4.48771	7.20E-06	8.55E-05	4.541232	4.818971	4.580013	5.454347	6.520817	6.630478
AT1G75820	1561.791	-1.45787	0.135732	-10.7408	6.55E-27	4.61E-25	9.784591	9.472484	9.776487	11.26365	11.19296	11.04139
AT4G12520	26.88915	-1.45103	0.37081	-3.91313	9.11E-05	0.000871	3.506448	3.711561	4.138765	4.997462	5.046837	5.913005
AT5G63600	22.27803	-1.44909	0.412119	-3.51618	0.000438	0.003484	2.628119	4.055712	3.499911	5.050236	4.483533	5.644966
AT4G16690	141.1709	-1.44899	0.232348	-6.23632	4.48E-10	9.51E-09	6.399741	6.141156	5.865288	7.507017	8.150563	7.440091
AT2G45360	19.79612	-1.44615	0.415634	-3.47937	0.000503	0.003934	4.002555	2.917335	2.868369	5.288529	4.089834	5.239508
AT2G39705	154.1286	-1.44172	0.195665	-7.3683	1.73E-13	5.05E-12	6.451914	6.250291	6.333412	8.031448	7.850087	7.600575
AT5G05880	12.56207	-1.43182	0.445519	-3.21383	0.00131	0.00906	2.851726	2.469294	2.977342	4.997462	2.665087	4.488922
AT4G37450	84.3451	-1.43084	0.238155	-6.00804	1.88E-09	3.77E-08	5.354131	5.259657	5.668068	6.892686	7.284819	6.744835

AT3G16380	105.5182	-1.42928	0.20581	-6.94466	3.79E-12	9.89E-11	5.870916	5.906661	5.635183	7.326244	7.33342	7.220496
AT1G10060	253.0373	-1.42903	0.169316	-8.44003	3.17E-17	1.25E-15	7.159792	6.84483	7.222966	8.634151	8.48767	8.494318
AT3G15450	20931.22	-1.42746	0.143202	-9.96813	2.10E-23	1.22E-21	13.60274	13.24341	13.47831	15.09564	14.86907	14.72622
AT5G02540	1656.285	-1.42617	0.191175	-7.46002	8.65E-14	2.61E-12	9.649622	9.637738	10.00442	11.57916	11.24152	10.84224
AT3G04070	28.08822	-1.42395	0.361041	-3.944	8.01E-05	0.000774	3.855363	3.766043	3.639105	4.700053	6.024848	5.42247
AT5G05320	34.72437	-1.4219	0.326313	-4.35747	1.32E-05	0.000149	4.508759	3.965467	3.937685	5.414645	5.90133	5.863234
AT5G10430	74.18797	-1.42154	0.243216	-5.84477	5.07E-09	9.62E-08	5.199953	5.161454	5.361282	6.588138	7.182411	6.585144
AT4G08950	3339.254	-1.41897	0.169986	-8.34758	6.97E-17	2.70E-15	10.7808	10.50739	11.05435	11.98705	12.36327	12.39117
AT2G29090	27.06627	-1.41765	0.39302	-3.60708	0.00031	0.002558	2.628119	3.918135	4.184943	5.197878	5.766238	5.239508
AT5G54090	326.5794	-1.41752	0.180992	-7.83192	4.80E-15	1.60E-13	7.640086	7.306589	7.319272	8.947549	9.134371	8.615763
AT1G09460	46.37974	-1.41572	0.293601	-4.8219	1.42E-06	1.91E-05	4.956834	4.296832	4.47242	6.001749	6.244072	6.159727
AT5G57560	7622.111	-1.4111	0.128908	-10.9466	6.90E-28	5.05E-26	12.05233	11.88125	12.08307	13.26076	13.4338	13.6096
AT1G80920	7835.426	-1.41109	0.14373	-9.81765	9.45E-23	5.28E-21	12.15933	11.9461	12.01719	13.69691	13.48847	13.22594
AT2G22450	407.6118	-1.40984	0.142798	-9.87296	5.45E-23	3.08E-21	7.861981	7.687943	7.821247	9.354005	9.148022	9.141961
AT2G22720	1106.28	-1.40949	0.140432	-10.0368	1.05E-23	6.18E-22	9.299479	9.183201	9.154652	10.62274	10.88185	10.44976
AT2G37130	164.892	-1.40866	0.26293	-5.35754	8.44E-08	1.37E-06	6.799356	6.221336	6.216008	7.409395	7.883112	8.361702
AT5G06690	880.4047	-1.40304	0.147668	-9.50132	2.07E-21	1.06E-19	8.884414	8.780202	9.016241	10.51187	10.33754	10.109
AT1G69890	410.9057	-1.40199	0.161776	-8.66623	4.47E-18	1.86E-16	7.988066	7.719346	7.638356	9.035249	9.33865	9.311602
AT1G08500	41.81167	-1.3981	0.303921	-4.60021	4.22E-06	5.21E-05	4.57299	4.181303	4.434696	5.603056	6.434336	5.863234
AT1G28230	45.61031	-1.39588	0.291874	-4.78247	1.73E-06	2.27E-05	4.406711	4.403791	4.832959	5.831289	6.434336	6.030403
AT5G10435	59.4444	-1.39529	0.25662	-5.4372	5.41E-08	9.02E-07	4.907866	4.844514	5.121171	6.313781	6.752923	6.352426
AT4G38580	165.2926	-1.39222	0.214425	-6.49279	8.43E-11	1.92E-09	6.190467	6.360626	6.831246	8.083015	7.850087	7.825159
AT1G08803	21.18013	-1.38981	0.384323	-3.61625	0.000299	0.002482	3.215913	3.470386	3.424931	4.408199	5.617177	4.984091
AT1G20620	38242.82	-1.38975	0.136557	-10.1771	2.51E-24	1.53E-22	14.47859	14.19541	14.35664	15.66674	15.97205	15.62678
AT1G04903	1730.261	-1.38895	0.197171	-7.0444	1.86E-12	5.01E-11	9.888419	9.932385	9.745045	10.76766	11.66118	11.35674
AT1G04907	1730.09	-1.38876	0.197161	-7.0438	1.87E-12	5.02E-11	9.888419	9.932385	9.745045	10.76766	11.66118	11.35617
AT3G11580	51.66241	-1.38724	0.302333	-4.58845	4.47E-06	5.48E-05	4.932558	4.655421	4.712022	6.640707	6.342338	5.730732
AT1G55810	425.4094	-1.3857	0.169277	-8.18597	2.70E-16	9.94E-15	7.999797	7.655841	7.835792	9.204868	9.548767	9.058728
AT5G49450	799.142	-1.38346	0.167976	-8.23609	1.78E-16	6.66E-15	8.909507	8.477577	8.854225	10.26074	10.34348	9.928694
AT2G28120	446.3229	-1.37886	0.154257	-8.93872	3.94E-19	1.74E-17	8.079347	7.736502	7.943807	9.382583	9.463995	9.180574
AT5G52190	249.412	-1.37721	0.174034	-7.91344	2.50E-15	8.55E-14	7.068052	6.97195	7.143347	8.405146	8.813085	8.288168
AT5G01600	4867.665	-1.37718	0.215211	-6.39921	1.56E-10	3.46E-09	11.55966	10.85045	11.54208	12.70179	13.15749	12.44239
AT5G14180	19.86348	-1.3762	0.374925	-3.67059	0.000242	0.002065	3.368482	3.332773	3.703969	5.197878	4.483533	5.029925

AT1G65845	128.1522	-1.37618	0.298137	-4.61593	3.91E-06	4.86E-05	6.566781	5.333673	5.987571	7.082003	7.946977	7.600575
AT3G26512	762.0433	-1.37429	0.195265	-7.03809	1.95E-12	5.22E-11	8.767477	8.501859	8.754402	10.02401	10.52183	9.708563
AT4G01250	423.056	-1.36569	0.198584	-6.87714	6.11E-12	1.56E-10	8.185881	7.574224	7.683285	9.275834	9.496371	9.0081
AT4G39090	9008.746	-1.36063	0.135439	-10.0461	9.56E-24	5.63E-22	12.12977	12.20125	12.4955	13.73933	13.73394	13.52561
AT1G26558	28.61001	-1.35899	0.346715	-3.91963	8.87E-05	0.00085	3.95514	3.654941	4.315217	5.800813	5.046837	5.352044
AT1G19380	243.5152	-1.35732	0.239903	-5.65778	1.53E-08	2.72E-07	7.246046	6.596882	7.195033	7.977969	8.591227	8.762127
AT1G19780	75.04636	-1.35716	0.238333	-5.69437	1.24E-08	2.23E-07	5.620423	5.333673	5.167905	6.818564	6.679627	6.850789
AT1G62480	3471.597	-1.35321	0.185856	-7.28093	3.32E-13	9.45E-12	11.20263	10.70291	10.72382	12.12105	12.61991	12.09393
AT2G37030	44.95913	-1.35225	0.279152	-4.84413	1.27E-06	1.72E-05	4.57299	4.535085	4.803662	6.17809	6.024848	5.984584
AT5G51790	126.4547	-1.35119	0.225772	-5.98475	2.17E-09	4.33E-08	6.408569	6.034228	5.762452	7.429455	7.78168	7.388246
AT3G61070	519.6149	-1.35047	0.163456	-8.26198	1.43E-16	5.43E-15	8.287481	8.000682	8.161518	9.338176	9.836987	9.465464
AT5G26740	576.9019	-1.34804	0.142732	-9.44459	3.57E-21	1.80E-19	8.471447	8.223893	8.248344	9.662309	9.87031	9.575804
AT2G26750	84.36653	-1.34359	0.217209	-6.18573	6.18E-10	1.29E-08	5.605144	5.535346	5.531776	7.017205	6.952811	6.876105
AT3G45970	815.3888	-1.34184	0.207286	-6.47339	9.58E-11	2.17E-09	8.688132	8.36956	9.226784	10.17125	10.40162	10.03648
AT5G03240	4354.941	-1.34106	0.12543	-10.6917	1.11E-26	7.79E-25	11.32948	11.1455	11.27387	12.68338	12.72497	12.41808
AT5G58600	146.2018	-1.33873	0.228634	-5.85533	4.76E-09	9.08E-08	6.180193	6.141156	6.534049	7.709258	8.12318	7.316087
AT1G02620	33.57401	-1.33759	0.393969	-3.39516	0.000686	0.005176	4.907866	3.470386	3.499911	6.001749	5.617177	5.387687
AT1G26665	317.5781	-1.33659	0.192452	-6.94504	3.78E-12	9.88E-11	7.802944	7.269354	7.261186	9.008827	8.795846	8.717307
AT1G30110	63.18616	-1.32665	0.273035	-4.8589	1.18E-06	1.61E-05	5.240069	4.626265	5.381252	6.533581	6.679627	6.422834
AT2G01080	99.7678	-1.32534	0.220002	-6.02422	1.70E-09	3.43E-08	5.792154	5.596757	6.06369	7.27173	7.013727	7.230345
AT4G03510	960.7088	-1.32406	0.149346	-8.86569	7.60E-19	3.31E-17	9.156142	8.917407	9.160441	10.6379	10.39018	10.23909
AT1G21680	2749.22	-1.32072	0.136511	-9.67487	3.86E-22	2.07E-20	10.61289	10.57619	10.60572	11.98958	12.13384	11.68372
AT5G10860	3181.27	-1.31943	0.126679	-10.4155	2.11E-25	1.35E-23	10.87617	10.68269	10.87231	12.16994	12.29896	11.98325
AT1G22740	755.4847	-1.31895	0.160806	-8.20211	2.36E-16	8.74E-15	8.918805	8.591329	8.659472	10.22532	10.18698	9.817115
AT1G54740	2028.625	-1.31808	0.146766	-8.98082	2.69E-19	1.19E-17	10.35937	10.02046	10.07968	11.61682	11.59793	11.28943
AT3G51330	102.4915	-1.3175	0.259757	-5.07206	3.94E-07	5.77E-06	6.289368	5.596757	5.400949	7.144016	7.380437	7.170215
AT5G54960	216.7397	-1.31623	0.169899	-7.74714	9.40E-15	3.05E-13	6.779077	7.03432	7.052865	8.358826	8.095267	8.370636
AT5G58650	141.0127	-1.31453	0.224222	-5.86262	4.56E-09	8.72E-08	6.417342	5.977637	6.353768	7.571623	8.066803	7.352617
AT5G24490	7134.396	-1.31289	0.135362	-9.69914	3.04E-22	1.64E-20	11.85898	11.97114	12.10744	13.45996	13.38131	13.09208
AT5G18937	24.85796	-1.31157	0.354723	-3.69745	0.000218	0.001884	4.048462	3.470386	3.703969	4.997462	5.450922	5.278004
AT3G20395	104.3402	-1.31131	0.250802	-5.22847	1.71E-07	2.65E-06	6.220856	5.487509	5.716032	7.304685	7.47011	6.961367
AT1G62770	33.32968	-1.30867	0.333099	-3.92879	8.54E-05	0.000821	3.95514	4.055712	4.614164	5.947137	5.450922	5.456434
AT1G08115	15303.68	-1.30691	0.190921	-6.8453	7.63E-12	1.93E-10	13.14505	13.05122	13.01032	13.89215	14.76079	14.49345

AT3G51430	254.4008	-1.30547	0.195487	-6.67804	2.42E-11	5.88E-10	7.386711	6.918835	7.119768	8.204403	8.706416	8.615763
AT5G22920	3533.224	-1.30543	0.117365	-11.1228	9.72E-29	7.63E-27	11.04702	10.90458	10.97936	12.39064	12.3662	12.13711
AT3G26210	285.5202	-1.30327	0.270493	-4.81811	1.45E-06	1.94E-05	7.578837	7.201787	7.034067	7.849861	8.896321	9.083391
AT5G18130	769.7053	-1.29854	0.136044	-9.54495	1.36E-21	7.05E-20	8.935698	8.69501	8.701774	10.19781	10.13309	9.974829
AT5G57565	222.896	-1.29534	0.173674	-7.45847	8.75E-14	2.63E-12	7.017014	6.900687	7.008616	8.405146	8.48767	8.064187
AT5G21170	1881.56	-1.28944	0.164451	-7.84088	4.47E-15	1.49E-13	10.18794	9.843339	10.14246	11.43653	11.61769	11.08071
AT4G12470	185.9981	-1.28913	0.263419	-4.89383	9.89E-07	1.37E-05	6.673172	6.181803	7.021398	7.607282	8.571102	7.919495
AT5G07745	4644.906	-1.28731	0.248788	-5.17432	2.29E-07	3.48E-06	11.44769	11.46402	11.06442	11.84585	13.11034	12.89022
AT1G12710	113.0943	-1.28551	0.212738	-6.04267	1.52E-09	3.07E-08	5.921133	5.918735	6.281227	7.488014	7.072174	7.423016
AT4G03210	2004.527	-1.28347	0.099855	-12.8534	8.24E-38	9.44E-36	10.16728	10.13227	10.24137	11.46171	11.4922	11.4764
AT4G27130	913.7794	-1.27863	0.160536	-7.96474	1.66E-15	5.75E-14	9.264654	8.743336	9.058711	10.45429	10.36702	10.21449
AT1G15330	18.06456	-1.27712	0.383833	-3.32727	0.000877	0.006439	3.215913	3.332773	3.639105	4.885743	4.483533	4.936752
AT5G01215	353.5373	-1.27697	0.190894	-6.68945	2.24E-11	5.45E-10	7.336712	7.666621	7.878569	8.857876	9.277863	8.789033
AT5G01210	353.5373	-1.27697	0.190894	-6.68945	2.24E-11	5.45E-10	7.336712	7.666621	7.878569	8.857876	9.277863	8.789033
AT4G09030	155.835	-1.27665	0.282345	-4.5216	6.14E-06	7.39E-05	6.354774	6.181803	6.725863	7.017205	7.946977	8.249946
AT5G02935	33.38964	-1.2763	0.325377	-3.92253	8.76E-05	0.000842	4.475539	3.766043	4.356153	5.603056	5.766238	5.553778
AT5G64570	1071.109	-1.27593	0.194778	-6.55068	5.73E-11	1.34E-09	9.683658	8.961847	9.001802	10.61291	10.66637	10.45922
AT1G12780	3176.692	-1.27422	0.101174	-12.5943	2.27E-36	2.48E-34	10.87577	10.854	10.81978	12.17292	12.19594	12.0428
AT2G26740	666.8761	-1.27345	0.12567	-10.1333	3.93E-24	2.37E-22	8.608007	8.564682	8.592528	9.905487	10.01141	9.750233
AT5G16850	18.26503	-1.26957	0.383758	-3.30827	0.000939	0.006822	3.294214	3.332773	3.639105	4.764642	4.483533	5.074348
AT4G02520	3490.524	-1.2681	0.233088	-5.44042	5.32E-08	8.87E-07	11.41602	10.77525	10.49266	11.82423	12.55094	12.39774
AT2G30600	2451.818	-1.26674	0.108136	-11.7143	1.08E-31	9.50E-30	10.42555	10.51387	10.49896	11.80421	11.8358	11.6442
AT5G64572	618.3199	-1.26297	0.192128	-6.57358	4.91E-11	1.16E-09	8.87966	8.223893	8.215351	9.809252	9.84539	9.699728
AT1G06560	318.8195	-1.25629	0.192266	-6.53411	6.40E-11	1.48E-09	7.748358	7.425602	7.370122	9.04828	8.896321	8.494318
AT5G07100	161.4557	-1.25209	0.231977	-5.39745	6.76E-08	1.11E-06	6.68775	6.495726	6.383775	7.315505	8.037767	8.107583
AT1G13920	28.65946	-1.24983	0.338059	-3.69709	0.000218	0.001886	4.048462	4.011295	4.138765	5.769679	5.262981	5.117444
AT1G62440	36.14991	-1.24786	0.337257	-3.70001	0.000216	0.001867	4.475539	3.869198	4.395959	5.243916	6.342338	5.522054
AT5G53970	448.0745	-1.24756	0.16803	-7.42464	1.13E-13	3.37E-12	8.277878	7.866861	7.889068	9.346112	9.453039	9.134113
AT2G18260	48.62537	-1.24509	0.2836	-4.3903	1.13E-05	0.00013	4.831149	4.712023	4.997248	6.476879	5.90133	5.961117
AT5G20630	531.2267	-1.24338	0.279011	-4.4564	8.33E-06	9.75E-05	8.943312	7.719346	7.638356	9.726546	9.419665	9.568058
AT1G12240	2261.786	-1.24216	0.15805	-7.85931	3.86E-15	1.30E-13	10.2774	10.40918	10.40759	11.48825	11.96674	11.42104
AT2G31810	2623.564	-1.24209	0.164147	-7.56693	3.82E-14	1.18E-12	10.81191	10.35633	10.54007	11.93844	12.02523	11.57776
AT5G02160	7399.739	-1.24135	0.146742	-8.45938	2.69E-17	1.07E-15	12.0488	11.90212	12.27641	13.43366	13.47905	13.119

AT3G13790	187.2285	-1.23904	0.260008	-4.76541	1.88E-06	2.47E-05	6.751587	6.876128	6.595172	7.336904	8.150563	8.4984
AT3G45730	702.9467	-1.23744	0.158829	-7.79102	6.65E-15	2.19E-13	8.751953	8.574255	8.737072	9.666541	10.00392	10.16142
AT2G17880	614.7029	-1.23692	0.191204	-6.4691	9.86E-11	2.22E-09	8.450229	8.112663	8.795908	9.724518	9.988833	9.571936
AT1G08513	42.71016	-1.23553	0.296584	-4.16585	3.10E-05	0.000325	4.907866	4.296832	4.580013	5.831289	6.13862	5.937262
AT1G29395	1341.614	-1.2348	0.172964	-7.13905	9.40E-13	2.57E-11	9.793885	9.313522	9.703019	11.06399	10.96902	10.60072
AT1G22490	27.27238	-1.2323	0.366846	-3.35918	0.000782	0.00581	3.57078	3.766043	4.273086	5.454347	5.766238	4.730144
AT1G05835	177.9929	-1.22917	0.17882	-6.87381	6.25E-12	1.59E-10	6.877737	6.684352	6.645579	8.144986	7.78168	7.967393
AT1G02400	109.443	-1.2289	0.216666	-5.67189	1.41E-08	2.52E-07	6.180193	5.894484	5.961278	7.507017	7.234523	7.085933
AT5G01740	276.894	-1.22856	0.215375	-5.7043	1.17E-08	2.11E-07	7.779316	7.067237	7.052865	8.726222	8.571102	8.558274
AT2G19810	1084.82	-1.22716	0.160845	-7.62947	2.36E-14	7.49E-13	9.531344	9.099486	9.305966	10.76963	10.57332	10.37071
AT1G21120	44.45263	-1.22652	0.29711	-4.12817	3.66E-05	0.000377	4.804643	4.655421	4.832959	5.919035	5.450922	6.439912
AT2G28840	1124.599	-1.22541	0.16631	-7.36825	1.73E-13	5.05E-12	9.422246	9.290475	9.410478	10.9296	10.5834	10.31741
AT1G75030	20.03214	-1.22372	0.374496	-3.26765	0.001084	0.007708	3.368482	3.596007	3.499911	4.885743	5.262981	4.614569
AT2G15970	635.4462	-1.21853	0.170241	-7.15765	8.21E-13	2.26E-11	8.755417	8.223893	8.628478	9.901902	9.878522	9.632612
AT5G63160	1762.24	-1.21436	0.20362	-5.96385	2.46E-09	4.89E-08	10.46734	9.792571	9.646386	11.29269	11.4321	11.10568
AT1G12080	390.6359	-1.21053	0.193305	-6.2623	3.79E-10	8.12E-09	7.949268	7.848043	7.691305	9.13023	9.442	8.678262
AT4G14548	36.39277	-1.20982	0.338851	-3.57037	0.000356	0.002898	4.72207	3.711561	4.680135	5.890376	5.450922	5.811684
AT4G09035	137.0028	-1.20773	0.288793	-4.18199	2.89E-05	0.000305	6.116967	6.056257	6.653811	6.818564	7.78168	8.013753
AT5G49360	11808.18	-1.20721	0.147267	-8.1974	2.46E-16	9.07E-15	13.01326	12.63158	12.67166	13.92144	14.20839	13.89854
AT2G21130	41.17209	-1.20429	0.29657	-4.06074	4.89E-05	0.000493	4.77764	4.437751	4.545032	6.05437	6.024848	5.615206
AT1G09327	26.74917	-1.20399	0.331614	-3.6307	0.000283	0.002362	4.092952	4.011295	3.990643	5.373819	5.046837	5.42247
AT1G11260	13241.99	-1.20276	0.161266	-7.45825	8.77E-14	2.63E-12	13.19732	12.63695	12.95446	14.1789	14.34244	14.00476
AT3G23730	295.438	-1.20271	0.17662	-6.80957	9.79E-12	2.46E-10	7.48597	7.292738	7.646629	8.937078	8.466036	8.638132
AT1G03470	77.50714	-1.20146	0.248313	-4.83849	1.31E-06	1.77E-05	5.635541	5.404077	5.495591	7.017205	6.88921	6.422834
AT4G37608	73.91587	-1.2003	0.242068	-4.95851	7.10E-07	1.00E-05	5.694477	5.27852	5.320494	6.588138	6.88921	6.703006
AT5G23210	490.8758	-1.19771	0.157266	-7.61584	2.62E-14	8.28E-13	8.277878	7.969098	8.319931	9.501056	9.528036	9.247575
AT3G03470	495.7232	-1.18949	0.175942	-6.76069	1.37E-11	3.41E-10	8.206275	8.201828	8.206983	9.031972	9.686095	9.546538
AT2G40420	506.0542	-1.18881	0.156292	-7.60633	2.82E-14	8.89E-13	8.404627	8.039905	8.256476	9.433134	9.657748	9.311602
AT2G41105	2195.397	-1.18836	0.195019	-6.09358	1.10E-09	2.27E-08	10.46046	10.14774	10.44365	11.19793	11.46246	11.99124
AT5G45830	124.2021	-1.18783	0.208429	-5.69899	1.21E-08	2.18E-07	6.381922	6.067147	6.17083	7.589563	7.554534	7.210579
AT1G04433	136.3847	-1.18485	0.188913	-6.27195	3.57E-10	7.64E-09	6.502267	6.259814	6.403437	7.676061	7.380437	7.652777
AT1G11820	482.5786	-1.184	0.161667	-7.32369	2.41E-13	6.96E-12	8.409033	8.034366	8.06296	9.443035	9.528036	9.223121
AT2G15830	58.27619	-1.18038	0.283353	-4.16576	3.10E-05	0.000325	5.240069	5.120221	5.144727	6.863493	5.90133	6.159727

AT3G29575	349.7302	-1.17829	0.198046	-5.94957	2.69E-09	5.28E-08	7.605954	7.95451	7.476057	8.762258	9.302486	8.706763
AT3G60530	1044.809	-1.17812	0.182089	-6.47002	9.80E-11	2.21E-09	9.336956	8.983564	9.509062	10.61181	10.70382	10.1806
AT3G51000	821.453	-1.17763	0.134152	-8.77833	1.66E-18	7.09E-17	9.087712	8.902284	8.873739	10.20073	10.2767	10.00239
AT3G10300	389.342	-1.17276	0.181536	-6.46022	1.05E-10	2.35E-09	7.915604	7.684411	7.983539	8.726222	9.326696	9.170377
AT5G00820	127.983	-1.17213	0.217367	-5.3924	6.95E-08	1.14E-06	6.47731	6.034228	6.204845	7.562569	7.709866	7.200593
AT1G70300	213.0747	-1.16906	0.193808	-6.03202	1.62E-09	3.28E-08	7.265613	6.882307	6.809303	8.289179	8.376102	7.996542
AT3G01420	128.4567	-1.16856	0.338466	-3.45252	0.000555	0.004299	6.34561	6.589348	5.601531	6.963185	6.822676	8.288168
AT3G04000	89.31324	-1.16696	0.262716	-4.4419	8.92E-06	0.000104	5.792154	5.566378	5.70022	6.377123	7.47011	6.98483
AT1G15670	502.5505	-1.16553	0.152044	-7.66576	1.78E-14	5.68E-13	8.448089	8.107398	8.181585	9.305986	9.579315	9.467548
AT3G05880	1754.295	-1.16296	0.178748	-6.50612	7.71E-11	1.77E-09	9.719571	10.00214	10.38119	11.38353	11.03663	11.32031
AT1G28050	88.4347	-1.16292	0.238774	-4.87037	1.11E-06	1.53E-05	5.650502	6.077955	5.549533	7.017205	6.679627	7.128689
AT4G04620	234.3406	-1.16258	0.157755	-7.36956	1.71E-13	5.01E-12	7.068052	7.13608	7.277261	8.278159	8.399116	8.383934
AT5G57910	131.2923	-1.16049	0.221817	-5.23173	1.68E-07	2.61E-06	6.317763	6.412782	6.075995	7.525773	7.883112	7.139183
AT5G00365	443.7771	-1.15868	0.153842	-7.53159	5.01E-14	1.54E-12	8.273052	7.854343	8.111587	9.316796	9.240123	9.249998
AT1G28260	651.7052	-1.15626	0.144438	-8.00526	1.19E-15	4.20E-14	8.698967	8.574255	8.624295	9.882023	9.988833	9.579661
AT3G44300	927.9864	-1.15428	0.158087	-7.30155	2.84E-13	8.16E-12	9.287565	9.254008	8.861352	10.1442	10.44096	10.40045
AT2G35070	66.62283	-1.15296	0.26604	-4.33377	1.47E-05	0.000164	5.240069	5.535346	5.299659	6.935398	6.342338	6.29727
AT3G01695	17785.37	-1.15084	0.177182	-6.49526	8.29E-11	1.90E-09	13.55726	13.29788	13.34953	14.13644	14.74235	14.81065
AT3G01715	17784.35	-1.15081	0.177217	-6.49376	8.37E-11	1.91E-09	13.55719	13.29773	13.34953	14.13615	14.74235	14.8106
AT3G01970	69.77192	-1.15014	0.359374	-3.2004	0.001372	0.009429	5.179468	5.793186	4.680135	5.567291	7.234523	6.772062
AT4G21500	23.95697	-1.14942	0.352113	-3.26435	0.001097	0.007784	3.906114	3.596007	3.937685	4.997462	5.450922	4.984091
AT3G05675	17838.26	-1.145	0.176662	-6.48129	9.09E-11	2.06E-09	13.56474	13.30839	13.35766	14.1411	14.74404	14.81224
AT5G49520	169.5406	-1.14491	0.284375	-4.02605	5.67E-05	0.000565	6.526799	6.726176	6.67823	6.9213	8.229725	8.235348
AT3G05700	17856.32	-1.14372	0.17663	-6.47521	9.47E-11	2.14E-09	13.56758	13.3096	13.36065	14.14272	14.74432	14.81367
AT1G58190	78.258	-1.14361	0.306965	-3.72555	0.000195	0.001708	6.169846	5.297139	4.997248	7.003888	6.434336	6.876105
AT3G45290	144.7392	-1.14346	0.245033	-4.66655	3.06E-06	3.87E-05	6.877737	6.259814	6.06369	7.741709	7.850087	7.397017
AT5G11090	518.0018	-1.14346	0.148909	-7.67891	1.60E-14	5.15E-13	8.467228	8.083462	8.375744	9.49631	9.548767	9.421004
AT3G09390	3066.938	-1.13724	0.130917	-8.68671	3.73E-18	1.57E-16	10.88608	10.82582	10.96868	12.25846	11.90175	11.95494
AT3G46690	54.58384	-1.13716	0.335737	-3.38707	0.000706	0.005305	4.907866	5.27852	4.680135	5.331804	6.752923	6.50627
AT4G37610	739.3396	-1.13616	0.195564	-5.80965	6.26E-09	1.17E-07	9.242608	8.572346	8.546845	10.09951	10.01886	9.887273
AT5G24500	647.0658	-1.13581	0.135977	-8.35296	6.66E-17	2.59E-15	8.645829	8.698516	8.647155	9.965108	9.569204	9.833362
AT1G21130	1906.971	-1.135	0.162422	-6.98798	2.79E-12	7.39E-11	10.4647	9.972035	10.12035	11.51959	11.39823	11.15628
AT3G18830	499.7784	-1.13283	0.137059	-8.26532	1.39E-16	5.29E-15	8.231369	8.199356	8.380712	9.472338	9.528036	9.295273

AT2G32150	2123.81	-1.13242	0.135167	-8.37793	5.39E-17	2.11E-15	10.35538	10.21905	10.50921	11.68307	11.47063	11.37904
AT1G04467	54.23125	-1.1322	0.28289	-4.00226	6.27E-05	0.000619	5.004194	5.077776	5.167905	6.674719	6.024848	5.961117
AT4G09020	241.2396	-1.13161	0.205541	-5.50553	3.68E-08	6.24E-07	6.871365	7.351821	7.399794	8.664088	8.229725	8.2206
AT3G15500	241.3944	-1.12967	0.283098	-3.99039	6.60E-05	0.000647	7.570995	7.221417	6.507039	7.733664	8.795846	8.518635
AT1G21100	215.0654	-1.12949	0.183542	-6.15386	7.56E-10	1.58E-08	7.270463	6.819293	7.008616	8.215999	8.376102	8.080614
AT5G53730	30.19685	-1.12742	0.323285	-3.48739	0.000488	0.003831	4.092952	4.181303	4.315217	5.530617	5.617177	5.199956
AT5G08150	108.688	-1.12577	0.247046	-4.55693	5.19E-06	6.30E-05	6.270123	5.535346	6.216008	7.030399	7.47011	7.249844
AT5G02140	50.32132	-1.12554	0.30482	-3.69245	0.000222	0.001917	5.354131	4.712023	4.712022	6.496028	6.024848	5.888334
AT2G01450	1556.701	-1.12248	0.16993	-6.60554	3.96E-11	9.44E-10	10.12104	9.641386	9.931809	11.1152	11.27301	10.78491
AT3G56210	241.1736	-1.12226	0.168168	-6.67343	2.50E-11	6.06E-10	7.408878	7.211635	7.059077	8.474367	8.376102	8.292876
AT5G66650	54.55634	-1.12062	0.269915	-4.15176	3.30E-05	0.000343	4.907866	5.056075	5.213173	5.947137	6.434336	6.405552
AT4G37580	63.64077	-1.12021	0.262657	-4.26492	2.00E-05	0.000218	5.47672	5.315522	5.213173	6.848671	6.13862	6.334274
AT1G03620	28.18347	-1.11885	0.325615	-3.43611	0.00059	0.004547	4.136111	4.259341	4.041726	5.414645	5.262981	5.352044
AT1G01420	93.56765	-1.11695	0.251751	-4.43672	9.13E-06	0.000106	6.169846	5.641159	5.635183	7.203473	7.182411	6.688789
AT2G45170	1158.835	-1.11694	0.117552	-9.50164	2.07E-21	1.06E-19	9.583923	9.398967	9.5427	10.71245	10.5478	10.64004
AT1G05340	126.7863	-1.11668	0.273979	-4.07579	4.59E-05	0.000464	6.542925	6.201705	6.112292	6.976881	7.182411	8.019445
AT4G29190	1763.491	-1.11626	0.169026	-6.60405	4.00E-11	9.53E-10	10.31955	9.856737	10.08273	11.42908	11.322	10.95122
AT2G04690	817.4133	-1.11622	0.140286	-7.95671	1.77E-15	6.12E-14	9.055757	8.849646	9.071058	10.24385	10.20668	9.943738
AT4G08305	58.8441	-1.11399	0.299344	-3.72144	0.000198	0.001733	4.664273	5.161454	5.651719	6.417857	6.024848	6.600414
AT2G39570	2969.218	-1.11346	0.174734	-6.37232	1.86E-10	4.08E-09	11.14964	10.5695	10.7681	12.00846	12.19922	11.75794
AT5G57240	224.5737	-1.11118	0.201153	-5.52408	3.31E-08	5.65E-07	6.987859	7.13608	7.29842	8.625482	8.037767	8.080614
AT3G01475	312.3423	-1.11073	0.159807	-6.95045	3.64E-12	9.52E-11	7.559151	7.535541	7.703253	8.824179	8.896321	8.522648
AT4G36648	1206.543	-1.10948	0.147306	-7.53184	5.00E-14	1.54E-12	9.701726	9.443525	9.5427	10.52591	10.60831	10.91808
AT4G23870	656.8974	-1.10813	0.191632	-5.78259	7.36E-09	1.36E-07	8.938749	8.412889	8.640956	10.13201	9.704689	9.569998
AT1G25400	471.1167	-1.10645	0.246721	-4.4846	7.30E-06	8.67E-05	8.183311	8.365155	7.953843	8.621127	9.419665	9.790733
AT2G22880	38.92423	-1.10571	0.328041	-3.37064	0.00075	0.005591	5.072441	4.296832	4.434696	5.800813	5.262981	6.138962
AT1G52140	39.95451	-1.10466	0.31603	-3.49541	0.000473	0.003732	4.693461	4.296832	4.580013	5.454347	6.434336	5.522054
AT1G06180	47.32289	-1.1046	0.284726	-3.87952	0.000105	0.000983	4.980709	4.965854	4.545032	6.05437	6.342338	5.811684
AT1G21110	104.8546	-1.10458	0.22214	-4.97246	6.61E-07	9.35E-06	6.279777	5.739739	6.051279	7.27173	7.182411	7.10747
AT3G60930	101.5165	-1.1035	0.259591	-4.25091	2.13E-05	0.00023	6.02811	6.231052	5.618455	6.740426	7.128345	7.481917
AT1G16510	33.5738	-1.10115	0.341387	-3.22552	0.001257	0.008745	4.693461	4.098802	4.184943	5.861135	5.766238	5.074348
AT1G50040	361.6422	-1.10084	0.162972	-6.75478	1.43E-11	3.55E-10	7.884303	7.574224	7.973708	8.839252	9.106676	8.931683
AT2G25930	428.8664	-1.09971	0.152795	-7.1973	6.14E-13	1.71E-11	8.178158	8.003519	8.066048	9.400478	9.134371	9.042048

AT3G26510	1420.503	-1.09943	0.184548	-5.95743	2.56E-09	5.07E-08	9.928865	9.614728	9.803563	10.83876	11.30382	10.56711
AT5G09565	193.6213	-1.09894	0.24398	-4.50423	6.66E-06	7.96E-05	7.289704	6.250291	7.008616	8.070295	8.037767	8.102229
AT1G12730	98.48469	-1.0956	0.220017	-4.9796	6.37E-07	9.03E-06	6.200668	5.780009	5.822109	7.030399	7.182411	7.085933
AT4G37520	397.6243	-1.09397	0.206622	-5.29457	1.19E-07	1.89E-06	8.206275	7.876179	7.715103	8.647057	9.350505	9.223121
AT5G55970	318.4517	-1.09309	0.162137	-6.74174	1.57E-11	3.87E-10	7.669763	7.558875	7.719032	8.937078	8.813085	8.546497
AT1G04147	30.58546	-1.08907	0.324414	-3.35704	0.000788	0.005851	4.334434	4.333373	4.229687	5.243916	5.262981	5.758224
AT5G28145	43.23579	-1.08786	0.341276	-3.18763	0.001434	0.009785	5.298227	4.140642	4.395959	5.737858	6.244072	5.863234
AT5G44572	85.1322	-1.08624	0.234513	-4.63191	3.62E-06	4.52E-05	6.005013	5.655661	5.549533	7.017205	6.88921	6.744835
AT4G36900	45.25174	-1.08448	0.337301	-3.21518	0.001304	0.009024	4.441535	5.386795	4.273086	6.129868	6.13862	5.758224
AT2G01300	29.10334	-1.0836	0.336057	-3.22446	0.001262	0.008775	4.296891	3.869198	4.356153	5.567291	5.450922	5.117444
AT4G24040	105.3466	-1.08351	0.200361	-5.4078	6.38E-08	1.05E-06	6.148927	6.109902	5.961278	7.226586	7.128345	7.190538
AT2G47270	53.83038	-1.08115	0.265349	-4.07445	4.61E-05	0.000467	5.004194	5.201541	5.022899	6.377123	6.342338	5.984584
AT1G21910	488.18	-1.08075	0.162518	-6.65004	2.93E-11	7.06E-10	8.34839	7.966193	8.462658	9.377429	9.442	9.334613
AT3G56880	310.6329	-1.08032	0.208392	-5.18407	2.17E-07	3.31E-06	7.792865	7.181886	7.806553	8.554188	8.630654	8.985015
AT1G10140	291.7914	-1.07831	0.173941	-6.19928	5.67E-10	1.19E-08	7.523025	7.634036	7.447929	8.379596	8.649971	8.860536
AT1G75750	4888.736	-1.07677	0.165082	-6.52261	6.91E-11	1.59E-09	11.8732	11.25908	11.59755	12.68468	12.8482	12.56979
AT2G28110	59.44016	-1.07484	0.267998	-4.01062	6.06E-05	0.0006	5.279101	5.386795	5.048102	6.079977	6.342338	6.645278
AT1G67070	60.76808	-1.07309	0.261572	-4.10246	4.09E-05	0.000418	5.220151	5.297139	5.190717	6.001749	6.679627	6.50627
AT2G46600	1441.75	-1.07282	0.158416	-6.77216	1.27E-11	3.17E-10	10.02958	9.560851	9.902744	11.12061	10.88999	10.79324
AT3G49790	748.9979	-1.07066	0.175035	-6.11685	9.54E-10	1.97E-08	9.228967	8.686208	8.701774	10.08536	10.04098	9.863737
AT1G02660	1097.94	-1.06971	0.133219	-8.02968	9.77E-16	3.45E-14	9.527297	9.279982	9.532691	10.48097	10.6992	10.45292
AT4G36410	52.6389	-1.06516	0.272404	-3.9102	9.22E-05	0.000881	5.072441	5.240544	4.917441	5.974702	6.13862	6.439912
AT2G17230	401.0759	-1.06433	0.184201	-5.7781	7.56E-09	1.40E-07	7.918697	7.691466	8.30171	8.951022	9.240123	9.099602
AT1G10090	373.4867	-1.06414	0.154236	-6.89944	5.22E-12	1.34E-10	8.062666	7.816122	7.791708	9.035249	9.106676	8.834943
AT3G43670	235.0794	-1.0637	0.159397	-6.6733	2.50E-11	6.06E-10	7.128075	7.216535	7.355055	8.233219	8.376102	8.370636
AT3G54880	243.8233	-1.06114	0.178363	-5.9493	2.69E-09	5.29E-08	7.456477	7.264631	7.059077	8.192714	8.591227	8.357215
AT1G74550	41.26285	-1.06045	0.282053	-3.75977	0.00017	0.001512	4.77764	4.684	4.712022	5.890376	5.90133	5.785202
AT1G69870	326.1515	-1.05466	0.161241	-6.5409	6.12E-11	1.42E-09	7.510779	7.729664	7.867993	8.865258	8.8636	8.652855
AT4G20860	204.5455	-1.05305	0.245789	-4.28435	1.83E-05	0.000201	6.958103	7.226283	6.823968	7.488014	8.280186	8.473736
AT5G64310	322.573	-1.04985	0.281118	-3.73455	0.000188	0.001654	7.598258	7.333898	7.986802	8.037996	8.649971	9.38398
AT1G62420	52.93995	-1.04829	0.296315	-3.53778	0.000404	0.003236	5.47672	5.099155	4.580013	6.397634	6.024848	6.159727
AT2G26560	564.9833	-1.04812	0.284584	-3.683	0.000231	0.001982	8.543347	8.583766	8.242898	8.590275	9.609229	10.19199
AT3G26910	128.6469	-1.0463	0.187408	-5.58298	2.36E-08	4.11E-07	6.317763	6.43817	6.422836	7.409395	7.380437	7.562097

AT1G74790	237.9545	-1.04529	0.15728	-6.64604	3.01E-11	7.25E-10	7.336712	7.231133	7.211858	8.38987	8.42177	8.2057
AT3G57700	53.87288	-1.04471	0.308231	-3.38936	0.000701	0.005265	4.882744	5.240544	5.097223	5.530617	6.434336	6.569711
AT2G15090	1310.209	-1.04285	0.142902	-7.29765	2.93E-13	8.37E-12	9.871758	9.599807	9.715854	11.00003	10.65686	10.68907
AT1G34370	1087.192	-1.04072	0.11296	-9.21314	3.17E-20	1.51E-18	9.503804	9.402196	9.476944	10.50361	10.61815	10.44131
AT1G75800	1894.365	-1.03614	0.164041	-6.31634	2.68E-10	5.83E-09	10.34794	10.00639	10.39353	11.49897	11.40393	11.03016
AT3G05360	73.07451	-1.03358	0.287864	-3.59053	0.00033	0.002709	5.73715	5.550946	5.190717	6.001749	7.013727	6.837962
AT1G09430	1690.094	-1.03352	0.136661	-7.56262	3.95E-14	1.22E-12	10.20767	9.970585	10.10842	11.16465	11.33101	10.95493
AT4G34970	92.97319	-1.03163	0.223268	-4.62059	3.83E-06	4.77E-05	6.148927	5.684235	5.907211	6.963185	6.952811	7.053013
AT3G47800	708.7907	-1.02959	0.131266	-7.84355	4.38E-15	1.46E-13	8.964419	8.746726	8.877259	9.983881	9.741171	9.95269
AT1G58270	790.2101	-1.0295	0.147428	-6.98305	2.89E-12	7.63E-11	9.076677	8.977805	8.965885	10.25091	10.08422	9.812205
AT4G31550	284.9526	-1.02438	0.21706	-4.71934	2.37E-06	3.05E-05	7.452214	7.269354	7.810241	8.19857	8.611075	8.898094
AT3G47860	180.4656	-1.02273	0.200046	-5.11249	3.18E-07	4.73E-06	6.964104	6.746642	6.929452	8.215999	7.883112	7.652777
AT2G16365	280.1268	-1.02139	0.182542	-5.59539	2.20E-08	3.84E-07	7.559151	7.531616	7.438429	8.805112	8.611075	8.235348
AT5G57110	278.1378	-1.01959	0.15664	-6.50909	7.56E-11	1.74E-09	7.506673	7.663037	7.349997	8.590275	8.550692	8.518635
AT3G47160	445.8916	-1.01939	0.154292	-6.6069	3.92E-11	9.36E-10	8.122909	8.22875	8.25106	9.467495	9.06411	9.115632
AT5G55250	69.82485	-1.01884	0.272135	-3.74387	0.000181	0.0016	5.158689	5.421153	5.684234	6.457472	7.072174	6.180197
AT2G31010	395.6266	-1.01831	0.162909	-6.25077	4.08E-10	8.71E-09	8.133597	7.844882	8.031715	9.163548	9.188216	8.815446
AT2G23080	411.0467	-1.01499	0.156302	-6.4938	8.37E-11	1.91E-09	8.263352	7.992137	8.009433	9.190246	8.8636	9.2354
AT5G67310	138.9806	-1.01489	0.238876	-4.24859	2.15E-05	0.000232	6.390859	6.38694	6.67823	7.043474	7.672573	7.869951
AT5G44130	416.9219	-1.01472	0.199443	-5.08777	3.62E-07	5.33E-06	8.079347	7.670197	8.388133	9.086682	9.362265	8.95563
AT5G20230	905.9437	-1.01454	0.260847	-3.88939	0.0001	0.00095	9.365572	9.20555	8.932439	9.367065	10.4073	10.71824
AT1G32170	156.7054	-1.01388	0.182938	-5.54218	2.99E-08	5.12E-07	6.709344	6.604377	6.67823	7.765576	7.915398	7.514525
AT4G11280	459.8421	-1.01349	0.259508	-3.90544	9.41E-05	0.000897	8.339185	8.204297	8.084436	8.634151	9.02025	9.904292
AT1G53450	355.8356	-1.01173	0.178056	-5.68208	1.33E-08	2.39E-07	7.912504	7.903778	7.765357	9.142435	8.959616	8.569957
AT3G19690	39.60092	-1.01104	0.311234	-3.24848	0.00116	0.008174	5.094491	4.535085	4.545032	5.861135	5.262981	6.052778
AT1G29760	369.1844	-1.00827	0.145404	-6.93428	4.08E-12	1.06E-10	7.993944	7.841715	7.89255	8.901615	9.148022	8.825227
AT3G58750	683.7978	-1.008	0.134579	-7.49008	6.88E-14	2.08E-12	8.849183	8.69501	8.892993	9.935605	9.894806	9.690838
AT5G57887	164.281	-1.00683	0.212268	-4.74322	2.10E-06	2.73E-05	7.079152	6.404219	6.718032	7.879329	7.709866	7.792291
AT4G32480	966.7929	-1.00672	0.159972	-6.29311	3.11E-10	6.72E-09	9.55936	9.188197	9.150294	10.49054	10.37284	10.14979
AT1G46768	74.30338	-1.00626	0.241745	-4.16249	3.15E-05	0.000329	5.694477	5.43803	5.792589	6.907064	6.342338	6.645278
AT4G26530	1582.401	-1.00612	0.17449	-5.76608	8.11E-09	1.49E-07	9.782895	9.918923	10.3107	11.30088	10.93005	10.89737
AT3G28290	309.826	-1.00391	0.227672	-4.40944	1.04E-05	0.00012	7.265613	7.832171	7.817587	9.083521	8.630654	8.316185
AT5G49015	38.71137	-1.00321	0.306831	-3.26958	0.001077	0.007669	4.750123	4.369011	4.944537	6.001749	5.450922	5.702706

AT3G28300	309.9215	-1.00255	0.227803	-4.40093	1.08E-05	0.000124	7.265613	7.832171	7.821247	9.083521	8.630654	8.316185
AT1G31812	4549.448	-1.00151	0.135955	-7.36643	1.75E-13	5.12E-12	11.5688	11.39331	11.68249	12.68989	12.6466	12.36319
AT2G47890	114.8501	-1.00064	0.194042	-5.1568	2.51E-07	3.79E-06	6.148927	6.306513	6.312764	7.27173	7.234523	7.325306
AT3G50650	113.4539	-0.99817	0.211965	-4.70911	2.49E-06	3.18E-05	6.270123	6.045285	6.216008	7.094621	7.63429	7.06407
AT1G35140	1436.371	-0.99592	0.183648	-5.42298	5.86E-08	9.72E-07	9.883678	9.627659	10.09714	10.51774	11.07286	11.08547
AT1G55110	193.9613	-0.99485	0.197307	-5.04214	4.60E-07	6.67E-06	7.1857	6.900687	6.809303	8.037996	8.304769	7.738093
AT1G48210	186.0585	-0.99368	0.189124	-5.25414	1.49E-07	2.33E-06	7.144021	6.677262	6.962967	7.842399	8.037767	8.030761
AT3G62650	1904.496	-0.99308	0.140781	-7.05405	1.74E-12	4.67E-11	10.32655	10.12317	10.43949	11.45561	11.34885	11.12164
AT3G19990	299.6646	-0.98887	0.165877	-5.96147	2.50E-09	4.96E-08	7.636334	7.670197	7.570401	8.781895	8.778398	8.375082
AT3G48690	987.7423	-0.98763	0.14563	-6.78177	1.19E-11	2.97E-10	9.469414	9.297428	9.278278	10.39432	10.56317	10.12229
AT2G33810	706.7124	-0.98428	0.168588	-5.83839	5.27E-09	9.96E-08	9.041639	8.628563	8.930747	10.11662	9.820033	9.681894
AT1G13990	356.5717	-0.9827	0.172039	-5.71206	1.12E-08	2.02E-07	7.937117	7.857483	7.828538	8.549613	9.12059	8.993715
AT2G33815	705.3715	-0.98265	0.169152	-5.80928	6.27E-09	1.17E-07	9.041639	8.623039	8.930747	10.11508	9.81148	9.681894
AT2G30930	1009.769	-0.98073	0.176346	-5.5614	2.68E-08	4.64E-07	9.629942	9.034389	9.422574	10.27051	10.62304	10.28706
AT2G35230	191.1002	-0.98052	0.205959	-4.76073	1.93E-06	2.52E-05	7.068052	6.894586	6.859993	7.849861	8.444073	7.674584
AT4G01870	873.6031	-0.98001	0.167929	-5.83587	5.35E-09	1.01E-07	9.035954	9.365173	9.102976	9.869229	10.41297	10.24639
AT1G78600	222.6937	-0.97995	0.18777	-5.21887	1.80E-07	2.77E-06	6.958103	7.425602	7.160782	8.215999	8.42177	8.036386
AT1G76070	138.7717	-0.97958	0.238791	-4.10223	4.09E-05	0.000418	6.558872	6.297293	6.733651	7.226586	7.33342	7.955567
AT5G42200	85.52993	-0.97883	0.251358	-3.89417	9.85E-05	0.000933	6.050842	5.386795	6.013393	7.017205	6.679627	6.837962
AT4G29950	1504.671	-0.97831	0.109594	-8.92667	4.39E-19	1.93E-17	10.08087	9.929404	9.915639	10.93487	10.99569	10.97338
AT5G58090	578.1819	-0.97736	0.155098	-6.30157	2.95E-10	6.38E-09	8.767477	8.485717	8.464999	9.48916	9.820033	9.461289
AT5G24800	328.508	-0.97579	0.14079	-6.93085	4.18E-12	1.08E-10	7.709596	7.83854	7.780474	8.79355	8.846958	8.696141
AT2G41100	6279.368	-0.97527	0.187022	-5.21471	1.84E-07	2.83E-06	12.16145	11.80605	12.08649	12.69222	12.8955	13.41647
AT1G50420	175.7206	-0.97503	0.19655	-4.96074	7.02E-07	9.91E-06	6.999592	6.726176	6.845691	8.044514	8.008134	7.569875
AT1G01300	170.6104	-0.97423	0.206042	-4.72833	2.26E-06	2.93E-05	6.737643	6.73303	6.915825	7.901041	8.150563	7.431579
AT3G25780	60.67397	-0.97161	0.302701	-3.20981	0.001328	0.009158	5.40795	5.315522	5.381252	5.947137	5.90133	6.961367
AT3G63010	65.65435	-0.97157	0.239562	-4.0556	5.00E-05	0.000504	5.605144	5.351599	5.477153	6.551997	6.342338	6.50627
AT3G50500	1386.164	-0.9691	0.122226	-7.92874	2.21E-15	7.62E-14	9.945642	9.75434	9.892345	10.9721	10.80207	10.75538
AT4G17245	176.9404	-0.96755	0.269522	-3.58987	0.000331	0.002714	7.231194	6.221336	6.881184	7.804499	8.328941	7.490138
AT2G45720	133.9507	-0.96471	0.207783	-4.64286	3.44E-06	4.31E-05	6.597987	6.297293	6.441977	7.44924	7.816289	7.200593
AT3G60690	125.7442	-0.96362	0.198981	-4.84277	1.28E-06	1.73E-05	6.36388	6.429757	6.507039	7.347485	7.128345	7.630636
AT4G36040	3133.902	-0.96279	0.157326	-6.11974	9.37E-10	1.94E-08	11.20041	10.72628	11.13852	12.02339	12.18605	11.84746
AT5G10450	5723.988	-0.961	0.131629	-7.30082	2.86E-13	8.19E-12	12.02281	11.71681	11.97962	12.96621	12.96656	12.72654

AT4G23170	75.52081	-0.95995	0.280479	-3.42253	0.00062	0.004752	5.832072	5.503631	5.567075	6.05437	6.822676	7.019324
AT3G01470	1001.245	-0.95814	0.164749	-5.81573	6.04E-09	1.13E-07	9.328841	9.190689	9.6277	10.55016	10.41861	10.13807
AT2G02710	1624.064	-0.95462	0.151522	-6.30018	2.97E-10	6.43E-09	10.30367	9.986447	9.969398	11.18471	11.17307	10.83983
AT4G21560	283.904	-0.9544	0.146335	-6.52202	6.94E-11	1.60E-09	7.632571	7.535541	7.5529	8.526517	8.687846	8.477876
AT2G35743	1095.364	-0.95375	0.165729	-5.7549	8.67E-09	1.59E-07	9.661672	9.446656	9.476944	10.26772	10.37284	10.81304
AT5G06370	450.4645	-0.95355	0.16709	-5.70677	1.15E-08	2.08E-07	8.329921	8.031589	8.322515	9.270283	9.430875	8.958595
AT1G06573	604.3589	-0.95353	0.167436	-5.69489	1.23E-08	2.23E-07	8.8637	8.342924	8.733192	9.559112	9.81148	9.575804
AT3G61460	1046.771	-0.95329	0.206655	-4.61293	3.97E-06	4.92E-05	9.642156	9.098159	9.549334	10.67723	10.60336	9.961586
AT5G63620	471.436	-0.95279	0.128887	-7.39248	1.44E-13	4.26E-12	8.364359	8.236006	8.337925	9.242206	9.373929	9.254831
AT1G77000	87.10934	-0.95219	0.217472	-4.37845	1.20E-05	0.000136	5.9456	5.76671	5.851038	6.833696	7.072174	6.731026
AT2G01170	148.661	-0.95143	0.213143	-4.46384	8.05E-06	9.46E-05	6.502267	6.395605	6.908963	7.439382	7.915398	7.554276
AT5G23575	360.866	-0.9508	0.136273	-6.97715	3.01E-12	7.94E-11	7.961317	7.90984	7.926923	8.954487	8.944051	8.812171
AT3G50770	191.0644	-0.94783	0.253482	-3.73922	0.000185	0.001626	6.590249	7.20672	7.143347	7.553458	7.850087	8.444419
AT5G18170	2032.046	-0.94638	0.155301	-6.09385	1.10E-09	2.27E-08	10.62241	10.23543	10.37808	11.34841	11.62014	11.18632
AT5G61600	332.9073	-0.94621	0.155448	-6.08699	1.15E-09	2.36E-08	7.782716	7.648609	8.012637	8.797415	8.778398	8.782353
AT4G33490	341.1433	-0.94501	0.175188	-5.39428	6.88E-08	1.13E-06	7.955305	7.701985	7.89255	9.064405	8.795846	8.566073
AT2G13610	278.5581	-0.94441	0.154362	-6.11813	9.47E-10	1.96E-08	7.720271	7.442428	7.535184	8.563295	8.48767	8.530642
AT5G11520	665.4919	-0.94322	0.145265	-6.49309	8.41E-11	1.92E-09	8.772614	8.832197	8.807023	9.608337	10.05554	9.667467
AT3G42052	87.47291	-0.94236	0.22803	-4.13262	3.59E-05	0.00037	5.792154	5.819185	6.03876	6.623396	6.952811	7.030642
AT1G22500	221.9873	-0.94221	0.21975	-4.28767	1.81E-05	0.000198	7.430709	6.912811	7.266564	7.781272	8.328941	8.448643
AT4G38400	179.2689	-0.94122	0.236919	-3.97275	7.10E-05	0.000693	6.839079	6.519708	7.222966	7.936518	8.229725	7.498313
AT5G64430	596.642	-0.93972	0.162142	-5.79566	6.81E-09	1.27E-07	8.541342	8.560835	8.821712	9.48916	9.910909	9.455002
AT5G18540	64.48816	-0.93952	0.257397	-3.6501	0.000262	0.002215	5.558315	5.333673	5.320494	6.496028	6.752923	6.074811
AT5G54930	51.36611	-0.93653	0.268747	-3.48481	0.000492	0.003864	4.932558	5.315522	5.072871	5.974702	6.244072	6.159727
AT5G65390	105.7073	-0.9338	0.253768	-3.67975	0.000233	0.002	5.896243	6.011857	6.353768	6.724278	7.672573	6.961367
AT3G01035	93.44977	-0.93374	0.215393	-4.33507	1.46E-05	0.000163	5.870916	6.045285	6.026132	7.056432	7.072174	6.772062
AT5G44340	4273.899	-0.9315	0.135022	-6.89883	5.24E-12	1.34E-10	11.5742	11.34128	11.60606	12.6418	12.40082	12.31338
AT1G14890	193.0079	-0.9313	0.186925	-4.9822	6.29E-07	8.93E-06	7.216189	6.906762	6.942951	8.108124	8.12318	7.765446
AT3G23020	65.55254	-0.93073	0.248785	-3.74111	0.000183	0.001615	5.354131	5.550946	5.381252	6.377123	6.822676	6.200381
AT5G42050	2262.772	-0.92981	0.143572	-6.47626	9.40E-11	2.13E-09	10.65523	10.67826	10.43233	11.29679	11.6029	11.69843
AT3G54810	922.6506	-0.92828	0.14543	-6.383	1.74E-10	3.82E-09	9.258564	9.28815	9.339527	9.965108	10.39018	10.36065
AT4G34860	100.6385	-0.92821	0.210671	-4.40596	1.05E-05	0.000121	5.96966	6.099331	6.281227	7.191776	6.952811	7.030642
AT4G37530	101.0317	-0.92751	0.268253	-3.4576	0.000545	0.004231	6.180193	6.181803	5.747144	6.4378	7.51294	7.170215

AT1G76210	148.4468	-0.92637	0.213587	-4.3372	1.44E-05	0.000162	6.493996	6.604377	6.908963	7.915337	7.42597	7.456967
AT3G10410	512.7348	-0.92602	0.144799	-6.3952	1.60E-10	3.54E-09	8.275467	8.606338	8.4859	9.49631	9.326696	9.366223
AT3G13270	59.1939	-0.92489	0.255353	-3.62198	0.000292	0.002432	5.40795	5.315522	5.167905	6.292034	6.602406	6.074811
AT4G35750	2073.402	-0.9245	0.140831	-6.56465	5.22E-11	1.23E-09	10.58736	10.36187	10.44602	11.47504	11.57538	11.16658
AT3G20810	62.52844	-0.9237	0.256049	-3.60752	0.000309	0.002554	5.442744	5.120221	5.618455	6.269954	6.520817	6.405552
AT1G22890	643.1259	-0.92304	0.232724	-3.96625	7.30E-05	0.000711	9.29115	8.481653	8.317342	9.901902	9.759072	9.52869
AT2G40000	3819.635	-0.92255	0.152171	-6.06254	1.34E-09	2.73E-08	11.57199	11.38136	11.05746	12.17663	12.28048	12.41889
AT1G35670	299.3063	-0.9223	0.158721	-5.81081	6.22E-09	1.16E-07	7.748358	7.578035	7.642499	8.540419	8.880053	8.461244
AT4G26670	192.5631	-0.92138	0.171887	-5.36036	8.31E-08	1.35E-06	6.970079	6.954462	7.222966	8.063893	7.946977	7.955567
AT1G32460	620.8743	-0.9189	0.164537	-5.58479	2.34E-08	4.07E-07	8.901712	8.434075	8.795908	9.58617	9.87031	9.566114
AT5G46180	616.3336	-0.91788	0.120149	-7.63951	2.18E-14	6.93E-13	8.695365	8.727978	8.746725	9.636654	9.759072	9.594989
AT1G03290	478.0579	-0.91742	0.131299	-6.9873	2.80E-12	7.42E-11	8.368889	8.415021	8.288552	9.338176	9.362265	9.190698
AT3G18035	1141.224	-0.91351	0.143751	-6.35484	2.09E-10	4.56E-09	9.601352	9.497857	9.72565	10.59192	10.72675	10.30931
AT5G66400	222.5406	-0.91228	0.263561	-3.46135	0.000537	0.004178	6.518668	7.446604	7.49451	8.250236	8.529989	7.785626
AT2G38820	405.4303	-0.91212	0.160579	-5.68017	1.35E-08	2.41E-07	8.318257	8.003519	7.986802	9.002146	9.252813	8.925634
AT1G52200	155.8227	-0.91205	0.273307	-3.33708	0.000847	0.006239	6.73062	6.589348	6.874155	7.069274	7.47011	8.273953
AT4G05065	77.49686	-0.91201	0.244418	-3.73136	0.00019	0.001673	5.933418	5.471204	5.851038	6.9213	6.434336	6.645278
AT1G79700	839.8316	-0.91128	0.153071	-5.95332	2.63E-09	5.17E-08	9.212678	9.039928	9.237779	10.13964	10.32557	9.830128
AT2G07175	93.2091	-0.9111	0.227233	-4.00954	6.08E-05	0.000603	5.921133	5.753287	6.259813	6.907064	7.072174	6.900984
AT4G20930	133.6359	-0.90983	0.215621	-4.21955	2.45E-05	0.000262	6.716471	6.463119	6.432438	7.789056	7.234523	7.288068
AT5G26340	387.3603	-0.90763	0.271601	-3.34177	0.000832	0.006149	7.964314	8.156661	7.967117	8.126672	8.975015	9.573871
AT3G14050	524.1995	-0.90743	0.181476	-5.00025	5.73E-07	8.20E-06	8.649557	8.450802	8.345569	9.038518	9.579315	9.613923
AT4G34138	500.1425	-0.90731	0.151103	-6.00457	1.92E-09	3.85E-08	8.404627	8.598853	8.248344	9.390279	9.474868	9.213222
AT2G26650	175.549	-0.90586	0.208174	-4.35144	1.35E-05	0.000152	7.275298	6.719289	6.702243	7.89384	7.78168	7.88873
AT2G01190	281.3845	-0.90467	0.222744	-4.06147	4.88E-05	0.000492	7.464966	7.425602	7.757739	8.305552	9.049638	8.144512
AT3G61430	4275.11	-0.90422	0.138081	-6.54842	5.81E-11	1.35E-09	11.4163	11.45421	11.6975	12.53794	12.55735	12.23544
AT1G17460	60.04192	-0.90212	0.268576	-3.3589	0.000783	0.005813	5.335736	5.503631	5.299659	6.657813	6.244072	6.007675
AT5G54520	86.72704	-0.90126	0.243454	-3.70198	0.000214	0.001856	6.062076	5.550946	6.013393	6.990448	6.952811	6.600414
AT2G38470	678.3033	-0.90036	0.257118	-3.50173	0.000462	0.003658	8.971883	8.982127	8.555659	9.086682	9.695422	10.36513
AT4G13250	848.5347	-0.89856	0.122389	-7.34186	2.11E-13	6.11E-12	9.161374	9.112685	9.322844	10.05016	10.167	10.1302
AT3G14020	76.8782	-0.8985	0.225092	-3.9917	6.56E-05	0.000644	5.818889	5.655661	5.684234	6.640707	6.822676	6.585144
AT5G37540	45.27514	-0.89761	0.278515	-3.22285	0.001269	0.008816	5.050049	4.818971	4.971133	5.70532	6.13862	5.961117
AT5G26865	48.55199	-0.89701	0.278054	-3.22603	0.001255	0.008731	5.372295	4.965854	4.889826	6.105138	5.90133	6.052778

AT1G76090	1045.402	-0.89633	0.156437	-5.72966	1.01E-08	1.83E-07	9.389179	9.321502	9.737318	10.35553	10.60831	10.27997
AT5G04720	746.5197	-0.89608	0.112365	-7.97472	1.53E-15	5.32E-14	9.020201	9.012021	9.033695	9.954764	9.942585	9.890382
AT1G20630	723.4129	-0.89456	0.16511	-5.41795	6.03E-08	9.99E-07	8.706145	8.917407	9.21987	9.867392	10.04828	9.74165
AT1G28280	270.9137	-0.89368	0.175815	-5.08306	3.71E-07	5.46E-06	7.518954	7.360699	7.691305	8.369248	8.760736	8.288168
AT3G10020	948.299	-0.89342	0.183327	-4.87338	1.10E-06	1.51E-05	9.148258	9.704646	9.128307	10.34236	10.41297	10.06702
AT1G33050	2102.447	-0.89168	0.141898	-6.28394	3.30E-10	7.12E-09	10.67868	10.38544	10.45665	11.56223	11.47605	11.20829
AT1G23050	540.7058	-0.89144	0.20189	-4.41549	1.01E-05	0.000117	8.649557	8.358522	8.6201	9.863711	9.350505	9.083391
AT1G23052	540.7058	-0.89144	0.20189	-4.41549	1.01E-05	0.000117	8.649557	8.358522	8.6201	9.863711	9.350505	9.083391
AT1G16110	97.95876	-0.88788	0.236125	-3.76023	0.00017	0.001511	5.723066	6.161623	6.373842	7.069274	6.822676	7.118118
AT3G13445	220.3448	-0.88781	0.162351	-5.46845	4.54E-08	7.63E-07	7.336712	7.161707	7.293159	8.272618	8.177436	8.064187
AT4G24220	1812.82	-0.88757	0.13303	-6.67194	2.52E-11	6.11E-10	10.30426	10.21661	10.37684	11.29679	11.32501	10.9785
AT5G60870	117.1813	-0.88571	0.207893	-4.26041	2.04E-05	0.000222	6.408569	6.297293	6.204845	7.056432	7.63429	7.128689
AT1G77640	152.996	-0.88531	0.23266	-3.80517	0.000142	0.001284	6.744632	6.306513	7.059077	7.459032	7.594963	7.876237
AT3G05890	76.42407	-0.88525	0.25522	-3.46858	0.000523	0.004077	5.47672	5.581648	6.112292	6.640707	6.520817	6.798784
AT5G06860	260.6859	-0.88367	0.168486	-5.24476	1.56E-07	2.44E-06	7.643829	7.360699	7.452655	8.215999	8.611075	8.418806
AT3G49780	186.9629	-0.883	0.189077	-4.67007	3.01E-06	3.81E-05	7.056865	6.966144	6.969577	7.59845	8.229725	7.967393
AT1G05753	88.39023	-0.88283	0.249239	-3.54212	0.000397	0.00319	6.148927	5.655661	5.920919	6.476879	7.072174	7.030642
AT1G80190	172.1012	-0.87898	0.203132	-4.32715	1.51E-05	0.000168	7.111951	6.73303	6.794486	7.922432	8.008134	7.514525
AT3G23050	4368.894	-0.87885	0.118937	-7.38919	1.48E-13	4.36E-12	11.6394	11.47301	11.62319	12.60479	12.46221	12.33323
AT5G49440	1191.543	-0.87847	0.158376	-5.54675	2.91E-08	5.01E-07	9.907228	9.360754	9.778371	10.63898	10.62792	10.53369
AT3G13750	16754.57	-0.87796	0.158388	-5.54306	2.97E-08	5.10E-07	13.78449	13.41745	13.29127	14.51439	14.50806	14.19492
AT5G52170	382.1064	-0.87789	0.147527	-5.95069	2.67E-09	5.25E-08	8.101292	7.936808	8.096566	8.846731	9.174942	8.866864
AT2G28900	1023.739	-0.87662	0.196958	-4.4508	8.55E-06	0.0001	9.530334	9.195659	9.669916	10.76174	10.20668	10.0902
AT5G51970	2832.567	-0.87404	0.114181	-7.65486	1.94E-14	6.17E-13	11.01559	10.89506	10.95542	11.85978	11.96288	11.70241
AT5G19120	3241.729	-0.8735	0.147991	-5.90237	3.58E-09	6.95E-08	11.34537	10.92418	11.14218	12.10321	12.16102	11.84225
AT2G40070	214.3558	-0.87329	0.172377	-5.06616	4.06E-07	5.94E-06	7.308691	7.181886	7.200663	8.294657	8.150563	7.92557
AT1G59900	2164.103	-0.87195	0.143057	-6.09516	1.09E-09	2.25E-08	10.57957	10.44823	10.66445	11.6201	11.52395	11.19703
AT5G47180	262.0369	-0.87146	0.195249	-4.4633	8.07E-06	9.48E-05	7.741388	7.20672	7.561677	8.621127	8.399116	8.210684
AT2G41410	1124.026	-0.87135	0.149008	-5.84768	4.98E-09	9.48E-08	9.74941	9.380533	9.714871	10.36467	10.5007	10.65748
AT4G21980	358.3656	-0.87034	0.155186	-5.60839	2.04E-08	3.58E-07	8.109437	7.796625	7.967117	8.742349	9.035019	8.828473
AT3G27210	65.39704	-0.86872	0.26477	-3.28105	0.001034	0.007403	5.40795	5.535346	5.531776	5.947137	6.679627	6.630478
AT1G09027	328.4195	-0.86854	0.18264	-4.75549	1.98E-06	2.58E-05	8.138912	7.753456	7.650749	8.797415	8.48767	8.888795
AT1G30360	2302.417	-0.86851	0.122726	-7.07678	1.48E-12	4.00E-11	10.66953	10.5695	10.74179	11.6615	11.5779	11.37904

AT4G01120	494.4234	-0.86846	0.177309	-4.89798	9.68E-07	1.34E-05	8.660684	8.22875	8.373253	9.49631	9.419665	9.04763
AT5G57220	99.44605	-0.86819	0.26619	-3.26153	0.001108	0.007848	6.460429	6.00054	5.907211	6.772191	6.679627	7.481917
AT2G28200	213.6418	-0.86758	0.181266	-4.78625	1.70E-06	2.24E-05	7.391172	7.104714	7.18938	8.316365	8.095267	7.931619
AT5G44260	1518.195	-0.86724	0.173787	-4.99026	6.03E-07	8.61E-06	10.20261	9.713337	10.18781	11.05029	11.09064	10.6702
AT1G07420	175.0805	-0.86686	0.202524	-4.28029	1.87E-05	0.000205	6.582468	6.97195	7.166548	7.757664	8.008134	7.765446
AT4G28720	353.6178	-0.8655	0.177899	-4.86511	1.14E-06	1.56E-05	8.031574	7.691466	8.123492	9.04828	8.795846	8.667428
AT5G06870	239.8886	-0.86495	0.19242	-4.49511	6.95E-06	8.29E-05	7.284918	7.515804	7.266564	8.051003	8.687846	8.102229
AT1G20693	1647.781	-0.86369	0.142236	-6.07226	1.26E-09	2.57E-08	9.974167	10.22939	10.32043	11.22257	11.01817	10.92492
AT4G39900	352.7455	-0.86354	0.188153	-4.58956	4.44E-06	5.46E-05	8.011433	7.698487	8.090514	9.00549	8.990252	8.4984
AT4G12290	217.2281	-0.86308	0.207682	-4.15576	3.24E-05	0.000337	6.839079	7.425602	7.40468	7.929492	8.229725	8.273953
AT5G41260	649.8513	-0.86103	0.127733	-6.74092	1.57E-11	3.88E-10	8.914164	8.748419	8.852438	9.774385	9.776754	9.602593
AT1G10760	1374.718	-0.85829	0.156031	-5.50076	3.78E-08	6.41E-07	9.681838	10.06188	9.996353	11.01089	10.72675	10.62984
AT3G16180	117.9875	-0.85816	0.203757	-4.21167	2.53E-05	0.000271	6.210797	6.351747	6.595172	7.35799	7.182411	7.249844
AT5G15150	68.3147	-0.85745	0.241886	-3.54485	0.000393	0.003164	5.708842	5.369304	5.618455	6.457472	6.679627	6.370353
AT1G75220	829.2414	-0.85629	0.127302	-6.72645	1.74E-11	4.26E-10	9.252448	9.256389	9.054053	10.00074	10.19358	10.01242
AT1G45976	436.1775	-0.85465	0.154934	-5.51622	3.46E-08	5.89E-07	8.424347	8.11791	8.204183	9.077177	9.350505	9.005234
AT2G19460	134.9	-0.85396	0.226602	-3.76854	0.000164	0.001468	6.871365	6.221336	6.534049	7.616061	7.51294	7.249844
AT3G23710	138.484	-0.85352	0.189061	-4.5145	6.35E-06	7.61E-05	6.792628	6.551075	6.488747	7.516426	7.51294	7.465332
AT5G19860	585.205	-0.85198	0.150424	-5.66385	1.48E-08	2.64E-07	8.656984	8.558907	8.819884	9.547686	9.776754	9.363988
AT4G05060	292.0458	-0.85196	0.176209	-4.83494	1.33E-06	1.80E-05	7.748358	7.551139	7.726857	8.701689	8.724751	8.249946
AT2G29630	1366.771	-0.85175	0.128912	-6.6072	3.92E-11	9.36E-10	9.908006	9.916667	9.932655	10.92519	10.8362	10.57872
AT5G52450	121.1913	-0.85154	0.202099	-4.21349	2.51E-05	0.000269	6.651025	6.306513	6.302328	7.315505	7.284819	7.316087
AT5G02150	421.0041	-0.85104	0.187292	-4.54394	5.52E-06	6.69E-05	8.393554	7.951575	8.248344	9.281363	9.201369	8.758729
AT3G55880	69.38622	-0.85099	0.248833	-3.41991	0.000626	0.004785	5.650502	5.684235	5.341032	6.356317	6.88921	6.315891
AT5G51070	2281.298	-0.85081	0.131037	-6.4929	8.42E-11	1.92E-09	10.65893	10.66264	10.6429	11.32517	11.74216	11.48465
AT5G14420	288.5383	-0.85039	0.159453	-5.33316	9.65E-08	1.55E-06	7.647563	7.623008	7.683285	8.49355	8.813085	8.339123
AT3G46440	157.6537	-0.84999	0.181359	-4.68681	2.78E-06	3.53E-05	6.651025	6.930809	6.831246	7.757664	7.672573	7.608149
AT5G58640	198.7301	-0.84991	0.186053	-4.56812	4.92E-06	6.00E-05	7.079152	7.011951	7.200663	7.86467	8.376102	7.792291
AT3G03970	57.60112	-0.84971	0.253938	-3.34614	0.000819	0.006065	5.279101	5.503631	5.320494	6.417857	6.13862	6.159727
AT5G51550	1030.057	-0.84913	0.142572	-5.95582	2.59E-09	5.11E-08	9.602314	9.246842	9.657687	10.37505	10.38442	10.37626
AT1G22190	527.3634	-0.84775	0.190807	-4.44296	8.87E-06	0.000103	8.873297	8.216575	8.460313	9.603931	9.373929	9.252416
AT2G48030	89.85167	-0.84774	0.255327	-3.32023	0.000899	0.006583	5.981541	5.535346	6.343626	6.756396	6.88921	6.99642
AT1G04503	854.8221	-0.84667	0.156417	-5.41291	6.20E-08	1.03E-06	8.985222	9.26469	9.44528	10.23817	10.11235	9.967487

AT1G20450	807.6555	-0.84602	0.165866	-5.10059	3.39E-07	5.02E-06	9.271928	9.147733	9.030537	10.15929	10.20668	9.676501
AT5G27280	401.3455	-0.84501	0.159512	-5.29746	1.17E-07	1.87E-06	8.048616	8.086142	8.30171	9.14547	9.106676	8.795682
AT1G66180	502.4392	-0.84468	0.15898	-5.31313	1.08E-07	1.72E-06	8.58288	8.354082	8.441416	9.32217	9.599327	9.094218
AT1G07640	87.33369	-0.84467	0.242964	-3.47653	0.000508	0.003971	5.73715	6.201705	5.822109	6.533581	7.013727	6.961367
AT5G58375	239.2406	-0.8428	0.166428	-5.06403	4.10E-07	6.00E-06	7.539193	7.240783	7.399794	8.278159	8.42177	8.139294
AT1G09570	6619.684	-0.84178	0.14295	-5.8886	3.89E-09	7.53E-08	12.19086	12.18384	12.22416	13.21797	13.17009	12.74534
AT4G12730	392.2338	-0.84055	0.184221	-4.56272	5.05E-06	6.15E-05	7.949268	7.969098	8.353172	8.746353	9.290227	8.904259
AT5G26570	796.8401	-0.83994	0.148505	-5.65592	1.55E-08	2.75E-07	8.982269	9.284655	9.163328	10.17571	9.996398	9.823636
AT3G11690	231.6252	-0.83992	0.20695	-4.05857	4.94E-05	0.000497	7.658705	7.083418	7.211858	8.221762	8.48767	7.979124
AT4G13530	670.8813	-0.83896	0.137743	-6.09076	1.12E-09	2.30E-08	8.831239	8.932373	8.908557	9.778301	9.934731	9.550475
AT1G08980	1319.055	-0.83847	0.141469	-5.92688	3.09E-09	6.02E-08	10.02097	9.660387	9.925023	10.8509	10.73582	10.59596
AT4G13510	581.7016	-0.83725	0.175273	-4.77684	1.78E-06	2.33E-05	8.753686	8.608203	8.703758	9.169524	9.628833	9.797374
AT2G36320	527.9055	-0.83535	0.136653	-6.11293	9.78E-10	2.02E-08	8.508872	8.505867	8.626388	9.415642	9.569204	9.247575
AT1G25560	2117.728	-0.83475	0.153174	-5.44968	5.05E-08	8.45E-07	10.69185	10.30838	10.64961	11.59145	11.41244	11.21202
AT4G13505	582.3174	-0.83309	0.175192	-4.7553	1.98E-06	2.58E-05	8.757146	8.613785	8.707718	9.169524	9.628833	9.797374
AT5G26280	63.45387	-0.83295	0.251735	-3.30885	0.000937	0.00681	5.635541	5.421153	5.513797	6.20161	6.244072	6.630478
AT5G06865	264.997	-0.82978	0.168668	-4.91963	8.67E-07	1.21E-05	7.713163	7.421365	7.49451	8.233219	8.630654	8.39711
AT1G21610	414.828	-0.82951	0.145768	-5.69059	1.27E-08	2.27E-07	8.301767	8.181928	8.111587	9.05152	9.240123	8.898094
AT1G20696	1059.914	-0.82935	0.141094	-5.87798	4.15E-09	8.00E-08	9.436343	9.653178	9.615107	10.6151	10.31351	10.27878
AT1G10210	182.0865	-0.82889	0.196099	-4.22689	2.37E-05	0.000254	7.289704	6.766821	6.969577	7.977969	7.883112	7.778931
AT1G09530	269.5298	-0.82649	0.154452	-5.35112	8.74E-08	1.41E-06	7.662401	7.491758	7.617462	8.521853	8.48767	8.311553
AT3G26220	477.4511	-0.82642	0.207801	-3.97697	6.98E-05	0.000681	8.758873	8.320346	8.084436	9.086682	9.078439	9.573871
AT1G01650	311.3171	-0.82638	0.157276	-5.25436	1.49E-07	2.33E-06	7.680736	7.879272	7.772935	8.51248	8.896321	8.542549
AT2G40960	151.0514	-0.82447	0.186972	-4.40957	1.04E-05	0.00012	6.702182	6.719289	6.801913	7.684432	7.78168	7.370542
AT2G19180	232.043	-0.82316	0.170488	-4.82825	1.38E-06	1.85E-05	7.294474	7.278753	7.489919	8.024871	8.466036	8.190645
AT1G53320	566.2677	-0.82283	0.145056	-5.67248	1.41E-08	2.52E-07	8.649557	8.600728	8.731248	9.621476	9.609229	9.276383
AT1G56220	8188.216	-0.82256	0.135028	-6.09177	1.12E-09	2.29E-08	12.65351	12.29716	12.58862	13.4919	13.33358	13.23728
AT3G14990	1971.389	-0.82233	0.119815	-6.86332	6.73E-12	1.71E-10	10.53693	10.4903	10.36435	11.16391	11.42931	11.31048
AT1G71920	92.51752	-0.82148	0.21514	-3.81834	0.000134	0.001225	6.095261	5.869819	6.159311	6.9213	7.013727	6.811962
AT3G26580	856.8209	-0.82031	0.147659	-5.55545	2.77E-08	4.78E-07	9.148258	9.395731	9.230917	10.23675	10.18698	9.865318
AT1G07310	309.303	-0.81995	0.22031	-3.72179	0.000198	0.001732	7.677087	7.499818	8.044294	8.445107	9.092627	8.311553
AT4G08425	203.6443	-0.81987	0.193666	-4.23341	2.30E-05	0.000247	7.408878	7.226283	6.867092	7.89384	8.12318	8.102229
AT3G13672	250.8433	-0.81935	0.150882	-5.43041	5.62E-08	9.34E-07	7.514872	7.454919	7.476057	8.272618	8.48767	8.259597

AT2G02060	88.1431	-0.81927	0.227792	-3.59656	0.000322	0.002652	5.993325	5.894484	5.907211	6.756396	7.234523	6.538339
AT1G19370	137.3898	-0.8183	0.182686	-4.47928	7.49E-06	8.85E-05	6.673172	6.558811	6.67823	7.562569	7.42597	7.414402
AT1G03610	976.8124	-0.81813	0.146016	-5.60304	2.11E-08	3.69E-07	9.515088	9.296271	9.532691	10.39941	10.40162	10.05322
AT2G42890	888.5814	-0.81733	0.121281	-6.73917	1.59E-11	3.93E-10	9.354194	9.307795	9.284919	10.16079	10.2829	10.01527
AT1G20840	462.499	-0.81724	0.174078	-4.69469	2.67E-06	3.40E-05	8.339185	8.410753	8.30171	9.022099	9.599327	8.982103
AT5G57880	201.9258	-0.81698	0.204743	-3.99026	6.60E-05	0.000647	7.481794	6.825719	7.160782	8.132803	8.008134	7.931619
AT4G36730	1446.106	-0.8164	0.158635	-5.14641	2.66E-07	4.00E-06	10.20894	9.83858	9.970222	10.7196	11.12211	10.70152
AT3G20410	489.7209	-0.81556	0.149617	-5.45099	5.01E-08	8.39E-07	8.565224	8.342924	8.419856	9.163548	9.538439	9.183111
AT1G49720	261.0511	-0.81225	0.157942	-5.14272	2.71E-07	4.07E-06	7.539193	7.495793	7.683285	8.5358	8.229725	8.361702
AT1G26270	627.9244	-0.81156	0.151594	-5.35352	8.63E-08	1.40E-06	8.989641	8.852796	8.601068	9.772423	9.648174	9.526693
AT1G02860	294.5433	-0.81051	0.140274	-5.77808	7.56E-09	1.40E-07	7.772494	7.722794	7.726857	8.576848	8.529989	8.569957
AT1G06460	478.8233	-0.81047	0.181076	-4.47582	7.61E-06	8.99E-05	8.329921	8.308923	8.634731	9.547686	9.188216	8.996603
AT4G36850	2699.208	-0.80942	0.142207	-5.69181	1.26E-08	2.26E-07	11.11332	10.77774	10.86258	11.74641	11.92374	11.57342
AT4G38540	374.9243	-0.80922	0.184505	-4.38588	1.16E-05	0.000132	8.251135	7.969098	7.957173	8.540419	9.106676	9.044842
AT5G04375	190.2757	-0.80805	0.181197	-4.45951	8.21E-06	9.63E-05	6.97603	7.023179	7.250369	7.991526	8.095267	7.72422
AT2G01090	300.1923	-0.80802	0.250263	-3.22868	0.001244	0.008662	7.769071	7.615609	7.734639	8.28368	9.240123	8.041989
AT2G15695	471.4321	-0.80746	0.145652	-5.54374	2.96E-08	5.09E-07	8.359814	8.380515	8.453256	9.245038	9.442	9.022342
AT1G12090	2832.106	-0.80656	0.165668	-4.86853	1.12E-06	1.54E-05	10.69681	10.91743	11.30393	11.90704	11.87327	11.68147
AT2G21500	187.5664	-0.80605	0.179115	-4.50018	6.79E-06	8.10E-05	7.299229	6.960315	6.99572	7.886602	7.850087	7.990759
AT1G09970	659.6074	-0.80603	0.177507	-4.54087	5.60E-06	6.78E-05	8.982269	8.983564	8.663555	9.359243	9.950397	9.813844
AT5G62470	444.8141	-0.80302	0.160895	-4.99097	6.01E-07	8.58E-06	8.541342	8.189423	8.212567	9.213571	9.265393	8.979186
AT2G09510	59.9495	-0.80282	0.245629	-3.26843	0.001081	0.007696	5.526228	5.421153	5.495591	6.335206	6.024848	6.422834
AT3G12400	356.0116	-0.80262	0.160722	-4.99387	5.92E-07	8.46E-06	8.07658	7.854343	8.031715	8.801269	9.078439	8.615763
AT5G65110	846.7842	-0.8021	0.11478	-6.98818	2.78E-12	7.39E-11	9.265869	9.255199	9.252761	9.994019	10.18698	10.05599
AT5G24460	148.2272	-0.80076	0.213465	-3.75125	0.000176	0.001558	6.620957	6.511758	6.949654	7.39926	7.946977	7.379421
AT3G19930	286.5599	-0.80059	0.187481	-4.27024	1.95E-05	0.000213	7.842569	7.507833	7.646629	8.192714	8.830121	8.514611
AT1G27100	1139.333	-0.79855	0.122709	-6.50771	7.63E-11	1.75E-09	9.775236	9.620284	9.67498	10.35814	10.56825	10.57968
AT4G28270	467.0611	-0.79818	0.149453	-5.34063	9.26E-08	1.50E-06	8.320597	8.320346	8.566601	9.354005	9.214403	9.080671
AT1G64230	3458.18	-0.79681	0.154212	-5.16699	2.38E-07	3.60E-06	11.42584	11.01696	11.39576	12.25178	12.15765	11.88846
AT2G25450	2428.972	-0.79667	0.107463	-7.41342	1.23E-13	3.65E-12	10.86619	10.78311	10.70364	11.56223	11.68009	11.54015
AT5G19530	330.4944	-0.79657	0.169216	-4.70742	2.51E-06	3.21E-05	7.758752	7.989277	7.916697	8.808946	8.912407	8.435932
AT3G55430	727.3671	-0.79548	0.162784	-4.88673	1.03E-06	1.41E-05	8.986697	9.017646	9.104479	9.501056	10.02627	10.00383
AT4G19850	80.76421	-0.7951	0.232206	-3.4241	0.000617	0.004729	5.832072	5.966048	6.00054	6.9213	6.13862	6.850789

AT4G26060	93.06509	-0.79375	0.242823	-3.26883	0.00108	0.007687	6.005013	5.793186	6.248986	6.570181	7.33342	6.811962
AT2G42580	1533.626	-0.79271	0.136861	-5.79208	6.95E-09	1.29E-07	10.03172	10.06188	10.26373	11.05836	10.98812	10.73043
AT1G09560	137.0286	-0.79164	0.222592	-3.55645	0.000376	0.003039	6.709344	6.395605	6.67823	7.030399	7.816289	7.498313
AT4G35790	1019.993	-0.79144	0.121152	-6.53261	6.46E-11	1.50E-09	9.596532	9.542435	9.451196	10.28434	10.49002	10.25365
AT5G04105	7783.806	-0.7911	0.158689	-4.98523	6.19E-07	8.80E-06	12.45024	12.5291	12.40923	12.90058	13.50912	13.35224
AT1G28680	102.7884	-0.79052	0.220854	-3.57939	0.000344	0.002817	6.138352	6.120395	6.27056	7.003888	7.42597	6.717084
AT3G62090	149.6828	-0.78988	0.207048	-3.81496	0.000136	0.001239	6.590249	6.838488	6.888179	7.86467	7.47011	7.361607
AT1G01820	603.4784	-0.78976	0.133083	-5.93436	2.95E-09	5.77E-08	8.871702	8.722823	8.742872	9.685432	9.619065	9.446577
AT5G67490	450.3268	-0.7895	0.159653	-4.94511	7.61E-07	1.07E-05	8.332243	8.11791	8.575295	9.175475	9.240123	9.086105
AT3G46970	534.2856	-0.78924	0.164133	-4.80852	1.52E-06	2.02E-05	8.512971	8.639548	8.695807	9.689596	9.106676	9.357262
AT1G21000	753.8399	-0.78904	0.117208	-6.73193	1.67E-11	4.11E-10	9.183402	9.071367	9.058711	9.942601	9.910909	9.860569
AT5G46710	309.7769	-0.78901	0.170807	-4.61931	3.85E-06	4.79E-05	7.868394	7.680871	7.94716	8.545023	8.399116	8.870017
AT3G01310	1054.483	-0.78882	0.149717	-5.26877	1.37E-07	2.16E-06	9.418973	9.646841	9.672957	10.56724	10.42423	10.15368
AT2G30360	86.16593	-0.78857	0.243111	-3.24367	0.00118	0.008284	5.858084	6.201705	5.684234	6.551997	7.128345	6.731026
AT2G02390	485.8591	-0.78808	0.126869	-6.21172	5.24E-10	1.10E-08	8.437346	8.487744	8.481282	9.181402	9.326696	9.311602
AT4G08850	572.8812	-0.78687	0.188058	-4.18421	2.86E-05	0.000302	8.881246	8.626724	8.583937	9.154537	9.474868	9.831746
AT3G02040	293.0462	-0.78679	0.161053	-4.88531	1.03E-06	1.42E-05	7.87796	7.666621	7.691305	8.722162	8.466036	8.423107
AT3G56140	668.8504	-0.78664	0.136256	-5.77327	7.77E-09	1.43E-07	8.866906	8.924909	8.998573	9.869229	9.759072	9.55636
AT3G16720	262.3374	-0.78612	0.182188	-4.31492	1.60E-05	0.000177	7.647563	7.28343	7.772935	8.267055	8.376102	8.510575
AT1G05010	4113.877	-0.78612	0.133908	-5.8706	4.34E-09	8.32E-08	11.62952	11.36904	11.64116	12.49486	12.36181	12.18189
AT4G38150	92.27215	-0.78526	0.219663	-3.57482	0.00035	0.002855	6.289368	5.894484	6.051279	6.892686	6.822676	6.949491
AT3G19680	1546.738	-0.78399	0.176308	-4.44673	8.72E-06	0.000102	10.02169	9.797484	10.49782	10.94274	10.97286	10.91656
AT2G35470	274.7333	-0.78263	0.158403	-4.94076	7.78E-07	1.09E-05	7.67343	7.467304	7.780474	8.38987	8.529989	8.444419
AT3G22200	1095.215	-0.78097	0.157685	-4.95274	7.32E-07	1.03E-05	9.415693	9.653178	9.816455	10.2138	10.63763	10.45817
AT1G12790	119.7927	-0.78016	0.219577	-3.553	0.000381	0.003077	6.518668	6.297293	6.383775	7.203473	7.672573	6.900984
AT4G19530	2488.646	-0.78007	0.137908	-5.65646	1.55E-08	2.74E-07	10.85574	10.75645	10.86922	11.7389	11.75113	11.35505
AT5G55140	215.1774	-0.77988	0.192005	-4.06175	4.87E-05	0.000492	7.547209	7.191871	7.211858	8.192714	7.709866	8.292876
AT3G07780	772.7882	-0.77961	0.143093	-5.44829	5.09E-08	8.50E-07	9.2327	9.229982	8.927355	9.928575	10.0771	9.812205
AT2G18050	2173.13	-0.77934	0.18606	-4.18863	2.81E-05	0.000298	10.51971	10.63363	10.72675	11.8332	11.27301	11.08343
AT4G27450	3337.666	-0.77932	0.152895	-5.0971	3.45E-07	5.10E-06	11.41191	11.04271	11.2732	12.15834	12.17776	11.78074
AT2G33150	2495.168	-0.77901	0.139418	-5.58761	2.30E-08	4.01E-07	10.63938	10.93759	10.89853	11.48466	11.82097	11.5642
AT2G30362	86.71422	-0.77891	0.242419	-3.21308	0.001313	0.009076	5.870916	6.221336	5.70022	6.570181	7.128345	6.731026
AT5G63650	164.5611	-0.77858	0.24436	-3.18621	0.001441	0.009822	6.590249	6.882307	7.211858	8.138907	7.47011	7.423016

AT2G19340	111.0689	-0.77825	0.22069	-3.52645	0.000421	0.003366	6.279777	6.056257	6.612171	7.082003	7.284819	7.118118
AT3G09745	129.9051	-0.77761	0.2037	-3.8174	0.000135	0.001229	6.399741	6.648549	6.686279	7.589563	7.284819	7.220496
AT1G78080	852.9774	-0.77757	0.178199	-4.36349	1.28E-05	0.000145	9.550414	8.980688	9.243245	10.15327	10.24529	9.830128
AT3G11570	202.5374	-0.77745	0.171404	-4.53576	5.74E-06	6.94E-05	7.255862	7.211635	7.083661	7.886602	8.280186	7.88873
AT5G41990	332.8228	-0.77733	0.140805	-5.52061	3.38E-08	5.76E-07	8.011433	7.933836	7.835792	8.718091	8.778398	8.68544
AT1G32090	201.2156	-0.77622	0.188247	-4.12338	3.73E-05	0.000384	7.13341	7.176868	7.287879	8.227502	8.037767	7.710211
AT2G39900	124.8281	-0.77614	0.205238	-3.78164	0.000156	0.001399	6.298895	6.7534	6.460867	7.336904	7.33342	7.297468
AT5G09130	780.9648	-0.77614	0.145981	-5.3167	1.06E-07	1.69E-06	9.30185	9.039928	9.132731	9.766522	9.950397	10.11964
AT5G53130	596.19	-0.77537	0.134858	-5.74956	8.95E-09	1.64E-07	8.829597	8.619344	8.864902	9.63881	9.559022	9.486164
AT5G09855	435.7478	-0.77531	0.18001	-4.30707	1.65E-05	0.000183	8.260917	7.994991	8.622199	9.045033	9.148022	9.152359
AT5G30495	146.8986	-0.77409	0.180298	-4.29337	1.76E-05	0.000194	6.799356	6.691408	6.823968	7.516426	7.42597	7.674584
AT5G67488	451.7392	-0.77399	0.161051	-4.80587	1.54E-06	2.05E-05	8.343795	8.125744	8.596804	9.169524	9.240123	9.088815
AT3G01290	2145.924	-0.77393	0.208213	-3.71698	0.000202	0.001758	10.52632	10.56326	10.73016	10.90204	11.31597	11.86574
AT4G04220	111.6791	-0.77101	0.204794	-3.76481	0.000167	0.001486	6.417342	6.240703	6.302328	6.976881	7.47011	7.053013
AT1G49050	281.5549	-0.77049	0.191681	-4.01966	5.83E-05	0.00058	7.955305	7.387012	7.621665	8.316365	8.706416	8.435932
AT2G41430	7761.238	-0.7701	0.142391	-5.40833	6.36E-08	1.05E-06	12.63365	12.24596	12.52356	13.41004	13.26789	13.0909
AT5G67630	312.2279	-0.76846	0.180063	-4.26771	1.97E-05	0.000215	7.94624	7.95744	7.634201	8.479187	8.466036	8.891901
AT5G43430	221.5303	-0.76701	0.172474	-4.44712	8.70E-06	0.000102	7.345933	7.196838	7.471407	8.233219	8.229725	7.949617
AT3G51500	243.3254	-0.76666	0.166855	-4.59473	4.33E-06	5.33E-05	7.578837	7.320308	7.587692	8.41524	8.12318	8.24023
AT4G02370	333.531	-0.76638	0.163649	-4.68307	2.83E-06	3.59E-05	8.090361	7.746698	8.015834	8.808946	8.48767	8.825227
AT1G53430	376.0147	-0.76207	0.150707	-5.05665	4.27E-07	6.21E-06	8.233855	8.096809	7.999777	8.730271	8.959616	8.990821
AT2G30590	240.8795	-0.76188	0.16769	-4.5434	5.54E-06	6.70E-05	7.605954	7.404291	7.344921	8.250236	8.444073	8.086048
AT5G04325	438.4325	-0.76184	0.186317	-4.08897	4.33E-05	0.000441	8.448089	8.306627	8.204183	8.689264	9.314642	9.290574
AT5G04315	438.4325	-0.76184	0.186317	-4.08897	4.33E-05	0.000441	8.448089	8.306627	8.204183	8.689264	9.314642	9.290574
AT1G66890	153.7783	-0.76118	0.178876	-4.25533	2.09E-05	0.000226	6.909179	6.746642	6.838487	7.717439	7.594963	7.514525
AT1G11530	365.9611	-0.76068	0.161052	-4.72322	2.32E-06	3.00E-05	8.167796	8.009177	8.053657	9.057977	8.846958	8.634428
AT4G05150	2051.663	-0.75975	0.139973	-5.42782	5.70E-08	9.47E-07	10.57369	10.34127	10.75285	11.4501	11.30991	11.24822
AT2G18170	270.5981	-0.75941	0.156188	-4.86213	1.16E-06	1.58E-05	7.713163	7.551139	7.642499	8.498306	8.508985	8.259597
AT4G32340	267.0504	-0.7593	0.172411	-4.40401	1.06E-05	0.000122	7.632571	7.38266	7.817587	8.469531	8.444073	8.297568
AT3G20680	272.512	-0.75913	0.167627	-4.5287	5.93E-06	7.15E-05	7.651287	7.596945	7.663036	8.526517	8.611075	8.149711
AT1G21770	574.046	-0.75891	0.161386	-4.70244	2.57E-06	3.28E-05	8.950886	8.450802	8.721491	9.510504	9.599327	9.405874
AT5G57550	268.0858	-0.75838	0.161935	-4.68322	2.82E-06	3.59E-05	7.826191	7.507833	7.566045	8.405146	8.328941	8.473736
AT5G05080	771.679	-0.75828	0.143576	-5.28142	1.28E-07	2.03E-06	9.175666	9.02883	9.240514	10.04041	10.02627	9.705035

AT3G53530	213.7985	-0.75791	0.164552	-4.60589	4.11E-06	5.08E-05	7.426369	7.171832	7.319272	8.151039	8.066803	8.025114
AT1G69850	358.9454	-0.75684	0.165069	-4.58501	4.54E-06	5.57E-05	8.246219	7.799893	8.047422	8.770145	8.959616	8.762127
AT3G49160	99.11275	-0.75616	0.218995	-3.45287	0.000555	0.004294	6.127699	6.333823	6.051279	6.724278	7.182411	7.053013
AT1G27630	227.1785	-0.75513	0.172037	-4.38936	1.14E-05	0.00013	7.231194	7.487711	7.49451	8.343049	8.008134	8.112917
AT5G11670	865.1911	-0.75482	0.13375	-5.6435	1.67E-08	2.94E-07	9.442803	9.27764	9.224022	9.928575	10.21967	10.12361
AT5G16110	2137.185	-0.75452	0.167125	-4.51472	6.34E-06	7.60E-05	10.88726	10.38218	10.55003	11.55311	11.46519	11.14786
AT4G23180	346.9464	-0.75428	0.210267	-3.58724	0.000334	0.002739	8.208804	7.977781	7.757739	8.327098	8.8636	9.066996
AT5G24030	325.6491	-0.75335	0.188579	-3.9949	6.47E-05	0.000637	7.819587	7.698487	8.135299	8.770145	8.896321	8.375082
AT5G55700	421.1816	-0.75085	0.154211	-4.86896	1.12E-06	1.54E-05	8.277878	8.184431	8.330241	8.879911	9.33865	8.94071
AT4G13830	922.0975	-0.75069	0.161194	-4.65708	3.21E-06	4.04E-05	9.43202	9.188197	9.580977	10.27051	10.33754	9.921112
AT4G24690	1950.201	-0.75029	0.103321	-7.26174	3.82E-13	1.08E-11	10.49713	10.49434	10.50409	11.25875	11.34589	11.17616
AT5G65430	2677.955	-0.75024	0.122406	-6.12908	8.84E-10	1.83E-08	10.98972	10.7827	11.08092	11.75836	11.76448	11.62985
AT3G59080	177.5376	-0.7497	0.18974	-3.95119	7.78E-05	0.000754	7.255862	6.894586	6.989228	8.004957	7.672573	7.72422
AT1G03870	1616.254	-0.74572	0.158264	-4.7119	2.45E-06	3.15E-05	10.27137	9.909121	10.44246	10.91544	11.13587	10.91656
AT4G39660	364.7761	-0.74537	0.166051	-4.48881	7.16E-06	8.52E-05	8.152113	7.903778	8.108595	8.709913	9.148022	8.67466
AT4G18010	702.4317	-0.74476	0.146363	-5.08846	3.61E-07	5.32E-06	8.882831	9.113999	9.093929	9.649539	9.704689	9.980676
AT4G19190	430.7576	-0.74156	0.163595	-4.53287	5.82E-06	7.03E-05	8.294642	8.457025	8.201378	9.270283	9.12059	8.834943
AT4G35560	164.2834	-0.74118	0.220261	-3.36501	0.000765	0.005699	6.574646	6.88846	7.166548	7.525773	8.066803	7.506442
AT1G78420	300.8771	-0.74108	0.157169	-4.7152	2.41E-06	3.11E-05	7.890617	7.860616	7.683285	8.722162	8.508985	8.448643
AT1G22070	151.1338	-0.74059	0.210078	-3.5253	0.000423	0.003378	6.758509	6.726176	7.046626	7.589563	7.128345	7.869951
AT4G25500	622.9341	-0.74048	0.134641	-5.49962	3.81E-08	6.45E-07	8.841054	8.793377	8.917132	9.664427	9.741171	9.431715
AT4G36780	651.5322	-0.73999	0.151893	-4.87176	1.11E-06	1.52E-05	9.005729	8.746726	8.962575	9.619294	9.918893	9.488218
AT1G76460	319.9867	-0.73925	0.190289	-3.88486	0.000102	0.000966	7.909398	7.816122	7.882077	8.701689	8.990252	8.225533
AT1G76180	3423.3	-0.73912	0.109281	-6.76349	1.35E-11	3.35E-10	11.35882	11.35411	11.23975	12.12721	12.12524	11.94488
AT3G63160	1006.401	-0.73775	0.18708	-3.94349	8.03E-05	0.000776	9.570219	9.418232	9.651534	10.70424	10.0771	10.03226
AT5G10650	197.5609	-0.73748	0.165126	-4.46616	7.96E-06	9.36E-05	7.336712	7.161707	7.119768	7.991526	7.946977	7.943643
AT1G35580	1167.756	-0.73744	0.179522	-4.10776	4.00E-05	0.000409	9.993365	9.552157	9.67498	10.38536	10.84879	10.27997
AT4G31290	1770.787	-0.73373	0.151606	-4.8397	1.30E-06	1.76E-05	10.44391	10.17568	10.47128	11.35169	11.02558	10.93926
AT5G62020	203.7446	-0.73298	0.172111	-4.25874	2.06E-05	0.000223	7.355095	7.333898	7.113813	7.991526	7.816289	8.154892
AT5G56550	1141.721	-0.73295	0.185612	-3.94884	7.85E-05	0.00076	10.067	9.558923	9.496529	10.71756	10.44096	10.26806
AT1G04310	190.2024	-0.732	0.179783	-4.07155	4.67E-05	0.000472	6.952077	7.13608	7.303661	7.901041	8.066803	7.765446
AT4G04221	115.3882	-0.73198	0.198851	-3.68104	0.000232	0.001993	6.468894	6.342813	6.383775	7.043474	7.47011	7.085933
AT1G71030	1362.372	-0.73149	0.141031	-5.18669	2.14E-07	3.27E-06	10.08845	9.788464	10.08273	10.88399	10.72219	10.59215

AT3G57750	91.33177	-0.73132	0.215599	-3.39204	0.000694	0.005225	6.220856	6.141156	6.03876	7.030399	6.520817	6.913264
AT4G25690	329.313	-0.73078	0.144714	-5.04979	4.42E-07	6.43E-06	8.048616	7.869974	7.889068	8.680921	8.795846	8.612001
AT1G68945	579.6149	-0.73007	0.152265	-4.79472	1.63E-06	2.15E-05	8.970394	8.595096	8.719531	9.526889	9.33865	9.623298
AT4G25620	424.6531	-0.72842	0.151324	-4.81365	1.48E-06	1.98E-05	8.492358	8.16935	8.283256	9.181402	8.959616	9.010959
AT5G62900	116.8448	-0.72756	0.212941	-3.41673	0.000634	0.004824	6.651025	6.412782	6.182258	7.107129	7.42597	7.118118
AT2G27260	121.6607	-0.72586	0.201953	-3.5942	0.000325	0.002675	6.582468	6.306513	6.569293	7.082003	7.47011	7.259495
AT5G63030	375.8844	-0.72359	0.139952	-5.17027	2.34E-07	3.54E-06	8.183311	8.133535	8.120525	9.002146	8.846958	8.775643
AT1G51610	121.5692	-0.72317	0.197473	-3.66214	0.00025	0.002125	6.518668	6.360626	6.569293	7.107129	7.51294	7.190538
AT2G36410	541.3374	-0.72285	0.145346	-4.97334	6.58E-07	9.31E-06	8.753686	8.513849	8.756314	9.552267	9.22732	9.397156
AT1G74840	763.129	-0.72274	0.174776	-4.1352	3.55E-05	0.000367	9.421156	9.002125	8.996956	10.04854	10.00392	9.625165
AT1G63830	351.1248	-0.72263	0.166213	-4.34759	1.38E-05	0.000155	8.087616	7.766877	8.234689	8.730271	8.795846	8.821974
AT5G03730	696.7028	-0.72191	0.131616	-5.48496	4.14E-08	6.99E-07	8.996976	9.125765	8.97577	9.896507	9.75015	9.641866
AT4G35730	110.3996	-0.72103	0.201661	-3.57545	0.00035	0.002849	6.327105	6.324777	6.479514	7.249335	7.128345	6.98483
AT2G30250	1024.925	-0.72044	0.206352	-3.49132	0.000481	0.003783	9.479922	9.640475	9.61616	9.822586	10.25794	10.75538
AT4G37260	715.0965	-0.72006	0.115775	-6.21948	4.99E-10	1.05E-08	9.030245	9.110055	9.096951	9.835799	9.732137	9.828507
AT1G27290	787.0924	-0.71997	0.147426	-4.88358	1.04E-06	1.43E-05	9.342725	8.944235	9.311181	10.00909	9.910909	9.904292
AT5G01520	149.1908	-0.71908	0.198142	-3.62912	0.000284	0.002373	6.765398	6.780119	6.809303	7.659171	7.78168	7.210579
AT3G32980	255.4108	-0.71836	0.192709	-3.72771	0.000193	0.001694	7.887464	7.369524	7.399794	8.343049	8.466036	8.175431
AT5G37780	618.5919	-0.71822	0.124388	-5.77405	7.74E-09	1.43E-07	8.757146	8.880842	8.954267	9.599511	9.589355	9.591173
AT4G31510	246.741	-0.71769	0.162222	-4.42411	9.68E-06	0.000112	7.695238	7.408578	7.489919	8.327098	8.304769	8.190645
AT5G20620	627.0591	-0.71702	0.136995	-5.23389	1.66E-07	2.58E-06	8.686319	8.977805	8.974127	9.625829	9.599327	9.613923
AT3G03870	743.2904	-0.71607	0.154151	-4.64527	3.40E-06	4.26E-05	9.382473	8.911377	9.058711	9.883841	9.785514	9.901212
AT2G02950	550.5869	-0.71543	0.185559	-3.85556	0.000115	0.001072	8.94483	8.479616	8.634731	9.728572	9.201369	9.288218
AT1G03220	287.663	-0.71518	0.184531	-3.87564	0.000106	0.000997	8.002715	7.659443	7.512729	8.332435	8.724751	8.431669
AT3G56720	364.1628	-0.71449	0.158098	-4.51931	6.20E-06	7.46E-05	7.890617	8.184431	8.164402	8.797415	9.02025	8.68544
AT1G12440	2089.09	-0.71431	0.137172	-5.20736	1.92E-07	2.94E-06	10.77441	10.40489	10.66241	11.47685	11.27301	11.28057
AT4G00355	768.4435	-0.71394	0.136613	-5.22596	1.73E-07	2.68E-06	9.215196	9.035776	9.247331	9.874726	10.08422	9.757062
AT1G63180	130.1927	-0.71372	0.195752	-3.64606	0.000266	0.002243	6.620957	6.421295	6.779516	7.282799	7.42597	7.370542
AT3G53460	2390.679	-0.71357	0.148275	-4.81244	1.49E-06	1.99E-05	10.99449	10.61427	10.80276	11.6514	11.63718	11.31858
AT2G22980	1324.899	-0.71355	0.166726	-4.27977	1.87E-05	0.000205	10.04877	9.992172	9.820117	10.88489	10.81923	10.31741
AT2G31800	747.0468	-0.71307	0.135963	-5.24457	1.57E-07	2.44E-06	9.268296	9.056416	9.084824	9.995702	9.853745	9.731283
AT5G48450	96.16093	-0.71106	0.220653	-3.22253	0.001271	0.008822	6.190467	5.942585	6.323125	6.9213	7.128345	6.744835
AT5G66050	285.6986	-0.71002	0.144483	-4.91423	8.91E-07	1.24E-05	7.723812	7.719346	7.79915	8.479187	8.571102	8.401475

AT2G02760	984.2183	-0.70956	0.134706	-5.26749	1.38E-07	2.18E-06	9.561341	9.349648	9.685057	10.33041	10.27047	10.17679
AT1G68410	449.6089	-0.70955	0.191316	-3.70877	0.000208	0.001811	8.251135	8.429862	8.504228	8.701689	9.22732	9.399341
AT1G02816	239.3223	-0.70948	0.169325	-4.19004	2.79E-05	0.000296	7.41765	7.467304	7.638356	8.101887	8.12318	8.414493
AT2G13360	13189.28	-0.70878	0.13755	-5.15288	2.57E-07	3.87E-06	13.2714	13.1575	13.40293	13.97659	14.20839	13.7972
AT4G21570	381.3264	-0.70813	0.148283	-4.7755	1.79E-06	2.35E-05	8.117535	8.196879	8.138236	8.842996	9.134371	8.713801
AT3G53540	733.2158	-0.70785	0.162252	-4.36266	1.28E-05	0.000145	9.264654	9.039928	8.993717	9.947826	10.00392	9.530684
AT4G36720	367.4162	-0.70683	0.160173	-4.41296	1.02E-05	0.000118	8.299396	7.936808	8.053657	8.766207	9.02025	8.748484
AT5G61210	671.9124	-0.70658	0.189873	-3.72134	0.000198	0.001733	9.116283	8.945711	8.863128	9.247865	9.836987	9.967487
AT2G36390	445.504	-0.70516	0.157292	-4.48311	7.36E-06	8.72E-05	8.294642	8.410753	8.497382	9.343472	9.02025	8.94968
AT3G03150	1909.743	-0.7051	0.146744	-4.80499	1.55E-06	2.05E-05	10.53994	10.32208	10.60094	11.31105	11.3518	10.95716
AT4G17230	325.4603	-0.70459	0.179778	-3.91919	8.88E-05	0.000851	7.927936	8.050918	7.828538	8.332435	8.742856	8.882563
AT5G05140	334.3054	-0.70458	0.143112	-4.92327	8.51E-07	1.19E-05	8.106727	7.948634	7.906398	8.746353	8.669033	8.689016
AT3G46600	1476.185	-0.70447	0.12313	-5.72131	1.06E-08	1.92E-07	10.13971	10.17064	10.07815	10.71756	10.7847	11.00384
AT2G19270	365.8684	-0.70335	0.138418	-5.08133	3.75E-07	5.51E-06	8.098567	8.026019	8.215351	8.839252	8.8636	8.798995
AT1G75540	364.064	-0.70311	0.192351	-3.65534	0.000257	0.002177	8.152113	7.851196	8.264562	9.070806	8.880053	8.48612
AT1G33420	134.1517	-0.70262	0.217156	-3.23555	0.001214	0.008484	6.408569	6.780119	6.741397	7.056432	7.554534	7.554276
AT2G45740	938.2148	-0.70262	0.123877	-5.67187	1.41E-08	2.52E-07	9.503804	9.379441	9.515853	10.18164	10.32557	10.05876
AT4G14230	220.5195	-0.70226	0.201006	-3.49372	0.000476	0.003752	7.022775	7.38266	7.650749	8.180929	8.203818	7.943643
AT1G07877	107.3874	-0.70213	0.219614	-3.19712	0.001388	0.009519	6.534884	6.109902	6.333412	7.191776	7.182411	6.863502
AT2G30440	1470.478	-0.70115	0.133747	-5.24235	1.59E-07	2.47E-06	10.28821	9.991458	10.05658	10.75281	10.98812	10.76388
AT1G02610	355.7878	-0.70105	0.169303	-4.14078	3.46E-05	0.000359	8.327596	7.930858	7.967117	8.930054	8.611075	8.8023
AT5G01750	2396.399	-0.69978	0.107926	-6.48389	8.94E-11	2.03E-09	10.87298	10.7077	10.90372	11.55938	11.53957	11.51469
AT5G23700	143.1196	-0.69906	0.187048	-3.73735	0.000186	0.001637	6.651025	6.912811	6.686279	7.389052	7.594963	7.490138
AT1G23020	607.2629	-0.69871	0.143657	-4.86376	1.15E-06	1.57E-05	8.985222	8.700266	8.827182	9.61711	9.667259	9.397156
AT3G02030	140.8198	-0.69844	0.190531	-3.66577	0.000247	0.002099	6.871365	6.648549	6.741397	7.642081	7.33342	7.370542
AT4G17150	203.2632	-0.69827	0.16971	-4.1145	3.88E-05	0.000398	7.289704	7.20672	7.255788	8.024871	8.177436	7.772204
AT1G51740	142.8284	-0.69816	0.194085	-3.59717	0.000322	0.002648	6.915386	6.684352	6.794486	7.642081	7.072174	7.600575
AT4G38545	355.995	-0.69783	0.185919	-3.75341	0.000174	0.001546	8.248679	7.933836	7.976993	8.425264	9.035019	8.885683
AT1G33970	397.9177	-0.69765	0.166704	-4.18495	2.85E-05	0.000302	8.017217	8.327157	8.345569	9.124089	8.959616	8.755322
AT1G14520	268.3164	-0.69677	0.203059	-3.43134	0.000601	0.004619	7.590521	7.442428	7.943807	8.680921	8.255176	8.165198
AT4G34180	532.3616	-0.69653	0.154265	-4.51514	6.33E-06	7.60E-05	8.8637	8.513849	8.564419	9.21646	9.442	9.450796
AT5G62910	310.735	-0.6958	0.139488	-4.98825	6.09E-07	8.68E-06	7.852308	7.912861	7.916697	8.647057	8.529989	8.593042
AT4G37770	550.0614	-0.69532	0.144456	-4.8134	1.48E-06	1.98E-05	8.604169	8.624883	8.8982	9.522227	9.314642	9.394969

AT3G45040	138.6702	-0.6944	0.188202	-3.68968	0.000225	0.001936	6.785868	6.604377	6.710159	7.260576	7.63429	7.440091
AT4G20890	609.7356	-0.69418	0.130203	-5.33158	9.74E-08	1.57E-06	8.709721	8.930883	8.932439	9.597296	9.538439	9.552439
AT2G25730	550.5496	-0.69403	0.166657	-4.16443	3.12E-05	0.000326	8.448089	8.964762	8.645091	9.435616	9.528036	9.297617
AT1G76490	8317.122	-0.69385	0.144674	-4.79595	1.62E-06	2.14E-05	12.69897	12.47467	12.68779	13.41916	13.49249	13.05168
AT5G67300	1759.619	-0.69318	0.140406	-4.93693	7.94E-07	1.11E-05	10.37465	10.14838	10.59881	11.04867	11.11517	11.11235
AT4G11360	271.7624	-0.69308	0.173598	-3.99244	6.54E-05	0.000643	7.915604	7.450767	7.691305	8.479187	8.328941	8.388339
AT3G25900	108.6267	-0.69295	0.203376	-3.40725	0.000656	0.004974	6.250617	6.395605	6.441977	6.9213	7.182411	7.170215
AT3G03330	367.7383	-0.69274	0.148755	-4.6569	3.21E-06	4.04E-05	8.243755	7.997839	8.161518	8.964833	8.742856	8.779002
AT2G28400	545.2299	-0.69272	0.167861	-4.12675	3.68E-05	0.000379	8.868506	8.570434	8.643025	9.190246	9.314642	9.660198
AT4G17460	838.4875	-0.69159	0.169614	-4.07746	4.55E-05	0.000461	9.50689	9.010612	9.361055	10.03715	10.21967	9.802335
AT4G14030	1342.078	-0.69087	0.1243	-5.55808	2.73E-08	4.72E-07	10.07534	9.944985	9.972692	10.83689	10.69455	10.55344
AT5G64090	169.7635	-0.68795	0.194706	-3.53329	0.00041	0.003286	7.068052	6.819293	7.059077	7.741709	8.008134	7.431579
AT3G52800	555.9224	-0.68776	0.209734	-3.27918	0.001041	0.007441	8.819703	8.32262	8.920548	8.998793	9.676708	9.560269
AT2G08780	359.5561	-0.68761	0.207927	-3.30698	0.000943	0.00685	8.280285	8.16935	7.784228	8.430251	8.795846	9.115632
AT1G33600	244.7849	-0.68752	0.19116	-3.59654	0.000322	0.002652	7.748358	7.421365	7.365117	7.957392	8.508985	8.278707
AT3G25910	1055.949	-0.68689	0.131671	-5.21673	1.82E-07	2.80E-06	9.764961	9.610082	9.573403	10.45673	10.41297	10.17933
AT4G21450	1043.035	-0.68643	0.128009	-5.36239	8.21E-08	1.34E-06	9.79641	9.508882	9.600274	10.40575	10.2829	10.30814
AT5G14270	586.9798	-0.68639	0.143703	-4.77647	1.78E-06	2.34E-05	8.760598	8.87	8.769633	9.621476	9.609229	9.278758
AT2G40940	714.0547	-0.68616	0.127631	-5.37617	7.61E-08	1.24E-06	9.131026	9.060509	9.063354	9.850752	9.886687	9.627031
AT3G07760	284.3786	-0.68469	0.150449	-4.55099	5.34E-06	6.48E-05	7.849069	7.641341	7.79915	8.521853	8.466036	8.401475
AT5G65210	132.5699	-0.6846	0.213699	-3.20357	0.001357	0.009338	6.812719	6.619252	6.497922	7.030399	7.51294	7.546413
AT2G46270	196.8127	-0.68436	0.179698	-3.80842	0.00014	0.00127	7.336712	7.311177	6.99572	7.984764	8.037767	7.798924
AT1G78700	290.8423	-0.68355	0.162584	-4.20429	2.62E-05	0.000279	7.782716	7.623008	7.953843	8.576848	8.550692	8.361702
AT4G27990	196.581	-0.68262	0.190164	-3.58963	0.000331	0.002716	7.022775	7.161707	7.419241	7.957392	8.150563	7.703156
AT5G54980	123.0203	-0.68255	0.201015	-3.39553	0.000685	0.005171	6.758509	6.36945	6.516099	7.27173	7.284819	7.240127
AT1G71695	939.6455	-0.68248	0.177537	-3.84419	0.000121	0.001116	9.587814	9.305497	9.520362	10.30756	10.41297	9.775678
AT2G37480	716.8232	-0.68124	0.129687	-5.25294	1.50E-07	2.34E-06	9.14033	9.002125	9.145924	9.883841	9.836987	9.651061
AT3G61260	5340.132	-0.68019	0.140772	-4.83186	1.35E-06	1.82E-05	12.0255	11.84473	12.10237	12.87269	12.70548	12.45714
AT5G63640	308.3771	-0.67997	0.158204	-4.29804	1.72E-05	0.00019	7.918697	7.835359	7.882077	8.730271	8.630654	8.361702
AT2G31360	794.0188	-0.67982	0.148982	-4.56312	5.04E-06	6.14E-05	9.007183	9.238437	9.462957	9.994019	9.910909	9.911962
AT4G26080	708.5794	-0.67916	0.163068	-4.16487	3.12E-05	0.000326	8.887575	9.270591	9.061808	9.983881	9.75015	9.566114
AT4G25970	778.3971	-0.67908	0.15747	-4.31245	1.61E-05	0.000179	9.357617	9.094172	9.198925	10.16079	9.81148	9.719094
AT2G34590	1307.164	-0.6774	0.145353	-4.66036	3.16E-06	3.98E-05	10.05159	9.745893	10.07662	10.78528	10.69455	10.47897

AT4G31420	460.2692	-0.67738	0.140921	-4.8068	1.53E-06	2.04E-05	8.527226	8.415021	8.402861	9.157547	9.326696	8.982103
AT1G75730	118.8134	-0.67732	0.199939	-3.38762	0.000705	0.005296	6.502267	6.351747	6.67823	7.293783	7.182411	7.149602
AT5G65730	2174.539	-0.67597	0.198929	-3.39805	0.000679	0.005129	11.14701	10.32037	10.47361	11.49362	11.36647	11.32261
AT5G39050	357.9147	-0.67501	0.20609	-3.27532	0.001055	0.007529	8.017217	8.206761	8.009433	8.255864	9.049638	8.982103
AT3G52240	852.9028	-0.67397	0.12839	-5.24941	1.53E-07	2.39E-06	9.45989	9.308942	9.280938	10.11817	10.09835	9.89967
AT4G32760	1214.329	-0.67331	0.150262	-4.48089	7.43E-06	8.80E-05	9.968209	9.749278	9.834674	10.48576	10.81496	10.32318
AT2G36220	434.951	-0.67259	0.200859	-3.34858	0.000812	0.006019	8.586774	8.43197	8.066048	8.730271	9.06411	9.36175
AT3G02420	174.3796	-0.67244	0.178795	-3.76094	0.000169	0.001507	7.144021	6.85743	7.166548	7.757664	7.816289	7.710211
AT1G19770	667.5066	-0.67227	0.170705	-3.93823	8.21E-05	0.000792	9.273137	8.758531	8.887768	9.547686	9.878522	9.636321
AT2G27830	515.4508	-0.67153	0.199758	-3.3617	0.000775	0.005763	8.878072	8.209221	8.695807	9.529215	9.33865	9.053189
AT2G29670	884.5069	-0.6715	0.128024	-5.24512	1.56E-07	2.44E-06	9.388063	9.323774	9.487346	10.09166	10.23893	9.943738
AT1G15400	523.9519	-0.67148	0.152199	-4.41182	1.03E-05	0.000119	8.83288	8.427751	8.655378	9.239367	9.419665	9.359508
AT4G30550	563.5716	-0.67142	0.142292	-4.71859	2.37E-06	3.06E-05	8.8637	8.604471	8.794047	9.556834	9.453039	9.309281
AT1G13270	259.898	-0.67125	0.154698	-4.3391	1.43E-05	0.00016	7.598258	7.619314	7.699282	8.425264	8.376102	8.185591
AT4G33940	330.2063	-0.67107	0.153402	-4.37459	1.22E-05	0.000138	8.011433	7.841715	8.044294	8.563295	8.896321	8.57383
AT1G78240	779.0832	-0.67017	0.138863	-4.82615	1.39E-06	1.87E-05	9.375737	9.137437	9.132731	9.947826	10.02627	9.750233
AT5G45550	651.3152	-0.66987	0.139587	-4.79894	1.60E-06	2.11E-05	9.030245	8.771905	9.096951	9.718417	9.579315	9.641866
AT2G21240	543.2693	-0.66849	0.157519	-4.24386	2.20E-05	0.000237	8.798031	8.576163	8.711666	9.517549	9.517558	9.112973
AT1G73840	215.7717	-0.66821	0.183943	-3.6327	0.00028	0.002348	7.386711	7.1257	7.517248	8.018263	8.304769	7.876237
AT5G47720	159.1068	-0.66819	0.177645	-3.7614	0.000169	0.001505	6.812719	6.989229	6.942951	7.553458	7.816289	7.530557
AT3G02320	143.3478	-0.66723	0.193705	-3.44458	0.000572	0.004414	6.785868	6.812837	6.874155	7.725575	7.072174	7.490138
AT1G18720	341.2584	-0.66547	0.15206	-4.37636	1.21E-05	0.000137	8.048616	7.857483	8.190101	8.730271	8.742856	8.68544
AT1G27150	398.2629	-0.66513	0.142183	-4.67802	2.90E-06	3.67E-05	8.301767	8.181928	8.285907	9.028689	9.005329	8.785697
AT5G61530	473.4455	-0.66484	0.124982	-5.31947	1.04E-07	1.67E-06	8.563249	8.485717	8.499668	9.219343	9.161545	9.178031
AT4G05320	18116.14	-0.66422	0.115897	-5.73117	9.97E-09	1.82E-07	13.69909	13.71346	13.88863	14.30114	14.48135	14.53526
AT5G02100	120.1763	-0.66396	0.196544	-3.37815	0.00073	0.005459	6.534884	6.487643	6.479514	7.030399	7.51294	7.149602
AT5G04040	915.3425	-0.66214	0.140446	-4.71453	2.42E-06	3.11E-05	9.499679	9.438292	9.412905	10.04204	10.39591	9.961586
AT5G55850	885.6732	-0.66073	0.162342	-4.06998	4.70E-05	0.000475	9.410209	9.20555	9.60771	10.31836	10.00392	9.915018
AT5G13730	330.0587	-0.65788	0.173059	-3.80149	0.000144	0.001301	7.806288	8.056393	8.066048	8.781895	8.830121	8.379515
AT3G16740	223.5958	-0.65785	0.155252	-4.23731	2.26E-05	0.000244	7.518954	7.421365	7.380081	8.151039	8.066803	8.096855
AT5G10960	1810.578	-0.65624	0.127481	-5.14771	2.64E-07	3.98E-06	10.60907	10.3141	10.41609	11.21175	11.11865	11.01742
AT4G36760	783.8429	-0.65589	0.12156	-5.39559	6.83E-08	1.12E-06	9.167888	9.27412	9.266247	9.820689	10.04828	9.870051
AT5G56180	317.8709	-0.65568	0.159582	-4.10873	3.98E-05	0.000408	8.020099	7.915876	7.846606	8.697559	8.760736	8.379515

AT5G66160	242.3629	-0.65541	0.17543	-3.736	0.000187	0.001645	7.56311	7.387012	7.630034	8.267055	8.444073	7.961492
AT5G62540	268.8504	-0.65499	0.151558	-4.32173	1.55E-05	0.000172	7.758752	7.708954	7.609019	8.405146	8.466036	8.25478
AT3G11560	1667.092	-0.65494	0.132371	-4.94774	7.51E-07	1.06E-05	10.33468	10.36794	10.27778	11.0299	11.1764	10.77233
AT4G34139	189.0348	-0.65452	0.188765	-3.46741	0.000526	0.004093	7.170211	7.408578	6.969577	7.998257	7.850087	7.74498
AT5G06700	758.7122	-0.65411	0.123951	-5.27719	1.31E-07	2.07E-06	9.187254	9.173156	9.208737	9.86002	10.01141	9.722587
AT3G15290	321.2817	-0.65406	0.167461	-3.90575	9.39E-05	0.000896	7.881135	7.776861	8.158628	8.664088	8.760736	8.46542
AT3G04560	241.5272	-0.65371	0.180748	-3.6167	0.000298	0.00248	7.527084	7.369524	7.642499	8.163069	8.529989	7.961492
AT5G51150	381.9071	-0.65313	0.155995	-4.18685	2.83E-05	0.0003	8.334561	8.067282	8.18443	8.957944	8.990252	8.678262
AT5G37710	257.6563	-0.65283	0.152953	-4.26814	1.97E-05	0.000215	7.688005	7.641341	7.570401	8.358826	8.399116	8.18052
AT4G16500	503.2356	-0.65266	0.145981	-4.47087	7.79E-06	9.17E-05	8.684503	8.415021	8.677754	9.273061	9.408366	9.141961
AT5G49645	344.2373	-0.65188	0.149915	-4.34831	1.37E-05	0.000154	8.203742	7.933836	7.999777	8.672529	8.846958	8.671049
AT1G16240	1128.881	-0.65155	0.140323	-4.64323	3.43E-06	4.30E-05	9.872556	9.611942	9.815538	10.57626	10.46842	10.24639
AT4G23270	305.0756	-0.6515	0.185198	-3.51787	0.000435	0.003465	7.918697	7.770213	7.970416	8.255864	8.48767	8.847796
AT1G06400	792.8023	-0.65148	0.146107	-4.45893	8.24E-06	9.65E-05	9.386947	9.072718	9.287567	9.956493	10.0771	9.74165
AT5G58800	262.8883	-0.65112	0.178683	-3.64399	0.000268	0.002257	7.590521	7.499818	7.85377	8.410202	8.508985	8.096855
AT1G21450	706.0893	-0.65082	0.121422	-5.36001	8.32E-08	1.35E-06	9.095933	9.117931	9.05094	9.726546	9.894806	9.662019
AT1G18850	167.0264	-0.6498	0.197682	-3.2871	0.001012	0.007258	6.909179	7.017576	7.089742	7.347485	7.816289	7.869951
AT4G27870	979.9735	-0.64892	0.135721	-4.78127	1.74E-06	2.29E-05	9.622373	9.526742	9.562512	10.41207	10.21319	10.04208
AT3G62550	1898.577	-0.64702	0.17095	-3.78484	0.000154	0.001384	10.34219	10.55071	10.66241	11.5102	10.91416	11.05808
AT1G59710	274.224	-0.64639	0.164308	-3.93401	8.35E-05	0.000804	7.802944	7.523732	7.828538	8.26147	8.529989	8.410167
AT1G69830	903.6544	-0.64587	0.168965	-3.82252	0.000132	0.001207	9.146939	9.623976	9.529339	10.22819	10.22612	9.870051
AT5G63880	165.7397	-0.6449	0.190887	-3.37843	0.000729	0.005455	6.92772	6.882307	7.125699	7.409395	7.946977	7.674584
AT4G17140	1212.672	-0.6449	0.16608	-3.88305	0.000103	0.000972	9.723114	10.16559	9.672957	10.4965	10.71762	10.3917
AT2G18440	1907.426	-0.64457	0.126419	-5.09867	3.42E-07	5.06E-06	10.6732	10.33678	10.57246	11.22759	11.18966	11.14721
AT3G22960	3121.831	-0.64393	0.137925	-4.66871	3.03E-06	3.83E-05	11.29895	11.11434	11.30556	12.0275	11.99345	11.65155
AT1G53400	454.8117	-0.64361	0.140541	-4.5795	4.66E-06	5.71E-05	8.563249	8.32942	8.511041	9.222221	9.078439	9.055961
AT2G20130	208.8618	-0.64337	0.176185	-3.65165	0.000261	0.002204	7.36873	7.1257	7.503648	7.901041	8.066803	8.053131
AT4G03915	221.7736	-0.64315	0.176791	-3.6379	0.000275	0.002306	7.201025	7.562728	7.462062	7.929492	8.255176	8.102229
AT1G12230	805.247	-0.64232	0.157572	-4.07633	4.58E-05	0.000463	9.207629	9.154131	9.455912	9.92151	10.18698	9.712081
AT5G21940	3175.426	-0.64213	0.150117	-4.27752	1.89E-05	0.000207	11.50951	11.0724	11.18648	12.07603	11.90778	11.77276
AT3G05120	449.5225	-0.64194	0.145458	-4.41324	1.02E-05	0.000118	8.551341	8.376143	8.429478	9.264711	9.06411	8.967456
AT4G11600	392.9987	-0.6415	0.14065	-4.56095	5.09E-06	6.19E-05	8.322934	8.199356	8.259177	8.797415	8.896321	9.028
AT2G21540	195.1513	-0.64148	0.197534	-3.24744	0.001164	0.008196	7.386711	7.006304	7.329587	8.144986	7.850087	7.68894

AT1G78895	185.617	-0.64043	0.196137	-3.26521	0.001094	0.007766	7.426369	6.924835	7.143347	7.984764	7.78168	7.72422
AT1G55520	271.6617	-0.6394	0.156295	-4.09098	4.30E-05	0.000438	7.78949	7.663037	7.734639	8.526517	8.352714	8.245097
AT5G45490	830.954	-0.63914	0.160582	-3.9801	6.89E-05	0.000673	9.475728	9.174415	9.354757	10.2495	9.87031	9.803985
AT2G37035	151.948	-0.63867	0.189991	-3.36156	0.000775	0.005764	6.981957	6.88846	6.718032	7.580621	7.74622	7.334468
AT2G34430	62767.6	-0.63821	0.176766	-3.61049	0.000306	0.002528	15.65594	15.24242	15.76519	16.52037	16.10736	16.00856
AT5G61590	1597.926	-0.63794	0.164813	-3.87066	0.000109	0.001015	10.5584	10.01696	10.21171	11.10744	10.86128	10.8114
AT5G13500	167.4633	-0.63722	0.19565	-3.25694	0.001126	0.007959	6.958103	7.072651	6.915825	7.44924	8.095267	7.514525
AT5G57900	201.7617	-0.63647	0.169008	-3.76593	0.000166	0.001481	7.303968	7.245585	7.266564	7.915337	8.203818	7.758657
AT3G42150	165.381	-0.6358	0.184631	-3.44365	0.000574	0.004425	6.915386	6.912811	7.217423	7.773445	7.594963	7.615684
AT4G10925	239.2511	-0.63501	0.171728	-3.69776	0.000218	0.001882	7.706019	7.347361	7.512729	8.076669	8.376102	8.154892
AT5G13710	1850.137	-0.63466	0.136526	-4.6486	3.34E-06	4.20E-05	10.64219	10.32094	10.4961	11.1906	11.2542	10.96971
AT2G18280	1693.913	-0.63457	0.137965	-4.59951	4.23E-06	5.22E-05	10.48362	10.18758	10.41063	11.11597	11.0871	10.82609
AT4G14270	1026.704	-0.63431	0.144626	-4.38585	1.16E-05	0.000132	9.534372	9.579034	9.819202	10.48097	10.18698	10.18187
AT1G56700	322.3178	-0.63426	0.148732	-4.26445	2.00E-05	0.000218	8.087616	7.885438	7.990057	8.607986	8.48767	8.741614
AT4G16330	305.4474	-0.63354	0.157073	-4.03341	5.50E-05	0.000549	7.996873	7.776861	7.909839	8.672529	8.571102	8.388339
AT5G08350	115.9263	-0.63315	0.195977	-3.23072	0.001235	0.008608	6.613341	6.421295	6.507039	7.27173	7.072174	7.10747
AT1G21780	816.4969	-0.63302	0.143777	-4.40278	1.07E-05	0.000123	9.455637	9.128367	9.327998	9.994019	10.0913	9.797374
AT5G17860	126.3047	-0.63282	0.196358	-3.2228	0.001269	0.008816	6.451914	6.773485	6.645579	7.203473	7.284819	7.352617
AT1G15690	5670.229	-0.63256	0.11622	-5.44283	5.24E-08	8.76E-07	12.12526	12.02065	12.18511	12.71027	12.90357	12.64029
AT4G32040	452.5519	-0.63255	0.152985	-4.1347	3.55E-05	0.000367	8.541342	8.338437	8.457965	9.083521	9.350505	8.90118
AT5G49640	310.5349	-0.63191	0.151904	-4.15994	3.18E-05	0.000332	8.057062	7.786777	7.89255	8.49355	8.669033	8.558274
AT2G27500	615.8677	-0.63186	0.134776	-4.68821	2.76E-06	3.50E-05	9.008635	8.743336	8.957596	9.477164	9.628833	9.56222
AT3G48150	217.1124	-0.63179	0.177143	-3.56653	0.000362	0.002936	7.640086	7.269354	7.271923	8.057462	8.095267	8.025114
AT1G61667	191.076	-0.63172	0.198562	-3.18145	0.001465	0.009959	7.190827	7.006304	7.49451	7.879329	7.594963	8.069683
AT4G30910	229.5245	-0.63134	0.184225	-3.42702	0.00061	0.004682	7.655001	7.351821	7.438429	8.348327	8.12318	7.907267
AT1G06450	126.8857	-0.63092	0.195275	-3.23094	0.001234	0.008606	6.628533	6.705416	6.525102	7.082003	7.42597	7.361607
AT5G63860	816.8875	-0.63046	0.134265	-4.69567	2.66E-06	3.38E-05	9.264654	9.327175	9.362312	10.11042	9.981227	9.773996
AT4G16190	3564.254	-0.63013	0.107972	-5.83605	5.35E-09	1.01E-07	11.41821	11.38735	11.52843	12.16097	12.10263	11.98179
AT5G08510	218.4538	-0.62936	0.19454	-3.2351	0.001216	0.008496	7.609787	7.231133	7.349997	8.233219	8.177436	7.765446
AT5G18310	226.918	-0.62927	0.17182	-3.66241	0.00025	0.002124	7.666086	7.387012	7.365117	8.031448	8.066803	8.24023
AT5G63260	194.2875	-0.62923	0.194627	-3.23298	0.001225	0.008552	7.250962	7.191871	7.18938	7.89384	8.255176	7.522564
AT5G14240	675.6385	-0.62923	0.158867	-3.96071	7.47E-05	0.000726	9.084961	8.89619	9.122387	9.826374	9.820033	9.390583
AT3G02340	314.6751	-0.62885	0.166307	-3.78127	0.000156	0.001401	8.128263	7.888511	7.802856	8.750346	8.550692	8.444419

AT5G43460	710.1683	-0.6283	0.150243	-4.1819	2.89E-05	0.000305	9.299479	8.917407	9.116442	9.898308	9.723045	9.641866
AT1G26920	740.6181	-0.62828	0.160289	-3.91964	8.87E-05	0.00085	9.202563	9.003543	9.316376	10.05662	9.75015	9.613923
AT1G67910	203.6076	-0.62714	0.193331	-3.24385	0.001179	0.008284	7.260746	7.115245	7.517248	8.108124	8.066803	7.703156
AT4G25670	317.3685	-0.62713	0.145825	-4.30055	1.70E-05	0.000188	7.927936	8.048173	7.943807	8.594723	8.444073	8.717307
AT1G63800	721.915	-0.62654	0.128603	-4.8719	1.11E-06	1.52E-05	9.125683	9.086164	9.218483	9.896507	9.785514	9.651061
AT5G14930	301.0826	-0.62633	0.178589	-3.5071	0.000453	0.003593	7.934063	7.885438	7.828538	8.221762	8.508985	8.785697
AT3G12490	569.5047	-0.62605	0.149839	-4.17816	2.94E-05	0.000309	8.845937	8.66844	8.913708	9.636654	9.22732	9.405874
AT5G44020	4933.51	-0.62595	0.172003	-3.63916	0.000274	0.002297	12.19946	11.59794	11.86972	12.65544	12.67163	12.31222
AT1G13260	981.6462	-0.62568	0.146215	-4.27918	1.88E-05	0.000205	9.793885	9.341822	9.586363	10.16976	10.24529	10.25847
AT5G08520	595.1314	-0.62495	0.176557	-3.53966	0.000401	0.003217	9.045889	8.746726	8.769633	9.752656	9.528036	9.183111
AT5G24060	242.3767	-0.62431	0.175157	-3.56427	0.000365	0.002958	7.43935	7.786777	7.452655	8.316365	8.229725	8.086048
AT3G55840	325.7158	-0.62363	0.194112	-3.21276	0.001315	0.009081	7.982165	7.83854	8.187268	8.469531	8.352714	8.982103
AT2G42790	629.7546	-0.62331	0.132084	-4.71906	2.37E-06	3.05E-05	8.920349	8.909865	8.992095	9.636654	9.704689	9.408045
AT5G38860	138.6312	-0.62309	0.195782	-3.18259	0.00146	0.00993	6.799356	6.604377	6.895141	7.571623	7.380437	7.259495
AT4G01000	1096.534	-0.62273	0.125389	-4.9664	6.82E-07	9.63E-06	9.787132	9.812931	9.637073	10.47736	10.41861	10.24274
AT2G20120	553.114	-0.62161	0.152436	-4.07784	4.55E-05	0.000461	8.858877	8.56276	8.814386	9.415642	9.569204	9.203255
AT2G16600	2317.215	-0.62131	0.101289	-6.13402	8.57E-10	1.78E-08	10.8521	10.79012	10.85054	11.38803	11.46791	11.5172
AT5G01240	594.8282	-0.62074	0.173611	-3.57547	0.00035	0.002849	9.131026	8.564682	8.832631	9.595078	9.569204	9.336894
AT1G05277	126.8666	-0.61914	0.188922	-3.27721	0.001048	0.007489	6.636069	6.589348	6.620596	7.203473	7.51294	7.159945
AT5G57660	5397.023	-0.61895	0.128454	-4.81844	1.45E-06	1.94E-05	12.15048	11.90269	12.08269	12.77686	12.75204	12.49159
AT5G02290	667.9285	-0.61656	0.139437	-4.42174	9.79E-06	0.000113	9.193652	8.838567	9.061808	9.693749	9.657748	9.64371
AT1G27300	323.7188	-0.6164	0.14448	-4.2663	1.99E-05	0.000216	7.934063	7.951575	8.114572	8.689264	8.550692	8.612001
AT1G43700	705.1634	-0.61623	0.148415	-4.15207	3.29E-05	0.000343	9.325349	8.945711	9.035271	9.788045	9.820033	9.625165
AT3G23750	993.8901	-0.61512	0.122841	-5.00741	5.52E-07	7.93E-06	9.499679	9.740801	9.592801	10.19342	10.21319	10.29881
AT5G43440	382.2826	-0.61429	0.155469	-3.95118	7.78E-05	0.000754	8.167796	8.148993	8.412597	9.018792	8.669033	8.850992
AT3G13670	859.2727	-0.61408	0.116249	-5.28241	1.27E-07	2.02E-06	9.44495	9.423538	9.324134	9.970252	10.11929	9.996634
AT2G44080	292.9636	-0.61308	0.167377	-3.66284	0.000249	0.002121	7.861981	7.630369	7.986802	8.343049	8.687846	8.418806
AT5G18110	1084.038	-0.61267	0.120962	-5.06498	4.08E-07	5.98E-06	9.799769	9.6138	9.792421	10.41459	10.40162	10.26567
AT2G26430	786.4516	-0.61247	0.147612	-4.14918	3.34E-05	0.000347	9.359894	9.179442	9.252761	9.966824	10.0771	9.638171
AT5G66880	460.3287	-0.61225	0.129831	-4.71573	2.41E-06	3.10E-05	8.590658	8.465281	8.462658	9.178441	9.106676	9.091519
AT4G02510	4892.57	-0.61218	0.167396	-3.6571	0.000255	0.002163	11.99267	12.05473	11.63727	12.25038	12.78627	12.52898
AT4G15080	250.1298	-0.61184	0.158839	-3.85197	0.000117	0.001085	7.723812	7.585629	7.521753	8.278159	8.376102	8.112917
AT4G23060	538.6723	-0.61103	0.16699	-3.6591	0.000253	0.002149	8.594531	8.623039	8.965885	9.570448	9.161545	9.285859

AT3G21330	647.4827	-0.61076	0.161918	-3.77202	0.000162	0.00145	9.066952	8.893133	9.016241	9.86002	9.619065	9.334613
AT1G53190	267.1246	-0.61024	0.187967	-3.24651	0.001168	0.008216	7.87159	7.450767	7.734639	8.348327	8.611075	8.075159
AT3G48990	3212.081	-0.60995	0.131834	-4.62667	3.72E-06	4.64E-05	11.37157	11.14775	11.38033	11.92128	12.09031	11.75453
AT5G15260	159.3319	-0.60848	0.177105	-3.43569	0.000591	0.004551	7.084671	6.838488	6.99572	7.580621	7.63429	7.600575
AT3G62720	557.2982	-0.60826	0.153075	-3.97361	7.08E-05	0.00069	8.729231	8.796652	8.799622	9.117922	9.507003	9.554401
AT3G17810	468.4297	-0.60752	0.125293	-4.84879	1.24E-06	1.69E-05	8.531273	8.505867	8.562234	9.184356	9.161545	9.104965
AT2G23320	443.9675	-0.60617	0.188119	-3.22227	0.001272	0.008828	8.698967	8.171874	8.439036	8.827962	9.049638	9.309281
AT5G02970	251.8743	-0.6056	0.170853	-3.54456	0.000393	0.003166	7.636334	7.812891	7.40468	8.186833	8.376102	8.230449
AT1G47410	161.2056	-0.60515	0.173157	-3.49479	0.000474	0.00374	6.987859	7.078044	6.936218	7.562569	7.594963	7.68894
AT3G21870	151.9169	-0.60461	0.183033	-3.30326	0.000956	0.006912	6.97603	6.806353	6.859993	7.459032	7.78168	7.388246
AT5G44250	639.3303	-0.6045	0.15545	-3.8887	0.000101	0.000952	9.135021	8.783507	9.014644	9.789986	9.507003	9.469628
AT1G76580	237.5034	-0.6042	0.174781	-3.45689	0.000546	0.00424	7.359654	7.786777	7.489919	8.169047	8.150563	8.200699
AT4G32030	281.6335	-0.60373	0.145688	-4.14398	3.41E-05	0.000354	7.832764	7.719346	7.860899	8.39498	8.399116	8.452856
AT3G57340	716.0411	-0.60353	0.144621	-4.17315	3.00E-05	0.000316	9.281571	8.974917	9.134203	9.726546	9.942585	9.613923
AT4G28300	461.9184	-0.60344	0.165946	-3.63636	0.000277	0.002316	8.604169	8.340682	8.517822	9.022099	9.442	8.913459
AT3G51840	3289.165	-0.60297	0.120873	-4.98848	6.09E-07	8.67E-06	11.30013	11.35189	11.37505	11.94237	12.12869	11.78784
AT1G06760	1867.741	-0.60243	0.15583	-3.86591	0.000111	0.001032	10.24942	10.51983	10.7662	11.27201	11.12901	11.01742
AT4G32150	1262.79	-0.6021	0.156787	-3.84026	0.000123	0.001133	10.14633	9.682686	10.00523	10.53982	10.75824	10.4296
AT5G27520	141.8532	-0.60209	0.189187	-3.18252	0.00146	0.00993	6.819354	6.88846	6.670136	7.478418	7.594963	7.249844
AT2G40300	290.3003	-0.60192	0.18078	-3.32959	0.00087	0.006395	7.782716	7.593183	8.075271	8.479187	8.630654	8.283446
AT1G45050	164.1882	-0.60146	0.18539	-3.24427	0.001178	0.008276	7.165011	6.948585	6.989228	7.842399	7.51294	7.530557
AT2G44410	540.9286	-0.6002	0.134046	-4.47756	7.55E-06	8.92E-05	8.798031	8.587553	8.841668	9.372256	9.33865	9.350505
AT1G29400	2183.914	-0.59996	0.122749	-4.88768	1.02E-06	1.41E-05	10.74596	10.77898	10.74324	11.50312	11.40108	11.17616
AT1G17080	353.7221	-0.59971	0.163378	-3.67069	0.000242	0.002065	8.159976	7.882358	8.330241	8.777989	8.687846	8.758729
AT4G18950	345.1987	-0.59904	0.171689	-3.48913	0.000485	0.003813	8.193563	8.048173	8.047422	8.440172	8.687846	8.952658
AT1G14740	1472.762	-0.59859	0.133205	-4.49377	7.00E-06	8.34E-05	10.38251	10.11794	10.03862	10.83124	10.84879	10.71298
AT3G05900	3686.26	-0.5948	0.144009	-4.13032	3.62E-05	0.000374	11.6086	11.47352	11.4458	12.20854	12.28821	11.82691
AT1G17290	1245.892	-0.59443	0.105572	-5.6306	1.80E-08	3.16E-07	9.959975	9.907607	9.98253	10.56043	10.5984	10.50456
AT4G13270	187.8751	-0.5941	0.163577	-3.63193	0.000281	0.002353	7.289704	7.181886	7.200663	7.773445	7.850087	7.876237
AT3G07350	936.9379	-0.59205	0.157063	-3.76948	0.000164	0.001463	9.621424	9.335081	9.622466	10.23105	10.30744	9.871625
AT5G14780	4405.116	-0.59173	0.100844	-5.86784	4.42E-09	8.46E-08	11.7954	11.72693	11.80235	12.43565	12.40082	12.27999
AT1G54410	3042.821	-0.59147	0.15806	-3.74208	0.000183	0.001611	11.06807	11.41052	11.21179	12.07245	11.84421	11.56906
AT1G21760	397.1087	-0.59099	0.152438	-3.87691	0.000106	0.000992	8.479848	8.179421	8.240166	9.018792	8.880053	8.812171

AT5G44580	604.8152	-0.59092	0.152122	-3.88448	0.000103	0.000967	9.04022	8.686208	8.99047	9.660189	9.430875	9.42744
AT2G40830	267.8987	-0.59058	0.169002	-3.49454	0.000475	0.003742	7.874778	7.574224	7.675219	8.192714	8.591227	8.25478
AT4G12420	2257.464	-0.59037	0.1071	-5.51234	3.54E-08	6.01E-07	10.85008	10.72628	10.84875	11.41217	11.48684	11.32836
AT3G05905	3680.028	-0.58972	0.144088	-4.09277	4.26E-05	0.000435	11.60908	11.47403	11.44669	12.19908	12.28821	11.82285
AT4G30350	252.1421	-0.58878	0.176411	-3.33754	0.000845	0.006232	7.590521	7.763533	7.591983	8.004957	8.255176	8.46542
AT5G01155	5433.877	-0.58871	0.161802	-3.63844	0.000274	0.002303	12.28044	11.87293	12.04719	12.81071	12.36035	12.81551
AT5G62720	412.0422	-0.58858	0.173454	-3.39328	0.000691	0.005205	8.359814	8.120526	8.579623	9.163548	8.795846	8.879437
AT2G01100	614.8591	-0.58827	0.146853	-4.00584	6.18E-05	0.000611	9.033102	8.958927	8.767738	9.54539	9.713896	9.345982
AT4G25170	738.7387	-0.58703	0.126012	-4.65849	3.19E-06	4.02E-05	9.313647	9.098159	9.171951	9.797723	9.878522	9.72433
AT2G44490	948.5585	-0.58663	0.115727	-5.06904	4.00E-07	5.86E-06	9.601352	9.532647	9.534921	10.06306	10.2767	10.13938
AT4G37300	3131.573	-0.58632	0.139229	-4.21123	2.54E-05	0.000271	11.3184	11.15638	11.36663	12.05682	11.91379	11.64374
AT2G01650	218.0144	-0.58618	0.171647	-3.41502	0.000638	0.00485	7.555181	7.288092	7.399794	7.971143	8.280186	7.901114
AT1G13960	416.4745	-0.58599	0.155825	-3.76056	0.00017	0.001509	8.525198	8.315788	8.259177	9.105507	9.02025	8.785697
AT2G08865	209.4459	-0.58583	0.162532	-3.60441	0.000313	0.002583	7.345933	7.404291	7.334716	7.915337	8.203818	7.863636
AT3G12290	900.1861	-0.58472	0.121795	-4.80086	1.58E-06	2.09E-05	9.527297	9.370678	9.550437	10.10576	10.15353	9.989402
AT3G22440	2248.956	-0.58463	0.119687	-4.88465	1.04E-06	1.43E-05	10.94149	10.70901	10.76193	11.45561	11.45699	11.28943
AT3G03105	2826.765	-0.58458	0.168498	-3.46932	0.000522	0.004068	11.32279	10.96514	11.08853	11.96746	11.78865	11.39117
AT5G14840	162.0855	-0.5844	0.179939	-3.24777	0.001163	0.008188	6.993737	6.942684	7.125699	7.488014	7.594963	7.751835
AT3G30180	865.0481	-0.58421	0.146226	-3.99522	6.46E-05	0.000636	9.54642	9.365173	9.381028	10.24668	9.934731	9.858983
AT5G32440	689.9945	-0.58365	0.150428	-3.87993	0.000104	0.000982	9.14562	8.956	9.170518	9.74867	9.886687	9.450796
AT5G53550	512.3018	-0.58291	0.178356	-3.26822	0.001082	0.007699	8.918805	8.507867	8.537977	8.985305	9.385499	9.418852
AT1G57680	1066.947	-0.58282	0.141099	-4.13056	3.62E-05	0.000373	9.683658	9.65137	9.828323	10.17125	10.57332	10.22315
AT1G28200	250.0834	-0.58193	0.177401	-3.28033	0.001037	0.007417	7.609787	7.391351	7.860899	8.163069	8.352714	8.215651
AT3G23490	884.4314	-0.58103	0.128839	-4.50977	6.49E-06	7.77E-05	9.539405	9.352989	9.498816	10.1772	10.04098	9.936235
AT3G57420	228.5659	-0.58052	0.152932	-3.79591	0.000147	0.001327	7.578837	7.511824	7.480693	8.120516	8.037767	8.149711
AT4G34120	344.1293	-0.58026	0.171608	-3.38134	0.000721	0.005407	8.098567	8.014813	8.161518	8.364046	8.846958	8.850992
AT5G60860	565.5801	-0.57962	0.151372	-3.82914	0.000129	0.001179	8.789609	8.617493	9.009841	9.440566	9.507003	9.290574
AT2G02070	539.3592	-0.57909	0.149172	-3.88205	0.000104	0.000975	8.716846	8.621193	8.879016	9.403016	9.485659	9.136733
AT1G64810	246.387	-0.57873	0.153413	-3.77235	0.000162	0.001449	7.602111	7.60444	7.687301	8.327098	8.150563	8.149711
AT1G08930	1566.698	-0.57862	0.131551	-4.39843	1.09E-05	0.000125	10.42881	10.10082	10.3243	10.78528	10.87365	10.97044
AT5G47860	407.9607	-0.57826	0.147753	-3.91368	9.09E-05	0.000869	8.329921	8.278792	8.38319	8.905201	9.174942	8.748484
AT4G33300	859.0197	-0.57737	0.165749	-3.48344	0.000495	0.003881	9.492431	9.296271	9.469967	9.695821	10.01886	10.27045
AT2G44520	181.069	-0.57719	0.169463	-3.40599	0.000659	0.004996	7.265613	7.050872	7.211858	7.725575	7.816289	7.785626

AT1G66410	826.6735	-0.57701	0.113661	-5.07657	3.84E-07	5.64E-06	9.358756	9.366276	9.397055	10.03059	9.918893	9.908899
AT3G51550	2611.986	-0.57665	0.12418	-4.64365	3.42E-06	4.29E-05	11.01378	10.95565	11.10214	11.55254	11.80166	11.47795
AT2G22660	897.059	-0.57578	0.127862	-4.50314	6.70E-06	8.00E-05	9.427684	9.550218	9.496529	10.22962	10.00392	9.966014
AT5G01950	158.8947	-0.57563	0.176363	-3.26389	0.001099	0.00779	6.999592	7.050872	6.874155	7.507017	7.74622	7.530557
AT4G28650	279.0215	-0.57461	0.172518	-3.33071	0.000866	0.006371	7.751831	7.809652	7.846606	8.61676	8.399116	8.128801
AT4G23680	312.4475	-0.57459	0.166726	-3.44634	0.000568	0.004391	8.017217	8.037138	7.765357	8.498306	8.813085	8.366176
AT5G52920	3170.927	-0.57396	0.139844	-4.1043	4.06E-05	0.000415	11.34796	11.24356	11.33306	12.09696	11.91379	11.63542
AT5G59440	291.5769	-0.57392	0.168345	-3.40922	0.000651	0.004941	7.949268	7.733087	7.83217	8.364046	8.760736	8.249946
AT4G29510	305.1768	-0.57388	0.143425	-4.00126	6.30E-05	0.000621	7.940164	7.860616	8.006222	8.464679	8.529989	8.566073
AT5G01720	361.5204	-0.57331	0.137737	-4.16239	3.15E-05	0.000329	8.128263	8.125744	8.272603	8.758298	8.813085	8.727774
AT4G29900	1306.413	-0.57297	0.149954	-3.82095	0.000133	0.001213	10.2315	9.97131	9.847292	10.65185	10.76268	10.42103
AT3G20290	581.2696	-0.57286	0.13244	-4.32542	1.52E-05	0.000169	8.87966	8.755168	8.935819	9.48916	9.538439	9.318544
AT1G14400	1413.801	-0.57285	0.147532	-3.88293	0.000103	0.000972	10.22714	9.913653	10.27044	10.89575	10.68989	10.58257
AT2G17380	347.9008	-0.57271	0.149506	-3.83068	0.000128	0.001174	8.193563	7.977781	8.158628	8.722162	8.846958	8.569957
AT1G51940	636.0092	-0.57235	0.151296	-3.78299	0.000155	0.001393	8.781136	8.944235	9.21987	9.61711	9.589355	9.526693
AT3G09740	1166.978	-0.5699	0.129837	-4.38935	1.14E-05	0.00013	9.913443	9.735691	9.940247	10.47976	10.57837	10.28942
AT3G23620	383.8856	-0.56954	0.147973	-3.84896	0.000119	0.001097	8.382395	8.285802	8.155732	8.781895	8.742856	8.993715
AT5G45360	583.9443	-0.56878	0.140035	-4.0617	4.87E-05	0.000492	8.962922	8.766904	8.852438	9.477164	9.609229	9.274004
AT1G67530	236.8111	-0.56864	0.156557	-3.63218	0.000281	0.002352	7.643829	7.555012	7.512729	8.238914	8.177436	8.047571
AT1G61250	795.9342	-0.56864	0.133637	-4.25508	2.09E-05	0.000226	9.436343	9.124463	9.378546	9.86002	9.926834	9.910431
AT1G64040	760.9545	-0.56832	0.123654	-4.59608	4.31E-06	5.30E-05	9.355336	9.215372	9.174815	9.824481	9.926834	9.755358
AT4G36050	302.3172	-0.56802	0.156517	-3.62909	0.000284	0.002373	8.059867	7.819346	7.850192	8.5358	8.630654	8.361702
AT1G15350	289.2876	-0.56798	0.170874	-3.32395	0.000888	0.00651	7.993944	7.637693	7.940446	8.603578	8.376102	8.325404
AT4G02610	197.831	-0.56766	0.169332	-3.35231	0.000801	0.005947	7.350521	7.301987	7.355055	8.089333	7.672573	7.857294
AT3G53120	179.853	-0.56738	0.175616	-3.2308	0.001234	0.008608	7.22621	7.061803	7.206271	7.571623	7.883112	7.831643
AT3G49800	482.9162	-0.56733	0.136309	-4.16206	3.15E-05	0.000329	8.684503	8.457025	8.669657	9.196113	9.106676	9.21818
AT3G54850	322.7541	-0.56621	0.161284	-3.5106	0.000447	0.003551	8.206275	7.963281	7.896025	8.750346	8.48767	8.530642
AT3G60300	935.9994	-0.56438	0.151696	-3.72046	0.000199	0.001739	9.711568	9.451858	9.486194	10.32506	10.13309	9.907365
AT1G09363	315.7915	-0.56366	0.143196	-3.93632	8.27E-05	0.000797	7.958314	7.969098	8.006222	8.563295	8.706416	8.444419
AT1G22530	2145.751	-0.5613	0.105347	-5.32815	9.92E-08	1.59E-06	10.67548	10.73655	10.85054	11.31846	11.34589	11.30816
AT1G24160	1014.052	-0.56068	0.147666	-3.79696	0.000146	0.001322	9.595566	9.816162	9.599209	10.45061	10.20668	10.04766
AT3G51370	821.4271	-0.56013	0.137055	-4.08694	4.37E-05	0.000445	9.390293	9.389237	9.292848	9.8929	10.167	9.757062
AT3G58680	1814.132	-0.55982	0.133091	-4.20626	2.60E-05	0.000276	10.52277	10.38436	10.6294	11.26085	10.99191	10.97191

AT1G02305	1301.21	-0.55944	0.127853	-4.37568	1.21E-05	0.000138	10.03672	9.93981	10.11738	10.71756	10.64727	10.4328
AT3G61790	278.8048	-0.55868	0.172546	-3.23785	0.001204	0.008428	7.982165	7.539457	7.85377	8.305552	8.508985	8.357215
AT5G04455	324.1554	-0.55797	0.169691	-3.28815	0.001008	0.007237	8.104012	7.882358	8.015834	8.384742	8.928315	8.490225
AT5G04170	550.3806	-0.55797	0.145968	-3.82253	0.000132	0.001207	8.818048	8.69501	8.812549	9.242206	9.628833	9.21818
AT4G02725	739.9718	-0.55775	0.123538	-4.51483	6.34E-06	7.60E-05	9.249994	9.164308	9.278278	9.896507	9.648174	9.792396
AT1G48840	232.4125	-0.55729	0.16384	-3.40144	0.00067	0.005071	7.640086	7.391351	7.570401	8.101887	8.280186	8.008039
AT5G25280	3268.796	-0.55713	0.128269	-4.34341	1.40E-05	0.000157	11.47131	11.21294	11.39391	12.02915	11.98966	11.76007
AT1G67325	318.0909	-0.5569	0.15457	-3.60293	0.000315	0.002595	8.095837	7.860616	8.03487	8.664088	8.630654	8.427394
AT2G20960	843.88	-0.5567	0.124155	-4.48391	7.33E-06	8.69E-05	9.471521	9.362966	9.393372	9.833919	10.03364	10.06014
AT2G39950	337.5665	-0.55644	0.143775	-3.87021	0.000109	0.001017	8.042958	8.061848	8.206983	8.709913	8.508985	8.720804
AT5G04445	324.3373	-0.55602	0.169849	-3.27362	0.001062	0.007568	8.106727	7.882358	8.019024	8.384742	8.928315	8.490225
AT1G30200	407.1518	-0.55411	0.151964	-3.64631	0.000266	0.002243	8.486117	8.223893	8.368259	9.070806	8.912407	8.785697
AT5G39590	1147.339	-0.5509	0.115708	-4.76114	1.93E-06	2.52E-05	9.855709	9.883937	9.817371	10.40702	10.5478	10.30115
AT2G32600	220.92	-0.55057	0.170036	-3.23799	0.001204	0.008426	7.602111	7.29737	7.530721	8.132803	8.066803	7.943643
AT5G57340	342.1659	-0.55045	0.156821	-3.51003	0.000448	0.003558	8.084865	8.22875	8.053657	8.879911	8.529989	8.57383
AT1G72150	11352.2	-0.54963	0.103284	-5.32149	1.03E-07	1.65E-06	13.18654	13.16512	13.14727	13.81145	13.75026	13.59598
AT5G51980	260.0811	-0.54936	0.162236	-3.38618	0.000709	0.005319	7.706019	7.756823	7.74238	8.498306	8.095267	8.190645
AT3G23030	4037.015	-0.54858	0.144135	-3.80601	0.000141	0.00128	11.8682	11.56014	11.56545	12.37067	12.28821	12.016
AT1G29970	600.4693	-0.54749	0.127826	-4.28309	1.84E-05	0.000202	9.010086	8.804808	8.947586	9.477164	9.548767	9.431715
AT4G05070	1580.195	-0.54711	0.150452	-3.63642	0.000276	0.002316	10.50898	10.14518	10.28641	11.07517	10.83198	10.70417
AT2G20670	7875.511	-0.54708	0.147688	-3.7043	0.000212	0.00184	12.80139	12.51835	12.57595	13.41618	13.1751	12.95995
AT5G16880	1485.047	-0.54647	0.12487	-4.37628	1.21E-05	0.000137	10.38531	10.14518	10.16423	10.89214	10.72219	10.73908
AT2G17520	512.4988	-0.54641	0.154778	-3.53026	0.000415	0.003321	8.862094	8.606338	8.568779	9.247865	9.453039	9.069741
AT1G76150	519.3918	-0.54622	0.13067	-4.18019	2.91E-05	0.000307	8.821357	8.61564	8.699788	9.259118	9.33865	9.232952
AT5G01715	229.8956	-0.54611	0.159562	-3.42253	0.00062	0.004752	7.518954	7.471408	7.642499	8.19857	8.095267	8.008039
AT3G55770	1914.824	-0.54562	0.139889	-3.90036	9.60E-05	0.000913	10.70579	10.43094	10.65064	11.34511	11.09064	11.00456
AT2G36930	730.3492	-0.54559	0.129621	-4.20913	2.56E-05	0.000273	9.226473	9.151575	9.233666	9.588402	9.878522	9.825262
AT1G11660	385.869	-0.54499	0.140914	-3.86754	0.00011	0.001027	8.251135	8.262304	8.330241	8.89802	8.959616	8.689016
AT2G30260	436.2898	-0.54448	0.163046	-3.3394	0.00084	0.006196	8.586774	8.281133	8.457965	8.922997	9.302486	8.844594
AT1G72430	1129.293	-0.54435	0.150651	-3.61332	0.000302	0.002506	9.921175	9.54341	9.998779	10.33706	10.5007	10.34487
AT5G47560	1593.938	-0.54381	0.127942	-4.2504	2.13E-05	0.000231	10.39256	10.30378	10.30548	10.68038	10.96902	11.00169
AT1G80180	1933.164	-0.54323	0.13379	-4.06031	4.90E-05	0.000494	10.74335	10.42989	10.65474	11.2961	11.1529	11.04139
AT4G09695	437.8439	-0.54241	0.144963	-3.74168	0.000183	0.001613	8.557307	8.306627	8.511041	8.975105	9.188216	8.931683

AT1G19570	1021.196	-0.54202	0.141246	-3.83742	0.000124	0.001146	9.6589	9.678254	9.711917	10.14116	10.51657	10.06702
AT3G08760	282.4643	-0.54198	0.145614	-3.72201	0.000198	0.001731	7.906284	7.780174	7.85377	8.353586	8.376102	8.452856
AT4G05050	2502.01	-0.54052	0.12116	-4.46123	8.15E-06	9.56E-05	10.98825	10.84017	11.12156	11.44395	11.61277	11.55443
AT4G38250	441.6793	-0.54003	0.144839	-3.72846	0.000193	0.00169	8.580928	8.315788	8.522325	8.988689	9.188216	8.952658
AT5G05100	758.1604	-0.53982	0.13766	-3.92144	8.80E-05	0.000845	9.43202	9.071367	9.274279	9.813074	9.836987	9.802335
AT1G63860	359.0313	-0.53934	0.156121	-3.4546	0.000551	0.004272	8.198661	8.059123	8.30171	8.890804	8.742856	8.57383
AT5G19780	1895.608	-0.53909	0.126532	-4.26053	2.04E-05	0.000222	10.51103	10.46942	10.76715	11.17435	11.17973	11.05601
AT1G74900	240.6176	-0.53905	0.159025	-3.38972	0.0007	0.005261	7.709596	7.593183	7.539633	8.278159	8.150563	8.058669
AT5G04530	265.0475	-0.53902	0.162305	-3.32103	0.000897	0.006566	7.799592	7.551139	7.878569	8.332435	8.352714	8.235348
AT2G19470	291.5307	-0.53883	0.14236	-3.78496	0.000154	0.001384	7.918697	7.891577	7.899491	8.507771	8.328941	8.457056
AT1G65720	679.913	-0.53848	0.164483	-3.27378	0.001061	0.007567	9.2327	8.806434	9.21987	9.540787	9.87031	9.568058
AT5G02810	1276.95	-0.53811	0.133145	-4.04155	5.31E-05	0.000532	9.983798	10.14389	9.905333	10.58746	10.71304	10.39609
AT1G70090	893.5615	-0.53734	0.135937	-3.95289	7.72E-05	0.000749	9.623321	9.350763	9.533806	9.928575	10.04098	10.17551
AT3G10770	1379.704	-0.53716	0.145121	-3.70144	0.000214	0.001859	10.27378	9.930896	10.1599	10.77552	10.77153	10.47691
AT1G07230	522.8144	-0.53704	0.139704	-3.84415	0.000121	0.001116	8.704354	8.684441	8.799622	9.403016	9.326696	9.102286
AT2G45980	1080.78	-0.5357	0.148842	-3.59909	0.000319	0.00263	9.944125	9.563737	9.813702	10.4965	10.25163	10.21076
AT2G30700	235.7365	-0.53562	0.156637	-3.41948	0.000627	0.00479	7.590521	7.503831	7.604778	8.057462	8.328941	8.041989
AT3G60600	1048.275	-0.53552	0.140471	-3.81233	0.000138	0.001252	9.872556	9.53657	9.781192	10.40195	10.2767	10.15756
AT3G53670	187.392	-0.53547	0.166738	-3.21142	0.001321	0.009116	7.221208	7.311177	7.23399	7.901041	7.816289	7.696066
AT3G52500	634.9388	-0.53488	0.141024	-3.79279	0.000149	0.001342	8.926509	8.920412	9.157549	9.413126	9.713896	9.554401
AT3G12570	636.5053	-0.53474	0.141902	-3.76836	0.000164	0.001469	9.110885	8.838567	9.06026	9.522227	9.732137	9.440226
AT4G39140	223.771	-0.53408	0.16227	-3.2913	0.000997	0.00716	7.43935	7.475501	7.544069	7.936518	8.304769	7.961492
AT5G10480	418.8568	-0.5334	0.156001	-3.41924	0.000628	0.004793	8.500639	8.297408	8.464999	9.175475	8.830121	8.831712
AT5G01800	522.5541	-0.53266	0.164204	-3.24392	0.001179	0.008284	8.76576	8.493811	8.870211	9.139393	9.538439	9.154947
AT4G16150	956.1051	-0.53186	0.143312	-3.71122	0.000206	0.001794	9.593632	9.69239	9.532691	10.35945	10.09835	9.954176
AT1G73680	267.8501	-0.53099	0.155619	-3.4121	0.000645	0.004895	7.723812	7.828975	7.734639	8.38987	8.42177	8.144512
AT1G70590	258.8862	-0.53073	0.152601	-3.47787	0.000505	0.003954	7.802944	7.644979	7.746235	8.215999	8.203818	8.361702
AT1G14510	436.5933	-0.52942	0.131716	-4.01941	5.83E-05	0.00058	8.527226	8.419277	8.460313	8.940576	9.134371	8.990821
AT2G46260	742.9196	-0.52903	0.132075	-4.00552	6.19E-05	0.000611	9.361032	9.120547	9.252761	9.896507	9.732137	9.7068
AT5G13570	357.1404	-0.52902	0.158551	-3.3366	0.000848	0.006248	8.308857	8.102113	8.111587	8.816582	8.8636	8.506528
AT1G25682	297.3524	-0.52849	0.163985	-3.22282	0.001269	0.008816	8.017217	7.891577	7.824897	8.576848	8.591227	8.225533
AT3G22480	249.108	-0.52757	0.160294	-3.29129	0.000997	0.00716	7.809624	7.648609	7.609019	8.316365	7.977879	8.273953
AT2G03270	189.2842	-0.52531	0.164161	-3.19995	0.001375	0.009441	7.280116	7.235966	7.339828	7.908207	7.74622	7.772204

AT1G73980	365.5719	-0.52452	0.143888	-3.64531	0.000267	0.002247	8.175575	8.16175	8.296461	8.766207	8.912407	8.615763
AT5G49400	352.9553	-0.52435	0.145664	-3.59969	0.000319	0.002625	8.292259	8.078089	8.129408	8.750346	8.778398	8.608229
AT1G29390	374.4482	-0.52422	0.149765	-3.50026	0.000465	0.003674	8.133597	8.223893	8.410169	8.887182	8.795846	8.696141
AT3G15000	576.59	-0.52367	0.128393	-4.07868	4.53E-05	0.000459	8.821357	8.851222	8.970836	9.319486	9.442	9.482048
AT1G36980	872.9569	-0.52349	0.150062	-3.4885	0.000486	0.003817	9.550414	9.214148	9.650506	10.10264	9.918893	10.0067
AT2G06530	434.6853	-0.52332	0.154848	-3.37955	0.000726	0.005435	8.426522	8.442462	8.488204	8.940576	9.302486	8.782353
AT5G15550	296.1306	-0.52314	0.163407	-3.20145	0.001367	0.009397	7.921783	7.841715	7.937077	8.192714	8.706416	8.473736
AT5G35200	549.1064	-0.52209	0.130819	-3.9909	6.58E-05	0.000646	8.920349	8.783507	8.711666	9.28412	9.430875	9.327748
AT4G01610	800.3308	-0.52068	0.139483	-3.73291	0.000189	0.001663	9.350763	9.47453	9.264904	10.06466	9.686095	9.866897
AT1G20100	419.3049	-0.51986	0.137344	-3.78507	0.000154	0.001384	8.479848	8.384874	8.419856	8.842996	8.944051	9.066996
AT3G10740	1188.018	-0.51966	0.153218	-3.39163	0.000695	0.005229	9.847618	9.964046	9.930962	10.33041	10.76711	10.23787
AT5G23610	497.0995	-0.51795	0.155828	-3.3239	0.000888	0.00651	8.860486	8.525739	8.592528	9.30327	9.265393	9.028
AT4G18710	1867.105	-0.51775	0.136838	-3.78364	0.000155	0.00139	10.64453	10.473	10.60837	11.22472	11.20933	10.86454
AT2G34770	1186.503	-0.51765	0.140579	-3.68226	0.000231	0.001986	9.91886	9.765248	10.08578	10.61838	10.36117	10.35841
AT3G42170	405.6622	-0.51726	0.155831	-3.31936	0.000902	0.006596	8.292259	8.302025	8.520076	9.041779	8.959616	8.710286
AT4G19880	503.8515	-0.51693	0.152056	-3.39958	0.000675	0.005102	8.870105	8.655871	8.560045	9.172502	9.06411	9.38398
AT1G62040	363.554	-0.51634	0.156452	-3.30029	0.000966	0.006978	8.311213	8.050918	8.330241	8.887182	8.550692	8.762127
AT1G69690	297.2576	-0.51559	0.16036	-3.21522	0.001303	0.009024	7.890617	7.844882	8.015834	8.5358	8.611075	8.235348
AT2G45820	1397.485	-0.51499	0.130197	-3.95546	7.64E-05	0.000741	10.29179	10.07475	10.11365	10.80557	10.71762	10.52871
AT4G16760	1009.241	-0.51462	0.130686	-3.93781	8.22E-05	0.000793	9.69993	9.785994	9.570144	10.07106	10.37284	10.21076
AT4G15545	2704.621	-0.51452	0.133406	-3.85677	0.000115	0.001067	11.13609	10.95821	11.23461	11.69348	11.76227	11.4503
AT3G17020	1096.367	-0.51442	0.134317	-3.82988	0.000128	0.001177	9.745932	9.824209	9.893214	10.52475	10.13309	10.31279
AT2G26060	344.2637	-0.51415	0.142242	-3.61461	0.000301	0.002496	8.170394	8.112663	8.123492	8.714008	8.778398	8.526651
AT1G11480	712.8799	-0.51398	0.158463	-3.24352	0.001181	0.008284	9.192375	9.237232	9.119417	9.841425	9.894806	9.386185
AT3G55130	604.4534	-0.51382	0.145202	-3.53867	0.000402	0.003226	9.071128	8.825799	8.982322	9.662309	9.350505	9.392778
AT3G19860	760.3536	-0.51289	0.150009	-3.41904	0.000628	0.004793	9.418973	9.112685	9.316376	9.990648	9.776754	9.64002
AT5G58720	624.2746	-0.51184	0.141081	-3.62797	0.000286	0.002382	9.100026	9.055049	8.816221	9.51989	9.657748	9.405874
AT1G08570	727.7822	-0.51148	0.150004	-3.40978	0.00065	0.004933	9.38583	9.150295	9.128307	9.935605	9.704689	9.573871
AT5G07370	352.4937	-0.51143	0.149599	-3.41864	0.000629	0.004796	8.318257	8.034366	8.204183	8.781895	8.591227	8.710286
AT2G37410	811.7353	-0.51123	0.121124	-4.22072	2.44E-05	0.000261	9.424424	9.311234	9.424981	10.00743	9.84539	9.841418
AT4G38770	2781.078	-0.51098	0.160322	-3.1872	0.001437	0.009794	11.16014	10.92268	11.35089	11.82849	11.77552	11.40319
AT2G45620	1005.473	-0.51019	0.127794	-3.99225	6.54E-05	0.000643	9.73369	9.706388	9.619317	10.2802	10.31955	10.02803
AT3G46000	634.0675	-0.50997	0.13605	-3.74837	0.000178	0.001575	9.058564	8.849646	9.161885	9.577207	9.538439	9.522691

AT5G21160	1044.722	-0.50964	0.138026	-3.69234	0.000222	0.001917	9.854902	9.720252	9.643288	10.345	10.39018	10.05322
AT1G03080	776.9483	-0.50935	0.159803	-3.18733	0.001436	0.009792	9.311296	9.528713	9.078722	9.980486	9.862051	9.665653
AT3G49590	460.5276	-0.50835	0.142296	-3.57252	0.000354	0.002879	8.551341	8.425637	8.681785	9.022099	9.240123	9.0081
AT5G06360	818.5775	-0.50829	0.131106	-3.87694	0.000106	0.000992	9.496577	9.388152	9.307272	9.791924	9.886687	10.04766
AT1G71980	363.7659	-0.50821	0.144867	-3.50813	0.000451	0.003582	8.246219	8.094149	8.299088	8.709913	8.896321	8.649188
AT5G54430	489.903	-0.5079	0.138801	-3.65921	0.000253	0.002149	8.684503	8.670227	8.570954	9.16055	9.362265	9.010959
AT2G42880	878.9631	-0.50743	0.154847	-3.27695	0.001049	0.007494	9.522223	9.415039	9.524858	10.05662	10.25794	9.7068
AT5G64200	376.8853	-0.50677	0.13768	-3.6808	0.000233	0.001993	8.308857	8.16935	8.343025	8.770145	8.8636	8.765518
AT3G07220	413.5727	-0.50626	0.140365	-3.60674	0.00031	0.00256	8.456627	8.327157	8.499668	8.981913	8.742856	9.019505
AT3G61600	1266.04	-0.50519	0.129375	-3.90485	9.43E-05	0.000898	9.983059	9.936845	10.15121	10.65185	10.57332	10.38841
AT5G16160	401.0499	-0.50499	0.146969	-3.43602	0.00059	0.004547	8.329921	8.391387	8.397968	9.057977	8.846958	8.727774
AT2G36900	423.5531	-0.50404	0.151304	-3.33133	0.000864	0.006358	8.512971	8.327157	8.495093	9.111728	8.990252	8.775643
AT3G29240	3138.69	-0.50349	0.131469	-3.82971	0.000128	0.001177	11.50283	11.19195	11.29803	11.9323	11.91379	11.69354
AT3G50830	697.6774	-0.50279	0.142769	-3.52172	0.000429	0.003421	9.179539	9.007788	9.299422	9.517549	9.741171	9.779038
AT2G06950	871.213	-0.50173	0.112583	-4.45651	8.33E-06	9.75E-05	9.428769	9.491808	9.547126	9.988959	10.04098	9.974829
AT5G54170	695.4477	-0.50129	0.117243	-4.27563	1.91E-05	0.000208	9.13635	9.236026	9.14154	9.693749	9.619065	9.703268
AT5G53300	4595.825	-0.50093	0.149117	-3.35928	0.000781	0.00581	12.10198	11.62399	11.89815	12.52634	12.40651	12.25367
AT4G37870	2525.615	-0.49926	0.14054	-3.5524	0.000382	0.003083	11.07986	10.99717	10.99443	11.68776	11.63233	11.25125
AT5G48160	1690.724	-0.49821	0.124259	-4.00944	6.09E-05	0.000603	10.55344	10.44144	10.34287	11.06319	10.98051	10.81222
AT4G34020	353.1608	-0.49781	0.14717	-3.38255	0.000718	0.005385	8.263352	8.164287	8.16728	8.861572	8.611075	8.596854
AT1G80040	514.1982	-0.49711	0.129243	-3.84635	0.00012	0.001107	8.725703	8.666651	8.780952	9.136346	9.362265	9.230501
AT3G21215	321.4539	-0.49693	0.146504	-3.39193	0.000694	0.005225	8.138912	7.989277	7.993304	8.517175	8.724751	8.469584
AT5G21930	1479.334	-0.49652	0.132954	-3.73449	0.000188	0.001654	10.38363	10.17757	10.18355	10.75679	10.93005	10.59215
AT2G02220	331.2593	-0.49593	0.155214	-3.19511	0.001398	0.00957	8.218877	7.969098	8.129408	8.521853	8.508985	8.758729
AT1G62310	447.0967	-0.49573	0.153659	-3.22617	0.001255	0.00873	8.604169	8.579969	8.431873	9.259118	8.896321	8.913459
AT2G42870	1335.114	-0.49562	0.150192	-3.29992	0.000967	0.006983	10.17571	9.876218	10.2475	10.77552	10.61323	10.436
AT3G20770	2805.636	-0.49559	0.113667	-4.35996	1.30E-05	0.000147	11.28314	11.12513	11.12637	11.72375	11.76005	11.5642
AT3G48170	688.3676	-0.49497	0.124882	-3.96349	7.39E-05	0.000718	9.194929	9.065948	9.181948	9.666541	9.76794	9.548508
AT1G18470	514.4232	-0.49462	0.130283	-3.79652	0.000147	0.001324	8.818048	8.673793	8.689814	9.239367	9.33865	9.149766
AT1G78890	279.7751	-0.4939	0.153732	-3.21276	0.001315	0.009081	7.937117	7.701985	7.937077	8.400072	8.328941	8.352713
AT1G30820	895.4557	-0.49358	0.133178	-3.70616	0.00021	0.001828	9.54542	9.607287	9.446465	10.18016	10.04828	9.868475
AT4G32520	561.1562	-0.49303	0.134112	-3.67625	0.000237	0.002025	8.738013	8.87	8.939191	9.308696	9.517558	9.276383
AT4G33430	527.9662	-0.49263	0.14927	-3.30028	0.000966	0.006978	8.858877	8.780202	8.673711	9.064405	9.302486	9.440226

AT1G23870	1041.887	-0.49257	0.134973	-3.64937	0.000263	0.00222	9.874946	9.75434	9.599209	10.24385	10.4073	10.11433
AT1G08510	1594.397	-0.49192	0.122721	-4.00844	6.11E-05	0.000605	10.31604	10.36242	10.40759	10.85554	11.03663	10.70505
AT3G62420	1028.265	-0.48985	0.144029	-3.40106	0.000671	0.005076	9.802283	9.648654	9.770821	10.10576	10.08422	10.47069
AT5G24890	930.6501	-0.48869	0.13155	-3.71487	0.000203	0.001771	9.747672	9.549247	9.478103	10.19635	10.04098	10.02379
AT3G13200	1170.086	-0.48863	0.115381	-4.23493	2.29E-05	0.000246	9.961476	9.84966	9.942769	10.39432	10.54265	10.32777
AT4G29840	607.6334	-0.48756	0.147593	-3.30339	0.000955	0.006912	8.968902	8.836977	9.081776	9.4455	9.695422	9.288218
AT5G59950	462.0895	-0.4872	0.129105	-3.77369	0.000161	0.001442	8.551341	8.576163	8.643025	9.16055	9.035019	9.036445
AT3G26520	20616.48	-0.48719	0.13561	-3.59258	0.000327	0.002689	14.13174	13.83152	14.19716	14.46484	14.70475	14.49415
AT1G72710	1278.78	-0.48676	0.129537	-3.75768	0.000171	0.001522	10.15422	9.919674	10.06278	10.58858	10.66162	10.38951
AT3G03950	269.2634	-0.48657	0.146984	-3.31039	0.000932	0.006784	7.884303	7.783479	7.75008	8.278159	8.328941	8.311553
AT2G22540	761.5579	-0.48639	0.149544	-3.25247	0.001144	0.008072	9.346175	9.113999	9.438148	9.963389	9.785514	9.636321
AT2G03640	1270.826	-0.48615	0.126108	-3.85501	0.000116	0.001073	10.19114	9.974206	9.951978	10.603	10.57837	10.43174
AT3G14010	688.134	-0.48593	0.134514	-3.61248	0.000303	0.002512	9.131026	9.223913	9.089383	9.660189	9.828535	9.47792
AT1G30970	367.7201	-0.48466	0.142327	-3.40529	0.000661	0.005004	8.341492	8.171874	8.277939	8.865258	8.669033	8.69969
AT5G42520	399.7864	-0.48437	0.13807	-3.50811	0.000451	0.003582	8.364359	8.358522	8.395516	8.947549	8.944051	8.727774
AT3G15070	358.8049	-0.48425	0.14377	-3.36822	0.000757	0.005637	8.318257	8.138706	8.209778	8.812769	8.706416	8.623258
AT3G56130	790.4659	-0.48314	0.145731	-3.3153	0.000915	0.006687	9.384712	9.225129	9.475783	10.05501	9.75015	9.722587
AT1G71080	408.9146	-0.48311	0.145999	-3.30901	0.000936	0.006808	8.533293	8.32942	8.319931	8.85417	9.078439	8.795682
AT2G32520	362.1828	-0.48248	0.139245	-3.46496	0.00053	0.004124	8.268211	8.219018	8.23743	8.842996	8.687846	8.641827
AT4G02880	711.0043	-0.48149	0.118374	-4.06748	4.75E-05	0.00048	9.182115	9.209241	9.264904	9.728572	9.589355	9.757062
AT5G12120	880.3994	-0.4808	0.124673	-3.85649	0.000115	0.001068	9.500711	9.544384	9.491945	10.06145	10.11929	9.83981
AT4G17486	291.3442	-0.48044	0.147402	-3.2594	0.001116	0.007897	7.943205	7.812891	7.990057	8.39498	8.508985	8.357215
AT1G73030	729.4919	-0.47901	0.120985	-3.95928	7.52E-05	0.00073	9.216454	9.204317	9.303352	9.730594	9.84539	9.638171
AT1G05840	593.0783	-0.47857	0.126566	-3.78119	0.000156	0.001401	9.027382	8.906837	8.905113	9.48916	9.485659	9.339171
AT5G48385	758.9239	-0.47847	0.12779	-3.7442	0.000181	0.001599	9.416787	9.215372	9.264904	9.828264	9.87031	9.681894
AT4G15470	747.4625	-0.47783	0.120508	-3.96508	7.34E-05	0.000714	9.303034	9.212923	9.315079	9.679162	9.886687	9.751943
AT4G30440	1466.707	-0.47774	0.141968	-3.36511	0.000765	0.005699	10.29119	10.00497	10.44246	10.73479	10.68053	10.80483
AT2G37340	956.303	-0.47672	0.118175	-4.03404	5.48E-05	0.000548	9.61667	9.612871	9.666868	10.02731	10.2767	10.07525
AT1G67480	370.7213	-0.47668	0.13468	-3.53937	0.000401	0.00322	8.246219	8.304328	8.275274	8.824179	8.760736	8.692583
AT1G50480	2268.233	-0.47657	0.11412	-4.17606	2.97E-05	0.000312	10.96113	10.78105	10.90415	11.39059	11.45699	11.25848
AT3G53030	296.9499	-0.47633	0.146048	-3.26148	0.001108	0.007848	7.982165	7.912861	7.920113	8.400072	8.611075	8.334564
AT1G60170	667.689	-0.47633	0.122908	-3.87552	0.000106	0.000997	9.030245	9.130964	9.184791	9.570448	9.704689	9.554401
AT5G60160	545.9354	-0.47593	0.130217	-3.65487	0.000257	0.002179	8.774323	8.783507	8.922253	9.356627	9.385499	9.213222

AT5G52240	765.3199	-0.47563	0.126371	-3.76376	0.000167	0.001491	9.375737	9.210469	9.367326	9.891093	9.81148	9.701499
AT5G18640	467.3192	-0.47467	0.138937	-3.41646	0.000634	0.004827	8.684503	8.535574	8.601068	9.207775	9.106676	8.95563
AT4G12570	425.7663	-0.47441	0.135399	-3.50379	0.000459	0.003633	8.48403	8.469391	8.457965	9.035249	9.02025	8.821974
AT2G20490	321.1946	-0.47403	0.14383	-3.29577	0.000982	0.007065	7.979205	8.107398	8.123492	8.479187	8.550692	8.623258
AT5G65670	1426.402	-0.47386	0.114804	-4.12755	3.67E-05	0.000378	10.15553	10.22878	10.27378	10.80557	10.68053	10.60261
AT3G60340	721.5776	-0.47346	0.148327	-3.19203	0.001413	0.009655	9.216454	9.094172	9.381028	9.883841	9.713896	9.53665
AT3G01910	925.308	-0.47345	0.12132	-3.90248	9.52E-05	0.000906	9.608074	9.579034	9.573403	10.07902	10.21967	9.931715
AT5G19140	5572.71	-0.4734	0.144128	-3.28462	0.001021	0.00732	12.26503	11.96987	12.29364	12.86949	12.58395	12.51796
AT5G58290	986.8152	-0.47272	0.123259	-3.83515	0.000125	0.001156	9.623321	9.641386	9.789622	10.04692	10.21319	10.24031
AT1G18460	758.5087	-0.47202	0.134218	-3.51681	0.000437	0.003478	9.410209	9.316947	9.160441	9.720453	9.950397	9.703268
AT1G10200	622.8458	-0.47157	0.137705	-3.42449	0.000616	0.004723	8.989641	8.947185	9.157549	9.649539	9.350505	9.479985
AT1G50600	640.6198	-0.4701	0.125653	-3.74122	0.000183	0.001615	9.043057	8.977805	9.154652	9.498685	9.648174	9.496403
AT5G16030	6482.018	-0.46998	0.11774	-3.99169	6.56E-05	0.000644	12.36394	12.35731	12.48875	13.01245	12.89348	12.72284
AT1G04430	1594.715	-0.46843	0.127071	-3.68638	0.000227	0.001958	10.4108	10.2312	10.49266	10.96008	10.86954	10.73736
AT3G57410	1785.209	-0.46813	0.117369	-3.98851	6.65E-05	0.000651	10.59318	10.5695	10.46953	11.12369	11.04759	10.88183
AT1G16890	1192.279	-0.46633	0.119413	-3.90515	9.42E-05	0.000897	9.994098	9.918923	9.963614	10.4689	10.55807	10.28588
AT3G07890	310.3084	-0.46509	0.140769	-3.30396	0.000953	0.0069	8.020099	7.971998	8.072203	8.440172	8.550692	8.514611
AT4G34480	465.53	-0.46474	0.135393	-3.43249	0.000598	0.004601	8.580928	8.543394	8.659472	8.978513	9.265393	9.019505
AT5G60340	752.5773	-0.46459	0.141316	-3.28762	0.00101	0.007248	9.479922	9.100811	9.303352	9.830151	9.75015	9.739928
AT1G63500	322.7494	-0.46439	0.140819	-3.29776	0.000975	0.007023	8.057062	8.023225	8.147011	8.488778	8.630654	8.558274
AT1G76160	1116.997	-0.46432	0.128053	-3.62598	0.000288	0.002399	9.984536	9.767753	9.831955	10.26633	10.50601	10.25967
AT3G53990	1133.266	-0.46416	0.115243	-4.02763	5.63E-05	0.000562	9.892358	9.796666	9.975979	10.36597	10.42983	10.29881
AT3G27260	891.5992	-0.46386	0.12102	-3.83293	0.000127	0.001165	9.514066	9.615656	9.494239	10.05662	10.10537	9.895034
AT5G03470	387.8441	-0.46315	0.135765	-3.41139	0.000646	0.004907	8.339185	8.402178	8.345569	8.89802	8.649971	8.866864
AT1G19660	1850.611	-0.46302	0.112852	-4.10291	4.08E-05	0.000418	10.55791	10.63957	10.60201	11.16465	11.0977	10.94076
AT1G36730	1497.155	-0.46212	0.104085	-4.43989	9.00E-06	0.000105	10.27137	10.30205	10.30417	10.71858	10.84879	10.7287
AT5G03340	453.7061	-0.4621	0.141964	-3.25502	0.001134	0.008004	8.510923	8.659473	8.592528	9.199037	8.830121	9.050412
AT5G63195	2433.583	-0.46181	0.120644	-3.82786	0.000129	0.001185	10.99119	10.94093	11.04929	11.56052	11.53957	11.28176
AT1G20330	1696.767	-0.45877	0.132968	-3.45022	0.00056	0.00433	10.52936	10.26233	10.61735	11.01588	10.94577	10.86612
AT1G32130	674.1699	-0.45851	0.123917	-3.70014	0.000215	0.001866	9.102749	9.238437	9.081776	9.623654	9.686095	9.53665
AT3G26030	695.4449	-0.45795	0.13253	-3.45546	0.000549	0.004261	9.273137	9.178187	9.137142	9.816887	9.569204	9.56222
AT2G18090	541.3583	-0.45538	0.12422	-3.66594	0.000246	0.002098	8.834518	8.891602	8.76584	9.245038	9.326696	9.318544
AT5G25265	1397.953	-0.45497	0.133924	-3.39726	0.000681	0.005143	10.26773	10.05506	10.28044	10.83219	10.52183	10.60734

AT1G78040	3572.229	-0.45496	0.119668	-3.80186	0.000144	0.0013	11.60669	11.42831	11.62031	12.14248	11.99534	11.89661
AT1G53730	528.1621	-0.45299	0.12665	-3.57669	0.000348	0.00284	8.751953	8.785157	8.827182	9.184356	9.396978	9.210737
AT1G04300	929.0593	-0.45272	0.130107	-3.47958	0.000502	0.003932	9.701726	9.602616	9.499958	10.02566	10.24529	9.951202
AT1G53910	847.9248	-0.45262	0.136705	-3.3109	0.00093	0.006775	9.427684	9.324909	9.660754	9.956493	10.00392	9.866897
AT4G19860	1220.898	-0.45131	0.119856	-3.76545	0.000166	0.001483	10.00505	9.914407	10.1129	10.55702	10.3553	10.46653
AT4G30750	301.7971	-0.45071	0.141138	-3.19341	0.001406	0.009616	8.002715	7.936808	8.04116	8.425264	8.466036	8.469584
AT5G04410	733.0321	-0.44927	0.12588	-3.56904	0.000358	0.002911	9.259784	9.314665	9.218483	9.58617	9.87031	9.738203
AT1G18450	566.9693	-0.44837	0.140924	-3.18161	0.001465	0.009959	9.01443	8.790095	8.884273	9.457761	9.419665	9.198245
AT3G26730	674.466	-0.44663	0.125524	-3.55816	0.000373	0.003023	9.110885	9.14388	9.173384	9.526889	9.785514	9.524693
AT3G45770	615.4463	-0.44608	0.133659	-3.33742	0.000846	0.006233	9.030245	8.903803	9.123869	9.563657	9.496371	9.366223
AT3G48760	442.1899	-0.4451	0.129799	-3.42914	0.000605	0.004651	8.578975	8.541443	8.551258	8.988689	8.896321	9.088815
AT3G14180	322.8046	-0.44488	0.136853	-3.25079	0.001151	0.008118	8.093102	8.125744	8.072203	8.549613	8.529989	8.558274
AT1G68580	790.2305	-0.44443	0.134706	-3.29923	0.000969	0.006996	9.548419	9.357432	9.224022	9.871063	9.862051	9.777359
AT5G63190	3748.066	-0.44391	0.115002	-3.86004	0.000113	0.001054	11.62242	11.58616	11.67745	12.18292	12.12869	11.91581
AT3G61440	3014.195	-0.44359	0.125396	-3.53747	0.000404	0.003239	11.36705	11.34631	11.22567	11.89447	11.81456	11.571
AT1G80440	2321.121	-0.44192	0.13499	-3.27375	0.001061	0.007567	11.02674	10.74421	11.02331	11.47444	11.46246	11.21822
AT4G27000	1461.069	-0.44108	0.129028	-3.41852	0.00063	0.004797	10.34679	10.11794	10.3416	10.84157	10.71304	10.59596
AT5G05690	3991.437	-0.44101	0.104031	-4.23919	2.24E-05	0.000242	11.71206	11.7533	11.70147	12.2472	12.19922	12.05428
AT1G12360	857.6538	-0.4408	0.123237	-3.57685	0.000348	0.002839	9.590726	9.507883	9.393372	9.861867	10.04828	9.95269
AT1G04400	1961.694	-0.43951	0.118196	-3.71853	0.0002	0.001749	10.64639	10.72886	10.71991	11.27824	11.1393	11.00169
AT4G31170	686.081	-0.43775	0.125988	-3.47457	0.000512	0.003999	9.247536	9.137437	9.135673	9.533855	9.785514	9.579661
AT3G49490	687.2293	-0.43443	0.125363	-3.46539	0.000529	0.004119	9.163983	9.152853	9.218483	9.61273	9.785514	9.502512
AT3G23280	693.9316	-0.43419	0.130493	-3.32726	0.000877	0.006439	9.14562	9.091507	9.354757	9.634496	9.676708	9.625165
AT4G34100	1466.965	-0.43405	0.120996	-3.58731	0.000334	0.002739	10.37352	10.18883	10.26306	10.68768	10.86541	10.61676
AT3G10720	1018.53	-0.43025	0.126096	-3.41209	0.000645	0.004895	9.770964	9.603552	9.88537	10.22819	10.21967	10.13938
AT1G06457	1375.305	-0.42976	0.125985	-3.41117	0.000647	0.004909	10.27439	10.19132	10.1285	10.74583	10.41861	10.68371
AT1G16810	428.8911	-0.42922	0.13386	-3.2065	0.001344	0.009251	8.575059	8.42352	8.542418	9.015478	8.928315	8.898094
AT5G26751	710.2071	-0.42416	0.127035	-3.33894	0.000841	0.006203	9.310118	9.133557	9.255468	9.666541	9.794222	9.568058
AT1G72180	1081.463	-0.42247	0.130348	-3.24111	0.001191	0.008345	9.717797	9.897728	9.912212	10.34632	10.37284	10.113
AT1G53570	476.9671	-0.42213	0.127558	-3.30931	0.000935	0.006804	8.695365	8.657673	8.628478	9.067609	9.214403	9.030821
AT3G24503	879.6017	-0.41821	0.122223	-3.42169	0.000622	0.004762	9.644027	9.508882	9.495385	10.03059	10.04098	9.866897
AT3G59940	4866.972	-0.41782	0.128689	-3.24677	0.001167	0.008211	12.1276	11.85773	12.06215	12.52634	12.52633	12.27226
AT5G25270	867.8074	-0.41703	0.124655	-3.34544	0.000822	0.006077	9.606157	9.484719	9.497673	10.01574	10.04828	9.813844

AT1G77180	1612.354	-0.41653	0.113543	-3.66845	0.000244	0.00208	10.47524	10.43568	10.36561	10.89485	10.93399	10.72435
AT5G36160	563.1443	-0.41488	0.130235	-3.18563	0.001444	0.00984	8.903274	8.809679	9.025787	9.390279	9.314642	9.295273
AT2G36460	1209.656	-0.41305	0.113283	-3.64619	0.000266	0.002243	10.05088	9.966229	10.01565	10.42211	10.55294	10.33692
AT1G01490	909.7281	-0.41304	0.115725	-3.56912	0.000358	0.002911	9.637471	9.634081	9.541591	10.08536	10.04828	9.940742
AT1G26830	818.4723	-0.41179	0.120379	-3.42078	0.000624	0.004774	9.422246	9.466327	9.444094	9.807337	10.03364	9.785732
AT1G01620	5203.905	-0.41147	0.110769	-3.71465	0.000203	0.001772	12.16341	11.98251	12.20887	12.51583	12.61131	12.48877
AT3G07790	651.3651	-0.40898	0.117301	-3.48658	0.000489	0.003841	9.148258	9.128367	9.092415	9.547686	9.599327	9.486164
AT3G14600	1193.729	-0.40315	0.121554	-3.31663	0.000911	0.006657	10.036	9.904575	10.0527	10.45796	10.50601	10.27284
AT1G02840	1265.19	-0.39466	0.106655	-3.70039	0.000215	0.001866	10.14236	10.05301	10.08273	10.53866	10.49002	10.44554
AT5G03280	1147.776	-0.39425	0.122153	-3.22751	0.001249	0.008693	9.876538	10.04752	9.932655	10.47253	10.30744	10.26328
AT2G23090	1474.857	-0.39268	0.120472	-3.25949	0.001116	0.007897	10.41737	10.17883	10.34923	10.75082	10.60831	10.76473
AT1G53210	2499.475	-0.38879	0.116797	-3.32876	0.000872	0.00641	11.16535	10.95858	11.09308	11.47263	11.5779	11.36235
AT1G29150	745.1183	-0.38708	0.116832	-3.31316	0.000922	0.006731	9.275552	9.321502	9.387213	9.689596	9.802877	9.694401
AT4G09800	1552.536	-0.38613	0.103854	-3.71798	0.000201	0.001752	10.43043	10.32094	10.42273	10.76865	10.80638	10.77654
AT5G64270	1932.111	-0.38528	0.108117	-3.56353	0.000366	0.002964	10.6923	10.71031	10.70859	11.03318	11.23194	11.03227
AT2G43820	1097.574	-0.38373	0.105132	-3.64994	0.000262	0.002216	9.899421	9.863781	9.921618	10.27467	10.2767	10.29412
AT4G02350	597.1811	-0.38319	0.119789	-3.19884	0.00138	0.009467	9.002817	8.987869	9.071058	9.397935	9.362265	9.446577
AT1G70290	4015.376	-0.38196	0.113305	-3.37107	0.000749	0.005584	11.82414	11.80442	11.65891	12.21867	12.20902	12.02275
AT4G29380	830.0517	-0.37122	0.116708	-3.18076	0.001469	0.00998	9.572185	9.485734	9.434569	9.820689	9.878522	9.922632
AT3G07560	1302.088	-0.37043	0.111837	-3.31219	0.000926	0.00675	10.20767	10.14967	10.08349	10.59526	10.52706	10.44343
AT2G42680	863.4214	-0.37036	0.112349	-3.29649	0.000979	0.007049	9.580997	9.491808	9.588512	9.940855	9.958166	9.896581
AT2G01850	2232.676	-0.32215	0.101091	-3.18674	0.001439	0.009807	11.00253	10.90116	10.95334	11.31846	11.29158	11.22562
AT1G65930	2928.176	0.395448	0.119881	3.298683	0.000971	0.007006	11.86219	11.6203	11.6161	11.37062	11.22873	11.29765
AT2G18790	1400.701	0.398505	0.110617	3.602562	0.000315	0.002598	10.65616	10.65409	10.62836	10.29668	10.0913	10.29177
AT1G66200	2212.847	0.411313	0.106523	3.86125	0.000113	0.00105	11.39258	11.24446	11.27253	10.92695	10.90615	10.83661
AT3G56940	14012.47	0.416379	0.127852	3.256716	0.001127	0.007963	13.91785	13.8612	14.11872	13.6336	13.62895	13.36571
AT1G63980	775.5641	0.416966	0.117671	3.543497	0.000395	0.003176	9.832937	9.804008	9.772712	9.403016	9.252813	9.433847
AT1G77760	1141.491	0.420075	0.13151	3.194235	0.001402	0.009594	10.26044	10.28175	10.52332	10.01076	9.802877	9.946728
AT3G04120	2622.68	0.422469	0.122095	3.46016	0.00054	0.004194	11.48729	11.55652	11.61794	10.94883	11.17307	11.24701
AT1G73920	495.6872	0.427079	0.1339	3.189526	0.001425	0.009731	9.210156	9.220259	9.035271	8.738334	8.706416	8.720804
AT1G01320	2246.745	0.429401	0.126643	3.390641	0.000697	0.005245	11.29419	11.47378	11.21771	10.90204	11.02927	10.7698
AT3G56950	1224.682	0.432304	0.123324	3.505435	0.000456	0.003613	10.44445	10.35744	10.5865	10.10731	9.918893	10.02237
AT1G75350	1401.764	0.434327	0.135644	3.201954	0.001365	0.009386	10.50949	10.58568	10.86125	10.27744	10.14675	10.21325

AT2G44160	945.91	0.434675	0.125845	3.454046	0.000552	0.004278	10.08087	10.11335	10.07815	9.789986	9.638536	9.506571
AT2G38170	2722.207	0.434723	0.115884	3.751354	0.000176	0.001558	11.62124	11.4903	11.72608	11.25945	11.11517	11.13874
AT4G24280	1562.533	0.438871	0.117252	3.74296	0.000182	0.001605	10.70937	10.93833	10.79347	10.42336	10.33754	10.34714
AT3G62530	960.0104	0.444093	0.137903	3.220338	0.00128	0.008878	10.01809	10.13357	10.21588	9.63881	9.453039	9.843024
AT3G08785	706.2649	0.449885	0.140807	3.195051	0.001398	0.00957	9.568251	9.647748	9.806335	9.359243	9.201369	9.080671
AT3G15570	472.3742	0.454666	0.135249	3.361687	0.000775	0.005763	8.98817	9.137437	9.14154	8.594723	8.760736	8.585388
AT1G64510	1681.292	0.457558	0.137919	3.317579	0.000908	0.006636	10.81731	10.87429	11.09384	10.63898	10.33157	10.3917
AT3G09200	4020.997	0.460581	0.131678	3.497777	0.000469	0.003704	12.07347	12.15127	12.33246	11.59423	11.65162	11.89428
AT4G34540	589.3473	0.46405	0.142187	3.263668	0.0011	0.007794	9.287565	9.469409	9.505655	9.089837	8.912407	8.828473
AT5G57800	535.6851	0.464889	0.132834	3.499779	0.000466	0.003678	9.303034	9.315806	9.26893	8.947549	8.611075	8.812171
AT3G18490	1058.322	0.465244	0.128846	3.610851	0.000305	0.002526	10.13706	10.27882	10.35176	9.752656	9.958166	9.685478
AT1G08190	517.8624	0.466441	0.144129	3.236274	0.001211	0.008468	9.144299	9.404344	9.140075	8.805112	8.795846	8.692583
AT5G11420	3589.411	0.469037	0.145039	3.233867	0.001221	0.008528	11.80567	12.07645	12.18422	11.73689	11.4321	11.45818
AT5G20700	1246.197	0.469645	0.114499	4.101725	4.10E-05	0.000419	10.45088	10.55652	10.51261	10.1228	9.910909	10.02095
AT3G56910	1434.356	0.471682	0.140834	3.349216	0.00081	0.006008	10.63233	10.62767	10.85991	10.42461	10.09835	10.12096
AT5G22830	317.4514	0.473551	0.148666	3.185342	0.001446	0.009847	8.492358	8.513849	8.63265	8.157066	7.883112	8.058669
AT4G33220	471.3161	0.474649	0.132597	3.579634	0.000344	0.002815	9.11763	9.057782	9.166208	8.664088	8.466036	8.68544
AT3G27160	1320.817	0.47515	0.141449	3.359158	0.000782	0.00581	10.39145	10.62583	10.73259	10.23675	10.09835	9.970428
AT3G16520	794.7889	0.475587	0.142008	3.349008	0.000811	0.006011	9.831297	9.701155	10.00844	9.40808	9.496371	9.228045
AT1G67090	46287.95	0.47704	0.143316	3.328599	0.000873	0.006412	15.68435	15.47584	15.96211	15.31537	15.25048	15.1322
AT2G33450	904.2288	0.477237	0.147859	3.22765	0.001248	0.008691	9.883678	9.986447	10.24001	9.679162	9.569204	9.416697
AT1G67700	1391.914	0.478433	0.1441	3.320151	0.0009	0.006583	10.57761	10.60543	10.81749	10.39432	10.03364	10.06702
AT1G72450	648.9149	0.48394	0.128325	3.771193	0.000162	0.001454	9.568251	9.583782	9.590658	9.181402	8.846958	9.131487
AT2G20890	1449.397	0.484612	0.130392	3.716585	0.000202	0.00176	10.6105	10.66443	10.89506	10.33573	10.19358	10.16528
AT2G26910	1241.907	0.486458	0.116636	4.170748	3.04E-05	0.000318	10.5594	10.54439	10.41124	10.09951	9.965894	9.960107
AT3G53580	492.7083	0.486994	0.14912	3.265777	0.001092	0.007753	9.139005	9.099486	9.316376	8.689264	8.42177	8.834943
AT5G28020	761.6221	0.488608	0.128495	3.802542	0.000143	0.001297	9.777793	9.752655	9.896687	9.413126	9.12059	9.33004
AT4G20070	450.8901	0.488929	0.144672	3.379579	0.000726	0.005435	8.973372	8.997863	9.137142	8.603578	8.687846	8.379515
AT5G45170	266.4705	0.491222	0.151638	3.23945	0.001198	0.00839	8.208804	8.32262	8.343025	7.796798	7.78168	7.792291
AT1G03475	763.8541	0.492071	0.130581	3.768314	0.000164	0.001469	9.685475	9.828215	9.894952	9.385153	9.33865	9.200752
AT5G57180	251.0479	0.493573	0.153647	3.212393	0.001316	0.00909	8.275467	8.189423	8.170152	7.757664	7.63429	7.696066
AT4G39040	502.8459	0.495334	0.134341	3.687143	0.000227	0.001954	9.14033	9.164308	9.291529	8.730271	8.795846	8.604447
AT2G46030	354.6988	0.495335	0.155368	3.188143	0.001432	0.009773	8.630819	8.673793	8.852438	8.267055	7.915398	8.311553

AT3G26440	604.4088	0.495747	0.140148	3.53732	0.000404	0.00324	9.602314	9.454971	9.363567	9.102387	8.8636	8.904259
AT4G15110	405.1002	0.497122	0.153031	3.248508	0.00116	0.008174	8.831239	8.927899	8.913708	8.5358	8.466036	8.165198
AT2G20180	294.1203	0.498161	0.15036	3.313114	0.000923	0.006731	8.395775	8.47349	8.397968	7.936518	8.095267	7.798924
AT4G34980	677.0026	0.501042	0.143222	3.498352	0.000468	0.003697	9.601352	9.511874	9.788687	9.256313	9.106676	9.005234
AT3G23530	378.3397	0.501851	0.141961	3.535119	0.000408	0.003264	8.826306	8.862206	8.707718	8.364046	8.280186	8.2206
AT1G21590	451.1088	0.50193	0.139465	3.598972	0.000319	0.00263	8.941793	9.113999	9.114952	8.634151	8.444073	8.518635
AT2G46710	590.2635	0.502177	0.135463	3.707103	0.00021	0.001822	9.453506	9.392488	9.496529	9.092985	8.778398	8.866864
AT1G48650	410.1918	0.502398	0.144909	3.466989	0.000526	0.004098	9.004274	8.933861	8.814386	8.526517	8.352714	8.316185
AT5G64030	399.2066	0.502513	0.148688	3.379654	0.000726	0.005435	8.866906	9.026042	8.752486	8.41524	8.229725	8.410167
AT3G25660	411.6609	0.503364	0.141893	3.547495	0.000389	0.003134	8.906394	8.871554	9.000188	8.540419	8.328941	8.329991
AT1G25440	1751.362	0.50446	0.130097	3.877556	0.000106	0.000991	10.97196	11.02362	11.04107	10.70218	10.33754	10.4059
AT5G23010	774.0214	0.507668	0.145707	3.484161	0.000494	0.003871	9.69993	9.97131	9.787753	9.142435	9.528036	9.292925
AT3G01120	724.9205	0.507867	0.118299	4.293068	1.76E-05	0.000194	9.710676	9.706388	9.797074	9.196113	9.214403	9.257241
AT1G32500	370.5799	0.508221	0.146748	3.463219	0.000534	0.00415	8.655131	8.791737	8.879016	8.337752	8.177436	8.225533
AT2G32480	287.1506	0.509172	0.154632	3.292797	0.000992	0.007129	8.329921	8.469391	8.431873	8.018263	7.816289	7.798924
AT1G69740	1118.194	0.510682	0.139096	3.671424	0.000241	0.002061	10.26834	10.31696	10.48172	9.889283	10.01141	9.636321
AT1G02910	260.8113	0.513198	0.15804	3.247257	0.001165	0.008199	8.270634	8.211676	8.373253	7.849861	7.51294	7.785626
AT1G51805	288.9877	0.515876	0.15817	3.261537	0.001108	0.007848	8.55929	8.362947	8.325095	7.971143	7.883112	7.818645
AT1G10510	246.2155	0.517941	0.161591	3.205257	0.001349	0.009289	8.059867	8.281133	8.242898	7.70103	7.554534	7.68894
AT5G13650	1446.745	0.519677	0.13367	3.887767	0.000101	0.000955	10.80524	10.69679	10.71254	10.38021	10.22612	10.0067
AT1G41830	274.4483	0.52155	0.161907	3.221286	0.001276	0.008851	8.311213	8.378331	8.41502	8.004957	7.47011	7.825159
AT3G03710	344.6868	0.521828	0.16081	3.244995	0.001175	0.008257	8.563249	8.69501	8.792183	8.343049	7.946977	8.058669
AT4G37200	308.2607	0.522019	0.146402	3.565642	0.000363	0.002945	8.441653	8.495827	8.581781	7.950468	8.066803	7.955567
AT4G36808	282.9668	0.522373	0.150947	3.46063	0.000539	0.004188	8.311213	8.378331	8.481282	7.879329	7.850087	7.844526
AT1G37130	11829.38	0.522467	0.10118	5.163723	2.42E-07	3.66E-06	13.82302	13.71859	13.76405	13.20075	13.34621	13.17808
AT2G25670	471.4739	0.522971	0.161577	3.236677	0.001209	0.00846	9.090458	9.276468	9.035271	8.48399	8.376102	8.828473
AT1G60000	415.854	0.523402	0.15836	3.305143	0.000949	0.006887	9.001359	8.775229	9.044693	8.558748	8.42177	8.24023
AT1G22710	585.881	0.524213	0.151566	3.458651	0.000543	0.004216	9.249994	9.43934	9.622466	8.868935	8.795846	9.016662
AT5G46290	707.0237	0.524424	0.150322	3.488682	0.000485	0.003816	9.646827	9.663081	9.835579	9.40555	8.990252	9.064245
AT5G63570	585.71	0.524938	0.145669	3.603628	0.000314	0.002589	9.311296	9.352989	9.626655	8.940576	8.944051	8.834943
AT3G14210	13727.1	0.526261	0.139545	3.771271	0.000162	0.001454	13.85233	13.94859	14.14979	13.51115	13.21861	13.59622
AT1G49340	591.963	0.527482	0.149689	3.523845	0.000425	0.003394	9.273137	9.634081	9.448833	8.861572	8.846958	9.022342
AT3G12590	296.2347	0.528698	0.150157	3.520978	0.00043	0.003429	8.529251	8.446638	8.370758	7.915337	8.037767	7.844526

AT4G36810	282.2013	0.529517	0.151133	3.503658	0.000459	0.003633	8.308857	8.376143	8.481282	7.857284	7.850087	7.844526
AT5G25460	6951.42	0.529854	0.149316	3.548543	0.000387	0.003123	12.84716	13.01705	13.14991	12.70179	12.22843	12.42694
AT1G72600	6200.416	0.529993	0.152907	3.466112	0.000528	0.004109	13.00264	12.48397	12.98801	12.29377	12.29743	12.31454
AT2G42690	995.0015	0.530563	0.147521	3.596535	0.000322	0.002652	10.39922	10.09684	10.08806	9.752656	9.75015	9.488218
AT1G72610	6248.188	0.53257	0.15342	3.471329	0.000518	0.004041	13.01552	12.49346	13.00138	12.30433	12.30658	12.3232
AT1G44920	284.3875	0.53296	0.162694	3.275847	0.001053	0.007517	8.292259	8.32489	8.546845	7.872018	8.008134	7.731173
AT2G30950	4115.338	0.533658	0.14014	3.808037	0.00014	0.001272	12.00927	12.28933	12.43065	11.8133	11.71031	11.59788
AT4G33500	345.5914	0.534347	0.162005	3.298346	0.000973	0.007011	8.537323	8.670227	8.866674	8.175	7.915398	8.245097
AT5G15450	546.7846	0.535459	0.158692	3.374207	0.00074	0.00553	9.076677	9.495844	9.422574	8.842996	8.846958	8.710286
AT1G20020	2326.793	0.535494	0.147659	3.626566	0.000287	0.002394	11.21934	11.43332	11.62136	11.06639	10.78032	10.78408
AT5G45775	697.1225	0.536194	0.160705	3.336501	0.000848	0.006248	9.72753	9.674698	9.693068	8.868935	9.092627	9.414539
AT2G21960	435.237	0.537933	0.156181	3.444283	0.000573	0.004416	9.024514	8.887001	9.081776	8.445107	8.742856	8.249946
AT2G32870	499.9467	0.538289	0.162592	3.310675	0.000931	0.006779	8.985222	9.357432	9.299422	8.835499	8.550692	8.57383
AT3G25920	2357.306	0.538602	0.141907	3.795464	0.000147	0.001329	11.31047	11.40784	11.62581	11.09572	10.77593	10.80647
AT3G10405	481.552	0.539997	0.14095	3.831119	0.000128	0.001173	9.253673	9.035776	9.1947	8.705807	8.591227	8.534622
AT4G14550	242.8899	0.54106	0.157486	3.43561	0.000591	0.004551	8.172987	8.181928	8.190101	7.741709	7.594963	7.530557
AT4G34830	527.4488	0.541091	0.146955	3.682024	0.000231	0.001987	9.144299	9.350763	9.369827	8.85417	8.795846	8.581546
AT3G45850	702.1742	0.542591	0.157492	3.445207	0.000571	0.004406	9.488274	9.833008	9.795214	9.346112	9.02025	9.050412
AT5G50250	364.0258	0.544566	0.167551	3.250145	0.001153	0.008132	8.565224	8.827401	8.917132	8.369248	7.946977	8.210684
AT2G40400	277.3637	0.54622	0.147614	3.700323	0.000215	0.001866	8.377907	8.354082	8.393059	7.789056	7.78168	7.863636
AT5G47190	1073.422	0.548135	0.14832	3.695633	0.000219	0.001896	10.16858	10.353	10.44602	9.980486	9.589355	9.665653
AT2G08420	426.1861	0.54824	0.15106	3.629297	0.000284	0.002373	8.818048	8.996439	9.135673	8.48399	8.466036	8.352713
AT1G23310	14876.71	0.548322	0.104701	5.23705	1.63E-07	2.54E-06	14.07669	14.04796	14.2044	13.62276	13.58321	13.46211
AT3G14420	10053.24	0.548573	0.12272	4.470101	7.82E-06	9.20E-05	13.43527	13.47295	13.71597	13.06067	13.0306	12.8748
AT1G05560	545.6706	0.549029	0.150929	3.637673	0.000275	0.002307	9.157452	9.515854	9.333133	8.676731	8.880053	8.818714
AT1G05562	545.6706	0.549029	0.150929	3.637673	0.000275	0.002307	9.157452	9.515854	9.333133	8.676731	8.880053	8.818714
AT2G01110	470.2362	0.550322	0.142591	3.859442	0.000114	0.001057	9.055757	9.1061	9.235038	8.697559	8.550692	8.457056
AT1G03130	8430.553	0.55079	0.13221	4.166038	3.10E-05	0.000324	13.23612	13.15399	13.47483	12.87909	12.69738	12.61325
AT2G29970	281.2467	0.550985	0.151098	3.646537	0.000266	0.002242	8.359814	8.483686	8.330241	7.849861	7.850087	7.805528
AT1G09873	1130.86	0.551936	0.153874	3.586928	0.000335	0.002741	10.31369	10.46788	10.37995	9.710241	10.16028	9.621428
AT5G42390	1105.34	0.554899	0.148446	3.738065	0.000185	0.001633	10.11161	10.52971	10.41548	9.70819	9.934731	9.768937
AT1G29418	360.5307	0.555185	0.166043	3.343615	0.000827	0.006113	8.718622	8.66844	8.910276	8.120516	7.915398	8.410167
AT2G21385	236.9144	0.555243	0.169898	3.268106	0.001083	0.0077	8.216365	8.026019	8.248344	7.741709	7.33342	7.569875

AT2G26690	557.8593	0.555333	0.15816	3.511217	0.000446	0.003545	9.203831	9.57045	9.379788	8.812769	8.591227	8.970397
AT5G03520	760.9471	0.555414	0.132823	4.181592	2.89E-05	0.000305	9.811465	9.737397	9.951143	9.369663	9.092627	9.281129
AT5G64040	9599.308	0.556041	0.145492	3.821805	0.000132	0.00121	13.44334	13.26168	13.71425	13.06589	12.85446	12.81796
AT2G34510	660.6537	0.557316	0.134308	4.149529	3.33E-05	0.000346	9.680928	9.508882	9.69606	9.181402	8.944051	9.016662
AT5G24314	363.536	0.557389	0.159254	3.50001	0.000465	0.003676	8.660684	8.6809	8.922253	8.272618	8.304769	8.030761
AT3G41761	926.9669	0.558497	0.160839	3.472389	0.000516	0.004028	9.925025	10.11794	10.30483	9.559112	9.252813	9.739928
AT5G42240	228.1412	0.559785	0.16205	3.454401	0.000552	0.004274	8.037277	8.094149	8.126453	7.378772	7.672573	7.562097
AT1G06430	1192.652	0.560055	0.134632	4.159899	3.18E-05	0.000332	10.26894	10.557	10.58057	9.928575	9.973581	9.830128
AT3G59980	284.4299	0.562337	0.155819	3.608905	0.000307	0.002542	8.450229	8.302025	8.513305	7.922432	7.709866	7.844526
AT5G47840	677.7104	0.563476	0.135156	4.169087	3.06E-05	0.00032	9.581973	9.595112	9.803563	9.166539	9.106676	9.002363
AT3G26060	701.5287	0.563661	0.174397	3.232051	0.001229	0.008577	9.463072	9.641386	9.994734	9.286871	9.148022	8.961555
AT5G59690	337.2695	0.564314	0.163811	3.444914	0.000571	0.004409	8.653275	8.495827	8.845267	8.204403	7.915398	8.075159
AT4G20362	4744.317	0.564805	0.129689	4.355074	1.33E-05	0.00015	12.23665	12.52169	12.62597	11.89672	11.92175	11.87322
AT4G20360	4735.728	0.565477	0.129811	4.356157	1.32E-05	0.000149	12.23386	12.51959	12.62375	11.89357	11.91977	11.86929
AT1G08520	1269.507	0.565724	0.141054	4.010678	6.05E-05	0.0006	10.39089	10.59371	10.70166	10.08219	10.08422	9.813844
AT2G37920	205.1292	0.565849	0.166788	3.392631	0.000692	0.005215	7.874778	7.945686	7.96381	7.249335	7.594963	7.325306
AT1G11840	725.5221	0.566416	0.145813	3.884539	0.000103	0.000967	9.576108	9.953814	9.749853	9.256313	9.106676	9.180574
AT2G20410	4579.592	0.567084	0.167063	3.394436	0.000688	0.005187	12.4477	12.31654	12.50719	11.91062	11.42371	12.09595
AT2G44650	489.0113	0.567331	0.151469	3.745532	0.00018	0.001591	9.086337	9.078112	9.40195	8.599158	8.630654	8.626991
AT2G14880	219.963	0.568208	0.169433	3.35358	0.000798	0.005923	8.198661	7.939773	8.015834	7.525773	7.380437	7.465332
AT4G17300	220.9852	0.568973	0.172478	3.298822	0.000971	0.007004	7.915604	8.067282	8.129408	7.507017	7.672573	7.288068
AT5G24165	500.19	0.56915	0.175516	3.242725	0.001184	0.008305	9.108178	9.125765	9.454734	8.905201	8.48767	8.477876
AT2G04842	529.1108	0.569152	0.137442	4.141037	3.46E-05	0.000359	9.15876	9.378349	9.372324	8.730271	8.795846	8.68544
AT1G20810	281.7136	0.569689	0.178114	3.198454	0.001382	0.009478	8.318257	8.410753	8.53129	8.101887	7.51294	7.710211
AT1G65010	755.9956	0.569881	0.170415	3.344077	0.000826	0.006105	9.635592	10.06664	9.76228	9.479571	9.049638	9.134113
AT4G04040	578.8419	0.570349	0.124776	4.570997	4.85E-06	5.93E-05	9.403601	9.422478	9.490797	8.912345	8.8636	8.808888
AT3G11120	416.1282	0.570763	0.174725	3.266634	0.001088	0.007732	8.857266	9.027437	9.058711	8.278159	8.037767	8.681856
AT1G67740	4269.145	0.571886	0.179177	3.191742	0.001414	0.009662	12.11653	12.15606	12.63692	11.60475	12.0141	11.54903
AT1G73060	540.2873	0.573333	0.163154	3.514053	0.000441	0.003509	9.102749	9.376161	9.497673	8.777989	8.944051	8.566073
AT4G04210	205.983	0.575458	0.174471	3.298297	0.000973	0.007011	7.87796	7.860616	8.129408	7.378772	7.33342	7.379421
AT1G70410	769.7994	0.576199	0.1381	4.172343	3.01E-05	0.000317	9.858128	9.820996	9.915639	9.278601	8.990252	9.444463
AT1G35680	1232.898	0.576204	0.162571	3.544317	0.000394	0.003168	10.337	10.4383	10.78834	10.11353	9.934731	9.760465
AT1G14270	288.5155	0.576343	0.17444	3.303956	0.000953	0.0069	8.395775	8.367359	8.603195	8.095624	7.554534	7.778931

AT1G35460	167.9958	0.576853	0.173468	3.325405	0.000883	0.00648	7.632571	7.659443	7.707214	7.043474	7.072174	7.10747
AT3G21055	4618.235	0.577688	0.162862	3.547099	0.000389	0.003138	12.30562	12.19723	12.75318	11.9476	11.87942	11.70374
AT3G15190	1108.795	0.57779	0.174949	3.302614	0.000958	0.006926	10.21146	10.21661	10.68267	10.00409	9.569204	9.736476
AT3G27830	1445.841	0.578474	0.151724	3.812682	0.000137	0.00125	10.62667	10.61566	11.02172	10.27744	10.03364	10.18314
AT2G36880	492.9163	0.582006	0.135421	4.297766	1.73E-05	0.00019	9.109532	9.290475	9.247331	8.693418	8.550692	8.600655
AT2G22970	201.0022	0.583782	0.174719	3.341254	0.000834	0.006158	7.979205	7.863742	7.973708	7.525773	7.128345	7.240127
AT3G02570	352.309	0.585187	0.176786	3.310135	0.000933	0.006786	8.79971	8.619344	8.847063	8.278159	7.594963	8.306906
AT5G11480	313.1943	0.585227	0.150474	3.889228	0.000101	0.00095	8.537323	8.517823	8.659472	8.083015	7.816289	7.943643
AT5G19220	921.542	0.585832	0.150491	3.892815	9.91E-05	0.000938	10.08845	10.14646	10.12776	9.780255	9.408366	9.311602
AT1G78850	867.1002	0.585885	0.136483	4.292735	1.76E-05	0.000194	10.03814	9.944247	10.06818	9.261917	9.676708	9.379562
AT3G16470	675.3171	0.586049	0.161188	3.635819	0.000277	0.00232	9.543418	9.938328	9.486194	9.00549	9.078439	9.126221
AT5G14210	224.9741	0.586461	0.180926	3.241448	0.001189	0.008338	8.268211	7.960363	7.996544	7.580621	7.594963	7.306808
AT2G24860	193.6361	0.586554	0.18353	3.195965	0.001394	0.009547	7.730868	7.95451	7.990057	7.27173	6.952811	7.448554
AT5G14570	268.1574	0.58667	0.163843	3.580682	0.000343	0.002805	8.409033	8.308923	8.22919	7.650652	8.037767	7.600575
AT5G03940	998.5062	0.588981	0.136958	4.300435	1.70E-05	0.000188	10.16077	10.27178	10.28641	9.835799	9.430875	9.575804
AT5G01410	368.0728	0.589499	0.141384	4.169497	3.05E-05	0.00032	8.729231	8.854369	8.773416	8.180929	8.328941	8.118231
AT3G04340	597.9908	0.59097	0.167915	3.519472	0.000432	0.003447	9.293535	9.702028	9.487346	9.114828	8.724751	8.775643
AT1G29850	373.5841	0.591063	0.138563	4.265676	1.99E-05	0.000217	8.83288	8.806434	8.836253	8.180929	8.12318	8.311553
AT3G23590	213.3308	0.591832	0.164215	3.604007	0.000313	0.002586	8.062666	8.017623	8.003003	7.378772	7.234523	7.538507
AT4G14870	312.097	0.593916	0.179978	3.299932	0.000967	0.006983	8.435188	8.467338	8.779072	8.169047	7.850087	7.785626
AT4G03280	4417.598	0.594022	0.130246	4.56077	5.10E-06	6.19E-05	12.26412	12.28831	12.56807	11.89402	11.74216	11.68237
AT2G26710	285.8141	0.594621	0.170053	3.496684	0.000471	0.003718	8.473552	8.402178	8.385664	7.936518	8.037767	7.530557
AT5G19221	470.4556	0.595184	0.161653	3.681869	0.000232	0.001988	9.121662	9.183201	9.164768	8.801269	8.466036	8.306906
AT1G79790	375.5113	0.595634	0.149662	3.979869	6.90E-05	0.000673	8.715068	8.859076	8.920548	8.353586	8.12318	8.149711
AT1G51400	7711.884	0.595666	0.137846	4.321255	1.55E-05	0.000172	13.17777	13.02388	13.33568	12.74795	12.57261	12.39172
AT3G07230	477.0228	0.595998	0.175827	3.389673	0.0007	0.005261	9.201293	9.079457	9.295481	8.647057	8.037767	8.8023
AT1G73480	643.1884	0.59662	0.146215	4.080439	4.50E-05	0.000456	9.673623	9.65137	9.453556	8.789676	9.148022	9.058728
AT3G13330	405.2799	0.59785	0.161503	3.701779	0.000214	0.001857	8.784531	9.059146	8.960918	8.126672	8.352714	8.48612
AT1G60950	6838.508	0.598234	0.141567	4.225811	2.38E-05	0.000255	12.94824	12.79627	13.25452	12.4645	12.42905	12.31106
AT2G37600	302.6029	0.598474	0.164116	3.646655	0.000266	0.002242	8.473552	8.419277	8.657427	7.741709	7.915398	8.047571
AT3G15520	272.1694	0.598968	0.170764	3.507576	0.000452	0.003588	8.180737	8.395713	8.535751	7.86467	7.594963	7.74498
AT1G18010	269.0752	0.600175	0.163293	3.675438	0.000237	0.002031	8.393554	8.181928	8.448531	7.773445	7.816289	7.645434
AT3G63190	672.088	0.600864	0.165103	3.639324	0.000273	0.002296	9.532354	9.654081	9.824682	9.32217	8.896321	8.873164

AT1G18000	266.2923	0.602972	0.164552	3.664328	0.000248	0.002109	8.382395	8.16175	8.434265	7.749708	7.816289	7.623179
AT4G37760	413.9591	0.604077	0.144819	4.171261	3.03E-05	0.000318	8.962922	8.848069	9.081776	8.39498	8.42177	8.269184
AT1G68000	172.3599	0.604918	0.183174	3.302424	0.000959	0.006928	7.7622	7.708954	7.746235	7.043474	6.752923	7.316087
AT5G47110	1197.055	0.60494	0.15143	3.994855	6.47E-05	0.000637	10.54245	10.38544	10.58919	10.14268	9.657748	9.782389
AT5G20130	210.4418	0.605131	0.168367	3.594124	0.000325	0.002675	8.048616	7.95744	8.003003	7.525773	7.33342	7.259495
AT3G10270	192.4179	0.605845	0.169651	3.571127	0.000355	0.002891	7.858764	7.966193	7.828538	7.304685	7.072174	7.306808
AT5G30510	2380.429	0.606379	0.133681	4.53601	5.73E-06	6.93E-05	11.31195	11.45057	11.69301	10.94623	10.84461	10.83338
AT5G25190	490.5168	0.606511	0.187437	3.235815	0.001213	0.008479	8.977827	9.214148	9.39828	8.634151	8.896321	8.24023
AT1G66430	336.9363	0.606682	0.160496	3.780037	0.000157	0.001407	8.555321	8.679127	8.795908	8.221762	8.008134	7.913394
AT3G60750	4666.747	0.607709	0.126029	4.821978	1.42E-06	1.91E-05	12.27261	12.48079	12.61705	11.88769	11.89164	11.76134
AT5G51110	650.0485	0.607944	0.143803	4.227608	2.36E-05	0.000253	9.552407	9.505884	9.772712	9.008827	9.161545	8.863703
AT1G52400	1892.41	0.608334	0.180231	3.375294	0.000737	0.005513	10.85372	11.54342	11.01255	10.62709	10.46842	10.51266
AT1G79750	963.368	0.609332	0.148601	4.100446	4.12E-05	0.000421	10.20767	10.16115	10.17999	9.651675	9.776754	9.269234
AT5G01270	196.5319	0.609366	0.170275	3.578713	0.000345	0.002821	7.900038	7.986412	7.806553	7.326244	7.33342	7.200593
AT5G54630	386.0157	0.610534	0.16406	3.721411	0.000198	0.001733	8.91726	8.980688	8.758225	8.488778	7.977879	8.175431
AT4G33520	253.1447	0.610991	0.173234	3.526963	0.00042	0.003361	8.178158	8.313503	8.332807	7.872018	7.51294	7.473648
AT1G66330	425.4758	0.611304	0.155325	3.935642	8.30E-05	0.000799	8.821357	9.09284	9.102976	8.479187	8.444073	8.25478
AT1G79850	1420.11	0.611972	0.15748	3.886033	0.000102	0.000962	10.62856	10.54244	11.03439	10.18901	10.16028	10.02662
AT3G62110	242.2699	0.612084	0.164863	3.712681	0.000205	0.001785	8.346094	8.179421	8.102593	7.659171	7.47011	7.569875
AT1G58080	415.2847	0.613429	0.176049	3.484422	0.000493	0.003869	8.702561	8.961847	9.243245	8.425264	8.280186	8.329991
AT3G56010	249.9156	0.613938	0.181639	3.379998	0.000725	0.00543	8.191007	8.148993	8.393059	7.812159	7.672573	7.361607
AT5G22800	589.2226	0.614697	0.172282	3.567973	0.00036	0.002921	9.149575	9.682686	9.579898	8.777989	8.813085	8.958595
AT1G09927	1417.609	0.615216	0.15761	3.903417	9.48E-05	0.000903	10.62714	10.54098	11.0336	10.18607	10.15353	10.02237
AT3G24170	454.2774	0.615263	0.170187	3.615208	0.0003	0.002491	8.929578	9.342943	9.021022	8.294657	8.550692	8.593042
AT3G06680	183.4137	0.615441	0.177228	3.472597	0.000515	0.004026	7.822893	7.828975	7.846606	7.168094	6.822676	7.370542
AT4G04640	4643.423	0.615927	0.145604	4.230157	2.34E-05	0.000251	12.26731	12.39521	12.69117	11.98916	11.74889	11.74383
AT5G46240	216.6862	0.616642	0.168271	3.664569	0.000248	0.002108	8.011433	8.011998	8.072203	7.478418	7.594963	7.220496
AT1G51402	5884.922	0.61686	0.146411	4.213214	2.52E-05	0.000269	12.75823	12.65545	12.97708	12.32087	12.25074	11.92304
AT3G56370	224.4401	0.617064	0.170265	3.624127	0.00029	0.002415	8.149482	8.083462	8.144092	7.624787	7.013727	7.554276
AT4G30825	176.8011	0.617571	0.187562	3.292626	0.000993	0.00713	7.625016	7.912861	7.780474	7.11953	6.822676	7.288068
AT1G10630	368.4794	0.618788	0.139924	4.422319	9.76E-06	0.000113	8.839423	8.738235	8.839865	8.114333	8.229725	8.210684
AT3G17470	169.5104	0.619453	0.193123	3.20755	0.001339	0.009222	7.559151	7.888511	7.699282	7.191776	6.679627	7.139183
AT3G53130	199.2495	0.62071	0.173961	3.568104	0.00036	0.00292	7.861981	7.974893	8.012637	7.44924	6.952811	7.325306

AT1G73170	226.3941	0.621064	0.167095	3.716821	0.000202	0.001759	8.028714	8.136123	8.204183	7.624787	7.284819	7.440091
AT2G01750	135.0448	0.621098	0.193559	3.208829	0.001333	0.009186	7.447939	7.360699	7.370122	6.9213	6.434336	6.703006
AT1G21350	191.8281	0.621671	0.187697	3.312103	0.000926	0.00675	7.884303	7.729664	8.059866	7.39926	6.88921	7.269083
AT4G18570	264.9937	0.621965	0.168612	3.688734	0.000225	0.001942	8.329921	8.174394	8.457965	7.725575	7.883112	7.538507
AT2G24020	588.9889	0.622503	0.14001	4.446144	8.74E-06	0.000102	9.451372	9.486748	9.549334	9.041779	8.649971	8.795682
AT5G42270	2057.587	0.625797	0.161301	3.879686	0.000105	0.000983	10.9521	11.47761	11.38992	10.67723	10.77153	10.50152
AT3G28920	147.4991	0.625873	0.190617	3.283402	0.001026	0.007345	7.531131	7.351821	7.587692	6.740426	6.952811	6.900984
AT4G13930	500.3068	0.626221	0.141955	4.411388	1.03E-05	0.000119	9.123003	9.303196	9.352229	8.647057	8.444073	8.69969
AT4G18370	273.6612	0.631437	0.174903	3.610208	0.000306	0.00253	8.263352	8.360736	8.560045	7.950468	7.554534	7.638054
AT2G24060	309.5112	0.631807	0.173373	3.64421	0.000268	0.002256	8.565224	8.479616	8.701774	8.175	7.554534	7.869951
AT2G34660	431.7423	0.63276	0.196134	3.226155	0.001255	0.00873	8.777734	9.418232	8.861352	8.300115	8.399116	8.473736
AT5G22010	167.5034	0.632999	0.19392	3.264232	0.001098	0.007785	7.586637	7.89769	7.583389	6.976881	6.88921	7.170215
AT4G09890	271.7381	0.633017	0.187073	3.383791	0.000715	0.005362	8.506818	8.318069	8.375744	8.051003	7.284819	7.645434
AT1G05320	146.8274	0.633154	0.19669	3.21905	0.001286	0.008913	7.395619	7.670197	7.365117	6.787815	7.013727	6.785485
AT4G20410	257.7446	0.633953	0.175485	3.612583	0.000303	0.002512	8.263352	8.186929	8.478967	7.849861	7.47011	7.562097
AT1G72970	276.9614	0.63591	0.153875	4.13263	3.59E-05	0.00037	8.325267	8.44037	8.460313	7.849861	7.709866	7.696066
AT5G53460	2724.346	0.637146	0.136259	4.675978	2.93E-06	3.71E-05	11.53921	11.90326	11.63986	11.14128	10.91815	11.07731
AT5G09870	385.5193	0.637476	0.144917	4.398891	1.09E-05	0.000125	8.895446	8.942757	8.859573	8.28368	7.946977	8.357215
AT1G22410	314.4898	0.637873	0.167628	3.805291	0.000142	0.001283	8.473552	8.598853	8.705739	7.749708	7.883112	8.134057
AT5G01650	309.0382	0.638511	0.173732	3.675266	0.000238	0.002031	8.533293	8.410753	8.779072	7.929492	7.63429	8.064187
AT3G26450	504.5815	0.639347	0.157928	4.048351	5.16E-05	0.000518	9.418973	9.233612	9.177672	8.842996	8.42177	8.514611
AT5G09660	5497.111	0.640158	0.117363	5.45452	4.91E-08	8.23E-07	12.60189	12.64117	12.87878	12.08712	12.09031	12.01565
AT2G30570	15796.72	0.640757	0.146493	4.373988	1.22E-05	0.000139	14.1417	14.08321	14.46061	13.74747	13.38923	13.58942
AT4G36190	164.3623	0.641759	0.178243	3.600467	0.000318	0.002618	7.578837	7.746698	7.650749	7.003888	7.013727	6.99642
AT4G17740	196.4885	0.642887	0.187855	3.422253	0.000621	0.004755	7.751831	7.933836	8.038018	7.409395	7.33342	7.030642
AT1G71340	185.2069	0.643091	0.179284	3.586995	0.000335	0.002741	7.955305	7.780174	7.806553	7.11953	6.952811	7.343571
AT5G19855	246.0436	0.643149	0.171559	3.748851	0.000178	0.001573	8.256034	8.308923	8.242898	7.765576	7.072174	7.674584
AT5G65480	519.6813	0.643787	0.133136	4.835551	1.33E-06	1.79E-05	9.382473	9.350763	9.228163	8.680921	8.529989	8.727774
AT4G33010	2431.583	0.64502	0.186687	3.455082	0.00055	0.004266	11.13343	11.58047	11.82918	10.98147	11.00697	10.61488
AT4G38740	635.0097	0.645163	0.138998	4.641542	3.46E-06	4.33E-05	9.439577	9.702028	9.673969	8.968265	8.830121	9.010959
AT5G67580	122.7068	0.645226	0.194175	3.322906	0.000891	0.006529	7.260746	7.226283	7.261186	6.657813	6.520817	6.538339
AT1G76100	2009.602	0.645906	0.13674	4.723615	2.32E-06	2.99E-05	11.16926	11.16464	11.44847	10.75976	10.50601	10.53369
AT5G59250	488.9669	0.646633	0.160349	4.032655	5.52E-05	0.000551	9.303034	9.06459	9.33825	8.781895	8.328941	8.506528

AT1G47530	552.7655	0.647321	0.174038	3.719423	0.0002	0.001743	9.657975	9.208012	9.33825	8.957944	8.571102	8.638132
AT5G58330	1269.435	0.647502	0.155326	4.16865	3.06E-05	0.000321	10.36505	10.62444	10.77614	9.867392	10.167	9.794057
AT5G39830	186.4719	0.64937	0.182563	3.556967	0.000375	0.003034	7.67343	7.891577	7.990057	7.215076	7.013727	7.240127
AT4G28080	2987.332	0.649852	0.167112	3.888726	0.000101	0.000952	11.61243	12.10513	11.75267	11.22328	11.35475	10.91656
AT4G10340	38566.53	0.650064	0.160105	4.060227	4.90E-05	0.000494	15.47074	15.34357	15.74696	15.12582	14.6587	14.76299
AT2G29290	702.6406	0.650692	0.178501	3.645312	0.000267	0.002247	9.621424	9.736544	9.907916	9.415642	8.912407	8.831712
AT1G12250	225.6409	0.651914	0.179563	3.630557	0.000283	0.002363	7.991008	8.143859	8.253771	7.667641	7.234523	7.352617
AT1G77930	296.1011	0.652038	0.148056	4.404008	1.06E-05	0.000122	8.539334	8.521787	8.499668	7.94351	7.672573	7.850924
AT3G04255	123.0733	0.652291	0.195258	3.340668	0.000836	0.00617	7.211152	7.245585	7.271923	6.623396	6.679627	6.473473
AT5G02502	135.3594	0.652614	0.203519	3.206651	0.001343	0.009249	7.498428	7.181886	7.508196	6.740426	6.520817	6.798784
AT1G06680	22002.25	0.65292	0.144606	4.51516	6.33E-06	7.60E-05	14.53906	14.63249	14.95604	14.20541	14.02451	13.91753
AT5G47640	198.446	0.652968	0.18055	3.61656	0.000299	0.00248	8.07103	7.948634	7.86445	7.347485	6.822676	7.423016
AT1G32200	382.717	0.653101	0.152356	4.286681	1.81E-05	0.000199	8.821357	8.771905	9.03999	8.300115	8.150563	8.165198
AT4G24090	134.7998	0.653195	0.204699	3.191001	0.001418	0.009684	7.255862	7.360699	7.526244	6.892686	6.679627	6.522394
AT1G31280	121.3365	0.653206	0.200083	3.264667	0.001096	0.007779	7.260746	7.264631	7.183706	6.417857	6.520817	6.703006
AT5G58870	474.1911	0.653401	0.144688	4.515919	6.30E-06	7.57E-05	9.037377	9.322639	9.170518	8.498306	8.611075	8.477876
AT5G20720	2003.462	0.653748	0.128802	5.07562	3.86E-07	5.67E-06	11.187	11.17852	11.4216	10.6908	10.43541	10.64096
AT1G19720	795.5306	0.653791	0.163164	4.006952	6.15E-05	0.000608	9.931165	10.0995	9.757513	9.501056	9.188216	9.055961
AT1G70230	135.8447	0.653864	0.196956	3.319848	0.000901	0.006588	7.231194	7.471408	7.476057	6.803271	6.679627	6.659928
AT3G63410	1543.546	0.654053	0.160042	4.086743	4.37E-05	0.000445	10.72624	10.77981	11.13154	10.42336	10.15353	10.04905
AT4G25080	2521.48	0.654203	0.14881	4.396237	1.10E-05	0.000126	11.55296	11.54342	11.69525	11.20303	10.74033	10.78241
AT1G22750	122.9735	0.654411	0.200486	3.264124	0.001098	0.007786	7.201025	7.20672	7.394891	6.605875	6.342338	6.674431
AT3G62910	166.8919	0.654705	0.204639	3.199323	0.001378	0.009457	7.518954	7.780174	7.813919	7.304685	6.679627	6.888598
AT1G14790	147.3515	0.656743	0.192136	3.418122	0.000631	0.004802	7.506673	7.539457	7.389971	6.756396	7.182411	6.659928
AT1G32990	984.7075	0.656756	0.170375	3.854763	0.000116	0.001074	10.00651	10.17505	10.51487	9.768491	9.385499	9.502512
AT2G43710	1004.558	0.657238	0.139579	4.708718	2.49E-06	3.19E-05	10.3508	10.16432	10.31135	9.786101	9.362265	9.585428
AT3G06510	369.2374	0.657402	0.152435	4.312677	1.61E-05	0.000179	8.688132	8.91891	8.882523	8.076669	8.095267	8.273953
AT2G17695	136.4541	0.657879	0.201334	3.267593	0.001085	0.007708	7.284918	7.342887	7.604778	6.803271	6.520817	6.758512
AT5G18410	376.7436	0.658503	0.151447	4.348064	1.37E-05	0.000154	8.827953	8.874656	8.920548	8.374432	7.883112	8.18052
AT3G05350	417.0155	0.659989	0.149908	4.402622	1.07E-05	0.000123	8.842684	9.096831	9.087865	8.369248	8.177436	8.39711
AT1G31180	305.2555	0.66003	0.168285	3.922095	8.78E-05	0.000843	8.54735	8.479616	8.701774	8.063893	7.47011	7.937644
AT2G47490	121.8569	0.661168	0.19916	3.319779	0.000901	0.006588	7.211152	7.216535	7.308884	6.4378	6.520817	6.688789
AT1G18170	193.3275	0.66126	0.203562	3.248439	0.00116	0.008174	7.666086	7.848043	8.170152	7.389052	7.128345	7.096741

AT3G55610	1281.468	0.662128	0.127911	5.176457	2.26E-07	3.44E-06	10.72712	10.57141	10.53951	9.889283	10.13309	9.843024
AT3G52960	515.516	0.662958	0.161896	4.094953	4.22E-05	0.000431	9.148258	9.18695	9.558133	8.664088	8.687846	8.558274
AT5G60600	2257.491	0.663844	0.138351	4.79826	1.60E-06	2.11E-05	11.21589	11.5383	11.53764	10.83595	10.8362	10.61582
AT4G33680	697.2473	0.664534	0.159658	4.162246	3.15E-05	0.000329	9.569235	9.7619	9.925023	9.166539	8.742856	9.195734
AT5G03650	517.5878	0.665958	0.147808	4.505559	6.62E-06	7.92E-05	9.223975	9.330569	9.40683	8.824179	8.550692	8.502469
AT4G34530	473.8258	0.66643	0.159691	4.173237	3.00E-05	0.000316	8.991111	9.268234	9.305966	8.676731	8.444073	8.379515
AT1G72640	245.1259	0.667555	0.163473	4.083589	4.43E-05	0.000451	8.211329	8.259933	8.242898	7.684432	7.63429	7.379421
AT1G36310	159.1728	0.668223	0.192555	3.470302	0.00052	0.004054	7.531131	7.637693	7.667108	6.657813	7.128345	7.053013
AT3G59970	1154.917	0.668459	0.132584	5.041762	4.61E-07	6.68E-06	10.54345	10.41771	10.48749	9.910848	9.517558	9.882596
AT1G63610	203.3422	0.668632	0.190981	3.501038	0.000463	0.003667	7.887464	8.039905	8.078333	7.580621	6.822676	7.240127
AT2G25605	125.5748	0.669097	0.200564	3.336067	0.00085	0.006256	7.275298	7.161707	7.433656	6.623396	6.520817	6.615524
AT1G62960	372.4774	0.66921	0.163219	4.100083	4.13E-05	0.000422	8.841054	8.646826	9.043127	8.157066	8.066803	8.230449
AT4G09010	1583.667	0.670095	0.134966	4.964911	6.87E-07	9.70E-06	10.85775	10.86807	11.05435	10.38792	10.28907	10.05737
AT3G08740	649.0906	0.670358	0.134588	4.980833	6.33E-07	8.98E-06	9.565293	9.567577	9.796144	8.995433	8.928315	8.958595
AT2G20930	166.0928	0.670596	0.198924	3.371115	0.000749	0.005584	7.695238	7.491758	7.902948	6.990448	6.752923	7.139183
AT5G49910	511.2012	0.671754	0.137199	4.896186	9.77E-07	1.35E-05	9.304217	9.400044	9.218483	8.599158	8.48767	8.724293
AT3G57645	184.3922	0.673808	0.171812	3.921769	8.79E-05	0.000844	7.7622	7.863742	7.89255	7.094621	7.128345	7.210579
AT3G55040	219.216	0.675749	0.185352	3.645765	0.000267	0.002245	8.008533	7.989277	8.291194	7.304685	7.182411	7.592961
AT5G11450	275.6774	0.675875	0.164197	4.116253	3.85E-05	0.000396	8.397993	8.373952	8.535751	7.749708	7.380437	7.894935
AT1G80030	260.8486	0.675993	0.167351	4.039375	5.36E-05	0.000537	8.213849	8.444552	8.368259	7.812159	7.51294	7.554276
AT1G10830	127.6669	0.676154	0.201882	3.349257	0.00081	0.006008	7.255862	7.181886	7.476057	6.605875	6.679627	6.585144
AT5G16010	379.2745	0.676871	0.148965	4.543827	5.52E-06	6.69E-05	8.956916	8.82259	8.871976	8.353586	8.008134	8.123525
AT1G48030	754.2599	0.677013	0.159325	4.249257	2.14E-05	0.000232	9.556384	10.00001	10.00362	9.204868	9.12059	9.183111
AT4G25890	234.945	0.677415	0.173273	3.909517	9.25E-05	0.000883	8.211329	8.104758	8.29383	7.41946	7.234523	7.703156
AT3G12560	167.9779	0.678172	0.211423	3.207649	0.001338	0.009222	7.490135	7.992137	7.671169	7.156105	6.602406	7.085933
AT1G68590	641.8189	0.678311	0.168006	4.037428	5.40E-05	0.000541	9.587814	9.459111	9.854452	9.163548	8.687846	8.882563
AT5G01220	246.3908	0.679152	0.164372	4.131806	3.60E-05	0.000372	8.201204	8.209221	8.395516	7.650652	7.33342	7.608149
AT1G73110	184.4846	0.680608	0.208922	3.25771	0.001123	0.007942	7.643829	8.009177	7.909839	7.459032	6.822676	6.973146
AT3G20790	193.8463	0.681269	0.188017	3.623436	0.000291	0.002421	7.741388	7.89769	8.108595	7.35799	7.072174	7.149602
AT3G21755	230.0771	0.681325	0.200607	3.39631	0.000683	0.005159	7.893765	8.206761	8.400417	7.650652	7.072174	7.490138
AT2G34640	172.666	0.681964	0.186019	3.666096	0.000246	0.002098	7.647563	7.87308	7.707214	7.179983	7.072174	6.888598
AT4G15560	5908.158	0.682761	0.113439	6.018739	1.76E-09	3.54E-08	12.79772	12.84661	12.85788	12.30025	12.04725	12.05981
AT3G25805	139.9566	0.682915	0.20007	3.413379	0.000642	0.004877	7.447939	7.259893	7.600526	6.787815	6.822676	6.645278

AT3G54720	223.8962	0.683375	0.172703	3.956935	7.59E-05	0.000737	8.017217	8.128345	8.126453	7.282799	7.78168	7.278607
AT5G46800	369.1173	0.683473	0.159467	4.285977	1.82E-05	0.0002	8.831239	8.648639	9.017836	8.169047	8.12318	8.123525
AT1G07600	738.4796	0.684166	0.13106	5.220269	1.79E-07	2.75E-06	9.858934	9.881626	9.799859	9.275834	8.896321	9.172933
AT3G61080	171.1631	0.684515	0.208716	3.279654	0.001039	0.007431	7.822893	7.663037	7.761553	7.378772	6.822676	6.744835
AT1G61190	420.9381	0.684518	0.142358	4.808429	1.52E-06	2.02E-05	9.061365	9.1061	8.935819	8.440172	8.177436	8.320801
AT3G23805	126.0031	0.684984	0.212104	3.229477	0.00124	0.008643	7.443651	7.099419	7.389971	6.533581	6.342338	6.772062
AT1G51590	172.3862	0.686148	0.175643	3.906502	9.36E-05	0.000894	7.706019	7.83854	7.699282	7.030399	7.013727	7.075043
AT2G37660	1013.213	0.687249	0.143062	4.803848	1.56E-06	2.06E-05	10.17183	10.20802	10.49266	9.720453	9.474868	9.55636
AT3G53260	442.2057	0.689148	0.140849	4.892822	9.94E-07	1.37E-05	9.139005	9.087502	9.032117	8.400072	8.591227	8.249946
AT2G46915	190.458	0.689935	0.190076	3.629777	0.000284	0.002369	7.903165	7.83854	7.96381	7.459032	7.013727	6.973146
AT2G41720	159.4267	0.690576	0.214869	3.213944	0.001309	0.009059	7.443651	7.806407	7.75008	7.168094	6.244072	7.019324
AT4G35250	620.696	0.690628	0.131414	5.255345	1.48E-07	2.32E-06	9.504833	9.53559	9.706981	8.887182	8.944051	8.844594
AT1G77590	241.7954	0.690779	0.181877	3.798061	0.000146	0.001317	8.07103	8.186929	8.462658	7.642081	7.284819	7.554276
AT4G32260	2450.272	0.690935	0.154843	4.462174	8.11E-06	9.53E-05	11.48572	11.37891	11.81296	11.05191	10.76268	10.74682
AT5G17310	178.6578	0.691888	0.190233	3.637062	0.000276	0.002311	7.83604	7.882358	7.795434	7.282799	6.434336	7.220496
AT4G30960	2425.886	0.692546	0.13257	5.223992	1.75E-07	2.70E-06	11.36251	11.69985	11.58791	10.94536	10.71762	10.86691
AT1G48480	198.3901	0.692821	0.169721	4.08212	4.46E-05	0.000453	7.937117	8.053658	7.89255	7.304685	7.072174	7.278607
AT1G18735	214.2183	0.692873	0.18492	3.746885	0.000179	0.001583	7.906284	8.072696	8.212567	7.553458	7.234523	7.200593
AT3G27925	365.1409	0.693321	0.188964	3.66906	0.000243	0.002077	8.636466	8.684441	9.101472	8.227502	8.255176	7.863636
AT1G65295	128.5375	0.693661	0.215736	3.215325	0.001303	0.009024	7.535168	7.191871	7.228488	6.772191	6.679627	6.38806
AT2G15620	863.2947	0.693867	0.184241	3.766074	0.000166	0.001481	9.66628	10.18446	10.29367	9.533855	9.214403	9.274004
AT4G30620	159.2791	0.694015	0.192126	3.612299	0.000303	0.002513	7.7622	7.600697	7.561677	7.11953	6.822676	6.772062
AT1G75460	498.4671	0.69471	0.158548	4.381713	1.18E-05	0.000134	9.215196	9.168107	9.388447	8.549613	8.846958	8.329991
AT3G53920	197.7928	0.695109	0.176738	3.932988	8.39E-05	0.000807	7.839308	8.107398	7.882077	7.260576	7.234523	7.210579
AT5G50280	121.066	0.695655	0.203815	3.413167	0.000642	0.004879	7.201025	7.29737	7.277261	6.740426	6.342338	6.405552
AT1G70760	731.5404	0.695656	0.168822	4.120652	3.78E-05	0.000389	9.67728	9.808071	10.00844	9.395388	8.8636	8.996603
AT1G67860	275.8711	0.696323	0.210112	3.314052	0.00092	0.006713	7.991008	8.531648	8.695807	7.650652	7.594963	7.831643
AT4G12980	255.8357	0.696446	0.168795	4.125995	3.69E-05	0.00038	8.133597	8.406472	8.405301	7.692755	7.554534	7.546413
AT2G04955	415.1657	0.696662	0.160688	4.33548	1.45E-05	0.000163	8.858877	9.017646	9.11793	8.063893	8.508985	8.352713
AT3G08920	421.1215	0.696792	0.179106	3.890383	0.0001	0.000947	8.920349	8.954535	9.224022	8.585813	8.229725	8.075159
AT4G03110	437.7231	0.697168	0.152806	4.562443	5.06E-06	6.15E-05	9.097299	8.974917	9.18337	8.186833	8.42177	8.514611
AT5G02840	1585.093	0.697586	0.151927	4.591598	4.40E-06	5.41E-05	11.14602	10.73613	10.92556	10.41207	10.15353	10.10097
AT5G13400	151.1959	0.698551	0.187004	3.735499	0.000187	0.001648	7.502556	7.503831	7.687301	6.787815	6.822676	6.913264

AT5G40160	174.0328	0.701195	0.197845	3.544165	0.000394	0.003169	7.514872	7.796625	7.976993	7.069274	6.952811	7.075043
AT4G10360	100.8737	0.701819	0.211743	3.314483	0.000918	0.006704	6.958103	6.983493	6.976157	6.313781	6.434336	6.096513
AT2G21210	198.4962	0.702036	0.169409	4.144022	3.41E-05	0.000354	7.964314	7.900737	8.031715	7.347485	7.072174	7.220496
AT3G23340	115.971	0.702789	0.212686	3.304349	0.000952	0.006899	7.03992	7.25514	7.28258	6.657813	6.342338	6.315891
AT3G21750	142.8944	0.703007	0.219955	3.196144	0.001393	0.009546	7.250962	7.666621	7.530721	7.017205	6.679627	6.50627
AT1G15410	197.2005	0.704449	0.219309	3.212129	0.001318	0.009096	7.709596	8.151554	8.04116	7.553458	6.602406	7.200593
AT4G33000	174.4329	0.704797	0.183056	3.850174	0.000118	0.001093	7.806288	7.65223	7.89255	7.179983	6.822676	7.041871
AT1G64370	742.9904	0.705666	0.1586	4.449344	8.61E-06	0.000101	9.580021	9.978538	9.989865	9.196113	8.959616	9.200752
AT2G46820	3343.693	0.706455	0.136145	5.188997	2.11E-07	3.23E-06	11.8732	11.9268	12.2415	11.39888	11.27612	11.22931
AT5G37360	338.4544	0.706495	0.157916	4.473874	7.68E-06	9.06E-05	8.588717	8.700266	8.88077	8.101887	7.915398	7.955567
AT1G79075	185.5259	0.70653	0.18327	3.855122	0.000116	0.001073	7.748358	7.885438	7.98027	7.043474	6.952811	7.316087
AT3G50685	306.6175	0.708622	0.197861	3.581407	0.000342	0.002798	8.481941	8.452879	8.799622	8.138907	7.816289	7.530557
AT5G08280	1079.705	0.709297	0.149686	4.738577	2.15E-06	2.79E-05	10.16923	10.35633	10.61787	9.699955	9.732137	9.585428
AT1G07700	267.9707	0.709794	0.177587	3.996887	6.42E-05	0.000632	8.569166	8.285802	8.335368	7.544288	7.47011	7.876237
AT3G53750	96.46827	0.710488	0.222301	3.196068	0.001393	0.009546	7.005422	6.954462	6.962967	6.377123	5.450922	6.334274
AT1G11860	3262.789	0.712667	0.138721	5.137405	2.79E-07	4.17E-06	11.863	11.96569	12.13122	11.46354	11.19625	11.10434
AT3G01480	875.8204	0.712679	0.145453	4.899725	9.60E-07	1.33E-05	9.962225	10.05916	10.25834	9.540787	9.240123	9.281129
AT5G44930	116.5839	0.713901	0.208994	3.415884	0.000636	0.004836	7.034228	7.28343	7.314087	6.551997	6.244072	6.489965
AT1G64390	173.25	0.713954	0.199903	3.571499	0.000355	0.002888	7.755295	7.857483	7.79915	7.069274	6.342338	7.316087
AT3G16010	153.7103	0.714077	0.183955	3.88181	0.000104	0.000976	7.527084	7.570402	7.703253	6.907064	6.752923	6.876105
AT3G21760	218.9492	0.715385	0.200159	3.574078	0.000351	0.002863	7.819587	8.141285	8.350642	7.507017	7.072174	7.397017
AT5G44650	288.4063	0.715781	0.156629	4.569909	4.88E-06	5.96E-05	8.527226	8.545342	8.453256	7.936518	7.554534	7.717232
AT5G02940	263.0635	0.716338	0.194034	3.691809	0.000223	0.00192	8.203742	8.593214	8.32767	7.908207	7.284819	7.538507
AT1G23950	176.3463	0.717285	0.184181	3.894449	9.84E-05	0.000932	7.751831	7.783479	7.828538	6.818564	7.128345	7.210579
AT1G09193	474.5436	0.71794	0.175188	4.098122	4.17E-05	0.000425	9.004274	9.165576	9.455912	8.680921	8.352714	8.348197
AT3G47500	470.2156	0.718538	0.157106	4.573587	4.79E-06	5.86E-05	9.197478	9.229982	9.173384	8.680921	8.466036	8.2206
AT4G02770	13262.35	0.718822	0.150044	4.790733	1.66E-06	2.19E-05	13.8611	13.91305	14.24448	13.48548	13.20643	13.1266
AT3G24590	160.8	0.719791	0.195454	3.682665	0.000231	0.001984	7.578837	7.611896	7.802856	7.131825	6.822676	6.744835
AT2G00550	113.8799	0.721583	0.204083	3.535727	0.000407	0.003257	7.062469	7.196838	7.261186	6.417857	6.342338	6.473473
AT1G67870	811.0679	0.722981	0.217635	3.321994	0.000894	0.006549	9.568251	10.38708	9.929268	9.531537	8.912407	9.172933
AT2G33470	259.8097	0.723011	0.156498	4.619927	3.84E-06	4.78E-05	8.391329	8.271749	8.38319	7.692755	7.594963	7.538507
AT1G04420	542.1704	0.723156	0.156559	4.619053	3.85E-06	4.80E-05	9.269508	9.434092	9.523735	8.883551	8.550692	8.518635
AT3G56825	3686.666	0.723842	0.146608	4.937265	7.92E-07	1.11E-05	12.14008	12.17585	12.20695	11.60254	11.08	11.54262

AT1G74470	15145.2	0.727254	0.150595	4.82919	1.37E-06	1.85E-05	14.1947	14.09448	14.33481	13.74697	13.31856	13.28841
AT1G79560	285.2246	0.728359	0.168397	4.325243	1.52E-05	0.000169	8.268211	8.589442	8.583937	7.796798	7.709866	7.703156
AT3G21250	466.9672	0.728797	0.170917	4.264041	2.01E-05	0.000218	9.128357	9.441434	8.985587	8.321742	8.42177	8.581546
AT2G28740	142.3836	0.728797	0.220877	3.299555	0.000968	0.00699	7.275298	7.790067	7.419241	6.9213	6.434336	6.717084
AT5G48900	673.3383	0.729328	0.153617	4.747699	2.06E-06	2.67E-05	9.492431	9.718526	9.930962	9.015478	8.912407	8.987921
AT4G30950	2191.253	0.729973	0.140511	5.195124	2.05E-07	3.13E-06	11.32512	11.32095	11.60897	10.85461	10.55807	10.59501
AT1G16080	402.0583	0.729979	0.169463	4.3076	1.65E-05	0.000183	8.847561	8.969123	9.131258	8.474367	8.095267	8.058669
AT3G14810	162.3688	0.730339	0.196613	3.714603	0.000204	0.001772	7.543206	7.900737	7.561677	6.935398	6.952811	6.900984
AT4G13500	310.9028	0.731155	0.167963	4.35308	1.34E-05	0.000151	8.598394	8.515837	8.701774	8.037996	7.915398	7.623179
AT2G31320	96.09205	0.731463	0.21861	3.345965	0.00082	0.006067	6.799356	7.017576	6.949654	6.224753	6.244072	6.074811
AT1G29530	86.10834	0.733493	0.226371	3.240229	0.001194	0.008369	6.673172	6.7534	6.838487	5.861135	6.244072	6.007675
AT3G54660	670.6039	0.733979	0.128866	5.695666	1.23E-08	2.22E-07	9.700828	9.686222	9.778371	9.102387	8.846958	8.9226
AT4G00050	108.8582	0.734074	0.229295	3.201443	0.001367	0.009397	7.36873	6.851144	7.071421	6.377123	6.342338	6.315891
AT4G01690	847.2244	0.734514	0.13436	5.466738	4.58E-08	7.70E-07	9.934225	10.05643	10.17357	9.438093	9.201369	9.249998
AT1G11720	236.1486	0.734853	0.183207	4.011062	6.04E-05	0.0006	8.128263	8.404326	8.152831	7.692755	7.182411	7.388246
AT4G11175	199.3601	0.737132	0.185164	3.98097	6.86E-05	0.000671	7.906284	7.95451	8.117552	7.459032	6.88921	7.149602
AT1G69160	383.7828	0.73954	0.200748	3.683922	0.00023	0.001976	9.263438	8.708983	8.748648	8.38987	7.915398	8.069683
AT2G35370	1259.027	0.739919	0.149978	4.933502	8.08E-07	1.13E-05	10.57712	10.59935	10.70908	10.1228	9.802877	9.641866
AT5G01590	382.4284	0.740666	0.176884	4.187305	2.82E-05	0.000299	8.590658	9.016242	9.087865	8.101887	8.150563	8.200699
AT2G40490	776.2878	0.741766	0.139403	5.321021	1.03E-07	1.65E-06	9.829655	9.873894	10.09639	9.199037	9.005329	9.274004
AT2G09465	818.0049	0.741852	0.162224	4.573004	4.81E-06	5.88E-05	10.0788	10.04063	9.92842	9.127163	8.959616	9.546538
AT1G31800	317.4981	0.741915	0.189232	3.920673	8.83E-05	0.000847	8.292259	8.811299	8.78283	7.977969	7.883112	7.778931
AT5G04130	94.72106	0.742267	0.215447	3.445244	0.000571	0.004406	6.858537	6.948585	6.908963	6.17809	6.244072	6.052778
AT3G04260	455.908	0.743308	0.17142	4.336187	1.45E-05	0.000162	9.001359	9.308942	9.177672	8.634151	8.376102	8.160054
AT1G06717	174.5571	0.743663	0.194478	3.823899	0.000131	0.001202	7.702435	7.641341	8.009433	7.131825	6.952811	6.949491
AT5G17870	683.7139	0.743674	0.180009	4.131326	3.61E-05	0.000372	9.631828	9.577131	10.0339	9.175475	8.630654	9.044842
AT1G48520	368.2048	0.743891	0.19541	3.806817	0.000141	0.001277	8.575059	8.935347	9.072594	8.369248	7.74622	8.025114
AT5G41140	319.5747	0.746259	0.186311	4.005455	6.19E-05	0.000611	8.355255	8.900763	8.683797	8.004957	7.78168	7.844526
AT3G18390	484.5749	0.747939	0.186639	4.007407	6.14E-05	0.000607	8.979309	9.576178	9.144464	8.572344	8.550692	8.339123
AT1G32470	1372.937	0.748425	0.152756	4.89947	9.61E-07	1.33E-05	10.65847	10.67292	10.93403	10.22676	9.794222	9.895034
AT4G19710	260.7443	0.748706	0.18584	4.028759	5.61E-05	0.00056	8.413425	8.376143	8.407737	7.757664	6.88921	7.798924
AT4G34730	148.4076	0.748992	0.204152	3.668796	0.000244	0.002078	7.469191	7.417115	7.784228	6.9213	6.679627	6.703006
AT5G61130	111.8213	0.749104	0.213349	3.511167	0.000446	0.003545	7.336712	7.072651	7.065262	6.496028	6.244072	6.334274

AT1G78580	164.8433	0.749411	0.210209	3.565083	0.000364	0.00295	7.535168	7.924884	7.570401	6.963185	7.234523	6.659928
AT5G55790	122.1424	0.749474	0.203617	3.680804	0.000233	0.001993	7.34133	7.245585	7.271923	6.335206	6.434336	6.688789
AT1G23730	131.8273	0.751642	0.198709	3.782627	0.000155	0.001395	7.303968	7.412853	7.419241	6.397634	6.752923	6.717084
AT2G25830	128.2237	0.753673	0.202072	3.729723	0.000192	0.001682	7.275298	7.408578	7.428867	6.787815	6.244072	6.522394
AT1G78510	131.4853	0.753741	0.227587	3.311881	0.000927	0.006754	7.391172	7.511824	7.319272	7.003888	6.244072	6.334274
AT2G29360	283.2656	0.754904	0.190484	3.963075	7.40E-05	0.000719	8.313565	8.483686	8.695807	7.984764	7.33342	7.645434
AT2G29320	87.39416	0.755206	0.232818	3.243755	0.00118	0.008284	6.702182	6.712369	6.881184	5.974702	6.434336	5.758224
AT1G17220	1272.994	0.756826	0.136411	5.548144	2.89E-08	4.97E-07	10.49817	10.8097	10.61471	9.940855	9.958166	9.743371
AT5G21222	358.6141	0.757947	0.159422	4.754331	1.99E-06	2.59E-05	8.850803	8.926405	8.663555	8.126672	8.177436	7.876237
AT5G52510	178.856	0.758389	0.206606	3.670693	0.000242	0.002065	7.539193	8.011998	7.94716	7.215076	6.752923	7.041871
AT1G09533	302.6158	0.758528	0.230362	3.292766	0.000992	0.007129	8.467228	8.795016	8.390598	7.336904	8.376102	7.600575
AT1G03310	206.7574	0.75942	0.192256	3.950055	7.81E-05	0.000757	7.809624	8.11791	8.190101	7.44924	7.072174	7.159945
AT1G52220	1462.847	0.759859	0.161435	4.706904	2.52E-06	3.21E-05	10.7637	10.79953	11.00372	10.32774	9.713896	10.08206
AT4G34350	2363.982	0.761585	0.109517	6.954022	3.55E-12	9.29E-11	11.53518	11.50389	11.58711	10.87852	10.74483	10.68191
AT1G66940	372.5301	0.761743	0.174831	4.35703	1.32E-05	0.000149	9.086337	8.628563	8.922253	8.244586	7.946977	8.058669
AT3G54900	638.5509	0.762024	0.153749	4.956275	7.19E-07	1.01E-05	9.594599	9.60168	9.801712	9.096125	8.591227	8.850992
AT2G08330	80.81595	0.76317	0.237483	3.213578	0.001311	0.009065	6.723563	6.57416	6.787021	6.079977	5.90133	5.702706
AT3G56650	269.8357	0.76353	0.182487	4.184029	2.86E-05	0.000302	8.308857	8.32489	8.657427	7.789056	7.284819	7.68894
AT2G03220	77.5587	0.763589	0.233679	3.267677	0.001084	0.007708	6.582468	6.7534	6.628972	5.890376	5.617177	5.913005
AT3G07215	271.6768	0.765279	0.169883	4.504739	6.65E-06	7.95E-05	8.292259	8.34963	8.615892	7.684432	7.709866	7.562097
AT5G16710	562.5772	0.765329	0.159063	4.811495	1.50E-06	2.00E-05	9.271928	9.503881	9.638111	8.85417	8.649971	8.554359
AT5G09650	679.0511	0.7656	0.131366	5.827995	5.61E-09	1.05E-07	9.605197	9.796666	9.829232	8.947549	8.959616	8.996603
AT2G34460	449.4659	0.766114	0.145242	5.274729	1.33E-07	2.10E-06	9.152205	9.02883	9.233666	8.343049	8.571102	8.249946
AT4G02920	976.9067	0.766929	0.149163	5.141561	2.72E-07	4.09E-06	10.33988	10.16622	10.30809	9.710241	9.453039	9.266844
AT5G48930	540.0155	0.767245	0.19645	3.905554	9.40E-05	0.000896	9.79893	9.084825	9.294165	8.554188	8.591227	8.724293
AT5G24300	607.723	0.767819	0.141732	5.417415	6.05E-08	1.00E-06	9.40911	9.609151	9.722718	8.816582	8.846958	8.765518
AT2G37040	146.0736	0.767841	0.197152	3.894666	9.83E-05	0.000931	7.56311	7.519773	7.447929	6.476879	7.128345	6.731026
AT3G52380	610.486	0.768419	0.145987	5.263612	1.41E-07	2.22E-06	9.532354	9.548276	9.713887	9.002146	8.687846	8.696141
AT1G66350	91.37816	0.768793	0.236518	3.250468	0.001152	0.008125	6.673172	6.85743	7.101827	6.224753	5.90133	6.007675
AT1G62180	302.7816	0.769171	0.207934	3.699112	0.000216	0.001873	8.469339	8.768573	8.515565	7.336904	7.850087	8.096855
AT2G25480	79.81248	0.769673	0.23954	3.213123	0.001313	0.009076	6.574646	6.766821	6.661997	6.028299	6.024848	5.615206
AT3G11945	293.7304	0.770369	0.179612	4.289077	1.79E-05	0.000197	8.415616	8.4214	8.792183	7.901041	7.594963	7.703156
AT5G58520	100.5476	0.770699	0.222905	3.457531	0.000545	0.004231	6.915386	6.930809	7.143347	6.335206	6.342338	5.984584

AT5G43740	113.517	0.771183	0.205108	3.759892	0.00017	0.001512	7.270463	7.201787	7.149183	6.533581	6.024848	6.422834
AT2G44920	530.8953	0.772806	0.161032	4.799087	1.59E-06	2.11E-05	9.247536	9.349648	9.599209	8.781895	8.376102	8.581546
AT5G08650	538.2609	0.773144	0.143293	5.39555	6.83E-08	1.12E-06	9.354194	9.385979	9.467634	8.664088	8.830121	8.423107
AT4G22010	202.5907	0.773336	0.175599	4.403993	1.06E-05	0.000122	7.890617	8.107398	8.081388	7.260576	6.952811	7.325306
AT5G27360	86.40282	0.773807	0.234042	3.306273	0.000945	0.006865	6.792628	6.876128	6.686279	5.70532	6.13862	6.138962
AT2G22330	254.8286	0.774076	0.234255	3.304414	0.000952	0.006899	7.921783	8.686208	8.388133	7.44924	7.128345	7.850924
AT2G32560	324.0841	0.774133	0.148004	5.230495	1.69E-07	2.62E-06	8.713288	8.66844	8.715604	8.024871	7.672573	7.901114
AT3G19710	424.3434	0.774286	0.18966	4.082501	4.46E-05	0.000453	8.855653	9.366276	8.957596	8.044514	8.304769	8.457056
AT1G05057	1838.248	0.774661	0.15095	5.131922	2.87E-07	4.29E-06	11.06807	11.10381	11.38281	10.55702	10.11929	10.44976
AT1G05063	1838.248	0.774661	0.15095	5.131922	2.87E-07	4.29E-06	11.06807	11.10381	11.38281	10.55702	10.11929	10.44976
AT4G25700	205.2836	0.774719	0.198855	3.895909	9.78E-05	0.000928	7.943205	7.841715	8.267248	7.003888	7.33342	7.361607
AT5G55400	88.24489	0.774817	0.229404	3.377518	0.000731	0.00547	6.902945	6.812837	6.787021	6.247531	5.90133	5.83769
AT5G44190	989.3787	0.774867	0.14722	5.263318	1.41E-07	2.22E-06	10.30898	10.29802	10.30352	9.73463	9.161545	9.504543
AT4G38950	229.8965	0.776067	0.200914	3.862682	0.000112	0.001045	8.366626	8.184431	7.930315	7.326244	7.78168	7.128689
AT5G65460	205.5319	0.776385	0.173651	4.470956	7.79E-06	9.17E-05	7.896905	8.107398	8.087478	7.326244	7.234523	7.149602
AT5G03985	1287.346	0.776434	0.164153	4.729954	2.25E-06	2.91E-05	10.49402	10.59794	10.90501	10.10107	9.820033	9.676501
AT3G04890	163.236	0.778678	0.219342	3.550069	0.000385	0.003108	7.786107	7.519773	7.835792	7.249335	6.679627	6.615524
AT3G07860	264.5583	0.778726	0.160052	4.86545	1.14E-06	1.56E-05	8.382395	8.36956	8.4928	7.667641	7.284819	7.710211
AT3G22790	176.903	0.778909	0.20098	3.875549	0.000106	0.000997	7.713163	8.059123	7.75008	7.168094	6.520817	7.128689
AT2G44040	110.0984	0.779296	0.22613	3.446228	0.000568	0.004392	6.909179	7.083418	7.344921	6.313781	6.602406	6.159727
AT2G29980	629.0277	0.77998	0.138101	5.647876	1.62E-08	2.87E-07	9.731053	9.491808	9.699047	8.940576	8.795846	8.792361
AT5G63380	86.803	0.780439	0.229166	3.405557	0.00066	0.005002	6.909179	6.691408	6.888179	6.028299	5.617177	6.138962
AT3G56705	2607.001	0.781491	0.138333	5.649342	1.61E-08	2.85E-07	11.58787	11.66242	11.8285	11.01089	10.63278	10.99448
AT3G24430	497.4144	0.782538	0.186609	4.193461	2.75E-05	0.000292	9.033102	9.315806	9.563605	8.726222	8.177436	8.490225
AT4G36540	1034.518	0.784042	0.130279	6.018157	1.76E-09	3.55E-08	10.42935	10.34351	10.32494	9.736644	9.373929	9.522691
AT3G23400	558.081	0.785071	0.143873	5.456705	4.85E-08	8.13E-07	9.336956	9.421418	9.622466	8.655598	8.795846	8.593042
AT2G28550	201.1326	0.785158	0.184773	4.249303	2.14E-05	0.000232	8.068248	7.997839	7.96381	7.439382	7.128345	6.973146
AT3G02510	84.15514	0.785456	0.237289	3.310124	0.000933	0.006786	6.658445	6.819293	6.816654	6.17809	5.90133	5.702706
AT1G17100	107.0533	0.785901	0.217484	3.613608	0.000302	0.002504	7.159792	6.906762	7.24493	6.269954	6.244072	6.334274
AT5G51100	97.32347	0.785951	0.245382	3.202967	0.00136	0.009355	6.723563	6.924835	7.287879	6.313781	5.766238	6.180197
AT2G04030	974.2929	0.788423	0.140766	5.600946	2.13E-08	3.73E-07	10.05159	10.42302	10.33137	9.472338	9.442	9.498443
AT1G52870	540.4203	0.7891	0.142991	5.518537	3.42E-08	5.82E-07	9.508944	9.354101	9.408047	8.758298	8.649971	8.457056
AT4G32190	295.9047	0.789339	0.16303	4.841692	1.29E-06	1.74E-05	8.573098	8.65226	8.407737	7.773445	7.946977	7.592961

AT2G15290	249.7773	0.790598	0.177902	4.443999	8.83E-06	0.000103	8.154739	8.285802	8.517822	7.642081	7.380437	7.448554
AT2G20240	83.78346	0.791726	0.24858	3.184998	0.001448	0.009856	6.723563	6.780119	6.838487	6.313781	5.617177	5.644966
AT3G16560	88.65032	0.791857	0.239512	3.306128	0.000946	0.006866	6.958103	6.900687	6.787021	6.356317	5.262981	5.984584
AT3G58670	156.7788	0.794167	0.19463	4.080392	4.50E-05	0.000456	7.7622	7.558875	7.587692	6.935398	7.013727	6.600414
AT5G08130	923.4395	0.795344	0.12092	6.577416	4.79E-11	1.13E-09	10.13772	10.17757	10.27778	9.462636	9.408366	9.311602
AT5G54270	44410.71	0.796168	0.175485	4.536967	5.71E-06	6.90E-05	15.71852	15.57912	16.03802	15.28132	14.71682	14.85136
AT2G32230	163.7983	0.796227	0.198696	4.00726	6.14E-05	0.000608	7.636334	7.857483	7.646629	7.107129	6.88921	6.659928
AT3G55240	599.1234	0.797858	0.149768	5.327294	9.97E-08	1.60E-06	9.722229	9.427769	9.585288	8.92653	8.669033	8.663799
AT2G21340	358.9559	0.798881	0.158708	5.033658	4.81E-07	6.96E-06	8.782835	8.795016	8.960918	8.215999	7.915398	7.894935
AT4G28730	135.8254	0.799472	0.206046	3.880071	0.000104	0.000982	7.386711	7.356267	7.663036	6.756396	6.244072	6.688789
AT4G17870	328.1578	0.799879	0.166502	4.804014	1.56E-06	2.06E-05	8.912613	8.535574	8.679771	7.857284	7.850087	7.961492
AT5G10690	157.1973	0.800437	0.205386	3.897223	9.73E-05	0.000924	7.832764	7.574224	7.587692	7.094621	6.679627	6.630478
AT3G50340	171.9858	0.800802	0.223839	3.577584	0.000347	0.002832	7.494287	7.756823	8.096566	7.203473	6.752923	6.811962
AT5G44520	209.6437	0.801518	0.182656	4.388138	1.14E-05	0.000131	8.028714	8.056393	8.178736	7.507017	6.952811	7.139183
AT1G63360	230.9194	0.801603	0.168258	4.76412	1.90E-06	2.48E-05	8.223887	8.290456	8.069129	7.389052	7.554534	7.278607
AT2G28000	1745.213	0.805119	0.127732	6.303191	2.92E-10	6.32E-09	11.03674	11.08785	11.23358	10.44447	10.28907	10.164
AT4G13840	272.2698	0.805349	0.188505	4.272298	1.93E-05	0.000211	8.395775	8.672011	8.253771	7.812159	7.594963	7.423016
AT2G42610	88.26617	0.806177	0.23748	3.394717	0.000687	0.005183	7.062469	6.670137	6.809303	6.15418	5.766238	5.961117
AT5G25630	415.2678	0.806218	0.188772	4.270856	1.95E-05	0.000213	8.81639	9.303196	9.052497	8.479187	7.883112	8.185591
AT3G02690	453.0579	0.806663	0.156794	5.144727	2.68E-07	4.03E-06	9.235183	8.997863	9.316376	8.498306	8.177436	8.329991
AT2G27820	136.3448	0.807354	0.210594	3.833698	0.000126	0.001162	7.443651	7.292738	7.687301	6.756396	6.244072	6.688789
AT3G52750	171.431	0.808425	0.184035	4.392777	1.12E-05	0.000128	7.796233	7.691466	7.902948	7.107129	6.752923	6.913264
AT3G52535	63.76227	0.808619	0.252944	3.196834	0.001389	0.009526	6.502267	6.315674	6.373842	5.672031	5.262981	5.522054
AT1G20850	162.0411	0.809082	0.210261	3.847996	0.000119	0.001101	7.551201	7.945686	7.687301	6.907064	6.342338	7.096741
AT2G29450	541.193	0.809355	0.137735	5.876172	4.20E-09	8.06E-08	9.496577	9.51287	9.284919	8.599158	8.630654	8.615763
AT5G58140	1356.685	0.809921	0.157382	5.146219	2.66E-07	4.00E-06	10.73416	10.81132	10.75094	10.23675	9.776754	9.712081
AT4G22830	92.88643	0.810443	0.237428	3.41342	0.000642	0.004877	6.716471	6.882307	7.17229	6.129868	5.766238	6.159727
AT3G10520	348.4846	0.81112	0.189596	4.278155	1.88E-05	0.000206	8.617555	8.857509	8.983955	8.244586	7.47011	7.984953
AT3G46540	153.7536	0.811627	0.190544	4.259515	2.05E-05	0.000222	7.792865	7.574224	7.539633	6.848671	6.679627	6.811962
AT3G47560	219.5554	0.811938	0.190227	4.268259	1.97E-05	0.000215	8.198661	8.056393	8.242898	7.607282	6.822676	7.249844
AT2G41680	407.2305	0.812127	0.144876	5.605648	2.07E-08	3.63E-07	8.932642	9.060509	9.071058	8.278159	8.280186	8.064187
AT1G18730	581.544	0.812339	0.168737	4.814232	1.48E-06	1.98E-05	9.354194	9.586623	9.683047	8.961393	8.591227	8.514611
AT2G40020	80.05666	0.814189	0.244227	3.333736	0.000857	0.006305	6.896684	6.691408	6.586597	6.105138	5.450922	5.785202

AT1G63940	319.0251	0.815433	0.161639	5.044783	4.54E-07	6.59E-06	8.48403	8.783507	8.76394	7.849861	7.78168	7.882497
AT1G29500	320.4338	0.816378	0.183218	4.455776	8.36E-06	9.78E-05	8.94483	8.519806	8.605319	8.024871	7.554534	7.850924
AT5G35790	1101.877	0.816615	0.126857	6.437302	1.22E-10	2.72E-09	10.40474	10.50939	10.48057	9.793859	9.548767	9.52869
AT5G22880	225.3352	0.818331	0.166598	4.912009	9.01E-07	1.25E-05	8.065459	8.283469	8.187268	7.378772	7.33342	7.316087
AT2G30520	3413.139	0.818667	0.131019	6.248463	4.15E-10	8.83E-09	12.30163	11.91931	12.03602	11.28514	11.22873	11.26927
AT4G39710	212.0724	0.818681	0.211977	3.86212	0.000112	0.001047	7.884303	7.95744	8.407737	7.459032	7.182411	7.053013
AT5G18010	183.9913	0.819065	0.220832	3.708996	0.000208	0.00181	8.042958	7.746698	7.96381	7.44924	6.434336	6.913264
AT1G68660	314.3432	0.819834	0.149619	5.479479	4.27E-08	7.20E-07	8.619458	8.663067	8.719531	7.94351	7.672573	7.792291
AT1G49380	223.4269	0.821204	0.18277	4.493094	7.02E-06	8.36E-05	8.203742	8.174394	8.22367	7.589563	6.752923	7.352617
AT4G27080	129.7023	0.822724	0.235626	3.49165	0.00048	0.003779	7.523025	7.306589	7.49451	6.892686	5.450922	6.630478
AT5G18050	488.3873	0.822924	0.204748	4.019209	5.84E-05	0.00058	9.434183	9.06866	9.40195	8.850455	8.066803	8.235348
AT2G30695	135.4171	0.82384	0.211391	3.897229	9.73E-05	0.000924	7.395619	7.25514	7.671169	6.674719	6.679627	6.473473
AT3G52150	980.022	0.824599	0.157705	5.228727	1.71E-07	2.65E-06	10.03956	10.2957	10.53283	9.505788	9.314642	9.514653
AT1G43560	184.0426	0.825568	0.214218	3.853875	0.000116	0.001077	7.605954	7.790067	8.198567	6.907064	7.234523	7.019324
AT1G12900	4542.068	0.825753	0.141279	5.844853	5.07E-09	9.62E-08	12.33804	12.4922	12.6805	11.85562	11.59544	11.52872
AT5G48910	111.7964	0.82717	0.232414	3.559041	0.000372	0.003013	6.964104	7.306589	7.349997	6.605875	5.766238	6.29727
AT3G14930	816.912	0.827335	0.125009	6.618179	3.64E-11	8.70E-10	10.03387	10.01344	10.08044	9.32485	9.02025	9.185645
AT5G23820	233.3462	0.828229	0.174293	4.751942	2.01E-06	2.62E-05	8.373405	8.166821	8.231942	7.497547	6.952811	7.506442
AT4G25370	464.1146	0.828617	0.149854	5.529513	3.21E-08	5.48E-07	9.087712	9.183201	9.364821	8.420261	8.48767	8.249946
AT1G56570	479.2216	0.829644	0.134117	6.185951	6.17E-10	1.29E-08	9.226473	9.245644	9.345892	8.503046	8.328941	8.410167
AT1G73720	231.6812	0.831917	0.171729	4.844366	1.27E-06	1.72E-05	8.322934	8.141285	8.242898	7.562569	7.182411	7.288068
AT1G23970	65.82319	0.832027	0.26096	3.188337	0.001431	0.009769	6.298895	6.36945	6.603697	5.737858	5.617177	5.315499
AT2G35840	301.1078	0.832554	0.169089	4.923769	8.49E-07	1.18E-05	8.426522	8.803181	8.562234	7.733664	7.709866	7.792291
AT3G42640	80.3635	0.832631	0.243327	3.421862	0.000622	0.004761	6.933848	6.655781	6.551779	5.947137	5.766238	5.785202
AT1G48300	2140.602	0.832745	0.194708	4.276881	1.90E-05	0.000207	11.6887	11.46531	11.09233	10.88399	10.21319	10.51266
AT1G04680	317.2264	0.833784	0.152175	5.479095	4.28E-08	7.21E-07	8.594531	8.623039	8.78283	7.765576	7.946977	7.805528
AT4G08520	223.3273	0.834719	0.17496	4.770919	1.83E-06	2.40E-05	8.008533	8.283469	8.242898	7.429455	7.182411	7.288068
AT2G43910	675.6084	0.83538	0.137085	6.093902	1.10E-09	2.27E-08	9.860543	9.640475	9.801712	9.041779	8.795846	8.870017
AT5G08260	111.7905	0.835548	0.207804	4.020849	5.80E-05	0.000577	7.190827	7.13608	7.206271	6.129868	6.434336	6.422834
AT1G52827	99.59651	0.83558	0.254456	3.283791	0.001024	0.007337	6.85208	7.115245	7.222966	6.079977	5.262981	6.55411
AT5G23920	133.1977	0.835797	0.210856	3.963831	7.38E-05	0.000717	7.518954	7.347361	7.480693	6.863493	6.342338	6.370353
AT1G76110	339.9339	0.835828	0.142856	5.850825	4.89E-09	9.31E-08	8.801387	8.77024	8.733192	7.922432	8.037767	7.857294
AT4G01460	235.2991	0.835853	0.167653	4.985611	6.18E-07	8.79E-06	8.289872	8.226323	8.288552	7.589563	7.072174	7.370542

AT2G24762	74.99991	0.836102	0.246404	3.393216	0.000691	0.005205	6.723563	6.719289	6.534049	6.001749	5.046837	5.785202
AT5G15850	797.814	0.836239	0.137216	6.094344	1.10E-09	2.26E-08	10.16728	9.902296	9.91735	9.073995	9.252813	9.154947
AT1G06263	120.8588	0.836561	0.20858	4.010755	6.05E-05	0.0006	7.149297	7.25514	7.471407	6.4378	6.434336	6.422834
AT2G44830	101.5178	0.836676	0.229148	3.65125	0.000261	0.002207	6.877737	7.171832	7.046626	6.335206	6.342338	5.888334
AT2G17972	118.533	0.837442	0.221981	3.772587	0.000162	0.001448	7.170211	7.408578	7.293159	6.724278	6.024848	6.239921
AT1G55370	145.1848	0.837958	0.204264	4.10232	4.09E-05	0.000418	7.359654	7.630369	7.703253	6.863493	6.520817	6.615524
AT1G02380	129.1293	0.83914	0.202785	4.138079	3.50E-05	0.000363	7.473404	7.329382	7.466742	6.570181	6.024848	6.717084
AT2G04375	2411.258	0.840389	0.166232	5.055527	4.29E-07	6.25E-06	11.54572	11.58947	11.68826	10.90652	10.2829	10.91961
AT4G04465	62.60958	0.840638	0.264087	3.183192	0.001457	0.009912	6.30836	6.240703	6.577971	5.672031	5.262981	5.387687
AT2G28470	551.7073	0.841345	0.128406	6.55224	5.67E-11	1.32E-09	9.454572	9.540483	9.414118	8.607986	8.687846	8.58922
AT2G41870	156.7352	0.84141	0.195327	4.307704	1.65E-05	0.000183	7.647563	7.726233	7.583389	6.9213	7.013727	6.522394
AT2G35830	475.1387	0.841817	0.176033	4.78216	1.73E-06	2.28E-05	9.40801	9.127067	9.262215	8.693418	8.229725	8.18052
AT1G14840	264.3249	0.842166	0.165506	5.088419	3.61E-07	5.32E-06	8.343795	8.475535	8.368259	7.535061	7.850087	7.361607
AT1G22140	127.6952	0.842811	0.203694	4.137626	3.51E-05	0.000363	7.36873	7.412853	7.462062	6.588138	5.90133	6.703006
AT1G56580	461.8123	0.843118	0.135317	6.230694	4.64E-10	9.83E-09	9.185971	9.189444	9.295481	8.445107	8.280186	8.334564
AT2G32100	117.037	0.843378	0.228979	3.683209	0.00023	0.001981	7.12272	7.176868	7.503648	6.657813	6.024848	6.259292
AT5G53580	288.7835	0.843798	0.16581	5.088942	3.60E-07	5.31E-06	8.549347	8.568519	8.601068	7.781272	7.182411	7.850924
AT5G15845	806.7863	0.844161	0.136075	6.203638	5.52E-10	1.16E-08	10.18473	9.926417	9.934345	9.092985	9.252813	9.167817
AT2G20570	731.0078	0.846578	0.12674	6.679665	2.39E-11	5.82E-10	9.916541	9.826614	9.905333	9.136346	8.990252	8.928662
AT5G58340	60.3848	0.848288	0.258055	3.287243	0.001012	0.007256	6.34561	6.38694	6.259813	5.331804	5.262981	5.584819
AT5G43745	84.88979	0.84856	0.23991	3.536989	0.000405	0.003243	6.643567	6.900687	6.845691	6.129868	5.90133	5.674125
AT2G32500	98.56958	0.850032	0.230989	3.679975	0.000233	0.001999	6.92772	6.906762	7.250369	6.313781	5.766238	6.117894
AT2G47240	262.6306	0.850069	0.171975	4.942969	7.69E-07	1.08E-05	8.54535	8.408614	8.335368	7.749708	7.182411	7.530557
AT3G55330	342.759	0.850203	0.170419	4.988901	6.07E-07	8.66E-06	8.709721	8.791737	8.925656	8.180929	7.594963	7.850924
AT5G40950	1449.75	0.850561	0.135463	6.278917	3.41E-10	7.33E-09	10.70803	10.8214	11.06673	10.05339	9.934731	10.01669
AT5G59430	384.9183	0.851136	0.167596	5.078504	3.80E-07	5.59E-06	8.897015	9.128367	8.805177	7.879329	8.328941	8.102229
AT5G28750	535.9923	0.851455	0.150327	5.664011	1.48E-08	2.64E-07	9.252448	9.425655	9.612997	8.517175	8.591227	8.604447
AT1G47580	95.85256	0.85177	0.226812	3.755411	0.000173	0.001535	7.03992	6.924835	7.065262	6.356317	5.450922	6.096513
AT1G56190	555.4239	0.852481	0.151388	5.631122	1.79E-08	3.15E-07	9.311296	9.484719	9.650506	8.709913	8.649971	8.502469
AT1G71500	867.1886	0.852553	0.15677	5.438229	5.38E-08	8.97E-07	10.00796	10.07138	10.30156	9.455317	9.265393	9.033636
AT2G40205	270.1473	0.852841	0.195675	4.358452	1.31E-05	0.000148	8.362088	8.278792	8.713636	7.378772	7.47011	7.805528
AT4G22200	322.6973	0.855492	0.154304	5.544179	2.95E-08	5.08E-07	8.689944	8.841741	8.617997	7.879329	7.672573	7.894935
AT5G07015	68.6767	0.855785	0.247845	3.452897	0.000555	0.004294	6.550921	6.404219	6.542941	5.737858	5.617177	5.456434

AT5G40390	84.13078	0.856154	0.227749	3.7592	0.00017	0.001515	6.772254	6.739852	6.881184	6.001749	5.766238	5.863234
AT5G59070	63.51254	0.85639	0.262711	3.259824	0.001115	0.007889	6.542925	6.171748	6.488747	5.603056	5.262981	5.489617
AT5G62220	145.0696	0.856518	0.218162	3.92607	8.63E-05	0.00083	7.359654	7.507833	7.699282	6.377123	7.182411	6.55411
AT1G51140	117.8587	0.856841	0.235009	3.645993	0.000266	0.002243	6.915386	7.535541	7.308884	6.417857	6.342338	6.352426
AT3G55500	150.8725	0.857379	0.258124	3.321581	0.000895	0.006555	7.175393	7.715891	7.850192	6.17809	7.128345	6.825021
AT5G67190	103.1699	0.858339	0.223027	3.848587	0.000119	0.001099	7.201025	7.115245	7.046626	6.4378	5.450922	6.259292
AT5G12470	218.9302	0.85975	0.179655	4.785565	1.71E-06	2.24E-05	8.233855	8.136123	8.190101	7.409395	6.679627	7.431579
AT4G34770	85.05156	0.861091	0.241607	3.564019	0.000365	0.00296	6.88408	6.691408	6.949654	6.20161	5.262981	5.888334
AT1G14460	155.2916	0.861372	0.205843	4.184602	2.86E-05	0.000302	7.510779	7.857483	7.667108	7.017205	6.434336	6.703006
AT4G07975	114.9289	0.862464	0.21744	3.966445	7.30E-05	0.00071	7.090168	7.429827	7.250369	6.476879	5.90133	6.422834
AT3G07315	378.3016	0.864363	0.203843	4.240327	2.23E-05	0.000241	8.794668	8.887001	9.177672	8.425264	7.594963	7.919495
AT2G36430	164.3092	0.864609	0.21963	3.936652	8.26E-05	0.000796	7.765639	7.715891	7.885577	7.191776	5.90133	6.925441
AT2G34760	88.4507	0.865999	0.263967	3.280712	0.001035	0.007409	6.665827	6.626632	7.143347	5.919035	6.434336	5.584819
AT1G55120	60.98571	0.868007	0.262683	3.304397	0.000952	0.006899	6.200668	6.487643	6.393639	5.603056	5.046837	5.42247
AT3G44620	204.2845	0.868007	0.188238	4.611233	4.00E-06	4.96E-05	7.87796	8.039905	8.245624	7.304685	7.128345	7.041871
AT1G32540	212.2348	0.868851	0.164708	5.275088	1.33E-07	2.10E-06	8.120225	8.133535	8.126453	7.326244	7.013727	7.240127
AT3G11170	946.7288	0.869188	0.150468	5.776576	7.62E-09	1.41E-07	10.28161	10.15797	10.33714	9.531537	9.485659	9.099602
AT3G24190	412.2315	0.869722	0.143007	6.081694	1.19E-09	2.43E-08	9.064162	9.060509	9.049381	8.255864	8.304769	8.013753
AT5G01195	98.25277	0.869964	0.23048	3.774576	0.00016	0.001438	7.045591	7.141242	6.845691	5.861135	6.13862	6.29727
AT2G24090	976.2437	0.870282	0.153901	5.654812	1.56E-08	2.76E-07	10.18858	10.23543	10.5075	9.592856	9.148022	9.442346
AT5G61270	83.48444	0.870628	0.229169	3.79907	0.000145	0.001312	6.758509	6.825719	6.85286	5.919035	5.450922	6.030403
AT1G71810	77.97508	0.870657	0.243836	3.570673	0.000356	0.002896	6.566781	6.712369	6.867092	5.974702	5.262981	5.811684
AT2G29890	62.78358	0.871311	0.254241	3.427109	0.00061	0.004682	6.327105	6.43817	6.460867	5.603056	5.046837	5.522054
AT5G28770	528.3734	0.871688	0.149602	5.826698	5.65E-09	1.06E-07	9.45244	9.283488	9.511329	8.629823	8.649971	8.343667
AT5G64850	119.263	0.872204	0.229573	3.799248	0.000145	0.001312	7.265613	7.083418	7.574743	6.551997	5.90133	6.45679
AT2G26080	1599.522	0.872763	0.106143	8.222539	1.99E-16	7.43E-15	10.96674	11.04271	11.03675	10.11662	10.21319	10.09559
AT3G62070	237.2576	0.879493	0.252075	3.489012	0.000485	0.003813	8.471447	8.248019	8.06296	7.580621	7.74622	6.600414
AT3G06170	302.8854	0.880952	0.156933	5.613565	1.98E-08	3.48E-07	8.649557	8.535574	8.709693	7.86467	7.63429	7.645434
AT5G38520	370.3032	0.881049	0.203101	4.337977	1.44E-05	0.000161	8.782835	8.933861	9.087865	8.400072	7.47011	7.901114
AT1G08477	161.526	0.881234	0.204074	4.318198	1.57E-05	0.000175	7.598258	7.882358	7.683285	6.533581	6.88921	7.007918
AT3G16800	471.0524	0.881262	0.136609	6.450997	1.11E-10	2.50E-09	9.29115	9.271768	9.240514	8.498306	8.255176	8.302244
AT2G08440	66.94147	0.881693	0.260012	3.39097	0.000696	0.005241	6.47731	6.240703	6.670136	5.492986	5.617177	5.553778
AT1G14030	231.686	0.883193	0.177228	4.983372	6.25E-07	8.88E-06	8.188446	8.196879	8.395516	7.544288	7.013727	7.306808

AT3G17510	1079.52	0.883401	0.157357	5.614009	1.98E-08	3.47E-07	10.65291	10.35578	10.37435	9.74867	9.240123	9.58926
AT4G37080	350.8919	0.883565	0.152024	5.811995	6.17E-09	1.16E-07	8.739763	8.909865	8.891253	8.076669	7.78168	7.894935
AT5G46690	126.4249	0.884649	0.238766	3.705089	0.000211	0.001835	7.502556	7.454919	7.28258	6.907064	5.766238	6.278406
AT2G26220	84.89069	0.885179	0.232012	3.81522	0.000136	0.001239	6.92772	6.851144	6.771973	6.001749	5.262981	6.052778
AT2G21370	122.3246	0.885456	0.235587	3.758514	0.000171	0.001518	7.079152	7.369524	7.59626	6.691428	5.90133	6.370353
AT1G08380	14769.75	0.887715	0.146899	6.043046	1.51E-09	3.07E-08	14.10708	14.07826	14.48099	13.45965	13.15918	13.34433
AT4G00430	1174.102	0.888225	0.124208	7.151123	8.61E-13	2.37E-11	10.46205	10.59935	10.68116	9.764549	9.569204	9.674698
AT5G64580	123.6266	0.889011	0.212016	4.193139	2.75E-05	0.000292	7.404472	7.378294	7.37511	6.724278	5.766238	6.422834
AT4G15630	95.8587	0.889384	0.261922	3.3956	0.000685	0.005171	7.280116	6.760126	6.982708	6.457472	5.450922	5.888334
AT2G33850	546.8417	0.890244	0.190243	4.679515	2.88E-06	3.65E-05	9.565293	9.564698	9.300733	8.835499	8.649971	8.144512
AT1G12280	89.21564	0.890376	0.269478	3.30407	0.000953	0.0069	6.987859	7.02876	6.794486	6.457472	4.792505	5.83769
AT5G38980	326.9629	0.89042	0.199666	4.459546	8.21E-06	9.63E-05	8.921892	8.515837	8.758225	7.429455	7.946977	8.047571
AT2G01755	75.65438	0.892367	0.242182	3.68469	0.000229	0.001971	6.628533	6.670137	6.67823	5.947137	5.617177	5.522054
AT5G20070	130.1046	0.892681	0.210827	4.23419	2.29E-05	0.000247	7.469191	7.404291	7.314087	6.588138	6.822676	6.159727
AT3G46780	3929.844	0.892949	0.130705	6.831798	8.39E-12	2.11E-10	12.32075	12.19288	12.44703	11.56052	11.42931	11.24397
AT3G16000	844.2273	0.892997	0.135922	6.569931	5.03E-11	1.19E-09	10.06561	10.20679	10.05658	9.372256	9.078439	9.102286
AT1G54790	116.3132	0.894733	0.229179	3.904081	9.46E-05	0.000901	7.051239	7.306589	7.466742	6.605875	6.024848	6.200381
AT1G77460	167.8382	0.894962	0.185701	4.819377	1.44E-06	1.93E-05	7.887464	7.809652	7.726857	6.863493	6.520817	7.041871
AT5G66520	93.96987	0.894997	0.234902	3.810089	0.000139	0.001262	6.751587	6.97195	7.137489	6.17809	6.024848	5.863234
AT5G61455	2508.971	0.895697	0.170394	5.256619	1.47E-07	2.30E-06	11.5961	11.69218	11.76389	10.69391	10.38442	11.09291
AT2G46450	135.3636	0.895884	0.210432	4.257346	2.07E-05	0.000224	7.695238	7.425602	7.344921	6.476879	6.342338	6.731026
AT4G32590	337.523	0.896015	0.167975	5.334214	9.60E-08	1.55E-06	8.640218	8.731405	8.996956	7.827358	7.74622	7.990759
AT3G18750	90.146	0.896291	0.234517	3.821851	0.000132	0.00121	6.915386	6.942684	6.888179	6.269954	5.766238	5.730732
AT2G43375	1039.027	0.896702	0.155829	5.754396	8.70E-09	1.60E-07	10.39089	10.50289	10.34605	9.319486	9.302486	9.772312
AT1G08650	68.55717	0.897692	0.263618	3.405272	0.000661	0.005004	6.643567	6.698429	6.312764	5.70532	4.792505	5.758224
AT2G21530	362.6554	0.89827	0.16677	5.386289	7.19E-08	1.18E-06	8.720396	8.902284	9.077193	8.101887	7.709866	8.008039
AT1G73390	149.6376	0.898309	0.19066	4.711572	2.46E-06	3.15E-05	7.628798	7.750081	7.521753	6.724278	6.520817	6.772062
AT1G04350	269.9918	0.900551	0.156846	5.741634	9.38E-09	1.72E-07	8.4882	8.378331	8.520076	7.589563	7.63429	7.456967
AT4G28750	12954.35	0.90129	0.172661	5.220006	1.79E-07	2.76E-06	13.91223	13.84987	14.34338	13.38473	12.9146	13.027
AT5G38140	62.37485	0.90218	0.273008	3.304598	0.000951	0.006898	6.200668	6.633975	6.238077	5.492986	5.617177	5.239508
AT4G30020	330.4227	0.902578	0.191509	4.712985	2.44E-06	3.14E-05	8.424347	8.947185	8.8982	7.977969	7.594963	7.838099
AT2G04230	123.808	0.903798	0.214812	4.207386	2.58E-05	0.000275	7.22621	7.450767	7.471407	6.674719	5.90133	6.405552
AT1G33110	245.1715	0.904711	0.195061	4.638105	3.52E-06	4.40E-05	8.22888	8.384874	8.368259	6.990448	7.554534	7.623179

AT3G19720	637.7218	0.904977	0.159875	5.660519	1.51E-08	2.68E-07	9.622373	9.771087	9.745045	9.054752	8.630654	8.585388
AT1G29040	156.5417	0.907187	0.20426	4.441333	8.94E-06	0.000104	7.716721	7.475501	7.902948	6.772191	6.520817	6.863502
AT1G61500	53.5554	0.90746	0.268039	3.385558	0.00071	0.005329	6.127699	6.191788	6.182258	5.150324	5.262981	5.239508
AT2G21860	110.91	0.907996	0.220943	4.109645	3.96E-05	0.000406	7.284918	7.13608	7.293159	6.247531	5.617177	6.538339
AT1G72645	4569.554	0.908389	0.129184	7.031757	2.04E-12	5.44E-11	12.46446	12.71994	12.43469	11.61956	11.75113	11.49949
AT2G18560	107.0855	0.908695	0.221001	4.111732	3.93E-05	0.000403	7.022775	7.301987	7.083661	6.313781	6.342338	6.007675
AT5G43150	163.3245	0.90936	0.187509	4.849684	1.24E-06	1.68E-05	7.636334	7.89769	7.74238	6.863493	6.679627	6.850789
AT4G33110	197.9897	0.909662	0.172621	5.269716	1.37E-07	2.15E-06	8.020099	8.050918	8.028552	7.249335	7.013727	6.98483
AT5G55740	85.69734	0.911475	0.243305	3.746222	0.00018	0.001587	6.709344	7.000635	6.779516	6.105138	5.90133	5.615206
AT4G17810	146.9063	0.911547	0.212778	4.284026	1.84E-05	0.000201	7.498428	7.487711	7.85377	6.878163	6.342338	6.600414
AT4G17270	271.5485	0.912072	0.155194	5.876999	4.18E-09	8.03E-08	8.517058	8.406472	8.542418	7.59845	7.42597	7.577611
AT4G00975	109.9696	0.912475	0.227837	4.004949	6.20E-05	0.000612	7.404472	7.023179	7.211858	6.224753	5.766238	6.473473
AT2G29180	180.836	0.913482	0.175809	5.19586	2.04E-07	3.12E-06	7.881135	7.894637	7.976993	7.056432	6.679627	7.019324
AT2G06520	12572.47	0.914032	0.149477	6.114848	9.66E-10	1.99E-08	13.89457	13.83442	14.2657	13.24263	12.9598	13.00977
AT5G09585	2652.579	0.915362	0.17914	5.109756	3.23E-07	4.80E-06	11.86219	11.76547	11.69026	11.00087	10.30134	11.03719
AT4G14910	207.631	0.916068	0.189037	4.84597	1.26E-06	1.71E-05	8.062666	8.259933	8.006222	7.003888	6.952811	7.397017
AT5G12860	397.9532	0.917221	0.211902	4.328505	1.50E-05	0.000167	8.586774	9.071367	9.33825	7.849861	8.328941	8.086048
AT1G30380	10431.66	0.919163	0.16061	5.722963	1.05E-08	1.90E-07	13.5937	13.56837	14.02608	12.95198	12.57892	12.84566
AT5G49030	271.2003	0.920206	0.183869	5.004688	5.60E-07	8.03E-06	8.287481	8.66486	8.524572	7.749708	7.234523	7.498313
AT2G08060	71.06068	0.921207	0.271898	3.388062	0.000704	0.005289	6.36388	6.611834	6.771973	5.243916	5.450922	5.937262
AT3G26710	396.9337	0.922111	0.168985	5.456754	4.85E-08	8.13E-07	8.998439	9.110055	9.009841	8.369248	7.915398	7.882497
AT3G18980	282.6519	0.922161	0.15813	5.831675	5.49E-09	1.03E-07	8.596464	8.623039	8.434265	7.676061	7.42597	7.630636
AT4G37280	175.9286	0.923447	0.179741	5.137652	2.78E-07	4.17E-06	7.94624	7.760182	7.913272	6.949358	6.752923	6.973146
AT2G31790	151.6884	0.924335	0.194696	4.747576	2.06E-06	2.67E-05	7.477605	7.780174	7.675219	6.724278	6.752923	6.645278
AT3G22150	169.6581	0.924804	0.216303	4.275509	1.91E-05	0.000208	7.713163	7.90984	7.882077	7.249335	6.434336	6.630478
AT3G14440	262.5379	0.924867	0.187371	4.936014	7.97E-07	1.12E-05	8.260917	8.406472	8.613784	7.650652	7.594963	7.220496
AT4G28780	183.8489	0.924892	0.191365	4.833122	1.34E-06	1.81E-05	7.842569	7.939773	8.084436	7.056432	6.434336	7.159945
AT1G09200	136.5165	0.925151	0.204682	4.51994	6.19E-06	7.44E-05	7.359654	7.503831	7.638356	6.707947	6.520817	6.38806
AT1G15000	125.3197	0.925427	0.237583	3.895169	9.81E-05	0.00093	7.206097	7.221417	7.561677	6.17809	7.013727	6.138962
AT4G39280	233.8475	0.926827	0.163381	5.672779	1.40E-08	2.52E-07	8.260917	8.367359	8.212567	7.39926	7.182411	7.334468
AT5G64170	458.3568	0.926959	0.158462	5.849718	4.92E-09	9.37E-08	9.288761	9.369578	9.061808	8.459811	8.177436	8.195681
AT5G04360	187.4031	0.927667	0.229949	4.034233	5.48E-05	0.000548	7.744877	8.028807	8.147011	7.41946	6.520817	6.758512
AT5G22620	193.365	0.928001	0.179128	5.180671	2.21E-07	3.36E-06	8.014328	8.091485	7.871527	7.131825	7.128345	6.913264

AT1G09440	64.19858	0.928046	0.263732	3.518899	0.000433	0.003453	6.597987	6.306513	6.422836	5.70532	5.262981	5.278004
AT5G17600	60.6882	0.928644	0.287061	3.235007	0.001216	0.008496	6.485677	6.278675	6.516099	5.603056	3.546624	5.674125
AT1G56050	120.3109	0.929709	0.229378	4.053176	5.05E-05	0.000508	7.051239	7.446604	7.521753	6.397634	5.90133	6.538339
AT1G10900	177.2923	0.929864	0.193489	4.805777	1.54E-06	2.05E-05	7.698841	8.083462	7.860899	6.963185	6.752923	6.973146
AT4G22755	76.83988	0.930508	0.242814	3.832185	0.000127	0.001168	6.751587	6.719289	6.710159	6.001749	5.046837	5.702706
AT3G62390	74.06096	0.930528	0.249657	3.727221	0.000194	0.001697	6.723563	6.47134	6.577971	5.492986	6.13862	5.489617
AT4G28755	4971.047	0.93062	0.166606	5.585754	2.33E-08	4.05E-07	12.56991	12.45745	12.9637	11.94586	11.6299	11.56274
AT1G29910	55094.95	0.930899	0.184395	5.048388	4.46E-07	6.47E-06	15.99285	15.96986	16.44325	15.50061	14.87857	15.11925
AT4G04020	528.5364	0.93126	0.191638	4.859475	1.18E-06	1.60E-05	9.120319	9.427769	9.753688	8.38987	8.328941	8.68544
AT5G25590	77.28113	0.931771	0.259339	3.592875	0.000327	0.002687	6.485677	6.994943	6.620596	5.890376	5.450922	5.674125
AT5G66190	3439.615	0.932165	0.137184	6.795005	1.08E-11	2.71E-10	11.98973	12.16939	12.26722	11.37709	11.16302	11.02804
AT2G26215	45.14769	0.935274	0.290459	3.219986	0.001282	0.008886	6.062076	5.977637	5.934498	5.050236	4.089834	5.15929
AT1G09333	4417.437	0.935725	0.130993	7.143305	9.11E-13	2.50E-11	12.41767	12.69097	12.39088	11.54968	11.68244	11.43654
AT3G25480	77.37455	0.936039	0.244163	3.83366	0.000126	0.001162	6.723563	6.726176	6.801913	5.861135	4.792505	5.937262
AT5G49730	1933.637	0.93772	0.130595	7.180362	6.95E-13	1.92E-11	11.3031	11.29571	11.36161	10.57288	10.20014	10.27165
AT3G25717	463.24	0.939801	0.187031	5.02484	5.04E-07	7.26E-06	9.588785	9.10742	9.052497	8.255864	8.12318	8.444419
AT1G44790	104.2329	0.940402	0.229482	4.097941	4.17E-05	0.000425	6.909179	7.216535	7.293159	6.335206	5.766238	6.138962
AT1G05630	71.47334	0.940603	0.255505	3.681357	0.000232	0.001991	6.779077	6.64128	6.353768	5.603056	5.450922	5.674125
AT4G11900	108.7142	0.940685	0.270813	3.473564	0.000514	0.004013	7.180556	7.216535	7.166548	5.414645	6.342338	6.600414
AT3G54460	372.6119	0.942809	0.147284	6.401302	1.54E-10	3.42E-09	8.87966	9.006375	8.982322	7.950468	7.816289	8.118231
AT3G19800	200.6884	0.943172	0.185137	5.094446	3.50E-07	5.17E-06	7.884303	8.053658	8.248344	7.131825	6.952811	7.128689
AT3G60370	149.2168	0.943317	0.195561	4.823646	1.41E-06	1.89E-05	7.535168	7.611896	7.734639	6.787815	6.752923	6.473473
AT1G08250	45.61221	0.944679	0.296724	3.183694	0.001454	0.009898	6.084284	5.698312	6.051279	4.997462	5.046837	4.837144
AT3G12780	4904.264	0.945907	0.158878	5.953677	2.62E-09	5.17E-08	12.34395	12.69427	12.90294	11.83602	11.65162	11.58546
AT2G21280	182.5943	0.946421	0.188239	5.027763	4.96E-07	7.17E-06	7.779316	7.906812	8.102593	6.907064	6.822676	7.06407
AT3G47650	515.9068	0.949705	0.165737	5.730212	1.00E-08	1.83E-07	9.29115	9.338456	9.639148	8.61676	8.150563	8.46542
AT4G34290	128.4514	0.950128	0.207486	4.579233	4.67E-06	5.72E-05	7.33208	7.306589	7.604778	6.356317	6.520817	6.473473
AT1G60600	170.3297	0.950378	0.219268	4.334323	1.46E-05	0.000163	7.594395	7.860616	8.053657	7.156105	6.520817	6.674431
AT1G26218	135.8102	0.950419	0.197146	4.820888	1.43E-06	1.92E-05	7.602111	7.429827	7.548491	6.605875	6.13862	6.630478
AT4G06215	68.56343	0.95107	0.244165	3.895199	9.81E-05	0.00093	6.542925	6.535476	6.542941	5.567291	5.450922	5.553778
AT1G64680	325.8335	0.951191	0.161557	5.887646	3.92E-09	7.57E-08	8.697167	8.763561	8.762038	7.842399	8.037767	7.530557
AT1G64770	282.1828	0.95134	0.185988	5.11505	3.14E-07	4.68E-06	8.306498	8.708983	8.611672	7.741709	7.594963	7.370542
AT4G21210	334.5684	0.952562	0.18357	5.1891	2.11E-07	3.23E-06	8.479848	8.906837	8.979049	7.796798	7.709866	7.907267

AT5G66460	62.55018	0.95342	0.296981	3.210376	0.001326	0.009142	5.896243	6.543296	6.710159	5.373819	5.262981	5.456434
AT1G29460	267.3034	0.953463	0.190574	5.003112	5.64E-07	8.08E-06	8.563249	8.259933	8.588239	7.725575	7.47011	7.240127
AT3G10940	86.73877	0.953721	0.28499	3.346506	0.000818	0.006059	6.34561	7.078044	7.125699	6.15418	5.617177	5.644966
AT1G03940	136.6685	0.953929	0.220302	4.330092	1.49E-05	0.000166	7.395619	7.753456	7.462062	6.476879	6.024848	6.798784
AT5G35170	762.9755	0.954706	0.140803	6.780462	1.20E-11	2.99E-10	9.804793	10.02884	10.09412	9.086682	9.049638	8.90118
AT1G64400	69.83151	0.954723	0.261445	3.651716	0.00026	0.002204	6.832534	6.421295	6.497922	5.637956	5.046837	5.702706
AT2G33330	94.91889	0.955443	0.225592	4.235262	2.28E-05	0.000246	7.005422	7.006304	7.101827	6.15418	5.262981	6.159727
AT3G12345	892.3799	0.956155	0.13393	7.139197	9.39E-13	2.57E-11	10.13905	10.13033	10.32559	9.225093	9.408366	9.104965
AT1G70250	50.08792	0.957992	0.281462	3.403631	0.000665	0.005032	6.062076	6.045285	6.088196	4.764642	5.450922	5.117444
AT5G65840	233.9399	0.958063	0.205401	4.664351	3.10E-06	3.91E-05	7.964314	8.276448	8.58609	7.368418	7.128345	7.325306
AT3G20362	83.96383	0.958534	0.250996	3.818923	0.000134	0.001222	6.799356	6.780119	6.956326	6.20161	5.450922	5.553778
AT5G46280	48.48316	0.958698	0.281663	3.403704	0.000665	0.005032	6.084284	6.161623	5.94795	5.101148	4.792505	5.074348
AT5G28500	331.4462	0.95935	0.161996	5.922055	3.18E-09	6.19E-08	8.580928	8.887001	8.861352	7.819779	7.850087	7.758657
AT1G29465	371.8944	0.960007	0.161371	5.949077	2.70E-09	5.29E-08	8.865304	8.873106	9.138609	8.024871	7.709866	8.069683
AT1G48355	431.7159	0.960298	0.171188	5.609626	2.03E-08	3.56E-07	8.98817	9.10742	9.394601	8.358826	8.037767	8.091462
AT5G25140	107.8856	0.96096	0.218054	4.406986	1.05E-05	0.000121	7.350521	7.109989	7.052865	6.105138	6.244072	6.220286
AT3G50820	6386.633	0.961411	0.153625	6.258155	3.90E-10	8.31E-09	12.77241	13.0048	13.32016	12.06245	12.16439	11.97119
AT2G05310	743.1764	0.9616	0.174224	5.519336	3.40E-08	5.80E-07	9.921175	9.837785	10.10166	9.286871	8.706416	8.779002
AT4G39363	467.9219	0.96177	0.190019	5.061448	4.16E-07	6.07E-06	9.055757	9.461176	9.258171	8.175	8.706416	8.008039
AT1G10960	420.9756	0.963288	0.181743	5.300266	1.16E-07	1.84E-06	9.156142	8.905321	9.260868	8.083015	8.508985	7.863636
AT2G41170	126.9734	0.963299	0.219939	4.379855	1.19E-05	0.000135	7.308691	7.479582	7.334716	6.417857	6.88921	6.030403
AT3G27170	95.13496	0.963401	0.22069	4.36541	1.27E-05	0.000144	7.095645	6.966144	7.015021	6.15418	5.617177	6.030403
AT4G02530	641.1612	0.964514	0.163116	5.913049	3.36E-09	6.52E-08	9.501743	9.730563	9.954479	8.890804	8.669033	8.66016
AT4G17260	49.10531	0.964866	0.294519	3.276076	0.001053	0.007514	6.016608	5.844724	6.323125	4.885743	5.046837	5.15929
AT5G64050	374.9499	0.965911	0.140942	6.85325	7.22E-12	1.83E-10	8.931111	9.030222	8.939191	7.971143	7.915398	8.030761
AT1G48600	462.0315	0.967055	0.181848	5.317941	1.05E-07	1.68E-06	8.976343	9.431987	9.32026	8.037996	8.571102	8.235348
AT4G23740	230.5067	0.968109	0.165561	5.847438	4.99E-09	9.49E-08	8.216365	8.338437	8.291194	7.293783	7.013727	7.397017
AT3G07610	201.3232	0.968407	0.204861	4.727132	2.28E-06	2.94E-05	7.868394	8.318069	7.999777	6.833696	7.128345	7.240127
AT1G49430	197.9139	0.969846	0.175778	5.517449	3.44E-08	5.85E-07	7.991008	8.014813	8.178736	7.082003	6.822676	7.149602
AT3G02370	115.891	0.970184	0.228401	4.247725	2.16E-05	0.000233	7.502556	7.120482	7.137489	6.028299	6.602406	6.278406
AT1G30520	168.7211	0.970209	0.194267	4.994199	5.91E-07	8.45E-06	7.852308	7.663037	7.846606	6.724278	7.234523	6.615524
AT4G13100	50.75807	0.972215	0.284312	3.419539	0.000627	0.00479	6.210797	6.045285	6.204845	5.414645	4.483533	4.984091
AT5G12080	67.66683	0.972821	0.25209	3.859026	0.000114	0.001058	6.534884	6.670137	6.403437	5.567291	5.262981	5.553778

AT4G16770	163.8495	0.973845	0.197519	4.930385	8.21E-07	1.15E-05	7.632571	7.753456	7.899491	6.514927	7.013727	6.850789
AT3G21110	92.87358	0.974619	0.262016	3.719695	0.000199	0.001742	6.97603	7.104714	6.982708	5.737858	5.046837	6.439912
AT5G63310	500.1096	0.975843	0.173347	5.62942	1.81E-08	3.18E-07	9.231457	9.318087	9.614052	8.599158	8.037767	8.379515
AT1G27120	70.33916	0.976401	0.243889	4.003464	6.24E-05	0.000616	6.542925	6.648549	6.603697	5.637956	5.262981	5.615206
AT2G22240	147.9855	0.977009	0.210858	4.633496	3.60E-06	4.49E-05	7.845823	7.659443	7.37511	6.707947	6.602406	6.55411
AT1G06253	246.3266	0.977085	0.170291	5.737755	9.59E-09	1.75E-07	8.231369	8.338437	8.526815	7.378772	7.284819	7.397017
AT1G11410	130.1686	0.977833	0.2011	4.862423	1.16E-06	1.58E-05	7.36873	7.527679	7.535184	6.533581	6.024848	6.55411
AT4G18810	247.6109	0.978531	0.193097	5.067561	4.03E-07	5.90E-06	8.159976	8.583766	8.388133	7.59845	7.128345	7.259495
AT4G36360	360.7853	0.979603	0.144764	6.766877	1.32E-11	3.28E-10	8.906394	8.985001	8.88077	7.977969	7.709866	7.973271
AT4G39970	312.9843	0.979904	0.176235	5.560203	2.69E-08	4.66E-07	8.642091	8.527712	8.949259	7.796798	7.594963	7.68178
AT3G59068	879.5582	0.980337	0.211402	4.637308	3.53E-06	4.41E-05	10.26287	9.893146	10.43711	9.377429	8.550692	9.403699
AT3G53190	64.86307	0.980446	0.256934	3.815946	0.000136	0.001235	6.399741	6.38694	6.637299	5.530617	5.262981	5.42247
AT3G25120	85.27257	0.981752	0.250485	3.919398	8.88E-05	0.00085	6.909179	6.543296	7.034067	5.637956	6.024848	5.863234
AT5G02830	104.2062	0.981857	0.23369	4.201536	2.65E-05	0.000282	7.386711	6.918835	7.071421	6.20161	6.13862	6.007675
AT3G53900	109.7796	0.985515	0.251565	3.917531	8.95E-05	0.000856	6.981957	7.146386	7.570401	6.377123	5.450922	6.315891
AT5G56380	96.19553	0.985518	0.252556	3.902173	9.53E-05	0.000907	6.85208	7.067237	7.18938	6.377123	5.766238	5.644966
AT2G24545	69.06229	0.985691	0.254544	3.872388	0.000108	0.001009	6.694984	6.611834	6.36384	5.454347	5.450922	5.615206
AT1G65490	177.4789	0.987666	0.246912	4.000065	6.33E-05	0.000624	8.289872	7.539457	7.806553	6.551997	6.822676	7.139183
AT2G33620	150.0679	0.987966	0.202057	4.889546	1.01E-06	1.39E-05	7.422016	7.770213	7.757739	6.623396	6.679627	6.630478
AT1G20340	16192	0.988217	0.127301	7.762844	8.30E-15	2.71E-13	14.26588	14.31625	14.59407	13.49414	13.37334	13.3131
AT1G78230	134.3119	0.988304	0.214143	4.615149	3.93E-06	4.88E-05	7.221208	7.593183	7.621665	6.397634	6.679627	6.422834
AT3G02725	73.96516	0.988508	0.30226	3.27039	0.001074	0.007649	6.230845	6.977733	6.702243	5.050236	5.617177	5.961117
AT5G18070	76.02573	0.989161	0.236817	4.176894	2.96E-05	0.000311	6.665827	6.760126	6.661997	5.637956	5.617177	5.702706
AT1G26560	95.11559	0.990769	0.281015	3.525684	0.000422	0.003374	6.417342	7.301987	7.17229	5.737858	6.13862	6.030403
AT3G17040	885.6351	0.990903	0.153895	6.43883	1.20E-10	2.70E-09	10.17636	10.24625	10.1977	9.374845	9.314642	8.885683
AT5G18060	482.3655	0.991907	0.194779	5.092476	3.53E-07	5.22E-06	9.437422	9.150295	9.441718	8.689264	8.037767	8.064187
AT1G04530	163.9995	0.992112	0.189746	5.228618	1.71E-07	2.65E-06	7.906284	7.701985	7.734639	6.878163	6.822676	6.615524
AT4G22890	1706.26	0.993188	0.13361	7.433461	1.06E-13	3.17E-12	11.01487	11.1104	11.32051	10.22245	10.20014	10.01527
AT1G66840	60.94297	0.993296	0.302985	3.278367	0.001044	0.007461	6.250617	6.746642	6.193595	5.769679	4.792505	5.029925
AT3G47070	686.9987	0.993869	0.158729	6.261437	3.81E-10	8.15E-09	9.723998	9.730563	10.07279	8.992065	8.687846	8.762127
AT4G14540	131.0852	0.994036	0.221912	4.479403	7.49E-06	8.85E-05	7.386711	7.663037	7.360095	6.707947	6.342338	6.180197
AT1G15290	1014.611	0.994618	0.165706	6.002318	1.95E-09	3.90E-08	10.46998	10.60356	10.10917	9.52456	9.419665	9.213222
AT5G09820	64.91596	0.99495	0.264282	3.764725	0.000167	0.001486	6.673172	6.269276	6.525102	5.530617	5.046837	5.489617

AT3G61198	1170.508	0.995534	0.237861	4.18536	2.85E-05	0.000301	10.82144	10.44301	10.61049	9.364463	8.928315	10.12625
AT5G44600	43.28774	0.996744	0.306861	3.248191	0.001161	0.008179	5.870916	5.869819	6.147699	5.150324	3.546624	4.936752
AT3G02830	184.837	0.996758	0.195785	5.091088	3.56E-07	5.25E-06	8.112141	7.799893	8.03487	7.11953	6.434336	6.99642
AT5G59920	150.6543	0.998422	0.188813	5.287898	1.24E-07	1.96E-06	7.594395	7.659443	7.74238	6.533581	6.679627	6.717084
AT1G26220	110.3632	0.99957	0.217523	4.595245	4.32E-06	5.32E-05	7.382237	7.151511	7.23399	6.356317	5.617177	6.278406
AT1G06233	53.11242	0.999642	0.297168	3.363896	0.000769	0.005721	6.095261	6.421295	6.124191	4.764642	4.792505	5.522054
AT1G16720	1222.154	1.000544	0.141794	7.056304	1.71E-12	4.60E-11	10.53087	10.64458	10.83298	9.74867	9.75015	9.47792
AT2G29300	122.0686	1.000683	0.223116	4.485037	7.29E-06	8.65E-05	7.280116	7.43824	7.424062	6.657813	6.13862	6.052778
AT2G33250	128.1964	1.001453	0.215176	4.654109	3.25E-06	4.09E-05	7.22621	7.417115	7.579072	6.356317	6.752923	6.200381
AT4G33666	182.2746	1.001705	0.183075	5.471566	4.46E-08	7.50E-07	7.985118	7.857483	7.976993	6.724278	6.952811	7.053013
AT5G07020	625.8707	1.002266	0.179821	5.573696	2.49E-08	4.33E-07	9.425511	9.635911	10.02202	8.754327	8.742856	8.562179
AT4G23940	171.3572	1.002501	0.209804	4.778265	1.77E-06	2.32E-05	7.539193	8.042666	7.930315	6.892686	6.88921	6.688789
AT2G28950	731.042	1.002969	0.152343	6.583641	4.59E-11	1.09E-09	9.916541	9.833805	10.0873	9.096125	8.591227	8.943706
AT5G49170	62.17983	1.003163	0.259962	3.858879	0.000114	0.001058	6.542925	6.395605	6.403437	5.567291	4.792505	5.387687
AT3G01810	167.7599	1.004586	0.192234	5.22584	1.73E-07	2.68E-06	7.737889	7.906812	7.913272	6.963185	6.244072	6.876105
AT5G62790	683.7144	1.004727	0.180001	5.581781	2.38E-08	4.14E-07	9.466246	9.998586	10.00924	8.93357	8.813085	8.67466
AT5G10572	88.53208	1.005245	0.234927	4.278974	1.88E-05	0.000206	6.832534	6.812837	6.976157	5.603056	6.342338	5.785202
AT1G68830	686.6912	1.006302	0.134331	7.491205	6.82E-14	2.07E-12	9.826364	9.855168	9.894083	8.981913	8.571102	8.828473
AT3G14415	2985.222	1.007178	0.126937	7.934455	2.11E-15	7.29E-14	11.85535	11.90913	12.12808	11.0167	10.79341	10.99665
AT5G65890	109.0245	1.007209	0.227773	4.421978	9.78E-06	0.000113	6.93995	7.391351	7.277261	6.17809	6.13862	6.159727
AT2G42130	201.4592	1.008742	0.194411	5.188696	2.12E-07	3.23E-06	7.921783	8.099463	8.264562	7.282799	6.822676	6.925441
AT2G07690	69.63942	1.009324	0.26139	3.861371	0.000113	0.00105	6.716471	6.670137	6.460867	5.454347	4.792505	5.83769
AT1G05253	216.6814	1.009737	0.198798	5.079221	3.79E-07	5.57E-06	8.028714	8.091485	8.388133	7.226586	7.42597	6.850789
AT5G10470	291.6194	1.009795	0.17009	5.936822	2.91E-09	5.69E-08	8.537323	8.806434	8.544633	7.642081	7.284819	7.696066
AT2G24150	188.5538	1.010139	0.198101	5.099098	3.41E-07	5.06E-06	8.042958	7.912861	8.066048	6.707947	6.752923	7.249844
AT3G62410	789.4651	1.011415	0.155664	6.497413	8.17E-11	1.87E-09	9.897854	9.983576	10.24546	9.139393	9.092627	8.821974
AT3G59070	864.8958	1.012862	0.213058	4.753928	2.00E-06	2.60E-05	10.24696	9.880854	10.42694	9.33286	8.48767	9.359508
AT1G27210	557.628	1.012901	0.163326	6.201704	5.59E-10	1.17E-08	9.450304	9.597931	9.632915	8.79355	8.255176	8.383934
AT3G18110	153.4785	1.014734	0.201167	5.044237	4.55E-07	6.60E-06	7.527084	7.786777	7.828538	6.833696	6.342338	6.630478
AT4G15920	53.58407	1.014825	0.305347	3.323515	0.000889	0.006517	6.47731	5.918735	6.026132	4.942684	5.766238	4.784636
AT1G03930	313.3237	1.015041	0.161575	6.282158	3.34E-10	7.20E-09	8.617555	8.741637	8.839865	7.642081	7.47011	7.844526
AT1G07993	84.38924	1.015906	0.252189	4.028354	5.62E-05	0.00056	6.574646	6.869922	7.101827	5.919035	5.617177	5.730732
AT5G66580	699.6782	1.016452	0.163107	6.231826	4.61E-10	9.77E-09	10.09461	9.710735	9.816455	9.028689	8.778398	8.678262

AT1G15002	2209.528	1.017014	0.223463	4.551146	5.34E-06	6.48E-05	11.68393	11.39061	11.5679	10.69599	9.695422	10.78491
AT2G42380	220.3187	1.01754	0.173908	5.851013	4.89E-09	9.31E-08	8.268211	8.143859	8.259177	7.35799	7.072174	7.041871
AT3G56360	490.5509	1.017707	0.240539	4.230937	2.33E-05	0.00025	9.640284	9.644116	8.685805	8.454926	8.008134	8.370636
AT4G07080	226.7999	1.018422	0.237158	4.294272	1.75E-05	0.000193	7.921783	8.295095	8.50195	7.439382	7.42597	6.674431
AT1G72030	218.949	1.019827	0.188483	5.410705	6.28E-08	1.04E-06	8.180737	8.078089	8.402861	7.378772	6.88921	7.085933
AT4G12320	66.42801	1.01991	0.263269	3.87402	0.000107	0.001003	6.426063	6.463119	6.749102	5.492986	4.792505	5.644966
AT4G31390	198.5778	1.021206	0.179024	5.704311	1.17E-08	2.11E-07	8.014328	8.088816	8.031715	6.803271	7.380437	6.98483
AT1G06283	241.3503	1.021419	0.172446	5.923132	3.16E-09	6.15E-08	8.203742	8.32489	8.513305	7.293783	7.284819	7.325306
AT4G39330	631.449	1.022057	0.141871	7.204151	5.84E-13	1.63E-11	9.779496	9.672026	9.786817	8.808946	8.352714	8.785697
AT1G32550	230.2467	1.022106	0.204603	4.995569	5.87E-07	8.40E-06	7.985118	8.245625	8.58609	7.315505	7.128345	7.200593
AT2G38010	39.7517	1.022118	0.320053	3.193593	0.001405	0.009613	5.650502	5.581648	5.987571	4.324977	5.262981	4.614569
AT1G56600	280.1241	1.024488	0.18549	5.523158	3.33E-08	5.68E-07	8.391329	8.679127	8.590385	7.676061	7.672573	7.200593
AT3G44890	833.8747	1.025397	0.150827	6.798489	1.06E-11	2.65E-10	10.0152	10.10413	10.29828	9.278601	8.830121	9.072481
AT1G05385	99.79421	1.02656	0.225727	4.547801	5.42E-06	6.58E-05	7.051239	6.97195	7.287879	6.05437	5.766238	6.117894
AT4G03905	622.7835	1.027306	0.149975	6.849837	7.39E-12	1.87E-10	9.529322	9.721976	9.865128	8.531166	8.760736	8.724293
AT5G16715	268.001	1.02803	0.176102	5.837702	5.29E-09	9.99E-08	8.327596	8.677351	8.467337	7.516426	7.554534	7.306808
AT1G21500	1044.748	1.028091	0.172467	5.961095	2.51E-09	4.96E-08	10.27981	10.32037	10.74805	9.606136	9.350505	9.230501
AT3G23810	453.0798	1.028691	0.164364	6.258599	3.88E-10	8.30E-09	9.208893	9.40327	9.211528	8.38987	7.74622	8.288168
AT5G27240	109.479	1.029572	0.223731	4.601825	4.19E-06	5.17E-05	7.221208	7.425602	7.101827	6.335206	5.617177	6.220286
AT3G44020	52.63554	1.032126	0.29157	3.539889	0.0004	0.003216	6.169846	5.989134	6.27056	5.150324	5.617177	4.614569
AT4G07075	236.2931	1.032196	0.232628	4.437118	9.12E-06	0.000106	8.017217	8.347398	8.546845	7.468757	7.51294	6.717084
AT3G05345	56.74443	1.033146	0.269498	3.83359	0.000126	0.001162	6.148927	6.333823	6.343626	5.050236	5.450922	5.199956
AT3G17185	59.5285	1.033491	0.294051	3.514664	0.00044	0.003502	6.095261	6.221336	6.718032	5.454347	5.262981	5.029925
AT4G24350	60.5063	1.033899	0.275478	3.753103	0.000175	0.001548	6.468894	6.57416	6.135993	5.492986	5.046837	5.199956
AT4G08815	128.9462	1.035532	0.203108	5.098441	3.42E-07	5.07E-06	7.460728	7.429827	7.424062	6.129868	6.679627	6.439912
AT1G56500	485.2205	1.036233	0.155534	6.662433	2.69E-11	6.51E-10	9.243841	9.384892	9.474622	8.503046	8.037767	8.25478
AT4G26860	197.9041	1.036561	0.230958	4.488103	7.19E-06	8.54E-05	8.165194	8.028807	8.158628	7.293783	5.766238	7.210579
AT3G19080	34.60696	1.036918	0.320857	3.231715	0.00123	0.008585	5.708842	5.519576	5.601531	4.324977	4.483533	4.614569
AT2G46340	493.4404	1.037461	0.13958	7.432734	1.06E-13	3.18E-12	9.366705	9.491808	9.305966	8.410202	8.177436	8.339123
AT4G32980	47.82389	1.038928	0.310053	3.350811	0.000806	0.005977	5.818889	6.360626	6.00054	4.632436	4.792505	5.239508
AT1G29840	40.66473	1.038967	0.315124	3.297009	0.000977	0.00704	5.764914	6.034228	5.731672	5.050236	4.483533	4.421742
AT1G29980	39.82792	1.039605	0.325621	3.192686	0.00141	0.009638	5.818889	5.726062	6.03876	4.486882	3.546624	5.15929
AT4G07070	243.1124	1.039921	0.225192	4.617935	3.88E-06	4.82E-05	8.079347	8.400026	8.564419	7.488014	7.554534	6.785485

AT5G09065	462.7927	1.040815	0.146275	7.11549	1.12E-12	3.04E-11	9.139005	9.405417	9.303352	8.215999	8.328941	8.18052
AT1G02350	90.89734	1.042443	0.237327	4.392433	1.12E-05	0.000128	6.952077	6.894586	7.065262	6.15418	5.766238	5.615206
AT2G39800	1229.801	1.043281	0.193251	5.398595	6.72E-08	1.11E-06	10.28761	11.07543	10.65731	9.608337	9.496371	9.746806
AT2G08765	63.53722	1.043783	0.266013	3.923803	8.72E-05	0.000838	6.399741	6.519708	6.393639	5.530617	5.617177	4.984091
AT3G45210	80.1339	1.045086	0.262443	3.982151	6.83E-05	0.000668	7.051239	6.691408	6.764389	5.919035	4.483533	5.888334
AT4G24670	49.80355	1.048045	0.312636	3.352283	0.000801	0.005947	5.933418	6.503765	5.94795	5.331804	4.483533	4.837144
AT4G06665	72.3557	1.05054	0.276442	3.800214	0.000145	0.001307	6.933848	6.446534	6.637299	5.919035	4.792505	5.42247
AT3G57020	60.31406	1.051552	0.264192	3.980252	6.88E-05	0.000673	6.399741	6.429757	6.248986	5.101148	5.617177	5.239508
AT2G24820	245.4184	1.05544	0.187089	5.641371	1.69E-08	2.98E-07	8.218877	8.450802	8.517822	7.156105	7.072174	7.546413
AT1G50240	139.1019	1.05544	0.212171	4.974478	6.54E-07	9.27E-06	7.422016	7.750081	7.630034	6.551997	5.90133	6.674431
AT1G57770	113.8164	1.055483	0.260479	4.05209	5.08E-05	0.00051	6.812719	7.412853	7.476057	6.028299	6.679627	5.913005
AT4G06534	82.26808	1.056135	0.303223	3.483035	0.000496	0.003885	6.36388	6.760126	7.178009	5.769679	6.244072	5.074348
AT3G19170	1163.107	1.05861	0.168695	6.275275	3.49E-10	7.49E-09	10.2888	10.76525	10.7874	9.706135	9.408366	9.47378
AT3G15540	238.1038	1.058683	0.250995	4.217943	2.47E-05	0.000263	7.812953	8.675573	8.504228	7.409395	6.602406	7.414402
AT2G26692	53.28377	1.058946	0.279961	3.782484	0.000155	0.001395	6.240765	6.360626	6.051279	4.942684	5.046837	5.239508
AT3G03830	66.99342	1.059677	0.265384	3.992994	6.52E-05	0.000641	6.673172	6.45485	6.551779	5.769679	5.046837	5.199956
AT2G35130	86.40431	1.059831	0.250881	4.224445	2.40E-05	0.000257	6.915386	6.838488	7.002182	6.17809	5.450922	5.489617
AT5G16400	596.9802	1.061039	0.183447	5.783895	7.30E-09	1.35E-07	9.336956	9.614728	9.986609	8.621127	8.550692	8.546497
AT1G62750	1545.259	1.062585	0.117438	9.048026	1.46E-19	6.63E-18	10.96898	11.00922	11.12711	10.05984	9.886687	9.908899
AT1G26230	69.56321	1.062802	0.263676	4.030716	5.56E-05	0.000555	6.317763	6.73303	6.741397	5.454347	5.262981	5.584819
AT3G04350	282.7209	1.06322	0.164176	6.47609	9.41E-11	2.13E-09	8.700765	8.515837	8.564419	7.659171	7.42597	7.388246
AT1G77580	110.9942	1.064556	0.247307	4.30459	1.67E-05	0.000185	7.051239	7.527679	7.261186	6.496028	5.450922	6.074811
AT1G22160	45.47411	1.065167	0.298219	3.571756	0.000355	0.002886	6.005013	6.099331	6.013393	5.243916	4.089834	4.730144
AT2G21140	157.8437	1.06549	0.216673	4.917491	8.77E-07	1.22E-05	7.779316	7.648609	7.776709	6.803271	7.013727	6.180197
AT4G10770	169.7352	1.066605	0.20289	5.257047	1.46E-07	2.30E-06	7.605954	7.948634	8.031715	6.863493	6.602406	6.744835
AT2G18230	53.23991	1.067096	0.288107	3.703815	0.000212	0.001843	6.260403	6.378222	6.124191	4.885743	4.483533	5.456434
AT2G34490	45.78519	1.068268	0.302982	3.525852	0.000422	0.003373	6.180193	5.844724	6.00054	5.150324	4.792505	4.553113
AT4G13570	131.3293	1.068393	0.227388	4.698539	2.62E-06	3.34E-05	7.640086	7.663037	7.149183	6.476879	6.342338	6.315891
AT5G52780	284.6618	1.070278	0.180737	5.921736	3.19E-09	6.19E-08	8.675388	8.436176	8.721491	7.749708	7.284819	7.370542
AT4G28220	532.4773	1.071141	0.166491	6.433609	1.25E-10	2.78E-09	9.364438	9.654081	9.436956	8.332435	8.742856	8.190645
AT3G55800	2512.656	1.072501	0.115015	9.32484	1.11E-20	5.50E-19	11.64104	11.70727	11.86064	10.72062	10.62304	10.60829
AT2G04080	70.31159	1.07322	0.266077	4.033491	5.50E-05	0.000549	6.550921	6.684352	6.586597	4.997462	5.617177	5.758224
AT3G11670	555.2866	1.073653	0.136288	7.877821	3.33E-15	1.13E-13	9.53538	9.634996	9.55484	8.594723	8.229725	8.4984

AT1G12800	672.2843	1.074719	0.136303	7.884753	3.15E-15	1.07E-13	9.719571	9.864561	9.912212	8.79355	8.8636	8.612001
AT5G62740	179.4056	1.075334	0.178672	6.018491	1.76E-09	3.55E-08	7.858764	7.936808	8.022207	6.772191	6.88921	6.876105
AT1G16410	201.1277	1.07718	0.233776	4.607749	4.07E-06	5.04E-05	7.7622	8.537533	7.94716	6.976881	6.88921	7.053013
AT1G09390	112.6677	1.077245	0.212718	5.064186	4.10E-07	6.00E-06	7.22621	7.221417	7.287879	6.028299	6.602406	6.007675
AT1G70000	38.13174	1.077556	0.321863	3.347871	0.000814	0.006031	5.574094	5.977637	5.731672	4.324977	4.483533	4.837144
AT5G00660	92.48442	1.077714	0.283061	3.807361	0.00014	0.001275	6.825959	7.412853	6.771973	5.567291	5.450922	6.259292
AT5G45930	335.0801	1.079507	0.224228	4.814318	1.48E-06	1.98E-05	8.571133	8.91891	9.071058	8.126672	7.072174	7.645434
AT5G41400	74.45245	1.08132	0.252015	4.290703	1.78E-05	0.000196	6.765398	6.619252	6.823968	5.567291	4.792505	5.83769
AT1G02205	323.7549	1.082344	0.236866	4.569433	4.89E-06	5.97E-05	8.275467	9.019049	9.025787	7.991526	7.33342	7.514525
AT2G36870	94.29874	1.084982	0.22527	4.816363	1.46E-06	1.96E-05	7.149297	7.023179	6.942951	5.831289	5.766238	6.030403
AT1G50732	129.2684	1.086147	0.203339	5.341556	9.22E-08	1.49E-06	7.481794	7.365118	7.604778	6.457472	6.244072	6.315891
AT1G48350	1167.189	1.086607	0.168141	6.462473	1.03E-10	2.32E-09	10.52936	10.53364	10.85991	9.772423	9.188216	9.516667
AT1G32900	367.7138	1.087256	0.164565	6.606856	3.93E-11	9.36E-10	8.901712	9.06866	8.927355	7.984764	8.037767	7.600575
AT3G01510	89.94815	1.089702	0.233816	4.660515	3.15E-06	3.98E-05	6.839079	7.099419	6.902069	5.890376	6.024848	5.644966
AT1G29810	56.09866	1.090446	0.28164	3.871778	0.000108	0.001011	6.190467	6.288014	6.302328	5.243916	5.617177	4.673514
AT1G66830	86.53032	1.090502	0.262317	4.157195	3.22E-05	0.000335	6.92772	6.64128	7.228488	5.890376	5.046837	5.937262
AT4G17090	262.6943	1.09072	0.215773	5.054934	4.31E-07	6.26E-06	8.819703	8.373952	8.325095	7.624787	6.752923	7.431579
AT1G31190	110.832	1.090934	0.237015	4.602808	4.17E-06	5.15E-05	7.073613	7.288092	7.503648	6.397634	5.450922	6.138962
AT4G18480	1592.005	1.091782	0.149391	7.308206	2.71E-13	7.78E-12	10.89553	11.16844	11.19743	10.18754	9.794222	9.890382
AT4G33780	129.4503	1.092022	0.196582	5.555059	2.78E-08	4.79E-07	7.48597	7.471408	7.512729	6.457472	6.244072	6.315891
AT5G54190	583.683	1.093066	0.201158	5.433861	5.51E-08	9.17E-07	9.928098	9.376161	9.603466	8.835499	8.376102	8.24023
AT1G17360	377.1562	1.093423	0.156335	6.994106	2.67E-12	7.09E-11	8.901712	9.161771	8.955933	7.957392	7.977879	7.785626
AT5G02120	455.5313	1.093791	0.16755	6.52814	6.66E-11	1.54E-09	9.196204	9.137437	9.522612	8.310969	8.008134	8.123525
AT5G48790	485.8617	1.095207	0.150524	7.275951	3.44E-13	9.80E-12	9.393632	9.239641	9.486194	8.364046	8.328941	8.107583
AT1G70610	147.6846	1.095314	0.186457	5.87436	4.24E-09	8.14E-08	7.651287	7.673764	7.695299	6.551997	6.520817	6.55411
AT2G25840	128.2083	1.096669	0.20446	5.363735	8.15E-08	1.33E-06	7.350521	7.608172	7.443187	6.377123	6.342338	6.29727
AT2G35260	1281.001	1.098341	0.18283	6.007445	1.88E-09	3.79E-08	10.65616	10.9309	10.7643	10.01906	9.277863	9.508596
AT5G38150	100.4157	1.102002	0.299565	3.678673	0.000234	0.002007	6.958103	7.687943	6.661997	6.105138	5.262981	6.117894
AT2G35660	83.42071	1.10264	0.233998	4.712188	2.45E-06	3.15E-05	6.952077	6.746642	6.902069	5.769679	5.617177	5.702706
AT1G66820	85.09591	1.102967	0.262183	4.206855	2.59E-05	0.000276	6.902945	6.626632	7.206271	5.861135	5.046837	5.888334
AT3G19480	85.00686	1.103265	0.241455	4.569232	4.90E-06	5.97E-05	6.765398	6.97195	6.976157	6.001749	5.450922	5.584819
AT5G57030	261.8854	1.104872	0.169549	6.516537	7.19E-11	1.66E-09	8.397993	8.47349	8.588239	7.478418	7.47011	7.170215
AT1G01600	30.43261	1.10634	0.34659	3.19207	0.001413	0.009655	5.47672	5.712254	5.278519	4.142579	3.546624	4.553113

AT2G23600	286.0975	1.10665	0.216544	5.11052	3.21E-07	4.78E-06	9.004274	8.25041	8.517822	7.468757	7.594963	7.388246
AT5G05580	49.50841	1.108075	0.291951	3.795419	0.000147	0.001329	6.169846	6.141156	6.112292	5.288529	4.792505	4.614569
AT5G66470	163.721	1.10834	0.207242	5.34806	8.89E-08	1.44E-06	7.758752	7.806407	7.970416	7.003888	6.244072	6.538339
AT1G06207	31.62103	1.110787	0.347178	3.199476	0.001377	0.009454	5.723066	5.201541	5.684234	4.561494	3.546624	4.277199
AT4G29060	1676.844	1.111257	0.157196	7.069229	1.56E-12	4.21E-11	10.89356	11.39682	11.18577	10.17423	9.950397	9.957145
AT5G67280	67.98412	1.111469	0.268735	4.135938	3.54E-05	0.000366	6.47731	6.581774	6.771973	5.243916	4.792505	5.758224
AT1G76080	861.9182	1.114927	0.134182	8.309047	9.65E-17	3.69E-15	10.17636	10.07812	10.36749	9.070806	9.049638	9.104965
AT5G57170	81.45913	1.116943	0.255268	4.375565	1.21E-05	0.000138	6.628533	6.799839	7.089742	5.530617	5.450922	5.863234
AT5G66590	545.0356	1.118566	0.147462	7.585434	3.31E-14	1.04E-12	9.531344	9.389237	9.715854	8.41524	8.444073	8.388339
AT4G19200	718.602	1.11909	0.160439	6.975178	3.05E-12	8.04E-11	10.20451	9.855168	9.758468	8.722162	8.830121	8.870017
AT3G26310	63.92096	1.120374	0.256896	4.361187	1.29E-05	0.000146	6.390859	6.558811	6.534049	5.373819	5.262981	5.278004
AT1G14160	39.41185	1.121173	0.332254	3.374441	0.00074	0.005527	5.679967	5.596757	6.204845	4.885743	4.089834	4.488922
AT2G43560	503.8695	1.122087	0.163027	6.882815	5.87E-12	1.50E-10	9.245074	9.441434	9.632915	8.450025	8.150563	8.225533
AT3G28130	56.08178	1.123635	0.293872	3.823555	0.000132	0.001203	6.106155	6.551075	6.393639	5.492986	4.089834	5.074348
AT1G52510	435.217	1.123782	0.161226	6.970225	3.16E-12	8.32E-11	9.108178	9.134851	9.414118	8.144986	8.203818	7.92557
AT5G48300	1007.846	1.124943	0.154765	7.268735	3.63E-13	1.03E-11	10.14302	10.55941	10.57571	9.259118	9.373929	9.254831
AT4G00490	118.9293	1.125457	0.224899	5.004277	5.61E-07	8.04E-06	7.559151	7.38266	7.154994	6.001749	6.244072	6.352426
AT5G05060	161.3521	1.127177	0.19905	5.662789	1.49E-08	2.65E-07	7.758752	7.879272	7.810241	6.907064	6.520817	6.422834
AT1G29660	1275.694	1.128883	0.152478	7.403604	1.33E-13	3.93E-12	10.80357	10.71074	10.85501	9.660189	9.252813	9.854214
AT5G35630	7608.517	1.129823	0.143919	7.850427	4.15E-15	1.39E-13	13.05101	13.45019	13.52891	12.24083	12.14919	12.22162
AT3G06900	6060.294	1.13118	0.159196	7.10558	1.20E-12	3.27E-11	13.07391	12.75456	13.23047	11.93274	11.62259	12.02275
AT3G06895	6060.39	1.13121	0.159204	7.105409	1.20E-12	3.27E-11	13.07391	12.75456	13.23056	11.93274	11.62259	12.02275
AT4G24930	117.251	1.131216	0.216588	5.222899	1.76E-07	2.72E-06	7.201025	7.320308	7.517248	6.269954	6.244072	6.052778
AT2G42770	118.9713	1.131915	0.212249	5.332965	9.66E-08	1.55E-06	7.221208	7.387012	7.521753	6.17809	6.13862	6.259292
AT5G45680	361.6248	1.133204	0.161646	7.010404	2.38E-12	6.33E-11	8.855653	8.912886	9.135673	7.94351	7.709866	7.731173
AT5G53740	2673.866	1.13411	0.208129	5.449063	5.06E-08	8.47E-07	12.06617	11.64526	11.84387	10.52241	10.21319	11.11899
AT4G34190	627.0383	1.134365	0.16398	6.917683	4.59E-12	1.18E-10	9.681838	9.674698	9.947799	8.808946	8.229725	8.619516
AT1G54780	1080.688	1.134472	0.177165	6.40349	1.52E-10	3.37E-09	10.22652	10.48473	10.86036	9.477164	9.408366	9.247575
AT4G15510	130.213	1.137476	0.228997	4.967217	6.79E-07	9.60E-06	7.481794	7.342887	7.726857	6.640707	6.024848	6.117894
AT1G09800	999.2259	1.139829	0.201383	5.660009	1.51E-08	2.69E-07	10.17183	10.55652	10.57408	8.995433	8.959616	9.660198
AT5G59870	100.1061	1.140494	0.2318	4.920154	8.65E-07	1.20E-05	7.028513	7.050872	7.360095	6.105138	5.617177	5.937262
AT4G10150	42.19849	1.142148	0.322732	3.538997	0.000402	0.003223	5.509914	5.954364	6.159311	4.561494	5.046837	4.553113
AT1G68780	160.813	1.142499	0.191595	5.963097	2.48E-09	4.91E-08	7.921783	7.803153	7.699282	6.756396	6.602406	6.522394

AT1G58520	40.73487	1.142847	0.306118	3.733355	0.000189	0.001661	5.870916	6.034228	5.792589	4.826463	4.089834	4.673514
AT1G61300	165.5811	1.144141	0.186524	6.134007	8.57E-10	1.78E-08	7.816274	7.921888	7.810241	6.803271	6.679627	6.538339
AT4G21280	4963.71	1.14488	0.130806	8.752517	2.09E-18	8.84E-17	12.64151	12.65511	12.92415	11.71611	11.45699	11.551
AT3G26570	349.2234	1.14553	0.180701	6.339354	2.31E-10	5.03E-09	8.823009	8.780202	9.101472	7.781272	8.037767	7.465332
AT3G03780	286.6858	1.147175	0.213632	5.369859	7.88E-08	1.28E-06	8.203742	8.879298	8.792183	7.429455	7.234523	7.600575
AT1G55490	1642.784	1.148756	0.128235	8.958213	3.30E-19	1.46E-17	11.13742	11.16179	11.1618	10.17868	9.878522	9.857395
AT5G08865	750.6901	1.149638	0.163626	7.026017	2.13E-12	5.66E-11	10.0953	10.10479	9.8598	8.554188	8.912407	9.044842
AT5G24760	34.06768	1.151025	0.329904	3.488964	0.000485	0.003813	5.620423	5.739739	5.567075	4.700053	4.089834	4.116545
AT2G35720	85.44448	1.151194	0.237888	4.83922	1.30E-06	1.76E-05	6.871365	6.86369	6.99572	5.454347	5.766238	5.888334
AT3G61870	458.7977	1.152772	0.160228	7.194587	6.26E-13	1.74E-11	9.197478	9.223913	9.520362	8.26147	8.037767	8.075159
AT4G16410	553.7136	1.153047	0.159325	7.237068	4.58E-13	1.29E-11	9.605197	9.447698	9.737318	8.563295	8.037767	8.477876
AT3G14070	29.71703	1.153436	0.356376	3.236568	0.00121	0.008461	5.509914	5.535346	5.400949	3.689461	3.546624	4.673514
AT4G19985	70.62504	1.153958	0.263585	4.377944	1.20E-05	0.000136	6.658445	6.684352	6.586597	5.737858	5.450922	5.029925
AT3G56080	53.69639	1.155058	0.301945	3.825391	0.000131	0.001195	6.138352	6.43817	6.302328	4.632436	4.483533	5.489617
AT2G38740	99.37487	1.155247	0.223395	5.17131	2.32E-07	3.53E-06	7.175393	7.072651	7.034067	5.800813	6.342338	5.785202
AT2G33180	88.68611	1.155291	0.234774	4.920867	8.62E-07	1.20E-05	7.111951	6.812837	6.989228	5.861135	5.617177	5.730732
AT5G64770	415.6037	1.155941	0.214976	5.377065	7.57E-08	1.24E-06	9.089086	9.227558	9.247331	8.43522	7.74622	7.546413
AT5G02905	214.7474	1.156413	0.194012	5.960511	2.51E-09	4.98E-08	8.368889	8.351858	7.943807	7.069274	6.952811	7.053013
AT1G68890	197.0439	1.157069	0.210764	5.489879	4.02E-08	6.80E-07	7.943205	8.358522	7.999777	7.156105	6.822676	6.674431
AT5G14200	256.934	1.157603	0.209297	5.5309	3.19E-08	5.45E-07	8.297021	8.79992	8.343025	7.107129	7.128345	7.530557
AT2G03750	301.7336	1.158416	0.158118	7.326264	2.37E-13	6.84E-12	8.845937	8.672011	8.636809	7.580621	7.42597	7.546413
AT4G13560	229.4239	1.158546	0.24156	4.796108	1.62E-06	2.14E-05	8.393554	8.611927	7.983539	7.535061	6.520817	6.961367
AT5G39210	60.80087	1.158622	0.260952	4.439977	9.00E-06	0.000105	6.443349	6.429757	6.507039	5.288529	4.792505	5.315499
AT5G59350	64.08602	1.159627	0.278007	4.171213	3.03E-05	0.000318	6.210797	6.626632	6.733651	5.414645	4.792505	5.352044
AT4G03295	27.99208	1.160605	0.352202	3.295284	0.000983	0.007075	5.158689	5.535346	5.458476	4.236658	3.546624	4.116545
AT5G08710	36.16573	1.161129	0.354014	3.279891	0.001038	0.007427	5.240069	6.130813	5.584406	4.324977	4.792505	4.277199
AT5G66600	44.40875	1.16274	0.305447	3.80668	0.000141	0.001277	5.723066	6.151426	6.026132	4.632436	5.046837	4.673514
AT4G18440	138.9731	1.164313	0.207869	5.601176	2.13E-08	3.72E-07	7.400052	7.750081	7.617462	6.292034	6.602406	6.38806
AT5G05740	221.2391	1.165813	0.201265	5.792423	6.94E-09	1.29E-07	7.991008	8.415021	8.436652	7.156105	6.679627	7.190538
AT1G50250	917.9492	1.167124	0.137449	8.491345	2.04E-17	8.19E-16	10.117	10.40918	10.40577	9.127163	9.174942	9.102286
AT5G44510	159.6859	1.167259	0.195963	5.956525	2.58E-09	5.09E-08	7.786107	7.951575	7.675219	6.476879	6.602406	6.717084
AT1G65230	275.897	1.168512	0.176101	6.635448	3.24E-11	7.77E-10	8.439501	8.521787	8.758225	7.41946	7.594963	7.220496
AT1G66130	283.7518	1.17009	0.180978	6.465373	1.01E-10	2.28E-09	8.729231	8.568519	8.665592	7.709258	6.88921	7.414402

AT4G20030	66.54676	1.170931	0.25668	4.561841	5.07E-06	6.17E-05	6.665827	6.487643	6.551779	5.492986	5.262981	5.199956
AT2G18328	81.26984	1.172219	0.237749	4.930497	8.20E-07	1.15E-05	6.92772	6.799839	6.845691	5.800813	5.450922	5.522054
AT1G52030	60.39024	1.172382	0.31114	3.768026	0.000165	0.00147	6.127699	6.876128	6.312764	5.050236	4.483533	5.522054
AT5G67030	1077.482	1.173011	0.142133	8.25293	1.55E-16	5.82E-15	10.33584	10.6924	10.61419	9.423165	9.240123	9.377347
AT5G24770	194.0114	1.173225	0.287396	4.082253	4.46E-05	0.000453	7.539193	8.670227	7.860899	6.417857	6.952811	7.075043
AT2G47450	1788.424	1.173735	0.142788	8.220108	2.03E-16	7.57E-15	11.08331	11.49258	11.25031	10.17125	9.965894	10.09829
AT1G08747	109.6198	1.17515	0.245654	4.783765	1.72E-06	2.26E-05	7.128075	7.141242	7.40468	5.947137	6.679627	5.615206
AT3G09050	102.7354	1.175215	0.234495	5.01169	5.40E-07	7.76E-06	7.106536	7.25514	7.261186	6.313781	5.450922	5.785202
AT4G12030	42.91475	1.175533	0.302711	3.883343	0.000103	0.000971	6.016608	6.023086	5.865288	4.486882	4.483533	4.936752
AT3G56270	31.19859	1.175816	0.348641	3.37257	0.000745	0.005558	5.493412	5.43803	5.635183	4.632436	4.089834	3.728968
AT2G41290	32.05709	1.177645	0.337689	3.487362	0.000488	0.003831	5.708842	5.503631	5.549533	4.632436	3.546624	4.116545
AT3G23410	88.29764	1.178239	0.255624	4.609263	4.04E-06	5.01E-05	7.241112	6.900687	6.838487	6.05437	5.046837	5.674125
AT1G08633	44.24617	1.179573	0.304215	3.877437	0.000106	0.000991	5.896243	5.882204	6.238077	4.885743	4.483533	4.673514
AT4G20140	47.96463	1.179991	0.309092	3.817611	0.000135	0.001228	5.764914	6.130813	6.302328	4.885743	5.262981	4.488922
AT4G27600	648.3392	1.180027	0.146696	8.044009	8.69E-16	3.09E-14	9.831297	9.773582	9.884496	8.842996	8.399116	8.522648
AT1G64500	94.91343	1.181289	0.314403	3.757247	0.000172	0.001524	6.651025	7.673764	6.661997	5.919035	6.024848	5.456434
AT1G29920	62065.22	1.181968	0.163031	7.249945	4.17E-13	1.17E-11	16.35852	16.22703	16.60193	15.47882	14.95967	15.08145
AT2G07015	85.49268	1.182711	0.262268	4.50955	6.50E-06	7.78E-05	6.806053	7.072651	6.982708	6.129868	5.046837	5.42247
AT4G04335	130.5909	1.185427	0.207038	5.725638	1.03E-08	1.87E-07	7.36873	7.531616	7.675219	6.292034	6.244072	6.334274
AT1G75690	574.5228	1.185757	0.181252	6.542039	6.07E-11	1.41E-09	9.530334	9.523781	9.83829	8.321742	8.830121	8.165198
AT4G09975	87.3111	1.186815	0.273925	4.33263	1.47E-05	0.000164	6.825959	6.726176	7.293159	5.331804	5.617177	6.007675
AT4G24660	86.80653	1.187444	0.238855	4.971402	6.65E-07	9.40E-06	6.839079	7.061803	7.034067	5.919035	5.046837	5.758224
AT2G42220	1870.527	1.187579	0.172122	6.899623	5.21E-12	1.34E-10	11.33904	11.26086	11.47048	10.48097	9.802877	10.00096
AT2G28605	111.7314	1.192027	0.22189	5.372153	7.78E-08	1.27E-06	7.149297	7.29737	7.424062	6.17809	6.244072	5.83769
AT5G04660	29.15026	1.192423	0.353987	3.368551	0.000756	0.005631	5.390232	5.140985	5.635183	3.933736	4.483533	4.028969
AT3G03773	44.8391	1.19477	0.303403	3.937894	8.22E-05	0.000793	6.016608	5.882204	6.100294	4.324977	5.046837	4.887807
AT2G09805	208.3246	1.195189	0.196586	6.079715	1.20E-09	2.46E-08	8.106727	8.020427	8.455612	6.949358	6.822676	7.053013
AT5G38420	10489.77	1.19651	0.157566	7.593704	3.11E-14	9.74E-13	13.62095	13.6891	14.15809	12.67158	12.65857	12.51846
AT5G50100	137.6937	1.196864	0.200104	5.981207	2.21E-09	4.41E-08	7.535168	7.637693	7.5529	6.292034	6.752923	6.200381
AT1G11700	35.66726	1.197174	0.324731	3.686659	0.000227	0.001957	5.620423	5.726062	5.893371	4.408199	3.546624	4.673514
AT1G04733	960.1163	1.19841	0.207491	5.775715	7.66E-09	1.41E-07	10.12372	10.52823	10.53283	8.85417	8.8636	9.577734
AT3G07705	92.1194	1.198862	0.246828	4.857071	1.19E-06	1.62E-05	7.190827	6.780119	7.059077	5.890376	6.024848	5.489617
AT1G01900	26.95322	1.199136	0.36537	3.28198	0.001031	0.00738	5.050049	5.503631	5.278519	3.549829	4.483533	4.028969

AT3G48730	719.8688	1.19998	0.164377	7.300188	2.87E-13	8.22E-12	9.702624	10.02046	10.19417	8.789676	8.591227	8.825227
AT5G20935	42.29206	1.200628	0.312083	3.847146	0.00012	0.001104	5.792154	5.894484	6.204845	4.885743	4.089834	4.614569
AT1G74670	5161.345	1.201094	0.153066	7.846882	4.27E-15	1.43E-13	12.62279	12.83511	12.9875	11.79264	11.32501	11.60971
AT1G45474	458.5751	1.201661	0.170814	7.034915	1.99E-12	5.33E-11	9.102749	9.321502	9.550437	8.238914	8.008134	8.013753
AT3G55710	46.89726	1.201703	0.320152	3.753545	0.000174	0.001546	5.66531	6.324777	6.302328	4.997462	4.089834	4.837144
AT5G49100	184.4381	1.202635	0.187561	6.411982	1.44E-10	3.19E-09	8.025848	8.023225	8.078333	7.043474	6.434336	6.688789
AT4G04750	43.67803	1.204661	0.299164	4.026755	5.66E-05	0.000564	5.9456	6.011857	6.100294	4.942684	4.089834	4.673514
AT4G00955	283.8722	1.204896	0.175423	6.868526	6.49E-12	1.65E-10	8.584828	8.69501	8.723447	7.616061	6.822676	7.481917
AT5G08855	794.5463	1.206491	0.168692	7.152024	8.55E-13	2.35E-11	10.19178	10.24506	9.918204	8.599158	8.928315	9.096912
AT2G05620	929.6538	1.206695	0.132394	9.114437	7.91E-20	3.64E-18	10.41628	10.24625	10.38799	9.270283	9.02025	9.03925
AT3G09905	224.8213	1.207562	0.212295	5.688132	1.28E-08	2.31E-07	8.154739	8.179421	8.581781	7.336904	6.952811	6.798784
AT2G32540	153.0995	1.208165	0.19911	6.067838	1.30E-09	2.64E-08	7.730868	7.915876	7.671169	6.457472	6.244072	6.688789
AT4G25420	26.87556	1.208973	0.358226	3.374885	0.000738	0.005519	5.094491	5.369304	5.458476	4.041933	4.089834	3.836051
AT2G44230	213.0469	1.209873	0.174837	6.920006	4.52E-12	1.17E-10	8.170394	8.151554	8.363247	6.949358	7.013727	7.019324
AT3G13470	370.5945	1.210295	0.159702	7.578442	3.50E-14	1.09E-12	9.015875	9.083485	9.013045	8.024871	7.554534	7.674584
AT1G62290	41.02263	1.212229	0.307702	3.939617	8.16E-05	0.000788	5.805583	6.077955	5.836646	4.561494	4.483533	4.673514
AT2G04530	56.0502	1.214504	0.280625	4.32786	1.51E-05	0.000168	6.230845	6.269276	6.551779	4.942684	4.792505	5.239508
AT2G42975	47.62138	1.216222	0.29119	4.176736	2.96E-05	0.000311	6.127699	6.023086	6.182258	4.997462	4.792505	4.614569
AT1G29490	29.25668	1.218681	0.347696	3.505018	0.000457	0.003618	5.558315	5.351599	5.584406	4.236658	2.665087	4.35128
AT4G10300	355.1314	1.2194	0.181635	6.713471	1.90E-11	4.64E-10	8.791297	9.041309	9.122387	7.834898	7.182411	7.882497
AT4G06745	41.87524	1.222104	0.309654	3.946682	7.92E-05	0.000767	5.95768	6.088683	5.777599	4.408199	4.483533	4.837144
AT1G09340	2206.374	1.222166	0.149218	8.190479	2.60E-16	9.59E-15	11.36336	11.56207	11.83891	10.44447	10.34348	10.26447
AT5G65360	160.6927	1.222582	0.193622	6.314282	2.71E-10	5.90E-09	7.691626	7.95451	7.839406	6.476879	6.602406	6.645278
AT3G04140	40.87192	1.22385	0.304933	4.013502	5.98E-05	0.000594	5.921133	5.882204	5.987571	4.561494	4.089834	4.784636
AT3G51820	527.5432	1.22436	0.15371	7.965385	1.65E-15	5.73E-14	9.628053	9.43304	9.576654	8.503046	8.066803	8.200699
AT5G20140	172.3519	1.224974	0.208322	5.880185	4.10E-09	7.90E-08	7.849069	7.915876	8.105597	6.990448	6.13862	6.569711
AT1G47210	86.14176	1.226998	0.235162	5.217668	1.81E-07	2.79E-06	6.819354	7.02876	7.027746	5.672031	5.450922	5.758224
AT5G42070	127.7289	1.22792	0.224022	5.481257	4.22E-08	7.13E-07	7.56311	7.264631	7.703253	6.417857	6.024848	6.138962
AT1G49245	6448.36	1.228938	0.160081	7.676951	1.63E-14	5.22E-13	13.16879	12.88741	13.36308	11.958	11.6299	12.04524
AT5G07325	2658.311	1.230754	0.177551	6.931829	4.15E-12	1.08E-10	11.7475	11.70531	12.13471	10.27189	10.65207	10.84224
AT1G08470	72.70564	1.230962	0.27118	4.53928	5.64E-06	6.83E-05	6.628533	6.832118	6.694283	5.800813	5.262981	4.984091
AT1G68238	20.86815	1.231087	0.384816	3.199158	0.001378	0.00946	4.857177	5.034043	5.072871	3.689461	3.546624	3.487532
AT1G22630	178.967	1.232145	0.224157	5.496795	3.87E-08	6.55E-07	7.893765	8.039905	8.158628	6.848671	5.617177	7.007918

AT2G34860	380.9256	1.233236	0.182231	6.767428	1.31E-11	3.27E-10	8.829597	9.003543	9.354757	7.872018	7.74622	7.778931
AT1G04360	24.47255	1.233355	0.373297	3.303954	0.000953	0.0069	5.335736	5.120221	5.213173	3.395221	3.546624	4.199107
AT5G41471	53.39885	1.233439	0.294717	4.185165	2.85E-05	0.000301	6.526799	6.211554	6.051279	4.700053	5.046837	5.117444
AT4G16400	534.5064	1.2355	0.154521	7.995657	1.29E-15	4.52E-14	9.557377	9.427769	9.716837	8.454926	8.037767	8.302244
AT1G64150	115.4935	1.237064	0.229806	5.383069	7.32E-08	1.20E-06	7.211152	7.29737	7.600526	6.292034	5.90133	5.937262
AT5G55220	580.4879	1.237497	0.149923	8.254207	1.53E-16	5.77E-15	9.487232	9.761062	9.772712	8.503046	8.444073	8.311553
AT5G18080	243.3169	1.239255	0.177514	6.981188	2.93E-12	7.73E-11	8.555321	8.347398	8.436652	7.378772	6.822676	7.10747
AT1G49975	215.3971	1.24004	0.191685	6.469161	9.85E-11	2.22E-09	8.258478	8.159207	8.412597	7.249335	6.520817	6.925441
AT4G27030	86.613	1.241822	0.251868	4.930441	8.20E-07	1.15E-05	6.819354	6.954462	7.131606	5.974702	5.450922	5.387687
AT2G39470	675.7779	1.242575	0.16741	7.422368	1.15E-13	3.43E-12	9.725765	9.900014	10.07432	8.872603	8.508985	8.440181
AT5G64840	1643.667	1.244022	0.117508	10.58667	3.44E-26	2.32E-24	11.11804	11.28991	11.13007	10.00074	9.87031	9.876337
AT1G51110	282.1526	1.244056	0.195075	6.377336	1.80E-10	3.96E-09	8.426522	8.809679	8.746725	7.59845	6.822676	7.405736
AT4G13564	25.90313	1.244283	0.361652	3.440555	0.000581	0.004474	5.372295	5.259657	5.167905	4.041933	4.089834	3.613295
AT2G36145	118.3984	1.244459	0.240104	5.18299	2.18E-07	3.33E-06	7.101101	7.442428	7.679258	6.292034	5.766238	6.074811
AT5G36120	68.52288	1.245546	0.29461	4.227779	2.36E-05	0.000253	6.336387	6.495726	7.059077	5.530617	4.792505	5.278004
AT5G02915	191.4719	1.247032	0.195311	6.384857	1.72E-10	3.77E-09	8.216365	8.201828	7.839406	6.803271	6.752923	6.837962
AT3G09055	26.49584	1.247822	0.35952	3.4708	0.000519	0.004048	5.137605	5.386795	5.381252	3.689461	4.089834	4.028969
AT1G74640	76.54401	1.249827	0.25084	4.982571	6.27E-07	8.92E-06	6.605684	6.832118	6.915825	5.603056	5.450922	5.352044
AT5G24420	34.55003	1.250003	0.3556	3.515198	0.000439	0.003496	5.279101	6.067147	5.716032	4.632436	3.546624	4.199107
AT1G77090	174.1859	1.250754	0.191586	6.528415	6.65E-11	1.54E-09	7.858764	8.009177	8.022207	6.878163	6.520817	6.522394
AT1G52340	93.05908	1.253419	0.25042	5.00527	5.58E-07	8.01E-06	6.877737	7.288092	7.137489	5.947137	4.792505	5.913005
AT4G35290	34.96608	1.253734	0.335172	3.740568	0.000184	0.001618	5.493412	5.844724	5.70022	4.561494	4.483533	3.935733
AT5G53905	7504.958	1.254515	0.198959	6.305409	2.87E-10	6.24E-09	13.68241	13.07223	13.33368	12.05197	11.68244	12.40401
AT1G05207	2832.544	1.256465	0.157036	8.001122	1.23E-15	4.33E-14	11.78506	11.8471	12.24729	10.81513	10.50601	10.70594
AT1G09513	110.799	1.257604	0.235549	5.339041	9.34E-08	1.51E-06	7.280116	7.181886	7.526244	6.313781	5.617177	5.83769
AT2G34920	28.6862	1.257752	0.377174	3.334674	0.000854	0.006286	5.027304	5.519576	5.822109	3.933736	2.665087	4.421742
AT3G48187	24.73065	1.258277	0.391859	3.211045	0.001323	0.009125	5.240069	5.550946	4.997248	4.408199	2.665087	3.34975
AT2G44740	111.1455	1.258928	0.242975	5.181312	2.20E-07	3.35E-06	7.159792	7.231133	7.570401	5.70532	5.90133	6.278406
AT2G24540	55.14024	1.261001	0.280832	4.490228	7.11E-06	8.47E-05	6.426063	6.36945	6.124191	4.826463	5.262981	4.984091
AT1G06467	244.3439	1.26434	0.191826	6.591077	4.37E-11	1.04E-09	8.578975	8.541443	8.283256	7.326244	6.520817	7.259495
AT4G17880	89.04856	1.265056	0.281079	4.500707	6.77E-06	8.08E-05	7.265613	7.161707	6.718032	5.947137	4.089834	5.888334
AT1G58290	2674.424	1.265766	0.117448	10.77729	4.41E-27	3.14E-25	11.92948	11.8374	11.90226	10.75877	10.52183	10.51367
AT1G06690	160.4192	1.267885	0.226557	5.596321	2.19E-08	3.82E-07	7.551201	7.869974	8.102593	6.772191	6.342338	6.352426

AT1G60260	74.30981	1.268901	0.267129	4.750149	2.03E-06	2.64E-05	7.017014	6.705416	6.534049	5.243916	5.262981	5.615206
AT5G13770	729.4348	1.269041	0.156966	8.084833	6.22E-16	2.23E-14	9.942606	10.12773	10.00362	8.985305	8.508985	8.577693
AT1G31920	126.7511	1.271369	0.232101	5.477654	4.31E-08	7.26E-07	7.422016	7.596945	7.625856	6.570181	5.262981	6.138962
AT4G04800	86.55971	1.271682	0.243719	5.217829	1.81E-07	2.78E-06	6.999592	6.85743	6.969577	5.243916	6.024848	5.730732
AT3G21670	597.4351	1.272613	0.217303	5.856407	4.73E-09	9.03E-08	9.341573	10.00001	9.82377	8.126672	8.203818	8.772276
AT1G08103	38.02684	1.273658	0.315379	4.038494	5.38E-05	0.000539	5.921133	5.894484	5.762452	4.561494	3.546624	4.614569
AT2G21330	4123.674	1.273801	0.135195	9.421984	4.43E-21	2.23E-19	12.46181	12.4221	12.64996	11.1928	11.42371	11.03859
AT2G29650	465.7132	1.280369	0.151384	8.457768	2.73E-17	1.08E-15	9.254897	9.348533	9.524858	8.180929	7.977879	8.013753
AT2G30010	167.4309	1.281365	0.190582	6.723426	1.78E-11	4.34E-10	7.991008	7.780174	7.960495	6.707947	6.520817	6.50627
AT1G10370	45.80961	1.281438	0.320119	4.003007	6.25E-05	0.000617	5.805583	6.088683	6.353768	4.324977	4.483533	5.029925
AT1G19670	125.3975	1.281495	0.215661	5.942169	2.81E-09	5.51E-08	7.514872	7.623008	7.389971	6.417857	5.90133	6.030403
AT3G02020	201.1403	1.281879	0.202638	6.325948	2.52E-10	5.48E-09	7.964314	8.415021	8.093543	6.674719	6.952811	6.937516
AT1G06273	39.12549	1.282231	0.318547	4.02525	5.69E-05	0.000567	5.635541	5.954364	5.907211	4.236658	4.792505	4.488922
AT1G14700	122.7274	1.282312	0.204445	6.272176	3.56E-10	7.63E-09	7.498428	7.459059	7.503648	6.269954	5.766238	6.180197
AT3G47430	83.71916	1.283629	0.248296	5.169761	2.34E-07	3.55E-06	7.045591	6.825719	7.008616	5.890376	4.792505	5.553778
AT1G74730	392.8064	1.284044	0.15735	8.160418	3.34E-16	1.22E-14	9.073905	9.017646	9.302043	7.901041	7.74622	7.785626
AT2G47010	55.33366	1.285861	0.281768	4.563546	5.03E-06	6.13E-05	6.200668	6.395605	6.323125	4.632436	5.450922	4.984091
AT1G35180	31.01205	1.286258	0.368272	3.492684	0.000478	0.003766	5.635541	5.726062	5.341032	4.700053	3.546624	3.487532
AT4G19020	102.2772	1.288115	0.230483	5.588758	2.29E-08	3.99E-07	7.250962	7.360699	7.077554	5.800813	5.450922	6.096513
AT1G07803	29.71483	1.288186	0.350136	3.679102	0.000234	0.002004	5.390232	5.503631	5.716032	4.142579	2.665087	4.35128
AT5G24120	163.0663	1.292868	0.195094	6.626886	3.43E-11	8.21E-10	8.022977	7.819346	7.806553	6.707947	6.342338	6.473473
AT4G04485	181.4049	1.293932	0.25629	5.048699	4.45E-07	6.46E-06	8.057062	7.581837	8.395516	7.017205	6.434336	6.38806
AT3G28170	23.42517	1.294216	0.402403	3.216216	0.001299	0.008999	5.050049	5.698312	4.74322	3.816762	2.665087	3.836051
AT5G04190	419.3446	1.294935	0.1709	7.577162	3.53E-14	1.10E-12	9.160068	9.130964	9.410478	8.138907	7.709866	7.765446
AT3G58990	35.06774	1.296293	0.335189	3.867353	0.00011	0.001027	5.679967	5.857326	5.792589	4.236658	2.665087	4.730144
AT3G57040	197.7871	1.296624	0.174441	7.433016	1.06E-13	3.17E-12	8.128263	8.22875	8.093543	6.848671	6.822676	6.798784
AT5G65010	1104.558	1.297436	0.144259	8.993786	2.39E-19	1.07E-17	10.60954	10.65995	10.6036	9.505788	9.314642	9.058728
AT2G26870	40.75501	1.297454	0.312504	4.151802	3.30E-05	0.000343	5.832072	5.954364	6.03876	4.826463	4.089834	4.35128
AT1G15810	1072.297	1.300159	0.143116	9.084626	1.04E-19	4.76E-18	10.54945	10.76901	10.41851	9.369663	9.148022	9.225585
AT3G18280	33.60755	1.303881	0.328659	3.96728	7.27E-05	0.000708	5.635541	5.753287	5.601531	4.324977	4.089834	4.199107
AT1G29820	42.91688	1.305711	0.312247	4.181656	2.89E-05	0.000305	5.993325	5.93071	5.893371	4.561494	5.262981	4.116545
AT5G49740	713.1097	1.305945	0.138809	9.408235	5.05E-21	2.53E-19	9.968955	9.989313	10.05658	8.85417	8.42177	8.626991
AT2G33530	89.99144	1.306873	0.289587	4.512879	6.40E-06	7.66E-05	6.964104	6.966144	7.021398	4.826463	6.520817	5.522054

AT2G20723	18.23856	1.307563	0.410145	3.188049	0.001432	0.009773	5.072441	4.712023	4.944537	3.395221	0	3.728968
AT3G44205	19.23242	1.308985	0.400107	3.271588	0.001069	0.007619	4.77764	5.161454	4.861673	3.395221	2.665087	3.613295
AT4G17600	389.9487	1.309638	0.151035	8.671074	4.28E-18	1.79E-16	9.005729	9.129666	9.233666	7.834898	7.850087	7.717232
AT4G02630	68.8183	1.311276	0.2928	4.478406	7.52E-06	8.89E-05	6.381922	7.011951	6.637299	5.150324	4.483533	5.615206
AT3G59410	1016.617	1.3132	0.13279	9.889301	4.63E-23	2.64E-21	10.4831	10.41771	10.61577	9.286871	9.201369	9.033636
AT3G60440	48.26719	1.315864	0.291222	4.518423	6.23E-06	7.49E-05	6.084284	6.278675	6.159311	4.942684	4.483533	4.673514
AT4G30610	48.37609	1.316066	0.294336	4.471297	7.77E-06	9.16E-05	6.180193	6.023086	6.323125	4.942684	4.483533	4.673514
AT4G13770	424.8678	1.31768	0.178569	7.379106	1.59E-13	4.68E-12	9.066952	9.457042	9.24869	7.757664	7.709866	8.139294
AT2G46570	35.23464	1.317888	0.339874	3.877581	0.000106	0.000991	5.805583	5.43803	5.934498	4.561494	4.089834	4.028969
AT5G54075	8636.352	1.318518	0.20035	6.581075	4.67E-11	1.11E-09	13.90221	13.27362	13.57386	12.19139	11.84421	12.56591
AT5G03555	147.5183	1.318888	0.216761	6.084518	1.17E-09	2.39E-08	7.727344	7.581837	7.817587	6.417857	6.822676	5.984584
AT5G59480	87.7285	1.320849	0.242865	5.43861	5.37E-08	8.96E-07	7.190827	6.994943	6.867092	5.567291	5.262981	5.811684
AT5G44680	1039.871	1.321976	0.135657	9.744958	1.94E-22	1.06E-20	10.39423	10.55748	10.67663	9.311401	9.106676	9.152359
AT5G00590	73.47336	1.323074	0.275435	4.803579	1.56E-06	2.06E-05	6.744632	6.519708	7.046626	5.243916	4.792505	5.644966
AT1G79520	2003.886	1.326981	0.157217	8.440445	3.16E-17	1.25E-15	11.34422	11.59982	11.50985	10.01242	10.4575	9.922632
AT1G11870	844.4657	1.32759	0.159054	8.346763	7.02E-17	2.72E-15	10.15946	10.09817	10.47941	8.908777	8.649971	9.030821
AT4G22510	28.14318	1.327616	0.372627	3.562858	0.000367	0.002971	5.805583	5.333673	5.121171	4.236658	3.546624	3.728968
AT5G17300	200.6505	1.327902	0.287107	4.625104	3.74E-06	4.67E-05	8.615651	7.966193	7.813919	6.674719	7.42597	6.096513
AT1G13080	89.45064	1.327995	0.237409	5.593693	2.22E-08	3.88E-07	7.017014	7.1257	6.895141	5.414645	5.90133	5.702706
AT3G47342	68.91662	1.328969	0.322663	4.118755	3.81E-05	0.000392	6.758509	7.104714	5.94795	5.197878	5.262981	5.199956
AT5G17050	64.75423	1.330367	0.271165	4.906123	9.29E-07	1.29E-05	6.605684	6.604377	6.620596	5.567291	4.483533	4.984091
AT5G20410	37.93589	1.330391	0.329026	4.043418	5.27E-05	0.000528	5.574094	5.942585	6.06369	4.486882	3.546624	4.553113
AT1G50320	437.5205	1.332699	0.172325	7.733656	1.05E-14	3.39E-13	9.112236	9.199376	9.563605	7.915337	7.915398	7.967393
AT4G17730	457.2652	1.333981	0.169627	7.864191	3.71E-15	1.25E-13	9.537394	9.232403	9.356018	8.227502	7.63429	7.973271
AT3G63140	1280.453	1.336541	0.169201	7.899145	2.81E-15	9.57E-14	10.60571	10.75897	11.13816	9.610535	9.507003	9.309281
AT3G01500	5905.08	1.337316	0.167898	7.965056	1.65E-15	5.74E-14	12.74272	13.04271	13.32557	11.72171	11.84001	11.46914
AT4G38860	861.3099	1.337382	0.178136	7.507626	6.02E-14	1.83E-12	10.32247	10.20062	10.35492	9.184356	8.328941	8.976262
AT4G08300	26.47565	1.337394	0.381704	3.503743	0.000459	0.003633	5.199953	5.670019	5.381252	3.816762	0	4.35128
AT5G08050	416.5354	1.337656	0.185761	7.200961	5.98E-13	1.67E-11	9.166587	9.113999	9.44528	8.157066	7.47011	7.758657
AT1G05243	16.64771	1.344169	0.418235	3.21391	0.001309	0.009059	4.57299	4.655421	4.861673	2.797163	3.546624	3.197407
AT3G54490	57.58395	1.347327	0.311185	4.329665	1.49E-05	0.000166	6.597987	6.698429	6.051279	5.288529	3.546624	5.074348
AT5G53902	9628.789	1.347663	0.204956	6.575392	4.85E-11	1.15E-09	14.05791	13.43339	13.75768	12.31245	11.9493	12.7248
AT4G28740	128.8465	1.350266	0.214948	6.281819	3.35E-10	7.20E-09	7.555181	7.487711	7.625856	6.397634	6.13862	5.888334

AT1G18060	257.4422	1.351449	0.173157	7.804777	5.96E-15	1.97E-13	8.441653	8.505867	8.667626	7.27173	7.234523	6.99642
AT3G28070	19.62871	1.353387	0.408981	3.309165	0.000936	0.006806	4.541232	5.120221	4.971133	3.025182	4.089834	3.197407
AT1G07010	178.1152	1.353887	0.179555	7.54025	4.69E-14	1.44E-12	8.011433	8.059123	8.003003	6.605875	6.520817	6.703006
AT5G43630	92.99215	1.356037	0.254501	5.328228	9.92E-08	1.59E-06	7.221208	7.211635	6.956326	6.079977	4.792505	5.553778
AT5G62430	267.8985	1.356131	0.161955	8.373512	5.59E-17	2.19E-15	8.638343	8.587553	8.605319	7.368418	7.072174	7.139183
AT5G01515	122.89	1.356339	0.207911	6.523645	6.86E-11	1.59E-09	7.498428	7.483652	7.476057	6.224753	6.13862	5.913005
AT4G13494	99.44745	1.356394	0.243838	5.562692	2.66E-08	4.60E-07	7.395619	7.269354	6.895141	5.800813	5.617177	5.811684
AT4G13493	63.67361	1.35663	0.282579	4.800895	1.58E-06	2.09E-05	6.680479	6.691408	6.204845	4.997462	5.450922	5.029925
AT3G28080	37.94378	1.357972	0.327938	4.140947	3.46E-05	0.000359	5.792154	5.857326	5.865288	3.816762	4.483533	4.673514
AT3G28760	50.25552	1.358191	0.294323	4.614632	3.94E-06	4.88E-05	6.016608	6.297293	6.403437	4.885743	4.483533	4.784636
AT2G23000	33.0899	1.358591	0.397397	3.41873	0.000629	0.004796	4.956834	6.011857	5.747144	3.025182	4.792505	4.277199
AT3G05936	27.95506	1.358734	0.367669	3.695539	0.000219	0.001896	5.050049	5.550946	5.584406	4.041933	4.089834	3.613295
AT1G10360	217.4145	1.35922	0.198621	6.843301	7.74E-12	1.95E-10	8.557307	8.080778	8.2806	6.976881	6.679627	6.949491
AT3G51075	18.68697	1.359963	0.410532	3.312685	0.000924	0.00674	4.750123	5.161454	4.647527	3.395221	3.546624	3.027056
AT4G12390	107.2603	1.365392	0.231154	5.906843	3.49E-09	6.77E-08	7.34133	7.109989	7.476057	5.800813	5.617177	6.052778
AT2G37250	846.7635	1.3669	0.142488	9.593065	8.55E-22	4.48E-20	10.21272	10.3379	10.25969	8.831735	8.591227	9.072481
AT1G04223	47.69451	1.37078	0.291376	4.704502	2.54E-06	3.25E-05	6.169846	6.141156	6.259813	4.826463	4.089834	4.784636
AT3G05715	905.4677	1.371747	0.159067	8.623708	6.48E-18	2.69E-16	10.49039	10.12317	10.45665	8.857876	8.813085	9.144567
AT1G07393	19.2955	1.377684	0.425369	3.238796	0.0012	0.008405	4.804643	5.404077	4.580013	3.816762	2.665087	2.833864
AT4G38850	71.74062	1.379585	0.264941	5.207133	1.92E-07	2.94E-06	6.526799	6.780119	6.749102	5.101148	5.90133	5.029925
AT1G10470	255.1355	1.379785	0.269321	5.1232	3.00E-07	4.48E-06	8.430861	8.515837	8.739008	6.356317	6.822676	7.667351
AT5G63180	382.5143	1.381681	0.175252	7.883966	3.17E-15	1.08E-13	9.295915	8.960388	9.120903	7.936518	7.51294	7.569875
AT2G04110	97.20908	1.382589	0.234428	5.897718	3.69E-09	7.13E-08	7.117346	7.141242	7.200663	5.919035	5.90133	5.387687
AT3G54500	2504.154	1.38285	0.167392	8.261125	1.44E-16	5.45E-15	12.0816	11.75688	11.60976	10.40449	10.64727	10.16912
AT2G00850	263.4241	1.384172	0.191784	7.21733	5.30E-13	1.48E-11	8.649557	8.376143	8.769633	7.378772	6.752923	7.149602
AT2G09685	48.67743	1.38507	0.292796	4.730492	2.24E-06	2.90E-05	6.127699	6.191788	6.193595	4.408199	5.046837	4.784636
AT1G26945	265.0317	1.387424	0.210395	6.594365	4.27E-11	1.01E-09	8.218877	8.650451	8.884273	7.144016	7.013727	7.259495
AT1G32780	19.74179	1.389581	0.406468	3.418675	0.000629	0.004796	4.932558	4.655421	5.235286	3.025182	3.546624	3.487532
AT4G07135	50.37022	1.389878	0.314111	4.424795	9.65E-06	0.000112	6.582468	6.00054	6.147699	4.486882	4.483533	5.029925
AT3G08405	96.4616	1.390722	0.263251	5.282873	1.27E-07	2.02E-06	7.502556	7.061803	6.831246	5.769679	5.766238	5.553778
AT5G67150	37.50888	1.39132	0.320167	4.345606	1.39E-05	0.000156	5.981541	5.844724	5.777599	4.486882	3.546624	4.421742
AT1G10640	26.28934	1.39133	0.361577	3.847954	0.000119	0.001101	5.259717	5.421153	5.299659	3.689461	4.089834	3.728968
AT5G04950	67.47987	1.392661	0.280004	4.973715	6.57E-07	9.30E-06	6.558872	6.589348	6.922655	5.530617	4.089834	5.15929

AT1G06227	352.2658	1.393127	0.227083	6.134885	8.52E-10	1.77E-08	8.821357	9.347417	8.794047	7.069274	7.78168	7.765446
AT3G23000	323.1352	1.395674	0.20059	6.957836	3.46E-12	9.05E-11	9.132359	8.945711	8.557854	7.347485	7.234523	7.645434
AT5G40270	45.89308	1.396062	0.305189	4.574413	4.78E-06	5.84E-05	5.95768	6.191788	6.204845	4.885743	4.483533	4.35128
AT4G06305	164.9322	1.396775	0.226284	6.172661	6.71E-10	1.40E-08	8.185881	7.641341	7.973708	6.514927	5.90133	6.674431
AT1G04250	128.548	1.398216	0.24312	5.75114	8.86E-09	1.63E-07	7.284918	7.680871	7.683285	5.637956	6.342338	6.315891
AT5G17170	269.1499	1.399173	0.189642	7.377988	1.61E-13	4.72E-12	8.402419	8.617493	8.859573	7.27173	6.88921	7.249844
AT3G06145	13.83961	1.400458	0.440194	3.181457	0.001465	0.009959	4.296891	4.470931	4.74322	2.797163	2.665087	2.833864
AT1G27480	61.87427	1.400928	0.269309	5.201929	1.97E-07	3.02E-06	6.51049	6.47134	6.694283	5.197878	4.483533	5.117444
AT4G24700	19.69149	1.402721	0.39749	3.528945	0.000417	0.003337	4.907866	5.077776	4.997248	3.689461	2.665087	3.197407
AT5G02890	102.5066	1.403107	0.219679	6.387087	1.69E-10	3.73E-09	7.221208	7.292738	7.277261	5.831289	5.450922	5.888334
AT5G63530	88.68425	1.407382	0.32203	4.37034	1.24E-05	0.000141	6.451914	7.666621	6.779516	5.373819	5.617177	5.553778
AT3G02730	583.516	1.407853	0.192674	7.306925	2.73E-13	7.85E-12	9.484104	9.634996	10.06895	8.48399	7.977879	8.283446
AT5G28626	36.53312	1.41016	0.389935	3.616398	0.000299	0.002481	5.993325	5.684235	5.865288	4.997462	3.546624	3.027056
AT4G23290	162.791	1.411043	0.205595	6.863218	6.73E-12	1.71E-10	7.723812	7.89769	8.111587	6.417857	6.244072	6.569711
AT1G29720	19.43533	1.411165	0.412615	3.420052	0.000626	0.004785	5.179468	4.844514	4.614164	2.797163	4.089834	3.197407
AT4G06255	162.4705	1.412572	0.227036	6.2218	4.91E-10	1.04E-08	8.165194	7.611896	7.967117	6.476879	5.90133	6.630478
AT3G02045	176.7479	1.420266	0.187326	7.58178	3.41E-14	1.06E-12	7.988066	8.011998	8.138236	6.674719	6.13862	6.645278
AT2G12020	40.76144	1.420886	0.349219	4.068754	4.73E-05	0.000478	5.620423	6.43817	5.762452	4.236658	4.089834	4.614569
AT5G18020	266.8077	1.420909	0.171286	8.295546	1.08E-16	4.12E-15	8.549347	8.595096	8.679771	7.293783	7.284819	6.937516
AT1G29071	19.07746	1.422439	0.408835	3.479251	0.000503	0.003934	4.634483	5.201541	4.997248	3.222033	2.665087	3.487532
AT3G02795	190.833	1.423147	0.209199	6.802849	1.03E-11	2.58E-10	8.167796	8.362947	7.828538	6.657813	6.752923	6.615524
AT3G05655	74.39437	1.423954	0.255155	5.58075	2.39E-08	4.16E-07	6.785868	6.948585	6.653811	5.414645	5.262981	5.199956
AT3G46130	14.15576	1.426496	0.445131	3.204668	0.001352	0.009305	4.634483	4.818971	4.138765	2.526213	2.665087	3.027056
AT1G27030	55.91666	1.429703	0.279031	5.123822	2.99E-07	4.47E-06	6.408569	6.487643	6.259813	4.885743	5.046837	4.784636
AT4G28250	46.31737	1.429936	0.30786	4.644767	3.40E-06	4.27E-05	5.908741	6.181803	6.323125	4.408199	4.483533	4.784636
AT1G75100	648.1494	1.430463	0.145379	9.839574	7.60E-23	4.26E-21	9.97491	9.856737	9.867784	8.642768	8.255176	8.329991
AT3G48200	333.3123	1.430525	0.173686	8.236278	1.78E-16	6.66E-15	8.777734	9.108738	8.918841	7.63346	7.42597	7.325306
AT3G53830	45.06697	1.431978	0.312946	4.575804	4.74E-06	5.81E-05	6.084284	6.306513	5.836646	4.324977	4.792505	4.614569
AT4G07145	48.82689	1.432332	0.323254	4.430981	9.38E-06	0.000109	6.582468	5.977637	6.112292	4.408199	4.089834	5.029925
AT4G00163	35.96875	1.433222	0.342755	4.181478	2.90E-05	0.000305	5.574094	5.832011	6.03876	3.933736	3.546624	4.614569
AT3G54600	106.6166	1.434114	0.268134	5.348504	8.87E-08	1.43E-06	7.017014	7.459059	7.517248	6.247531	5.046837	5.584819
AT4G38970	6972.156	1.434913	0.137367	10.44586	1.53E-25	9.90E-24	13.13145	13.23755	13.55702	11.79988	11.91379	11.86534
AT5G55260	54.08866	1.434918	0.281725	5.093332	3.52E-07	5.20E-06	6.47731	6.250291	6.333412	4.826463	4.792505	4.837144

AT1G16820	2099.267	1.437728	0.154993	9.276109	1.76E-20	8.60E-19	11.54522	11.63615	11.60765	10.07902	9.776754	10.39936
AT1G29510	215.9562	1.440141	0.187195	7.693255	1.43E-14	4.62E-13	8.437346	8.264671	8.29383	7.094621	6.520817	6.717084
AT3G47380	21.62733	1.442415	0.41796	3.451086	0.000558	0.004318	4.634483	5.27852	5.213173	3.689461	4.089834	2.346685
AT4G25050	1452.637	1.444075	0.14967	9.648364	4.99E-22	2.65E-20	11.09771	10.92642	11.17079	9.776345	9.252813	9.628893
AT1G04013	125.195	1.446293	0.238752	6.057716	1.38E-09	2.81E-08	7.506673	7.329382	7.817587	6.313781	5.617177	5.913005
AT1G05427	27.7309	1.446427	0.357394	4.047147	5.18E-05	0.000521	5.542361	5.333673	5.477153	3.689461	3.546624	4.028969
AT3G57765	2075.609	1.446601	0.159555	9.066469	1.23E-19	5.62E-18	11.49249	11.63043	11.62109	9.980486	9.802877	10.40372
AT4G04850	204.129	1.44681	0.193163	7.49009	6.88E-14	2.08E-12	8.130932	8.393552	8.256476	6.740426	6.244072	6.973146
AT2G32340	30.09885	1.448976	0.357759	4.05014	5.12E-05	0.000514	5.259717	5.670019	5.584406	3.549829	4.483533	3.935733
AT3G01550	40.89218	1.449777	0.312926	4.632969	3.60E-06	4.50E-05	5.921133	5.942585	6.088196	4.632436	4.089834	4.277199
AT1G22430	297.3651	1.451671	0.178471	8.133948	4.16E-16	1.51E-14	8.827953	8.755168	8.816221	7.589563	6.822676	7.200593
AT4G00165	39.27267	1.455368	0.335748	4.3347	1.46E-05	0.000163	5.679967	5.918735	6.17083	3.933736	4.089834	4.673514
AT1G09883	25.28579	1.456182	0.369438	3.941617	8.09E-05	0.000782	5.259717	5.519576	5.278519	3.816762	2.665087	3.836051
AT3G48500	146.9469	1.456342	0.227596	6.398805	1.57E-10	3.46E-09	7.881135	7.885438	7.591983	6.605875	5.90133	6.030403
AT1G62780	189.3684	1.456344	0.198986	7.318831	2.50E-13	7.20E-12	7.927936	8.201828	8.285907	6.803271	6.434336	6.55411
AT5G05860	89.5241	1.456431	0.242853	5.997176	2.01E-09	4.02E-08	7.154554	6.948585	7.002182	5.243916	6.024848	5.522054
AT5G10930	61.87733	1.458808	0.281394	5.184222	2.17E-07	3.31E-06	6.354774	6.691408	6.702243	5.050236	4.089834	5.239508
AT2G10735	19.5779	1.459095	0.402949	3.621045	0.000293	0.00244	5.072441	4.739512	5.048102	3.025182	3.546624	3.34975
AT4G08735	39.29209	1.459506	0.322261	4.528954	5.93E-06	7.15E-05	5.764914	6.141156	5.865288	4.324977	4.089834	4.421742
AT1G32220	219.9847	1.459653	0.211325	6.907138	4.95E-12	1.27E-10	8.04012	8.320346	8.622199	6.878163	7.013727	6.674431
AT5G06790	16.96506	1.461128	0.420851	3.471841	0.000517	0.004035	4.980709	4.684	4.889826	3.549829	0	3.027056
AT4G38840	347.0063	1.461223	0.168492	8.672349	4.23E-18	1.77E-16	9.197478	8.866887	8.930747	7.429455	7.51294	7.577611
AT4G34060	16.90207	1.461317	0.442058	3.305714	0.000947	0.006875	4.136111	5.077776	5.048102	3.395221	2.665087	2.610742
AT3G14170	29.95991	1.467178	0.347839	4.217977	2.47E-05	0.000263	5.542361	5.655661	5.495591	4.142579	3.546624	3.836051
AT5G02255	22.72406	1.468131	0.433561	3.386219	0.000709	0.005319	4.72207	4.626265	5.822109	3.395221	4.089834	2.833864
AT5G14565	20.4712	1.468278	0.393714	3.729297	0.000192	0.001685	4.956834	4.988944	5.121171	3.222033	3.546624	3.34975
AT2G23672	138.2311	1.471885	0.218241	6.744317	1.54E-11	3.81E-10	7.796233	7.574224	7.784228	6.457472	5.450922	6.138962
AT5G63980	93.73007	1.476265	0.245877	6.004073	1.92E-09	3.86E-08	7.017014	7.211635	7.154994	5.831289	5.766238	5.199956
AT1G15820	17630.61	1.477717	0.161352	9.158337	5.27E-20	2.47E-18	14.46995	14.54537	14.95661	13.31922	12.94032	13.18427
AT4G19865	27.92565	1.482356	0.366472	4.044938	5.23E-05	0.000525	5.679967	5.503631	5.190717	4.041933	3.546624	3.613295
AT4G12830	22.56627	1.483378	0.383658	3.866403	0.00011	0.001031	5.11621	5.140985	5.235286	3.689461	3.546624	3.197407
AT2G15128	13.7298	1.485333	0.444343	3.342762	0.000829	0.006128	4.77764	4.369011	4.395959	2.526213	2.665087	2.833864
AT5G14545	76.32585	1.48696	0.314843	4.722855	2.33E-06	3.00E-05	7.195935	6.558811	6.915825	5.831289	3.546624	5.074348

AT2G40520	71.50267	1.488938	0.262824	5.665147	1.47E-08	2.62E-07	6.964104	6.719289	6.603697	5.197878	5.046837	5.239508
AT5G59750	177.4048	1.492941	0.187691	7.954267	1.80E-15	6.23E-14	8.014328	8.080778	8.069129	6.691428	6.434336	6.370353
AT4G26850	520.8973	1.49352	0.158135	9.444589	3.57E-21	1.80E-19	9.76152	9.454971	9.589586	8.057462	7.816289	8.225533
AT2G39330	26.33929	1.493934	0.430626	3.469216	0.000522	0.004068	4.441535	6.067147	5.320494	3.222033	3.546624	3.836051
AT5G22390	68.0745	1.49455	0.262542	5.692616	1.25E-08	2.25E-07	6.758509	6.558811	6.801913	5.243916	4.792505	5.117444
AT2G40670	16.43456	1.495388	0.424309	3.524285	0.000425	0.00339	4.634483	4.844514	4.971133	3.395221	0	3.027056
AT4G01883	58.74394	1.495825	0.277988	5.380902	7.41E-08	1.21E-06	6.317763	6.551075	6.595172	4.997462	4.792505	4.837144
AT3G03820	179.6161	1.498179	0.210547	7.115661	1.11E-12	3.04E-11	8.04012	8.128345	8.161518	6.787815	5.450922	6.645278
AT5G19730	18.2895	1.500697	0.414214	3.622995	0.000291	0.002424	4.980709	4.894281	4.74322	2.526213	3.546624	3.34975
AT2G23670	137.0227	1.50152	0.21725	6.911487	4.80E-12	1.24E-10	7.796233	7.562728	7.776709	6.397634	5.450922	6.117894
AT3G46900	51.64848	1.503049	0.304037	4.943645	7.67E-07	1.08E-05	6.230845	6.201705	6.525102	4.324977	4.483533	5.029925
AT3G01145	77.37924	1.503291	0.320543	4.689828	2.73E-06	3.48E-05	6.806053	7.115245	6.497922	4.700053	6.244072	4.784636
AT3G54050	2208.277	1.505049	0.1511	9.960634	2.27E-23	1.31E-21	11.52507	11.65206	11.85596	10.37117	10.01886	9.996634
AT1G05273	2035.283	1.505126	0.155986	9.649106	4.96E-22	2.64E-20	11.51335	11.61868	11.57713	9.980486	9.676708	10.30582
AT5G27660	224.9762	1.506435	0.204084	7.38143	1.57E-13	4.61E-12	8.125588	8.380515	8.63265	6.657813	6.952811	6.925441
AT1G03630	582.3976	1.507847	0.150468	10.02106	1.23E-23	7.21E-22	9.63277	9.762738	9.88537	8.379596	8.150563	8.102229
AT3G04790	336.5016	1.509476	0.183138	8.242304	1.69E-16	6.35E-15	8.709721	8.977805	9.20454	7.39926	7.380437	7.465332
AT5G57345	730.9038	1.511105	0.163002	9.270447	1.85E-20	9.04E-19	9.953203	10.10082	10.20472	8.758298	8.571102	8.269184
AT5G18030	365.0758	1.511739	0.175025	8.637266	5.76E-18	2.39E-16	9.171782	8.957464	9.177672	7.804499	7.182411	7.448554
AT3G10840	40.3895	1.512664	0.31992	4.728254	2.26E-06	2.93E-05	5.9456	5.857326	6.193595	4.486882	3.546624	4.421742
AT4G27595	67.57768	1.512853	0.303686	4.981635	6.30E-07	8.95E-06	6.298895	7.109989	6.645579	5.101148	4.483533	5.239508
AT3G47347	113.1343	1.513033	0.218595	6.92163	4.46E-12	1.15E-10	7.464966	7.503831	7.28258	5.890376	5.766238	5.83769
AT3G59780	1086.324	1.514033	0.141367	10.70994	9.14E-27	6.42E-25	10.5141	10.75097	10.71352	9.292358	9.02025	9.002363
AT5G51720	23.67334	1.514205	0.380291	3.981706	6.84E-05	0.000669	5.158689	5.181636	5.477153	3.689461	2.665087	3.613295
AT1G06087	86.03618	1.514316	0.282412	5.362086	8.23E-08	1.34E-06	6.958103	7.274061	6.787021	4.764642	5.766238	5.674125
AT3G04165	453.3772	1.516081	0.204322	7.420057	1.17E-13	3.48E-12	9.118975	9.645025	9.374816	7.488014	8.255176	7.785626
AT1G64860	822.1413	1.516737	0.178948	8.475858	2.33E-17	9.33E-16	10.09188	10.52231	10.15484	8.968265	8.48767	8.558274
AT4G27700	550.4701	1.517626	0.178343	8.509582	1.75E-17	7.05E-16	9.540409	9.747587	9.793353	8.440172	7.709866	8.025114
AT2G30766	66.58864	1.518898	0.363378	4.179943	2.92E-05	0.000307	6.819354	6.760126	6.281227	3.549829	6.024848	4.887807
AT2G13550	18.63238	1.519076	0.42458	3.577829	0.000346	0.00283	5.220151	5.01167	4.580013	2.526213	2.665087	3.613295
AT3G07715	1010.656	1.520231	0.171879	8.844765	9.17E-19	3.99E-17	10.75421	10.65228	10.24886	9.05152	8.724751	9.136733
AT5G38430	4851.056	1.522804	0.156412	9.735827	2.12E-22	1.16E-20	12.64747	12.64083	13.1232	11.25524	11.36647	11.16914
AT4G02850	29.36786	1.522922	0.357515	4.259745	2.05E-05	0.000222	5.679967	5.315522	5.635183	4.041933	3.546624	3.728968

AT1G29450	295.7468	1.524056	0.214536	7.10397	1.21E-12	3.30E-11	8.865304	8.639548	8.88077	7.580621	7.182411	6.744835
AT4G10060	405.7664	1.525963	0.159987	9.538068	1.46E-21	7.52E-20	9.104108	9.365173	9.279609	7.842399	7.51294	7.623179
AT1G63240	156.1052	1.526033	0.235154	6.489504	8.61E-11	1.96E-09	7.559151	8.083462	7.976993	6.570181	6.13862	6.030403
AT1G04277	89.3133	1.528424	0.253383	6.032069	1.62E-09	3.28E-08	6.921566	7.104714	7.089742	5.414645	6.13862	5.074348
AT1G62540	22.19737	1.529349	0.392387	3.897554	9.72E-05	0.000923	5.158689	5.297139	5.235286	3.933736	0	3.34975
AT4G32770	79.60872	1.530379	0.277516	5.514557	3.50E-08	5.94E-07	6.636069	7.067237	7.160782	5.492986	4.089834	5.489617
AT5G58770	20.44012	1.5315	0.421682	3.631883	0.000281	0.002353	5.425452	5.034043	4.889826	2.797163	0	3.935733
AT1G52240	1212.573	1.532824	0.196555	7.798436	6.27E-15	2.06E-13	10.72535	10.71897	11.04929	9.472338	8.611075	9.414539
AT5G61420	72.55435	1.532867	0.268178	5.715848	1.09E-08	1.98E-07	6.643567	7.017576	6.794486	5.150324	4.483533	5.42247
AT5G04140	5691.162	1.537225	0.184212	8.344885	7.13E-17	2.75E-15	12.54885	13.303	13.21838	11.43839	11.53698	11.4392
AT1G08643	69.2738	1.540177	0.261666	5.886041	3.96E-09	7.63E-08	6.636069	6.851144	6.702243	5.243916	5.046837	4.984091
AT1G45201	159.6237	1.540469	0.213064	7.230069	4.83E-13	1.35E-11	7.772494	8.146428	7.761553	6.292034	6.520817	6.220286
AT1G78370	428.664	1.54838	0.205364	7.539697	4.71E-14	1.45E-12	8.943312	9.584729	9.441718	7.725575	7.380437	7.931619
AT1G44575	3208.789	1.549626	0.152955	10.13127	4.01E-24	2.41E-22	12.00382	12.15319	12.49793	10.75976	10.62304	10.55246
AT1G14150	173.6383	1.550804	0.213501	7.263672	3.77E-13	1.07E-11	7.890617	7.903778	8.264562	6.551997	6.602406	6.180197
AT1G62560	44.41299	1.551364	0.356017	4.357553	1.32E-05	0.000149	5.845137	6.511758	6.051279	3.816762	3.546624	4.984091
AT3G17890	188.3359	1.554189	0.185777	8.365903	5.97E-17	2.33E-15	8.106727	8.120526	8.155732	6.496028	6.952811	6.370353
AT4G37930	5777.126	1.554216	0.140843	11.03508	2.59E-28	1.95E-26	12.80808	13.11279	13.28233	11.48885	11.53439	11.46966
AT1G06537	27.90024	1.555767	0.361651	4.301843	1.69E-05	0.000187	5.390232	5.670019	5.381252	3.689461	3.546624	3.836051
AT1G35560	56.76195	1.562886	0.277056	5.641047	1.69E-08	2.98E-07	6.460429	6.495726	6.460867	4.764642	4.483533	4.936752
AT1G12570	29.24674	1.56338	0.370547	4.219111	2.45E-05	0.000262	5.372295	5.712254	5.495591	3.025182	4.089834	4.116545
AT4G25290	86.41818	1.564634	0.250472	6.246751	4.19E-10	8.92E-09	7.090168	7.171832	6.956326	5.737858	4.792505	5.278004
AT5G01505	84.68895	1.56547	0.247822	6.316921	2.67E-10	5.81E-09	6.832534	7.151511	7.077554	5.414645	5.262981	5.387687
AT5G08155	2972.531	1.566452	0.211156	7.41844	1.19E-13	3.52E-12	12.46658	11.72112	12.13085	10.39304	10.14675	10.84705
AT1G16070	21.0791	1.567657	0.416957	3.75976	0.00017	0.001512	5.137605	5.077776	5.072871	3.816762	3.546624	2.023222
AT5G67370	141.0214	1.568146	0.203413	7.709169	1.27E-14	4.09E-13	7.826191	7.729664	7.630034	6.028299	6.342338	6.096513
AT4G22513	20.25848	1.569087	0.410616	3.821306	0.000133	0.001212	5.425452	4.988944	4.74322	3.395221	2.665087	3.197407
AT3G62030	2077.406	1.57188	0.188911	8.320724	8.74E-17	3.36E-15	11.2206	11.63592	11.91858	10.1442	9.695422	10.0629
AT4G29030	60.57051	1.572927	0.401171	3.920842	8.82E-05	0.000846	5.574094	7.429827	6.259813	4.826463	3.546624	5.029925
AT4G06110	11.70054	1.573822	0.459122	3.427896	0.000608	0.004671	4.334434	4.503365	4.356153	2.526213	0	2.610742
AT4G13495	1550.626	1.574542	0.13528	11.63912	2.61E-31	2.26E-29	11.31342	11.17789	11.07978	9.736644	9.362265	9.593082
AT1G07490	12.77702	1.574731	0.454214	3.466939	0.000526	0.004098	4.604064	4.22085	4.680135	2.797163	0	2.610742
AT5G54070	7667.434	1.575318	0.191821	8.21243	2.17E-16	8.05E-15	13.79189	13.16409	13.48402	11.81044	11.54732	12.16947

AT4G09650	3092.888	1.57559	0.139799	11.27037	1.84E-29	1.49E-27	11.93675	12.19831	12.38502	10.56497	10.5984	10.57872
AT3G18890	540.6569	1.578982	0.164293	9.610755	7.20E-22	3.78E-20	9.455637	9.738249	9.831048	8.101887	7.78168	8.170323
AT5G04425	58.46634	1.580402	0.292166	5.409251	6.33E-08	1.04E-06	6.51049	6.495726	6.323125	4.632436	5.617177	4.421742
AT3G03190	15.89453	1.581935	0.438413	3.60832	0.000308	0.002547	4.57299	4.684	5.097223	3.395221	0	2.610742
AT1G62630	85.81501	1.583361	0.241544	6.555168	5.56E-11	1.30E-09	7.01123	7.146386	7.046626	5.567291	4.792505	5.42247
AT2G04790	36.2368	1.585968	0.343669	4.61481	3.93E-06	4.88E-05	5.95768	5.857326	5.934498	4.142579	0	4.553113
AT5G17305	23.75286	1.586756	0.402305	3.944164	8.01E-05	0.000774	5.679967	4.844514	5.167905	3.549829	3.546624	3.197407
AT3G10060	253.0146	1.58789	0.195779	8.110618	5.04E-16	1.82E-14	8.308857	8.648639	8.758225	7.056432	7.013727	6.785485
AT1G13609	9.185806	1.591322	0.485746	3.276041	0.001053	0.007514	4.002555	4.181303	3.825555	1.757146	2.665087	1.605623
AT3G59400	791.8888	1.594193	0.143344	11.12142	9.87E-29	7.73E-27	10.21775	10.06528	10.37746	8.680921	8.591227	8.526651
AT5G23730	20.08135	1.594501	0.416299	3.830184	0.000128	0.001176	5.004194	5.161454	5.048102	3.816762	2.665087	2.346685
AT5G06835	75.59979	1.594857	0.264447	6.030916	1.63E-09	3.30E-08	6.845594	6.994943	6.881184	5.567291	4.089834	5.117444
AT5G59130	10.60115	1.595088	0.484318	3.29347	0.00099	0.007115	4.002555	4.403791	3.937685	1.757146	3.546624	1.015523
AT3G62500	23.64961	1.599371	0.396263	4.036131	5.43E-05	0.000544	5.094491	5.259657	5.213173	2.526213	4.483533	3.34975
AT2G40610	742.018	1.599794	0.185653	8.617138	6.86E-18	2.84E-16	9.840296	10.09751	10.43293	8.685098	8.255176	8.435932
AT2G07774	83.71192	1.605187	0.246787	6.504329	7.80E-11	1.79E-09	7.056865	6.85743	7.125699	5.331804	5.262981	5.352044
AT5G38410	14932.63	1.606259	0.163555	9.820935	9.15E-23	5.12E-21	14.18301	14.33344	14.80855	12.84122	12.78627	12.8257
AT3G27700	1597.175	1.606281	0.152877	10.50702	8.02E-26	5.31E-24	11.16177	11.1551	11.39637	9.830151	9.538439	9.38398
AT3G03770	68.44567	1.609871	0.273901	5.877566	4.16E-09	8.01E-08	6.590249	6.918835	6.749102	4.885743	4.483533	5.315499
AT3G55090	21.41586	1.611397	0.394196	4.087804	4.35E-05	0.000443	5.158689	4.988944	5.320494	3.395221	2.665087	3.34975
AT1G07180	264.0202	1.612064	0.201971	7.98166	1.44E-15	5.05E-14	8.460877	8.738235	8.750569	6.623396	7.013727	7.249844
AT5G58260	158.4733	1.617921	0.222894	7.258714	3.91E-13	1.10E-11	7.734383	7.786777	8.198567	6.335206	6.342338	6.074811
AT1G65860	66.24793	1.622168	0.296096	5.478511	4.29E-08	7.23E-07	6.327105	6.954462	6.764389	4.700053	4.792505	5.199956
AT2G15030	9.644985	1.622244	0.488381	3.321675	0.000895	0.006554	4.57299	4.181303	3.639105	1.757146	0	2.346685
AT1G29430	155.889	1.62453	0.214584	7.570588	3.72E-14	1.15E-12	7.985118	7.701985	8.012637	6.4378	6.13862	6.007675
AT1G80570	39.00815	1.625741	0.337896	4.811361	1.50E-06	2.00E-05	6.210797	5.61171	6.00054	4.142579	4.089834	4.199107
AT2G21595	21.65119	1.625918	0.426973	3.808015	0.00014	0.001272	5.240069	4.596507	5.668068	3.689461	0	3.197407
AT1G26770	120.2715	1.626457	0.214748	7.573805	3.62E-14	1.13E-12	7.506673	7.623008	7.49451	6.001749	5.617177	5.785202
AT4G08755	37.73845	1.626932	0.332679	4.890395	1.01E-06	1.39E-05	5.73715	6.130813	5.836646	4.041933	4.089834	4.199107
AT5G06845	29.36281	1.628066	0.377856	4.308689	1.64E-05	0.000182	5.493412	5.62651	5.635183	2.797163	3.546624	4.277199
AT2G41250	422.1529	1.628548	0.169435	9.611616	7.14E-22	3.76E-20	9.496577	9.308942	9.134203	7.676061	7.883112	7.490138
AT3G51238	21.9075	1.629267	0.418267	3.895276	9.81E-05	0.00093	4.932558	5.201541	5.439554	3.933736	2.665087	2.346685
AT1G69530	1587.016	1.629514	0.155204	10.4992	8.71E-26	5.75E-24	10.92774	11.37371	11.37131	9.51989	9.528036	9.652893

AT1G44000	272.8201	1.630138	0.181597	8.976688	2.79E-19	1.24E-17	8.666216	8.6809	8.786579	7.304685	6.822676	6.825021
AT1G19050	68.02571	1.63516	0.26339	6.208136	5.36E-10	1.13E-08	6.723563	6.691408	6.725863	4.764642	5.262981	5.074348
AT1G74070	133.2048	1.638707	0.211912	7.732964	1.05E-14	3.40E-13	7.67343	7.630369	7.787973	5.890376	5.617177	6.200381
AT5G54585	72.43553	1.639072	0.274856	5.963386	2.47E-09	4.90E-08	7.03992	6.633975	6.725863	5.243916	5.262981	4.784636
AT5G43750	240.5111	1.639862	0.192934	8.499604	1.90E-17	7.66E-16	8.311213	8.511857	8.737072	6.949358	6.752923	6.758512
AT4G00880	37.9927	1.640322	0.334177	4.908544	9.18E-07	1.27E-05	5.883635	6.151426	5.851038	4.486882	2.665087	4.116545
AT1G58280	15.00611	1.645882	0.454727	3.619498	0.000295	0.002454	4.136111	4.626265	5.144727	2.797163	2.665087	2.346685
AT5G00765	58.2898	1.646772	0.297525	5.534907	3.11E-08	5.33E-07	6.200668	6.655781	6.694283	4.632436	4.483533	4.936752
AT5G06825	1901.683	1.649222	0.138314	11.92377	8.90E-33	8.22E-31	11.29687	11.54098	11.63649	9.728572	9.981227	9.777359
AT5G14060	1075.862	1.650288	0.124714	13.23258	5.69E-40	7.27E-38	10.61432	10.72155	10.71401	9.127163	8.846958	8.982103
AT4G08112	12.61837	1.65269	0.478812	3.451645	0.000557	0.00431	4.178017	4.965854	4.091061	2.797163	2.665087	1.015523
AT4G37800	458.1676	1.653809	0.203577	8.123754	4.52E-16	1.64E-14	9.11763	9.457042	9.751772	7.991526	7.554534	7.585307
AT3G51240	22.27122	1.655203	0.416959	3.9697	7.20E-05	0.000701	4.980709	5.201541	5.477153	3.933736	2.665087	2.346685
AT4G36280	19.12204	1.656127	0.4065	4.074112	4.62E-05	0.000467	5.072441	4.942389	5.022899	3.222033	2.665087	3.027056
AT5G27330	15.39473	1.656922	0.463389	3.575665	0.000349	0.002849	4.296891	5.369304	4.273086	2.192331	2.665087	2.833864
AT3G10570	32.51018	1.658361	0.344755	4.810256	1.51E-06	2.01E-05	5.73715	5.844724	5.584406	3.933736	3.546624	3.935733
AT3G55850	958.8762	1.660631	0.194752	8.52688	1.50E-17	6.10E-16	10.2894	10.29339	10.89115	8.599158	9.035019	8.785697
AT2G30760	10.77237	1.660715	0.477688	3.47657	0.000508	0.003971	4.604064	4.055712	3.990643	1.757146	2.665087	2.023222
AT4G28660	235.5784	1.662156	0.191477	8.680733	3.93E-18	1.65E-16	8.327596	8.44037	8.717569	6.707947	6.752923	6.876105
AT3G48280	9.781437	1.662205	0.48462	3.429912	0.000604	0.004642	3.748168	4.259341	4.273086	1.757146	2.665087	1.605623
AT3G01505	113.983	1.664836	0.259206	6.42283	1.34E-10	2.98E-09	7.211152	7.535541	7.738515	5.831289	4.483533	5.984584
AT1G29440	331.0663	1.669896	0.199864	8.355155	6.53E-17	2.55E-15	9.041639	8.849646	9.116442	7.642081	6.88921	7.030642
AT3G46300	7.945572	1.672402	0.499521	3.348009	0.000814	0.006029	3.906114	3.596007	4.273086	1.131026	0	2.023222
AT2G46192	3145.837	1.67834	0.19129	8.773801	1.73E-18	7.36E-17	12.28824	12.00993	12.40832	10.57288	9.988833	10.79739
AT5G05040	12.71406	1.679745	0.456768	3.677459	0.000236	0.002016	4.72207	4.566123	4.273086	2.526213	0	2.610742
AT1G70260	9.360018	1.684121	0.492439	3.419958	0.000626	0.004785	4.178017	4.437751	3.766043	2.526213	0	1.015523
AT5G59860	13.62745	1.686221	0.447007	3.772251	0.000162	0.001449	4.604064	4.712023	4.545032	2.526213	0	2.833864
AT5G17670	207.1408	1.686382	0.198129	8.511517	1.72E-17	6.96E-16	8.329921	8.091485	8.497382	6.588138	6.752923	6.473473
AT1G19450	179.4561	1.686977	0.22692	7.43423	1.05E-13	3.15E-12	7.772494	8.269393	8.277939	6.129868	6.342338	6.55411
AT5G23940	198.439	1.687005	0.1962	8.598388	8.08E-18	3.33E-16	8.087616	8.327157	8.355697	6.691428	6.520817	6.334274
AT5G44410	44.03545	1.688129	0.322342	5.23708	1.63E-07	2.54E-06	5.896243	6.141156	6.373842	4.142579	4.089834	4.488922
AT1G06667	8.176467	1.693257	0.502294	3.371047	0.000749	0.005584	4.048462	3.470386	4.091061	0	2.665087	1.605623
AT5G13630	7623.829	1.694941	0.118698	14.27947	2.94E-46	4.66E-44	13.39523	13.5599	13.57739	11.89582	11.83791	11.64558

AT3G09775	5.272381	1.700703	0.514851	3.303289	0.000956	0.006912	3.294214	3.869198	3.173324	1.131026	0	0
AT3G19450	122.4938	1.701123	0.219665	7.744174	9.62E-15	3.12E-13	7.741388	7.503831	7.499086	5.890376	5.617177	5.811684
AT3G07385	8.115974	1.70355	0.498248	3.419078	0.000628	0.004793	3.95514	3.654941	4.273086	1.131026	0	2.023222
AT2G35960	26.53139	1.706928	0.370941	4.601611	4.19E-06	5.17E-05	5.390232	5.454712	5.495591	3.222033	3.546624	3.728968
AT1G04180	53.44699	1.708172	0.291698	5.855959	4.74E-09	9.05E-08	6.434732	6.278675	6.422836	4.408199	5.046837	4.488922
AT2G04435	1291.364	1.711545	0.154539	11.07516	1.66E-28	1.27E-26	10.85936	11.05404	10.97854	9.111728	8.928315	9.475851
AT2G04455	1290.473	1.714558	0.155674	11.01376	3.28E-28	2.46E-26	10.85936	11.05404	10.97854	9.111728	8.912407	9.475851
AT2G05070	18459.05	1.718731	0.152196	11.2929	1.42E-29	1.16E-27	14.7502	14.64487	14.98066	13.28574	12.82502	12.96751
AT2G40100	36.34845	1.719319	0.358768	4.792292	1.65E-06	2.17E-05	5.459832	6.034228	6.193595	4.142579	2.665087	4.116545
AT5G07725	1061.477	1.720698	0.208843	8.239203	1.73E-16	6.51E-15	10.43152	10.88817	10.67965	8.621127	9.408366	8.58922
AT2G32645	131.3247	1.725143	0.259312	6.652771	2.88E-11	6.94E-10	7.691626	7.666621	7.83217	6.356317	4.089834	5.83769
AT2G39730	26955.08	1.730293	0.123005	14.06688	6.07E-45	9.28E-43	15.14913	15.3549	15.5059	13.61004	13.62773	13.52661
AT5G36700	600.2174	1.732023	0.160699	10.77805	4.37E-27	3.12E-25	9.66536	9.868457	10.0394	8.233219	7.946977	8.025114
AT5G36790	601.4632	1.732713	0.161007	10.7617	5.22E-27	3.68E-25	9.6672	9.873894	10.04176	8.238914	7.946977	8.025114
AT5G35970	616.3487	1.734213	0.152196	11.39461	4.45E-30	3.69E-28	9.71602	10.06392	9.900152	8.095624	8.095267	8.18052
AT5G09935	1523.821	1.734706	0.129133	13.43348	3.85E-41	5.12E-39	11.10452	11.32123	11.18329	9.435616	9.265393	9.560269
AT1G04897	672.1836	1.734945	0.194396	8.924818	4.46E-19	1.96E-17	9.934988	9.852023	10.29236	8.132803	7.816289	8.554359
AT3G24612	30.24375	1.739109	0.398849	4.360325	1.30E-05	0.000147	5.66531	6.00054	4.944537	3.222033	4.483533	3.34975
AT5G01015	17.83724	1.739696	0.434053	4.008027	6.12E-05	0.000606	4.604064	5.01167	5.278519	3.395221	0	2.610742
AT1G52230	4612.479	1.739849	0.163711	10.62754	2.22E-26	1.52E-24	12.68701	12.69778	13.01022	11.23758	10.67111	11.06841
AT1G44446	1744.434	1.749584	0.132725	13.18201	1.11E-39	1.39E-37	11.44581	11.39898	11.36913	9.822586	9.385499	9.575804
AT1G09855	20.57746	1.752067	0.420812	4.16354	3.13E-05	0.000327	5.179468	5.386795	4.971133	2.526213	0	3.728968
AT5G04305	669.7555	1.753347	0.18449	9.503746	2.02E-21	1.04E-19	9.928098	9.860655	10.28575	8.186833	7.816289	8.473736
AT5G35490	25.927	1.75366	0.401025	4.37294	1.23E-05	0.000139	5.050049	5.712254	5.567075	2.797163	2.665087	3.935733
AT3G45851	9.010789	1.75758	0.501261	3.506321	0.000454	0.003602	3.294214	4.296832	4.545032	1.131026	0	2.023222
AT1G06100	7.789492	1.762652	0.505703	3.485549	0.000491	0.003855	3.506448	4.503365	3.639105	1.131026	0	1.605623
AT1G17050	78.54861	1.764256	0.269626	6.543356	6.02E-11	1.40E-09	6.765398	6.942684	7.17229	4.826463	5.046837	5.315499
AT2G37240	88.92592	1.770111	0.244866	7.228884	4.87E-13	1.36E-11	6.999592	7.13608	7.24493	5.197878	5.450922	5.278004
AT5G44530	126.9137	1.771315	0.257131	6.888757	5.63E-12	1.44E-10	7.809624	7.770213	7.389971	6.224753	5.262981	5.456434
AT2G20750	22.81376	1.771347	0.401644	4.410247	1.03E-05	0.000119	5.179468	5.099155	5.549533	2.797163	2.665087	3.613295
AT3G52720	170.9372	1.771524	0.265559	6.670936	2.54E-11	6.15E-10	7.547209	8.271749	8.358218	6.476879	5.450922	6.220286
AT1G23205	4.459062	1.775863	0.514442	3.45202	0.000556	0.004305	3.294214	3.010545	3.571186	0	0	0
AT3G28270	29.732	1.777962	0.39311	4.522804	6.10E-06	7.35E-05	5.199953	6.099331	5.635183	3.816762	0	3.836051

AT1G70185	944.1722	1.779137	0.215362	8.261155	1.44E-16	5.45E-15	10.32072	10.24326	10.91874	8.353586	9.049638	8.596854
AT3G47420	215.5146	1.780623	0.195121	9.125745	7.12E-20	3.29E-18	8.282688	8.589442	8.31215	6.397634	6.602406	6.688789
AT3G25740	55.2067	1.785753	0.296364	6.025531	1.69E-09	3.40E-08	6.468894	6.306513	6.637299	4.764642	4.483533	4.35128
AT3G16320	9.597599	1.788947	0.487794	3.667422	0.000245	0.002088	3.95514	4.181303	4.184943	1.757146	2.665087	1.015523
AT5G07690	55.71411	1.792158	0.31219	5.740599	9.43E-09	1.73E-07	6.518668	6.558811	6.193595	4.324977	5.450922	4.116545
AT3G20760	100.2616	1.792815	0.23921	7.494736	6.64E-14	2.01E-12	7.250962	7.425602	7.365117	5.492986	4.483533	5.674125
AT3G59710	51.97056	1.79703	0.312959	5.74206	9.35E-09	1.71E-07	6.190467	6.519708	6.507039	4.041933	4.089834	4.837144
AT5G48490	582.0251	1.797445	0.214506	8.379459	5.32E-17	2.09E-15	10.01593	9.595112	9.911354	8.384742	7.33342	7.850924
AT1G10657	16.11658	1.799998	0.44754	4.021981	5.77E-05	0.000575	5.027304	4.844514	4.74322	1.757146	0	3.34975
AT2G48120	88.08675	1.801156	0.249721	7.212687	5.49E-13	1.53E-11	7.017014	7.13608	7.23947	4.997462	5.262981	5.42247
AT4G03060	25.76258	1.80123	0.39305	4.5827	4.59E-06	5.63E-05	5.240069	5.793186	5.167905	3.395221	3.546624	3.197407
AT3G08770	89.58531	1.802839	0.311735	5.783242	7.33E-09	1.36E-07	6.825959	7.404291	7.324439	4.764642	4.089834	5.811684
AT4G24780	278.949	1.805226	0.202399	8.919139	4.70E-19	2.06E-17	8.658835	8.882384	8.85601	7.260576	6.13862	6.863502
AT4G06235	89.26829	1.80657	0.266311	6.78368	1.17E-11	2.93E-10	6.915386	7.360699	7.24493	5.150324	4.483533	5.553778
AT5G50740	59.24849	1.806951	0.345747	5.226216	1.73E-07	2.68E-06	6.016608	7.02876	6.694283	4.942684	2.665087	4.673514
AT1G32520	74.91881	1.809372	0.257243	7.033711	2.01E-12	5.37E-11	6.877737	6.977733	6.922655	5.150324	4.483533	5.029925
AT4G00883	29.9462	1.81103	0.368119	4.919684	8.67E-07	1.21E-05	5.509914	5.857326	5.618455	4.041933	2.665087	3.34975
AT5G17230	1097.692	1.812833	0.121702	14.89565	3.52E-50	6.50E-48	10.75464	10.71594	10.78366	8.998793	8.706416	8.937707
AT5G13170	54.23112	1.815432	0.374721	4.844752	1.27E-06	1.72E-05	5.66531	6.936758	6.603697	4.885743	4.089834	3.836051
AT5G01017	15.48626	1.815886	0.447924	4.054008	5.03E-05	0.000507	4.441535	4.844514	5.097223	3.025182	0	2.346685
AT1G06553	88.46926	1.8183	0.272009	6.6847	2.31E-11	5.63E-10	6.915386	6.983493	7.476057	5.101148	5.262981	5.278004
AT1G23740	396.2139	1.818426	0.158048	11.50553	1.24E-30	1.05E-28	9.198751	9.255199	9.384742	7.553458	7.42597	7.288068
AT5G64940	1695.041	1.81928	0.130342	13.95778	2.82E-44	4.24E-42	11.4291	11.44875	11.23015	9.61273	9.589355	9.38398
AT1G04227	80.52456	1.828191	0.275615	6.633121	3.29E-11	7.89E-10	6.772254	7.28343	7.046626	5.288529	4.089834	5.15929
AT1G08123	24.56951	1.836761	0.385996	4.758498	1.95E-06	2.55E-05	5.390232	5.503631	5.299659	3.025182	2.665087	3.613295
AT2G09695	2754.975	1.837368	0.217098	8.463294	2.60E-17	1.04E-15	12.16145	11.81557	12.2694	10.35945	9.463995	10.44449
AT1G45191	29.19851	1.837804	0.367117	5.00605	5.56E-07	7.98E-06	5.723066	5.487509	5.601531	3.816762	3.546624	3.197407
AT5G03155	8.692138	1.841571	0.503818	3.655234	0.000257	0.002177	3.57078	4.22085	4.395959	2.192331	0	0
AT3G09225	49.62415	1.851824	0.322703	5.738476	9.55E-09	1.75E-07	6.116967	6.487643	6.441977	3.816762	4.089834	4.730144
AT2G46830	570.2813	1.856853	0.225574	8.231679	1.85E-16	6.90E-15	10.19114	9.676477	9.521487	8.192714	7.883112	7.465332
AT1G14345	148.5873	1.861407	0.216339	8.604127	7.69E-18	3.17E-16	7.855539	7.726233	8.087478	6.05437	5.766238	5.937262
AT3G03850	74.74638	1.869766	0.293888	6.362165	1.99E-10	4.35E-09	7.221208	6.760126	6.859993	5.373819	3.546624	4.784636
AT1G63710	6.316757	1.875908	0.513894	3.650381	0.000262	0.002213	3.133118	4.22085	3.639105	0	0	1.015523

AT3G02380	326.8214	1.877665	0.185567	10.1185	4.57E-24	2.73E-22	9.220219	8.8306	8.974127	7.215076	7.182411	6.888598
AT2G09885	43.32205	1.878298	0.365223	5.142884	2.71E-07	4.07E-06	6.270123	5.819185	6.159311	3.025182	5.262981	3.836051
AT1G65450	19.93666	1.8801	0.431358	4.358562	1.31E-05	0.000148	4.804643	5.43803	5.190717	3.549829	0	2.346685
AT1G01790	425.7746	1.883715	0.1731	10.88226	1.40E-27	1.02E-25	9.161374	9.43304	9.559229	7.459032	7.672573	7.343571
AT5G21100	130.6647	1.883997	0.226548	8.316113	9.09E-17	3.49E-15	7.7622	7.803153	7.489919	5.530617	6.13862	5.730732
AT5G42800	8.440076	1.886472	0.510933	3.692209	0.000222	0.001918	3.215913	4.766488	3.882709	1.757146	0	0
AT5G25130	78.81963	1.887472	0.279563	6.751517	1.46E-11	3.63E-10	6.839079	7.1257	7.101827	5.454347	4.089834	4.730144
AT5G66562	11.57479	1.887792	0.473251	3.98899	6.64E-05	0.00065	4.21874	4.739512	4.356153	2.192331	0	2.023222
AT5G27780	40.52216	1.891923	0.349664	5.410681	6.28E-08	1.04E-06	6.159424	5.655661	6.373842	3.933736	3.546624	4.116545
AT4G39364	284.6752	1.893636	0.209537	9.037242	1.61E-19	7.31E-18	8.592596	8.914395	8.88077	6.640707	7.47011	6.600414
AT2G06855	20.93157	1.894386	0.436168	4.343249	1.40E-05	0.000157	5.442744	5.201541	5.072871	1.757146	0	3.728968
AT2G04032	5.992549	1.895169	0.512917	3.694884	0.00022	0.0019	3.802761	3.596007	3.571186	0	0	1.015523
AT2G32650	170.4934	1.895292	0.241224	7.856993	3.93E-15	1.32E-13	8.098567	8.069991	8.231942	6.533581	4.483533	6.138962
AT1G29435	71.64209	1.898101	0.304798	6.227409	4.74E-10	1.00E-08	6.92772	6.589348	7.119768	5.331804	4.089834	4.421742
AT3G09600	156.121	1.902369	0.266275	7.144374	9.04E-13	2.48E-11	8.400208	7.857483	7.574743	6.224753	5.766238	5.730732
AT2G08860	609.0503	1.903795	0.18192	10.465	1.25E-25	8.20E-24	9.854902	9.987164	9.971869	8.051003	7.234523	8.225533
AT2G07739	1209.252	1.90988	0.145756	13.10327	3.15E-39	3.88E-37	10.74466	10.89009	11.08092	9.105507	8.813085	8.910399
AT2G47750	39.3372	1.917528	0.360436	5.320018	1.04E-07	1.66E-06	5.921133	6.269276	5.731672	3.025182	4.792505	3.935733
AT2G18120	8.473533	1.920667	0.49757	3.860093	0.000113	0.001054	4.092952	3.869198	4.229687	1.757146	0	1.015523
AT3G04775	218.1729	1.923484	0.218362	8.808687	1.27E-18	5.44E-17	8.196114	8.746726	8.335368	6.476879	6.679627	6.278406
AT4G39800	1427.339	1.924429	0.17236	11.1652	6.04E-29	4.77E-27	10.88687	11.22484	11.32696	9.430648	8.896321	9.096912
AT5G42280	16.12401	1.924638	0.440317	4.37103	1.24E-05	0.00014	4.604064	4.988944	4.889826	2.192331	2.665087	2.610742
AT4G09845	167.722	1.926591	0.212698	9.057855	1.33E-19	6.07E-18	8.022977	8.231173	8.012637	6.377123	5.617177	5.937262
AT1G09867	243.1815	1.930194	0.178447	10.8166	2.87E-27	2.07E-25	8.561271	8.581869	8.605319	6.496028	7.072174	6.489965
AT5G21430	243.5565	1.937516	0.185396	10.45071	1.45E-25	9.48E-24	8.48403	8.560835	8.76394	6.707947	6.679627	6.489965
AT3G12320	674.8653	1.941101	0.164138	11.82602	2.86E-32	2.60E-30	10.27439	10.00143	9.9335	7.908207	8.066803	8.25478
AT4G38960	33.29702	1.943844	0.360253	5.395776	6.82E-08	1.12E-06	6.050842	5.76671	5.601531	3.395221	3.546624	3.836051
AT5G09995	57.72774	1.945002	0.304998	6.377106	1.80E-10	3.96E-09	6.620957	6.43817	6.733651	4.942684	3.546624	4.199107
AT2G34620	294.1132	1.945543	0.164018	11.86179	1.87E-32	1.71E-30	8.928044	8.840155	8.88077	7.003888	6.822676	6.811962
AT5G01645	20.55837	1.946746	0.424051	4.59083	4.41E-06	5.43E-05	5.050049	4.988944	5.549533	3.395221	0	2.610742
AT1G42970	5205.312	1.948221	0.146294	13.31719	1.84E-40	2.40E-38	12.74544	13.02911	13.24783	11.09415	11.01444	10.98724
AT3G01060	140.5709	1.951636	0.210837	9.256591	2.11E-20	1.02E-18	7.940164	7.763533	7.817587	5.831289	5.450922	5.913005
AT2G09395	88.24552	1.952767	0.271879	7.182497	6.84E-13	1.89E-11	7.426369	6.936758	7.027746	4.942684	5.617177	4.936752

AT3G57770	932.7849	1.953311	0.171582	11.38413	5.02E-30	4.13E-28	10.23523	10.71378	10.64445	8.358826	8.571102	8.67466
AT4G13572	107.8502	1.962196	0.268227	7.31544	2.57E-13	7.38E-12	7.590521	7.39999	7.370122	5.800813	5.450922	4.730144
AT2G05100	19498.87	1.970292	0.162287	12.14077	6.42E-34	6.13E-32	14.85629	14.77502	15.14401	13.17685	12.6466	12.85742
AT2G39250	26.31599	1.971706	0.389427	5.063095	4.13E-07	6.02E-06	5.66531	5.566378	5.299659	3.689461	2.665087	2.833864
AT1G23935	21.57438	1.977314	0.419883	4.709205	2.49E-06	3.18E-05	5.335736	5.297139	5.121171	1.757146	2.665087	3.487532
AT5G23060	2743.627	1.985533	0.146668	13.5376	9.38E-42	1.31E-39	11.96881	12.05113	12.28157	10.28296	9.996398	9.921112
AT4G04195	36.41797	1.986113	0.375006	5.296219	1.18E-07	1.88E-06	5.832072	5.670019	6.193595	2.526213	4.483533	3.935733
AT4G04185	36.41797	1.986113	0.375006	5.296219	1.18E-07	1.88E-06	5.832072	5.670019	6.193595	2.526213	4.483533	3.935733
AT5G45650	76.71769	1.987621	0.285583	6.959869	3.41E-12	8.94E-11	6.651025	6.983493	7.261186	4.885743	4.792505	4.837144
AT5G04415	154.7699	1.988287	0.245197	8.108923	5.11E-16	1.84E-14	7.666086	7.866861	8.291194	5.637956	6.244072	5.888334
AT3G03435	563.0675	1.988305	0.200682	9.907739	3.85E-23	2.21E-21	9.776089	10.0612	9.631873	7.429455	7.63429	8.102229
AT1G55480	457.4712	1.988638	0.168011	11.83633	2.53E-32	2.31E-30	9.328841	9.59323	9.664833	7.525773	7.182411	7.600575
AT1G30350	5.420128	1.992502	0.515065	3.868447	0.00011	0.001024	3.368482	3.534563	3.766043	0	0	0
AT3G61220	63.42837	1.995769	0.30997	6.438581	1.21E-10	2.70E-09	6.270123	6.876128	6.936218	4.561494	4.483533	4.553113
AT4G22570	302.65	2.005061	0.181082	11.07267	1.70E-28	1.30E-26	8.779436	8.982127	9.028955	6.640707	6.952811	7.030642
AT3G55580	5.613678	2.006243	0.515055	3.895202	9.81E-05	0.00093	3.748168	3.180636	3.825555	0	0	0
AT2G01590	90.14876	2.006685	0.266664	7.525151	5.27E-14	1.61E-12	7.284918	6.924835	7.419241	5.243916	4.792505	5.029925
AT1G04263	852.7826	2.01466	0.214093	9.410199	4.95E-21	2.48E-19	10.2007	10.14582	10.83931	8.031448	8.630654	8.334564
AT3G02870	100.8799	2.021933	0.25844	7.823621	5.13E-15	1.70E-13	7.154554	7.507833	7.530721	5.492986	4.089834	5.315499
AT3G02832	182.6103	2.027833	0.252553	8.029322	9.80E-16	3.46E-14	8.005627	8.402178	8.135299	5.567291	6.88921	5.888334
AT3G55920	58.57483	2.033537	0.293155	6.936728	4.01E-12	1.05E-10	6.493996	6.766821	6.595172	4.408199	4.089834	4.553113
AT3G03445	554.6826	2.038122	0.200016	10.18979	2.20E-24	1.35E-22	9.76152	10.05369	9.615107	7.368418	7.594963	8.025114
AT3G03585	143.1741	2.046538	0.220849	9.266665	1.92E-20	9.33E-19	7.83604	7.869974	7.839406	5.831289	6.244072	5.352044
AT2G04965	7927.483	2.049177	0.144342	14.19668	9.61E-46	1.50E-43	13.53305	13.50308	13.88066	11.6971	11.39823	11.55296
AT3G08565	818.5248	2.063836	0.213679	9.658596	4.52E-22	2.41E-20	10.14236	10.10281	10.78974	7.971143	8.550692	8.185591
AT1G05247	256.583	2.067616	0.474634	4.356237	1.32E-05	0.000149	8.313565	9.316947	8.681785	4.942684	6.88921	4.887807
AT2G31380	167.2346	2.080017	0.212437	9.791207	1.23E-22	6.81E-21	8.226385	8.072696	8.025383	5.70532	5.766238	6.200381
AT3G58070	8.47271	2.082003	0.502415	4.143988	3.41E-05	0.000354	3.748168	4.098802	4.395959	1.131026	0	1.015523
AT1G05853	806.7519	2.087113	0.211957	9.846857	7.07E-23	3.98E-21	10.12707	10.08818	10.77047	7.964284	8.508985	8.123525
AT2G08375	28.13785	2.088365	0.403728	5.172708	2.31E-07	3.50E-06	5.620423	6.00054	5.299659	3.816762	0	2.833864
AT4G03925	412.9095	2.091898	0.177614	11.77776	5.08E-32	4.55E-30	9.231457	9.376161	9.584211	7.094621	7.128345	7.440091
AT2G15020	17.25156	2.097977	0.445044	4.714092	2.43E-06	3.12E-05	4.541232	5.099155	5.167905	2.192331	2.665087	2.346685
AT5G00760	14.43586	2.104036	0.457914	4.594829	4.33E-06	5.33E-05	4.882744	4.566123	4.680135	1.757146	2.665087	2.023222

AT1G54820	159.0805	2.10584	0.208113	10.11873	4.56E-24	2.73E-22	8.028714	8.099463	8.050543	6.079977	5.046837	5.888334
AT4G06115	18.62541	2.109903	0.430273	4.903642	9.41E-07	1.30E-05	5.279101	5.161454	4.861673	2.797163	0	2.610742
AT1G07897	537.7841	2.110717	0.449423	4.696499	2.65E-06	3.37E-05	9.370099	10.07475	10.08882	6.224753	7.977879	6.259292
AT3G48420	351.2274	2.116338	0.205416	10.30271	6.85E-25	4.28E-23	8.83779	9.138728	9.478103	6.907064	7.013727	7.019324
AT1G08347	11.16595	2.121123	0.487473	4.351261	1.35E-05	0.000152	4.541232	4.296832	4.273086	1.757146	2.665087	0
AT1G74456	86.44568	2.121163	0.265871	7.978159	1.49E-15	5.19E-14	7.175393	7.156618	7.183706	4.408199	5.046837	5.239508
AT1G19150	318.097	2.123699	0.235302	9.025403	1.79E-19	8.08E-18	8.695365	9.02045	9.345892	7.179983	6.602406	6.45679
AT2G21320	43.29171	2.125647	0.337828	6.292092	3.13E-10	6.76E-09	6.073222	6.240703	6.159311	3.222033	4.792505	3.836051
AT1G06243	216.9269	2.130922	0.283214	7.524056	5.31E-14	1.62E-12	8.241286	8.891602	8.187268	5.530617	6.952811	6.096513
AT1G53541	527.3302	2.13626	0.460201	4.642012	3.45E-06	4.32E-05	9.349617	10.05984	10.07968	6.079977	7.883112	6.007675
AT4G03995	1065.627	2.136451	0.136332	15.67099	2.39E-55	5.20E-53	10.73679	10.90154	10.67258	8.714008	8.444073	8.569957
AT1G09125	38.48004	2.137503	0.346593	6.167176	6.95E-10	1.45E-08	5.883635	6.099331	6.026132	3.395221	4.483533	3.487532
AT3G24615	177.4085	2.141956	0.238325	8.987547	2.53E-19	1.13E-17	7.868394	8.483686	8.173019	6.079977	6.13862	5.702706
AT5G33370	51.96117	2.151359	0.369791	5.817772	5.96E-09	1.12E-07	5.832072	6.799839	6.771973	4.142579	0	4.421742
AT1G15980	487.4963	2.151782	0.205446	10.47371	1.14E-25	7.49E-24	9.2389	9.777731	9.875724	7.607282	7.33342	7.259495
AT5G56850	191.2666	2.156101	0.220659	9.771173	1.50E-22	8.25E-21	8.185881	8.56276	8.164402	6.292034	5.90133	5.888334
AT4G34610	55.32157	2.162692	0.309814	6.980614	2.94E-12	7.75E-11	6.737643	6.412782	6.560562	4.561494	3.546624	4.028969
AT2G04039	187.1623	2.164816	0.207231	10.44637	1.52E-25	9.88E-24	8.208804	8.143859	8.478967	6.028299	5.90133	6.096513
AT1G73870	311.6985	2.16579	0.189996	11.39913	4.22E-30	3.51E-28	8.827953	9.211697	8.962575	6.588138	6.822676	6.913264
AT1G15990	47.0369	2.171003	0.33943	6.396028	1.59E-10	3.53E-09	6.005013	6.404219	6.628972	4.142579	2.665087	4.028969
AT2G07754	44.52511	2.171487	0.32751	6.6303	3.35E-11	8.03E-10	6.36388	6.151426	6.281227	3.689461	3.546624	4.116545
AT5G39860	153.3034	2.185439	0.22728	9.615605	6.87E-22	3.63E-20	7.999797	7.903778	8.081388	6.028299	5.766238	5.315499
AT1G06453	3433.681	2.185631	0.154459	14.15025	1.86E-45	2.90E-43	12.36748	12.50026	12.52132	10.01906	10.57332	10.0902
AT2G07807	10.46969	2.196148	0.489409	4.48735	7.21E-06	8.56E-05	4.57299	4.403791	4.138765	1.131026	0	1.605623
AT3G01440	40.83455	2.202575	0.353276	6.234715	4.53E-10	9.60E-09	5.95768	6.201705	6.343626	3.222033	2.665087	4.199107
AT5G24150	22.54703	2.204167	0.441761	4.989507	6.05E-07	8.63E-06	4.604064	5.780009	5.458476	2.192331	2.665087	2.833864
AT1G09087	37.31514	2.204358	0.370148	5.955345	2.60E-09	5.12E-08	5.694477	5.894484	6.343626	3.025182	4.089834	3.613295
AT5G40400	86.83712	2.208973	0.29335	7.530155	5.07E-14	1.55E-12	7.165011	6.825719	7.512729	4.997462	5.046837	4.488922
AT4G39366	421.1002	2.211342	0.181079	12.21202	2.68E-34	2.61E-32	9.339266	9.533629	9.444094	6.787815	7.47011	7.316087
AT1G04217	60.69357	2.218234	0.301492	7.357516	1.87E-13	5.46E-12	6.716471	6.463119	6.859993	4.236658	4.483533	4.277199
AT5G02865	31.50845	2.234456	0.380244	5.876371	4.19E-09	8.06E-08	5.73715	5.739739	5.94795	3.816762	2.665087	2.610742
AT4G09350	37.51666	2.237563	0.383093	5.840786	5.20E-09	9.84E-08	5.509914	6.269276	6.238077	2.797163	3.546624	3.836051
AT3G55630	64.84421	2.266106	0.294434	7.696491	1.40E-14	4.51E-13	6.812719	6.611834	6.85286	4.236658	5.046837	4.116545

AT3G27865	172.1372	2.267325	0.220598	10.2781	8.85E-25	5.50E-23	8.079347	8.28813	8.078333	5.454347	6.434336	5.785202
AT1G61275	2323.315	2.27117	0.221932	10.23362	1.40E-24	8.68E-23	12.10164	11.87893	11.79841	9.102387	9.22732	10.0684
AT4G13577	113.2123	2.27228	0.251771	9.02519	1.79E-19	8.08E-18	7.698841	7.429827	7.600526	5.492986	5.262981	4.784636
AT5G04085	528.0286	2.275916	0.193081	11.78739	4.53E-32	4.09E-30	9.671791	9.637738	10.05813	7.589563	6.822676	7.569875
AT1G20015	26.43835	2.290506	0.396887	5.771186	7.87E-09	1.45E-07	5.335736	5.726062	5.635183	3.025182	2.665087	2.833864
AT4G15430	53.53048	2.295679	0.323597	7.094261	1.30E-12	3.53E-11	6.68775	6.527613	6.36384	4.408199	4.089834	3.487532
AT1G22590	31.6584	2.300528	0.384606	5.981525	2.21E-09	4.41E-08	5.858084	5.550946	6.112292	3.549829	0	3.197407
AT5G08860	39.54008	2.307605	0.416326	5.542786	2.98E-08	5.11E-07	6.116967	6.773485	5.190717	3.222033	2.665087	3.613295
AT3G15354	117.766	2.315906	0.237378	9.756198	1.74E-22	9.53E-21	7.609787	7.743307	7.600526	4.997462	5.046837	5.456434
AT4G06225	73.95295	2.323619	0.296344	7.840947	4.47E-15	1.49E-13	6.702182	7.216535	7.089742	4.486882	3.546624	4.730144
AT2G08900	32.91193	2.328739	0.406907	5.723024	1.05E-08	1.90E-07	5.66531	6.211554	5.477153	2.192331	4.483533	2.833864
AT5G01775	309.125	2.328739	0.246285	9.455469	3.22E-21	1.64E-19	8.914164	9.293955	8.779072	6.17809	7.33342	6.278406
AT3G59320	38.02204	2.333041	0.359638	6.487189	8.75E-11	1.99E-09	6.084284	6.011857	5.94795	3.395221	4.483533	2.833864
AT2G05565	15.97037	2.335037	0.454707	5.135256	2.82E-07	4.22E-06	5.050049	4.739512	5.022899	1.757146	0	2.346685
AT2G32180	107.9837	2.336467	0.247688	9.433115	3.98E-21	2.01E-19	7.543206	7.412853	7.663036	5.331804	4.483533	4.984091
AT3G55860	111.2922	2.33685	0.270217	8.648042	5.24E-18	2.18E-16	7.400052	7.324852	7.909839	4.997462	5.262981	5.074348
AT4G39510	99.96956	2.350747	0.256925	9.149559	5.72E-20	2.67E-18	7.231194	7.56657	7.485313	4.942684	4.483533	5.074348
AT2G32640	57.3878	2.352661	0.312021	7.540078	4.70E-14	1.45E-12	6.426063	6.780119	6.645579	4.142579	4.483533	3.836051
AT4G06010	164.2424	2.355633	0.251027	9.383987	6.35E-21	3.16E-19	7.772494	8.436176	8.084436	5.737858	5.90133	5.387687
AT1G15550	27.70087	2.363812	0.418535	5.647827	1.62E-08	2.87E-07	5.027304	5.819185	5.974485	2.526213	2.665087	3.027056
AT2G32179	111.5083	2.369063	0.245049	9.667724	4.13E-22	2.22E-20	7.602111	7.463187	7.707214	5.331804	4.483533	5.029925
AT3G47348	309.1211	2.378436	0.175457	13.55564	7.34E-42	1.04E-39	8.989641	9.155407	8.944234	6.496028	6.602406	6.674431
AT1G04640	109.8063	2.3816	0.26003	9.15896	5.24E-20	2.46E-18	7.535168	7.306589	7.757739	4.764642	5.450922	5.074348
AT5G18660	188.6003	2.383932	0.22847	10.43434	1.73E-25	1.11E-23	8.062666	8.436176	8.453256	6.079977	5.90133	5.522054
AT5G08030	7.814644	2.384827	0.511278	4.664448	3.09E-06	3.91E-05	3.855363	4.369011	3.882709	0	0	0
AT3G56290	74.53028	2.397111	0.279997	8.561205	1.12E-17	4.57E-16	7.068052	6.876128	7.083661	4.324977	4.483533	4.553113
AT1G75163	49.19721	2.397409	0.356426	6.726238	1.74E-11	4.26E-10	6.190467	6.819293	6.124191	3.689461	4.483533	3.34975
AT3G46370	19.06493	2.403318	0.457802	5.249687	1.52E-07	2.38E-06	4.57299	5.519576	5.213173	1.131026	2.665087	2.346685
AT3G21805	770.4306	2.404966	0.475595	5.056751	4.26E-07	6.21E-06	9.976396	10.86418	10.43113	5.70532	7.946977	6.278406
AT3G27690	6927.129	2.407697	0.152294	15.80957	2.67E-56	6.08E-54	13.40788	13.37118	13.74509	11.26365	10.96131	10.90815
AT1G07487	12.40907	2.414931	0.480557	5.025278	5.03E-07	7.25E-06	4.541232	4.684	4.647527	1.757146	0	1.015523
AT3G44990	21.75369	2.417345	0.450865	5.361567	8.25E-08	1.34E-06	4.604064	5.566378	5.731672	2.192331	0	2.610742
AT3G48390	85.50496	2.417528	0.329049	7.34701	2.03E-13	5.90E-12	7.617422	7.03432	6.962967	5.150324	4.089834	3.836051

AT5G09505	94.10781	2.419213	0.350666	6.898908	5.24E-12	1.34E-10	7.289704	7.942733	6.497922	4.408199	5.046837	4.553113
AT4G15258	75.30757	2.420686	0.295154	8.20144	2.38E-16	8.78E-15	6.758509	7.231133	7.107833	4.236658	4.089834	4.673514
AT1G31835	6000.001	2.426237	0.145851	16.63508	3.88E-62	1.06E-59	13.23464	13.32931	13.36418	10.63575	11.1393	10.71736
AT4G39361	264.3939	2.428293	0.211187	11.49831	1.35E-30	1.13E-28	8.519097	9.044068	8.839865	6.15418	6.434336	6.352426
AT4G37925	235.442	2.449051	0.206153	11.8798	1.51E-32	1.38E-30	8.655131	8.661271	8.709693	6.496028	5.262981	5.984584
AT3G05335	86.90184	2.449369	0.284938	8.59616	8.24E-18	3.38E-16	7.13341	7.324852	7.113813	4.142579	5.617177	4.421742
AT1G08937	129.8594	2.453711	0.250659	9.789055	1.25E-22	6.95E-21	7.758752	7.608172	8.047422	4.997462	5.046837	5.456434
AT5G08515	94.12318	2.483131	0.313485	7.921045	2.36E-15	8.07E-14	7.280116	7.763533	6.85286	4.561494	5.262981	4.35128
AT1G26761	56.87587	2.489208	0.324325	7.675032	1.65E-14	5.29E-13	6.534884	6.918835	6.441977	3.933736	4.089834	3.836051
AT1G08353	29.3666	2.500842	0.402664	6.210735	5.27E-10	1.11E-08	5.751099	5.404077	6.03876	2.526213	2.665087	3.027056
AT5G10250	27.55039	2.510783	0.402961	6.230832	4.64E-10	9.83E-09	5.896243	5.471204	5.584406	2.797163	2.665087	2.610742
AT2G07759	41.4221	2.527283	0.348869	7.244216	4.35E-13	1.22E-11	6.270123	6.109902	6.248986	3.222033	3.546624	3.487532
AT3G45090	50.23225	2.533036	0.325313	7.786453	6.89E-15	2.26E-13	6.460429	6.581774	6.470221	3.933736	2.665087	3.728968
AT3G03175	19.84779	2.534677	0.445211	5.693205	1.25E-08	2.25E-07	4.932558	5.315522	5.361282	1.131026	2.665087	2.346685
AT5G01785	273.4038	2.534856	0.238786	10.61562	2.52E-26	1.72E-24	8.784531	9.169371	8.590385	6.05437	6.88921	5.888334
AT2G35820	229.7069	2.535911	0.201368	12.59341	2.30E-36	2.50E-34	8.762321	8.479616	8.596804	5.861135	6.434336	5.937262
AT1G08117	27.69218	2.536125	0.39966	6.345703	2.21E-10	4.83E-09	5.509914	5.698312	5.792589	2.526213	2.665087	2.833864
AT1G73600	125.0519	2.539954	0.266819	9.51938	1.74E-21	8.97E-20	7.447939	8.059123	7.765357	5.288529	4.792505	4.936752
AT5G02055	86.77278	2.555812	0.361256	7.074796	1.50E-12	4.05E-11	7.241112	7.773541	6.432438	4.041933	5.262981	3.935733
AT1G15580	34.38586	2.557773	0.392266	6.5205	7.01E-11	1.61E-09	5.509914	6.088683	6.248986	3.025182	2.665087	3.027056
AT5G09515	91.11307	2.565159	0.356332	7.198786	6.08E-13	1.69E-11	7.260746	7.927874	6.470221	4.324977	4.792505	4.277199
AT1G12013	1167.339	2.566641	0.189722	13.52845	1.06E-41	1.47E-39	11.02674	10.66802	11.19814	8.114333	8.12318	8.612001
AT4G08991	10.7801	2.571151	0.498593	5.15681	2.51E-07	3.79E-06	4.508759	4.684	4.184943	0	0	1.015523
AT5G07105	742.3641	2.581663	0.184726	13.97564	2.20E-44	3.32E-42	10.34104	10.49434	10.08197	7.468757	8.12318	7.498313
AT5G04465	131.5979	2.604592	0.250224	10.40905	2.25E-25	1.44E-23	7.796233	7.666621	8.099583	5.197878	4.483533	5.199956
AT5G04485	131.5979	2.604592	0.250224	10.40905	2.25E-25	1.44E-23	7.796233	7.666621	8.099583	5.197878	4.483533	5.199956
AT5G02645	183.0614	2.61569	0.210389	12.43266	1.74E-35	1.83E-33	8.251135	8.427751	8.240166	5.530617	5.90133	5.553778
AT5G13225	84.20015	2.621689	0.376737	6.958941	3.43E-12	8.99E-11	7.206097	7.756823	6.36384	3.933736	5.262981	3.487532
AT1G09787	192.2225	2.624957	0.298008	8.80836	1.27E-18	5.45E-17	8.106727	8.574255	8.462658	4.324977	6.13862	5.888334
AT1G32080	393.6622	2.625581	0.216795	12.11087	9.25E-34	8.80E-32	9.095933	9.436194	9.705991	6.640707	6.13862	6.937516
AT1G60590	52.98193	2.656146	0.330153	8.045203	8.61E-16	3.06E-14	6.434732	6.766821	6.569293	3.689461	2.665087	3.836051
AT4G06130	48.82304	2.661634	0.348038	7.647548	2.05E-14	6.52E-13	6.68775	6.511758	6.281227	3.395221	0	3.935733
AT4G12917	28.75486	2.666472	0.403785	6.603689	4.01E-11	9.54E-10	5.751099	5.519576	5.920919	2.526213	2.665087	2.610742

AT1G16635	268.3331	2.667804	0.306941	8.691597	3.57E-18	1.51E-16	8.384633	9.344063	8.729302	5.492986	6.88921	5.456434
AT3G09855	171.3376	2.694283	0.213464	12.62175	1.60E-36	1.76E-34	8.165194	8.313503	8.220903	5.454347	5.450922	5.42247
AT4G06250	78.88235	2.698631	0.291249	9.26573	1.94E-20	9.39E-19	6.987859	7.274061	7.18938	4.041933	3.546624	4.553113
AT3G15310	10.51302	2.708417	0.505232	5.360739	8.29E-08	1.35E-06	4.048462	4.470931	4.773757	0	0	0
AT4G06310	78.54378	2.724208	0.293689	9.275834	1.76E-20	8.61E-19	6.958103	7.28343	7.195033	4.041933	3.546624	4.488922
AT5G13930	118.3482	2.746381	0.268829	10.21609	1.68E-24	1.03E-22	7.404472	7.806407	7.84301	4.700053	5.450922	4.553113
AT5G03195	105.741	2.746559	0.287075	9.567396	1.10E-21	5.72E-20	7.494287	7.240783	7.889068	4.700053	4.483533	4.553113
AT4G06240	68.34893	2.754114	0.323726	8.507554	1.78E-17	7.16E-16	6.620957	7.156618	7.046626	3.549829	3.546624	4.277199
AT3G09865	169.1305	2.774068	0.217325	12.7646	2.58E-37	2.94E-35	8.149482	8.302025	8.215351	5.373819	5.450922	5.278004
AT3G14450	38.12738	2.778217	0.37869	7.336394	2.19E-13	6.35E-12	6.005013	6.151426	6.333412	3.549829	0	2.610742
AT2G08725	69.04875	2.796152	0.314816	8.88185	6.58E-19	2.87E-17	6.680479	7.067237	7.101827	3.689461	4.089834	4.028969
AT5G02665	63.359	2.801092	0.33565	8.345282	7.10E-17	2.74E-15	6.723563	7.201787	6.560562	3.689461	3.546624	3.836051
AT3G59330	28.20703	2.824278	0.413561	6.829172	8.54E-12	2.15E-10	5.896243	5.535346	5.731672	2.526213	2.665087	2.023222
AT4G13575	844.885	2.841561	0.198171	14.3389	1.25E-46	2.06E-44	10.75248	10.557	10.32945	7.94351	6.952811	7.546413
AT3G50825	141.4412	2.901704	0.246214	11.78527	4.65E-32	4.18E-30	7.961317	8.102113	7.990057	5.101148	2.665087	5.15929
AT2G09705	292.2482	2.919391	0.207337	14.08044	5.01E-45	7.71E-43	8.834518	9.010612	9.170518	5.769679	6.602406	5.888334
AT3G01155	92.5799	2.949553	0.316892	9.307752	1.31E-20	6.41E-19	7.062469	7.543361	7.452655	3.222033	5.262981	4.199107
AT4G16250	85.33817	2.962201	0.287475	10.3042	6.74E-25	4.23E-23	7.180556	7.342887	7.344921	4.324977	3.546624	4.028969
AT5G02655	69.46363	2.962621	0.320366	9.247614	2.30E-20	1.10E-18	7.180556	7.045376	6.733651	3.933736	3.546624	3.613295
AT2G35747	317.2163	3.02151	0.261648	11.54799	7.56E-31	6.47E-29	8.998439	9.623976	8.697799	5.890376	6.342338	5.730732
AT5G40395	67.32717	3.035402	0.343575	8.834764	1.00E-18	4.34E-17	6.921566	6.558811	7.28258	3.395221	4.089834	3.487532
AT2G35744	384.2327	3.091728	0.237957	12.99278	1.34E-38	1.61E-36	8.991111	9.817775	9.415329	6.292034	6.13862	6.096513
AT1G08875	23.68573	3.123194	0.443849	7.036615	1.97E-12	5.27E-11	5.493412	5.503631	5.651719	1.757146	0	1.605623
AT3G03595	106.5874	3.123448	0.273626	11.41502	3.52E-30	2.93E-28	7.582742	7.596945	7.613246	4.486882	4.792505	3.836051
AT1G05917	518.5292	3.176379	0.411036	7.72774	1.09E-14	3.54E-13	9.79809	10.2306	9.694066	5.454347	6.88921	5.117444
AT1G01060	394.5568	3.184763	0.250678	12.70461	5.57E-37	6.25E-35	9.962225	9.16304	9.208737	6.224753	6.244072	5.913005
AT5G51174	228.8545	3.18536	0.26026	12.23916	1.92E-34	1.90E-32	8.246219	9.016242	8.756314	5.373819	5.617177	5.117444
AT1G04517	23.21914	3.237233	0.451976	7.162405	7.93E-13	2.19E-11	5.459832	5.503631	5.635183	1.131026	0	1.605623
AT1G07593	24.9173	3.240698	0.458959	7.060975	1.65E-12	4.46E-11	5.372295	5.421153	6.026132	0	0	2.023222
AT3G02445	110.3553	3.266266	0.341667	9.5598	1.18E-21	6.15E-20	7.447939	8.226323	7.149183	4.041933	4.483533	3.728968
AT4G07635	126.0993	3.275759	0.263044	12.45328	1.34E-35	1.42E-33	7.849069	7.974893	7.757739	4.408199	4.483533	4.35128
AT1G13650	25.75422	3.334866	0.450634	7.400392	1.36E-13	4.02E-12	5.650502	5.918735	5.439554	1.757146	0	1.015523
AT1G04527	24.59338	3.3889	0.463	7.319434	2.49E-13	7.18E-12	5.896243	5.201541	5.70022	1.131026	0	1.015523

AT4G06120	62.5778	3.407727	0.34778	9.798512	1.14E-22	6.35E-21	7.045591	6.918835	6.710159	3.025182	2.665087	3.197407
AT2G32810	258.6063	3.423077	0.216455	15.81424	2.48E-56	5.69E-54	8.769191	9.06459	8.852438	5.288529	5.450922	5.352044
AT4G07625	36.64404	3.518317	0.430751	8.167872	3.14E-16	1.15E-14	5.818889	6.324777	6.193595	1.757146	3.546624	0
AT2G35387	83.81693	3.595747	0.324663	11.07533	1.65E-28	1.27E-26	7.175393	7.446604	7.271923	3.025182	4.089834	3.34975
AT1G09937	1264.193	3.608617	0.231181	15.60951	6.27E-55	1.33E-52	11.02423	10.9789	11.52787	6.935398	8.037767	7.249844
AT1G09943	1264.193	3.608617	0.231181	15.60951	6.27E-55	1.33E-52	11.02423	10.9789	11.52787	6.935398	8.037767	7.249844
AT1G05913	524.3437	3.712163	0.264492	14.03507	9.51E-45	1.45E-42	9.808133	10.23483	9.703019	5.530617	6.952811	5.553778
AT5G46315	253.9667	3.741873	0.218332	17.13846	7.67E-66	2.37E-63	8.918805	8.843326	8.945911	4.997462	4.792505	5.074348
AT5G09115	263.1151	3.783059	0.217153	17.42118	5.70E-68	1.93E-65	8.964419	8.909865	9.005023	5.101148	4.483533	5.074348
AT3G13857	262.7885	3.803196	0.21808	17.43944	4.14E-68	1.42E-65	8.961423	8.912886	9.003413	4.997462	4.483533	5.117444
AT2G08910	91.12694	3.805671	0.330687	11.50839	1.20E-30	1.01E-28	7.452214	7.648609	7.23947	3.222033	2.665087	3.34975
AT5G66564	122.9951	3.805936	0.290495	13.10155	3.23E-39	3.95E-37	7.819587	7.992137	7.83217	3.689461	2.665087	4.028969
AT3G14735	262.3432	3.821211	0.218705	17.47195	2.34E-68	8.25E-66	8.961423	8.911377	9.000188	4.997462	4.483533	5.074348
AT3G13855	262.2289	3.841986	0.219651	17.49128	1.67E-68	6.04E-66	8.961423	8.912886	9.000188	4.942684	4.483533	5.074348
AT4G04615	262.5177	3.843355	0.219782	17.4871	1.80E-68	6.41E-66	8.962922	8.911377	9.005023	4.942684	4.483533	5.074348
AT4G07875	262.4994	3.843371	0.219716	17.49244	1.64E-68	6.00E-66	8.965915	8.911377	9.001802	4.942684	4.483533	5.074348
AT5G00750	332.7655	4.10723	0.241032	17.04021	4.13E-65	1.25E-62	9.273137	9.071367	9.544915	4.632436	5.262981	5.15929
AT2G07605	338.4515	4.267051	0.235085	18.15113	1.26E-73	5.39E-71	9.101388	9.469409	9.427384	4.826463	5.046837	4.837144
AT4G09135	186.4274	4.606734	0.297715	15.47364	5.23E-54	1.09E-51	8.377907	8.64501	8.448531	3.549829	4.483533	3.027056
AT5G34853	142.9717	5.682773	0.395418	14.37154	7.81E-47	1.31E-44	8.15736	8.214128	8.087478	0	2.665087	1.015523
AT5G34850	874.9935	7.971466	0.356232	22.37719	6.56E-111	8.43E-108	10.75594	10.76943	10.7944	1.131026	0	1.015523
