# Supplementary material for: Induction of epigenetic variation in Arabidopsis by over-expression of DNA METHYLTRANSFERASE1 (MET1)
Source: PLoS One. 2018 Feb 21;13(2):e0192170. doi: 10.1371/journal.pone.0192170 (PMC5821449; doi:10.1371/journal.pone.0192170)
Supplement: S3 Table — (PDF) [file pone.0192170.s008.pdf]

S3 Table: List of genes with altered transcript levels in line A2+

	baseMean	log2FoldCh	lfcSE	stat	pvalue	padj	control 1	control 2	control 3	A2+ 4	A2+ 5	A2+ 6
AT4G06566	356.2203	-4.057801	0.168732	-24.04874	8.61E-128	1.14E-123	1.523549	2.703491	1.024858	9.327026	9.779157	9.25497
AT5G49160	4864.811	-3.941186	0.149715	-26.32459	1.00E-152	2.67E-148	7.593837	8.478316	7.997466	13.25077	13.22985	13.14137
AT1G59930	481.386	-3.66341	0.167799	-21.83216	1.15E-105	1.02E-101	4.370668	2.893891	4.29467	9.606873	10.22347	9.766956
AT2G06180	374.445	-3.487712	0.177469	-19.6525	5.50E-86	2.93E-82	0	0	1.024858	8.985359	10.10463	9.323682
AT3G06465	397.158	-3.40005	0.177586	-19.14592	1.05E-81	3.98E-78	1.523549	1.909536	1.024858	9.433616	10.19863	9.004551
AT1G42050	193.1229	-3.370772	0.175987	-19.15357	9.04E-82	3.98E-78	0.954186	0	1.024858	8.524804	8.961088	8.195116
AT1G59920	264.3615	-3.358866	0.168179	-19.97192	9.67E-89	6.43E-85	3.72108	3.062067	3.853184	8.747784	9.331975	8.912373
AT4G04170	528.8392	-3.169234	0.167848	-18.88161	1.62E-79	5.37E-76	5.400859	5.495379	3.952951	9.973666	10.22217	9.775158
AT4G04430	198.9275	-3.1542	0.17787	-17.73319	2.32E-70	6.87E-67	1.523549	0.940325	0	8.14022	9.105943	8.494364
AT4G06506	167.8216	-3.12812	0.177483	-17.62489	1.59E-69	4.22E-66	1.523549	0	1.024858	8.61817	8.590884	7.836044
AT2G11780	157.7282	-3.031526	0.17738	-17.09059	1.74E-65	4.22E-62	0.954186	1.504835	2.037128	8.251261	8.68049	7.836044
AT2G13160	188.7258	-3.015596	0.17912	-16.83565	1.34E-63	2.96E-60	0	0	0	7.888656	9.021979	8.564184
AT2G34130	147.3262	-2.970182	0.178734	-16.61788	5.17E-62	1.06E-58	0	0.940325	0	7.716367	8.642213	8.109488
AT2G11778	150.8647	-2.911102	0.179285	-16.23729	2.75E-59	4.87E-56	0	0	0	8.303733	8.638329	7.597891
AT3G30393	91.68757	-2.893088	0.177483	-16.30064	9.77E-60	1.86E-56	0.954186	0.940325	0	7.599114	7.610569	7.339299
AT4G02314	137.5216	-2.835124	0.179381	-15.80506	2.87E-56	4.77E-53	0.954186	0	0	7.560428	8.566564	8.018455
AT3G32230	120.169	-2.809611	0.179265	-15.67299	2.31E-55	3.62E-52	0.954186	0	0	7.437728	8.331809	7.829758
AT1G42410	130.6466	-2.70727	0.179854	-15.05263	3.32E-51	4.90E-48	0	0	1.024858	7.488067	8.55012	7.854739
AT1G36260	102.355	-2.68953	0.179732	-14.9641	1.26E-50	1.76E-47	0	0	0	7.11361	7.960873	7.842303
AT5G36655	125.5457	-2.524351	0.18018	-14.0102	1.35E-44	1.80E-41	1.523549	0.940325	0	7.226639	8.464963	7.96783
AT4G07942	77.19431	-2.503879	0.180068	-13.90518	5.89E-44	7.46E-41	0	0	0	7.429165	7.512127	6.797952
AT4G25530	84.59673	-2.502674	0.180141	-13.89285	7.00E-44	8.46E-41	0	0	0	7.102881	7.864414	7.130131
AT4G06485	79.8609	-2.486526	0.179533	-13.85	1.27E-43	1.47E-40	0.954186	1.504835	1.618012	7.446241	7.529007	6.88541
AT3G30620	80.40007	-2.468684	0.179299	-13.7685	3.94E-43	4.37E-40	1.930722	1.504835	1.618012	7.552565	7.415578	6.909454
AT3G30780	86.92852	-2.438085	0.180249	-13.52621	1.10E-41	1.12E-38	0	0	0	6.831152	7.851051	7.490411
AT4G06720	70.56623	-2.437183	0.180084	-13.53361	9.90E-42	1.05E-38	0	0.940325	0	7.294214	7.350594	6.718523
AT1G33130	152.6022	-2.398624	0.180177	-13.3126	1.96E-40	1.86E-37	0	0.940325	0	7.275228	8.847924	8.238471
AT3G42716	104.6838	-2.395898	0.180267	-13.29084	2.62E-40	2.40E-37	0.954186	0.940325	0	7.844257	8.125306	6.909454
AT3G32226	107.5595	-2.346893	0.180182	-13.02515	8.80E-39	7.81E-36	0	0	0	6.831152	8.051286	8.072696
AT1G67105	103.7252	-2.339764	0.175022	-13.36837	9.25E-41	9.12E-38	4.2336	3.062067	3.043225	7.771603	7.626342	7.466299
AT1G52850	96.30345	-2.325936	0.180225	-12.90575	4.18E-38	3.58E-35	0.954186	0	0	6.869634	8.119745	7.529722
AT2G06720	55.14439	-2.297664	0.18021	-12.74994	3.12E-37	2.59E-34	0	0	1.024858	6.750962	6.997302	6.605319
AT3G42650	103.401	-2.292794	0.180088	-12.7315	3.95E-37	3.19E-34	0	0	0	6.764641	7.903775	8.104289
AT3G32880	52.51944	-2.276144	0.180259	-12.62705	1.50E-36	1.17E-33	0	0	0	6.818092	6.843857	6.498412
AT2G04770	67.72652	-2.232791	0.180177	-12.3922	2.88E-35	2.19E-32	0	0	0	7.367755	7.292464	6.450052

AT5G33050	60.16304	-2.21835	0.180248	-12.30719	8.29E-35	6.12E-32	0.954186	0	0	6.804914	7.302316	6.545202
AT2G09187	62.31674	-2.163971	0.180141	-12.01267	3.05E-33	2.19E-30	0.954186	0	0	6.750962	7.43362	6.575574
AT2G14230	76.95287	-2.139728	0.179911	-11.89328	1.28E-32	8.98E-30	2.727903	2.484087	2.361498	6.723211	7.634165	7.190249
AT1G36190	81.61812	-2.136262	0.179717	-11.88682	1.39E-32	9.45E-30	0	0	0	6.446026	7.74668	7.575524
AT1G35300	46.17448	-2.116591	0.180145	-11.74935	7.12E-32	4.73E-29	0	0	0	6.636601	6.687246	6.276037
AT5G28165	50.18872	-2.092501	0.180245	-11.60923	3.70E-31	2.40E-28	0.954186	0.940325	1.024858	6.651402	6.909787	6.33048
AT1G43840	81.15094	-2.066788	0.179501	-11.51409	1.12E-30	7.10E-28	0	0	1.024858	6.376464	7.680229	7.648771
AT4G08093	50.74363	-2.059273	0.180101	-11.43399	2.83E-30	1.71E-27	0	0.940325	1.024858	6.358536	6.528427	7.034979
AT1G36200	69.31377	-2.057129	0.179481	-11.46152	2.06E-30	1.27E-27	0	0	0	6.265396	7.341066	7.490411
AT1G42500	60.31659	-2.054377	0.179785	-11.42685	3.07E-30	1.82E-27	0	0	1.024858	6.340383	7.397308	6.836085
AT5G33389	63.99713	-2.039826	0.179775	-11.34653	7.72E-30	4.37E-27	0	0.940325	1.024858	7.275228	7.272555	6.238565
AT2G15810	195.4569	-2.037843	0.178821	-11.396	4.38E-30	2.53E-27	4.434609	5.435368	3.366369	8.508638	8.779035	8.228949
AT3G32240	49.74617	-2.012901	0.179646	-11.20485	3.86E-29	2.14E-26	0	0	0	6.226389	7.068309	6.529773
AT1G34530	62.39218	-2.005754	0.17947	-11.17598	5.35E-29	2.90E-26	0.954186	0	0	6.284511	7.477761	6.909454
AT2G13870	55.8699	-1.988732	0.179339	-11.08923	1.42E-28	7.53E-26	0	0	0	6.246024	7.321821	6.677105
AT2G12520	63.48532	-1.987575	0.179331	-11.08325	1.51E-28	7.89E-26	0.954186	0	0	6.303377	7.537374	6.88541
AT2G06740	44.90918	-1.958519	0.17946	-10.91338	9.95E-28	5.09E-25	0	0	0	6.651402	6.746181	6.013027
AT3G43862	49.49425	-1.942656	0.179615	-10.81565	2.90E-27	1.46E-24	0.954186	0	1.024858	6.575831	7.045026	6.140393
AT5G35057	55.28998	-1.918046	0.178828	-10.72563	7.72E-27	3.80E-24	0	0	0	7.196681	6.947945	6.013027
AT2G17690	80.35864	-1.894522	0.180267	-10.50954	7.81E-26	3.71E-23	2.507778	3.473601	2.626158	7.166087	7.789339	6.648818
AT4G07605	41.31245	-1.88636	0.179068	-10.53431	6.00E-26	2.90E-23	0	0	0	6.186297	6.77477	6.099175
AT3G30765	40.23082	-1.876447	0.179034	-10.48093	1.06E-25	4.93E-23	0	0	0	6.479588	6.577935	5.897562
AT2G13310	51.11748	-1.839123	0.178512	-10.3025	6.87E-25	3.15E-22	0.954186	0	0	5.967203	7.168667	6.677105
AT1G35590	33.34706	-1.822651	0.178931	-10.18635	2.28E-24	1.03E-21	0	0	0	6.12399	6.124155	5.990661
AT2G11110	45.43486	-1.791419	0.177964	-10.06621	7.79E-24	3.45E-21	0	0	0	5.764295	6.857286	6.718523
AT1G40310	39.76646	-1.785246	0.178177	-10.0195	1.25E-23	5.46E-21	0	0	0	6.496081	6.610021	5.745576
AT3G42719	36.45308	-1.769906	0.1782	-9.932148	3.02E-23	1.29E-20	0	0	0	6.303377	6.47716	5.745576
AT2G03965	37.47779	-1.7623	0.179062	-9.841844	7.43E-23	3.14E-20	0.954186	0.940325	1.024858	5.81776	6.47716	6.312559
AT2G01840	40.77592	-1.746687	0.178679	-9.775582	1.43E-22	5.87E-20	0.954186	1.504835	0	6.05887	6.802803	6.056744
AT5G29408	44.61763	-1.730669	0.177278	-9.76245	1.63E-22	6.58E-20	0	0	0	5.68019	6.883775	6.663031
AT3G33072	37.03582	-1.729177	0.177683	-9.731817	2.21E-22	8.76E-20	0	0	0	6.226389	6.610021	5.718612
AT3G43128	62.56331	-1.726891	0.176564	-9.780517	1.37E-22	5.67E-20	0	0	0	5.791275	7.331476	7.339299
AT3G42720	44.57817	-1.698543	0.177574	-9.565294	1.12E-21	4.38E-19	0.954186	0.940325	0	6.226389	7.056714	5.921406
AT1G39110	34.17418	-1.697925	0.177899	-9.544316	1.37E-21	5.21E-19	0	0.940325	0	5.843767	6.47716	5.921406
AT4G06587	37.75332	-1.695713	0.177604	-9.547725	1.33E-21	5.11E-19	0	0.940325	0	5.68019	6.641409	6.276037
AT4G06718	40.13446	-1.683343	0.178378	-9.436928	3.84E-21	1.44E-18	1.523549	0.940325	1.024858	6.102608	6.802803	5.897562
AT4G06570	30.16553	-1.670285	0.177426	-9.413964	4.78E-21	1.77E-18	0	0	0	5.791275	6.189318	5.798037

AT3G46484	33.37746	-1.664641	0.177077	-9.400666	5.42E-21	1.98E-18	0	0	0	6.462905	6.00855	5.663124
AT5G35120	43.33248	-1.645103	0.176976	-9.295632	1.46E-20	5.26E-18	0	0	1.618012	6.512388	5.655788	6.897482
AT3G46487	35.87113	-1.603099	0.178155	-8.998342	2.29E-19	8.13E-17	1.930722	0	1.618012	5.791275	6.545119	6.013027
AT4G04293	43.00788	-1.578185	0.180226	-8.756718	2.01E-18	6.68E-16	2.507778	3.473601	2.84971	6.165826	6.40584	6.382943
AT1G36520	34.80807	-1.573259	0.176575	-8.909856	5.11E-19	1.77E-16	0	0	1.618012	6.145059	6.528427	5.545304
AT1G35600	29.92061	-1.572906	0.175943	-8.939844	3.90E-19	1.36E-16	0	0	0	5.81776	6.311428	5.545304
AT2G12980	30.12187	-1.55719	0.175647	-8.865476	7.62E-19	2.60E-16	0	0	0	5.393725	6.23118	6.056744
AT2G12260	25.46327	-1.54041	0.175857	-8.759435	1.96E-18	6.61E-16	0	0	0	5.708773	5.801843	5.575673
AT5G45082	29.07102	-1.526606	0.175197	-8.713649	2.94E-18	9.66E-16	0	0	0	5.708773	6.311428	5.514281
AT5G28927	26.04079	-1.52509	0.175506	-8.689689	3.63E-18	1.18E-15	0	0	0	5.708773	5.959583	5.482577
AT5G31719	30.26273	-1.490582	0.175601	-8.488443	2.09E-17	6.71E-15	0	0.940325	1.024858	6.013764	6.271862	5.348296
AT5G29975	29.55461	-1.488639	0.175654	-8.47484	2.35E-17	7.45E-15	0	0.940325	1.024858	5.990671	6.210401	5.348296
AT4G08091	31.02613	-1.482909	0.175321	-8.458271	2.71E-17	8.49E-15	0	1.504835	0	6.303377	6.079019	5.348296
AT2G14180	30.35084	-1.482489	0.176545	-8.397237	4.57E-17	1.40E-14	1.930722	0	1.024858	6.036494	6.167922	5.45016
AT1G42040	25.91822	-1.47396	0.174516	-8.446007	3.01E-17	9.32E-15	0	0	0	5.651028	6.055909	5.383056
AT1G49090	29.68856	-1.468637	0.175803	-8.353856	6.61E-17	1.97E-14	1.523549	0.940325	0	5.559832	6.330811	5.663124
AT1G36470	23.55811	-1.462888	0.174597	-8.378651	5.35E-17	1.62E-14	0	0	0	5.462479	5.715996	5.575673
AT2G06904	27.5272	-1.44921	0.173803	-8.338238	7.54E-17	2.23E-14	0	0	0	5.843767	6.189318	5.23869
AT4G03900	22.35066	-1.421169	0.173867	-8.173891	2.99E-16	8.73E-14	0	0	0	5.495665	5.592958	5.45016
AT2G11775	25.25853	-1.415238	0.173321	-8.16543	3.20E-16	9.26E-14	0	0	0	5.590875	6.079019	5.276159
AT5G32623	28.6842	-1.41087	0.173543	-8.129808	4.30E-16	1.22E-13	0	0	1.024858	5.245529	6.330811	5.798037
AT2G04365	23.68937	-1.4108	0.173426	-8.134888	4.12E-16	1.18E-13	0	0	0	5.919088	5.422755	5.383056
AT5G32566	24.19211	-1.40016	0.173112	-8.088155	6.06E-16	1.70E-13	0	0	0	5.393725	6.00855	5.383056
AT2G06790	21.79746	-1.397861	0.173413	-8.060858	7.58E-16	2.10E-13	0	0	0	5.559832	5.422755	5.45016
AT3G29732	25.48089	-1.391869	0.175051	-7.951201	1.85E-15	4.96E-13	0	0	2.037128	5.462479	5.882868	5.634559
AT2G06800	22.16054	-1.385773	0.173066	-8.007186	1.17E-15	3.22E-13	0	0	0	5.245529	5.624715	5.605416
AT2G06002	43.38386	-1.375224	0.179652	-7.654928	1.93E-14	4.64E-12	3.72108	3.793422	3.366369	6.206482	6.349937	6.200094
AT5G28926	22.18812	-1.374082	0.172777	-7.952913	1.82E-15	4.94E-13	0	0	0	5.736801	5.493264	5.23869
AT5G32107	23.44913	-1.361568	0.173942	-7.827713	4.97E-15	1.28E-12	0	0.940325	1.024858	5.590875	5.801843	5.23869
AT4G03860	25.59549	-1.359147	0.173503	-7.833575	4.74E-15	1.24E-12	0.954186	0	1.024858	5.708773	6.055909	5.160702
AT2G10480	21.5469	-1.359021	0.172507	-7.878047	3.33E-15	8.76E-13	0	0	0	5.393725	5.686206	5.276159
AT4G28970	26.52768	-1.357365	0.171772	-7.90215	2.74E-15	7.29E-13	0	0	0	5.358078	6.311428	5.383056
AT4G06505	30.56068	-1.334242	0.170763	-7.813417	5.57E-15	1.42E-12	0	0	0	6.145059	6.40584	4.945016
AT5G32624	23.17477	-1.333314	0.17159	-7.770335	7.83E-15	1.98E-12	0	0	0	5.651028	5.882868	5.035195
AT1G50850	22.44253	-1.330828	0.171633	-7.7539	8.91E-15	2.22E-12	0	0	0	5.708773	5.715996	5.035195
AT4G06517	25.61987	-1.329504	0.171161	-7.767569	8.00E-15	2.01E-12	0	0	0	5.943346	6.00855	4.945016
AT3G30396	20.10371	-1.325077	0.171871	-7.709696	1.26E-14	3.08E-12	0	0	0	5.321529	5.422755	5.348296

AT5G32306	22.47468	-1.322307	0.171402	-7.714646	1.21E-14	2.99E-12	0	0	0	5.590875	5.82936	5.035195
AT4G08131	21.11596	-1.317826	0.173364	-7.601487	2.93E-14	6.95E-12	0	0.940325	1.024858	5.393725	5.45844	5.383056
AT2G19840	25.12515	-1.312257	0.176058	-7.453562	9.09E-14	2.03E-11	1.523549	1.504835	2.037128	5.495665	5.908896	5.383056
AT2G12540	27.20024	-1.303751	0.172524	-7.556945	4.13E-14	9.63E-12	1.523549	0.940325	0	5.035921	6.189318	5.848657
AT5G19015	37.70703	-1.298299	0.169279	-7.669592	1.73E-14	4.17E-12	0	0	0	4.942736	6.870592	6.34818
AT3G30749	22.11942	-1.294501	0.170692	-7.583835	3.35E-14	7.90E-12	0	0	0	5.68019	5.745183	4.945016
AT5G45570	23.84342	-1.289994	0.172922	-7.459981	8.65E-14	1.95E-11	0	0.940325	1.618012	5.358078	5.984274	5.276159
AT3G33067	24.98147	-1.283963	0.169995	-7.552925	4.26E-14	9.84E-12	0	0	0	5.736801	6.124155	4.89772
AT1G36460	21.27483	-1.27452	0.170257	-7.485843	7.11E-14	1.63E-11	0	0	0	5.245529	5.856362	5.120067
AT1G44060	21.75318	-1.267361	0.171935	-7.371145	1.69E-13	3.66E-11	0	0	1.618012	5.393725	5.745183	5.160702
AT2G10180	19.77424	-1.266942	0.171366	-7.393195	1.43E-13	3.15E-11	0	0	1.024858	5.462479	5.348623	5.160702
AT4G03910	22.88077	-1.261555	0.16963	-7.437087	1.03E-13	2.28E-11	0	0	0	5.205974	6.079019	5.160702
AT3G30695	21.15458	-1.261553	0.172752	-7.302672	2.82E-13	5.86E-11	0	1.504835	1.024858	5.28403	5.624715	5.276159
AT4G03790	22.69604	-1.25905	0.172232	-7.310211	2.67E-13	5.59E-11	0	1.909536	0	5.708773	5.624715	5.120067
AT5G34790	104.5599	-1.248199	0.166884	-7.479454	7.46E-14	1.70E-11	5.493835	5.552993	5.602852	7.552565	7.43362	7.045866
AT2G10490	18.96077	-1.247841	0.169878	-7.345504	2.05E-13	4.36E-11	0	0	0	5.245529	5.493264	5.078255
AT4G06698	21.19688	-1.245941	0.17135	-7.271301	3.56E-13	7.18E-11	1.523549	0	0	5.205974	5.745183	5.23869
AT1G33135	28.69035	-1.243771	0.168417	-7.385075	1.52E-13	3.32E-11	0	0	0	4.790597	6.424003	5.944862
AT5G32228	20.0513	-1.241839	0.169491	-7.326876	2.36E-13	4.97E-11	0	0	0	5.358078	5.686206	4.945016
AT2G13000	26.07062	-1.240434	0.168584	-7.357944	1.87E-13	4.01E-11	0	0	0	4.736097	5.959583	6.140393
AT3G30767	18.39953	-1.235982	0.169623	-7.286623	3.18E-13	6.55E-11	0	0	0	5.358078	5.270475	5.078255
AT4G06686	18.60811	-1.235294	0.169561	-7.285234	3.21E-13	6.57E-11	0	0	0	5.28403	5.422755	5.035195
AT3G29730	21.21175	-1.233208	0.171952	-7.171802	7.40E-13	1.45E-10	0.954186	0.940325	1.024858	5.462479	5.686206	4.99081
AT1G50860	18.24178	-1.228419	0.16942	-7.250729	4.15E-13	8.29E-11	0	0	0	5.123452	5.386166	5.160702
AT2G12300	21.31523	-1.227945	0.168848	-7.272487	3.53E-13	7.17E-11	0	0	0	5.245529	5.934462	4.99081
AT3G42360	18.03059	-1.227803	0.169443	-7.246122	4.29E-13	8.51E-11	0	0	0	5.28403	5.187849	5.160702
AT5G32197	20.25727	-1.209437	0.168436	-7.180384	6.95E-13	1.37E-10	0	0	0	5.358078	5.773791	4.848821
AT3G30680	18.06035	-1.208503	0.168819	-7.158572	8.15E-13	1.58E-10	0	0	0	5.393725	5.229754	4.99081
AT1G42745	17.6938	-1.205127	0.168786	-7.139966	9.34E-13	1.79E-10	0	0	0	5.035921	5.270475	5.23869
AT5G28810	18.29304	-1.200149	0.168501	-7.122517	1.06E-12	2.01E-10	0	0	0	5.205974	5.493264	4.945016
AT1G34590	23.27288	-1.195743	0.168522	-7.095452	1.29E-12	2.43E-10	0	0	1.024858	5.393725	6.124155	4.89772
AT2G13050	36.00922	-1.188526	0.166165	-7.152692	8.51E-13	1.64E-10	0	0	0	4.620502	6.687246	6.514178
AT3G44215	18.76794	-1.182214	0.170076	-6.951093	3.62E-12	6.60E-10	0	0.940325	1.024858	5.08035	5.493264	5.120067
AT5G45085	17.92974	-1.180825	0.167928	-7.031718	2.04E-12	3.82E-10	0	0	0	5.358078	5.348623	4.848821
AT2G04655	19.17174	-1.175363	0.1675	-7.017108	2.27E-12	4.21E-10	0	0	0	4.843113	5.715996	5.200223
AT2G15555	21.86542	-1.171308	0.17167	-6.823039	8.91E-12	1.56E-09	1.930722	1.504835	0	5.205974	5.82936	5.160702
AT5G36650	20.53391	-1.165027	0.166909	-6.980028	2.95E-12	5.45E-10	0	0	0	4.679457	5.82936	5.45016

AT2G00430	22.14834	-1.163892	0.174866	-6.655889	2.82E-11	4.77E-09	1.930722	1.909536	2.037128	5.28403	5.686206	5.200223
AT3G44042	18.78933	-1.160659	0.167063	-6.947434	3.72E-12	6.73E-10	0	0	0	4.736097	5.624715	5.312678
AT4G06544	19.11505	-1.157069	0.166876	-6.933707	4.10E-12	7.37E-10	0	0	0	4.679457	5.624715	5.416998
AT1G49070	16.84464	-1.156916	0.167334	-6.913828	4.72E-12	8.42E-10	0	0	0	5.28403	5.100203	4.945016
AT4G07586	18.00316	-1.145433	0.168997	-6.777838	1.22E-11	2.11E-09	0	1.504835	0	5.358078	5.144692	5.035195
AT1G37340	18.73807	-1.138019	0.166268	-6.844492	7.67E-12	1.36E-09	0	0	0	4.620502	5.527266	5.482577
AT1G50735	18.89185	-1.124512	0.165756	-6.78415	1.17E-11	2.03E-09	0	0	0	4.942736	5.801843	4.89772
AT1G44070	16.7136	-1.121168	0.166067	-6.751288	1.47E-11	2.51E-09	0	0	0	4.790597	5.386166	5.078255
AT4G04223	153.2606	-1.116415	0.160082	-6.974004	3.08E-12	5.65E-10	6.594304	6.009174	6.277467	7.771603	8.015798	7.711657
AT2G12460	39.97239	-1.114586	0.170275	-6.545812	5.92E-11	9.60E-09	2.507778	2.225226	2.037128	4.942736	7.045026	6.099175
AT2G10640	18.49327	-1.106963	0.167568	-6.606033	3.95E-11	6.60E-09	0	0.940325	1.024858	4.990081	5.655788	4.89772
AT1G40121	16.94673	-1.105172	0.165416	-6.681159	2.37E-11	4.04E-09	0	0	0	5.035921	5.493264	4.745752
AT5G29562	17.23119	-1.101052	0.166451	-6.614859	3.72E-11	6.26E-09	0.954186	0	0	5.358078	5.270475	4.691318
AT5G26270	26.01006	-1.098398	0.178519	-6.15282	7.61E-10	1.07E-07	3.087445	2.893891	2.626158	5.358078	5.592958	5.663124
AT1G40137	16.05956	-1.087728	0.164939	-6.594721	4.26E-11	7.08E-09	0	0	0	4.990081	5.348623	4.745752
AT3G42721	15.30039	-1.086407	0.165067	-6.581609	4.65E-11	7.64E-09	0	0	0	5.08035	5.006885	4.848821
AT5G28785	15.62046	-1.085872	0.164964	-6.582463	4.63E-11	7.64E-09	0	0	0	4.736097	5.100203	5.160702
AT4G08050	43.06509	-1.084279	0.179763	-6.031699	1.62E-09	2.21E-07	3.238386	4.5834	3.043225	6.303377	6.251664	6.078115
AT3G43863	19.72199	-1.081102	0.167436	-6.456797	1.07E-10	1.66E-08	0.954186	0	1.618012	5.428511	5.655788	4.634749
AT4G06573	15.88595	-1.078578	0.164618	-6.552002	5.68E-11	9.26E-09	0	0	0	5.035921	5.310078	4.691318
AT3G33225	15.14546	-1.077206	0.164742	-6.538766	6.20E-11	9.82E-09	0	0	0	4.942736	5.100203	4.848821
AT3G26530	18.86731	-1.071387	0.163734	-6.543467	6.01E-11	9.63E-09	0	0	0	4.559035	5.82936	5.160702
AT5G44890	18.86731	-1.071387	0.163734	-6.543467	6.01E-11	9.63E-09	0	0	0	4.559035	5.82936	5.160702
AT1G08740	19.04635	-1.070107	0.163653	-6.53888	6.20E-11	9.82E-09	0	0	0	4.559035	5.856362	5.160702
AT5G29040	20.20016	-1.06492	0.164364	-6.479031	9.23E-11	1.44E-08	0.954186	0	0	5.843767	5.45844	4.450377
AT4G06615	16.07585	-1.063033	0.163949	-6.48393	8.94E-11	1.41E-08	0	0	0	5.123452	5.348623	4.575871
AT2G29240	18.84451	-1.05456	0.164242	-6.420784	1.36E-10	2.08E-08	0.954186	0	0	4.559035	5.801843	5.160702
AT2G12240	16.09192	-1.052564	0.163517	-6.437026	1.22E-10	1.88E-08	0	0	0	5.035921	5.422755	4.575871
AT1G37735	14.6218	-1.051802	0.163838	-6.419779	1.36E-10	2.09E-08	0	0	0	4.942736	5.006885	4.798207
AT3G43563	16.21183	-1.045411	0.16596	-6.299185	2.99E-10	4.47E-08	0.954186	0	1.024858	4.893784	5.348623	4.798207
AT1G37040	15.85066	-1.039369	0.165832	-6.267607	3.67E-10	5.36E-08	0	0.940325	1.024858	4.893784	5.270475	4.798207
AT1G21020	17.48342	-1.030708	0.16232	-6.349865	2.16E-10	3.28E-08	0	0	0	4.494833	5.715996	5.035195
AT1G20400	17.64857	-1.027393	0.163409	-6.28725	3.23E-10	4.80E-08	0.954186	0	0	5.393725	5.493264	4.383283
AT2G11500	14.09272	-1.026659	0.162909	-6.302035	2.94E-10	4.41E-08	0	0	0	4.893784	4.907112	4.798207
AT2G06906	17.33341	-1.026649	0.163537	-6.277773	3.43E-10	5.05E-08	0	0	1.024858	5.205974	5.560486	4.450377
AT1G40112	14.85189	-1.026005	0.162687	-6.306616	2.85E-10	4.31E-08	0	0	0	4.790597	5.270475	4.691318
AT4G03760	17.14851	-1.024306	0.163486	-6.265407	3.72E-10	5.41E-08	0	0	1.024858	4.679457	5.655788	4.848821

AT5G32404	14.67567	-1.019936	0.162468	-6.27778	3.43E-10	5.05E-08	0	0	0	4.942736	5.187849	4.575871
AT3G30663	15.22626	-1.014684	0.162105	-6.259412	3.86E-10	5.59E-08	0	0	0	4.942736	5.348623	4.514488
AT3G42916	15.33813	-1.01189	0.164896	-6.136527	8.43E-10	1.17E-07	0.954186	0	1.024858	4.843113	5.229754	4.745752
AT5G33255	14.74238	-1.010649	0.163508	-6.18105	6.37E-10	9.01E-08	0	0.940325	0	4.620502	5.006885	5.078255
AT2G05914	17.88995	-1.00982	0.170992	-5.905673	3.51E-09	4.65E-07	2.247911	1.909536	1.024858	5.035921	5.054299	5.200223
AT3G32897	14.5127	-1.005376	0.161868	-6.211105	5.26E-10	7.57E-08	0	0	0	4.843113	5.229754	4.575871
AT1G39830	14.08707	-1.002692	0.161852	-6.195115	5.82E-10	8.29E-08	0	0	0	4.559035	5.006885	4.99081
AT1G40074	14.08707	-1.002692	0.161852	-6.195115	5.82E-10	8.29E-08	0	0	0	4.559035	5.006885	4.99081
AT2G06562	16.46304	-0.993684	0.160909	-6.175425	6.60E-10	9.29E-08	0	0	0	4.494833	5.655788	4.848821
AT4G03310	14.82786	-0.988089	0.162484	-6.081149	1.19E-09	1.65E-07	0.954186	0	0	4.990081	4.490884	5.200223
AT2G10630	13.23791	-0.974085	0.160773	-6.058745	1.37E-09	1.89E-07	0	0	0	4.893784	4.799922	4.634749
AT4G06736	14.90054	-0.966199	0.159984	-6.039347	1.55E-09	2.12E-07	0	0	0	4.990081	5.348623	4.312916
AT2G14950	13.57842	-0.962615	0.161762	-5.950817	2.67E-09	3.58E-07	0.954186	0	0	4.679457	4.957861	4.745752
AT1G38260	17.98752	-0.960953	0.169002	-5.686061	1.30E-08	1.63E-06	2.507778	1.504835	1.024858	5.245529	5.270475	4.745752
AT1G38330	17.98752	-0.960953	0.169002	-5.686061	1.30E-08	1.63E-06	2.507778	1.504835	1.024858	5.245529	5.270475	4.745752
AT2G06590	15.24472	-0.956274	0.159439	-5.997751	2.00E-09	2.72E-07	0	0	0	4.427639	5.527266	4.745752
AT3G32210	15.09377	-0.954972	0.159401	-5.991002	2.09E-09	2.82E-07	0	0	0	4.204956	5.144692	5.312678
AT5G32511	14.3367	-0.94565	0.159133	-5.942514	2.81E-09	3.75E-07	0	0	0	5.205974	5.054299	4.238941
AT4G05587	14.50373	-0.942327	0.16056	-5.868995	4.38E-09	5.77E-07	0	0	1.024858	5.165303	5.054299	4.312916
AT1G40123	13.4895	-0.93981	0.159062	-5.908468	3.45E-09	4.59E-07	0	0	0	4.679457	5.187849	4.450377
AT5G28923	14.16914	-0.9371	0.160311	-5.845501	5.05E-09	6.62E-07	0	0.940325	0	4.843113	5.229754	4.383283
AT5G33391	14.0162	-0.922297	0.158059	-5.835139	5.37E-09	6.97E-07	0	0	0	5.08035	5.144692	4.160966
AT1G23915	14.57982	-0.921716	0.157903	-5.837236	5.31E-09	6.92E-07	0	0	0	4.559035	5.493264	4.450377
AT4G08016	14.01222	-0.912729	0.159267	-5.730794	1.00E-08	1.28E-06	0	0	1.024858	4.990081	5.144692	4.238941
AT3G30790	15.09522	-0.911987	0.158872	-5.740403	9.45E-09	1.21E-06	0	0	1.024858	4.122374	5.144692	5.312678
AT3G30713	12.85247	-0.909194	0.157681	-5.766044	8.12E-09	1.05E-06	0	0	0	4.283066	4.799922	5.035195
AT4G06484	13.82501	-0.908365	0.159014	-5.712475	1.11E-08	1.42E-06	0.954186	0	0	4.942736	5.144692	4.238941
AT4G07490	13.88502	-0.906307	0.158877	-5.704455	1.17E-08	1.48E-06	0.954186	0	0	4.204956	5.006885	5.120067
AT3G43523	13.59863	-0.896591	0.160127	-5.599245	2.15E-08	2.63E-06	1.523549	0	0	4.559035	5.100203	4.634749
AT1G60190	151.5828	-0.892742	0.180256	-4.952641	7.32E-07	7.70E-05	5.885676	6.551747	5.72077	8.61817	7.312102	7.303455
AT4G02312	15.53822	-0.891145	0.162024	-5.500082	3.80E-08	4.51E-06	1.523549	0.940325	1.024858	4.427639	5.493264	4.691318
AT2G06965	12.54282	-0.89026	0.156778	-5.678472	1.36E-08	1.70E-06	0	0	0	4.204956	4.907112	4.89772
AT3G01345	16.68053	-0.882513	0.163585	-5.394822	6.86E-08	7.93E-06	1.930722	1.909536	0	4.559035	5.527266	4.798207
AT2G11650	12.26836	-0.880896	0.15636	-5.633758	1.76E-08	2.18E-06	0	0	0	4.427639	5.054299	4.450377
AT5G35052	12.96346	-0.878052	0.15602	-5.627833	1.82E-08	2.25E-06	0	0	0	5.035921	4.957861	4.078535
AT3G47330	13.3615	-0.87623	0.155826	-5.623112	1.88E-08	2.30E-06	0	0	0	4.494833	5.348623	4.312916
AT3G31356	12.2499	-0.87071	0.155811	-5.588257	2.29E-08	2.79E-06	0	0	0	4.942736	4.799922	4.160966

AT5G28760	12.03651	-0.867763	0.157533	-5.508435	3.62E-08	4.32E-06	0	0.940325	0	4.427639	4.854512	4.575871
AT4G06704	11.73949	-0.863026	0.155536	-5.548735	2.88E-08	3.48E-06	0	0	0	4.679457	4.854512	4.238941
AT1G77960	80.99931	-0.856421	0.17876	-4.790911	1.66E-06	1.64E-04	4.665316	5.6877	5.663016	6.606536	7.282544	6.797952
AT2G06670	11.36773	-0.855903	0.155249	-5.513115	3.53E-08	4.22E-06	0	0	0	4.357163	4.799922	4.514488
AT1G43880	17.35405	-0.852489	0.155208	-5.492548	3.96E-08	4.68E-06	0	0	1.024858	3.941514	5.856362	5.035195
AT3G43867	13.9003	-0.84573	0.155755	-5.429873	5.64E-08	6.61E-06	0	0	1.024858	4.736097	5.386166	4.078535
AT4G29200	13.50026	-0.8443	0.157453	-5.362245	8.22E-08	9.47E-06	0	0.940325	1.024858	4.843113	5.187849	4.078535
AT4G07530	13.81479	-0.83632	0.153521	-5.447601	5.11E-08	6.01E-06	0	0	0	3.841804	5.348623	4.945016
AT2G12305	10.8065	-0.836301	0.154303	-5.419876	5.96E-08	6.93E-06	0	0	0	4.559035	4.420256	4.514488
AT1G40101	13.03296	-0.832448	0.153477	-5.423918	5.83E-08	6.80E-06	0	0	0	4.357163	5.386166	4.238941
AT4G16215	12.27511	-0.824465	0.155016	-5.318577	1.05E-07	1.20E-05	0.954186	0	0	4.283066	5.144692	4.383283
AT5G41830	15.44618	-0.820062	0.165927	-4.942307	7.72E-07	8.09E-05	2.247911	0.940325	2.361498	4.736097	5.144692	4.634749
AT1G39190	11.57277	-0.808731	0.154492	-5.234788	1.65E-07	1.87E-05	0	0	1.024858	4.736097	4.799922	4.078535
AT3G31540	10.47274	-0.805246	0.152565	-5.278035	1.31E-07	1.49E-05	0	0	0	4.679457	4.345992	4.312916
AT4G09380	12.8135	-0.795447	0.154833	-5.13744	2.79E-07	3.10E-05	0.954186	0.940325	0	4.620502	5.229754	3.991107
AT3G33118	12.10905	-0.793191	0.153196	-5.177635	2.25E-07	2.51E-05	0	0.940325	0	4.122374	5.187849	4.383283
AT3G30170	10.99374	-0.789869	0.15564	-5.074976	3.88E-07	4.26E-05	0.954186	0	1.024858	4.357163	4.622542	4.450377
AT4G06628	10.36082	-0.785734	0.151403	-5.189682	2.11E-07	2.36E-05	0	0	0	4.357163	4.743185	4.160966
AT3G25770	165.7009	-0.783625	0.170228	-4.603385	4.16E-06	0.000379	6.016788	6.923433	6.919246	7.979431	7.594621	7.950552
AT5G32475	10.88332	-0.78194	0.153169	-5.105091	3.31E-07	3.66E-05	0	0	1.024858	4.427639	4.799922	4.160966
AT4G07920	14.44617	-0.780573	0.150137	-5.199085	2.00E-07	2.26E-05	0	0	0	3.841804	5.686206	4.575871
AT3G56970	25.61612	-0.77148	0.177011	-4.358376	1.31E-05	1.09E-03	3.087445	3.886005	3.366369	4.990081	5.310078	5.772045
AT3G06315	11.04637	-0.765449	0.151926	-5.038289	4.70E-07	5.12E-05	0.954186	0	0	4.736097	4.743185	3.898037
AT1G38450	10.68286	-0.764838	0.152053	-5.03007	4.90E-07	5.32E-05	0.954186	0	0	4.679457	4.622542	3.991107
AT2G10310	10.37711	-0.762769	0.149938	-5.087218	3.63E-07	4.01E-05	0	0	0	4.559035	4.743185	3.898037
AT1G11210	276.7094	-0.760235	0.173328	-4.386109	1.15E-05	9.87E-04	7.145912	7.471191	7.556823	9.176552	8.125306	8.233718
AT4G08080	10.50381	-0.756742	0.151614	-4.991243	6.00E-07	6.38E-05	0.954186	0	0	4.679457	4.558214	3.991107
AT1G38430	13.25435	-0.750796	0.156991	-4.782399	1.73E-06	1.70E-04	1.523549	1.909536	0	4.357163	5.229754	4.312916
AT3G32010	10.02706	-0.749073	0.149151	-5.022255	5.11E-07	5.52E-05	0	0	0	4.494833	4.684124	3.898037
AT2G16140	18.47623	-0.748735	0.171012	-4.378261	1.20E-05	1.01E-03	2.247911	2.484087	3.366369	5.08035	5.229754	4.634749
AT3G30720	79.46087	-0.748027	0.166121	-4.502901	6.70E-06	5.92E-04	5.636604	5.713195	5.748803	6.737153	6.830302	6.732068
AT2G07400	10.94377	-0.745359	0.148646	-5.014315	5.32E-07	5.73E-05	0	0	0	4.427639	5.054299	3.798547
AT1G40230	12.38413	-0.740793	0.148013	-5.004909	5.59E-07	5.97E-05	0	0	0	4.357163	5.422755	3.798547
AT4G06622	10.04314	-0.740056	0.148547	-4.981951	6.29E-07	6.67E-05	0	0	0	4.357163	4.799922	3.898037
AT2G14240	9.599877	-0.733098	0.150476	-4.871858	1.11E-06	0.000114	0	0.940325	0	4.283066	4.345992	4.312916
AT2G23720	10.59372	-0.732978	0.147916	-4.95536	7.22E-07	7.62E-05	0	0	0	4.357163	5.006885	3.798547
AT2G06220	12.4673	-0.732512	0.152953	-4.789141	1.67E-06	1.65E-04	0.954186	0.940325	1.024858	3.941514	5.310078	4.312916

AT1G40106	9.685054	-0.730588	0.148019	-4.935774	7.98E-07	8.33E-05	0	0	0	4.357163	4.684124	3.898037
AT5G29560	9.811545	-0.729355	0.150138	-4.857895	1.19E-06	1.22E-04	0.954186	0	0	4.122374	4.622542	4.238941
AT5G32495	9.811762	-0.72707	0.149981	-4.847737	1.25E-06	0.000127	0.954186	0	0	4.494833	4.490884	3.991107
AT2G11050	10.50068	-0.723395	0.153795	-4.703617	2.56E-06	2.44E-04	0.954186	0.940325	1.024858	4.204956	4.743185	4.160966
AT4G03770	13.70714	-0.718202	0.148007	-4.852474	1.22E-06	0.000125	0	0	1.024858	3.493167	5.527266	4.745752
AT4G06542	9.436909	-0.717629	0.149501	-4.800154	1.59E-06	1.60E-04	0	0.940325	0	4.122374	4.420256	4.312916
AT2G13547	10.13784	-0.71449	0.149074	-4.792838	1.64E-06	1.63E-04	0	0	1.024858	4.843113	4.184908	3.991107
AT4G07458	8.928275	-0.705422	0.146512	-4.814781	1.47E-06	1.49E-04	0	0	0	3.941514	4.345992	4.383283
AT2G01422	10.43336	-0.703839	0.145959	-4.822168	1.42E-06	0.000144	0	0	0	4.034777	5.100203	3.898037
AT1G35650	9.834122	-0.69945	0.145806	-4.797127	1.61E-06	1.61E-04	0	0	0	3.618975	4.799922	4.450377
AT3G47320	9.866485	-0.699282	0.145787	-4.796583	1.61E-06	0.000161	0	0	0	3.734688	4.907112	4.238941
AT2G14730	10.75626	-0.697485	0.14543	-4.796027	1.62E-06	1.61E-04	0	0	0	3.618975	5.144692	4.312916
AT2G11120	10.5622	-0.696697	0.147475	-4.72417	2.31E-06	2.21E-04	0	0.940325	0	3.841804	5.054299	4.160966
AT2G14330	8.966284	-0.69271	0.14559	-4.757955	1.96E-06	1.91E-04	0	0	0	4.494833	4.345992	3.798547
AT1G49080	9.359119	-0.69079	0.14533	-4.753258	2.00E-06	1.94E-04	0	0	0	4.034777	4.799922	3.898037
AT4G06531	11.1213	-0.689991	0.146793	-4.700451	2.60E-06	2.47E-04	0.954186	0	0	3.941514	5.229754	3.991107
AT1G43870	9.870764	-0.689354	0.147438	-4.675547	2.93E-06	2.76E-04	0	0	1.024858	3.941514	4.854512	4.078535
AT2G10250	15.74819	-0.688897	0.168351	-4.092025	4.28E-05	0.003241	1.930722	3.062067	2.626158	4.559035	4.743185	4.99081
AT4G06748	8.616023	-0.687609	0.145338	-4.731094	2.23E-06	2.16E-04	0	0	0	4.034777	4.420256	4.078535
AT5G47230	61.62908	-0.686638	0.179318	-3.829153	1.29E-04	0.008638	5.663538	5.010989	4.854918	6.831152	6.330811	6.119931
AT2G06710	8.79528	-0.685581	0.145128	-4.723977	2.31E-06	2.21E-04	0	0	0	4.427639	4.345992	3.798547
AT4G07518	9.127264	-0.68369	0.147247	-4.643137	3.43E-06	3.19E-04	0	0.940325	0	4.034777	4.558214	4.078535
AT5G20150	231.0495	-0.67923	0.139255	-4.877612	1.07E-06	1.12E-04	7.316827	7.515339	7.315767	8.226765	8.114163	8.294323
AT5G28235	8.618629	-0.675854	0.144469	-4.6782	2.89E-06	2.74E-04	0	0	0	3.841804	4.490884	4.160966
AT4G05275	8.816783	-0.67484	0.144329	-4.675695	2.93E-06	0.000276	0	0	0	4.357163	4.490884	3.691684
AT1G41720	8.77117	-0.670836	0.144041	-4.657272	3.20E-06	3.00E-04	0	0	0	4.620502	4.097077	3.798547
AT4G08593	8.228401	-0.669284	0.144109	-4.644305	3.41E-06	3.18E-04	0	0	0	4.204956	4.00355	4.160966
AT5G28826	9.957102	-0.664925	0.14765	-4.503382	6.69E-06	5.92E-04	0.954186	0.940325	0	4.843113	4.267697	3.691684
AT3G33166	18.25249	-0.662644	0.17088	-3.877841	0.000105	0.007358	2.918845	3.588268	2.037128	4.843113	5.100203	4.848821
AT1G35647	10.50008	-0.661134	0.144893	-4.56291	5.04E-06	4.56E-04	0	0.940325	0	3.35533	4.907112	4.634749
AT1G41900	9.882558	-0.660271	0.142935	-4.619366	3.85E-06	3.56E-04	0	0	0	3.493167	5.006885	4.238941
AT2G44910	112.9452	-0.655304	0.164474	-3.984246	6.77E-05	4.88E-03	6.507774	6.387267	5.931117	7.385569	7.135984	7.130131
AT5G30762	8.279661	-0.650686	0.142672	-4.560717	5.10E-06	0.00046	0	0	0	4.357163	4.267697	3.691684
AT1G20450	1444.252	-0.649906	0.152995	-4.247878	2.16E-05	1.71E-03	10.12612	10.01261	9.882132	11.27127	10.46738	10.72269
AT5G19097	13.48742	-0.646827	0.153175	-4.222793	2.41E-05	1.89E-03	1.930722	1.909536	1.024858	4.204956	5.45844	3.898037
AT3G15760	231.3175	-0.646536	0.155027	-4.170483	3.04E-05	0.002357	7.628425	7.058861	7.37092	8.424973	8.195714	8.023972
AT3G30837	9.296714	-0.640539	0.14623	-4.380341	1.18E-05	1.00E-03	0	1.504835	0	4.283066	4.558214	3.798547

AT1G40139	8.311807	-0.639732	0.141808	-4.511256	6.44E-06	0.000575	0	0	0	4.034777	4.558214	3.691684
AT3G30836	8.493455	-0.637907	0.141606	-4.504793	6.64E-06	0.000591	0	0	0	3.841804	4.684124	3.798547
AT5G57240	349.7937	-0.632079	0.147672	-4.280289	1.87E-05	0.001491	7.838002	7.997474	8.14715	8.873036	8.469342	9.004551
AT2G39980	309.0708	-0.630897	0.164448	-3.836461	1.25E-04	8.47E-03	7.935145	7.698817	7.634005	9.064901	8.130846	8.619893
AT5G32516	7.921579	-0.630099	0.141169	-4.463429	8.07E-06	7.08E-04	0	0	0	4.357163	4.097077	3.691684
AT1G36085	8.135373	-0.629098	0.141021	-4.461037	8.16E-06	7.09E-04	0	0	0	3.841804	4.558214	3.798547
AT5G30480	8.867826	-0.627891	0.140704	-4.462507	8.10E-06	0.000709	0	0	0	4.122374	4.799922	3.450809
AT5G03090	9.385835	-0.626695	0.140471	-4.461394	8.14E-06	7.09E-04	0	0	0	3.493167	5.006885	3.991107
AT2G12500	13.1611	-0.623529	0.161374	-3.863878	1.12E-04	0.007751	1.930722	2.703491	2.037128	4.357163	5.006885	4.160966
AT5G30450	8.096507	-0.61935	0.142899	-4.33418	1.46E-05	1.20E-03	0.954186	0	0	4.034777	4.345992	3.798547
AT3G33169	9.787642	-0.614989	0.151391	-4.062258	4.86E-05	0.003632	0.954186	0.940325	2.037128	4.427639	4.345992	3.898037
AT4G03865	7.969799	-0.613237	0.139788	-4.386921	1.15E-05	0.000987	0	0	0	3.841804	4.558214	3.691684
AT2G06340	7.756005	-0.612155	0.139766	-4.379867	1.19E-05	0.001003	0	0	0	4.357163	4.097077	3.576268
AT2G11670	7.603681	-0.609152	0.139572	-4.36442	1.27E-05	0.001063	0	0	0	3.941514	4.345992	3.691684
AT5G29056	7.405744	-0.608703	0.139604	-4.360226	1.30E-05	1.08E-03	0	0	0	3.841804	4.184908	3.898037
AT1G36630	478.5635	-0.607228	0.137925	-4.402586	1.07E-05	9.24E-04	2.507778	4.343278	1.618012	9.773424	10.29676	9.482379
AT1G42510	8.730121	-0.607149	0.139053	-4.36633	1.26E-05	1.06E-03	0	0	0	3.202919	4.345992	4.691318
AT1G36270	7.720603	-0.605562	0.139227	-4.349472	1.36E-05	1.12E-03	0	0	0	3.493167	4.184908	4.312916
AT5G37385	8.525818	-0.604589	0.138908	-4.352429	1.35E-05	0.001112	0	0	0	3.941514	4.799922	3.450809
AT3G02040	516.578	-0.604481	0.137337	-4.401428	1.08E-05	0.000926	8.73045	8.529443	8.541014	9.578113	9.252894	9.140276
AT5G32103	8.795181	-0.603843	0.143834	-4.198205	2.69E-05	2.10E-03	0	0.940325	1.024858	4.494833	4.345992	3.450809
AT4G15242	9.188281	-0.602987	0.143487	-4.202387	2.64E-05	2.07E-03	0.954186	0	1.024858	3.734688	4.854512	3.798547
AT3G47260	8.049046	-0.600858	0.144248	-4.16544	3.11E-05	2.40E-03	0	0.940325	1.024858	3.841804	4.097077	4.160966
AT4G06555	8.875646	-0.598692	0.138326	-4.328128	1.50E-05	1.23E-03	0	0	0	3.493167	4.957861	3.798547
AT5G34834	8.560287	-0.596656	0.146027	-4.08594	4.39E-05	3.32E-03	0	1.504835	1.024858	3.841804	4.267697	4.160966
AT3G42718	7.053092	-0.588021	0.137977	-4.261723	2.03E-05	1.62E-03	0	0	0	3.941514	4.00355	3.798547
AT2G13040	12.30811	-0.587477	0.136776	-4.295185	1.75E-05	1.41E-03	0	0	0	2.615916	5.422755	4.798207
AT3G26512	959.7842	-0.583855	0.120218	-4.856641	1.19E-06	1.23E-04	9.62123	9.366092	9.605737	10.3085	10.16662	10.13007
AT4G08092	7.22692	-0.581926	0.137385	-4.235732	2.28E-05	0.001793	0	0	0	4.283066	3.903537	3.576268
AT3G54730	11.03196	-0.581805	0.151329	-3.844636	1.21E-04	8.23E-03	0.954186	1.909536	2.037128	3.841804	5.006885	3.991107
AT5G43800	7.382686	-0.576749	0.139713	-4.128102	3.66E-05	2.80E-03	0	0.940325	0	4.122374	4.00355	3.691684
AT3G56210	355.0207	-0.576568	0.135504	-4.254986	2.09E-05	0.00166	8.260311	8.073266	7.907067	8.655358	8.854635	8.826583
AT4G23680	576.4402	-0.575797	0.124811	-4.613349	3.96E-06	0.000365	8.869959	8.900688	8.615221	9.536861	9.397784	9.470306
AT3G32970	7.069382	-0.568449	0.136256	-4.171932	3.02E-05	0.002349	0	0	0	4.204956	4.00355	3.450809
AT2G11773	7.096099	-0.566172	0.136046	-4.16161	3.16E-05	0.00243	0	0	0	3.734688	4.345992	3.576268
AT5G07010	1042.837	-0.564604	0.13008	-4.34043	1.42E-05	1.17E-03	9.631498	9.789599	9.535057	10.49545	10.35591	10.08855
AT3G32677	7.605214	-0.557184	0.137845	-4.0421	5.30E-05	3.89E-03	0.954186	0	0	4.122374	4.345992	3.313392

AT3G30827	7.86599	-0.556842	0.137631	-4.045892	5.21E-05	3.86E-03	0.954186	0	0	3.202919	4.184908	4.450377
AT3G30610	7.941528	-0.556794	0.13756	-4.047646	5.17E-05	3.84E-03	0	0.940325	0	4.427639	4.267697	3.161495
AT3G07350	1713.157	-0.555135	0.121615	-4.5647	5.00E-06	0.000454	10.47584	10.2001	10.47448	11.16891	10.95736	10.94471
AT4G07494	7.239953	-0.553964	0.134896	-4.106597	4.02E-05	0.00306	0	0	0	3.202919	4.345992	4.078535
AT1G36185	6.700224	-0.552965	0.134986	-4.096447	4.20E-05	3.19E-03	0	0	0	3.493167	4.00355	3.991107
AT2G28400	960.5699	-0.550069	0.124026	-4.4351	9.20E-06	7.97E-04	9.72236	9.43475	9.49424	10.32475	10.16122	10.08196
AT2G05000	6.919665	-0.549718	0.134616	-4.08359	4.43E-05	3.33E-03	0	0	0	3.493167	4.345992	3.691684
AT5G31087	6.727157	-0.549095	0.134624	-4.078721	4.53E-05	3.39E-03	0	0	0	3.493167	4.184908	3.798547
AT3G44265	7.679966	-0.549002	0.139818	-3.926554	8.62E-05	6.11E-03	0.954186	0.940325	0	3.35533	4.097077	4.312916
AT3G02030	252.2305	-0.546555	0.141972	-3.84974	1.18E-04	8.11E-03	7.721079	7.508074	7.588193	8.475752	8.169039	8.180369
AT3G14440	807.5526	-0.541452	0.139767	-3.873957	1.07E-04	7.46E-03	9.114047	9.270582	9.464966	10.15077	9.659696	10.00454
AT4G04530	6.875369	-0.539593	0.136726	-3.946514	7.93E-05	5.66E-03	0.954186	0	0	3.618975	4.097077	3.798547
AT5G38365	7.821754	-0.539101	0.136222	-3.957502	7.57E-05	5.43E-03	0	0	1.024858	3.493167	4.684124	3.576268
AT1G42727	6.676331	-0.534891	0.133313	-4.012296	6.01E-05	0.004406	0	0	0	4.283066	3.415245	3.691684
AT5G04925	6.879698	-0.530556	0.132839	-3.993976	6.50E-05	4.71E-03	0	0	0	4.427639	3.679952	3.313392
AT1G25560	3448.778	-0.527549	0.121992	-4.324465	1.53E-05	0.001244	11.54669	11.17389	11.50203	11.98177	11.96942	12.1169
AT5G37125	6.896872	-0.526281	0.13544	-3.885701	1.02E-04	7.16E-03	0.954186	0	0	3.493167	4.267697	3.691684
AT2G10280	6.537257	-0.523534	0.132282	-3.957697	7.57E-05	5.43E-03	0	0	0	3.202919	4.097077	3.991107
AT3G45520	93.54737	-0.522043	0.130662	-3.995365	6.46E-05	4.69E-03	0	1.909536	0	6.544458	8.267882	7.321488
AT3G32966	6.522717	-0.520916	0.135207	-3.852717	1.17E-04	0.008029	0.954186	0	0	3.734688	3.903537	3.691684
AT4G03880	6.902518	-0.513376	0.134175	-3.826167	1.30E-04	0.008699	0.954186	0	0	4.122374	4.097077	3.161495
AT3G62475	6.203718	-0.511685	0.131247	-3.898634	9.67E-05	6.81E-03	0	0	0	4.034777	3.796072	3.313392
AT5G29574	6.233258	-0.502894	0.130369	-3.857475	1.15E-04	0.007916	0	0	0	3.841804	4.097077	3.161495
AT2G06760	5.848243	-0.497563	0.129966	-3.8284	1.29E-04	8.64E-03	0	0	0	3.841804	3.679952	3.450809
AT3G32020	6.610019	-0.4966	0.129621	-3.831161	1.28E-04	0.008589	0	0	0	3.35533	4.490884	3.313392
AT1G44040	5.818487	-0.494266	0.129639	-3.812636	1.37E-04	9.12E-03	0	0	0	3.493167	3.553661	3.898037
AT5G54510	1424.607	-0.469236	0.122945	-3.816635	1.35E-04	0.008997	10.20272	10.17395	10.16689	10.94017	10.55755	10.64247
AT3G26510	1961.073	-0.466696	0.121609	-3.837678	1.24E-04	8.45E-03	10.78344	10.47993	10.65567	11.33339	11.11729	11.08129
AT4G15760	4859.7	0.441755	0.102989	4.289348	1.79E-05	1.44E-03	12.46555	12.437	12.50436	11.87034	12.05276	12.02256
AT1G61100	1623.498	0.511305	0.129683	3.942718	8.06E-05	5.73E-03	10.85747	10.99253	10.95466	10.04686	10.53579	10.37422
AT5G44680	2240.122	0.511517	0.134897	3.791907	1.49E-04	0.009892	11.24898	11.42308	11.52906	10.47479	10.86731	10.98062
AT5G58120	399.2086	0.528084	0.130632	4.042537	5.29E-05	3.89E-03	8.867065	8.872678	9.023613	8.280119	8.451744	8.150417
AT1G70090	1065.955	0.556875	0.142801	3.899642	9.63E-05	6.81E-03	10.47774	10.2158	10.38576	9.289671	9.910566	9.748746
AT1G23030	757.0578	0.578462	0.151526	3.817586	1.35E-04	8.98E-03	9.930717	9.763906	9.975541	8.636884	9.329568	9.354661
AT5G01810	449.5639	0.585283	0.146058	4.007198	6.14E-05	0.00449	9.06691	9.175194	9.168149	8.053732	8.365092	8.708211
AT2G03760	1812.482	0.585637	0.130299	4.494563	6.97E-06	6.14E-04	10.87486	11.22297	11.26055	10.25378	10.50306	10.53838
AT5G62670	2069.407	0.589732	0.117646	5.012769	5.37E-07	5.75E-05	11.15839	11.44131	11.32365	10.50973	10.70038	10.70648

AT4G16680	718.3733	0.591808	0.14978	3.951167	7.78E-05	0.005561	9.529044	10.17624	9.674943	9.229296	9.024958	8.912373
AT3G61630	706.6826	0.592047	0.153525	3.856349	0.000115	0.007932	9.696512	9.653007	10.03081	8.556602	9.232427	9.197543
AT1G03300	111.2496	0.607942	0.16039	3.790408	0.00015	0.009928	7.164887	7.253881	7.07129	6.246024	6.56162	6.180467
AT1G10020	544.6861	0.612443	0.135292	4.526835	5.99E-06	0.000536	9.474953	9.46508	9.292389	8.560528	8.926469	8.510174
AT5G24640	283.8165	0.62006	0.161165	3.84737	1.19E-04	8.16E-03	8.446287	8.50045	8.603755	7.275228	8.119745	7.458172
AT3G46090	1441.183	0.628888	0.161297	3.89895	9.66E-05	0.006808	10.73347	11.03421	10.80749	9.508689	10.48154	9.818659
AT3G44260	801.1133	0.6297	0.146302	4.304096	1.68E-05	0.00136	10.00762	9.753192	10.17982	9.067663	9.501918	8.962075
AT3G22910	1791.519	0.640781	0.146209	4.382639	1.17E-05	0.000996	11.08121	11.28326	11.09435	10.15468	10.72623	10.07134
AT4G01360	685.2385	0.645	0.161265	3.999639	6.34E-05	4.62E-03	10.01025	9.897788	9.428601	8.662682	9.329568	8.623531
AT5G16360	117.1634	0.645905	0.159712	4.044185	5.25E-05	3.88E-03	7.342054	7.39447	7.003101	6.394171	6.368813	6.382943
AT5G13210	1148.974	0.648468	0.169205	3.832436	1.27E-04	8.59E-03	10.29173	10.7039	10.68044	8.834645	9.704965	10.03081
AT2G47000	2263.708	0.653328	0.170528	3.831211	1.28E-04	8.59E-03	11.11393	11.72751	11.74233	9.800236	10.86814	10.8091
AT5G40690	191.4017	0.662061	0.163548	4.048129	5.16E-05	3.84E-03	7.935145	8.14995	7.830434	6.694915	7.46904	6.873236
AT5G55150	2063.212	0.66273	0.156358	4.238538	2.25E-05	1.78E-03	11.28195	11.28433	11.5748	10.03703	10.92185	10.40943
AT1G05880	342.4253	0.666923	0.171726	3.883652	1.03E-04	7.20E-03	8.846645	8.76404	8.922636	7.206736	8.424939	7.560418
AT3G13080	5721.075	0.694298	0.166745	4.163833	3.13E-05	0.002413	12.42234	13.08602	13.09385	11.25501	12.13569	12.12334
AT5G58750	83.54496	0.694361	0.179948	3.858664	1.14E-04	0.007898	6.477738	6.724671	7.379911	4.843113	6.032424	5.691135
AT3G19680	1508.186	0.696562	0.170575	4.083608	4.43E-05	0.003332	10.8763	10.66278	11.3502	9.201909	10.31269	10.08063
AT2G18193	3204.629	0.696916	0.161956	4.303117	1.68E-05	1.36E-03	11.77842	12.25694	12.07788	10.43898	11.3375	11.3034
AT5G62480	368.8228	0.70396	0.176583	3.986564	6.70E-05	0.004846	8.904238	8.855607	9.216629	6.919398	8.442864	7.732024
AT3G00800	94.37819	0.724027	0.168793	4.289437	1.79E-05	1.44E-03	6.826528	6.879069	7.287379	5.791275	5.856362	6.160569
AT1G50040	292.0555	0.725422	0.176426	4.111761	3.93E-05	0.003001	8.736804	8.436833	8.823971	6.446026	7.877654	7.711657
AT2G36800	1139.422	0.745219	0.157678	4.726196	2.29E-06	0.00022	10.18888	10.67824	10.78143	9.111139	9.678731	9.820245
AT4G08985	356.7989	0.757971	0.166431	4.554272	5.26E-06	4.72E-04	8.858349	9.1706	8.718061	7.429165	8.331809	7.575524
AT5G45340	55.99935	0.762933	0.174797	4.364681	1.27E-05	1.06E-03	6.317392	6.403019	6.218547	5.08035	5.229754	4.99081
AT2G36792	2263.461	0.786594	0.170552	4.612041	3.99E-06	3.66E-04	11.2462	11.60476	11.93078	9.651729	10.69852	10.70819
AT4G34410	647.4361	0.787481	0.165073	4.7705	1.84E-06	1.80E-04	9.749325	10.06182	9.52496	8.221815	9.150258	8.482392
AT2G36790	2253.865	0.788485	0.171043	4.609868	4.03E-06	3.68E-04	11.24174	11.60348	11.92733	9.62198	10.6901	10.69788
AT3G10986	73.97447	0.789824	0.180175	4.383639	1.17E-05	0.000995	6.537198	6.434017	7.277791	4.427639	5.527266	5.575673
AT5G14730	411.8747	0.81825	0.147902	5.532379	3.16E-08	3.80E-06	9.028576	9.022958	9.285229	7.778361	8.383772	8.007357
AT1G22830	189.1244	0.823893	0.177681	4.636932	3.54E-06	3.28E-04	7.733842	8.048442	8.458543	5.764295	7.009382	7.001813
AT5G25240	101.2061	0.848436	0.161764	5.244902	1.56E-07	1.78E-05	7.005604	7.209782	7.208827	6.013764	6.055909	5.848657
AT2G23290	92.29739	0.854007	0.16874	5.061078	4.17E-07	4.56E-05	6.973677	7.245168	6.869045	5.68019	6.055909	5.575673
AT5G64870	764.2008	0.882584	0.155551	5.673904	1.40E-08	1.74E-06	9.936249	9.865464	10.30782	8.463224	9.222084	8.848514
AT1G76650	370.5589	0.976081	0.142847	6.833043	8.31E-12	1.46E-09	9.106706	9.165991	8.749618	7.876109	7.910232	7.627185
