# Supplementary material for: Induction of epigenetic variation in Arabidopsis by over-expression of DNA METHYLTRANSFERASE1 (MET1)
Source: PLoS One. 2018 Feb 21;13(2):e0192170. doi: 10.1371/journal.pone.0192170 (PMC5821449; doi:10.1371/journal.pone.0192170)
Supplement: S4 Table — (PDF) [file pone.0192170.s009.pdf]

Supplementary table 4: List of genes with altered transcript levels in line A2-

	baseMean	log2FoldCha	lfcSE	stat	pvalue	padj	control 1	control 2	control 3	A2-4	A2-5	A2-6
AT4G25580	82.954602	-5.57654	0.3377799	-16.50939	3.14E-61	3.65E-58	0.9769677	0.9592852	1.3855946	7.2350505	7.3637291	7.506598
AT4G33467	114.23502	-4.660157	0.3175133	-14.67704	9.04E-49	6.71E-46	2.8667312	2.7368381	1.8828362	7.7347315	7.2894272	8.249459
AT4G33465	88.605696	-4.640625	0.3282985	-14.13538	2.30E-45	1.49E-42	2.5463685	2.2562212	1.6555327	7.2743201	7.0221311	7.9013487
AT1G10070	1321.8585	-4.056488	0.2183247	-18.58006	4.66E-77	5.14E-73	7.2017997	7.0107677	6.7182815	10.739356	11.489301	11.536866
AT1G80160	132.84895	-3.89843	0.2182144	-17.86514	2.20E-71	9.73E-68	3.9565084	4.0098434	3.7972589	7.8431732	8.0796573	8.0078328
AT1G15380	113.12957	-3.856333	0.2935646	-13.13623	2.04E-39	9.80E-37	4.1655639	3.2477933	2.6726488	7.5866041	7.9886655	7.677659
AT4G33150	1530.6515	-3.706227	0.1540353	-24.0609	6.42E-128	1.42E-123	7.854626	7.6726689	7.4615383	11.263119	11.589467	11.569289
AT1G08630	318.83918	-3.65673	0.2631786	-13.89448	6.84E-44	4.20E-41	5.6409784	5.24144	4.7151872	8.6137933	9.4891466	9.4401143
AT4G33980	53.319348	-3.575918	0.3266282	-10.94798	6.79E-28	1.50E-25	2.1339363	2.7368381	3.0919337	6.0770468	6.7613149	7.0261521
AT5G42900	41.134378	-3.442357	0.3284045	-10.48206	1.04E-25	1.95E-23	2.6612438	2.7368381	1.8828362	6.0770468	6.2047119	6.5677036
AT4G08093	13.793023	-3.426282	0.4350551	-7.875514	3.39E-15	2.65E-13	0	0.5579473	0.620679	4.1397727	5.0904916	5.0465438
AT5G39520	168.42461	-3.419424	0.2282978	-14.97791	1.02E-50	8.69E-48	4.8378037	4.3137483	4.7444162	8.6290213	8.1230878	8.0582656
AT5G47240	117.24561	-3.318498	0.218718	-15.1725	5.38E-52	4.75E-49	4.1261063	4.1314298	4.5919633	7.7347315	7.7435613	7.8012572
AT1G68050	81.596309	-3.188035	0.2559064	-12.45782	1.27E-35	4.59E-33	3.2802015	3.9669257	4.1428676	7.2350505	7.2271013	7.2487732
AT3G42258	23.689489	-3.153998	0.4403597	-7.162321	7.93E-13	4.53E-11	0.5696486	1.2729306	0.620679	6.4642672	5.2241406	4.0895494
AT5G23240	130.60801	-3.045552	0.225164	-13.52593	1.10E-41	5.92E-39	4.683262	4.3476018	5.008125	7.6476919	8.0619123	7.9287155
AT5G23235	120.24088	-3.001784	0.2391404	-12.5524	3.86E-36	1.52E-33	4.5404893	4.2071437	4.9590991	7.5228149	7.9980273	7.7484809
AT3G61060	814.37409	-2.936941	0.2159552	-13.59977	4.02E-42	2.27E-39	7.8425041	7.0054757	7.2176151	10.285492	10.540337	10.686588
AT5G60100	77.164918	-2.933466	0.2844355	-10.31329	6.14E-25	1.10E-22	4.0008133	4.1697834	3.6193629	6.9254163	6.7831397	7.5774743
AT3G20340	163.71946	-2.930905	0.2983963	-9.822188	9.04E-23	1.31E-20	5.3470024	4.3476018	4.8287095	8.786708	7.6751769	7.9819395
AT3G33595	25.203309	-2.825249	0.4507683	-6.267629	3.67E-10	1.40E-08	0.5696486	1.2729306	0.620679	6.7692438	4.8634794	3.7645543
AT5G17460	84.103257	-2.788121	0.3282177	-8.494732	1.98E-17	1.89E-15	3.8635952	3.922692	4.186117	7.9440305	6.3286119	7.0881471
AT2G21660	1938.0129	-2.779638	0.2348678	-11.83491	2.58E-32	8.01E-30	8.1236506	9.2618146	8.6646672	11.835631	11.672935	11.737833
AT2G03865	544755.25	-2.759027	0.4525171	-6.097067	1.08E-09	3.83E-08	14.051843	14.225909	14.63744	21.090797	19.327004	18.268909
AT5G56870	3536.1097	-2.733571	0.2720592	-10.04771	9.40E-24	1.54E-21	10.073194	9.1713746	9.3606453	11.877675	12.851195	12.921692
AT5G24470	193.74574	-2.708686	0.2299463	-11.77965	4.97E-32	1.52E-29	4.9092797	5.8495183	5.7024326	8.3996454	8.4371141	8.4278759
AT2G42530	500.60416	-2.700941	0.300294	-8.994321	2.38E-19	2.66E-17	6.7994621	6.4881382	6.8518556	10.56822	9.2238563	9.1717801
AT3G06365	4537134	-2.669116	0.4519374	-5.905942	3.51E-09	1.12E-07	17.459451	17.740404	17.977399	24.138364	22.37902	21.334878
AT2G03875	1820017.5	-2.66526	0.4522919	-5.892788	3.80E-09	1.20E-07	16.19303	16.201924	16.671227	22.824068	21.054331	20.018699
AT3G33575	18.11128	-2.63844	0.455166	-5.796654	6.77E-09	2.05E-07	0	0.5579473	0	6.3946946	4.2622301	2.9759397
AT2G23030	80.550242	-2.632644	0.2681782	-9.81677	9.53E-23	1.38E-20	4.7881068	4.0098434	4.0522736	6.875214	7.3781411	7.1821107

AT3G33377	12.293117	-2.612679	0.4549845	-5.742348	9.34E-09	2.75E-07	0	0.5579473	0	5.669115	4.1322253	2.9759397
AT5G52310	912.7899	-2.608891	0.4050596	-6.440757	1.19E-10	4.76E-09	7.0624282	7.3272788	6.8915908	11.575626	10.055618	9.8968158
AT3G34299	11.600296	-2.608031	0.4549318	-5.732796	9.88E-09	2.90E-07	0	0.5579473	0	5.5461594	4.1322253	2.9759397
AT3G33205	22.257573	-2.587461	0.448983	-5.762938	8.27E-09	2.46E-07	1.5541704	0.5579473	1.3855946	6.4642672	5.0904916	3.3443906
AT3G57520	2290.8142	-2.544105	0.2367329	-10.74673	6.14E-27	1.26E-24	9.2979303	9.2739695	9.0831121	11.203334	12.138476	12.314411
AT1G73120	55.715505	-2.539512	0.3775624	-6.726073	1.74E-11	7.85E-10	3.2802015	3.0967291	3.8519957	7.5550621	5.7961798	5.9989583
AT3G19390	45.630384	-2.444544	0.3569014	-6.849354	7.42E-12	3.55E-10	3.2064485	3.7308031	3.5548313	4.9116189	6.9273135	6.5501893
AT1G76410	106.82083	-2.437158	0.2316168	-10.52237	6.81E-26	1.31E-23	5.1446787	4.7532581	5.008125	7.2350505	7.434391	7.8378064
AT3G51325	25.275723	-2.4236	0.342937	-7.067189	1.58E-12	8.58E-11	3.0465662	2.6308491	2.6726488	5.2634978	5.7525968	5.4755828
AT1G65970	141.39103	-2.419308	0.2743871	-8.817134	1.17E-18	1.23E-16	5.4937351	5.3622846	4.9838203	7.4215092	8.4977674	7.669571
AT3G41768	4039253.4	-2.396709	0.4549461	-5.268115	1.38E-07	3.32E-06	16.608688	18.202397	17.260225	23.930812	22.229958	21.399519
AT2G01010	3526008.2	-2.380492	0.4549308	-5.232647	1.67E-07	3.95E-06	16.486261	18.064305	17.135149	23.735443	22.024229	21.203357
AT1G15040	138.01983	-2.379448	0.3637474	-6.541485	6.09E-11	2.52E-09	5.2585368	5.2049685	4.4921063	6.163252	8.4232821	8.3286394
AT1G21400	2514.6632	-2.368648	0.1730951	-13.68408	1.26E-42	7.34E-40	9.6959263	9.5495814	9.5463633	11.674344	12.079067	12.336609
AT2G36270	13.434343	-2.354026	0.4089829	-5.755805	8.62E-09	2.56E-07	1.9653122	1.2729306	2.0791536	4.4452387	4.6894999	4.8314086
AT3G45300	2154.1126	-2.34521	0.1876911	-12.49505	7.94E-36	3.02E-33	9.7101726	9.1854828	9.1798937	11.575626	11.826646	12.052016
AT4G15530	2054.2744	-2.343364	0.1929367	-12.14577	6.04E-34	2.02E-31	9.3913819	9.3500528	9.1772168	11.284914	12.077964	11.817771
AT2G15880	304.37086	-2.343089	0.1796961	-13.03917	7.32E-39	3.44E-36	6.2670382	6.8068484	6.6348919	8.8530562	9.1075825	9.0493366
AT3G61890	594.62996	-2.337107	0.172728	-13.53056	1.03E-41	5.70E-39	7.7481902	7.5688625	7.2887833	10.051231	9.7244723	10.110553
AT4G26290	95.499992	-2.326256	0.2989845	-7.780524	7.22E-15	5.46E-13	5.2215736	4.564641	4.4921063	6.6548684	7.3344659	7.8663935
AT2G01021	68107.91	-2.294251	0.445889	-5.145341	2.67E-07	6.06E-06	12.887996	12.664754	13.29904	17.95247	16.37814	15.479212
AT1G19530	62.899504	-2.285636	0.2983489	-7.660952	1.85E-14	1.33E-12	4.2778476	4.3137483	4.2281074	5.8874513	7.2894272	6.7779245
AT1G76590	284.44493	-2.272956	0.1622582	-14.00826	1.39E-44	8.75E-42	6.4379048	6.4651914	6.7474485	8.965297	8.7568314	8.9593144
AT3G15440	19.245766	-2.271184	0.3885373	-5.845473	5.05E-09	1.55E-07	2.959449	1.7488329	2.2519284	5.0982599	4.8634794	5.3993805
AT2G21820	27.642343	-2.269695	0.3437145	-6.603432	4.02E-11	1.73E-09	3.2802015	2.9279768	2.6726488	5.9853613	5.1588632	5.6168815
AT1G27670	39.363801	-2.263143	0.3452188	-6.555678	5.54E-11	2.31E-09	4.085539	2.8355706	3.3418688	5.669115	6.5227227	6.0245506
AT3G33131	11.522376	-2.254466	0.4537882	-4.968102	6.76E-07	1.41E-05	0	0.5579473	0	5.4117401	4.6894999	1.7177495
AT5G15970	1563.4615	-2.236922	0.3891481	-5.748254	9.02E-09	2.67E-07	9.1409948	7.886394	8.0699823	12.001313	10.933339	11.143137
AT3G33201	10.381592	-2.232408	0.4523938	-4.934656	8.03E-07	1.66E-05	0	1.2729306	1.0532486	5.2634978	3.8307199	3.3443906
AT5G61380	353.30994	-2.2258	0.1373816	-16.20158	4.91E-59	5.43E-56	6.8366047	6.9894822	6.9617125	9.1455296	9.2632116	9.1774952
AT3G39230	13.444265	-2.21999	0.4551234	-4.877777	1.07E-06	2.15E-05	0.5696486	0.5579473	1.0532486	5.9853613	3.2122368	3.1718957
AT4G15990	43.882884	-2.209266	0.2989332	-7.390502	1.46E-13	9.31E-12	4.085539	3.9669257	3.3418688	5.8874513	6.5227227	6.2364917
AT2G33830	6308.2105	-2.202072	0.1870803	-11.77073	5.52E-32	1.67E-29	11.243311	10.732418	11.138665	13.15568	13.190287	13.666521

AT5G34780	93.485269	-2.199493	0.2443789	-9.000338	2.25E-19	2.53E-17	4.977381	4.9643561	5.0555394	6.7131893	7.3637291	7.6532575
AT1G13930	3021.9822	-2.194601	0.1982677	-11.06887	1.78E-28	4.04E-26	9.7657916	9.973428	10.186658	12.691453	12.040512	12.059773
AT1G30720	151.76947	-2.190784	0.2908344	-7.532756	4.97E-14	3.42E-12	5.4303553	5.673849	5.6415889	7.0208656	8.5811075	8.0206072
AT1G07040	786.60638	-2.181592	0.1416418	-15.40218	1.58E-53	1.46E-50	8.0090216	8.0469745	8.2766794	10.221633	10.290888	10.498498
AT2G15970	1057.6693	-2.180303	0.2197062	-9.923721	3.28E-23	4.96E-21	8.6575785	8.1295629	8.5368515	11.195684	10.357863	10.67855
AT1G11810	5.9180957	-2.176938	0.4541854	-4.793061	1.64E-06	3.15E-05	0.5696486	0	0	3.219378	4.3814822	2.9759397
AT3G33136	13.185147	-2.159174	0.4537214	-4.758809	1.95E-06	3.67E-05	0	0.9592852	0	5.8874513	4.1322253	2.148558
AT2G40080	15.479273	-2.125392	0.4042402	-5.257745	1.46E-07	3.50E-06	1.9653122	2.392196	1.8828362	5.4117401	4.5939523	4.2715627
AT3G33178	19.738591	-2.119575	0.4532224	-4.676677	2.92E-06	5.24E-05	0.5696486	0.5579473	0.620679	6.6548684	3.9893347	1.7177495
AT2G01029	23.802058	-2.105347	0.3651828	-5.765186	8.16E-09	2.43E-07	2.6612438	2.7368381	3.0919337	5.9853613	4.7791107	5.1891546
AT1G20440	3532.9998	-2.098567	0.1416994	-14.81	1.26E-49	9.95E-47	10.434385	10.397733	10.178649	12.682358	12.297626	12.477914
AT1G53885	38.982177	-2.093015	0.2895047	-7.22964	4.84E-13	2.85E-11	4.043798	3.6785597	3.6193629	5.8874513	6.0691631	6.1692457
AT1G53903	38.982177	-2.093015	0.2895047	-7.22964	4.84E-13	2.85E-11	4.043798	3.6785597	3.6193629	5.8874513	6.0691631	6.1692457
AT3G33148	8.4660426	-2.085982	0.4526409	-4.608471	4.06E-06	7.07E-05	0	0	0.620679	5.2634978	3.2122368	2.4798665
AT2G41190	178.47986	-2.073977	0.226234	-9.167397	4.85E-20	5.72E-18	5.8370045	6.027144	6.0336839	8.673764	7.8814647	7.9354769
AT5G15960	86.959092	-2.068351	0.2966954	-6.971295	3.14E-12	1.62E-10	5.2215736	4.5058886	4.6853538	7.7626156	6.6469455	6.961373
AT1G20450	1470.3917	-2.019465	0.2328499	-8.672819	4.22E-18	4.17E-16	9.1740192	9.0532483	8.9388516	11.768458	10.727846	10.997276
AT2G15890	3242.3692	-2.00679	0.1554884	-12.90636	4.14E-38	1.87E-35	10.258521	10.152874	10.437679	12.243167	12.169879	12.61057
AT1G11210	236.15479	-1.996052	0.2209216	-9.035112	1.64E-19	1.86E-17	6.2021101	6.518177	6.6191983	8.9410983	8.1230878	8.6037167
AT5G53970	621.02743	-1.974738	0.1668524	-11.83524	2.57E-32	8.01E-30	8.1801315	7.7726235	7.7976011	9.8835937	9.8435016	10.151781
AT5G52300	24.484034	-1.960183	0.4223956	-4.640633	3.47E-06	6.16E-05	1.5541704	3.3177801	2.6726488	6.3215962	4.7791107	4.710477
AT3G11550	14.162307	-1.959775	0.4054483	-4.8336	1.34E-06	2.62E-05	2.6612438	1.5304167	2.2519284	4.9116189	4.5939523	4.5076474
AT3G33193	15.015904	-1.957735	0.4432439	-4.416834	1.00E-05	1.58E-04	0.9769677	1.9384886	2.2519284	5.7824096	4.2622301	3.4984464
AT3G33151	7.9448211	-1.952676	0.4546058	-4.295316	1.74E-05	0.0002573	0.9769677	0.5579473	0	5.0982599	2.5754861	3.1718957
AT4G16146	137.556	-1.947255	0.2618297	-7.437105	1.03E-13	6.74E-12	5.2585368	5.4890323	6.158366	8.0382954	7.4205328	7.9219223
AT1G54100	3592.813	-1.936755	0.1087222	-17.8138	5.52E-71	2.03E-67	10.520598	10.525697	10.509669	12.438401	12.380493	12.616368
AT5G26280	106.91816	-1.933394	0.2292732	-8.432707	3.38E-17	3.16E-15	5.5395071	5.328778	5.424005	6.875214	7.5786433	7.7095654
AT1G56300	273.30031	-1.929855	0.176626	-10.92623	8.64E-28	1.87E-25	6.5760926	6.7884791	6.949214	8.8268819	8.5496208	8.9291436
AT4G37220	91.87239	-1.91481	0.2447962	-7.822058	5.20E-15	3.98E-13	5.0841925	5.2233195	5.3307272	6.7692438	7.1282532	7.6200665
AT1G72060	309.42126	-1.913633	0.2696456	-7.096844	1.28E-12	7.08E-11	7.4496782	6.3446409	6.5872896	8.5828465	9.0547314	9.2168792
AT1G53580	792.72401	-1.90661	0.1208036	-15.78272	4.09E-56	4.11E-53	8.3778654	8.335489	8.3687161	10.304586	10.173919	10.405379
AT4G35770	16396.005	-1.902347	0.1785493	-10.65446	1.66E-26	3.28E-24	12.823451	12.453285	12.778558	14.37215	14.612198	14.976983
AT5G66650	76.675647	-1.900134	0.2424053	-7.838665	4.55E-15	3.50E-13	4.8131692	4.9643561	5.1238615	6.5306385	7.2740963	6.961373

AT2G37130	200.4203	-1.899215	0.2077681	-9.141031	6.19E-20	7.23E-18	6.7021936	6.1279897	6.1254163	8.126777	8.4508147	8.3889965
AT3G33139	7.9148517	-1.898957	0.4489699	-4.229586	2.34E-05	3.35E-04	0	0	0.620679	5.2634978	2.5754861	2.4798665
AT1G77380	34.058572	-1.898615	0.3110538	-6.103816	1.04E-09	3.69E-08	4.0008133	3.5680299	3.6193629	5.5461594	6.1719999	5.6826588
AT3G15460	647.51186	-1.888581	0.2139706	-8.826358	1.08E-18	1.14E-16	8.2890982	7.9092437	7.8354458	9.5652174	9.9891016	10.383295
AT3G38525	7.2959528	-1.88656	0.4486516	-4.204955	2.61E-05	3.68E-04	0	0.5579473	0	5.0982599	2.9287097	2.148558
AT1G72070	321.51454	-1.872305	0.2650978	-7.062693	1.63E-12	8.84E-11	7.5198648	6.4101853	6.7618138	8.6290213	9.1119006	9.2498017
AT5G19230	61.258238	-1.87155	0.2819073	-6.638882	3.16E-11	1.39E-09	4.977381	4.6485132	4.1428676	6.163252	6.8672798	6.7928944
AT3G61070	632.63441	-1.867592	0.1496356	-12.48093	9.49E-36	3.55E-33	8.1897325	7.9064072	8.0699823	9.7645358	9.9749482	10.135724
AT1G13920	40.314843	-1.861408	0.2857136	-6.514943	7.27E-11	2.98E-09	3.9565084	3.922692	4.0522736	6.3946946	5.9195455	5.8058471
AT3G33124	7.7193092	-1.85998	0.4432937	-4.195818	2.72E-05	0.0003811	0	0	0	5.2634978	2.9287097	1.7177495
AT1G22770	809.17684	-1.849217	0.1539929	-12.00846	3.21E-33	1.06E-30	8.1410757	8.49315	8.5719221	10.453151	10.260093	10.240449
AT1G04157	115.41116	-1.844237	0.1968978	-9.366467	7.50E-21	9.41E-19	5.6687059	5.673849	5.5116165	7.4215092	7.4205328	7.7016547
AT1G80920	9227.9419	-1.839787	0.1253561	-14.67648	9.12E-49	6.71E-46	12.06128	11.851469	11.925351	13.816753	13.667662	13.97231
AT3G03470	649.90094	-1.834326	0.1431625	-12.8129	1.39E-37	5.78E-35	8.1085455	8.1075036	8.1154378	10.206486	9.9867523	9.7814647
AT3G04070	30.973847	-1.830366	0.3141208	-5.826949	5.64E-09	1.72E-07	3.7642837	3.6785597	3.5548313	5.4117401	6.0331881	5.5122259
AT1G05147	50.075779	-1.829317	0.4522723	-4.044725	5.24E-05	6.77E-04	2.5463685	2.2562212	1.0532486	3.7517613	7.1948986	7.0636679
AT3G33154	9.8723351	-1.827694	0.44887	-4.071767	4.67E-05	0.0006128	0.5696486	0.5579473	0	5.669115	2.5754861	2.148558
AT5G57640	21.095763	-1.824549	0.3722163	-4.901851	9.49E-07	1.92E-05	3.4172774	2.7368381	2.8974698	4.9116189	4.7791107	5.650145
AT1G53100	22.178034	-1.820867	0.3650508	-4.987984	6.10E-07	1.28E-05	2.7676429	2.8355706	3.4164043	5.669115	5.2241406	4.710477
AT3G52310	15.430023	-1.818733	0.3902617	-4.660292	3.16E-06	5.65E-05	2.4215486	2.8355706	2.2519284	4.9116189	4.9431857	4.3546248
AT3G26740	11165.353	-1.813401	0.1553527	-11.67279	1.76E-31	5.10E-29	12.108865	12.053116	12.484367	14.155273	13.911794	14.209395
AT4G26288	287.38483	-1.812839	0.2204541	-8.223203	1.98E-16	1.72E-14	7.173047	6.8068484	6.7618138	8.3817769	8.7787236	9.2113183
AT2G39855	10.438976	-1.809771	0.433089	-4.17875	2.93E-05	4.08E-04	2.2848979	1.9384886	1.3855946	3.7517613	3.8307199	4.888274
AT4G30650	144.09404	-1.805603	0.2854255	-6.326003	2.52E-10	9.75E-09	5.4464632	5.8960167	6.0454746	8.4858118	7.2894272	7.5424714
AT5G24910	11.487089	-1.80535	0.4180402	-4.318604	1.57E-05	2.35E-04	2.4215486	2.1060843	1.6555327	4.1397727	4.7791107	4.0895494
AT4G39780	107.12473	-1.800939	0.2247625	-8.012632	1.12E-15	9.25E-14	5.7872795	5.1863811	5.5617383	7.1531516	7.5278411	7.5156503
AT3G45290	192.4473	-1.799597	0.226063	-7.960601	1.71E-15	1.39E-13	6.780526	6.1664337	5.9732405	8.1051601	8.2924378	8.3540958
AT5G44572	117.80082	-1.795536	0.2099512	-8.552156	1.21E-17	1.16E-15	5.9085191	5.5629435	5.4596906	7.6476919	7.3491717	7.6284362
AT2G45360	24.609649	-1.780864	0.3720755	-4.786299	1.70E-06	3.24E-05	3.9107996	2.8355706	2.7894342	4.9116189	5.707656	5.3993805
AT1G66060	8.2478828	-1.777961	0.4471555	-3.976158	7.00E-05	8.81E-04	2.1339363	0.5579473	1.3855946	3.7517613	3.8307199	4.2715627
AT5G54080	1910.2533	-1.777661	0.165505	-10.74083	6.55E-27	1.33E-24	9.9351757	9.4845053	9.6648008	11.454775	11.379869	11.768449
AT3G25770	130.76899	-1.7775	0.2694771	-6.596106	4.22E-11	1.81E-09	5.0841925	5.9739483	5.9855335	8.0832144	7.5786433	7.3932938
AT4G25480	17.546083	-1.77559	0.3839322	-4.62475	3.75E-06	6.59E-05	2.7676429	2.7368381	2.8974698	4.9116189	4.2622301	5.3597148

AT3G51000	1056.7645	-1.772066	0.1487796	-11.91067	1.04E-32	3.38E-30	8.9898253	8.8078313	8.7820749	10.866861	10.435712	10.729042
AT3G07105	30.403541	-1.771769	0.3548171	-4.993473	5.93E-07	1.25E-05	4.1655639	3.5680299	2.7894342	5.2634978	5.7525968	5.8917921
AT4G24050	206.82097	-1.771753	0.2405435	-7.365624	1.76E-13	1.11E-11	6.761338	6.2582871	6.3512009	7.8431732	8.2924378	8.7718135
AT3G33142	5.2174897	-1.767102	0.4466077	-3.956721	7.60E-05	0.0009436	0	0.5579473	0	4.4452387	2.9287097	2.148558
AT3G33181	8.0674693	-1.766667	0.4482791	-3.940998	8.11E-05	1.00E-03	0	0.9592852	0	5.2634978	2.9287097	2.148558
AT5G54960	261.66112	-1.766012	0.1541412	-11.45711	2.17E-30	5.63E-28	6.6819274	6.9404095	6.9617125	8.6590034	8.6539849	8.7100637
AT1G21670	492.51782	-1.756503	0.1521024	-11.54816	7.54E-31	2.06E-28	7.7416779	7.6793308	7.9338808	9.4239412	9.521966	9.7739396
AT4G34030	1791.3882	-1.755757	0.1641248	-10.69769	1.04E-26	2.08E-24	9.673862	9.6066153	9.6250829	11.100568	11.451468	11.719342
AT1G03090	3595.643	-1.753447	0.1665637	-10.52719	6.47E-26	1.25E-23	10.730433	10.587654	10.590864	12.12998	12.408408	12.748461
AT3G42057	7.4172676	-1.751749	0.4542457	-3.85639	1.15E-04	0.0013514	1.774338	0	1.0532486	4.4452387	3.4490907	3.4984464
AT5G41080	103.43356	-1.740896	0.32557	-5.347225	8.93E-08	2.24E-06	5.3470024	5.3456285	5.5116165	5.8874513	7.8088504	7.7561396
AT2G38465	32.248432	-1.739501	0.3092531	-5.624846	1.86E-08	5.18E-07	3.8635952	3.922692	3.4164043	5.9853613	5.6133433	5.5479613
AT2G39980	246.1879	-1.72016	0.1695901	-10.14305	3.56E-24	5.95E-22	6.987345	6.7446853	6.6960127	8.4519603	8.4977674	8.7604349
AT3G42100	5.8280072	-1.718923	0.4550974	-3.777044	1.59E-04	1.80E-03	0.5696486	0.5579473	1.3855946	4.1397727	2.9287097	3.3443906
AT2G38400	759.28227	-1.714943	0.1600717	-10.7136	8.79E-27	1.76E-24	8.6225627	8.189126	8.464044	10.016912	10.202545	10.353312
AT5G20630	672.77331	-1.707909	0.2881739	-5.926661	3.09E-09	9.94E-08	8.8454448	7.6251537	7.5469651	10.016912	9.8225776	10.295358
AT3G33537	8.2521076	-1.701062	0.4492257	-3.786653	1.53E-04	0.0017387	0	1.2729306	0	5.2634978	2.5754861	2.7491208
AT1G22490	32.445098	-1.699296	0.3147321	-5.399184	6.69E-08	1.71E-06	3.4812217	3.6785597	4.186117	5.8874513	5.5124312	5.7455674
AT1G60740	22.584521	-1.694553	0.344065	-4.925096	8.43E-07	1.73E-05	3.4172774	3.0967291	3.4164043	4.9116189	5.5637694	5.0956639
AT5G50450	138.56566	-1.691157	0.2193685	-7.709206	1.27E-14	9.29E-13	6.1440327	5.8376558	5.9732405	7.5550621	7.514856	8.1069947
AT3G05870	223.12234	-1.690953	0.1611696	-10.49176	9.43E-26	1.79E-23	6.6264497	6.6797225	6.7760374	8.4347319	8.277139	8.5606317
AT1G09420	148.19462	-1.679793	0.2166	-7.755278	8.82E-15	6.55E-13	6.06277	6.1376972	6.1364834	7.2743201	7.9602099	8.1483231
AT1G03100	228.36461	-1.67836	0.2001894	-8.383857	5.12E-17	4.67E-15	6.9928396	6.5764409	6.5463849	8.308004	8.3150859	8.7296459
AT3G33166	55.571379	-1.672591	0.4488981	-3.725992	1.95E-04	2.14E-03	2.1339363	2.7368381	1.3855946	7.9194695	6.0331881	4.1834245
AT1G21360	12.345127	-1.665927	0.4124169	-4.039425	5.36E-05	0.0006899	2.8667312	2.2562212	1.8828362	3.7517613	4.6894999	4.6459844
AT4G04330	242.34203	-1.664467	0.2222152	-7.490338	6.87E-14	4.61E-12	6.3887355	6.7698728	7.0810805	8.8659674	8.246046	8.4980657
AT4G36900	62.614517	-1.66403	0.3236578	-5.141326	2.73E-07	6.18E-06	4.3481248	5.2944746	4.186117	7.11039	6.496541	6.2364917
AT2G02120	7.425152	-1.659393	0.4551125	-3.646116	2.66E-04	2.80E-03	0.9769677	1.7488329	0	4.6971932	3.2122368	3.1718957
AT4G15610	117.46361	-1.651224	0.2263815	-7.293989	3.01E-13	1.83E-11	5.8491725	5.8136339	5.6570421	7.2350505	7.9015576	7.2915629
AT5G09440	397.70558	-1.648288	0.2899067	-5.68558	1.30E-08	3.73E-07	7.9816486	6.9514597	7.1753136	8.5188872	9.6008897	9.5074583
AT3G22640	8.1784249	-1.644382	0.4467611	-3.680674	2.33E-04	2.50E-03	0.5696486	1.9384886	2.0791536	3.219378	4.2622301	4.0895494
AT2G28110	76.817684	-1.643812	0.2298636	-7.151249	8.60E-13	4.86E-11	5.1836384	5.2944746	4.9590991	6.9739302	7.0403691	6.6846883
AT1G11362	4.3659203	-1.641166	0.443176	-3.703193	2.13E-04	0.0023225	0	0	0.620679	4.1397727	2.106732	2.7491208

AT1G08803	21.073265	-1.63957	0.3451045	-4.750938	2.02E-06	3.78E-05	3.128721	3.3845282	3.3418688	4.6971932	5.3464515	5.1891546
AT3G51430	281.56522	-1.637136	0.1686278	-9.70858	2.77E-22	3.85E-20	7.2892443	6.8249868	7.0285834	8.786708	8.7787236	8.6942042
AT4G39260	272.66239	-1.636766	0.3252597	-5.032182	4.85E-07	1.04E-05	5.8612387	7.6077975	6.4784249	8.9890964	8.8372686	8.3840621
AT5G07440	2838.8014	-1.634733	0.2260795	-7.230789	4.80E-13	2.84E-11	10.506262	10.216091	10.307864	11.402564	12.353396	12.340754
AT4G13540	31.996499	-1.631366	0.3430051	-4.756099	1.97E-06	3.70E-05	3.8635952	4.3137483	3.0919337	5.2634978	5.8384847	5.8917921
AT1G22990	22.655159	-1.629763	0.3769513	-4.323536	1.54E-05	2.30E-04	3.5424517	3.3177801	2.6726488	5.669115	4.3814822	5.3993805
AT1G06570	2390.4022	-1.628517	0.1432003	-11.3723	5.75E-30	1.44E-27	10.233791	10.032508	10.209764	11.690765	11.707585	12.05745
AT1G49000	23.380207	-1.627348	0.3827095	-4.252176	2.12E-05	3.06E-04	3.6576272	2.1060843	3.6193629	5.4117401	5.1588632	5.2769537
AT3G55450	211.48694	-1.627315	0.1607548	-10.12297	4.37E-24	7.25E-22	6.7677624	6.5906465	6.6270664	8.3268039	8.4508147	8.2275891
AT1G70300	254.75892	-1.626164	0.1834715	-8.863307	7.77E-19	8.33E-17	7.1681987	6.7884791	6.7182815	8.8136145	8.4778316	8.5252126
AT3G62950	255.08033	-1.625293	0.272361	-5.967424	2.41E-09	7.88E-08	7.3632303	6.4496882	6.5628862	8.126777	8.5935123	9.0336677
AT4G28040	445.72118	-1.620587	0.171624	-9.442658	3.63E-21	4.67E-19	7.7019741	7.5937606	7.9147238	9.2077062	9.3052974	9.6683113
AT1G07985	46.435109	-1.615751	0.2790833	-5.789493	7.06E-09	2.13E-07	4.3134141	4.5931436	4.5261657	5.7824096	6.1042628	6.5501893
AT1G73680	415.9539	-1.607139	0.1385559	-11.5992	4.16E-31	1.16E-28	7.6262184	7.7347485	7.6432179	9.2077062	9.2278405	9.4519519
AT5G48180	818.68983	-1.604409	0.2023056	-7.930623	2.18E-15	1.73E-13	8.309057	8.9633084	8.4552317	9.9994417	10.31356	10.506467
AT5G52120	65.408308	-1.604058	0.3021516	-5.308785	1.10E-07	2.72E-06	5.4303553	4.5355639	4.62377	6.0770468	6.9467821	6.8654991
AT1G13990	453.16158	-1.602502	0.1317223	-12.16576	4.73E-34	1.61E-31	7.8394577	7.7632475	7.7370884	9.5572792	9.4001486	9.3134751
AT2G07766	11.120649	-1.600418	0.4081567	-3.921088	8.82E-05	1.07E-03	2.1339363	2.392196	2.4062073	4.1397727	4.3814822	4.2715627
AT4G16690	138.21196	-1.600273	0.1978239	-8.089378	6.00E-16	5.07E-14	6.3028689	6.047885	5.7750472	7.7062978	7.7323866	7.7407813
AT5G48250	83.447626	-1.599454	0.2252972	-7.099308	1.25E-12	6.99E-11	5.042412	5.5629435	5.3113254	7.0208656	7.0221311	6.9876347
AT1G07430	51.60401	-1.594062	0.3823958	-4.168619	3.06E-05	0.0004237	3.4812217	4.8029102	4.4214759	7.1531516	5.2241406	6.2364917
AT5G05965	17.514624	-1.579162	0.3707127	-4.2598	2.05E-05	2.96E-04	3.3503668	2.7368381	2.9979758	4.4452387	4.8634794	5.1431664
AT1G04467	67.361378	-1.577287	0.2389277	-6.601525	4.07E-11	1.75E-09	4.9092797	4.9860128	5.0786748	7.0208656	6.4150185	6.6357088
AT3G62550	2802.5317	-1.575919	0.1455622	-10.82643	2.58E-27	5.37E-25	10.244194	10.456119	10.570607	11.932403	11.935408	12.246028
AT4G32340	377.87344	-1.574439	0.1650289	-9.540382	1.42E-21	1.87E-19	7.5350088	7.2885889	7.7261412	9.3245555	9.0725644	9.0771187
AT3G33127	5.0896552	-1.572497	0.4306943	-3.651074	2.61E-04	2.75E-03	0	0	0	4.6971932	2.106732	1.7177495
AT5G03240	4613.3734	-1.568626	0.1341636	-11.69189	1.40E-31	4.13E-29	11.231449	11.050889	11.182038	12.911352	12.537204	12.811611
AT3G13610	37.506169	-1.567094	0.3866317	-4.053195	5.05E-05	0.0006573	4.1261063	4.1314298	3.6193629	5.2634978	6.8258231	4.9956923
AT5G05880	13.790207	-1.56296	0.3946303	-3.960567	7.48E-05	0.0009311	2.7676429	2.392196	2.8974698	4.1397727	4.3814822	4.9429826
AT2G07793	11.210249	-1.559491	0.4076132	-3.82591	0.0001303	0.0015172	2.1339363	2.392196	2.5455702	4.1397727	4.3814822	4.2715627
AT4G27260	2184.6072	-1.552074	0.1707815	-9.088068	1.01E-19	1.16E-17	10.301773	9.8430297	10.049943	11.469654	11.61479	11.929518
AT2G39920	28.795575	-1.546573	0.3192412	-4.84453	1.27E-06	2.49E-05	3.8635952	3.8299356	3.4872774	5.8874513	5.2241406	5.3993805
AT2G15960	3756.0363	-1.546055	0.1875241	-8.244569	1.66E-16	1.45E-14	11.167293	10.620477	10.706307	12.271342	12.347457	12.740279

AT3G54830	91.661311	-1.542647	0.2144971	-7.191925	6.39E-13	3.70E-11	5.6959106	5.3950307	5.4058257	7.1946821	6.9467821	7.2268928
AT2G29670	1257.2735	-1.540699	0.1244857	-12.37652	3.50E-35	1.25E-32	9.2901413	9.22927	9.3956113	10.984008	10.71513	10.924375
AT1G20620	38707.713	-1.539041	0.1220114	-12.61391	1.77E-36	7.11E-34	14.380517	14.100756	14.264783	15.918275	15.699311	15.837051
AT1G58190	94.106466	-1.537454	0.2910043	-5.283268	1.27E-07	3.08E-06	6.0731817	5.2049685	4.9083485	6.9739302	7.1785237	7.3735134
AT1G10760	1855.9969	-1.534623	0.2054844	-7.468319	8.12E-14	5.39E-12	9.5838884	9.9673166	9.9045794	11.877675	11.149338	11.217142
AT5G59260	12.636431	-1.531619	0.4379427	-3.497305	4.70E-04	4.54E-03	3.128721	0.9592852	2.0791536	4.4452387	4.6894999	4.2715627
AT5G23050	485.88971	-1.530003	0.1333842	-11.47064	1.85E-30	4.87E-28	7.9593699	7.9729899	7.937049	9.3338789	9.5089277	9.6478725
AT1G29395	1463.4467	-1.529802	0.1547692	-9.88441	4.86E-23	7.16E-21	9.6959263	9.2190188	9.6112664	11.127624	10.989565	11.172767
AT5G39410	140.74742	-1.522895	0.1982046	-7.683452	1.55E-14	1.13E-12	6.3803752	5.8495183	6.1474662	7.7626156	7.7211247	7.7174329
AT5G59570	35.980898	-1.522685	0.3049259	-4.993623	5.93E-07	1.25E-05	4.085539	4.4446417	3.9047314	5.5461594	5.6612702	6.1461153
AT1G19540	213.95098	-1.522671	0.1650011	-9.228242	2.75E-20	3.28E-18	6.8846859	6.8309826	6.5546591	8.2889558	8.307576	8.3840621
AT2G21130	44.828608	-1.522288	0.2725512	-5.585328	2.33E-08	6.42E-07	4.683262	4.3476018	4.4572233	5.8874513	6.0331881	6.3622531
AT1G09421	43.407493	-1.521669	0.2838306	-5.361186	8.27E-08	2.08E-06	4.3134141	4.6485132	4.3848202	5.5461594	6.4150185	6.0987114
AT2G47190	20.629351	-1.521008	0.414191	-3.672239	2.40E-04	2.57E-03	2.959449	3.8299356	1.8828362	4.9116189	5.6133433	4.6459844
AT5G02580	76.374248	-1.51987	0.2687007	-5.656368	1.55E-08	4.35E-07	5.6823724	4.8973644	5.008125	6.6548684	6.8046392	7.112218
AT2G29660	71.509736	-1.517637	0.2240883	-6.772495	1.27E-11	5.81E-10	5.3297379	5.1291373	5.1014451	6.5940902	6.8258231	6.8512676
AT5G14470	38.824639	-1.51257	0.2965101	-5.101245	3.37E-07	7.50E-06	4.6278367	4.0920287	4.0522736	5.669115	5.8795843	6.1461153
AT5G63450	8.2804079	-1.508129	0.4367954	-3.452713	5.55E-04	5.26E-03	1.9653122	1.9384886	1.8828362	3.219378	4.4916251	3.6376272
AT1G52000	666.56154	-1.506576	0.1785869	-8.436095	3.28E-17	3.08E-15	8.3545808	8.6607377	8.132121	9.8120105	10.184208	9.8617492
AT1G11080	123.74016	-1.50419	0.2456509	-6.123281	9.17E-10	3.30E-08	5.6823724	6.3446409	5.6570421	7.5866041	7.7656542	7.2809839
AT3G51730	2420.1114	-1.499422	0.1390207	-10.7856	4.03E-27	8.31E-25	10.38666	10.114677	10.327916	11.61631	11.837117	11.964234
AT3G21090	78.887401	-1.498387	0.2872347	-5.216594	1.82E-07	4.27E-06	5.6549088	5.0073492	5.1459349	6.3946946	6.7391548	7.4128066
AT3G04060	31.519195	-1.495856	0.318857	-4.691308	2.71E-06	4.92E-05	3.9565084	3.9669257	3.6811312	6.163252	5.3464515	5.2769537
AT3G15635	173.59596	-1.494876	0.2198515	-6.79948	1.05E-11	4.90E-10	6.7088862	6.2582871	6.3979365	7.7626156	7.8507907	8.3840621
AT4G28703	39.740228	-1.493923	0.3010896	-4.961721	6.99E-07	1.46E-05	4.6558155	4.2071437	3.9556072	5.7824096	6.2366987	5.776022
AT5G63810	615.18634	-1.486366	0.173093	-8.587091	8.92E-18	8.64E-16	8.0726726	8.5193775	8.2965495	9.5572792	9.9218314	9.998789
AT5G26340	479.33185	-1.486024	0.172793	-8.600024	7.97E-18	7.79E-16	7.8666469	8.0623465	7.8756292	9.5169193	9.7131482	9.1660423
AT1G32560	5.8644186	-1.484568	0.454075	-3.269434	1.08E-03	9.28E-03	0.9769677	1.5304167	1.0532486	4.1397727	2.9287097	3.1718957
AT1G15330	19.777554	-1.480834	0.3728124	-3.972061	7.13E-05	8.93E-04	3.128721	3.2477933	3.5548313	4.1397727	4.8634794	5.5828329
AT5G54165	22.876612	-1.478378	0.3790811	-3.8999	9.62E-05	0.0011617	3.8635952	3.5094183	2.7894342	4.4452387	5.7961798	5.0465438
AT1G70850	111.71467	-1.478354	0.2804041	-5.272225	1.35E-07	3.26E-06	5.9429895	6.0984692	5.008125	7.7062978	7.3924105	7.1002328
AT3G13450	1028.3453	-1.472311	0.1975577	-7.452563	9.15E-14	6.05E-12	9.1385182	8.9189619	9.0585945	10.175706	10.474793	10.976938
AT5G54090	309.4586	-1.470786	0.1610471	-9.132641	6.68E-20	7.77E-18	7.5425215	7.2125501	7.2279995	8.786708	8.7732816	8.9626281

AT1G79440	478.42793	-1.470255	0.1435958	-10.23885	1.33E-24	2.31E-22	7.8636511	8.0418139	7.9959604	9.4239412	9.3277462	9.6354688
AT5G02020	863.34134	-1.469019	0.2015523	-7.288526	3.13E-13	1.89E-11	8.7018644	8.7910132	8.8339847	10.74286	9.8819344	10.244539
AT3G41345	10.343007	-1.462221	0.4409379	-3.31616	9.13E-04	8.07E-03	0.9769677	0.9592852	0	5.7824096	2.5754861	1.1007184
AT4G24040	120.05841	-1.460791	0.1940194	-7.529097	5.11E-14	3.50E-12	6.0522826	6.0166606	5.8709334	7.3860957	7.3637291	7.677659
AT5G18130	780.24707	-1.455429	0.1252083	-11.62406	3.11E-31	8.80E-29	8.8378322	8.6005893	8.6101356	10.154815	10.140493	10.20731
AT1G28230	44.293514	-1.449176	0.2792922	-5.188746	2.12E-07	4.91E-06	4.3134141	4.3137483	4.7444162	6.3946946	5.9195455	5.9193391
AT3G14280	30.858232	-1.447257	0.3512513	-4.120288	3.78E-05	5.11E-04	3.8635952	3.922692	3.6193629	6.3215962	5.3464515	4.8314086
AT1G58180	1186.429	-1.443359	0.1401024	-10.30218	6.89E-25	1.22E-22	9.4384505	9.3119769	9.1226072	10.630075	10.743237	10.920972
AT4G38580	161.5824	-1.442097	0.2089937	-6.900194	5.19E-12	2.58E-10	6.0937824	6.2671589	6.7402118	7.9681804	7.9311818	7.8378064
AT1G28330	3397.7007	-1.436486	0.2011289	-7.142113	9.19E-13	5.18E-11	10.968651	10.531692	10.822218	11.904516	12.215738	12.667774
AT1G56550	24.758412	-1.432674	0.3379078	-4.239838	2.24E-05	3.21E-04	3.8147938	3.8299356	3.1801448	5.4117401	5.0187179	5.3993805
AT1G18710	139.38558	-1.430543	0.1927485	-7.421811	1.16E-13	7.46E-12	6.4053121	6.1946089	5.9483358	7.789971	7.6516422	7.6450311
AT4G04620	249.94756	-1.428011	0.149395	-9.55863	1.19E-21	1.59E-19	6.9707344	7.0421185	7.1860058	8.4519603	8.5685951	8.5430308
AT4G26530	1886.1363	-1.424421	0.2227677	-6.394197	1.61E-10	6.39E-09	9.6849363	9.824367	10.218904	11.829053	10.935777	11.453354
AT5G01520	195.93379	-1.421888	0.1642197	-8.65845	4.78E-18	4.71E-16	6.6682567	6.686352	6.7182815	8.3996454	8.0439463	8.0767324
AT2G02100	727.82905	-1.415713	0.2097708	-6.748855	1.49E-11	6.78E-10	8.8424046	8.2214376	8.6061604	9.8644206	9.8512705	10.419917
AT1G58270	902.89991	-1.415102	0.1570535	-9.010321	2.05E-19	2.33E-17	8.9787919	8.8833422	8.8742083	10.465928	10.053374	10.551175
AT3G61900	16.60882	-1.408279	0.3986748	-3.532402	0.0004118	0.0040736	2.6612438	3.5094183	2.4062073	5.2634978	4.2622301	4.6459844
AT5G57240	251.41493	-1.406975	0.2207895	-6.372471	1.86E-10	7.28E-09	6.8905849	7.0421185	7.2071554	8.5188872	8.0708121	8.9086734
AT5G57630	395.98149	-1.406789	0.1907425	-7.375331	1.64E-13	1.04E-11	8.0305521	7.5325314	7.5552361	9.0240752	9.085796	9.4161437
AT5G55970	345.69867	-1.403613	0.1632507	-8.597899	8.12E-18	7.90E-16	7.5721874	7.4647369	7.6276151	8.6883751	9.0045226	9.2029366
AT4G17243	10.775883	-1.402982	0.4275059	-3.281785	1.03E-03	8.94E-03	2.8667312	1.5304167	2.2519284	4.4452387	4.2622301	3.8812119
AT2G14170	1219.7036	-1.400277	0.1410032	-9.930817	3.06E-23	4.66E-21	9.4202148	9.2717671	9.4150555	10.572165	10.796519	10.992421
AT2G18260	49.409003	-1.395982	0.2675998	-5.21668	1.82E-07	4.27E-06	4.7366366	4.621094	4.9083485	5.669115	6.496541	6.3215387
AT4G35750	2445.3146	-1.395058	0.1437406	-9.705385	2.86E-22	3.95E-20	10.489355	10.267287	10.354225	11.54384	11.861042	11.963408
AT1G77145	20.115325	-1.393925	0.3445054	-4.046163	5.21E-05	6.74E-04	3.5424517	3.3177801	3.3418688	4.9116189	5.0904916	4.9429826
AT3G29575	360.22004	-1.392675	0.1896835	-7.342099	2.10E-13	1.31E-11	7.5084016	7.8602479	7.3847234	8.9532484	8.8476625	9.2978183
AT3G63215	75.171275	-1.38979	0.22427	-6.196951	5.76E-10	2.14E-08	5.4781493	5.1675511	5.3113254	6.9739302	6.6469455	6.7928944
AT3G60690	150.15132	-1.388325	0.1799702	-7.714195	1.22E-14	8.96E-13	6.2670382	6.3362345	6.4162149	7.8431732	7.9115001	7.5946624
AT1G02620	34.071057	-1.38726	0.3908796	-3.549072	3.87E-04	3.86E-03	4.8131692	3.3845282	3.4164043	4.9116189	5.707656	6.3007423
AT1G77000	99.38862	-1.386738	0.1966423	-7.052087	1.76E-12	9.49E-11	5.8491725	5.673849	5.7608137	7.1531516	7.1452054	7.2809839
AT3G60140	31.661184	-1.385134	0.3816752	-3.62909	2.84E-04	0.0029649	3.6576272	4.5355639	3.4872774	4.4452387	6.3286119	5.5479613
AT1G67265	212.30243	-1.384996	0.1953461	-7.08996	1.34E-12	7.40E-11	7.1387614	6.7446853	6.6191983	8.0609297	8.3150859	8.4421897

AT1G26450	31.204477	-1.384608	0.3084084	-4.489529	7.14E-06	1.16E-04	4.1655639	4.0098434	3.7972589	5.8874513	5.5637694	5.2769537
AT3G47340	23807.313	-1.382603	0.1757822	-7.86543	3.68E-15	2.86E-13	13.737944	13.550219	13.649799	14.724898	15.040425	15.410731
AT2G19450	110.10297	-1.379659	0.1980134	-6.967504	3.23E-12	1.66E-10	5.7872795	5.9847455	6.0098096	7.0663221	7.4481174	7.4510579
AT1G65845	120.94328	-1.377858	0.3138102	-4.390736	1.13E-05	0.0001765	6.4697773	5.24144	5.8971983	6.7131893	7.861088	7.7016547
AT3G26210	270.50959	-1.377181	0.2099486	-6.559611	5.39E-11	2.26E-09	7.4812945	7.1077946	6.9429239	8.2500881	8.9134569	8.6537756
AT1G21680	2638.3841	-1.373881	0.1177587	-11.66691	1.88E-31	5.39E-29	10.514881	10.481594	10.513918	11.988087	11.718245	11.983529
AT3G15630	673.92377	-1.371563	0.2268448	-6.046264	1.48E-09	5.12E-08	8.7786246	8.2395816	8.408063	9.50871	9.8225776	10.382058
AT2G35170	61.814797	-1.368859	0.2406605	-5.687926	1.29E-08	3.69E-07	5.2401736	4.9200424	5.1238615	6.2445954	6.5737024	6.6846883
AT1G01420	98.369251	-1.353245	0.2257587	-5.99421	2.04E-09	6.78E-08	6.0731817	5.5484611	5.5452237	7.2350505	7.0761677	7.2268928
AT1G26665	302.20546	-1.351182	0.1959059	-6.897097	5.31E-12	2.63E-10	7.7053248	7.1753313	7.1699376	8.7453832	8.6300992	8.9559931
AT1G03620	31.177891	-1.350306	0.3025422	-4.4632	8.07E-06	0.0001296	4.043798	4.1697834	3.9556072	5.669115	5.707656	5.3189277
AT3G32980	325.76287	-1.341438	0.1904625	-7.043056	1.88E-12	1.01E-10	7.7898186	7.2754582	7.3084886	9.0805521	8.8631143	8.7100637
AT3G15620	104.46775	-1.336495	0.2274719	-5.875426	4.22E-09	1.31E-07	6.0522826	5.8495183	5.6874605	6.9739302	7.1110995	7.5946624
AT3G24420	40.048559	-1.334259	0.3616229	-3.689641	2.25E-04	2.43E-03	4.9550358	4.0098434	4.0047499	4.6971932	6.2047119	6.3821874
AT4G23870	697.09809	-1.334076	0.1937018	-6.887266	5.69E-12	2.80E-10	8.840882	8.3185188	8.5493269	10.016912	9.6492252	10.225355
AT3G28510	45.817606	-1.333206	0.404752	-3.293883	9.88E-04	0.0086267	4.043798	4.7782977	4.3472088	5.0982599	7.2110898	5.0956639
AT3G46970	673.3729	-1.332287	0.1985244	-6.710949	1.93E-11	8.66E-10	8.4151751	8.5451367	8.6041688	10.360396	9.5916437	9.7415127
AT5G57655	6293.0834	-1.331103	0.1656386	-8.036193	9.27E-16	7.72E-14	11.933177	11.690757	11.681053	12.841166	13.138316	13.413406
AT1G12240	2213.1972	-1.329217	0.1626133	-8.174097	2.98E-16	2.55E-14	10.179411	10.314591	10.315796	11.275268	11.730232	11.85391
AT3G06850	711.18985	-1.328792	0.2135215	-6.223221	4.87E-10	1.84E-08	8.6261028	8.5100651	8.7023937	9.4326457	10.046623	10.396831
AT1G69480	21.173654	-1.328149	0.3477786	-3.818951	0.000134	0.0015566	3.3503668	3.5680299	3.4872774	5.4117401	4.5939523	5.0465438
AT1G05340	131.69913	-1.328108	0.2199225	-6.038984	1.55E-09	5.32E-08	6.4459391	6.1083766	6.0217961	7.2350505	7.9015576	7.5335863
AT1G08050	51.465048	-1.320961	0.3897601	-3.389165	7.01E-04	6.44E-03	4.4475108	5.1095399	4.2689102	4.9116189	7.2586008	5.8058471
AT2G43400	670.64396	-1.320691	0.1612532	-8.190171	2.61E-16	2.24E-14	8.6662013	8.5286301	8.4880038	9.6866255	9.8199407	10.189037
AT3G47160	485.76409	-1.320374	0.142217	-9.284225	1.63E-20	1.98E-18	8.0251996	8.1344195	8.1595047	9.3151715	9.3680164	9.6354688
AT5G16370	1443.1408	-1.319072	0.1719554	-7.671012	1.71E-14	1.24E-12	9.8769379	9.467286	9.6003169	10.794422	10.941853	11.286214
AT2G19810	1086.4659	-1.318807	0.213086	-6.189084	6.05E-10	2.25E-08	9.4334081	9.005007	9.2142497	10.30932	10.36873	11.059724
AT1G22370	197.87817	-1.313716	0.1767154	-7.434076	1.05E-13	6.84E-12	6.5314847	6.912408	6.9366063	8.126777	8.1569148	8.1941497
AT3G22460	70.242347	-1.31363	0.2354044	-5.580312	2.40E-08	6.58E-07	5.3470024	5.3950307	5.2717211	6.163252	6.9075785	6.8368942
AT4G09500	29.755583	-1.312706	0.3269847	-4.014579	5.96E-05	7.58E-04	3.4172774	4.2790814	4.186117	5.5461594	5.5637694	5.3993805
AT3G47480	226.48828	-1.311706	0.3162121	-4.148186	3.35E-05	0.0004584	6.8427032	6.8007513	6.9985713	8.0153003	9.1668905	7.4698099
AT5G52450	143.34883	-1.303222	0.1874632	-6.95188	3.60E-12	1.84E-10	6.5539611	6.2130913	6.211663	7.789971	7.6277174	7.677659
AT4G23700	30.338172	-1.302101	0.3450917	-3.773204	0.0001612	0.001822	4.1655639	4.2790814	3.4872774	5.4117401	5.996293	5.0465438

AT3G59880	13.590817	-1.299744	0.3906991	-3.326714	8.79E-04	0.0078172	3.0465662	2.5164518	3.0919337	4.1397727	4.6894999	4.433164
AT4G13250	974.44441	-1.298882	0.1486941	-8.735262	2.43E-18	2.45E-16	9.0634778	9.0182051	9.2311256	10.693013	10.287074	10.336808
AT5G10860	2941.0409	-1.298835	0.1151711	-11.27744	1.70E-29	4.12E-27	10.778149	10.588096	10.780497	11.946147	12.019406	12.135863
AT1G30730	41.792197	-1.297242	0.3089563	-4.198788	2.68E-05	3.77E-04	4.6558155	4.701836	4.3085906	5.0982599	6.4698754	5.9729037
AT3G43850	104.45806	-1.295674	0.2728745	-4.748241	2.05E-06	3.82E-05	6.3549996	5.2944746	5.6415889	7.3497909	7.3344659	7.1002328
AT1G06460	583.24492	-1.292288	0.2153198	-6.001717	1.95E-09	6.52E-08	8.2321632	8.2145744	8.5431027	10.154815	9.278658	9.5954717
AT4G39090	8191.8908	-1.29158	0.146623	-8.808849	1.26E-18	1.31E-16	12.031723	12.106613	12.403646	13.426336	13.411344	13.692449
AT4G26080	897.14746	-1.287017	0.1748441	-7.360942	1.83E-13	1.15E-11	8.7897154	9.1760927	8.9701186	10.595609	10.042105	10.304528
AT1G62510	491.96177	-1.283498	0.145286	-8.834283	1.01E-18	1.06E-16	8.1161178	7.9566301	8.335487	9.4412981	9.4690905	9.4613525
AT1G49720	309.21394	-1.28339	0.1488032	-8.624747	6.42E-18	6.30E-16	7.4416647	7.4016785	7.5918794	8.7453832	8.6832965	8.9223524
AT3G12750	29.396708	-1.280869	0.3316481	-3.862133	0.0001124	0.0013278	4.043798	4.2435609	3.4872774	5.8874513	5.4039261	5.0956639
AT4G24230	1218.9803	-1.280171	0.1804007	-7.096266	1.28E-12	7.09E-11	9.6082563	9.3830745	9.3175358	10.44026	10.702301	11.088826
AT4G01870	954.10949	-1.279258	0.1802194	-7.098338	1.26E-12	7.02E-11	8.9380734	9.2706647	9.011282	10.587836	10.565742	10.038585
AT5G16340	147.98451	-1.27848	0.2072393	-6.169098	6.87E-10	2.53E-08	6.583395	6.2582871	6.3887097	7.5228149	7.5661095	8.0458217
AT1G17665	19.722333	-1.276563	0.3532064	-3.614214	0.0003013	0.0031096	3.6011885	3.2477933	3.3418688	5.2634978	4.8634794	4.6459844
AT5G64572	588.27697	-1.275841	0.1998535	-6.383881	1.73E-10	6.81E-09	8.7818021	8.1295629	8.1238035	9.8120105	9.5761007	9.7720522
AT5G64570	1008.0886	-1.270436	0.2002143	-6.34538	2.22E-10	8.64E-09	9.5857077	8.8673865	8.91012	10.52817	10.391996	10.56759
AT3G07650	109.6944	-1.268748	0.2338316	-5.425907	5.77E-08	1.49E-06	6.1538761	6.0166606	5.7608137	7.0208656	7.1110995	7.685702
AT1G68500	18.536556	-1.261254	0.3577131	-3.525882	4.22E-04	4.16E-03	3.128721	3.3177801	3.5548313	5.0982599	4.7791107	4.6459844
AT3G61160	151.9362	-1.259965	0.2407719	-5.233022	1.67E-07	3.94E-06	6.8787626	5.9847455	6.2529269	7.8690562	7.7323866	7.7407813
AT3G09390	3062.0986	-1.258155	0.125429	-10.03082	1.12E-23	1.81E-21	10.788059	10.731216	10.876859	12.223237	11.893384	12.117771
AT5G49015	42.143799	-1.254888	0.2930896	-4.281587	1.86E-05	2.72E-04	4.6558155	4.2790814	4.8557473	5.669115	5.7961798	6.3007423
AT3G20470	44.895138	-1.254681	0.3638642	-3.448211	0.0005643	0.0053382	4.4791742	4.9200424	3.9556072	6.7131893	6.1385289	4.9956923
AT4G16870	90.192453	-1.25396	0.2727455	-4.597545	4.27E-06	7.39E-05	5.7357793	5.577282	5.6570421	7.0208656	7.501753	6.40185
AT3G02040	350.73549	-1.250471	0.2296249	-5.445713	5.16E-08	1.35E-06	7.7803181	7.5724458	7.5998976	8.4689853	8.8002886	9.4636932
AT4G11910	15.696069	-1.248858	0.3757093	-3.324002	0.0008874	0.0078899	3.2802015	2.9279768	3.0919337	4.9116189	4.2622301	4.6459844
AT2G39800	2808.6214	-1.248564	0.2562387	-4.872661	1.10E-06	2.20E-05	10.189615	10.980823	10.565498	12.519287	11.582483	11.651151
AT5G63160	1691.932	-1.248391	0.2079274	-6.003977	1.93E-09	6.44E-08	10.369338	9.6980243	9.5546376	11.175082	11.157716	11.374692
AT1G62515	319.95621	-1.246502	0.1656507	-7.524883	5.28E-14	3.61E-12	7.5045602	7.3144971	7.7800652	8.8136145	8.8733244	8.8017248
AT5G59220	98.343878	-1.246362	0.3110262	-4.007257	6.14E-05	0.000779	5.4623933	6.0984692	5.4772077	7.8690562	6.5227227	6.8223762
AT3G51400	54.101628	-1.246358	0.2414854	-5.161215	2.45E-07	5.62E-06	4.9550358	4.9643561	4.9838203	6.3946946	6.2047119	6.3007423
AT5G58350	223.81339	-1.245348	0.1781699	-6.989666	2.76E-12	1.44E-10	7.0934478	7.1753313	6.7402118	8.4689853	8.277139	8.2109663
AT1G21100	212.66479	-1.245179	0.2284933	-5.449521	5.05E-08	1.33E-06	7.173047	6.7255017	6.9174857	7.6476919	8.3737984	8.5649985

AT1G06980	75.541588	-1.244582	0.2613429	-4.762257	1.91E-06	3.61E-05	5.3297379	5.1095399	5.6723315	7.2350505	6.496541	6.5324599
AT2G21060	36.654492	-1.243416	0.3148719	-3.948959	7.85E-05	9.72E-04	4.203971	4.8271098	3.9047314	5.7824096	5.8795843	5.650145
AT4G09020	241.84153	-1.24178	0.1862512	-6.667235	2.61E-11	1.16E-09	6.7741583	7.2577626	7.3084886	8.5188872	8.3737984	8.3988151
AT4G19160	2092.8358	-1.240726	0.1813028	-6.843391	7.73E-12	3.69E-10	10.461884	10.129956	10.1234	11.25578	11.432605	11.856138
AT3G11690	260.1049	-1.239402	0.190305	-6.512716	7.38E-11	3.02E-09	7.5611341	6.9894822	7.1206306	8.4858118	8.4711247	8.6372815
AT5G57887	177.09259	-1.238581	0.2294667	-5.397652	6.75E-08	1.73E-06	6.9818294	6.3107171	6.6270664	8.126777	7.6156045	8.1714184
AT4G36450	30.546897	-1.237918	0.3340037	-3.706301	0.0002103	0.0022999	4.5101575	3.624353	4.0982816	5.4117401	5.2241406	5.835068
AT1G18270	1245.9355	-1.237056	0.1645373	-7.518392	5.55E-14	3.78E-12	9.5535354	9.5459408	9.4364816	10.396438	10.943065	10.9463
AT2G04690	806.92874	-1.234552	0.1321962	-9.338788	9.74E-21	1.21E-18	8.9578741	8.7552016	8.9793681	9.9935709	10.212632	10.237716
AT4G19850	96.53905	-1.229844	0.2046735	-6.008808	1.87E-09	6.31E-08	5.7357793	5.8729549	5.9101537	7.0208656	6.9849484	7.2915629
AT1G08890	276.00592	-1.228445	0.1620685	-7.579789	3.46E-14	2.42E-12	7.2438821	7.3357375	7.4571312	8.6440903	8.4022823	8.7642378
AT4G37608	68.402451	-1.227601	0.2587925	-4.743573	2.10E-06	3.89E-05	5.5983615	5.1863811	5.2309989	6.0770468	6.6705562	6.961373
AT2G28840	1056.241	-1.225744	0.1383588	-8.859172	8.06E-19	8.60E-17	9.3243205	9.1959742	9.3187509	10.753321	10.350573	10.497357
AT5G57110	288.24224	-1.225722	0.1511475	-8.109439	5.09E-16	4.32E-14	7.4091579	7.5688625	7.2587115	8.6290213	8.6716432	8.7100637
AT5G07100	145.12919	-1.22268	0.200916	-6.085527	1.16E-09	4.10E-08	6.5906606	6.4021533	6.2930433	7.3497909	7.9697576	7.669571
AT2G39705	129.86906	-1.220317	0.2069508	-5.896655	3.71E-09	1.17E-07	6.3549996	6.1569183	6.2427211	7.6174713	7.1785237	7.7484809
AT1G60190	67.847412	-1.218287	0.3352224	-3.634265	2.79E-04	0.0029129	4.9550358	5.6055384	4.8011554	7.3860957	6.2366987	5.9729037
AT3G25760	37.148874	-1.213702	0.2956888	-4.104659	4.05E-05	5.42E-04	4.4475108	4.5058886	4.4572233	5.2634978	6.2047119	5.650145
AT3G63210	243.9034	-1.211025	0.1831309	-6.612888	3.77E-11	1.63E-09	7.2937031	6.9236739	7.2383096	8.6590034	8.1817745	8.432663
AT1G55510	339.50425	-1.207004	0.1850301	-6.523286	6.88E-11	2.83E-09	7.6918751	7.6216992	7.6890385	8.4858118	8.9085009	9.2141014
AT1G19550	123.75407	-1.199158	0.2097125	-5.718106	1.08E-08	3.14E-07	6.2487836	6.2582871	5.9732405	7.8168173	7.2271013	7.3020649
AT1G17710	22.253269	-1.198198	0.3622232	-3.307901	9.40E-04	0.0082751	3.6576272	3.6785597	3.4164043	5.5461594	5.2241406	4.2715627
AT1G28050	86.975424	-1.197127	0.2278488	-5.25404	1.49E-07	3.56E-06	5.5544473	5.9847455	5.4596906	7.0208656	6.8672798	6.9876347
AT5G06690	741.44524	-1.194983	0.1488063	-8.030463	9.71E-16	8.06E-14	8.7865553	8.6857672	8.9245573	9.87084	9.9242887	10.237716
AT5G45830	116.25566	-1.194688	0.2301291	-5.19138	2.09E-07	4.85E-06	6.2850648	5.9739483	6.0802791	7.2350505	7.0403691	7.7330404
AT1G17860	89.405907	-1.192623	0.2229273	-5.34983	8.80E-08	2.21E-06	5.8850726	5.6871314	5.5780659	7.0663221	7.1785237	6.7164413
AT1G64660	2153.6287	-1.192466	0.1904752	-6.260478	3.84E-10	1.46E-08	10.059513	10.402773	10.46592	11.210944	11.515613	11.8896
AT2G22450	343.85769	-1.191912	0.1450034	-8.219891	2.04E-16	1.76E-14	7.7643436	7.5937606	7.7297995	8.9040219	8.7841451	9.0399557
AT2G46270	234.55418	-1.191109	0.178186	-6.684638	2.31E-11	1.03E-09	7.2392665	7.2171356	6.9045963	8.4858118	8.1316192	8.4703982
AT3G15500	231.56764	-1.190395	0.2758395	-4.315535	1.59E-05	0.0002375	7.4734552	7.1274156	6.4162149	7.7626156	8.5368311	8.6578698
AT5G43840	27.580152	-1.189518	0.3158171	-3.766476	1.66E-04	1.86E-03	4.043798	4.0920287	4.0522736	5.0982599	5.1588632	5.6826588
AT1G17460	64.177047	-1.189495	0.2324679	-5.116815	3.11E-07	6.96E-06	5.2401736	5.4111291	5.2101985	6.2445954	6.6705562	6.5850078
AT4G36930	43.662799	-1.182993	0.2633603	-4.491918	7.06E-06	0.0001155	4.6278367	4.7532581	4.6853538	6.163252	5.8384847	5.94637

AT2G29630	1505.8817	-1.182654	0.1418264	-8.338744	7.51E-17	6.77E-15	9.8100382	9.8221112	9.8408853	11.228546	10.770807	11.08655
AT1G23060	79.371887	-1.181984	0.2329672	-5.073608	3.90E-07	8.56E-06	5.3297379	5.7002926	5.7319184	6.5306385	6.8046392	7.0512707
AT2G22660	1113.9793	-1.177615	0.1234707	-9.537614	1.46E-21	1.91E-19	9.3297582	9.4556911	9.4047942	10.746355	10.447719	10.599869
AT3G02030	168.48179	-1.177369	0.2360829	-4.987101	6.13E-07	1.29E-05	6.7741583	6.5548667	6.6504165	7.5228149	7.6156045	8.3791108
AT1G79360	105.95735	-1.176126	0.3195858	-3.680159	2.33E-04	2.51E-03	6.1241421	5.7002926	5.9732405	6.0770468	7.1785237	7.9354769
AT4G34000	623.01876	-1.175931	0.1737402	-6.768326	1.30E-11	5.97E-10	8.5386472	8.70385	8.3569362	10.073665	9.5635447	9.6313104
AT2G39710	39.522913	-1.173946	0.3480868	-3.372567	7.45E-04	6.79E-03	4.4475108	3.8770591	4.8557473	6.2445954	6.1042628	5.0465438
AT3G51330	89.144503	-1.17376	0.2632586	-4.458583	8.25E-06	1.32E-04	6.1925915	5.5041206	5.3113254	6.9254163	7.1282532	6.9210618
AT2G22080	198.46912	-1.170539	0.1860547	-6.291369	3.15E-10	1.20E-08	6.7868657	7.0677336	6.8518556	8.1690626	7.8507907	8.3131472
AT3G17790	232.66522	-1.165583	0.2179021	-5.349115	8.84E-08	2.22E-06	7.0466646	7.3525075	6.8652226	8.7453832	7.9886655	8.2602708
AT5G64260	1203.982	-1.165045	0.1954504	-5.960821	2.51E-09	8.19E-08	9.6959263	9.1354883	9.6112664	10.360396	10.926001	10.810375
AT1G71030	1567.4838	-1.164822	0.1362585	-8.548614	1.25E-17	1.20E-15	9.9904671	9.6939178	9.9909537	11.18542	10.95393	11.126245
AT4G16750	27.776088	-1.163999	0.3093281	-3.762992	1.68E-04	1.89E-03	4.085539	3.922692	4.0982816	5.5461594	5.2865918	5.233722
AT2G25450	2697.7222	-1.163361	0.1022468	-11.37797	5.38E-30	1.37E-27	10.76817	10.688513	10.611831	11.912317	11.893384	11.80352
AT3G48390	235.66947	-1.162173	0.2443395	-4.756387	1.97E-06	3.70E-05	7.5198648	6.9404095	6.87186	7.9919327	8.2302446	8.7868467
AT2G02930	100.13411	-1.158971	0.2722261	-4.257386	2.07E-05	2.99E-04	6.3719661	5.7517687	5.3113254	7.2743201	7.2271013	6.9745636
AT5G25110	47.433441	-1.157639	0.2657489	-4.356139	1.32E-05	0.000202	4.6558155	4.8029102	4.9590991	6.3215962	6.1385289	5.8058471
AT1G19570	1258.1578	-1.156856	0.1231183	-9.3963	5.65E-21	7.13E-19	9.5609526	9.5837165	9.6201636	10.914246	10.592255	10.790934
AT1G63720	50.384465	-1.156229	0.3020392	-3.828077	1.29E-04	0.0015047	4.6558155	5.0490981	5.008125	5.7824096	6.7391548	5.8058471
AT2G15060	43.222159	-1.155866	0.3192008	-3.621126	2.93E-04	3.05E-03	4.3820199	5.0283747	4.3472088	6.3215962	6.1385289	5.3597148
AT5G45310	116.77282	-1.154076	0.1923262	-6.000615	1.97E-09	6.55E-08	6.114093	6.027144	6.2930433	7.4898305	7.2110898	7.3735134
AT1G55810	347.74791	-1.153017	0.171409	-6.726702	1.74E-11	7.84E-10	7.9021201	7.5616691	7.7443407	8.6137933	9.0945501	8.9985851
AT5G66170	50.299896	-1.151434	0.2615966	-4.401565	1.07E-05	1.69E-04	4.9092797	5.1095399	4.6548905	6.3946946	6.1042628	6.0245506
AT1G77210	931.84779	-1.149934	0.2163107	-5.316121	1.06E-07	2.62E-06	9.361961	8.7863923	9.1945289	9.9578353	10.248373	10.731958
AT3G05880	1648.075	-1.147234	0.1912005	-6.00016	1.97E-09	6.55E-08	9.6216183	9.9075754	10.28939	11.124942	10.961128	11.381517
AT3G26512	624.33513	-1.14536	0.1735012	-6.601453	4.07E-11	1.75E-09	8.669636	8.4074723	8.6627547	9.4326457	9.7606735	10.024384
AT3G27210	70.802826	-1.144796	0.2294043	-4.9903	6.03E-07	1.27E-05	5.3122644	5.442797	5.4419581	6.9739302	6.4427077	6.5145098
AT5G38200	111.9563	-1.143446	0.1954345	-5.850786	4.89E-09	1.50E-07	5.920101	6.2222444	6.1799216	7.1946821	7.3924105	7.2809839
AT1G02470	31.840364	-1.140733	0.3009918	-3.789915	0.0001507	0.001724	4.2778476	4.0098434	4.4921063	5.5461594	5.6133433	5.4379847
AT1G52200	165.58853	-1.139823	0.2619105	-4.351956	1.35E-05	2.05E-04	6.6335022	6.4957067	6.783097	7.3125489	8.4508147	7.5774743
AT3G26220	521.58603	-1.136523	0.1880293	-6.044393	1.50E-09	5.17E-08	8.6610338	8.2259951	7.9929191	9.3796127	9.5540554	9.567452
AT3G14050	544.63705	-1.133985	0.1781385	-6.36575	1.94E-10	7.59E-09	8.5517361	8.3564247	8.2539934	9.2477168	9.8589978	9.5097253
AT1G28260	597.85181	-1.133547	0.1275122	-8.889718	6.13E-19	6.63E-17	8.6011379	8.4798552	8.5326689	9.6719966	9.5761007	9.8111786

AT1G62360	24.011456	-1.13262	0.3338559	-3.392541	6.92E-04	6.37E-03	3.8635952	3.922692	4.0047499	4.4452387	5.1588632	5.5479613
AT2G22980	1504.0217	-1.132137	0.1553811	-7.286198	3.19E-13	1.92E-11	9.9507874	9.8976116	9.7283552	10.739356	11.032337	11.249381
AT1G53180	34.551234	-1.128417	0.3012065	-3.746322	1.79E-04	2.00E-03	4.415137	4.5931436	4.0522736	5.8874513	5.3464515	5.6826588
AT4G20820	36.451741	-1.127612	0.311977	-3.614406	0.000301	0.0031096	4.8378037	4.2071437	4.2689102	5.5461594	6.0691631	5.4755828
AT4G08305	56.940644	-1.126853	0.3048897	-3.695937	2.19E-04	2.38E-03	4.5701966	5.069528	5.5617383	5.8874513	6.2986201	6.7164413
AT1G06180	44.57532	-1.124683	0.2717504	-4.138664	3.49E-05	4.76E-04	4.8858456	4.8743242	4.4572233	6.163252	5.996293	5.835068
AT2G01940	180.53403	-1.120259	0.2284349	-4.904064	9.39E-07	1.91E-05	7.0985535	6.8129199	6.3700772	7.9440305	7.7211247	8.2275891
AT1G19510	27.034984	-1.116835	0.3331951	-3.351895	0.0008026	0.0072555	4.3481248	4.0920287	3.7972589	4.4452387	5.6612702	5.4755828
AT2G45560	206.6648	-1.116041	0.1846609	-6.043734	1.51E-09	5.18E-08	6.9023111	6.8953424	7.1317342	8.4858118	7.9792425	8.0014029
AT1G78070	285.29044	-1.115058	0.2229294	-5.001843	5.68E-07	1.20E-05	7.3589816	7.4414118	7.4790331	9.1455296	8.2222785	8.3939142
AT3G10985	739.55831	-1.114367	0.2351665	-4.738629	2.15E-06	3.98E-05	9.2169078	8.3953846	8.7153684	9.7366892	9.806683	10.390694
AT1G21000	819.54162	-1.114345	0.1266039	-8.801821	1.35E-18	1.39E-16	9.0855028	8.9768917	8.9670222	9.9758138	10.142605	10.274178
AT4G02005	64.19547	-1.113055	0.2568117	-4.334129	1.46E-05	0.0002201	5.6687059	5.2049685	5.008125	6.3215962	6.5737024	6.5850078
AT4G34138	513.01887	-1.110464	0.1544831	-7.188253	6.56E-13	3.78E-11	8.3068529	8.5044488	8.1567896	9.3614928	9.3788069	9.6354688
AT1G16110	103.22623	-1.108688	0.2399428	-4.620633	3.83E-06	6.71E-05	5.6269122	6.068332	6.2831183	6.7692438	7.3781411	7.2809839
AT1G71910	27.487822	-1.108533	0.3376123	-3.28345	1.03E-03	0.0088962	4.1261063	3.922692	3.8519957	5.9853613	4.7791107	5.0465438
AT3G05165	214.20755	-1.108029	0.1833131	-6.044461	1.50E-09	5.17E-08	7.0037665	7.2798484	6.8315699	8.3453619	8.2538823	8.0395593
AT2G45720	128.88803	-1.107326	0.1996381	-5.54667	2.91E-08	7.90E-07	6.5009609	6.2038797	6.3512009	7.1531516	7.514856	7.7016547
AT5G10946	154.90355	-1.10725	0.205976	-5.375624	7.63E-08	1.93E-06	6.8057194	6.6528956	6.2427211	7.8168173	7.5661095	7.8592997
AT2G38820	412.11162	-1.106203	0.1747533	-6.330083	2.45E-10	9.51E-09	8.2205015	7.9092437	7.8953089	8.9040219	9.1032514	9.3942242
AT3G17810	552.09682	-1.10282	0.1212377	-9.096339	9.34E-20	1.08E-17	8.4334742	8.4114791	8.4706181	9.6719966	9.4623427	9.5630926
AT4G27460	29.01533	-1.102578	0.3040483	-3.626325	0.0002875	0.0029926	4.043798	4.1697834	4.3472088	5.2634978	5.4039261	5.4755828
AT5G11610	113.81345	-1.101439	0.1892743	-5.819277	5.91E-09	1.80E-07	6.1925915	6.047885	6.1799216	7.4898305	7.2271013	7.2046755
AT1G05347	93.602041	-1.100715	0.2190832	-5.024187	5.06E-07	1.08E-05	6.0095537	5.8136339	5.7319184	7.11039	7.2110898	6.7475104
AT1G10090	353.38286	-1.100123	0.1440151	-7.638937	2.19E-14	1.56E-12	7.9649719	7.7218992	7.7002699	8.9040219	8.9085009	8.9593144
AT4G03510	814.93428	-1.099908	0.2009278	-5.474148	4.40E-08	1.17E-06	9.0582462	8.8229522	9.0687405	9.6793296	10.09323	10.512132
AT1G20696	1137.1697	-1.099774	0.1416402	-7.764562	8.19E-15	6.11E-13	9.3384161	9.5586429	9.523361	10.814542	10.433989	10.557763
AT5G53730	28.198282	-1.099649	0.307796	-3.572654	0.0003534	0.0035758	4.0008133	4.0920287	4.2281074	5.5461594	5.2865918	5.233722
AT1G08230	142.39007	-1.095457	0.2224218	-4.925133	8.43E-07	1.73E-05	6.818153	6.2847403	6.211663	7.7347315	7.4065402	7.7330404
AT1G55110	185.21329	-1.095068	0.1921633	-5.698631	1.21E-08	3.49E-07	7.0883239	6.8068484	6.7182815	7.6476919	8.1569148	8.1189241
AT2G39350	70.925673	-1.093394	0.2547443	-4.292122	1.77E-05	2.61E-04	5.3470024	5.3950307	5.7172508	6.3215962	6.4427077	7.0134269
AT2G47890	114.40032	-1.093178	0.189071	-5.781839	7.39E-09	2.23E-07	6.0522826	6.2130913	6.2220902	7.3860957	7.3637291	7.1821107
AT5G46180	622.48907	-1.092324	0.1129303	-9.672546	3.94E-22	5.34E-20	8.5975359	8.633552	8.6550793	9.7082944	9.7045964	9.7852125

AT1G46768	74.606685	-1.090336	0.2330162	-4.679228	2.88E-06	5.19E-05	5.5983615	5.3456285	5.7024326	6.7131893	6.4698754	6.8935479
AT2G21560	86.212534	-1.089704	0.2068574	-5.267899	1.38E-07	3.32E-06	5.824733	5.8014713	5.6259685	7.0208656	6.8875699	6.8223762
AT5G00365	402.61518	-1.089529	0.1565452	-6.959836	3.41E-12	1.75E-10	8.175307	7.7601086	8.0200631	9.0240752	9.1204982	9.1774952
AT2G16365	268.95627	-1.087082	0.1431476	-7.594132	3.10E-14	2.18E-12	7.4616157	7.4374874	7.3471095	8.5512213	8.4371141	8.5909257
AT3G61490	69.097617	-1.086854	0.226385	-4.800908	1.58E-06	3.04E-05	5.6127075	5.4270499	5.3113254	6.5306385	6.5484377	6.6846883
AT1G73330	218.90268	-1.08598	0.1593596	-6.814648	9.45E-12	4.46E-10	7.234636	7.1225353	6.9554768	8.3268039	8.1981128	8.1828288
AT4G34139	215.3624	-1.083069	0.1821205	-5.946992	2.73E-09	8.85E-08	7.0728423	7.3144971	6.8784669	8.0382954	8.1899668	8.3741423
AT3G49790	708.4132	-1.082793	0.190871	-5.672906	1.40E-08	3.98E-07	9.1310628	8.5917882	8.6101356	9.7082944	9.8894996	10.147419
AT4G11330	266.34089	-1.082733	0.1565138	-6.917812	4.59E-12	2.30E-10	7.5611341	7.3357375	7.2688053	8.5984029	8.5303935	8.4182539
AT3G48530	1351.8713	-1.079714	0.1336341	-8.079634	6.50E-16	5.47E-14	9.8635039	9.6623208	9.7219406	10.667429	10.845312	11.011745
AT3G03870	828.71895	-1.07958	0.1583471	-6.817806	9.24E-12	4.37E-10	9.2845519	8.8169228	8.9670222	10.090264	10.271718	10.072719
AT1G07390	49.48082	-1.079516	0.263811	-4.092005	4.28E-05	0.0005686	4.8620246	5.0896727	4.8287095	6.3215962	6.2366987	5.8058471
AT1G01470	436.80707	-1.077757	0.2008704	-5.365434	8.08E-08	2.04E-06	8.1873382	8.0418139	8.0140751	9.6646261	9.0412104	8.8913902
AT2G09400	46.786029	-1.076603	0.2855045	-3.770879	1.63E-04	1.84E-03	4.8378037	4.621094	5.008125	6.4642672	5.5124312	6.0744123
AT4G04410	126.14194	-1.075272	0.2621416	-4.101873	4.10E-05	5.47E-04	6.8057194	5.8612842	6.0454746	7.2743201	7.434391	7.6367575
AT2G46680	306.27075	-1.073738	0.2414879	-4.446342	8.73E-06	0.0001394	7.2438821	7.9007175	7.4393671	9.1975278	8.4093163	8.4610565
AT1G22500	219.0956	-1.071635	0.2140508	-5.006452	5.54E-07	1.18E-05	7.3332236	6.818966	7.1753136	7.789971	8.5109065	8.3027258
AT5G57880	215.77464	-1.065221	0.220427	-4.832534	1.35E-06	2.63E-05	7.3842879	6.7319246	7.0695785	8.2889558	7.8814647	8.4278759
AT2G40420	440.16705	-1.062227	0.1543336	-6.882667	5.87E-12	2.88E-10	8.3068529	7.9456196	8.1649196	9.058226	9.2748119	9.3315292
AT3G55500	296.18566	-1.062175	0.242912	-4.372674	1.23E-05	1.89E-04	7.0780213	7.6216992	7.7587368	9.1349012	8.4778316	8.3027258
AT4G20860	189.99349	-1.061581	0.2256883	-4.703748	2.55E-06	4.66E-05	6.8608456	7.1322794	6.7329387	7.6174713	8.4508147	7.8874681
AT3G02525	35.043264	-1.061293	0.2990866	-3.548448	3.88E-04	3.86E-03	4.3134141	4.4446417	4.4921063	5.9853613	5.707656	5.1891546
AT2G35070	60.773479	-1.061069	0.2365538	-4.485531	7.27E-06	0.0001184	5.1446787	5.442797	5.2101985	6.4642672	6.4150185	6.3420395
AT1G21110	95.669064	-1.060998	0.2263726	-4.686956	2.77E-06	5.02E-05	6.1830096	5.6469116	5.9608419	6.7692438	7.2271013	7.0881471
AT3G45730	606.13864	-1.059701	0.1755721	-6.035703	1.58E-09	5.42E-08	8.6541149	8.4798552	8.6454274	9.5572792	10.014694	9.4401143
AT2G43830	42.047427	-1.058694	0.2779864	-3.808438	1.40E-04	1.62E-03	4.8131692	4.7782977	4.8011554	5.2634978	5.9584295	6.2144228
AT5G52250	96.732568	-1.057427	0.2820675	-3.748843	0.0001777	0.001985	6.4776366	5.458374	5.610177	6.8232017	7.1785237	7.1934372
AT5G11090	463.63443	-1.0571	0.1544977	-6.842172	7.80E-12	3.71E-10	8.3694417	7.9891663	8.2841628	9.1665543	9.3277462	9.3966762
AT3G15450	16486.047	-1.056798	0.1847099	-5.721397	1.06E-08	3.09E-07	13.504673	13.148768	13.386449	14.145709	14.340941	14.80536
AT4G38060	170.58769	-1.054759	0.1880033	-5.610323	2.02E-08	5.58E-07	6.987345	6.757334	6.5042865	7.9681804	7.861088	7.7863742
AT4G11360	304.52839	-1.053957	0.1807377	-5.831415	5.50E-09	1.68E-07	7.8179504	7.3566697	7.5998976	8.786708	8.4711247	8.8237582
AT1G18870	30.802753	-1.046885	0.2981636	-3.511109	4.46E-04	4.35E-03	4.1655639	4.3137483	4.3472088	5.669115	5.3464515	5.3597148
AT3G55880	70.108227	-1.043653	0.231855	-4.501315	6.75E-06	0.0001112	5.5544473	5.5914794	5.2515037	6.7692438	6.6229419	6.4212482

AT5G27920	91.710807	-1.040932	0.225313	-4.619937	3.84E-06	6.72E-05	6.0731817	5.5914794	5.9977226	6.7131893	7.1452054	7.0261521
AT5G47860	469.17929	-1.039708	0.1430958	-7.265817	3.71E-13	2.22E-11	8.2321632	8.1844504	8.2916075	9.50871	9.3052974	9.116327
AT5G43440	439.97365	-1.039285	0.1437113	-7.231752	4.77E-13	2.83E-11	8.0700758	8.054681	8.3210083	9.2962184	9.1075825	9.2552165
AT5G23575	350.288	-1.037552	0.1305849	-7.945421	1.94E-15	1.55E-13	7.8636511	7.8155898	7.8354458	8.8136145	8.971637	8.8738974
AT4G15490	200.25059	-1.037148	0.1864975	-5.561192	2.68E-08	7.29E-07	6.7484027	7.0317438	7.1860058	8.2889558	8.0796573	7.9082392
AT3G47800	668.72964	-1.034595	0.141121	-7.331266	2.28E-13	1.42E-11	8.866549	8.652297	8.7855942	9.6347618	9.806683	9.9906965
AT4G14270	1160.9163	-1.031827	0.1632094	-6.322105	2.58E-10	9.94E-09	9.4364357	9.4845053	9.7274406	10.544324	10.393771	10.873351
AT4G16190	4003.7953	-1.030015	0.1117589	-9.2164	3.07E-20	3.65E-18	11.320175	11.292731	11.436599	12.475731	12.262127	12.446899
AT3G01310	1105.8456	-1.029115	0.1384055	-7.435505	1.04E-13	6.78E-12	9.321048	9.5523058	9.5812062	10.652603	10.363307	10.605179
AT4G09560	84.818726	-1.027173	0.2246705	-4.57191	4.83E-06	8.20E-05	5.8850726	5.4737846	5.8971983	7.0208656	6.8672798	6.7164413
AT4G30662	95.453818	-1.025864	0.275103	-3.729017	1.92E-04	2.12E-03	5.3122644	5.9074109	6.3887097	6.8232017	7.0583794	7.2158269
AT3G28220	413.12268	-1.024444	0.1746839	-5.864557	4.50E-09	1.38E-07	7.8023894	8.2145744	8.132121	9.2477168	9.2397275	8.88092
AT5G40240	83.308273	-1.024049	0.2357627	-4.343559	1.40E-05	0.0002117	5.7998723	5.6469116	5.7464384	6.9739302	7.0761677	6.4212482
AT4G36040	3021.3771	-1.022562	0.1538635	-6.645908	3.01E-11	1.33E-09	11.102377	10.631684	11.046698	11.912317	11.952965	12.108078
AT4G11600	445.0727	-1.022387	0.1582061	-6.462377	1.03E-10	4.17E-09	8.2251775	8.1050316	8.1676195	9.5169193	9.0091599	9.1457784
AT5G16380	66.292307	-1.022101	0.2228927	-4.585619	4.53E-06	7.77E-05	5.3809236	5.442797	5.4772077	6.5306385	6.5484377	6.4592793
AT2G27310	54.435869	-1.019593	0.2879121	-3.541335	3.98E-04	0.0039558	4.9993853	5.3787505	4.8011554	6.5940902	6.3579929	5.7144559
AT2G46690	192.33536	-1.016312	0.1886757	-5.386555	7.18E-08	1.83E-06	7.182695	6.7319246	6.9554768	8.1051601	7.8299729	8.1366353
AT5G60553	77.915682	-1.011157	0.2397108	-4.218236	2.46E-05	0.000351	5.4781493	6.0061005	5.4596906	6.6548684	6.7831397	6.7779245
AT2G18170	286.99007	-1.011134	0.1552731	-6.511972	7.42E-11	3.03E-09	7.6155729	7.4570037	7.5511065	8.800224	8.5811075	8.4085672
AT1G54290	92.877101	-1.01083	0.2143777	-4.715183	2.41E-06	4.42E-05	5.9429895	5.776834	5.9732405	7.2743201	6.6937867	6.9745636
AT5G66053	130.24409	-1.009874	0.2551137	-3.958524	7.54E-05	9.38E-04	6.7994621	5.8136339	6.4162149	7.2743201	7.514856	7.6200665
AT3G44880	574.47907	-1.009646	0.1876047	-5.38177	7.38E-08	1.87E-06	8.2980029	8.4741197	8.7978449	9.8962356	9.4657205	9.4040074
AT5G42440	75.481356	-1.008334	0.2626306	-3.839362	1.23E-04	0.0014418	5.8850726	5.5338318	5.2916592	6.8232017	6.9467821	6.2796418
AT5G02140	45.250147	-1.00714	0.2896179	-3.477477	5.06E-04	0.0048483	5.2585368	4.621094	4.62377	6.0770468	5.7525968	6.0987114
AT2G09660	46.575136	-1.003836	0.2968476	-3.381654	7.21E-04	6.61E-03	5.0634535	4.9200424	4.933947	5.0982599	6.0331881	6.5145098
AT3G06500	285.05498	-1.003097	0.1739901	-5.765253	8.15E-09	2.43E-07	7.6918751	7.6182363	7.3035874	8.5671205	8.7568314	8.4230729
AT3G23430	84.167951	-1.002424	0.22128	-4.530116	5.90E-06	9.86E-05	5.5395071	5.9739483	5.7891416	6.9739302	6.7166491	6.8512676
AT2G15830	53.256532	-1.000023	0.2555723	-3.912877	9.12E-05	1.11E-03	5.1446787	5.0283747	5.0555394	6.5940902	6.0691631	5.9193391
AT3G26180	617.34197	-0.998315	0.1305092	-7.649384	2.02E-14	1.45E-12	8.599338	8.7329599	8.691179	9.6572178	9.8356906	9.5739666
AT4G38470	1204.7349	-0.99773	0.189352	-5.269181	1.37E-07	3.30E-06	9.6695799	9.5890332	9.6211488	10.144255	10.830977	10.898656
AT4G35790	1047.1653	-0.997146	0.1122343	-8.884503	6.42E-19	6.92E-17	9.4985894	9.4479092	9.3594651	10.552333	10.40261	10.404161
AT2G27150	236.6835	-0.995506	0.2018904	-4.930923	8.18E-07	1.69E-05	7.2392665	7.2666375	7.2485466	8.731341	8.2302446	7.9287155

AT4G37610	650.79851	-0.994329	0.2192107	-4.535948	5.73E-06	9.62E-05	9.1447017	8.4779459	8.4552317	9.4584487	9.8013457	10.024384
AT1G70820	277.95933	-0.991806	0.2825388	-3.510334	4.48E-04	4.36E-03	6.7548849	7.7757353	7.6315317	9.0805521	8.3665887	8.1129718
AT4G27950	92.032056	-0.986794	0.1994587	-4.947358	7.52E-07	1.56E-05	5.9315906	5.9299327	5.9229937	7.0663221	6.8258231	7.0005885
AT1G20693	1636.2212	-0.984836	0.140423	-7.013348	2.33E-12	1.24E-10	9.8761948	10.134811	10.228633	11.056195	10.987217	11.228886
AT4G13530	664.40201	-0.984635	0.1134726	-8.677293	4.05E-18	4.03E-16	8.7333885	8.8379162	8.8168881	9.7506797	9.7852142	9.8366855
AT5G51070	2260.7167	-0.984029	0.118896	-8.276383	1.27E-16	1.13E-14	10.560922	10.568044	10.551096	11.398126	11.717537	11.538533
AT5G26570	806.03124	-0.983776	0.1613869	-6.095762	1.09E-09	3.86E-08	8.8843958	9.1901551	9.0716263	10.332761	9.8486855	10.016434
AT4G10120	155.79455	-0.983567	0.1844512	-5.332398	9.69E-08	2.41E-06	6.4618749	6.8605934	6.7034739	7.8168173	7.6983337	7.6367575
AT1G69260	74.189311	-0.983542	0.2527328	-3.891627	9.96E-05	0.0011935	5.4781493	5.7390709	5.4419581	7.1531516	6.2679916	6.4963335
AT5G66790	154.19105	-0.983531	0.2132528	-4.612046	3.99E-06	6.97E-05	6.9197236	6.2759764	6.7474485	7.6772924	7.5910692	7.8305703
AT2G42890	889.71162	-0.983233	0.1169492	-8.407357	4.19E-17	3.86E-15	9.2562754	9.2132921	9.1932046	10.062491	10.248373	10.30844
AT4G33490	325.34767	-0.981992	0.1522976	-6.447849	1.13E-10	4.57E-09	7.8576407	7.6077975	7.8010828	8.8136145	8.6119217	8.8668404
AT1G22930	1568.2198	-0.981462	0.1442609	-6.803383	1.02E-11	4.79E-10	9.8997848	10.266734	9.8776255	11.030622	11.099109	10.967072
AT1G05835	148.89841	-0.9812	0.2241781	-4.376876	1.20E-05	1.86E-04	6.780526	6.5906465	6.5546591	7.11039	7.6277174	8.0395593
AT1G56220	8297.3024	-0.980073	0.1591496	-6.158187	7.36E-10	2.70E-08	12.555456	12.202518	12.496766	13.252882	13.337903	13.684067
AT2G31980	75.779016	-0.97727	0.2751478	-3.551801	3.83E-04	3.83E-03	6.0731817	5.2049685	5.424005	6.8232017	6.8258231	6.4403891
AT5G57900	217.36897	-0.976281	0.1611334	-6.058836	1.37E-09	4.76E-08	7.2065366	7.1515724	7.1753136	8.2889558	7.9697576	8.2869512
AT5G65110	852.31113	-0.976194	0.1267153	-7.70384	1.32E-14	9.65E-13	9.1679603	9.1607021	9.1610499	9.9578353	10.161475	10.299295
AT3G28290	290.63909	-0.97542	0.222659	-4.380781	1.18E-05	1.83E-04	7.1681987	7.737943	7.7261412	8.9164869	8.1981128	8.6578698
AT3G28300	290.72869	-0.974059	0.2228272	-4.371363	1.23E-05	1.90E-04	7.1681987	7.737943	7.7297995	8.9164869	8.1981128	8.6578698
AT1G03470	62.887083	-0.972405	0.2357325	-4.125038	3.71E-05	0.0005012	5.5395071	5.3117282	5.4058257	6.0770468	6.5985322	6.4963335
AT3G48690	916.62369	-0.971511	0.1524136	-6.374178	1.84E-10	7.23E-09	9.3714833	9.2029263	9.1865645	10.342031	9.9773168	10.415087
AT1G62810	198.52316	-0.969937	0.163217	-5.942625	2.80E-09	9.05E-08	7.182695	7.0265283	6.9924932	7.8690562	8.1817745	8.0767324
AT1G70560	65.485774	-0.968848	0.2581944	-3.752398	1.75E-04	0.00196	5.6409784	5.1291373	5.5617383	6.3215962	6.2679916	6.7779245
AT3G13790	147.76169	-0.968714	0.1978725	-4.89565	9.80E-07	1.98E-05	6.6544552	6.7823036	6.5042865	7.1946821	7.798172	7.7938349
AT5G57910	106.73908	-0.967944	0.2156821	-4.487828	7.20E-06	1.17E-04	6.2209611	6.3192731	5.9855335	6.8232017	7.1452054	7.4415898
AT3G49110	180.83914	-0.967242	0.2009467	-4.813425	1.48E-06	2.87E-05	7.0728423	6.9948331	6.7182815	7.4898305	8.0165706	8.1541316
AT5G52190	185.46414	-0.967115	0.222559	-4.34543	1.39E-05	0.0002104	6.9707344	6.8780725	7.0521516	7.2743201	8.1316192	8.2656465
AT3G26510	1239.6862	-0.966788	0.1768192	-5.467663	4.56E-08	1.21E-06	9.8308961	9.5201961	9.7118026	10.360396	10.655739	10.989175
AT3G18830	432.66686	-0.96636	0.1979279	-4.882385	1.05E-06	2.10E-05	8.1336336	8.1050316	8.2891302	8.800224	9.0457315	9.567452
AT1G80315	45.131859	-0.96179	0.2886953	-3.331505	8.64E-04	7.71E-03	4.6278367	4.6754209	5.2717211	6.163252	5.707656	5.9989583
AT3G47860	167.14578	-0.961752	0.2117305	-4.542341	5.56E-06	9.35E-05	6.8668427	6.6528956	6.8383635	8.0382954	7.3344659	7.9819395
AT4G20110	137.88257	-0.960112	0.2241401	-4.283538	1.84E-05	0.0002697	6.2760797	6.7884791	6.4162149	7.6174713	7.75465	7.2046755

AT5G17860	139.69451	-0.959139	0.1875175	-5.114929	3.14E-07	7.01E-06	6.3549996	6.6797225	6.5546591	7.7347315	7.501753	7.4224649
AT1G06560	255.27258	-0.957331	0.1822449	-5.252992	1.50E-07	3.57E-06	7.6507567	7.3315144	7.2788289	8.2696528	8.2924378	8.6372815
AT4G23170	69.872914	-0.956545	0.2614299	-3.658896	2.53E-04	2.68E-03	5.7357793	5.4111291	5.4772077	6.3215962	7.0036595	6.258228
AT3G29035	199.8317	-0.956184	0.1585743	-6.029879	1.64E-09	5.57E-08	7.128814	6.9569533	7.1038131	8.1897496	8.0073288	7.9884565
AT3G27250	57.331755	-0.95527	0.2845862	-3.3567	0.0007888	0.0071389	5.0634535	5.328778	5.1890938	6.8232017	5.6133433	6.2796418
AT3G26290	341.03558	-0.953309	0.2419869	-3.939506	8.16E-05	0.0010066	7.6402914	7.9648332	7.7623135	9.3975079	8.5811075	8.3591336
AT1G05753	83.612543	-0.951597	0.2345995	-4.056262	4.99E-05	0.0006495	6.0522826	5.5629435	5.8306171	6.5306385	7.0761677	6.7779245
AT1G75780	107.33261	-0.950864	0.2334714	-4.072719	4.65E-05	6.11E-04	5.7745758	6.4021533	6.3029005	6.9254163	7.1619607	7.3834375
AT5G49360	9783.1033	-0.950773	0.1455426	-6.532609	6.46E-11	2.66E-09	12.915198	12.536934	12.579811	13.495473	13.75256	13.727568
AT4G27820	71.04816	-0.950402	0.2711923	-3.504532	4.57E-04	4.45E-03	5.2215736	5.776834	5.4772077	7.11039	6.3579929	6.2364917
AT1G02400	89.378319	-0.949376	0.2114158	-4.49056	7.10E-06	1.16E-04	6.0835188	5.8014713	5.8709334	6.875214	6.7831397	7.0512707
AT4G16000	118.48839	-0.948709	0.1868117	-5.078424	3.81E-07	8.35E-06	6.4053121	6.2671589	6.3700772	7.0663221	7.3924105	7.4128066
AT4G29190	1531.9513	-0.945686	0.1977796	-4.781512	1.74E-06	3.31E-05	10.221557	9.7621861	9.9909537	10.728793	10.797859	11.366585
AT3G19580	142.61	-0.942433	0.1866174	-5.050081	4.42E-07	9.61E-06	6.5239141	6.686352	6.512805	7.7347315	7.6516422	7.33312
AT3G16380	77.356657	-0.940259	0.2266227	-4.149008	3.34E-05	0.0004576	5.7745758	5.8136339	5.5452237	6.7131893	6.4698754	6.8935479
AT3G22121	435.48009	-0.939683	0.1680663	-5.591147	2.26E-08	6.23E-07	8.2665931	8.054681	8.2360988	9.4837982	9.0366751	8.9725238
AT5G20250	7090.7478	-0.938463	0.2102093	-4.464419	8.03E-06	0.0001292	12.359244	12.116618	12.146408	12.608417	13.353486	13.503183
AT3G22120	435.64148	-0.938101	0.1682631	-5.575206	2.47E-08	6.76E-07	8.2711223	8.054681	8.2360988	9.4837982	9.0366751	8.9725238
AT4G36670	390.54907	-0.933735	0.2101614	-4.442944	8.87E-06	0.0001416	8.2063819	7.8425502	8.0814811	8.4858118	9.0901797	9.3442881
AT1G22640	447.89455	-0.930732	0.194116	-4.794719	1.63E-06	3.12E-05	8.5348856	8.0183599	8.132121	9.1349012	8.9905204	9.4753396
AT2G17730	45.39008	-0.930274	0.2625422	-3.54333	3.95E-04	3.93E-03	5.1446787	4.8029102	4.933947	5.7824096	6.0331881	5.9989583
AT2G23840	175.87363	-0.926128	0.2215549	-4.18013	2.91E-05	4.06E-04	6.605083	6.9624259	7.0106512	8.2696528	7.4617144	7.8159882
AT5G56100	301.37964	-0.925767	0.2154363	-4.297175	1.73E-05	0.0002558	7.9981346	7.3144971	7.6470923	8.2889558	8.7120244	8.8668404
AT5G39610	327.67265	-0.925321	0.1574284	-5.877727	4.16E-09	1.29E-07	7.9537461	7.6592521	7.7800652	8.8659674	8.5748649	8.8273979
AT1G69890	303.78836	-0.924286	0.2168899	-4.261547	2.03E-05	2.94E-04	7.8903923	7.6251537	7.5469651	8.0832144	9.0045226	8.7218449
AT1G67070	50.788084	-0.921217	0.2619575	-3.516666	0.000437	0.0042826	5.1247971	5.2049685	5.1014451	5.669115	6.4698754	6.0245506
AT3G10410	483.56948	-0.919622	0.1455124	-6.319888	2.62E-10	1.01E-08	8.1777213	8.5119324	8.3942981	9.4326457	9.2436681	9.2740097
AT2G32150	1800.6825	-0.919601	0.1422458	-6.464871	1.01E-10	4.11E-09	10.25738	10.124474	10.417405	11.019108	11.203956	11.381517
AT1G30260	62.958912	-0.917602	0.2458525	-3.732325	1.90E-04	2.10E-03	5.6127075	5.1484721	5.4596906	6.5306385	6.2047119	6.4779254
AT4G31650	110.66478	-0.916675	0.2242696	-4.087379	4.36E-05	5.78E-04	5.9655205	6.4881382	6.2630611	7.0208656	7.0761677	7.4604644
AT3G53800	264.37503	-0.9163	0.1793194	-5.109878	3.22E-07	7.17E-06	7.2484831	7.6251537	7.5634598	8.6590034	8.2142682	8.4610565
AT4G02370	338.61542	-0.915299	0.1554514	-5.888007	3.91E-09	1.23E-07	7.9926602	7.6524966	7.9243341	8.800224	8.7120244	8.8879085
AT5G06860	244.07644	-0.915265	0.1555931	-5.88243	4.04E-09	1.26E-07	7.5462633	7.2666375	7.3613299	8.2696528	8.3881107	8.3286394

AT2G23450	400.21748	-0.914785	0.16947	-5.397914	6.74E-08	1.72E-06	8.2552075	7.9918448	8.0289989	9.0355488	9.2670887	8.7980196
AT5G28050	592.83628	-0.912694	0.1832337	-4.981038	6.32E-07	1.32E-05	8.6798914	8.5970753	8.7373427	9.1560802	9.6727989	9.8950825
AT2G33150	2476.0072	-0.912582	0.1226464	-7.440755	1.00E-13	6.60E-12	10.54137	10.842986	10.806714	11.639052	11.701868	11.654229
AT3G26580	833.46512	-0.912373	0.1316292	-6.931388	4.17E-12	2.10E-10	9.0503631	9.3012194	9.1392082	10.062491	10.000791	10.214277
AT3G13270	53.464904	-0.912289	0.2449656	-3.724153	0.000196	0.002157	5.3122644	5.2233195	5.0786748	6.0770468	6.2986201	6.122608
AT2G26695	60.58955	-0.912204	0.2376924	-3.83775	1.24E-04	1.45E-03	5.2766692	5.2944746	5.4945147	6.5306385	6.3579929	6.1692457
AT5G06505	106.88761	-0.910956	0.2097585	-4.342878	1.41E-05	2.12E-04	6.2670382	5.9187157	6.3794235	7.1531516	7.0583794	7.2378745
AT5G58650	104.77461	-0.909917	0.2117873	-4.296373	1.74E-05	0.0002564	6.3204559	5.8845319	6.2630611	7.11039	7.242937	7.0134269
AT5G41810	102.50953	-0.909665	0.2156645	-4.217963	2.47E-05	3.51E-04	6.3204559	5.9630696	6.0802791	7.2350505	6.8046392	7.2158269
AT4G30660	185.29043	-0.909563	0.2706393	-3.360794	7.77E-04	0.0070499	6.3028689	6.9067419	7.4571312	7.7347315	7.8404194	8.2330877
AT5G19860	561.12004	-0.908466	0.1429836	-6.353636	2.10E-10	8.20E-09	8.5591624	8.4645096	8.7282273	9.7011075	9.3966135	9.4753396
AT2G40000	3561.3208	-0.907631	0.1558431	-5.824007	5.75E-09	1.75E-07	11.473951	11.286745	10.965634	12.04738	12.251403	12.265276
AT4G05150	2064.3803	-0.907418	0.1509045	-6.013191	1.82E-09	6.16E-08	10.475685	10.24669	10.661041	11.465418	11.218045	11.513898
AT3G18080	1428.1033	-0.906524	0.1699238	-5.334884	9.56E-08	2.38E-06	9.895391	9.946066	9.9484129	11.228546	10.680662	10.650059
AT3G24518	103.1416	-0.905561	0.2522504	-3.589931	0.0003308	0.0033826	6.0203552	6.2934513	5.9229937	7.6174713	6.9659915	6.6357088
AT4G37580	54.164193	-0.90506	0.2452071	-3.691004	0.0002234	0.0024187	5.3809236	5.2233195	5.1238615	5.9853613	6.2047119	6.3215387
AT2G13360	13488.26	-0.903154	0.1303445	-6.928978	4.24E-12	2.13E-10	13.17334	13.062852	13.311071	14.078662	13.955097	14.272752
AT5G24490	5484.4561	-0.902868	0.1322769	-6.825589	8.76E-12	4.15E-10	11.76093	11.876506	12.015593	12.758764	12.670154	12.982535
AT5G11150	102.15032	-0.902696	0.2009839	-4.491387	7.08E-06	1.16E-04	6.0835188	6.0581447	6.2930433	7.0663221	6.9467821	7.2158269
AT1G12080	310.52583	-0.902526	0.1617273	-5.58054	2.40E-08	6.58E-07	7.8516051	7.7538103	7.5998976	8.800224	8.4371141	8.7680306
AT1G22070	158.54868	-0.90073	0.1893847	-4.756089	1.97E-06	3.70E-05	6.6613724	6.6324429	6.9554768	7.9440305	7.514856	7.669571
AT4G37870	2811.8215	-0.899472	0.1228996	-7.318754	2.50E-13	1.54E-11	10.981836	10.90256	10.902612	11.780441	11.710435	12.02136
AT4G11320	160.01295	-0.898525	0.2249376	-3.994551	6.48E-05	8.19E-04	6.6682567	7.0472781	6.5212736	7.8690562	7.9311818	7.3433248
AT5G10650	201.57473	-0.898423	0.1689733	-5.316954	1.06E-07	2.61E-06	7.2392665	7.0677336	7.0285834	8.1480747	8.1485321	7.8592997
AT2G02710	1479.3715	-0.896729	0.1500879	-5.974692	2.31E-09	7.59E-08	10.205675	9.891887	9.8776255	10.863647	10.777617	11.097895
AT5G64430	541.55086	-0.895113	0.1378584	-6.49299	8.41E-11	3.43E-09	8.443541	8.4664368	8.730055	9.5250821	9.4315808	9.4543078
AT1G12010	105.41577	-0.894315	0.2537385	-3.524552	0.0004242	0.0041831	6.4053121	5.633252	6.3224152	7.3497909	6.8258231	7.2046755
AT2G18050	2136.1487	-0.893474	0.1322658	-6.75514	1.43E-11	6.51E-10	10.42171	10.539036	10.63494	11.45691	11.265331	11.598339
AT1G53870	77.860973	-0.893258	0.2543377	-3.512093	4.45E-04	4.34E-03	6.0310764	5.4111291	5.610177	7.0663221	6.4698754	6.5501893
AT1G53890	77.860973	-0.893258	0.2543377	-3.512093	4.45E-04	0.0043396	6.0310764	5.4111291	5.610177	7.0663221	6.4698754	6.5501893
AT1G80310	127.62821	-0.891265	0.2413723	-3.692493	2.22E-04	2.41E-03	6.0835188	6.3695703	6.7618138	7.7347315	7.0403691	7.3932938
AT5G24460	141.53569	-0.89097	0.1935463	-4.603394	4.16E-06	7.22E-05	6.5239141	6.4181729	6.8585546	7.6174713	7.5278411	7.4974886
AT1G04433	111.32997	-0.886876	0.2351071	-3.772223	1.62E-04	1.83E-03	6.4053121	6.1664337	6.3126908	6.6548684	7.1452054	7.6116481

AT3G24520	101.14945	-0.885788	0.248225	-3.56849	3.59E-04	3.62E-03	5.9655205	6.3107171	5.9229937	7.5550621	6.9273135	6.6522207
AT3G07280	238.14961	-0.885492	0.1746521	-5.070032	3.98E-07	8.69E-06	7.3375486	7.6043011	7.1482312	8.2302543	8.2616763	8.3641539
AT2G43535	61.401992	-0.885239	0.2594923	-3.411426	0.0006462	0.0060052	5.5983615	5.458374	5.1676757	6.3215962	6.0331881	6.6522207
AT5G13870	77.128408	-0.884302	0.2234847	-3.95688	7.59E-05	0.0009435	5.7617592	5.5338318	5.9483358	6.5940902	6.6937867	6.7320595
AT4G01610	885.39592	-0.883679	0.1201335	-7.35581	1.90E-13	1.19E-11	9.2528447	9.3800106	9.173192	10.236622	10.086663	10.193274
AT3G10740	1293.0948	-0.883603	0.1102325	-8.015817	1.09E-15	9.05E-14	9.7496545	9.8694869	9.8391928	10.756791	10.61525	10.775944
AT4G21980	334.76986	-0.88209	0.1555882	-5.669389	1.43E-08	4.05E-07	8.0117306	7.7024082	7.8756292	8.731341	8.9134569	8.6660236
AT3G53530	210.99089	-0.881801	0.1690358	-5.216653	1.82E-07	4.27E-06	7.3288855	7.0778536	7.2279995	7.9681804	8.0348786	8.2922286
AT1G48210	165.18907	-0.881681	0.1899264	-4.642224	3.45E-06	6.12E-05	7.0466646	6.5835612	6.87186	7.7347315	7.8713123	7.677659
AT1G22250	76.194606	-0.879359	0.2646186	-3.323121	8.90E-04	0.0079027	5.954299	5.328778	5.7891416	6.7692438	6.2366987	6.9210618
AT2G00600	55.122274	-0.878694	0.2695579	-3.25976	0.0011151	0.0095573	5.2585368	5.5629435	4.8557473	6.163252	6.3579929	6.0987114
AT1G10210	173.58613	-0.878356	0.1980757	-4.434447	9.23E-06	0.0001469	7.192279	6.6730624	6.8784669	7.6174713	7.8814647	7.9622099
AT5G65210	137.59377	-0.87768	0.2110442	-4.158749	3.20E-05	0.0004405	6.7155479	6.5255899	6.4071046	7.7626156	7.5910692	7.1241044
AT5G39030	157.3679	-0.875577	0.1930653	-4.535135	5.76E-06	9.65E-05	6.9539304	6.5548667	6.7901222	7.7062978	7.8507907	7.506598
AT1G03290	435.56326	-0.874772	0.1379749	-6.340084	2.30E-10	8.93E-09	8.2711223	8.320651	8.1969891	8.965297	9.3015218	9.1457784
AT3G16460	745.80076	-0.87323	0.1659394	-5.262342	1.42E-07	3.42E-06	9.2180811	9.0634701	8.7714649	9.6866255	9.991447	10.065032
AT2G26650	162.44683	-0.87272	0.2128892	-4.099409	4.14E-05	5.52E-04	7.1778791	6.6255604	6.6112871	7.5866041	7.7323866	7.8734526
AT2G27830	531.40023	-0.872292	0.1894429	-4.604514	4.13E-06	7.19E-05	8.7802142	8.1148942	8.6041688	9.5731122	9.3090632	9.4353519
AT3G26170	280.15326	-0.871339	0.1718486	-5.070391	3.97E-07	8.69E-06	7.6986156	7.6286001	7.5344691	8.3268039	8.7949275	8.3691568
AT3G62650	1683.6712	-0.870575	0.1358034	-6.410557	1.45E-10	5.76E-09	10.22856	10.0286	10.347692	11.010413	11.024342	11.237803
AT4G02410	130.23786	-0.868127	0.2054221	-4.226065	2.38E-05	3.40E-04	6.605083	6.4340166	6.4432054	7.5228149	7.6277174	7.0759594
AT4G27130	702.26371	-0.867421	0.1706075	-5.084309	3.69E-07	8.11E-06	9.1667455	8.6489069	8.9670222	9.7366892	9.7440781	10.011643
AT5G66052	522.90139	-0.865062	0.235462	-3.673894	0.0002389	0.0025541	8.9034839	7.9702761	8.4771623	9.124194	9.3823858	9.7004227
AT5G03700	50.908517	-0.863226	0.2597832	-3.322869	8.91E-04	7.91E-03	5.2027307	5.1095399	5.2916592	5.669115	6.0331881	6.40185
AT2G30860	2065.9684	-0.861287	0.1182789	-7.281832	3.29E-13	1.98E-11	10.437914	10.663532	10.41343	11.393675	11.430877	11.331772
AT1G19660	2051.9597	-0.858857	0.1065408	-8.061302	7.55E-16	6.34E-14	10.459902	10.544975	10.510201	11.313472	11.313931	11.484808
AT4G02520	2663.61	-0.858757	0.2170782	-3.95598	7.62E-05	9.46E-04	11.317988	10.680649	10.400864	11.587367	11.883947	11.807672
AT2G36390	450.1011	-0.856	0.1433725	-5.970462	2.37E-09	7.76E-08	8.1968915	8.3163834	8.405778	9.3057261	9.0045226	9.2498017
AT2G23080	360.26941	-0.855589	0.1498935	-5.707979	1.14E-08	3.32E-07	8.1656092	7.8978641	7.9179343	8.7730642	8.8320434	8.9856133
AT2G38530	335.88404	-0.854225	0.1670403	-5.113889	3.16E-07	7.05E-06	7.9649719	7.7973321	7.882219	8.9164869	8.4576165	8.9017849
AT1G73750	163.62849	-0.853704	0.1855131	-4.601852	4.19E-06	7.26E-05	6.8427032	6.6324429	7.0106512	7.9440305	7.6156045	7.6614373
AT2G25730	554.73329	-0.852628	0.1736503	-4.91003	9.11E-07	1.86E-05	8.3503066	8.8703007	8.5534616	9.5169193	9.6252598	9.3263939
AT3G46640	274.19182	-0.850841	0.1568749	-5.423692	5.84E-08	1.51E-06	7.465573	7.6043011	7.7297995	8.4858118	8.3373841	8.5909257

AT1G27150	403.01974	-0.850169	0.1272272	-6.682288	2.35E-11	1.05E-09	8.2040152	8.087608	8.1943437	9.0240752	8.9858227	9.0679173
AT5G52910	106.96891	-0.847981	0.2152955	-3.938686	8.19E-05	0.0010089	5.9429895	6.3529987	6.3606699	7.2743201	6.9075785	7.2046755
AT4G14620	288.54908	-0.847942	0.1468006	-5.776147	7.64E-09	2.30E-07	7.8055151	7.6558783	7.5958941	8.4172953	8.5811075	8.6205967
AT5G23980	261.89379	-0.847691	0.1976035	-4.289857	1.79E-05	2.63E-04	7.3842879	7.4914813	7.6432179	8.7592901	8.0073288	8.4085672
AT1G70420	263.34051	-0.847262	0.2153041	-3.935188	8.31E-05	1.02E-03	7.6884929	7.6693264	7.1372542	8.5671205	8.6058113	8.0582656
AT5G36910	105.93549	-0.845965	0.2519075	-3.358237	7.84E-04	7.11E-03	6.5687531	5.8612842	6.1691841	6.9254163	6.9075785	7.4510579
AT3G59210	158.9135	-0.845714	0.1804933	-4.685568	2.79E-06	5.05E-05	6.7931777	6.8780725	6.7034739	7.9681804	7.4885299	7.6200665
AT1G03960	48.992892	-0.844934	0.2588445	-3.264254	1.10E-03	9.43E-03	5.2585368	5.0490981	5.032027	5.9853613	6.2366987	5.835068
AT5G06865	245.45386	-0.841439	0.1580905	-5.322513	1.02E-07	2.53E-06	7.6155729	7.3272788	7.4031689	8.2302543	8.3952139	8.3027258
AT2G25930	354.00109	-0.841423	0.1543655	-5.450853	5.01E-08	1.32E-06	8.0804351	7.9092437	7.9745351	8.5984029	8.8320434	9.0336677
AT5G00820	100.95224	-0.840374	0.2203979	-3.812986	0.0001373	0.0015913	6.3803752	5.9410631	6.1142637	7.1531516	6.7831397	7.1821107
AT1G76180	3350.4572	-0.83949	0.0957593	-8.766674	1.84E-18	1.87E-16	11.260781	11.259495	11.147923	12.119237	12.060756	12.040694
AT5G01215	262.65164	-0.838387	0.1864279	-4.497112	6.89E-06	0.000113	7.2392665	7.5724458	7.7871051	8.2500881	8.4232821	8.5385969
AT5G01210	262.65164	-0.838387	0.1864279	-4.497112	6.89E-06	1.13E-04	7.2392665	7.5724458	7.7871051	8.2500881	8.4232821	8.5385969
AT2G45740	924.95257	-0.833349	0.1131296	-7.366325	1.75E-13	1.11E-11	9.4058704	9.2849313	9.4241158	10.186039	10.169783	10.287452
AT5G63620	413.28114	-0.833252	0.1407957	-5.918167	3.26E-09	1.04E-07	8.2665931	8.1416739	8.2463515	8.8268819	9.1459878	9.1573926
AT1G71040	513.59357	-0.83291	0.1555372	-5.355052	8.55E-08	2.15E-06	8.5921161	8.3480868	8.5901495	9.3705812	9.5540554	9.1717801
AT1G21770	555.11806	-0.832802	0.1639471	-5.079697	3.78E-07	8.31E-06	8.8530175	8.3564247	8.6298486	9.4753978	9.3895172	9.5847592
AT2G24240	185.70709	-0.83243	0.1890007	-4.404374	1.06E-05	1.67E-04	7.2484831	7.0160404	6.9110554	7.6476919	7.8713123	8.1483231
AT1G08570	783.56309	-0.831437	0.1337354	-6.217034	5.07E-10	1.91E-08	9.2879082	9.0558105	9.0366098	9.9517924	9.8894996	10.084934
AT5G49450	569.17333	-0.831274	0.1899195	-4.376983	1.20E-05	0.0001863	8.8116445	8.3831948	8.7625633	9.1975278	9.4924623	9.8074976
AT2G30360	79.69026	-0.829672	0.2343876	-3.539745	4.01E-04	3.97E-03	5.7617592	6.1083766	5.5942108	6.5306385	6.8875699	6.6685459
AT5G57340	368.93479	-0.829589	0.148557	-5.584318	2.35E-08	6.45E-07	7.9871649	8.1344195	7.9621477	9.1134066	8.7623357	8.8054205
AT3G01475	252.31476	-0.829424	0.1474201	-5.626263	1.84E-08	5.15E-07	7.4616157	7.4414118	7.6118418	8.2500881	8.3809723	8.3939142
AT5G02810	1350.6522	-0.828363	0.1156931	-7.160004	8.07E-13	4.59E-11	9.8858248	10.049321	9.8135649	10.749842	10.730656	10.800224
AT2G30140	317.33251	-0.827681	0.220485	-3.753912	1.74E-04	1.95E-03	7.7898186	7.9120747	7.7224736	8.5188872	9.1375412	8.2602708
AT2G30362	80.710766	-0.827501	0.2331075	-3.54987	3.85E-04	3.85E-03	5.7745758	6.1279897	5.610177	6.5940902	6.8875699	6.6685459
AT5G27930	131.31355	-0.825986	0.1798059	-4.593765	4.35E-06	7.51E-05	6.6544552	6.562094	6.487097	7.3125489	7.3637291	7.5335863
AT1G21130	1546.7413	-0.825359	0.1755609	-4.701266	2.59E-06	4.71E-05	10.366695	9.8774752	10.028568	10.870069	10.773535	11.203902
AT5G40720	176.4923	-0.82426	0.1574134	-5.236274	1.64E-07	3.89E-06	6.9651548	6.9841114	7.0285834	7.789971	7.8194503	7.8804773
AT5G55700	400.07822	-0.82425	0.1578708	-5.221043	1.78E-07	4.19E-06	8.1801315	8.09011	8.2386688	8.8787641	8.8528315	9.2498017
AT5G58600	103.83838	-0.823873	0.2327426	-3.539848	4.00E-04	0.0039744	6.0835188	6.047885	6.4432054	7.2350505	6.6705562	7.2809839
AT2G46610	122.33803	-0.823178	0.1981639	-4.154024	3.27E-05	4.49E-04	6.6264497	6.3446409	6.4432054	7.0663221	7.2740963	7.5246462

AT3G50500	1216.4575	-0.822634	0.1225823	-6.710877	1.93E-11	8.66E-10	9.8476712	9.6597966	9.8005782	10.618677	10.483151	10.713391
AT3G63010	56.844749	-0.822489	0.2530691	-3.250057	0.0011538	0.0098322	5.5091544	5.2593357	5.3874144	5.7824096	6.2047119	6.5324599
AT5G11520	578.38716	-0.822223	0.1304794	-6.301552	2.95E-10	1.13E-08	8.6747728	8.7377549	8.7153684	9.3245555	9.6039585	9.6458126
AT2G42790	642.6444	-0.820842	0.1300143	-6.313473	2.73E-10	1.05E-08	8.8224853	8.8154115	8.9004142	9.8186668	9.5089277	9.7298916
AT3G10020	861.76331	-0.82065	0.1883181	-4.357783	1.31E-05	2.01E-04	9.0503631	9.6101061	9.0366098	10.27585	9.9291908	10.135724
AT4G34860	89.899462	-0.820096	0.2087496	-3.928613	8.54E-05	1.04E-03	5.8732049	6.0061005	6.1905799	6.7131893	6.9467821	6.9210618
AT5G55860	200.0929	-0.819288	0.1587557	-5.160683	2.46E-07	5.63E-06	7.2253302	7.1419581	7.0924916	8.1897496	7.9213745	7.9354769
AT1G56700	327.79869	-0.816467	0.1464843	-5.573753	2.49E-08	6.81E-07	7.9899152	7.7911945	7.8985629	8.5351448	8.751306	8.83465
AT3G51830	326.51538	-0.815987	0.1363917	-5.982676	2.20E-09	7.24E-08	7.931029	7.8543728	7.8320461	8.8136145	8.6058113	8.7218449
AT4G27450	3186.5046	-0.815639	0.1698071	-4.803329	1.56E-06	3.00E-05	11.313878	10.948096	11.181371	11.802435	11.85139	12.291865
AT4G01130	166.3265	-0.815587	0.1757595	-4.64036	3.48E-06	6.16E-05	6.9081386	6.8007513	6.9924932	7.8168173	7.5407104	7.8663935
AT2G18670	222.21343	-0.814977	0.1620522	-5.029104	4.93E-07	1.06E-05	7.445677	7.3357375	7.1591252	8.1897496	8.238167	8.0582656
AT1G45249	171.56503	-0.812697	0.2493043	-3.259861	1.11E-03	0.0095573	6.761338	7.2217066	6.6426751	8.308004	7.501753	7.4883212
AT4G25500	596.35797	-0.8125	0.1407594	-5.772263	7.82E-09	2.34E-07	8.7432021	8.6989408	8.8254617	9.388588	9.5348876	9.7720522
AT5G05890	157.02011	-0.81231	0.1820316	-4.462467	8.10E-06	0.0001299	6.9197236	6.6047136	6.9429239	7.5866041	7.6397294	7.7484809
AT1G21410	213.74689	-0.81208	0.193185	-4.20364	2.63E-05	3.70E-04	7.4009154	7.0626468	7.2279995	8.4519603	7.9792425	7.8663935
AT4G21570	373.29113	-0.811146	0.1486056	-5.458383	4.80E-08	1.27E-06	8.019827	8.1025553	8.0467059	9.1560802	8.767819	8.783103
AT1G49032	582.49281	-0.810883	0.1897266	-4.273955	1.92E-05	2.80E-04	9.0490451	8.3791085	8.6001771	9.5572792	9.4281219	9.6581281
AT5G23660	288.67269	-0.809907	0.2075648	-3.901951	9.54E-05	1.15E-03	7.211258	7.8336193	7.937049	8.4172953	8.5873232	8.6079552
AT5G62720	431.95027	-0.808658	0.1722771	-4.693936	2.68E-06	4.87E-05	8.2620497	8.0262203	8.4880038	9.3245555	8.9525032	9.0617503
AT1G80910	277.77527	-0.808522	0.1761883	-4.588964	4.45E-06	7.66E-05	7.7675527	7.5471738	7.6548102	8.2302543	8.3809723	8.7566221
AT4G19860	1334.6463	-0.80749	0.1159917	-6.961621	3.36E-12	1.73E-10	9.9070783	9.8198518	10.021123	10.660035	10.71513	10.823192
AT4G17900	353.46258	-0.807305	0.1457149	-5.540304	3.02E-08	8.16E-07	8.1484797	7.9756986	7.8690092	8.731341	8.9085009	8.8201092
AT2G20960	882.03173	-0.806015	0.1228088	-6.563166	5.27E-11	2.21E-09	9.3735908	9.2684572	9.3016464	10.196299	10.228627	9.9858191
AT3G50740	727.81524	-0.802501	0.1689637	-4.74955	2.04E-06	3.80E-05	9.2979303	8.7724399	8.9992074	9.6866255	9.9389453	9.9495384
AT4G28490	132.60969	-0.799886	0.1765772	-4.529952	5.90E-06	9.86E-05	6.5687531	6.6116961	6.5380629	7.5866041	7.3344659	7.3433248
AT2G47180	156.55064	-0.798874	0.2387351	-3.346276	8.19E-04	7.38E-03	7.0519383	6.6392927	6.5953332	8.1480747	7.4205328	7.33312
AT4G27745	103.95839	-0.797094	0.192222	-4.146734	3.37E-05	4.61E-04	6.2487836	6.1852782	6.3224152	6.9739302	7.0221311	7.1821107
AT1G12730	79.794607	-0.79616	0.2248899	-3.540222	4.00E-04	3.97E-03	6.1039734	5.6871314	5.7319184	6.7131893	6.6937867	6.6846883
AT1G10410	210.01461	-0.794666	0.1936631	-4.103345	4.07E-05	5.44E-04	7.215964	7.3566697	7.1094408	8.2696528	8.2222785	7.7016547
AT4G24690	1858.9778	-0.794187	0.0957127	-8.297609	1.06E-16	9.46E-15	10.399128	10.399751	10.412292	11.243464	11.141967	11.23575
AT4G21650	266.0571	-0.793637	0.2199011	-3.609061	3.07E-04	3.16E-03	7.0624282	7.9064072	7.6276151	8.5512213	8.3952139	8.3183298
AT1G12780	2385.666	-0.792997	0.1679866	-4.720595	2.35E-06	4.33E-05	10.777751	10.759392	10.727962	11.231043	11.530205	11.881745

AT1G54410	3119.4413	-0.792297	0.1470691	-5.387247	7.15E-08	1.82E-06	10.970045	11.315896	11.119968	12.147271	11.781279	11.924004
AT5G15150	62.773824	-0.792274	0.2418854	-3.27541	1.06E-03	9.11E-03	5.6127075	5.2770121	5.528518	6.6548684	6.2047119	6.2144228
AT3G18930	124.73766	-0.792078	0.2018632	-3.923835	8.72E-05	1.06E-03	6.7287798	6.4181729	6.3606699	7.2350505	7.2110898	7.506598
AT3G22200	1026.58	-0.791486	0.1398718	-5.658651	1.53E-08	4.30E-07	9.317768	9.5586429	9.7246932	10.378529	10.339569	10.344449
AT2G07707	2302.0873	-0.791278	0.2133339	-3.709108	2.08E-04	2.28E-03	10.554899	10.995578	10.464274	11.10602	11.896516	11.473238
AT4G34230	123.60976	-0.789347	0.2210594	-3.570748	3.56E-04	3.60E-03	6.3378311	6.6663714	6.3606699	7.5228149	7.4751844	6.9210618
AT4G17230	315.17314	-0.78904	0.1435066	-5.498285	3.84E-08	1.03E-06	7.8302795	7.9566301	7.7370884	8.6440903	8.7289905	8.582335
AT5G27280	364.05918	-0.785852	0.1422857	-5.523052	3.33E-08	8.96E-07	7.9509259	7.9918448	8.2101439	8.8659674	8.8162534	8.8913902
AT2G01450	1238.1058	-0.784532	0.1594294	-4.920874	8.62E-07	1.76E-05	10.023055	9.5468518	9.8400393	10.52817	10.540337	10.784395
AT3G26910	107.37151	-0.78309	0.1882981	-4.158778	3.20E-05	4.40E-04	6.2209611	6.3446409	6.3320744	7.0663221	7.1785237	7.0759594
AT3G56140	622.03299	-0.782565	0.1216898	-6.430819	1.27E-10	5.07E-09	8.7690498	8.8304536	8.906892	9.5412705	9.6039585	9.7201354
AT3G26230	210	-0.781927	0.1565685	-4.994152	5.91E-07	1.25E-05	7.3759017	7.1419581	7.2738258	8.0382954	8.0884486	8.070603
AT5G02100	114.27005	-0.781526	0.1857434	-4.207556	2.58E-05	0.0003647	6.4379048	6.3940763	6.3887097	6.9739302	7.3045969	7.2595902
AT3G13750	15060.875	-0.781081	0.1677342	-4.656656	3.21E-06	5.74E-05	13.686422	13.322803	13.19941	14.101359	14.101523	14.449955
AT4G26670	171.04892	-0.779908	0.1835027	-4.250117	2.14E-05	3.08E-04	6.872815	6.8605934	7.1317342	7.894483	7.5407104	7.8734526
AT2G02760	952.77196	-0.779181	0.1423258	-5.474631	4.38E-08	1.17E-06	9.4634015	9.2551412	9.5933055	10.271004	10.095412	10.348254
AT5G54500	690.90951	-0.777509	0.1364987	-5.696094	1.23E-08	3.53E-07	8.9966785	8.9005495	9.073067	9.5652174	9.8093443	9.9208649
AT5G07010	575.50495	-0.777321	0.2300856	-3.378398	0.0007291	0.006674	8.6798914	8.8304536	8.5921606	8.8268819	9.9316356	9.6018612
AT3G29670	179.06957	-0.776177	0.1896887	-4.091846	4.28E-05	5.69E-04	7.2937031	6.7884791	6.9617125	7.8690562	7.8404194	7.8159882
AT2G45510	123.54233	-0.776154	0.1972859	-3.93416	8.35E-05	1.02E-03	6.6193626	6.5692853	6.2529269	7.3860957	7.3781411	7.1706945
AT3G19930	258.14494	-0.775397	0.1805186	-4.295387	1.74E-05	2.57E-04	7.7449377	7.4137138	7.5552361	8.0153003	8.4371141	8.5562516
AT3G09260	2078.727	-0.774682	0.1300286	-5.95778	2.56E-09	8.31E-08	10.68151	10.619612	10.408873	11.296882	11.278836	11.503129
AT1G78670	569.77115	-0.773543	0.11374	-6.800976	1.04E-11	4.86E-10	8.711894	8.7136187	8.7023937	9.5412705	9.4453338	9.5051876
AT1G09490	250.48739	-0.772284	0.1716993	-4.497883	6.86E-06	0.0001127	7.2981481	7.6077975	7.6315317	8.3636843	8.1401005	8.4374342
AT1G69490	601.37149	-0.771806	0.1509656	-5.112464	3.18E-07	7.09E-06	8.8799548	8.7863923	8.6665771	9.7082944	9.320302	9.6784231
AT2G22540	808.04375	-0.770804	0.1576439	-4.889524	1.01E-06	2.03E-05	9.2482577	9.0195182	9.3464181	9.9698458	9.8013457	10.205913
AT5G03200	117.56105	-0.77039	0.1827631	-4.215239	2.50E-05	0.0003543	6.5239141	6.4101853	6.3979365	7.1531516	7.2271013	7.3020649
AT5G22505	197.01986	-0.769009	0.2334388	-3.294264	9.87E-04	8.63E-03	6.9197236	7.1176385	7.4304023	7.8168173	7.6156045	8.3840621
AT1G73980	374.52367	-0.767603	0.135655	-5.658495	1.53E-08	4.30E-07	8.0778522	8.0674343	8.2048963	8.7592901	8.9085009	8.982352
AT5G35560	391.23755	-0.7668	0.1306899	-5.867327	4.43E-09	1.37E-07	8.1631746	8.2030626	8.1567896	8.9532484	8.8528315	9.0462164
AT3G10120	97.54945	-0.762988	0.2227916	-3.424673	6.16E-04	0.0057587	6.3970476	6.2038797	5.9101537	6.6548684	7.1619607	7.0134269
AT3G20250	497.76614	-0.762958	0.1404445	-5.43245	5.56E-08	1.45E-06	8.6985056	8.4548351	8.3942981	9.2477168	9.349851	9.2978183
AT1G62480	2431.2046	-0.762819	0.170152	-4.483163	7.35E-06	0.0001196	11.104599	10.60831	10.63201	11.413598	11.596417	11.739282

AT1G09430	1389.4009	-0.76102	0.1377623	-5.524151	3.31E-08	8.91E-07	10.109686	9.876026	10.016638	10.611029	10.734862	10.961286
AT1G01780	78.760384	-0.760884	0.2275657	-3.343578	8.27E-04	7.43E-03	5.8123562	5.8845319	5.9229937	6.5940902	6.3867874	6.9346244
AT5G22920	2585.6943	-0.760719	0.2022692	-3.760924	1.69E-04	1.90E-03	10.948999	10.809975	10.887543	11.177674	11.653812	12.075162
AT3G20410	439.27152	-0.75971	0.1375991	-5.521184	3.37E-08	9.03E-07	8.4674185	8.2485688	8.3282658	8.9772457	9.1459878	9.2085298
AT5G02160	5551.1765	-0.759686	0.1823634	-4.165782	3.10E-05	4.28E-04	11.950753	11.807484	12.184563	12.620848	12.528751	13.120764
AT5G63760	101.09772	-0.757889	0.2198653	-3.44706	5.67E-04	5.35E-03	6.4854533	6.0166606	6.1364834	6.875214	7.2110898	6.9346244
AT1G80325	154.26334	-0.75784	0.21196	-3.575392	3.50E-04	3.55E-03	6.5163035	6.8129199	7.0462956	7.894483	7.3491717	7.6031803
AT1G66890	145.3593	-0.756588	0.1967582	-3.845269	1.20E-04	1.41E-03	6.8119495	6.6528956	6.7474485	7.6772924	7.1785237	7.7016547
AT5G59960	419.66346	-0.755583	0.155523	-4.858333	1.18E-06	2.34E-05	8.3945667	8.2053723	8.2489033	8.9040219	8.9331121	9.2766746
AT5G24800	278.1127	-0.754631	0.1404127	-5.374378	7.68E-08	1.95E-06	7.6120069	7.7443108	7.6890385	8.5351448	8.4022823	8.4469296
AT5G63860	808.92688	-0.754158	0.1310788	-5.753466	8.74E-09	2.59E-07	9.1667455	9.2326709	9.2705891	10.084752	9.7959885	10.091003
AT5G24500	510.54662	-0.753778	0.1254671	-6.007775	1.88E-09	6.34E-08	8.5480085	8.6040947	8.5555244	9.277013	9.255426	9.4472285
AT3G48360	2277.4612	-0.753272	0.2210263	-3.408066	6.54E-04	0.0060694	11.018663	10.502406	10.513918	11.024876	11.469247	11.87824
AT3G56720	344.25857	-0.752731	0.1506461	-4.996687	5.83E-07	1.23E-05	7.7929716	8.09011	8.0728656	8.8268819	8.6832965	8.7905806
AT1G18330	411.37192	-0.752009	0.1790962	-4.19891	2.68E-05	3.77E-04	8.515929	8.0209848	8.1595047	8.9890964	8.8682284	9.1973216
AT4G32940	901.23843	-0.751916	0.1416719	-5.307449	1.11E-07	2.73E-06	9.5729247	9.2098451	9.3452262	10.056872	10.254245	10.132785
AT4G16150	987.4221	-0.751873	0.1228587	-6.119819	9.37E-10	3.36E-08	9.4956896	9.597851	9.4409521	10.383027	10.134139	10.321403
AT3G16180	104.86758	-0.750762	0.210566	-3.565447	3.63E-04	3.65E-03	6.114093	6.2582871	6.5042865	6.8232017	7.0583794	7.2487732
AT1G11530	339.53084	-0.748355	0.1470659	-5.088569	3.61E-07	7.97E-06	8.0700758	7.9149001	7.9621477	8.6883751	8.6180063	8.9017849
AT3G05020	209.33081	-0.748117	0.1752657	-4.268476	1.97E-05	2.87E-04	7.211258	7.0978832	7.4920164	8.1051601	7.9409229	8.1069947
AT2G36320	470.39228	-0.747539	0.1271214	-5.880509	4.09E-09	1.28E-07	8.4110769	8.4114791	8.5347617	9.187277	9.1459878	9.2925613
AT4G15540	300.93525	-0.747455	0.141332	-5.288649	1.23E-07	3.01E-06	7.7834919	7.7569629	7.8690092	8.6290213	8.4439806	8.6331284
AT1G75820	1041.6008	-0.746611	0.1852263	-4.030806	5.56E-05	7.13E-04	9.6866325	9.3779644	9.6847284	9.93963	10.447719	10.611526
AT2G08780	344.82641	-0.746129	0.1831001	-4.07498	4.60E-05	0.0006055	8.1825378	8.0750326	7.692792	8.6137933	8.9811096	8.6821941
AT1G03080	805.48489	-0.742759	0.148229	-5.010891	5.42E-07	1.15E-05	9.213382	9.4341889	8.9870309	10.022689	9.9606547	9.9792901
AT4G20880	156.67258	-0.742199	0.1902916	-3.900326	9.61E-05	1.16E-03	6.7677624	6.9624259	6.7618138	7.9681804	7.4885299	7.4415898
AT2G39310	412.0868	-0.741561	0.198915	-3.728028	1.93E-04	0.0021315	8.5793901	8.1961111	7.905049	8.9040219	8.923318	9.2251805
AT3G17770	325.83515	-0.741445	0.1535189	-4.829665	1.37E-06	2.66E-05	8.0117306	7.9837943	7.7587368	8.7028397	8.5368311	8.7980196
AT1G14890	164.33691	-0.739749	0.2029685	-3.644649	0.0002678	0.0028111	7.1187975	6.8129199	6.8518556	7.7062978	7.3924105	7.9555729
AT3G13445	193.60877	-0.739495	0.1574347	-4.697154	2.64E-06	4.80E-05	7.2392665	7.0677336	7.201897	7.9919327	7.8814647	7.9354769
AT2G36220	419.05481	-0.739361	0.1747167	-4.231768	2.32E-05	3.32E-04	8.4889643	8.3375963	7.9745351	8.9532484	9.1997173	8.9725238
AT2G28900	902.4076	-0.739223	0.1745053	-4.236106	2.27E-05	0.0003261	9.4323975	9.1011694	9.5781655	10.241585	9.8844606	10.299295
AT2G39900	114.92978	-0.739169	0.2130532	-3.469412	5.22E-04	0.0049898	6.2021101	6.6596492	6.3700772	7.1531516	7.0221311	7.3735134

AT1G09530	241.76764	-0.738598	0.1675301	-4.408747	1.04E-05	0.0001637	7.5648279	7.3976444	7.5260779	8.2302543	8.0439463	8.451654
AT5G54510	812.96126	-0.736308	0.2266618	-3.248487	1.16E-03	9.88E-03	9.250553	9.2144392	9.2233612	9.2962184	10.117055	10.391923
AT1G01650	275.56773	-0.736293	0.1435827	-5.128011	2.93E-07	6.61E-06	7.5831567	7.7850307	7.6815021	8.4519603	8.4302147	8.4374342
AT2G18160	325.10129	-0.735724	0.1378467	-5.337263	9.44E-08	2.35E-06	7.8962681	7.8602479	8.0200631	8.673764	8.6119217	8.737405
AT1G12230	780.39948	-0.731201	0.152657	-4.789829	1.67E-06	3.19E-05	9.1097276	9.0596454	9.3641803	10.022689	9.7159876	10.052646
AT3G10113	221.89088	-0.730319	0.1737189	-4.204029	2.62E-05	3.69E-04	7.5940432	7.2217066	7.3035874	8.0153003	8.0971867	8.249459
AT1G21610	365.58697	-0.729813	0.1501203	-4.861524	1.16E-06	2.30E-05	8.2040152	8.087608	8.0200631	8.6883751	8.7732816	9.0305134
AT2G17880	449.54321	-0.729155	0.218093	-3.343322	8.28E-04	7.43E-03	8.3524453	8.0183599	8.7042544	8.8136145	9.0813989	9.468363
AT1G33970	379.76037	-0.728866	0.1545371	-4.716449	2.40E-06	4.41E-05	7.9195348	8.2328043	8.2539934	9.0240752	8.8320434	8.8454601
AT5G62470	402.38981	-0.727221	0.1873856	-3.88088	0.0001041	0.0012408	8.443541	8.0951011	8.1210203	9.0008505	8.6657811	9.2334343
AT2G31800	703.77884	-0.727185	0.1290356	-5.63554	1.75E-08	4.90E-07	9.1703869	8.961943	8.993132	9.7714143	9.6902298	9.8828913
AT3G13672	225.19314	-0.72714	0.1612098	-4.510521	6.47E-06	0.0001069	7.4173535	7.3608199	7.3847234	8.3268039	7.9115001	8.1941497
AT4G38540	332.73433	-0.72638	0.1573676	-4.615816	3.92E-06	6.85E-05	8.1533946	7.874832	7.8656878	8.5188872	8.8002886	8.7718135
AT2G36420	86.959565	-0.725972	0.2188776	-3.316796	9.11E-04	8.06E-03	5.9429895	6.027144	6.0217961	7.11039	6.4427077	6.7928944
AT1G74840	713.42456	-0.725876	0.167528	-4.332862	1.47E-05	2.21E-04	9.3232305	8.907659	8.9052753	9.7437014	9.6786327	9.9792901
AT3G08760	289.95812	-0.725608	0.14607	-4.967537	6.78E-07	1.42E-05	7.808634	7.6859621	7.7623135	8.6590034	8.5109065	8.3641539
AT2G15080	251.00431	-0.725318	0.2006835	-3.614237	3.01E-04	0.0031096	7.8333454	7.4335522	7.3754114	7.9194695	8.5043519	8.4085672
AT5G18525	321.82437	-0.724746	0.1402121	-5.168925	2.35E-07	5.42E-06	7.8333454	8.0078123	7.8789279	8.7730642	8.6058113	8.6037167
AT3G53960	115.61296	-0.723762	0.1991322	-3.634581	0.0002784	0.0029108	6.3116892	6.5692853	6.3416693	7.4560742	7.1110995	7.0261521
AT5G09130	712.6346	-0.723687	0.1556447	-4.649611	3.33E-06	5.91E-05	9.2039377	8.9454567	9.0410336	9.5004538	9.9193699	9.936116
AT2G15695	418.07807	-0.723601	0.1412169	-5.124039	2.99E-07	6.74E-06	8.2620497	8.2861516	8.3616597	8.8914483	8.9621018	9.2113183
AT5G39570	1666.4948	-0.722757	0.1683006	-4.294444	1.75E-05	0.0002581	10.051637	10.606127	10.11643	11.100568	10.990738	10.997276
AT4G27020	226.51659	-0.721319	0.1508241	-4.782519	1.73E-06	3.30E-05	7.4295603	7.3272788	7.4527106	8.2500881	8.1652491	8.052057
AT4G20930	118.52335	-0.720084	0.201786	-3.568551	3.59E-04	0.0036231	6.6193626	6.3695703	6.3416693	7.5228149	7.0403691	7.1241044
AT5G03470	407.32982	-0.717717	0.12774	-5.618576	1.93E-08	5.35E-07	8.2414252	8.3078102	8.2539934	8.9288451	8.9525032	9.0832206
AT5G21170	1336.9208	-0.717405	0.1645234	-4.360503	1.30E-05	1.98E-04	10.089954	9.7487884	10.050675	10.52817	10.589161	10.965421
AT2G31585	140.99335	-0.716598	0.1855277	-3.862487	1.12E-04	1.33E-03	6.7287798	6.547603	6.8451253	7.6772924	7.3924105	7.3635206
AT1G53320	500.3121	-0.716403	0.1258	-5.69478	1.24E-08	3.55E-07	8.5517361	8.5063233	8.639605	9.1665543	9.3240289	9.3544146
AT1G34370	873.46682	-0.71546	0.1452242	-4.926588	8.37E-07	1.72E-05	9.4058704	9.3076835	9.3852102	9.8384535	10.080067	10.30844
AT1G53590	290.13737	-0.715039	0.1402125	-5.099683	3.40E-07	7.55E-06	7.7771374	7.8455149	7.692792	8.5351448	8.4371141	8.5430308
AT3G20550	218.09483	-0.714397	0.1756851	-4.066349	4.78E-05	6.26E-04	7.2530694	7.5937606	7.1913224	8.126777	8.0257537	8.142491
AT4G39660	328.36569	-0.713919	0.1673636	-4.265678	1.99E-05	2.89E-04	8.054396	7.8095295	8.0170722	8.3453619	8.8056298	8.8273979
AT5G14420	246.41171	-0.713597	0.1704982	-4.185361	2.85E-05	3.99E-04	7.5499954	7.5288475	7.5918794	7.9194695	8.2924378	8.5071716

AT1G03220	269.92512	-0.713189	0.1939212	-3.677727	2.35E-04	2.53E-03	7.9050372	7.5652703	7.4213815	8.6883751	8.3150859	8.1997771
AT4G25670	312.61034	-0.712552	0.1364464	-5.222214	1.77E-07	4.17E-06	7.8302795	7.9538853	7.8523251	8.673764	8.5432401	8.6205967
AT3G48990	3134.7987	-0.710519	0.1135346	-6.258173	3.90E-10	1.48E-08	11.273535	11.053135	11.288504	11.950699	11.876987	11.963408
AT3G15040	117.62502	-0.710339	0.1810362	-3.923739	8.72E-05	1.06E-03	6.4217005	6.4805297	6.5296927	7.1946821	7.242937	7.1934372
AT2G02060	75.450516	-0.709878	0.2141476	-3.314902	9.17E-04	0.0081061	5.8968434	5.8014713	5.816924	6.5940902	6.4698754	6.6685459
AT1G10060	169.00437	-0.708598	0.193123	-3.669153	2.43E-04	0.0025882	7.0624282	6.7510235	7.1317342	7.8168173	7.4885299	7.8663935
AT1G73480	966.10286	-0.706598	0.1577355	-4.479639	7.48E-06	0.0001213	9.5756735	9.5568352	9.3618246	10.503594	9.9937887	10.176249
AT4G02715	317.91533	-0.706416	0.1562438	-4.521238	6.15E-06	0.0001022	8.0700758	7.9092437	7.7370884	8.5512213	8.7568314	8.582335
AT4G14990	342.23684	-0.706266	0.1332023	-5.302207	1.14E-07	2.81E-06	8.0958359	7.9945183	7.9496529	8.786708	8.7289905	8.7061151
AT5G19120	2817.6524	-0.703991	0.1667548	-4.221716	2.42E-05	0.000346	11.24734	10.829573	11.05036	11.555842	11.71328	12.026515
AT2G18950	83.300736	-0.703622	0.2099418	-3.351512	0.0008037	0.0072607	5.9085191	6.068332	5.9732405	6.6548684	6.8672798	6.6190056
AT1G03210	103.25162	-0.703121	0.1943871	-3.617118	2.98E-04	3.08E-03	6.3970476	6.1946089	6.2731246	7.0663221	6.9273135	7.0759594
AT4G33700	113.29982	-0.702711	0.1936498	-3.628772	2.85E-04	0.0029672	6.2760797	6.5692853	6.4342646	7.11039	7.0937393	7.2487732
AT2G28910	806.91676	-0.701066	0.1966208	-3.565572	0.0003631	0.0036536	9.1235687	9.5960917	8.9117313	10.160066	9.991447	9.7682699
AT5G14270	547.86705	-0.700682	0.1209768	-5.791872	6.96E-09	2.10E-07	8.6627583	8.7755521	8.677984	9.3151715	9.4521614	9.4566599
AT1G62660	251.09023	-0.699591	0.1673946	-4.179294	2.92E-05	4.07E-04	7.445677	7.7757353	7.4438287	8.4172953	8.2062131	8.2656465
AT1G04620	170.6282	-0.699012	0.1867209	-3.743616	0.0001814	0.0020196	6.9707344	6.8780725	7.1806696	7.789971	7.501753	7.9013487
AT1G20880	84.780943	-0.698072	0.2140451	-3.261331	1.11E-03	0.0095193	5.8850726	6.0581447	6.1030242	6.7131893	6.5737024	6.9073704
AT5G03230	968.30127	-0.696902	0.1215669	-5.732661	9.89E-09	2.90E-07	9.6383677	9.4371399	9.4805752	10.295071	10.127756	10.271509
AT1G48840	227.15736	-0.695623	0.1591735	-4.370218	1.24E-05	1.91E-04	7.5425215	7.2972768	7.4790331	8.0153003	8.1485321	8.249459
AT1G02660	861.58511	-0.695122	0.1630095	-4.264303	2.01E-05	2.91E-04	9.4293615	9.1854828	9.4409521	9.7437014	10.127756	10.267495
AT1G21760	388.63431	-0.69434	0.1498606	-4.63324	3.60E-06	6.35E-05	8.3820589	8.0851017	8.1486135	8.8530562	8.8528315	9.0336677
AT4G30630	95.89595	-0.693653	0.2113745	-3.281632	1.03E-03	8.94E-03	6.2021101	6.0061005	6.2529269	7.2350505	6.7391548	6.7779245
AT5G47560	1586.1757	-0.69301	0.1008471	-6.871891	6.34E-12	3.09E-10	10.294561	10.209202	10.213688	10.920448	11.001251	10.897791
AT2G02220	341.25221	-0.692915	0.1908884	-3.62995	2.83E-04	2.96E-03	8.1211441	7.874832	8.0378796	8.5828465	9.0813989	8.4610565
AT5G48160	1715.0959	-0.690896	0.1138637	-6.06775	1.30E-09	4.52E-08	10.455431	10.346856	10.251081	10.995803	11.016302	11.140945
AT2G41140	324.97926	-0.690731	0.1444127	-4.783035	1.73E-06	3.29E-05	8.0225158	7.9345244	7.8556774	8.8400284	8.562298	8.582335
AT1G11820	355.94175	-0.689243	0.1819725	-3.787625	0.0001521	0.0017348	8.3112576	7.9400826	7.9714482	8.6590034	8.6240654	9.0462164
AT1G69252	589.28312	-0.688161	0.1657114	-4.15277	3.28E-05	4.51E-04	8.9676735	8.5249362	8.8808045	9.6271982	9.3277462	9.5847592
AT5G40170	314.76809	-0.687937	0.1414288	-4.864194	1.15E-06	2.28E-05	8.0008641	7.8306299	7.905049	8.5188872	8.717702	8.582335
AT3G46440	138.82539	-0.685436	0.1828108	-3.749428	0.0001772	0.0019814	6.5539611	6.8369536	6.7402118	7.5866041	7.3924105	7.353458
AT5G24060	231.78368	-0.684922	0.1758903	-3.894029	9.86E-05	0.0011837	7.3418608	7.6925631	7.3613299	8.0382954	8.1230878	8.3286394
AT1G79970	244.59345	-0.68391	0.1833448	-3.730182	1.91E-04	2.12E-03	7.5976539	7.4762596	7.5048839	8.5188872	7.8713123	8.3183298

AT4G17098	221.82081	-0.683463	0.1814251	-3.767189	1.65E-04	1.86E-03	7.173047	7.3731995	7.5838165	8.3453619	7.9792425	8.0078328
AT1G55280	108.93162	-0.680667	0.1903245	-3.57635	0.0003484	0.0035397	6.5086526	6.2934513	6.3512009	7.0663221	7.0761677	7.1241044
AT3G02340	302.8837	-0.680286	0.1559738	-4.361542	1.29E-05	0.0001976	8.0305521	7.7942666	7.7114144	8.6290213	8.4508147	8.5866368
AT5G18170	1690.0319	-0.680081	0.1973112	-3.446742	5.67E-04	0.0053559	10.524397	10.140857	10.286288	10.591728	11.048196	11.363455
AT4G14030	1249.7297	-0.679358	0.1257329	-5.403182	6.55E-08	1.68E-06	9.9773615	9.8504277	9.8809196	10.52817	10.474793	10.756984
AT3G01100	172.55765	-0.676839	0.184132	-3.675836	2.37E-04	2.54E-03	7.0200031	6.8722697	7.1806696	7.9194695	7.8088504	7.5156503
AT3G19990	238.74754	-0.676472	0.1580856	-4.279152	1.88E-05	2.74E-04	7.53877	7.5760202	7.4790331	8.0153003	8.1652491	8.3988151
AT2G26740	466.47705	-0.676214	0.1490429	-4.537045	5.70E-06	9.58E-05	8.5101932	8.4702834	8.500907	8.8914483	9.1915803	9.3744567
AT2G32240	774.88897	-0.673251	0.1332139	-5.05391	4.33E-07	9.43E-06	9.3155773	9.2805566	9.0395605	9.8384535	9.9984606	9.863523
AT1G28280	227.84665	-0.672396	0.1809659	-3.715593	2.03E-04	0.0022269	7.4214339	7.2666375	7.5998976	8.2889558	8.2538823	7.8804773
AT2G31280	160.18785	-0.669765	0.1851426	-3.617561	0.0002974	0.0030812	7.0780213	7.0369405	6.6809735	7.6476919	7.5910692	7.677659
AT5G57250	160.20574	-0.66938	0.2045149	-3.273012	1.06E-03	9.17E-03	6.9254813	7.0524192	6.783097	7.894483	7.2271013	7.7484809
AT2G37480	666.13422	-0.66847	0.1283733	-5.207238	1.92E-07	4.46E-06	9.0424367	8.907659	9.0542243	9.6793296	9.5572254	9.798254
AT3G62750	682.47873	-0.668234	0.1272375	-5.251865	1.51E-07	3.59E-06	8.9801757	8.9996938	9.1086256	9.8579725	9.5729719	9.7181762
AT5G56180	294.45605	-0.667788	0.1434914	-4.653852	3.26E-06	5.80E-05	7.9224169	7.8216247	7.7551512	8.3817769	8.5174315	8.5994656
AT5G05140	306.90763	-0.667624	0.1462759	-4.564146	5.02E-06	8.49E-05	8.0090216	7.8543728	7.8149264	8.3996454	8.6300992	8.6414228
AT1G64950	284.35017	-0.667574	0.1659794	-4.022028	5.77E-05	0.0007372	7.5758531	7.9483801	7.7334486	8.6137933	8.4439806	8.3286394
AT3G30390	1519.7052	-0.667272	0.1168635	-5.709843	1.13E-08	3.29E-07	10.254524	10.077964	10.227986	10.986965	10.778976	10.842207
AT5G44580	587.55603	-0.666032	0.1431048	-4.654159	3.25E-06	5.80E-05	8.9423394	8.5917882	8.8987902	9.5250821	9.4957705	9.4868928
AT1G77760	1615.5359	-0.666001	0.1894514	-3.515419	0.0004391	0.0042989	10.162446	10.187168	10.431514	11.18542	11.119625	10.504195
AT1G11545	238.77151	-0.665645	0.1824095	-3.649181	0.0002631	0.0027712	7.215964	7.6043011	7.662487	8.3817769	8.1817745	8.070603
AT5G13800	770.76268	-0.663498	0.1277004	-5.195741	2.04E-07	4.74E-06	9.1385182	9.2439498	9.2349922	10.062491	9.8564266	9.7511259
AT4G01120	418.4861	-0.663368	0.1770551	-3.746673	1.79E-04	2.00E-03	8.5628614	8.1344195	8.2816726	8.7592901	9.0045226	9.227937
AT3G11570	174.82207	-0.662693	0.1718189	-3.856928	1.15E-04	1.35E-03	7.1584528	7.1176385	6.9924932	7.5866041	7.6983337	7.9422067
AT2G07741	3117.5293	-0.662067	0.1630714	-4.059986	4.91E-05	6.41E-04	11.237826	11.366309	11.056928	11.600946	12.139534	11.932056
AT2G28400	504.13914	-0.661714	0.1376392	-4.8076	1.53E-06	2.94E-05	8.77065	8.4760341	8.5513957	9.2477168	9.2863196	9.3004396
AT4G38545	322.41755	-0.661491	0.1554683	-4.254827	2.09E-05	0.0003021	8.1509392	7.8395794	7.8855026	8.5024443	8.689088	8.6981854
AT1G01300	133.84717	-0.661472	0.1799405	-3.676059	2.37E-04	2.54E-03	6.6405204	6.6392927	6.8247441	7.2743201	7.3491717	7.4790952
AT4G23880	168.72955	-0.660583	0.1985028	-3.327825	0.0008753	0.0077903	7.2757848	6.7761016	6.9802596	7.6772924	7.5661095	7.8663935
AT2G45820	1394.2403	-0.6595	0.1371271	-4.809403	1.51E-06	2.92E-05	10.193796	9.9801881	10.021869	10.844205	10.532305	10.838604
AT3G14067	997.04635	-0.659071	0.1743827	-3.779454	1.57E-04	1.78E-03	9.2332483	9.6765419	9.7662552	10.461682	10.13626	10.181946
AT2G39570	2250.3715	-0.657958	0.1838826	-3.57814	3.46E-04	3.52E-03	11.051615	10.474908	10.676288	11.221029	11.378973	11.677606
AT5G44750	456.63951	-0.656245	0.1533831	-4.278473	1.88E-05	2.75E-04	8.6154564	8.5137973	8.2386688	9.0469319	9.2475981	9.1013727

AT1G53450	278.62709	-0.655602	0.1536773	-4.266097	1.99E-05	0.0002893	7.8148516	7.8095295	7.6739261	8.1897496	8.4508147	8.5780204
AT5G15230	1327.6186	-0.65558	0.1719974	-3.81157	1.38E-04	1.60E-03	9.827825	9.9598119	10.173286	10.840939	10.32848	10.807614
AT1G75410	466.16253	-0.655022	0.1469556	-4.457279	8.30E-06	1.33E-04	8.5044344	8.4912582	8.4419116	9.4239412	8.9905204	9.0679173
AT3G26280	892.33001	-0.653712	0.1235962	-5.28909	1.23E-07	3.00E-06	9.5082134	9.3448242	9.4274989	10.211552	9.9582586	10.119487
AT3G12490	541.91701	-0.653611	0.1367429	-4.779858	1.75E-06	3.33E-05	8.7480839	8.5740234	8.8220384	9.277013	9.349851	9.4937806
AT2G21500	166.4366	-0.652624	0.1838088	-3.550559	3.84E-04	3.84E-03	7.2017997	6.8664434	6.9045963	7.789971	7.7435613	7.5335863
AT1G55520	253.21211	-0.650742	0.1560087	-4.17119	3.03E-05	0.0004197	7.6918751	7.5688625	7.6432179	8.0153003	8.3520603	8.4230729
AT3G13061	173.28331	-0.650124	0.1709737	-3.802478	1.43E-04	1.65E-03	7.025375	7.112725	7.0521516	7.8690562	7.514856	7.8305703
AT1G79270	512.43968	-0.649697	0.1649872	-3.937863	8.22E-05	1.01E-03	8.7068879	8.7677589	8.3663678	9.4063729	9.3859559	9.1013727
AT2G35743	891.48752	-0.649545	0.1432703	-4.533702	5.80E-06	9.71E-05	9.5637242	9.3521389	9.3852102	9.9087678	10.053374	10.290092
AT4G05320	16878.278	-0.649143	0.1228262	-5.285054	1.26E-07	3.06E-06	13.601024	13.618809	13.796774	14.21704	14.490935	14.285543
AT4G22920	197.20212	-0.649107	0.1944624	-3.337958	8.44E-04	7.56E-03	7.211258	7.0107677	7.4438287	8.2101441	7.861088	7.7095654
AT2G28890	169.75028	-0.648766	0.1797363	-3.609544	0.0003067	0.0031606	7.009199	6.9514597	7.1913224	7.4898305	7.9213745	7.669571
AT3G51420	478.75129	-0.648043	0.147528	-4.392679	1.12E-05	1.75E-04	8.4752906	8.4874672	8.6061604	9.4063729	9.2037686	8.982352
AT2G02390	425.24471	-0.647912	0.1237918	-5.23388	1.66E-07	3.93E-06	8.3395652	8.3933601	8.3896804	8.9410983	9.0813989	9.0462164
AT5G08520	560.38633	-0.647395	0.1417412	-4.567441	4.94E-06	8.37E-05	8.9480077	8.652297	8.677984	9.3975079	9.3644016	9.5097253
AT1G71695	859.43748	-0.646852	0.1449142	-4.463693	8.06E-06	1.29E-04	9.4898725	9.210995	9.4286248	10.016912	9.8819344	10.20731
AT1G04400	2001.532	-0.646342	0.0989396	-6.532691	6.46E-11	2.66E-09	10.548383	10.634258	10.628095	11.265557	11.19686	11.308522
AT5G51970	2397.4434	-0.646276	0.1442587	-4.47998	7.47E-06	0.0001212	10.917565	10.800458	10.863599	11.301641	11.454871	11.764183
AT1G02860	259.45826	-0.646256	0.1430166	-4.518748	6.22E-06	0.0001032	7.6748846	7.6286001	7.6354376	8.4519603	8.2302446	8.2816545
AT1G19370	119.85723	-0.646138	0.1828925	-3.532883	4.11E-04	4.07E-03	6.5760926	6.4651914	6.5872896	7.1946821	7.3045969	7.1358938
AT4G36550	193.36994	-0.646113	0.1861304	-3.471294	5.18E-04	4.96E-03	7.4376412	7.1894011	7.0166534	7.894483	8.0529572	7.7407813
AT3G01470	814.49819	-0.646002	0.1561395	-4.13734	3.51E-05	4.78E-04	9.2309252	9.0961994	9.5359531	10.039882	9.8252098	10.025969
AT2G18280	1594.7714	-0.645065	0.1484699	-4.344752	1.39E-05	2.11E-04	10.385616	10.093011	10.318836	10.960123	10.712289	11.101658
AT3G62700	785.12006	-0.644669	0.123989	-5.199402	2.00E-07	4.65E-06	9.1655296	9.3500528	9.2207638	10.039882	9.8460959	9.8456869
AT5G11670	773.03402	-0.643831	0.137515	-4.681896	2.84E-06	5.13E-05	9.3448756	9.183141	9.1323143	10.045568	9.8970254	9.7162143
AT3G07780	675.50673	-0.640997	0.1521015	-4.214273	2.51E-05	0.0003553	9.1347953	9.1354883	8.8356832	9.4669481	9.782508	9.7963981
AT1G72680	255.39876	-0.640993	0.1873947	-3.420552	6.25E-04	5.84E-03	7.4050425	7.9064072	7.5344691	8.2302543	8.4576165	8.1885004
AT4G17140	1131.0594	-0.640172	0.1611084	-3.97355	7.08E-05	8.88E-04	9.6251607	10.071015	9.5812062	10.422891	10.496423	10.391923
AT3G53540	662.05917	-0.639693	0.1457163	-4.389987	1.13E-05	0.000177	9.1667455	8.9454567	8.9020364	9.6572178	9.5023641	9.8130155
AT1G66760	407.93341	-0.638052	0.1873449	-3.40576	0.0006598	0.0061132	8.5957316	8.0078123	8.2257728	9.1665543	8.8682284	8.8703732
AT1G18720	317.45717	-0.636557	0.1556455	-4.089787	4.32E-05	5.73E-04	7.9509259	7.7632475	8.0985593	8.731341	8.5109065	8.5780204
AT5G09220	507.26782	-0.636423	0.1508992	-4.217541	2.47E-05	3.51E-04	8.8424046	8.4489991	8.5513957	9.3245555	9.2397275	9.2766746

AT2G45170	873.72176	-0.636171	0.1565184	-4.064514	4.81E-05	0.0006307	9.4859814	9.3044551	9.4509604	9.7225617	10.186257	10.215666
AT1G21780	762.39773	-0.636104	0.1357686	-4.685207	2.80E-06	5.05E-05	9.3577086	9.033885	9.2362787	9.87084	9.7906114	9.9293576
AT1G50570	361.03735	-0.635633	0.1438689	-4.418142	9.96E-06	0.0001577	8.2298384	7.9998506	8.1810435	8.786708	8.689088	8.88092
AT5G48412	395.94207	-0.635179	0.1456155	-4.362029	1.29E-05	1.97E-04	8.2935574	8.1222472	8.3663678	9.058226	8.7732816	8.9325272
AT4G25690	291.40348	-0.635063	0.1522262	-4.171836	3.02E-05	4.19E-04	7.9509259	7.7757353	7.7976011	8.2696528	8.5043519	8.6205967
AT5G64370	299.47132	-0.634718	0.1516903	-4.184305	2.86E-05	0.0003997	7.8962681	7.7881159	7.9527868	8.308004	8.689088	8.5162203
AT1G20840	389.34346	-0.634182	0.1294418	-4.899358	9.62E-07	1.94E-05	8.2414252	8.3163834	8.2101439	8.786708	8.9525032	8.9291436
AT4G28650	266.89485	-0.632627	0.1483033	-4.265763	1.99E-05	2.89E-04	7.6542284	7.7154315	7.7551512	8.3453619	8.2222785	8.4750465
AT3G16450	192.11696	-0.632547	0.1879235	-3.365978	7.63E-04	6.94E-03	7.3245343	7.3230308	6.949214	8.126777	7.7211247	7.8086415
AT1G30270	372.77224	-0.631816	0.1353104	-4.669379	3.02E-06	5.42E-05	8.1311443	8.2773962	8.1703143	8.7171607	8.9331121	8.8273979
AT3G02455	152.2424	-0.631443	0.1823231	-3.463315	5.34E-04	0.0050922	6.872815	7.0317438	6.8040708	7.4560742	7.4205328	7.7330404
AT5G58375	200.9782	-0.631013	0.1675331	-3.766498	1.66E-04	1.86E-03	7.4416647	7.1467733	7.3084886	7.8690562	7.8915461	8.0582656
AT5G52170	313.47365	-0.630886	0.167958	-3.756214	1.73E-04	1.93E-03	8.0035884	7.8425502	8.0050462	8.2302543	8.6420915	8.7943049
AT2G24270	3280.8843	-0.630464	0.1193159	-5.283987	1.26E-07	3.07E-06	11.398335	11.216916	11.35397	11.918527	11.859759	12.105457
AT4G26130	313.97225	-0.630449	0.1759587	-3.582938	3.40E-04	3.46E-03	7.9166469	7.7347485	8.092889	8.8136145	8.6058113	8.3337668
AT1G48540	314.8903	-0.629412	0.155903	-4.037202	5.41E-05	6.95E-04	7.931029	8.0851017	7.7729907	8.731341	8.4576165	8.5866368
AT4G25970	716.16923	-0.629351	0.136178	-4.621534	3.81E-06	6.69E-05	9.259698	8.9996938	9.10722	9.8644206	9.622236	9.8239884
AT5G61820	1035.6331	-0.629261	0.1678203	-3.749614	0.0001771	0.0019809	9.5319962	9.7723481	9.6309641	10.630075	10.106274	10.135724
AT2G37640	351.40397	-0.628136	0.1661126	-3.781384	1.56E-04	1.77E-03	8.2063819	7.9345244	8.1098335	9.0008505	8.5109065	8.7179285
AT3G19290	460.16953	-0.627875	0.1323397	-4.744417	2.09E-06	3.88E-05	8.5848578	8.5006923	8.3942981	9.124194	9.0502385	9.2196516
AT3G20820	1107.1582	-0.626979	0.1453748	-4.312847	1.61E-05	0.0002399	9.756131	9.5739181	9.919072	10.44457	10.224645	10.51552
AT3G10250	256.60794	-0.625137	0.1460884	-4.27917	1.88E-05	0.0002741	7.6782987	7.6859621	7.5838165	8.3636843	8.1735355	8.3439671
AT5G27520	134.36529	-0.624554	0.1850234	-3.375538	0.0007367	0.0067354	6.722179	6.7946282	6.5792009	7.5866041	7.1948986	7.33312
AT3G06860	592.19471	-0.624171	0.1396867	-4.468366	7.88E-06	1.27E-04	8.6968233	9.0208302	8.8254617	9.5887733	9.4384737	9.46603
AT5G66880	432.99555	-0.623342	0.1681682	-3.706659	2.10E-04	2.30E-03	8.4928474	8.370901	8.3710605	8.8659674	8.8682284	9.3391981
AT2G46370	336.14934	-0.621713	0.1599973	-3.885772	1.02E-04	1.22E-03	7.8636511	7.9864828	8.2257728	8.8400284	8.5935123	8.6163952
AT1G32090	178.24454	-0.621298	0.1727108	-3.597331	3.21E-04	0.003297	7.0360591	7.0828872	7.1966194	8.0382954	7.6277174	7.6532575
AT3G51840	3096.9334	-0.620761	0.1247036	-4.977889	6.43E-07	1.35E-05	11.202102	11.257271	11.283223	11.778735	11.76492	12.069411
AT3G54140	433.10056	-0.620114	0.1409876	-4.398356	1.09E-05	0.0001712	8.5517361	8.3933601	8.2965495	8.9040219	9.0769884	9.1281798
AT1G07630	238.20614	-0.619234	0.1698271	-3.646259	2.66E-04	2.80E-03	7.6918751	7.3813939	7.5593538	8.1051601	8.3665887	8.0644475
AT2G32440	238.46992	-0.615081	0.1668168	-3.687162	2.27E-04	0.0024527	7.5904235	7.6007961	7.4833739	8.126777	7.9886655	8.3988151
AT2G37520	244.71229	-0.614966	0.1581917	-3.887473	0.0001013	0.0012115	7.6542284	7.6659761	7.4790331	8.0153003	8.3737984	8.2330877
AT5G03630	489.2901	-0.614429	0.1751052	-3.508914	0.0004499	0.004384	8.6610338	8.6369784	8.4216979	9.3431423	9.4036751	8.8738974

AT1G80440	2333.4715	-0.612988	0.1344856	-4.558018	5.16E-06	8.71E-05	10.928717	10.649605	10.931492	11.441898	11.349107	11.601531
AT5G52920	3007.8059	-0.610059	0.1068221	-5.710983	1.12E-08	3.27E-07	11.249923	11.148944	11.241228	11.777027	11.762175	11.945097
AT1G15350	275.20498	-0.609487	0.1749112	-3.48455	4.93E-04	4.74E-03	7.8962681	7.5435271	7.848965	8.2101441	8.322557	8.5909257
AT5G53450	378.83935	-0.608623	0.1515097	-4.017058	5.89E-05	7.51E-04	8.2298384	8.3729573	8.0408277	8.9040219	8.7289905	8.9086734
AT3G22440	2127.1419	-0.60859	0.1249239	-4.87169	1.11E-06	2.20E-05	10.843471	10.614407	10.670113	11.329873	11.204967	11.450996
AT2G38230	799.8923	-0.607919	0.1416944	-4.290354	1.78E-05	0.0002622	9.1046613	9.4759213	9.2880635	10.011112	9.9169042	9.8438911
AT4G25170	693.16036	-0.607271	0.1278004	-4.751713	2.02E-06	3.77E-05	9.2157335	9.0036805	9.0802492	9.5887733	9.7385036	9.8001074
AT5G03280	1172.8465	-0.607173	0.1135429	-5.34752	8.92E-08	2.23E-06	9.7785725	9.9529553	9.8408853	10.387511	10.479814	10.540127
AT1G33170	259.85673	-0.60667	0.163692	-3.706165	2.10E-04	0.0022999	7.8425041	7.6354681	7.5511065	8.3268039	8.1485321	8.4278759
AT3G07310	796.00863	-0.60616	0.1238768	-4.893247	9.92E-07	2.00E-05	9.2551328	9.3141188	9.3077785	10.039882	9.7468573	9.9495384
AT2G07699	3126.634	-0.606057	0.1644027	-3.686417	2.27E-04	2.46E-03	11.272405	11.40576	11.101732	11.575626	12.128921	11.91077
AT5G63880	147.62405	-0.605084	0.1851815	-3.26752	1.08E-03	0.0093317	6.8304803	6.7884791	7.0345117	7.1946821	7.6516422	7.560079
AT4G28652	259.57542	-0.603746	0.1489882	-4.052307	5.07E-05	6.59E-04	7.6226786	7.6826503	7.7407191	8.3453619	8.1569148	8.3939142
AT5G51570	150.67706	-0.603457	0.1710399	-3.528163	0.0004185	0.004132	6.987345	6.8309826	6.87186	7.5228149	7.603389	7.4415898
AT5G35370	149.85074	-0.602951	0.1773067	-3.40061	0.0006724	0.0062191	6.9197236	6.9787205	6.7329387	7.6476919	7.3924105	7.5156503
AT5G58070	841.23275	-0.602749	0.120973	-4.98251	6.28E-07	1.32E-05	9.3714833	9.2936412	9.4520681	10.106675	9.9891016	9.8828913
AT5G14780	4143.7804	-0.601648	0.0997079	-6.034103	1.60E-09	5.45E-08	11.697358	11.632303	11.710512	12.326093	12.188909	12.349324
AT1G35580	1022.7307	-0.601503	0.182766	-3.291111	9.98E-04	0.0087019	9.895391	9.4576301	9.5832298	9.945724	10.294692	10.528994
AT4G27870	901.4107	-0.600443	0.1556772	-3.856973	1.15E-04	1.35E-03	9.5244283	9.4322181	9.470771	10.117512	9.7986696	10.317526
AT4G21810	620.18812	-0.60034	0.1448839	-4.143596	3.42E-05	4.67E-04	9.0647828	8.7677589	8.9654715	9.6042663	9.3895172	9.6560828
AT4G27410	224.08854	-0.599632	0.1819803	-3.29504	0.0009841	0.0086049	7.5462633	7.5866906	7.2176151	8.2696528	7.861088	8.1366353
AT2G27200	138.56019	-0.59963	0.1735095	-3.455892	5.48E-04	5.21E-03	6.6887145	6.8369536	6.8315699	7.3497909	7.4205328	7.4224649
AT4G37470	436.66528	-0.59961	0.174279	-3.440515	5.81E-04	5.47E-03	8.5610131	8.198432	8.4771623	9.2962184	8.7841451	9.0462164
AT1G78420	264.48796	-0.599529	0.1480646	-4.049106	5.14E-05	6.67E-04	7.7929716	7.7663796	7.5918794	8.2302543	8.3593428	8.3741423
AT1G10200	617.84752	-0.599349	0.1432726	-4.183276	2.87E-05	4.01E-04	8.8917673	8.8527266	9.0658489	9.4412981	9.4419078	9.7298916
AT3G47680	131.22979	-0.598901	0.179466	-3.337128	8.46E-04	7.58E-03	6.7741583	6.6797225	6.6809735	7.1946821	7.4751844	7.2703267
AT1G02610	319.36658	-0.598042	0.1759571	-3.398792	0.0006768	0.0062527	8.2298384	7.8366024	7.8756292	8.3453619	8.6832965	8.7218449
AT2G03440	730.9658	-0.597781	0.1417385	-4.217493	2.47E-05	3.51E-04	9.2811879	8.9728301	9.2478064	9.9025153	9.6669415	9.7963981
AT5G04375	158.40487	-0.597745	0.1840807	-3.24719	1.17E-03	9.92E-03	6.8787626	6.9292741	7.1591252	7.3125489	7.6516422	7.7407813
AT4G28300	423.27615	-0.597686	0.1534803	-3.894221	9.85E-05	1.18E-03	8.5063565	8.2463272	8.4262144	8.8787641	8.9035279	9.1916847
AT2G34590	1182.4043	-0.596254	0.1464527	-4.071307	4.68E-05	6.14E-04	9.9536079	9.6513504	9.9848434	10.499458	10.321039	10.611526
AT5G05080	669.72266	-0.596091	0.1438054	-4.14512	3.40E-05	0.0004637	9.0777677	8.9343603	9.1488046	9.5887733	9.5187175	9.8510609
AT5G24160	178.41857	-0.595493	0.1721616	-3.45892	5.42E-04	0.0051592	7.0728423	7.2577626	7.0924916	7.7626156	7.9115001	7.6031803

AT3G26100	320.07348	-0.595117	0.1354756	-4.392797	1.12E-05	0.0001751	8.0035884	8.0314369	7.9147238	8.6883751	8.5748649	8.5430308
AT1G19400	1236.4126	-0.594169	0.1329031	-4.470691	7.80E-06	1.26E-04	10.00073	9.9026021	9.9166667	10.474384	10.391996	10.739704
AT4G00355	675.94167	-0.594166	0.1289365	-4.608206	4.06E-06	7.08E-05	9.1172937	8.9413055	9.1556204	9.549297	9.698867	9.7549533
AT3G09770	247.49959	-0.593844	0.1604879	-3.700239	2.15E-04	2.35E-03	7.5648279	7.4952618	7.7443407	8.4172953	8.1817745	8.1009928
AT1G62310	437.34701	-0.593516	0.1290921	-4.597612	4.27E-06	7.39E-05	8.5063565	8.485568	8.3402811	8.9772457	9.0945501	9.0586569
AT2G39400	533.96338	-0.592943	0.1772596	-3.345054	0.0008227	0.0074107	9.0437608	8.5025718	8.5555244	9.3705812	9.259324	9.3843743
AT3G53280	204.72118	-0.592721	0.1640558	-3.612925	3.03E-04	3.12E-03	7.4812945	7.2398472	7.2738258	8.1051601	7.8713123	7.9082392
AT3G26730	668.18897	-0.592664	0.1295181	-4.575915	4.74E-06	8.08E-05	9.0129947	9.0493964	9.0816813	9.7782602	9.4690905	9.7142498
AT1G78080	733.77804	-0.592232	0.1725075	-3.433079	5.97E-04	5.60E-03	9.4524762	8.8862243	9.1515348	9.8053233	9.6640039	9.9071717
AT3G52060	427.34361	-0.59005	0.1600515	-3.686622	2.27E-04	0.0024547	8.5884915	8.2259951	8.3850479	9.0355488	8.8424749	9.1486907
AT2G31810	1808.2253	-0.589115	0.1637449	-3.597757	3.21E-04	3.29E-03	10.713893	10.261749	10.448265	10.951064	10.990738	11.308522
AT3G14990	1661.8456	-0.588645	0.1418851	-4.14874	3.34E-05	0.0004579	10.438921	10.395711	10.272559	10.696632	11.051572	11.118096
AT4G09760	378.95457	-0.586944	0.1642754	-3.572925	0.000353	0.0035753	8.4475481	8.165595	8.0378796	8.9772457	8.8424749	8.7061151
AT5G05060	262.46873	-0.5869	0.1485358	-3.951236	7.77E-05	9.63E-04	7.6611467	7.7850307	7.7187966	8.1480747	8.307576	8.4374342
AT5G18640	454.92334	-0.586582	0.1401803	-4.184482	2.86E-05	0.0003997	8.5866758	8.4411808	8.5094454	8.8659674	9.1792882	9.2113183
AT2G46550	759.56703	-0.586434	0.1362288	-4.304778	1.67E-05	2.48E-04	9.0970284	9.305532	9.3041024	9.9087678	9.6698732	9.9293576
AT4G16760	966.92847	-0.584768	0.1213574	-4.818559	1.45E-06	2.81E-05	9.6019782	9.6914483	9.4784022	10.101225	10.279417	10.166224
AT5G16110	1855.2393	-0.584239	0.1542506	-3.787595	1.52E-04	0.0017348	10.789243	10.287595	10.458223	11.078552	11.090383	11.209492
AT4G34180	471.43776	-0.583797	0.1628924	-3.583945	0.0003384	0.0034515	8.7658441	8.4194596	8.4728028	8.9040219	9.2318137	9.2793346
AT5G51980	248.57146	-0.58293	0.1552311	-3.755244	1.73E-04	1.94E-03	7.608432	7.662618	7.6509564	8.1690626	8.0884486	8.4036994
AT1G58030	1176.7358	-0.581398	0.1046712	-5.55451	2.78E-08	7.56E-07	9.9507874	9.8160783	9.8602071	10.48279	10.471437	10.446195
AT4G34020	341.10487	-0.581335	0.1643662	-3.536826	4.05E-04	0.0040149	8.1656092	8.0699715	8.0757431	8.3453619	8.7063244	8.9189448
AT3G53030	287.0637	-0.580724	0.1453412	-3.995594	6.45E-05	0.0008163	7.8844925	7.8186104	7.8286383	8.4519603	8.2924378	8.5518581
AT3G59820	157.67457	-0.579643	0.1677019	-3.456391	5.47E-04	0.0052011	6.9312162	7.0828872	6.9110554	7.6174713	7.5534658	7.5688029
AT5G20650	303.29824	-0.579237	0.1766666	-3.278704	1.04E-03	9.02E-03	7.9166469	7.7283382	8.078615	8.6290213	8.238167	8.6578698
AT1G69830	823.30803	-0.578424	0.1671148	-3.46124	5.38E-04	5.13E-03	9.0490451	9.5294434	9.4376005	10.117512	9.7879153	9.9495384
AT3G15840	1135.6429	-0.576813	0.1478931	-3.9002	9.61E-05	1.16E-03	9.6687219	9.7151449	10.053597	10.495309	10.294692	10.454457
AT5G53130	512.73558	-0.57675	0.1320225	-4.368575	1.25E-05	0.000192	8.7317464	8.5249362	8.7732387	9.277013	9.278658	9.2525116
AT1G69270	285.46101	-0.576402	0.1385905	-4.15903	3.20E-05	0.0004405	7.7961177	7.8690161	7.8590219	8.3636843	8.5109065	8.3939142
AT5G05110	197.70021	-0.57283	0.1530957	-3.741648	1.83E-04	2.03E-03	7.3245343	7.3230308	7.2587115	7.9919327	7.8713123	7.8450065
AT3G13062	1001.3631	-0.572371	0.1248269	-4.585319	4.53E-06	7.77E-05	9.7817502	9.4939836	9.6580967	10.211552	10.212632	10.267495
AT1G59700	257.00971	-0.571646	0.150874	-3.788895	0.0001513	0.0017284	7.6402914	7.6286001	7.7658814	8.4172953	8.1230878	8.2869512
AT4G17100	257.75884	-0.571031	0.1689163	-3.380554	0.0007234	0.0066301	7.4773802	7.7283382	7.8080212	8.4858118	8.1230878	8.2330877

AT4G34710	1260.9914	-0.570645	0.1531493	-3.726068	0.0001945	0.0021445	9.9993661	9.9591277	9.959361	10.797795	10.598423	10.260781
AT5G47720	141.14816	-0.569739	0.173962	-3.275075	0.0010563	0.0091141	6.7155479	6.8953424	6.8518556	7.4560742	7.434391	7.3635206
AT4G16330	275.55666	-0.568981	0.1564084	-3.637791	2.75E-04	2.88E-03	7.899197	7.6826503	7.8183666	8.1897496	8.3520603	8.5341492
AT1G70160	952.96538	-0.5682	0.1166281	-4.871892	1.11E-06	2.20E-05	9.5572488	9.5685453	9.6003169	10.290289	10.026179	10.154681
AT1G04970	319.03328	-0.567136	0.1501367	-3.777463	1.58E-04	1.80E-03	7.8576407	8.0183599	8.095727	8.6883751	8.6300992	8.4374342
AT2G41430	6670.6109	-0.566771	0.1641693	-3.452355	0.0005557	0.0052682	12.535594	12.151325	12.431714	12.740477	12.901013	13.217728
AT5G22000	932.57373	-0.565839	0.1236163	-4.57738	4.71E-06	8.03E-05	9.673862	9.4410653	9.5180816	10.175706	10.16771	10.035441
AT3G16420	1445.0829	-0.56569	0.1400805	-4.038317	5.38E-05	0.0006923	10.321558	10.110986	10.105911	10.587836	10.709442	10.947973
AT2G44130	726.26924	-0.565572	0.1555276	-3.636475	0.0002764	0.0028936	9.4202148	9.005007	9.1058129	9.7225617	9.6757188	9.8933472
AT3G30380	167.27307	-0.564735	0.1711518	-3.299613	9.68E-04	8.48E-03	7.0307269	7.112725	7.0981635	7.5228149	7.8507907	7.560079
AT1G11910	4444.7011	-0.564222	0.0958235	-5.888141	3.91E-09	1.23E-07	11.720104	11.86267	11.826739	12.406722	12.377806	12.342027
AT1G78490	201.0382	-0.564025	0.1540838	-3.660506	2.52E-04	2.67E-03	7.2712702	7.3525075	7.4123039	7.8431732	7.9697576	7.9287155
AT2G44490	873.19362	-0.563994	0.1122765	-5.023259	5.08E-07	1.09E-05	9.5034094	9.4381223	9.4431822	9.921192	10.117055	10.035441
AT5G28840	2338.8357	-0.563264	0.1420259	-3.965928	7.31E-05	9.15E-04	11.013604	10.805415	10.803246	11.231043	11.453171	11.638258
AT5G10450	4501.4221	-0.562537	0.162962	-3.451954	5.57E-04	0.0052738	11.924758	11.622182	11.887776	12.178656	12.292879	12.673094
AT1G04280	469.29771	-0.561879	0.1701065	-3.3031	9.56E-04	0.0083913	8.8163005	8.4094771	8.4239579	9.0240752	9.0547314	9.3108774
AT2G34170	524.79991	-0.561582	0.1342807	-4.182149	2.89E-05	4.03E-04	8.7035408	8.6352662	8.8186069	9.3705812	9.1459878	9.3669735
AT1G78680	359.65204	-0.561205	0.1711543	-3.278941	1.04E-03	9.01E-03	8.0804351	8.2259951	8.1595047	9.091587	8.6300992	8.5162203
AT5G62540	238.40013	-0.561108	0.1643041	-3.415057	0.0006377	0.0059364	7.6611467	7.6147651	7.5176377	7.9919327	8.0619123	8.3791108
AT1G76460	268.74436	-0.560529	0.1482035	-3.782157	1.55E-04	0.0017685	7.8117462	7.7218992	7.7906123	8.1051601	8.4302147	8.4134187
AT5G01750	2114.6114	-0.560306	0.1113296	-5.032859	4.83E-07	1.04E-05	10.774963	10.613102	10.8119	11.263119	11.264362	11.377178
AT3G55130	579.49775	-0.559555	0.1329863	-4.20761	2.58E-05	3.65E-04	8.9732434	8.731358	8.8906427	9.4412981	9.3461903	9.5210075
AT4G36648	882.10216	-0.558825	0.152336	-3.668371	2.44E-04	2.59E-03	9.6037747	9.3490086	9.4509604	9.9578353	10.273647	9.8705961
AT1G23020	531.15951	-0.55861	0.1409884	-3.962095	7.43E-05	0.0009268	8.8873489	8.6058443	8.7355242	9.2077062	9.278658	9.4329649
AT5G63640	271.42535	-0.557695	0.1396301	-3.99409	6.49E-05	8.20E-04	7.8210426	7.7411304	7.7906123	8.2500881	8.3809723	8.3889965
AT3G61420	311.56267	-0.557556	0.1465025	-3.805779	1.41E-04	1.63E-03	8.085587	7.9289446	7.8756292	8.673764	8.4302147	8.5385969
AT1G68820	181.02164	-0.557548	0.1587474	-3.512169	4.44E-04	0.0043396	7.1970472	7.2353334	7.1806696	7.6174713	7.861088	7.7863742
AT1G29400	2001.9212	-0.556776	0.1033353	-5.388051	7.12E-08	1.82E-06	10.647948	10.68438	10.651429	11.124942	11.212024	11.318255
AT1G67530	221.6064	-0.555378	0.1513408	-3.669716	2.43E-04	2.58E-03	7.5462633	7.4608755	7.4213815	8.1690626	7.9602099	8.0458217
AT1G03400	272.91726	-0.553963	0.1673835	-3.309545	9.34E-04	0.0082365	7.9050372	7.5652703	7.8887788	8.1690626	8.4022823	8.4563629
AT5G41410	199.18444	-0.553799	0.1630842	-3.395785	6.84E-04	6.31E-03	7.2892443	7.4097132	7.2383096	8.1051601	7.8299729	7.7863742
AT5G22290	234.8191	-0.553619	0.1571419	-3.523053	4.27E-04	4.20E-03	7.6472767	7.4838905	7.5428118	8.2889558	7.9602099	8.1656791
AT2G01490	761.81223	-0.553415	0.1445432	-3.828716	1.29E-04	1.50E-03	9.4313862	9.1306353	9.2246582	9.7366892	9.7412935	9.9923186

AT5G51150	337.71882	-0.553386	0.1600297	-3.45802	0.0005442	0.005172	8.2368016	7.9729899	8.092889	8.3996454	8.6948564	8.8273979
AT3G22960	2808.1202	-0.553281	0.1151313	-4.805654	1.54E-06	2.97E-05	11.200915	11.01973	11.213734	11.737178	11.6018	11.784454
AT2G03340	287.60691	-0.552624	0.1528413	-3.615674	3.00E-04	3.10E-03	7.8455442	7.8125628	7.9082811	8.5512213	8.2062131	8.5162203
AT5G61530	423.76164	-0.552555	0.1223602	-4.515807	6.31E-06	1.05E-04	8.4654437	8.3913328	8.408063	8.9890964	8.9952029	8.9692328
AT3G45600	275.51642	-0.552511	0.1659662	-3.329056	0.0008714	0.0077674	7.7771374	7.6859621	7.8656878	8.6590034	8.2616763	8.1771349
AT3G50210	183.65705	-0.552413	0.16991	-3.251207	1.15E-03	9.80E-03	7.3632303	7.0978832	7.2071554	7.6174713	7.9015576	7.8012572
AT1G63800	654.26717	-0.551753	0.1412582	-3.905988	9.38E-05	0.0011359	9.0277908	8.9916871	9.1267754	9.5572792	9.4419078	9.8019585
AT3G60850	179.98195	-0.551373	0.1671123	-3.299417	0.0009689	0.0084853	7.3418608	7.1028474	7.1261931	7.6772924	7.7656542	7.8086415
AT4G19190	371.36104	-0.550962	0.147271	-3.741145	1.83E-04	0.0020365	8.1968915	8.3626467	8.1098335	8.8787641	8.6480505	8.8561899
AT1G12050	353.13986	-0.550791	0.1542849	-3.569959	3.57E-04	3.61E-03	8.0752647	8.1319932	8.2766794	8.6590034	8.5685951	8.9086734
AT5G43850	445.31248	-0.549952	0.1416459	-3.882583	0.0001034	0.0012328	8.4253699	8.4054647	8.6121191	9.2278502	8.9476798	8.9953531
AT3G08510	1307.9031	-0.54895	0.109417	-5.017049	5.25E-07	1.12E-05	9.9752812	10.122642	10.055056	10.511833	10.643861	10.651087
AT1G03380	533.34651	-0.547734	0.1222115	-4.481853	7.40E-06	0.0001202	8.7152217	8.8379162	8.7061127	9.3338789	9.3535024	9.2552165
AT4G24220	1461.9939	-0.547538	0.1238696	-4.42028	9.86E-06	1.56E-04	10.206266	10.122031	10.285045	10.904893	10.607625	10.778766
AT1G29760	277.13495	-0.545581	0.1461414	-3.73324	1.89E-04	2.09E-03	7.8962681	7.7474843	7.8010828	8.3636843	8.2694284	8.4703982
AT1G28580	239.74347	-0.544892	0.1496612	-3.640836	2.72E-04	2.85E-03	7.6952493	7.6077975	7.4963183	8.2101441	8.1145057	8.1714184
AT5G37780	535.45175	-0.543645	0.1363494	-3.987149	6.69E-05	8.43E-04	8.6593072	8.7863923	8.8625918	9.1455296	9.3388409	9.4377351
AT2G22430	1630.6297	-0.54357	0.1294144	-4.200226	2.67E-05	0.000375	10.33514	10.430187	10.323686	11.127624	10.777617	10.84849
AT5G18630	702.21364	-0.543322	0.1385645	-3.921074	8.82E-05	0.0010724	9.1618759	9.20177	9.1239979	9.4498989	9.7551629	9.8705961
AT1G35612	319.63775	-0.542146	0.1606072	-3.375602	7.37E-04	6.74E-03	8.1484797	7.8631766	8.0496361	8.5024443	8.4439806	8.7527991
AT2G26690	778.94399	-0.540989	0.1471574	-3.676262	0.0002367	0.0025379	9.1059295	9.4759213	9.2880635	10.011112	9.7879153	9.7739396
AT1G01770	188.49492	-0.539409	0.1572421	-3.430433	6.03E-04	5.65E-03	7.3504466	7.2125501	7.2331638	7.7626156	7.861088	7.8159882
AT5G04040	801.87388	-0.538592	0.1382097	-3.89692	9.74E-05	1.17E-03	9.4017456	9.3437762	9.3211779	9.6422861	9.9365128	10.066573
AT1G68490	459.52005	-0.538188	0.1404684	-3.831383	0.0001274	0.0014862	8.6627583	8.5614566	8.4307168	8.9164869	9.1501926	9.1888579
AT4G39140	208.28372	-0.537027	0.1583555	-3.391271	6.96E-04	0.0063947	7.3418608	7.3813939	7.4527106	8.1480747	7.8088504	7.9287155
AT3G17000	376.86235	-0.535662	0.1403117	-3.817659	1.35E-04	1.56E-03	8.3352462	8.1464899	8.269157	8.965297	8.7063244	8.7642378
AT2G18700	3057.8447	-0.5354	0.1163003	-4.603597	4.15E-06	7.22E-05	11.284497	11.285926	11.279484	11.685312	11.78534	11.984752
AT2G35940	778.26673	-0.534958	0.1523151	-3.512178	4.44E-04	4.34E-03	9.4089562	9.3594168	9.1100299	10.022689	9.8894996	9.6396152
AT5G26600	211.32988	-0.53482	0.1586356	-3.371378	7.48E-04	6.82E-03	7.445677	7.389542	7.3939756	8.2101441	7.8507907	7.8874681
AT3G11560	1473.209	-0.53421	0.1166633	-4.579072	4.67E-06	7.97E-05	10.236688	10.273354	10.185992	10.656324	10.725029	10.914143
AT4G34120	315.03206	-0.53316	0.1505444	-3.541549	3.98E-04	3.95E-03	8.0008641	7.9205343	8.0699823	8.6137933	8.6716432	8.3641539
AT1G67310	461.71009	-0.532718	0.1543354	-3.451687	5.57E-04	5.28E-03	8.5830376	8.5119324	8.6081494	8.7592901	9.2037686	9.2633007
AT1G56280	5129.5599	-0.532437	0.1616763	-3.293229	9.90E-04	8.64E-03	12.281124	11.75129	11.988173	12.697786	12.409285	12.601033

AT5G43930	285.68173	-0.531856	0.1453406	-3.659373	2.53E-04	2.68E-03	7.9761111	7.8034437	7.8388375	8.2889558	8.4163161	8.4980657
AT1G01820	501.4202	-0.531809	0.1385343	-3.838826	1.24E-04	1.44E-03	8.7738451	8.6283972	8.6512263	9.0125096	9.2397275	9.361963
AT3G03105	2589.7374	-0.5314	0.1519988	-3.496078	4.72E-04	0.0045582	11.224752	10.870536	10.996712	11.773606	11.373589	11.589257
AT1G45976	350.19822	-0.530545	0.14699	-3.609396	3.07E-04	3.16E-03	8.3265692	8.0236049	8.1126384	8.6883751	8.6948564	8.7218449
AT5G13590	477.72446	-0.530306	0.1342915	-3.948919	7.85E-05	9.72E-04	8.5957316	8.7217091	8.4793371	9.2178134	9.1627341	9.070991
AT1G09053	239.55143	-0.52998	0.147461	-3.594033	3.26E-04	3.34E-03	7.6262184	7.6354681	7.5838165	8.1051601	8.2694284	8.0828359
AT2G16600	2089.9132	-0.529947	0.0979264	-5.411689	6.24E-08	1.61E-06	10.754084	10.695513	10.758727	11.313472	11.198891	11.305915
AT2G42620	548.56557	-0.52943	0.1445331	-3.663038	2.49E-04	2.64E-03	8.9093067	8.8659272	8.6239629	9.3796127	9.2078086	9.4472285
AT1G24440	288.02057	-0.529375	0.1391945	-3.803129	1.43E-04	1.65E-03	7.899197	7.8514262	7.9018096	8.3817769	8.3520603	8.511703
AT4G21450	914.15685	-0.527803	0.1357942	-3.886784	1.02E-04	0.0012143	9.6984506	9.4143593	9.5085298	10.034174	9.9891016	10.210101
AT1G30360	1854.7686	-0.525566	0.1137539	-4.620202	3.83E-06	6.72E-05	10.571518	10.474908	10.649981	11.086848	11.007058	11.198291
AT1G75800	1405.8228	-0.525205	0.1549754	-3.38896	7.02E-04	0.0064435	10.249942	9.9118246	10.301732	10.614858	10.587611	10.88475
AT5G62350	690.13065	-0.522635	0.1344273	-3.887865	1.01E-04	1.21E-03	9.1691741	9.0532483	9.1705026	9.8835937	9.5854466	9.552136
AT3G11730	293.82042	-0.522518	0.1532576	-3.409407	6.51E-04	0.0060447	8.0063076	7.7569629	7.9621477	8.4519603	8.3299896	8.5474512
AT3G18035	892.74811	-0.521751	0.1381018	-3.778015	1.58E-04	1.79E-03	9.5034094	9.4033357	9.6338956	9.93963	9.9486343	10.222593
AT3G20770	2649.6261	-0.521582	0.1056708	-4.935915	7.98E-07	1.65E-05	11.185103	11.030517	11.034548	11.533762	11.64862	11.648582
AT1G75380	3811.592	-0.521181	0.1196761	-4.354933	1.33E-05	0.000203	11.499749	11.579637	11.736202	12.011516	12.158444	12.230992
AT3G22270	248.83814	-0.518266	0.1464849	-3.538015	4.03E-04	4.00E-03	7.7219626	7.7056751	7.5797681	8.2500881	8.238167	8.1366353
AT4G36400	204.60571	-0.518164	0.1524221	-3.399535	6.75E-04	0.0062384	7.3967765	7.4016785	7.3847234	7.9440305	7.8299729	7.9949442
AT2G45380	536.99431	-0.517439	0.1195744	-4.327336	1.51E-05	0.0002264	8.8424046	8.7599234	8.7571959	9.2866477	9.2632116	9.3744567
AT5G47040	1406.8771	-0.51713	0.1146337	-4.511153	6.45E-06	1.07E-04	10.155726	10.155863	10.217602	10.587836	10.65426	10.833183
AT1G65580	541.8036	-0.516327	0.1474703	-3.501227	4.63E-04	0.0044867	8.6730625	9.0129403	8.6741917	9.3057261	9.3388409	9.3289639
AT3G10420	797.21839	-0.513752	0.1397359	-3.676594	2.36E-04	2.54E-03	9.2274336	9.3521389	9.4509604	10.079219	9.8225776	9.7298916
AT2G14080	1026.6399	-0.513111	0.1296717	-3.956997	7.59E-05	9.43E-04	9.7880845	9.7739051	9.5741011	10.360396	10.263979	10.103065
AT2G21620	484.85207	-0.513016	0.1327752	-3.863794	0.0001116	0.001322	8.5775629	8.6641001	8.639605	9.3523467	9.0813989	9.0586569
AT3G17020	1030.7628	-0.512841	0.1123736	-4.56371	5.03E-06	8.50E-05	9.6479766	9.7296597	9.8014476	10.256369	10.175983	10.307137
AT3G17800	916.06416	-0.512683	0.1193139	-4.296922	1.73E-05	2.56E-04	9.526324	9.468248	9.6600153	10.165298	9.9961265	10.074252
AT2G23170	384.14683	-0.511252	0.127363	-4.014134	5.97E-05	7.59E-04	8.2733816	8.3333786	8.2891302	8.9164869	8.8002886	8.7718135
AT5G39590	1051.4306	-0.511252	0.1023645	-4.994427	5.90E-07	1.25E-05	9.7577456	9.7893838	9.7256096	10.280679	10.236558	10.307137
AT2G01100	551.64925	-0.511237	0.1324626	-3.859483	1.14E-04	0.0013373	8.9352224	8.8644664	8.6760891	9.277013	9.3644016	9.3917681
AT1G72160	1361.2886	-0.51091	0.1268148	-4.028789	5.61E-05	7.19E-04	9.9745871	10.191835	10.195945	10.787653	10.565742	10.590261
AT3G49590	428.35685	-0.507973	0.1449729	-3.503917	4.58E-04	4.45E-03	8.453538	8.3312651	8.5901495	9.0240752	8.8372686	9.0771187
AT3G27020	1121.2445	-0.506945	0.1247552	-4.063518	4.83E-05	6.33E-04	9.8014533	9.8069815	9.9444826	10.351243	10.234579	10.50078

AT4G02510	4370.5681	-0.506748	0.1394059	-3.635054	2.78E-04	2.91E-03	11.894621	11.960093	11.545435	12.3061	12.394739	12.287577
AT2G15900	419.55037	-0.506398	0.1252158	-4.044205	5.25E-05	0.0006783	8.4495475	8.4798552	8.3827261	8.9040219	9.0091599	8.9325272
AT3G16950	616.86315	-0.505831	0.1124405	-4.498651	6.84E-06	1.12E-04	9.0237705	8.9863245	8.9732083	9.4669481	9.4957705	9.5455219
AT3G10490	247.99527	-0.505153	0.1464553	-3.449194	5.62E-04	5.32E-03	7.7481902	7.662618	7.6470923	8.1051601	8.1652491	8.2922286
AT3G13670	764.13936	-0.50509	0.117009	-4.316676	1.58E-05	2.36E-04	9.3470224	9.3290237	9.2324156	9.7576244	9.763421	9.9071717
AT3G53990	1076.4085	-0.50397	0.1151748	-4.375695	1.21E-05	1.87E-04	9.7943912	9.7021191	9.8842062	10.30932	10.232598	10.373369
AT1G32130	638.39554	-0.503919	0.1261209	-3.995519	6.46E-05	0.0008163	9.0048597	9.143942	8.9900847	9.4063729	9.6161692	9.616661
AT5G41370	470.26099	-0.503276	0.1366215	-3.683727	2.30E-04	2.48E-03	8.5330011	8.7409428	8.5136958	9.1455296	9.0275616	9.1689141
AT1G28960	368.63937	-0.502081	0.1527026	-3.287965	1.01E-03	0.0087824	8.2205015	8.2883321	8.2101439	8.9532484	8.5043519	8.8201092
AT3G14830	226.81111	-0.499464	0.1519422	-3.2872	1.01E-03	8.80E-03	7.6012556	7.5724458	7.4482765	8.1897496	8.0257537	7.9949442
AT4G05050	2300.8293	-0.498833	0.1169632	-4.264873	2.00E-05	0.0002903	10.890224	10.745571	11.029733	11.382487	11.429148	11.387077
AT2G39950	308.84121	-0.494718	0.1453951	-3.402574	6.68E-04	0.0061801	7.945269	7.9675572	8.1154378	8.3268039	8.5935123	8.5606317
AT2G33770	845.36644	-0.493559	0.1195683	-4.127845	3.66E-05	0.0004963	9.386172	9.5248272	9.4308739	10.028443	10.000791	9.8348784
AT1G50640	371.28612	-0.493417	0.1520069	-3.24602	0.0011703	0.0099535	8.2913295	8.1124349	8.401197	8.6440903	8.7233573	8.9155291
AT3G23920	616.64531	-0.493164	0.1162523	-4.242187	2.21E-05	0.0003182	9.0251118	9.0260663	8.9293379	9.5731122	9.4657205	9.46603
AT3G49490	650.75884	-0.492713	0.1265967	-3.891991	9.94E-05	0.0011924	9.0660865	9.0583682	9.1267754	9.4063729	9.5792227	9.7122826
AT4G29950	1136.2577	-0.492303	0.1472517	-3.343277	0.000828	0.0074341	9.9828942	9.8348479	9.8238708	10.084752	10.540337	10.46851
AT4G37790	234.89922	-0.492065	0.1463043	-3.363298	7.70E-04	7.00E-03	7.5685124	7.6007961	7.6276151	8.2302543	8.0348786	8.0828359
AT5G04170	494.41034	-0.491038	0.1251843	-3.922522	8.76E-05	0.0010671	8.720199	8.6005893	8.7208934	9.1560802	9.259324	9.1222656
AT1G32230	2105.7732	-0.4908	0.1393233	-3.522744	0.0004271	0.0042023	10.695478	10.851122	10.756488	11.127624	11.499224	11.15042
AT4G36760	679.42486	-0.490771	0.115514	-4.248585	2.15E-05	3.10E-04	9.0699908	9.1796212	9.1745348	9.6042663	9.6039585	9.6984366
AT1G23870	969.2111	-0.490525	0.1442103	-3.401453	6.70E-04	6.20E-03	9.7769811	9.6597966	9.5074646	10.051231	10.030747	10.344449
AT5G56240	579.39776	-0.489386	0.125697	-3.893381	9.89E-05	1.19E-03	8.9732434	8.8571402	8.8758602	9.549297	9.3015218	9.379424
AT3G25910	905.7462	-0.489383	0.1187203	-4.122154	3.75E-05	5.07E-04	9.6670045	9.5155501	9.4816604	9.9994417	10.019299	10.128366
AT2G02570	449.96909	-0.487606	0.1499332	-3.252157	0.0011453	0.0097749	8.4049077	8.7457114	8.46843	8.9772457	9.1290449	9.0210089
AT3G56130	742.95025	-0.487479	0.1259826	-3.869416	1.09E-04	0.0012957	9.2867903	9.1306353	9.3840499	9.7506797	9.7244723	9.8185124
AT4G34890	676.20888	-0.486459	0.1432536	-3.395792	0.0006843	0.006306	9.0089329	9.2178753	9.1812304	9.4326457	9.8146522	9.6082226
AT1G34220	349.61114	-0.486404	0.1383079	-3.516819	4.37E-04	0.0042826	8.1533946	8.1099713	8.2940806	8.6440903	8.6300992	8.7566221
AT3G56310	818.63948	-0.484614	0.1062244	-4.562175	5.06E-06	8.55E-05	9.4494822	9.3718083	9.4127815	9.921192	9.8995253	9.8916098
AT1G72710	1190.7412	-0.484416	0.1229567	-3.939731	8.16E-05	1.01E-03	10.056237	9.8251182	9.9709998	10.374017	10.397313	10.550074
AT4G02880	673.1607	-0.483932	0.1191051	-4.06307	4.84E-05	6.34E-04	9.0842165	9.1147494	9.173192	9.7154457	9.6432711	9.5119888
AT3G55770	1750.0161	-0.483873	0.1314837	-3.680101	2.33E-04	2.51E-03	10.60778	10.336357	10.558829	11.033485	10.855648	11.100154
AT3G23490	795.17278	-0.483131	0.1272851	-3.795664	1.47E-04	1.69E-03	9.4414675	9.2584817	9.4070808	9.8579725	9.7440781	9.9727314

AT4G11960	437.64481	-0.482776	0.1262974	-3.822531	0.0001321	0.0015358	8.515929	8.4969261	8.4923177	9.1349012	8.9134569	8.9659342
AT5G26751	677.52137	-0.481276	0.1185493	-4.059706	4.91E-05	0.0006415	9.2122048	9.039074	9.163757	9.6422861	9.6581105	9.5911962
AT1G24160	916.59818	-0.480749	0.1246891	-3.855577	1.15E-04	1.36E-03	9.4976234	9.721614	9.5074646	9.9817571	10.080067	10.125412
AT1G66410	747.25335	-0.479619	0.1148996	-4.174243	2.99E-05	4.15E-04	9.2608371	9.2717671	9.3053288	9.8644206	9.6610602	9.7870828
AT4G36980	768.11351	-0.478895	0.1424222	-3.362505	0.0007724	0.007015	9.2642489	9.4892522	9.1838999	9.8579725	9.906999	9.6642466
AT1G80300	479.05073	-0.478458	0.1443922	-3.313598	0.000921	0.0081375	8.5082761	8.7136187	8.6608397	9.3431423	9.0137824	9.0368151
AT5G48385	709.00494	-0.477421	0.126074	-3.786834	0.0001526	0.0017383	9.3188622	9.1208801	9.173192	9.7437014	9.5854466	9.7511259
AT2G30440	1245.6864	-0.477408	0.134611	-3.546577	3.90E-04	3.89E-03	10.190213	9.8968973	9.9648041	10.355826	10.581397	10.566501
AT3G23640	1209.3419	-0.476941	0.1041355	-4.580004	4.65E-06	7.95E-05	9.9676279	9.9673166	10.012138	10.40088	10.439153	10.537907
AT5G16160	372.09426	-0.476072	0.1446639	-3.290884	9.99E-04	8.70E-03	8.2321632	8.2970215	8.3063828	8.8136145	8.5685951	8.8983283
AT4G36195	449.71205	-0.474918	0.1331941	-3.56561	0.000363	0.0036536	8.4253699	8.5811556	8.6550793	8.9772457	9.0813989	9.0462164
AT2G19270	309.49083	-0.474023	0.1403627	-3.37713	7.32E-04	0.0067021	8.0008641	7.9317372	8.1238035	8.4347319	8.523927	8.5296878
AT3G27260	836.20347	-0.469826	0.1131341	-4.152826	3.28E-05	4.51E-04	9.4161309	9.5211235	9.4025039	10.011112	9.8692369	9.9088904
AT2G24280	515.5185	-0.468761	0.1235389	-3.794443	0.000148	0.0016955	8.7642385	8.7661952	8.7571959	9.0805521	9.3277462	9.2579163
AT4G32040	387.7206	-0.467431	0.1397989	-3.343593	8.27E-04	7.43E-03	8.443541	8.2440822	8.3663678	8.6590034	8.8682284	8.9052333
AT1G18470	472.35131	-0.467044	0.1253207	-3.726792	0.0001939	0.0021398	8.720199	8.5793759	8.5981772	9.0355488	9.1247779	9.1428601
AT3G05120	388.81011	-0.465054	0.1431743	-3.248165	0.0011615	0.0098864	8.453538	8.2817805	8.3378861	8.6590034	8.8002886	8.9758073
AT1G47128	3782.8995	-0.462925	0.1191604	-3.884892	1.02E-04	1.22E-03	11.546735	11.758582	11.574462	12.221982	11.996235	12.081653
AT2G43680	1018.4363	-0.46171	0.1246036	-3.705428	2.11E-04	2.31E-03	9.8769379	9.7053866	9.617204	10.256369	10.242478	10.131314
AT4G29010	1413.1586	-0.460937	0.1150145	-4.007639	6.13E-05	0.0007782	10.10462	10.273905	10.245342	10.784256	10.590708	10.667424
AT5G54940	1488.2367	-0.460653	0.1349946	-3.412381	6.44E-04	5.99E-03	10.474704	10.24276	10.111531	10.831096	10.686464	10.752205
AT1G09910	324.21057	-0.460116	0.1389891	-3.310447	9.31E-04	8.21E-03	8.0491313	8.0951011	8.1567896	8.4347319	8.5432401	8.6741315
AT3G15730	2837.8945	-0.459664	0.1132531	-4.058729	4.93E-05	6.44E-04	11.233481	11.214625	11.200941	11.840545	11.558955	11.648582
AT1G21450	603.18267	-0.459321	0.1207866	-3.80275	1.43E-04	1.65E-03	8.9980453	9.0234507	8.9592521	9.3245555	9.4589569	9.552136
AT1G55310	489.95144	-0.458841	0.1366753	-3.357161	0.0007875	0.0071344	8.8378322	8.650603	8.5616954	9.124194	9.1290449	9.2057359
AT3G59940	4631.0731	-0.457687	0.133114	-3.438311	5.85E-04	5.51E-03	12.029553	11.763097	11.970308	12.31201	12.284296	12.562788
AT3G61600	1160.866	-0.456938	0.1282055	-3.564107	3.65E-04	0.0036681	9.8850863	9.8422878	10.059423	10.374017	10.279417	10.522273
AT2G22720	659.83419	-0.454772	0.1312144	-3.465867	0.0005285	0.0050495	9.2015669	9.0887121	9.0629515	9.580964	9.4350313	9.7122826
AT2G38800	1129.2331	-0.454651	0.1310619	-3.468978	5.22E-04	0.0049957	9.7849208	10.069113	9.7962231	10.422891	10.361495	10.284807
AT3G13060	366.41588	-0.453879	0.1372723	-3.306411	0.000945	0.0083126	8.2228414	8.2663768	8.3210083	8.7730642	8.5935123	8.8273979
AT1G27100	915.58914	-0.453458	0.1397454	-3.244883	1.17E-03	9.99E-03	9.6772785	9.5257516	9.5832298	9.7850737	10.161475	10.166224
AT5G63195	2268.1704	-0.451854	0.123576	-3.656485	0.0002557	0.0027038	10.893165	10.84632	10.957467	11.382487	11.179479	11.499141
AT4G01000	953.02765	-0.451797	0.1386884	-3.257638	1.12E-03	9.61E-03	9.6891731	9.7183831	9.5453256	9.9758138	10.012386	10.309741

AT5G63190	3519.0682	-0.450954	0.1150372	-3.920067	8.85E-05	1.08E-03	11.524382	11.491536	11.585609	12.002775	11.841674	12.118885
AT3G26165	344.06189	-0.449465	0.1362613	-3.298548	0.0009719	0.0085048	8.1385993	8.1416739	8.2816726	8.5512213	8.7120244	8.6455522
AT5G45500	576.58077	-0.447277	0.1376699	-3.248911	1.16E-03	0.0098681	9.0170451	8.935752	8.8441462	9.1975278	9.5476944	9.3719666
AT1G04440	681.96872	-0.443047	0.1200053	-3.691892	2.23E-04	0.0024139	9.2378832	9.1595114	9.1350758	9.5004538	9.7244723	9.6250503
AT2G25490	943.11049	-0.440734	0.1141004	-3.862682	1.12E-04	0.0013266	9.7043235	9.5721294	9.6329191	10.180882	10.088856	10.002013
AT3G09275	4435.1164	-0.43975	0.1346596	-3.265643	1.09E-03	0.0093864	11.967419	11.696105	11.942005	12.23821	12.220744	12.490595
AT1G14000	430.11178	-0.436238	0.1341094	-3.252849	1.14E-03	9.76E-03	8.4986525	8.4645096	8.5780235	8.8914483	9.0945501	8.8703732
AT3G63500	991.0578	-0.434705	0.1219691	-3.564057	3.65E-04	0.0036681	9.7085039	9.8430297	9.5832298	10.165298	10.194424	10.116515
AT3G16857	633.52299	-0.434083	0.1303452	-3.330258	8.68E-04	7.74E-03	9.0424367	9.183141	9.0022355	9.3705812	9.5154616	9.6292267
AT2G44100	1064.9197	-0.432462	0.1025741	-4.216093	2.49E-05	3.53E-04	9.8544779	9.7863013	9.8195856	10.30932	10.224645	10.249973
AT4G24020	801.73081	-0.430582	0.1110124	-3.878684	1.05E-04	1.25E-03	9.4222524	9.3645929	9.4608997	9.7918552	9.9169042	9.8348784
AT3G22970	884.98244	-0.426467	0.1298016	-3.285526	1.02E-03	0.0088448	9.6549249	9.5071493	9.4849113	10.165298	9.8692369	9.9394833
AT5G53350	817.17114	-0.424424	0.1192149	-3.560163	3.71E-04	3.71E-03	9.3340936	9.4653599	9.5170234	9.9638531	9.8093443	9.8581953
AT1G50480	2074.5382	-0.42363	0.1111231	-3.812257	0.0001377	0.0015943	10.863104	10.686448	10.812332	11.198238	11.144077	11.305915
AT4G18120	543.32387	-0.423472	0.1236345	-3.42519	0.0006144	0.0057502	8.9194404	8.8289564	8.8424576	9.1455296	9.3680164	9.3238194
AT5G58200	412.86086	-0.423379	0.1267454	-3.340389	8.37E-04	7.50E-03	8.4314524	8.4912582	8.4463653	8.9772457	8.8002886	8.9052333
AT5G44290	465.74518	-0.42259	0.1235179	-3.421288	6.23E-04	0.005827	8.6985056	8.6110802	8.5981772	9.0355488	9.1290449	9.0273522
AT3G24503	820.28503	-0.42232	0.1248904	-3.381528	7.21E-04	6.61E-03	9.5460799	9.4143593	9.4036495	9.7576244	9.8512705	10.010043
AT1G30820	815.87783	-0.421472	0.1225046	-3.440459	5.81E-04	5.47E-03	9.4474827	9.5127553	9.3547344	9.9758138	9.7440781	9.8968158
AT4G32850	665.87607	-0.415093	0.1223062	-3.393879	6.89E-04	6.34E-03	9.1667455	9.2213031	9.0916668	9.4584487	9.5698364	9.680437
AT4G30600	958.70645	-0.414305	0.1092406	-3.792589	1.49E-04	1.71E-03	9.7184876	9.6889746	9.6377952	10.062491	10.184208	10.051091
AT3G52240	713.0473	-0.411983	0.125448	-3.284096	1.02E-03	8.88E-03	9.361961	9.2144392	9.1892242	9.6422861	9.5916437	9.777707
AT3G57300	1091.6488	-0.411881	0.1176147	-3.501949	4.62E-04	4.48E-03	9.8642535	9.9481363	9.8049201	10.165298	10.283251	10.395606
AT3G51370	714.90581	-0.411052	0.1169146	-3.515827	4.38E-04	4.29E-03	9.292371	9.2947262	9.2011325	9.6195946	9.6312886	9.7682699
AT5G42950	1242.3332	-0.409465	0.1157822	-3.53651	4.05E-04	4.02E-03	10.066044	10.131171	9.9601399	10.56822	10.370533	10.480118
AT3G27090	2198.0577	-0.409057	0.1086853	-3.763686	0.0001674	0.0018822	10.971089	10.769963	10.889588	11.352985	11.238924	11.294127
AT5G59420	856.43826	-0.40813	0.120304	-3.392488	6.93E-04	6.37E-03	9.5938658	9.534045	9.4465208	9.8053233	10.039841	9.9445196
AT3G22380	2129.011	-0.407663	0.1036638	-3.932549	8.40E-05	1.03E-03	10.794759	10.938756	10.768098	11.231043	11.255607	11.257497
AT4G32060	1533.6215	-0.406322	0.1206348	-3.368201	7.57E-04	6.89E-03	10.419672	10.269496	10.400864	10.689386	10.702301	10.917562
AT2G07180	645.75777	-0.400362	0.1203088	-3.327786	0.0008754	0.0077903	9.1788481	9.0986866	9.1001711	9.3796127	9.5885484	9.5847592
AT3G48000	1092.1637	-0.400357	0.1227736	-3.260941	1.11E-03	9.53E-03	9.7464053	9.9598119	9.9150609	10.290289	10.184208	10.36963
AT1G15740	1489.3489	-0.399711	0.122517	-3.262496	1.10E-03	0.0094839	10.32755	10.421769	10.216298	10.725255	10.61068	10.844903
AT3G45190	591.4069	-0.391443	0.1184122	-3.305768	9.47E-04	8.33E-03	9.0529956	9.0129403	8.9165545	9.4326457	9.407193	9.3518896

AT4G19110	937.23341	-0.382879	0.1069673	-3.579404	0.0003444	0.0035039	9.6427433	9.7053866	9.6426549	10.106675	10.071224	9.9906965
AT2G27860	1996.7936	-0.38287	0.1147999	-3.335105	0.0008527	0.007625	10.774963	10.638965	10.844323	11.223539	11.045941	11.164877
AT5G65670	1291.2901	-0.382301	0.1119411	-3.415197	6.37E-04	0.0059364	10.057548	10.134205	10.181992	10.622487	10.41664	10.511
AT1G26830	749.78304	-0.380841	0.1137104	-3.349219	8.10E-04	7.31E-03	9.3243205	9.3718083	9.3523632	9.6793296	9.6815407	9.8239884
AT3G60190	756.87555	-0.377984	0.1149433	-3.288443	1.01E-03	0.0087709	9.300148	9.4083569	9.3852102	9.6719966	9.7272895	9.8221654
AT3G09840	3677.5538	-0.346415	0.0926588	-3.738615	1.85E-04	2.05E-03	11.616912	11.686837	11.670384	12.067081	11.95777	12.003373
AT5G05690	3591.6041	-0.344109	0.1037409	-3.317002	9.10E-04	8.06E-03	11.614018	11.658669	11.609634	11.879267	11.945121	12.087736
AT1G16890	1059.096	-0.34407	0.1026515	-3.351826	8.03E-04	7.26E-03	9.8961242	9.824367	9.8718427	10.241585	10.173919	10.226734
AT3G11130	2418.257	0.3483067	0.1070232	3.2544972	1.14E-03	0.0097097	11.285337	11.480612	11.445804	11.036344	11.077194	11.044168
AT2G41840	2362.411	0.3493843	0.1052887	3.318347	9.06E-04	8.03E-03	11.272688	11.453907	11.384752	11.047721	10.973045	11.036327
AT5G20290	3197.4362	0.3544627	0.1013999	3.4956921	4.73E-04	0.0045628	11.76093	11.756418	11.910317	11.482286	11.427417	11.449225
AT5G02960	1929.8722	0.3646577	0.1019143	3.5780821	0.0003461	0.0035185	11.139387	11.000574	11.119271	10.728793	10.696562	10.730986
AT1G05850	3487.0153	0.3653737	0.1112543	3.2841311	1.02E-03	0.0088794	11.975247	11.825427	12.0184	11.567745	11.486809	11.652178
AT3G58610	3021.6469	0.3694038	0.1127275	3.276961	1.05E-03	9.07E-03	11.611341	11.741177	11.850667	11.325206	11.3307	11.424806
AT4G39080	1439.5059	0.370308	0.1001885	3.6961139	0.0002189	0.0023811	10.610463	10.701662	10.688559	10.295071	10.29659	10.287452
AT5G26742	1751.5705	0.3743853	0.1080205	3.4658718	5.29E-04	5.05E-03	11.005812	10.902204	10.940202	10.67845	10.478142	10.565412
AT4G15000	1048.9392	0.3754679	0.1157152	3.2447598	1.18E-03	0.0099899	10.155113	10.152874	10.327312	9.8449892	9.7986696	9.8546325
AT3G22890	1463.9907	0.3777719	0.0995303	3.7955459	1.47E-04	1.69E-03	10.689145	10.743189	10.652875	10.299836	10.317305	10.320112
AT5G39740	1815.2158	0.3793136	0.107416	3.5312572	4.14E-04	4.09E-03	10.985974	11.050247	10.97144	10.746355	10.554682	10.568677
AT3G08580	3861.9267	0.3879039	0.1073044	3.6149876	3.00E-04	3.10E-03	12.078986	12.038455	12.172181	11.804113	11.599496	11.710007
AT2G36910	820.10443	0.3932877	0.117876	3.336453	8.49E-04	7.59E-03	9.8053619	9.9529553	9.8281432	9.5572792	9.4315808	9.4257798
AT1G72370	1923.4465	0.396483	0.1038474	3.8179366	1.35E-04	0.0015622	11.095051	11.027583	11.160176	10.760253	10.695124	10.6356
AT3G62870	1980.2577	0.4053809	0.1121263	3.6153959	0.0002999	0.0031013	11.088009	11.10168	11.22997	10.834385	10.628873	10.732928
AT2G39770	1075.502	0.4067041	0.1185947	3.4293625	6.05E-04	5.67E-03	10.343228	10.217236	10.225397	9.9025153	9.7244723	9.9208649
AT2G39460	1149.5069	0.4067576	0.1141439	3.5635499	3.66E-04	0.0036719	10.371976	10.348425	10.350665	10.005289	10.023889	9.8130155
AT2G09990	708.44023	0.4085211	0.1225783	3.3327357	8.60E-04	7.68E-03	9.7076688	9.5532129	9.7274406	9.2278502	9.2078086	9.3004396
AT2G22125	1535.0799	0.4093651	0.123164	3.3237391	8.88E-04	0.0078915	10.703459	10.877432	10.759175	10.191178	10.474793	10.390694
AT1G15930	1324.2233	0.409654	0.1136524	3.604447	3.13E-04	3.22E-03	10.597898	10.458542	10.639323	10.079219	10.148922	10.20731
AT1G75680	485.787	0.4114663	0.1242341	3.3120231	9.26E-04	8.17E-03	9.1298165	9.0874605	9.1610499	8.5984029	8.7623357	8.7218449
AT5G64740	1541.2975	0.4117221	0.1009354	4.0790656	4.52E-05	5.96E-04	10.795938	10.803129	10.759623	10.290289	10.427074	10.373369
AT2G23350	850.53909	0.4137985	0.1108316	3.7335784	1.89E-04	2.09E-03	9.8850863	9.9550157	9.9546792	9.4326457	9.5187175	9.55872
AT5G56710	891.98872	0.4155395	0.1148812	3.6171226	2.98E-04	3.08E-03	10.068648	9.970715	9.9397521	9.6719966	9.5380999	9.5299701
AT5G15200	2452.4698	0.4169762	0.1193748	3.493001	4.78E-04	4.60E-03	11.470755	11.302203	11.585104	11.036344	10.981329	11.078936

AT5G23740	846.18301	0.4199106	0.1157244	3.6285412	0.000285	0.0029685	9.9244337	9.8385726	10.007625	9.4753978	9.4891466	9.5232533
AT4G26230	617.60073	0.421005	0.1210101	3.4790891	5.03E-04	4.82E-03	9.5139571	9.4759213	9.4047942	9.1455296	9.0275616	8.9725238
AT1G04270	1608.7108	0.4212834	0.1091138	3.8609536	0.0001129	0.0013328	10.86685	10.768011	10.918727	10.355826	10.404371	10.497357
AT2G27530	1463.0439	0.4213067	0.1018589	4.1361782	3.53E-05	4.80E-04	10.755701	10.671907	10.717406	10.221633	10.304157	10.323982
AT4G18700	904.88365	0.4216804	0.1147117	3.6760023	2.37E-04	0.002538	9.9718075	10.067845	10.009883	9.7082944	9.5056496	9.5761316
AT1G08360	1524.4396	0.423107	0.1109517	3.8134329	0.000137	0.0015892	10.747192	10.749929	10.83076	10.261264	10.29659	10.456808
AT3G49010	1257.1764	0.4234792	0.1094631	3.8686944	0.0001094	0.0012989	10.452444	10.443945	10.588851	10.039882	10.106274	10.049533
AT4G21105	564.17931	0.4242217	0.1270922	3.3379059	0.0008441	0.0075608	9.2699173	9.3300825	9.4376005	8.800224	8.9525032	8.9526641
AT5G27850	1764.5749	0.4295841	0.1060344	4.051364	5.09E-05	0.0006613	10.98149	10.955661	11.025275	10.552333	10.457932	10.637674
AT1G18080	2052.5183	0.4307719	0.108299	3.9776172	6.96E-05	8.76E-04	11.15448	11.133866	11.321305	10.774018	10.783043	10.750289
AT5G62300	1275.1828	0.4322712	0.124069	3.4841206	0.0004938	0.004746	10.549782	10.37688	10.622694	10.133618	9.9820423	10.125412
AT3G25520	2560.0924	0.4379132	0.0913919	4.7915979	1.65E-06	3.17E-05	11.535013	11.51965	11.525841	11.089602	11.047069	11.115865
AT3G56340	791.55148	0.4401533	0.1175634	3.7439648	1.81E-04	2.02E-03	9.8739633	9.7392558	9.8842062	9.4498989	9.3859559	9.3417453
AT5G08690	1430.3225	0.4418049	0.1094987	4.0347957	5.46E-05	7.02E-04	10.580669	10.759785	10.722464	10.24653	10.244446	10.234979
AT2G18300	1943.5223	0.4426895	0.1094089	4.0461915	5.21E-05	0.0006738	11.156624	11.06971	11.172325	10.648873	10.59996	10.789069
AT3G18820	633.87631	0.4435652	0.1315427	3.3720243	0.0007462	0.0068022	9.5319962	9.4566609	9.5882765	8.8530562	9.1833972	9.1103639
AT5G14800	599.93215	0.4437885	0.1242757	3.5709995	3.56E-04	3.60E-03	9.5139571	9.3645929	9.4409521	8.965297	8.9282233	9.0586569
AT2G18020	1763.3697	0.4443765	0.1247316	3.5626607	0.0003671	0.0036827	10.917204	10.88141	11.162884	10.587836	10.538734	10.498498
AT4G34350	2416.6859	0.4486475	0.1090862	4.1127806	3.91E-05	5.25E-04	11.437142	11.40927	11.49527	11.084088	10.862071	11.03239
AT1G34030	634.73476	0.4495115	0.1298417	3.4619971	5.36E-04	5.11E-03	9.6171781	9.4083569	9.5212515	9.1769528	9.0502385	8.982352
AT3G44010	757.81114	0.450426	0.1362914	3.3048758	9.50E-04	8.35E-03	9.8612527	9.5916842	9.8618751	9.3796127	9.3425203	9.2416412
AT2G34480	2008.826	0.4519169	0.1118771	4.0394037	5.36E-05	6.90E-04	11.167901	11.077296	11.295309	10.774018	10.726438	10.67855
AT1G70940	1404.3795	0.4526968	0.1285817	3.5206932	0.0004304	0.0042293	10.686605	10.663952	10.671065	10.011112	10.175983	10.390694
AT3G47370	374.22256	0.4527889	0.1364259	3.3189359	9.04E-04	8.01E-03	8.7350287	8.7281488	8.8424576	8.1897496	8.3952139	8.2922286
AT3G04840	1402.7224	0.4530912	0.1203307	3.7653818	1.66E-04	0.0018714	10.549782	10.6914	10.747944	10.256369	10.271718	10.091003
AT3G52590	1970.1847	0.4535112	0.1122963	4.0385242	5.38E-05	0.0006921	11.153867	11.069076	11.246664	10.637624	10.649812	10.790002
AT1G06410	1315.885	0.4535973	0.1285948	3.5273374	0.0004198	0.004143	10.461884	10.733219	10.517627	10.095755	10.200519	10.038585
AT3G04920	1012.5191	0.4574072	0.1085186	4.2150126	2.50E-05	3.54E-04	10.185422	10.167756	10.250445	9.6497713	9.771632	9.766375
AT1G23300	1089.2497	0.4574196	0.128002	3.5735334	3.52E-04	3.57E-03	10.276653	10.226931	10.419106	9.6572178	9.8844606	9.9378006
AT1G23290	1087.7758	0.4577543	0.1278401	3.5806795	3.43E-04	0.0034885	10.274399	10.224656	10.417972	9.6572178	9.8819344	9.9344293
AT5G08650	546.04485	0.4598849	0.1216477	3.7804654	1.57E-04	1.78E-03	9.2562754	9.2914686	9.3759014	8.8787641	8.8215359	8.83465
AT1G56570	499.08248	0.4606311	0.133464	3.4513518	5.58E-04	5.28E-03	9.1285691	9.1511488	9.2541711	8.7453832	8.8109514	8.582335
AT5G19190	971.52166	0.4613745	0.1359255	3.3943184	6.88E-04	6.33E-03	10.290663	9.9788386	10.150948	9.5412705	9.7103033	9.7376494

AT1G05190	1947.6157	0.4639751	0.1363607	3.4025565	0.0006676	0.0061801	11.065697	11.048318	11.310352	10.67845	10.516106	10.803924
AT4G13495	1887.0211	0.4655007	0.1173886	3.9654693	7.33E-05	0.0009158	11.215382	11.083274	10.987955	10.67845	10.667521	10.535684
AT1G52300	1522.29	0.4658794	0.1183885	3.9351727	8.31E-05	0.0010192	10.821638	10.684793	10.851897	10.431602	10.19849	10.321403
AT1G33140	1915.4795	0.4662201	0.1132009	4.1185184	3.81E-05	5.14E-04	11.131002	11.002567	11.22025	10.700241	10.584507	10.661319
AT4G12420	1437.5337	0.4662511	0.1433438	3.252678	1.14E-03	0.0097646	10.75206	10.631684	10.756936	9.9273642	10.341409	10.364629
AT4G29410	700.3651	0.466426	0.1225328	3.8065401	0.0001409	0.0016259	9.7267549	9.5721294	9.7136512	9.1665543	9.2709555	9.1486907
AT3G10920	726.29032	0.4679836	0.1293594	3.6177009	2.97E-04	3.08E-03	9.5911516	9.7360642	9.835802	9.2866477	9.1710349	9.2872852
AT2G26980	1073.779	0.4703447	0.1341163	3.5069908	0.0004532	0.00441	10.389266	10.374316	10.084641	9.8449892	9.7523997	9.8330691
AT5G18410	376.47705	0.4705529	0.1315766	3.5762647	3.49E-04	3.54E-03	8.7301024	8.7802079	8.8288769	8.308004	8.2694284	8.3234938
AT1G03475	720.50604	0.4707949	0.1280521	3.676588	2.36E-04	2.54E-03	9.5875246	9.7336658	9.8031849	9.3431423	9.1585657	9.2141014
AT3G14240	418.27759	0.4713835	0.130247	3.6191493	0.0002956	0.0030667	8.8947054	8.8876633	9.0097782	8.4519603	8.4711247	8.432663
AT3G59540	800.42789	0.4715168	0.1373221	3.4336556	0.0005955	0.0055902	9.9337481	9.7566845	9.9262641	9.1455296	9.4758069	9.479972
AT2G43460	752.69482	0.4723227	0.1156644	4.0835625	4.44E-05	0.000586	9.8377822	9.74403	9.7519342	9.2962184	9.235776	9.3569351
AT4G32470	536.28549	0.4725697	0.1407743	3.3569313	0.0007881	0.0071374	9.1569898	9.3012194	9.3979125	8.8914483	8.6598951	8.8703732
AT1G51650	485.13719	0.4725862	0.1450157	3.2588617	1.12E-03	9.58E-03	9.1409948	9.0849539	9.2426944	8.4172953	8.6657811	8.8310285
AT1G75840	296.25256	0.4775664	0.1443056	3.3094094	0.0009349	0.0082372	8.4693905	8.3750106	8.4771623	7.9440305	7.861088	8.0332695
AT5G03850	603.63161	0.4784536	0.120518	3.9699766	7.19E-05	9.01E-04	9.4181743	9.4488842	9.5201957	8.9532484	9.0457315	8.925752
AT5G35790	1130.5059	0.4784914	0.1170717	4.0871643	4.37E-05	5.78E-04	10.306745	10.4148	10.388766	9.7645358	9.8512705	9.998789
AT4G30010	561.22361	0.478861	0.1432209	3.3435131	0.0008272	0.0074338	9.2122048	9.4083569	9.4663921	8.673764	8.971637	8.9155291
AT1G22610	562.50244	0.479163	0.1288857	3.7177359	2.01E-04	2.21E-03	9.2676526	9.3769402	9.4498518	8.7592901	8.9331121	8.9017849
AT1G33240	958.08922	0.4792764	0.1226439	3.9078688	9.31E-05	0.0011284	10.057548	10.067845	10.259331	9.580964	9.6252598	9.7043869
AT2G01008	3151.1033	0.4794584	0.1421098	3.3738593	7.41E-04	0.0067682	11.628872	11.888234	11.996169	11.469654	11.39146	11.196885
AT5G13120	404.74631	0.4797587	0.1407597	3.4083537	6.54E-04	6.07E-03	8.7738451	8.8905368	9.0037472	8.3453619	8.3665887	8.4657349
AT5G13450	766.84514	0.4801823	0.1261363	3.8068523	1.41E-04	1.63E-03	9.7085039	9.8251182	9.890757	9.3057261	9.2238563	9.4161437
AT5G52900	1510.7236	0.4805339	0.1232263	3.8996068	9.63E-05	1.16E-03	10.879882	10.731216	10.742522	10.318742	10.14682	10.411455
AT5G61410	2905.0035	0.4810278	0.1373826	3.5013728	0.0004629	0.0044862	11.725684	11.61525	11.837799	11.359847	11.008217	11.324064
AT1G14280	1516.9431	0.4811375	0.1456186	3.3040931	9.53E-04	8.37E-03	10.84954	10.67441	10.86235	10.056872	10.281335	10.508735
AT4G18100	1327.9934	0.4814532	0.1024983	4.6971809	2.64E-06	4.80E-05	10.567842	10.597799	10.636402	10.073665	10.088856	10.164786
AT5G60670	348.4331	0.4816873	0.1458393	3.3028646	9.57E-04	0.008395	8.720199	8.5578459	8.7079686	8.3636843	8.1316192	8.0767324
AT2G19940	341.30183	0.483434	0.1470599	3.2873269	1.01E-03	8.80E-03	8.5517361	8.6421027	8.7571959	7.9919327	8.2616763	8.1541316
AT4G36130	747.83468	0.48391	0.1249851	3.8717408	1.08E-04	0.0012855	9.7936043	9.6748761	9.8618751	9.1560802	9.3128192	9.3468264
AT3G48930	957.24609	0.48518	0.1238024	3.9189887	8.89E-05	0.0010805	10.142809	10.055095	10.199906	9.5004538	9.771632	9.6082226
AT3G18740	865.05282	0.4852827	0.1392981	3.4837717	4.94E-04	4.75E-03	10.045701	9.8303654	10.083212	9.3151715	9.4891466	9.6229575

AT2G29510	376.38916	0.4880522	0.1368638	3.5659692	3.63E-04	3.65E-03	8.8499932	8.803264	8.7190541	8.1897496	8.3881107	8.2656465
AT3G14420	9565.362	0.4884745	0.1369557	3.5666604	0.0003616	0.0036435	13.337203	13.378299	13.624106	12.985693	12.770187	13.083201
AT5G16130	1270.0958	0.4901974	0.1105898	4.4325733	9.31E-06	0.0001481	10.535735	10.459026	10.611831	10.106675	10.046623	9.9792901
AT1G09590	663.02914	0.4901999	0.1375034	3.5650009	0.0003639	0.0036583	9.4879283	9.6343084	9.671474	9.1769528	9.2037686	8.9291436
AT2G33370	697.68824	0.4913108	0.1469413	3.3435859	8.27E-04	7.43E-03	9.764989	9.4806965	9.7751341	9.1665543	9.0457315	9.2951922
AT4G15560	5839.8906	0.493012	0.0932607	5.2863879	1.25E-07	3.04E-06	12.699663	12.751969	12.76603	12.268914	12.166769	12.286586
AT1G57660	607.2222	0.4934474	0.1274996	3.8701871	1.09E-04	1.29E-03	9.4820798	9.5183395	9.4498518	8.8136145	9.1162058	8.9559931
AT3G14310	1083.571	0.4946116	0.1198024	4.1285608	3.65E-05	4.95E-04	10.286754	10.319397	10.34352	9.6497713	9.9510464	9.7945399
AT4G10480	689.40384	0.4953835	0.1146061	4.3224872	1.54E-05	2.31E-04	9.637491	9.6538895	9.6875528	9.1665543	9.2118373	9.0983632
AT1G14320	1842.5003	0.4963926	0.1189497	4.1731313	3.00E-05	0.0004167	10.993531	11.082646	11.160853	10.457423	10.70659	10.537907
AT1G72020	524.33782	0.4972585	0.1356164	3.6666549	2.46E-04	2.61E-03	9.2901413	9.1618919	9.3618246	8.673764	8.7063244	8.8738974
AT4G12600	389.88137	0.4975613	0.150887	3.2975766	9.75E-04	0.0085309	8.7268088	8.8379162	8.9451595	8.3817769	8.4576165	8.1599169
AT3G22230	624.86887	0.5013879	0.1399578	3.5824227	3.40E-04	3.47E-03	9.5301079	9.398297	9.6221334	9.0805521	9.1075825	8.8454601
AT5G09870	379.33872	0.5016758	0.1452614	3.4536083	5.53E-04	5.25E-03	8.7975855	8.8482994	8.7679109	8.2500881	8.4643864	8.142491
AT1G07320	1749.9014	0.5037711	0.1521055	3.3119852	0.0009264	0.0081747	10.99627	10.883575	11.145528	10.337403	10.354223	10.735836
AT1G56580	475.99341	0.5047734	0.1379216	3.6598577	0.0002524	0.0026736	9.0880721	9.0949542	9.2037655	8.6440903	8.7401914	8.4657349
AT5G27770	647.72258	0.5048336	0.1272089	3.9685389	7.23E-05	9.06E-04	9.700131	9.4854559	9.5328154	9.058226	9.0769884	9.0493366
AT4G38520	311.95984	0.5066102	0.1399382	3.6202418	0.0002943	0.0030581	8.5063565	8.5175198	8.5637466	7.9194695	8.1058722	7.9753929
AT2G32060	480.26104	0.5070857	0.1353339	3.7469244	0.000179	0.0019992	9.2367259	9.0142583	9.169156	8.7028397	8.5873232	8.6079552
AT1G17220	1275.1085	0.5079701	0.1297732	3.9142904	9.07E-05	0.0011	10.400162	10.715097	10.522908	10.101225	10.000791	10.008441
AT2G29530	218.59863	0.5080223	0.1562024	3.2523344	1.14E-03	9.77E-03	8.019827	7.9373062	8.0843416	7.5228149	7.4481174	7.5156503
AT5G13650	1359.9612	0.5097399	0.1050214	4.8536774	1.21E-06	2.38E-05	10.707224	10.602188	10.620725	10.180882	10.075652	10.135724
AT1G33120	1597.4005	0.5118914	0.113554	4.50791	6.55E-06	0.0001079	10.889488	10.771523	10.972984	10.318742	10.324764	10.425931
AT5G09125	1383.0277	0.5132992	0.1216953	4.2179066	2.47E-05	0.000351	10.718875	10.72197	10.584311	9.9698458	10.234579	10.212886
AT5G25460	6546.1193	0.5140877	0.1399158	3.6742647	2.39E-04	0.0025517	12.749101	12.922409	13.058058	12.559582	12.209203	12.381247
AT4G27700	661.01615	0.5148669	0.1576281	3.266339	1.09E-03	9.37E-03	9.4424718	9.6530436	9.7015928	9.3523467	8.8834626	9.0018099
AT1G63980	712.43848	0.5152265	0.1308688	3.9369681	8.25E-05	0.001015	9.7349751	9.7094606	9.6809538	9.4151838	9.0725644	9.1013727
AT4G19710	267.37442	0.5167704	0.145339	3.5556206	0.0003771	0.0037741	8.315649	8.2817805	8.3161496	7.8168173	7.8404194	7.6937004
AT4G29350	1306.4189	0.5176789	0.1177306	4.3971501	1.10E-05	1.72E-04	10.584314	10.510834	10.685736	9.9335101	10.101939	10.137191
AT4G13500	308.63697	0.5185152	0.1479907	3.5037013	4.59E-04	4.45E-03	8.5005824	8.4214478	8.6101356	8.0609297	7.8713123	8.0269523
AT2G46030	333.63839	0.5191615	0.1536423	3.3790277	7.27E-04	6.66E-03	8.5330011	8.5793759	8.7607764	8.1480747	7.9409229	8.1885004
AT1G70490	731.64197	0.5193876	0.1366929	3.7996671	1.45E-04	1.67E-03	9.7585522	9.6135885	9.8915737	9.2278502	9.1247779	9.3186565
AT5G47840	641.18943	0.5201738	0.1328641	3.9150824	9.04E-05	0.001097	9.4840319	9.5005815	9.7118026	9.0240752	8.9811096	9.0983632

AT1G61190	415.17318	0.5210476	0.1336395	3.8989046	9.66E-05	1.16E-03	8.9634819	9.0116211	8.8441462	8.4347319	8.3737984	8.4278759
AT1G32200	374.62269	0.5216634	0.153559	3.3971535	6.81E-04	0.0062824	8.7235077	8.6774722	8.9483031	8.4347319	8.1735355	8.1941497
AT1G68660	324.11252	0.5220173	0.1580109	3.3036793	0.0009542	0.0083773	8.5216422	8.568651	8.6278894	8.3453619	7.8299729	7.9949442
AT4G35100	6010.7158	0.5221289	0.1595114	3.2733021	1.06E-03	9.16E-03	12.687643	12.701933	12.987518	11.991037	12.2558	12.483692
AT1G67700	1288.3579	0.5222562	0.1491388	3.5018125	4.62E-04	4.48E-03	10.479604	10.510834	10.725674	10.068089	9.806683	10.212886
AT3G10860	434.60512	0.5226517	0.1549328	3.373409	0.0007424	0.0067765	8.8755	9.0416615	9.1226072	8.3636843	8.3447408	8.6619525
AT4G31985	648.90172	0.5226792	0.135037	3.8706376	0.0001086	0.0012906	9.6348577	9.4440024	9.6762217	9.0240752	8.9668773	9.1515972
AT5G52060	251.33502	0.5230803	0.1593938	3.2816853	1.03E-03	8.94E-03	8.2574919	8.2418337	8.1265813	7.9194695	7.501753	7.6614373
AT1G18540	1164.3665	0.5274341	0.1119783	4.7101442	2.48E-06	4.53E-05	10.336761	10.456119	10.49577	9.8384535	9.9437979	9.8916098
AT1G08520	1200.1371	0.5276219	0.1346903	3.9172969	8.95E-05	0.0010875	10.292891	10.499115	10.609848	10.016912	9.8252098	9.964491
AT4G16450	349.61622	0.5277358	0.160381	3.2905138	1.00E-03	8.71E-03	8.6401768	8.5632586	8.8808045	8.1690626	8.0257537	8.2602708
AT3G59970	1131.2429	0.5278648	0.121271	4.352769	1.34E-05	2.05E-04	10.445448	10.323124	10.395692	9.87084	9.7103033	9.9611815
AT3G21200	287.77767	0.5278771	0.1598305	3.3027305	9.57E-04	8.40E-03	8.3374074	8.3893026	8.5326689	7.789971	8.0619123	7.7484809
AT5G48900	666.60289	0.5288036	0.154257	3.4280682	6.08E-04	0.0056944	9.3944988	9.6239856	9.8391928	9.0469319	9.1075825	9.0953474
AT3G14390	209.84069	0.5299064	0.1620999	3.269011	1.08E-03	9.29E-03	7.8485778	7.9837943	8.0612976	7.4215092	7.4205328	7.4224649
AT1G72180	726.01263	0.5302749	0.1260951	4.2053585	2.61E-05	0.0003678	9.6198439	9.8031741	9.8204436	9.2278502	9.1501926	9.2525116
AT5G50460	214.75281	0.5307262	0.1608881	3.2987289	9.71E-04	0.0085027	8.0932805	7.9233432	8.0110718	7.2743201	7.514856	7.5156503
AT1G19835	1203.6682	0.5312122	0.1157168	4.5906225	4.42E-06	7.61E-05	10.446449	10.540866	10.459876	9.8252926	9.9094817	10.057303
AT2G35880	597.43976	0.5319577	0.1500188	3.5459399	3.91E-04	0.0038961	9.3243205	9.5432043	9.5638899	8.6590034	8.9952029	9.0555568
AT5G28020	702.54727	0.5321849	0.1595169	3.3362291	0.0008492	0.0075973	9.6798356	9.6581113	9.8049201	8.7730642	9.3571446	9.2416412
AT1G72640	238.97754	0.5327713	0.1509907	3.5285047	0.0004179	0.0041285	8.1135981	8.165595	8.151344	7.7626156	7.5534658	7.5424714
AT1G08380	15181.791	0.5338965	0.1583209	3.3722419	0.0007456	0.0067996	14.009016	13.983608	14.389132	13.641062	13.372056	13.728906
AT5G02500	11732.358	0.5340234	0.1428989	3.7370718	1.86E-04	0.0020656	13.939629	13.574043	13.760963	13.394944	13.154628	13.090035
AT1G04230	334.62439	0.5348544	0.1585225	3.3739965	0.0007409	0.0067676	8.7497075	8.5378237	8.6608397	7.8431732	8.0619123	8.2763382
AT4G35250	603.84567	0.5358903	0.1536322	3.4881389	4.86E-04	0.0046834	9.4068997	9.4410653	9.6152275	8.965297	8.7006018	9.116327
AT5G17710	578.35262	0.5360929	0.1332686	4.0226494	5.75E-05	7.36E-04	9.3416495	9.3820539	9.5296708	9.0240752	8.7787236	8.8490456
AT3G26650	10264.855	0.5367066	0.1233808	4.3500022	1.36E-05	0.0002067	13.578818	13.462963	13.670766	13.030821	12.8698	13.163146
AT2G01250	1291.3062	0.5374385	0.108451	4.9555886	7.21E-07	1.50E-05	10.514881	10.588539	10.641752	10.022689	10.101939	9.9858191
AT3G45030	606.9662	0.5376265	0.1379756	3.8965328	9.76E-05	0.0011741	9.5572488	9.3162575	9.5983171	8.9772457	8.9035279	8.9626281
AT5G63570	546.65584	0.5384474	0.1493617	3.6049888	3.12E-04	0.0032102	9.213382	9.2584817	9.5349079	8.9040219	8.7895464	8.7021557
AT3G02170	1332.1176	0.5400675	0.1234056	4.3763616	1.21E-05	1.87E-04	10.63087	10.610055	10.662477	9.8772309	10.095412	10.222593
AT5G51110	615.99272	0.5405401	0.1516851	3.5635675	0.0003658	0.0036719	9.4544688	9.4113612	9.6809538	9.0008505	8.767819	9.104376
AT4G24280	1421.3683	0.5408363	0.1196483	4.5202169	6.18E-06	1.03E-04	10.611356	10.843728	10.701658	10.196299	10.226637	10.097047

AT1G27400	852.77849	0.5410014	0.1243007	4.3523608	1.35E-05	2.05E-04	10.000048	9.9604957	10.01963	9.2575485	9.4281219	9.5761316
AT1G67900	3518.4295	0.5414254	0.1653094	3.2752245	0.0010558	0.0091128	12.033057	11.993555	12.080245	11.195684	11.351847	11.797038
AT5G65440	362.64514	0.5419955	0.1652381	3.2800881	0.0010377	0.0089782	8.686688	8.7771057	8.8322841	8.0832144	7.9886655	8.451654
AT3G18130	305.77028	0.5420903	0.1618256	3.3498435	8.09E-04	0.0073016	8.3460196	8.5704441	8.6239629	7.8168173	8.1145057	7.8944251
AT1G56340	830.396	0.5429692	0.1587569	3.4201299	6.26E-04	5.84E-03	9.9186717	10.035757	9.9134533	9.124194	9.6757188	9.3004396
AT3G54900	641.53372	0.5431701	0.1466151	3.7047346	0.0002116	0.0023107	9.4966568	9.5071493	9.7099516	9.187277	8.8109514	9.0648371
AT3G55750	569.96739	0.5436525	0.1243077	4.3734407	1.22E-05	1.89E-04	9.3438011	9.4459571	9.413919	8.9772457	8.767819	8.83465
AT5G24300	603.07988	0.5437271	0.140658	3.8655961	1.11E-04	1.31E-03	9.3111857	9.5146191	9.6309641	8.8914483	8.9763811	8.925752
AT4G18030	794.70771	0.5437984	0.1231651	4.4151999	1.01E-05	0.0001594	9.7665937	9.9795135	9.9029601	9.3614928	9.2824939	9.3569351
AT2G18730	236.21312	0.5438176	0.151372	3.5925906	3.27E-04	3.35E-03	8.1336336	8.1173494	8.1730041	7.6174713	7.4885299	7.6450311
AT2G36170	491.13945	0.5444749	0.1374791	3.9604209	7.48E-05	9.31E-04	9.250553	9.107358	9.2349922	8.5024443	8.7895464	8.5866368
AT3G53260	425.64479	0.5449626	0.1398182	3.8976516	9.71E-05	1.17E-03	9.0411114	8.9930246	8.9404312	8.3996454	8.2924378	8.5736928
AT5G19760	1117.1944	0.5461583	0.1326665	4.1167768	3.84E-05	0.0005176	10.358738	10.301156	10.472486	9.8514955	9.6372923	9.9562031
AT1G52220	1461.0529	0.5479456	0.1274282	4.3000336	1.71E-05	2.53E-04	10.665687	10.70493	10.9119	10.285492	10.086663	10.248616
AT2G32650	220.23545	0.5491339	0.1652397	3.3232563	8.90E-04	0.007902	8.0008641	7.9756986	8.1403908	7.5866041	7.2894272	7.560079
AT3G14930	828.6218	0.5506778	0.1268504	4.341158	1.42E-05	0.0002138	9.935889	9.918879	9.9886654	9.4669481	9.2078086	9.4822826
AT2G27290	408.88946	0.5514528	0.1508884	3.6547055	2.57E-04	0.0027161	8.8424046	8.8379162	9.1015836	8.4347319	8.2538823	8.4182539
AT5G22440	456.70479	0.5523209	0.1516334	3.6424759	2.70E-04	2.83E-03	8.9480077	9.1511488	9.1474376	8.7028397	8.5685951	8.3234938
AT2G01110	440.00024	0.5542678	0.1501526	3.6913638	0.0002231	0.0024177	8.9578741	9.0116211	9.1433288	8.5512213	8.2538823	8.595202
AT5G02870	1571.3073	0.5555571	0.1187014	4.6802912	2.86E-06	5.16E-05	10.745972	10.881049	10.982982	10.314039	10.370533	10.240449
AT4G37920	197.40036	0.5564071	0.1638717	3.3953819	0.0006853	0.0063126	7.8874454	7.8064898	7.9714482	7.3497909	7.2271013	7.3735134
AT3G62400	218.27289	0.5568657	0.1551519	3.5891631	3.32E-04	3.39E-03	8.0144344	8.0078123	8.0985593	7.3497909	7.514856	7.4883212
AT5G67220	401.06485	0.5573285	0.1554833	3.5844902	3.38E-04	3.45E-03	9.011642	8.7863923	8.9545698	8.0382954	8.4845075	8.3988151
AT4G10060	479.44567	0.5574679	0.1442822	3.8637334	1.12E-04	0.001322	9.0062187	9.2706647	9.187895	8.7453832	8.5043519	8.5518581
AT3G04400	1893.9109	0.5575229	0.138139	4.035954	5.44E-05	0.0006985	11.205359	10.971354	11.25208	10.387511	10.59996	10.705501
AT1G77460	174.63616	0.5577261	0.1677923	3.3239077	0.0008877	0.0078899	7.7898186	7.7154315	7.6354376	7.1531516	7.1110995	7.1591873
AT1G31180	298.09629	0.5578368	0.1498716	3.7220983	1.98E-04	2.17E-03	8.4495475	8.3852336	8.6101356	7.8690562	7.9213745	7.9287155
AT5G59750	208.65313	0.5590352	0.1607471	3.4777314	0.0005057	0.0048458	7.9166469	7.9864828	7.9776154	7.5228149	7.4481174	7.2487732
AT2G36830	866.49138	0.5590775	0.1683765	3.3204005	0.0008989	0.0079737	9.9794387	9.8236155	10.236369	9.3975079	9.235776	9.6540346
AT5G59430	386.20957	0.5591019	0.1549391	3.6085266	0.0003079	0.0031682	8.7991544	9.033885	8.713522	8.2696528	8.3665887	8.2053825
AT3G53430	1081.5291	0.5604431	0.1093977	5.1229902	3.01E-07	6.77E-06	10.290663	10.311378	10.411153	9.7082944	9.7906114	9.7945399
AT2G05070	22289.984	0.5616176	0.1500046	3.7440029	1.81E-04	2.02E-03	14.652133	14.550216	14.888798	14.305294	13.89889	14.147683
AT1G69620	1160.4136	0.56208	0.1161573	4.8389549	1.31E-06	2.55E-05	10.409436	10.396722	10.520798	9.7296427	9.9020208	9.9445196

AT3G06700	931.82844	0.5631786	0.1231411	4.573442	4.80E-06	8.15E-05	10.098899	10.072281	10.187988	9.6195946	9.6312886	9.4040074
AT4G24830	245.8259	0.5633382	0.1541171	3.6552612	2.57E-04	2.71E-03	8.2344843	8.2191535	8.2048963	7.3860957	7.7765751	7.6450311
AT3G55250	314.41903	0.5634972	0.1716443	3.2829355	0.0010273	0.0089055	8.5386472	8.4874672	8.657002	8.0609297	7.6634575	8.1714184
AT3G08740	627.79875	0.5649053	0.1490707	3.7895133	1.51E-04	0.0017259	9.4673539	9.4730486	9.7043844	9.1455296	8.7732816	9.0114413
AT1G78630	2445.3656	0.5649378	0.1680773	3.3611793	0.0007761	0.007043	11.426777	11.342635	11.756743	10.932772	10.686464	11.145326
AT5G08280	1050.3732	0.5664808	0.1518105	3.7314983	1.90E-04	2.11E-03	10.071247	10.261749	10.526067	9.7986049	9.6757188	9.6824481
AT3G53890	496.81289	0.5666817	0.1501368	3.7744364	1.60E-04	1.81E-03	9.089355	9.1913208	9.3863696	8.4519603	8.7623357	8.6578698
AT5G52650	691.25042	0.5675053	0.1380497	4.1108774	3.94E-05	5.29E-04	9.7176583	9.5559304	9.8118401	9.0694322	9.0183901	9.2389108
AT5G49740	806.44347	0.5675799	0.1546653	3.6697293	0.0002428	0.0025837	9.8709826	9.8947521	9.9648041	9.6497713	9.2118373	9.1311278
AT3G17040	923.93535	0.567601	0.1156755	4.9068376	9.26E-07	1.88E-05	10.078372	10.151677	10.105911	9.5731122	9.4211793	9.6103368
AT5G50900	259.24933	0.5678277	0.1533187	3.7035786	0.0002126	0.0023201	8.2846252	8.3501758	8.2075225	7.7347315	7.798172	7.586094
AT1G63940	320.13412	0.5682523	0.158196	3.5920768	3.28E-04	3.36E-03	8.3862403	8.6890719	8.6722918	8.0153003	8.0348786	7.9688164
AT5G52840	422.58017	0.5683399	0.155459	3.6558823	2.56E-04	0.0027076	8.9223228	8.9509731	9.1001711	8.3636843	8.2062131	8.5994656
AT3G01480	854.95273	0.5693163	0.1326661	4.2913484	1.78E-05	0.0002614	9.8642535	9.9645921	10.166553	9.388588	9.4453338	9.4257798
AT2G33850	554.9682	0.5694674	0.1572413	3.6216134	0.0002928	0.0030462	9.4673539	9.4701702	9.2090171	8.6883751	8.6539849	8.9985851
AT5G57030	278.55244	0.5696509	0.1624303	3.5070492	0.0004531	0.00441	8.3002205	8.3791085	8.4966187	7.789971	7.6156045	7.9688164
AT3G62250	1539.5935	0.570458	0.1135656	5.0231566	5.08E-07	1.09E-05	10.825877	10.747157	10.968347	10.271004	10.273647	10.267495
AT2G47240	267.4767	0.5706613	0.1750801	3.2594298	1.12E-03	9.56E-03	8.4475481	8.3142449	8.2437951	7.7347315	7.4751844	7.9622099
AT4G31700	1352.0191	0.5716038	0.1278974	4.4692381	7.85E-06	0.0001267	10.506742	10.737215	10.730703	10.149545	10.13626	9.959524
AT5G20130	199.93762	0.5721063	0.1717189	3.3316446	8.63E-04	0.0077111	7.9509259	7.8631766	7.911506	7.4898305	7.0583794	7.4224649
AT2G16280	682.5476	0.572657	0.1190274	4.8111354	1.50E-06	2.90E-05	9.7209728	9.6648406	9.6648008	8.965297	9.1833972	9.1103639
AT1G65290	509.50274	0.5729459	0.137271	4.1738299	3.00E-05	0.0004157	9.3746435	9.1415317	9.2579765	8.6440903	8.6058113	8.7566221
AT3G15850	1069.7328	0.5735422	0.1313555	4.3663368	1.26E-05	1.94E-04	10.362457	10.197647	10.416271	9.7437014	9.6039585	9.863523
AT5G54630	364.71202	0.5739028	0.1605019	3.5756761	3.49E-04	0.0035461	8.8193962	8.8862243	8.6665771	7.8431732	8.2848087	8.3286394
AT4G26542	459.14722	0.5740423	0.1653806	3.4710367	5.18E-04	4.96E-03	8.9746325	8.970116	9.3452262	8.6590034	8.3952139	8.511703
AT3G11510	904.34415	0.5753752	0.1280938	4.4918276	7.06E-06	0.0001155	10.039741	10.004929	10.217602	9.3614928	9.531668	9.5761316
AT1G20810	264.372	0.5764303	0.1592337	3.620026	2.95E-04	3.06E-03	8.2205015	8.3163834	8.4396796	7.5550621	7.6983337	7.8592997
AT1G14920	1575.5088	0.5767985	0.1648479	3.4989739	4.67E-04	4.51E-03	10.789638	10.818299	11.057292	10.062491	10.178043	10.574103
AT5G52520	314.3342	0.5776629	0.1448308	3.9885357	6.65E-05	0.0008388	8.477252	8.6421027	8.5901495	7.894483	7.9505986	8.052057
AT3G62630	325.90558	0.5783082	0.167236	3.4580354	5.44E-04	5.17E-03	8.6454194	8.5359897	8.7061127	7.8431732	7.861088	8.2763382
AT1G18010	253.25854	0.5789946	0.1625509	3.5619275	3.68E-04	3.69E-03	8.2957819	8.087608	8.3569362	7.7626156	7.6751769	7.5688029
AT4G33520	240.17525	0.5795716	0.1548868	3.7419053	0.0001826	0.0020324	8.0804351	8.2191535	8.2412343	7.5866041	7.5278411	7.6367575
AT1G76200	440.33306	0.5806418	0.1336771	4.3436133	1.40E-05	0.0002117	9.0738844	8.9728301	9.1239979	8.3268039	8.4977674	8.5162203

AT1G54040	208.27384	0.5812124	0.174285	3.3348378	8.53E-04	7.63E-03	7.945269	8.0314369	7.9211377	7.5228149	7.514856	7.112218
AT5G49910	495.27529	0.5823013	0.1349431	4.3151613	1.59E-05	2.38E-04	9.2063046	9.305532	9.1267754	8.7592901	8.6240654	8.5207235
AT5G67385	679.30173	0.5836835	0.1476695	3.9526341	7.73E-05	9.58E-04	9.8642535	9.5043382	9.6474982	9.0694322	8.9905204	9.1689141
AT4G33110	202.19574	0.584069	0.1660839	3.5167103	0.0004369	0.0042826	7.9224169	7.9566301	7.937049	7.3497909	7.1452054	7.4790952
AT5G10920	222.82925	0.5846835	0.164201	3.5607797	0.0003698	0.0037041	7.9844094	8.1148942	8.1126384	7.5866041	7.2740963	7.560079
AT1G41880	599.47851	0.5853932	0.1441151	4.0619828	4.87E-05	6.36E-04	9.4293615	9.6066153	9.4420676	8.965297	9.0275616	8.7021557
AT5G35970	743.46199	0.5884623	0.1618614	3.6355944	2.77E-04	0.0029021	9.6180672	9.9693566	9.8083842	9.4498989	8.9952029	9.1602816
AT5G04590	1633.956	0.588807	0.1128326	5.2184129	1.80E-07	4.24E-06	10.900492	11.055057	10.86651	10.337403	10.400846	10.296672
AT2G33460	586.23162	0.5890588	0.1712388	3.4399844	0.0005817	0.0054769	9.3034682	9.4440024	9.6358467	9.0240752	8.5432401	8.982352
AT2G08770	16710.627	0.5895572	0.180112	3.2732804	1.06E-03	9.16E-03	14.383003	14.099632	14.408484	13.454389	13.49885	14.036316
AT5G03040	813.14806	0.5905705	0.1333008	4.4303613	9.41E-06	0.0001495	9.8657516	9.8452531	10.102388	9.2378177	9.375219	9.3769425
AT2G35040	512.202	0.5908137	0.1282233	4.6076946	4.07E-06	7.09E-05	9.2110267	9.2947262	9.2905426	8.800224	8.6716432	8.5649985
AT3G16310	169.92478	0.5908856	0.1695585	3.4848486	0.0004924	0.0047393	7.7086677	7.6388899	7.7297995	7.0663221	6.9849484	7.1706945
AT4G11175	198.05026	0.5909029	0.1755441	3.3661222	0.0007623	0.0069408	7.808634	7.8602479	8.0260264	7.4898305	7.0761677	7.3433248
AT1G07010	202.52767	0.5914079	0.1592658	3.7133383	0.0002045	0.0022446	7.9137532	7.9648332	7.911506	7.4898305	7.2894272	7.2595902
AT3G50820	6568.5268	0.5917587	0.1691679	3.4980547	0.0004687	0.0045285	12.674347	12.91016	13.228308	12.499746	12.148495	12.359415
AT1G64510	1514.0196	0.592887	0.1625174	3.6481439	0.0002641	0.0027811	10.719289	10.779683	11.002016	10.299836	9.9193699	10.416296
AT1G24050	329.35761	0.5944056	0.1510286	3.935715	8.29E-05	1.02E-03	8.6384251	8.5829332	8.6722918	8.2302543	7.8088504	8.0644475
AT3G07010	461.5494	0.594616	0.1575676	3.7737204	1.61E-04	0.0018192	9.0397848	8.9916871	9.3332521	8.4858118	8.4163161	8.624786
AT1G32900	389.75327	0.5946175	0.148776	3.9967311	6.42E-05	0.0008131	8.8038509	8.9741852	8.8356832	8.4858118	8.0971867	8.254875
AT5G13410	299.15998	0.5975762	0.1736148	3.441966	5.78E-04	5.44E-03	8.3588424	8.49315	8.6722918	7.7626156	7.7323866	8.1009928
AT2G32450	273.05397	0.5976361	0.1650328	3.6213172	0.0002931	0.0030483	8.3588424	8.4352892	8.3827261	7.3497909	7.8194503	7.9489053
AT5G03940	935.28846	0.5981587	0.1308314	4.5719822	4.83E-06	8.20E-05	10.062782	10.1772	10.194621	9.5004538	9.3859559	9.6884645
AT1G67740	3959.9696	0.5989784	0.1737214	3.4479253	5.65E-04	5.34E-03	12.018484	12.061428	12.545063	11.777027	11.436913	11.590864
AT1G71500	870.0356	0.5993741	0.1702097	3.521387	4.29E-04	4.22E-03	9.9099854	9.976812	10.209764	9.7154457	9.1375412	9.3843743
AT1G22530	1330.587	0.5995551	0.1740058	3.4456039	5.70E-04	5.37E-03	10.577473	10.641952	10.758727	9.6195946	10.315434	10.06349
AT3G19720	650.95814	0.6001078	0.1584693	3.786903	0.0001525	0.0017383	9.5244283	9.6765419	9.6532888	9.2178134	8.689088	9.0862618
AT1G18000	248.90317	0.6004696	0.163504	3.6725063	2.40E-04	2.57E-03	8.2846252	8.0674343	8.3426722	7.7062978	7.6397294	7.5424714
AT4G14320	818.77197	0.6006657	0.1252029	4.7975373	1.61E-06	3.08E-05	9.9669301	9.8825357	10.024106	9.1560802	9.407193	9.4161437
AT2G35260	1370.9444	0.6010154	0.1484259	4.0492631	5.14E-05	6.67E-04	10.558145	10.836295	10.672491	10.318742	9.9169042	9.993939
AT1G70600	774.5716	0.6024415	0.1174791	5.1280761	2.93E-07	6.61E-06	9.8438758	9.9412239	9.8425757	9.1560802	9.3351522	9.2686652
AT1G23490	820.34214	0.6037016	0.1314042	4.5942337	4.34E-06	7.50E-05	10.05558	9.8206053	9.9993134	9.1975278	9.3788069	9.4161437
AT1G62750	1627.6236	0.6054814	0.1276412	4.7436192	2.10E-06	3.89E-05	10.870958	10.914609	11.035287	10.503594	10.171853	10.304528

AT5G16715	276.68545	0.6093463	0.1729114	3.5240386	0.000425	0.0041893	8.2298384	8.5829332	8.3757381	7.6174713	7.9409229	7.7016547
AT3G59980	264.97296	0.6102364	0.1540046	3.9624545	7.42E-05	0.0009259	8.3524453	8.2076783	8.4216979	7.789971	7.6751769	7.677659
AT1G56500	509.66869	0.6106958	0.1371838	4.4516608	8.52E-06	1.36E-04	9.1459353	9.2903812	9.3828886	8.5984029	8.7346019	8.6079552
AT4G34540	527.39969	0.6107636	0.1507096	4.0525844	5.07E-05	6.59E-04	9.1896542	9.3748897	9.413919	8.6290213	8.5432401	8.88092
AT2G23930	357.69947	0.6109986	0.1397336	4.3725954	1.23E-05	1.89E-04	8.7035408	8.7925503	8.8237511	7.9681804	8.1899668	8.2109663
AT5G17560	287.70549	0.6133334	0.1485298	4.1293618	3.64E-05	0.0004937	8.4192617	8.3953846	8.5513957	7.789971	7.7656542	7.9013487
AT5G52882	254.25267	0.6135457	0.1823153	3.3652995	7.65E-04	6.96E-03	8.5330011	8.1584602	8.1293538	7.4898305	7.7435613	7.6532575
AT4G23740	239.38369	0.6138751	0.162019	3.788907	1.51E-04	1.73E-03	8.1186331	8.2440822	8.1996297	7.6476919	7.3491717	7.669571
AT5G05740	241.30931	0.6149638	0.1872031	3.2850087	0.0010198	0.0088576	7.8933332	8.320651	8.3450594	7.6772924	7.501753	7.5335863
AT4G02770	12763.466	0.615186	0.1529215	4.0228875	5.75E-05	7.35E-04	13.763032	13.818402	14.152614	13.436108	13.087148	13.32203
AT2G41680	400.81009	0.6154525	0.1342221	4.5853276	4.53E-06	7.77E-05	8.8347758	8.9660353	8.9793681	8.3453619	8.2848087	8.2869512
AT3G56020	488.46023	0.616063	0.1633257	3.7719911	1.62E-04	0.001829	9.1557657	9.1391174	9.3747336	8.3453619	8.4977674	8.8201092
AT5G61170	522.78317	0.6165088	0.1249797	4.9328716	8.10E-07	1.67E-05	9.3100857	9.2673522	9.3559185	8.6883751	8.7568314	8.6121814
AT5G02610	375.29775	0.6175479	0.1407535	4.3874441	1.15E-05	1.78E-04	8.8332452	8.7265416	8.9341026	8.2696528	8.1899668	8.1714184
AT1G52870	528.03544	0.6180235	0.139904	4.4174841	9.99E-06	1.58E-04	9.4110098	9.2595935	9.3163198	8.7028397	8.5303935	8.83465
AT2G40490	755.18622	0.6187335	0.1381963	4.4772059	7.56E-06	0.0001225	9.7316926	9.7793416	10.004608	9.2673136	9.0945501	9.2713399
AT3G63490	2051.7402	0.6192665	0.1513425	4.0918217	4.28E-05	5.69E-04	11.03972	11.270291	11.510769	10.718152	10.533915	10.693585
AT2G35750	17032.373	0.6198754	0.1770912	3.5003182	4.65E-04	4.50E-03	14.416961	14.160938	14.435759	13.467155	13.508299	14.042201
AT3G53580	445.40406	0.6215223	0.1519922	4.0891728	4.33E-05	5.74E-04	9.0411114	9.005007	9.2246582	8.308004	8.3520603	8.6289632
AT1G03930	330.11357	0.6220621	0.1655624	3.7572658	1.72E-04	1.93E-03	8.5197403	8.6472089	8.7482056	8.3268039	7.8507907	7.8874681
AT1G01100	1201.4289	0.6228539	0.1282234	4.8575692	1.19E-06	2.34E-05	10.474213	10.411802	10.6548	9.7918552	9.8356906	9.9906965
AT5G65470	345.4091	0.6235425	0.1757099	3.5487038	3.87E-04	3.86E-03	8.650643	8.5006923	8.9762915	8.0832144	8.1401005	8.0078328
AT5G11740	1219.8151	0.6243149	0.1868139	3.341908	0.000832	0.0074648	10.463369	10.228635	10.868171	9.7576244	10.084468	9.8019585
AT3G05590	1500.9999	0.6247694	0.105884	5.9005058	3.62E-09	1.15E-07	10.82241	10.805795	10.865679	10.299836	10.196458	10.118002
AT5G67300	1024.1791	0.6256337	0.1693151	3.6950843	2.20E-04	2.39E-03	10.276653	10.053814	10.507006	9.5250821	9.8666839	9.4960692
AT1G57680	646.22301	0.6260645	0.1361451	4.5985087	4.26E-06	7.37E-05	9.5857077	9.5568352	9.7365609	8.800224	9.1247779	8.9790834
AT4G15802	349.17455	0.6261323	0.143458	4.3645687	1.27E-05	1.95E-04	8.7102272	8.70385	8.7750103	8.2500881	7.9213745	8.1307557
AT1G74470	14557.344	0.6265427	0.1580077	3.9652679	7.33E-05	9.16E-04	14.096629	13.999833	14.242948	13.684528	13.153843	13.52408
AT1G73060	494.63707	0.6268096	0.1646821	3.8061782	1.41E-04	0.0016272	9.0048597	9.2816515	9.4059379	8.6440903	8.4163161	8.7061151
AT5G11450	268.46102	0.6275585	0.1695687	3.7009106	0.0002148	0.0023411	8.3002205	8.27959	8.4441402	8.0382954	7.6277174	7.506598
AT3G45850	640.23847	0.6280159	0.1592417	3.9437897	8.02E-05	0.0009915	9.3903414	9.7384585	9.7034545	8.731341	9.0091599	9.0983632
AT4G27720	209.99571	0.6280502	0.1875775	3.348217	8.13E-04	7.34E-03	7.9844094	8.0314369	8.0843416	6.7692438	7.603389	7.4604644
AT3G56910	1285.5638	0.6283418	0.1334514	4.7083955	2.50E-06	4.57E-05	10.534322	10.533072	10.768098	9.945724	9.8564266	10.100059

AT4G00585	276.77966	0.628513	0.1452104	4.3282921	1.50E-05	2.26E-04	8.3966409	8.3729573	8.4574399	7.6772924	7.7435613	7.8305703
AT5G03520	704.66806	0.6286192	0.1388439	4.5275245	5.97E-06	9.94E-05	9.7135044	9.6428546	9.8593723	9.3057261	9.0366751	8.9888672
AT1G20090	414.70561	0.6289004	0.160311	3.9230027	8.75E-05	0.0010656	9.0686905	8.8138987	9.0945072	8.0609297	8.4093163	8.4657349
AT4G26540	593.74625	0.6300141	0.1516946	4.1531743	3.28E-05	4.51E-04	9.360899	9.4103605	9.7071707	8.9532484	8.751306	8.8703732
AT5G64840	1785.9494	0.6300579	0.1474596	4.2727501	1.93E-05	0.0002816	11.020009	11.195291	11.038241	10.711014	10.321039	10.278173
AT5G13100	391.86252	0.6309258	0.1690372	3.7324675	1.90E-04	2.10E-03	9.1372783	8.7739968	8.7803119	8.2889558	8.1058722	8.3591336
AT1G14290	515.8517	0.6316425	0.1745964	3.6177292	2.97E-04	0.003081	9.3986443	9.1690098	9.3559185	8.4689853	8.4371141	8.9493274
AT2G18790	1227.8509	0.6326614	0.1068851	5.9190771	3.24E-09	1.03E-07	10.558145	10.559493	10.536548	9.8252926	9.8844606	9.9923186
AT3G23400	544.13224	0.6328169	0.1763921	3.5875583	3.34E-04	3.41E-03	9.2390396	9.3269038	9.5307197	9.0805521	8.5368311	8.5385969
AT1G51110	310.65464	0.6328924	0.1730936	3.6563598	2.56E-04	2.70E-03	8.3287433	8.7152404	8.6550793	7.9194695	7.787414	8.0332695
AT5G49030	278.87388	0.6331185	0.1854387	3.4141669	6.40E-04	0.0059526	8.1897325	8.5704441	8.4329627	8.0832144	7.5534658	7.6614373
AT5G64580	127.29904	0.634386	0.185149	3.4263543	6.12E-04	5.73E-03	7.3069972	7.2842253	7.2838147	6.7131893	6.5227227	6.6846883
AT2G35370	1213.9216	0.6343865	0.1614295	3.9298053	8.50E-05	0.0010393	10.479115	10.504752	10.617273	10.062491	9.5252073	10.011643
AT1G45688	369.62111	0.636898	0.1406267	4.5289966	5.93E-06	9.90E-05	8.77065	8.7724399	8.9020364	8.308004	8.0619123	8.1656791
AT5G66920	113.69018	0.6392788	0.1954273	3.2711847	1.07E-03	9.23E-03	7.182695	7.0626468	7.1699376	6.4642672	6.3579929	6.5677036
AT3G08785	625.1878	0.6397344	0.1402438	4.5615888	5.08E-06	8.57E-05	9.4703111	9.5532129	9.7145746	8.9040219	8.8056298	9.0493366
AT5G11420	3197.0554	0.6405184	0.1485652	4.3113617	1.62E-05	0.0002414	11.707628	11.981814	12.092374	11.140965	11.214034	11.450406
AT3G27700	1855.0597	0.6406309	0.1506997	4.2510436	2.13E-05	3.07E-04	11.06374	11.06049	11.304538	10.703841	10.252291	10.49507
AT2G40610	864.62523	0.6412914	0.1875987	3.4184213	6.30E-04	5.88E-03	9.7423335	10.002939	10.34113	9.6195946	9.3277462	9.1916847
AT3G57800	867.70537	0.6413974	0.1746828	3.6717831	2.41E-04	2.57E-03	10.267049	9.8138095	10.063778	9.3151715	9.1874946	9.6250503
AT1G26208	297.28611	0.6414445	0.1782696	3.5981716	3.20E-04	3.29E-03	8.4515441	8.3480868	8.6893014	8.126777	7.6156045	7.8159882
AT5G01810	236.53286	0.642974	0.1525857	4.2138546	2.51E-05	0.0003558	8.1161178	8.2168658	8.2257728	7.4898305	7.501753	7.5688029
AT2G43130	121.2079	0.6434429	0.1872085	3.437039	5.88E-04	5.53E-03	7.211258	7.2171356	7.2383096	6.6548684	6.5227227	6.5324599
AT3G52750	169.83911	0.6435566	0.1832939	3.5110638	0.0004463	0.0043526	7.6986156	7.5972826	7.8114779	7.11039	7.1948986	6.8368942
AT1G30440	533.2061	0.6442999	0.1501444	4.2912012	1.78E-05	2.61E-04	9.2039377	9.429257	9.4431822	8.4347319	8.8109514	8.7642378
AT1G32540	213.63166	0.6447133	0.1678058	3.8420192	1.22E-04	1.43E-03	8.0225158	8.0392266	8.0349254	7.5550621	7.1110995	7.4604644
AT3G63540	673.6643	0.6462165	0.166615	3.8785014	0.0001051	0.0012516	9.6295765	9.5783802	9.8434202	9.2575485	8.7120244	9.0801729
AT2G28150	266.10055	0.6470214	0.1899395	3.4064605	6.58E-04	6.10E-03	8.453538	8.2773962	8.3757381	7.4898305	7.4205328	8.0142341
AT1G18730	568.68275	0.6477416	0.1622163	3.9930733	6.52E-05	8.23E-04	9.2562754	9.4920929	9.591296	8.7171607	8.6058113	8.9790834
AT4G39970	321.71888	0.6480037	0.1885128	3.4374528	0.0005872	0.0055194	8.5442712	8.43332	8.8575845	8.2302543	7.6868017	7.9489053
AT3G06150	348.06339	0.648484	0.1604266	4.0422471	5.29E-05	0.0006832	8.6917645	8.6824549	8.8575845	8.0609297	7.861088	8.2602708
AT4G18730	1210.4046	0.6485192	0.1086125	5.970942	2.36E-09	7.75E-08	10.597447	10.456604	10.545398	9.8772309	9.8435016	9.9071717
AT3G54720	205.84999	0.6486852	0.1637612	3.9611662	7.46E-05	9.29E-04	7.9195348	8.0340382	8.0349254	7.1946821	7.2586008	7.4510579

AT4G39350	994.2963	0.649232	0.1358345	4.7795805	1.76E-06	3.33E-05	10.098899	10.346333	10.299272	9.6497713	9.4315808	9.6784231
AT1G69740	1003.5232	0.6501852	0.1215091	5.3509154	8.75E-08	2.20E-06	10.170347	10.222377	10.389923	9.6572178	9.5603885	9.5997345
AT1G76520	324.5966	0.6502149	0.1641325	3.9615239	7.45E-05	0.0009285	8.5957316	8.652297	8.6930542	7.8168173	8.238167	7.8012572
AT1G77670	110.37938	0.6508136	0.1969474	3.3045053	9.51E-04	8.36E-03	7.0934478	7.0107677	7.1806696	6.4642672	6.3867874	6.4212482
AT3G57610	419.24491	0.6511855	0.161113	4.0417927	5.30E-05	6.84E-04	8.8814366	8.9903483	9.1419566	8.3817769	8.5109065	8.1189241
AT3G59310	212.01112	0.6514466	0.1993325	3.2681408	1.08E-03	0.0093149	7.931029	7.8455149	8.2866486	7.4560742	7.0937393	7.4883212
AT3G21055	4223.1131	0.6516174	0.1883139	3.460272	5.40E-04	5.14E-03	12.207567	12.102591	12.66133	11.86808	11.360946	11.718852
AT4G39040	449.49143	0.6524334	0.1672835	3.9001668	9.61E-05	0.001161	9.0424367	9.0698221	9.1998142	8.6883751	8.0971867	8.5071716
AT4G25700	198.42402	0.6524722	0.1902074	3.4303197	6.03E-04	5.65E-03	7.8455442	7.7474843	8.1756889	7.3497909	7.2894272	7.1591873
AT4G00430	1170.2925	0.6527413	0.1333174	4.8961441	9.77E-07	1.97E-05	10.364048	10.504752	10.589354	9.8579725	9.6551548	9.9394833
AT1G14270	267.86544	0.6531575	0.1679392	3.8892502	1.01E-04	1.20E-03	8.2980029	8.2729985	8.5115722	7.8168173	7.4751844	7.7863742
AT5G57290	550.55528	0.6542563	0.1438083	4.5495029	5.38E-06	9.05E-05	9.4140847	9.237193	9.5525735	8.6440903	8.8002886	8.737405
AT5G67470	104.87325	0.656161	0.2018783	3.2502802	0.0011529	0.0098283	7.0985535	7.0054757	6.9366063	6.5306385	6.2679916	6.2796418
AT2G27720	1185.6088	0.6563056	0.1410534	4.6528867	3.27E-06	5.82E-05	10.516312	10.361428	10.647566	9.7154457	9.7579208	10.006836
AT1G04240	545.94283	0.6568454	0.1578769	4.1604904	3.18E-05	0.0004379	9.5149122	9.2772668	9.4059379	8.4858118	8.6420915	8.945983
AT1G55370	145.04999	0.6588206	0.199108	3.3088604	9.37E-04	8.25E-03	7.2621985	7.536206	7.6118418	7.0663221	6.6937867	6.7006521
AT5G60600	2114.3789	0.6590314	0.1491735	4.417885	9.97E-06	1.58E-04	11.117861	11.443671	11.445804	10.857195	10.51121	10.626228
AT2G32560	315.25515	0.6596997	0.1396411	4.7242498	2.31E-06	4.26E-05	8.6154564	8.5740234	8.6239629	7.9194695	7.861088	7.9949442
AT3G16080	464.0194	0.6598857	0.1438134	4.5884845	4.46E-06	7.67E-05	9.1285691	9.1642684	9.2142497	8.2101441	8.6657811	8.4934912
AT2G05620	997.62397	0.659897	0.133841	4.9304535	8.20E-07	1.69E-05	10.318279	10.151677	10.296192	9.7645358	9.407193	9.5911962
AT4G37770	316.8238	0.6603886	0.1640178	4.0263221	5.67E-05	0.0007256	8.5063565	8.5304735	8.806532	7.7347315	8.0348786	7.9688164
AT2G42870	833.17743	0.6605413	0.1854745	3.5613596	3.69E-04	0.0036976	10.077725	9.7816652	10.155715	9.0240752	9.1956545	9.6375435
AT4G17560	708.89629	0.66094	0.1593185	4.1485454	3.35E-05	4.58E-04	9.6145073	9.7086467	9.9616963	9.2378177	8.8631143	9.1602816
AT3G56650	263.89618	0.6610413	0.190873	3.463252	5.34E-04	0.0050922	8.2111038	8.2305382	8.5657948	7.894483	7.3491717	7.7330404
AT5G51545	201.1161	0.6611464	0.1780298	3.7136842	2.04E-04	2.24E-03	7.9367419	7.8573134	8.095727	7.2350505	7.0937393	7.4510579
AT5G40160	165.02077	0.6612559	0.1977179	3.3444405	0.0008245	0.0074237	7.4173535	7.7024082	7.8855026	6.9739302	6.9849484	7.0005885
AT3G05560	671.23338	0.6616883	0.1177843	5.6177992	1.93E-08	5.36E-07	9.6687219	9.6840144	9.732008	8.9040219	9.0457315	9.0740581
AT1G76450	436.15733	0.6639338	0.1626153	4.0828505	4.45E-05	0.0005873	8.971853	9.0076563	9.2207638	8.5188872	8.1230878	8.5071716
AT3G53870	1350.4207	0.6640992	0.1100854	6.0325796	1.61E-09	5.49E-08	10.622033	10.726399	10.741163	10.028443	10.09323	9.9512075
AT3G62600	186.23056	0.6644679	0.1829125	3.6327097	2.80E-04	2.93E-03	7.9367419	7.9511353	7.6315317	7.2350505	7.2110898	7.0636679
AT5G48790	507.54886	0.6656586	0.153553	4.3350406	1.46E-05	2.19E-04	9.2957092	9.1451456	9.3944593	8.8659674	8.4643864	8.5071716
AT5G20080	220.502	0.6660446	0.1661867	4.0078092	6.13E-05	7.78E-04	7.9338882	8.15129	8.1863783	7.3860957	7.4481174	7.3834375
AT1G04430	1006.8582	0.6662627	0.1729455	3.8524433	1.17E-04	1.37E-03	10.312798	10.136628	10.400864	9.2575485	9.531668	9.8846392

AT5G21060	140.83368	0.6678798	0.1987413	3.3605487	7.78E-04	7.05E-03	7.2667415	7.468588	7.6158013	6.7692438	6.5985322	6.8795917
AT1G76160	703.36383	0.6685276	0.1420633	4.7058445	2.53E-06	4.62E-05	9.8865629	9.6732084	9.7401929	8.8268819	9.2037686	9.139936
AT3G25660	371.00839	0.6693172	0.148309	4.5129898	6.39E-06	0.0001058	8.8085321	8.7771057	8.9085069	8.3636843	7.9602099	8.1599169
AT2G21280	186.08701	0.6698975	0.1901166	3.5236146	4.26E-04	4.19E-03	7.6817048	7.8125628	8.0110718	7.4215092	6.9075785	7.1591873
AT5G06290	351.61664	0.6700683	0.1963435	3.4127348	0.0006431	0.0059815	8.6593072	8.5829332	9.0022355	8.3996454	7.7435613	8.0395593
AT1G71880	461.51423	0.6705477	0.1721362	3.8954486	9.80E-05	0.0011787	9.3089849	9.0208302	9.1460693	8.2696528	8.7457594	8.3079459
AT2G02510	217.93961	0.6708417	0.1967328	3.4099131	0.0006498	0.0060361	8.1484797	7.8573134	8.2590657	7.0663221	7.3196087	7.6284362
AT4G03280	4045.0985	0.6708979	0.148819	4.5081466	6.54E-06	1.08E-04	12.166068	12.193675	12.476223	11.73366	11.366378	11.667489
AT2G33450	799.85494	0.672228	0.1765998	3.8065039	1.41E-04	1.63E-03	9.7857124	9.891887	10.148217	9.4326457	8.9085009	9.379424
AT3G46560	124.11769	0.6740631	0.2046236	3.2941613	9.87E-04	0.008625	7.2667415	7.2488327	7.3182411	6.4642672	6.8672798	6.3215387
AT1G26210	183.92734	0.6747708	0.2073084	3.2549137	1.13E-03	9.70E-03	7.7086677	7.7089345	8.0349254	7.4898305	6.7613149	7.1358938
AT5G66470	174.01255	0.6751467	0.1781138	3.7905356	0.0001503	0.0017206	7.6611467	7.7121867	7.8789279	7.11039	7.1619607	6.9210618
AT2G44920	511.32056	0.6763798	0.1776251	3.8079066	1.40E-04	1.62E-03	9.1496295	9.2551412	9.5074646	8.5984029	8.3299896	8.8382624
AT5G56750	334.67212	0.6775982	0.153487	4.414693	1.01E-05	0.0001596	8.7561838	8.5758098	8.7803119	7.8168173	7.9792425	8.142491
AT5G27560	273.67646	0.6783389	0.2023121	3.3529329	8.00E-04	7.23E-03	8.2251775	8.4194596	8.6140998	7.3497909	7.5661095	8.0332695
AT1G23180	130.97207	0.6796889	0.1925716	3.529539	0.0004163	0.0041142	7.2484831	7.3608199	7.4348917	6.7692438	6.4427077	6.7320595
AT1G04480	702.91477	0.6797196	0.1346034	5.0497972	4.42E-07	9.61E-06	9.8270562	9.597851	9.8602071	9.0240752	9.1332993	9.0462164
AT4G03110	408.94078	0.6801389	0.1459536	4.6599664	3.16E-06	5.65E-05	8.9994107	8.8804542	9.0916668	8.1051601	8.4232821	8.2869512
AT2G29980	606.38428	0.6810409	0.1998516	3.4077328	0.000655	0.0060743	9.6330994	9.3972872	9.6072944	9.3057261	8.5559733	8.595202
AT5G51120	248.08271	0.6811498	0.1660887	4.1011206	4.11E-05	5.48E-04	8.1825378	8.235067	8.3592999	7.6772924	7.6868017	7.353458
AT5G11070	344.99072	0.6812291	0.1751967	3.8883665	0.0001009	0.0012084	8.7849726	8.6472089	8.8424576	7.5550621	8.238167	8.1541316
AT5G41520	942.41037	0.682501	0.1322521	5.1606077	2.46E-07	5.63E-06	10.010921	10.269496	10.263754	9.5731122	9.4142031	9.4937806
AT1G57860	424.8199	0.6832458	0.1399828	4.8809259	1.06E-06	2.12E-05	9.0048597	9.0786682	9.0716263	8.0832144	8.4711247	8.3939142
AT4G22890	1724.8062	0.684078	0.1356525	5.0428692	4.59E-07	9.94E-06	10.916843	11.015788	11.228678	10.448867	10.218651	10.411455
AT2G27710	712.11937	0.6841463	0.1184997	5.7733996	7.77E-09	2.33E-07	9.7201449	9.7707893	9.8551915	9.1025381	9.1247779	9.0430894
AT5G11270	184.55006	0.6844254	0.1676369	4.0827851	4.45E-05	5.87E-04	7.7803181	7.8395794	7.8855026	7.3125489	7.0036595	7.1358938
AT1G59710	157.35999	0.6845358	0.1895459	3.6114509	3.04E-04	3.14E-03	7.7053248	7.4296063	7.7370884	6.7131893	6.9659915	6.9745636
AT3G25920	2119.685	0.6846283	0.1529004	4.4776088	7.55E-06	0.0001224	11.212441	11.313221	11.533972	10.74286	10.40261	10.792797
AT3G55330	339.77996	0.6849898	0.1642901	4.1693919	3.05E-05	0.0004225	8.6118901	8.6973007	8.8339847	8.1897496	7.7435613	8.1069947
AT5G16710	537.03301	0.6856991	0.1683514	4.0730238	4.64E-05	6.10E-04	9.1740192	9.409359	9.5463633	8.673764	8.4508147	8.8597488
AT5G02095	276.87194	0.685881	0.2109319	3.2516706	1.15E-03	9.79E-03	8.1728886	8.2418337	8.789105	7.9194695	7.5534658	7.669571
AT1G67865	874.89735	0.6864506	0.1680628	4.0844885	4.42E-05	5.84E-04	9.9904671	10.08174	10.159789	9.7225617	9.1032514	9.2659854
AT3G11700	683.96959	0.6865284	0.1520468	4.5152449	6.32E-06	0.0001048	9.8739633	9.5322061	9.7804353	8.8136145	9.0945501	9.116327

AT3G28200	134.04195	0.6866216	0.2010292	3.4155317	6.37E-04	5.93E-03	7.2667415	7.3102113	7.5757083	6.6548684	6.8258231	6.5324599
AT1G09690	1230.8339	0.6870287	0.1344019	5.111749	3.19E-07	7.11E-06	10.451945	10.487774	10.763643	9.8962356	9.9119601	9.8130155
AT4G16980	1406.9411	0.688096	0.193877	3.5491365	0.0003865	0.0038587	10.910688	10.288139	11.022294	10.165298	9.9678192	10.025969
AT4G33680	654.55551	0.6886897	0.1450651	4.7474516	2.06E-06	3.83E-05	9.4712955	9.667356	9.8332536	9.0240752	8.923318	8.9426308
AT5G65360	171.58723	0.6887961	0.1868242	3.6868669	2.27E-04	2.45E-03	7.5940432	7.8602479	7.7479532	7.1946821	6.7831397	7.112218
AT4G33865	709.63186	0.6890735	0.1675187	4.1134122	3.90E-05	5.24E-04	9.8657516	9.5676479	9.9214734	8.7453832	9.1833972	9.2085298
AT5G43270	224.91554	0.689362	0.1699558	4.0561246	4.99E-05	0.0006495	8.1385993	8.0078123	8.2127605	7.5550621	7.514856	7.2158269
AT4G16983	1402.8614	0.689976	0.1940753	3.5551981	3.78E-04	3.78E-03	10.907418	10.283774	11.019308	10.160066	9.9630468	10.01962
AT2G31490	281.4367	0.6904496	0.1876309	3.6798289	2.33E-04	2.51E-03	8.4909072	8.4194596	8.4944698	7.5228149	7.4751844	8.0767324
AT3G60245	1304.0022	0.690473	0.1124585	6.1398048	8.26E-10	3.02E-08	10.648819	10.581888	10.746139	9.87084	10.003117	9.9825583
AT4G30440	923.42439	0.6907577	0.1562779	4.4200612	9.87E-06	0.0001564	10.193199	9.9104096	10.350665	9.5250821	9.5187175	9.3160682
AT3G54210	1267.9241	0.6914828	0.1624813	4.2557679	2.08E-05	0.000301	10.463864	10.504283	10.859431	10.079219	9.6959937	9.936116
AT5G16000	332.7703	0.6918376	0.1636357	4.227913	2.36E-05	0.0003371	8.6917645	8.7297543	8.7172124	7.5550621	8.0439463	8.1885004
AT5G28500	332.16216	0.6920055	0.1596577	4.3343063	1.46E-05	0.0002201	8.48312	8.7925503	8.769689	8.0609297	7.9886655	7.9082392
AT3G48200	372.30009	0.6925875	0.1922042	3.6033938	0.0003141	0.003227	8.6798914	9.0142583	8.8271703	8.5024443	8.0971867	7.7938349
AT5G54600	1431.0278	0.6927157	0.1459773	4.7453664	2.08E-06	3.86E-05	10.607332	10.790493	10.970667	10.051231	9.9534545	10.230862
AT1G75350	1216.2598	0.6930334	0.1576116	4.3970956	1.10E-05	0.0001718	10.411489	10.491091	10.769431	9.7225617	9.7272895	10.055753
AT3G23730	133.07996	0.6933753	0.2030368	3.415023	6.38E-04	5.94E-03	7.3884628	7.1987053	7.5552361	6.6548684	6.496541	6.7928944
AT1G37130	10544.908	0.6954224	0.1121562	6.2004788	5.63E-10	2.10E-08	13.724951	13.623944	13.672193	13.042241	13.045429	12.807241
AT1G03870	893.60269	0.6962098	0.1980817	3.5147611	4.40E-04	4.31E-03	10.173375	9.8145662	10.350665	9.0008505	9.6698732	9.4088742
AT1G50450	158.03747	0.6968606	0.1930675	3.6094145	0.0003069	0.0031606	7.5122328	7.6354681	7.7297995	7.0208656	6.5985322	7.0636679
AT4G17870	314.64873	0.697007	0.1705699	4.086341	4.38E-05	5.80E-04	8.8147502	8.4411808	8.5881355	7.8168173	7.798172	8.0395593
AT3G61620	132.53102	0.6972141	0.2003906	3.479275	5.03E-04	4.82E-03	7.2937031	7.3230308	7.4304023	7.0663221	6.5227227	6.4212482
AT3G63410	1428.0372	0.6990066	0.1689017	4.1385419	3.50E-05	4.76E-04	10.628224	10.685207	11.039715	10.241585	9.8093443	10.144504
AT1G68780	168.04009	0.6995131	0.1988352	3.5180546	4.35E-04	4.27E-03	7.8241282	7.7089345	7.6078713	7.1531516	6.5985322	7.1706945
AT5G62280	1369.9382	0.6999216	0.1211209	5.7787005	7.53E-09	2.26E-07	10.73945	10.80351	10.656723	10.011112	9.8945212	10.143045
AT1G80030	243.5283	0.7004568	0.160163	4.3733985	1.22E-05	0.0001887	8.1161178	8.3501758	8.2766794	7.4898305	7.5910692	7.4974886
AT4G20360	4266.4925	0.7005554	0.1429501	4.9006987	9.55E-07	1.93E-05	12.135811	12.424949	12.531896	11.742439	11.509898	11.706058
AT3G62530	841.15151	0.7007239	0.1247969	5.6149155	1.97E-08	5.45E-07	9.9201144	10.038998	10.124095	9.3614928	9.2238563	9.3669735
AT4G20362	4273.1946	0.7007593	0.1429121	4.9034286	9.42E-07	1.91E-05	12.138602	12.427054	12.534121	11.744188	11.511533	11.709021
AT5G53370	283.39475	0.7015151	0.1598871	4.3875651	1.15E-05	1.78E-04	8.4792106	8.4293734	8.4793371	7.789971	7.9213745	7.5246462
AT3G02570	322.28252	0.7029145	0.1564477	4.4929693	7.02E-06	0.000115	8.7018644	8.5249362	8.7554023	7.7062978	7.9505986	8.0582656
AT3G28180	627.05147	0.7060755	0.1523635	4.6341499	3.58E-06	6.33E-05	9.4771878	9.5495814	9.7997082	8.7592901	9.0457315	8.8237582

AT5G56670	625.07768	0.7067401	0.1216413	5.8100337	6.25E-09	1.89E-07	9.5637242	9.5658514	9.6790628	8.9288451	8.8267992	8.9086734
AT3G47450	204.70303	0.7075059	0.191507	3.6944126	2.20E-04	2.39E-03	7.7153305	8.0800759	8.1703143	7.3860957	7.1785237	7.2595902
AT2G37660	946.19194	0.7079327	0.1453946	4.8690442	1.12E-06	2.23E-05	10.073842	10.113448	10.400864	9.5731122	9.3425203	9.5210075
AT1G26880	544.42293	0.7080128	0.1691722	4.1851609	2.85E-05	3.99E-04	9.3416495	9.4262897	9.4913912	8.2696528	8.9525032	8.6862086
AT3G27160	1155.999	0.7087717	0.1462389	4.8466702	1.26E-06	2.46E-05	10.293448	10.531232	10.640781	9.7437014	9.6312886	9.9071717
AT2G24060	287.47398	0.7092613	0.1779403	3.9859509	6.72E-05	0.000847	8.4674185	8.3852336	8.6101356	7.894483	7.4205328	7.9219223
AT3G11945	282.28795	0.7095529	0.1797242	3.9480098	7.88E-05	9.75E-04	8.3178396	8.3270288	8.7005306	7.9194695	7.6277174	7.669571
AT4G25080	2326.9659	0.7096808	0.1462176	4.8535939	1.21E-06	2.38E-05	11.454918	11.448798	11.603416	10.777438	10.524228	10.984293
AT1G22882	150.05319	0.7098361	0.1905351	3.7254864	0.0001949	0.0021477	7.4496782	7.6726689	7.5428118	6.8232017	7.0036595	6.6357088
AT4G17390	1517.2143	0.7098596	0.1192487	5.9527642	2.64E-09	8.56E-08	10.904142	10.76057	10.979913	10.149545	10.244446	10.089488
AT1G11860	3054.7895	0.7109711	0.1357158	5.2386767	1.62E-07	3.84E-06	11.76495	11.87106	12.039372	11.138306	11.016302	11.329849
AT1G29070	1201.353	0.7110504	0.2022927	3.5149573	4.40E-04	4.30E-03	10.374609	10.326841	10.897742	9.9638531	9.4487516	9.9611815
AT5G48460	190.24678	0.711556	0.1769855	4.0204204	5.81E-05	7.41E-04	7.7611274	7.8631766	8.0612976	7.1946821	7.0937393	7.2046755
AT3G24050	337.87722	0.7124069	0.1634219	4.3593115	1.30E-05	0.0001994	8.7448312	8.6249504	8.8254617	7.7347315	8.2302446	7.9219223
AT5G65380	1363.4046	0.713055	0.1056644	6.7483006	1.50E-11	6.80E-10	10.743122	10.673159	10.778291	9.9638531	9.9867523	10.06349
AT3G23750	582.03618	0.7141043	0.1666853	4.284148	1.83E-05	2.69E-04	9.4017456	9.6462589	9.5010567	8.3636843	8.9379844	8.8948634
AT4G06695	271.41265	0.7145834	0.1706928	4.1863709	2.83E-05	3.97E-04	8.443541	8.2237182	8.5555244	7.6772924	7.5278411	7.7938349
AT3G44890	848.00864	0.71591	0.1556539	4.599372	4.24E-06	7.34E-05	9.9172276	10.009562	10.206485	9.4412981	9.0547314	9.4257798
AT3G03780	300.51954	0.7159881	0.2138815	3.3475928	8.15E-04	7.35E-03	8.1060125	8.7848486	8.7005306	7.9440305	7.8814647	7.6031803
AT3G23530	329.07804	0.7164168	0.1659292	4.3176063	1.58E-05	2.36E-04	8.7284566	8.7677589	8.6160778	7.5550621	7.9886655	8.1599169
AT5G47190	959.29376	0.7171884	0.1458603	4.9169536	8.79E-07	1.80E-05	10.070598	10.258416	10.354225	9.3705812	9.3930697	9.6764064
AT4G09010	1463.1578	0.7173448	0.1390616	5.1584662	2.49E-07	5.68E-06	10.759735	10.77347	10.962528	10.231643	9.8819344	10.1791
AT3G62410	793.3326	0.7187122	0.1601309	4.4882793	7.18E-06	0.0001171	9.7998869	9.8890161	10.153674	9.3245555	8.9811096	9.3238194
AT1G60950	6180.9007	0.7193478	0.1641435	4.382433	1.17E-05	1.82E-04	12.850184	12.701626	13.162666	12.375461	11.966738	12.154818
AT5G08330	499.82109	0.7196683	0.1727443	4.1660895	3.10E-05	0.0004281	9.3513064	9.0155751	9.4564907	8.7730642	8.4022823	8.4750465
AT4G02790	211.07292	0.7197473	0.1790093	4.0207261	5.80E-05	7.41E-04	7.9338882	8.1075036	8.1486135	7.11039	7.1785237	7.5335863
AT3G13520	582.94245	0.7199675	0.1912251	3.7650261	1.67E-04	1.87E-03	9.5992791	9.1866523	9.7338308	8.4347319	8.8320434	8.9426308
AT1G49840	87.175236	0.7210163	0.2121484	3.3986417	6.77E-04	6.25E-03	6.818153	6.7823036	6.7689431	6.0770468	5.9584295	6.0744123
AT3G07230	434.11069	0.7234729	0.1509315	4.7933853	1.64E-06	3.14E-05	9.103392	8.9849807	9.2037655	8.0609297	8.4439806	8.4469296
AT2G29180	177.27911	0.7240264	0.209206	3.4608298	0.0005385	0.0051316	7.7834919	7.8003911	7.8855026	6.8232017	6.7166491	7.4224649
AT2G40765	206.98414	0.7245457	0.1724165	4.2023002	2.64E-05	0.0003718	8.033221	7.9120747	8.1622147	7.0208656	7.3781411	7.3433248
AT5G47700	634.83927	0.7254705	0.1236039	5.8693182	4.38E-09	1.35E-07	9.605569	9.6109775	9.6978621	8.8268819	8.8424749	8.9953531
AT5G13400	139.03728	0.7273096	0.1961784	3.7073885	0.0002094	0.0022923	7.4050425	7.4097132	7.5958941	6.5306385	6.5484377	6.9210618

AT3G14415	3000.5772	0.7274759	0.1198793	6.068401	1.29E-09	4.51E-08	11.757302	11.814499	12.036237	11.162055	11.087097	11.154048
AT3G15190	996.45839	0.7287636	0.1906132	3.8232594	1.32E-04	0.0015328	10.113474	10.122031	10.590864	9.6195946	9.2078086	9.7181762
AT3G05910	745.8283	0.7289036	0.1284191	5.6759747	1.38E-08	3.94E-07	9.7928171	9.8818138	9.946056	8.965297	9.1204982	9.2470867
AT3G52380	578.74167	0.7312722	0.1347381	5.4273593	5.72E-08	1.49E-06	9.434418	9.4537496	9.6221334	8.7592901	8.6539849	8.8490456
AT5G54640	220.28514	0.7315182	0.1751945	4.1754634	2.97E-05	4.13E-04	8.0674743	8.0418139	8.2741762	7.1531516	7.2894272	7.5513021
AT2G44740	119.34772	0.7316913	0.2176149	3.3623211	7.73E-04	7.02E-03	7.0624282	7.1371269	7.4790331	6.6548684	6.2679916	6.5145098
AT1G21050	621.93018	0.7317489	0.1752433	4.1756169	2.97E-05	0.0004134	9.8146994	9.3033773	9.6894327	8.6440903	8.9085009	8.9626281
AT5G08050	455.41925	0.7322588	0.1779892	4.1140623	3.89E-05	5.22E-04	9.0686905	9.0195182	9.3535493	8.7171607	8.1485321	8.3388759
AT3G26060	625.81965	0.7324892	0.1897679	3.8599219	1.13E-04	0.0013363	9.3651421	9.5468518	9.9029601	9.0008505	8.562298	8.982352
AT5G53580	282.76971	0.7331635	0.163141	4.4940495	6.99E-06	1.15E-04	8.4515441	8.4741197	8.5094454	7.7626156	7.4617144	7.9013487
AT1G31800	297.05285	0.7334345	0.1899774	3.8606409	1.13E-04	1.33E-03	8.1945091	8.7168603	8.691179	7.7626156	7.7656542	7.8232977
AT1G67250	156.88364	0.7344164	0.2105312	3.4883962	4.86E-04	0.004681	7.5831567	7.5065441	7.8690092	6.3215962	6.9659915	7.0759594
AT1G65230	286.57917	0.7346438	0.1746853	4.2055279	2.60E-05	0.0003677	8.3417199	8.4273961	8.6665771	8.0153003	7.6156045	7.6116481
AT5G57345	811.48231	0.7350343	0.2060835	3.5666812	3.62E-04	3.64E-03	9.8552323	10.006254	10.112932	9.6793296	8.7895464	9.104376
AT5G04130	88.423553	0.7351957	0.2163832	3.3976566	6.80E-04	6.27E-03	6.761338	6.8547197	6.8178859	6.0770468	6.2047119	5.8637088
AT3G17390	2174.7847	0.7353674	0.142	5.1786432	2.24E-07	5.17E-06	11.285896	11.369659	11.582325	10.44026	10.770807	10.738738
AT5G60860	330.01426	0.7355716	0.1981895	3.7114555	2.06E-04	0.0022602	8.6917645	8.5230856	8.9181586	7.4560742	7.9602099	8.2109663
AT1G29460	261.87815	0.7357315	0.205116	3.5869045	0.0003346	0.0034157	8.4654437	8.165595	8.4966187	7.4215092	7.3045969	7.9422067
AT4G24090	122.42026	0.7357326	0.2142668	3.4337214	5.95E-04	5.59E-03	7.1584528	7.2666375	7.4348917	6.4642672	6.2047119	6.7627976
AT5G20140	187.04859	0.7382697	0.1943726	3.79822	0.0001457	0.0016742	7.7514354	7.8216247	8.0140751	7.5228149	6.8875699	6.9745636
AT5G56040	206.28321	0.7387262	0.1611815	4.5831938	4.58E-06	7.83E-05	8.0117306	7.962104	8.0814811	7.3860957	7.1785237	7.2595902
AT2G14880	197.86197	0.7387415	0.1755451	4.2082727	2.57E-05	0.000364	8.1009332	7.8455149	7.9243341	7.3497909	7.0403691	7.2378745
AT5G02890	112.11923	0.7397476	0.2030345	3.6434574	2.69E-04	2.82E-03	7.1238145	7.1987053	7.1860058	6.2445954	6.2047119	6.6021069
AT1G78370	481.83442	0.7399692	0.1916157	3.861735	1.13E-04	0.0013293	8.8454448	9.4901997	9.3499881	8.3636843	8.5303935	8.5162203
AT3G51140	244.68879	0.740017	0.2010261	3.6811978	2.32E-04	0.002499	8.1161178	8.1632207	8.4793371	7.8690562	7.1619607	7.4604644
AT3G47560	212.03529	0.7407964	0.1703311	4.3491557	1.37E-05	2.07E-04	8.1009332	7.962104	8.151344	7.2350505	7.1619607	7.4604644
AT3G62720	322.66466	0.7408718	0.1840554	4.0252645	5.69E-05	7.28E-04	8.6313967	8.7022155	8.7079686	7.5866041	8.2616763	7.7252578
AT5G20720	1837.4015	0.7410658	0.1419442	5.2208258	1.78E-07	4.19E-06	11.088971	11.083902	11.329767	10.591728	10.220651	10.421122
AT1G17650	178.25314	0.7446421	0.220403	3.3785471	7.29E-04	6.67E-03	7.5758531	7.7443108	8.1238035	6.9739302	6.7831397	7.2809839
AT2G41310	191304.85	0.7446494	0.2026598	3.6743822	0.0002384	0.0025517	17.967561	17.513407	18.110727	16.698034	17.162633	17.361004
AT2G09250	191202.75	0.7446826	0.2027953	3.6720902	2.41E-04	2.57E-03	17.966964	17.512354	18.110078	16.69651	17.162048	17.360396
AT3G28460	108.37805	0.7447825	0.2265697	3.2872114	0.0010118	0.0087959	6.9651548	7.2533046	7.1591252	5.8874513	6.6937867	6.1920111
AT1G58290	2824.7201	0.7449458	0.1559882	4.7756562	1.79E-06	3.40E-05	11.831434	11.742768	11.81042	11.322867	10.755708	10.966247

AT3G27830	1291.0715	0.745353	0.1785145	4.1753087	2.98E-05	4.13E-04	10.528659	10.521069	10.929902	9.87084	9.6342936	10.134256
AT5G46580	214.45826	0.7456952	0.1734504	4.2991833	1.71E-05	0.0002537	7.9395899	8.2395816	8.058391	7.3125489	7.4065402	7.2268928
AT5G61130	104.57163	0.7458935	0.2111126	3.5331545	4.11E-04	0.0040656	7.2392665	6.9787205	6.9741037	6.163252	6.2366987	6.3821874
AT5G13510	536.70061	0.7465242	0.1882411	3.9657871	7.32E-05	0.0009151	9.112254	9.3066082	9.7310956	8.7171607	8.4977674	8.6700833
AT1G14360	91.707507	0.7469391	0.2134729	3.4989887	0.000467	0.0045149	6.9928396	6.8309826	6.7971134	6.0770468	6.0331881	6.1461153
AT3G16800	456.83824	0.7471015	0.1421758	5.2547714	1.48E-07	3.55E-06	9.1932384	9.1772698	9.1488046	8.3996454	8.2222785	8.5649985
AT5G05370	90.484232	0.7474478	0.2271193	3.290992	0.0009983	0.008702	6.8057194	6.8369536	7.0106512	5.4117401	6.3286119	6.1461153
AT5G03985	1215.468	0.7505914	0.1710718	4.3875807	1.15E-05	1.78E-04	10.396021	10.503345	10.813194	10.068089	9.5885484	9.7434405
AT2G26080	1541.9234	0.7532168	0.1027727	7.3289558	2.32E-13	1.44E-11	10.868719	10.948096	10.944931	10.226647	10.112752	10.148874
AT1G01540	587.00716	0.7556146	0.1584256	4.7695247	1.85E-06	3.50E-05	9.5820669	9.4133606	9.627046	8.5828465	8.6180063	9.0018099
AT5G52780	288.80252	0.7566974	0.1865252	4.0568116	4.97E-05	0.0006484	8.5775629	8.3418016	8.6298486	7.7626156	7.4065402	7.9622099
AT1G77090	184.47534	0.7574867	0.1845744	4.1039632	4.06E-05	0.0005435	7.7611274	7.9149001	7.9307056	7.2350505	6.7831397	7.2268928
AT5G45775	615.25514	0.757712	0.1172635	6.4616175	1.04E-10	4.18E-09	9.6295765	9.5801611	9.6013158	8.786708	8.8215359	8.8738974
AT5G07860	109.91652	0.7583931	0.2303715	3.2920433	9.95E-04	8.68E-03	7.0676446	7.1467733	7.2587115	6.0770468	5.996293	6.7164413
AT3G19450	141.85552	0.7586952	0.1925953	3.9393231	8.17E-05	1.01E-03	7.6437883	7.4097132	7.4077436	6.875214	6.5484377	6.7320595
AT3G51820	557.30125	0.7588808	0.148572	5.1078325	3.26E-07	7.25E-06	9.5301079	9.3385247	9.4849113	8.9288451	8.4911527	8.6414228
AT3G59410	1082.4552	0.7590113	0.1238617	6.1278914	8.91E-10	3.23E-08	10.385094	10.323124	10.523962	9.7506797	9.6521931	9.5388774
AT2G42320	497.26191	0.759533	0.1433709	5.2976799	1.17E-07	2.87E-06	9.2285984	9.3479636	9.3464181	8.2302543	8.5935123	8.6455522
AT3G03025	154.09013	0.7600636	0.2295097	3.3116835	9.27E-04	0.0081803	7.4295603	7.4838905	7.911506	6.9254163	6.4150185	7.0387661
AT1G10830	116.28243	0.761514	0.203614	3.7399887	1.84E-04	2.04E-03	7.1584528	7.0879032	7.3847234	6.4642672	6.3867874	6.4212482
AT1G68590	592.37949	0.7618959	0.1643401	4.6360911	3.55E-06	6.28E-05	9.4898725	9.3645929	9.7626882	8.9410983	8.5748649	8.783103
AT4G04020	518.95479	0.7627776	0.2030292	3.7569847	0.000172	0.0019294	9.0224279	9.3332541	9.6619314	8.786708	8.6180063	8.2763382
AT2G46820	3070.778	0.7635801	0.1919683	3.9776353	6.96E-05	8.76E-04	11.77515	11.832169	12.149655	11.471767	10.752946	11.083509
AT1G79790	337.14892	0.763813	0.1567807	4.8718563	1.11E-06	2.20E-05	8.6172363	8.7646298	8.8288769	8.0382954	7.75465	8.0644475
AT1G16080	375.08469	0.7639397	0.1740359	4.3895531	1.14E-05	0.0001771	8.7497075	8.874661	9.0395605	8.2696528	7.787414	8.2330877
AT2G44230	223.30013	0.7641563	0.1776469	4.3015454	1.70E-05	0.0002515	8.0726726	8.0572407	8.2716688	7.6476919	7.1110995	7.3433248
AT5G56850	236.39269	0.7654252	0.1944944	3.9354621	8.30E-05	1.02E-03	8.088156	8.4683614	8.0728656	7.7347315	7.3344659	7.2703267
AT4G37040	233.18258	0.7674548	0.1809989	4.2401076	2.23E-05	3.21E-04	8.1777213	8.0648927	8.4126223	7.2350505	7.3491717	7.5946624
AT1G61100	795.64853	0.7687732	0.1336614	5.7516473	8.84E-09	2.62E-07	9.9048942	10.032508	10.010635	8.965297	9.3461903	9.2085298
AT1G79850	1271.7646	0.769407	0.1733385	4.4387551	9.05E-06	0.0001442	10.530549	10.447852	10.942569	9.9994417	9.6432711	9.9208649
AT1G09927	1270.4501	0.7702331	0.1735752	4.4374602	9.10E-06	0.000145	10.529132	10.446388	10.94178	9.9994417	9.6402848	9.9174537
AT2G24860	175.3429	0.7702511	0.1863284	4.1338355	3.57E-05	4.85E-04	7.6332721	7.8602479	7.8985629	7.1531516	6.7831397	7.0881471
AT3G51860	131.65087	0.7709912	0.2190751	3.5193008	4.33E-04	0.0042497	7.0831818	7.5435271	7.5176377	6.6548684	6.7166491	6.40185

AT5G38520	363.05519	0.7717212	0.1643799	4.6947429	2.67E-06	4.85E-05	8.6849918	8.8394041	8.9961729	8.2302543	7.8404194	8.1009928
AT1G67090	39807.736	0.7721556	0.1712378	4.5092594	6.51E-06	0.0001073	15.586274	15.381185	15.870249	14.909263	14.54113	14.986731
AT2G25840	129.8798	0.7733973	0.1943571	3.9792584	6.91E-05	8.71E-04	7.2530694	7.5140169	7.3518652	6.5940902	6.6229419	6.5145098
AT5G18500	448.45285	0.7734009	0.1467404	5.2705366	1.36E-07	3.28E-06	9.0983034	9.1379087	9.2529004	8.0832144	8.5174315	8.3988151
AT3G56070	203.86873	0.7741937	0.185873	4.1651762	3.11E-05	0.0004293	8.033221	8.2191535	7.8149264	7.0663221	7.3344659	7.2158269
AT3G15354	148.24685	0.7743847	0.1894335	4.0878961	4.35E-05	0.0005772	7.5122328	7.6491069	7.5091477	6.875214	6.9075785	6.5324599
AT1G17360	380.81289	0.7745916	0.1652011	4.6887794	2.75E-06	4.98E-05	8.8038509	9.0672847	8.864257	8.2101441	8.2694284	7.8874681
AT3G14210	11989.923	0.7771819	0.1513306	5.1356574	2.81E-07	6.37E-06	13.754263	13.853939	14.057928	13.337007	12.92176	12.994118
AT1G23740	461.26344	0.7773194	0.1700936	4.5699506	4.88E-06	8.27E-05	9.1008499	9.1607021	9.2930175	8.7453832	8.1230878	8.3079459
AT1G27385	194.39318	0.7776087	0.1907032	4.0775855	4.55E-05	5.99E-04	7.8210426	7.9205343	8.1622147	6.7692438	7.3196087	7.2158269
AT3G03475	762.76304	0.7780257	0.1484008	5.2427332	1.58E-07	3.77E-06	9.764186	9.918879	10.082497	8.9040219	9.1792882	9.2334343
AT1G73530	180.93861	0.7781233	0.1866264	4.1694174	3.05E-05	4.23E-04	7.8696366	7.6958523	8.0260264	6.7692438	7.0937393	7.1706945
AT4G01690	786.19158	0.7783869	0.1289961	6.0341911	1.60E-09	5.45E-08	9.8362548	9.9618625	10.081781	9.187277	9.1032514	9.2168792
AT3G25500	208.49395	0.7788112	0.2089439	3.7273696	1.93E-04	2.14E-03	7.8485778	8.0926077	8.269157	6.7131893	7.5278411	7.2809839
AT1G64860	908.68925	0.7792914	0.188822	4.1271218	3.67E-05	0.0004973	9.9938963	10.427716	10.063053	9.6938847	9.0813989	9.2793346
AT2G34770	712.27097	0.7802684	0.1800402	4.3338574	1.47E-05	0.0002202	9.820891	9.6707031	9.9939992	8.731341	8.8834626	9.3289639
AT2G24020	530.79788	0.7806202	0.1703132	4.5834392	4.57E-06	7.83E-05	9.3534436	9.3922273	9.4575942	8.7028397	8.2222785	8.8017248
AT4G24780	329.88393	0.781558	0.2140462	3.6513521	0.0002609	0.0027505	8.5610131	8.7879342	8.7643481	8.4172953	7.4885299	7.7095654
AT5G46800	332.8651	0.7824691	0.1951625	4.00932	6.09E-05	7.74E-04	8.7333885	8.5542261	8.9261526	7.6476919	7.7323866	8.2385654
AT3G09905	233.33811	0.7826797	0.190815	4.1017729	4.10E-05	5.47E-04	8.0570212	8.0851017	8.4901624	7.4215092	7.2894272	7.4974886
AT3G06680	168.07304	0.7827413	0.1749081	4.4751579	7.64E-06	1.23E-04	7.7252672	7.7347485	7.7551512	7.1531516	6.7391548	6.961373
AT5G13770	769.72117	0.7833889	0.1276836	6.1353942	8.49E-10	3.10E-08	9.8446357	10.033158	9.9118439	9.2278502	9.0183901	9.1689141
AT3G41761	819.0552	0.7834989	0.1558579	5.0270065	4.98E-07	1.07E-05	9.8270562	10.023374	10.213035	9.1560802	9.0945501	9.3819013
AT1G70410	690.2428	0.7851785	0.129181	6.078126	1.22E-09	4.28E-08	9.7601641	9.7264468	9.8238708	9.1769528	8.8579821	8.9189448
AT4G29060	1698.8687	0.7856297	0.1632772	4.8116322	1.50E-06	2.89E-05	10.795545	11.302203	11.093947	10.44457	10.161475	10.194684
AT3G23410	92.582475	0.7857288	0.2279524	3.4468992	5.67E-04	0.0053551	7.1437095	6.8068484	6.7474485	5.9853613	6.1042628	6.0987114
AT3G07860	253.38549	0.7866277	0.1684749	4.6691102	3.03E-06	5.43E-05	8.2846252	8.2751991	8.401197	7.8690562	7.3637291	7.3932938
AT1G55335	528.6221	0.7870897	0.1784867	4.409795	1.03E-05	0.0001631	9.3662009	9.2783642	9.5700253	8.2500881	8.5043519	8.8738974
AT1G55330	528.7028	0.7874224	0.1784617	4.4122763	1.02E-05	1.61E-04	9.3672589	9.2783642	9.5700253	8.2500881	8.5043519	8.8738974
AT5G28750	509.64119	0.7883945	0.1550313	5.085388	3.67E-07	8.08E-06	9.1545406	9.3311404	9.5212515	8.6137933	8.4093163	8.582335
AT1G48600	449.23813	0.788558	0.1701195	4.6353173	3.56E-06	6.30E-05	8.8784714	9.3374722	9.2285421	8.4858118	8.2694284	8.3079459
AT3G03840	67.961636	0.7891653	0.2381535	3.313684	9.21E-04	8.14E-03	6.5760926	6.3695703	6.4162149	5.7824096	5.5637694	5.5828329
AT4G28740	138.33072	0.7896406	0.1849325	4.2698857	1.96E-05	2.85E-04	7.4576475	7.3935989	7.5344691	6.8232017	6.6229419	6.5677036

AT3G02730	631.55951	0.7897463	0.1851176	4.266187	1.99E-05	2.89E-04	9.386172	9.5404626	9.9771689	8.9532484	8.6832965	8.8668404
AT2G23672	156.51491	0.7899992	0.2081287	3.7957251	0.0001472	0.0016888	7.6986156	7.4800801	7.692792	7.2743201	6.7391548	6.4963335
AT4G24660	92.695643	0.790301	0.2327255	3.3958504	6.84E-04	0.006306	6.7418914	6.9678779	6.9429239	6.4642672	5.6133433	6.122608
AT5G30510	2115.8244	0.7905151	0.157198	5.0287843	4.94E-07	1.06E-05	11.213913	11.35595	11.601171	10.544324	10.393771	10.780645
AT2G06520	12111.861	0.7907182	0.1689107	4.6812799	2.85E-06	5.14E-05	13.796505	13.739765	14.173836	13.222459	12.816561	13.215993
AT1G32470	1271.4471	0.7918442	0.1555367	5.091043	3.56E-07	7.87E-06	10.56046	10.578328	10.842212	9.7850737	9.6551548	10.065032
AT5G65010	1165.3595	0.7919722	0.1228801	6.4450808	1.16E-10	4.65E-09	10.511535	10.565349	10.511795	9.9025153	9.7188214	9.5761316
AT4G28750	12448.434	0.7920128	0.1774114	4.4642719	8.03E-06	1.29E-04	13.814167	13.755219	14.251523	13.257788	12.848287	13.257393
AT4G26690	626.49222	0.7921544	0.1528692	5.1819104	2.20E-07	5.08E-06	9.5139571	9.5229765	9.8668675	8.8136145	8.8885052	8.783103
AT4G04570	416.06459	0.7923194	0.2095924	3.7802858	1.57E-04	1.78E-03	9.4614212	8.7755521	8.8592556	8.0832144	8.3593428	8.2053825
AT1G35560	64.674728	0.79399	0.2436648	3.2585335	0.0011199	0.0095875	6.3635078	6.4021533	6.3700772	5.7824096	5.6612702	5.2769537
AT5G66590	548.85305	0.7956244	0.1494716	5.3229155	1.02E-07	2.53E-06	9.4334081	9.2947262	9.6241004	8.5828465	8.562298	8.7451226
AT4G32790	83.043633	0.7969093	0.2316671	3.4398893	0.000582	0.0054769	6.8608456	6.686352	6.7971134	5.2634978	6.2047119	5.94637
AT1G03630	640.46051	0.7972971	0.1642513	4.8541313	1.21E-06	2.38E-05	9.534824	9.6681935	9.7936038	9.1769528	8.6774817	8.7061151
AT5G09855	230.56036	0.7985059	0.2102668	3.7975842	1.46E-04	0.0016776	8.1631746	7.9007175	8.530573	7.1946821	7.5407104	7.33312
AT3G52150	924.54566	0.7996927	0.1627945	4.9122834	9.00E-07	1.84E-05	9.9415828	10.201123	10.44103	9.4063729	9.2632116	9.4706923
AT5G62790	671.7227	0.8005462	0.1778754	4.5006006	6.78E-06	0.0001115	9.3683161	9.9040248	9.9174689	8.8914483	8.9134569	8.945983
AT2G47160	161.1155	0.8011975	0.1841663	4.350403	1.36E-05	0.0002065	7.6748846	7.7089345	7.7729907	6.3946946	7.0403691	6.961373
AT5G06980	795.11737	0.8032915	0.1620801	4.9561382	7.19E-07	1.50E-05	10.206857	9.8592555	9.8899398	9.1455296	8.971637	9.3417453
AT1G48355	421.60416	0.8053882	0.1778499	4.5284724	5.94E-06	9.91E-05	8.890296	9.0129403	9.3028749	8.3636843	7.9886655	8.3641539
AT2G23670	155.27283	0.8055698	0.2098447	3.8388853	1.24E-04	1.44E-03	7.6986156	7.468588	7.6852752	7.2743201	6.6705562	6.4963335
AT4G17600	411.4763	0.8062033	0.1675573	4.8115069	1.50E-06	2.89E-05	8.9078532	9.035184	9.1419566	8.5512213	8.0529572	8.0458217
AT2G17230	183.66885	0.806247	0.2243195	3.5941911	3.25E-04	3.34E-03	7.8210426	7.5972826	8.2101439	6.875214	7.3045969	6.8654991
AT2G03350	183.52466	0.8064046	0.1737833	4.6402886	3.48E-06	6.16E-05	7.8455442	7.9566301	7.905049	6.7131893	7.0937393	7.2046755
AT1G53730	320.03285	0.8066594	0.1471921	5.4803161	4.25E-08	1.13E-06	8.6541149	8.6907214	8.7355242	7.5866041	7.9409229	7.9422067
AT2G37600	268.09766	0.8067622	0.1583635	5.094371	3.50E-07	7.75E-06	8.3757641	8.324906	8.5657948	7.6476919	7.5534658	7.6031803
AT5G12470	213.72053	0.808583	0.1810736	4.465494	7.99E-06	0.0001286	8.1361186	8.0418139	8.0985593	7.5550621	6.9075785	7.33312
AT1G50250	933.17208	0.8087151	0.1372661	5.8915882	3.83E-09	1.20E-07	10.019022	10.314591	10.31397	9.4151838	9.3351522	9.4353519
AT2G33250	124.44614	0.8111107	0.2127593	3.8123397	1.38E-04	0.0015943	7.128814	7.3230308	7.4877016	6.7692438	6.2047119	6.4963335
AT1G75100	700.13203	0.8128636	0.1182819	6.8722593	6.32E-12	3.09E-10	9.8769379	9.7621861	9.776019	9.0355488	8.9525032	8.9692328
AT1G01790	491.77658	0.8131008	0.1636157	4.9695778	6.71E-07	1.40E-05	9.0634778	9.3385247	9.467488	8.5828465	8.5109065	8.3131472
AT5G18050	459.44882	0.8153671	0.1981225	4.1154697	3.86E-05	0.0005202	9.3362565	8.9741852	9.310224	8.2889558	8.0073288	8.6741315
AT1G70230	121.67855	0.8157369	0.1999835	4.0790215	4.52E-05	5.96E-04	7.1337963	7.3773025	7.3847234	6.4642672	6.3867874	6.4963335

AT1G14150	190.49513	0.8160825	0.2095389	3.894658	9.83E-05	1.18E-03	7.7929716	7.8095295	8.1730041	7.3860957	6.7166491	7.1475875
AT4G15800	275.9509	0.8160941	0.2190695	3.7252744	1.95E-04	2.15E-03	8.3799637	8.2663768	8.7607764	7.4560742	7.9792425	7.3124911
AT4G01883	65.024016	0.817794	0.2476335	3.3024364	0.0009585	0.0084011	6.2209611	6.4574606	6.5042865	5.7824096	5.5124312	5.3993805
AT4G39800	1662.5198	0.8180588	0.1999196	4.091938	4.28E-05	5.69E-04	10.788849	11.130224	11.235127	10.607189	9.9606547	9.9710871
AT5G01590	351.24837	0.8191312	0.183031	4.4753688	7.63E-06	1.23E-04	8.4928474	8.9217739	8.9961729	8.1051601	7.8915461	7.9354769
AT4G32590	322.39449	0.8195234	0.1682154	4.8718693	1.11E-06	2.20E-05	8.542399	8.6369784	8.9052753	7.789971	7.7765751	7.9622099
AT5G08130	858.3975	0.8197826	0.1188917	6.8952063	5.38E-12	2.66E-10	10.039741	10.082997	10.185992	9.2962184	9.1915803	9.3238194
AT4G26860	201.27854	0.8198173	0.1640181	4.9983349	5.78E-07	1.22E-05	8.0674743	7.9345244	8.0670932	7.11039	7.242937	7.1591873
AT1G52240	1336.1887	0.8202272	0.1737477	4.7207943	2.35E-06	4.33E-05	10.627341	10.624366	10.957467	10.186039	9.622236	9.8474804
AT1G10020	269.96463	0.821023	0.1820624	4.5095699	6.50E-06	1.07E-04	8.5235416	8.5063233	8.3498218	7.11039	7.8713123	7.6116481
AT3G11120	366.9434	0.8211438	0.1483665	5.5345622	3.12E-08	8.41E-07	8.7594111	8.9329672	8.9670222	7.9440305	7.9886655	8.1541316
AT4G24930	117.84735	0.8221401	0.2057023	3.9967469	6.42E-05	0.0008131	7.1036412	7.2262632	7.425899	6.4642672	6.2986201	6.4403891
AT1G12800	668.03317	0.8232928	0.1518989	5.4200058	5.96E-08	1.54E-06	9.6216183	9.7700093	9.8204436	9.1665543	8.6832965	8.8561899
AT4G25890	213.3813	0.8235856	0.1635307	5.0362753	4.75E-07	1.02E-05	8.1135981	8.0104564	8.2022654	7.1946821	7.242937	7.3124911
AT2G47440	530.30949	0.8239837	0.1896191	4.3454672	1.39E-05	2.10E-04	9.3133832	9.20177	9.7043844	8.4172953	8.8109514	8.3988151
AT4G14560	303.97888	0.8251402	0.2142602	3.8511135	1.18E-04	1.38E-03	8.7545675	8.6075917	8.5051825	7.789971	7.2271013	8.0949659
AT1G04420	497.09426	0.8266929	0.1604226	5.1532187	2.56E-07	5.83E-06	9.1715987	9.3395766	9.4319972	8.673764	8.2062131	8.5296878
AT1G63090	247.51535	0.8271886	0.1857676	4.4528133	8.48E-06	1.35E-04	8.3799637	8.2485688	8.3827261	7.0208656	7.787414	7.4030832
AT1G33811	245.73061	0.8286495	0.1771509	4.677647	2.90E-06	5.22E-05	8.4733266	8.0775564	8.3640157	7.4215092	7.501753	7.432059
AT2G05100	22763.456	0.829316	0.1552408	5.3421246	9.19E-08	2.29E-06	14.758224	14.68037	15.052144	14.193546	13.785878	13.946184
AT2G00550	103.78585	0.8310237	0.2067641	4.0191879	5.84E-05	7.44E-04	6.9651548	7.1028474	7.1699376	6.2445954	6.1042628	6.2796418
AT1G72790	208.83366	0.8310904	0.2212944	3.755587	1.73E-04	0.0019382	8.038544	7.7757353	8.3687161	7.3125489	7.4065402	6.8795917
AT5G65810	100.51588	0.8321442	0.2129688	3.9073519	9.33E-05	1.13E-03	6.9539304	7.0212939	7.0637929	6.4642672	6.2047119	5.8917921
AT2G26550	120.79969	0.8322891	0.2006976	4.1469815	3.37E-05	0.0004606	7.1584528	7.4296063	7.2838147	6.4642672	6.496541	6.3420395
AT2G36620	546.74304	0.8330987	0.1419683	5.8682004	4.41E-09	1.36E-07	9.3254096	9.486406	9.5801933	8.4347319	8.6832965	8.6619525
AT3G20362	83.055331	0.8336439	0.2291856	3.637418	2.75E-04	2.88E-03	6.7021936	6.686352	6.8652226	6.163252	5.5637694	5.94637
AT5G62220	133.86989	0.8340363	0.2333833	3.5736762	3.52E-04	3.57E-03	7.2621985	7.4137138	7.6078713	6.9739302	5.996293	6.6522207
AT4G23940	167.29851	0.8350104	0.2076795	4.0206687	5.80E-05	7.41E-04	7.4416647	7.9483801	7.8388375	7.0663221	6.8467003	6.7928944
AT2G24090	927.44768	0.8351889	0.1538298	5.4293061	5.66E-08	1.47E-06	10.090595	10.140857	10.415703	9.5004538	9.1543852	9.4329649
AT4G17300	189.89579	0.8353454	0.1711195	4.8816499	1.05E-06	2.11E-05	7.8179504	7.9729899	8.0378796	7.0663221	7.0583794	7.112218
AT4G21210	324.79486	0.8358595	0.2263558	3.6926803	2.22E-04	2.41E-03	8.3820589	8.8123842	8.8873708	8.3268039	7.5910692	7.5246462
AT3G01810	166.35275	0.8362825	0.1847097	4.5275517	5.97E-06	9.94E-05	7.6402914	7.8125628	7.8217986	6.875214	6.6937867	7.0512707
AT2G42220	1905.4671	0.8367551	0.169638	4.9325935	8.11E-07	1.67E-05	11.241004	11.16624	11.378648	10.591728	10.010075	10.534571

AT1G06717	160.5143	0.8368778	0.1994491	4.1959472	2.72E-05	0.0003811	7.6048482	7.5471738	7.9179343	7.0208656	6.6469455	6.8512676
AT1G03940	135.14216	0.8386952	0.2205999	3.8018835	1.44E-04	1.65E-03	7.2981481	7.6592521	7.3707328	6.7692438	6.7613149	6.2144228
AT2G28000	1618.341	0.8390382	0.1648965	5.0882714	3.61E-07	7.97E-06	10.938715	10.993241	11.141757	10.461682	9.8564266	10.12689
AT1G44446	1974.4685	0.8395303	0.128066	6.5554508	5.55E-11	2.31E-09	11.347774	11.30436	11.277298	10.663737	10.281335	10.418711
AT4G30620	140.51871	0.8412518	0.213389	3.9423386	8.07E-05	0.000997	7.6645935	7.5065441	7.4703122	5.9853613	6.6469455	6.9480607
AT1G09200	131.50102	0.8422819	0.2519608	3.3429082	8.29E-04	7.44E-03	7.2621985	7.4097132	7.5469651	7.0663221	5.7961798	6.5677036
AT3G07460	754.37734	0.8434159	0.1549022	5.4448275	5.19E-08	1.36E-06	9.9092592	9.7029367	10.148217	9.0240752	9.1204982	9.0368151
AT4G39363	447.66729	0.8437654	0.1986428	4.2476523	2.16E-05	3.11E-04	8.9578741	9.3666581	9.166459	8.7171607	8.0348786	8.1129718
AT1G78230	129.82643	0.8438821	0.2146069	3.932223	8.42E-05	0.0010295	7.1238145	7.4990324	7.5302796	6.7692438	6.3286119	6.4963335
AT2G04160	104.28862	0.8439526	0.2448309	3.4470835	0.0005667	0.0053542	6.7548849	7.3854737	7.0462956	6.4642672	6.1719999	5.9989583
AT1G48480	178.96278	0.8445824	0.1732119	4.876006	1.08E-06	2.17E-05	7.8394577	7.9593696	7.8010828	6.9254163	6.9075785	7.1002328
AT2G47450	1810.2263	0.8449048	0.154585	5.465634	4.61E-08	1.22E-06	10.985286	11.397955	11.15848	10.520024	10.240507	10.201712
AT1G76090	517.41125	0.8453702	0.1802037	4.6911913	2.72E-06	4.92E-05	9.2912566	9.2269982	9.6455628	8.1690626	8.6480505	8.624786
AT3G03770	76.702206	0.8464758	0.2351003	3.6004886	3.18E-04	0.0032617	6.4932279	6.8249868	6.6581167	5.669115	5.8384847	5.7455674
AT5G39210	62.599922	0.8468776	0.2500467	3.3868774	7.07E-04	0.0064899	6.3464409	6.3362345	6.4162149	5.669115	5.0904916	5.6168815
AT3G47650	501.05512	0.8475497	0.1716311	4.9382056	7.88E-07	1.63E-05	9.1932384	9.2439498	9.5474002	8.731341	8.2538823	8.4182539
AT4G34530	420.93417	0.8487451	0.1675294	5.0662471	4.06E-07	8.85E-06	8.8932371	9.1737356	9.2142497	8.0609297	8.1058722	8.4182539
AT1G76110	311.21187	0.8488445	0.1664739	5.098966	3.42E-07	7.57E-06	8.7035408	8.6758074	8.6415484	7.4560742	7.7097742	8.0395593
AT4G18740	313.34782	0.8500906	0.1547927	5.4918018	3.98E-08	1.06E-06	8.8038509	8.568651	8.6337591	7.6772924	7.9015576	7.7713361
AT5G40950	1357.7798	0.8524396	0.1498818	5.6874112	1.29E-08	3.69E-07	10.610016	10.726801	10.974912	10.039882	9.7216496	9.9445196
AT1G56045	553.67676	0.8537617	0.1436196	5.9446043	2.77E-09	8.96E-08	9.5460799	9.3109047	9.5993174	8.5351448	8.5996749	8.6821941
AT1G49975	220.11818	0.8544321	0.2155631	3.9637217	7.38E-05	9.22E-04	8.1607358	8.0648927	8.3210083	6.9254163	6.9849484	7.677659
AT5G54380	360.0116	0.8546054	0.1757718	4.8620172	1.16E-06	2.30E-05	8.7833882	8.8541993	8.997691	7.6476919	8.2848087	7.9013487
AT4G08870	182.16973	0.8546814	0.2446194	3.4939228	4.76E-04	4.59E-03	7.4851983	8.2508068	7.848965	7.1946821	7.1282532	6.6190056
AT2G21340	330.54671	0.8550569	0.1500792	5.697372	1.22E-08	3.51E-07	8.6849918	8.7005791	8.8692413	7.7062978	7.8814647	7.9622099
AT1G50320	456.1387	0.85592	0.1762023	4.8575976	1.19E-06	2.34E-05	9.0143461	9.1048858	9.4718636	8.4347319	8.1485321	8.3889965
AT1G47380	101.93638	0.8567772	0.2220053	3.8592644	0.0001137	0.0013378	6.8668427	7.1274156	7.1753136	6.3215962	5.8795843	6.2796418
AT3G11170	886.45273	0.8582541	0.1665903	5.1518855	2.58E-07	5.86E-06	10.183621	10.063395	10.245342	9.5169193	8.9183958	9.3694722
AT5G18010	173.06627	0.8622151	0.1973358	4.3692785	1.25E-05	1.92E-04	7.945269	7.6524966	7.872323	6.9254163	6.6469455	7.1358938
AT4G38860	908.74345	0.8624611	0.1444961	5.9687508	2.39E-09	7.83E-08	10.224479	10.10605	10.263123	9.5412705	9.0813989	9.3315292
AT2G36792	1241.0115	0.863097	0.2435369	3.5440095	3.94E-04	3.92E-03	10.293448	10.644508	10.986427	9.9994417	9.9630468	9.0923252
AT3G55040	199.2028	0.8643507	0.1912097	4.5204334	6.17E-06	1.03E-04	7.9108537	7.8950051	8.1996297	7.4560742	7.0036595	6.961373
AT3G26710	377.62756	0.8644973	0.1688417	5.1201661	3.05E-07	6.85E-06	8.9005637	9.0155751	8.9181586	8.0609297	7.7435613	8.2816545

AT2G36790	1237.7828	0.8647544	0.2440976	3.5426588	0.0003961	0.0039396	10.288989	10.64323	10.982982	9.9935709	9.9606547	9.0832206
AT2G44650	417.97894	0.8655195	0.1914261	4.5214292	6.14E-06	0.0001022	8.9884507	8.9836357	9.310224	7.9194695	8.0165706	8.5071716
AT1G24170	340.9797	0.8656144	0.2139141	4.0465513	5.20E-05	0.0006735	8.7738451	8.7184784	8.9020364	7.9440305	8.246046	7.3635206
AT4G18480	1577.8562	0.8658925	0.14124	6.1306467	8.75E-10	3.18E-08	10.797508	11.073824	11.105609	10.261264	9.9678192	10.110553
AT2G36870	94.842407	0.866961	0.2414	3.5913879	3.29E-04	0.0033653	7.0519383	6.9292741	6.8518556	6.5306385	5.4591985	6.0987114
AT4G28755	4717.0121	0.8674911	0.1795503	4.8314664	1.36E-06	2.64E-05	12.471854	12.362806	12.871841	11.825752	11.383445	11.798893
AT3G09200	3374.6933	0.8683227	0.1228452	7.0684308	1.57E-12	8.54E-11	11.975421	12.056638	12.240609	11.315826	11.204967	11.115121
AT2G17972	109.88093	0.8703688	0.2105494	4.1337981	3.57E-05	0.0004845	7.0728423	7.3144971	7.201897	5.8874513	6.2679916	6.4592793
AT1G45474	465.36903	0.8709946	0.1722528	5.0564905	4.27E-07	9.31E-06	9.0048597	9.2269982	9.4586968	8.5188872	8.1735355	8.3490403
AT1G12900	4203.1397	0.8712825	0.1420945	6.1317125	8.69E-10	3.16E-08	12.239985	12.397562	12.588652	11.610567	11.333476	11.60948
AT4G14680	77.181539	0.8719081	0.2476	3.5214382	4.29E-04	4.22E-03	6.6405204	6.9010534	6.5628862	5.2634978	5.6612702	6.0496968
AT3G54500	2615.199	0.8721475	0.1528941	5.7042572	1.17E-08	3.38E-07	11.98355	11.662246	11.517928	10.700241	10.89877	10.892589
AT3G12345	846.44992	0.8729469	0.1549096	5.6352028	1.75E-08	4.90E-07	10.041068	10.035757	10.233795	9.2962184	8.9282233	9.3694722
AT3G50800	66.887076	0.8747248	0.2560768	3.4158696	6.36E-04	5.93E-03	6.4298254	6.4728809	6.4697003	5.7824096	5.7961798	5.0465438
AT4G03210	827.48989	0.8753033	0.173769	5.0371659	4.72E-07	1.02E-05	10.069298	10.037702	10.149583	8.731341	9.1874946	9.4543078
AT3G48720	474.21563	0.8762358	0.1933588	4.5316563	5.85E-06	9.80E-05	9.3767464	8.978243	9.4597987	8.126777	8.2302446	8.6372815
AT2G32230	150.77756	0.8768771	0.188703	4.6468646	3.37E-06	5.99E-05	7.53877	7.7632475	7.5552361	6.8232017	6.7831397	6.5850078
AT5G17870	621.36037	0.8784483	0.1750759	5.0175278	5.23E-07	1.12E-05	9.533882	9.4826022	9.9421193	8.7453832	8.5935123	8.9017849
AT1G48350	1154.3668	0.8784577	0.1497551	5.8659602	4.47E-09	1.38E-07	10.431353	10.439046	10.768098	9.7366892	9.4924623	9.7240458
AT4G04565	11752.211	0.8791372	0.2394605	3.6713243	0.0002413	0.0025725	14.011176	13.757729	13.976356	12.512119	12.61494	13.541635
AT1G43560	167.85416	0.8822386	0.2289694	3.8530855	0.0001166	0.0013684	7.5084016	7.6958523	8.1070232	6.9739302	6.5227227	7.0261521
AT1G06690	164.26457	0.8846091	0.2248897	3.9335253	8.37E-05	1.03E-03	7.4536684	7.7757353	8.0110718	6.875214	6.496541	7.0387661
AT3G59780	1166.1321	0.8856077	0.1453699	6.0920982	1.11E-09	3.94E-08	10.416097	10.656374	10.62171	9.87084	9.4589569	9.6560828
AT5G62430	280.47339	0.8858615	0.1449578	6.1111674	9.89E-10	3.55E-08	8.5405243	8.49315	8.5136958	7.6772924	7.6156045	7.5688029
AT5G16400	580.462	0.8871041	0.1905744	4.6548956	3.24E-06	5.78E-05	9.2390396	9.5201961	9.8948362	8.5671205	8.5811075	8.7680306
AT3G12930	199.76403	0.8889173	0.1735832	5.1209873	3.04E-07	6.83E-06	7.931029	7.9756986	8.1916935	7.11039	7.1282532	7.112218
AT4G28210	98.346793	0.8898113	0.2532862	3.5130663	0.000443	0.0043314	6.9312162	6.8664434	7.3133731	5.8874513	5.707656	6.4403891
AT3G13470	376.39658	0.889978	0.1352111	6.5821369	4.64E-11	1.96E-09	8.9179971	8.9890082	8.9213615	8.0609297	7.9697576	8.0767324
AT1G56050	116.49071	0.8901075	0.2337985	3.8071559	1.41E-04	1.62E-03	6.9539304	7.3525075	7.4304023	6.5306385	6.4698754	5.9989583
AT1G80640	219.36632	0.8915222	0.1843713	4.8354714	1.33E-06	2.60E-05	8.3545808	8.0262203	8.1622147	6.9739302	7.2110898	7.4415898
AT1G09390	107.55296	0.8916992	0.1996561	4.4661748	7.96E-06	1.28E-04	7.128814	7.1274156	7.1966194	6.2445954	6.2679916	6.1692457
AT3G47070	660.35271	0.8922727	0.1638893	5.4443612	5.20E-08	1.36E-06	9.6260449	9.6360216	9.9810112	8.8914483	8.6361078	8.9626281
AT1G49380	206.78384	0.8928424	0.1692167	5.2763239	1.32E-07	3.19E-06	8.1060125	8.0800759	8.132121	6.7692438	7.2586008	7.3020649

AT5G12860	372.43991	0.8939465	0.2445852	3.6549494	2.57E-04	2.71E-03	8.4889643	8.9768917	9.2465301	8.4689853	7.7211247	7.677659
AT3G23390	399.56485	0.8945089	0.1485355	6.0221894	1.72E-09	5.84E-08	8.9704612	9.0660143	9.0845414	7.8690562	8.307576	8.0889136
AT4G13572	123.06303	0.8947888	0.1999389	4.4753109	7.63E-06	1.23E-04	7.4929743	7.3059127	7.2788289	6.163252	6.4150185	6.5501893
AT2G34070	285.11023	0.8950528	0.1868142	4.7911399	1.66E-06	3.17E-05	8.5848578	8.4293734	8.6855387	7.0663221	7.798172	7.7788748
AT5G10400	89.851801	0.8951258	0.2572044	3.4802118	5.01E-04	4.81E-03	6.6193626	6.9236739	7.1372542	6.0770468	5.4591985	6.1920111
AT3G12780	4638.0581	0.8963139	0.1727532	5.1884079	2.12E-07	4.91E-06	12.245893	12.599627	12.811083	11.799073	11.424816	11.675588
AT1G32990	864.64216	0.8992305	0.1900084	4.7325834	2.22E-06	4.10E-05	9.9085326	10.080483	10.423065	9.2278502	8.9085009	9.444861
AT1G05207	2872.4512	0.9000094	0.1725396	5.2162492	1.83E-07	4.27E-06	11.687015	11.752475	12.155447	11.143618	10.705161	10.965421
AT4G08930	237.8683	0.9018796	0.1720416	5.2422167	1.59E-07	3.77E-06	8.4595031	8.1368417	8.2716688	7.2743201	7.4481174	7.33312
AT1G27460	271.57224	0.9020612	0.1693205	5.3275377	9.96E-08	2.47E-06	8.2711223	8.5847085	8.5800516	7.4898305	7.5278411	7.6116481
AT2G33570	165.15738	0.903363	0.2435114	3.7097353	2.07E-04	2.27E-03	7.8241282	7.3935989	8.0349254	6.9254163	7.0761677	6.3821874
AT4G30950	1964.2913	0.9035638	0.1538164	5.8742998	4.25E-09	1.31E-07	11.227085	11.226329	11.517134	10.580022	10.157303	10.446195
AT2G01755	70.241609	0.9041314	0.2386664	3.7882649	1.52E-04	1.73E-03	6.5314847	6.5764409	6.5872896	5.5461594	5.3464515	5.8058471
AT5G15850	730.72018	0.9060613	0.1385362	6.5402515	6.14E-11	2.54E-09	10.069298	9.8077417	9.8255813	8.9410983	9.0681268	8.9189448
AT1G14453	163.70922	0.9073124	0.2294409	3.9544497	7.67E-05	0.0009515	7.6851029	7.6077975	8.0437698	6.0770468	6.9467821	7.0512707
AT5G51460	135.80995	0.908093	0.2140692	4.2420537	2.21E-05	3.18E-04	7.6748846	7.4762596	7.4077436	5.8874513	6.6229419	6.7928944
AT5G15845	739.92252	0.908595	0.1381429	6.5772108	4.79E-11	2.02E-09	10.086746	9.8318611	9.8425757	8.9410983	9.0901797	8.9426308
AT3G14870	74.714635	0.9089098	0.2656759	3.4211229	6.24E-04	5.83E-03	6.4932279	6.5107257	6.9174857	5.5461594	6.0331881	5.3189277
AT2G23120	736.85873	0.9097145	0.1321167	6.885691	5.75E-12	2.83E-10	10.00073	9.7653205	9.9679053	9.0805521	8.9282233	8.9725238
AT4G00810	286.71118	0.9100634	0.1702183	5.3464472	8.97E-08	2.24E-06	8.4233367	8.5304735	8.7373427	7.5228149	7.514856	7.7788748
AT4G12390	112.80322	0.9109665	0.2158527	4.2203148	2.44E-05	3.48E-04	7.2438821	7.0160404	7.3847234	6.3946946	6.3579929	6.0987114
AT3G55800	2440.4518	0.912469	0.1283799	7.1075709	1.18E-12	6.62E-11	11.542994	11.612642	11.768796	10.784256	10.543537	10.803924
AT4G16410	554.39628	0.9128583	0.1522837	5.994457	2.04E-09	6.78E-08	9.5072539	9.3531809	9.6455628	8.731341	8.3809723	8.6121814
AT1G64770	266.24712	0.9130445	0.1813406	5.0349711	4.78E-07	1.03E-05	8.2087448	8.6145602	8.5200479	7.5866041	7.3491717	7.5946624
AT5G59870	97.25768	0.9133342	0.2639525	3.4602222	0.0005397	0.0051387	6.9312162	6.9569533	7.2688053	5.4117401	5.7525968	6.5324599
AT1G19440	132.79817	0.9166396	0.2262983	4.0505812	5.11E-05	6.63E-04	7.2392665	7.3272788	7.7765323	6.5940902	6.3286119	6.5677036
AT4G17810	138.88933	0.9168711	0.2054029	4.4637686	8.05E-06	1.29E-04	7.4009154	7.3935989	7.7623135	6.5940902	6.5484377	6.5677036
AT1G07600	658.05345	0.9173926	0.1258429	7.2899813	3.10E-13	1.88E-11	9.7609694	9.7870726	9.7080983	8.9532484	8.6832965	8.8454601
AT3G61870	454.85723	0.9180315	0.1821457	5.0400944	4.65E-07	1.01E-05	9.0995772	9.1294195	9.4286248	8.5024443	7.9311818	8.3691568
AT3G59400	854.72864	0.9183171	0.152866	6.0073338	1.89E-09	6.35E-08	10.119766	9.970715	10.285667	9.4326457	9.0769884	9.0740581
AT5G48300	986.40496	0.9183213	0.1854436	4.9520245	7.34E-07	1.53E-05	10.04504	10.464821	10.483904	9.6497713	9.0725644	9.4113014
AT4G02540	315.68425	0.9183482	0.1945722	4.7198324	2.36E-06	4.34E-05	8.5348856	8.5249362	9.0052573	7.789971	7.8814647	7.5688029
AT3G12110	69.983706	0.920475	0.238444	3.8603398	1.13E-04	1.33E-03	6.4217005	6.6255604	6.6270664	5.5461594	5.5124312	5.650145

AT2G41250	455.20744	0.9207261	0.1554055	5.9246686	3.13E-09	1.00E-07	9.3986443	9.2144392	9.0425052	8.3996454	8.2616763	8.2109663
AT5G09650	609.2944	0.9207421	0.1372479	6.7086076	1.96E-11	8.76E-10	9.5072539	9.7021191	9.7374697	8.6137933	8.6774817	8.8017248
AT5G66190	3225.0563	0.9224273	0.1521559	6.0623828	1.34E-09	4.67E-08	11.891683	12.074753	12.17537	11.318177	10.849197	11.115865
AT1G04680	290.10945	0.9241344	0.1567179	5.8968011	3.71E-09	1.17E-07	8.4967201	8.5286301	8.691179	7.8168173	7.514856	7.6031803
AT2G24540	55.279141	0.9241808	0.2628002	3.5166666	4.37E-04	0.0042826	6.3291697	6.2759764	6.0336839	5.4117401	5.0904916	5.233722
AT1G63260	186.2035	0.9243933	0.1757307	5.2602846	1.44E-07	3.45E-06	7.9395899	7.8806246	8.0641983	6.7131893	7.0221311	7.1241044
AT1G56190	509.21363	0.9297067	0.1530611	6.0740871	1.25E-09	4.37E-08	9.213382	9.3901984	9.5587571	8.4858118	8.322557	8.5071716
AT1G29910	51546.07	0.9305594	0.1752448	5.310054	1.10E-07	2.70E-06	15.894782	15.875205	16.351387	15.278801	14.832281	15.124253
AT4G08805	89.519415	0.931459	0.2505731	3.7173139	0.0002014	0.0022129	6.8608456	6.9678779	7.0345117	4.9116189	5.9195455	6.3215387
AT3G05490	222.69396	0.9332888	0.2371086	3.9361235	8.28E-05	1.02E-03	8.2801383	7.8306299	8.4771623	7.1531516	7.5661095	6.8512676
AT5G13630	8274.2156	0.9336753	0.1717799	5.4353016	5.47E-08	1.43E-06	13.297168	13.465253	13.485527	12.798871	12.244537	12.237162
AT1G74670	5138.8193	0.9343556	0.1460532	6.3973653	1.58E-10	6.27E-09	12.52473	12.740468	12.895649	11.915425	11.579368	11.797502
AT3G07470	594.3003	0.934404	0.1888261	4.9484888	7.48E-07	1.55E-05	9.6330994	9.4193423	9.8118401	8.308004	8.971637	8.5518581
AT3G18890	583.36365	0.9352696	0.1940587	4.8195181	1.44E-06	2.79E-05	9.3577086	9.6437064	9.7392858	9.0125096	8.4911527	8.3183298
AT2G34620	335.75361	0.9363568	0.2024166	4.6258898	3.73E-06	6.56E-05	8.8301791	8.7457114	8.789105	8.308004	7.3781411	7.7174329
AT1G35680	1049.47	0.9364625	0.1641945	5.7033736	1.17E-08	3.39E-07	10.239002	10.343714	10.696526	9.5652174	9.2977362	9.54773
AT5G18255	722.36195	0.9375448	0.2273054	4.124604	3.71E-05	0.0005018	10.091875	9.5739181	10.0404	8.5188872	8.6832965	9.3569351
AT2G43560	494.3576	0.9378625	0.1590013	5.8984594	3.67E-09	1.16E-07	9.1471677	9.3469179	9.5411676	8.4689853	8.277139	8.4278759
AT2G10940	2910.6329	0.9379793	0.197274	4.7547034	1.99E-06	3.72E-05	11.666962	11.623046	12.344627	11.013317	10.856935	10.922675
AT3G52720	192.37807	0.9381548	0.24288	3.8626263	1.12E-04	1.33E-03	7.4496782	8.1774085	8.2666407	7.1946821	6.8258231	7.0005885
AT2G34860	381.32313	0.9390965	0.1852013	5.0706799	3.96E-07	8.68E-06	8.7317464	8.9090767	9.2630348	8.0832144	7.861088	8.0767324
AT1G35140	654.15417	0.9398785	0.2805925	3.3496209	0.0008092	0.0073045	9.7857124	9.5331259	10.005363	8.5512213	9.3680164	7.9287155
AT4G34290	118.74699	0.9403554	0.2125476	4.4242108	9.68E-06	1.54E-04	7.234636	7.2125501	7.5133989	6.163252	6.2047119	6.5145098
AT3G07705	91.253022	0.9414962	0.2550736	3.691077	0.0002233	0.0024187	7.0934478	6.686352	6.9679214	6.3946946	5.3464515	5.9989583
AT1G74070	143.66946	0.9420226	0.2180635	4.3199465	1.56E-05	2.33E-04	7.5758531	7.536206	7.6965358	6.3946946	6.2366987	6.961373
AT1G72450	543.06662	0.9421003	0.1372261	6.8653148	6.63E-12	3.20E-10	9.4703111	9.4892522	9.4989144	8.5828465	8.6598951	8.3439671
AT5G49170	58.831784	0.9432921	0.2562434	3.681235	2.32E-04	0.002499	6.4459391	6.30211	6.3126908	4.6971932	5.3464515	5.5479613
AT2G42130	193.92545	0.9440444	0.184204	5.1249952	2.98E-07	6.71E-06	7.8241282	8.0051632	8.1730041	7.1946821	6.9273135	7.0134269
AT3G25805	122.71285	0.9451502	0.2196778	4.3024391	1.69E-05	2.51E-04	7.3504466	7.1658746	7.5091477	6.7131893	5.996293	6.40185
AT4G36540	927.7392	0.9456874	0.1556337	6.0763676	1.23E-09	4.32E-08	10.33135	10.248931	10.23315	9.0125096	9.2118373	9.55872
AT5G45930	326.35487	0.9457938	0.1890449	5.0030105	5.64E-07	1.20E-05	8.4733266	8.8244556	8.9793681	7.5228149	7.8088504	7.9219223
AT5G52970	282.71369	0.9458343	0.2150875	4.3974393	1.10E-05	1.72E-04	8.2913295	8.49315	8.8475175	7.6772924	7.1785237	7.7713361
AT5G17170	282.26864	0.9476193	0.1988058	4.7665582	1.87E-06	3.55E-05	8.3046455	8.5230856	8.7679109	7.9194695	7.4617144	7.33312

AT3G13000	146.54322	0.9483058	0.1837863	5.1598279	2.47E-07	5.64E-06	7.5574307	7.6693264	7.6548102	6.3946946	6.6469455	6.7475104
AT2G46970	73.142807	0.9493224	0.2726227	3.4821839	4.97E-04	4.77E-03	6.8119495	6.4805297	6.5380629	6.0770468	4.8634794	5.7455674
AT3G08405	99.632181	0.9494424	0.2541875	3.7352052	1.88E-04	2.08E-03	7.4050425	6.9678779	6.7402118	6.2445954	6.1385289	5.8058471
AT4G09320	1337.0852	0.9497349	0.1386876	6.8480138	7.49E-12	3.58E-10	10.732076	10.658483	10.973756	9.6793296	9.8330775	9.9191603
AT1G14345	167.89266	0.9505095	0.1953862	4.8647721	1.15E-06	2.27E-05	7.7579039	7.6320382	7.9959604	7.0208656	6.6469455	6.8223762
AT1G21500	997.36243	0.9510922	0.1659466	5.7313158	9.97E-09	2.92E-07	10.181819	10.225794	10.656243	9.50871	9.3090632	9.3544146
AT5G35630	7405.478	0.9532943	0.1524266	6.2541196	4.00E-10	1.51E-08	12.952951	13.355538	13.437048	12.270128	12.192481	12.376596
AT5G24314	304.19804	0.9539278	0.160446	5.9454738	2.76E-09	8.92E-08	8.5628614	8.5864817	8.8305815	7.6476919	7.6277174	7.7484809
AT2G32500	89.571477	0.9543003	0.2386786	3.9982649	6.38E-05	0.0008087	6.8304803	6.8129199	7.1591252	5.5461594	5.7961798	6.1692457
AT5G08980	101.17592	0.9550087	0.2179046	4.3826928	1.17E-05	0.0001823	7.0200031	6.9894822	7.2738258	5.9853613	5.996293	6.2144228
AT1G70090	508.8616	0.956037	0.1751386	5.4587463	4.80E-08	1.27E-06	9.5253764	9.2562555	9.4420676	8.126777	8.717702	8.2974868
AT4G02530	602.61875	0.9583753	0.1747074	5.4856026	4.12E-08	1.10E-06	9.4038095	9.6360216	9.8627083	8.8530562	8.4232821	8.6862086
AT1G30380	9648.7263	0.958486	0.1763593	5.4348495	5.48E-08	1.43E-06	13.49563	13.473721	13.934218	12.603607	12.422817	12.892454
AT2G31751	456.56796	0.961692	0.1312607	7.3265781	2.36E-13	1.46E-11	9.2574172	9.1737356	9.3090018	8.2302543	8.2924378	8.2656465
AT1G75690	561.21118	0.9617021	0.1917059	5.0165497	5.26E-07	1.12E-05	9.4323975	9.429257	9.746527	8.9040219	8.1735355	8.5162203
AT5G64750	62.962826	0.9630174	0.2891019	3.3310653	0.0008651	0.0077178	6.0937824	6.6663714	6.5296927	4.9116189	5.8384847	5.0956639
AT2G27402	65.481602	0.9630339	0.2622495	3.6722048	2.40E-04	2.57E-03	6.6613724	6.1946089	6.6033322	5.0982599	5.5637694	5.5122259
AT1G49700	1244.4821	0.9640204	0.2557246	3.7697607	0.0001634	0.0018435	10.971437	10.290861	10.808445	9.2278502	9.3128192	10.208706
AT5G35480	61.262291	0.964035	0.2628988	3.6669437	0.0002455	0.0026081	6.583395	6.2759764	6.2930433	5.4117401	5.0187179	5.5479613
AT5G59920	143.28483	0.9643475	0.1895556	5.0874127	3.63E-07	8.00E-06	7.4968466	7.5652703	7.6509564	6.8232017	6.5985322	6.40185
AT2G19620	57.603454	0.9646088	0.2718914	3.5477731	3.89E-04	0.0038726	6.1925915	6.1083766	6.5628862	5.2634978	5.4039261	5.0956639
AT3G07315	347.56025	0.9668353	0.181194	5.3359125	9.51E-08	2.37E-06	8.6968233	8.7925503	9.0859693	7.7347315	7.6983337	8.070603
AT2G15290	224.86736	0.9688322	0.1843	5.2568206	1.47E-07	3.51E-06	8.0570212	8.1914581	8.4262144	7.3497909	7.0403691	7.3020649
AT3G01060	162.59967	0.9689339	0.19256	5.0318556	4.86E-07	1.04E-05	7.8425041	7.6693264	7.7261412	7.1531516	6.6705562	6.5324599
AT3G57645	157.74318	0.9691594	0.1875096	5.1685869	2.36E-07	5.42E-06	7.6645935	7.7695049	7.8010828	6.3946946	6.6705562	6.9346244
AT1G52510	417.77195	0.9694872	0.1736591	5.5827017	2.37E-08	6.50E-07	9.0102881	9.0403684	9.3223899	8.0382954	7.9213745	8.3439671
AT1G60600	160.77642	0.9699749	0.202939	4.7796378	1.76E-06	3.33E-05	7.4968466	7.7663796	7.9621477	6.8232017	6.7391548	6.6846883
AT4G39710	190.49754	0.9702939	0.2373644	4.0877817	4.36E-05	5.77E-04	7.7866588	7.8631766	8.3161496	7.11039	6.4698754	7.2268928
AT5G18060	456.12366	0.9704957	0.186329	5.2085048	1.90E-07	4.44E-06	9.3394947	9.0558105	9.3499881	8.3453619	7.861088	8.4610565
AT4G18440	134.97224	0.9716015	0.2232349	4.3523733	1.35E-05	0.0002049	7.3025794	7.6558783	7.5260779	6.5940902	6.7391548	6.0987114
AT1G15810	1080.779	0.9730948	0.143318	6.7897578	1.12E-11	5.22E-10	10.451446	10.67441	10.326708	9.5965406	9.3680164	9.5254957
AT1G18400	139.5277	0.9733887	0.1941424	5.0137876	5.34E-07	1.14E-05	7.4173535	7.5214513	7.6815021	6.7131893	6.496541	6.4592793
AT1G31190	110.02473	0.9740763	0.2240618	4.3473561	1.38E-05	2.09E-04	6.9762926	7.1940607	7.4123039	6.4642672	6.0331881	6.122608

AT5G22880	202.95327	0.9741887	0.178568	5.45556	4.88E-08	1.29E-06	7.9677648	8.189126	8.095727	7.1946821	6.8467003	7.1934372
AT4G34190	615.85331	0.9751205	0.1525192	6.3934283	1.62E-10	6.41E-09	9.5838884	9.5801611	9.8560286	8.8659674	8.5174315	8.6741315
AT4G14910	191.30103	0.9755011	0.1848595	5.2769868	1.31E-07	3.19E-06	7.9649719	8.165595	7.9147238	6.7692438	6.9075785	7.1934372
AT4G16400	535.28433	0.9767507	0.1476767	6.6141173	3.74E-11	1.62E-09	9.4594382	9.3332541	9.6250829	8.6290213	8.3593428	8.4703982
AT2G31750	427.57911	0.9774424	0.1354835	7.2144751	5.41E-13	3.16E-11	9.1630948	9.0698221	9.2349922	8.1690626	8.1401005	8.1714184
AT4G22830	84.446258	0.9797665	0.2404092	4.0754123	4.59E-05	6.05E-04	6.6193626	6.7884791	7.0810805	5.8874513	5.7961798	5.7455674
AT2G06950	497.80386	0.9815665	0.1778711	5.518414	3.42E-08	9.17E-07	9.3308433	9.3972872	9.4553863	7.8431732	8.6300992	8.4374342
AT3G26700	72.830472	0.9831801	0.2367157	4.1534213	3.28E-05	4.50E-04	6.6405204	6.547603	6.6504165	5.8874513	5.6133433	5.3597148
AT3G03830	63.679626	0.9864034	0.2506244	3.9357839	8.29E-05	0.0010186	6.5760926	6.3613083	6.4609226	5.0982599	5.2865918	5.6168815
AT1G72416	88.94486	0.986523	0.2551459	3.8665054	1.10E-04	1.31E-03	7.187495	6.6255604	6.9302609	5.669115	6.1719999	5.650145
AT1G65490	167.39728	0.9867717	0.2429349	4.0618776	4.87E-05	0.0006364	8.1921228	7.4453256	7.7151102	7.11039	6.5484377	6.6685459
AT4G02630	71.376265	0.9869249	0.2933544	3.3642746	7.67E-04	0.0069759	6.2850648	6.9180519	6.5463849	5.7824096	5.8384847	4.888274
AT3G02045	186.7652	0.9870705	0.2149361	4.5923907	4.38E-06	7.55E-05	7.8903923	7.9177199	8.0467059	7.4898305	6.7613149	6.5677036
AT5G07020	587.25289	0.9874694	0.1884876	5.2389082	1.62E-07	3.84E-06	9.3275855	9.5413771	9.9302443	8.786708	8.4576165	8.5430308
AT1G20340	15136.537	0.9880363	0.1377695	7.171662	7.41E-13	4.25E-11	14.167807	14.221601	14.502205	13.302994	13.146232	13.417034
AT4G04335	128.63589	0.9893305	0.2048449	4.8296562	1.37E-06	2.66E-05	7.2712702	7.4374874	7.5838165	6.5306385	6.496541	6.2364917
AT2G09465	723.03687	0.9909381	0.1747919	5.6692442	1.43E-08	4.05E-07	9.9808219	9.946066	9.8366504	9.0469319	8.4845075	9.0923252
AT5G02502	114.48741	0.9916777	0.2280274	4.34894	1.37E-05	2.07E-04	7.4009154	7.0879032	7.4168499	5.5461594	6.496541	6.3215387
AT3G05320	91.88521	0.9924535	0.2808902	3.5332433	4.10E-04	4.07E-03	6.8964599	6.7698728	7.2176151	5.9853613	6.3286119	5.1891546
AT5G45680	348.40942	0.9933945	0.1735576	5.723717	1.04E-08	3.05E-07	8.7577984	8.8184325	9.0439753	7.7626156	7.6397294	8.0644475
AT1G05057	1622.6357	0.9964409	0.197351	5.0490798	4.44E-07	9.63E-06	10.970045	11.009193	11.290982	9.9335101	9.6757188	10.423529
AT1G05063	1622.6357	0.9964409	0.197351	5.0490798	4.44E-07	9.63E-06	10.970045	11.009193	11.290982	9.9335101	9.6757188	10.423529
AT3G46900	54.022325	0.9969488	0.2733983	3.6465065	0.0002658	0.0027949	6.1341217	6.1083766	6.4342646	5.0982599	4.8634794	5.3597148
AT2G38170	2197.1804	0.998406	0.1323666	7.5427327	4.60E-14	3.19E-12	11.523196	11.395682	11.634242	10.682104	10.466387	10.360866
AT1G62520	43.008049	0.9987678	0.2866205	3.4846346	4.93E-04	4.74E-03	5.7226118	6.0375518	5.9229937	4.9116189	4.7791107	4.7722096
AT5G09730	12100.439	1.0008793	0.2179295	4.5926742	4.38E-06	7.55E-05	14.159587	13.641108	14.141585	12.549613	12.770529	13.354871
AT3G24430	443.44071	1.0009176	0.1918917	5.2160556	1.83E-07	4.27E-06	8.9352224	9.2213031	9.4718636	8.2500881	7.9115001	8.3439671
AT5G18020	271.1704	1.0014091	0.1700398	5.8892625	3.88E-09	1.22E-07	8.4515441	8.5006923	8.5881355	7.5550621	7.1948986	7.6532575
AT5G63180	389.44906	1.0026505	0.1642042	6.1061214	1.02E-09	3.65E-08	9.1980034	8.8659272	9.0292065	7.9919327	7.8404194	8.142491
AT4G20760	108.48239	1.0068399	0.2175506	4.6280719	3.69E-06	6.50E-05	7.0985535	7.1753313	7.342338	6.2445954	5.8384847	6.3215387
AT3G54050	2291.2599	1.0082117	0.1647132	6.1210123	9.30E-10	3.34E-08	11.42703	11.557433	11.76412	10.804517	10.271718	10.534571
AT1G73870	356.84021	1.0095798	0.2495295	4.0459343	5.21E-05	0.0006741	8.7301024	9.1172048	8.8708988	8.4519603	7.2586008	7.6116481
AT3G19170	1103.256	1.0100229	0.170187	5.9347818	2.94E-09	9.47E-08	10.190811	10.670654	10.695591	9.6347618	9.4521614	9.3942242

AT3G27690	8151.2551	1.0100992	0.1718147	5.8790017	4.13E-09	1.29E-07	13.309812	13.276528	13.653235	12.629392	12.091146	12.359101
AT1G48750	39.9131	1.0119062	0.29363	3.4461944	5.69E-04	5.36E-03	5.8612387	5.8136339	5.6570421	5.0982599	4.4916251	4.5784734
AT5G45650	85.86887	1.0129486	0.2523624	4.0138648	5.97E-05	0.0007597	6.5539611	6.8896087	7.1699376	5.669115	5.7961798	5.8637088
AT1G04223	48.816266	1.0146382	0.2736443	3.7078729	2.09E-04	2.29E-03	6.0731817	6.047885	6.1691841	4.6971932	5.2865918	4.8314086
AT3G61970	88.041923	1.0160448	0.2342849	4.3367918	1.45E-05	0.0002179	6.8119495	7.0524192	6.8040708	6.2445954	5.5124312	5.8058471
AT1G45545	45.530016	1.0165829	0.2943479	3.4536786	0.000553	0.0052483	5.7488277	5.9630696	6.2427211	4.9116189	4.5939523	5.0465438
AT2G36145	116.55718	1.0172514	0.2351631	4.3257273	1.52E-05	2.28E-04	7.0037665	7.3483332	7.5878536	5.9853613	6.2047119	6.40185
AT4G38850	70.587532	1.0174149	0.2517116	4.041987	5.30E-05	6.84E-04	6.4298254	6.686352	6.6581167	5.7824096	5.0904916	5.650145
AT4G25810	1073.8871	1.0181563	0.3078178	3.3076594	9.41E-04	8.28E-03	10.38248	10.53445	10.682908	9.5572792	9.9094817	8.0889136
AT1G52230	4969.763	1.0181862	0.1708356	5.9600334	2.52E-09	8.21E-08	12.588947	12.603135	12.918369	11.952214	11.415239	11.553993
AT1G29500	285.80952	1.0182456	0.2078747	4.8983625	9.66E-07	1.95E-05	8.8469625	8.4254161	8.5136958	7.4898305	7.1785237	7.8232977
AT3G43720	186.25637	1.0188328	0.2095307	4.862451	1.16E-06	2.30E-05	7.8636511	7.8425502	8.2641201	6.5306385	6.9273135	7.1241044
AT2G30695	118.4867	1.0206008	0.2217676	4.6021175	4.18E-06	7.26E-05	7.2981481	7.1611229	7.5797681	5.9853613	6.2047119	6.4592793
AT2G42380	204.04257	1.0221896	0.1732429	5.9003273	3.63E-09	1.15E-07	8.1704663	8.0495479	8.1676195	6.7131893	7.0761677	7.2268928
AT4G37925	284.77041	1.0227255	0.1689327	6.0540417	1.41E-09	4.90E-08	8.5573094	8.5668558	8.6180531	7.8168173	7.2110898	7.586094
AT1G16720	1134.216	1.022915	0.1760168	5.8114619	6.19E-09	1.88E-07	10.43287	10.549982	10.741163	9.8186668	9.1751674	9.5254957
AT1G52340	94.794766	1.0258846	0.2347434	4.3702382	1.24E-05	1.91E-04	6.780526	7.1940607	7.0462956	6.163252	5.6612702	5.9989583
AT1G19670	127.18905	1.0266551	0.219969	4.6672719	3.05E-06	5.47E-05	7.4173535	7.5288475	7.2986696	6.7692438	6.4150185	5.94637
AT2G21330	4063.041	1.0270702	0.142122	7.2266772	4.95E-13	2.91E-11	12.363757	12.327457	12.558112	11.551853	11.140911	11.389541
AT4G09650	3215.9853	1.0297164	0.1902934	5.4112035	6.26E-08	1.61E-06	11.838699	12.103675	12.293169	11.373474	10.768073	10.831372
AT5G07240	327.84373	1.030774	0.1810707	5.6926601	1.25E-08	3.59E-07	8.9676735	8.7184784	8.7427843	7.3125489	7.7211247	7.9819395
AT3G27960	114.00272	1.0311729	0.2421484	4.2584344	2.06E-05	2.98E-04	7.1187975	7.4914813	7.3327475	5.5461594	6.0691631	6.5850078
AT2G47750	42.01604	1.031277	0.3045381	3.3863649	7.08E-04	6.50E-03	5.824733	6.1758867	5.6415889	4.6971932	4.4916251	4.9429826
AT3G58120	665.22223	1.0315689	0.2101618	4.9084518	9.18E-07	1.87E-05	9.8139236	9.683186	9.9871378	8.0609297	8.9525032	8.9593144
AT4G06215	59.802109	1.033485	0.3020882	3.4211364	0.0006236	0.005827	6.4459391	6.4418737	6.4520912	3.219378	5.2865918	5.776022
AT3G04790	348.60852	1.0336661	0.1897186	5.4484171	5.08E-08	1.33E-06	8.6118901	8.8833422	9.1128343	8.0382954	7.5786433	7.8232977
AT3G53900	102.84667	1.0406167	0.2478228	4.1990358	2.68E-05	0.0003767	6.8846859	7.0524192	7.4790331	5.7824096	6.2679916	5.94637
AT2G04955	354.09701	1.04093	0.1547276	6.7274986	1.73E-11	7.81E-10	8.761022	8.9231778	9.0262345	7.9194695	7.7211247	7.8874681
AT4G28900	109.38523	1.0415582	0.2204907	4.7238186	2.31E-06	4.27E-05	7.215964	7.1706107	7.3707328	5.7824096	5.9584295	6.4403891
AT4G06805	1193.9805	1.042737	0.1761385	5.9199835	3.22E-09	1.03E-07	10.572895	10.506626	10.898148	9.2178134	9.7606735	9.680437
AT4G06810	1194.0592	1.0428835	0.176104	5.9219754	3.18E-09	1.02E-07	10.572895	10.507094	10.898148	9.2178134	9.7606735	9.680437
AT4G21280	4752.501	1.0430215	0.1378993	7.5636449	3.92E-14	2.73E-12	12.543455	12.560468	12.832295	11.68349	11.404719	11.651665
AT1G71140	68.76639	1.0437451	0.2572293	4.0576452	4.96E-05	6.46E-04	6.5314847	6.7125695	6.4957174	5.2634978	5.8384847	5.1431664

AT4G31000	145.51867	1.0440163	0.2544237	4.1034549	4.07E-05	0.0005439	7.2802853	7.886394	7.7479532	6.163252	6.9659915	6.2364917
AT1G31290	63.700864	1.0458479	0.2663193	3.9270445	8.60E-05	0.0010496	6.2579398	6.532965	6.5380629	5.669115	5.5124312	4.888274
AT5G42070	124.32779	1.0472568	0.2247331	4.6600033	3.16E-06	5.65E-05	7.465573	7.1706107	7.6118418	6.2445954	6.0691631	6.5324599
AT3G59010	43.274458	1.0482721	0.2921904	3.5876342	0.0003337	0.0034088	5.8370045	6.1664337	5.7891416	4.6971932	4.6894999	4.888274
AT1G09513	111.5953	1.0488276	0.2274714	4.6108116	4.01E-06	7.01E-05	7.182695	7.0879032	7.4348917	6.5940902	5.8384847	6.0744123
AT1G04030	149.2018	1.0495124	0.2153498	4.8735229	1.10E-06	2.19E-05	7.6297496	7.5866906	7.7941109	6.8232017	6.0691631	6.7627976
AT1G55490	1573.5002	1.0502893	0.1550477	6.7739744	1.25E-11	5.76E-10	11.039388	11.067172	11.069975	10.24653	9.6640039	9.9955575
AT4G10300	351.1753	1.0541103	0.1837458	5.736787	9.65E-09	2.84E-07	8.6934528	8.9468378	9.0306902	8.1051601	7.4885299	7.8305703
AT5G18080	239.01172	1.0550719	0.1905518	5.5369284	3.08E-08	8.31E-07	8.4575175	8.2530414	8.3450594	7.3497909	6.8672798	7.4790952
AT5G17670	216.86614	1.0558769	0.2050565	5.1492006	2.62E-07	5.94E-06	8.2321632	7.9971869	8.405778	7.11039	6.7831397	7.353458
AT1G15410	170.60646	1.0594383	0.2104919	5.0331552	4.82E-07	1.04E-05	7.6120069	8.0572407	7.9496529	6.5940902	6.5985322	6.9876347
AT5G14545	81.246787	1.0608444	0.2585062	4.1037484	4.07E-05	0.0005436	7.0985535	6.4651914	6.8247441	5.7824096	5.6612702	5.6168815
AT2G33180	85.166512	1.0644006	0.2331723	4.5648681	5.00E-06	8.46E-05	7.0146112	6.7190501	6.8981082	5.8874513	5.5124312	5.8637088
AT5G18030	377.74925	1.0649331	0.1583957	6.7232455	1.78E-11	7.99E-10	9.0738844	8.8630041	9.0859693	7.9681804	7.7211247	8.0395593
AT2G04030	854.1272	1.066168	0.1655162	6.4414735	1.18E-10	4.75E-09	9.9536079	10.328432	10.23958	9.3523467	8.9183958	8.9856133
AT1G29465	343.20086	1.0674415	0.1848506	5.7746177	7.71E-09	2.31E-07	8.7674478	8.7786576	9.046911	8.0382954	7.4065402	7.8378064
AT1G29920	59500.199	1.0704462	0.1572549	6.807077	9.96E-12	4.68E-10	16.260443	16.132372	16.510066	15.373529	14.944725	15.264181
AT1G04237	79.606491	1.0732598	0.2318143	4.6298256	3.66E-06	6.45E-05	6.761338	6.7319246	6.7971134	6.0770468	5.5124312	5.4755828
AT5G25140	98.11199	1.0735853	0.2204977	4.868919	1.12E-06	2.23E-05	7.2530694	7.0160404	6.9617125	6.0770468	5.8795843	5.94637
AT3G01505	124.73122	1.0750143	0.2604703	4.127205	3.67E-05	4.97E-04	7.1137631	7.4414118	7.6470923	6.875214	5.707656	6.1692457
AT4G13330	36.642318	1.0771021	0.3233336	3.3312412	8.65E-04	0.007716	5.954299	5.7643558	5.424005	4.1397727	4.9431857	4.1834245
AT4G06130	60.809848	1.0775738	0.2623195	4.1078685	3.99E-05	5.35E-04	6.5906606	6.4181729	6.1905799	5.4117401	5.1588632	5.233722
AT4G38840	347.30935	1.0778695	0.1882836	5.7247109	1.04E-08	3.03E-07	9.0995772	8.7724399	8.8390744	7.4560742	7.6634575	8.0644475
AT5G18660	219.87886	1.079517	0.1940749	5.5623719	2.66E-08	7.25E-07	7.9649719	8.3418016	8.3616597	7.3497909	6.9273135	7.1002328
AT1G52827	87.199768	1.0795577	0.2919185	3.6981476	2.17E-04	2.36E-03	6.7548849	7.0212939	7.1317342	4.6971932	5.4591985	6.3420395
AT3G08770	99.764861	1.0797318	0.2552669	4.2298154	2.34E-05	3.35E-04	6.7287798	7.3102113	7.2331638	6.2445954	5.5637694	6.0496968
AT2G31380	188.40532	1.0823565	0.1746129	6.1986054	5.70E-10	2.12E-08	8.1286507	7.9784022	7.9338808	6.9739302	6.8046392	6.9346244
AT4G00490	113.60282	1.0831234	0.2296887	4.7156144	2.41E-06	4.42E-05	7.4616157	7.2885889	7.0637929	6.5940902	5.8384847	6.0744123
AT4G15430	61.464487	1.083372	0.2632712	4.1150424	3.87E-05	0.0005208	6.5906606	6.4340166	6.2731246	5.0982599	5.5637694	5.0465438
AT3G56200	65.667749	1.0851539	0.264296	4.1058271	4.03E-05	0.0005397	6.4854533	6.4805297	6.6348919	5.0982599	5.7961798	4.9956923
AT1G05253	197.51006	1.0888593	0.1920364	5.6700674	1.43E-08	4.04E-07	7.931029	7.9971869	8.2965495	7.1531516	6.7831397	6.961373
AT5G14060	1127.6718	1.0927369	0.1546223	7.0671373	1.58E-12	8.58E-11	10.516312	10.626953	10.622202	9.7918552	9.2901352	9.3160682
AT1G04180	53.9922	1.0939012	0.2840367	3.8512677	1.18E-04	1.38E-03	6.3378311	6.1852782	6.3320744	4.6971932	4.5939523	5.5122259

AT3G27540	78.523104	1.0965772	0.260247	4.2136014	2.51E-05	3.56E-04	6.6193626	6.6797225	7.0404157	5.4117401	5.9584295	5.3189277
AT1G04250	126.08481	1.0969009	0.2308041	4.7525195	2.01E-06	3.76E-05	7.187495	7.5866906	7.5918794	5.9853613	6.6229419	6.122608
AT2G28950	672.41423	1.098091	0.1408951	7.7936793	6.51E-15	4.96E-13	9.8185723	9.7392558	9.9955196	8.5984029	8.6948564	8.8454601
AT1G44000	288.39834	1.0996751	0.1700943	6.4650906	1.01E-10	4.11E-09	8.568392	8.5864817	8.6949269	7.8690562	7.3781411	7.3020649
AT4G00163	36.546295	1.1013863	0.3142049	3.5053125	0.0004561	0.0044359	5.4781493	5.7390709	5.9483358	4.1397727	4.7791107	4.3546248
AT1G26761	68.200093	1.1020548	0.2849182	3.8679688	0.0001097	0.001302	6.4379048	6.8249868	6.3512009	6.0770468	5.0904916	4.9956923
AT1G06263	106.45044	1.1029515	0.2575321	4.2827736	1.85E-05	0.0002704	7.0519383	7.1611229	7.3800749	6.5306385	5.2865918	6.1461153
AT5G63310	459.41223	1.1056789	0.1793479	6.1649954	7.05E-10	2.59E-08	9.1335522	9.2235839	9.5223066	8.4172953	7.8915461	8.1714184
AT1G48745	46.166416	1.1064578	0.2896832	3.8195437	0.0001337	0.0015537	5.9877051	5.8495183	6.211663	5.0982599	4.9431857	4.5076474
AT1G67860	233.27586	1.1068404	0.2184007	5.0679336	4.02E-07	8.78E-06	7.8933332	8.4372558	8.6041688	7.1946821	7.1452054	7.1706945
AT4G00165	39.721976	1.1075595	0.3059974	3.619506	0.0002952	0.0030639	5.5838715	5.8256948	6.0802791	4.4452387	4.7791107	4.5076474
AT2G18328	78.114469	1.1099003	0.2304982	4.8152237	1.47E-06	2.85E-05	6.8304803	6.7060597	6.754649	5.7824096	5.6133433	5.4755828
AT1G69530	1644	1.1104128	0.1616521	6.869154	6.46E-12	3.13E-10	10.82972	11.27909	11.279484	10.149545	9.9020208	9.9461945
AT4G13575	1023.7675	1.1110479	0.1809032	6.1416702	8.17E-10	2.98E-08	10.654463	10.462409	10.237654	9.6347618	9.0457315	9.2168792
AT3G01500	5774.2443	1.1122643	0.1820521	6.1095948	9.99E-10	3.57E-08	12.644658	12.948065	13.233712	11.8951	11.521305	11.953033
AT1G18060	253.63758	1.1123611	0.1641739	6.7755065	1.24E-11	5.73E-10	8.3438714	8.4114791	8.5759926	7.4560742	7.2586008	7.2487732
AT4G32770	85.164509	1.1140292	0.2705748	4.1172691	3.83E-05	0.0005168	6.5390158	6.9733094	7.0695785	6.3215962	5.3464515	5.4379847
AT1G32550	210.38674	1.1144881	0.2102735	5.3001837	1.16E-07	2.84E-06	7.8874454	8.15129	8.4944698	6.9254163	6.9849484	7.112218
AT4G39510	116.67996	1.1183249	0.2166625	5.1615996	2.45E-07	5.61E-06	7.1337963	7.4724289	7.3939756	6.3215962	6.2679916	5.9729037
AT4G34760	516.23849	1.1191754	0.1637527	6.8345455	8.23E-12	3.91E-10	9.5553933	9.3269038	9.5628648	8.1690626	8.1652491	8.5606317
AT1G29418	291.46477	1.1196803	0.2014274	5.558728	2.72E-08	7.39E-07	8.6207894	8.5740234	8.8186069	7.1946821	7.2271013	7.8592997
AT1G76100	1673.8517	1.1206854	0.1346938	8.3202446	8.78E-17	7.85E-15	11.071226	11.070027	11.356634	10.011112	9.9413736	10.116515
AT1G02350	81.095615	1.1210667	0.3066056	3.6563808	2.56E-04	2.70E-03	6.8548234	6.8007513	6.9741037	5.2634978	4.6894999	6.2364917
AT5G04190	404.12029	1.1220969	0.1880945	5.9656007	2.44E-09	7.96E-08	9.0621717	9.0364818	9.3187509	7.4898305	7.9697576	8.2385654
AT1G72470	88.764719	1.1228889	0.2366065	4.7458083	2.08E-06	3.86E-05	6.7155479	6.9894822	7.1427531	5.8874513	5.707656	5.7455674
AT3G48730	687.71876	1.1240558	0.1761739	6.3803758	1.77E-10	6.95E-09	9.6046722	9.925899	10.102388	8.9410983	8.5368311	8.7100637
AT2G31141	33939.428	1.1254603	0.1983559	5.6739446	1.40E-08	3.97E-07	15.652932	15.159262	15.702463	14.013795	14.277488	14.65531
AT1G68870	43.72646	1.1266756	0.2929551	3.8458981	0.0001201	0.0014061	6.1538761	5.8612842	5.9608419	4.1397727	5.0904916	4.6459844
AT2G01590	99.526039	1.1277194	0.2366372	4.7656059	1.88E-06	3.56E-05	7.187495	6.8309826	7.3279282	5.9853613	5.7961798	5.9989583
AT2G09690	158.61493	1.128857	0.2180113	5.1779755	2.24E-07	5.18E-06	7.9367419	7.8660993	7.6432179	5.8874513	6.6469455	6.9073704
AT5G02905	202.91151	1.129971	0.1950496	5.7932482	6.90E-09	2.09E-07	8.2711223	8.2575003	7.8523251	7.0663221	6.9467821	6.9073704
AT1G79520	1938.2478	1.1309075	0.2009113	5.6288889	1.81E-08	5.08E-07	11.24619	11.505193	11.418012	10.660035	9.9119601	9.9394833
AT3G60320	865.57177	1.1327318	0.1302354	8.697575	3.39E-18	3.39E-16	10.27609	10.142666	10.268163	8.8659674	9.085796	9.1945059

AT1G64150	111.60058	1.1328415	0.2214532	5.11549	3.13E-07	7.00E-06	7.1137631	7.203335	7.5091477	6.2445954	6.0331881	6.0496968
AT2G42065	35.059782	1.1352471	0.3426198	3.3134315	9.22E-04	8.14E-03	5.5544473	5.5190527	5.8441814	4.9116189	4.6894999	3.3443906
AT3G28160	269.88532	1.1381524	0.2328266	4.8884122	1.02E-06	2.04E-05	8.1921228	8.6180319	8.789105	7.6772924	7.4617144	6.8654991
AT2G07015	82.1975	1.1391862	0.2322479	4.9050432	9.34E-07	1.90E-05	6.7088862	6.9787205	6.8915908	5.5461594	5.7525968	5.6168815
AT4G34950	89.066795	1.1410019	0.2805383	4.0671884	4.76E-05	0.0006242	6.4932279	7.0107677	7.2887833	6.163252	5.707656	5.3597148
AT5G25240	51.377443	1.1438225	0.2849795	4.0137012	5.98E-05	7.60E-04	6.06277	6.2582871	6.2731246	4.9116189	5.3464515	4.5076474
AT3G23810	418.82914	1.1447674	0.1458032	7.8514551	4.11E-15	3.17E-13	9.1109914	9.3087581	9.1198217	8.0609297	7.9115001	8.0644475
AT1G15820	17618.34	1.144887	0.1664086	6.879976	5.99E-12	2.93E-10	14.371878	14.450721	14.864746	13.526806	13.182866	13.449281
AT1G55480	501.82967	1.1465672	0.1635668	7.0097776	2.39E-12	1.27E-10	9.2309252	9.4986994	9.5730833	8.4689853	8.0708121	8.2656465
AT3G08920	351.89342	1.1511882	0.1742293	6.6073164	3.91E-11	1.69E-09	8.8224853	8.8600752	9.1323143	7.6476919	7.5786433	7.9489053
AT2G43375	919.85868	1.1515754	0.1651857	6.9713984	3.14E-12	1.62E-10	10.292891	10.408298	10.25426	9.3151715	8.7623357	9.2740097
AT4G04595	36016.751	1.1515996	0.2417111	4.7643635	1.89E-06	3.58E-05	15.746785	15.304549	15.788321	14.066129	13.946056	14.925697
AT2G42770	110.23804	1.1563063	0.2152469	5.372	7.79E-08	1.97E-06	7.1238145	7.2929394	7.4304023	5.9853613	5.9584295	6.1920111
AT5G55220	552.22749	1.1601875	0.1562882	7.4233872	1.14E-13	7.39E-12	9.3893002	9.666518	9.6809538	8.5351448	8.2062131	8.4469296
AT2G04145	30.673368	1.1608097	0.325438	3.5669153	3.61E-04	3.64E-03	5.5244106	5.5484611	5.5116165	3.7517613	3.8307199	4.5784734
AT3G63140	1238.2751	1.1670345	0.1774007	6.5785233	4.75E-11	2.00E-09	10.507702	10.664371	11.046332	9.6271982	9.3240289	9.66221
AT5G17230	1163.0028	1.1679851	0.1179822	9.8996742	4.18E-23	6.19E-21	10.656628	10.621342	10.691845	9.5887733	9.3277462	9.5074583
AT4G02970	6810.1277	1.1684234	0.1693308	6.9002422	5.19E-12	2.58E-10	13.334365	12.989111	13.302038	11.74768	11.92563	12.28162
AT1G02205	298.77056	1.1693104	0.257006	4.5497389	5.37E-06	9.05E-05	8.1777213	8.9245804	8.9341026	7.894483	6.9467821	7.432059
AT1G12570	29.16376	1.1696702	0.3347268	3.494403	0.0004751	0.0045829	5.2766692	5.6194617	5.4058257	4.1397727	3.6524875	4.3546248
AT4G37080	305.77241	1.1697056	0.1616707	7.2351126	4.65E-13	2.77E-11	8.6419265	8.8154115	8.7995865	7.1946821	7.6156045	7.6614373
AT5G66580	626.28223	1.175759	0.195929	6.0009433	1.96E-09	6.54E-08	9.9966338	9.6161948	9.7246932	8.2500881	8.3593428	8.9017849
AT4G34260	610.0484	1.1806152	0.178075	6.6298768	3.36E-11	1.46E-09	9.565569	9.7684481	9.8948362	8.2302543	8.3952139	8.8054205
AT4G38960	35.394473	1.1827799	0.3222588	3.6702802	0.0002423	0.0025805	5.954299	5.673849	5.5116165	4.1397727	3.9893347	4.710477
AT3G07735	30.700835	1.1830143	0.3311297	3.5726614	3.53E-04	3.58E-03	5.3470024	5.6604432	5.3874144	4.6971932	4.1322253	3.7645543
AT1G04107	128.57307	1.183579	0.2387179	4.9580656	7.12E-07	1.48E-05	7.7219626	7.1847264	7.6276151	5.9853613	6.0691631	6.5324599
AT3G29370	263.93615	1.1845554	0.2491709	4.7539869	1.99E-06	3.73E-05	8.3945667	8.5740234	8.7172124	6.6548684	6.9659915	7.8159882
AT1G50040	147.4213	1.1866514	0.2498689	4.7490963	2.04E-06	3.81E-05	7.7866588	7.4800801	7.882219	5.9853613	6.9467821	6.0987114
AT5G23750	149.0248	1.1869874	0.2292859	5.1768888	2.26E-07	5.21E-06	7.4968466	7.7121867	7.9559138	6.3946946	6.1042628	6.7627976
AT1G04040	636.14988	1.1873212	0.1422165	8.3486901	6.90E-17	6.25E-15	9.8131474	9.6538895	9.9174689	8.6137933	8.4576165	8.6700833
AT3G23805	103.69306	1.1896732	0.2462363	4.8314284	1.36E-06	2.64E-05	7.3461601	7.0054757	7.2986696	5.7824096	5.5124312	6.3007423
AT2G21140	140.53411	1.1919007	0.2006693	5.9396271	2.86E-09	9.21E-08	7.6817048	7.5544397	7.6852752	6.3946946	6.1042628	6.6021069
AT3G04985	27.839708	1.1979187	0.3399573	3.5237331	4.26E-04	4.19E-03	5.1642901	5.6055384	5.4419581	3.219378	4.1322253	4.1834245

AT5G23940	202.11556	1.1988863	0.2037734	5.8834295	4.02E-09	1.26E-07	7.9899152	8.2328043	8.2641201	6.875214	6.5484377	7.1821107
AT3G62070	204.47132	1.2002492	0.2156779	5.5650073	2.62E-08	7.15E-07	8.3736597	8.153684	7.9714482	7.1531516	6.4427077	7.0881471
AT1G26945	254.94139	1.2030271	0.2330208	5.1627456	2.43E-07	5.59E-06	8.1211441	8.5560371	8.7926073	6.8232017	7.0937393	7.5513021
AT5G25130	87.368335	1.2036158	0.2537829	4.7426978	2.11E-06	3.90E-05	6.7418914	7.0317438	7.0106512	6.3215962	5.4039261	5.3597148
AT1G72430	603.07088	1.2052774	0.1842102	6.5429448	6.03E-11	2.50E-09	9.8232061	9.4488842	9.907005	8.1690626	8.7120244	8.4610565
AT1G74730	376.09042	1.2071229	0.163284	7.3927793	1.44E-13	9.18E-12	8.9760203	8.9231778	9.210327	8.0382954	7.6751769	7.7407813
AT5G58670	212.81938	1.2074821	0.2581413	4.6776014	2.90E-06	5.22E-05	8.0464918	8.2328043	8.4441402	6.9254163	7.4205328	6.258228
AT5G08495	175.33871	1.2099704	0.3384459	3.5750777	3.50E-04	3.55E-03	8.0983868	8.2773962	7.5918794	6.3215962	5.3464515	7.3228424
AT2G30766	62.769801	1.2116362	0.2944575	4.1148084	3.88E-05	0.000521	6.722179	6.6663714	6.1905799	4.1397727	5.6612702	5.0956639
AT2G35290	99.778836	1.2118761	0.3322061	3.6479649	2.64E-04	0.0027817	6.8366047	6.6663714	7.7941109	5.9853613	6.1385289	5.0465438
AT5G01375	54.915907	1.2118985	0.3049403	3.9742154	7.06E-05	0.0008865	6.5978898	6.2130913	6.2831183	3.7517613	4.7791107	5.5122259
AT1G29510	210.86516	1.2121873	0.1888547	6.4186245	1.38E-10	5.47E-09	8.3395652	8.170332	8.2022654	6.6548684	6.8046392	7.2487732
AT3G07215	225.74396	1.213593	0.2027683	5.9851209	2.16E-09	7.15E-08	8.1945091	8.2552726	8.5242672	6.9739302	6.7391548	7.33312
AT5G36700	619.91861	1.2140576	0.1624445	7.4736762	7.80E-14	5.20E-12	9.5674115	9.7739051	9.9476277	8.6137933	8.3150859	8.6205967
AT1G07803	29.116744	1.2150666	0.3344944	3.6325466	0.0002806	0.0029283	5.2945766	5.4111291	5.6259685	4.1397727	3.6524875	4.2715627
AT5G15350	443.12894	1.2153949	0.2239988	5.4259001	5.77E-08	1.49E-06	9.1310628	9.0849539	9.6426549	7.5228149	8.3593428	7.9622099
AT3G02870	112.23839	1.2165786	0.2252105	5.4019627	6.59E-08	1.69E-06	7.0571928	7.4137138	7.4393671	6.3215962	5.9195455	5.94637
AT5G36790	620.89967	1.217161	0.1624984	7.4902947	6.87E-14	4.61E-12	9.5692516	9.7793416	9.949982	8.6137933	8.3150859	8.6205967
AT2G20800	227.98334	1.2204004	0.3192242	3.82302	1.32E-04	1.53E-03	7.9733343	8.3013467	8.7409727	7.2743201	7.4065402	5.776022
AT4G06705	103.29083	1.2267437	0.2794633	4.3896413	1.14E-05	1.77E-04	7.0728423	7.0001642	7.5344691	6.163252	5.0904916	6.1692457
AT1G09950	69.427418	1.2277632	0.3507881	3.5000141	4.65E-04	4.50E-03	6.4217005	6.1182165	7.2434372	5.669115	5.5124312	4.2715627
AT4G35320	91.682062	1.2313657	0.2591613	4.7513489	2.02E-06	3.77E-05	6.9254813	6.7636171	7.3939756	5.669115	5.8795843	5.5828329
AT1G06273	37.178372	1.2345632	0.3334693	3.7021792	0.0002138	0.0023306	5.5395071	5.8612842	5.816924	5.0982599	4.4916251	3.4984464
AT2G39470	635.74053	1.2351539	0.1823157	6.7748064	1.25E-11	5.74E-10	9.6278118	9.8054597	9.9825453	8.8400284	8.1817745	8.5474512
AT1G62780	187.62697	1.2379406	0.1964345	6.3020518	2.94E-10	1.13E-08	7.8302795	8.1075036	8.1943437	7.0663221	6.6229419	6.6846883
AT4G27600	601.76975	1.2392374	0.1565084	7.9180246	2.41E-15	1.91E-13	9.7333348	9.679037	9.7927296	8.5512213	8.1569148	8.6372815
AT4G08950	1154.9491	1.2427985	0.2603595	4.7733948	1.81E-06	3.43E-05	10.682785	10.412802	10.962528	8.8136145	9.9413736	8.9659342
AT3G51238	22.931062	1.2454607	0.3823443	3.2574324	1.12E-03	0.0096173	4.8378037	5.1095399	5.3498714	4.4452387	2.106732	3.7645543
AT4G02130	243.94469	1.2532587	0.180418	6.9464177	3.75E-12	1.91E-10	8.4889643	8.2485688	8.5616954	7.2350505	7.2586008	6.961373
AT5G64940	1753.8552	1.2563627	0.1384126	9.0769401	1.12E-19	1.28E-17	11.331061	11.35413	11.138321	10.216601	9.8819344	9.8881287
AT1G08643	69.919174	1.2573123	0.2745568	4.579425	4.66E-06	7.96E-05	6.5390158	6.757334	6.6112871	5.8874513	5.4039261	4.6459844
AT3G10060	252.76817	1.2608373	0.2106401	5.9857424	2.15E-09	7.13E-08	8.2111038	8.5542261	8.6665771	7.5866041	6.8258231	7.112218
AT3G29250	111.80531	1.2612406	0.3599544	3.5038901	4.59E-04	4.45E-03	7.1238145	6.8605934	7.9590341	5.669115	6.5737024	4.710477

AT3G24480	155.28453	1.263272	0.2091358	6.0404388	1.54E-09	5.28E-08	7.5904235	7.7569629	8.0289989	6.4642672	6.5985322	6.3622531
AT5G04140	5589.1997	1.266018	0.2001748	6.3245617	2.54E-10	9.82E-09	12.450798	13.208351	13.126524	11.802435	11.620105	11.45923
AT2G33330	85.043359	1.266624	0.2256497	5.6132305	1.99E-08	5.49E-07	6.9081386	6.912408	7.0106512	5.669115	5.5124312	5.650145
AT2G21320	45.625909	1.2667971	0.2839696	4.4610312	8.16E-06	1.31E-04	5.9766555	6.1473397	6.0687706	4.9116189	4.4916251	4.710477
AT5G49215	72.932874	1.2688689	0.2690533	4.71605	2.40E-06	4.41E-05	6.5086526	6.6255604	7.0345117	5.2634978	5.6133433	5.1431664
AT1G65060	17.228166	1.2701863	0.3856161	3.2939145	9.88E-04	8.63E-03	4.977381	4.6485132	4.6548905	3.219378	3.2122368	3.1718957
AT5G00660	83.848143	1.2704059	0.274949	4.6205138	3.83E-06	6.71E-05	6.7287798	7.3187703	6.6809735	5.669115	5.2241406	5.7144559
AT5G01515	118.13358	1.2704929	0.2345808	5.4160136	6.09E-08	1.57E-06	7.4009154	7.389542	7.3847234	6.6548684	5.4591985	6.0496968
AT2G40205	227.57215	1.2730177	0.2042065	6.2339725	4.55E-10	1.72E-08	8.2643232	8.1844504	8.6219957	6.5940902	7.0761677	7.2046755
AT4G27654	44.714233	1.2742606	0.338319	3.7664469	0.0001656	0.0018644	5.7617592	5.9521083	6.3029005	5.4117401	4.5939523	3.6376272
AT1G32220	212.75511	1.2747673	0.2123012	6.0045232	1.92E-09	6.43E-08	7.9424322	8.2259951	8.530573	7.11039	6.9273135	6.7475104
AT2G41330	31.455259	1.2752022	0.348053	3.663816	2.48E-04	2.64E-03	5.6269122	5.1863811	5.7319184	4.9116189	3.4490907	3.8812119
AT4G31890	72.078757	1.2822584	0.2459258	5.2140064	1.85E-07	4.32E-06	6.6335022	6.7125695	6.7901222	5.5461594	5.0187179	5.4755828
AT4G28780	159.025	1.2860095	0.2033851	6.3230281	2.56E-10	9.90E-09	7.7449377	7.8455149	7.9929191	5.8874513	6.7391548	6.5677036
AT1G62180	253.34031	1.2867783	0.1827398	7.0415888	1.90E-12	1.02E-10	8.3715523	8.6741408	8.4239579	7.4560742	7.1452054	6.9745636
AT3G10520	294.42139	1.2871628	0.1763841	7.2974975	2.93E-13	1.79E-11	8.5197403	8.7630627	8.8922759	7.1531516	7.4617144	7.4698099
AT5G58260	155.72912	1.2904441	0.2235249	5.773156	7.78E-09	2.33E-07	7.636786	7.6925631	8.1070232	6.3215962	6.2679916	6.6685459
AT5G21430	256.46961	1.291946	0.1946377	6.6376959	3.19E-11	1.39E-09	8.3862403	8.4664368	8.6722918	7.6174713	6.8875699	7.0636679
AT1G09883	24.105014	1.2965445	0.3537846	3.6647848	2.48E-04	2.63E-03	5.1642901	5.4270499	5.1890938	2.3665114	3.8307199	3.9891381
AT2G08765	55.004132	1.2978179	0.264819	4.9007737	9.55E-07	1.93E-05	6.3028689	6.4261165	6.3029005	4.9116189	5.0187179	4.888274
AT1G29660	1157.986	1.2994695	0.1188126	10.937136	7.66E-28	1.67E-25	10.705552	10.616144	10.763197	9.2378177	9.4555632	9.3893077
AT4G00955	261.92299	1.3016605	0.1813682	7.1768953	7.13E-13	4.10E-11	8.4870189	8.6005893	8.6318052	6.9254163	7.0403691	7.4974886
AT5G09065	404.05684	1.301848	0.2125561	6.1247279	9.08E-10	3.28E-08	9.0411114	9.3109047	9.2116357	7.9194695	7.2586008	8.1307557
AT1G23030	339.00316	1.302582	0.1638698	7.9488846	1.88E-15	1.51E-13	8.9787919	8.804788	9.0321724	7.2350505	7.7097742	7.677659
AT1G79075	149.74302	1.3127247	0.209378	6.2696399	3.62E-10	1.38E-08	7.6507567	7.7911945	7.8887788	6.163252	6.1385289	6.6846883
AT4G28660	235.03776	1.3153428	0.1894425	6.9432283	3.83E-12	1.94E-10	8.2298384	8.3459948	8.6259275	7.11039	6.8672798	7.1358938
AT5G07200	18.384421	1.3185628	0.3807179	3.4633593	5.33E-04	5.09E-03	4.9092797	4.9200424	4.9590991	0	3.6524875	3.4984464
AT1G08477	137.57652	1.3215624	0.2055803	6.4284486	1.29E-10	5.14E-09	7.5007086	7.7881159	7.5918794	6.5306385	6.2366987	6.0987114
AT1G53490	27.486263	1.3223651	0.3929893	3.3648883	7.66E-04	6.96E-03	5.7745758	4.8973644	5.6415889	0	4.5939523	3.4984464
AT5G65610	172.48143	1.3233277	0.2011341	6.5793319	4.73E-11	1.99E-09	7.9079484	7.886394	8.1540694	6.0770468	6.8258231	6.6190056
AT4G28220	463.16908	1.3236482	0.1908761	6.9345926	4.07E-12	2.06E-10	9.2665189	9.559546	9.3452262	7.8690562	7.6983337	8.3337668
AT2G40670	16.110307	1.3268816	0.393235	3.3742711	7.40E-04	6.76E-03	4.5404893	4.7532581	4.8822876	2.3665114	2.9287097	3.3443906
AT5G00875	160.66124	1.3288371	0.2269777	5.854484	4.78E-09	1.47E-07	7.8485778	7.8835122	7.8985629	6.5940902	5.8384847	6.7928944

AT1G07180	260.90367	1.3297397	0.1737851	7.6516316	1.98E-14	1.43E-12	8.3630915	8.6438068	8.6589221	7.1946821	7.2586008	7.112218
AT1G05137	1126.2779	1.331038	0.2328013	5.7174852	1.08E-08	3.15E-07	10.870585	10.505221	10.658164	9.0355488	8.751306	9.7587706
AT1G15405	220000.47	1.3384108	0.2716948	4.926156	8.39E-07	1.72E-05	18.393527	17.9348	18.526958	16.198919	16.488451	17.459303
AT1G05163	220004.55	1.3384159	0.271692	4.9262252	8.38E-07	1.72E-05	18.393551	17.934835	18.526982	16.198919	16.488529	17.459313
AT4G06120	74.676946	1.3433231	0.2637605	5.0929649	3.53E-07	7.80E-06	6.9482853	6.8249868	6.6191983	5.2634978	4.8634794	5.6826588
AT5G09495	20.256999	1.3433958	0.3762623	3.5703704	3.56E-04	3.60E-03	4.7366366	5.1863811	5.1676757	2.3665114	3.4490907	3.6376272
AT2G02680	30.852976	1.3437774	0.393017	3.4191327	6.28E-04	5.86E-03	5.3975898	5.3950307	5.9229937	4.1397727	4.5939523	2.148558
AT2G25770	70.157361	1.343845	0.3257413	4.1254974	3.70E-05	0.0005005	6.0835188	6.6324429	7.1913224	5.7824096	4.8634794	4.888274
AT5G35490	26.414741	1.3443718	0.3635831	3.6975646	0.0002177	0.0023687	4.9550358	5.6194617	5.4772077	3.7517613	3.2122368	4.0895494
AT1G06233	45.671078	1.3463579	0.2937199	4.5838164	4.57E-06	7.82E-05	5.9986708	6.3277787	6.0336839	4.1397727	4.5939523	4.8314086
AT5G44680	971.38963	1.3508994	0.1413636	9.5562063	1.22E-21	1.61E-19	10.296228	10.462892	10.584816	9.2477168	9.0366751	8.9559931
AT5G02760	2826.5165	1.3527477	0.1721968	7.8558243	3.97E-15	3.08E-13	11.865902	11.918113	12.206062	10.378529	10.454535	10.894325
AT4G37930	5598.2408	1.355265	0.1555488	8.7127961	2.96E-18	2.98E-16	12.710021	13.018142	13.190477	11.687132	11.420471	11.64961
AT1G72645	3817.6149	1.3575402	0.2269782	5.9809277	2.22E-09	7.31E-08	12.366405	12.625299	12.342836	10.817868	10.483151	11.492852
AT5G44130	169.72675	1.3585217	0.2653423	5.119883	3.06E-07	6.85E-06	7.9816486	7.5760202	8.2965495	6.163252	6.9659915	6.122608
AT4G23290	154.72042	1.3605644	0.2062946	6.5952499	4.25E-11	1.81E-09	7.6262184	7.8034437	8.0200631	6.3946946	6.5227227	6.2796418
AT1G73340	19.631091	1.3610885	0.393435	3.4595004	5.41E-04	5.15E-03	4.8131692	4.701836	5.4058257	2.3665114	3.8307199	2.9759397
AT1G10470	244.74076	1.3619587	0.1958394	6.9544676	3.54E-12	1.81E-10	8.3330818	8.4214478	8.6473629	7.2743201	7.2110898	6.7164413
AT4G00883	31.465266	1.3620498	0.3456926	3.9400611	8.15E-05	1.01E-03	5.4140656	5.7643558	5.528518	4.4452387	4.3814822	3.1718957
AT5G50740	62.040918	1.3646906	0.3224257	4.2325746	2.31E-05	0.000331	5.920101	6.9348525	6.6033322	5.4117401	4.4916251	5.0465438
AT2G23130	128.18011	1.3668037	0.25244	5.4143704	6.15E-08	1.58E-06	7.4616157	7.2533046	7.911506	6.0770468	6.3579929	5.8058471
AT3G56825	2926.1936	1.3688478	0.2585137	5.2950687	1.19E-07	2.91E-06	12.04203	12.081213	12.115105	10.332761	9.8794038	11.195477
AT5G14565	18.639594	1.3716945	0.3775125	3.6335075	2.80E-04	2.92E-03	4.8620246	4.8973644	5.032027	2.3665114	3.4490907	3.3443906
AT4G13340	336.36443	1.3723164	0.2209024	6.2123214	5.22E-10	1.96E-08	8.7738451	8.8289564	9.2181617	7.2350505	7.9015576	7.1934372
AT4G25050	1385.3709	1.3739216	0.126737	10.840726	2.21E-27	4.64E-25	10.999685	10.831817	11.078966	9.5887733	9.4791534	9.6458126
AT2G33530	80.996777	1.3747829	0.2339861	5.8754886	4.22E-09	1.31E-07	6.8668427	6.8722697	6.9302609	5.7824096	5.4039261	5.2769537
AT2G20721	32.039287	1.3761093	0.3313741	4.1527361	3.29E-05	0.0004505	5.3809236	5.6604432	5.8441814	3.7517613	4.2622301	3.9891381
AT1G08103	34.677095	1.3761604	0.3150787	4.3676718	1.26E-05	0.0001927	5.824733	5.8014713	5.6723315	3.219378	4.3814822	4.3546248
AT3G50060	189.16158	1.3795595	0.2528974	5.4550175	4.90E-08	1.29E-06	8.0090216	7.8155898	8.4596446	6.9739302	6.8046392	6.0987114
AT1G72760	18.025959	1.3799328	0.4205456	3.2812915	0.0010333	0.0089435	4.203971	5.24144	5.032027	3.219378	3.6524875	1.7177495
AT3G47347	108.3221	1.3806768	0.2667108	5.1766812	2.26E-07	5.21E-06	7.3674665	7.4097132	7.1913224	5.9853613	6.2679916	5.0956639
AT3G56290	83.056083	1.3809031	0.248988	5.5460629	2.92E-08	7.92E-07	6.9707344	6.7823036	6.9924932	6.0770468	5.0904916	5.3189277
AT5G09585	2253.182	1.3829293	0.139395	9.9209371	3.38E-23	5.07E-21	11.764147	11.670838	11.598422	10.295071	10.028465	10.435503

AT2G20724	118.76836	1.3848808	0.2288676	6.051013	1.44E-09	4.98E-08	7.4576475	7.1894011	7.6890385	5.9853613	6.0331881	5.94637
AT3G04975	147.42341	1.3856378	0.3486622	3.9741552	7.06E-05	8.87E-04	7.7053248	8.0051632	7.7800652	5.2634978	5.0904916	7.0512707
AT3G19680	708.28841	1.3877891	0.2546852	5.4490365	5.06E-08	1.33E-06	9.9237147	9.7029367	10.406018	7.9440305	9.0275616	8.3490403
AT5G16023	124.97609	1.3884639	0.2285595	6.0748471	1.24E-09	4.36E-08	7.4009154	7.3813939	7.7976011	5.9853613	5.8795843	6.2364917
AT1G52830	40.926394	1.3903688	0.3395077	4.0952496	4.22E-05	0.0005616	5.4303553	6.068332	6.2930433	4.6971932	3.8307199	4.5076474
AT5G24030	152.27588	1.3948048	0.2248063	6.2044731	5.49E-10	2.05E-08	7.7219626	7.6043011	8.0437698	6.6548684	6.4150185	5.9989583
AT1G22830	96.633206	1.3965189	0.2833213	4.9290989	8.26E-07	1.70E-05	6.7868657	7.0929018	7.5176377	6.0770468	5.7525968	5.0956639
AT3G15540	208.2531	1.3967532	0.2485143	5.6204135	1.91E-08	5.30E-07	7.7153305	8.5811556	8.4126223	6.5306385	6.7391548	6.9346244
AT2G39730	26689.214	1.3970822	0.1431954	9.7564725	1.73E-22	2.45E-20	15.05106	15.260246	15.41404	13.989665	13.663991	13.777224
AT5G39860	162.5831	1.3993423	0.2123405	6.5900863	4.40E-11	1.87E-09	7.9021201	7.8095295	7.9898713	6.7131893	5.9195455	6.6190056
AT2G23110	20.500473	1.4026625	0.4281584	3.276036	1.05E-03	9.09E-03	4.6558155	5.2593357	5.1676757	4.1397727	3.4490907	0
AT5G02915	174.00814	1.4037933	0.200719	6.9938224	2.67E-12	1.40E-10	8.1186331	8.1075036	7.7479532	6.6548684	6.496541	6.4963335
AT5G27660	214.08354	1.4048445	0.2116388	6.6379336	3.18E-11	1.39E-09	8.0278783	8.2861516	8.5410219	7.0663221	6.5227227	6.8935479
AT4G07080	188.12961	1.4050264	0.3395965	4.1373405	3.51E-05	4.78E-04	7.8241282	8.2007491	8.4103445	6.9739302	4.8634794	6.8935479
AT5G61455	2049.7393	1.4106706	0.2913173	4.8423849	1.28E-06	2.51E-05	11.498059	11.597553	11.672049	9.4239412	9.3128192	10.754118
AT1G06467	223.00403	1.4139443	0.2121276	6.6655373	2.64E-11	1.17E-09	8.4811667	8.4470485	8.1916935	6.6548684	6.5484377	7.2378745
AT2G31730	39.01916	1.418728	0.3100495	4.5758107	4.74E-06	8.08E-05	6.0417185	5.7892052	5.9855335	3.219378	4.6894999	4.3546248
AT1G26770	117.75285	1.4205485	0.2094836	6.7811911	1.19E-11	5.53E-10	7.4091579	7.5288475	7.4031689	5.9853613	6.1385289	5.776022
AT2G23600	247.7658	1.4210215	0.2299673	6.1792316	6.44E-10	2.38E-08	8.9063983	8.1560741	8.4262144	6.9254163	6.8672798	7.1821107
AT1G09340	1976.3084	1.4235775	0.1723185	8.2613168	1.44E-16	1.26E-14	11.265329	11.467441	11.747073	10.056872	9.7797967	10.215666
AT4G33560	11.174977	1.4257567	0.4297667	3.3175135	9.08E-04	8.04E-03	4.3820199	4.0098434	4.5261657	0	2.9287097	2.148558
AT3G56705	2078.9352	1.4294933	0.2505251	5.7059883	1.16E-08	3.35E-07	11.489825	11.567793	11.736656	9.7437014	9.4589569	10.638711
AT5G02255	19.637192	1.4297791	0.4355052	3.2830357	0.001027	0.0089055	4.6278367	4.5355639	5.7319184	3.219378	0	3.6376272
AT4G38970	6510.2835	1.4345782	0.1533513	9.3548474	8.37E-21	1.04E-18	13.033385	13.142905	13.465165	11.86808	11.568412	11.795645
AT2G41170	103.13543	1.4353576	0.2187788	6.5607722	5.35E-11	2.24E-09	7.211258	7.3854737	7.2434372	5.4117401	5.9195455	5.776022
AT1G09333	3651.8549	1.4362389	0.2369504	6.0613485	1.35E-09	4.69E-08	12.31962	12.596331	12.299029	10.637624	10.324764	11.397521
AT5G59320	29.225247	1.438022	0.4299866	3.3443417	0.0008248	0.0074237	3.9107996	6.0581447	6.0336839	3.7517613	2.5754861	3.8812119
AT2G23100	58.829993	1.4412695	0.2733239	5.2731194	1.34E-07	3.25E-06	6.4298254	6.3859538	6.6112871	4.9116189	5.2241406	4.5784734
AT3G12965	126.65553	1.4418526	0.2189456	6.5854381	4.54E-11	1.92E-09	7.3674665	7.6007961	7.662487	6.3215962	6.1385289	5.7455674
AT1G75040	134.27617	1.4430085	0.3700823	3.8991555	9.65E-05	0.001164	7.9166469	8.165595	6.6191983	6.3946946	6.2366987	4.6459844
AT5G65613	178.93057	1.4451137	0.213553	6.767001	1.31E-11	6.01E-10	7.9565608	7.9373062	8.3063828	5.9853613	6.7831397	6.5850078
AT2G04375	1940.4618	1.4468877	0.2724793	5.3100836	1.10E-07	2.70E-06	11.447684	11.494845	11.59642	9.6572178	9.1162058	10.561046
AT4G28190	100.69138	1.4477789	0.2371194	6.1056959	1.02E-09	3.65E-08	7.2253302	7.0212939	7.4703122	5.5461594	5.6612702	5.8058471

AT3G59068	698.13906	1.4515133	0.3980414	3.6466389	0.0002657	0.0027948	10.164882	9.798592	10.34531	7.6476919	7.2740963	8.9659342
AT3G07725	100.21037	1.4534767	0.2409079	6.03333	1.61E-09	5.47E-08	6.9928396	7.3357375	7.3847234	5.5461594	5.4591985	5.9193391
AT4G07075	194.50456	1.4535909	0.3380293	4.3001925	1.71E-05	2.53E-04	7.9195348	8.2530414	8.4552317	6.9739302	4.8634794	6.9073704
AT4G00975	92.805451	1.4596307	0.2382346	6.1268617	8.96E-10	3.24E-08	7.3069972	6.9292741	7.1206306	5.669115	5.4039261	5.650145
AT5G43750	232.54032	1.4609844	0.1973011	7.4048452	1.31E-13	8.40E-12	8.213459	8.4174686	8.6454274	7.0208656	6.6705562	7.0261521
AT1G44575	3035.0546	1.4655867	0.1880523	7.7935056	6.52E-15	4.96E-13	11.905769	12.058556	12.406078	10.827801	10.246411	10.69159
AT2G21595	21.016972	1.4667087	0.3977967	3.6870813	0.0002268	0.0024527	5.1446787	4.5058886	5.5780659	2.3665114	3.4490907	3.3443906
AT3G03850	75.255634	1.467935	0.2736706	5.3638744	8.15E-08	2.06E-06	7.1238145	6.6663714	6.7689431	4.6971932	5.0187179	5.6168815
AT5G54030	32.621718	1.4688895	0.4191231	3.504673	4.57E-04	4.44E-03	5.5091544	5.0073492	6.3320744	4.6971932	3.8307199	1.7177495
AT3G04155	34.497353	1.4691497	0.3246868	4.524821	6.04E-06	1.01E-04	5.7617592	5.9739483	5.5452237	3.7517613	4.1322253	4.1834245
AT5G03670	12.261661	1.4695462	0.4274314	3.4380866	5.86E-04	5.51E-03	4.6278367	4.6485132	4.0522736	0	2.106732	2.9759397
AT5G23730	18.631671	1.4701149	0.3930357	3.7404109	1.84E-04	2.04E-03	4.9092797	5.069528	4.9590991	2.3665114	2.106732	3.7645543
AT3G59070	688.75705	1.4706387	0.3993911	3.6822017	0.0002312	0.0024916	10.148974	9.7863013	10.335139	7.5866041	7.2271013	8.925752
AT5G48850	9.3531733	1.4731421	0.438873	3.3566476	7.89E-04	7.14E-03	4.0008133	4.0515212	4.2689102	0	2.5754861	1.7177495
AT2G26560	195.35411	1.477083	0.4424628	3.3383211	0.0008429	0.0075557	8.445546	8.4893639	8.151344	5.669115	6.8672798	4.1834245
AT3G03820	171.22399	1.4799629	0.2019417	7.328663	2.32E-13	1.44E-11	7.9424322	8.0340382	8.0699823	6.2445954	6.1719999	6.7627976
AT1G29430	150.67258	1.4805009	0.2247932	6.5860576	4.52E-11	1.92E-09	7.8874454	7.6077975	7.9211377	6.6548684	5.7525968	6.3420395
AT4G04485	164.80702	1.4830464	0.2676631	5.5407208	3.01E-08	8.15E-07	7.9593699	7.4876909	8.3039307	6.8232017	5.8384847	6.3215387
AT1G60840	5.1369223	1.4835318	0.4549737	3.2606979	1.11E-03	9.53E-03	3.7119409	3.3177801	3.2632718	0	0	1.1007184
AT5G41471	47.502875	1.4854921	0.3004123	4.944844	7.62E-07	1.58E-05	6.4298254	6.1182165	5.9608419	4.9116189	4.4916251	4.3546248
AT4G08040	88.107022	1.487395	0.2905058	5.1200185	3.06E-07	6.85E-06	7.2576412	6.9624259	7.1150465	3.219378	5.9584295	5.5479613
AT5G48490	574.09433	1.4877596	0.2094092	7.1045585	1.21E-12	6.75E-11	9.9179499	9.5005815	9.8195856	7.789971	7.9886655	8.5693522
AT4G29310	137.52051	1.4908192	0.2145387	6.9489509	3.68E-12	1.88E-10	7.5721874	7.6216992	7.8920476	5.8874513	6.3579929	6.0245506
AT1G53480	27.688779	1.4933256	0.3954876	3.7759104	1.59E-04	1.81E-03	5.8123562	4.8271098	5.7464384	2.3665114	4.3814822	2.9759397
AT5G06845	29.118793	1.4945351	0.3486485	4.2866533	1.81E-05	0.0002663	5.3975898	5.5338318	5.5452237	4.4452387	3.9893347	2.9759397
AT3G07385	8.0109721	1.4972819	0.4498193	3.3286299	8.73E-04	0.007773	3.8635952	3.5680299	4.186117	2.3665114	0	1.7177495
AT3G61198	918.6162	1.4978555	0.4067376	3.6826091	2.31E-04	2.49E-03	10.723427	10.348425	10.518684	7.9681804	7.434391	9.3056679
AT2G30190	7.194642	1.498043	0.4500056	3.3289432	0.0008718	0.0077674	3.6576272	3.922692	3.8519957	0	1.4079361	1.7177495
AT5G05860	81.06528	1.4989208	0.2458404	6.0971297	1.08E-09	3.83E-08	7.0571928	6.8547197	6.9110554	5.5461594	4.9431857	5.5122259
AT2G37540	111.97504	1.4998711	0.2151065	6.9726915	3.11E-12	1.62E-10	7.3418608	7.3854737	7.521864	5.4117401	5.996293	5.8637088
AT4G07070	200.45406	1.5012592	0.3110315	4.8267107	1.39E-06	2.70E-05	7.9816486	8.3056589	8.4728028	6.9739302	5.2241406	6.9210618
AT5G01195	79.126641	1.5015086	0.245187	6.1239314	9.13E-10	3.29E-08	6.9482853	7.0472781	6.754649	5.0982599	5.4591985	5.2769537
AT1G52030	53.086994	1.5036168	0.312265	4.8151945	1.47E-06	2.85E-05	6.0310764	6.7823036	6.2220902	4.4452387	4.5939523	4.8314086

AT3G12320	678.14491	1.5037061	0.1509805	9.9596045	2.29E-23	3.54E-21	10.176396	9.906866	9.8417308	8.4172953	8.5174315	8.3741423
AT1G68570	110.9423	1.5087663	0.2436822	6.1915336	5.96E-10	2.21E-08	7.1681987	7.5288475	7.3939756	6.3215962	5.7525968	5.3597148
AT2G09805	180.61701	1.5095318	0.2276001	6.6323856	3.30E-11	1.44E-09	8.0090216	7.9261466	8.3640157	5.7824096	6.8046392	6.5324599
AT1G51820	82.565466	1.5098186	0.320105	4.7166348	2.40E-06	4.41E-05	6.6544552	7.3483332	6.8585546	5.7824096	5.5637694	4.2715627
AT1G19376	5.4345746	1.5102344	0.4551706	3.3179528	9.07E-04	8.03E-03	3.4172774	3.3845282	3.6811312	0	1.4079361	0
AT1G04197	8.4731014	1.5106861	0.4442804	3.4002987	6.73E-04	6.22E-03	3.7642837	4.1314298	4.0982816	0	2.106732	1.7177495
AT2G04039	195.73368	1.5119948	0.2100028	7.1998795	6.03E-13	3.50E-11	8.111074	8.0495479	8.387366	6.9739302	6.6705562	6.258228
AT2G29350	74.600069	1.5154225	0.3555878	4.2617393	2.03E-05	2.94E-04	6.3803752	7.5544397	6.3416693	5.2634978	5.4039261	4.3546248
AT3G27170	80.218304	1.5256158	0.2369838	6.4376362	1.21E-10	4.85E-09	6.9983134	6.8722697	6.9238874	5.4117401	5.3464515	5.233722
AT5G53880	91.786516	1.5304588	0.2475187	6.1832039	6.28E-10	2.33E-08	7.128814	7.3731995	6.9110554	5.0982599	5.5637694	5.6168815
AT1G15002	1862.6158	1.5319201	0.201382	7.6070369	2.80E-14	1.98E-12	11.58589	11.295986	11.476068	9.7082944	9.4384737	10.245899
AT3G06895	5198.8032	1.5379034	0.1979804	7.767957	7.98E-15	5.99E-13	12.975849	12.65992	13.138701	11.042043	11.126044	11.692152
AT3G06900	5198.3321	1.538399	0.1978434	7.7758419	7.49E-15	5.65E-13	12.975849	12.65992	13.138615	11.042043	11.126044	11.691153
AT1G68238	17.525028	1.5384824	0.4108299	3.7448165	0.0001805	0.002013	4.7626012	4.9423694	4.9838203	3.219378	0	3.4984464
AT5G01495	15.719796	1.5409075	0.4070041	3.7859756	1.53E-04	1.74E-03	4.5993045	5.0896727	4.5261657	2.3665114	2.5754861	2.9759397
AT1G07887	27.15329	1.5456848	0.393367	3.9293708	8.52E-05	0.0010406	5.7998723	4.8271098	5.7172508	2.3665114	4.2622301	2.9759397
AT1G15980	499.71508	1.5476938	0.1998339	7.7448998	9.57E-15	7.06E-13	9.1409948	9.683186	9.7839587	7.9440305	8.0529572	7.8086415
AT5G10572	69.569045	1.5511724	0.3390082	4.575619	4.75E-06	8.08E-05	6.7353505	6.7190501	6.8850438	5.2634978	3.2122368	5.5122259
AT3G50340	135.26723	1.5534379	0.2524366	6.1537754	7.57E-10	2.77E-08	7.3967765	7.662618	8.0050462	5.7824096	5.707656	6.3622531
AT4G03295	23.632794	1.5555813	0.3659879	4.2503629	2.13E-05	0.0003076	5.0634535	5.442797	5.368765	2.3665114	3.8307199	3.3443906
AT5G08525	36.495547	1.5561502	0.4031955	3.8595425	0.0001136	0.0013373	5.9986708	6.5107257	4.8011554	3.219378	4.4916251	3.3443906
AT3G48420	357.2367	1.5563565	0.204949	7.5938722	3.10E-14	2.18E-12	8.7399383	9.0442444	9.3863696	7.5550621	7.2740963	7.506598
AT1G09075	8.6035961	1.5596139	0.4478574	3.4823893	0.000497	0.0047727	3.6576272	3.8770591	4.3472088	2.3665114	1.4079361	1.1007184
AT5G03120	184.94547	1.5600721	0.1856592	8.4028823	4.36E-17	3.99E-15	8.1311443	8.082591	8.1890383	6.5306385	6.2679916	6.6846883
AT5G02865	32.899574	1.5644757	0.3572297	4.3794676	1.19E-05	0.0001844	5.6409784	5.6469116	5.8576194	4.4452387	2.106732	4.2715627
AT2G07774	76.979044	1.5698668	0.2544621	6.1693549	6.86E-10	2.53E-08	6.9595535	6.7636171	7.0345117	4.4452387	5.2241406	5.4755828
AT5G03155	8.3268629	1.5744112	0.4499234	3.4992873	4.67E-04	4.51E-03	3.4812217	4.1314298	4.3085906	0	2.106732	1.1007184
AT5G00590	63.97343	1.5778548	0.3053565	5.1672554	2.38E-07	5.46E-06	6.6475046	6.4261165	6.9554768	3.219378	4.8634794	5.3597148
AT3G17890	173.50412	1.5793233	0.1874382	8.4258347	3.58E-17	3.34E-15	8.0090216	8.0262203	8.0641983	6.7131893	6.1385289	6.4212482
AT1G08123	23.47245	1.5930932	0.3635489	4.3820605	1.18E-05	1.83E-04	5.2945766	5.4111291	5.2101985	2.3665114	3.2122368	3.7645543
AT2G09775	5.9636347	1.5960996	0.4551078	3.5070804	0.0004531	0.00441	3.4172774	3.8770591	3.5548313	0	1.4079361	0
AT1G29435	70.653629	1.6011358	0.2831991	5.653746	1.57E-08	4.41E-07	6.8304803	6.4957067	7.0285834	4.9116189	4.5939523	5.3597148
AT1G04013	114.5146	1.6026137	0.2424989	6.6087456	3.88E-11	1.68E-09	7.4091579	7.2353334	7.7261412	5.7824096	5.4591985	5.94637

AT3G02380	317.85035	1.6054545	0.179189	8.9595573	3.26E-19	3.62E-17	9.1223159	8.7361583	8.8824489	7.1946821	7.3924105	7.1591873
AT1G09800	834.87792	1.6088236	0.2928051	5.4945206	3.92E-08	1.05E-06	10.073842	10.461926	10.482279	7.9440305	7.8814647	9.2633007
AT3G09600	153.07749	1.6103283	0.2593079	6.2101	5.30E-10	1.98E-08	8.3024347	7.7632475	7.4833739	6.163252	6.3286119	5.9193391
AT1G06537	24.470043	1.6123304	0.3681608	4.379419	1.19E-05	0.0001844	5.2945766	5.577282	5.2916592	0	3.4490907	3.8812119
AT3G62030	1930.0723	1.6133709	0.1925322	8.3797442	5.30E-17	4.82E-15	11.122568	11.541296	11.826739	9.9758138	9.6161692	9.8950825
AT3G04165	414.23537	1.6165944	0.2030119	7.9630536	1.68E-15	1.37E-13	9.0210841	9.5504901	9.2830924	7.9681804	7.5407104	7.3433248
AT5G38430	4451.1767	1.6168387	0.1712715	9.4402063	3.72E-21	4.75E-19	12.54941	12.546189	13.031348	11.16467	10.844015	11.124027
AT5G38420	9013.2638	1.6199677	0.1812565	8.937433	3.98E-19	4.40E-17	13.522887	13.594453	14.066226	11.976228	11.874447	12.290217
AT2G08370	5.0901033	1.6240886	0.4533732	3.5822334	3.41E-04	0.0034694	2.959449	3.5680299	3.7972589	0	0	0
AT3G05355	15.282497	1.6241202	0.4192338	3.8740202	1.07E-04	0.0012742	4.9550358	5.0490981	4.2281074	0	2.5754861	2.9759397
AT1G49245	5564.7035	1.6255464	0.1965017	8.272428	1.31E-16	1.15E-14	13.070723	12.792765	13.271225	11.078552	11.15458	11.716891
AT4G07145	43.758665	1.6258271	0.3228206	5.0363179	4.75E-07	1.02E-05	6.4854533	5.8845319	6.0217961	3.7517613	4.1322253	4.5784734
AT4G04465	50.411722	1.6261938	0.2988492	5.4415189	5.28E-08	1.38E-06	6.2115664	6.1473397	6.487097	5.0982599	4.4916251	4.0895494
AT2G37250	755.41363	1.6277198	0.1598677	10.181671	2.39E-24	4.10E-22	10.114735	10.243322	10.167902	8.2889558	8.3150859	8.7680306
AT1G19050	64.468874	1.6306331	0.2754714	5.919429	3.23E-09	1.03E-07	6.6264497	6.5976972	6.6348919	5.669115	4.5939523	4.5076474
AT1G06227	314.37083	1.6314005	0.2053876	7.9430317	1.97E-15	1.57E-13	8.7235077	9.2529098	8.7023937	7.2743201	7.1110995	7.2268928
AT1G29071	16.717075	1.635471	0.4059698	4.0285535	5.61E-05	0.0007192	4.5404893	5.1095399	4.9083485	2.3665114	2.106732	3.1718957
AT5G51720	22.382605	1.6435948	0.3812342	4.3112468	1.62E-05	2.41E-04	5.0634535	5.0896727	5.3874144	4.1397727	2.106732	3.1718957
AT4G07135	44.184855	1.6445306	0.3205229	5.1307749	2.89E-07	6.53E-06	6.4854533	5.9074109	6.0571696	3.7517613	4.1322253	4.5784734
AT1G29440	314.61647	1.6455133	0.164218	10.020297	1.24E-23	2.00E-21	8.9437585	8.7552016	9.0247462	7.2743201	7.1785237	7.2158269
AT1G19450	169.35529	1.6474492	0.235281	7.0020488	2.52E-12	1.33E-10	7.6748846	8.1750535	8.1863783	6.4642672	6.4698754	5.9193391
AT1G21910	186.17379	1.6482839	0.2343436	7.0336189	2.01E-12	1.07E-10	8.250628	7.871927	8.3710605	6.163252	6.8046392	6.122608
AT5G00760	14.426208	1.6505067	0.4152775	3.9744667	7.05E-05	0.0008864	4.7881068	4.4755902	4.5919633	3.219378	2.5754861	1.7177495
AT1G72960	12.449973	1.6526422	0.4255225	3.8837949	1.03E-04	1.23E-03	4.1655639	4.564641	4.6548905	2.3665114	2.106732	2.148558
AT1G72600	4463.9624	1.6622627	0.1958306	8.4882679	2.10E-17	1.99E-15	12.904579	12.389332	12.896157	10.599479	11.171227	11.152598
AT1G04733	801.48426	1.6666378	0.307181	5.425588	5.78E-08	1.50E-06	10.025738	10.433638	10.44103	7.789971	7.6277174	9.1803443
AT1G30350	5.0762873	1.6670213	0.4541546	3.6706033	0.000242	0.0025785	3.2802015	3.4483243	3.6811312	0	0	0
AT5G53740	2250.2984	1.6670381	0.2198453	7.5827778	3.38E-14	2.37E-12	11.968117	11.550637	11.752029	10.095755	9.3966135	10.326556
AT5G53905	6464.0593	1.6672002	0.2213249	7.5328199	4.97E-14	3.42E-12	13.584345	12.977587	13.241822	11.668829	10.965906	11.772703
AT2G16660	172.91566	1.6710534	0.2224349	7.5125498	5.80E-14	3.93E-12	7.7318539	8.1075036	8.3014745	6.4642672	6.2679916	6.2144228
AT1G72610	4492.895	1.6723638	0.1978068	8.4545294	2.80E-17	2.64E-15	12.917457	12.398824	12.909525	10.587836	11.179479	11.151146
AT1G06667	7.1963355	1.6747396	0.4544193	3.6854499	2.28E-04	0.0024636	3.9565084	3.3845282	4.0047499	2.3665114	0	0
AT3G05655	64.863173	1.6820389	0.2964766	5.6734294	1.40E-08	3.98E-07	6.6887145	6.8547197	6.5628862	4.6971932	3.9893347	5.3189277

AT3G24612	27.225314	1.6860953	0.3831616	4.4004808	1.08E-05	1.70E-04	5.5692344	5.9074109	4.8557473	3.7517613	3.2122368	3.3443906
AT2G40100	34.69634	1.6875891	0.3548196	4.7561895	1.97E-06	3.70E-05	5.3640627	5.9410631	6.1030242	3.7517613	4.2622301	3.3443906
AT3G04175	25.203686	1.6882881	0.3618799	4.6653271	3.08E-06	5.52E-05	5.3809236	5.458374	5.3113254	3.7517613	2.5754861	3.6376272
AT5G27780	38.891606	1.6994791	0.3471577	4.895409	9.81E-07	1.98E-05	6.06277	5.5629435	6.2831183	4.1397727	3.2122368	4.3546248
AT2G00850	231.45947	1.7043997	0.2312453	7.3705269	1.70E-13	1.08E-11	8.5517361	8.2817805	8.677984	6.3215962	6.3286119	7.112218
AT2G34760	67.524518	1.7182288	0.2861408	6.0048359	1.92E-09	6.43E-08	6.5687531	6.532965	7.0521516	5.2634978	4.7791107	4.6459844
AT5G42800	7.7244298	1.7186228	0.4551803	3.7756966	1.60E-04	1.81E-03	3.128721	4.6754209	3.7972589	0	0	1.1007184
AT5G64850	95.98065	1.7191265	0.2580588	6.6617623	2.71E-11	1.19E-09	7.1681987	6.9894822	7.4833739	5.8874513	5.0904916	5.2769537
AT1G29450	265.93725	1.725429	0.1788254	9.6486778	4.98E-22	6.70E-20	8.7674478	8.5451367	8.789105	6.5940902	6.8875699	7.0759594
AT5G07725	990.76099	1.7267733	0.1777505	9.7145888	2.61E-22	3.65E-20	10.333517	10.793566	10.587843	9.0805521	8.7233573	8.5780204
AT5G54075	7439.9181	1.742944	0.2243989	7.7671681	8.03E-15	6.01E-13	13.804143	13.178977	13.482006	11.810806	11.088193	11.912913
AT1G09797	701.36087	1.7437345	0.2459501	7.0897893	1.34E-12	7.40E-11	9.9507874	10.048034	10.327916	7.5866041	7.9792425	8.7680306
AT1G42970	5002.8575	1.7446561	0.178322	9.7837389	1.32E-22	1.90E-20	12.647383	12.934467	13.155969	11.366676	10.849197	11.081224
AT5G07325	2056.8371	1.7465725	0.4218894	4.1398824	3.47E-05	4.74E-04	11.649457	11.610683	12.042869	8.7171607	8.1569148	10.07884
AT4G03905	495.41159	1.7525169	0.2505741	6.9940075	2.67E-12	1.40E-10	9.4313862	9.6274348	9.7733627	7.5866041	7.0221311	8.2275891
AT1G06253	191.36116	1.7558572	0.3053102	5.7510602	8.87E-09	2.62E-07	8.1336336	8.2440822	8.4352051	5.8874513	5.2865918	6.9745636
AT2G09395	83.059975	1.7571573	0.2665811	6.5914558	4.36E-11	1.86E-09	7.3288855	6.8429	6.9366063	5.2634978	4.8634794	5.2769537
AT1G04227	78.370662	1.7574123	0.2712428	6.479111	9.23E-11	3.75E-09	6.6751082	7.1894011	6.9554768	5.4117401	5.0904916	4.7722096
AT2G07739	1160.5682	1.7592242	0.1439047	12.224922	2.29E-34	7.89E-32	10.646642	10.795484	10.9891	9.058226	9.0901797	8.9017849
AT5G06835	70.610805	1.7643065	0.2571323	6.8614737	6.82E-12	3.28E-10	6.7484027	6.9010534	6.7901222	5.0982599	4.6894999	4.9956923
AT5G02315	21.696532	1.7677304	0.4225241	4.1837385	2.87E-05	4.00E-04	5.2766692	5.8612842	4.2689102	2.3665114	3.2122368	2.4798665
AT3G47342	57.718176	1.7804726	0.3418994	5.2075915	1.91E-07	4.46E-06	6.6613724	7.0107677	5.8576194	4.1397727	4.2622301	4.7722096
AT5G38410	13514.219	1.7895825	0.1811667	9.8780991	5.18E-23	7.58E-21	14.084939	14.238792	14.716689	12.590054	12.308954	12.601565
AT3G05325	22.131661	1.7900826	0.3882945	4.610116	4.02E-06	7.02E-05	5.3470024	5.5041206	4.8287095	3.219378	2.5754861	3.1718957
AT1G57770	88.20446	1.7948232	0.2816127	6.3733753	1.85E-10	7.25E-09	6.7155479	7.3187703	7.3847234	4.6971932	5.0904916	5.4379847
AT1G06283	184.79899	1.7973698	0.3488337	5.1525121	2.57E-07	5.85E-06	8.1060125	8.2305382	8.4216979	5.5461594	4.6894999	6.9480607
AT1G15990	46.978845	1.8075631	0.3184958	5.6753126	1.38E-08	3.95E-07	5.9085191	6.3107171	6.5380629	4.1397727	4.3814822	4.0895494
AT1G11870	720.3017	1.8075633	0.218442	8.2747968	1.29E-16	1.14E-14	10.061475	10.003602	10.387609	7.9681804	7.8713123	8.6741315
AT5G53902	8268.0555	1.8083475	0.2203021	8.2084902	2.24E-16	1.93E-14	13.959847	13.338737	13.665822	11.87608	11.221047	12.028493
AT1G13650	27.58196	1.8105127	0.3727983	4.8565475	1.19E-06	2.35E-05	5.5544473	5.8256948	5.3498714	2.3665114	2.5754861	3.9891381
AT2G09685	40.193597	1.8165445	0.3122537	5.8175281	5.97E-09	1.82E-07	6.0310764	6.0984692	6.1030242	3.219378	3.9893347	4.2715627
AT1G09533	215.20164	1.8177446	0.3403178	5.3413152	9.23E-08	2.30E-06	8.3694417	8.7005791	8.2990141	6.5306385	4.5939523	6.8935479
AT1G09793	604.7961	1.8290748	0.2448889	7.4689989	8.08E-14	5.37E-12	9.756131	9.858522	10.131028	7.3125489	7.6751769	8.4796798

AT1G43605	6.1603834	1.8400132	0.4551446	4.0427006	5.28E-05	6.82E-04	4.0008133	3.7308031	3.4164043	0	0	0
AT2G34510	465.97549	1.8494114	0.1704916	10.847521	2.05E-27	4.35E-25	9.582978	9.4143593	9.6043081	7.3125489	7.4885299	7.8804773
AT3G04775	205.65092	1.8543905	0.2348376	7.8964796	2.87E-15	2.26E-13	8.0983868	8.652297	8.2437951	6.8232017	6.3579929	6.0245506
AT3G20760	93.922363	1.8645892	0.2604881	7.1580605	8.18E-13	4.63E-11	7.1535551	7.3315144	7.2738258	4.9116189	5.7525968	4.7722096
AT4G06235	84.162052	1.8655875	0.264167	7.0621517	1.64E-12	8.85E-11	6.818153	7.2666375	7.1536885	5.0982599	5.2865918	4.8314086
AT3G15310	10.589572	1.8660573	0.4433902	4.2086117	2.57E-05	0.0003637	3.9565084	4.380679	4.6853538	0	2.106732	1.1007184
AT4G37800	414.27221	1.8670027	0.2009827	9.2893722	1.55E-20	1.90E-18	9.019739	9.3625247	9.6600153	7.2743201	7.501753	7.4030832
AT1G10550	98.367045	1.8713458	0.2835522	6.5996514	4.12E-11	1.77E-09	7.0934478	7.0575421	7.7587368	4.6971932	5.4591985	5.3189277
AT2G39330	22.187925	1.8783368	0.4242801	4.427115	9.55E-06	1.52E-04	4.3481248	5.9739483	5.2309989	2.3665114	2.9287097	2.4798665
AT2G30010	138.20333	1.8793422	0.2211854	8.4966828	1.95E-17	1.86E-15	7.8933332	7.6859621	7.8690092	4.9116189	6.0691631	5.9193391
AT5G00765	51.899388	1.882999	0.3132613	6.010953	1.84E-09	6.24E-08	6.1039734	6.562094	6.6033322	4.1397727	3.8307199	4.6459844
AT5G08865	596.15023	1.8876532	0.2883703	6.5459354	5.91E-11	2.46E-09	9.9973173	10.010223	9.7680353	7.1946821	7.1452054	8.5296878
AT3G57765	1768.2675	1.8903099	0.2722626	6.9429654	3.84E-12	1.94E-10	11.394448	11.5358	11.529256	8.8914483	8.7895464	10.07884
AT1G16820	1794.3518	1.8947191	0.2487684	7.6163989	2.61E-14	1.85E-12	11.447183	11.541525	11.515811	9.0694322	8.8985377	10.044851
AT5G66562	10.460583	1.9070547	0.4419406	4.3151833	1.59E-05	2.38E-04	4.1261063	4.6485132	4.2689102	0	1.4079361	1.7177495
AT4G06305	141.69586	1.9073098	0.2664275	7.1588315	8.14E-13	4.62E-11	8.088156	7.5471738	7.882219	5.7824096	5.0904916	6.1692457
AT1G07393	15.717015	1.9121468	0.4263682	4.4847318	7.30E-06	0.0001188	4.7101961	5.3117282	4.4921063	0	2.9287097	1.7177495
AT5G01015	16.409895	1.9161567	0.4214239	4.5468625	5.45E-06	9.16E-05	4.5101575	4.9200424	5.1890938	2.3665114	2.9287097	1.1007184
AT5G23060	2587.2869	1.9295579	0.1495877	12.899177	4.55E-38	2.01E-35	11.87076	11.956498	12.189719	10.122901	9.7986696	10.145963
AT5G08855	636.60035	1.9323997	0.2813542	6.8682089	6.50E-12	3.14E-10	10.093794	10.150479	9.8264358	7.2743201	7.2740963	8.5649985
AT1G19150	306.98541	1.9345543	0.2174131	8.8980574	5.68E-19	6.18E-17	8.5975359	8.9259816	9.2541711	7.11039	6.6705562	6.9480607
AT4G08735	32.936684	1.9388911	0.344923	5.6212287	1.90E-08	5.28E-07	5.6687059	6.047885	5.7750472	2.3665114	3.8307199	3.6376272
AT4G06255	138.8916	1.9462158	0.2699108	7.2105878	5.57E-13	3.25E-11	8.0674743	7.5177389	7.8756292	5.669115	5.0187179	6.122608
AT3G45851	7.7779184	1.9485758	0.4551787	4.2809025	1.86E-05	0.0002723	3.2064485	4.2071437	4.4572233	0	0	0
AT3G03585	134.85889	1.9511623	0.2475403	7.8822012	3.22E-15	2.52E-13	7.7384106	7.7757353	7.7479532	6.4642672	5.0187179	5.5479613
AT3G02795	162.90213	1.9513969	0.2504907	7.7902975	6.69E-15	5.07E-13	8.0700758	8.2685874	7.7370884	6.4642672	5.3464515	5.9989583
AT1G61275	1951.57	1.9543017	0.4539682	4.3049307	1.67E-05	2.48E-04	12.003592	11.784303	11.706572	7.2350505	5.9195455	9.0462164
AT2G07807	10.180326	1.9588131	0.445135	4.4004926	1.08E-05	0.0001696	4.4791742	4.3137483	4.0522736	2.3665114	0	1.1007184
AT4G39364	259.91643	1.9632679	0.187261	10.484123	1.02E-25	1.93E-23	8.4947851	8.8199406	8.789105	6.8232017	6.6937867	6.5501893
AT5G40400	83.762477	1.9703574	0.2875502	6.85222	7.27E-12	3.49E-10	7.0676446	6.7319246	7.4213815	5.4117401	4.8634794	4.6459844
AT4G13577	108.6535	1.9706342	0.2477069	7.9555067	1.78E-15	1.45E-13	7.6012556	7.3357375	7.5091477	5.2634978	4.8634794	5.7144559
AT5G08155	2582.3147	1.9725388	0.2544053	7.7535275	8.94E-15	6.62E-13	12.36852	11.626497	12.039004	10.028443	9.1792882	10.249973
AT3G07715	875.62313	1.9750543	0.1944064	10.15941	3.01E-24	5.07E-22	10.656196	10.557686	10.157075	8.0382954	8.3520603	8.6700833

AT1G05273	1734.5265	1.9810864	0.2636836	7.5131191	5.77E-14	3.92E-12	11.415314	11.524052	11.485295	8.9040219	8.6598951	9.9461945
AT5G01505	72.563471	1.992348	0.2731214	7.2947349	2.99E-13	1.83E-11	6.7353505	7.0575421	6.9863893	4.1397727	4.5939523	5.0465438
AT1G32080	399.03745	2.0101045	0.2158131	9.3141	1.23E-20	1.52E-18	8.9980453	9.3416779	9.6142382	7.5866041	6.9075785	7.1706945
AT2G38240	114.87144	2.0187975	0.3613021	5.5875612	2.30E-08	6.35E-07	7.2253302	7.8276344	7.7151102	5.4117401	5.7961798	3.3443906
AT5G01017	14.187569	2.0236883	0.4300779	4.7053998	2.53E-06	4.62E-05	4.3481248	4.7532581	5.008125	2.3665114	2.106732	1.1007184
AT5G54070	6641.8511	2.0259784	0.2253323	8.9910687	2.45E-19	2.73E-17	13.69382	13.069444	13.39216	11.415795	10.671915	11.499712
AT3G56380	13.302527	2.0289221	0.4380163	4.6320695	3.62E-06	6.38E-05	4.5701966	4.2790814	5.008125	2.3665114	2.106732	0
AT3G03445	459.93966	2.0444887	0.4451804	4.5924952	4.38E-06	7.55E-05	9.6635635	9.9591277	9.523361	5.0982599	5.0187179	7.2915629
AT1G10522	2119.2362	2.0462418	0.2925883	6.9935876	2.68E-12	1.40E-10	12.042195	11.472245	11.79129	9.124194	8.6598951	10.174821
AT5G04415	141.41574	2.0499946	0.2596837	7.8941969	2.92E-15	2.30E-13	7.5685124	7.7726235	8.1996297	5.8874513	5.1588632	5.835068
AT5G59860	11.802343	2.053447	0.4361629	4.7079822	2.50E-06	4.57E-05	4.5101575	4.621094	4.4572233	0	2.106732	1.1007184
AT3G25180	11.732505	2.0582562	0.4516703	4.5569883	5.19E-06	8.75E-05	3.8635952	5.1675511	4.2281074	2.3665114	0	0
AT3G24615	167.10616	2.0788538	0.2581632	8.0524778	8.11E-16	6.79E-14	7.7707547	8.3893026	8.0814811	6.3946946	5.8795843	5.3597148
AT4G22570	281.25779	2.0796473	0.1834045	11.339129	8.40E-30	2.06E-27	8.6815935	8.8876633	8.9372704	7.0208656	6.4698754	6.6522207
AT5G08860	37.585713	2.0863048	0.3986096	5.2339558	1.66E-07	3.93E-06	6.0203552	6.6797225	5.1014451	3.219378	3.6524875	2.9759397
AT1G15580	34.267955	2.0899874	0.3682661	5.6752101	1.39E-08	3.95E-07	5.4140656	5.9954625	6.158366	4.1397727	2.5754861	3.3443906
AT2G46830	516.51273	2.0991666	0.2104232	9.9759296	1.94E-23	3.04E-21	10.093155	9.5819399	9.4297498	7.7626156	7.3924105	7.4224649
AT5G08795	79.362191	2.1077122	0.3816433	5.5227284	3.34E-08	8.97E-07	7.7546733	7.1225353	5.8841256	5.0982599	3.8307199	4.2715627
AT1G08117	26.947695	2.1191618	0.3688508	5.7453096	9.18E-09	2.71E-07	5.4140656	5.6055384	5.7024326	3.219378	2.5754861	3.1718957
AT2G28210	14.975237	2.1286479	0.4469965	4.7621126	1.92E-06	3.61E-05	4.9092797	5.4111291	3.8519957	2.3665114	1.4079361	0
AT1G08347	9.4395243	2.1363436	0.4491204	4.7567279	1.97E-06	3.70E-05	4.4475108	4.2071437	4.186117	0	0	1.1007184
AT2G22990	2291.8069	2.1384336	0.1201573	17.796949	7.46E-71	2.35E-67	11.827018	11.95667	11.828233	9.8579725	9.6757188	9.5826071
AT2G06855	19.233713	2.1417382	0.4047864	5.2910327	1.22E-07	2.97E-06	5.3470024	5.1095399	4.9838203	2.3665114	1.4079361	2.7491208
AT4G08755	31.499846	2.1442262	0.3570439	6.0054971	1.91E-09	6.41E-08	5.6409784	6.0375518	5.7464384	2.3665114	3.2122368	3.4984464
AT3G03435	469.39206	2.1523658	0.4300509	5.0049095	5.59E-07	1.19E-05	9.6781314	9.966636	9.5401262	5.669115	5.2865918	7.3932938
AT5G06825	1621.3538	2.1540804	0.2417153	8.9116417	5.03E-19	5.52E-17	11.198837	11.446359	11.544656	8.8530562	8.4977674	9.6103368
AT1G76210	55.270888	2.1567854	0.3093525	6.9719353	3.13E-12	1.62E-10	6.3970476	6.5107257	6.8178859	3.219378	4.5939523	3.9891381
AT2G04435	1110.1088	2.168286	0.2727695	7.9491513	1.88E-15	1.51E-13	10.761345	10.959429	10.886724	7.9440305	7.861088	9.1486907
AT2G04455	1110.1088	2.168286	0.2727695	7.9491513	1.88E-15	1.51E-13	10.761345	10.959429	10.886724	7.9440305	7.861088	9.1486907
AT4G06010	156.62084	2.1727385	0.2581156	8.4176955	3.84E-17	3.55E-15	7.6748846	8.3418016	7.9929191	6.163252	5.6612702	5.3189277
AT3G57770	831.30381	2.1850287	0.2487672	8.7834263	1.59E-18	1.63E-16	10.137238	10.619179	10.552646	7.4560742	7.8194503	8.6121814
AT3G59710	46.580092	2.1911267	0.3231342	6.7808564	1.19E-11	5.53E-10	6.0937824	6.4261165	6.4162149	4.4452387	3.2122368	3.8812119
AT5G25190	330.91816	2.1920324	0.190131	11.529066	9.42E-31	2.54E-28	8.8799548	9.119656	9.3065541	6.5306385	6.8875699	6.9073704

AT1G09867	217.06635	2.2199911	0.1870605	11.86777	1.74E-32	5.57E-30	8.4634662	8.4874672	8.5136958	6.5940902	6.1385289	5.9989583
AT4G15258	71.814583	2.2200761	0.2959191	7.5023084	6.27E-14	4.23E-12	6.6613724	7.1371269	7.0166534	4.1397727	4.9431857	4.0895494
AT4G09845	150.75675	2.2276504	0.2344997	9.4995857	2.11E-21	2.72E-19	7.9252933	8.1368417	7.9211377	5.5461594	5.0904916	5.9729037
AT3G02725	54.413235	2.2284803	0.3272312	6.8101094	9.75E-12	4.59E-10	6.1341217	6.8838522	6.6112871	3.7517613	3.9893347	4.0895494
AT3G01145	60.609706	2.2447233	0.3331354	6.7381718	1.60E-11	7.27E-10	6.7088862	7.0212939	6.4071046	4.1397727	2.9287097	4.6459844
AT1G04217	56.324576	2.2605762	0.3099329	7.2937592	3.01E-13	1.83E-11	6.6193626	6.3695703	6.7689431	4.6971932	3.4490907	4.0895494
AT3G05715	717.00928	2.2688301	0.3030239	7.4872967	7.03E-14	4.70E-12	10.392388	10.0286	10.364851	6.9739302	6.9659915	8.451654
AT1G09855	17.564141	2.2694024	0.4218214	5.3800079	7.45E-08	1.89E-06	5.0841925	5.2944746	4.8822876	0	0	2.7491208
AT5G09935	1301.9762	2.2860025	0.2397389	9.5353835	1.49E-21	1.94E-19	11.006492	11.226613	11.091461	8.1897496	8.2222785	9.2085298
AT2G08860	538.28215	2.2902045	0.2477968	9.2422685	2.41E-20	2.91E-18	9.7569385	9.8926038	9.8800968	6.8232017	6.8875699	7.9422067
AT5G08845	73.536871	2.2969878	0.405104	5.6701193	1.43E-08	4.04E-07	7.7285643	7.0421185	5.7464384	4.9116189	2.5754861	3.7645543
AT2G46192	2682.4166	2.2989644	0.2239568	10.265214	1.01E-24	1.77E-22	12.190184	11.915294	12.316469	9.2278502	9.4690905	10.174821
AT1G06553	76.035118	2.3094862	0.3196396	7.2252821	5.00E-13	2.93E-11	6.818153	6.8896087	7.3847234	4.6971932	3.2122368	4.8314086
AT4G03925	378.52076	2.3117083	0.1999446	11.561747	6.44E-31	1.78E-28	9.1335522	9.2816515	9.4924683	7.3860957	6.5985322	6.7627976
AT5G04305	577.08969	2.3185322	0.2318838	9.9986812	1.54E-23	2.44E-21	9.8301289	9.766103	10.19396	7.1946821	7.0036595	7.915097
AT2G10735	14.687068	2.3253645	0.4317666	5.3856985	7.22E-08	1.84E-06	4.977381	4.6485132	4.9590991	0	0	2.148558
AT4G06225	70.3751	2.3267078	0.3031807	7.674328	1.66E-14	1.21E-12	6.605083	7.1225353	6.9985713	4.6971932	4.5939523	3.7645543
AT2G04965	7116.1089	2.3358831	0.1888649	12.368008	3.89E-35	1.36E-32	13.434987	13.408434	13.788795	10.663737	11.281714	11.282241
AT1G07487	11.279868	2.3446687	0.4446822	5.2726835	1.34E-07	3.25E-06	4.4475108	4.5931436	4.5594396	0	0	1.1007184
AT1G74456	76.980778	2.3537816	0.2795854	8.4188274	3.80E-17	3.53E-15	7.0780213	7.0626468	7.0924916	4.1397727	3.8307199	4.9429826
AT5G01775	285.35903	2.3623947	0.2382453	9.9158094	3.55E-23	5.30E-21	8.8163005	9.1994545	8.6874213	7.0208656	6.1042628	6.0987114
AT3G55850	806.94245	2.3702032	0.2144086	11.05461	2.08E-28	4.69E-26	10.191408	10.198807	10.799334	7.9440305	7.603389	8.1307557
AT1G04897	571.39977	2.3727679	0.2571025	9.2288793	2.73E-20	3.28E-18	9.8370187	9.7574717	10.200566	6.9254163	6.8258231	7.9219223
AT5G04425	46.391529	2.39638	0.3229126	7.42114	1.16E-13	7.47E-12	6.4135296	6.4021533	6.2324425	4.1397727	3.2122368	3.6376272
AT1G20015	24.278653	2.3998143	0.3945416	6.0825387	1.18E-09	4.17E-08	5.2401736	5.633252	5.5452237	3.219378	1.4079361	2.4798665
AT5G08515	85.989308	2.4001167	0.353107	6.797137	1.07E-11	4.97E-10	7.182695	7.6693264	6.7618138	4.6971932	4.9431857	2.9759397
AT5G01785	255.9516	2.4055148	0.2630048	9.1462779	5.89E-20	6.92E-18	8.686688	9.0748837	8.4987644	6.875214	5.9584295	5.6168815
AT2G09695	2378.8158	2.4193329	0.2397169	10.092457	5.97E-24	9.83E-22	12.063403	11.720937	12.177548	8.9532484	9.0321256	9.924268
AT1G70185	760.35376	2.4218033	0.3989629	6.0702464	1.28E-09	4.47E-08	10.222727	10.14868	10.826923	7.0663221	5.9584295	7.8159882
AT1G06243	193.97859	2.4221971	0.2759608	8.7773221	1.67E-18	1.71E-16	8.1435479	8.7971516	8.095727	6.3946946	5.4039261	5.3993805
AT1G04277	69.405184	2.4327182	0.3269674	7.4402471	1.00E-13	6.60E-12	6.8243298	7.0107677	6.9985713	4.4452387	2.106732	4.710477
AT3G09225	43.341763	2.4446136	0.3399704	7.190665	6.45E-13	3.73E-11	6.0203552	6.3940763	6.3512009	4.1397727	2.9287097	3.3443906
AT2G05565	14.326006	2.4478485	0.4352947	5.6234276	1.87E-08	5.22E-07	4.9550358	4.6485132	4.933947	0	0	1.7177495

AT3G27865	155.69714	2.4501414	0.2202478	11.124475	9.54E-29	2.22E-26	7.9816486	8.1937865	7.9868171	5.8874513	5.3464515	5.3597148
AT5G33370	47.618616	2.4576866	0.368816	6.6637196	2.67E-11	1.18E-09	5.7357793	6.7060597	6.6809735	3.219378	3.8307199	2.9759397
AT2G08900	28.080058	2.475544	0.3974611	6.2283926	4.71E-10	1.78E-08	5.5692344	6.1182165	5.3874144	2.3665114	1.4079361	2.9759397
AT2G39030	34.227805	2.4766961	0.4212718	5.8790925	4.13E-09	1.29E-07	5.6823724	6.7190501	5.2515037	2.3665114	3.2122368	1.1007184
AT5G50915	71.383463	2.4950503	0.2803291	8.9004338	5.56E-19	6.08E-17	6.9254813	6.9894822	7.0285834	4.1397727	3.9893347	4.433164
AT5G04085	484.39578	2.4955474	0.1879093	13.280596	3.00E-40	1.51E-37	9.5738416	9.5432043	9.9663555	6.875214	7.0937393	7.2268928
AT5G02055	78.698431	2.5048905	0.3535103	7.0857635	1.38E-12	7.59E-11	7.1437095	7.6793308	6.3416693	3.7517613	4.4916251	3.8812119
AT5G04485	122.72278	2.5331547	0.2610614	9.7032915	2.92E-22	4.00E-20	7.6986156	7.5724458	8.0080621	3.7517613	5.1588632	5.233722
AT4G06240	65.406785	2.5425544	0.3191516	7.9666051	1.63E-15	1.34E-13	6.5239141	7.0626468	6.9554768	4.1397727	4.3814822	3.3443906
AT2G09885	34.146669	2.5441704	0.3692428	6.8902373	5.57E-12	2.75E-10	6.1733636	5.7262603	6.0687706	0	3.4490907	2.7491208
AT4G39361	243.61429	2.5526745	0.2441505	10.455331	1.39E-25	2.57E-23	8.4213006	8.949596	8.7482056	6.4642672	6.1042628	5.4379847
AT5G04465	122.44718	2.5558965	0.2618363	9.7614301	1.65E-22	2.35E-20	7.6986156	7.5724458	8.0080621	3.7517613	5.0904916	5.233722
AT3G47348	282.78739	2.5754513	0.1881095	13.691231	1.15E-42	6.84E-40	8.8917673	9.0609214	8.8525598	6.3946946	5.996293	6.3821874
AT2G08725	63.951043	2.5973663	0.3121775	8.320159	8.78E-17	7.85E-15	6.583395	6.9733094	7.0106512	2.3665114	3.9893347	4.1834245
AT3G05335	78.051182	2.6017579	0.2938073	8.8553202	8.34E-19	8.86E-17	7.0360591	7.2308054	7.0226307	5.0982599	3.2122368	4.2715627
AT1G06087	65.104585	2.6180028	0.374155	6.997108	2.61E-12	1.38E-10	6.8608456	7.1800365	6.6960127	2.3665114	1.4079361	4.5784734
AT1G75163	43.990716	2.624451	0.3679302	7.133014	9.82E-13	5.52E-11	6.0937824	6.7255017	6.0336839	4.1397727	2.5754861	2.7491208
AT5G13225	75.989401	2.6297157	0.3627231	7.2499264	4.17E-13	2.49E-11	7.108711	7.662618	6.2731246	3.7517613	4.2622301	3.4984464
AT2G08375	24.468135	2.6531768	0.4086321	6.4928252	8.42E-11	3.43E-09	5.5244106	5.9074109	5.2101985	0	2.106732	2.148558
AT4G39366	370.44518	2.6535109	0.179006	14.823587	1.03E-49	8.43E-47	9.2413497	9.439104	9.3523632	6.163252	6.5227227	6.8077106
AT3G03175	17.312618	2.6559317	0.4303407	6.1716956	6.76E-10	2.49E-08	4.8378037	5.2233195	5.2717211	0	0	1.7177495
AT4G07625	35.42415	2.6599551	0.3760415	7.0735686	1.51E-12	8.25E-11	5.7226118	6.2313399	6.1030242	3.219378	3.2122368	1.7177495
AT2G35747	303.2958	2.6678908	0.2679348	9.9572401	2.34E-23	3.60E-21	8.9005637	9.5294434	8.6061604	6.5940902	6.0331881	5.835068
AT5G09505	83.328881	2.6718322	0.3607043	7.4072644	1.29E-13	8.28E-12	7.192279	7.8484736	6.4071046	3.219378	4.2622301	3.9891381
AT2G07754	38.659715	2.6722019	0.3534616	7.5600905	4.03E-14	2.80E-12	6.2670382	6.0581447	6.1905799	2.3665114	2.5754861	3.3443906
AT4G21680	103.67261	2.6930455	0.3530012	7.6289975	2.37E-14	1.69E-12	6.9983134	7.421682	8.0496361	4.9116189	4.6894999	2.9759397
AT1G09125	31.14422	2.6969724	0.3904192	6.9078889	4.92E-12	2.46E-10	5.7872795	6.0061005	5.9357204	0	0	3.3443906
AT5G09515	81.800904	2.7088379	0.3643021	7.435692	1.04E-13	6.78E-12	7.163334	7.8336193	6.3794235	3.219378	4.2622301	3.7645543
AT5G03195	97.741857	2.7384995	0.2949677	9.2840659	1.63E-20	1.98E-18	7.3967765	7.1467733	7.7976011	3.7517613	4.6894999	4.3546248
AT4G03995	923.19148	2.7653183	0.2205779	12.536696	4.70E-36	1.82E-33	10.638777	10.806937	10.580771	7.5228149	7.2271013	8.1997771
AT4G04195	28.939961	2.7656996	0.4025181	6.8709937	6.38E-12	3.09E-10	5.7357793	5.577282	6.1030242	0	0	2.9759397
AT4G04185	28.939961	2.7656996	0.4025181	6.8709937	6.38E-12	3.09E-10	5.7357793	5.577282	6.1030242	0	0	2.9759397
AT1G09087	31.983828	2.768443	0.3951621	7.0058423	2.46E-12	1.30E-10	5.5983615	5.8014713	6.2529269	3.219378	2.5754861	1.1007184

AT1G02380	89.270544	2.7685463	0.2724508	10.161639	2.94E-24	4.99E-22	7.3759017	7.2353334	7.3754114	4.1397727	4.5939523	4.0895494
AT5G13930	108.2862	2.7819663	0.2782386	9.9984923	1.55E-23	2.44E-21	7.3069972	7.7121867	7.7515567	4.9116189	3.9893347	4.710477
AT2G35744	366.27336	2.8017397	0.2709601	10.340046	4.64E-25	8.40E-23	8.8932371	9.7232267	9.3236008	6.8232017	6.2986201	5.650145
AT3G02832	151.39799	2.8101524	0.2560999	10.972877	5.16E-28	1.15E-25	7.9079484	8.3078102	8.0437698	4.6971932	4.5939523	5.4379847
AT1G04527	23.252455	2.8235328	0.4208175	6.7096374	1.95E-11	8.72E-10	5.7998723	5.1095399	5.610177	0	0	2.148558
AT5G13170	43.821205	2.8460841	0.3971863	7.1656156	7.74E-13	4.43E-11	5.5692344	6.8429	6.512805	0	2.9287097	2.7491208
AT5G02655	66.059554	2.8998605	0.3291774	8.8094167	1.26E-18	1.31E-16	7.0831818	6.9514597	6.6426751	4.6971932	3.2122368	2.9759397
AT1G07593	23.242549	2.944716	0.4251295	6.9266329	4.31E-12	2.16E-10	5.2766692	5.328778	5.9357204	0	0	1.7177495
AT1G04263	708.82589	2.9498799	0.2960594	9.9638094	2.19E-23	3.41E-21	10.102715	10.051248	10.747493	7.0663221	5.9584295	7.5156503
AT1G04517	21.705127	2.9537402	0.4194672	7.0416483	1.90E-12	1.02E-10	5.3640627	5.4111291	5.5452237	0	1.4079361	1.1007184
AT4G06250	72.385533	2.9572797	0.3042292	9.7205638	2.46E-22	3.47E-20	6.8905849	7.1800365	7.0981635	3.7517613	3.6524875	3.7645543
AT1G01060	373.66459	2.9609359	0.2586256	11.448735	2.39E-30	6.13E-28	9.8642535	9.0685539	9.1170308	6.5940902	6.0331881	5.94637
AT1G08875	21.956114	2.9681212	0.4190409	7.0831305	1.41E-12	7.72E-11	5.3975898	5.4111291	5.5617383	0	1.4079361	1.1007184
AT3G08565	684.24735	2.9761837	0.2888972	10.301879	6.91E-25	1.22E-22	10.044379	10.00824	10.697927	6.9739302	5.996293	7.4510579
AT5G51174	214.39405	2.981479	0.264606	11.267616	1.90E-29	4.55E-27	8.1484797	8.9217739	8.6646672	4.9116189	5.6133433	5.2769537
AT1G05853	674.46391	2.9883551	0.2984228	10.013829	1.33E-23	2.12E-21	10.029085	9.9936141	10.678656	6.9739302	5.7961798	7.3932938
AT4G07635	121.26384	3.0038614	0.2687279	11.178077	5.22E-29	1.24E-26	7.7514354	7.8806246	7.6663101	5.4117401	4.3814822	3.9891381
AT4G06310	71.119009	3.0076698	0.310916	9.6735767	3.90E-22	5.32E-20	6.8608456	7.1894011	7.1038131	2.3665114	3.8307199	3.7645543
AT1G06453	2909.2632	3.0085106	0.2646433	11.368173	6.02E-30	1.49E-27	12.269428	12.405619	12.429466	8.7592901	8.3299896	9.6924616
AT1G08353	25.274927	3.0651383	0.4196394	7.3042192	2.79E-13	1.71E-11	5.6549088	5.3117282	5.9483358	0	1.4079361	1.1007184
AT5G07105	655.87564	3.0740365	0.2464276	12.474403	1.03E-35	3.79E-33	10.243042	10.399751	9.9901913	7.2350505	6.0331881	7.1821107
AT3G03595	99.8122	3.0787937	0.2823346	10.904771	1.09E-27	2.34E-25	7.4851983	7.5027931	7.521864	5.0982599	3.2122368	4.0895494
AT5G40395	61.627352	3.0887031	0.3528747	8.7529732	2.08E-18	2.10E-16	6.8243298	6.4651914	7.1913224	3.7517613	2.106732	3.3443906
AT2G07759	35.452878	3.1029167	0.3824355	8.1135683	4.92E-16	4.19E-14	6.1733636	6.0166606	6.158366	0	1.4079361	2.7491208
AT1G16635	239.3398	3.1137092	0.2802744	11.109502	1.13E-28	2.59E-26	8.2868634	9.2495563	8.637659	5.5461594	5.3464515	5.1431664
AT1G08937	112.92637	3.1246058	0.3005841	10.395114	2.61E-25	4.76E-23	7.6611467	7.5140169	7.9559138	3.7517613	3.2122368	4.7722096
AT1G31835	5234.2885	3.1461505	0.1971329	15.959537	2.45E-57	2.57E-54	13.136582	13.234667	13.272322	9.8579725	9.4350313	10.291411
AT5G02645	162.59499	3.1565002	0.2394061	13.18471	1.07E-39	5.27E-37	8.1533946	8.3333786	8.1486135	5.2634978	4.4916251	4.888274
AT1G12013	1018.2135	3.2581262	0.2832875	11.501128	1.30E-30	3.46E-28	10.928717	10.573418	11.106312	6.4642672	6.7391548	7.9219223
AT3G55860	93.482406	3.2816856	0.3212702	10.214722	1.70E-24	2.94E-22	7.3025794	7.2308054	7.8183666	2.3665114	3.4490907	4.0895494
AT3G09855	152.70772	3.2877278	0.2454122	13.396761	6.32E-41	3.24E-38	8.0674743	8.2191535	8.1293538	4.9116189	4.3814822	4.6459844
AT2G35387	77.251891	3.3436159	0.3199509	10.450402	1.46E-25	2.68E-23	7.0780213	7.3525075	7.1806696	0	3.9893347	3.1718957
AT3G02445	100.52375	3.3681893	0.3503929	9.612607	7.07E-22	9.47E-20	7.3504466	8.1319932	7.057984	3.219378	3.2122368	3.7645543

AT3G09865	149.71405	3.40665	0.2530896	13.460253	2.68E-41	1.41E-38	8.0517661	8.2076783	8.1238035	4.4452387	4.1322253	4.6459844
AT3G50825	129.73624	3.4073302	0.2645669	12.878898	5.92E-38	2.56E-35	7.8636511	8.0078123	7.8985629	4.1397727	4.3814822	4.0895494
AT2G09705	260.59487	3.4138159	0.2652397	12.870683	6.58E-38	2.79E-35	8.7366671	8.9161445	9.0788156	5.9853613	3.9893347	5.3189277
AT1G05247	225.6679	3.5251882	0.3005948	11.727374	9.23E-32	2.75E-29	8.2158103	9.2224439	8.5901495	4.6971932	5.0187179	4.433164
AT3G01155	80.346875	3.5393768	0.3336805	10.607084	2.76E-26	5.40E-24	6.9651548	7.4492288	7.3613299	2.3665114	3.4490907	2.9759397
AT5G02665	55.125681	3.5635035	0.3777329	9.433923	3.95E-21	5.01E-19	6.6264497	7.1077946	6.4697003	0	2.5754861	2.148558
AT2G08910	85.157825	3.6501074	0.3277737	11.136058	8.37E-29	1.97E-26	7.3547204	7.5544397	7.1482312	2.3665114	3.2122368	3.1718957
AT5G66564	115.6202	3.7827074	0.2960866	12.775678	2.24E-37	9.17E-35	7.7219626	7.8978641	7.7407191	3.7517613	3.6524875	3.3443906
AT1G07897	461.63003	3.9092054	0.2726772	14.336384	1.30E-46	8.94E-44	9.2721785	9.9801881	9.9970383	5.0982599	4.9431857	5.9193391
AT3G21805	696.92475	3.9092219	0.2478886	15.770077	5.00E-56	4.80E-53	9.8784228	10.769573	10.339335	5.7824096	6.3286119	6.122608
AT1G05913	483.13673	3.9467849	0.2303047	17.13723	7.83E-66	1.15E-62	9.7101726	10.140254	9.6112664	6.0770468	5.5124312	5.4379847
AT1G09787	162.34738	3.9627019	0.3046485	13.007455	1.11E-38	5.10E-36	8.0090216	8.4798552	8.3710605	2.3665114	2.9287097	4.433164
AT1G53541	454.15564	4.0621552	0.2850534	14.250507	4.45E-46	2.98E-43	9.2516993	9.9652737	9.9879018	4.9116189	4.4916251	5.7455674
AT4G09135	176.42014	4.102493	0.2822829	14.533268	7.46E-48	5.31E-45	8.2801383	8.5505972	8.3569362	4.6971932	3.9893347	3.1718957
AT2G07605	316.63301	4.1216597	0.2403776	17.146605	6.66E-66	1.05E-62	9.0034994	9.3748897	9.3356549	4.6971932	4.6894999	4.9429826
AT1G05917	476.34405	4.1549269	0.2349226	17.686368	5.34E-70	1.47E-66	9.700131	10.136022	9.6023139	5.669115	5.4039261	5.1891546
AT3G13857	242.96927	4.190939	0.2452626	17.087558	1.84E-65	2.54E-62	8.863553	8.8184325	8.9117313	4.6971932	4.3814822	4.1834245
AT5G46315	232.7394	4.3088034	0.2559009	16.837784	1.29E-63	1.68E-60	8.8209416	8.7488817	8.8542367	4.6971932	3.8307199	4.0895494
AT1G09937	1141.7187	4.3273864	0.2353471	18.387248	1.66E-75	9.17E-72	10.926206	10.884296	11.436039	5.8874513	6.2047119	6.8654991
AT1G09943	1141.7187	4.3273864	0.2353471	18.387248	1.66E-75	9.17E-72	10.926206	10.884296	11.436039	5.8874513	6.2047119	6.8654991
AT5G09115	241.62808	4.3328205	0.252661	17.148751	6.42E-66	1.05E-62	8.866549	8.8154115	8.9133408	4.4452387	4.1322253	4.0895494
AT3G13855	241.27046	4.3339458	0.2527271	17.148718	6.43E-66	1.05E-62	8.863553	8.8184325	8.9085069	4.4452387	3.9893347	4.1834245
AT5G00750	307.15831	4.3345365	0.2581871	16.788356	2.97E-63	3.64E-60	9.1752279	8.9768917	9.453175	4.4452387	4.2622301	4.6459844
AT3G14735	241.001	4.3680418	0.2546299	17.15447	5.82E-66	1.05E-62	8.863553	8.8169228	8.9085069	4.4452387	3.9893347	4.0895494
AT4G04615	241.35049	4.3696455	0.2547087	17.15546	5.72E-66	1.05E-62	8.8650518	8.8169228	8.9133408	4.4452387	3.9893347	4.0895494
AT4G07875	241.33268	4.3698242	0.2546438	17.160535	5.24E-66	1.05E-62	8.8680447	8.8169228	8.91012	4.4452387	3.9893347	4.0895494
