# Supplementary material for: Induction of epigenetic variation in Arabidopsis by over-expression of DNA METHYLTRANSFERASE1 (MET1)
Source: PLoS One. 2018 Feb 21;13(2):e0192170. doi: 10.1371/journal.pone.0192170 (PMC5821449; doi:10.1371/journal.pone.0192170)
Supplement: S5 Table — (PDF) [file pone.0192170.s010.pdf]

S5 Table: List of transposable elements with at least log2-fold increases (negative log2-fold change) or decreases (positive log2-fold change) of 2.5 in at least one of the four lines A1+, A1-, A2+ or A2-.

	baseMean	log2FoldC	lfcSE	stat	pvalue	padj	1	2	3	4	5	6		
AT3G30765	14.6841	-4.1818	0.55546	-7.5285	5.13E-14	1.57E-12	0	0	0	4.16454	5.31093	5.06504	A1+	CACTA-like transposase family (En/Spm)
AT3G30765	40.2308	-1.8764	0.17903	-10.481	1.06E-25	4.93E-23	0	0	0	6.47959	6.57793	5.89756	A2+	CACTA-like transposase family (En/Spm)
AT3G32230	24.9516	-4.3726	0.54547	-8.0163	1.09E-15	3.88E-14	0.64015	0	0	6.47656	5.39625	4.39129	A1+	CACTA-like transposase family (En/Spm)
AT3G32230	120.169	-2.8096	0.17926	-15.673	2.31E-55	3.62E-52	0.95419	0	0	7.43773	8.33181	7.82976	A2+	CACTA-like transposase family (En/Spm)
AT5G28927	10.499	-3.6534	0.57304	-6.3755	1.82E-10	3.78E-09	0	0	0	3.48023	4.78679	4.76714	A1+	CACTA-like transposase family (En/Spm)
AT5G28927	26.0408	-1.5251	0.17551	-8.6897	3.63E-18	1.18E-15	0	0	0	5.70877	5.95958	5.48258	A2+	CACTA-like transposase family (En/Spm)
AT1G33130	206.788	-7.1397	0.47209	-15.124	1.13E-51	3.16E-49	0	0.62641	0	7.35364	9.27081	8.85145	A1+	CACTA-like transposase family (Ptta/En/Spm)
AT1G33130	152.602	-2.3986	0.18018	-13.313	1.96E-40	1.86E-37	0	0.94033	0	7.27523	8.84792	8.23847	A2+	CACTA-like transposase family (Ptta/En/Spm)
AT1G33130	4.04861	-1.9453	0.51292	-3.7926	0.000149	0.001343	0	0.58899	0	2.79716	3.54662	3.02706	A1-	CACTA-like transposase family (Ptta/En/Spm)
AT1G36460	171.743	-4.0442	0.5825	-6.9428	3.84E-12	9.79E-11	0	0	0	6.44887	8.72874	9.02752	A1+	CACTA-like transposase family (Ptta/En/Spm)
AT1G36460	7.33409	-2.8544	0.51338	-5.56	2.70E-08	4.67E-07	0	0	0	3.22203	4.48353	3.93573	A1-	CACTA-like transposase family (Ptta/En/Spm)
AT1G36460	21.2748	-1.2745	0.17026	-7.4858	7.11E-14	1.63E-11	0	0	0	5.24553	5.85636	5.12007	A2+	CACTA-like transposase family (Ptta/En/Spm)
AT1G39110	288.187	-7.6555	0.45711	-16.748	5.88E-63	2.56E-60	0	0.62641	0	7.97503	9.47582	9.58482	A1+	CACTA-like transposase family (Ptta/En/Spm)
AT1G39110	24.923	-4.4474	0.46564	-9.5511	1.28E-21	6.68E-20	0	0.58899	0	4.94268	6.02485	5.81168	A1-	CACTA-like transposase family (Ptta/En/Spm)
AT1G39110	34.1742	-1.6979	0.1779	-9.5443	1.37E-21	5.21E-19	0	0.94033	0	5.84377	6.47716	5.92141	A2+	CACTA-like transposase family (Ptta/En/Spm)
AT1G40118	38.5434	-5.5376	0.50935	-10.872	1.57E-27	1.29E-25	0	0	0	5.6469	6.37902	6.65714	A1+	CACTA-like transposase family (Ptta/En/Spm)
AT1G40118	4.41923	-2.148	0.51141	-4.2002	2.67E-05	0.000283	0	0	0	2.19233	3.54662	3.72897	A1-	CACTA-like transposase family (Ptta/En/Spm)
AT1G40124	10.6002	-3.8275	0.56532	-6.7705	1.28E-11	3.06E-10	0	0	0	4.16454	4.6545	4.55355	A1+	CACTA-like transposase family (Ptta/En/Spm)
AT1G42500	20.8688	-4.4442	0.53148	-8.3619	6.17E-17	2.44E-15	0	0	0.69459	5.74125	5.62557	4.62832	A1+	CACTA-like transposase family (Ptta/En/Spm)
AT1G42500	60.3166	-2.0544	0.17979	-11.427	3.07E-30	1.82E-27	0	0	1.02486	6.34038	7.39731	6.83609	A2+	CACTA-like transposase family (Ptta/En/Spm)
AT1G43840	47.5903	-5.7159	0.48217	-11.855	2.04E-32	2.27E-30	0	0	0.69459	6.3018	6.9582	6.40407	A1+	CACTA-like transposase family (Ptta/En/Spm)
AT1G43840	81.1509	-2.0668	0.1795	-11.514	1.12E-30	7.10E-28	0	0	1.02486	6.37646	7.68023	7.64877	A2+	CACTA-like transposase family (Ptta/En/Spm)
AT1G49080	103.51	-4.6495	0.56914	-8.1693	3.10E-16	1.16E-14	0	0	0	6.06692	8.18598	8.05421	A1+	CACTA-like transposase family (Ptta/En/Spm)
AT1G49080	3.22732	-1.759	0.4998	-3.5195	0.000432	0.003447	0	0	0	2.19233	3.54662	2.61074	A1-	CACTA-like transposase family (Ptta/En/Spm)
AT1G49080	9.35912	-0.6908	0.14533	-4.7533	2.00E-06	1.94E-04	0	0	0	4.03478	4.79992	3.89804	A2+	CACTA-like transposase family (Ptta/En/Spm)
AT1G49090	299.586	-7.5399	0.43674	-17.264	8.76E-67	4.79E-64	1.08201	0.62641	0	8.14863	9.54284	9.58717	A1+	CACTA-like transposase family (Ptta/En/Spm)
AT1G49090	15.05	-3.2843	0.48787	-6.732	1.67E-11	4.11E-10	1.02605	0.58899	0	3.81676	5.61718	4.83714	A1-	CACTA-like transposase family (Ptta/En/Spm)
AT1G49090	29.6886	-1.4686	0.1758	-8.3539	6.61E-17	1.97E-14	1.52355	0.94033	0	5.55983	6.33081	5.66312	A2+	CACTA-like transposase family (Ptta/En/Spm)
AT1G50850	159.35	-7.1604	0.47357	-15.12	1.20E-51	3.30E-49	0	0	0	7.30812	8.51647	8.76296	A1+	CACTA-like transposase family (Ptta/En/Spm)
AT1G50850	5.33775	-2.3745	0.51452	-4.6149	3.93E-06	4.88E-05	0	0	0	2.52621	4.08983	3.61329	A1-	CACTA-like transposase family (Ptta/En/Spm)
AT1G50850	22.4425	-1.3308	0.17163	-7.7539	8.91E-15	2.22E-12	0	0	0	5.70877	5.716	5.03519	A2+	CACTA-like transposase family (Ptta/En/Spm)
AT2G06250	62.2787	-5.8497	0.43399	-13.479	2.08E-41	2.83E-39	0	0	0	6.69143	7.47011	6.58514	A1-	CACTA-like transposase family (Ptta/En/Spm)
AT2G06250	43.4201	-5.3386	0.52709	-10.129	4.13E-24	2.87E-22	0	0	0	7.29262	5.47681	5.95894	A1+	CACTA-like transposase family (Ptta/En/Spm)
AT2G06490	42.7423	-5.2818	0.4889	-10.803	3.31E-27	2.66E-25	0.64015	0.62641	0	6.47656	6.87146	5.7063	A1+	CACTA-like transposase family (Ptta/En/Spm)
AT2G06490	43.2074	-5.2372	0.42964	-12.19	3.53E-34	3.42E-32	0.60238	0.58899	0	6.86349	6.02485	6.31589	A1-	CACTA-like transposase family (Ptta/En/Spm)
AT2G06590	278.716	-7.9087	0.38426	-20.582	4.00E-94	3.31E-91	0	0	0	8.91946	9.39698	9.01381	A1-	CACTA-like transposase family (Ptta/En/Spm)
AT2G06590	66.1754	-6.0071	0.50385	-11.922	9.05E-33	1.02E-30	0	0	0	7.74386	6.81059	6.19861	A1+	CACTA-like transposase family (Ptta/En/Spm)
AT2G06590	15.2447	-0.9563	0.15944	-5.9978	2.00E-09	2.72E-07	0	0	0	4.42764	5.52727	4.74575	A2+	CACTA-like transposase family (Ptta/En/Spm)
AT2G06670	204.547	-7.5714	0.38849	-19.49	1.35E-84	8.24E-82	0	0	0	8.57685	8.83012	8.61952	A1-	CACTA-like transposase family (Ptta/En/Spm)
AT2G06670	65.2755	-5.9298	0.50814	-11.67	1.82E-31	1.91E-29	0	0	0	7.78874	6.68057	6.14868	A1+	CACTA-like transposase family (Ptta/En/Spm)
AT2G06670	11.3677	-0.8559	0.15525	-5.5131	3.53E-08	4.22E-06	0	0	0	4.35716	4.79992	4.51449	A2+	CACTA-like transposase family (Ptta/En/Spm)
AT2G06720	158.172	-7.2118	0.44792	-16.101	2.52E-58	9.25E-56	0	0	0.69459	8.69406	8.33388	7.74544	A1+	CACTA-like transposase family (Ptta/En/Spm)
AT2G06720	59.9711	-5.5088	0.43862	-12.559	3.54E-36	3.82E-34	0	0	0.65347	6.05437	7.47011	6.8886	A1-	CACTA-like transposase family (Ptta/En/Spm)
AT2G06720	55.1444	-2.2977	0.18021	-12.75	3.12E-37	2.59E-34	0	0	1.02486	6.75096	6.9973	6.60532	A2+	CACTA-like transposase family (Ptta/En/Spm)
AT2G06800	45.421	-5.5083	0.51853	-10.623	2.33E-26	1.80E-24	0	0	0	5.31997	6.84135	6.91779	A1+	CACTA-like transposase family (Ptta/En/Spm)
AT2G06800	40.1664	-4.9957	0.46414	-10.763	5.12E-27	3.63E-25	0	0	0	5.67203	7.12834	5.73073	A1-	CACTA-like transposase family (Ptta/En/Spm)
AT2G06800	22.1605	-1.3858	0.17307	-8.0072	1.17E-15	3.22E-13	0	0	0	5.24553	5.62471	5.60542	A2+	CACTA-like transposase family (Ptta/En/Spm)
AT2G10000	50.6116	-5.4726	0.4315	-12.683	7.38E-37	8.21E-35	0	0	0.65347	6.41786	7.12834	6.33427	A1-	CACTA-like transposase family (Ptta/En/Spm)
AT2G10000	6.35742	-2.7932	0.58396	-4.7831	1.73E-06	1.98E-05	0	0	0.69459	3.68404	4.16427	3.28443	A1+	CACTA-like transposase family (Ptta/En/Spm)
AT2G10640	73.3693	-5.8021	0.48632	-11.931	8.20E-33	9.28E-31	0	0.62641	0.69459	5.99213	7.4721	7.64998	A1+	CACTA-like transposase family (Ptta/En/Spm)
AT2G12300	113.427	-3.812	0.58517	-6.5143	7.30E-11	1.60E-09	0	0	0	5.78621	8.16181	8.41783	A1+	CACTA-like transposase family (Ptta/En/Spm)
AT2G12300	10.4711	-2.8575	0.51417	-5.5576	2.74E-08	4.73E-07	0	0	0	2.52621	5.45092	4.02897	A1-	CACTA-like transposase family (Ptta/En/Spm)
AT2G12300	21.3152	-1.2279	0.16885	-7.2725	3.53E-13	7.17E-11	0	0	0	5.24553	5.93446	4.99081	A2+	CACTA-like transposase family (Ptta/En/Spm)
AT2G13160	101.994	-6.7869	0.47359	-14.331	1.41E-46	2.94E-44	0	0	0	7.98471	7.85024	7.03235	A1+	CACTA-like transposase family (Ptta/En/Spm)
AT2G13160	10.0958	-3.3894	0.50378	-6.7279	1.72E-11	4.22E-10	0	0	0	4.04193	4.7925	4.2772	A1-	CACTA-like transposase family (Ptta/En/Spm)

AT2G13160	188.726	-3.0156	0.17912	-16.836	1.34E-63	2.96E-60	0	0	0	7.88866	9.02198	8.56418	A2+	CACTA-like transposase family (Ptta/En/Spm)
AT2G13175	33.5971	-4.8378	0.4379	-11.048	2.24E-28	1.70E-26	0	0.58899	0.65347	5.80081	6.43434	5.93726	A1-	CACTA-like transposase family (Ptta/En/Spm)
AT2G13175	17.3241	-4.1806	0.51967	-8.0447	8.64E-16	3.10E-14	0	0.62641	0.69459	5.31997	5.12347	4.95315	A1+	CACTA-like transposase family (Ptta/En/Spm)
AT2G13870	178.891	-4.0137	0.58301	-6.8845	5.80E-12	1.45E-10	0	0	0	6.47656	8.85467	9.03441	A1+	CACTA-like transposase family (Ptta/En/Spm)
AT2G13870	20.2952	-3.8956	0.49586	-7.8563	3.96E-15	1.33E-13	0	0	0	3.93374	6.24407	5.07435	A1-	CACTA-like transposase family (Ptta/En/Spm)
AT2G13870	55.8699	-1.9887	0.17934	-11.089	1.42E-28	7.53E-26	0	0	0	6.24602	7.32182	6.67711	A2+	CACTA-like transposase family (Ptta/En/Spm)
AT2G14230	230.524	-6.5806	0.33379	-19.715	1.60E-86	1.96E-83	2.12133	1.89477	1.79647	9.17596	8.67853	8.60533	A1+	CACTA-like transposase family (Ptta/En/Spm)
AT2G14230	76.9529	-2.1397	0.17991	-11.893	1.28E-32	8.98E-30	2.7279	2.48409	2.3615	6.72321	7.63416	7.19025	A2+	CACTA-like transposase family (Ptta/En/Spm)
AT2G14970	15.2081	-4.1544	0.55776	-7.4484	9.45E-14	2.83E-12	0	0	0	4.02145	5.47681	5.06504	A1+	CACTA-like transposase family (Ptta/En/Spm)
AT3G29634	31.4577	-4.7568	0.51406	-9.2532	2.18E-20	1.13E-18	0.64015	0.62641	0	5.05192	5.88373	6.62098	A1+	CACTA-like transposase family (Ptta/En/Spm)
AT3G29650	38.2969	-4.802	0.47231	-10.167	2.78E-24	1.69E-22	0	0	0	5.19788	7.12834	5.81168	A1-	CACTA-like transposase family (Ptta/En/Spm)
AT3G29730	124.661	-6.4628	0.45391	-14.238	5.31E-46	1.08E-43	0.64015	0.62641	0.69459	6.96128	8.16181	8.40193	A1+	CACTA-like transposase family (Ptta/En/Spm)
AT3G29730	7.94052	-2.54	0.50515	-5.0282	4.95E-07	7.15E-06	0.60238	0.58899	0.65347	3.22203	4.48353	4.11654	A1-	CACTA-like transposase family (Ptta/En/Spm)
AT3G29730	21.2118	-1.2332	0.17195	-7.1718	7.40E-13	1.45E-10	0.95419	0.94033	1.02486	5.46248	5.68621	4.99081	A2+	CACTA-like transposase family (Ptta/En/Spm)
AT3G29734	149.003	-5.5674	0.32874	-16.935	2.47E-64	1.17E-61	1.6933	2.80343	2.41385	8.55808	7.89491	8.0744	A1+	CACTA-like transposase family (Ptta/En/Spm)
AT3G29734	85.491	-4.7167	0.32562	-14.485	1.50E-47	2.61E-45	1.61959	2.71064	2.32499	7.0304	7.67257	7.40574	A1-	CACTA-like transposase family (Ptta/En/Spm)
AT3G29736	125.207	-5.6657	0.34771	-16.294	1.09E-59	4.23E-57	1.41979	2.55913	1.79647	8.24034	7.60349	7.95603	A1+	CACTA-like transposase family (Ptta/En/Spm)
AT3G29736	66.0799	-4.6873	0.34666	-13.521	1.17E-41	1.60E-39	1.35313	2.46929	1.71887	6.74043	7.18241	7.11812	A1-	CACTA-like transposase family (Ptta/En/Spm)
AT3G29739	86.7203	-5.6416	0.3924	-14.377	7.21E-47	1.56E-44	1.41979	1.3957	1.51363	7.77765	7.04001	7.38479	A1+	CACTA-like transposase family (Ptta/En/Spm)
AT3G29739	57.8484	-4.7896	0.39042	-12.268	1.35E-34	1.36E-32	1.35313	1.32917	1.44299	6.41786	7.42597	6.45679	A1-	CACTA-like transposase family (Ptta/En/Spm)
AT3G30218	69.9228	-5.8868	0.45365	-12.977	1.66E-38	2.54E-36	0	1.06174	0.69459	6.50374	7.37033	7.36306	A1+	CACTA-like transposase family (Ptta/En/Spm)
AT3G30396	95.6345	-4.2065	0.57827	-7.2743	3.48E-13	9.78E-12	0	0	0	5.78621	7.83504	8.19614	A1+	CACTA-like transposase family (Ptta/En/Spm)
AT3G30396	5.5642	-2.5051	0.51512	-4.8631	1.16E-06	1.58E-05	0	0	0	3.02518	4.08983	3.48753	A1-	CACTA-like transposase family (Ptta/En/Spm)
AT3G30396	20.1037	-1.3251	0.17187	-7.7097	1.26E-14	3.08E-12	0	0	0	5.32153	5.42276	5.3483	A2+	CACTA-like transposase family (Ptta/En/Spm)
AT3G30663	132.313	-6.8027	0.48689	-13.972	2.32E-44	4.45E-42	0	0	0	6.79525	8.34466	8.49478	A1+	CACTA-like transposase family (Ptta/En/Spm)
AT3G30663	15.2263	-1.0147	0.16211	-6.2594	3.86E-10	5.59E-08	0	0	0	4.94274	5.34862	4.51449	A2+	CACTA-like transposase family (Ptta/En/Spm)
AT3G30744	83.6618	-6.5612	0.45345	-14.47	1.88E-47	4.36E-45	0	0	0.69459	7.24511	7.34909	7.56688	A1+	CACTA-like transposase family (Ptta/En/Spm)
AT3G30744	8.68255	-2.4763	0.5147	-4.8112	1.50E-06	2.00E-05	0	0	0.65347	2.79716	5.26298	3.19741	A1-	CACTA-like transposase family (Ptta/En/Spm)
AT3G30746	24.2371	-5.0291	0.52177	-9.6385	5.50E-22	3.20E-20	0	0	0	5.43737	5.82339	5.59885	A1+	CACTA-like transposase family (Ptta/En/Spm)
AT3G30746	16.3051	-3.8831	0.4923	-7.8877	3.08E-15	1.05E-13	0	0	0	4.70005	5.76624	4.35128	A1-	CACTA-like transposase family (Ptta/En/Spm)
AT3G30767	47.1731	-5.4514	0.52361	-10.411	2.20E-25	1.63E-23	0	0	0	5.19216	6.77917	7.12563	A1+	CACTA-like transposase family (Ptta/En/Spm)
AT3G30767	2.70551	-1.6996	0.49678	-3.4213	0.000623	0.004766	0	0	0	2.52621	2.66509	2.83386	A1-	CACTA-like transposase family (Ptta/En/Spm)
AT3G30767	18.3995	-1.236	0.16962	-7.2866	3.18E-13	6.55E-11	0	0	0	5.35808	5.27047	5.07826	A2+	CACTA-like transposase family (Ptta/En/Spm)
AT3G30780	42.5528	-5.5962	0.50953	-10.983	4.62E-28	3.92E-26	0	0	0	6.83858	6.53766	5.67137	A1+	CACTA-like transposase family (Ptta/En/Spm)
AT3G30780	7.49336	-2.9629	0.51229	-5.7836	7.31E-09	1.35E-07	0	0	0	3.39522	4.08983	4.35128	A1-	CACTA-like transposase family (Ptta/En/Spm)
AT3G30780	86.9285	-2.4381	0.18025	-13.526	1.10E-41	1.12E-38	0	0	0	6.83115	7.85105	7.49041	A2+	CACTA-like transposase family (Ptta/En/Spm)
AT3G31920	24.5628	-4.4482	0.55624	-7.9969	1.28E-15	4.52E-14	0	0	0	4.02145	5.55311	6.44602	A1+	CACTA-like transposase family (Ptta/En/Spm)
AT3G32226	14.0812	-3.7572	0.57334	-6.5531	5.63E-11	1.25E-09	0	0	0	4.6268	5.69457	3.45845	A1+	CACTA-like transposase family (Ptta/En/Spm)
AT3G32226	107.56	-2.3469	0.18018	-13.025	8.80E-39	7.81E-36	0	0	0	6.83115	8.05129	8.0727	A2+	CACTA-like transposase family (Ptta/En/Spm)
AT3G32240	163.909	-7.1915	0.39992	-17.982	2.68E-72	1.13E-69	0	0	0	8.02487	8.68785	8.29288	A1-	CACTA-like transposase family (Ptta/En/Spm)
AT3G32240	142.499	-6.9759	0.47971	-14.542	6.58E-48	1.63E-45	0	0	0	8.887	7.49161	7.67664	A1+	CACTA-like transposase family (Ptta/En/Spm)
AT3G32240	49.7462	-2.0129	0.17965	-11.205	3.86E-29	2.14E-26	0	0	0	6.22639	7.06831	6.52977	A2+	CACTA-like transposase family (Ptta/En/Spm)
AT3G32677	71.7235	-5.8474	0.49987	-11.698	1.31E-31	1.39E-29	0.64015	0	0	5.82982	7.4721	7.63192	A1+	CACTA-like transposase family (Ptta/En/Spm)
AT3G33166	97.5228	-4.548	0.43686	-10.411	2.22E-25	1.63E-23	2.29556	2.91163	1.51363	8.01335	7.93823	6.17386	A1+	CACTA-like transposase family (Ptta/En/Spm)
AT3G33166	69.0081	-2.5253	0.51225	-4.9299	8.23E-07	1.15E-05	2.21024	2.81769	1.44299	6.15418	8.17744	5.48962	A1-	CACTA-like transposase family (Ptta/En/Spm)
AT3G33166	18.2525	-0.6626	0.17088	-3.8778	0.000105	0.007358	2.91885	3.58827	2.03713	4.84311	5.1002	4.84882	A2+	CACTA-like transposase family (Ptta/En/Spm)
AT3G42720	333.089	-7.6547	0.45144	-16.956	1.73E-64	8.41E-62	0.64015	0.62641	0	10.1894	8.69546	8.70801	A1+	CACTA-like transposase family (Ptta/En/Spm)
AT3G42720	190.448	-7.0289	0.39155	-17.951	4.68E-72	1.94E-69	0.60238	0.58899	0	7.95047	8.95962	8.64183	A1-	CACTA-like transposase family (Ptta/En/Spm)
AT3G42720	44.5782	-1.6985	0.17757	-9.5653	1.12E-21	4.38E-19	0.95419	0.94033	0	6.22639	7.05671	5.92141	A2+	CACTA-like transposase family (Ptta/En/Spm)
AT3G46487	439.503	-8.2178	0.37506	-21.911	2.05E-106	3.52E-103	1.41979	0	1.16147	9.43434	9.90498	9.94619	A1+	CACTA-like transposase family (Ptta/En/Spm)
AT3G46487	43.0087	-4.2224	0.46125	-9.1542	5.47E-20	2.56E-18	1.35313	0	1.10158	4.99746	7.28482	6.13896	A1-	CACTA-like transposase family (Ptta/En/Spm)
AT3G46487	35.8711	-1.6031	0.17815	-8.9983	2.29E-19	8.13E-17	1.93072	0	1.61801	5.79128	6.54512	6.01303	A2+	CACTA-like transposase family (Ptta/En/Spm)
AT4G03775	17.0843	-3.8175	0.56301	-6.7806	1.20E-11	2.86E-10	0.64015	0	0	5.91325	5.01973	3.61372	A1+	CACTA-like transposase family (Ptta/En/Spm)
AT4G03910	237.406	-7.7136	0.4591	-16.802	2.37E-63	1.05E-60	0	0	0	7.97503	9.17748	9.22111	A1+	CACTA-like transposase family (Ptta/En/Spm)
AT4G03910	5.70029	-2.0042	0.50791	-3.9461	7.94E-05	0.000769	0	0	0	1.75715	4.7925	2.61074	A1-	CACTA-like transposase family (Ptta/En/Spm)
AT4G03910	22.8808	-1.2616	0.16963	-7.4371	1.03E-13	2.28E-11	0	0	0	5.20597	6.07902	5.1607	A2+	CACTA-like transposase family (Ptta/En/Spm)
AT4G04170	1209.68	-5.4349	0.41822	-12.995	1.30E-38	1.57E-36	4.57299	4.65542	3.17332	10.5751	11.2447	11.6679	A1-	CACTA-like transposase family (Ptta/En/Spm)

AT4G04170	528.839	-3.1692	0.16785	-18.882	1.62E-79	5.37E-76	5.40086	5.49538	3.95295	9.97367	10.2222	9.77516	A2+	CACTA-like transposase family (Ptta/En/Spm)
AT4G04430	94.5697	-6.3001	0.44151	-14.27	3.39E-46	6.96E-44	1.08201	0.62641	0	7.62514	7.90949	7.03235	A1+	CACTA-like transposase family (Ptta/En/Spm)
AT4G04430	198.927	-3.1542	0.17787	-17.733	2.32E-70	6.87E-67	1.52355	0.94033	0	8.14022	9.10594	8.49436	A2+	CACTA-like transposase family (Ptta/En/Spm)
AT4G06698	224	-7.7266	0.41103	-18.798	7.84E-79	6.71E-76	1.08201	0	0	8.71746	8.72049	8.97477	A1+	CACTA-like transposase family (Ptta/En/Spm)
AT4G06698	69.6374	-5.2318	0.45161	-11.585	4.92E-31	4.24E-29	1.02605	0	0	5.70532	7.85009	7.09674	A1-	CACTA-like transposase family (Ptta/En/Spm)
AT4G06698	21.1969	-1.2459	0.17135	-7.2713	3.56E-13	7.18E-11	1.52355	0	0	5.20597	5.74518	5.23869	A2+	CACTA-like transposase family (Ptta/En/Spm)
AT4G07518	84.2498	-6.3364	0.47347	-13.383	7.61E-41	1.27E-38	0	0.62641	0	6.65684	7.49161	7.82706	A1+	CACTA-like transposase family (Ptta/En/Spm)
AT4G07518	9.12726	-0.6837	0.14725	-4.6431	3.43E-06	3.19E-04	0	0.94033	0	4.03478	4.55821	4.07853	A2+	CACTA-like transposase family (Ptta/En/Spm)
AT4G08091	385.49	-8.4053	0.40096	-20.963	1.44E-97	2.05E-94	0	1.06174	0	9.71865	9.32622	9.69763	A1+	CACTA-like transposase family (Ptta/En/Spm)
AT4G08091	39.6691	-4.8372	0.44779	-10.802	3.35E-27	2.40E-25	0	1.00603	0	5.45435	6.88921	6.27841	A1-	CACTA-like transposase family (Ptta/En/Spm)
AT4G08091	31.0261	-1.4829	0.17532	-8.4583	2.71E-17	8.49E-15	0	1.50483	0	6.30338	6.07902	5.3483	A2+	CACTA-like transposase family (Ptta/En/Spm)
AT4G08598	45.5674	-5.4681	0.44742	-12.221	2.39E-34	2.33E-32	0	0	0	6.10514	6.24407	7.04187	A1-	CACTA-like transposase family (Ptta/En/Spm)
AT4G08598	30.3989	-5.0941	0.52803	-9.6473	5.05E-22	2.96E-20	0	0	0	6.58232	5.12347	5.77373	A1+	CACTA-like transposase family (Ptta/En/Spm)
AT4G32340	451.696	-1.6716	0.1733	-9.6461	5.11E-22	2.98E-20	7.74001	7.49093	7.92732	9.16323	9.64661	9.45989	A1+	CACTA-like transposase family (Ptta/En/Spm)
AT5G19015	13.7584	-4.1043	0.55734	-7.3641	1.78E-13	5.17E-12	0	0	0	4.89656	5.22024	4.20843	A1+	CACTA-like transposase family (Ptta/En/Spm)
AT5G19015	37.707	-1.2983	0.16928	-7.6696	1.73E-14	4.17E-12	0	0	0	4.94274	6.87059	6.34818	A2+	CACTA-like transposase family (Ptta/En/Spm)
AT5G28923	137.976	-6.4236	0.49672	-12.932	2.97E-38	4.38E-36	0	0.62641	0	6.33243	8.47785	8.61914	A1+	CACTA-like transposase family (Ptta/En/Spm)
AT5G28923	14.1691	-0.9371	0.16031	-5.8455	5.05E-09	6.62E-07	0	0.94033	0	4.84311	5.22975	4.38328	A2+	CACTA-like transposase family (Ptta/En/Spm)
AT5G29408	44.6176	-1.7307	0.17728	-9.7624	1.63E-22	6.58E-20	0	0	0	5.68019	6.88378	6.66303	A2+	CACTA-like transposase family (Ptta/En/Spm)
AT5G29568	5.59228	-2.5257	0.51514	-4.9029	9.44E-07	1.31E-05	0	0	0	3.22203	4.08983	3.34975	A1-	CACTA-like transposase family (Ptta/En/Spm)
AT5G29568	62.8942	-1.9798	0.58218	-3.4007	6.72E-04	4.30E-03	0	0	0	3.48023	7.26084	7.75381	A1+	CACTA-like transposase family (Ptta/En/Spm)
AT5G30450	63.3663	-5.8329	0.42338	-13.777	3.51E-43	5.18E-41	0.60238	0	0	6.60587	6.95281	7.33447	A1-	CACTA-like transposase family (Ptta/En/Spm)
AT5G30450	8.09651	-0.6193	0.1429	-4.3342	1.46E-05	1.20E-03	0.95419	0	0	4.03478	4.34599	3.79855	A2+	CACTA-like transposase family (Ptta/En/Spm)
AT5G30480	23.9581	-4.273	0.48708	-8.7728	1.74E-18	7.42E-17	0	0	0	4.32498	5.61718	6.27841	A1-	CACTA-like transposase family (Ptta/En/Spm)
AT5G30480	8.86783	-0.6279	0.1407	-4.4625	8.10E-06	0.000709	0	0	0	4.12237	4.79992	3.45081	A2+	CACTA-like transposase family (Ptta/En/Spm)
AT5G32563	26.6796	-4.3558	0.56172	-7.7544	8.88E-15	2.90E-13	0	0	0	3.48023	6.24743	6.24686	A1+	CACTA-like transposase family (Ptta/En/Spm)
AT5G32566	17.127	-3.8918	0.49534	-7.8569	3.94E-15	1.32E-13	0	0	0	3.93374	5.26298	5.70271	A1-	CACTA-like transposase family (Ptta/En/Spm)
AT5G32566	217.374	-2.8129	0.59236	-4.7486	2.05E-06	2.33E-05	0	0	0	6.03001	9.36378	9.18751	A1+	CACTA-like transposase family (Ptta/En/Spm)
AT5G32566	24.1921	-1.4002	0.17311	-8.0882	6.06E-16	1.70E-13	0	0	0	5.39372	6.00855	5.38306	A2+	CACTA-like transposase family (Ptta/En/Spm)
AT5G34790	103.379	-2.5282	0.28777	-8.7855	1.56E-18	6.99E-17	4.76813	4.81692	4.88007	7.52259	7.92393	6.84128	A1+	CACTA-like transposase family (Ptta/En/Spm)
AT5G34790	104.56	-1.2482	0.16688	-7.4795	7.46E-14	1.70E-11	5.49384	5.55299	5.60285	7.55257	7.43362	7.04587	A2+	CACTA-like transposase family (Ptta/En/Spm)
AT5G36655	320.787	-6.9282	0.48666	-14.236	5.47E-46	1.11E-43	1.08201	0.62641	0	7.07436	9.91947	9.68455	A1+	CACTA-like transposase family (Ptta/En/Spm)
AT5G36655	8.65157	-2.5588	0.5058	-5.059	4.21E-07	6.15E-06	1.02605	0.58899	0	3.22203	4.7925	4.02897	A1-	CACTA-like transposase family (Ptta/En/Spm)
AT5G36655	125.546	-2.5244	0.18018	-14.01	1.35E-44	1.80E-41	1.52355	0.94033	0	7.22664	8.46496	7.96783	A2+	CACTA-like transposase family (Ptta/En/Spm)
AT2G13310	179.219	-6.9259	0.47809	-14.487	1.47E-47	3.47E-45	0.64015	0	0	7.03764	8.94239	8.82785	A1+	CACTA-like transposase family (Ptta/En/Spm),
AT2G13310	27.1257	-4.5374	0.46259	-9.8088	1.03E-22	5.75E-21	0.60238	0	0	5.80081	6.24407	5.07435	A1-	CACTA-like transposase family (Ptta/En/Spm),
AT2G13310	51.1175	-1.8391	0.17851	-10.302	6.87E-25	3.15E-22	0.95419	0	0	5.9672	7.16867	6.67711	A2+	CACTA-like transposase family (Ptta/En/Spm),
AT1G33570	97.2394	-2.7719	0.59233	-4.6797	2.87E-06	3.19E-05	0	0	0	4.89656	8.04777	8.18998	A1+	CACTA-like transposase family (Tnp1/En/Spm)
AT1G34530	29.1211	-4.9206	0.51452	-9.5635	1.14E-21	6.47E-20	0.64015	0	0	5.69485	6.42035	5.31183	A1+	CACTA-like transposase family (Tnp1/En/Spm)
AT1G34530	62.3922	-2.0058	0.17947	-11.176	5.35E-29	2.90E-26	0.95419	0	0	6.28451	7.47776	6.90945	A2+	CACTA-like transposase family (Tnp1/En/Spm)
AT1G35600	293.662	-5.6343	0.55272	-10.194	2.11E-24	1.49E-22	0	0	0	7.73242	9.55681	9.64233	A1+	CACTA-like transposase family (Tnp1/En/Spm)
AT1G35600	29.9206	-1.5729	0.17594	-8.9398	3.90E-19	1.36E-16	0	0	0	5.81776	6.31143	5.5453	A2+	CACTA-like transposase family (Tnp1/En/Spm)
AT1G36630	1211.52	-4.9248	0.56023	-8.7906	1.49E-18	6.72E-17	1.92313	3.63438	1.16147	10.5681	11.7754	11.1248	A1+	CACTA-like transposase family (Tnp1/En/Spm)
AT1G39190	6.07521	-2.7205	0.58564	-4.6454	3.39E-06	3.71E-05	0	0	0.69459	3.48023	4.16427	3.28443	A1+	CACTA-like transposase family (Tnp1/En/Spm)
AT1G39190	11.5728	-0.8087	0.15449	-5.2348	1.65E-07	1.87E-05	0	0	1.02486	4.7361	4.79992	4.07853	A2+	CACTA-like transposase family (Tnp1/En/Spm)
AT1G42410	56.5877	-5.9027	0.47897	-12.324	6.76E-35	8.43E-33	0	0	0.69459	6.96128	7.11744	6.29355	A1+	CACTA-like transposase family (Tnp1/En/Spm)
AT1G42410	130.647	-2.7073	0.17985	-15.053	3.32E-51	4.90E-48	0	0	1.02486	7.48807	8.55012	7.85474	A2+	CACTA-like transposase family (Tnp1/En/Spm)
AT1G42410	3.49308	-1.6712	0.50692	-3.2967	0.000978	0.007045	0	0	0.65347	2.19233	3.54662	2.83386	A1-	CACTA-like transposase family (Tnp1/En/Spm)
AT1G52850	350.503	-3.4327	0.58982	-5.8199	5.89E-09	9.83E-08	0.64015	0	0	7.03764	9.84175	10.045	A1+	CACTA-like transposase family (Tnp1/En/Spm)
AT1G52850	7.23309	-2.4722	0.51442	-4.8059	1.54E-06	2.05E-05	0.60238	0	0	2.79716	4.7925	3.48753	A1-	CACTA-like transposase family (Tnp1/En/Spm)
AT1G52850	96.3034	-2.3259	0.18022	-12.906	4.18E-38	3.58E-35	0.95419	0	0	6.86963	8.11975	7.52972	A2+	CACTA-like transposase family (Tnp1/En/Spm)
AT3G30836	252.469	-8.0261	0.44152	-18.178	7.65E-74	4.79E-71	0	0	0	9.05257	8.50691	9.28317	A1+	CACTA-like transposase family (Tnp1/En/Spm)
AT3G30836	229.964	-7.6069	0.39247	-19.382	1.10E-83	6.48E-81	0	0	0	8.83174	9.16154	8.46958	A1-	CACTA-like transposase family (Tnp1/En/Spm)
AT3G30836	8.49345	-0.6379	0.14161	-4.5048	6.64E-06	0.000591	0	0	0	3.8418	4.68412	3.79855	A2+	CACTA-like transposase family (Tnp1/En/Spm)
AT3G32950	15.4903	-4.2187	0.55489	-7.6029	2.90E-14	9.05E-13	0	0	0	4.16454	5.47681	5.06504	A1+	CACTA-like transposase family (Tnp1/En/Spm)
AT3G42650	12.2885	-3.4404	0.58231	-5.9083	3.46E-09	5.96E-08	0	0	0	5.54593	4.50885	2.85705	A1+	CACTA-like transposase family (Tnp1/En/Spm)
AT3G42650	103.401	-2.2928	0.18009	-12.732	3.95E-37	3.19E-34	0	0	0	6.76464	7.90377	8.10429	A2+	CACTA-like transposase family (Tnp1/En/Spm)

AT3G42650	4.23186	-1.8226	0.5019	-3.6313	0.000282	0.002357	0	0	0	1.13103	4.08983	3.19741	A1-	CACTA-like transposase family (Tnp1/En/Spm)
AT5G28524	8.84681	-3.4628	0.57752	-5.996	2.02E-09	3.65E-08	0	0	0	3.48023	4.6545	4.30275	A1+	CACTA-like transposase family (Tnp1/En/Spm)
AT1G35590	5.90501	-2.9239	0.58914	-4.963	6.94E-07	8.54E-06	0	0	0	4.16454	3.04536	3.61372	A1+	CACTA-like transposase family (Tnp2/En/Spm)
AT1G35590	33.3471	-1.8227	0.17893	-10.186	2.28E-24	1.03E-21	0	0	0	6.12399	6.12416	5.99066	A2+	CACTA-like transposase family (Tnp2/En/Spm)
AT1G36200	10.4535	-3.6028	0.57453	-6.2709	3.59E-10	7.12E-09	0	0	0	4.6268	4.90796	3.45845	A1+	CACTA-like transposase family (Tnp2/En/Spm)
AT1G36200	69.3138	-2.0571	0.17948	-11.462	2.06E-30	1.27E-27	0	0	0	6.2654	7.34107	7.49041	A2+	CACTA-like transposase family (Tnp2/En/Spm)
AT1G36470	7.63756	-3.3112	0.5811	-5.6982	1.21E-08	1.93E-07	0	0	0	3.48023	4.16427	4.30275	A1+	CACTA-like transposase family (Tnp2/En/Spm)
AT1G36470	23.5581	-1.4629	0.1746	-8.3787	5.35E-17	1.62E-14	0	0	0	5.46248	5.716	5.57567	A2+	CACTA-like transposase family (Tnp2/En/Spm)
AT1G49070	66.0007	-3.7389	0.58514	-6.3898	1.66E-10	3.46E-09	0	0	0	5.05192	7.49161	7.53808	A1+	CACTA-like transposase family (Tnp2/En/Spm)
AT1G49070	16.8446	-1.1569	0.16733	-6.9138	4.72E-12	8.42E-10	0	0	0	5.28403	5.1002	4.94502	A2+	CACTA-like transposase family (Tnp2/En/Spm)
AT1G50860	6.54917	-3.0636	0.58685	-5.2204	1.79E-07	2.42E-06	0	0	0	3.24283	3.71068	4.30275	A1+	CACTA-like transposase family (Tnp2/En/Spm)
AT1G50860	18.2418	-1.2284	0.16942	-7.2507	4.15E-13	8.29E-11	0	0	0	5.12345	5.38617	5.1607	A2+	CACTA-like transposase family (Tnp2/En/Spm)
AT2G04770	526.66	-3.9904	0.58424	-6.83	8.49E-12	2.08E-10	0	0	0	7.9456	10.5582	10.4601	A1+	CACTA-like transposase family (Tnp2/En/Spm)
AT2G04770	67.7265	-2.2328	0.18018	-12.392	2.88E-35	2.19E-32	0	0	0	7.36775	7.29246	6.45005	A2+	CACTA-like transposase family (Tnp2/En/Spm)
AT2G04770	3.53978	-1.8299	0.50246	-3.6418	0.000271	0.002275	0	0	0	1.75715	3.54662	3.19741	A1-	CACTA-like transposase family (Tnp2/En/Spm)
AT2G06740	15.9945	-4.4299	0.54468	-8.133	4.19E-16	1.54E-14	0	0	0	5.19216	4.6545	5.21812	A1+	CACTA-like transposase family (Tnp2/En/Spm)
AT2G12210	10.6865	-3.1684	0.50957	-6.2179	5.04E-10	1.06E-08	0	0	0	3.22203	5.26298	4.2772	A1-	CACTA-like transposase family (Tnp2/En/Spm)
AT2G12260	8.21525	-3.4471	0.57742	-5.9698	2.38E-09	4.23E-08	0	0	0	3.86258	3.95523	4.4747	A1+	CACTA-like transposase family (Tnp2/En/Spm)
AT2G12260	25.4633	-1.5404	0.17586	-8.7594	1.96E-18	6.61E-16	0	0	0	5.70877	5.80184	5.57567	A2+	CACTA-like transposase family (Tnp2/En/Spm)
AT2G12980	13.2617	-4.0061	0.56166	-7.1326	9.85E-13	2.68E-11	0	0	0	5.19216	4.90796	3.99899	A1+	CACTA-like transposase family (Tnp2/En/Spm)
AT2G12980	30.1219	-1.5572	0.17565	-8.8655	7.62E-19	2.60E-16	0	0	0	5.39372	6.23118	6.05674	A2+	CACTA-like transposase family (Tnp2/En/Spm)
AT2G13000	11.7912	-3.27	0.58607	-5.5796	2.41E-08	3.69E-07	0	0	0	5.19216	5.01973	2.24707	A1+	CACTA-like transposase family (Tnp2/En/Spm)
AT2G13000	26.0706	-1.2404	0.16858	-7.3579	1.87E-13	4.01E-11	0	0	0	4.7361	5.95958	6.14039	A2+	CACTA-like transposase family (Tnp2/En/Spm)
AT3G29648	128.172	-6.7696	0.46857	-14.447	2.61E-47	5.87E-45	0	0.62641	0	8.67625	7.4119	7.58576	A1+	CACTA-like transposase family (Tnp2/En/Spm)
AT3G29648	20.2712	-4.0123	0.48078	-8.3454	7.09E-17	2.74E-15	0	0.58899	0	4.4082	6.02485	5.23951	A1-	CACTA-like transposase family (Tnp2/En/Spm)
AT3G29732	8.65861	-2.7435	0.57308	-4.7873	1.69E-06	1.94E-05	0	0	1.51363	3.68404	3.95523	4.62832	A1+	CACTA-like transposase family (Tnp2/En/Spm)
AT3G29732	25.4809	-1.3919	0.17505	-7.9512	1.85E-15	4.96E-13	0	0	2.03713	5.46248	5.88287	5.63456	A2+	CACTA-like transposase family (Tnp2/En/Spm)
AT3G30393	57.6264	-5.5317	0.49244	-11.233	2.80E-29	2.54E-27	0.64015	0.62641	0	6.1029	6.46052	7.57635	A1+	CACTA-like transposase family (Tnp2/En/Spm)
AT3G30393	91.6876	-2.8931	0.17748	-16.301	9.77E-60	1.86E-56	0.95419	0.94033	0	7.59911	7.61057	7.3393	A2+	CACTA-like transposase family (Tnp2/En/Spm)
AT3G30837	164.358	-7.2635	0.42542	-17.074	2.32E-65	1.17E-62	0	1.06174	0	8.47126	7.99404	8.56307	A1+	CACTA-like transposase family (Tnp2/En/Spm)
AT3G30837	152.964	-6.8746	0.38651	-17.786	9.03E-71	3.51E-68	0	1.00603	0	8.08933	8.61108	8.0023	A1-	CACTA-like transposase family (Tnp2/En/Spm)
AT3G30837	9.29671	-0.6405	0.14623	-4.3803	1.18E-05	1.00E-03	0	1.50483	0	4.28307	4.55821	3.79855	A2+	CACTA-like transposase family (Tnp2/En/Spm)
AT3G33151	39.0683	-4.8783	0.49953	-9.7658	1.58E-22	9.75E-21	1.08201	0.62641	0	6.92153	6.20076	5.3565	A1+	CACTA-like transposase family (Tnp2/En/Spm)
AT3G33151	19.1148	-3.2534	0.49588	-6.5609	5.35E-11	1.26E-09	1.02605	0.58899	0	4.14258	6.34234	4.11654	A1-	CACTA-like transposase family (Tnp2/En/Spm)
AT3G33151	7.94482	-1.9527	0.45461	-4.2953	1.74E-05	0.000257	0.97697	0.55795	0	5.09826	2.57549	3.1719	A2-	CACTA-like transposase family (Tnp2/En/Spm)
AT3G42721	7.18764	-3.2714	0.58181	-5.6228	1.88E-08	2.92E-07	0	0	0	3.86258	3.71068	4.20843	A1+	CACTA-like transposase family (Tnp2/En/Spm)
AT3G42721	15.3004	-1.0864	0.16507	-6.5816	4.65E-11	7.64E-09	0	0	0	5.08035	5.00689	4.84882	A2+	CACTA-like transposase family (Tnp2/En/Spm)
AT3G43128	16.2857	-3.9958	0.56704	-7.0467	1.83E-12	4.83E-11	0	0	0	5.95323	4.50885	3.99899	A1+	CACTA-like transposase family (Tnp2/En/Spm)
AT3G43128	15.4037	-3.9669	0.48841	-8.1221	4.58E-16	1.66E-14	0	0	0	4.76464	5.45092	4.61457	A1-	CACTA-like transposase family (Tnp2/En/Spm)
AT3G43128	62.5633	-1.7269	0.17656	-9.7805	1.37E-22	5.67E-20	0	0	0	5.79128	7.33148	7.3393	A2+	CACTA-like transposase family (Tnp2/En/Spm)
AT4G03900	7.17437	-3.2801	0.58131	-5.6426	1.68E-08	2.62E-07	0	0	0	3.86258	3.95523	3.99899	A1+	CACTA-like transposase family (Tnp2/En/Spm)
AT4G03900	22.3507	-1.4212	0.17387	-8.1739	2.99E-16	8.73E-14	0	0	0	5.49567	5.59296	5.45016	A2+	CACTA-like transposase family (Tnp2/En/Spm)
AT4G04270	15.1132	-3.6025	0.50125	-7.187	6.62E-13	1.84E-11	0	0	0	4.70005	5.76624	3.72897	A1-	CACTA-like transposase family (Tnp2/En/Spm)
AT4G06588	9.39818	-2.9193	0.50076	-5.8297	5.55E-09	1.04E-07	0.60238	0	0.65347	3.93374	4.7925	3.93573	A1-	CACTA-like transposase family (Tnp2/En/Spm)
AT4G06650	9.50491	-3.3783	0.58137	-5.8109	6.21E-09	1.03E-07	0	0	0	2.9585	4.90796	4.4747	A1+	CACTA-like transposase family (Tnp2/En/Spm)
AT4G08016	88.1914	-3.6799	0.58529	-6.2873	3.23E-10	6.44E-09	0	0	0.69459	5.54593	7.4119	8.29699	A1+	CACTA-like transposase family (Tnp2/En/Spm)
AT4G08016	14.0122	-0.9127	0.15927	-5.7308	1.00E-08	1.28E-06	0	0	1.02486	4.99008	5.14469	4.23894	A2+	CACTA-like transposase family (Tnp2/En/Spm)
AT4G08092	5.68992	-2.8442	0.5901	-4.8198	1.44E-06	1.67E-05	0	0	0	3.24283	4.16427	3.28443	A1+	CACTA-like transposase family (Tnp2/En/Spm)
AT4G08092	7.22692	-0.5819	0.13738	-4.2357	2.28E-05	0.001793	0	0	0	4.28307	3.90354	3.57627	A2+	CACTA-like transposase family (Tnp2/En/Spm)
AT5G28165	345.548	-6.6242	0.50648	-13.079	4.35E-39	6.81E-37	0.64015	0.62641	0.69459	6.44887	9.98291	9.92966	A1+	CACTA-like transposase family (Tnp2/En/Spm)
AT5G28165	9.35887	-2.8229	0.49842	-5.6636	1.48E-08	2.64E-07	0.60238	0.58899	0.65347	3.54983	4.48353	4.55311	A1-	CACTA-like transposase family (Tnp2/En/Spm)
AT5G28165	50.1887	-2.0925	0.18024	-11.609	3.70E-31	2.40E-28	0.95419	0.94033	1.02486	6.6514	6.90979	6.33048	A2+	CACTA-like transposase family (Tnp2/En/Spm)
AT5G28926	5.32664	-2.7704	0.59113	-4.6866	2.78E-06	3.10E-05	0	0	0	3.24283	3.04536	4.10751	A1+	CACTA-like transposase family (Tnp2/En/Spm)
AT5G28926	22.1881	-1.3741	0.17278	-7.9529	1.82E-15	4.94E-13	0	0	0	5.7368	5.49326	5.23869	A2+	CACTA-like transposase family (Tnp2/En/Spm)
AT5G32825	22.4894	-2.8876	0.58997	-4.8945	9.86E-07	1.18E-05	0.64015	0	0.69459	0	6.20076	5.95894	A1+	CACTA-like transposase family (Tnp2/En/Spm)
AT5G45085	5.7702	-2.8323	0.59063	-4.7954	1.62E-06	1.87E-05	0	0	0	3.86258	2.54524	4.10751	A1+	CACTA-like transposase family (Tnp2/En/Spm)
AT5G45085	17.9297	-1.1808	0.16793	-7.0317	2.04E-12	3.82E-10	0	0	0	5.35808	5.34862	4.84882	A2+	CACTA-like transposase family (Tnp2/En/Spm)

AT1G40075	19.8433	-3.3937	0.57647	-5.8871	3.93E-09	6.72E-08	0.64015	0.62641	0	2.1331	5.62557	6.0704	A1+	CACTA-like transposase family, putative
AT2G06790	8.09438	-3.4209	0.57828	-5.9157	3.31E-09	5.73E-08	0	0	0	4.02145	3.71068	4.4747	A1+	CACTA-like transposase family, putative
AT2G06790	21.7975	-1.3979	0.17341	-8.0609	7.58E-16	2.10E-13	0	0	0	5.55983	5.42276	5.45016	A2+	CACTA-like transposase family, putative
AT2G10480	6.72445	-3.156	0.58461	-5.3985	6.72E-08	9.68E-07	0	0	0	4.16454	3.41604	3.88164	A1+	CACTA-like transposase family, putative
AT2G10480	21.5469	-1.359	0.17251	-7.878	3.33E-15	8.76E-13	0	0	0	5.39372	5.68621	5.27616	A2+	CACTA-like transposase family, putative
AT5G30470	14.7057	-2.9993	0.51197	-5.8582	4.68E-09	8.94E-08	0	0.58899	0	2.19233	5.04684	5.73073	A1-	CACTA-like transposase family, putative
AT5G32103	17.6444	-3.0992	0.58358	-5.3107	1.09E-07	1.53E-06	0	0.62641	0.69459	1.42937	5.88373	5.5225	A1+	CACTA-like transposase family, putative
AT5G32103	8.79518	-0.6038	0.14383	-4.1982	2.69E-05	2.10E-03	0	0.94033	1.02486	4.49483	4.34599	3.45081	A2+	CACTA-like transposase family, putative
AT1G30180	10.7074	2.95024	0.57539	5.1274	2.94E-07	3.84E-06	4.05639	4.89808	4.37779	0	0	0	A1+	copia-like retrotransposon family (Ty1_Copia-element)
AT1G34967	12.6885	-3.3934	0.57453	-5.9065	3.50E-09	6.02E-08	0.64015	0	0	3.24283	5.62557	4.30275	A1+	copia-like retrotransposon family (Ty1_Copia-element)
AT1G47650	26.0544	-3.9836	0.57454	-6.9335	4.11E-12	1.04E-10	0	0	0	2.60404	6.20076	6.31635	A1+	copia-like retrotransposon family (Ty1_Copia-element)
AT2G06930	15.4598	-3.9568	0.48899	-8.0917	5.88E-16	2.11E-14	0	0	0	4.88574	5.45092	4.48892	A1-	copia-like retrotransposon family (Ty1_Copia-element)
AT2G07400	106.371	-6.7209	0.4072	-16.505	3.37E-61	8.58E-59	0	0	0	7.63346	7.9154	7.65278	A1-	copia-like retrotransposon family (Ty1_Copia-element)
AT2G07400	10.9438	-0.7454	0.14865	-5.0143	5.32E-07	5.73E-05	0	0	0	4.42764	5.0543	3.79855	A2+	copia-like retrotransposon family (Ty1_Copia-element)
AT2G07550	16.4592	-3.1826	0.47151	-6.7498	1.48E-11	3.67E-10	1.02605	0.58899	1.71887	5.05024	3.54662	5.73073	A1-	copia-like retrotransposon family (Ty1_Copia-element)
AT2G09830	38.1889	-5.1583	0.43975	-11.73	8.93E-32	7.91E-30	0	0.58899	0	6.31378	6.52082	5.91301	A1-	copia-like retrotransposon family (Ty1_Copia-element)
AT2G11950	12.4214	-3.931	0.56345	-6.9768	3.02E-12	7.75E-11	0	0	0	4.52436	5.22024	4.10751	A1+	copia-like retrotransposon family (Ty1_Copia-element)
AT2G11950	12.1593	-3.5418	0.50053	-7.0762	1.48E-12	4.01E-11	0	0	0	4.4082	5.26298	4.02897	A1-	copia-like retrotransposon family (Ty1_Copia-element)
AT2G13930	7.1136	-3.2349	0.58289	-5.5497	2.86E-08	4.34E-07	0	0	0	3.68404	3.71068	4.30275	A1+	copia-like retrotransposon family (Ty1_Copia-element)
AT2G16000	43.5404	-5.6492	0.4816	-11.73	8.95E-32	9.59E-30	0	0	0.69459	6.53041	6.61089	6.19861	A1+	copia-like retrotransposon family (Ty1_Copia-element)
AT2G16000	8.38662	-2.9962	0.50797	-5.8985	3.67E-09	7.10E-08	0	0	0.65347	4.70005	2.66509	4.35128	A1-	copia-like retrotransposon family (Ty1_Copia-element)
AT2G16670	46.2901	-5.0249	0.51719	-9.7158	2.58E-22	1.56E-20	0	1.06174	0	7.39777	6.05094	5.48276	A1+	copia-like retrotransposon family (Ty1_Copia-element)
AT2G16670	11.8898	-3.1968	0.49574	-6.4486	1.13E-10	2.53E-09	0	1.00603	0	4.70005	5.04684	3.83605	A1-	copia-like retrotransposon family (Ty1_Copia-element)
AT2G19840	59.4956	-5.0627	0.39052	-12.964	1.95E-38	2.32E-36	1.02605	1.00603	1.44299	6.7564	7.33342	6.43991	A1-	copia-like retrotransposon family (Ty1_Copia-element)
AT2G19840	25.1252	-1.3123	0.17606	-7.4536	9.09E-14	2.03E-11	1.52355	1.50483	2.03713	5.49567	5.9089	5.38306	A2+	copia-like retrotransposon family (Ty1_Copia-element)
AT2G21460	28.7249	-3.9465	0.44643	-8.8401	9.56E-19	4.15E-17	1.02605	1.593	0.65347	5.41464	5.04684	6.58514	A1-	copia-like retrotransposon family (Ty1_Copia-element)
AT3G27327	12.7428	-4.0718	0.55774	-7.3006	2.86E-13	8.12E-12	0	0	0	4.29471	4.78679	5.01018	A1+	copia-like retrotransposon family (Ty1_Copia-element)
AT3G27327	2.76168	-1.7043	0.49697	-3.4293	0.000605	0.00465	0	0	0	3.02518	2.66509	2.34668	A1-	copia-like retrotransposon family (Ty1_Copia-element)
AT3G29156	53.4349	3.74835	0.4499	8.33153	7.98E-17	3.11E-15	6.20171	6.95914	6.83506	3.48023	0	1.16932	A1+	copia-like retrotransposon family (Ty1_Copia-element)
AT3G30582	26.6228	-3.4723	0.46145	-7.5248	5.28E-14	1.61E-12	1.02605	1.81597	0.65347	4.32498	6.43434	5.70271	A1-	copia-like retrotransposon family (Ty1_Copia-element)
AT3G43867	164.242	-7.11	0.38966	-18.247	2.20E-74	9.74E-72	0	0	0.65347	8.31637	8.63065	8.09146	A1-	copia-like retrotransposon family (Ty1_Copia-element)
AT3G43867	129.458	-7.0214	0.44808	-15.67	2.43E-55	8.00E-53	0	0	0.69459	8.31127	8.08679	7.56688	A1+	copia-like retrotransposon family (Ty1_Copia-element)
AT3G43867	13.9003	-0.8457	0.15575	-5.4299	5.64E-08	6.61E-06	0	0	1.02486	4.7361	5.38617	4.07853	A2+	copia-like retrotransposon family (Ty1_Copia-element)
AT3G44215	125.366	-4.0208	0.57948	-6.9387	3.96E-12	1.00E-10	0	0.62641	0.69459	9.02	6.61089	7.09959	A1+	copia-like retrotransposon family (Ty1_Copia-element)
AT3G44215	8.08669	-2.8448	0.50465	-5.6371	1.73E-08	3.05E-07	0	0.58899	0.65347	3.93374	3.54662	4.55311	A1-	copia-like retrotransposon family (Ty1_Copia-element)
AT3G44215	18.7679	-1.1822	0.17008	-6.9511	3.62E-12	6.60E-10	0	0.94033	1.02486	5.08035	5.49326	5.12007	A2+	copia-like retrotransposon family (Ty1_Copia-element)
AT3G44325	30.6598	-5.1163	0.50304	-10.171	2.68E-24	1.87E-22	0.64015	0	0	6.3018	5.88373	5.59885	A1+	copia-like retrotransposon family (Ty1_Copia-element)
AT3G45775	12.0999	-2.5677	0.59115	-4.3436	1.40E-05	1.35E-04	0	0.62641	0.69459	5.91325	3.41604	1.80658	A1+	copia-like retrotransposon family (Ty1_Copia-element)
AT4G04426	10.0131	-3.4678	0.56558	-6.1314	8.71E-10	1.64E-08	0	0.62641	0	4.89656	3.95523	4.10751	A1+	copia-like retrotransposon family (Ty1_Copia-element)
AT4G05592	22.8707	-4.4774	0.47504	-9.4252	4.29E-21	2.16E-19	0	0	0	4.82646	5.90133	5.70271	A1-	copia-like retrotransposon family (Ty1_Copia-element)
AT4G05592	10.9513	-2.9303	0.59121	-4.9565	7.18E-07	8.80E-06	0	0	0	5.69485	1.77337	3.75389	A1+	copia-like retrotransposon family (Ty1_Copia-element)
AT4G06682	10.5484	-3.3035	0.49814	-6.6316	3.32E-11	7.96E-10	0	0.58899	0	4.14258	4.7925	4.35128	A1-	copia-like retrotransposon family (Ty1_Copia-element)
AT4G06684	7.77598	-3.0971	0.50987	-6.0743	1.25E-09	2.54E-08	0	0	0	3.93374	4.08983	4.11654	A1-	copia-like retrotransposon family (Ty1_Copia-element)
AT4G06684	3.49352	-2.051	0.58661	-3.4964	4.72E-04	3.16E-03	0	0	0	3.86258	1.77337	2.58406	A1+	copia-like retrotransposon family (Ty1_Copia-element)
AT4G07810	15.6289	-3.3328	0.58098	-5.7365	9.67E-09	1.56E-07	0.64015	0	0	2.1331	5.39625	5.63557	A1+	copia-like retrotransposon family (Ty1_Copia-element)
AT4G09316	16.0714	-3.3022	0.47616	-6.935	4.06E-12	1.06E-10	0.60238	0.58899	1.10158	4.48688	5.76624	4.35128	A1-	copia-like retrotransposon family (Ty1_Copia-element)
AT4G09425	32.0208	-3.6395	0.38508	-9.4513	3.35E-21	1.70E-19	1.35313	2.33157	1.95037	5.73786	5.90133	6.20038	A1-	copia-like retrotransposon family (Ty1_Copia-element)
AT4G18420	16.6186	-3.7673	0.57566	-6.5444	5.97E-11	1.32E-09	0	0	0	2.9585	5.94164	5.06504	A1+	copia-like retrotransposon family (Ty1_Copia-element)
AT5G17125	21.3348	-3.935	0.57334	-6.8633	6.73E-12	1.67E-10	0	0	0	3.24283	6.46052	5.06504	A1+	copia-like retrotransposon family (Ty1_Copia-element)
AT5G19097	137.669	-4.6675	0.54898	-8.5021	1.86E-17	7.71E-16	1.41979	1.3957	0.69459	3.68404	8.61765	8.71231	A1+	copia-like retrotransposon family (Ty1_Copia-element)
AT5G19097	5.59115	-1.6596	0.51019	-3.253	0.001142	0.00806	1.35313	1.32917	0.65347	3.81676	3.54662	2.83386	A1-	copia-like retrotransposon family (Ty1_Copia-element)
AT5G19097	13.4874	-0.6468	0.15318	-4.2228	2.41E-05	1.89E-03	1.93072	1.90954	1.02486	4.20496	5.45844	3.89804	A2+	copia-like retrotransposon family (Ty1_Copia-element)
AT5G32702	6.70894	-2.5096	0.51396	-4.8829	1.05E-06	1.44E-05	0	0.58899	0	3.02518	4.48353	3.61329	A1-	copia-like retrotransposon family (Ty1_Copia-element)
AT5G35052	94.5319	-6.5075	0.41402	-15.718	1.14E-55	2.52E-53	0	0	0	7.44924	7.88311	7.31609	A1-	copia-like retrotransposon family (Ty1_Copia-element)
AT5G35052	12.9635	-0.8781	0.15602	-5.6278	1.82E-08	2.25E-06	0	0	0	5.03592	4.95786	4.07853	A2+	copia-like retrotransposon family (Ty1_Copia-element)
AT1G36540	10.5938	-3.8343	0.56528	-6.7829	1.18E-11	2.83E-10	0	0	0	4.72243	4.16427	4.4747	A1+	gypsy-like retrotransposon family (Athila)
AT1G36540	13.198	-3.8176	0.4925	-7.7516	9.08E-15	2.95E-13	0	0	0	4.56149	5.04684	4.67351	A1-	gypsy-like retrotransposon family (Athila)

AT1G36795	8.12752	-3.0856	0.50993	-6.051	1.44E-09	2.92E-08	0	0	0	3.81676	4.48353	3.93573	A1-	gypsy-like retrotransposon family (Athila)
AT1G36795	4.20603	-1.9801	0.58411	-3.39	6.99E-04	4.44E-03	0	0	0	4.4141	1.77337	1.80658	A1+	gypsy-like retrotransposon family (Athila)
AT1G37160	34.4386	-4.9534	0.54032	-9.1674	4.85E-20	2.45E-18	0	0	0	7.03764	5.22024	5.3565	A1+	gypsy-like retrotransposon family (Athila)
AT1G37160	24.0701	-4.627	0.46795	-9.8879	4.70E-23	2.66E-21	0	0	0	5.37382	6.02485	5.35204	A1-	gypsy-like retrotransposon family (Athila)
AT1G37340	40.7492	-5.778	0.49489	-11.675	1.71E-31	1.79E-29	0	0	0	6.42063	6.29264	6.38264	A1+	gypsy-like retrotransposon family (Athila)
AT1G37340	18.7381	-1.138	0.16627	-6.8445	7.67E-12	1.36E-09	0	0	0	4.6205	5.52727	5.48258	A2+	gypsy-like retrotransposon family (Athila)
AT1G38167	17.4171	-4.2112	0.48068	-8.7608	1.94E-18	8.23E-17	0	0	0	5.05024	5.45092	4.93675	A1-	gypsy-like retrotransposon family (Athila)
AT1G38167	13.2894	-3.4829	0.58206	-5.9838	2.18E-09	3.91E-08	0	0	0	5.87214	3.41604	3.75389	A1+	gypsy-like retrotransposon family (Athila)
AT1G38185	67.4166	-5.8426	0.42225	-13.837	1.53E-43	2.27E-41	0	0	0.65347	6.80327	7.55453	6.74483	A1-	gypsy-like retrotransposon family (Athila)
AT1G38185	50.7526	-5.3454	0.51532	-10.373	3.29E-25	2.41E-23	0	0	0.69459	7.53581	5.82339	6.01574	A1+	gypsy-like retrotransposon family (Athila)
AT1G38260	42.3242	-4.171	0.50569	-8.2483	1.61E-16	6.15E-15	1.92313	1.06174	0.69459	7.27696	5.99732	5.06504	A1+	gypsy-like retrotransposon family (Athila)
AT1G38260	17.9875	-0.961	0.169	-5.6861	1.30E-08	1.63E-06	2.50778	1.50483	1.02486	5.24553	5.27047	4.74575	A2+	gypsy-like retrotransposon family (Athila)
AT1G38330	42.3242	-4.171	0.50569	-8.2483	1.61E-16	6.15E-15	1.92313	1.06174	0.69459	7.27696	5.99732	5.06504	A1+	gypsy-like retrotransposon family (Athila)
AT1G38330	17.9875	-0.961	0.169	-5.6861	1.30E-08	1.63E-06	2.50778	1.50483	1.02486	5.24553	5.27047	4.74575	A2+	gypsy-like retrotransposon family (Athila)
AT1G38360	63.9099	-5.846	0.47157	-12.397	2.72E-35	3.52E-33	0.64015	0	0.69459	7.5619	6.53766	6.69242	A1+	gypsy-like retrotransposon family (Athila)
AT1G38360	53.55	-5.4947	0.41783	-13.151	1.69E-39	2.10E-37	0.60238	0	0.65347	6.64071	7.07217	6.47347	A1-	gypsy-like retrotransposon family (Athila)
AT1G38430	38.4637	-4.5706	0.49395	-9.2532	2.18E-20	1.13E-18	1.08201	1.3957	0	6.88065	6.24743	5.21812	A1+	gypsy-like retrotransposon family (Athila)
AT1G38430	13.2543	-0.7508	0.15699	-4.7824	1.73E-06	1.70E-04	1.52355	1.90954	0	4.35716	5.22975	4.31292	A2+	gypsy-like retrotransposon family (Athila)
AT1G38450	83.1514	-5.8538	0.43338	-13.507	1.41E-41	1.93E-39	0.60238	0	0	6.60587	8.0668	7.08593	A1-	gypsy-like retrotransposon family (Athila)
AT1G38450	10.6829	-0.7648	0.15205	-5.0301	4.90E-07	5.32E-05	0.95419	0	0	4.67946	4.62254	3.99111	A2+	gypsy-like retrotransposon family (Athila)
AT1G38460	10.1787	-2.8646	0.59164	-4.8418	1.29E-06	1.51E-05	0	0	0	5.5973	1.77337	3.61372	A1+	gypsy-like retrotransposon family (Athila)
AT1G39830	30.6205	-5.2752	0.51614	-10.22	1.61E-24	1.14E-22	0	0	0	6.39182	5.69457	5.67137	A1+	gypsy-like retrotransposon family (Athila)
AT1G39830	14.0871	-1.0027	0.16185	-6.1951	5.82E-10	8.29E-08	0	0	0	4.55904	5.00689	4.99081	A2+	gypsy-like retrotransposon family (Athila)
AT1G39990	7.41733	-2.7959	0.59163	-4.7257	2.29E-06	2.58E-05	0	0	0	5.05192	2.54524	3.08651	A1+	gypsy-like retrotransposon family (Athila)
AT1G40074	29.8143	-5.2207	0.51872	-10.065	7.93E-24	5.38E-22	0	0	0	6.39182	5.55311	5.67137	A1+	gypsy-like retrotransposon family (Athila)
AT1G40074	14.0871	-1.0027	0.16185	-6.1951	5.82E-10	8.29E-08	0	0	0	4.55904	5.00689	4.99081	A2+	gypsy-like retrotransposon family (Athila)
AT1G40077	32.5651	-4.9688	0.53729	-9.2479	2.29E-20	1.18E-18	0	0	0	4.6268	6.61089	6.24686	A1+	gypsy-like retrotransposon family (Athila)
AT1G40077	8.22886	-3.1746	0.50857	-6.2423	4.31E-10	9.17E-09	0	0	0	4.32498	4.08983	3.93573	A1-	gypsy-like retrotransposon family (Athila)
AT1G40101	179.932	-7.2186	0.40306	-17.91	9.94E-72	4.05E-69	0	0	0	8.051	8.94405	8.34367	A1-	gypsy-like retrotransposon family (Athila)
AT1G40101	13.033	-0.8324	0.15348	-5.4239	5.83E-08	6.80E-06	0	0	0	4.35716	5.38617	4.23894	A2+	gypsy-like retrotransposon family (Athila)
AT1G41775	35.9858	-5.0787	0.45619	-11.133	8.68E-29	6.84E-27	0	0	0	5.83129	6.75292	5.75822	A1-	gypsy-like retrotransposon family (Athila)
AT1G41775	25.1001	-4.5271	0.55285	-8.1886	2.64E-16	9.97E-15	0	0	0	6.60759	4.78679	4.83185	A1+	gypsy-like retrotransposon family (Athila)
AT1G42370	31.9321	-5.049	0.45466	-11.105	1.18E-28	9.19E-27	0	0	0	5.70532	6.34234	5.93726	A1-	gypsy-like retrotransposon family (Athila)
AT1G43060	36.7038	-3.2313	0.52525	-6.1521	7.65E-10	1.45E-08	2.45099	2.09165	1.16147	3.68404	6.49961	6.79335	A1+	gypsy-like retrotransposon family (Athila)
AT2G06885	12.1754	-2.6588	0.50783	-5.2356	1.64E-07	2.56E-06	0	0.58899	1.10158	2.79716	5.61718	4.19911	A1-	gypsy-like retrotransposon family (Athila)
AT2G10180	326.935	-7.8193	0.38519	-20.3	1.28E-91	9.99E-89	0	0	0.65347	9.07718	9.82003	9.02234	A1-	gypsy-like retrotransposon family (Athila)
AT2G10180	19.7742	-1.2669	0.17137	-7.3932	1.43E-13	3.15E-11	0	0	1.02486	5.46248	5.34862	5.1607	A2+	gypsy-like retrotransposon family (Athila)
AT2G10190	42.9982	-5.5436	0.49111	-11.288	1.51E-29	1.39E-27	0	0.62641	0	6.90123	6.10263	6.17386	A1+	gypsy-like retrotransposon family (Athila)
AT2G10190	40.4227	-5.1852	0.44048	-11.772	5.45E-32	4.86E-30	0	0.58899	0	6.39763	6.67963	5.86323	A1-	gypsy-like retrotransposon family (Athila)
AT2G10250	16.3385	-2.569	0.47689	-5.3869	7.17E-08	1.03E-06	1.41979	2.41948	2.03285	4.4141	5.39625	4.83185	A1+	gypsy-like retrotransposon family (Athila)
AT2G10250	13.6474	-2.346	0.44433	-5.2799	1.29E-07	2.04E-06	1.35313	2.33157	1.95037	4.88574	4.48353	4.61457	A1-	gypsy-like retrotransposon family (Athila)
AT2G10250	15.7482	-0.6889	0.16835	-4.092	4.28E-05	0.003241	1.93072	3.06207	2.62616	4.55904	4.74318	4.99081	A2+	gypsy-like retrotransposon family (Athila)
AT2G10280	65.3167	-5.569	0.45322	-12.288	1.06E-34	1.07E-32	0	0	0	7.39926	7.42597	5.75822	A1-	gypsy-like retrotransposon family (Athila)
AT2G10280	6.53726	-0.5235	0.13228	-3.9577	7.57E-05	5.43E-03	0	0	0	3.20292	4.09708	3.99111	A2+	gypsy-like retrotransposon family (Athila)
AT2G10310	136.806	-6.9488	0.4053	-17.145	6.89E-66	2.16E-63	0	0	0	7.95739	8.46604	7.79229	A1-	gypsy-like retrotransposon family (Athila)
AT2G10310	10.3771	-0.7628	0.14994	-5.0872	3.63E-07	4.01E-05	0	0	0	4.55904	4.74318	3.89804	A2+	gypsy-like retrotransposon family (Athila)
AT2G10660	67.5742	-5.6549	0.43503	-12.999	1.24E-38	1.50E-36	0	0.58899	0	6.69143	7.74622	6.48996	A1-	gypsy-like retrotransposon family (Athila)
AT2G11050	21.3941	-4.2622	0.50778	-8.3939	4.70E-17	1.88E-15	0.64015	0.62641	0.69459	5.87214	4.78679	5.44189	A1+	gypsy-like retrotransposon family (Athila)
AT2G11050	10.5007	-0.7234	0.1538	-4.7036	2.56E-06	2.44E-04	0.95419	0.94033	1.02486	4.20496	4.74318	4.16097	A2+	gypsy-like retrotransposon family (Athila)
AT2G11100	5.94992	-2.7273	0.51453	-5.3007	1.15E-07	1.84E-06	0	0	0	3.54983	3.54662	3.93573	A1-	gypsy-like retrotransposon family (Athila)
AT2G11120	40.9294	-5.1786	0.44463	-11.647	2.38E-31	2.08E-29	0	0.58899	0	6.8337	6.24407	5.86323	A1-	gypsy-like retrotransposon family (Athila)
AT2G11120	10.5622	-0.6967	0.14748	-4.7242	2.31E-06	2.21E-04	0	0.94033	0	3.8418	5.0543	4.16097	A2+	gypsy-like retrotransposon family (Athila)
AT2G11340	22.4533	-4.3416	0.55879	-7.7697	7.87E-15	2.59E-13	0	0	0	6.47656	4.6545	4.55355	A1+	gypsy-like retrotransposon family (Athila)
AT2G11340	17.5312	-4.2245	0.48028	-8.7959	1.42E-18	6.07E-17	0	0	0	4.94268	5.45092	5.07435	A1-	gypsy-like retrotransposon family (Athila)
AT2G11770	5.79174	-2.556	0.58167	-4.3943	1.11E-05	1.09E-04	0.64015	0	0.69459	3.48023	3.41604	3.88164	A1+	gypsy-like retrotransposon family (Athila)
AT2G12040	20.537	-4.2363	0.4579	-9.2517	2.21E-20	1.07E-18	0.60238	0	0.65347	5.45435	5.26298	5.42247	A1-	gypsy-like retrotransposon family (Athila)
AT2G12040	6.10116	-2.58	0.58202	-4.4329	9.30E-06	9.28E-05	0.64015	0	0.69459	4.16454	3.04536	3.61372	A1+	gypsy-like retrotransposon family (Athila)

AT2G12500	59.2428	-4.0872	0.51174	-7.9869	1.38E-15	4.88E-14	1.41979	2.09165	1.51363	7.95548	6.10263	5.11789	A1+	gypsy-like retrotransposon family (Athila)
AT2G12500	13.1611	-0.6235	0.16137	-3.8639	1.12E-04	0.007751	1.93072	2.70349	2.03713	4.35716	5.00689	4.16097	A2+	gypsy-like retrotransposon family (Athila)
AT2G13390	47.2708	-5.1958	0.41756	-12.443	1.52E-35	1.61E-33	1.02605	0.58899	0	6.20161	6.88921	6.53834	A1-	gypsy-like retrotransposon family (Athila)
AT2G14180	402.54	-7.699	0.37306	-20.637	1.28E-94	1.09E-91	1.35313	0	0.65347	9.3087	10.1936	9.25483	A1-	gypsy-like retrotransposon family (Athila)
AT2G14180	275.72	-7.397	0.42529	-17.393	9.36E-68	5.47E-65	1.41979	0	0.69459	9.75585	8.67853	8.57257	A1+	gypsy-like retrotransposon family (Athila)
AT2G14180	30.3508	-1.4825	0.17654	-8.3972	4.57E-17	1.40E-14	1.93072	0	1.02486	6.03649	6.16792	5.45016	A2+	gypsy-like retrotransposon family (Athila)
AT3G30400	6.11319	-2.978	0.58829	-5.0621	4.15E-07	5.28E-06	0	0	0	4.16454	3.04536	3.75389	A1+	gypsy-like retrotransposon family (Athila)
AT3G30418	8.16784	-3.1032	0.57834	-5.3657	8.06E-08	1.15E-06	0.64015	0	0	4.6268	3.04536	4.20843	A1+	gypsy-like retrotransposon family (Athila)
AT3G30418	5.25995	-2.3339	0.51514	-4.5306	5.88E-06	7.10E-05	0.60238	0	0	3.22203	2.66509	4.19911	A1-	gypsy-like retrotransposon family (Athila)
AT3G30620	1192.13	-9.2872	0.355	-26.161	7.31E-151	2.68E-147	1.41979	1.06174	1.16147	11.6097	10.8859	11.0596	A1+	gypsy-like retrotransposon family (Athila)
AT3G30620	1296.3	-9.1995	0.32516	-28.292	4.31E-176	3.69E-172	1.35313	1.00603	1.10158	11.3292	11.6396	10.9756	A1-	gypsy-like retrotransposon family (Athila)
AT3G30620	80.4001	-2.4687	0.1793	-13.768	3.94E-43	4.37E-40	1.93072	1.50483	1.61801	7.55257	7.41558	6.90945	A2+	gypsy-like retrotransposon family (Athila)
AT3G30668	40.4216	-5.4524	0.44138	-12.353	4.69E-35	4.84E-33	0	0	0	6.29203	6.43434	6.33427	A1-	gypsy-like retrotransposon family (Athila)
AT3G30695	231.505	-7.3929	0.42558	-17.371	1.37E-67	7.63E-65	0	1.06174	0.69459	9.40941	8.40766	8.5342	A1+	gypsy-like retrotransposon family (Athila)
AT3G30695	251.949	-7.3882	0.37106	-19.911	3.26E-88	2.33E-85	0	1.00603	0.65347	8.6851	9.36226	8.79236	A1-	gypsy-like retrotransposon family (Athila)
AT3G30695	21.1546	-1.2616	0.17275	-7.3027	2.82E-13	5.86E-11	0	1.50483	1.02486	5.28403	5.62471	5.27616	A2+	gypsy-like retrotransposon family (Athila)
AT3G30713	45.0553	-5.9137	0.49025	-12.063	1.66E-33	1.92E-31	0	0	0	6.50374	6.53766	6.48677	A1+	gypsy-like retrotransposon family (Athila)
AT3G30713	3.36951	-1.7722	0.50015	-3.5432	0.000395	0.003178	0	0	0	1.75715	3.54662	3.02706	A1-	gypsy-like retrotransposon family (Athila)
AT3G30713	12.8525	-0.9092	0.15768	-5.766	8.12E-09	1.05E-06	0	0	0	4.28307	4.79992	5.03519	A2+	gypsy-like retrotransposon family (Athila)
AT3G30749	382.136	-8.3458	0.37511	-22.249	1.15E-109	1.41E-106	0	0	0	9.55455	9.75907	9.4037	A1-	gypsy-like retrotransposon family (Athila)
AT3G30749	22.1194	-1.2945	0.17069	-7.5838	3.35E-14	7.90E-12	0	0	0	5.68019	5.74518	4.94502	A2+	gypsy-like retrotransposon family (Athila)
AT3G31356	15.964	-4.1383	0.48411	-8.5484	1.25E-17	5.09E-16	0	0	0	5.37382	4.7925	4.88781	A1-	gypsy-like retrotransposon family (Athila)
AT3G31356	12.2499	-0.8707	0.15581	-5.5883	2.29E-08	2.79E-06	0	0	0	4.94274	4.79992	4.16097	A2+	gypsy-like retrotransposon family (Athila)
AT3G32010	190.042	-7.3489	0.39795	-18.467	3.82E-76	1.85E-73	0	0	0	8.46953	8.94405	8.21068	A1-	gypsy-like retrotransposon family (Athila)
AT3G32010	10.0271	-0.7491	0.14915	-5.0223	5.11E-07	5.52E-05	0	0	0	4.49483	4.68412	3.89804	A2+	gypsy-like retrotransposon family (Athila)
AT3G32880	677.252	-8.7506	0.38238	-22.885	6.58E-116	9.39E-113	0	0	0	10.1335	10.9497	9.91502	A1-	gypsy-like retrotransposon family (Athila)
AT3G32880	52.5194	-2.2761	0.18026	-12.627	1.50E-36	1.17E-33	0	0	0	6.81809	6.84386	6.49841	A2+	gypsy-like retrotransposon family (Athila)
AT3G33124	5.49568	-2.5022	0.59234	-4.2242	2.40E-05	0.00022	0	0	0	3.86258	4.16427	1.80658	A1+	gypsy-like retrotransposon family (Athila)
AT3G33136	15.6748	-2.9355	0.58672	-5.0032	5.64E-07	7.03E-06	0	1.06174	0	5.78621	5.22024	1.80658	A1+	gypsy-like retrotransposon family (Athila)
AT3G33136	14.0161	-2.7037	0.51276	-5.2728	1.34E-07	2.12E-06	0	1.00603	0	4.4082	5.90133	2.34668	A1-	gypsy-like retrotransposon family (Athila)
AT3G33136	13.1851	-2.1592	0.45372	-4.7588	1.95E-06	3.67E-05	0	0.95929	0	5.88745	4.13223	2.14856	A2-	gypsy-like retrotransposon family (Athila)
AT3G33142	6.12353	-2.5959	0.58931	-4.4051	1.06E-05	0.000105	0	0.62641	0	3.68404	4.34682	2.58406	A1+	gypsy-like retrotransposon family (Athila)
AT3G33142	5.21749	-1.7671	0.44661	-3.9567	7.60E-05	0.000944	0	0.55795	0	4.44524	2.92871	2.14856	A2-	gypsy-like retrotransposon family (Athila)
AT3G33142	5.00622	-1.7058	0.50562	-3.3738	0.000741	0.005537	0	0.58899	0	3.02518	4.48353	1.01552	A1-	gypsy-like retrotransposon family (Athila)
AT3G33172	17.46	-3.1394	0.56884	-5.5191	3.41E-08	5.12E-07	0	1.3957	0.69459	5.37986	5.82339	2.85705	A1+	gypsy-like retrotransposon family (Athila)
AT3G37820	24.9877	-4.3883	0.54327	-8.0776	6.61E-16	2.39E-14	0	0	0.69459	5.91325	6.20076	4.20843	A1+	gypsy-like retrotransposon family (Athila)
AT3G37820	31.4912	-1.5906	0.49377	-3.2213	0.001276	0.008851	0	0	0.65347	4.88574	7.23452	3.48753	A1-	gypsy-like retrotransposon family (Athila)
AT3G42083	23.065	-3.9302	0.47214	-8.3242	8.49E-17	3.27E-15	0.60238	0.58899	0.65347	6.20161	5.45092	4.48892	A1-	gypsy-like retrotransposon family (Athila)
AT3G42083	14.3893	-2.9668	0.57926	-5.1218	3.03E-07	3.94E-06	0.64015	0.62641	0.69459	6.06692	2.54524	3.88164	A1+	gypsy-like retrotransposon family (Athila)
AT3G42252	6.11409	-2.5115	0.51513	-4.8756	1.09E-06	1.49E-05	0	0	0	3.02518	4.48353	3.19741	A1-	gypsy-like retrotransposon family (Athila)
AT3G42256	36.4887	-4.0696	0.39205	-10.38	3.05E-25	1.94E-23	1.61959	1.32917	1.71887	5.94714	6.60241	5.83769	A1-	gypsy-like retrotransposon family (Athila)
AT3G42256	23.4768	-3.0393	0.53553	-5.6753	1.38E-08	2.19E-07	1.6933	1.3957	1.79647	6.60759	4.50885	4.10751	A1+	gypsy-like retrotransposon family (Athila)
AT3G42258	21.0326	-3.7024	0.52399	-7.0656	1.60E-12	4.23E-11	0.64015	1.3957	0.69459	5.6469	5.88373	4.10751	A1+	gypsy-like retrotransposon family (Athila)
AT3G42258	23.6895	-3.154	0.44036	-7.1623	7.93E-13	4.53E-11	0.56965	1.27293	0.62068	6.46427	5.22414	4.08955	A2-	gypsy-like retrotransposon family (Athila)
AT3G42356	8.16976	-3.067	0.58803	-5.2158	1.83E-07	2.48E-06	0	0	0	4.81212	4.16427	2.58406	A1+	gypsy-like retrotransposon family (Athila)
AT3G42431	104.953	-6.6091	0.48764	-13.553	7.57E-42	1.34E-39	0	0	0	8.41512	7.37033	6.97621	A1+	gypsy-like retrotransposon family (Athila)
AT3G42431	91.379	-6.5454	0.41114	-15.92	4.58E-57	1.06E-54	0	0	0	7.52577	7.59496	7.44009	A1-	gypsy-like retrotransposon family (Athila)
AT3G42445	9.13025	-3.3087	0.58326	-5.6728	1.41E-08	2.22E-07	0	0	0	5.12374	3.41604	3.61372	A1+	gypsy-like retrotransposon family (Athila)
AT3G42445	9.85223	-3.1937	0.50839	-6.282	3.34E-10	7.20E-09	0	0	0	3.54983	5.04684	4.11654	A1-	gypsy-like retrotransposon family (Athila)
AT3G42716	1225.92	-9.3978	0.35639	-26.369	3.09E-153	9.92E-150	0.60238	0.58899	0	11.2383	11.6299	10.7916	A1-	gypsy-like retrotransposon family (Athila)
AT3G42716	104.684	-2.3959	0.18027	-13.291	2.62E-40	2.40E-37	0.95419	0.94033	0	7.84426	8.12531	6.90945	A2+	gypsy-like retrotransposon family (Athila)
AT3G43304	106.683	-6.6024	0.39855	-16.566	1.23E-61	3.19E-59	0	0.58899	0	7.59845	7.9154	7.69607	A1-	gypsy-like retrotransposon family (Athila)
AT3G43862	532.776	-8.4457	0.36248	-23.3	4.44E-120	6.71E-117	0.60238	0	0.65347	9.81116	10.413	9.87162	A1-	gypsy-like retrotransposon family (Athila)
AT3G43862	489.493	-8.4213	0.4208	-20.013	4.27E-89	5.49E-86	0.64015	0	0.69459	10.524	9.47091	9.56355	A1+	gypsy-like retrotransposon family (Athila)
AT3G43862	49.4942	-1.9427	0.17962	-10.816	2.90E-27	1.46E-24	0.95419	0	1.02486	6.57583	7.04503	6.14039	A2+	gypsy-like retrotransposon family (Athila)
AT4G03790	465.25	-8.01	0.3725	-21.503	1.45E-102	1.56E-99	0	1.32917	0	9.64311	10.3553	9.4167	A1-	gypsy-like retrotransposon family (Athila)
AT4G03790	22.696	-1.259	0.17223	-7.3102	2.67E-13	5.59E-11	0	1.90954	0	5.70877	5.62471	5.12007	A2+	gypsy-like retrotransposon family (Athila)

AT4G03795	9.93649	-3.2348	0.50736	-6.3758	1.82E-10	3.99E-09	0	0	0	3.93374	5.04684	3.83605	A1-	gypsy-like retrotransposon family (Athila)
AT4G03860	357.689	-7.7349	0.38426	-20.13	4.06E-90	3.07E-87	0.60238	0	0.65347	9.11792	10.0263	9.10229	A1-	gypsy-like retrotransposon family (Athila)
AT4G03860	310.65	-7.5912	0.45062	-16.846	1.12E-63	5.12E-61	0.64015	0	0.69459	10.0558	8.42806	8.83576	A1+	gypsy-like retrotransposon family (Athila)
AT4G03860	25.5955	-1.3591	0.1735	-7.8336	4.74E-15	1.24E-12	0.95419	0	1.02486	5.70877	6.05591	5.1607	A2+	gypsy-like retrotransposon family (Athila)
AT4G05556	14.1055	-3.9378	0.48902	-8.0524	8.12E-16	2.90E-14	0	0	0	4.70005	5.04684	4.83714	A1-	gypsy-like retrotransposon family (Athila)
AT4G05638	6.1031	-2.6679	0.51481	-5.1822	2.19E-07	3.34E-06	0	0	0	3.22203	4.08983	3.72897	A1-	gypsy-like retrotransposon family (Athila)
AT4G06485	775.445	-8.6526	0.34237	-25.272	6.44E-141	1.27E-137	0.60238	1.00603	1.10158	10.3854	10.9729	10.3505	A1-	gypsy-like retrotransposon family (Athila)
AT4G06485	674.227	-8.171	0.42767	-19.106	2.26E-81	2.15E-78	0.64015	1.06174	1.16147	11.2291	9.46105	9.87697	A1+	gypsy-like retrotransposon family (Athila)
AT4G06485	79.8609	-2.4865	0.17953	-13.85	1.27E-43	1.47E-40	0.95419	1.50483	1.61801	7.44624	7.52901	6.88541	A2+	gypsy-like retrotransposon family (Athila)
AT4G06506	2193.09	-9.6911	0.36607	-26.473	1.98E-154	7.27E-151	1.02605	0	0.65347	11.6919	12.7158	11.6092	A1-	gypsy-like retrotransposon family (Athila)
AT4G06506	167.822	-3.1281	0.17748	-17.625	1.59E-69	4.22E-66	1.52355	0	1.02486	8.61817	8.59088	7.83604	A2+	gypsy-like retrotransposon family (Athila)
AT4G06540	20.3603	-4.2925	0.46651	-9.2012	3.54E-20	1.68E-18	0	0	0.65347	5.19788	5.61718	5.278	A1-	gypsy-like retrotransposon family (Athila)
AT4G06573	198.177	-7.3703	0.39896	-18.474	3.37E-76	1.67E-73	0	0	0	8.57234	9.02025	8.19064	A1-	gypsy-like retrotransposon family (Athila)
AT4G06573	15.8859	-1.0786	0.16462	-6.552	5.68E-11	9.26E-09	0	0	0	5.03592	5.31008	4.69132	A2+	gypsy-like retrotransposon family (Athila)
AT4G06578	7.02629	-3.209	0.58343	-5.5003	3.79E-08	5.65E-07	0	0	0	3.48023	3.95523	4.20843	A1+	gypsy-like retrotransposon family (Athila)
AT4G06578	7.57762	-3.0499	0.51067	-5.9722	2.34E-09	4.66E-08	0	0	0	3.81676	4.08983	4.11654	A1-	gypsy-like retrotransposon family (Athila)
AT4G06585	6.87584	-2.9366	0.58108	-5.0538	4.33E-07	5.50E-06	0	0.62641	0	4.29471	3.71068	3.45845	A1+	gypsy-like retrotransposon family (Athila)
AT4G06624	37.1667	-5.2018	0.45091	-11.536	8.67E-31	7.38E-29	0	0	0	5.86113	6.67963	6.0304	A1-	gypsy-like retrotransposon family (Athila)
AT4G06656	6.59917	-2.5984	0.51366	-5.0586	4.22E-07	6.16E-06	0	0.58899	0	4.4082	3.54662	3.19741	A1-	gypsy-like retrotransposon family (Athila)
AT4G07600	11.4412	-3.7691	0.56971	-6.6158	3.70E-11	8.36E-10	0	0	0	5.19216	4.34682	3.88164	A1+	gypsy-like retrotransposon family (Athila)
AT4G07600	12.0061	-3.6211	0.49813	-7.2693	3.61E-13	1.03E-11	0	0	0	4.56149	5.04684	4.19911	A1-	gypsy-like retrotransposon family (Athila)
AT4G07937	12.4326	-3.6982	0.49599	-7.4561	8.91E-14	2.67E-12	0	0	0	4.32498	5.04684	4.61457	A1-	gypsy-like retrotransposon family (Athila)
AT4G07937	6.55675	-2.9204	0.58977	-4.9517	7.36E-07	9.00E-06	0	0	0	4.52436	2.54524	3.75389	A1+	gypsy-like retrotransposon family (Athila)
AT4G08050	641.255	-6.8726	0.26616	-25.821	5.14E-147	1.32E-143	2.50182	3.76604	2.32499	10.2898	10.5834	10.0266	A1-	gypsy-like retrotransposon family (Athila)
AT4G08050	43.0651	-1.0843	0.17976	-6.0317	1.62E-09	2.21E-07	3.23839	4.5834	3.04322	6.30338	6.25166	6.07812	A2+	gypsy-like retrotransposon family (Athila)
AT4G08078	34.8299	-5.1061	0.45386	-11.25	2.31E-29	1.86E-27	0	0	0	5.76968	6.60241	5.91301	A1-	gypsy-like retrotransposon family (Athila)
AT4G08080	139.223	-6.793	0.40092	-16.943	2.15E-64	6.35E-62	0.60238	0	0	7.8045	8.5711	7.86995	A1-	gypsy-like retrotransposon family (Athila)
AT4G08080	106.337	-6.3207	0.48943	-12.915	3.73E-38	5.47E-36	0.64015	0	0	8.57098	6.98599	7.05962	A1+	gypsy-like retrotransposon family (Athila)
AT4G08080	10.5038	-0.7567	0.15161	-4.9912	6.00E-07	6.38E-05	0.95419	0	0	4.67946	4.55821	3.99111	A2+	gypsy-like retrotransposon family (Athila)
AT5G04965	22.8562	-4.621	0.46762	-9.882	4.98E-23	2.82E-21	0	0	0	5.45435	5.76624	5.38769	A1-	gypsy-like retrotransposon family (Athila)
AT5G04965	11.2538	-3.2637	0.58609	-5.5686	2.57E-08	3.92E-07	0	0	0	5.6469	3.41604	3.28443	A1+	gypsy-like retrotransposon family (Athila)
AT5G29032	6.93329	-3.0466	0.58746	-5.1861	2.15E-07	2.87E-06	0	0	0	4.52436	3.71068	3.08651	A1+	gypsy-like retrotransposon family (Athila)
AT5G29032	5.57519	-2.3038	0.51397	-4.4823	7.38E-06	8.74E-05	0	0	0	2.79716	4.48353	2.83386	A1-	gypsy-like retrotransposon family (Athila)
AT5G29975	169.604	-7.0061	0.38394	-18.248	2.15E-74	9.69E-72	0	0.58899	0.65347	8.12052	8.76074	8.2644	A1-	gypsy-like retrotransposon family (Athila)
AT5G29975	75.3201	-5.5553	0.50684	-10.961	5.90E-28	4.99E-26	0	0.62641	0.69459	8.19104	6.46052	6.19861	A1+	gypsy-like retrotransposon family (Athila)
AT5G29975	29.5546	-1.4886	0.17565	-8.4748	2.35E-17	7.45E-15	0	0.94033	1.02486	5.99067	6.2104	5.3483	A2+	gypsy-like retrotransposon family (Athila)
AT5G31087	14.1938	-4.1006	0.55876	-7.3387	2.16E-13	6.20E-12	0	0	0	4.02145	5.31093	5.01018	A1+	gypsy-like retrotransposon family (Athila)
AT5G31087	6.72716	-0.5491	0.13462	-4.0787	4.53E-05	3.39E-03	0	0	0	3.49317	4.18491	3.79855	A2+	gypsy-like retrotransposon family (Athila)
AT5G31719	176.418	-7.0512	0.38354	-18.385	1.75E-75	8.17E-73	0	0.58899	0.65347	8.1328	8.81308	8.36618	A1-	gypsy-like retrotransposon family (Athila)
AT5G31719	76.0257	-5.581	0.50553	-11.04	2.45E-28	2.12E-26	0	0.62641	0.69459	8.18266	6.61089	6.12306	A1+	gypsy-like retrotransposon family (Athila)
AT5G31719	30.2627	-1.4906	0.1756	-8.4884	2.09E-17	6.71E-15	0	0.94033	1.02486	6.01376	6.27186	5.3483	A2+	gypsy-like retrotransposon family (Athila)
AT5G31804	5.77964	-2.6884	0.51475	-5.2228	1.76E-07	2.72E-06	0	0	0	3.54983	3.54662	3.83605	A1-	gypsy-like retrotransposon family (Athila)
AT5G31804	3.68843	-2.1551	0.58904	-3.6587	2.53E-04	1.82E-03	0	0	0	3.86258	2.54524	2.24707	A1+	gypsy-like retrotransposon family (Athila)
AT5G31963	45.8349	-5.4695	0.444	-12.319	7.19E-35	7.33E-33	0	0	0	6.0283	6.95281	6.47347	A1-	gypsy-like retrotransposon family (Athila)
AT5G32306	138.797	-6.9852	0.40382	-17.298	4.89E-67	1.57E-64	0	0	0	7.85728	8.46604	7.96739	A1-	gypsy-like retrotransposon family (Athila)
AT5G32306	62.1331	-5.5873	0.52597	-10.623	2.33E-26	1.80E-24	0	0	0	7.91557	6.29264	5.80629	A1+	gypsy-like retrotransposon family (Athila)
AT5G32306	22.4747	-1.3223	0.1714	-7.7146	1.21E-14	2.99E-12	0	0	0	5.59088	5.82936	5.03519	A2+	gypsy-like retrotransposon family (Athila)
AT5G32423	42.1718	-5.3742	0.44676	-12.029	2.50E-33	2.34E-31	0	0	0	5.89038	6.75292	6.47347	A1-	gypsy-like retrotransposon family (Athila)
AT5G32475	170.318	-7.0317	0.39703	-17.711	3.46E-70	1.33E-67	0	0	0.65347	8.2044	8.8636	8.04199	A1-	gypsy-like retrotransposon family (Athila)
AT5G32475	10.8833	-0.7819	0.15317	-5.1051	3.31E-07	3.66E-05	0	0	1.02486	4.42764	4.79992	4.16097	A2+	gypsy-like retrotransposon family (Athila)
AT5G33050	182.357	-7.1723	0.39215	-18.289	1.00E-74	4.61E-72	0.60238	0	0	8.11433	8.88005	8.44442	A1-	gypsy-like retrotransposon family (Athila)
AT5G33050	132.042	-7.165	0.4382	-16.351	4.28E-60	1.72E-57	0.64015	0	0	8.20766	7.85024	8.0677	A1+	gypsy-like retrotransposon family (Athila)
AT5G33050	60.163	-2.2184	0.18025	-12.307	8.29E-35	6.12E-32	0.95419	0	0	6.80491	7.30232	6.5452	A2+	gypsy-like retrotransposon family (Athila)
AT5G34834	9.95057	-3.046	0.55966	-5.4426	5.25E-08	7.68E-07	0	1.06174	0.69459	3.48023	4.78679	4.4747	A1+	gypsy-like retrotransposon family (Athila)
AT5G34834	10.5484	-2.9146	0.49903	-5.8405	5.20E-09	9.85E-08	0	1.00603	0.65347	3.54983	4.48353	4.93675	A1-	gypsy-like retrotransposon family (Athila)
AT5G34834	8.56029	-0.5967	0.14603	-4.0859	4.39E-05	3.32E-03	0	1.50483	1.02486	3.8418	4.2677	4.16097	A2+	gypsy-like retrotransposon family (Athila)
AT5G35057	379.238	-8.2308	0.38159	-21.57	3.44E-103	3.84E-100	0	0	0	9.3168	9.93473	9.37068	A1-	gypsy-like retrotransposon family (Athila)

AT5G35057	656.002	-3.0313	0.5922	-5.1187	3.08E-07	4.00E-06	0	0	0	7.75521	10.7385	10.9762	A1+	gypsy-like retrotransposon family (Athila)
AT5G35057	55.29	-1.918	0.17883	-10.726	7.72E-27	3.80E-24	0	0	0	7.19668	6.94794	6.01303	A2+	gypsy-like retrotransposon family (Athila)
AT1G42070	9.04863	-3.6239	0.57161	-6.3398	2.30E-10	4.70E-09	0	0	0	4.29471	4.16427	4.30275	A1+	gypsy-like retrotransposon family (Ty3-element)
AT1G42070	6.01884	-2.5187	0.5151	-4.8897	1.01E-06	1.39E-05	0	0	0	2.52621	4.08983	4.02897	A1-	gypsy-like retrotransposon family (Ty3-element)
AT2G01026	16.2382	-3.8732	0.48172	-8.0404	8.95E-16	3.18E-14	0	0	0.65347	4.48688	5.45092	5.07435	A1-	gypsy-like retrotransposon family (Ty3-element)
AT2G01028	14.8758	-3.5519	0.48316	-7.3513	1.96E-13	5.72E-12	0	0.58899	0.65347	4.23666	5.45092	4.83714	A1-	gypsy-like retrotransposon family (Ty3-element)
AT2G01034	15.8617	-3.4489	0.47927	-7.1962	6.19E-13	1.72E-11	0	0.58899	1.10158	4.23666	5.61718	4.83714	A1-	gypsy-like retrotransposon family (Ty3-element)
AT2G01037	24.9755	-3.718	0.44085	-8.4339	3.34E-17	1.32E-15	0.60238	1.00603	1.71887	4.88574	6.13862	5.61521	A1-	gypsy-like retrotransposon family (Ty3-element)
AT2G01037	7.62626	-2.1396	0.55934	-3.8252	1.31E-04	1.01E-03	0.64015	1.06174	1.79647	3.86258	4.34682	3.28443	A1+	gypsy-like retrotransposon family (Ty3-element)
AT2G06470	118.966	-6.5729	0.40724	-16.14	1.33E-58	3.26E-56	0	0.58899	0	7.32624	8.25518	7.96739	A1-	gypsy-like retrotransposon family (Ty3-element)
AT2G09920	41.4377	-5.2622	0.45124	-11.662	2.00E-31	1.75E-29	0	0	0	5.89038	6.95281	6.09651	A1-	gypsy-like retrotransposon family (Ty3-element)
AT2G10540	12.1305	-2.7199	0.58899	-4.6179	3.88E-06	4.18E-05	0	1.06174	0	1.42937	5.22024	5.11789	A1+	gypsy-like retrotransposon family (Ty3-element)
AT2G10540	8.1363	-2.5671	0.51017	-5.0318	4.86E-07	7.02E-06	0	1.00603	0	3.68946	4.7925	3.34975	A1-	gypsy-like retrotransposon family (Ty3-element)
AT2G10780	19.8418	-4.3134	0.47961	-8.9937	2.39E-19	1.07E-17	0	0	0	4.70005	5.61718	5.55378	A1-	gypsy-like retrotransposon family (Ty3-element)
AT2G15410	6.75801	-3.1859	0.58356	-5.4594	4.78E-08	7.02E-07	0	0	0	3.86258	3.95523	3.75389	A1+	gypsy-like retrotransposon family (Ty3-element)
AT3G29615	14.9028	-3.7699	0.48662	-7.7471	9.40E-15	3.05E-13	0.60238	0	0	4.32498	5.04684	5.278	A1-	gypsy-like retrotransposon family (Ty3-element)
AT3G29792	11.0668	-2.7622	0.57306	-4.8202	1.43E-06	1.67E-05	0.64015	1.06174	0.69459	5.54593	3.41604	3.28443	A1+	gypsy-like retrotransposon family (Ty3-element)
AT3G32195	23.0214	-2.65	0.43126	-6.1449	8.00E-10	1.67E-08	1.35313	2.71064	2.1498	4.56149	6.02485	5.38769	A1-	gypsy-like retrotransposon family (Ty3-element)
AT3G32290	8.04889	-3.0203	0.58887	-5.129	2.91E-07	3.81E-06	0	0	0	4.89656	3.95523	2.58406	A1+	gypsy-like retrotransposon family (Ty3-element)
AT3G32295	35.5739	-5.1395	0.53081	-9.6823	3.59E-22	2.14E-20	0	0	0	6.88065	5.99732	5.11789	A1+	gypsy-like retrotransposon family (Ty3-element)
AT3G32295	16.6144	-4.0812	0.48658	-8.3874	4.97E-17	1.96E-15	0	0	0	4.48688	5.26298	5.38769	A1-	gypsy-like retrotransposon family (Ty3-element)
AT3G33130	12.9297	-2.6519	0.57742	-4.5927	4.37E-06	4.65E-05	1.41979	0.62641	0.69459	5.87214	3.41604	3.08651	A1+	gypsy-like retrotransposon family (Ty3-element)
AT3G33130	8.86642	-1.7648	0.51514	-3.4259	0.000613	0.004701	1.35313	0.58899	0.65347	2.52621	5.45092	2.02322	A1-	gypsy-like retrotransposon family (Ty3-element)
AT3G33154	10.6175	-3.0783	0.57389	-5.3639	8.15E-08	1.16E-06	0.64015	0.62641	0	5.05192	4.6545	2.85705	A1+	gypsy-like retrotransposon family (Ty3-element)
AT3G33154	10.4523	-2.3959	0.51454	-4.6563	3.22E-06	4.05E-05	0.60238	0.58899	0	3.39522	5.61718	2.34668	A1-	gypsy-like retrotransposon family (Ty3-element)
AT3G33154	9.87234	-1.8277	0.44887	-4.0718	4.67E-05	0.000613	0.56965	0.55795	0	5.66912	2.57549	2.14856	A2-	gypsy-like retrotransposon family (Ty3-element)
AT3G33178	10.9624	-3.0008	0.56683	-5.2941	1.20E-07	1.66E-06	0.64015	0.62641	0.69459	4.89656	4.90796	2.85705	A1+	gypsy-like retrotransposon family (Ty3-element)
AT3G33178	17.7038	-2.7135	0.51188	-5.3009	1.15E-07	1.84E-06	0.60238	0.58899	0.65347	4.48688	6.34234	2.02322	A1-	gypsy-like retrotransposon family (Ty3-element)
AT3G33178	19.7386	-2.1196	0.45322	-4.6767	2.92E-06	5.24E-05	0.56965	0.55795	0.62068	6.65487	3.98933	1.71775	A2-	gypsy-like retrotransposon family (Ty3-element)
AT3G33193	18.5704	-2.505	0.53349	-4.6954	2.66E-06	2.97E-05	1.08201	2.09165	2.41385	6.06692	4.6545	3.75389	A1+	gypsy-like retrotransposon family (Ty3-element)
AT3G33193	25.3477	-2.4738	0.49127	-5.0355	4.77E-07	6.90E-06	1.02605	2.00904	2.32499	4.04193	6.82268	4.11654	A1-	gypsy-like retrotransposon family (Ty3-element)
AT3G33193	15.0159	-1.9577	0.44324	-4.4168	1.00E-05	1.58E-04	0.97697	1.93849	2.25193	5.78241	4.26223	3.49845	A2-	gypsy-like retrotransposon family (Ty3-element)
AT3G42057	12.1526	-2.723	0.5443	-5.0027	5.65E-07	7.04E-06	1.92313	0	1.16147	4.6268	5.01973	3.88164	A1+	gypsy-like retrotransposon family (Ty3-element)
AT3G42622	30.1255	-4.6885	0.44528	-10.529	6.34E-26	4.24E-24	0.60238	0	0.65347	5.49299	6.02485	6.1802	A1-	gypsy-like retrotransposon family (Ty3-element)
AT3G42622	27.0022	-4.2972	0.53873	-7.9765	1.51E-15	5.30E-14	0.64015	0	0.69459	6.68085	4.50885	5.26573	A1+	gypsy-like retrotransposon family (Ty3-element)
AT3G42993	6.8783	-2.9339	0.58106	-5.0491	4.44E-07	5.62E-06	0.64015	0	0	4.29471	3.71068	3.45845	A1+	gypsy-like retrotransposon family (Ty3-element)
AT3G42996	9.85846	-3.0859	0.49726	-6.2059	5.44E-10	1.14E-08	0.60238	0.58899	0	3.93374	4.48353	4.55311	A1-	gypsy-like retrotransposon family (Ty3-element)
AT3G43307	40.4291	-5.0057	0.45023	-11.118	1.02E-28	7.99E-27	0.60238	0	0	5.76968	7.01373	5.93726	A1-	gypsy-like retrotransposon family (Ty3-element)
AT3G45446	4.95143	-2.5112	0.59237	-4.2392	2.24E-05	0.000207	0	0	0	4.29471	2.54524	2.85705	A1+	gypsy-like retrotransposon family (Ty3-element)
AT3G62475	21.6907	-4.8198	0.53202	-9.0593	1.31E-19	6.51E-18	0	0	0	5.5973	4.90796	5.77373	A1+	gypsy-like retrotransposon family (Ty3-element)
AT3G62475	24.1227	-4.4225	0.47904	-9.2321	2.65E-20	1.27E-18	0	0	0	5.86113	6.02485	4.61457	A1-	gypsy-like retrotransposon family (Ty3-element)
AT3G62475	6.20372	-0.5117	0.13125	-3.8986	9.67E-05	6.81E-03	0	0	0	4.03478	3.79607	3.31339	A2+	gypsy-like retrotransposon family (Ty3-element)
AT4G03770	22.7355	-4.6721	0.51882	-9.0053	2.15E-19	1.05E-17	0	0	0.69459	5.49267	5.88373	5.11789	A1+	gypsy-like retrotransposon family (Ty3-element)
AT4G03770	13.7071	-0.7182	0.14801	-4.8525	1.22E-06	0.000125	0	0	1.02486	3.49317	5.52727	4.74575	A2+	gypsy-like retrotransposon family (Ty3-element)
AT4G03840	11.9688	-3.6229	0.49905	-7.2596	3.88E-13	1.10E-11	0	0	0	4.04193	4.7925	4.93675	A1-	gypsy-like retrotransposon family (Ty3-element)
AT4G05593	27.6458	-4.842	0.46214	-10.477	1.10E-25	7.24E-24	0	0	0	5.37382	6.02485	5.96112	A1-	gypsy-like retrotransposon family (Ty3-element)
AT4G05593	8.91571	-2.7191	0.59228	-4.591	4.41E-06	4.69E-05	0	0	0	5.43737	1.77337	3.28443	A1+	gypsy-like retrotransposon family (Ty3-element)
AT4G06566	984.522	-8.6738	0.37382	-23.203	4.24E-119	1.09E-115	1.08201	2.09165	0.69459	11.4891	10.8102	10.2704	A1+	gypsy-like retrotransposon family (Ty3-element)
AT4G06566	356.22	-4.0578	0.16873	-24.049	8.61E-128	1.14E-123	1.52355	2.70349	1.02486	9.32703	9.77916	9.25497	A2+	gypsy-like retrotransposon family (Ty3-element)
AT4G06566	35.9793	-3.9421	0.43332	-9.0974	9.25E-20	4.25E-18	1.02605	2.00904	0.65347	5.19788	6.82268	6.00768	A1-	gypsy-like retrotransposon family (Ty3-element)
AT4G06604	5.48865	-2.7951	0.59084	-4.7308	2.24E-06	2.52E-05	0	0	0	4.16454	3.04536	3.28443	A1+	gypsy-like retrotransposon family (Ty3-element)
AT4G06628	99.9598	-6.9524	0.46013	-15.11	1.40E-51	3.82E-49	0	0	0	7.64968	7.78846	7.49877	A1+	gypsy-like retrotransposon family (Ty3-element)
AT4G06628	72.223	-6.0783	0.42709	-14.232	5.81E-46	9.15E-44	0	0	0	6.8337	7.63429	6.94949	A1-	gypsy-like retrotransposon family (Ty3-element)
AT4G06686	123.189	-6.9496	0.40154	-17.308	4.13E-67	1.34E-64	0	0	0	7.90821	7.9154	8.02511	A1-	gypsy-like retrotransposon family (Ty3-element)
AT4G06686	18.6081	-1.2353	0.16956	-7.2852	3.21E-13	6.57E-11	0	0	0	5.28403	5.42276	5.03519	A2+	gypsy-like retrotransposon family (Ty3-element)
AT4G07850	48.6177	-3.2137	0.40197	-7.9948	1.30E-15	4.55E-14	2.21024	3.53456	1.71887	5.70532	6.95281	6.67443	A1-	gypsy-like retrotransposon family (Ty3-element)
AT4G22415	24.4997	-4.5711	0.50979	-8.9665	3.06E-19	1.48E-17	0.64015	0	0.69459	4.97633	5.99732	5.74041	A1+	gypsy-like retrotransposon family (Ty3-element)

AT4G22415	10.2087	-3.1586	0.4942	-6.3914	1.64E-10	3.63E-09	0.60238	0	0.65347	4.48688	4.48353	4.19911	A1-	gypsy-like retrotransposon family (Ty3-element)
AT5G28865	18.0636	-3.7017	0.56961	-6.4987	8.10E-11	1.76E-09	0	0	0.69459	2.9585	5.47681	5.8693	A1+	gypsy-like retrotransposon family (Ty3-element)
AT5G29075	15.5181	-3.5901	0.47189	-7.6079	2.79E-14	8.79E-13	0.60238	0.58899	0.65347	4.48688	5.26298	5.07435	A1-	gypsy-like retrotransposon family (Ty3-element)
AT5G29562	21.7414	-4.4072	0.4632	-9.5146	1.82E-21	9.37E-20	0.60238	0	0	5.24392	5.61718	5.52205	A1-	gypsy-like retrotransposon family (Ty3-element)
AT5G29562	117.799	-3.7835	0.58463	-6.4717	9.69E-11	2.08E-09	0.64015	0	0	5.78621	8.24467	8.45425	A1+	gypsy-like retrotransposon family (Ty3-element)
AT5G29562	17.2312	-1.1011	0.16645	-6.6149	3.72E-11	6.26E-09	0.95419	0	0	5.35808	5.27047	4.69132	A2+	gypsy-like retrotransposon family (Ty3-element)
AT5G31981	10.3996	-3.7083	0.57056	-6.4994	8.06E-11	1.75E-09	0	0	0	4.97633	4.16427	3.99899	A1+	gypsy-like retrotransposon family (Ty3-element)
AT5G31981	9.6038	-3.2601	0.50768	-6.4216	1.35E-10	3.00E-09	0	0	0	3.54983	4.48353	4.73014	A1-	gypsy-like retrotransposon family (Ty3-element)
AT5G32436	15.2271	-3.2377	0.58815	-5.5049	3.69E-08	5.52E-07	0	0	0	1.42937	5.55311	5.48276	A1+	gypsy-like retrotransposon family (Ty3-element)
AT5G32436	7.59961	-2.7795	0.5141	-5.4065	6.43E-08	1.06E-06	0	0	0	3.54983	4.7925	3.19741	A1-	gypsy-like retrotransposon family (Ty3-element)
AT1G37603	34.6788	-4.9866	0.4454	-11.196	4.27E-29	3.41E-27	0.60238	0	0	5.73786	6.52082	6.0304	A1-	gypsy-like retrotransposon pseudogene (Athila)
AT4G05587	289.26	-7.793	0.37915	-20.554	7.10E-94	5.70E-91	0	0	0.65347	8.9159	9.51756	9.028	A1-	gypsy-like retrotransposon pseudogene (Athila)
AT4G05587	14.5037	-0.9423	0.16056	-5.869	4.38E-09	5.77E-07	0	0	1.02486	5.1653	5.0543	4.31292	A2+	gypsy-like retrotransposon pseudogene (Athila)
AT4G07942	1858.62	-10.027	0.36432	-27.522	9.56E-167	6.14E-163	0	0	0	11.5876	12.3691	11.4445	A1-	gypsy-like retrotransposon pseudogene (Athila)
AT4G07942	995.549	-4.6314	0.57552	-8.0473	8.46E-16	3.04E-14	0	0	0	11.9407	9.72291	10.2289	A1+	gypsy-like retrotransposon pseudogene (Athila)
AT4G07942	77.1943	-2.5039	0.18007	-13.905	5.89E-44	7.46E-41	0	0	0	7.42916	7.51213	6.79795	A2+	gypsy-like retrotransposon pseudogene (Athila)
AT5G32404	170.402	-7.3952	0.46154	-16.023	8.88E-58	3.11E-55	0	0	0	8.96729	7.92393	8.13975	A1+	gypsy-like retrotransposon pseudogene (Athila)
AT5G32404	179.922	-7.331	0.3961	-18.508	1.79E-76	9.02E-74	0	0	0	8.26147	8.81308	8.3482	A1-	gypsy-like retrotransposon pseudogene (Athila)
AT5G32404	14.6757	-1.0199	0.16247	-6.2778	3.43E-10	5.05E-08	0	0	0	4.94274	5.18785	4.57587	A2+	gypsy-like retrotransposon pseudogene (Athila)
AT5G32624	144.935	-7.1259	0.39959	-17.833	3.90E-71	1.54E-68	0	0	0	8.06389	8.03777	8.41881	A1-	gypsy-like retrotransposon pseudogene (Athila)
AT5G32624	137.994	-4.7964	0.56759	-8.4505	2.90E-17	1.17E-15	0	0	0	6.47656	8.51647	8.55351	A1+	gypsy-like retrotransposon pseudogene (Athila)
AT5G32624	23.1748	-1.3333	0.17159	-7.7703	7.83E-15	1.98E-12	0	0	0	5.65103	5.88287	5.03519	A2+	gypsy-like retrotransposon pseudogene (Athila)
AT5G33389	356.088	-8.0163	0.36478	-21.976	4.92E-107	5.74E-104	0	0.58899	0.65347	9.1814	9.53844	9.66928	A1-	gypsy-like retrotransposon pseudogene (Athila)
AT5G33389	311.716	-7.8795	0.42778	-18.419	9.17E-76	6.55E-73	0	0.62641	0.69459	8.55808	9.37958	9.69545	A1+	gypsy-like retrotransposon pseudogene (Athila)
AT5G33389	63.9971	-2.0398	0.17978	-11.347	7.72E-30	4.37E-27	0	0.94033	1.02486	7.27523	7.27255	6.23857	A2+	gypsy-like retrotransposon pseudogene (Athila)
AT1G42110	10.6477	-3.7737	0.56758	-6.6487	2.96E-11	6.79E-10	0	0	0	4.29471	4.90796	4.10751	A1+	hAT-like transposase family (hobo/Ac/Tam3)
AT1G42110	7.08389	-2.9991	0.51168	-5.8613	4.59E-09	8.78E-08	0	0	0	4.04193	3.54662	4.11654	A1-	hAT-like transposase family (hobo/Ac/Tam3)
AT2G05700	50.6876	-5.8738	0.4735	-12.405	2.45E-35	3.20E-33	0	0	0.69459	6.65684	6.84135	6.50673	A1+	hAT-like transposase family (hobo/Ac/Tam3)
AT2G05700	52.1101	-5.5825	0.42816	-13.038	7.41E-39	9.02E-37	0	0	0.65347	6.9213	6.82268	6.33427	A1-	hAT-like transposase family (hobo/Ac/Tam3)
AT2G06760	159.253	-7.2713	0.39452	-18.431	7.42E-76	3.53E-73	0	0	0	8.39498	8.32894	8.23045	A1-	hAT-like transposase family (hobo/Ac/Tam3)
AT2G06760	50.8742	-6.0176	0.48969	-12.288	1.04E-34	1.28E-32	0	0	0	6.99997	6.46052	6.52641	A1+	hAT-like transposase family (hobo/Ac/Tam3)
AT2G06760	5.84824	-0.4976	0.12997	-3.8284	1.29E-04	8.64E-03	0	0	0	3.8418	3.67995	3.45081	A2+	hAT-like transposase family (hobo/Ac/Tam3)
AT2G14950	32.4335	-5.1793	0.50138	-10.33	5.15E-25	3.73E-23	0.64015	0	0	6.36244	6.05094	5.59885	A1+	hAT-like transposase family (hobo/Ac/Tam3)
AT2G14950	13.5784	-0.9626	0.16176	-5.9508	2.67E-09	3.58E-07	0.95419	0	0	4.67946	4.95786	4.74575	A2+	hAT-like transposase family (hobo/Ac/Tam3)
AT2G15160	7.1513	-2.8257	0.50974	-5.5434	2.97E-08	5.09E-07	0	0	0.65347	3.93374	3.54662	4.19911	A1-	hAT-like transposase family (hobo/Ac/Tam3)
AT2G15160	4.14017	-2.1152	0.59232	-3.571	3.56E-04	2.46E-03	0	0	0.69459	2.1331	3.71068	3.28443	A1+	hAT-like transposase family (hobo/Ac/Tam3)
AT2G15940	45.1425	-5.0975	0.40786	-12.498	7.62E-36	8.16E-34	0.60238	0.58899	1.10158	6.552	6.60241	6.33427	A1-	hAT-like transposase family (hobo/Ac/Tam3)
AT2G15940	25.5103	-4.0069	0.52735	-7.5983	3.00E-14	9.36E-13	0.64015	0.62641	1.16147	6.5566	5.12347	4.62832	A1+	hAT-like transposase family (hobo/Ac/Tam3)
AT3G23085	26.4264	-4.0355	0.45538	-8.8619	7.86E-19	3.64E-17	0.64015	1.89477	1.16147	5.54593	5.62557	5.92967	A1+	hAT-like transposase family (hobo/Ac/Tam3)
AT3G23085	14.0982	-2.9735	0.4626	-6.4277	1.30E-10	2.89E-09	0.60238	1.81597	1.10158	5.15032	4.48353	4.67351	A1-	hAT-like transposase family (hobo/Ac/Tam3)
AT3G43523	29.4627	-4.9206	0.4924	-9.9932	1.63E-23	1.09E-21	1.08201	0	0	6.1029	5.94164	5.59885	A1+	hAT-like transposase family (hobo/Ac/Tam3)
AT3G43523	13.5986	-0.8966	0.16013	-5.5992	2.15E-08	2.63E-06	1.52355	0	0	4.55904	5.1002	4.63475	A2+	hAT-like transposase family (hobo/Ac/Tam3)
AT4G05510	17.339	-2.9612	0.5867	-5.0472	4.48E-07	5.68E-06	0	0	1.16147	6.39182	2.54524	3.99899	A1+	hAT-like transposase family (hobo/Ac/Tam3)
AT4G05510	9.31205	-2.8992	0.50298	-5.7642	8.21E-09	1.51E-07	0	0	1.10158	3.68946	4.48353	4.48892	A1-	hAT-like transposase family (hobo/Ac/Tam3)
AT5G35608	14.9926	-3.7096	0.49172	-7.5441	4.56E-14	1.40E-12	0	0.58899	0	4.32498	4.48353	5.64497	A1-	hAT-like transposase family (hobo/Ac/Tam3)
AT5G35608	9.67593	-2.3357	0.59205	-3.9452	7.97E-05	6.50E-04	0	0.62641	0	0	5.22024	4.4747	A1+	hAT-like transposase family (hobo/Ac/Tam3)
AT1G37063	5.99298	-2.6602	0.59219	-4.4922	7.05E-06	7.22E-05	0	0	0	4.6268	3.04536	2.58406	A1+	hAT-like transposase family (hobo/Ac/Tam3), putative
AT3G45270	59.0437	-5.5634	0.48964	-11.362	6.44E-30	6.08E-28	0	0	1.16147	7.60018	6.20076	6.46654	A1+	hAT-like transposase family (hobo/Ac/Tam3), putative
AT3G45270	23.0411	-4.2095	0.46052	-9.1407	6.20E-20	2.88E-18	0	0	1.10158	5.3318	6.02485	5.11744	A1-	hAT-like transposase family (hobo/Ac/Tam3), putative
AT5G34853	142.972	5.68277	0.39542	14.3715	7.81E-47	1.31E-44	8.15736	8.21413	8.08748	0	2.66509	1.01552	A1-	MUG8 MUSTANG 8 Encodes a member of a domesticated transposable element gene family MUSTANG. Members of this family are derived from transposable elements genes but gained function in plant fitness and flower development. Known members include: AT3G04605 (MUG1), AT2G30640 (MUG2), AT1G06740 (MUG3), AT5G16505 (MUG4), AT3G06940 (MUG5), AT5G48965 (MUG6), AT3G05850 (MUG7) and AT5G34853
AT5G34853	153.539	5.96887	0.43779	13.6342	2.51E-42	4.57E-40	8.26496	8.32268	8.19729	1.42937	0	1.16932	A1+	MUG8 MUSTANG 8 Encodes a member of a domesticated transposable element gene family MUSTANG. Members of this family are derived from transposable elements genes but gained function in plant fitness and flower development. Known members include:

														AT3G04605 (MUG1), AT2G30640 (MUG2), AT1G06740 (MUG3), AT5G16505 (MUG4), AT3G06940 (MUG5), AT5G48965 (MUG6), AT3G05850 (MUG7) and AT5G34853
AT1G35995	91.8519	-4.9595	0.54811	-9.0483	1.45E-19	7.16E-18	0.64015	0.62641	0	4.02145	7.80415	8.29124	A1+	Mutator-like transposase family
AT1G36085	49.6259	-5.6468	0.43917	-12.858	7.77E-38	8.95E-36	0	0	0	6.97688	6.60241	6.27841	A1-	Mutator-like transposase family
AT1G36085	34.0077	-4.6611	0.55391	-8.4148	3.93E-17	1.58E-15	0	0	0	3.86258	6.57474	6.60256	A1+	Mutator-like transposase family
AT1G36085	8.13537	-0.6291	0.14102	-4.461	8.16E-06	7.09E-04	0	0	0	3.8418	4.55821	3.79855	A2+	Mutator-like transposase family
AT1G67240	107.037	-6.7687	0.4306	-15.719	1.11E-55	3.72E-53	0	0.62641	0.69459	7.76647	7.65633	7.8111	A1+	Mutator-like transposase family
AT1G67240	31.6153	-4.603	0.45182	-10.188	2.25E-24	1.38E-22	0	0.58899	0.65347	5.19788	6.43434	6.09651	A1-	Mutator-like transposase family
AT1G78350	12.2923	-3.4792	0.58121	-5.9861	2.15E-09	3.86E-08	0	0	0	2.60404	5.31093	4.95315	A1+	Mutator-like transposase family
AT2G04310	7.70773	-3.1267	0.57464	-5.4411	5.30E-08	7.73E-07	0	0	0.69459	4.29471	3.95523	3.75389	A1+	Mutator-like transposase family
AT2G04310	3.41316	-1.7399	0.50668	-3.4339	0.000595	0.004578	0	0	0.65347	3.54983	0	3.34975	A1-	Mutator-like transposase family
AT2G10955	8.02441	-2.8498	0.51357	-5.5489	2.87E-08	4.95E-07	0	0	0	3.93374	4.7925	3.02706	A1-	Mutator-like transposase family
AT2G12066	11.9512	-3.9857	0.5602	-7.1148	1.12E-12	3.04E-11	0	0	0	4.89656	4.6545	4.30275	A1+	Mutator-like transposase family
AT2G12066	5.60937	-2.6462	0.51493	-5.1389	2.76E-07	4.15E-06	0	0	0	3.54983	3.54662	3.72897	A1-	Mutator-like transposase family
AT2G14570	14.4131	-3.9679	0.49076	-8.0852	6.21E-16	2.23E-14	0	0	0	4.88574	4.08983	5.42247	A1-	Mutator-like transposase family
AT2G15810	235.696	-3.5939	0.46032	-7.8074	5.84E-15	1.93E-13	3.63236	4.59651	2.62211	8.41524	9.40837	8.47788	A1-	Mutator-like transposase family
AT2G15810	195.457	-2.0378	0.17882	-11.396	4.38E-30	2.53E-27	4.43461	5.43537	3.36637	8.50864	8.77904	8.22895	A2+	Mutator-like transposase family
AT2G23500	17.5469	-4.2204	0.5399	-7.8171	5.41E-15	1.82E-13	0.64015	0	0	5.69485	5.01973	4.55355	A1+	Mutator-like transposase family
AT2G23500	8.46351	-3.0307	0.50744	-5.9725	2.34E-09	4.65E-08	0.60238	0	0	4.48688	2.66509	4.61457	A1-	Mutator-like transposase family
AT2G23720	57.5032	-5.8611	0.43288	-13.54	9.10E-42	1.28E-39	0	0	0	7.19178	6.75292	6.55411	A1-	Mutator-like transposase family
AT2G23720	42.3502	-4.9169	0.54771	-8.9772	2.78E-19	1.35E-17	0	0	0	4.16454	6.90096	6.90281	A1+	Mutator-like transposase family
AT2G23720	10.5937	-0.733	0.14792	-4.9554	7.22E-07	7.62E-05	0	0	0	4.35716	5.00689	3.79855	A2+	Mutator-like transposase family
AT3G29695	7.75274	-3.2835	0.58204	-5.6413	1.69E-08	2.64E-07	0	0	0	4.52436	3.95523	3.45845	A1+	Mutator-like transposase family
AT3G30170	266.528	-7.9231	0.40931	-19.357	1.78E-83	1.83E-80	0.64015	0	0.69459	9.29351	8.86219	8.98903	A1+	Mutator-like transposase family
AT3G30170	63.2888	-5.8334	0.40702	-14.332	1.38E-46	2.26E-44	0.60238	0	0.65347	7.09462	6.82268	7.04187	A1-	Mutator-like transposase family
AT3G30170	10.9937	-0.7899	0.15564	-5.075	3.88E-07	4.26E-05	0.95419	0	1.02486	4.35716	4.62254	4.45038	A2+	Mutator-like transposase family
AT3G30585	9.01235	-2.5231	0.51267	-4.9214	8.59E-07	1.20E-05	0	0	1.10158	2.52621	4.7925	4.48892	A1-	Mutator-like transposase family
AT3G31450	11.3532	-3.9189	0.56259	-6.9658	3.27E-12	8.36E-11	0	0	0	4.89656	4.34682	4.39129	A1+	Mutator-like transposase family
AT3G31450	9.6753	-3.4403	0.50302	-6.8392	7.96E-12	2.01E-10	0	0	0	4.56149	4.08983	4.35128	A1-	Mutator-like transposase family
AT3G31909	9.82124	-3.7376	0.56817	-6.5783	4.76E-11	1.07E-09	0	0	0	4.52436	4.16427	4.39129	A1+	Mutator-like transposase family
AT3G31909	4.20811	-2.2762	0.51349	-4.4327	9.31E-06	0.000108	0	0	0	3.54983	2.66509	3.34975	A1-	Mutator-like transposase family
AT3G33160	16.8562	-4.2394	0.48003	-8.8315	1.03E-18	4.46E-17	0	0	0	5.10115	5.04684	5.19996	A1-	Mutator-like transposase family
AT3G33160	14.9701	-2.9627	0.59143	-5.0093	5.46E-07	6.83E-06	0	0	0	6.20578	3.95523	1.80658	A1+	Mutator-like transposase family
AT3G33377	7.38653	-2.9495	0.58149	-5.0723	3.93E-07	5.02E-06	0	0.62641	0	4.16454	4.34682	3.08651	A1+	Mutator-like transposase family
AT3G33377	12.2931	-2.6127	0.45498	-5.7423	9.34E-09	2.75E-07	0	0.55795	0	5.66912	4.13223	2.97594	A2-	Mutator-like transposase family
AT3G33377	13.0092	-2.5868	0.51496	-5.0233	5.08E-07	7.32E-06	0	0.58899	0	3.22203	6.02485	2.61074	A1-	Mutator-like transposase family
AT3G34299	7.38653	-2.9495	0.58149	-5.0723	3.93E-07	5.02E-06	0	0.62641	0	4.16454	4.34682	3.08651	A1+	Mutator-like transposase family
AT3G34299	11.6003	-2.608	0.45493	-5.7328	9.88E-09	2.90E-07	0	0.55795	0	5.54616	4.13223	2.97594	A2-	Mutator-like transposase family
AT3G34299	11.9485	-2.4847	0.51514	-4.8234	1.41E-06	1.90E-05	0	0.58899	0	3.22203	5.90133	2.34668	A1-	Mutator-like transposase family
AT3G42535	13.4083	-3.3562	0.48037	-6.9867	2.81E-12	7.44E-11	1.02605	0	0.65347	4.82646	5.04684	4.35128	A1-	Mutator-like transposase family
AT3G42712	8.29102	-2.6126	0.51143	-5.1084	3.25E-07	4.83E-06	1.02605	0	0	3.02518	4.08983	4.73014	A1-	Mutator-like transposase family
AT3G42712	9.94852	-2.3971	0.59211	-4.0484	5.16E-05	0.000438	1.08201	0	0	5.5973	1.77337	3.28443	A1+	Mutator-like transposase family
AT4G08720	27.1456	-4.8013	0.49713	-9.6579	4.55E-22	2.68E-20	0	1.06174	0	5.49267	6.05094	5.74041	A1+	Mutator-like transposase family
AT4G08720	19.0377	-4.1202	0.46358	-8.8877	6.24E-19	2.73E-17	0	1.00603	0	5.3318	5.04684	5.42247	A1-	Mutator-like transposase family
AT4G09380	56.7942	-5.579	0.41931	-13.305	2.15E-40	2.79E-38	0.60238	0.58899	0	7.17998	6.75292	6.48996	A1-	Mutator-like transposase family
AT4G09380	44.0994	-4.7767	0.53143	-8.9884	2.51E-19	1.22E-17	0.64015	0.62641	0	4.4141	6.98599	6.88767	A1+	Mutator-like transposase family
AT4G09380	12.8135	-0.7954	0.15483	-5.1374	2.79E-07	3.10E-05	0.95419	0.94033	0	4.6205	5.22975	3.99111	A2+	Mutator-like transposase family
AT4G28970	226.584	-7.6653	0.38925	-19.692	2.50E-86	1.69E-83	0	0	0	9.06119	8.7784	8.60445	A1-	Mutator-like transposase family
AT4G28970	118.313	-2.889	0.59238	-4.877	1.08E-06	1.28E-05	0	0	0	5.25748	8.49729	8.29124	A1+	Mutator-like transposase family
AT4G28970	26.5277	-1.3574	0.17177	-7.9021	2.74E-15	7.29E-13	0	0	0	5.35808	6.31143	5.38306	A2+	Mutator-like transposase family
AT5G15995	17.1044	-2.5593	0.59121	-4.3289	1.50E-05	0.000144	0.64015	0	1.16147	0	5.62557	5.74041	A1+	Mutator-like transposase family
AT5G35791	5.52799	-2.5727	0.58888	-4.3688	1.25E-05	1.22E-04	0.64015	0	0	3.68404	3.95523	2.85705	A1+	Mutator-like transposase family
AT5G35792	30.3568	-5.1538	0.52308	-9.8528	6.66E-23	4.19E-21	0	0	0	5.31997	6.49961	5.77373	A1+	Mutator-like transposase family
AT5G35792	8.38814	-3.2423	0.5077	-6.3862	1.70E-10	3.75E-09	0	0	0	4.4082	3.54662	4.35128	A1-	Mutator-like transposase family
AT5G44416	34.5866	-5.1646	0.45334	-11.392	4.57E-30	3.78E-28	0	0	0	6.49603	6.02485	5.7852	A1-	Mutator-like transposase family
AT5G44416	23.8182	-4.2866	0.56235	-7.6226	2.49E-14	7.83E-13	0	0	0	3.48023	6.05094	6.09697	A1+	Mutator-like transposase family
AT3G42353	7.59139	-3.282	0.58173	-5.6418	1.68E-08	2.63E-07	0	0	0	4.29471	4.16427	3.45845	A1+	Mutator-like transposase pseudogene

AT1G25430	133.452	-3.9235	0.37613	-10.431	1.79E-25	1.33E-23	3.22918	4.15829	3.44556	6.83858	8.23312	8.46449	A1+	non-LTR retrotransposon family (LINE)
AT1G25430	51.7972	-2.7014	0.34318	-7.8718	3.50E-15	1.18E-13	3.13312	4.05571	3.34584	7.00389	6.43434	6.05278	A1-	non-LTR retrotransposon family (LINE)
AT1G41840	10.1579	-3.5772	0.57569	-6.2138	5.17E-10	1.00E-08	0	0	0	5.12374	3.71068	3.99899	A1+	non-LTR retrotransposon family (LINE)
AT1G41840	7.0668	-2.9393	0.51223	-5.7381	9.57E-09	1.75E-07	0	0	0	3.81676	4.08983	3.83605	A1-	non-LTR retrotransposon family (LINE)
AT2G10820	6.82327	-2.736	0.51438	-5.319	1.04E-07	1.67E-06	0	0	0	3.22203	4.48353	3.61329	A1-	non-LTR retrotransposon family (LINE)
AT2G11410	17.6386	-4.3205	0.47826	-9.0338	1.66E-19	7.51E-18	0	0	0	5.3318	4.7925	5.35204	A1-	non-LTR retrotransposon family (LINE)
AT2G11410	5.11913	-2.7421	0.59122	-4.6381	3.52E-06	3.83E-05	0	0	0	3.86258	3.41604	3.08651	A1+	non-LTR retrotransposon family (LINE)
AT2G12650	34.8496	-4.9109	0.43635	-11.254	2.20E-29	1.78E-27	0.60238	0.58899	0	6.22475	6.34234	5.7852	A1-	non-LTR retrotransposon family (LINE)
AT2G12650	25.7452	-4.7166	0.50031	-9.4272	4.21E-21	2.30E-19	0.64015	0.62641	0	5.78621	5.94164	5.31183	A1+	non-LTR retrotransposon family (LINE)
AT2G15510	13.4425	-3.4624	0.47755	-7.2504	4.15E-13	1.17E-11	1.02605	0	0.65347	5.05024	4.48353	4.73014	A1-	non-LTR retrotransposon family (LINE)
AT3G32043	102.874	-6.8518	0.4444	-15.418	1.24E-53	3.93E-51	0.64015	0	0	7.69754	7.75654	7.61363	A1+	non-LTR retrotransposon family (LINE)
AT3G32043	77.496	-6.1828	0.40877	-15.125	1.10E-51	2.16E-49	0.60238	0	0	7.27173	7.42597	7.13918	A1-	non-LTR retrotransposon family (LINE)
AT3G43546	6.60094	-2.7196	0.51158	-5.3161	1.06E-07	1.69E-06	0	0.58899	0	3.81676	3.54662	4.02897	A1-	non-LTR retrotransposon family (LINE)
AT3G43573	18.1765	-4.0627	0.55212	-7.3584	1.86E-13	5.39E-12	0	0.62641	0	6.03001	4.6545	4.39129	A1+	non-LTR retrotransposon family (LINE)
AT3G43573	4.30314	-2.0087	0.51363	-3.9107	9.20E-05	0.000879	0	0.58899	0	3.39522	3.54662	2.61074	A1-	non-LTR retrotransposon family (LINE)
AT3G43575	8.24771	-3.163	0.50952	-6.2079	5.37E-10	1.13E-08	0	0	0	3.93374	3.54662	4.67351	A1-	non-LTR retrotransposon family (LINE)
AT5G13475	74.1744	-3.7723	0.57942	-6.5105	7.49E-11	1.63E-09	0	1.06174	0.69459	1.42937	7.69051	7.88919	A1+	non-LTR retrotransposon family (LINE)
AT5G28523	104.744	-4.0316	0.57632	-6.9955	2.64E-12	6.84E-11	0.64015	0.62641	0.69459	1.42937	8.17394	8.40193	A1+	non-LTR retrotransposon family (LINE)
AT5G37665	8.59378	-2.6437	0.59109	-4.4727	7.73E-06	7.82E-05	0	0.62641	0	1.42937	4.34682	4.95315	A1+	non-LTR retrotransposon family (LINE)
AT5G38365	5.71895	-2.6008	0.58828	-4.4211	9.82E-06	9.75E-05	0	0	0.69459	2.9585	4.16427	3.45845	A1+	non-LTR retrotransposon family (LINE)
AT5G38365	7.82175	-0.5391	0.13622	-3.9575	7.57E-05	5.43E-03	0	0	1.02486	3.49317	4.68412	3.57627	A2+	non-LTR retrotransposon family (LINE)
AT2G01840	96.6059	-5.9984	0.40645	-14.758	2.74E-49	4.93E-47	0.60238	1.00603	0	7.58062	8.0668	6.92544	A1-	non-LTR retrotransposon family (LINE), putative
AT2G01840	36.4172	-4.894	0.49059	-9.9758	1.95E-23	1.29E-21	0.64015	1.06174	0	6.20578	6.68057	5.44189	A1+	non-LTR retrotransposon family (LINE), putative
AT2G01840	40.7759	-1.7467	0.17868	-9.7756	1.43E-22	5.87E-20	0.95419	1.50483	0	6.05887	6.8028	6.05674	A2+	non-LTR retrotransposon family (LINE), putative
AT4G07355	27.1973	-4.8865	0.46016	-10.619	2.43E-26	1.66E-24	0	0	0	5.56729	5.76624	6.00768	A1-	non-LTR retrotransposon family (LINE), putative
AT4G02960	21.1437	-4.0561	0.48217	-8.4121	4.03E-17	1.62E-15	0.64015	1.06174	1.16147	5.19216	5.55311	5.44189	A1+	RE2, retro element 2, a copia-type retrotransposon element containing LTRs and encoding a polyprotein. This retro element exists in two loci in Landsberg erecta but only once in Columbia
AT4G02960	14.9494	-3.3134	0.46672	-7.0994	1.25E-12	3.41E-11	0.60238	1.00603	1.10158	4.4082	5.04684	5.15929	A1-	RE2, retro element 2, a copia-type retrotransposon element containing LTRs and encoding a polyprotein. This retro element exists in two loci in Landsberg erecta but only once in Columbia
AT3G30820	44.4028	-5.6818	0.48183	-11.792	4.29E-32	4.70E-30	0.64015	0	0	6.70447	6.15253	6.54583	A1+	retrotransposon ORF-1 protein
AT3G30820	39.6412	-5.2505	0.43571	-12.051	1.93E-33	1.81E-31	0.60238	0	0	6.10514	6.52082	6.31589	A1-	retrotransposon ORF-1 protein
AT3G42658	74.3439	-5.0277	0.37493	-13.41	5.32E-41	8.93E-39	2.12133	1.66671	1.79647	7.42645	7.32753	6.76048	A1+	SADHU NON-CODING RETROTRANSPOSON 3-2, SADHU3-2
AT3G42658	56.4702	-4.6664	0.35275	-13.229	6.00E-40	7.63E-38	2.0389	1.593	1.71887	6.99045	6.52082	6.85079	A1-	SADHU NON-CODING RETROTRANSPOSON 3-2, SADHU3-2
AT3G02515	111.494	-3.9218	0.2421	-16.199	5.10E-59	1.27E-56	3.63236	3.53456	3.82555	7.72557	7.74622	7.71723	A1-	SADHU NON-CODING RETROTRANSPOSON 6-1, SADHU6-1
AT3G02515	105.517	-3.7622	0.25947	-14.499	1.22E-47	2.91E-45	3.73192	3.63438	3.92825	7.75521	7.75654	7.37397	A1+	SADHU NON-CODING RETROTRANSPOSON 6-1, SADHU6-1
AT3G31442	9.71336	-3.179	0.56518	-5.6247	1.86E-08	2.89E-07	0	1.06174	0	3.68404	4.78679	4.30275	A1+	SADHU NON-CODING RETROTRANSPOSON 7-2, SADHU7-2
AT3G31442	3.86536	-1.7507	0.51232	-3.4171	0.000633	0.004819	0	1.00603	0	3.54983	2.66509	2.83386	A1-	SADHU NON-CODING RETROTRANSPOSON 7-2, SADHU7-2
AT1G50735	122.615	-6.8548	0.47925	-14.303	2.10E-46	4.35E-44	0	0	0	7.11016	8.54478	7.82706	A1+	SADHU NON-CODING RETROTRANSPOSON 8-1, SADHU8-1
AT1G50735	18.8918	-1.1245	0.16576	-6.7841	1.17E-11	2.03E-09	0	0	0	4.94274	5.80184	4.89772	A2+	SADHU NON-CODING RETROTRANSPOSON 8-1, SADHU8-1
AT2G10010	31.796	-5.1034	0.45245	-11.279	1.66E-29	1.35E-27	0	0	0	5.83129	6.13862	6.05278	A1-	TNP2-like transposon protein, putative
AT2G10010	6.56377	-2.9673	0.58869	-5.0404	4.64E-07	5.87E-06	0	0	0	4.29471	3.95523	2.85705	A1+	TNP2-like transposon protein, putative
AT1G23930	12.946	-3.766	0.49581	-7.5956	3.06E-14	9.62E-13	0	0	0	5.24392	4.48353	4.35128	A1-	transposable element gene
AT1G34590	229.856	-2.7306	0.59224	-4.6105	4.02E-06	4.31E-05	0	0	0.69459	5.95323	9.43103	9.29756	A1+	transposable element gene
AT1G34590	23.2729	-1.1957	0.16852	-7.0955	1.29E-12	2.43E-10	0	0	1.02486	5.39372	6.12416	4.89772	A2+	transposable element gene
AT1G36763	8.38204	-3.1254	0.50931	-6.1366	8.43E-10	1.75E-08	0	0	0	4.14258	4.48353	3.72897	A1-	transposable element gene
AT1G36763	6.22837	-2.4603	0.59204	-4.1557	3.24E-05	2.88E-04	0	0	0	4.89656	2.54524	2.24707	A1+	transposable element gene
AT1G38194	11.9448	-3.8622	0.5666	-6.8164	9.34E-12	2.28E-10	0	0	0	5.25748	4.16427	4.20843	A1+	transposable element gene
AT1G38194	10.2319	-3.1309	0.50981	-6.1413	8.18E-10	1.70E-08	0	0	0	3.54983	5.26298	3.83605	A1-	transposable element gene
AT1G40115	23.7967	-4.5161	0.47289	-9.5501	1.30E-21	6.73E-20	0	0	0	5.41464	6.13862	5.02993	A1-	transposable element gene
AT1G40115	16.547	-4.279	0.55369	-7.7281	1.09E-14	3.56E-13	0	0	0	5.6469	5.01973	4.30275	A1+	transposable element gene
AT1G40125	85.911	-3.9421	0.58262	-6.7663	1.32E-11	3.15E-10	0	0	0	5.49267	7.9664	7.80306	A1+	transposable element gene
AT1G40230	145.703	-7.4001	0.45121	-16.401	1.89E-60	7.97E-58	0	0	0	8.46436	8.0739	7.99186	A1+	transposable element gene
AT1G40230	12.3841	-0.7408	0.14801	-5.0049	5.59E-07	5.97E-05	0	0	0	4.35716	5.42276	3.79855	A2+	transposable element gene
AT1G42367	13.0167	-3.8462	0.49188	-7.8194	5.31E-15	1.76E-13	0	0	0	4.63244	4.7925	4.83714	A1-	transposable element gene
AT1G42367	11.1526	-3.5293	0.5787	-6.0986	1.07E-09	2.00E-08	0	0	0	5.43737	3.71068	3.75389	A1+	transposable element gene

AT1G43590	68.2332	-3.5001	0.32324	-10.828	2.53E-27	2.05E-25	3.84837	3.35661	2.41385	7.12774	6.84135	7.03235	A1+	transposable element gene
AT2G04000	20.4361	-4.7314	0.53416	-8.8576	8.17E-19	3.78E-17	0	0	0	5.6469	5.47681	4.95315	A1+	transposable element gene
AT2G04000	13.2261	-3.8221	0.49234	-7.7633	8.28E-15	2.71E-13	0	0	0	4.63244	5.04684	4.61457	A1-	transposable element gene
AT2G04320	36.2758	-5.2936	0.4988	-10.613	2.60E-26	2.00E-24	0.64015	0	0	6.20578	6.57474	5.67137	A1+	transposable element gene
AT2G04320	23.0048	-4.5371	0.45884	-9.888	4.69E-23	2.66E-21	0.60238	0	0	5.63796	5.45092	5.55378	A1-	transposable element gene
AT2G04330	4.58826	-2.5174	0.59239	-4.2496	2.14E-05	0.000198	0	0	0	2.9585	3.95523	2.85705	A1+	transposable element gene
AT2G04330	2.50715	-1.5918	0.4909	-3.2425	0.001185	0.008308	0	0	0	2.19233	2.66509	2.83386	A1-	transposable element gene
AT2G06140	8.31488	-3.2041	0.50797	-6.3076	2.83E-10	6.15E-09	0	0	0	4.04193	4.08983	4.2772	A1-	transposable element gene
AT2G06480	32.3257	-5.0937	0.45462	-11.204	3.88E-29	3.10E-27	0	0	0	5.70532	6.02485	6.31589	A1-	transposable element gene
AT2G06914	6.44188	-2.6962	0.51474	-5.238	1.62E-07	2.53E-06	0	0	0	4.04193	4.08983	3.02706	A1-	transposable element gene
AT2G10070	11.1925	-2.7044	0.59135	-4.5733	4.80E-06	5.07E-05	0.64015	0	0	5.78621	1.77337	3.45845	A1+	transposable element gene
AT2G10285	17.5448	-4.1177	0.4863	-8.4675	2.51E-17	1.00E-15	0	0	0	5.56729	5.26298	4.48892	A1-	transposable element gene
AT2G12320	14.8367	-3.8899	0.49076	-7.9263	2.26E-15	7.74E-14	0	0	0	4.63244	5.45092	4.55311	A1-	transposable element gene
AT2G12320	9.54727	-3.138	0.58764	-5.34	9.30E-08	1.31E-06	0	0	0	5.31997	3.71068	2.85705	A1+	transposable element gene
AT2G12460	26.9246	-3.4243	0.48858	-7.0088	2.40E-12	6.25E-11	1.92313	1.66671	1.51363	4.29471	6.29264	5.8998	A1+	transposable element gene
AT2G12460	39.9724	-1.1146	0.17027	-6.5458	5.92E-11	9.60E-09	2.50778	2.22523	2.03713	4.94274	7.04503	6.09917	A2+	transposable element gene
AT2G12520	36.0449	-4.99	0.52179	-9.5633	1.14E-21	6.47E-20	0.64015	0	0	5.05192	6.87146	6.09697	A1+	transposable element gene
AT2G13070	5.45454	-2.5744	0.59237	-4.3459	1.39E-05	1.34E-04	0	0	0	2.1331	4.34682	3.45845	A1+	transposable element gene
AT2G13320	23.026	-4.4942	0.55204	-8.1411	3.92E-16	1.45E-14	0	0	0	4.02145	5.94164	5.98762	A1+	transposable element gene
AT2G13320	7.40125	-2.7238	0.51452	-5.2939	1.20E-07	1.90E-06	0	0	0	3.39522	4.7925	3.19741	A1-	transposable element gene
AT2G13431	54.833	-4.2908	0.35028	-12.25	1.69E-34	1.67E-32	2.74425	1.32917	1.95037	6.90706	6.43434	6.85079	A1-	transposable element gene
AT2G13431	45.3826	-3.9923	0.384	-10.397	2.57E-25	1.89E-23	2.83661	1.3957	2.03285	6.50374	6.61089	6.24686	A1+	transposable element gene
AT2G14240	37.9469	-5.3499	0.49818	-10.739	6.68E-27	5.35E-25	0	0.62641	0	6.70447	6.15253	5.77373	A1+	transposable element gene
AT2G14240	9.59988	-0.7331	0.15048	-4.8719	1.11E-06	0.000114	0	0.94033	0	4.28307	4.34599	4.31292	A2+	transposable element gene
AT2G14730	112.496	-6.8047	0.40564	-16.775	3.72E-63	1.06E-60	0	0	0	7.93652	7.85009	7.66008	A1-	transposable element gene
AT2G14730	75.1087	-6.2963	0.49124	-12.817	1.32E-37	1.87E-35	0	0	0	7.86407	6.81059	6.76048	A1+	transposable element gene
AT2G14730	10.7563	-0.6975	0.14543	-4.796	1.62E-06	1.61E-04	0	0	0	3.61898	5.14469	4.31292	A2+	transposable element gene
AT2G15550	10.0914	-2.9058	0.51367	-5.657	1.54E-08	2.74E-07	0	0	0	2.52621	5.26298	4.2772	A1-	transposable element gene
AT2G15800	58.5332	-3.4701	0.41551	-8.3513	6.75E-17	2.65E-15	3.22918	3.19469	2.03285	7.46844	5.88373	6.62098	A1+	transposable element gene
AT2G15800	58.3215	-3.2178	0.41146	-7.8205	5.26E-15	1.74E-13	3.13312	3.0981	1.95037	5.83129	7.70987	6.09651	A1-	transposable element gene
AT2G15815	7.46599	-3.0141	0.57929	-5.2032	1.96E-07	2.64E-06	0	0	0.69459	4.52436	3.41604	3.75389	A1+	transposable element gene
AT2G22710	22.2255	-2.7572	0.39335	-7.0094	2.39E-12	6.36E-11	2.50182	2.46929	1.71887	5.3318	5.45092	5.35204	A1-	transposable element gene
AT2G22710	25.2579	-2.5996	0.50996	-5.0976	3.44E-07	4.43E-06	2.5913	2.55913	1.79647	6.58232	4.34682	4.6994	A1+	transposable element gene
AT2G23480	15	-4.0434	0.54391	-7.434	1.05E-13	3.14E-12	0.64015	0	0	4.81212	5.39625	4.4747	A1+	transposable element gene
AT2G23480	10.9927	-3.4449	0.4943	-6.9693	3.18E-12	8.36E-11	0.60238	0	0	4.56149	4.48353	4.48892	A1-	transposable element gene
AT2G23710	6.0183	-2.535	0.59236	-4.2795	1.87E-05	1.76E-04	0	0	0	1.42937	4.16427	4.20843	A1+	transposable element gene
AT3G15310	13.4089	1.85232	0.50741	3.65056	2.62E-04	1.87E-03	4.15013	4.57509	4.88007	2.60404	2.54524	1.80658	A1+	transposable element gene
AT3G15310	10.5896	1.86606	0.44339	4.20861	2.57E-05	0.000364	3.95651	4.38068	4.68535	0	2.10673	1.10072	A2-	transposable element gene
AT3G15310	10.513	2.70842	0.50523	5.36074	8.29E-08	1.35E-06	4.04846	4.47093	4.77376	0	0	0	A1-	transposable element gene
AT3G29610	8.69238	-3.554	0.57392	-6.1924	5.92E-10	1.14E-08	0	0	0	4.02145	4.16427	4.39129	A1+	transposable element gene
AT3G29610	4.80318	-2.427	0.51476	-4.7147	2.42E-06	3.11E-05	0	0	0	3.93374	2.66509	3.34975	A1-	transposable element gene
AT3G29700	34.8987	-5.5085	0.50553	-10.896	1.20E-27	9.93E-26	0	0	0	6.06692	6.42035	5.8998	A1+	transposable element gene
AT3G29700	20.1159	-4.494	0.47283	-9.5044	2.01E-21	1.03E-19	0	0	0	5.63796	5.04684	5.35204	A1-	transposable element gene
AT3G29710	5.24332	-2.5056	0.58989	-4.2476	2.16E-05	0.0002	0	0.62641	0	3.48023	3.95523	2.85705	A1+	transposable element gene
AT3G30360	7.93092	-2.7773	0.51456	-5.3975	6.76E-08	1.11E-06	0	0	0	2.52621	4.48353	4.48892	A1-	transposable element gene
AT3G30670	34.9298	-5.1768	0.52783	-9.8078	1.04E-22	6.50E-21	0	0	0	4.97633	6.64615	6.33878	A1+	transposable element gene
AT3G30690	9.28958	-3.3181	0.50544	-6.5648	5.21E-11	1.23E-09	0	0	0	4.32498	4.48353	4.02897	A1-	transposable element gene
AT3G30690	8.90178	-2.9016	0.59105	-4.9092	9.15E-07	1.10E-05	0	0	0	5.25748	1.77337	3.88164	A1+	transposable element gene
AT3G30700	10.1409	-3.4795	0.50168	-6.9357	4.04E-12	1.05E-10	0	0	0	4.32498	4.48353	4.42174	A1-	transposable element gene
AT3G30700	7.08828	-2.5062	0.59217	-4.2323	2.31E-05	2.13E-04	0	0	0	5.12374	1.77337	2.85705	A1+	transposable element gene
AT3G31310	36.36	-5.3266	0.44542	-11.959	5.84E-33	5.44E-31	0	0	0	6.26995	6.13862	6.20038	A1-	transposable element gene
AT3G31540	115.292	-7.0701	0.46082	-15.342	3.98E-53	1.25E-50	0	0	0	8.19104	7.67352	7.63192	A1+	transposable element gene
AT3G31540	46.8943	-5.4218	0.44915	-12.071	1.50E-33	1.41E-31	0	0	0	5.80081	6.82268	6.85079	A1-	transposable element gene
AT3G31540	10.4727	-0.8052	0.15257	-5.278	1.31E-07	1.49E-05	0	0	0	4.67946	4.34599	4.31292	A2+	transposable element gene
AT3G31915	5.50125	-2.5887	0.59238	-4.37	1.24E-05	1.21E-04	0	0	0	3.68404	1.77337	4.30275	A1+	transposable element gene
AT3G31955	7.34898	-3.277	0.58198	-5.6308	1.79E-08	2.79E-07	0	0	0	4.16454	3.41604	4.20843	A1+	transposable element gene
AT3G32070	36.9679	-5.5846	0.50305	-11.101	1.24E-28	1.11E-26	0	0	0	6.17229	6.49961	5.95894	A1+	transposable element gene

AT3G32070	22.9124	-4.6182	0.46787	-9.8706	5.58E-23	3.14E-21	0	0	0	5.53062	5.76624	5.3155	A1-	transposable element gene
AT3G32897	10.4302	-3.1856	0.50874	-6.2618	3.81E-10	8.14E-09	0	0	0	3.68946	5.26298	3.83605	A1-	transposable element gene
AT3G32897	14.5127	-1.0054	0.16187	-6.2111	5.26E-10	7.57E-08	0	0	0	4.84311	5.22975	4.57587	A2+	transposable element gene
AT3G33073	77.2854	-5.8552	0.44293	-13.219	6.80E-40	8.56E-38	0	0	0	6.4378	8.00813	6.93752	A1-	transposable element gene
AT3G42090	5.7126	-2.6647	0.58685	-4.5407	5.61E-06	5.83E-05	0	0	0.69459	4.02145	3.41604	3.28443	A1+	transposable element gene
AT3G42090	6.4812	-2.5782	0.51305	-5.0252	5.03E-07	7.25E-06	0	0	0.65347	3.81676	4.08983	3.34975	A1-	transposable element gene
AT3G42110	5.07413	-2.5406	0.58876	-4.3153	1.59E-05	0.000152	0	0	0.69459	3.48023	3.41604	3.45845	A1+	transposable element gene
AT3G42110	5.31914	-2.2315	0.51514	-4.3318	1.48E-05	0.000165	0	0	0.65347	3.02518	4.08983	3.19741	A1-	transposable element gene
AT3G42181	17.3348	-3.5181	0.58331	-6.0313	1.63E-09	2.96E-08	0	0	0	6.33243	4.16427	3.08651	A1+	transposable element gene
AT3G43680	18.1593	-3.8363	0.56416	-6.8	1.05E-11	2.53E-10	0.64015	0	0	6.20578	4.34682	4.10751	A1+	transposable element gene
AT3G43680	12.1643	-3.3884	0.50107	-6.7624	1.36E-11	3.38E-10	0.60238	0	0	4.23666	3.54662	5.48962	A1-	transposable element gene
AT3G43863	275.336	-7.4498	0.37324	-19.959	1.24E-88	9.11E-86	0.60238	0	1.10158	8.70581	9.52804	8.9586	A1-	transposable element gene
AT3G43863	206.737	-7.2789	0.42454	-17.145	6.82E-66	3.58E-63	0.64015	0	1.16147	9.20935	8.33388	8.35317	A1+	transposable element gene
AT3G43863	19.722	-1.0811	0.16744	-6.4568	1.07E-10	1.66E-08	0.95419	0	1.61801	5.42851	5.65579	4.63475	A2+	transposable element gene
AT3G45380	11.2735	-3.6086	0.57603	-6.2647	3.74E-10	7.39E-09	0	0	0	5.37986	3.95523	3.75389	A1+	transposable element gene
AT3G47330	9.55865	-3.6116	0.57316	-6.3011	2.96E-10	5.95E-09	0	0	0	3.68404	4.50885	4.62832	A1+	transposable element gene
AT3G47330	3.49894	-2.0142	0.50849	-3.9611	7.46E-05	0.000725	0	0	0	3.39522	2.66509	2.83386	A1-	transposable element gene
AT3G47330	13.3615	-0.8762	0.15583	-5.6231	1.88E-08	2.30E-06	0	0	0	4.49483	5.34862	4.31292	A2+	transposable element gene
AT4G05633	108.866	-6.6203	0.4138	-15.999	1.30E-57	3.07E-55	0	0	0	7.48801	8.20382	7.50644	A1-	transposable element gene
AT4G06603	48.5154	-3.244	0.347	-9.3487	8.88E-21	4.73E-19	2.83661	3.50217	2.71497	6.77308	6.49961	6.17386	A1+	transposable element gene
AT4G06603	23.2991	-2.1338	0.39248	-5.4366	5.43E-08	9.04E-07	2.74425	3.40322	2.62211	5.05024	5.04684	5.81168	A1-	transposable element gene
AT4G06672	19.5014	-4.7287	0.53253	-8.8798	6.70E-19	3.12E-17	0	0	0	5.25748	5.47681	5.21812	A1+	transposable element gene
AT4G06672	5.24074	-2.5403	0.51514	-4.9312	8.17E-07	1.14E-05	0	0	0	3.39522	3.54662	3.61329	A1-	transposable element gene
AT4G07485	9.23149	-3.0196	0.5896	-5.1215	3.03E-07	3.94E-06	0	0	0	5.37986	3.04536	3.08651	A1+	transposable element gene
AT4G07490	38.7643	-5.0117	0.52466	-9.5521	1.27E-21	7.17E-20	0.64015	0	0	7.12774	5.76042	5.3565	A1+	transposable element gene
AT4G07490	13.885	-0.9063	0.15888	-5.7045	1.17E-08	1.48E-06	0.95419	0	0	4.20496	5.00689	5.12007	A2+	transposable element gene
AT4G07493	18.7585	-3.8215	0.57577	-6.6372	3.20E-11	7.31E-10	0	0	0	6.39182	3.71068	4.20843	A1+	transposable element gene
AT4G07520	9.91491	-3.7246	0.56884	-6.5478	5.84E-11	1.29E-09	0	0	0	4.02145	4.50885	4.55355	A1+	transposable element gene
AT4G08710	19.7545	-4.5002	0.52633	-8.5502	1.23E-17	5.19E-16	0.64015	0	0	4.97633	5.22024	5.7063	A1+	transposable element gene
AT4G08710	18.2425	-4.1122	0.47372	-8.6807	3.93E-18	1.65E-16	0.60238	0	0	4.82646	5.45092	5.3155	A1-	transposable element gene
AT4G09370	7.67045	-2.7286	0.59212	-4.6082	4.06E-06	4.35E-05	0	0	0	1.42937	4.34682	4.6994	A1+	transposable element gene
AT4G09370	4.81418	-2.3101	0.51389	-4.4953	6.95E-06	8.28E-05	0	0	0	3.81676	3.54662	2.61074	A1-	transposable element gene
AT4G09425	43.8942	-4.0301	0.42381	-9.5091	1.92E-21	1.07E-19	1.41979	2.41948	2.03285	6.77308	5.55311	6.65714	A1+	transposable element gene
AT4G22450	6.32778	-2.5044	0.51503	-4.8627	1.16E-06	1.58E-05	0	0	0	4.23666	4.08983	2.34668	A1-	transposable element gene
AT4G28960	9.9206	-2.9342	0.59092	-4.9655	6.85E-07	8.44E-06	0	0	0	1.42937	4.78679	5.01018	A1+	transposable element gene
AT4G28960	7.00887	-2.8315	0.51376	-5.5113	3.56E-08	6.04E-07	0	0	0	4.23666	4.08983	3.19741	A1-	transposable element gene
AT5G27160	15.6024	-3.3364	0.58076	-5.7449	9.20E-09	1.49E-07	0.64015	0	0	2.1331	5.55311	5.48276	A1+	transposable element gene
AT5G27180	14.3943	-3.1968	0.58857	-5.4316	5.59E-08	8.13E-07	0	0	0	1.42937	5.55311	5.31183	A1+	transposable element gene
AT5G28430	54.3953	-2.8357	0.51177	-5.541	3.01E-08	4.55E-07	3.14059	3.19469	2.71497	3.68404	7.04001	7.35207	A1+	transposable element gene
AT5G28430	23.1946	-1.9313	0.42126	-4.5847	4.55E-06	5.58E-05	3.04528	3.0981	2.62211	4.04193	5.90133	5.52205	A1-	transposable element gene
AT5G29408	22.9204	-4.8453	0.53128	-9.1201	7.50E-20	3.74E-18	0	0	0	5.05192	5.99732	5.44189	A1+	transposable element gene
AT5G32312	5.25783	-2.5718	0.51514	-4.9924	5.96E-07	8.52E-06	0	0	0	3.68946	2.66509	3.93573	A1-	transposable element gene
AT5G32312	2.57283	-1.8275	0.57944	-3.1539	1.61E-03	0.009109	0	0	0	2.9585	1.77337	2.85705	A1+	transposable element gene
AT5G32405	6.5581	-2.6166	0.51271	-5.1036	3.33E-07	4.94E-06	0.60238	0	0	3.39522	4.08983	3.83605	A1-	transposable element gene
AT5G32405	4.04979	-2.2074	0.59236	-3.7265	1.94E-04	0.001433	0.64015	0	0	3.48023	2.54524	3.28443	A1+	transposable element gene
AT5G32775	13.198	-3.8176	0.4925	-7.7516	9.08E-15	2.95E-13	0	0	0	4.56149	5.04684	4.67351	A1-	transposable element gene
AT5G33240	18.7149	-4.6375	0.53692	-8.6372	5.76E-18	2.48E-16	0	0	0	4.89656	5.47681	5.3565	A1+	transposable element gene
AT5G33240	14.5364	-4.0413	0.48694	-8.2995	1.05E-16	3.99E-15	0	0	0	5.05024	4.48353	5.11744	A1-	transposable element gene
AT5G35794	5.2729	-2.5997	0.59231	-4.389	1.14E-05	0.000112	0	0	0	2.1331	3.95523	3.88164	A1+	transposable element gene
AT5G35794	4.64567	-2.3146	0.51401	-4.503	6.70E-06	8.00E-05	0	0	0	2.79716	3.54662	3.61329	A1-	transposable element gene
AT5G36650	55.6017	-2.9148	0.59231	-4.9211	8.61E-07	1.04E-05	0	0	0	4.29471	7.19092	7.41678	A1+	transposable element gene
AT5G36650	20.5339	-1.165	0.16691	-6.98	2.95E-12	5.45E-10	0	0	0	4.67946	5.82936	5.45016	A2+	transposable element gene
AT5G37385	49.9665	-5.6667	0.4383	-12.929	3.10E-38	3.67E-36	0	0	0	6.97688	6.60241	6.31589	A1-	transposable element gene
AT5G37385	33.8464	-4.4905	0.56085	-8.0066	1.18E-15	4.19E-14	0	0	0	3.48023	6.61089	6.60256	A1+	transposable element gene
AT5G37385	8.52582	-0.6046	0.13891	-4.3524	1.35E-05	0.001112	0	0	0	3.94151	4.79992	3.45081	A2+	transposable element gene
AT5G37390	6.22648	-2.563	0.59239	-4.3265	1.52E-05	0.000145	0	0	0	1.42937	4.16427	4.30275	A1+	transposable element gene
AT5G37390	2.93195	-1.795	0.50111	-3.582	0.000341	0.002792	0	0	0	3.02518	2.66509	2.61074	A1-	transposable element gene

AT5G38595	13.3667	-3.1445	0.58908	-5.3379	9.40E-08	1.32E-06	0	0	0	1.42937	5.47681	5.16887	A1+	transposable element gene
AT5G38595	9.16134	-2.9719	0.51362	-5.7862	7.20E-09	1.34E-07	0	0	0	4.4082	0	5.15929	A1-	transposable element gene
AT1G38390	4.95143	-2.5112	0.59237	-4.2392	2.24E-05	2.07E-04	0	0	0	4.29471	2.54524	2.85705	A1+	transposable element gene; pseudogene, hypothetical protein
AT1G38390	5.16748	-2.3301	0.51418	-4.5316	5.85E-06	7.07E-05	0	0	0	2.52621	4.08983	3.48753	A1-	transposable element gene; pseudogene, hypothetical protein
AT1G40072	9.4865	-3.2699	0.58465	-5.593	2.23E-08	3.43E-07	0	0	0	5.25748	3.41604	3.45845	A1+	transposable element gene; pseudogene, hypothetical protein
AT1G40076	4.97106	-2.7514	0.59104	-4.6551	3.24E-06	3.55E-05	0	0	0	3.48023	3.41604	3.45845	A1+	transposable element gene; pseudogene, hypothetical protein
AT1G40119	33.676	-4.9383	0.44772	-11.03	2.74E-28	2.06E-26	0.60238	0	0	5.60306	6.43434	6.11789	A1-	transposable element gene; pseudogene, hypothetical protein
AT1G40121	288.464	-8.1302	0.44225	-18.384	1.78E-75	1.20E-72	0	0	0	8.57739	9.44612	9.35372	A1+	transposable element gene; pseudogene, hypothetical protein
AT1G40121	137.563	-7.0067	0.4023	-17.417	6.17E-68	2.06E-65	0	0	0	7.8424	8.3761	8.05867	A1-	transposable element gene; pseudogene, hypothetical protein
AT1G40121	16.9467	-1.1052	0.16542	-6.6812	2.37E-11	4.04E-09	0	0	0	5.03592	5.49326	4.74575	A2+	transposable element gene; pseudogene, hypothetical protein
AT1G40135	57.3646	-5.9346	0.47873	-12.397	2.73E-35	3.52E-33	0.64015	0	0	6.3018	6.9582	7.16383	A1+	transposable element gene; pseudogene, hypothetical protein
AT1G40135	8.24373	-2.7944	0.51106	-5.4678	4.56E-08	7.65E-07	0.60238	0	0	3.02518	4.48353	4.42174	A1-	transposable element gene; pseudogene, hypothetical protein
AT1G40310	396.23	-8.4938	0.43718	-19.429	4.42E-84	4.73E-81	0	0	0	10.1187	9.15318	9.44962	A1+	transposable element gene; pseudogene, hypothetical protein
AT1G40310	95.6513	-6.5165	0.41443	-15.724	1.04E-55	2.32E-53	0	0	0	7.60728	7.85009	7.24013	A1-	transposable element gene; pseudogene, hypothetical protein
AT1G40310	39.7665	-1.7852	0.17818	-10.019	1.25E-23	5.46E-21	0	0	0	6.49608	6.61002	5.74558	A2+	transposable element gene; pseudogene, hypothetical protein
AT1G41680	17.0621	-4.4754	0.54372	-8.231	1.86E-16	7.09E-15	0	0	0	4.6268	5.31093	5.3565	A1+	transposable element gene; pseudogene, hypothetical protein
AT1G41680	10.7641	-3.5691	0.49957	-7.1442	9.05E-13	2.48E-11	0	0	0	4.63244	4.48353	4.35128	A1-	transposable element gene; pseudogene, hypothetical protein
AT1G42040	397.141	-8.6473	0.42637	-20.281	1.88E-91	2.54E-88	0	0	0	9.93373	9.53346	9.381	A1+	transposable element gene; pseudogene, hypothetical protein
AT1G42040	271.15	-6.3458	0.45836	-13.845	1.37E-43	2.05E-41	0	0	0	8.38474	9.72305	8.81545	A1-	transposable element gene; pseudogene, hypothetical protein
AT1G42040	25.9182	-1.474	0.17452	-8.446	3.01E-17	9.32E-15	0	0	0	5.65103	6.05591	5.38306	A2+	transposable element gene; pseudogene, hypothetical protein
AT1G42050	2313.88	-10.648	0.37437	-28.443	6.02E-178	3.09E-174	0.64015	0	0.69459	12.4897	11.9981	11.9812	A1+	transposable element gene; pseudogene, hypothetical protein
AT1G42050	1711.46	-9.5996	0.36481	-26.314	1.31E-152	3.74E-149	0.60238	0	0.65347	11.1121	12.2665	11.6121	A1-	transposable element gene; pseudogene, hypothetical protein
AT1G42050	193.123	-3.3708	0.17599	-19.154	9.04E-82	3.98E-78	0.95419	0	1.02486	8.5248	8.96109	8.19512	A2+	transposable element gene; pseudogene, hypothetical protein
AT1G42360	31.7337	-5.0344	0.45524	-11.059	1.99E-28	1.52E-26	0	0	0	5.67203	6.34234	5.93726	A1-	transposable element gene; pseudogene, hypothetical protein
AT1G42745	30.9731	-4.8381	0.54273	-8.9144	4.90E-19	2.33E-17	0	0	0	4.4141	6.57474	6.17386	A1+	transposable element gene; pseudogene, hypothetical protein
AT1G42745	17.6938	-1.2051	0.16879	-7.14	9.34E-13	1.79E-10	0	0	0	5.03592	5.27047	5.23869	A2+	transposable element gene; pseudogene, hypothetical protein
AT2G04990	6.69155	-2.8776	0.59043	-4.8738	1.09E-06	1.30E-05	0	0	0	4.72243	3.04536	3.08651	A1+	transposable element gene; pseudogene, hypothetical protein
AT2G06180	114.391	-6.446	0.48377	-13.325	1.67E-40	2.74E-38	0	0	0.69459	6.98075	8.61765	7.40619	A1+	transposable element gene; pseudogene, hypothetical protein
AT2G06180	374.445	-3.4877	0.17747	-19.653	5.50E-86	2.93E-82	0	0	1.02486	8.98536	10.1046	9.32368	A2+	transposable element gene; pseudogene, hypothetical protein
AT2G06245	10.8892	-3.5333	0.50022	-7.0635	1.62E-12	4.38E-11	0	0	0	4.4082	4.7925	4.2772	A1-	transposable element gene; pseudogene, hypothetical protein
AT2G06245	10.6154	-3.3103	0.58457	-5.6627	1.49E-08	2.35E-07	0	0	0	5.49267	3.41604	3.45845	A1+	transposable element gene; pseudogene, hypothetical protein
AT2G06330	140.006	-6.8677	0.39601	-17.342	2.27E-67	7.46E-65	0	0	0.65347	7.95739	8.48767	7.87624	A1-	transposable element gene; pseudogene, hypothetical protein
AT2G06335	11.5593	-3.6858	0.49665	-7.4213	1.16E-13	3.45E-12	0	0	0	4.48688	4.48353	4.78464	A1-	transposable element gene; pseudogene, hypothetical protein
AT2G06335	7.77358	-2.7415	0.59207	-4.6304	3.65E-06	3.96E-05	0	0	0	5.19216	2.54524	2.85705	A1+	transposable element gene; pseudogene, hypothetical protein
AT2G06340	21.9675	-4.5651	0.47005	-9.7121	2.68E-22	1.45E-20	0	0	0	5.19788	5.61718	5.61521	A1-	transposable element gene; pseudogene, hypothetical protein
AT2G06340	7.75601	-0.6122	0.13977	-4.3799	1.19E-05	0.001003	0	0	0	4.35716	4.09708	3.57627	A2+	transposable element gene; pseudogene, hypothetical protein
AT2G06370	39.8128	-5.3345	0.52358	-10.188	2.23E-24	1.57E-22	0	0	0	5.12374	6.77917	6.60256	A1+	transposable element gene; pseudogene, hypothetical protein
AT2G06460	28.3596	-4.7968	0.45217	-10.608	2.72E-26	1.85E-24	0	0	0.65347	5.67203	5.61718	6.1802	A1-	transposable element gene; pseudogene, hypothetical protein
AT2G06965	20.6246	-4.6682	0.53866	-8.6663	4.46E-18	1.94E-16	0	0	0	5.87214	5.31093	4.83185	A1+	transposable element gene; pseudogene, hypothetical protein
AT2G06965	13.588	-3.9131	0.49166	-7.9589	1.74E-15	6.02E-14	0	0	0	5.24392	4.08983	4.88781	A1-	transposable element gene; pseudogene, hypothetical protein
AT2G06965	12.5428	-0.8903	0.15678	-5.6785	1.36E-08	1.70E-06	0	0	0	4.20496	4.90711	4.89772	A2+	transposable element gene; pseudogene, hypothetical protein
AT2G07460	31.6153	-4.8998	0.44727	-10.955	6.29E-28	4.65E-26	0	0	0.65347	6.0283	6.24407	5.67413	A1-	transposable element gene; pseudogene, hypothetical protein
AT2G07460	25.6696	-4.8474	0.51293	-9.4504	3.37E-21	1.85E-19	0	0	0.69459	5.19216	5.99732	5.80629	A1+	transposable element gene; pseudogene, hypothetical protein
AT2G09187	931.402	-9.1979	0.36229	-25.389	3.37E-142	7.86E-139	0.60238	0	0	10.6357	11.2447	10.6196	A1-	transposable element gene; pseudogene, hypothetical protein
AT2G09187	62.3167	-2.164	0.18014	-12.013	3.05E-33	2.19E-30	0.95419	0	0	6.75096	7.43362	6.57557	A2+	transposable element gene; pseudogene, hypothetical protein
AT2G09930	42.5387	-5.4347	0.44328	-12.26	1.48E-34	1.48E-32	0	0	0	6.07998	6.75292	6.37035	A1-	transposable element gene; pseudogene, hypothetical protein
AT2G11640	13.1461	-3.8875	0.4912	-7.9144	2.49E-15	8.50E-14	0	0	0	4.99746	4.48353	4.78464	A1-	transposable element gene; pseudogene, hypothetical protein
AT2G11640	10.4065	-3.4166	0.58157	-5.8748	4.23E-09	7.20E-08	0	0	0	5.31997	3.04536	4.10751	A1+	transposable element gene; pseudogene, hypothetical protein
AT2G11650	40.5533	-5.3408	0.44653	-11.961	5.72E-33	5.34E-31	0	0	0	5.9747	6.75292	6.23992	A1-	transposable element gene; pseudogene, hypothetical protein
AT2G11650	31.1527	-5.0745	0.52973	-9.5794	9.76E-22	5.60E-20	0	0	0	6.65684	5.69457	5.21812	A1+	transposable element gene; pseudogene, hypothetical protein
AT2G11650	12.2684	-0.8809	0.15636	-5.6338	1.76E-08	2.18E-06	0	0	0	4.42764	5.0543	4.45038	A2+	transposable element gene; pseudogene, hypothetical protein
AT2G11780	2722.87	-10.371	0.30863	-33.603	1.54E-247	3.95E-243	0.60238	1.00603	1.44299	12.4978	12.5106	12.2039	A1-	transposable element gene; pseudogene, hypothetical protein
AT2G11780	2589.92	-10.326	0.35658	-28.958	2.22E-184	1.42E-180	0.64015	1.06174	1.51363	11.775	12.5923	12.5165	A1+	transposable element gene; pseudogene, hypothetical protein
AT2G11780	157.728	-3.0315	0.17738	-17.091	1.74E-65	4.22E-62	0.95419	1.50483	2.03713	8.25126	8.68049	7.83604	A2+	transposable element gene; pseudogene, hypothetical protein
AT2G12380	28.7905	-5.2212	0.51705	-10.098	5.64E-24	3.86E-22	0	0	0	5.43737	5.94164	6.14868	A1+	transposable element gene; pseudogene, hypothetical protein
AT2G12385	50.9152	-5.0603	0.48895	-10.35	4.21E-25	3.06E-23	0	1.66671	0	5.5973	6.90096	7.11267	A1+	transposable element gene; pseudogene, hypothetical protein
AT2G12540	10.9643	-3.3358	0.54286	-6.1449	8.00E-10	1.51E-08	1.08201	0.62641	0	4.29471	4.50885	4.62832	A1+	transposable element gene; pseudogene, hypothetical protein

AT2G12540	27.2002	-1.3038	0.17252	-7.5569	4.13E-14	9.63E-12	1.52355	0.94033	0	5.03592	6.18932	5.84866	A2+	transposable element gene; pseudogene, hypothetical protein
AT2G12810	9.96363	-3.3112	0.58395	-5.6704	1.42E-08	2.25E-07	0	0	0	5.31997	3.71068	3.28443	A1+	transposable element gene; pseudogene, hypothetical protein
AT2G12810	7.28225	-3.0397	0.51109	-5.9475	2.72E-09	5.34E-08	0	0	0	4.14258	3.54662	4.11654	A1-	transposable element gene; pseudogene, hypothetical protein
AT2G12830	12.496	-3.8585	0.56762	-6.7977	1.06E-11	2.57E-10	0	0	0	5.37986	4.34682	3.99899	A1+	transposable element gene; pseudogene, hypothetical protein
AT2G12830	11.0766	-3.6264	0.49802	-7.2817	3.30E-13	9.41E-12	0	0	0	4.56149	4.48353	4.55311	A1-	transposable element gene; pseudogene, hypothetical protein
AT2G12840	12.4492	-3.7584	0.57186	-6.5723	4.96E-11	1.11E-09	0	0	0	5.49267	4.16427	3.88164	A1+	transposable element gene; pseudogene, hypothetical protein
AT2G12840	11.0485	-3.6211	0.49819	-7.2685	3.63E-13	1.03E-11	0	0	0	4.48688	4.48353	4.61457	A1-	transposable element gene; pseudogene, hypothetical protein
AT2G12910	28.4003	-4.3263	0.47999	-9.0133	2.00E-19	8.99E-18	0	0.58899	0	4.70005	5.45092	6.68879	A1-	transposable element gene; pseudogene, hypothetical protein
AT2G12910	11.5122	-3.3243	0.57614	-5.77	7.93E-09	1.30E-07	0	0.62641	0	5.49267	3.95523	3.45845	A1+	transposable element gene; pseudogene, hypothetical protein
AT2G13040	40.9481	-5.3126	0.52597	-10.101	5.49E-24	3.76E-22	0	0	0	5.05192	6.87146	6.62098	A1+	transposable element gene; pseudogene, hypothetical protein
AT2G13040	12.3081	-0.5875	0.13678	-4.2952	1.75E-05	1.41E-03	0	0	0	2.61592	5.42276	4.79821	A2+	transposable element gene; pseudogene, hypothetical protein
AT2G13050	123.058	-6.8715	0.47835	-14.365	8.60E-47	1.84E-44	0	0	0	7.11016	8.52597	7.87391	A1+	transposable element gene; pseudogene, hypothetical protein
AT2G13050	36.0092	-1.1885	0.16616	-7.1527	8.51E-13	1.64E-10	0	0	0	4.6205	6.68725	6.51418	A2+	transposable element gene; pseudogene, hypothetical protein
AT2G13400	99.2959	-6.8084	0.47075	-14.463	2.08E-47	4.77E-45	0	0	0	7.07436	7.86529	7.85067	A1+	transposable element gene; pseudogene, hypothetical protein
AT2G13400	55.7647	-5.8095	0.43226	-13.44	3.53E-41	4.75E-39	0	0	0	6.51493	7.12834	6.73103	A1-	transposable element gene; pseudogene, hypothetical protein
AT3G29612	56.3017	-5.9003	0.47993	-12.294	9.74E-35	1.21E-32	0	0.62641	0	6.2385	7.11744	6.97621	A1+	transposable element gene; pseudogene, hypothetical protein
AT3G29612	51.2746	-5.5875	0.42583	-13.121	2.48E-39	3.07E-37	0	0.58899	0	6.60587	6.95281	6.47347	A1-	transposable element gene; pseudogene, hypothetical protein
AT3G29738	12.6421	-3.9415	0.56473	-6.9796	2.96E-12	7.61E-11	0	0	0	4.89656	3.71068	5.16887	A1+	transposable element gene; pseudogene, hypothetical protein
AT3G29738	7.74613	-3.0112	0.51159	-5.8859	3.96E-09	7.63E-08	0	0	0	4.4082	4.08983	3.48753	A1-	transposable element gene; pseudogene, hypothetical protein
AT3G30665	25.2312	-5.1029	0.51877	-9.8365	7.84E-23	4.90E-21	0	0	0	5.54593	5.76042	5.74041	A1+	transposable element gene; pseudogene, hypothetical protein
AT3G30665	3.96458	-2.0982	0.5108	-4.1077	4.00E-05	0.000409	0	0	0	2.79716	3.54662	3.02706	A1-	transposable element gene; pseudogene, hypothetical protein
AT3G31440	11.3187	-3.4637	0.55335	-6.2594	3.86E-10	7.61E-09	0	1.06174	0	4.02145	4.78679	4.6994	A1+	transposable element gene; pseudogene, hypothetical protein
AT3G31440	4.03563	-1.8229	0.51345	-3.5502	0.000385	0.003107	0	1.00603	0	3.54983	2.66509	3.02706	A1-	transposable element gene; pseudogene, hypothetical protein
AT3G32966	129.249	-6.6725	0.4761	-14.015	1.27E-44	2.45E-42	0.64015	0	0	6.83858	8.32303	8.42309	A1+	transposable element gene; pseudogene, hypothetical protein
AT3G32966	6.52272	-0.5209	0.13521	-3.8527	1.17E-04	0.008029	0.95419	0	0	3.73469	3.90354	3.69168	A2+	transposable element gene; pseudogene, hypothetical protein
AT3G33072	406.864	-6.7485	0.45282	-14.903	3.14E-50	5.85E-48	0	0	0	8.95794	10.2891	9.44235	A1-	transposable element gene; pseudogene, hypothetical protein
AT3G33072	37.0358	-1.7292	0.17768	-9.7318	2.21E-22	8.76E-20	0	0	0	6.22639	6.61002	5.71861	A2+	transposable element gene; pseudogene, hypothetical protein
AT3G33081	28.4942	-4.6824	0.46931	-9.9771	1.92E-23	1.12E-21	0	0	0	5.3318	6.52082	5.38769	A1-	transposable element gene; pseudogene, hypothetical protein
AT3G33082	5.52512	-2.5532	0.51514	-4.9564	7.18E-07	1.01E-05	0	0	0	3.02518	3.54662	4.02897	A1-	transposable element gene; pseudogene, hypothetical protein
AT3G33085	21.8925	-4.5421	0.47022	-9.6595	4.48E-22	2.39E-20	0	0	0	5.28853	5.76624	5.35204	A1-	transposable element gene; pseudogene, hypothetical protein
AT3G33118	38.3686	-4.6747	0.46901	-9.9671	2.12E-23	1.23E-21	0	0.58899	0	4.82646	6.88921	6.42283	A1-	transposable element gene; pseudogene, hypothetical protein
AT3G33118	12.1091	-0.7932	0.1532	-5.1776	2.25E-07	2.51E-05	0	0.94033	0	4.12237	5.18785	4.38328	A2+	transposable element gene; pseudogene, hypothetical protein
AT3G33225	112.833	-6.6529	0.48854	-13.618	3.14E-42	5.63E-40	0	0	0	8.57739	7.26084	7.17635	A1+	transposable element gene; pseudogene, hypothetical protein
AT3G33225	29.3091	-4.9037	0.45969	-10.667	1.45E-26	1.01E-24	0	0	0	5.86113	6.24407	5.48962	A1-	transposable element gene; pseudogene, hypothetical protein
AT3G33225	15.1455	-1.0772	0.16474	-6.5388	6.20E-11	9.82E-09	0	0	0	4.94274	5.1002	4.84882	A2+	transposable element gene; pseudogene, hypothetical protein
AT3G33575	13.9845	-3.3493	0.57841	-5.7905	7.02E-09	1.16E-07	0	0.62641	0	5.25748	5.39625	2.58406	A1+	transposable element gene; pseudogene, hypothetical protein
AT3G33575	12.4874	-2.6864	0.51418	-5.2246	1.75E-07	2.70E-06	0	0.58899	0	3.39522	5.90133	2.83386	A1-	transposable element gene; pseudogene, hypothetical protein
AT3G33575	18.1113	-2.6384	0.45517	-5.7967	6.77E-09	2.05E-07	0	0.55795	0	6.39469	4.26223	2.97594	A2-	transposable element gene; pseudogene, hypothetical protein
AT3G33595	22.0666	-3.8269	0.51484	-7.4333	1.06E-13	3.15E-12	0.64015	1.3957	0.69459	5.37986	6.10263	4.4747	A1+	transposable element gene; pseudogene, hypothetical protein
AT3G33595	26.7433	-3.2492	0.49539	-6.5588	5.43E-11	1.27E-09	0.60238	1.32917	0.65347	4.70005	6.88921	4.02897	A1-	transposable element gene; pseudogene, hypothetical protein
AT3G33595	25.2033	-2.8252	0.45077	-6.2676	3.67E-10	1.40E-08	0.56965	1.27293	0.62068	6.76924	4.86348	3.76455	A2-	transposable element gene; pseudogene, hypothetical protein
AT3G35707	36.8495	-5.3273	0.52096	-10.226	1.52E-24	1.08E-22	0	0	0	6.88065	5.88373	5.56118	A1+	transposable element gene; pseudogene, hypothetical protein
AT3G35707	9.82238	-3.169	0.50892	-6.2271	4.75E-10	1.00E-08	0	0	0	4.14258	5.04684	3.48753	A1-	transposable element gene; pseudogene, hypothetical protein
AT3G38525	40.8797	-5.0402	0.44866	-11.234	2.78E-29	2.24E-27	0	0.58899	0	5.89038	7.01373	5.88833	A1-	transposable element gene; pseudogene, hypothetical protein
AT3G42360	61.8892	-5.881	0.50849	-11.566	6.16E-31	6.13E-29	0	0	0	7.46844	7.14235	5.80629	A1+	transposable element gene; pseudogene, hypothetical protein
AT3G42360	5.33166	-1.7765	0.49976	-3.5547	0.000378	0.003058	0	0	0	1.13103	4.7925	2.34668	A1-	transposable element gene; pseudogene, hypothetical protein
AT3G42360	18.0306	-1.2278	0.16944	-7.2461	4.29E-13	8.51E-11	0	0	0	5.28403	5.18785	5.1607	A2+	transposable element gene; pseudogene, hypothetical protein
AT3G42718	87.6279	-6.4222	0.41574	-15.448	7.81E-54	1.62E-51	0	0	0	7.21508	7.74622	7.37054	A1-	transposable element gene; pseudogene, hypothetical protein
AT3G42718	7.05309	-0.588	0.13798	-4.2617	2.03E-05	1.62E-03	0	0	0	3.94151	4.00355	3.79855	A2+	transposable element gene; pseudogene, hypothetical protein
AT3G42719	545.389	-8.6045	0.37933	-22.683	6.58E-114	8.89E-111	0	0	0	10.0108	10.5007	9.63447	A1-	transposable element gene; pseudogene, hypothetical protein
AT3G42719	36.4531	-1.7699	0.1782	-9.9321	3.02E-23	1.29E-20	0	0	0	6.30338	6.47716	5.74558	A2+	transposable element gene; pseudogene, hypothetical protein
AT3G43000	14.0014	-3.6977	0.49015	-7.5441	4.56E-14	1.40E-12	0.60238	0	0	4.4082	4.48353	5.42247	A1-	transposable element gene; pseudogene, hypothetical protein
AT3G43000	14.4779	-3.3687	0.57849	-5.8232	5.77E-09	9.66E-08	0.64015	0	0	5.82982	4.6545	2.85705	A1+	transposable element gene; pseudogene, hypothetical protein
AT3G43154	27.6811	-4.7818	0.52288	-9.145	5.96E-20	2.98E-18	0	0	0.69459	6.47656	5.31093	5.31183	A1+	transposable element gene; pseudogene, hypothetical protein
AT3G43154	27.5522	-4.6936	0.45361	-10.347	4.31E-25	2.72E-23	0	0	0.65347	5.53062	6.13862	5.67413	A1-	transposable element gene; pseudogene, hypothetical protein
AT3G43303	6.44364	-2.7446	0.51438	-5.3358	9.51E-08	1.53E-06	0	0	0	3.22203	4.08983	3.93573	A1-	transposable element gene; pseudogene, hypothetical protein
AT4G03760	103.891	-6.4834	0.47571	-13.629	2.69E-42	4.88E-40	0	0	0.69459	8.3862	7.06629	7.29584	A1+	transposable element gene; pseudogene, hypothetical protein

AT4G03760	37.8378	-4.4154	0.48275	-9.1463	5.89E-20	2.74E-18	0	0	0.65347	4.56149	5.90133	7.19054	A1-	transposable element gene; pseudogene, hypothetical protein
AT4G03760	17.1485	-1.0243	0.16349	-6.2654	3.72E-10	5.41E-08	0	0	1.02486	4.67946	5.65579	4.84882	A2+	transposable element gene; pseudogene, hypothetical protein
AT4G03825	8.11043	-2.9403	0.51227	-5.7398	9.48E-09	1.73E-07	0	0	0	3.54983	4.7925	3.61329	A1-	transposable element gene; pseudogene, hypothetical protein
AT4G03825	4.68315	-2.0158	0.585	-3.4458	5.69E-04	3.73E-03	0	0	0	4.52436	2.54524	1.16932	A1+	transposable element gene; pseudogene, hypothetical protein
AT4G04165	49.3894	-5.2638	0.4481	-11.747	7.32E-32	6.51E-30	0.60238	0	0	5.76968	6.60241	7.20059	A1-	transposable element gene; pseudogene, hypothetical protein
AT4G04293	32.5835	-3.2508	0.40054	-8.116	4.82E-16	1.74E-14	1.84442	2.71064	2.1498	6.58814	5.45092	5.55378	A1-	transposable element gene; pseudogene, hypothetical protein
AT4G04293	33.6643	-3.0865	0.47527	-6.4943	8.34E-11	1.81E-09	1.92313	2.80343	2.23589	6.79525	5.76042	4.76714	A1+	transposable element gene; pseudogene, hypothetical protein
AT4G04293	43.0079	-1.5782	0.18023	-8.7567	2.01E-18	6.68E-16	2.50778	3.4736	2.84971	6.16583	6.40584	6.38294	A2+	transposable element gene; pseudogene, hypothetical protein
AT4G05588	63.1055	-5.8552	0.43456	-13.474	2.23E-41	3.02E-39	0	0	0	6.45747	7.47011	6.8635	A1-	transposable element gene; pseudogene, hypothetical protein
AT4G05591	124.299	-6.5766	0.42283	-15.554	1.50E-54	3.15E-52	0	0	0	7.45903	8.61108	7.50644	A1-	transposable element gene; pseudogene, hypothetical protein
AT4G06507	11.6954	-3.5752	0.49935	-7.1597	8.09E-13	2.23E-11	0	0	0	4.14258	5.04684	4.48892	A1-	transposable element gene; pseudogene, hypothetical protein
AT4G06507	3.77573	-2.1045	0.58779	-3.5804	3.43E-04	2.38E-03	0	0	0	4.02145	1.77337	2.58406	A1+	transposable element gene; pseudogene, hypothetical protein
AT4G06511	14.44	-3.832	0.49253	-7.7803	7.23E-15	2.37E-13	0	0	0	4.48688	5.45092	4.55311	A1-	transposable element gene; pseudogene, hypothetical protein
AT4G06511	16.192	-3.775	0.57534	-6.5614	5.33E-11	1.18E-09	0	0	0	6.1029	4.16427	3.75389	A1+	transposable element gene; pseudogene, hypothetical protein
AT4G06517	267.082	-7.7127	0.39387	-19.582	2.21E-85	1.39E-82	0	0	0	8.73431	9.52804	8.7857	A1-	transposable element gene; pseudogene, hypothetical protein
AT4G06517	141.598	-4.266	0.57849	-7.3744	1.65E-13	4.81E-12	0	0	0	9.12873	6.92986	7.41678	A1+	transposable element gene; pseudogene, hypothetical protein
AT4G06517	25.6199	-1.3295	0.17116	-7.7676	8.00E-15	2.01E-12	0	0	0	5.94335	6.00855	4.94502	A2+	transposable element gene; pseudogene, hypothetical protein
AT4G06518	13.8001	-3.6194	0.57836	-6.2581	3.90E-10	7.66E-09	0	0	0	5.82982	4.16427	3.45845	A1+	transposable element gene; pseudogene, hypothetical protein
AT4G06518	5.97191	-2.4481	0.51498	-4.7538	2.00E-06	2.60E-05	0	0	0	3.22203	4.48353	2.83386	A1-	transposable element gene; pseudogene, hypothetical protein
AT4G06530	254.594	-7.503	0.40385	-18.579	4.78E-77	2.45E-74	0	0	0	8.60799	9.59933	8.51057	A1-	transposable element gene; pseudogene, hypothetical protein
AT4G06531	292.917	-7.6941	0.3869	-19.886	5.36E-88	3.72E-85	0.60238	0	0	9.10239	9.61906	8.72777	A1-	transposable element gene; pseudogene, hypothetical protein
AT4G06531	11.1213	-0.69	0.14679	-4.7005	2.60E-06	2.47E-04	0.95419	0	0	3.94151	5.22975	3.99111	A2+	transposable element gene; pseudogene, hypothetical protein
AT4G06533	11.0424	-3.4652	0.50204	-6.9022	5.12E-12	1.32E-10	0	0	0	4.23666	5.04684	4.11654	A1-	transposable element gene; pseudogene, hypothetical protein
AT4G06533	6.15433	-2.5558	0.59238	-4.3145	1.60E-05	1.52E-04	0	0	0	4.81212	2.54524	2.58406	A1+	transposable element gene; pseudogene, hypothetical protein
AT4G06541	15.3655	-3.3565	0.58611	-5.7267	1.02E-08	1.65E-07	0	0	0	6.17229	2.54524	4.10751	A1+	transposable element gene; pseudogene, hypothetical protein
AT4G06541	8.55898	-2.5829	0.51514	-5.0139	5.33E-07	7.68E-06	0	0	0	2.52621	5.26298	3.34975	A1-	transposable element gene; pseudogene, hypothetical protein
AT4G06544	21.3383	-4.3921	0.47667	-9.2141	3.14E-20	1.50E-18	0	0	0	4.82646	5.90133	5.42247	A1-	transposable element gene; pseudogene, hypothetical protein
AT4G06544	19.115	-1.1571	0.16688	-6.9337	4.10E-12	7.37E-10	0	0	0	4.67946	5.62471	5.417	A2+	transposable element gene; pseudogene, hypothetical protein
AT4G06575	24.4351	-4.5218	0.55231	-8.1869	2.68E-16	1.01E-14	0	0	0	6.53041	5.01973	4.62832	A1+	transposable element gene; pseudogene, hypothetical protein
AT4G06575	23.0441	-4.3738	0.47848	-9.141	6.19E-20	2.87E-18	0	0	0	4.94268	6.24407	5.11744	A1-	transposable element gene; pseudogene, hypothetical protein
AT4G06592	15.6841	-3.6238	0.4914	-7.3745	1.65E-13	4.84E-12	0.60238	0	0	4.14258	5.76624	4.61457	A1-	transposable element gene; pseudogene, hypothetical protein
AT4G06615	202.635	-7.7921	0.44398	-17.55	5.90E-69	3.52E-66	0	0	0	8.98186	8.35535	8.59138	A1+	transposable element gene; pseudogene, hypothetical protein
AT4G06615	237.528	-7.6638	0.3906	-19.62	1.03E-85	6.64E-83	0	0	0	8.82039	9.2144	8.57769	A1-	transposable element gene; pseudogene, hypothetical protein
AT4G06615	16.0758	-1.063	0.16395	-6.4839	8.94E-11	1.41E-08	0	0	0	5.12345	5.34862	4.57587	A2+	transposable element gene; pseudogene, hypothetical protein
AT4G06664	6.51514	-2.7458	0.51459	-5.3359	9.51E-08	1.53E-06	0	0	0	4.4082	3.54662	3.19741	A1-	transposable element gene; pseudogene, hypothetical protein
AT4G06704	139.674	-6.8404	0.41287	-16.568	1.18E-61	3.11E-59	0	0	0	7.75766	8.66903	7.76545	A1-	transposable element gene; pseudogene, hypothetical protein
AT4G06704	11.7395	-0.863	0.15554	-5.5487	2.88E-08	3.48E-06	0	0	0	4.67946	4.85451	4.23894	A2+	transposable element gene; pseudogene, hypothetical protein
AT4G06712	8.73791	-3.1254	0.51028	-6.1248	9.08E-10	1.88E-08	0	0	0	4.76464	4.08983	3.48753	A1-	transposable element gene; pseudogene, hypothetical protein
AT4G06720	992.039	-9.2331	0.36409	-25.359	7.10E-142	1.52E-138	0	0.58899	0	10.7872	11.3606	10.6055	A1-	transposable element gene; pseudogene, hypothetical protein
AT4G06720	70.5662	-2.4372	0.18008	-13.534	9.90E-42	1.05E-38	0	0.94033	0	7.29421	7.35059	6.71852	A2+	transposable element gene; pseudogene, hypothetical protein
AT4G06736	397.523	-8.1114	0.39139	-20.725	2.07E-95	1.97E-92	0	0	0	9.53154	10.1331	9.03645	A1-	transposable element gene; pseudogene, hypothetical protein
AT4G06736	14.9005	-0.9662	0.15998	-6.0393	1.55E-09	2.12E-07	0	0	0	4.99008	5.34862	4.31292	A2+	transposable element gene; pseudogene, hypothetical protein
AT4G06738	53.8198	-5.5593	0.44486	-12.497	7.78E-36	8.29E-34	0	0	0	6.58814	7.33342	6.09651	A1-	transposable element gene; pseudogene, hypothetical protein
AT4G07570	12.6652	-3.8316	0.49275	-7.7759	7.49E-15	2.45E-13	0	0	0	4.70005	4.48353	4.93675	A1-	transposable element gene; pseudogene, hypothetical protein
AT4G07570	4.42747	-1.876	0.58039	-3.2323	0.001228	0.007235	0	0	0	4.4141	0	2.85705	A1+	transposable element gene; pseudogene, hypothetical protein
AT4G07605	325.035	-8.053	0.38398	-20.973	1.17E-97	1.15E-94	0	0	0	9.30055	9.65775	9.0081	A1-	transposable element gene; pseudogene, hypothetical protein
AT4G07605	41.3124	-1.8864	0.17907	-10.534	6.00E-26	2.90E-23	0	0	0	6.1863	6.77477	6.09917	A2+	transposable element gene; pseudogene, hypothetical protein
AT4G07936	7.03872	-2.9286	0.5124	-5.7155	1.09E-08	1.98E-07	0	0	0	3.68946	4.08983	3.93573	A1-	transposable element gene; pseudogene, hypothetical protein
AT4G07939	9.28164	-3.0312	0.58302	-5.1992	2.00E-07	2.69E-06	0	0.62641	0	5.19216	3.71068	3.08651	A1+	transposable element gene; pseudogene, hypothetical protein
AT4G07939	5.47796	-2.2984	0.5151	-4.462	8.12E-06	9.53E-05	0	0.58899	0	3.02518	4.08983	3.34975	A1-	transposable element gene; pseudogene, hypothetical protein
AT5G28870	7.25593	-2.8542	0.51394	-5.5535	2.80E-08	4.83E-07	0	0	0	3.22203	3.54662	4.67351	A1-	transposable element gene; pseudogene, hypothetical protein
AT5G29058	8.98095	-3.4252	0.57919	-5.9138	3.34E-09	5.78E-08	0	0	0	3.24283	4.6545	4.4747	A1+	transposable element gene; pseudogene, hypothetical protein
AT5G30762	87.8641	-6.7706	0.46626	-14.521	8.92E-48	2.18E-45	0	0	0	7.24511	7.45231	7.66781	A1+	transposable element gene; pseudogene, hypothetical protein
AT5G30762	88.271	-6.2151	0.42823	-14.513	9.97E-48	1.74E-45	0	0	0	6.87816	8.03777	7.24984	A1-	transposable element gene; pseudogene, hypothetical protein
AT5G30762	8.27966	-0.6507	0.14267	-4.5607	5.10E-06	0.00046	0	0	0	4.35716	4.2677	3.69168	A2+	transposable element gene; pseudogene, hypothetical protein
AT5G30852	20.3746	-4.2555	0.48195	-8.8298	1.05E-18	4.52E-17	0	0	0	4.56149	5.90133	5.38769	A1-	transposable element gene; pseudogene, hypothetical protein
AT5G31302	14.2179	-3.938	0.48918	-8.0501	8.27E-16	2.95E-14	0	0	0	4.94268	5.04684	4.61457	A1-	transposable element gene; pseudogene, hypothetical protein

AT5G31302	6.15433	-2.5558	0.59238	-4.3145	1.60E-05	0.000152	0	0	0	4.81212	2.54524	2.58406	A1+	transposable element gene; pseudogene, hypothetical protein
AT5G32107	136.358	-6.7831	0.38531	-17.604	2.28E-69	8.61E-67	0	0.58899	0.65347	7.85728	8.32894	8.05867	A1-	transposable element gene; pseudogene, hypothetical protein
AT5G32107	66.2424	-5.5374	0.50028	-11.069	1.78E-28	1.56E-26	0	0.62641	0.69459	7.91557	6.42035	6.17386	A1+	transposable element gene; pseudogene, hypothetical protein
AT5G32107	23.4491	-1.3616	0.17394	-7.8277	4.97E-15	1.28E-12	0	0.94033	1.02486	5.59088	5.80184	5.23869	A2+	transposable element gene; pseudogene, hypothetical protein
AT5G32228	108.728	-6.7452	0.40709	-16.569	1.16E-61	3.07E-59	0	0	0	7.57162	7.9154	7.80553	A1-	transposable element gene; pseudogene, hypothetical protein
AT5G32228	63.9852	-5.897	0.5094	-11.576	5.43E-31	5.43E-29	0	0	0	7.79974	6.42035	6.29355	A1+	transposable element gene; pseudogene, hypothetical protein
AT5G32228	20.0513	-1.2418	0.16949	-7.3269	2.36E-13	4.97E-11	0	0	0	5.35808	5.68621	4.94502	A2+	transposable element gene; pseudogene, hypothetical protein
AT5G32241	13.284	-3.7972	0.49365	-7.6922	1.45E-14	4.65E-13	0	0	0	4.32498	5.04684	4.88781	A1-	transposable element gene; pseudogene, hypothetical protein
AT5G32420	8.23677	-3.1027	0.58753	-5.2809	1.29E-07	1.78E-06	0	0	0	5.05192	3.04536	3.45845	A1+	transposable element gene; pseudogene, hypothetical protein
AT5G32420	7.63379	-3.0671	0.51034	-6.0099	1.86E-09	3.73E-08	0	0	0	4.04193	4.08983	3.93573	A1-	transposable element gene; pseudogene, hypothetical protein
AT5G32434	6.41503	-3.0904	0.58595	-5.2742	1.33E-07	1.85E-06	0	0	0	3.48023	3.71068	4.10751	A1+	transposable element gene; pseudogene, hypothetical protein
AT5G32434	6.23253	-2.7905	0.51406	-5.4283	5.69E-08	9.45E-07	0	0	0	4.04193	3.54662	3.61329	A1-	transposable element gene; pseudogene, hypothetical protein
AT5G32473	17.0441	-3.987	0.48918	-8.1503	3.63E-16	1.33E-14	0	0	0	4.4082	5.76624	4.88781	A1-	transposable element gene; pseudogene, hypothetical protein
AT5G32473	9.92248	-3.6599	0.57187	-6.4	1.55E-10	3.25E-09	0	0	0	4.89656	3.95523	4.10751	A1+	transposable element gene; pseudogene, hypothetical protein
AT5G32483	7.23707	-2.9789	0.5117	-5.8217	5.83E-09	1.09E-07	0	0	0	3.81676	4.08983	3.93573	A1-	transposable element gene; pseudogene, hypothetical protein
AT5G32483	3.00313	-1.8776	0.58122	-3.2305	1.24E-03	0.007273	0	0	0	3.68404	1.77337	2.24707	A1+	transposable element gene; pseudogene, hypothetical protein
AT5G32488	9.00696	-3.2646	0.50661	-6.4439	1.16E-10	2.61E-09	0	0	0	3.93374	4.48353	4.2772	A1-	transposable element gene; pseudogene, hypothetical protein
AT5G32490	13.7711	-3.9592	0.48926	-8.0921	5.86E-16	2.11E-14	0	0	0	4.88574	4.48353	5.07435	A1-	transposable element gene; pseudogene, hypothetical protein
AT5G32490	7.33002	-2.8497	0.59106	-4.8213	1.43E-06	1.66E-05	0	0	0	4.97633	3.04536	2.85705	A1+	transposable element gene; pseudogene, hypothetical protein
AT5G32495	213.137	-7.3658	0.38866	-18.952	4.27E-80	2.29E-77	0.60238	0	0	8.53117	9.13437	8.44864	A1-	transposable element gene; pseudogene, hypothetical protein
AT5G32495	9.81176	-0.7271	0.14998	-4.8477	1.25E-06	0.000127	0.95419	0	0	4.49483	4.49088	3.99111	A2+	transposable element gene; pseudogene, hypothetical protein
AT5G32510	7.1919	-2.8441	0.51339	-5.5399	3.03E-08	5.18E-07	0	0	0	3.39522	4.48353	3.72897	A1-	transposable element gene; pseudogene, hypothetical protein
AT5G32511	87.2971	-6.7429	0.46803	-14.407	4.68E-47	1.03E-44	0	0	0	7.72088	7.16684	7.42729	A1+	transposable element gene; pseudogene, hypothetical protein
AT5G32511	105.497	-6.4761	0.42094	-15.385	2.07E-53	4.22E-51	0	0	0	7.3369	8.28019	7.35262	A1-	transposable element gene; pseudogene, hypothetical protein
AT5G32511	14.3367	-0.9457	0.15913	-5.9425	2.81E-09	3.75E-07	0	0	0	5.20597	5.0543	4.23894	A2+	transposable element gene; pseudogene, hypothetical protein
AT5G32515	26.2208	-4.8674	0.46032	-10.574	3.93E-26	2.65E-24	0	0	0	5.67203	5.61718	5.91301	A1-	transposable element gene; pseudogene, hypothetical protein
AT5G32515	101.442	-4.0812	0.58074	-7.0276	2.10E-12	5.49E-11	0	0	0	5.78621	8.2675	7.95603	A1+	transposable element gene; pseudogene, hypothetical protein
AT5G32516	14.7594	-4.2522	0.55203	-7.7029	1.33E-14	4.29E-13	0	0	0	5.37986	4.6545	4.62832	A1+	transposable element gene; pseudogene, hypothetical protein
AT5G32516	12.6345	-3.2469	0.51	-6.3666	1.93E-10	4.23E-09	0	0	0	2.79716	5.04684	5.278	A1-	transposable element gene; pseudogene, hypothetical protein
AT5G32516	7.92158	-0.6301	0.14117	-4.4634	8.07E-06	7.08E-04	0	0	0	4.35716	4.09708	3.69168	A2+	transposable element gene; pseudogene, hypothetical protein
AT5G32517	18.9654	-4.0575	0.48797	-8.3151	9.16E-17	3.51E-15	0	0	0	4.76464	6.02485	4.61457	A1-	transposable element gene; pseudogene, hypothetical protein
AT5G32517	13.9615	-3.5556	0.58045	-6.1256	9.03E-10	1.69E-08	0	0	0	5.91325	3.95523	3.45845	A1+	transposable element gene; pseudogene, hypothetical protein
AT5G32520	6.84822	-2.6304	0.51514	-5.1061	3.29E-07	4.88E-06	0	0	0	3.02518	2.66509	4.88781	A1-	transposable element gene; pseudogene, hypothetical protein
AT5G32520	5.93099	-2.107	0.58697	-3.5895	3.31E-04	2.31E-03	0	0	0	0	4.34682	4.10751	A1+	transposable element gene; pseudogene, hypothetical protein
AT5G32591	5.87847	-2.8479	0.59025	-4.825	1.40E-06	1.63E-05	0	0	0	4.16454	3.71068	2.85705	A1+	transposable element gene; pseudogene, hypothetical protein
AT5G32591	7.57152	-2.7816	0.51407	-5.411	6.27E-08	1.04E-06	0	0	0	3.39522	4.7925	3.34975	A1-	transposable element gene; pseudogene, hypothetical protein
AT5G32623	62.219	-2.8812	0.59226	-4.8647	1.15E-06	1.36E-05	0	0	0.69459	4.4141	7.21461	7.68543	A1+	transposable element gene; pseudogene, hypothetical protein
AT5G32623	28.6842	-1.4109	0.17354	-8.1298	4.30E-16	1.22E-13	0	0	1.02486	5.24553	6.33081	5.79804	A2+	transposable element gene; pseudogene, hypothetical protein
AT5G32630	35.2266	-3.0102	0.58921	-5.109	3.24E-07	4.19E-06	0.64015	1.06174	0	0	6.33648	7.03235	A1+	transposable element gene; pseudogene, hypothetical protein
AT5G32630	4.75792	-1.8956	0.515	-3.6807	0.000233	0.001993	0.60238	1.00603	0	3.02518	3.54662	3.34975	A1-	transposable element gene; pseudogene, hypothetical protein
AT5G32726	26.8005	-4.852	0.46154	-10.513	7.55E-26	5.03E-24	0	0	0	5.49299	5.76624	6.00768	A1-	transposable element gene; pseudogene, hypothetical protein
AT5G33150	6.12019	-2.7634	0.51429	-5.3731	7.74E-08	1.26E-06	0	0	0	3.54983	3.54662	4.02897	A1-	transposable element gene; pseudogene, hypothetical protein
AT5G33150	6.20116	-2.7631	0.59153	-4.6712	2.99E-06	3.31E-05	0	0	0	4.6268	3.04536	2.85705	A1+	transposable element gene; pseudogene, hypothetical protein
AT5G33254	47.5555	-5.6527	0.43556	-12.978	1.63E-38	1.94E-36	0	0	0	6.53358	6.75292	6.45679	A1-	transposable element gene; pseudogene, hypothetical protein
AT5G33254	22.471	-4.9259	0.52575	-9.3693	7.30E-21	3.92E-19	0	0	0	5.74125	5.39625	5.39982	A1+	transposable element gene; pseudogene, hypothetical protein
AT5G33255	105.447	-6.5797	0.40227	-16.356	3.93E-60	9.89E-58	0	0.58899	0	7.62479	7.47011	8.02511	A1-	transposable element gene; pseudogene, hypothetical protein
AT5G33255	57.3445	-6.0538	0.46792	-12.938	2.76E-38	4.09E-36	0	0.62641	0	6.68085	6.98599	6.87237	A1+	transposable element gene; pseudogene, hypothetical protein
AT5G33255	14.7424	-1.0106	0.16351	-6.1811	6.37E-10	9.01E-08	0	0.94033	0	4.6205	5.00689	5.07826	A2+	transposable element gene; pseudogene, hypothetical protein
AT5G33384	7.16052	-2.9718	0.58038	-5.1203	3.05E-07	3.96E-06	0.64015	0	0	4.4141	3.71068	3.45845	A1+	transposable element gene; pseudogene, hypothetical protein
AT5G33387	7.54953	-3.0291	0.51109	-5.9268	3.09E-09	6.02E-08	0	0	0	3.68946	4.08983	4.19911	A1-	transposable element gene; pseudogene, hypothetical protein
AT5G33387	5.89743	-2.7053	0.59192	-4.5703	4.87E-06	5.12E-05	0	0	0	2.1331	3.95523	4.20843	A1+	transposable element gene; pseudogene, hypothetical protein
AT5G33391	87.363	-6.4942	0.41359	-15.702	1.46E-55	3.21E-53	0	0	0	7.4094	7.28482	7.65278	A1-	transposable element gene; pseudogene, hypothetical protein
AT5G33391	57.1486	-5.9394	0.50163	-11.84	2.42E-32	2.68E-30	0	0	0	6.03001	6.84135	7.37397	A1+	transposable element gene; pseudogene, hypothetical protein
AT5G33391	14.0162	-0.9223	0.15806	-5.8351	5.37E-09	6.97E-07	0	0	0	5.08035	5.14469	4.16097	A2+	transposable element gene; pseudogene, hypothetical protein
AT5G33427	9.97689	-3.3455	0.5831	-5.7374	9.61E-09	1.55E-07	0	0	0	5.31997	3.41604	3.61372	A1+	transposable element gene; pseudogene, hypothetical protein
AT5G34623	6.47844	-2.7193	0.58754	-4.6284	3.69E-06	3.99E-05	0	0.62641	0	2.60404	3.95523	4.30275	A1+	transposable element gene; pseudogene, hypothetical protein
AT5G34623	5.17159	-2.3853	0.51501	-4.6317	3.63E-06	4.53E-05	0	0.58899	0	3.68946	2.66509	3.83605	A1-	transposable element gene; pseudogene, hypothetical protein

AT5G34835	6.61243	-2.8183	0.58377	-4.8278	1.38E-06	1.61E-05	0	0	0.69459	3.24283	4.34682	3.61372	A1+	transposable element gene; pseudogene, hypothetical protein
AT5G34835	3.42592	-1.6431	0.50386	-3.2609	0.00111	0.007861	0	0	0.65347	1.75715	2.66509	3.72897	A1-	transposable element gene; pseudogene, hypothetical protein
AT5G34843	5.51586	-2.5784	0.59238	-4.3526	1.35E-05	0.000131	0	0	0	4.52436	2.54524	2.85705	A1+	transposable element gene; pseudogene, hypothetical protein
AT5G35048	17.1672	-4.0548	0.47644	-8.5105	1.73E-17	7.01E-16	0.60238	0	0	5.45435	5.04684	4.83714	A1-	transposable element gene; pseudogene, hypothetical protein
AT5G35048	6.19869	-2.5543	0.59054	-4.3253	1.52E-05	1.46E-04	0.64015	0	0	2.1331	3.95523	4.30275	A1+	transposable element gene; pseudogene, hypothetical protein
AT5G35061	6.30052	-2.7716	0.59148	-4.6859	2.79E-06	3.11E-05	0	0	0	2.1331	4.16427	4.20843	A1+	transposable element gene; pseudogene, hypothetical protein
AT5G35061	4.86602	-1.9574	0.50692	-3.8614	0.000113	0.00105	0	0	0	2.52621	4.48353	2.02322	A1-	transposable element gene; pseudogene, hypothetical protein
AT5G35145	37.3602	-5.3069	0.44636	-11.889	1.35E-32	1.24E-30	0	0	0	6.00175	6.43434	6.25929	A1-	transposable element gene; pseudogene, hypothetical protein
AT5G35146	32.4003	-5.1026	0.45269	-11.272	1.81E-29	1.47E-27	0	0	0	6.05437	6.24407	5.7852	A1-	transposable element gene; pseudogene, hypothetical protein
AT5G35146	16.8033	-3.8976	0.57152	-6.8196	9.13E-12	2.23E-10	0	0	0	6.1029	4.34682	3.88164	A1+	transposable element gene; pseudogene, hypothetical protein
AT4G06474	9.75751	-2.9288	0.5794	-5.055	4.30E-07	5.47E-06	0.64015	0.62641	0	5.31997	3.04536	3.61372	A1+	transposable element gene; retroelement pol polyprotein
AT2G09910	21.1278	-3.7172	0.48525	-7.6603	1.85E-14	5.92E-13	0.60238	0.58899	0	4.32498	6.34234	4.78464	A1-	transposable element gene; similar to ASY2, DNA binding
AT1G54430	11.5387	-3.5274	0.55033	-6.4096	1.46E-10	3.06E-09	1.08201	0	0	4.6268	4.16427	4.83185	A1+	transposable element gene; similar to AT hook motif-containing protein-related
AT3G42100	20.4708	-3.9851	0.48687	-8.1851	2.72E-16	1.02E-14	0.64015	0.62641	1.51363	5.12374	5.47681	5.44189	A1+	transposable element gene; similar to AT hook motif-containing protein-related
AT3G42100	18.0222	-3.6673	0.45121	-8.1278	4.37E-16	1.59E-14	0.60238	0.58899	1.44299	5.15032	5.04684	5.3155	A1-	transposable element gene; similar to AT hook motif-containing protein-related
AT3G42100	5.82801	-1.7189	0.4551	-3.777	1.59E-04	1.80E-03	0.56965	0.55795	1.38559	4.13977	2.92871	3.34439	A2-	transposable element gene; similar to AT hook motif-containing protein-related
AT4G01980	26.7861	-3.7002	0.40324	-9.1762	4.47E-20	2.11E-18	1.61959	1.593	1.44299	5.49299	5.90133	5.73073	A1-	transposable element gene; similar to myb family protein
AT4G01980	21.7167	-3.1832	0.50614	-6.2892	3.19E-10	6.38E-09	1.6933	1.66671	1.51363	6.27049	4.78679	4.4747	A1+	transposable element gene; similar to myb family protein
AT1G08740	7.67179	-3.2134	0.58377	-5.5045	3.70E-08	5.52E-07	0	0	0	3.68404	4.6545	3.45845	A1+	transposable element gene; similar to Ulp1 protease family protein
AT1G08740	19.0463	-1.0701	0.16365	-6.5389	6.20E-11	9.82E-09	0	0	0	4.55904	5.85636	5.1607	A2+	transposable element gene; similar to Ulp1 protease family protein
AT1G21020	7.18139	-3.0689	0.58696	-5.2284	1.71E-07	2.32E-06	0	0	0	3.48023	4.6545	3.28443	A1+	transposable element gene; similar to Ulp1 protease family protein
AT1G21020	17.4834	-1.0307	0.16232	-6.3499	2.16E-10	3.28E-08	0	0	0	4.49483	5.716	5.03519	A2+	transposable element gene; similar to Ulp1 protease family protein
AT1G34610	8.90633	-3.3543	0.56774	-5.9082	3.46E-09	5.96E-08	0.64015	0	0	3.86258	4.34682	4.39129	A1+	transposable element gene; similar to Ulp1 protease family protein
AT2G07510	6.05126	-2.758	0.51451	-5.3605	8.30E-08	1.35E-06	0	0	0	4.14258	2.66509	3.93573	A1-	transposable element gene; similar to Ulp1 protease family protein
AT2G14010	8.22739	-2.8234	0.58727	-4.8077	1.53E-06	1.77E-05	0.64015	0	0	2.1331	4.6545	4.4747	A1+	transposable element gene; similar to Ulp1 protease family protein
AT2G14010	4.00577	-1.6556	0.5058	-3.2731	0.001064	0.007579	0.60238	0	0	1.75715	4.08983	2.61074	A1-	transposable element gene; similar to Ulp1 protease family protein
AT2G29240	7.36178	-2.9769	0.58013	-5.1314	2.88E-07	3.77E-06	0.64015	0	0	3.68404	4.50885	3.45845	A1+	transposable element gene; similar to Ulp1 protease family protein
AT2G29240	18.8445	-1.0546	0.16424	-6.4208	1.36E-10	2.08E-08	0.95419	0	0	4.55904	5.80184	5.1607	A2+	transposable element gene; similar to Ulp1 protease family protein
AT3G09170	8.20776	-3.2189	0.5723	-5.6245	1.86E-08	2.89E-07	0.64015	0	0	3.68404	4.34682	4.20843	A1+	transposable element gene; similar to Ulp1 protease family protein
AT3G26530	7.67179	-3.2134	0.58377	-5.5045	3.70E-08	5.52E-07	0	0	0	3.68404	4.6545	3.45845	A1+	transposable element gene; similar to Ulp1 protease family protein
AT3G26530	18.8673	-1.0714	0.16373	-6.5435	6.01E-11	9.63E-09	0	0	0	4.55904	5.82936	5.1607	A2+	transposable element gene; similar to Ulp1 protease family protein
AT4G04400	14.1285	-3.9694	0.5654	-7.0206	2.21E-12	5.77E-11	0	0	0	5.6469	4.16427	4.30275	A1+	transposable element gene; similar to Ulp1 protease family protein
AT4G04530	7.15294	-2.9063	0.58275	-4.9872	6.13E-07	7.60E-06	0.64015	0	0	2.9585	4.34682	4.10751	A1+	transposable element gene; similar to Ulp1 protease family protein
AT4G04530	6.0192	-2.4662	0.51425	-4.7956	1.62E-06	2.14E-05	0.60238	0	0	3.22203	4.08983	3.61329	A1-	transposable element gene; similar to Ulp1 protease family protein
AT4G04530	6.87537	-0.5396	0.13673	-3.9465	7.93E-05	5.66E-03	0.95419	0	0	3.61898	4.09708	3.79855	A2+	transposable element gene; similar to Ulp1 protease family protein
AT4G07580	6.06402	-2.6928	0.51482	-5.2306	1.69E-07	2.62E-06	0	0	0	3.22203	3.54662	4.19911	A1-	transposable element gene; similar to Ulp1 protease family protein
AT4G07580	2.92909	-1.918	0.5828	-3.291	0.000998	0.006033	0	0	0	3.48023	1.77337	2.58406	A1+	transposable element gene; similar to Ulp1 protease family protein
AT5G44890	7.67179	-3.2134	0.58377	-5.5045	3.70E-08	5.52E-07	0	0	0	3.68404	4.6545	3.45845	A1+	transposable element gene; similar to Ulp1 protease family protein
AT5G44890	18.8673	-1.0714	0.16373	-6.5435	6.01E-11	9.63E-09	0	0	0	4.55904	5.82936	5.1607	A2+	transposable element gene; similar to Ulp1 protease family protein
AT1G17277	8.06525	-2.7068	0.51475	-5.2585	1.45E-07	2.28E-06	0	0	0	3.02518	5.04684	3.34975	A1-	transposable element gene; similar to zinc finger protein
AT2G15520	13.559	-3.5969	0.48983	-7.3431	2.09E-13	6.06E-12	0	0	0.65347	4.70005	5.26298	4.2772	A1-	transposable element gene; similar to zinc finger protein
AT5G27500	12.9636	-3.1269	0.58924	-5.3067	1.12E-07	1.56E-06	0	0	0	1.42937	5.39625	5.16887	A1+	transposable element gene; similar to zinc finger protein
AT1G61510	12.7007	-3.4635	0.50507	-6.8575	7.01E-12	1.78E-10	0	0	0	5.3318	4.7925	3.48753	A1-	transposable element gene; transposase IS4 family protein
AT1G61510	6.596	-2.9307	0.58957	-4.9709	6.66E-07	8.21E-06	0	0	0	2.60404	3.95523	4.39129	A1+	transposable element gene; transposase IS4 family protein
