# Supplementary material for: Induction of epigenetic variation in Arabidopsis by over-expression of DNA METHYLTRANSFERASE1 (MET1)
Source: PLoS One. 2018 Feb 21;13(2):e0192170. doi: 10.1371/journal.pone.0192170 (PMC5821449; doi:10.1371/journal.pone.0192170)
Supplement: S6 Table — (PDF) [file pone.0192170.s011.pdf]

S6 Table: List of non-coding RNAs with at least log2-fold increases (negative log2-fold change) or decreases (positive log2-fold change) of 2.5 in at least one of the four lines A1+, A1-, A2+ or A2-

	baseMean	log2FoldC	lfcSE	stat	pvalue	padj	1	2	3	4	5	6		
MIRNAs														
AT4G06130	48.823	2.66163	0.34804	7.64755	2.05E-14	6.52E-13	6.68775	6.51176	6.28123	3.39522	0	3.93573	A1-	MIR3932b
AT4G06130	56.135	2.34314	0.33176	7.06266	1.63E-12	4.31E-11	6.79471	6.61951	6.39006	4.16454	3.41604	4.39129	A1+	MIR3932b
AT4G06130	60.8098	1.07757	0.26232	4.10787	3.99E-05	5.35E-04	6.59066	6.41817	6.19058	5.41174	5.15886	5.23372	A2-	MIR3932b
AT5G04935	18.4763	-3.8198	0.57564	-6.6358	3.23E-11	7.37E-10	0	0	0	6.36244	3.71068	4.20843	A1+	MIR854b
AT5G04935	29.9511	-5.0032	0.45548	-10.985	4.54E-28	3.38E-26	0	0	0	5.86113	6.13862	5.75822	A1-	MIR854b
AT5G04985	8.67342	-3.3077	0.58259	-5.6776	1.37E-08	2.16E-07	0	0	0	4.89656	3.95523	3.28443	A1+	MIR854c
AT5G04985	18.993	-4.3878	0.47556	-9.2265	2.80E-20	1.34E-18	0	0	0	5.45435	5.26298	5.11744	A1-	MIR854c
AT5G04995	9.5194	-3.5707	0.57492	-6.2107	5.27E-10	1.02E-08	0	0	0	4.89656	3.71068	4.10751	A1+	MIR854a
AT5G04995	16.5718	-4.208	0.481	-8.7484	2.16E-18	9.14E-17	0	0	0	5.19788	5.04684	5.02993	A1-	MIR854a
NATs														
AT1G05147	55.603	-3.9888	0.38135	-10.46	1.32E-25	8.65E-24	2.62812	2.33157	1.10158	6.6234	7.33342	6.05278	A1-	NAT overlaps with AT1G15040
AT1G05147	36.5931	-3.5032	0.42623	-8.219	2.05E-16	7.77E-15	2.71916	2.41948	1.16147	5.91325	6.64615	5.67137	A1+	NAT overlaps with AT1G15040
AT1G05147	50.0758	-1.8293	0.45227	-4.0447	5.24E-05	6.77E-04	2.54637	2.25622	1.05325	3.75176	7.1949	7.06367	A2-	NAT overlaps with AT1G15040
AT1G20515	36.49	3.21983	0.47125	6.83259	8.34E-12	2.05E-10	5.93821	5.68836	6.61607	3.24283	0	1.80658	A1+	NAT overlaps with AT1G20520
AT2G05914	86.9176	-5.3054	0.45979	-11.539	8.41E-31	8.32E-29	1.6933	1.3957	0.69459	8.24034	7.01325	6.46654	A1+	NAT overlaps with AT2G05915~Potential natural antisense
AT2G05914	130.979	-6.1585	0.3527	-17.461	2.82E-68	9.80E-66	1.61959	1.32917	0.65347	7.86467	8.35271	7.8121	A1-	NAT overlaps with AT2G05915~Potential natural antisense
AT2G05914	17.89	-1.0098	0.17099	-5.9057	3.51E-09	4.65E-07	2.24791	1.90954	1.02486	5.03592	5.0543	5.20022	A2+	NAT overlaps with AT2G05915~Potential natural antisense
AT2G07213	61.6042	-2.7126	0.33336	-8.1372	4.04E-16	1.47E-14	3.74817	3.40322	4.09106	5.9747	7.12834	7.04187	A1-	NAT overlaps with hypothetical protein AT2G07215
AT2G07335	204.713	4.2595	0.30796	13.8312	1.65E-43	3.10E-41	8.34644	8.6383	8.85475	3.24283	3.41604	4.76714	A1+	NAT overlaps with AT2G20720
AT2G09885	42.0454	2.35017	0.36889	6.37099	1.88E-10	3.88E-09	6.37674	5.92623	6.26802	3.24283	3.71068	3.88164	A1+	NAT overlaps with AT2G47380
AT2G09885	43.322	1.8783	0.36522	5.14288	2.71E-07	4.07E-06	6.27012	5.81919	6.15931	3.02518	5.26298	3.83605	A1-	NAT overlaps with AT2G47380
AT2G09885	34.1467	2.54417	0.36924	6.89024	5.57E-12	2.75E-10	6.17336	5.72626	6.06877	0	3.44909	2.74912	A2-	NAT overlaps with AT2G47380
AT2G36792	982.815	2.76912	0.56163	4.93049	8.20E-07	9.97E-06	10.4993	10.8479	11.1884	7.69754	4.90796	5.3565	A1+	NAT overlaps with AT2G36790
AT2G36792	2263.46	0.78659	0.17055	4.61204	3.99E-06	3.66E-04	11.2462	11.6048	11.9308	9.65173	10.6985	10.7082	A2+	NAT overlaps with AT2G36790
AT2G36792	1241.01	0.8631	0.24354	3.54401	3.94E-04	3.92E-03	10.2934	10.6445	10.9864	9.99944	9.96305	9.09233	A2-	NAT overlaps with AT2G36790
AT3G01055	54.3909	3.02208	0.3739	8.08265	6.34E-16	2.31E-14	6.61732	6.7047	6.66085	4.16454	0	3.28443	A1+	NAT overlaps with AT3G01830
AT4G08285	17.5425	2.90922	0.52318	5.56064	2.69E-08	4.09E-07	4.93546	4.84448	5.4689	2.1331	0	1.16932	A1+	NAT overlaps with AT4G30430
AT4G09715	20.3617	2.97014	0.49928	5.94879	2.70E-09	4.75E-08	5.28456	5.2261	5.40716	2.60404	0	1.16932	A1+	NAT overlaps with AT4G38560
AT5G03195	118.879	2.3742	0.28058	8.4618	2.63E-17	1.07E-15	7.60168	7.34899	7.99882	4.97633	4.90796	5.48276	A1+	NAT overlaps with AT5G20720
AT5G03195	105.741	2.74656	0.28707	9.5674	1.10E-21	5.72E-20	7.49429	7.24078	7.88907	4.70005	4.48353	4.55311	A1-	NAT overlaps with AT5G20720
AT5G03195	97.7419	2.7385	0.29497	9.28407	1.63E-20	1.98E-18	7.39678	7.14677	7.7976	3.75176	4.6895	4.35462	A2-	NAT overlaps with AT5G20720
ncRNA														
AT1G05913	524.344	3.71216	0.26449	14.0351	9.51E-45	1.45E-42	9.80813	10.2348	9.70302	5.53062	6.95281	5.55378	A1-	ncRNA
AT1G05913	550.205	4.3675	0.23246	18.7879	9.49E-79	7.87E-76	9.91598	10.3436	9.81309	5.5973	5.88373	5.11789	A1+	ncRNA
AT1G05913	483.137	3.94678	0.2303	17.1372	7.83E-66	1.15E-62	9.71017	10.1403	9.61127	6.07705	5.51243	5.43798	A2-	ncRNA
AT1G06407	10.3041	-3.6327	0.57366	-6.3325	2.41E-10	4.92E-09	0	0	0	3.48023	4.6545	4.83185	A1+	ncRNA
AT1G06963	14.6039	-3.8021	0.55793	-6.8146	9.45E-12	2.30E-10	0.64015	0	0	4.6268	5.62557	3.99899	A1+	ncRNA
AT1G07343	6.66937	-2.5483	0.59234	-4.3022	1.69E-05	1.60E-04	0	0	0	1.42937	3.71068	4.76714	A1+	ncRNA

AT1G07347	7.98054	-3.408	0.57803	-5.896	3.72E-09	6.39E-08	0	0	0	3.86258	4.34682	3.99899	A1+	ncRNA
AT1G08757	16.7006	3.38556	0.56449	5.99756	2.00E-09	3.62E-08	5.06145	4.36275	5.62167	0	0	0	A1+	ncRNA
AT1G08997	22.6761	-3.7267	0.58028	-6.4222	1.34E-10	2.84E-09	0	0	0	2.1331	6.10263	6.04333	A1+	ncRNA
AT1G09937	1353.51	3.76154	0.19127	19.6664	4.19E-86	4.89E-83	11.1321	11.0878	11.638	7.53581	7.11744	7.6044	A1+	ncRNA
AT1G09937	1264.19	3.60862	0.23118	15.6095	6.27E-55	1.33E-52	11.0242	10.9789	11.5279	6.9354	8.03777	7.24984	A1-	ncRNA
AT1G09937	1141.72	4.32739	0.23535	18.3872	1.66E-75	9.17E-72	10.9262	10.8843	11.436	5.88745	6.20471	6.8655	A2-	ncRNA
AT1G16635	271.512	3.5185	0.28543	12.3271	6.47E-35	8.15E-33	8.49229	9.4528	8.83925	5.05192	5.47681	5.26573	A1+	ncRNA
AT1G16635	268.333	2.6678	0.30694	8.6916	3.57E-18	1.51E-16	8.38463	9.34406	8.7293	5.49299	6.88921	5.45643	A1-	ncRNA
AT1G16635	239.34	3.11371	0.28027	11.1095	1.13E-28	2.59E-26	8.28686	9.24956	8.63766	5.54616	5.34645	5.14317	A2-	ncRNA
AT1G26558	113.705	-3.255	0.38496	-8.4552	2.79E-17	1.13E-15	4.05639	3.75549	4.42008	6.33243	8.13724	8.1009	A1+	ncRNA
AT1G26558	28.61	-1.359	0.34671	-3.9196	8.87E-05	0.00085	3.95514	3.65494	4.31522	5.80081	5.04684	5.35204	A1-	ncRNA
AT1G67105	1941.54	-8.8146	0.232	-37.995	0.00E+00	0.00E+00	3.53746	2.41948	2.41385	11.9757	11.8874	11.8982	A1+	ncRNA
AT1G67105	935.662	-7.5258	0.2821	-26.677	8.67E-157	3.71E-153	3.43911	2.33157	2.32499	11.2769	10.7084	10.4964	A1-	ncRNA
AT1G67105	103.725	-2.3398	0.17502	-13.368	9.25E-41	9.12E-38	4.2336	3.06207	3.04322	7.7716	7.62634	7.4663	A2+	ncRNA
AT2G01422	50.4161	-2.8961	0.59234	-4.8893	1.01E-06	1.20E-05	0	0	0	4.16454	7.01325	7.30727	A1+	ncRNA
AT2G01422	3.18648	-1.7901	0.50061	-3.5758	0.000349	0.002848	0	0	0	3.54983	2.66509	2.02322	A1-	ncRNA
AT2G01422	10.4334	-0.7038	0.14596	-4.8222	1.42E-06	0.000144	0	0	0	4.03478	5.1002	3.89804	A2+	ncRNA
AT2G04365	7.71226	-3.2962	0.58138	-5.6697	1.43E-08	2.26E-07	0	0	0	4.16454	4.34682	3.45845	A1+	ncRNA
AT2G04365	23.6894	-1.4108	0.17343	-8.1349	4.12E-16	1.18E-13	0	0	0	5.91909	5.42276	5.38306	A2+	ncRNA
AT2G04655	99.4895	-6.9588	0.45972	-15.137	9.23E-52	2.61E-49	0	0	0	7.77765	7.56715	7.57635	A1+	ncRNA
AT2G04655	111.023	-6.7403	0.40779	-16.529	2.28E-61	5.85E-59	0	0	0	7.58956	8.0668	7.70316	A1-	ncRNA
AT2G04655	19.1717	-1.1754	0.1675	-7.0171	2.27E-12	4.21E-10	0	0	0	4.84311	5.716	5.20022	A2+	ncRNA
AT2G04885	42.2685	-5.0077	0.52825	-9.4799	2.55E-21	1.41E-19	0	0	0.69459	7.30812	5.88373	5.26573	A1+	ncRNA
AT2G04885	33.4998	-5.07	0.44234	-11.462	2.05E-30	1.72E-28	0	0	0.65347	6.31378	5.76624	6.11789	A1-	ncRNA
AT2G05015	15.9149	-4.3406	0.54873	-7.9102	2.57E-15	8.87E-14	0	0	0	5.19216	5.31093	4.4747	A1+	ncRNA
AT2G05015	13.0527	-3.7708	0.49661	-7.5931	3.12E-14	9.77E-13	0	0	0	4.48688	4.08983	5.38769	A1-	ncRNA
AT2G05695	16.9634	-4.3614	0.54997	-7.9302	2.19E-15	7.65E-14	0	0	0	5.6469	5.01973	4.4747	A1+	ncRNA
AT2G05695	18.1043	-4.3475	0.47693	-9.1157	7.82E-20	3.61E-18	0	0	0	5.19788	5.04684	5.38769	A1-	ncRNA
AT2G05705	4.45619	-2.1927	0.58942	-3.7201	1.99E-04	1.47E-03	0.64015	0.62641	0	3.24283	3.41604	3.08651	A1+	ncRNA
AT2G05705	8.14474	-2.8596	0.50286	-5.6867	1.30E-08	2.32E-07	0.60238	0.58899	0	4.04193	4.08983	4.11654	A1-	ncRNA
AT2G06002	148.679	-5.0923	0.27213	-18.713	3.90E-78	3.13E-75	3.04621	3.10637	2.71497	8.11378	8.08679	8.34765	A1+	ncRNA
AT2G06002	66.0513	-3.922	0.30653	-12.795	1.75E-37	2.00E-35	2.95175	3.01054	2.62211	7.14402	6.52082	7.20059	A1-	nc RNA
AT2G06002	43.3839	-1.3752	0.17965	-7.6549	1.93E-14	4.64E-12	3.72108	3.79342	3.36637	6.20648	6.34994	6.20009	A2+	ncRNA
AT2G06562	15.8757	-4.0557	0.5637	-7.1948	6.26E-13	1.73E-11	0	0	0	5.74125	4.90796	3.88164	A1+	ncRNA
AT2G06562	10.2489	-3.3006	0.506	-6.5228	6.90E-11	1.59E-09	0	0	0	3.81676	5.04684	4.11654	A1-	ncRNA
AT2G06562	16.463	-0.9937	0.16091	-6.1754	6.60E-10	9.29E-08	0	0	0	4.49483	5.65579	4.84882	A2+	ncRNA
AT2G15555	159.394	-6.4518	0.44714	-14.429	3.41E-47	7.61E-45	1.41979	1.06174	0	9.08442	7.67352	7.71145	A1+	ncRNA
AT2G15555	176.074	-6.8768	0.35539	-19.35	2.03E-83	1.16E-80	1.35313	1.00603	0	8.31637	8.59123	8.46124	A1-	ncRNA
AT2G15555	21.8654	-1.1713	0.17167	-6.823	8.91E-12	1.56E-09	1.93072	1.50483	0	5.20597	5.82936	5.1607	A2+	ncRNA
AT3G02832	193.665	1.99649	0.22139	9.01782	1.92E-19	9.41E-18	8.11319	8.51077	8.24512	5.95323	6.05094	6.56498	A1+	ncRNA
AT3G02832	182.61	2.02783	0.25255	8.02932	9.80E-16	3.46E-14	8.00563	8.40218	8.1353	5.56729	6.88921	5.88833	A1-	ncRNA
AT3G02832	151.398	2.81015	0.2561	10.9729	5.16E-28	1.15E-25	7.90795	8.30781	8.04377	4.69719	4.59395	5.43798	A2-	ncRNA
AT3G03595	120.608	2.52084	0.2497	10.0955	5.78E-24	3.94E-22	7.69017	7.7053	7.7229	4.4141	5.39625	5.31183	A1+	ncRNA
AT3G03595	106.587	3.12345	0.27363	11.415	3.52E-30	2.93E-28	7.58274	7.59694	7.61325	4.48688	4.7925	3.83605	A1-	ncRNA
AT3G03595	99.8122	3.07879	0.28233	10.9048	1.09E-27	2.34E-25	7.4852	7.50279	7.52186	5.09826	3.21224	4.08955	A2-	ncRNA
AT3G05595	12.6784	-3.459	0.57241	-6.0429	1.51E-09	2.76E-08	0	0.62641	0	2.9585	5.22024	5.06504	A1+	ncRNA
AT3G05955	10.8952	-2.6946	0.53541	-5.0328	4.83E-07	6.09E-06	0	1.3957	1.79647	4.52436	4.50885	4.20843	A1+	ncRNA
AT3G05955	11.1204	-2.3221	0.49586	-4.683	2.83E-06	3.59E-05	0	1.32917	1.71887	3.93374	5.26298	3.61329	A1-	ncRNA

AT3G06095	14.1896	-2.315	0.46869	-4.9394	7.84E-07	9.54E-06	2.12133	2.26486	2.03285	4.97633	4.16427	4.83185	A1+	ncRNA
AT3G06095	37.3989	-3.7629	0.36177	-10.401	2.44E-25	1.55E-23	2.0389	2.1793	1.95037	6.17809	6.34234	6.00768	A1-	ncRNA
AT3G06115	7.34329	-2.8539	0.59105	-4.8284	1.38E-06	1.61E-05	0	0	0	4.97633	2.54524	3.28443	A1+	ncRNA
AT3G06115	6.49981	-2.7936	0.51389	-5.4362	5.44E-08	9.05E-07	0	0	0	3.54983	4.08983	3.72897	A1-	ncRNA
AT3G06355	28019.6	-2.5225	0.56327	-4.4783	7.53E-06	7.65E-05	11.4682	10.4701	12.5264	15.5667	16.5452	13.7534	A1+	ncRNA
AT3G06465	1978.68	-9.4392	0.40928	-23.063	1.09E-117	2.55E-114	1.08201	1.3957	0.69459	12.7632	11.4914	10.9949	A1+	ncRNA
AT3G06465	1813.53	-9.7805	0.31876	-30.683	9.56E-207	1.23E-202	1.02605	1.32917	0.65347	11.9739	11.9454	11.5091	A1-	ncRNA
AT3G06465	397.158	-3.4	0.17759	-19.146	1.05E-81	3.98E-78	1.52355	1.90954	1.02486	9.43362	10.1986	9.00455	A2+	ncRNA
AT3G06495	4.79008	-2.5838	0.5923	-4.3623	1.29E-05	0.000125	0	0	0	4.02145	3.04536	2.85705	A1+	ncRNA
AT3G06715	16.702	-4.092	0.5634	-7.263	3.79E-13	1.06E-11	0	0	0	5.95323	4.50885	4.20843	A1+	ncRNA
AT3G06715	11.5795	-3.4309	0.5041	-6.8058	1.00E-11	2.53E-10	0	0	0	4.76464	5.04684	3.61329	A1-	ncRNA
AT3G06955	83.2251	-5.7123	0.39254	-14.552	5.67E-48	1.42E-45	1.41979	1.3957	1.16147	7.64968	7.19092	7.23733	A1+	ncRNA
AT3G06955	62.6378	-5.2388	0.37164	-14.096	4.00E-45	6.20E-43	1.35313	1.32917	1.10158	6.96319	6.75292	7.1496	A1-	ncRNA
AT3G09855	181.833	2.88711	0.21632	13.3467	1.24E-40	2.05E-38	8.2728	8.42207	8.33075	5.25748	5.22024	5.56118	A1+	ncRNA
AT3G09855	171.338	2.69428	0.21346	12.6217	1.60E-36	1.76E-34	8.16519	8.3135	8.2209	5.45435	5.45092	5.42247	A1-	ncRNA
AT3G09855	152.708	3.28773	0.24541	13.3968	6.32E-41	3.24E-38	8.06747	8.21915	8.12935	4.91162	4.38148	4.64598	A2-	ncRNA
AT3G60176	10.8432	-3.6809	0.57261	-6.4283	1.29E-10	2.73E-09	0	0	0	5.19216	3.95523	3.99899	A1+	ncRNA
AT3G60176	15.3414	-3.7346	0.49671	-7.5188	5.53E-14	1.69E-12	0	0	0	4.4082	5.76624	4.2772	A1-	ncRNA
AT4G04195	36.283	2.39521	0.4134	5.79399	6.87E-09	1.14E-07	5.93821	5.77686	6.30234	2.1331	3.04536	3.99899	A1+	ncRNA
AT4G04195	36.418	1.98611	0.37501	5.29622	1.18E-07	1.88E-06	5.83207	5.67002	6.1936	2.52621	4.48353	3.93573	A1-	ncRNA
AT4G04195	28.94	2.7657	0.40252	6.87099	6.38E-12	3.09E-10	5.73578	5.57728	6.10302	0	0	2.97594	A2-	ncRNA
AT4G04223	150.452	-2.3697	0.20785	-11.401	4.13E-30	3.96E-28	5.85713	5.26742	5.54731	8.07806	7.86529	8.03372	A1+	ncRNA
AT4G04223	157.563	-2.5207	0.20698	-12.179	4.03E-34	3.88E-32	5.7511	5.16145	5.43955	8.08302	8.20382	7.97912	A1-	ncRNA
AT4G04223	153.261	-1.1164	0.16008	-6.974	3.08E-12	5.65E-10	6.5943	6.00917	6.27747	7.7716	8.0158	7.71166	A2+	ncRNA
AT4G04945	9.01574	-3.4878	0.57706	-6.0441	1.50E-09	2.75E-08	0	0	0	4.81212	3.95523	3.75389	A1+	ncRNA
AT4G04945	7.7369	-3.0977	0.5104	-6.0692	1.29E-09	2.62E-08	0	0	0	3.93374	3.54662	4.48892	A1-	ncRNA
AT4G05025	14.3158	-4.2528	0.5509	-7.7198	1.17E-14	3.79E-13	0	0	0	5.19216	4.78679	4.62832	A1+	ncRNA
AT4G05025	14.4912	-4.0232	0.48681	-8.2645	1.40E-16	5.32E-15	0	0	0	4.88574	4.7925	5.02993	A1-	ncRNA
AT4G05205	29.7175	-4.8192	0.45126	-10.679	1.27E-26	8.88E-25	0.60238	0	0	5.53062	6.02485	6.11789	A1-	ncRNA
AT4G05275	95.9977	-6.8314	0.46727	-14.62	2.10E-48	5.30E-46	0	0	0	7.93566	7.28341	7.47871	A1+	ncRNA
AT4G05275	31.9252	-4.5658	0.47921	-9.5277	1.61E-21	8.29E-20	0	0	0	4.56149	6.67963	6.09651	A1-	ncRNA
AT4G05275	8.81678	-0.6748	0.14433	-4.6757	2.93E-06	0.000276	0	0	0	4.35716	4.49088	3.69168	A2+	ncRNA
AT4G05715	63.737	-6.3093	0.48157	-13.102	3.22E-39	5.08E-37	0	0	0	7.24511	6.57474	7.11267	A1+	ncRNA
AT4G05715	53.4201	-5.786	0.43636	-13.26	3.97E-40	5.11E-38	0	0	0	7.0304	6.02485	6.99642	A1-	ncRNA
AT4G06225	77.8908	2.70326	0.32586	8.29585	1.08E-16	4.17E-15	6.80915	7.32473	7.19916	4.6268	3.04536	4.39129	A1+	ncRNA
AT4G06225	73.9529	2.32362	0.29634	7.84095	4.47E-15	1.49E-13	6.70218	7.21653	7.08974	4.48688	3.54662	4.73014	A1-	ncRNA
AT4G06225	70.3751	2.32671	0.30318	7.67433	1.66E-14	1.21E-12	6.60508	7.12254	6.99857	4.69719	4.59395	3.76455	A2-	ncRNA
AT4G06835	36.6034	-5.4522	0.48696	-11.196	4.25E-29	3.83E-27	0.64015	0	0	6.20578	6.20076	6.22294	A1+	ncRNA
AT4G06835	42.1724	-5.3755	0.43347	-12.401	2.58E-35	2.68E-33	0.60238	0	0	6.69143	6.13862	6.35243	A1-	ncRNA
AT4G07250	7.26167	-3.2943	0.58113	-5.6688	1.44E-08	2.27E-07	0	0	0	4.02145	3.71068	4.10751	A1+	ncRNA
AT4G07250	4.94537	-2.4905	0.51504	-4.8356	1.33E-06	1.79E-05	0	0	0	3.81676	2.66509	3.61329	A1-	ncRNA
AT4G12917	35.4533	1.76577	0.35961	4.91017	9.10E-07	1.10E-05	5.85713	5.62619	6.02937	3.86258	3.95523	3.99899	A1+	ncRNA
AT4G12917	28.7549	2.66647	0.40379	6.60369	4.01E-11	9.54E-10	5.7511	5.51958	5.92092	2.52621	2.66509	2.61074	A1-	ncRNA
AT4G15242	24.6206	-4.5447	0.51247	-8.8683	7.43E-19	3.45E-17	0.64015	0	0.69459	4.89656	6.05094	5.74041	A1+	ncRNA
AT4G15242	40.0767	-5.1988	0.42689	-12.178	4.06E-34	3.90E-32	0.60238	0	0.65347	6.39763	6.02485	6.53834	A1-	ncRNA
AT4G15242	9.18828	-0.603	0.14349	-4.2024	2.64E-05	2.07E-03	0.95419	0	1.02486	3.73469	4.85451	3.79855	A2+	ncRNA
AT5G02055	88.6003	3.09923	0.39142	7.91795	2.41E-15	8.34E-14	7.34839	7.88196	6.54141	4.16454	4.16427	3.28443	A1+	ncRNA
AT5G02055	86.7728	2.55581	0.36126	7.0748	1.50E-12	4.05E-11	7.24111	7.77354	6.43244	4.04193	5.26298	3.93573	A1-	ncRNA

AT5G02055	78.6984	2.50489	0.35351	7.08576	1.38E-12	7.59E-11	7.14371	7.67933	6.34167	3.75176	4.49163	3.88121	A2-	ncRNA
AT5G02645	190.29	2.92884	0.22398	13.0762	4.50E-39	7.02E-37	8.35876	8.53635	8.35001	5.43737	4.78679	5.67137	A1+	ncRNA
AT5G02645	183.061	2.61569	0.21039	12.4327	1.74E-35	1.83E-33	8.25114	8.42775	8.24017	5.53062	5.90133	5.55378	A1-	ncRNA
AT5G02645	162.595	3.1565	0.23941	13.1847	1.07E-39	5.27E-37	8.15339	8.33338	8.14861	5.2635	4.49163	4.88827	A2-	ncRNA
AT5G05005	11.2538	-3.2637	0.58609	-5.5686	2.57E-08	3.92E-07	0	0	0	5.6469	3.41604	3.28443	A1+	ncRNA
AT5G05005	21.7674	-4.5728	0.46911	-9.7478	1.88E-22	1.03E-20	0	0	0	5.41464	5.61718	5.38769	A1-	ncRNA
AT5G09505	95.9383	3.0653	0.38984	7.86295	3.75E-15	1.28E-13	7.39701	8.05121	6.60695	3.68404	4.50885	3.88164	A1+	ncRNA
AT5G09505	94.1078	2.41921	0.35067	6.89891	5.24E-12	1.34E-10	7.2897	7.94273	6.49792	4.4082	5.04684	4.55311	A1-	ncRNA
AT5G09505	83.3289	2.67183	0.3607	7.40726	1.29E-13	8.28E-12	7.19228	7.84847	6.4071	3.21938	4.26223	3.98914	A2-	ncRNA
AT5G38005	69.5746	-3.2502	0.47336	-6.8661	6.60E-12	1.64E-10	3.14059	3.01228	3.17624	4.72243	7.70731	7.341	A1+	ncRNA
AT5G38005	33.7313	-2.713	0.3673	-7.3864	1.51E-13	4.45E-12	3.04528	2.91734	3.07866	5.86113	5.04684	6.53834	A1-	ncRNA

rRNAs

AT2G01010	3295391	-3.7511	0.50786	-7.3859	1.51E-13	4.44E-12	16.6923	18.2679	17.3372	22.4289	23.3183	21.5544	A1+	rRNA
AT2G01010	3526008	-2.3805	0.45493	-5.2326	1.67E-07	3.95E-06	16.4863	18.0643	17.1351	23.7354	22.0242	21.2034	A2-	rRNA
AT3G41768	3791353	-3.787	0.50986	-7.4275	1.11E-13	3.28E-12	16.8147	18.406	17.4623	22.631	23.5256	21.7527	A1+	rRNA
AT3G41768	4039253	-2.3967	0.45495	-5.2681	1.38E-07	3.32E-06	16.6087	18.2024	17.2602	23.9308	22.23	21.3995	A2-	rRNA

snoRNAs

AT1G04517	23.2191	3.23723	0.45198	7.16241	7.93E-13	2.19E-11	5.45983	5.50363	5.63518	1.13103	0	1.60562	A1-	snoRNA
AT1G04517	25.2941	3.29343	0.49126	6.7041	2.03E-11	4.73E-10	5.56543	5.61022	5.74325	0	0	2.58406	A1+	snoRNA
AT1G04517	21.7051	2.95374	0.41947	7.04165	1.90E-12	1.02E-10	5.36406	5.41113	5.54522	0	1.40794	1.10072	A2-	snoRNA
AT1G04527	27.0323	3.33254	0.49364	6.75098	1.47E-11	3.48E-10	6.00246	5.30759	5.80838	1.42937	0	2.24707	A1+	snoRNA
AT1G04527	24.5934	3.3889	0.463	7.31943	2.49E-13	7.18E-12	5.89624	5.20154	5.70022	1.13103	0	1.01552	A1-	snoRNA
AT1G04527	23.2525	2.82353	0.42082	6.70964	1.95E-11	8.72E-10	5.79987	5.10954	5.61018	0	0	2.14856	A2-	snoRNA
AT1G05247	259.679	3.80791	0.30531	12.4725	1.06E-35	1.40E-33	8.42121	9.42568	8.79173	4.6268	5.31093	4.6994	A1+	snoRNA
AT1G05247	256.583	2.06762	0.47463	4.35624	1.32E-05	0.000149	8.31356	9.31695	8.68179	4.94268	6.88921	4.88781	A1-	snoRNA
AT1G05247	225.668	3.52519	0.30059	11.7274	9.23E-32	2.75E-29	8.21581	9.22244	8.59015	4.69719	5.01872	4.43316	A2-	snoRNA
AT1G05917	544.954	4.51852	0.24304	18.5917	3.75E-77	2.92E-74	9.90594	10.3394	9.80414	5.37986	5.82339	4.83185	A1+	snoRNA
AT1G05917	518.529	3.17638	0.41104	7.72774	1.09E-14	3.54E-13	9.79809	10.2306	9.69407	5.45435	6.88921	5.11744	A1-	snoRNA
AT1G05917	476.344	4.15493	0.23492	17.6864	5.34E-70	1.47E-66	9.70013	10.136	9.60231	5.66912	5.40393	5.18915	A2-	snoRNA
AT1G06087	93.8706	1.42236	0.25318	5.61801	1.93E-08	3.00E-07	7.06523	7.38228	6.89626	5.6469	5.47681	5.77373	A1+	snoRNA
AT1G06087	86.0362	1.51432	0.28241	5.36209	8.23E-08	1.34E-06	6.9581	7.27406	6.78702	4.76464	5.76624	5.67413	A1-	snoRNA
AT1G06087	65.1046	2.618	0.37415	6.99711	2.61E-12	1.38E-10	6.86085	7.18004	6.69601	2.36651	1.40794	4.57847	A2-	snoRNA
AT1G06243	214.125	2.9489	0.27184	10.8477	2.04E-27	1.66E-25	8.34891	9.00028	8.2971	5.54593	5.82339	5.11789	A1+	snoRNA
AT1G06243	216.927	2.13092	0.28321	7.52406	5.31E-14	1.62E-12	8.24129	8.8916	8.18727	5.53062	6.95281	6.09651	A1-	snoRNA
AT1G06243	193.979	2.4222	0.27596	8.77732	1.67E-18	1.71E-16	8.14355	8.79715	8.09573	6.39469	5.40393	5.39938	A2-	snoRNA
AT1G06453	3653.03	2.3135	0.10148	22.7976	4.84E-115	8.89E-112	12.4754	12.6091	12.6315	10.1187	10.2753	10.3276	A1+	snoRNA
AT1G06453	3433.68	2.18563	0.15446	14.1503	1.86E-45	2.90E-43	12.3675	12.5003	12.5213	10.0191	10.5733	10.0902	A1-	snoRNA
AT1G06453	2909.26	3.00851	0.26464	11.3682	6.02E-30	1.49E-27	12.2694	12.4056	12.4295	8.75929	8.32999	9.69246	A2-	snoRNA
AT1G07593	28.6164	2.74968	0.45345	6.06395	1.33E-09	2.45E-08	5.47775	5.5276	6.1347	2.60404	1.77337	2.85705	A1+	snoRNA
AT1G07593	24.9173	3.2407	0.45896	7.06097	1.65E-12	4.46E-11	5.37229	5.42115	6.02613	0	0	2.02322	A1-	snoRNA
AT1G07593	23.2425	2.94472	0.42513	6.92663	4.31E-12	2.16E-10	5.27667	5.32878	5.93572	0	0	1.71775	A2-	snoRNA
AT1G07897	586.649	2.60385	0.22082	11.7919	4.30E-32	4.70E-30	9.47791	10.1836	10.1989	7.03764	7.54864	7.28433	A1+	snoRNA
AT1G07897	537.784	2.11072	0.44942	4.6965	2.65E-06	3.37E-05	9.3701	10.0748	10.0888	6.22475	7.97788	6.25929	A1-	snoRNA
AT1G07897	461.63	3.90921	0.27268	14.3364	1.30E-46	8.94E-44	9.27218	9.98019	9.99704	5.09826	4.94319	5.91934	A2-	snoRNA
AT1G08117	31.5781	2.23211	0.39642	5.6306	1.80E-08	2.80E-07	5.6156	5.80519	5.90087	3.68404	3.41604	2.85705	A1+	snoRNA
AT1G08117	27.6922	2.53613	0.39966	6.3457	2.21E-10	4.83E-09	5.50991	5.69831	5.79259	2.52621	2.66509	2.83386	A1-	snoRNA

AT1G08117	26.9477	2.11916	0.36885	5.74531	9.18E-09	2.71E-07	5.41407	5.60554	5.70243	3.21938	2.57549	3.1719	A2-	snoRNA
AT1G08353	31.7291	2.58154	0.43695	5.90814	3.46E-09	5.96E-08	5.85713	5.5105	6.14734	1.42937	3.04536	3.45845	A1+	snoRNA
AT1G08353	29.3666	2.50084	0.40266	6.21073	5.27E-10	1.11E-08	5.7511	5.40408	6.03876	2.52621	2.66509	3.02706	A1-	snoRNA
AT1G08353	25.2749	3.06514	0.41964	7.30422	2.79E-13	1.71E-11	5.65491	5.31173	5.94834	0	1.40794	1.10072	A2-	snoRNA
AT1G08937	141.786	2.3906	0.25257	9.46525	2.93E-21	1.61E-19	7.86624	7.71654	8.15722	5.05192	5.31093	5.77373	A1+	snoRNA
AT1G08937	129.859	2.45371	0.25066	9.78906	1.25E-22	6.95E-21	7.75875	7.60817	8.04742	4.99746	5.04684	5.45643	A1-	snoRNA
AT1G08937	112.926	3.12461	0.30058	10.3951	2.61E-25	4.76E-23	7.66115	7.51402	7.95591	3.75176	3.21224	4.77221	A2-	snoRNA
AT1G09087	39.5854	2.25042	0.3866	5.82102	5.85E-09	9.77E-08	5.80043	6.00162	6.45252	3.24283	3.41604	3.99899	A1+	snoRNA
AT1G09087	37.3151	2.20436	0.37015	5.95535	2.60E-09	5.12E-08	5.69448	5.89448	6.34363	3.02518	4.08983	3.61329	A1-	snoRNA
AT1G09087	31.9838	2.76844	0.39516	7.00584	2.46E-12	1.30E-10	5.59836	5.80147	6.25293	3.21938	2.57549	1.10072	A2-	snoRNA
AT1G09787	206.985	2.71737	0.23767	11.4336	2.84E-30	2.76E-28	8.21432	8.68288	8.57256	5.82982	5.99732	5.26573	A1+	snoRNA
AT1G09787	192.223	2.62496	0.29801	8.80836	1.27E-18	5.45E-17	8.10673	8.57426	8.46266	4.32498	6.13862	5.88833	A1-	snoRNA
AT1G09787	162.347	3.9627	0.30465	13.0075	1.11E-38	5.10E-36	8.00902	8.47986	8.37106	2.36651	2.92871	4.43316	A2-	snoRNA
AT1G12013	1278.11	2.45185	0.18945	12.9421	2.60E-38	3.89E-36	11.1347	10.7769	11.3083	8.42936	8.28997	8.9013	A1+	snoRNA, SNOR111 small nucleolar RNA111, Encodes a H/ACA-box snoRNA (snoR111)
AT1G12013	1167.34	2.56664	0.18972	13.5284	1.06E-41	1.47E-39	11.0267	10.668	11.1981	8.11433	8.12318	8.612	A1-	snoRNA, SNOR111 small nucleolar RNA111, Encodes a H/ACA-box snoRNA (snoR111)
AT1G12013	1018.21	3.25813	0.28329	11.5011	1.30E-30	3.46E-28	10.9287	10.5734	11.1063	6.46427	6.73915	7.92192	A2-	snoRNA, SNOR111 small nucleolar RNA111, Encodes a H/ACA-box snoRNA (snoR111)
AT1G74456	88.7294	2.62364	0.27414	9.57053	1.06E-21	6.09E-20	7.28264	7.26478	7.29317	4.6268	4.78679	4.30275	A1+	snoRNA
AT1G74456	86.4457	2.12116	0.26587	7.97816	1.49E-15	5.19E-14	7.17539	7.15662	7.18371	4.4082	5.04684	5.23951	A1-	snoRNA
AT1G74456	76.9808	2.35378	0.27959	8.41883	3.80E-17	3.53E-15	7.07802	7.06265	7.09249	4.13977	3.83072	4.94298	A2-	snoRNA
AT1G75163	51.0537	2.65433	0.38269	6.93597	4.03E-12	1.02E-10	6.29701	6.92726	6.23287	2.9585	3.95523	3.75389	A1+	snoRNA
AT1G75163	49.1972	2.39741	0.35643	6.72624	1.74E-11	4.26E-10	6.19047	6.81929	6.12419	3.68946	4.48353	3.34975	A1-	snoRNA
AT1G75163	43.9907	2.62445	0.36793	7.13301	9.82E-13	5.52E-11	6.09378	6.7255	6.03368	4.13977	2.57549	2.74912	A2-	snoRNA
AT2G06855	20.9093	3.14331	0.50819	6.18533	6.20E-10	1.19E-08	5.54832	5.30759	5.17991	2.1331	1.77337	0	A1+	snoRNA
AT2G06855	20.9316	1.89439	0.43617	4.34325	1.40E-05	0.000157	5.44274	5.20154	5.07287	1.75715	0	3.72897	A1-	snoRNA
AT2G06855	19.2337	2.14174	0.40479	5.29103	1.22E-07	2.97E-06	5.347	5.10954	4.98382	2.36651	1.40794	2.74912	A2-	snoRNA
AT2G07605	366.177	4.22881	0.23108	18.3	8.27E-75	5.45E-72	9.20917	9.57816	9.53743	4.81212	5.39625	5.06504	A1+	snoRNA
AT2G07605	338.451	4.26705	0.23508	18.1511	1.26E-73	5.39E-71	9.10139	9.46941	9.42738	4.82646	5.04684	4.83714	A1-	snoRNA
AT2G07605	316.633	4.12166	0.24038	17.1466	6.66E-66	1.05E-62	9.0035	9.37489	9.33565	4.69719	4.6895	4.94298	A2-	snoRNA
AT2G08375	28.287	3.10888	0.47521	6.54217	6.06E-11	1.33E-09	5.72627	6.1078	5.40716	2.1331	0	2.58406	A1+	snoRNA
AT2G08375	28.1379	2.08836	0.40373	5.17271	2.31E-07	3.50E-06	5.62042	6.00054	5.29966	3.81676	0	2.83386	A1-	snoRNA
AT2G08375	24.4681	2.65318	0.40863	6.49283	8.42E-11	3.43E-09	5.52441	5.90741	5.2102	0	2.10673	2.14856	A2-	snoRNA
AT2G08725	75.553	2.69771	0.31962	8.44036	3.16E-17	1.28E-15	6.78743	7.17536	7.21125	4.6268	4.16427	3.75389	A1+	snoRNA
AT2G08725	69.0487	2.79615	0.31482	8.88185	6.58E-19	2.87E-17	6.68048	7.06724	7.10183	3.68946	4.08983	4.02897	A1-	snoRNA
AT2G08725	63.951	2.59737	0.31218	8.32016	8.78E-17	7.85E-15	6.5834	6.97331	7.01065	2.36651	3.98933	4.18342	A2-	snoRNA
AT2G08900	31.5145	3.11811	0.46749	6.6699	2.56E-11	5.91E-10	5.77123	6.31904	5.58497	2.1331	0	2.85705	A1+	snoRNA
AT2G08900	32.9119	2.32874	0.40691	5.72302	1.05E-08	1.90E-07	5.66531	6.21155	5.47715	2.19233	4.48353	2.83386	A1-	snoRNA
AT2G08900	28.0801	2.47554	0.39746	6.22839	4.71E-10	1.78E-08	5.56923	6.11822	5.38741	2.36651	1.40794	2.97594	A2-	snoRNA
AT2G08910	99.5398	3.7655	0.33344	11.2929	1.42E-29	1.32E-27	7.55959	7.75699	7.34897	3.48023	3.71068	3.45845	A1+	snoRNA
AT2G08910	91.1269	3.80567	0.33069	11.5084	1.20E-30	1.01E-28	7.45221	7.64861	7.23947	3.22203	2.66509	3.34975	A1-	snoRNA
AT2G08910	85.1578	3.65011	0.32777	11.1361	8.37E-29	1.97E-26	7.35472	7.55444	7.14823	2.36651	3.21224	3.1719	A2-	snoRNA
AT2G09705	327.917	2.4078	0.18287	13.1665	1.37E-39	2.20E-37	8.94226	9.11931	9.28053	6.39182	6.74705	6.8095	A1+	snoRNA
AT2G09705	292.248	2.91939	0.20734	14.0804	5.01E-45	7.71E-43	8.83452	9.01061	9.17052	5.76968	6.60241	5.88833	A1-	snoRNA

AT2G09705	260.595	3.41382	0.26524	12.8707	6.58E-38	2.79E-35	8.73667	8.91614	9.07882	5.98536	3.98933	5.31893	A2-	snoRNA
AT2G35387	89.6719	3.69075	0.33537	11.0051	3.61E-28	3.09E-26	7.28264	7.5549	7.38143	3.24283	3.71068	3.45845	A1+	snoRNA
AT2G35387	83.8169	3.59575	0.32466	11.0753	1.65E-28	1.27E-26	7.17539	7.4466	7.27192	3.02518	4.08983	3.34975	A1-	snoRNA
AT2G35387	77.2519	3.34362	0.31995	10.4504	1.46E-25	2.68E-23	7.07802	7.35251	7.18067	0	3.98933	3.1719	A2-	snoRNA
AT2G35744	413.449	3.19713	0.24074	13.2803	3.01E-40	4.93E-38	9.09887	9.92656	9.52537	6.2385	6.46052	6.01574	A1+	snoRNA
AT2G35744	384.233	3.09173	0.23796	12.9928	1.34E-38	1.61E-36	8.99111	9.81778	9.41533	6.29203	6.13862	6.09651	A1-	snoRNA
AT2G35744	366.273	2.80174	0.27096	10.34	4.64E-25	8.40E-23	8.89324	9.72323	9.3236	6.8232	6.29862	5.65014	A2-	snoRNA
AT2G35747	335.381	3.30122	0.26305	12.5499	3.98E-36	5.41E-34	9.1062	9.73274	8.80774	5.82982	5.82339	5.77373	A1+	snoRNA
AT2G35747	317.216	3.02151	0.26165	11.548	7.56E-31	6.47E-29	8.99844	9.62398	8.6978	5.89038	6.34234	5.73073	A1-	snoRNA
AT2G35747	303.296	2.66789	0.26793	9.95724	2.34E-23	3.60E-21	8.90056	9.52944	8.60616	6.59409	6.03319	5.83507	A2-	snoRNA
AT3G01155	102.551	2.52621	0.279	9.05467	1.37E-19	6.78E-18	7.16966	7.6517	7.56225	4.72243	5.12347	4.6994	A1+	snoRNA
AT3G01155	92.5799	2.94955	0.31689	9.30775	1.31E-20	6.41E-19	7.06247	7.54336	7.45266	3.22203	5.26298	4.19911	A1-	snoRNA
AT3G01155	80.3469	3.53938	0.33368	10.6071	2.76E-26	5.40E-24	6.96515	7.44923	7.36133	2.36651	3.44909	2.97594	A2-	snoRNA
AT3G02445	119.844	3.11353	0.35442	8.78477	1.57E-18	7.03E-17	7.55531	8.33487	7.25863	3.86258	4.16427	4.83185	A1+	snoRNA
AT3G02445	110.355	3.26627	0.34167	9.5598	1.18E-21	6.15E-20	7.44794	8.22632	7.14918	4.04193	4.48353	3.72897	A1-	snoRNA
AT3G02445	100.524	3.36819	0.35039	9.61261	7.07E-22	9.47E-20	7.35045	8.13199	7.05798	3.21938	3.21224	3.76455	A2-	snoRNA
AT3G03175	21.5676	2.57279	0.47108	5.46148	4.72E-08	6.95E-07	5.03712	5.42179	5.4689	2.60404	2.54524	1.80658	A1+	snoRNA
AT3G03175	19.8478	2.53468	0.44521	5.69321	1.25E-08	2.25E-07	4.93256	5.31552	5.36128	1.13103	2.66509	2.34668	A1-	snoRNA
AT3G03175	17.3126	2.65593	0.43034	6.1717	6.76E-10	2.49E-08	4.8378	5.22332	5.27172	0	0	1.71775	A2-	snoRNA
AT3G05335	90.3758	2.5158	0.27337	9.20285	3.49E-20	1.78E-18	7.24064	7.4331	7.22324	4.6268	4.34682	4.89377	A1+	snoRNA
AT3G05335	86.9018	2.44937	0.28494	8.59616	8.24E-18	3.38E-16	7.13341	7.32485	7.11381	4.14258	5.61718	4.42174	A1-	snoRNA
AT3G05335	78.0512	2.60176	0.29381	8.85532	8.34E-19	8.86E-17	7.03606	7.23081	7.02263	5.09826	3.21224	4.27156	A2-	snoRNA
AT3G09865	179.782	2.94659	0.2179	13.5229	1.15E-41	2.00E-39	8.25708	8.41059	8.32519	5.25748	5.22024	5.39982	A1+	snoRNA
AT3G09865	169.13	2.77407	0.21733	12.7646	2.58E-37	2.94E-35	8.14948	8.30203	8.21535	5.37382	5.45092	5.278	A1-	snoRNA
AT3G09865	149.714	3.40665	0.25309	13.4603	2.68E-41	1.41E-38	8.05177	8.20768	8.1238	4.44524	4.13223	4.64598	A2-	snoRNA
AT3G13855	288.398	3.4684	0.22624	15.3309	4.76E-53	1.45E-50	9.06918	9.02157	9.11018	5.25748	4.6545	5.98762	A1+	snoRNA, U6 small nucleolar RNA26
AT3G13855	262.229	3.84199	0.21965	17.4913	1.67E-68	6.04E-66	8.96142	8.91289	9.00019	4.94268	4.48353	5.07435	A1-	snoRNA, U6 small nucleolar RNA26
AT3G13855	241.27	4.33395	0.25273	17.1487	6.43E-66	1.05E-62	8.86355	8.81843	8.90851	4.44524	3.98933	4.18342	A2-	snoRNA, U6 small nucleolar RNA26
AT3G14735	288.307	3.46788	0.2263	15.3243	5.26E-53	1.57E-50	9.06918	9.02006	9.11018	5.25748	4.6545	5.98762	A1+	snoRNA, U6 small nucleolar RNA1
AT3G14735	262.343	3.82121	0.21871	17.472	2.34E-68	8.25E-66	8.96142	8.91138	9.00019	4.99746	4.48353	5.07435	A1-	snoRNA, U6 small nucleolar RNA1
AT3G21805	794.107	4.39164	0.27174	16.1613	9.46E-59	3.52E-56	10.0843	10.973	10.5413	6.50374	5.62557	5.48276	A1+	snoRNA
AT3G21805	770.431	2.40497	0.4756	5.05675	4.26E-07	6.21E-06	9.9764	10.8642	10.4311	5.70532	7.94698	6.27841	A1-	snoRNA
AT3G21805	696.925	3.90922	0.24789	15.7701	5.00E-56	4.80E-53	9.87842	10.7696	10.3393	5.78241	6.32861	6.12261	A2-	snoRNA
AT3G47348	326.141	2.6319	0.18289	14.3906	5.93E-47	1.29E-44	9.0974	9.26412	9.05422	6.13802	6.81059	6.4252	A1+	snoRNA
AT3G47348	309.121	2.37844	0.17546	13.5556	7.34E-42	1.04E-39	8.98964	9.15541	8.94423	6.49603	6.60241	6.67443	A1-	snoRNA
AT3G47348	282.787	2.57545	0.18811	13.6912	1.15E-42	6.84E-40	8.89177	9.06092	8.85256	6.39469	5.99629	6.38219	A2-	snoRNA
AT3G50825	157.044	2.84409	0.22593	12.5883	2.45E-36	3.35E-34	8.06887	8.21063	8.09984	5.25748	5.01973	5.26573	A1+	snoRNA
AT3G50825	141.441	2.9017	0.24621	11.7853	4.65E-32	4.18E-30	7.96132	8.10211	7.99006	5.10115	2.66509	5.15929	A1-	snoRNA
AT3G50825	129.736	3.40733	0.26457	12.8789	5.92E-38	2.56E-35	7.86365	8.00781	7.89856	4.13977	4.38148	4.08955	A2-	snoRNA
AT4G06010	172.485	2.59451	0.25577	10.1439	3.53E-24	2.46E-22	7.87999	8.54477	8.19424	5.74125	5.47681	5.3565	A1+	snoRNA
AT4G06010	164.242	2.35563	0.25103	9.38399	6.35E-21	3.16E-19	7.77249	8.43618	8.08444	5.73786	5.90133	5.38769	A1-	snoRNA
AT4G06010	156.621	2.17274	0.25812	8.4177	3.84E-17	3.55E-15	7.67488	8.3418	7.99292	6.16325	5.66127	5.31893	A2-	snoRNA
AT4G06240	72.3979	3.11588	0.34639	8.99517	2.36E-19	1.15E-17	6.72787	7.26478	7.15602	4.02145	3.04536	3.75389	A1+	snoRNA
AT4G06240	68.3489	2.75411	0.32373	8.50755	1.78E-17	7.16E-16	6.62096	7.15662	7.04663	3.54983	3.54662	4.2772	A1-	snoRNA
AT4G06240	65.4068	2.54255	0.31915	7.96661	1.63E-15	1.34E-13	6.52391	7.06265	6.95548	4.13977	4.38148	3.34439	A2-	snoRNA
AT4G06250	83.8119	3.15545	0.31751	9.93803	2.84E-23	1.85E-21	7.09501	7.38228	7.29885	3.68404	4.50885	3.61372	A1+	snoRNA
AT4G06250	78.8823	2.69863	0.29125	9.26573	1.94E-20	9.39E-19	6.98786	7.27406	7.18938	4.04193	3.54662	4.55311	A1-	snoRNA
AT4G06250	72.3855	2.95728	0.30423	9.72056	2.46E-22	3.47E-20	6.89058	7.18004	7.09816	3.75176	3.65249	3.76455	A2-	snoRNA

AT4G06310	84.4838	3.01907	0.32626	9.25361	2.17E-20	1.13E-18	7.06523	7.39166	7.30451	3.24283	4.90796	3.75389	A1+	snoRNA
AT4G06310	78.5438	2.72421	0.29369	9.27583	1.76E-20	8.61E-19	6.9581	7.28343	7.19503	4.04193	3.54662	4.48892	A1-	snoRNA
AT4G06310	71.119	3.00767	0.31092	9.67358	3.90E-22	5.32E-20	6.86085	7.1894	7.10381	2.36651	3.83072	3.76455	A2-	snoRNA
AT4G07625	38.7661	3.55326	0.44997	7.89672	2.86E-15	9.84E-14	5.92501	6.43237	6.30234	2.1331	1.77337	2.24707	A1+	snoRNA
AT4G07625	36.644	3.51832	0.43075	8.16787	3.14E-16	1.15E-14	5.81889	6.32478	6.1936	1.75715	3.54662	0	A1-	snoRNA
AT4G07625	35.4242	2.65996	0.37604	7.07357	1.51E-12	8.25E-11	5.72261	6.23134	6.10302	3.21938	3.21224	1.71775	A2-	snoRNA
AT4G07635	131.827	3.9066	0.31866	12.2594	1.50E-34	1.82E-32	7.95659	8.08338	7.86745	4.6268	3.71068	2.58406	A1+	snoRNA
AT4G07635	126.099	3.27576	0.26304	12.4533	1.34E-35	1.42E-33	7.84907	7.97489	7.75774	4.4082	4.48353	4.35128	A1-	snoRNA
AT4G07635	121.264	3.00386	0.26873	11.1781	5.22E-29	1.24E-26	7.75144	7.88062	7.66631	5.41174	4.38148	3.98914	A2-	snoRNA
AT4G15258	81.0574	2.60506	0.30344	8.58522	9.07E-18	3.86E-16	6.86552	7.33934	7.21726	4.4141	4.6545	4.20843	A1+	snoRNA, SNOR37-2 small nucleolar RNA37-2 Encodes a C/D box snoRNA (snoR37-2)
AT4G39361	279.067	2.63854	0.20948	12.5959	2.22E-36	3.06E-34	8.62678	9.15277	8.94983	6.2385	6.10263	6.2704	A1+	snoRNA
AT4G39361	264.394	2.42829	0.21119	11.4983	1.35E-30	1.13E-28	8.5191	9.04407	8.83987	6.15418	6.43434	6.35243	A1-	snoRNA
AT4G39361	243.614	2.55267	0.24415	10.4553	1.39E-25	2.57E-23	8.4213	8.9496	8.74821	6.46427	6.10426	5.43798	A2-	snoRNA
AT4G39366	441.525	2.41368	0.16172	14.9247	2.28E-50	6.09E-48	9.44707	9.64239	9.55414	6.85977	7.01325	7.32984	A1+	snoRNA
AT4G39366	421.1	2.21134	0.18108	12.212	2.68E-34	2.61E-32	9.33927	9.53363	9.44409	6.78781	7.47011	7.31609	A1-	snoRNA
AT4G39366	370.445	2.65351	0.17901	14.8236	1.03E-49	8.43E-47	9.24135	9.4391	9.35236	6.16325	6.52272	6.80771	A2-	snoRNA
AT5G00750	367.933	3.48393	0.23705	14.6972	6.72E-49	1.71E-46	9.38094	9.18007	9.65497	5.74125	5.01973	6.22294	A1+	snoRNA
AT5G00750	332.766	4.10723	0.24103	17.0402	4.13E-65	1.25E-62	9.27314	9.07137	9.54491	4.63244	5.26298	5.15929	A1-	snoRNA
AT5G00750	307.158	4.33454	0.25819	16.7884	2.97E-63	3.64E-60	9.17523	8.97689	9.45318	4.44524	4.26223	4.64598	A2-	snoRNA
AT5G01785	303.015	2.16025	0.24555	8.79746	1.40E-18	6.34E-17	8.89226	9.27809	8.70031	6.33243	6.42035	7.18875	A1+	snoRNA
AT5G01785	273.404	2.53486	0.23879	10.6156	2.52E-26	1.72E-24	8.78453	9.16937	8.59039	6.05437	6.88921	5.88833	A1-	snoRNA
AT5G01785	255.952	2.40551	0.263	9.14628	5.89E-20	6.92E-18	8.68669	9.07488	8.49876	6.87521	5.95843	5.61688	A2-	snoRNA
AT5G02655	72.8369	3.50307	0.35986	9.73447	2.15E-22	1.31E-20	7.28781	7.15348	6.84285	3.68404	3.04536	3.08651	A1+	snoRNA
AT5G02655	69.4636	2.96262	0.32037	9.24761	2.30E-20	1.10E-18	7.18056	7.04538	6.73365	3.93374	3.54662	3.61329	A1-	snoRNA
AT5G02655	66.0596	2.89986	0.32918	8.80942	1.26E-18	1.31E-16	7.08318	6.95146	6.64268	4.69719	3.21224	2.97594	A2-	snoRNA
AT5G02665	66.0346	3.40189	0.37939	8.96663	3.06E-19	1.48E-17	6.83055	7.30998	6.66964	3.48023	3.04536	3.08651	A1+	snoRNA
AT5G02665	63.359	2.80109	0.33565	8.34528	7.10E-17	2.74E-15	6.72356	7.20179	6.56056	3.68946	3.54662	3.83605	A1-	snoRNA
AT5G02665	55.1257	3.5635	0.37773	9.43392	3.95E-21	5.01E-19	6.62645	7.10779	6.4697	0	2.57549	2.14856	A2-	snoRNA
AT5G07105	810.558	2.43218	0.15042	16.1694	8.29E-59	3.13E-56	10.4489	10.6032	10.1921	7.89519	8.00766	7.97047	A1+	snoRNA
AT5G07105	742.364	2.58166	0.18473	13.9756	2.20E-44	3.32E-42	10.341	10.4943	10.082	7.46876	8.12318	7.49831	A1-	snoRNA
AT5G07105	655.876	3.07404	0.24643	12.4744	1.03E-35	3.79E-33	10.243	10.3998	9.99019	7.23505	6.03319	7.18211	A2-	snoRNA
AT5G08515	95.5186	3.0859	0.34507	8.94273	3.80E-19	1.83E-17	7.38741	7.87195	6.96214	4.4141	4.16427	3.75389	A1+	snoRNA
AT5G08515	94.1232	2.48313	0.31349	7.92104	2.36E-15	8.07E-14	7.28012	7.76353	6.85286	4.56149	5.26298	4.35128	A1-	snoRNA
AT5G08515	85.9893	2.40012	0.35311	6.79714	1.07E-11	4.97E-10	7.1827	7.66933	6.76181	4.69719	4.94319	2.97594	A2-	snoRNA
AT5G09515	93.5292	3.19495	0.39532	8.08184	6.38E-16	2.32E-14	7.36803	8.03634	6.57922	3.48023	4.16427	3.88164	A1+	snoRNA
AT5G09515	91.1131	2.56516	0.35633	7.19879	6.08E-13	1.69E-11	7.26075	7.92787	6.47022	4.32498	4.7925	4.2772	A1-	snoRNA
AT5G09515	81.8009	2.70884	0.3643	7.43569	1.04E-13	6.78E-12	7.16333	7.83362	6.37942	3.21938	4.26223	3.76455	A2-	snoRNA
AT5G13225	84.7923	3.39106	0.40748	8.32206	8.65E-17	3.36E-15	7.31336	7.86524	6.47275	3.86258	3.41604	3.08651	A1+	snoRNA
AT5G13225	84.2001	2.62169	0.37674	6.95894	3.43E-12	8.99E-11	7.2061	7.75682	6.36384	3.93374	5.26298	3.48753	A1-	snoRNA
AT5G13225	75.9894	2.62972	0.36272	7.24993	4.17E-13	2.49E-11	7.10871	7.66262	6.27312	3.75176	4.26223	3.49845	A2-	snoRNA
AT5G46315	277.822	3.4462	0.22853	15.0798	2.20E-51	5.95E-49	9.02656	8.952	9.05589	5.19216	4.6545	5.95894	A1+	snoRNA, U6 small nucleolar RNA29
AT5G46315	253.967	3.74187	0.21833	17.1385	7.67E-66	2.37E-63	8.91881	8.84333	8.94591	4.99746	4.7925	5.07435	A1-	snoRNA, U6 small nucleolar RNA29
AT5G46315	232.739	4.3088	0.2559	16.8378	1.29E-63	1.68E-60	8.82094	8.74888	8.85424	4.69719	3.83072	4.08955	A2-	snoRNA, U6 small nucleolar RNA29
AT5G51174	242.412	3.50398	0.27473	12.7541	2.96E-37	4.13E-35	8.35384	9.12494	8.86627	5.19216	5.47681	4.76714	A1+	snoRNA, SNO30, Encodes a C/D box snoRNA (snoR30)
AT5G51174	228.855	3.18536	0.26026	12.2392	1.92E-34	1.90E-32	8.24622	9.01624	8.75631	5.37382	5.61718	5.11744	A1-	snoRNA, SNO30, Encodes a C/D box

AT5G51174	214.394	2.98148	0.26461	11.2676	1.90E-29	4.55E-27	8.14848	8.92177	8.66467	4.91162	5.61334	5.27695	A2-	snoRNA (snoR30) snoRNA, SNO30, Encodes a C/D box snoRNA (snoR30)
AT5G66564	137.262	3.42164	0.2666	12.8342	1.05E-37	1.50E-35	7.92709	8.10063	7.9419	4.29471	4.6545	4.39129	A1+	snoRNA
AT5G66564	122.995	3.80594	0.2905	13.1015	3.23E-39	3.95E-37	7.81959	7.99214	7.83217	3.68946	2.66509	4.02897	A1-	snoRNA
AT5G66564	115.62	3.78271	0.29609	12.7757	2.24E-37	9.17E-35	7.72196	7.89786	7.74072	3.75176	3.65249	3.34439	A2-	snoRNA
snRNAs														
AT1G04263	852.783	2.01466	0.21409	9.4102	4.95E-21	2.48E-19	10.2007	10.1458	10.8393	8.03145	8.63065	8.33456	A1-	snRNA
AT1G04263	929.475	1.94291	0.20044	9.69345	3.21E-22	1.93E-20	10.3086	10.2546	10.9494	8.37152	8.52597	8.69936	A1+	snRNA
AT1G04263	708.826	2.94988	0.29606	9.96381	2.19E-23	3.41E-21	10.1027	10.0512	10.7475	7.06632	5.95843	7.51565	A2-	snRNA
AT1G05853	878.299	2.00917	0.20756	9.67976	3.68E-22	2.19E-20	10.2349	10.197	10.8806	8.16574	8.36596	8.62372	A1+	snRNA
AT1G05853	806.752	2.08711	0.21196	9.84686	7.07E-23	3.98E-21	10.1271	10.0882	10.7705	7.96428	8.50898	8.12353	A1-	snRNA
AT1G05853	674.464	2.98836	0.29842	10.0138	1.33E-23	2.12E-21	10.0291	9.99361	10.6787	6.97393	5.79618	7.39329	A2-	snRNA
AT4G03995	923.191	2.76532	0.22058	12.5367	4.70E-36	1.82E-33	10.6388	10.8069	10.5808	7.52281	7.2271	8.19978	A2-	snRNA
AT4G04185	36.283	2.39521	0.4134	5.79399	6.87E-09	1.14E-07	5.93821	5.77686	6.30234	2.1331	3.04536	3.99899	A1+	snRNA
AT4G04185	36.418	1.98611	0.37501	5.29622	1.18E-07	1.88E-06	5.83207	5.67002	6.1936	2.52621	4.48353	3.93573	A1-	snRNA
AT4G04185	28.94	2.7657	0.40252	6.87099	6.38E-12	3.09E-10	5.73578	5.57728	6.10302	0	0	2.97594	A2-	snRNA
AT4G04615	288.71	3.46987	0.22642	15.3248	5.22E-53	1.57E-50	9.07068	9.02006	9.11501	5.25748	4.6545	5.98762	A1+	snRNA
AT4G04615	262.518	3.84336	0.21978	17.4871	1.80E-68	6.41E-66	8.96292	8.91138	9.00502	4.94268	4.48353	5.07435	A1-	snRNA
AT4G04615	241.35	4.36965	0.25471	17.1555	5.72E-66	1.05E-62	8.86505	8.81692	8.91334	4.44524	3.98933	4.08955	A2-	snRNA
AT4G07875	288.69	3.46985	0.22632	15.3316	4.71E-53	1.45E-50	9.07367	9.02006	9.11179	5.25748	4.6545	5.98762	A1+	snRNA
AT4G07875	262.499	3.84337	0.21972	17.4924	1.64E-68	6.00E-66	8.96592	8.91138	9.0018	4.94268	4.48353	5.07435	A1-	snRNA
AT4G07875	241.333	4.36982	0.25464	17.1605	5.24E-66	1.05E-62	8.86804	8.81692	8.91012	4.44524	3.98933	4.08955	A2-	snRNA
AT4G09135	206.411	3.77317	0.24862	15.1763	5.08E-52	1.48E-49	8.48556	8.75365	8.55843	4.4141	4.78679	4.83185	A1+	snRNA
AT4G09135	186.427	4.60673	0.29772	15.4736	5.23E-54	1.09E-51	8.37791	8.64501	8.44853	3.54983	4.48353	3.02706	A1-	snRNA
AT4G09135	176.42	4.10249	0.28228	14.5333	7.46E-48	5.31E-45	8.28014	8.5506	8.35694	4.69719	3.98933	3.1719	A2-	snRNA
AT5G04465	156.678	1.84011	0.27813	6.6161	3.69E-11	8.35E-10	7.90373	7.77501	8.20939	5.49267	5.62557	6.60256	A1+	snRNA
AT5G04465	131.598	2.60459	0.25022	10.4091	2.25E-25	1.44E-23	7.79623	7.66662	8.09958	5.19788	4.48353	5.19996	A1-	snRNA
AT5G04465	122.447	2.5559	0.26184	9.76143	1.65E-22	2.35E-20	7.69862	7.57245	8.00806	3.75176	5.09049	5.23372	A2-	snRNA
AT5G40395	74.3097	2.62659	0.35338	7.43274	1.06E-13	3.16E-12	7.02868	6.6666	7.3921	4.02145	3.04536	4.76714	A1+	snRNA U6 acat
AT5G40395	67.3272	3.0354	0.34357	8.83476	1.00E-18	4.34E-17	6.92157	6.55881	7.28258	3.39522	4.08983	3.48753	A1-	snRNA U6 acat
AT5G40395	61.6274	3.0887	0.35287	8.75297	2.08E-18	2.10E-16	6.82433	6.46519	7.19132	3.75176	2.10673	3.34439	A2-	snRNA U6 acat
tRNAs														
AT2G07754	44.5251	2.17149	0.32751	6.6303	3.35E-11	8.03E-10	6.36388	6.15143	6.28123	3.68946	3.54662	4.11654	A1-	tRNA-Ser (anticodon: GCT)
AT2G07754	48.1959	2.28374	0.33459	6.82554	8.76E-12	2.14E-10	6.47058	6.25885	6.39006	3.86258	3.95523	3.99899	A1+	tRNA-Ser (anticodon: GCT)
AT2G07754	38.6597	2.6722	0.35346	7.56009	4.03E-14	2.80E-12	6.26704	6.05814	6.19058	2.36651	2.57549	3.34439	A2-	tRNA-Ser (anticodon: GCT)
AT2G07759	43.987	2.71814	0.36838	7.37864	1.60E-13	4.68E-12	6.37674	6.21728	6.35779	3.48023	3.04536	3.45845	A1+	tRNA-Ser (anticodon: GCT)
AT2G07759	41.4221	2.52728	0.34887	7.24422	4.35E-13	1.22E-11	6.27012	6.1099	6.24899	3.22203	3.54662	3.48753	A1-	tRNA-Ser (anticodon: GCT)
AT2G07759	35.4529	3.10292	0.38244	8.11357	4.92E-16	4.19E-14	6.17336	6.01666	6.15837	0	1.40794	2.74912	A2-	tRNA-Ser (anticodon: GCT)
