# Supplementary material for: Induction of epigenetic variation in Arabidopsis by over-expression of DNA METHYLTRANSFERASE1 (MET1)
Source: PLoS One. 2018 Feb 21;13(2):e0192170. doi: 10.1371/journal.pone.0192170 (PMC5821449; doi:10.1371/journal.pone.0192170)
Supplement: S7 Table — (PDF) [file pone.0192170.s012.pdf]

S7 Table: List of coding genes at least log2-fold increases (negative log2-fold change) or decreases (positive log2-fold change) of 2.5 in at least one of the four lines A1+, A1, A2+ or A2-

	baseMean	log2Fold(	lfcSE	stat	pvalue	padj	1	2	3	4	5	6		
AT1G01060	421.8555	2.94793	0.43508	6.77561	1.24E-11	2.96E-10	10.0701	9.27176	9.31876	6.81708	5.76042	5.26573	A1+	LHY1, LATE ELONGATED HYPOCOTYL 1, LHY encodes a myb-related putative transcription factor involved in circadian rhythm along with another myb transcription factor CCA1
AT1G01060	394.5568	3.18476	0.25068	12.7046	5.57E-37	6.25E-35	9.96223	9.16304	9.20874	6.22475	6.24407	5.91301	A1-	
AT1G01060	373.6646	2.96094	0.25863	11.4487	2.39E-30	6.13E-28	9.86425	9.06855	9.11703	6.59409	6.03319	5.94637	A2-	
AT1G01680	220.7745	4.44029	0.30587	14.5171	9.44E-48	2.29E-45	8.44683	8.7609	8.9731	4.6268	1.77337	4.10751	A1+	plant U-box 54 (PUB54); FUNCTIONS IN: ubiquitin-protein ligase activity; INVOLVED IN: response to stress, protein ubiquitination; LOCATED IN: ubiquitin ligase complex
AT1G02380	136.0454	1.02207	0.22449	4.55284	5.29E-06	5.53E-05	7.58079	7.43763	7.57634	6.90123	6.15253	6.24686	A1+	Transmembrane protein
AT1G02380	129.1293	0.83914	0.20278	4.13808	3.50E-05	0.0003628	7.4734	7.32938	7.46674	6.57018	6.02485	6.71708	A1-	
AT1G02380	89.27054	2.76855	0.27245	10.1616	2.94E-24	4.99E-22	7.3759	7.23533	7.37541	4.13977	4.59395	4.08955	A2-	
AT1G02520	59.71548	2.7839	0.36051	7.72214	1.14E-14	3.72E-13	6.39601	6.94647	6.89626	3.86258	2.54524	4.10751	A1+	ABCB11 ATP-binding cassette B11, P-glycoprotein 11 (PGP11); FUNCTIONS IN: ATPase activity, coupled to transmembrane movement of substances; Encodes an ATP-binding cassette (ABC) transporter. Expressed in the vascular tissue of primary stem
AT1G03445	16.94786	2.86819	0.55767	5.14313	2.70E-07	3.55E-06	4.32111	4.63945	5.83987	0	0	1.80658	A1+	BSU1, BRI1 SUPPRESSOR 1 (BSU1); FUNCTIONS IN: protein binding, protein serine/threonine phosphatase activity; INVOLVED IN: brassinosteroid mediated signaling pathway, regulation of protein localization, encodes a serine–threonine protein phosphatase with an N-terminal Kelch-repeat domain, which is nuclear localized and expressed preferentially in elongating cells. Genetic evidence suggest that this gene plays a redundant role (along with other members of the same gene family) in modulating growth in response to brassinosteroid
AT1G06137	17.77101	3.37315	0.5391	6.25699	3.92E-10	7.71E-09	5.38439	5.07139	5.02412	1.42937	0	0	A1+	Transmembrane protein
AT1G08630	321.5537	-3.626	0.25276	-14.345	1.14E-46	2.40E-44	5.84317	5.43997	4.91006	8.7965	9.56605	9.23314	A1+	THA1 threonine aldolase 1
AT1G08630	510.0416	-4.2961	0.25841	-16.625	4.57E-62	1.23E-59	5.73715	5.33367	4.80366	9.63017	10.441	9.57387	A1-	
AT1G08630	318.8392	-3.6567	0.26318	-13.894	6.84E-44	4.20E-41	5.64098	5.24144	4.71519	8.61379	9.48915	9.44011	A2-	
AT1G08860	18.31202	2.568	0.51272	5.00861	5.48E-07	6.85E-06	4.54471	4.99965	5.62167	2.1331	1.77337	1.80658	A1+	BON3; BONZAI 3 (BON3); Encodes a copine-like protein, which is a member of a newly identified class of calcium-dependent, phospholipid binding proteins that are present in a wide range of organisms. Overexpression of this gene suppresses bon1-1 phenotypes. Double mutant analyses with bon1-1 suggest that BON1 and BON3 have overlapping functions in maintaining cellular homeostasis and inhibiting cell death.
AT1G09932	148.0076	3.85374	0.34746	11.0911	1.39E-28	1.23E-26	8.05681	7.83464	8.46809	4.89656	2.54524	3.28443	A1+	Phosphoglycerate mutase family protein; CONTAINS InterPro DOMAIN/s: Histidine phosphatase superfamily, clade-1
AT1G09950	59.6729	4.10069	0.46657	8.789	1.51E-18	6.80E-17	6.6255	6.31904	7.44426	2.1331	1.77337	1.80658	A1+	RESPONSE TO ABA AND SALT 1
AT1G09950	69.42742	1.22776	0.35079	3.50001	4.65E-04	4.50E-03	6.4217	6.11822	7.24344	5.66912	5.51243	4.27156	A2-	
AT1G10070	2134.618	-4.7499	0.14542	-32.664	5.05E-234	6.48E-230	7.40654	7.21285	6.91855	11.8753	12.1655	11.9745	A1+	Encodes a chloroplast branched-chain amino acid aminotransferase. Complements the yeast leu/iso-leu/val auxotrophy mutant. Involved in cell wall development.
AT1G10070	2333.689	-4.9069	0.1795	-27.337	1.56E-164	8.00E-161	7.29923	7.10471	6.8093	12.1145	12.4206	11.8471	A1-	
AT1G10070	1321.858	-4.0565	0.21832	-18.58	4.66E-77	5.14E-73	7.2018	7.01077	6.71828	10.7394	11.4893	11.5369	A2-	
AT1G11810	5.332416	-2.5609	0.58929	-4.3458	1.39E-05	0.0001343	0.64015	0	0	2.9585	3.41604	3.99899	A1+	F-box associated ubiquitination effector family protein;
AT1G11810	5.303359	-2.2426	0.51475	-4.3567	1.32E-05	0.0001489	0.60238	0	0	4.32498	0	3.72897	A1-	
AT1G11810	5.918096	-2.1769	0.45419	-4.7931	1.64E-06	3.15E-05	0.56965	0	0	3.21938	4.38148	2.97594	A2-	
AT1G13310	49.92424	3.20513	0.39407	8.13351	4.17E-16	1.54E-14	6.35722	6.63538	6.66964	3.68404	0	3.08651	A1+	BRO1 Endosomal targeting BRO1-like domain-containing protein
AT1G15040	91.73669	-1.8755	0.28323	-6.6216	3.55E-11	8.06E-10	5.45955	5.40337	4.68577	7.03764	7.58543	6.91779	A1+	GAT1_2.1, Class I glutamine amidotransferase-like superfamily protein; FUNCTIONS IN: hydrolase activity; INVOLVED IN: glutamine metabolic process; overlaps with NAT antisense AT1G15047
AT1G15040	137.1009	-2.5379	0.2853	-8.8955	5.82E-19	2.55E-17	5.35413	5.29714	4.58001	7.66764	8.46604	7.38825	A1-	
AT1G15040	138.0198	-2.3794	0.36375	-6.5415	6.09E-11	2.52E-09	5.25854	5.20497	4.49211	6.16325	8.42328	8.32864	A2-	
AT1G15380	63.70528	-2.9384	0.35519	-8.2725	1.31E-16	5.04E-15	4.36087	3.43123	2.84483	6.68085	7.16684	6.69242	A1+	GLY I 4 Lactoylglutathione lyase / glyoxalase I family protein
AT1G15380	63.09437	-2.8676	0.36267	-7.9069	2.64E-15	9.00E-14	4.25834	3.33277	2.75049	6.12987	7.07217	7.17021	A1-	
AT1G15380	113.1296	-3.8563	0.29356	-13.136	2.04E-39	9.80E-37	4.16556	3.24779	2.67265	7.5866	7.98867	7.67766	A2-	
AT1G15580	35.15185	3.37972	0.46265	7.30514	2.77E-13	7.86E-12	5.6156	6.19604	6.35779	2.1331	1.77337	2.24707	A1+	IAA5 indole-3-acetic acid inducible 5; ATAUX2-27, AUXIN-INDUCIBLE 2-27, AUX2-27; FUNCTIONS IN: sequence-specific DNA binding transcription factor activity; INVOLVED IN: response to auxin stimulus, response to cyclopentenone, response to brassinosteroid stimulus;
AT1G15580	34.38586	2.55777	0.39227	6.5205	7.01E-11	1.61E-09	5.50991	6.08868	6.24899	3.02518	2.66509	3.02706	A1-	
AT1G15580	34.26796	2.08999	0.36827	5.67521	1.39E-08	3.95E-07	5.41407	5.99546	6.15837	4.13977	2.57549	3.34439	A2-	
AT1G17380	58.48942	2.60503	0.34123	7.63428	2.27E-14	7.17E-13	6.50648	6.83409	6.77115	4.29471	2.54524	4.10751	A1+	JAZ5 jasmonate-zim-domain protein 5
AT1G17960	63.33199	2.77221	0.37512	7.39026	1.47E-13	4.31E-12	6.44307	6.86133	7.16226	4.4141	1.77337	3.88164	A1+	Threonyl-tRNA synthetase
AT1G18050	5.581102	-2.5117	0.58269	-4.3106	1.63E-05	0.000155	0	0.62641	0.69459	3.48023	3.41604	3.75389	A1+	SWAP/surp RNA-binding domain protein, SWAP (Suppressor-of-White-APricot)/surp RNA-binding domain-containing protein; FUNCTIONS IN: RNA binding; INVOLVED IN: RNA processing
AT1G20310	18.86933	2.58322	0.51255	5.0399	4.66E-07	5.88E-06	5.15487	5.13974	5.20432	3.24283	0	0	A1+	syringolide-induced protein
AT1G20520	31.40585	3.41383	0.49896	6.84191	7.81E-12	1.93E-10	5.74141	5.43997	6.46267	2.60404	0	1.16932	A1+	DUF241 domain protein, putative (DUF241), overlaps with NAT AT1G20515

AT1G21400	3048.344	-2.512	0.1313	-19.131	1.38E-81	1.36E-78	9.90174	9.75289	9.74818	12.1028	12.5435	12.3576	A1+	Thiamin diphosphate-binding fold (THDP-binding) superfamily protein; FUNCTIONS IN: oxidoreductase activity, acting on the aldehyde or oxo group of donors, disulfide as acceptor, 3-methyl-2-oxobutanoate dehydrogenase (2-methylpropanoyl-transferring) activity
AT1G21400	3360.147	-2.7708	0.14543	-19.053	6.23E-81	3.41E-78	9.79389	9.64412	9.63811	12.4596	12.7764	12.2974	A1-	
AT1G21400	2514.663	-2.3686	0.1731	-13.684	1.26E-42	7.34E-40	9.69593	9.54958	9.54636	11.6743	12.0791	12.3366	A2-	
AT1G21850	34.03105	3.42352	0.48154	7.10956	1.16E-12	3.15E-11	5.69551	5.74796	6.55095	1.42937	1.77337	2.24707	A1+	SKS8, SKU5 similar 8 (sks8); FUNCTIONS IN: oxidoreductase activity, copper ion binding; INVOLVED IN: oxidation reduction
AT1G22240	18.83979	3.39933	0.54059	6.2882	3.21E-10	6.41E-09	4.7974	5.42179	5.44861	1.42937	0	0	A1+	PUM8, pumilio 8, Encodes a member of the Arabidopsis Pumilio (APUM) proteins containing PUF domain (eight repeats of approximately 36 amino acids each). PUF proteins regulate both mRNA stability and translation through sequence-specific binding to the 3' UTR of target mRNA transcripts.
AT1G24260	10.92815	-2.7081	0.56377	-4.8036	1.56E-06	1.80E-05	1.08201	0.62641	1.16147	2.60404	5.12347	4.62832	A1+	AGAMOUS-LIKE 9, AGL9, SEP3, SEPALLATA3 Member of the MADs box transcription factor family. SEP3 is redundant with SEP1 and 2. Flowers of SEP1/2/3 triple mutants show a conversion of petals and stamens to sepals.SEP3 forms heterotetrameric complexes with other MADS box family members and binds to the CArG box motif.
AT1G24260	10.70713	-2.9278	0.483	-6.0616	1.35E-09	2.74E-08	1.02605	0.58899	1.10158	4.82646	3.54662	4.61457	A1-	
AT1G27570	25.34111	-3.4677	0.56603	-6.1263	8.99E-10	1.69E-08	1.08201	1.06174	0	2.60404	6.33648	6.04333	A1+	Phosphatidylinositol 3- and 4-kinase family protein;
AT1G30190	80.6578	3.33862	0.42556	7.84518	4.32E-15	1.46E-13	6.95266	6.9208	7.7397	4.52436	1.77337	2.24707	A1+	Cotton fiber protein
AT1G30370	62.9448	2.56771	0.38396	6.6875	2.27E-11	5.27E-10	6.47964	6.82027	7.10509	4.52436	0	4.30275	A1+	DLAH alpha/beta-Hydrolases superfamily protein Encodes a mitochondria-localized class III phospholipase A1 that plays a role in seed viability.
AT1G31095	4.957119	-2.6252	0.59218	-4.4331	9.29E-06	9.27E-05	0	0	0	2.60404	3.41604	3.99899	A1+	Lactate/malate dehydrogenase, NAD-binding domain protein
AT1G32080	393.6622	2.62558	0.2168	12.1109	9.25E-34	8.80E-32	9.09593	9.43619	9.70599	6.64071	6.13862	6.93752	A1-	LrgB membrane protein, putative; Encodes a plant LrgAB/CidAB protein localized to the chloroplast envelope that is involved in chloroplast development, carbon partitioning and leaf senescence. The gene may have evolved from gene fusion of bacterial lrgA and lrgB.
AT1G32910	13.44612	2.83823	0.56592	5.01526	5.30E-07	6.64E-06	4.50977	4.06764	5.40716	1.42937	0	0	A1+	HXXXD-type acyl-transferase family protein; FUNCTIONS IN: transferase activity, transferring acyl groups other than amino-acyl groups, transferase activity
AT1G33720	197.6012	2.52336	0.33284	7.58142	3.42E-14	1.06E-12	8.19245	8.32022	8.73422	6.53041	4.6545	5.16887	A1+	CYP76C6 cytochrome P450, family 76, subfamily C, polypeptide 6" (CYP76C6); FUNCTIONS IN: electron carrier activity, monooxygenase activity, iron ion binding, oxygen binding, heme binding
AT1G33760	146.8185	4.26842	0.365	11.6944	1.36E-31	1.44E-29	8.0324	7.79634	8.53456	4.4141	1.77337	2.85705	A1+	ETHYLENE RESPONSE FACTOR022, ERF022, encodes a member of the DREB subfamily A-4 of ERF/AP2 transcription factor family. The protein contains one AP2 domain. There are 17 members in this subfamily including TINY.
AT1G36060	26.95504	-1.5042	0.37941	-3.9645	7.35E-05	6.06E-04	4.10402	3.10637	3.92825	5.31997	5.47681	5.39982	A1+	RAP2.4/TRANSLUCENT GREEN, TG Integrase-type DNA-binding superfamily protein, encodes a member of the DREB subfamily A-6 of ERF/AP2 transcription factor family. The protein contains one AP2 domain. There are 8 members in this subfamily including RAP2.4.Overexpression results in increased drought tolerance and vitrified leaves. Binds to DRE/GCC promoter elements and activates expression of aquaporin genes AtTIP1;1, AtTIP2;3, and AtPIP2;2
AT1G36060	50.89952	-2.6147	0.31003	-8.4336	3.35E-17	1.32E-15	4.00256	3.01054	3.82555	6.33521	6.67963	6.48996	A1-	
AT1G42980	14.40198	2.52785	0.54178	4.66585	3.07E-06	3.38E-05	4.23815	4.70107	5.27515	2.1331	1.77337	0	A1+	Actin-binding FH2 (formin homology 2) family protein; FUNCTIONS IN: actin binding
AT1G43910	394.4243	3.04111	0.54664	5.56329	2.65E-08	4.03E-07	9.15931	9.07345	10.1821	4.89656	3.04536	5.95894	A1+	P-loop containing nucleoside triphosphate hydrolases superfamily protein; FUNCTIONS IN: nucleoside-triphosphatase activity, ATPase activity, nucleotide binding,
AT1G44130	20.55873	2.6855	0.4851	5.536	3.09E-08	4.68E-07	5.08538	5.2261	5.5281	2.60404	0	2.24707	A1+	Eukaryotic aspartyl protease family protein; FUNCTIONS IN: aspartic-type endopeptidase activity; INVOLVED IN: proteolysis
AT1G47590	10.52504	2.9987	0.57241	5.23871	1.62E-07	2.20E-06	4.32111	4.43706	4.61472	0	0	0	A1+	PUP20, Member of a family of proteins related to PUP1, a purine transporter. May be involved in the transport of purine and purine derivatives such as cytokinins, across the plasma membrane.
AT1G48285	17.55682	-3.282	0.58805	-5.5812	2.39E-08	3.66E-07	0	0	0	1.42937	5.31093	6.04333	A1+	GRF zinc finger protein
AT1G51850	116.0955	2.61073	0.57228	4.56197	5.07E-06	5.31E-05	7.65462	7.43763	8.19729	4.6268	0	3.08651	A1+	Leucine-rich repeat protein kinase family protein; FUNCTIONS IN: kinase activity; INVOLVED IN: protein amino acid phosphorylation
AT1G55390	13.56513	2.62143	0.53316	4.91679	8.80E-07	1.06E-05	4.57882	4.60763	4.99644	1.42937	0	1.80658	A1+	Cysteine/Histidine-rich C1 domain family protein; FUNCTIONS IN: zinc ion binding
AT1G57650	24.79155	3.03994	0.4896	6.20905	5.33E-10	1.03E-08	5.42246	5.28764	5.9594	2.60404	0	1.80658	A1+	ATP binding protein; FUNCTIONS IN: ATP binding; INVOLVED IN: apoptosis, defense response
AT1G59920	83.40661	-4.0719	0.3705	-10.99	4.26E-28	3.62E-26	3.04621	2.41948	3.17624	7.17921	7.90949	6.62098	A1+	Q9XID9 MADS-box family protein
AT1G59920	264.3615	-3.3589	0.16818	-19.972	9.67E-89	6.43E-85	3.72108	3.06207	3.85318	8.74778	9.33197	8.91237	A2+	
AT1G59930	171.4759	-4.6003	0.37818	-12.164	4.82E-34	5.66E-32	3.66997	2.26486	3.6007	8.39348	8.95651	7.42729	A1+	MADS-box family protein
AT1G59930	481.386	-3.6634	0.1678	-21.832	1.15E-105	1.02E-101	4.37067	2.89389	4.29467	9.60687	10.2235	9.76696	A2+	
AT1G60590	76.76581	1.00523	0.24956	4.02801	5.63E-05	4.74E-04	6.5415	6.87476	6.67837	5.74125	5.76042	5.5225	A1+	Pectin lyase-like superfamily protein; FUNCTIONS IN: polygalacturonase activity; INVOLVED IN: carbohydrate metabolic process
AT1G60590	52.98193	2.65615	0.33015	8.0452	8.61E-16	3.06E-14	6.43473	6.76682	6.56929	3.68946	2.66509	3.83605	A1-	
AT1G61800	69.44008	2.68584	0.42894	6.26156	3.81E-10	7.53E-09	7.15277	6.11913	7.40794	3.68404	1.77337	4.62832	A1+	GPT2, ATGPT2, ARABIDOPSIS GLUCOSE-6-PHOSPHATE/PHOSPHATE TRANSLOCATOR 2, glucose-6-phosphate/phosphate translocator 2 (GPT2); FUNCTIONS IN: antiporter activity, glucose-6-phosphate transmembrane
AT1G62580	18.54191	-2.134	0.46119	-4.6272	3.71E-06	4.01E-05	1.6933	2.91163	2.96397	5.49267	4.6545	4.83185	A1+	NOGC1 nitric oxide-dependent guanylate cyclase 1, Flavin-binding monooxygenase family protein; FUNCTIONS IN:

AT1G62580	25.50751	-2.6365	0.40154	-6.566	5.17E-11	1.22E-09	1.61959	2.81769	2.86837	5.10115	5.61718	5.86323	A1-	NADP or NADPH binding, monooxygenase activity, FAD binding, flavin-containing monooxygenase activity;
AT1G65390	74.17779	3.99816	0.39531	10.1141	4.78E-24	3.30E-22	7.09501	7.07421	7.32691	3.86258	0	1.80658	A1+	ATPP2-A5, PHLOEM PROTEIN 2 A5, PP2-A5, FUNCTIONS IN: carbohydrate binding; INVOLVED IN: signal transduction, defense response, innate immune response;
AT1G65481	16.23705	3.50155	0.55653	6.29172	3.14E-10	6.29E-09	5.01236	4.81692	5.32051	0	0	0	A1+	Transmembrane protein
AT1G65610	24.46579	2.80077	0.47273	5.92461	3.13E-09	5.45E-08	5.32532	5.43997	5.82421	2.1331	0	2.85705	A1+	KOR2, KORRIGAN 2, UNCTIONS IN: hydrolase activity, hydrolyzing O-glycosyl compounds, catalytic activity; INVOLVED IN: carbohydrate metabolic process
AT1G67856	52.3631	2.57149	0.3953	6.50517	7.76E-11	1.69E-09	6.32743	6.28926	6.96214	4.4141	1.77337	3.61372	A1+	RING/U-box superfamily protein; FUNCTIONS IN: zinc ion binding
AT1G68050	102.6629	-3.3466	0.31574	-10.599	3.01E-26	2.30E-24	3.46634	4.15829	4.33422	6.92153	7.83504	7.80306	A1+	FLAVIN-BINDING, KELCH REPEAT, F BOX 1", ADO3, FKF1, a flavin-binding kelch repeat F box protein, is clock-controlled, regulates transition to flowering. Forms a complex with GI on the CO promoter to regulate CO expression.
AT1G68050	100.0817	-3.4548	0.26759	-12.91	3.93E-38	4.59E-36	3.36848	4.05571	4.22969	7.8045	7.38044	7.4144	A1-	
AT1G68050	81.59631	-3.188	0.25591	-12.458	1.27E-35	4.59E-33	3.2802	3.96693	4.14287	7.23505	7.2271	7.24877	A2-	
AT1G68765	65.97922	3.59263	0.38322	9.3748	6.93E-21	3.73E-19	7.16966	6.9401	6.81142	3.86258	0	2.85705	A1+	IDA, INFLORESCENCE DEFICIENT IN ABSCISSION, Putative membrane lipoprotein , encodes a small protein of 77 amino acids. Loss of function mutations are defective in the process of ethylene independent floral organ abscission. Although the mutants have a normal appearing abscission zone, the floral organs do not abscisce. The peptide appears to be secreted and may function as a ligand. Arabidopsis 35S:IDA lines constitutively overexpressing IDA exhibit earlier abscission of floral organs, showing that the abscission zones are responsive to IDA soon after the opening of the flowers. In addition, ectopic abscission was observed at the bases of the pedicel, branches of the inflorescence, and cauline leaves. The silique valves also dehisced prematurely
AT1G69930	43.88752	2.52091	0.44541	5.65971	1.52E-08	2.39E-07	6.05188	6.54586	6.3136	4.52436	0	2.58406	A1+	GSTU11glutathione S-transferase TAU 11 (GSTU11), Encodes glutathione transferase belonging to the tau class of GSTs. Naming convention according to Wagner et al. (2002)
AT1G73120	88.25249	-2.5154	0.55724	-4.514	6.36E-06	6.56E-05	3.46634	3.27792	4.04094	5.25748	8.30107	7.17635	A1+	F-box/RNI superfamily protein
AT1G73120	27.70764	-1.7783	0.38043	-4.6745	2.95E-06	3.73E-05	3.36848	3.18064	3.93769	6.05437	5.26298	5.02993	A1-	
AT1G73120	55.71551	-2.5395	0.37756	-6.7261	1.74E-11	7.85E-10	3.2802	3.09673	3.852	7.55506	5.79618	5.99896	A2-	
AT1G73340	18.35498	3.33204	0.54231	6.14417	8.04E-10	1.52E-08	5.01236	4.89808	5.60344	0	1.77337	0	A1+	Cytochrome P450 superfamily protein; FUNCTIONS IN: electron carrier activity, monooxygenase activity, iron ion binding, oxygen binding, heme binding
AT1G73340	19.63109	1.36109	0.39343	3.4595	5.41E-04	5.15E-03	4.81317	4.70184	5.40583	2.36651	3.83072	2.97594	A2-	
AT1G73600	125.0519	2.53995	0.26682	9.51938	1.74E-21	8.97E-20	7.44794	8.05912	7.76536	5.28853	4.7925	4.93675	A1-	S-adenosyl-L-methionine-dependent methyltransferases superfamily protein; FUNCTIONS IN: methyltransferase activity, phosphoethanolamine N-methyltransferase activity;
AT1G74710	615.4413	3.42598	0.2378	14.407	4.68E-47	1.03E-44	9.90258	10.1038	10.409	6.85977	5.62557	6.85691	A1+	SID2, EDS16, ENHANCED DISEASE SUSCEPTIBILITY TO ERYSIPHE ORONTII 16, ICS1, ATICS1, SALICYLIC ACID INDUCTION DEFICIENT 2, ARABIDOPSIS ISOCHORISMATE SYNTHASE 1, SID2, ISOCHORISMATE SYNTHASE 1, Encodes a protein with isochorismate synthase activity. Mutants fail to accumulate salicylic acid. Its function may be redundant with that of ICS2 (AT1G18870)
AT1G76410	117.5158	-2.387	0.2417	-9.876	5.29E-23	3.36E-21	5.34528	4.94976	5.20432	7.33863	7.99404	7.53808	A1+	ATL8, RING/U-box superfamily protein
AT1G76410	133.9113	-2.6803	0.21742	-12.328	6.42E-35	6.57E-33	5.24007	4.84451	5.09722	7.84986	8.12318	7.63805	A1-	
AT1G76410	106.8208	-2.4372	0.23162	-10.522	6.81E-26	1.31E-23	5.14468	4.75326	5.00812	7.23505	7.43439	7.83781	A2-	
AT1G76430	16.97418	2.59803	0.5259	4.94015	7.81E-07	9.51E-06	5.28456	4.73091	5.02412	2.9585	0	0	A1+	PHT1;9 phosphate transporter 1;9 Encodes Pht1;9, a member of the Pht1 family of phosphate transporters which include: Pht1;1/At5g43350, Pht1;2/At5g43370, Pht1;3/At5g43360, Pht1;4/At2g38940, Pht1;5/At2g32830, Pht1;6/At5g43340, Pht1;7/At3g54700, Pht1;8/At1g20860, Pht1;9/At1g76430.
AT1G80160	164.053	-4.0561	0.23968	-16.923	3.03E-64	1.41E-61	4.15013	4.20157	3.9857	8.31127	8.52597	7.97763	A1+	GLY I 7 Lactoylglutathione lyase / glyoxalase I family protein
AT1G80160	123.5302	-3.6012	0.26442	-13.619	3.08E-42	4.44E-40	4.04846	4.0988	3.88271	7.86467	8.22973	7.37054	A1-	
AT1G80160	132.849	-3.8984	0.21821	-17.865	2.20E-71	9.73E-68	3.95651	4.00984	3.79726	7.84317	8.07966	8.00783	A2-	
AT2G02680	28.5738	3.2148	0.48246	6.66332	2.68E-11	6.17E-10	5.59907	5.59407	6.12195	2.9585	0	1.16932	A1+	Cysteine/Histidine-rich C1 domain family protein; FUNCTIONS IN: zinc ion binding; INVOLVED IN: intracellular signaling pathway;
AT2G04040	238.7008	3.28529	0.27297	12.0355	2.31E-33	2.65E-31	8.2728	8.84177	9.10533	5.5973	5.01973	5.26573	A1+	DTX1 detoxification 1, ATDTX1, TX1, AtDTX1 (At2g04040) has been identified as a detoxifying efflux carrier for plant-derived antibiotics and other toxic compounds, including CD2 . Mistakenly referred to as At2g04070 in PMID:11739388
AT2G04070	179.6805	3.23849	0.54066	5.98988	2.10E-09	3.78E-08	8.24916	8.15117	8.83144	4.52436	0	3.99899	A1+	MATE efflux family protein; FUNCTIONS IN: antiporter activity, drug transmembrane transporter activity, transporter activity;
AT2G06425	8.363999	-3.4189	0.57834	-5.9115	3.39E-09	5.85E-08	0	0	0	4.52436	4.16427	3.61372	A1+	PttA/En/Spm family plant transposase
AT2G06425	8.205108	-3.0674	0.51169	-5.9947	2.04E-09	4.08E-08	0	0	0	4.88574	2.66509	4.02897	A1-	
AT2G06845	25.75645	-5.0463	0.52298	-9.6491	4.96E-22	2.91E-20	0	0	0	5.78621	5.99732	5.26573	A1+	Beta-galactosidase related protein
AT2G06845	28.99523	-4.9705	0.45744	-10.866	1.67E-27	1.21E-25	0	0	0	5.63796	5.90133	6.07481	A1-	
AT2G06904	38.91945	-5.2669	0.52655	-10.003	1.48E-23	9.96E-22	0	0	0	6.85977	6.42035	5.11789	A1+	Nucleic acid / zinc ion binding protein
AT2G06904	28.65092	-4.4571	0.48087	-9.2687	1.88E-20	9.17E-19	0	0	0	4.56149	6.60241	5.75822	A1-	
AT2G06904	27.5272	-1.4492	0.1738	-8.3382	7.54E-17	2.23E-14	0	0	0	5.84377	6.18932	5.23869	A2+	
AT2G07240	6.437787	-2.203	0.59213	-3.7204	1.99E-04	1.46E-03	0.64015	0	0.69459	4.89656	1.77337	2.85705	A1+	Cysteine-type peptidase

AT2G07240	19.20523	-3.7876	0.47771	-7.9286	2.22E-15	7.62E-14	0.60238	0	0.65347	4.48688	6.02485	4.88781	A1-	
AT2G09840	16.88487	-3.2835	0.58785	-5.5855	2.33E-08	3.58E-07	0	0	0	6.39182	3.71068	2.85705	A1+	Nucleic acid/zinc ion-binding protein
AT2G11778	758.0734	-9.4613	0.41229	-22.949	1.53E-116	3.01E-113	0	0	0	10.8507	10.5015	10.2935	A1+	Transmembrane protein
AT2G11778	606.4654	-8.9811	0.36327	-24.723	6.08E-135	1.12E-131	0	0	0	10.2939	10.1535	10.2847	A1-	
AT2G11778	150.8647	-2.9111	0.17928	-16.237	2.75E-59	4.87E-56	0	0	0	8.30373	8.63833	7.59789	A2+	
AT2G13810	48.31647	4.35329	0.52059	8.36222	6.16E-17	2.43E-15	5.71097	6.51188	7.20522	1.42937	0	0	A1+	EDTS5,eds two suppressor 5, AGD2-like defense response protein 1 (ALD1); FUNCTIONS IN: transferase activity, transferring nitrogenous groups, pyridoxal phosphate binding, transaminase activity, catalytic activity; INVOLVED IN: asparagine catabolic process, biosynthetic process, glutamate catabolic process to oxaloacetate, aspartate transamidation;
AT2G15420	7.571765	-2.6811	0.51216	-5.235	1.65E-07	2.57E-06	0	0	0.65347	3.02518	4.48353	4.11654	A1-	myosin heavy chain-like protein
AT2G15890	5072.829	-2.5591	0.14263	-17.942	5.56E-72	3.40E-69	10.4644	10.3563	10.6396	12.8034	13.2725	13.1485	A1+	maternal effect embryo arrest 14 (MEE14); FUNCTIONS IN: molecular_function unknown; INVOLVED IN: defense response to fungus, embryo development ending in seed dormancy;
AT2G15890	4038.251	-2.2921	0.14231	-16.106	2.31E-58	5.55E-56	10.3565	10.2475	10.5295	12.9035	12.7295	12.4962	A1-	
AT2G15890	3242.369	-2.0068	0.15549	-12.906	4.14E-38	1.87E-35	10.2585	10.1529	10.4377	12.2432	12.1699	12.6106	A2-	
AT2G17690	42.0385	-3.6086	0.42963	-8.3993	4.49E-17	1.80E-15	1.92313	2.80343	2.03285	6.88065	6.33648	5.48276	A1+	SDC, SUPPRESSOR OF DRM1 DRM2 CMT3, Encodes an F-box domain containing protein that is regulated by non-CG DNA methylation. In drm1 drm2 cmt3 triple mutant background SDC expression is no longer suppressed and plants display abnormal development including curled leaves and reduced stature. A maternally expressed imprinted gene.
AT2G17690	80.35864	-1.8945	0.18027	-10.51	7.81E-26	3.71E-23	2.50778	3.4736	2.62616	7.16609	7.78934	6.64882	A2+	
AT2G19800	1233.924	-1.7839	0.44572	-4.0023	6.27E-05	0.0005236	8.13914	9.07345	9.07917	10.2728	11.6892	10.559	A1+	MIOX2, MYO-INOSITOL OXYGENASE 2
AT2G19800	2490.611	-3.2642	0.22669	-14.399	5.26E-47	8.94E-45	8.03157	8.96476	8.96919	12.2104	12.3662	11.841	A1-	
AT2G19850	33.81868	-4.5668	0.41707	-10.95	6.66E-28	4.91E-26	1.02605	1.32917	0	6.0283	6.24407	5.96112	A1-	Transcription repressor
AT2G20350	17.89711	2.6522	0.5294	5.00982	5.45E-07	6.82E-06	4.70775	5.68836	4.72002	2.60404	0	1.16932	A1+	Integrase-type DNA-binding superfamily protein; FUNCTIONS IN: DNA binding, sequence-specific DNA binding transcription factor activity; INVOLVED IN: regulation of transcription, DNA-dependent. encodes a member of the ERF (ethylene response factor) subfamily B-6 of ERF/AP2 transcription factor family. The protein contains one AP2 domain. There are 12 members in this subfamily including RAP2.11
AT2G20720	266.7597	4.50557	0.28453	15.8352	1.78E-56	6.01E-54	8.77577	9.05882	9.18413	3.68404	3.41604	4.89377	A1+	Pentatricopeptide repeat (PPR) superfamily protein
AT2G21660	2116.119	-2.7843	0.23959	-11.621	3.22E-31	3.28E-29	8.329	9.46506	8.86627	11.5628	11.9425	12.0521	A1+	GLYCINE-RICH RNA-BINDING PROTEIN 7, cold, circadian rhythm, and rna binding 2" (CCR2); FUNCTIONS IN: double-stranded DNA binding, RNA binding, single-stranded DNA binding,Encodes a small glycine-rich RNA binding protein that is part of a negative-feedback loop through which AtGRP7 regulates the circadian oscillations of its own transcript. Gene expression is induced by cold. GRP7 appears to promote stomatal opening and reduce tolerance under salt and dehydration stress conditions, but, promotes stomatal closing and thereby increases stress tolerance under conditions of cold tolerance.
AT2G21660	1652.777	-2.4838	0.23312	-10.655	1.66E-26	1.15E-24	8.22138	9.35632	8.75631	11.6966	11.3459	11.3522	A1-	
AT2G21660	1938.013	-2.7796	0.23487	-11.835	2.58E-32	8.01E-30	8.12365	9.26181	8.66467	11.8356	11.6729	11.7378	A2-	
AT2G21900	77.13376	4.39781	0.41086	10.704	9.75E-27	7.73E-25	7.15842	7.10302	7.45447	2.60404	0	2.58406	A1+	WRKY DNA-binding protein 59 (WRKY59);
AT2G22470	200.0311	3.11337	0.31149	9.99513	1.60E-23	1.07E-21	8.44452	8.15393	8.86627	5.87214	3.95523	5.01018	A1+	AGP2 Encodes arabinogalactan-protein (AGP2)
AT2G23030	51.4888	-1.7246	0.31403	-5.4919	3.98E-08	5.90E-07	4.98718	4.20157	4.24293	6.17229	6.57474	6.29355	A1+	SNF1-related protein kinase 2.9 (SNRK2.9); FUNCTIONS IN: protein serine/threonine kinase activity, protein kinase activity, kinase activity, ATP binding; INVOLVED IN: protein amino acid phosphorylation, response to osmotic stress; EXPRESSED IN: hypocotyl, root
AT2G23030	48.43805	-1.7316	0.30865	-5.6102	2.02E-08	3.55E-07	4.88274	4.0988	4.13877	6.51493	6.13862	6.13896	A1-	
AT2G23030	80.55024	-2.6326	0.26818	-9.8168	9.53E-23	1.38E-20	4.78811	4.00984	4.05227	6.87521	7.37814	7.18211	A2-	
AT2G23270	40.56418	2.55925	0.47375	5.40206	6.59E-08	9.50E-07	6.07597	6.1853	6.42163	1.42937	0	4.30275	A1+	Transmembrane protein
AT2G25460	71.05939	2.50355	0.35154	7.12163	1.07E-12	2.89E-11	6.83055	7.21285	6.81934	5.05192	3.71068	3.45845	A1+	EEIG1/EHBP1 protein amino-terminal domain protein, CONTAINS InterPro DOMAIN/s: C2 calcium-dependent membrane targeting
AT2G27080	178.9118	2.98888	0.3201	9.33721	9.89E-21	5.21E-19	8.2116	7.95964	8.75089	5.82982	4.16427	4.83185	A1+	Late embryogenesis abundant (LEA) hydroxyproline-rich glycoprotein family; CONTAINS InterPro DOMAIN/s: Late embryogenesis abundant protein, group 2
AT2G27660	53.82416	2.53596	0.40183	6.31099	2.77E-10	5.61E-09	6.67365	6.39557	6.70427	4.72243	0	3.45845	A1+	Cysteine/Histidine-rich C1 domain family protein; FUNCTIONS IN: zinc ion binding
AT2G27690	132.6377	3.56402	0.34202	10.4205	2.00E-25	1.48E-23	7.69406	7.56736	8.46557	4.4141	3.95523	3.88164	A1+	CYP94C1, cytochrome P450, family 94, subfamily C, polypeptide 1, Encodes a CYP94C1. Has highest omega-hydroxylase activity with 9,10-epoxystearic acid, while also metabolized lauric acid (C12:0) and C18 unsaturated fatty acids. Gene expression is induced in response to wounding and jasmonic acid treatment
AT2G32020	52.53943	2.69604	0.37628	7.16489	7.79E-13	2.14E-11	6.27637	6.45042	6.92591	3.68404	2.54524	3.99899	A1+	Acyl-CoA N-acyltransferases (NAT) superfamily protein; FUNCTIONS IN: N-acetyltransferase activity; INVOLVED IN: response to abscisic acid stimulus, metabolic process
AT2G32810	285.3039	3.10929	0.21425	14.5125	1.01E-47	2.42E-45	8.87692	9.17329	8.96241	5.25748	6.20076	5.8998	A1+	BGAL9, beta galactosidase 9 (BGAL9); FUNCTIONS IN: sugar binding, cation binding, beta-galactosidase activity, hydrolase activity, hydrolyzing O-glycosyl compounds, catalytic activity; INVOLVED IN: lactose catabolic process, using glucoside 3-dehydrogenase, carbohydrate metabolic process, lactose catabolic process via UDP-galactose, lactose catabolic process
AT2G32810	258.6063	3.42308	0.21646	15.8142	2.48E-56	5.69E-54	8.76919	9.06459	8.85244	5.28853	5.45092	5.35204	A1-	
AT2G33830	8176.052	-2.4636	0.15152	-16.259	1.92E-59	7.35E-57	11.4493	10.9359	11.3406	13.8349	13.8692	13.5671	A1+	Dormancy/auxin associated family protein
AT2G33830	8691.835	-2.6584	0.16985	-15.651	3.25E-55	7.01E-53	11.3413	10.827	11.2305	14.1081	13.8709	13.6328	A1-	
AT2G33830	6308.211	-2.2021	0.18708	-11.771	5.52E-32	1.67E-29	11.2433	10.7324	11.1387	13.1557	13.1903	13.6665	A2-	

AT2G34130	79.88609	-6.3431	0.46794	-13.555	7.37E-42	1.32E-39	0	0.62641	0	7.33863	7.70731	6.79335	A1+	MEE19 maternal effect embryo arrest 19; hypothetical protein
AT2G34130	3.850254	-1.8551	0.51128	-3.6283	0.000285	0.0023799	0	0.58899	0	2.52621	3.54662	3.02706	A1-	
AT2G34130	147.3262	-2.9702	0.17873	-16.618	5.17E-62	1.06E-58	0	0.94033	0	7.71637	8.64221	8.10949	A2+	
AT2G35820	258.8268	2.07085	0.21143	9.79454	1.19E-22	7.38E-21	8.87005	8.58822	8.70673	7.03764	6.20076	6.38264	A1+	Ureidoglycolate hydrolases; FUNCTIONS IN: ureidoglycolate hydrolase activity; INVOLVED IN: allantoin catabolic process;
AT2G35820	229.7069	2.53591	0.20137	12.5934	2.30E-36	2.50E-34	8.76232	8.47962	8.5968	5.86113	6.43434	5.93726	A1-	
AT2G35930	93.08535	3.34331	0.32516	10.2819	8.50E-25	6.10E-23	7.3729	7.27495	7.65812	4.4141	2.54524	3.88164	A1+	PUB23, plant U-box 23, Encodes a cytoplasmically localized U-box domain containing E3 ubiquitin ligase that is involved in the response to water stress and acts as a negative regulator of PAMP-triggered immunity
AT2G36270	16.38157	-2.6918	0.46795	-5.7522	8.81E-09	1.44E-07	2.12133	1.3957	2.23589	4.52436	5.12347	5.11789	A1+	ABA INSENSITIVE 5 (ABI5), Encodes a member of the basic leucine zipper transcription factor family, involved in ABA signalling during seed maturation and germination. The Arabidopsis abscisic acid (ABA)-insensitive abi5 mutants have pleiotropic defects in ABA response, including decreased sensitivity to ABA inhibition of germination and altered expression of some ABA-regulated genes. Comparison of seed and ABA-inducible vegetative gene expression in wild-type and abi5-1 plants indicates that ABI5 regulates a subset of late embryogenesis-abundant genes during both developmental stages
AT2G36750	36.42553	3.87605	0.52066	7.4445	9.73E-14	2.91E-12	5.24261	6.37682	6.61607	0	0	1.80658	A1+	UGT73C1, UDP-glucosyl transferase 73C1
AT2G36790	980.0984	2.78387	0.56146	4.95822	7.11E-07	8.74E-06	10.4949	10.8467	11.185	7.6738	4.90796	5.31183	A1+	UGT73C6, UDP-glucosyl transferase 73C6, The At2g36790 gene encodes a UDP-glucose:flavonol-3-O-glycoside-7-O-glucosyltransferase (UGT73C6)attaching a glucosyl residue to the 7-O-position of the flavonols kaempferol, quercetin and their 3-O-glycoside derivatives. Overexpression of the UGT73C6 alters brassinosteroid glucoside formation in Arabidopsis thaliana
AT2G36790	2253.865	0.78848	0.17104	4.60987	4.03E-06	3.68E-04	11.2417	11.6035	11.9273	9.62198	10.6901	10.6979	A2+	
AT2G36790	1237.783	0.86475	0.2441	3.54266	0.000396	0.0039396	10.289	10.6432	10.983	9.99357	9.96065	9.08322	A2-	
AT2G36800	511.2186	2.82201	0.21804	12.9426	2.59E-38	3.89E-36	9.44245	9.92172	10.0394	7.17921	6.37902	6.99045	A1+	DOGT1, don-glucosyltransferase 1, Encodes a DON-Glucosyltransferase. The UGT73C5 glucosylates both brassinolide and castasterone in the 23-O position. The enzyme is presumably involved in the homeostasis of those steroid hormones hence regulating BR activity.
AT2G36800	1139.422	0.74522	0.15768	4.7262	2.29E-06	0.0002205	10.1889	10.6782	10.7814	9.11114	9.67873	9.82025	A2+	
AT2G37125	10.4983	-3.3463	0.58358	-5.734	9.81E-09	1.58E-07	0	0	0	2.60404	4.6545	5.11789	A1+	GRF zinc finger protein
AT2G39490	30.72113	2.82624	0.44495	6.35181	2.13E-10	4.37E-09	5.7564	5.67306	6.06977	3.48023	1.77337	1.80658	A1+	F-box family protein
AT2G40750	75.17003	3.08932	0.37401	8.25998	1.46E-16	5.59E-15	7.15842	6.73447	7.41841	4.29471	0	3.99899	A1+	WRKY54 WRKY DNA-binding protein 54, member of WRKY Transcription Factor; Group III
AT2G41280	4.245576	-1.7034	0.51508	-3.3069	0.000943	0.0068504	0.60238	1.00603	0.65347	3.68946	2.66509	2.83386	A1-	Glutamyl-tRNA (Gln) amidotransferase subunit C
AT2G42065	28.97269	3.63402	0.49131	7.39655	1.40E-13	4.13E-12	5.7564	5.71846	6.04296	1.42937	0	1.80658	A1+	DnaJ domain protein, DnaJ is a member of the hsp40 family of molecular chaperones, which is also called the J-protein family, the members of which regulate the activity of hsp70s
AT2G42065	35.05978	1.13525	0.34262	3.31343	9.22E-04	8.14E-03	5.55445	5.51905	5.84418	4.91162	4.6895	3.34439	A2-	
AT2G42530	516.0136	-2.7601	0.26902	-10.26	1.07E-24	7.67E-23	7.00378	6.68958	7.05229	8.94268	10.1853	10.0787	A1+	Cold regulated 15b (COR15B); INVOLVED IN: response to cold, defense response to fungus
AT2G42530	500.6042	-2.7009	0.30029	-8.9943	2.38E-19	2.66E-17	6.79946	6.48814	6.85186	10.5682	9.22386	9.17178	A2-	
AT2G44070	26.30665	2.55074	0.44801	5.69344	1.25E-08	1.98E-07	5.67988	5.76248	5.34266	3.24283	0	2.85705	A1+	NagB/RpiA/CoA transferase-like superfamily protein; FUNCTIONS IN: GTP binding, translation initiation factor activity; INVOLVED IN: translational initiation, cellular metabolic process; LOCATED IN: eukaryotic translation initiation factor 2B complex
AT2G44840	26.56838	3.06393	0.50564	6.0595	1.37E-09	2.51E-08	5.47775	5.11732	6.2564	1.42937	0	2.58406	A1+	ERF13, EREBP, ATERF13, ETHYLENE-RESPONSIVE ELEMENT BINDING FACTOR 13, encodes a member of the ERF (ethylene response factor) subfamily B-3 of ERF/AP2 transcription factor family. The protein contains one AP2 domain. There are 18 members in this subfamily including ATERF-1, ATERF-2, AND ATERF-5
AT2G47000	981.7918	3.24359	0.21804	14.876	4.72E-50	1.25E-47	10.3671	10.9707	11	7.78874	6.98599	7.43772	A1+	ABCB4, ATP binding cassette subfamily B4 , MDR4, ARABIDOPSIS P-GLYCOPROTEIN 4, AtABCB4, MULTIDRUG RESISTANCE 4, ATPGP4, PGP4, P-GLYCOPROTEIN 4, Encodes an auxin efflux transmembrane transporter that is a member of the multidrug resistance P-glycoprotein (MDR/PGP) subfamily of ABC transporters. Functions in the basipetal redirection of auxin from the root tip. Exhibits apolar plasma membrane localization in the root cap and polar localization in tissues above and is involved in root hair elongation.
AT3G01345	198.4049	-7.0759	0.38344	-18.454	4.86E-76	3.57E-73	1.41979	1.3957	0	8.92268	8.46803	8.45425	A1+	Expressed protein;(source:Araport11)
AT3G01345	176.2633	-6.8286	0.34752	-19.65	5.84E-86	3.85E-83	1.35313	1.32917	0	8.45493	8.42177	8.50653	A1-	
AT3G01345	16.68053	-0.8825	0.16359	-5.3948	6.86E-08	7.93E-06	1.93072	1.90954	0	4.55904	5.52727	4.79821	A2+	
AT3G01760	25.02919	3.22528	0.5016	6.43004	1.28E-10	2.70E-09	5.26374	5.62619	5.91573	0	0	2.58406	A1+	LHT6, lysine-histidine-like transporter 6, Encodes an amino acid transporter expressed in the root that is involved in the uptake of acidic amino acids, glutamine and alanine, and probably phenylalanine.
AT3G05320	81.68409	2.60865	0.32216	8.09735	5.62E-16	2.06E-14	7.10089	6.97169	7.41841	5.05192	3.71068	3.99899	A1+	O-fucosyltransferase family protein
AT3G05320	91.88521	0.99245	0.28089	3.53324	4.10E-04	4.07E-03	6.89646	6.76987	7.21762	5.98536	6.32861	5.18915	A2-	
AT3G06890	51.59412	3.66521	0.43311	8.46256	2.62E-17	1.07E-15	6.46147	6.31904	7.04555	2.60404	1.77337	2.58406	A1+	Transmembrane protein, downregulated in RETARDED GROWTH OF EMBRYO1 mutant, which has small seeds
AT3G07195	119.2622	2.63595	0.5439	4.84637	1.26E-06	1.48E-05	7.96306	7.74972	7.70591	5.12374	1.77337	3.28443	A1+	RPM1-interacting protein 4 (RIN4) family protein; CONTAINS InterPro DOMAIN/s: RPM1-interacting protein 4, defence response
AT3G09870	27.54803	3.3042	0.52593	6.28256	3.33E-10	6.62E-09	5.77123	4.78881	6.34687	2.1331	0	1.16932	A1+	SMALL AUXIN UPREGULATED RNA 48, SAUR48, SAUR-like auxin-responsive protein family ; FUNCTIONS IN: molecular_function unknown; INVOLVED IN: response to auxin stimulus

AT3G10986	33.47166	3.85317	0.51041	7.54913	4.38E-14	1.35E-12	5.80043	5.68836	6.54141	2.1331	0	0	A1+	LURP-one-like protein (DUF567)
AT3G10986	73.97447	0.78982	0.18018	4.38364	1.17E-05	0.0009951	6.5372	6.43402	7.27779	4.42764	5.52727	5.57567	A2+	
AT3G12900	23.92255	3.0851	0.53607	5.75507	8.66E-09	1.41E-07	4.85422	5.02396	6.32478	0	1.77337	1.80658	A1+	2-oxoglutarate (2OG) and Fe(II)-dependent oxygenase superfamily protein; FUNCTIONS IN: oxidoreductase activity, acting on paired donors, with incorporation or reduction of molecular oxygen, 2-oxoglutarate as one donor, and incorporation of one atom each of oxygen into both donors, oxidoreductase activity;
AT3G13080	2455.457	2.99961	0.4479	6.6971	2.13E-11	4.95E-10	11.6753	12.329	12.3513	9.242	7.43225	8.31972	A1+	ABCC3 ATP-binding cassette C3, MRP3, MRP3, MULTIDRUG RESISTANCE PROTEIN 3, multidrug resistance-associated protein 3, multidrug resistance-associated protein 3 (MRP3); FUNCTIONS IN: chlorophyll catabolite transmembrane transporter activity, ATPase activity, coupled to transmembrane movement of substances, glutathione S-conjugate-exporting ATPase activity; INVOLVED IN: transport, transmembrane transport
AT3G13080	5721.075	0.6943	0.16675	4.16383	3.13E-05	0.002413	12.4223	13.086	13.0939	11.255	12.1357	12.1233	A2+	
AT3G13100	55.05717	2.61247	0.39378	6.6344	3.26E-11	7.43E-10	6.05188	6.61151	7.05899	4.29471	3.04536	3.45845	A1+	ABCC7, ATP-binding cassette C7 multidrug resistance-associated protein 7 (MRP7); MRP7, multidrug resistance-associated protein 7, MRP7, multidrug resistance-associated protein 7, ATMRP7 FUNCTIONS IN: ATPase activity, coupled to transmembrane movement of substances; INVOLVED IN: response to other organism;
AT3G13600	82.23187	2.78731	0.30366	9.17899	4.35E-20	2.21E-18	7.06523	7.25967	7.2528	4.6268	4.50885	3.61372	A1+	Calmodulin-binding family protein;
AT3G14450	50.24215	1.47411	0.33029	4.46314	8.08E-06	8.15E-05	6.11136	6.25885	6.44229	4.29471	4.16427	5.21812	A1+	CID9, CTC-interacting domain 9, CTC-interacting domain 9 (CID9); FUNCTIONS IN: RNA binding, nucleotide binding, nucleic acid binding;
AT3G14450	38.12738	2.77822	0.37869	7.33639	2.19E-13	6.35E-12	6.00501	6.15143	6.33341	3.54983	0	2.61074	A1-	
AT3G14735	241.001	4.36804	0.25463	17.1545	5.82E-66	1.05E-62	8.86355	8.81692	8.90851	4.44524	3.98933	4.08955	A2-	AtALD1
AT3G15440	38.60537	-3.3277	0.39776	-8.3662	5.95E-17	2.36E-15	3.14059	1.89477	2.41385	5.87214	6.61089	5.98762	A1+	RING/U-box protein
AT3G15440	39.88521	-3.4068	0.36487	-9.3369	9.92E-21	4.92E-19	3.04528	1.81597	2.32499	6.51493	6.24407	5.93726	A1-	
AT3G15440	19.24577	-2.2712	0.38854	-5.8455	5.05E-09	1.55E-07	2.95945	1.74883	2.25193	5.09826	4.86348	5.39938	A2-	
AT3G16860	102.5916	2.58652	0.27	9.57975	9.73E-22	5.59E-20	7.47607	7.40097	7.59953	5.19216	3.95523	4.83185	A1+	COBL8; COBRA-like protein 8 precursor
AT3G17609	24.89941	3.12232	0.48083	6.49355	8.38E-11	1.82E-09	5.69551	5.43997	5.65746	1.42937	0	2.58406	A1+	HYH, HY5-homolog, Encodes a homolog of HY5 (HYH). Involved in phyB signaling pathway
AT3G18610	47.29103	2.76668	0.39851	6.94261	3.85E-12	9.79E-11	6.27637	6.58724	6.44229	4.29471	0	3.08651	A1+	PARLL1, NUC2, nucleolin like 2, PARALLEL1-LIKE 1, nucleolin 2, ATNUC-L2, nucleolin like 2 (NUC-L2); FUNCTIONS IN: nucleotide binding, nucleic acid binding
AT3G19390	45.84889	-2.4807	0.32932	-7.5328	4.97E-14	1.52E-12	3.39154	3.91999	3.74076	5.99213	6.71419	6.19861	A1+	Granulin repeat cysteine protease family protein; FUNCTIONS IN: cysteine-type endopeptidase activity, cysteine-type peptidase activity; INVOLVED IN: proteolysis
AT3G19390	61.8966	-2.8138	0.33997	-8.2765	1.27E-16	4.83E-15	3.29421	3.81854	3.6391	6.45747	7.51294	6.13896	A1-	
AT3G19390	45.63038	-2.4445	0.3569	-6.8494	7.42E-12	3.55E-10	3.20645	3.7308	3.55483	4.91162	6.92731	6.55019	A2-	
AT3G20340	165.3621	-2.9116	0.3144	-9.2607	2.03E-20	1.06E-18	5.54832	4.5418	5.02412	7.78874	8.79308	7.85067	A1+	Expression of the gene is downregulated in the presence of paraquat, an inducer of photoxidative stress.
AT3G20340	136.9047	-2.7703	0.25018	-11.073	1.69E-28	1.30E-26	5.44274	4.43775	4.91744	7.8424	8.12318	7.80553	A1-	
AT3G20340	163.7195	-2.9309	0.2984	-9.8222	9.04E-23	1.31E-20	5.347	4.3476	4.82871	8.78671	7.67518	7.98194	A2-	
AT3G21080	109.9014	4.68371	0.40619	11.5309	9.21E-31	9.07E-29	7.52501	7.49529	8.15409	2.9585	1.77337	2.24707	A1+	ABC transporter-like protein
AT3G21570	9.18913	-3.4506	0.57865	-5.9632	2.47E-09	4.39E-08	0	0	0	3.24283	4.6545	4.55355	A1+	Proline-rich nuclear receptor coactivator
AT3G21570	3.300579	-1.9488	0.50665	-3.8464	0.00012	0.0011067	0	0	0	3.22203	2.66509	2.83386	A1-	
AT3G24542	38.6852	-5.5514	0.50822	-10.923	8.93E-28	7.50E-26	0	0	0	5.6469	6.57474	6.48677	A1+	Beta-galactosidase related protein
AT3G24542	31.85273	-4.8891	0.46234	-10.575	3.90E-26	2.63E-24	0	0	0	5.45435	6.60241	5.73073	A1-	
AT3G27150	12.15375	2.63724	0.54511	4.83797	1.31E-06	1.54E-05	4.50977	4.5418	4.68577	1.42937	1.77337	0	A1+	Galactose oxidase/kelch repeat superfamily protein;Target gene of MIR2111-5p
AT3G27473	12.82813	-2.9842	0.51967	-5.7426	9.32E-09	1.51E-07	1.08201	0.62641	1.79647	5.05192	4.16427	4.62832	A1+	Cysteine/Histidine-rich C1 domain family protein
AT3G27473	6.510493	-1.7967	0.51326	-3.5006	0.000464	0.0036704	1.02605	0.58899	1.71887	3.54983	0	4.67351	A1-	
AT3G28193	12.67188	-3.8231	0.55088	-6.94	3.92E-12	9.95E-11	0	0	0.69459	4.4141	5.12347	4.4747	A1+	Transmembrane protein
AT3G28193	23.23429	-4.477	0.4608	-9.7158	2.58E-22	1.41E-20	0	0	0.65347	5.60306	5.76624	5.278	A1-	
AT3G28580	54.00222	3.07824	0.39686	7.75655	8.73E-15	2.86E-13	6.32743	6.89467	6.72128	3.24283	0	3.88164	A1+	P-loop containing nucleoside triphosphate hydrolases superfamily protein; FUNCTIONS IN: nucleoside-triphosphatase activity, ATPase activity, nucleotide binding, ATP binding; INVOLVED IN: response to abscisic acid stimulus
AT3G28917	7.990181	-2.8521	0.5776	-4.9378	7.90E-07	9.61E-06	0.64015	0.62641	0	4.81212	3.04536	3.75389	A1+	Mini zinc finger protein 2
AT3G29250	100.0304	4.00899	0.4037	9.93063	3.06E-23	1.99E-21	7.32848	7.06252	8.16034	2.9585	3.41604	2.85705	A1+	SDR4 short-chain dehydrogenase reductase 4, NAD(P)-binding Rossmann-fold superfamily protein; FUNCTIONS IN: oxidoreductase activity, copper ion binding; INVOLVED IN: oxidation reduction, metabolic process
AT3G29250	111.8053	1.26124	0.35995	3.50389	4.59E-04	4.45E-03	7.12381	6.86059	7.95903	5.66912	6.5737	4.71048	A2-	
AT3G30165	28.09512	-5.0919	0.52423	-9.7131	2.65E-22	1.59E-20	0	0	0	6.3018	5.76042	5.26573	A1+	Zinc ion-binding protein
AT3G30720	311.0296	-4.185	0.18175	-23.026	2.57E-117	5.50E-114	4.90888	4.97492	5.02412	9.04334	9.19545	9.37559	A1+	QQS qua-quine starch
AT3G30720	253.0322	-3.9513	0.19072	-20.717	2.41E-95	2.21E-92	4.80464	4.86961	4.91744	9.08984	8.79585	8.8056	A1-	
AT3G30720	79.46087	-0.748	0.16612	-4.5029	6.70E-06	5.92E-04	5.6366	5.7132	5.7488	6.73715	6.8303	6.73207	A2+	
AT3G30751	37.71811	-5.5928	0.50492	-11.077	1.63E-28	1.43E-26	0	0	0	6.2385	5.82339	6.60256	A1+	Putative uncharacterized protein
AT3G30770	35.71235	-5.0975	0.5147	-9.9038	4.01E-23	2.57E-21	0.64015	0	0	6.85977	5.39625	5.8693	A1+	Eukaryotic aspartyl protease family protein
AT3G30770	36.67141	-5.1491	0.44266	-11.632	2.83E-31	2.44E-29	0.60238	0	0	6.12987	5.76624	6.61552	A1-	

AT3G30775	1990.53	-1.205	0.21129	-5.7032	1.18E-08	1.88E-07	10.4547	9.8038	10.2822	11.406	11.8117	11.0349	A1+	EARLY RESPONSIVE TO DEHYDRATION 5 (ERD5); CONTAINS InterPro DOMAIN/s: Proline dehydrogenase (InterPro:IPR002872), Proline oxidase, Encodes a proline oxidase that is predicted to localize to the inner mitochondrial membrane, its mRNA expression induced by high levels of Al and by osmotic stress. The promoter contains an L-proline-inducible element.
AT3G30775	4479.675	-2.7185	0.2111	-12.878	6.01E-38	6.99E-36	10.3468	9.69502	10.1721	12.6705	13.3551	12.6886	A1-	
AT3G31910	31.3262	-4.8547	0.4821	-10.07	7.50E-24	5.10E-22	1.08201	0.62641	0	6.3018	5.55311	5.98762	A1+	Ulp1 protease family protein (DUF1985)
AT3G31910	16.35862	-3.7091	0.46699	-7.9426	1.98E-15	6.84E-14	1.02605	0.58899	0	4.88574	5.26298	4.98409	A1-	
AT3G42060	108.1823	-6.5237	0.40624	-16.059	4.98E-58	1.18E-55	0	0	0.65347	8.08933	7.74622	7.36161	A1-	Myosin heavy chain-like protein
AT3G42723	33.61586	-4.2053	0.53	-7.9344	2.11E-15	7.40E-14	0	0.62641	1.51363	7.03764	5.01973	5.26573	A1+	ATP binding / aminoacyl-tRNA ligase/ nucleotide binding protein
AT3G42723	10.65187	-2.8695	0.49135	-5.8401	5.22E-09	9.87E-08	0	0.58899	1.44299	4.70005	4.48353	4.02897	A1-	
AT3G44070	62.39451	-5.7091	0.5194	-10.992	4.19E-28	3.58E-26	0	0	0	7.84294	6.46052	5.8998	A1+	Glycosyl hydrolase family 35 protein
AT3G44070	55.27904	-5.6166	0.44248	-12.694	6.42E-37	7.17E-35	0	0	0	6.51493	7.38044	6.25929	A1-	
AT3G44265	87.00657	-6.3759	0.44867	-14.211	7.87E-46	1.57E-43	0.64015	0.62641	0	7.68572	7.56715	7.00455	A1+	Beta-galactosidase-like protein
AT3G44265	66.43804	-5.8655	0.40576	-14.456	2.31E-47	3.95E-45	0.60238	0.58899	0	6.94936	7.18241	7.04187	A1-	
AT3G44265	7.679966	-0.549	0.13982	-3.9266	8.62E-05	6.11E-03	0.95419	0.94033	0	3.35533	4.09708	4.31292	A2+	
AT3G44350	55.20153	2.83143	0.40421	7.00491	2.47E-12	6.41E-11	6.96561	6.71966	6.23287	4.29471	0	3.45845	A1+	NAC61, NAC domain containing protein 61, DOMAIN/s: No apical meristem (NAM) protein (InterPro:IPR003441);
AT3G45090	60.22633	1.82932	0.30056	6.08636	1.16E-09	2.14E-08	6.56722	6.68958	6.57922	4.6268	3.95523	5.06504	A1+	P-loop containing nucleoside triphosphate hydrolases superfamily protein
AT3G45090	50.23225	2.53304	0.32531	7.78645	6.89E-15	2.26E-13	6.46043	6.58177	6.47022	3.93374	2.66509	3.72897	A1-	
AT3G45300	3240.753	-2.8412	0.1634	-17.388	1.02E-67	5.80E-65	9.91598	9.38871	9.38163	12.2973	12.581	12.5475	A1+	Isovaleryl-CoA-dehydrogenase (IVD); FUNCTIONS IN: isovaleryl-CoA dehydrogenase activity, ATP binding; INVOLVED IN: leucine catabolic process
AT3G45300	2673.607	-2.6184	0.17856	-14.664	1.09E-48	1.95E-46	9.80813	9.27998	9.27161	12.2169	12.3633	11.9227	A1-	
AT3G45300	2154.113	-2.3452	0.18769	-12.495	7.94E-36	3.02E-33	9.71017	9.18548	9.17989	11.5756	11.8266	12.052	A2-	
AT3G45700	37.28339	4.09897	0.52707	7.77686	7.43E-15	2.46E-13	6.08786	5.47566	6.81934	0	0	1.16932	A1+	Major facilitator superfamily protein; FUNCTIONS IN: transporter activity; INVOLVED IN: oligopeptide transport
AT3G45960	37.73716	2.95511	0.46942	6.29527	3.07E-10	6.16E-09	6.03968	5.40337	6.72128	3.24283	2.54524	1.80658	A1+	EXLA3, EXPL3, expansin-like A3, ATEXLA3, ATEXPL3, ATHEXP BETA 2.3, expansin-like A3 (EXLA3); INVOLVED IN: plant-type cell wall organization, unidimensional cell growth, plant-type cell wall loosening;
AT3G48640	39.3615	3.5112	0.45654	7.69095	1.46E-14	4.69E-13	5.84317	6.45042	6.39006	2.9585	1.77337	1.16932	A1+	transmembrane protein
AT3G50770	76.3482	2.81603	0.35774	7.87169	3.50E-15	1.19E-13	6.69714	7.31491	7.2528	4.81212	3.04536	3.61372	A1+	CML41, calmodulin-like 41 FUNCTIONS IN: calcium ion binding
AT3G50770	191.0644	-0.9478	0.25348	-3.7392	0.000185	0.0016262	6.59025	7.20672	7.14335	7.55346	7.85009	8.44442	A1-	
AT3G53910	20.87058	-4.6824	0.53931	-8.6821	3.88E-18	1.70E-16	0	0	0	5.43737	4.6545	5.8998	A1+	Malate dehydrogenase-like protein
AT3G53910	12.98254	-3.7005	0.49604	-7.4602	8.64E-14	2.61E-12	0	0	0	4.32498	5.26298	4.48892	A1-	
AT3G54730	65.51468	-5.2216	0.38584	-13.533	9.98E-42	1.39E-39	0.60238	1.32917	1.44299	7.0304	7.38044	6.56971	A1-	Putative transmembrane protein
AT3G54730	11.03196	-0.5818	0.15133	-3.8446	1.21E-04	8.23E-03	0.95419	1.90954	2.03713	3.8418	5.00689	3.99111	A2+	
AT3G55150	17.32364	2.65396	0.52044	5.09943	3.41E-07	4.39E-06	4.96155	4.94976	5.25192	2.9585	0	0	A1+	EXO70H1, exocyst subunit exo70 family protein H1, INVOLVED IN: exocytosis, vesicle docking involved in exocytosis; A member of EXO70 gene family, putative exocyst subunits, conserved in land plants. Arabidopsis thaliana contains 23 putative EXO70 genes, which can be classified into eight clusters on the phylogenetic tree.
AT3G56380	14.09531	3.05577	0.55426	5.51329	3.52E-08	5.28E-07	4.76813	4.47283	5.20432	0	0	1.16932	A1+	RR17, response regulator 17 (RR17); CONTAINS InterPro DOMAIN/s: CheY-like (InterPro:IPR011006), Signal transduction response regulator
AT3G56380	13.30253	2.02892	0.43802	4.63207	3.62E-06	6.38E-05	4.5702	4.27908	5.00812	2.36651	2.10673	0	A2-	
AT3G56891	137.1618	4.77864	0.3926	12.1717	4.40E-34	5.21E-32	8.19245	7.60008	8.33903	3.68404	0	2.24707	A1+	Heavy metal transport/detoxification superfamily protein, FUNCTIONS IN: metal ion binding; INVOLVED IN: metal ion transport
AT3G57460	35.3446	2.6212	0.4524	5.79397	6.87E-09	1.14E-07	5.38439	6.08487	6.44229	3.24283	0	3.45845	A1+	Catalytics;metal ion binding; FUNCTIONS IN: catalytic activity, metal ion binding
AT3G57520	4407.309	-3.5127	0.14179	-24.774	1.71E-135	5.49E-132	9.50367	9.47722	9.28483	12.9701	13.2226	12.7323	A1+	ATSIP2 encodes a raffinose-specific alpha-galactosidase that catalyzes the breakdown of raffinose into alpha-galatose and sucrose. This enzyme may function in unloading raffinose from the phloem as part of sink metabolism. Although it was originally predicted to act as a raffinose synthase (RS), that activity was not observed for recombinant SIP2.
AT3G57520	5030.653	-3.7433	0.19314	-19.381	1.11E-83	6.48E-81	9.39585	9.36848	9.17481	12.9235	13.6417	12.9158	A1-	
AT3G57520	2290.814	-2.5441	0.23673	-10.747	6.14E-27	1.26E-24	9.29793	9.27397	9.08311	11.2033	12.1385	12.3144	A2-	
AT3G59330	31.8754	2.31716	0.41387	5.59882	2.16E-08	3.33E-07	6.00246	5.64198	5.83987	2.9585	1.77337	3.88164	A1+	Solute carrier family 35 protein (DUF914)
AT3G59330	28.20703	2.82428	0.41356	6.82917	8.54E-12	2.15E-10	5.89624	5.53535	5.73167	2.52621	2.66509	2.02322	A1-	
AT3G59930	57.04168	-2.773	0.35138	-7.8917	2.98E-15	1.02E-13	4.10402	3.63438	3.27171	7.21253	6.24743	6.40407	A1+	Defensin-like protein
AT3G59930	55.8357	-2.8179	0.30535	-9.2286	2.74E-20	1.31E-18	4.00256	3.53456	3.17332	6.78781	6.82268	6.33427	A1-	
AT3G61060	633.4733	-2.3053	0.29578	-7.7942	6.48E-15	2.16E-13	8.04771	7.20755	7.41841	9.15039	10.4919	10.2468	A1+	ATPP2-A13, PHLOEM PROTEIN 2-A13, PP2-A13, phloem protein 2-A13
AT3G61060	513.357	-2.0813	0.25013	-8.3209	8.73E-17	3.36E-15	7.94016	7.09942	7.30888	9.29236	10.1936	9.54457	A1-	
AT3G61060	814.3741	-2.9369	0.21596	-13.6	4.02E-42	2.27E-39	7.8425	7.00548	7.21762	10.2855	10.5403	10.6866	A2-	
AT3G61190	134.7409	3.0632	0.34287	8.93411	4.10E-19	1.97E-17	7.97911	7.45562	8.2971	5.37986	3.41604	4.30275	A1+	BAP, BON association protein 1, Encodes a protein with a C2 domain that binds to BON1 in yeast two hybrid analyses. Its ability to bind to phospholipids is enhanced by calcium ions. Involved in maintaining cell homeostasis.

AT3G62210	15.66843	3.20769	0.57207	5.60711	2.06E-08	3.18E-07	4.54471	4.28441	5.75981	0	0	0	A1+	EDA32 embryo sac development arrest 32, Putative endonuclease or glycosyl hydrolase
AT4G00130	23.70858	2.45229	0.45389	5.40289	6.56E-08	9.47E-07	5.28456	5.36582	5.62167	3.24283	2.54524	1.80658	A1+	DNA-binding storekeeper protein-related transcriptional regulator
AT4G00130	236.0499	-3.3549	0.20005	-16.77	4.03E-63	1.14E-60	5.17947	5.25966	5.5138	8.64277	8.5711	9.028	A1-	
AT4G02160	21.19472	3.34846	0.57263	5.84752	4.99E-09	8.42E-08	5.17731	3.9709	6.29099	0	0	0	A1+	Cotton fiber protein
AT4G02170	64.83101	3.97881	0.47833	8.31809	8.94E-17	3.47E-15	6.78743	6.07327	7.68004	1.42937	1.77337	2.58406	A1+	
AT4G03950	18.92254	-3.2464	0.58075	-5.5899	2.27E-08	3.49E-07	0.64015	0.62641	0	6.53041	3.04536	3.88164	A1+	Nucleotide/sugar transporter family protein
AT4G03950	11.74245	-3.2495	0.49437	-6.5731	4.93E-11	1.16E-09	0.60238	0.58899	0	3.81676	4.7925	4.93675	A1-	
AT4G04030	46.39197	-4.976	0.40854	-12.18	3.97E-34	3.83E-32	0.60238	1.593	0	6.7564	6.60241	6.20038	A1-	Ovate family protein 9 (OFP9)
AT4G04510	18.12962	2.75881	0.52217	5.28334	1.27E-07	1.76E-06	4.54471	5.5276	5.20432	1.42937	0	2.24707	A1+	CRK38, cysteine-rich RLK (RECEPTOR-like protein kinase) 38, FUNCTIONS IN: kinase activity; INVOLVED IN: protein amino acid phosphorylation;
AT4G08190	10.27703	-3.3124	0.50567	-6.5505	5.73E-11	1.34E-09	0	0	0	3.93374	5.04684	4.02897	A1-	P-loop containing nucleoside triphosphate hydrolases superfamily protein;
AT4G08691	65.54856	-5.7213	0.41643	-13.739	5.94E-43	8.62E-41	0.60238	0.58899	0	6.51493	7.23452	7.2595	A1-	Putative uncharacterized protein
AT4G09430	9.929713	-2.5306	0.5337	-4.7416	2.12E-06	2.40E-05	1.08201	1.3957	1.51363	4.4141	3.41604	4.6994	A1+	Disease resistance protein (TIR-NBS-LRR class) family; with Natural antisense transcript At4G09432, FUNCTIONS IN: transmembrane receptor activity, ATP binding;
AT4G09430	11.92829	-2.7206	0.46882	-5.8031	6.51E-09	1.21E-07	1.02605	1.32917	1.44299	4.88574	4.48353	4.19911	A1-	
AT4G11000	83.12398	2.87731	0.36998	7.77698	7.43E-15	2.46E-13	6.91976	7.21285	7.52882	4.97633	3.41604	3.08651	A1+	Ankyrin repeat family protein
AT4G11250	7.244885	2.55601	0.58455	4.37261	1.23E-05	1.20E-04	4.10402	3.81243	3.92825	0	0	0	A1+	AGL52, AGAMOUS-like 52 (AGL52); FUNCTIONS IN: sequence-specific DNA binding transcription factor activity; INVOLVED IN: regulation of transcription, DNA-dependent
AT4G13540	67.59473	-2.6337	0.36226	-7.2702	3.59E-13	1.01E-11	4.05639	4.50773	3.27171	6.3018	7.43225	6.79335	A1+	Golgin family A protein, unknown protein; INVOLVED IN: N-terminal protein myristoylation
AT4G13540	69.99772	-2.6743	0.34081	-7.8469	4.27E-15	1.43E-13	3.95514	4.40379	3.17332	6.90706	7.51294	6.27841	A1-	
AT4G13540	31.9965	-1.6314	0.34301	-4.7561	1.97E-06	3.70E-05	3.8636	4.31375	3.09193	5.2635	5.83848	5.89179	A2-	
AT4G14130	4892.723	-2.6006	0.23501	-11.066	1.84E-28	1.61E-26	10.4971	9.93299	10.5183	12.4982	13.5254	12.947	A1+	XTR7, xyloglucan endotransglycosylase 7, xyloglucan endotransglucosylase/hydrolase 15 (XTH15); FUNCTIONS IN: hydrolase activity, acting on glycosyl bonds, xyloglucan:xyloglucosyl transferase activity, hydrolase activity, hydrolyzing O-glycosyl compounds; INVOLVED IN: N-terminal protein myristoylation, carbohydrate metabolic process, cellular glucan metabolic process
AT4G14130	8997.816	-3.6954	0.1724	-21.435	6.30E-102	6.47E-99	10.3892	9.82421	10.4082	14.1114	14.1494	13.8253	A1-	
AT4G15530	2035.454	-2.1728	0.11395	-19.069	4.60E-81	4.08E-78	9.59714	9.55332	9.37896	11.6724	11.8472	11.5881	A1+	pyruvate orthophosphate dikinase (PPDK); FUNCTIONS IN: kinase activity, pyruvate, phosphate dikinase activity; INVOLVED IN: phosphorylation, response to absence of light, The product of this long transcript was shown to be targeted to the chloroplast, whereas the shorter transcript (no targeting sequence) accumulates in the cytosol. They were also found in slightly different tissues.
AT4G15530	2493.316	-2.5628	0.19839	-12.918	3.55E-38	4.17E-36	9.48931	9.44457	9.26893	11.7151	12.5289	11.8382	A1-	
AT4G15530	2054.274	-2.3434	0.19294	-12.146	6.04E-34	2.02E-31	9.39138	9.35005	9.17722	11.2849	12.078	11.8178	A2-	
AT4G16250	95.0387	2.69749	0.2753	9.79845	1.14E-22	7.11E-21	7.28781	7.45114	7.45447	4.81212	3.95523	4.6994	A1+	PHYD, phytochrome D, Encodes a phytochrome photoreceptor with a function similar to that of phyB that absorbs the red/far-red part of the light spectrum and is involved in light responses. It cannot compensate for phyB loss in Arabidopsis but can substitute for tobacco phyB in vivo.
AT4G16250	85.33817	2.9622	0.28748	10.3042	6.74E-25	4.23E-23	7.18056	7.34289	7.34492	4.32498	3.54662	4.02897	A1-	
AT4G16820	27.77998	3.96485	0.51617	7.68127	1.58E-14	5.03E-13	5.80043	5.74796	5.87069	1.42937	0	0	A1+	PLA-I{beta]2, phospholipase A I beta 2, alpha/beta-Hydrolases superfamily protein; DAD1-Like Lipase 1, DALL1, Encodes a lipase that hydrolyzes phosphatidylcholine, glycolipids as well as triacylglycerols FUNCTIONS IN: galactolipase activity, triglyceride lipase activity, phospholipase A1 activity; INVOLVED IN: lipid metabolic process
AT4G18150	49.63982	-5.6599	0.46688	-12.123	7.99E-34	9.29E-32	1.08201	0	0	6.3018	6.74705	6.82548	A1+	Serine/Threonine-kinase, putative
AT4G18150	50.77746	-5.3883	0.42287	-12.742	3.45E-37	3.91E-35	1.02605	0	0	6.64071	7.01373	6.27841	A1-	
AT4G21680	117.3354	3.12621	0.34061	9.17816	4.39E-20	2.22E-18	7.20285	7.62413	8.25099	4.52436	3.95523	4.4747	A1+	NRT1.8, NITRATE TRANSPORTER 1.8, NRT1/ PTR family 7.2, NPF7.2, AtNPF7.2, Encodes a nitrate transporter (NRT1.8). Functions in nitrate removal from the xylem sap. Mediates cadmium tolerance.
AT4G21680	103.6726	2.69305	0.353	7.629	2.37E-14	1.69E-12	6.99831	7.42168	8.04964	4.91162	4.6895	2.97594	A2-	
AT4G22590	120.3371	2.53574	0.37998	6.67342	2.50E-11	5.78E-10	7.69406	7.49529	7.98127	5.87214	3.04536	4.39129	A1+	TPPG Haloacid dehalogenase-like hydrolase (HAD) superfamily protein; FUNCTIONS IN: catalytic activity, trehalose-phosphatase activity; INVOLVED IN: trehalose biosynthetic process, metabolic process;
AT4G23070	16.04959	3.20211	0.54974	5.82476	5.72E-09	9.59E-08	4.82609	4.70107	5.44861	0	0	1.16932	A1+	RBL7, RHOMBOID-like protein 7 (RBL7); FUNCTIONS IN: serine-type endopeptidase activity;
AT4G24570	111.6137	2.89312	0.27158	10.6531	1.69E-26	1.32E-24	7.75874	7.46008	7.71018	4.89656	4.34682	4.55355	A1+	DIC2 dicarboxylate carrier 2 (DIC2); FUNCTIONS IN: binding, dicarboxylic acid transmembrane transporter activity
AT4G25530	983.3379	-10.316	0.44697	-23.081	7.26E-118	2.12E-114	0	0	0	0	11.1722	11.3504	A1+	FLOWERING WAGENINGEN, FWA, HDG6, HOMEODOMAIN GLABROUS 6
AT4G25530	104.4545	-3.8579	0.50802	0	3.10E-14	9.74E-13	0	0	0	6.53358	8.61108	7.19054	A1-	
AT4G25530	84.59673	-2.5027	0.18014	-13.893	7.00E-44	8.46E-41	0	0	0	7.10288	7.86441	7.13013	A2+	
AT4G25580	63.81279	-5.2969	0.42977	-12.325	6.65E-35	8.33E-33	1.08201	1.06174	1.51363	6.3018	7.28341	7.20105	A1+	Putative uncharacterized protein
AT4G25580	69.81524	-5.2557	0.38752	-13.563	6.68E-42	9.48E-40	1.02605	1.00603	1.44299	6.7564	7.63429	6.79878	A1-	
AT4G25580	82.9546	-5.5765	0.33778	-16.509	3.14E-61	3.65E-58	0.97697	0.95929	1.38559	7.23505	7.36373	7.5066	A2-	
AT4G27654	39.75652	2.96547	0.43548	6.8096	9.79E-12	2.37E-10	5.96426	6.1526	6.50258	3.86258	0	2.24707	A1+	Transmembrane protein
AT4G27654	44.71423	1.27426	0.33832	3.76645	0.000166	0.0018644	5.76176	5.95211	6.3029	5.41174	4.59395	3.63763	A2-	
AT4G29200	124.6455	-6.9681	0.42551	-16.376	2.84E-60	1.16E-57	0	0.62641	0.69459	7.97503	8.02115	7.89677	A1+	Beta-galactosidase related protein;(source:Araport11)

AT4G29200	85.43925	-5.7917	0.42575	-13.604	3.81E-42	5.47E-40	0	0.58899	0.65347	6.67472	8.09527	7.11812	A1-	
AT4G29200	13.50026	-0.8443	0.15745	-5.3622	8.22E-08	9.47E-06	0	0.94033	1.02486	4.84311	5.18785	4.07853	A2+	
AT4G30280	775.46	3.32806	0.51889	6.41378	1.42E-10	2.99E-09	10.3111	10.275	10.9358	6.99997	4.78679	5.11789	A1+	XYL18, xyloglucan endotransglucosylase/hydrolase 18, Encodes a xyloglucan endotransglucosylase/hydrolase with onlyy the endotransglucosylase (XET; EC 2.4.1.207) activity towards xyloglucan and non-detectable endohydrolytic (XEH; EC 3.2.1.151) activity. Expressed in the mature or basal regions of both the main and lateral roots, but not in the tip of these roots where cell division occurs.
AT4G30430	33.13238	3.27877	0.47623	6.88488	5.78E-12	1.44E-10	5.74141	5.65761	6.48276	2.1331	0	2.58406	A1+	TET9, Tetraspanin9 (TET9); FUNCTIONS IN: molecular_function unknown; INVOLVED IN: aging;
AT4G31800	218.3683	2.50357	0.31002	8.07553	6.72E-16	2.43E-14	8.30882	8.48692	8.86627	6.53041	4.50885	5.8693	A1+	WRKY DNA-binding protein 18 (WRKY18)
AT4G33150	1769.791	-3.7789	0.12217	-30.931	4.61E-210	3.95E-206	8.05984	7.8753	7.66253	11.6505	11.7553	11.6656	A1+	LKR/SDH locus. Encodes two proteins. One protein is the monofunctional saccharopine dehydrogenase involved in lysine degradation. The longer protein from the same LKR/SDH locus is bifunctional and also has saccharopine dehydrogenase activity. The monofunctional SDH functions mainly to enhance the flux of lysine catabolism. Gene expression is induced by abscisic acid, jasmonate, and under sucrose starvation.
AT4G33150	1266.716	-3.2826	0.19358	-16.957	1.70E-64	5.09E-62	7.95229	7.76688	7.5529	11.0365	11.5627	10.8358	A1-	
AT4G33150	1530.652	-3.7062	0.15404	-24.061	6.42E-128	1.42E-123	7.85463	7.67267	7.46154	11.2631	11.5895	11.5693	A2-	
AT4G33465	67.31289	-4.0057	0.45497	-8.8043	1.32E-18	6.01E-17	2.71916	2.41948	1.79647	5.43737	7.70731	7.15121	A1+	SCR-LIKE 22, SCRL22, Encodes a member of a family of small, secreted, cysteine rich proteins with sequence similarity to SCR (S locus cysteine-rich protein).
AT4G33465	31.17081	-3.1975	0.37634	-8.4963	1.96E-17	7.87E-16	2.62812	2.33157	1.71887	5.80081	6.24407	5.55378	A1-	
AT4G33465	88.6057	-4.6406	0.3283	-14.135	2.30E-45	1.49E-42	2.54637	2.25622	1.65553	7.27432	7.02213	7.90135	A2-	
AT4G34410	294.0708	2.92737	0.27902	10.4916	9.44E-26	7.16E-24	9.0032	9.30562	8.78365	6.63243	5.12347	5.63557	A1+	RRTF1, ERF109, ethylene response factor 109, redox responsive transcription factor 1 (RRTF1); CONTAINS InterPro DOMAIN/s: DNA-binding, integrase-type (InterPro:IPR016177), Pathogenesis-related transcriptional factor/ERF, DNA-binding
AT4G34410	647.4361	0.78748	0.16507	4.7705	1.84E-06	1.80E-04	9.74933	10.0618	9.52496	8.22181	9.15026	8.48239	A2+	
AT4G37220	132.9132	-2.2069	0.33305	-6.6264	3.44E-11	7.82E-10	5.28456	5.42179	5.5281	6.83858	8.4876	7.65892	A1+	Cold acclimation protein WCOR413 family, early CK response gene
AT4G37220	215.8884	-3.1489	0.21554	-14.609	2.46E-48	4.36E-46	5.17947	5.31552	5.42038	8.32174	9.02025	8.42311	A1-	
AT4G37220	91.87239	-1.9148	0.2448	-7.8221	5.20E-15	3.98E-13	5.08419	5.22332	5.33073	6.76924	7.12825	7.62007	A2-	
AT4G38560	123.6681	2.5617	0.3004	8.52749	1.50E-17	6.25E-16	7.63043	7.61616	7.99533	5.74125	4.50885	4.4747	A1+	Phospholipase-like protein (PEARLI 4) family protein. Overlaps with NAT AT4G09715
AT4G39030	273.6344	2.52542	0.2368	10.6648	1.49E-26	1.16E-24	8.60625	8.79309	9.19483	6.65684	5.82339	6.14868	A1+	EDS5 ENHANCED DISEASE SUSCEPTIBILITY 5 (EDS5);SCORD3, SID1, susceptible to coronatine-deficient Pst DC3000 3, SALICYLIC ACID INDUCTION DEFICIENT 1 Encodes an orphan multidrug and toxin extrusion transporter. Essential component of salicylic acid-dependent signaling for disease resistance. Member of the MATE-transporter family. Expression induced by salicylic acid. Mutants are salicylic acid-deficient
AT4G39070	50.59654	-1.5665	0.3263	-4.8008	1.58E-06	1.82E-05	4.82609	4.06764	4.84945	6.60759	6.29264	5.8998	A1+	Encodes BZS1, a brassinosteroids-regulated BZR1 target (BRBT) gene. BZS1 is a putative zinc finger transcription factor. Expression of BZS1 was increased under BR-deficient condition and repressed by BR. Transgenic Arabidopsis plants overexpressing BZS1 showed a hypersensitivity to the BR biosynthetic inhibitor brassinazole (BRZ). In contrast, transgenic plants expressing reduced level of BZS1 had longer hypocotyls than wild type when grown on BRZ. B-box zinc finger family protein; FUNCTIONS IN: sequence-specific DNA binding transcription factor activity, zinc ion binding; INVOLVED IN: response to karrikin, response to chitin, regulation of transcription;
AT4G39070	92.2453	-2.7087	0.26666	-10.158	3.05E-24	1.84E-22	4.72207	3.96547	4.74322	7.63346	7.12834	7.24984	A1-	
AT5G01080	10.05227	-2.5503	0.58688	-4.3454	1.39E-05	0.0001344	0.64015	0.62641	0.69459	1.42937	4.16427	5.3565	A1+	Beta-galactosidase related protein
AT5G01080	22.36107	-4.2179	0.44841	-9.4064	5.14E-21	2.57E-19	0.60238	0.58899	0.65347	5.67203	5.45092	5.35204	A1-	
AT5G01380	19.55855	3.11726	0.5113	6.09679	1.08E-09	2.02E-08	5.08538	5.42179	5.29801	1.42937	0	1.80658	A1+	Homeodomain-like superfamily protein; CONTAINS InterPro DOMAIN/s: SANT, DNA-binding (InterPro:IPR001005), MYB-like
AT5G10250	32.3457	1.98945	0.38315	5.19232	2.08E-07	2.78E-06	6.00246	5.57774	5.69239	3.48023	3.71068	3.61372	A1+	DOT3 DEFECTIVELY ORGANIZED TRIBUTARIES 3 (DOT3), Encodes a protein with an N-terminal BTB/POZ domain and a C-terminal NPH3 family domain. dot3 mutants have defects in shoot and primary root growth and produce an aberrant parallel venation pattern in juvenile leaves.
AT5G10250	27.55039	2.51078	0.40296	6.23083	4.64E-10	9.83E-09	5.89624	5.4712	5.58441	2.79716	2.66509	2.61074	A1-	
AT5G13170	54.23112	1.81543	0.37472	4.84475	1.27E-06	1.72E-05	5.66531	6.93676	6.6037	4.88574	4.08983	3.83605	A1-	SAG29, senescence-associated gene 29, Encodes a member of the SWEET sucrose efflux transporter family proteins
AT5G13170	43.8212	2.84608	0.39719	7.16562	7.74E-13	4.43E-11	5.56923	6.8429	6.51281	0	2.92871	2.74912	A2-	
AT5G13320	812.5962	2.53672	0.5663	4.47949	7.48E-06	7.62E-05	10.555	10.4618	10.7206	7.41218	3.71068	6.58389	A1+	PBS3, AVRPPHB SUSCEPTIBLE 3, AtGH3.12, GDG1, GH3.12, GH3-LIKE DEFENSE GENE 1, GRETCHEN HAGEN 3.12, WIN3, HOPW1-1-INTERACTING 3, Encodes an enzyme capable of conjugating amino acids to 4-substituted benzoates. 4-HBA (4-hydroxybenzoic acid) and pABA (4-aminobenzoate) may be targets of the enzyme in Arabidopsis, leading to the production of pABA-Glu, 4HBA-Glu, or other related compounds. This enzyme is involved in disease-resistance signaling. It is required for the accumulation of salicylic acid, activation of defense responses, and resistance to Pseudomonas syringae. Salicylic acid can decrease this enzyme's activity in vitro and may act as a competitive inhibitor.
AT5G13930	177.6488	0.73527	0.20759	3.54189	0.000397	0.0027085	7.51183	7.91484	7.95275	7.24511	7.09209	6.8095	A1+	TT4, TRANSPARENT TESTA 4, ATCHS, CHALCONE SYNTHASE, CHS, Encodes chalcone synthase (CHS), a key enzyme involved in the biosynthesis of flavonoids. Required for the accumulation of purple anthocyanins in leaves and stems. Also involved in the regulation of auxin transport and the modulation of root gravitropism.
AT5G13930	118.3482	2.74638	0.26883	10.2161	1.68E-24	1.03E-22	7.40447	7.80641	7.84301	4.70005	5.45092	4.55311	A1-	
AT5G13930	108.2862	2.78197	0.27824	9.99849	1.55E-23	2.44E-21	7.307	7.71219	7.75156	4.91162	3.98933	4.71048	A2-	
AT5G14920	1149.303	-1.4137	0.20845	-6.7823	1.18E-11	2.84E-10	9.17612	8.88389	9.62365	10.8652	10.8988	10.3304	A1+	GASA14, A-stimulated in Arabidopsis 14, Gibberellin-regulated family protein; INVOLVED IN: response to gibberellin

AT5G14920	2058.137	-2.5062	0.22549	-11.115	1.07E-28	8.29E-27	9.06835	8.77523	9.51359	11.6477	12.2204	11.3818	A1-	Stimulus
AT5G15360	47.79843	-4.2141	0.56253	-7.4914	6.81E-14	2.06E-12	0.64015	0	0.69459	2.9585	7.16684	7.09959	A1+	Transmembrane protein
AT5G15360	73.34113	-6.0211	0.40152	-14.996	7.80E-51	1.50E-48	0.60238	0	0.65347	7.14402	7.18241	7.27861	A1-	
AT5G17460	89.5997	-2.9729	0.31979	-9.2965	1.45E-20	7.59E-19	4.05639	4.11368	4.37779	6.53041	7.63893	7.62281	A1+	Glutamyl-tRNA (Gln) amidotransferase subunit C
AT5G17460	38.23128	-1.7012	0.3291	-5.1693	2.35E-07	3.56E-06	3.95514	4.01129	4.27309	6.33521	5.90133	5.45643	A1-	
AT5G17460	84.10326	-2.7881	0.32822	-8.4947	1.98E-17	1.89E-15	3.8636	3.92269	4.18612	7.94403	6.32861	7.08815	A2-	
AT5G20260	15.01639	-2.0249	0.49946	-4.0541	5.03E-05	4.29E-04	3.14059	1.89477	1.51363	4.29471	4.78679	5.06504	A1+	Exostosin family protein
AT5G20260	22.74582	-2.6376	0.42278	-6.2388	4.41E-10	9.37E-09	3.04528	1.81597	1.44299	5.53062	5.61718	5.02993	A1-	
AT5G22380	85.45932	4.141	0.38202	10.8399	2.23E-27	1.81E-25	7.26179	7.23385	7.61326	3.24283	0	3.08651	A1+	NAC090, NAC domain containing protein 90 (NAC090); FUNCTIONS IN: sequence-specific DNA binding transcription factor activity; INVOLVED IN: multicellular organismal development, regulation of transcription
AT5G22680	23.38055	3.01395	0.48239	6.248	4.16E-10	8.14E-09	5.32532	5.61022	5.54731	2.60404	0	1.80658	A1+	F-box protein
AT5G23240	131.497	-2.8814	0.25442	-11.326	9.78E-30	9.18E-28	4.88181	4.5418	5.20432	7.52259	7.86529	8.17135	A1+	ATDJC17, DJC76, DNA J PROTEIN C76, DNAJ heat shock N-terminal domain-containing protein;
AT5G23240	103.4181	-2.5439	0.27307	-9.316	1.21E-20	5.96E-19	4.77764	4.43775	5.09722	7.86467	7.47011	7.05301	A1-	
AT5G23240	130.608	-3.0456	0.22516	-13.526	1.10E-41	5.92E-39	4.68326	4.3476	5.00812	7.64769	8.06191	7.92872	A2-	
AT5G24080	13.98696	2.75335	0.55115	4.99566	5.86E-07	7.30E-06	4.43724	4.40039	5.36449	1.42937	0	1.16932	A1+	Protein kinase superfamily protein; FUNCTIONS IN: protein serine/threonine kinase activity, protein kinase activity, kinase activity, ATP binding; INVOLVED IN: protein amino acid phosphorylation;
AT5G24150	22.58289	3.091	0.52373	5.90187	3.59E-09	6.18E-08	4.70775	5.887	5.56626	2.1331	1.77337	0	A1+	SQP1 squalene monooxygenase gene homolog, FUNCTIONS IN: squalene monooxygenase activity; INVOLVED IN: sterol biosynthetic process;
AT5G24150	22.54703	2.20417	0.44176	4.98951	6.05E-07	8.63E-06	4.60406	5.78001	5.45848	2.19233	2.66509	2.83386	A1-	
AT5G24240	48.62145	-4.1443	0.54669	-7.5808	3.44E-14	1.06E-12	0.64015	1.3957	0.69459	3.48023	7.19092	7.07307	A1+	ATPI4Kγ3 Encodes PI4Kc3, localizes to the nucleus and has autophosphorylation activity, but no lipid kinase activity. Overexpression mutants display late-flowering phenotype.
AT5G24240	44.03725	-4.5235	0.44593	-10.144	3.52E-24	2.12E-22	0.60238	1.32917	0.65347	5.19788	6.60241	7.03064	A1-	
AT5G26270	181.761	-3.4697	0.57881	-5.9946	2.04E-09	3.68E-08	2.45099	2.26486	2.03285	5.54593	9.11593	8.90881	A1+	Transmembrane protein
AT5G26270	131.7881	-5.4112	0.32024	-16.897	4.71E-64	1.38E-61	2.36338	2.1793	1.95037	7.55346	8.32894	8.09686	A1-	
AT5G26270	26.01006	-1.0984	0.17852	-6.1528	7.61E-10	1.07E-07	3.08744	2.89389	2.62616	5.35808	5.59296	5.66312	A2+	
AT5G28235	15.78096	-4.0191	0.48985	-8.2049	2.31E-16	8.56E-15	0	0	0	5.63796	4.48353	4.67351	A1-	Ulp1 protease family protein
AT5G28235	8.618629	-0.6759	0.14447	-4.6782	2.89E-06	2.74E-04	0	0	0	3.8418	4.49088	4.16097	A2+	
AT5G28810	7.424436	-3.0632	0.51078	-5.9971	2.01E-09	4.02E-08	0	0	0	4.04193	3.54662	4.2772	A1-	
AT5G28810	18.29304	-1.2001	0.1685	-7.1225	1.06E-12	2.01E-10	0	0	0	5.20597	5.49326	4.94502	A2+	
AT5G34780	116.679	-2.3832	0.20581	-11.579	5.24E-31	5.26E-29	5.17731	5.16182	5.25192	7.60018	7.78846	7.49877	A1+	Thiamin diphosphate-binding fold (THDP-binding) superfamily protein, FUNCTIONS IN: oxidoreductase activity, acting on the aldehyde or oxo group of donors, disulfide as acceptor, 2-dehydropantoate 2-reductase activity; INVOLVED IN: pantothenate biosynthetic process
AT5G34780	129.7331	-2.6043	0.21213	-12.277	1.21E-34	1.22E-32	5.07244	5.05608	5.14473	7.76558	8.09527	7.57761	A1-	
AT5G34780	93.48527	-2.1995	0.24438	-9.0003	2.25E-19	2.53E-17	4.97738	4.96436	5.05554	6.71319	7.36373	7.65326	A2-	
AT5G34850	944.328	7.95557	0.36436	21.8345	1.09E-105	1.75E-102	10.8638	10.8783	10.9045	0	0	2.85705	A1+	Purple acid phosphatase 26
AT5G34850	874.9935	7.97147	0.35623	22.3772	6.56E-111	8.43E-108	10.7559	10.7694	10.7944	1.13103	0	1.01552	A1-	
AT5G35375	24.18782	-3.3788	0.57452	-5.881	4.08E-09	6.96E-08	1.08201	0	0.69459	2.1331	6.20076	6.09697	A1+	Transmembrane protein
AT5G35375	6.586777	-2.3977	0.50899	-4.7107	2.47E-06	3.16E-05	1.02605	0	0.65347	3.68946	3.54662	4.02897	A1-	
AT5G37490	45.76507	3.02402	0.41139	7.3507	1.97E-13	5.69E-12	6.25543	6.61951	6.36863	4.02145	0	2.58406	A1+	ARM repeat superfamily protein; FUNCTIONS IN: ubiquitin-protein ligase activity, binding; INVOLVED IN: response to chitin; LOCATED IN: ubiquitin ligase complex
AT5G38190	10.99572	-3.2398	0.58637	-5.5253	3.29E-08	4.95E-07	0	0	0	2.1331	5.12347	4.89377	A1+	Myosin heavy chain-like protein
AT5G38700	33.54536	3.63713	0.47727	7.62076	2.52E-14	7.93E-13	6.05188	5.74796	6.29099	2.1331	0	1.80658	A1+	Cotton fiber protein
AT5G39580	104.3411	2.59966	0.3553	7.3169	2.54E-13	7.25E-12	7.75501	7.64388	7.13078	5.54593	3.41604	4.20843	A1+	Peroxidase superfamily protein; FUNCTIONS IN: peroxidase activity, heme binding
AT5G41080	238.0994	-2.9989	0.2738	-10.953	6.45E-28	4.76E-26	5.44274	5.43803	5.60153	8.038	9.37393	8.54255	A1-	ATGDPD2, GDPD2, GLYCEROPHOSPHODIESTER PHOSPHODIESTERASE 2
AT5G41730	32.13856	3.61769	0.47286	7.65061	2.00E-14	6.35E-13	5.89824	5.9518	6.0961	2.1331	0	1.80658	A1+	Protein kinase family protein; FUNCTIONS IN: protein serine/threonine/tyrosine kinase activity, kinase activity; INVOLVED IN: protein amino acid phosphorylation
AT5G42900	53.91136	-3.7234	0.39379	-9.4553	3.22E-21	1.77E-19	2.83661	2.91163	2.03285	6.13802	6.42035	7.27272	A1+	COR27 cold regulated protein 27
AT5G42900	81.18587	-4.344	0.3258	-13.333	1.48E-40	1.94E-38	2.74425	2.81769	1.95037	7.30468	7.67257	6.82502	A1-	
AT5G42900	41.13438	-3.4424	0.3284	-10.482	1.04E-25	1.95E-23	2.66124	2.73684	1.88284	6.07705	6.20471	6.5677	A2-	
AT5G44440	28.15411	-2.501	0.54151	-4.6185	3.87E-06	4.17E-05	3.31264	1.3957	1.51363	3.86258	6.61089	5.56118	A1+	FAD-binding Berberine family protein; FUNCTIONS IN: electron carrier activity, oxidoreductase activity, FAD binding, catalytic activity
AT5G44440	27.09903	-2.79	0.43592	-6.4002	1.55E-10	3.44E-09	3.21591	1.32917	1.44299	5.9747	5.76624	5.19996	A1-	
AT5G45570	297.1668	-4.7587	0.56749	-8.3855	5.05E-17	2.01E-15	0	0.62641	1.16147	3.24283	9.6895	9.89032	A1+	Ulp1 protease family protein
AT5G45570	14.76882	-3.4868	0.48213	-7.2322	4.75E-13	1.33E-11	0	0.58899	1.10158	5.56729	4.08983	4.67351	A1-	
AT5G45570	23.84342	-1.29	0.17292	-7.46	8.65E-14	1.95E-11	0	0.94033	1.61801	5.35808	5.98427	5.27616	A2+	
AT5G45630	32.88955	2.73175	0.43584	6.26784	3.66E-10	7.25E-09	5.91169	5.49319	6.27955	2.60404	2.54524	3.08651	A1+	Senescence regulator (Protein of unknown function, DUF584)

AT5G47240	85.70836	-2.659	0.26188	-10.154	3.19E-24	2.23E-22	4.32111	4.32411	4.78619	6.92153	7.34909	7.39553	A1+	ATNUDT8, NUDIX HYDROLASE HOMOLOG 8, NUDT8, NUDX8
AT5G47240	65.64214	-2.2597	0.29658	-7.6195	2.55E-14	8.06E-13	4.21874	4.22085	4.68013	7.20347	6.75292	6.35243	A1-	
AT5G47240	117.2456	-3.3185	0.21872	-15.172	5.38E-52	4.75E-49	4.12611	4.13143	4.59196	7.73473	7.74356	7.80126	A2-	
AT5G47850	41.32965	2.77757	0.41238	6.73546	1.63E-11	3.84E-10	5.95129	6.44142	6.3136	4.02145	1.77337	2.58406	A1+	CCR4, CRINKLY4 related 4 (CCR4); FUNCTIONS IN: kinase activity; INVOLVED IN: protein amino acid phosphorylation;
AT5G49160	621.2418	-2.6561	0.23121	-11.488	1.52E-30	1.49E-28	6.85163	7.72398	7.25863	10.304	10.2031	9.66248	A1+	Methyltransferase 1;
AT5G49160	4864.811	-3.9412	0.14971	-26.325	1.00E-152	2.67E-148	7.59384	8.47832	7.99747	13.2508	13.2299	13.1414	A2+	
AT5G51000	22.13262	3.74654	0.55245	6.78172	1.19E-11	2.85E-10	4.85422	5.43997	5.98779	0	0	0	A1+	F-box and associated interaction domains-containing protein
AT5G52050	153.8491	3.01566	0.32467	9.28826	1.57E-20	8.19E-19	8.25971	8.07467	8.05021	5.78621	3.95523	4.10751	A1+	MATE efflux family protein; FUNCTIONS IN: antiporter activity, drug transmembrane transporter activity; INVOLVED IN: drug transmembrane transport, transmembrane transport;
AT5G52310	634.278	-2.3675	0.43362	-5.4598	4.77E-08	7.01E-07	7.26703	7.52965	7.09207	8.98668	10.0337	10.7984	A1+	RD29A, LTI78, LOW-TEMPERATURE-INDUCED 78, cold regulated gene, the 5' region of cor78 has cis-acting regulatory elements that can impart cold-regulated gene expression
AT5G52310	912.7899	-2.6089	0.40506	-6.4408	1.19E-10	4.76E-09	7.06243	7.32728	6.89159	11.5756	10.0556	9.89682	A2-	
AT5G52760	227.3983	3.40852	0.25281	13.4826	1.98E-41	3.39E-39	8.75167	8.56148	8.8412	5.6469	3.71068	5.31183	A1+	Copper transport protein family; BEST Arabidopsis thaliana protein match is: Heavy metal transport/detoxification superfamily protein (TAIR:AT5G52750.1).
AT5G54030	30.08113	3.81752	0.53469	7.13964	9.36E-13	2.55E-11	5.71097	5.20499	6.5318	1.42937	0	0	A1+	Cysteine/Histidine-rich C1 domain family protein
AT5G54030	32.62172	1.46889	0.41912	3.50467	4.57E-04	4.44E-03	5.50915	5.00735	6.33207	4.69719	3.83072	1.71775	A2-	
AT5G54710	317.2143	2.91831	0.54077	5.39661	6.79E-08	9.78E-07	9.0032	9.072	9.57066	6.06692	3.04536	4.4747	A1+	Ankyrin repeat family protein
AT5G54720	124.5492	4.25124	0.34855	12.1969	3.23E-34	3.86E-32	7.84186	7.68639	8.17276	4.02145	2.54524	2.85705	A1+	
AT5G56870	3218.963	-2.5524	0.21911	-11.649	2.33E-31	2.41E-29	10.2791	9.3746	9.56242	12.2313	12.699	12.3442	A1+	Beta-galactosidase 4 (BGAL4); INVOLVED IN: lactose catabolic process, using glucoside 3-dehydrogenase, carbohydrate metabolic process, lactose catabolic process via UDP-galactose, lactose catabolic process
AT5G56870	3255.329	-2.6462	0.22651	-11.683	1.57E-31	1.38E-29	10.1712	9.26587	9.45238	12.2931	12.8079	12.2525	A1-	
AT5G56870	3536.11	-2.7336	0.27206	-10.048	9.40E-24	1.54E-21	10.0732	9.17137	9.36065	11.8777	12.8512	12.9217	A2-	
AT5G56970	17.34139	2.56411	0.50721	5.05535	4.30E-07	5.46E-06	4.85422	4.84448	5.38598	2.60404	0	1.80658	A1+	CKX3 cytokinin oxidase 3 (CKX3); FUNCTIONS IN: primary amine oxidase activity, cytokinin dehydrogenase activity; INVOLVED IN: cytokinin catabolic process, It encodes a protein whose sequence is similar to cytokinin oxidase/dehydrogenase, which catalyzes the degradation of cytokinins
AT5G57010	26.81918	2.83445	0.45554	6.2222	4.90E-10	9.52E-09	5.7859	5.65761	5.56626	2.1331	0	3.08651	A1+	Calmodulin-binding family protein
AT5G57640	45.98899	-2.8497	0.4027	-7.0764	1.48E-12	3.94E-11	3.60523	2.91163	3.07401	5.49267	7.04001	6.2704	A1+	GCK domain-containing protein
AT5G57640	36.54749	-2.6125	0.34523	-7.5673	3.81E-14	1.18E-12	3.50645	2.81769	2.97734	6.10514	6.34234	5.61521	A1-	
AT5G57640	21.09576	-1.8245	0.37222	-4.9019	9.49E-07	1.92E-05	3.41728	2.73684	2.89747	4.91162	4.77911	5.65014	A2-	
AT5G58610	18.13549	2.60794	0.51217	5.0919	3.54E-07	4.55E-06	4.57882	5.13974	5.50863	2.1331	0	2.24707	A1+	PHD finger transcription factor, putative; FUNCTIONS IN: RNA binding, DNA binding, zinc ion binding; INVOLVED IN: N-terminal protein myristoylation, regulation of transcription, DNA-dependent, response to chitin;
AT5G58750	35.85393	4.14592	0.51821	8.00052	1.24E-15	4.39E-14	5.74141	5.97692	6.6431	1.42937	0	0	A1+	Disease resistance protein (TIR-NBS-LRR class) family; FUNCTIONS IN: transmembrane receptor activity, nucleoside-triphosphatase activity, nucleotide binding, ATP binding; INVOLVED IN: signal transduction, defense response, apoptosis, innate immune response
AT5G58750	83.54496	0.69436	0.17995	3.85866	1.14E-04	0.0078978	6.47774	6.72467	7.37991	4.84311	6.03242	5.69114	A2+	
AT5G60100	101.2135	-3.2351	0.29771	-10.867	1.66E-27	1.36E-25	4.19481	4.36275	3.806	6.92153	7.83504	7.72002	A1+	Encodes pseudo-response regulator 3 (APRR3/PRR3). PRR3 transcript levels vary in a circadian pattern with peak expression at dusk under long and short day conditions. PRR3 affects the period of the circadian clock and seedlings with reduced levels of PRR3 have shorter periods, based on transcriptional assays of clock-regulated genes. PRR3 is expressed in the vasculature of cotyledons and leaves where it may help stabilize the TOC1 protein by preventing interactions between TOC1 and the F-box protein ZTL.
AT5G60100	73.01443	-2.8578	0.25983	-10.999	3.87E-28	2.89E-26	4.09295	4.25934	3.70397	7.09462	7.07217	6.94949	A1-	
AT5G60100	77.16492	-2.9335	0.28444	-10.313	6.14E-25	1.10E-22	4.00081	4.16978	3.61936	6.92542	6.78314	7.57747	A2-	
AT5G62480	155.575	4.74371	0.3472	13.6627	1.70E-42	3.11E-40	8.15901	8.10063	8.47563	3.48023	0	3.45845	A1+	GST14, ATGSTU9, GST14B, GLUTATHIONE S-TRANSFERASE 14B, glutathione S-transferase tau 9, GLUTATHIONE S-TRANSFERASE 14, Encodes glutathione transferase belonging to the tau class of GSTs
AT5G62480	368.8228	0.70396	0.17658	3.98656	6.70E-05	0.0048458	8.90424	8.85561	9.21663	6.9194	8.44286	7.73202	A2+	
AT5G64810	97.06413	3.33198	0.31283	10.6512	1.72E-26	1.34E-24	7.41128	7.55907	7.53845	4.52436	1.77337	4.10751	A1+	WRKY51 WRKY DNA-binding protein 51, member of WRKY Transcription Factor; Group II-c. Involved in jasmonic acid inducible defense responses
AT5G66620	78.80702	2.68079	0.36757	7.29327	3.03E-13	8.54E-12	6.95266	7.22339	7.2528	5.12374	1.77337	3.88164	A1+	DAR6, DA1-related protein 6 (DAR6); FUNCTIONS IN: zinc ion binding
AT5G66640	90.33773	2.73678	0.40612	6.73892	1.60E-11	3.75E-10	6.93959	7.33934	7.70163	5.25748	1.77337	3.75389	A1+	DAR3, DA1-related protein 3
AT5G67450	47.39256	3.5928	0.42884	8.37797	5.38E-17	2.14E-15	6.52409	6.22779	6.76296	2.9585	0	2.58406	A1+	ZF1, zinc-finger protein 1, Encodes zinc-finger protein. mRNA levels are elevated in response to low temperature, cold temperatures and high salt. The protein is localized to the nucleus and acts as a transcriptional repressor
