# Supplementary material for: Induction of epigenetic variation in Arabidopsis by over-expression of DNA METHYLTRANSFERASE1 (MET1)
Source: PLoS One. 2018 Feb 21;13(2):e0192170. doi: 10.1371/journal.pone.0192170 (PMC5821449; doi:10.1371/journal.pone.0192170)
Supplement: S8 Table — (PDF) [file pone.0192170.s013.pdf]

S8 Table: List of primers		
AT number	Forward Primer	Reverse Primer
qPCR analysis		
AT3G01345	TTGCTGCCCACACCAAGTATCG	ACCAGCCCAAACAGAGGTAGAG
AT3G27473	GCCTCTGGATCTTAGCCTCCAATG	TGACGACAAGCTCGACATCTCC
AT3G30720	AAACCTCCTTTCGATCTGTCAGC	ATGGCTGACCGTGTGAGTCTTG
AT3G30820	CAGAGCATCTTCGCTGTACCTG	TTCGTCGCGGAGAGAAATGAGG
AT4G25530	TCCCATGACTTGCGTGACTCTG	CACGTTGACCCATTTGCCTGTG
AT5G34850	TCACAGTTGGAGACGGAGGAAATC	TGGCTGTGGTTCCGTAAACCTTC
Bisulphite Sequencing		
AT number	Forward Primer	Reverse Primer
AT3G01345	GTTGGTGAYAAAGAGAAGATG	ATAACAACATCAAAAAATTT
AT3G27473	ATAAAATATTAGGTTAAGTG	ATCTCRAATCAATATTTCCARCT
AT3G30720	GAGATATTGGYYTTTGATTTGTYTGTTT	TCTTRTTTCTTCTRATCTTCAAT
AT5G34850	GAATGTTGATTTYAAATYTAGAATGAAG	CAAACTTTTTCTTRACACCAAACTATTTC
ChIP analysis		
AT number	Forward Primer	Reverse Primer
AT3G01345	CGAGGCCAAAGCTTCCAAAC	GAGAGCGACAAGGGAACGAT
AT3G27473	ATCCACAACCGCCATGACTT	GAGAACCCATCACCAGACGA
AT3G30720	AGGTTCATTTTGCCTCACACT	GCCCGACCCATGATATGACC
AT5G34850	TGGGTTACACCTGATGAACCTG	TGGTAAGTCCCTTGAGCAACA
AT5G34850 mapping	Forward Primer	Reverse Primer
Pp1	CTCACTCGCATAGTTCCGACA	ATACAATCTGAGAAATTCGTTGTGA
Pp2	CAAACTTTTTCTTGACACCAAACTATTTC	ATACAATCTGAGAAATTCGTTGTGA
Pp3	AAGACCCAATCCATTTCCCTCA	TGGTAAGTCCCTTGAGCAACA
Pp4	AAGACCCAATCCATTTCCCTCA	CAATCTTGTAATAGTATTTTGTATC
